# Genome-scale metabolic network reconstruction and *in silico* flux analysis of the thermophilic bacterium *Thermus thermophilus* HB27

**DOI:** 10.1186/1475-2859-13-61

**Published:** 2014-04-28

**Authors:** Na-Rae Lee, Meiyappan Lakshmanan, Shilpi Aggarwal, Ji-Won Song, Iftekhar A Karimi, Dong-Yup Lee, Jin-Byung Park

**Affiliations:** 1 Department of Food Science & Engineering, Ewha Womans University, 11-1 Daehyun-dong, Seodaemun-gu, Seoul 120-750, Korea; 2 Department of Chemical and Biomolecular Engineering, National University of Singapore, 4 Engineering Drive 4, Singapore 117585, Singapore; 3 Bioprocessing Technology Institute, Agency for Science, Technology and Research (A*STAR), 20 Biopolis Way, #06-01, Centros, Singapore 138668, Singapore; 4 Global Top5 Research Program, Ewha Womans University, 11-1 Daehyeon-dong, Seodaemun-gu, Seoul 120750, Korea

**Keywords:** *Thermus thermophilus*, Thermophile, Genome-scale metabolic model, Constraints-based flux analysis, Ethanol

## Abstract

**Background:**

*Thermus thermophilus*, an extremely thermophilic bacterium, has been widely recognized as a model organism for studying how microbes can survive and adapt under high temperature environment. However, the thermotolerant mechanisms and cellular metabolism still remains mostly unravelled. Thus, it is highly required to consider systems biological approaches where *T. thermophilus* metabolic network model can be employed together with high throughput experimental data for elucidating its physiological characteristics under such harsh conditions.

**Results:**

We reconstructed a genome-scale metabolic model of *T. thermophilus*, *i*TT548, the first ever large-scale network of a thermophilic bacterium, accounting for 548 unique genes, 796 reactions and 635 unique metabolites. Our initial comparative analysis of the model with *Escherichia coli* has revealed several distinctive metabolic reactions, mainly in amino acid metabolism and carotenoid biosynthesis, producing relevant compounds to retain the cellular membrane for withstanding high temperature. Constraints-based flux analysis was, then, applied to simulate the metabolic state in glucose minimal and amino acid rich media. Remarkably, resulting growth predictions were highly consistent with the experimental observations. The subsequent comparative flux analysis under different environmental conditions highlighted that the cells consumed branched chain amino acids preferably and utilized them directly in the relevant anabolic pathways for the fatty acid synthesis. Finally, gene essentiality study was also conducted via single gene deletion analysis, to identify the conditional essential genes in glucose minimal and complex media.

**Conclusions:**

The reconstructed genome-scale metabolic model elucidates the phenotypes of *T. thermophilus*, thus allowing us to gain valuable insights into its cellular metabolism through *in silico* simulations. The information obtained from such analysis would not only shed light on the understanding of physiology of thermophiles but also helps us to devise metabolic engineering strategies to develop *T. thermophilus* as a thermostable microbial cell factory.

## Background

*Thermus thermophilus* is a gram-negative, obligate aerobic bacterium, representing one of the best-studied thermophiles. It usually colonizes the terrestrial volcanic hot springs (grows optimally between 65 and 72°C) and was originally isolated from a Japanese thermal spa [1]. In addition to the ability of surviving at such high temperatures, *T. thermophilus* is resistant to other stress such as harsh chemical conditions [2]. These properties motivated researchers to extract or isolate numerous proteins from *T. thermophilus*, making it as a model organism in structural genomics with significant industrial potential [3-6]. For example, several thermostable proteins are already used in commercial processes, including the DNA polymerase in PCR techniques, α-amylases and glucose isomerases in starch processing, esterases, lipases and proteases in organic synthesis, and xylanases in paper and pulp manufacturing [7,8]. Moreover, *T. thermophilus* is being recognized as a potential microbial cell factory for the low cost ethanol fermentation from lignocellulosic waste materials since it can grow by utilizing most of the C5/C6 carbon sources at relatively high temperatures, i.e. 70–80°C, thus reducing the energy costs: no cooling step is required following enzymatic hydrolysis, rendering it easier to distil subsequent fermentations [9].

Despite enormous potentials for biotechnological applications, the current knowledge on the unique cellular physiology of *T. thermophilus* is very limited; to date, the production of distinctive carotenoid molecules [10] and the use of adaptive protein synthesis strategies [11] are only two notable traits unravelled at the molecular level. Such limited studies are mostly due to the technical difficulties in cultivating and analysing thermophilic microbes; cell culture experiments require high amount of energy to maintain the optimal growth conditions. Hence, it is indeed required to develop more systematic approaches for improving our understanding of *T. thermophilus* cellular behaviour. In this regard, constraints-based *in silico* metabolic modeling and analysis can be considered as one of the promising techniques to characterize the physiological behaviour and metabolic states of an organism upon various environmental/genetic changes as they systematically capture the genotype-phenotype relationships from the entire genome information [12,13]. As a result, several genome-scale metabolic models (GSMMs) are now available for describing the metabolic organization of various organisms including *Escherichia coli*[14], *Bacillus subtilis*[15], *Saccharomyces cerevisiae*[16], *Pichia pastoris*[17], *Corynebacterium glutamicum*[18], *Ralstonia eutropha*[19], *Pseudomonas aeruginosa*[20], and even for multicellular eukaryotes such as *Mus musculus*[21] and *Homo sapiens*[22]. Moreover, with the availability of several conveniently accessible constraints-based modeling software tools [23], these models have been largely utilized to postulate various strain improvement strategies [17-19,24,25]. Thus, the development of *T. thermophilus* GSMM based on the currently available biochemical and genomic information and its subsequent *in silico* analysis enables us to elucidate its unique metabolic behaviour.

In thermophilic microbes regard, there have been only a few initial attempts to model their cellular metabolisms. First, an *in silico* model of *Thermotoga maritima* was presented, covering its central metabolism along with the 3D structures of all the enzymes accounted in the network [26]. Recently, the genome-scale metabolic model of thermophilic archeon, *Sulfolobus solfataricus*, was also developed, and used to describe its autotrophic growth in bicarbonate via hydroxypropionate-hydroxybutyrate cycle under aerobic conditions [27]. However, both models are not mature enough to explain the molecular mechanisms of high temperature adaptations as they do not consider the detailed biosynthetic machinery of biomolecules which help them to retain the integrity of their cell wall membranes. Therefore, in this work, we reconstructed the genome-scale metabolic model of *T. thermophilus* based on the genome annotation of HB27 wild-type strain [28] for investigating unique characteristics of thermophilic microbes. Additionally, the model was functionally characterized by gene essentiality studies to identify essential genes for cellular growth while growing in both glucose minimal and amino acid supplemented complex media.

## Results

### Reconstruction of *T. thermophilus* genome-scale metabolic network

**Figure 1:**
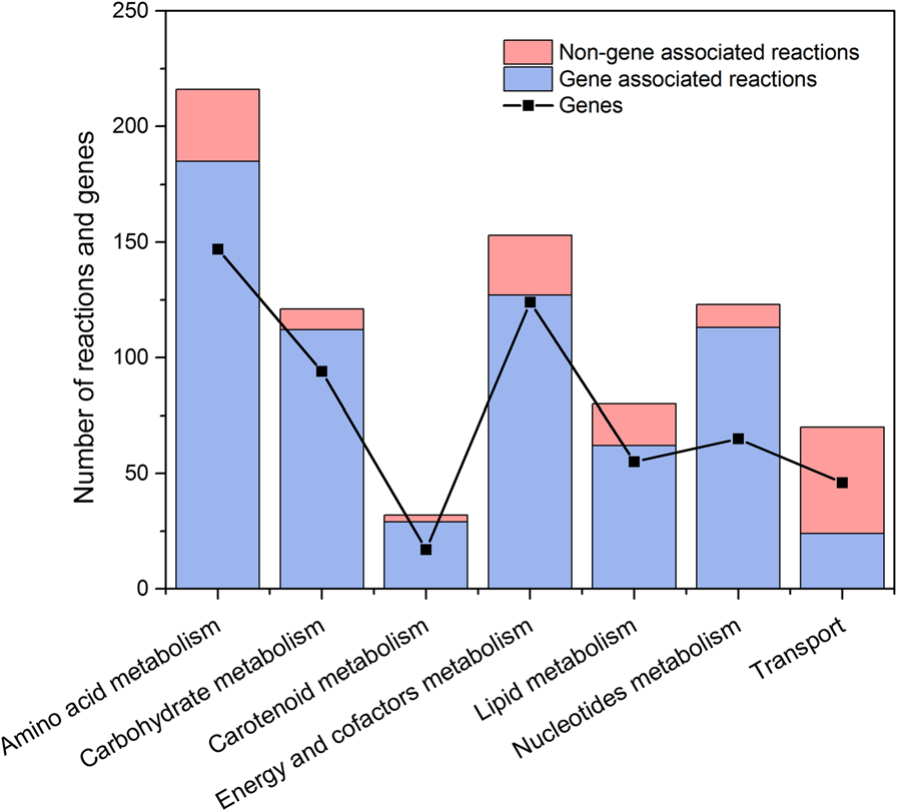
**Distribution of reactions and genes across various metabolic subsystems in *i*** **TT548.**

The genome-scale metabolic network of *T. thermophilus* HB27 was reconstructed through a three step procedure: (1) construction of draft network via compilation of genes, reactions and pathway information from biochemical databases based on the genome annotation of *T. thermophilus* HB27, (2) manual curation of the draft model by verifying the elemental balances in reactions and assigning proper gene-reaction relationships, and (3) gap filling using organism specific knowledge (see Methods). During the reconstruction process, significant efforts were highly required to identify and resolve the network gaps across various metabolic pathways. Such gaps exist due to the incomplete genome annotations which result in missing biochemical reactions and dead ends. These gaps can be appropriately filled by the addition of new reactions based on information obtained from the literature or inferred by the genome annotation of other organisms. For example, the initial model contained several metabolic gaps in the synthetic pathway of thermozeaxanthin and thermobiszeaxanthin, the unique type of carotenoids that are found only in thermophiles, enabling their cellular membrane to retain its fluidity even at very high temperatures [29-31]. Therefore, in order to fill such gaps, we added the reactions corresponding to glycosyltransferase and acyltransferase enzymes from *Staphylococcus aureus* subsp. *aureus* 11819–97 and *Halobacillus halophilus* DSM 2266 for the sequential glycosylation and esterification of zeaxanthin with glucose and branched-chain fatty acids, producing thermozeaxanthin. Similarly, the draft model also had several gaps in the carbon assimilatory pathways, and thus was unable to consume six carbon sources, namely, trehalose, palatinose, isomaltose, cellobiose, glutamate and mannose. However, earlier studies have reported that *T. thermophilus* can grow on all these carbon sources [32,33]. Such discrepancies were again resolved by adding new reactions corresponding to the α-glucosidase and mannokinase enzymes based on the information available from KEGG and MetaCyc databases. Overall, we included 74 new reactions for 63 enzymes, thereby improving its network connectivity (see Additional file 1 for complete list of reactions added). The gap filling of draft model was followed by the identification of genetic evidence for the newly added enzymes via sequence-based homology searches. For this purpose, a BLASTp search was performed in NCBI database for the enzymes that could resolve the network gaps using their amino acid sequences collected from various other organisms against the non-redundant protein sequences of *T. thermophilus* HB27 genome. In such a way, of the 63 enzymes added, we could assign a putative locus for 10 of them, thus providing possible new annotations (see Additional file 1 for the list of new annotations). The final genome-scale metabolic network of *T. thermophilus*, *i*TT548, contains 548 unique genes (ORF coverage – 24%), 796 reactions and 635 unique metabolites. In *i*TT548, all the 796 reactions were classified into seven major metabolic subsystems: carbohydrates, amino acid, energy and cofactors, lipids, nucleotides, carotenoids and transport. Among them, amino acid metabolism has largest number of reactions and genes, followed by carbohydrates and energy and cofactors metabolism (Figure 1). The detailed list of completely curated *T. thermophilus* HB27 metabolic network containing the various genes, reactions, and metabolites can be obtained from Additional file 1, and also available as Systems Biology Markup Language (SBML) file (level 2, version 1, http://sbml.org/) (Additional file 2).

### Network characteristics of *i*TT548 and its comparison with *E. coli* GSMM

**Figure 2:**
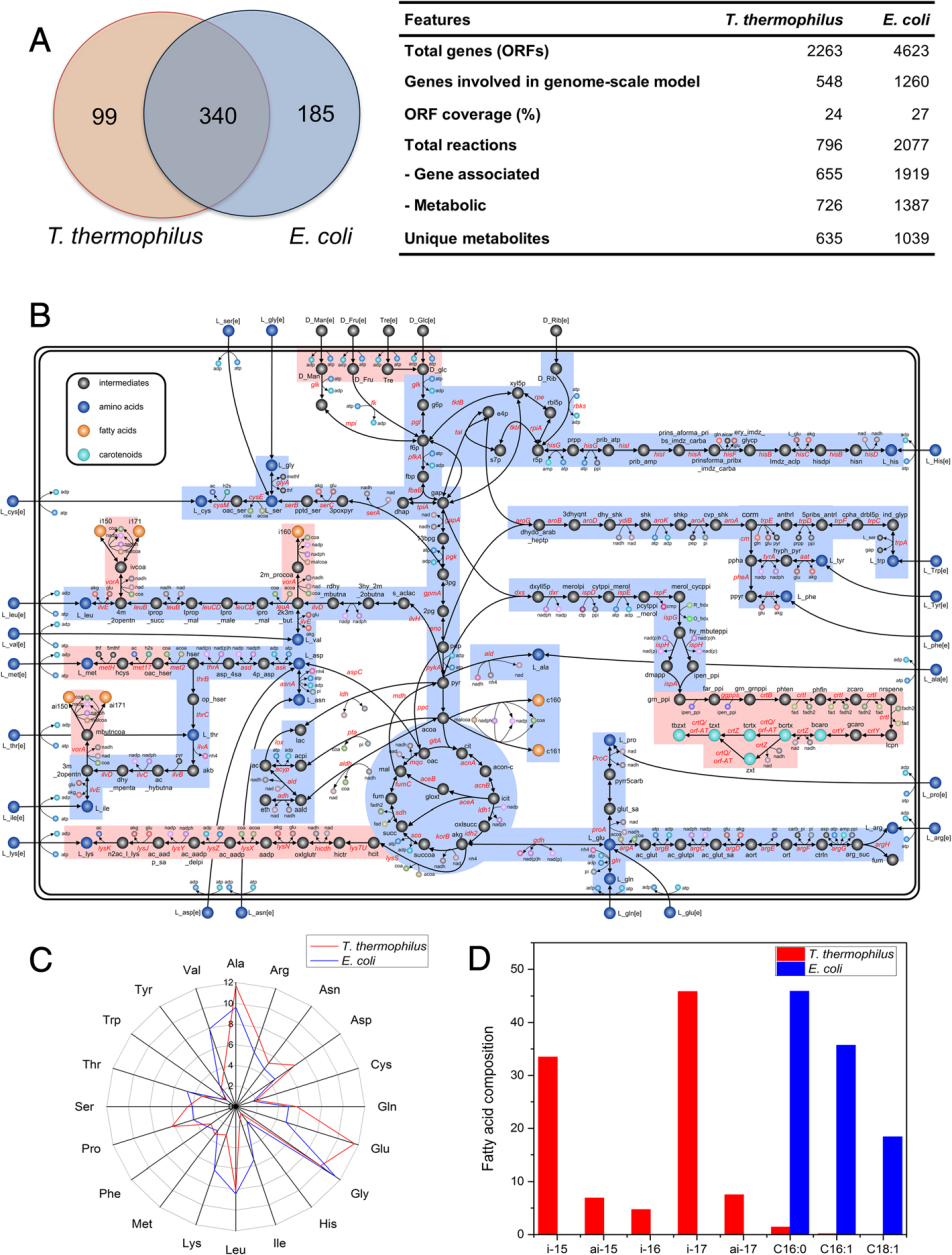
**Metabolic organization and biomass composition of *T. thermophilus *and *E. coli*. (A)** General features of the *i*TT548 in comparison with *E. coli i*AF1260 GSMM (Feist et al. 2007), **(B)** Central metabolic network of *T. thermophilus*, **(C)** amino acid composition (mol%) and **(D)** fatty acid composition (mol%). The numbers in the Venn diagram represents the enzymes in each organism. The common and unique pathways of *T. thermophilus* are highlighted with blue and red backgrounds, respectively. The number of unique and common enzymes was identified using the EC numbers. The biomass data for *E. coli* was obtained from *i*AF1260 GSMM. See supplementary 1 for metabolite and enzyme abbreviations used in the network diagram.

Figure 2A presents the overall network features of *i*TT548 and its comparison with *E. coli* using the metabolic model (*i*AF1260) [14] in terms of the EC numbers. Here, it should be noted that we were unable to fairly compare the metabolic characteristics of *T. thermophilus* with other thermophiles since the *T. maritima* model is only limited to the central metabolism while *S. solfataricus* GSMM was not accessible and no link was provided. From the comparison, there are 363 enzymes/genes are common between *E. coli* and *T. thermophilus*, mostly belonging to the central metabolic pathways such as glycolysis, pentose phosphate pathway and the TCA cycle. However, certain notable differences were observed in the amino acid synthetic pathways of *T. thermophilus*. For instance, lysine is synthesized via the alpha-aminoadipate pathway instead of diaminopimelate pathway. Similarly, the upstream of methionine synthetic pathway is not conserved with *E. coli*: the precursor molecule, homocysteine, is produced from O-acetyl-L-homoserine and hydrogen sulphide via O-acetyl-L-(homo) serine sulfhydrylase in *T. thermophilus* and from O-succinyl-L-homoserine and cysteine through O-succinyl-homoserine lyase in *E. coli*. The comparison between transport reactions further revealed that *T. thermophilus* lacks the phosphoenolpyruvate-dependent phosphotransferase system (PTS), a typical bacterial transport system, and thus consumes most of the carbohydrates including glucose via ATP-binding cassette (ABC) transporters.

Interestingly, as a unique feature of *T. thermophilus*, *i*TT548 contains the necessary biosynthetic machinery for synthesizing several molecules which help them in habituating high temperatures. Unlike many other gram-negative bacteria, *T. thermophilus* does not contain lipopolysaccharides in cell outer membrane [34]. Instead, it embeds complex carotenoid glucoside esters with various branched chain fatty acids, known as thermozeaxanthins and thermobiszeaxanthins, in the lipid bilayers. Such an arrangement offers multiple advantages including the retention of membrane fluidity at high temperatures and reduction of oxygen diffusion through the membrane for preventing oxidation damage [10,35]. Furthermore, *T. thermophilus* synthesizes several unique polyamines such as thermine, spermine, thermospermine and caldopentamine using a distinct pathway from L-arginine via aminopropyl agmatine [36]. These polyamines are essential for high temperature protein synthesis by ensuring the proper structure formation of the initiation complex among 30S ribosomal subunit, the messenger, and the initial aminoacyl-tRNA. As a notable exception to gram-negative bacterium, *T. thermophilus* also synthesizes branched chain fatty acids from amino acids such as valine, leucine and isoleucine via ketoisovalerate oxidoreductase (*vorA*) as in gram-positive bacteria such as *Bacillus*. Table 1 list the unique carotenoid and polyamine molecules, and relevant genes of the corresponding synthetic pathways accounted in *i*TT548; Figure 2 illustrates the central metabolic network of *T. thermophilus* where the branched chain fatty acids and carotenoid synthetic pathways are highlighted.

**Table 1:** **Biosynthetic machinery of unique molecules in *T. thermophilus***

**Molecules**	**No. of reactions**	**Genes involved**	**Major metabolic precursors**
**Branched fatty acids**	**19**	*ilvE* (TTC1870), *vorA* (TTC1756) and *vorB* (TTC1757), *FabF* (TTC0049 or TTC0045), *FabG* (TTC0047 or TTC0394), *FabZ* (TTC1463), *FabI* (TTC0343)	Leucine, valine, isoleucine and acetyl-coA
(i-15:0, ai-15:0, i-16:0, i-17:0, ai-17:1)			
**Carotenoids**	**18**	*CrtE* (TTC1986), *CrtB* (TT_P0057), *CrtI* (TT_P0066), *CrtY* (TT_P0060), *CrtZ* (TT_P0059), *CruC* (TT_P0062), *CruD* (TT_P0061)	Isopentenyl diphosphate (IPP), UDP-glucose and branched fatty acids
(thermocryptoxanthin, thermozeaxanthin and thermobiszeaxanthin)			
**Polyamines**	**16**	*SpeA* (TTC1277), *SpeB* (TTC0764 or TTC1132), *SpeD1* (TTC0473), *SpeD2* (TTC1093), *SpeE* (TTC0472)	Putrescine, spermidine and spermine
(1,3-diaminopropane, norspermidine, sym-homospermidine, thermine, thermospermine, homospermine, caldopentamine, thermopentamine, homocaldopentamine, caldohexamine, homocaldohexamine, tris(3-aminopropyl)amine, tetrakis(3-aminopropyl)ammonium)			

In *i*TT548, we have also included a biomass equation based on our amino acid compositional analysis and the data obtained from literature. Importantly, such biomass composition must be carefully formulated to avoid any erroneous conclusions from the flux balance analysis [37]. It should be noted that the earlier thermophile models, *T. maritima* and *S. solfataricus*, adopted the biomass equation from *E. coli* and *M. bakeri*, respectively, both grow at 37°C which is well below the optimal growing temperature range of thermophiles (50 ~ 80°C). In this regard, the compositions of some amino acids, valine, lysine, threonine, lysine, glutamine and isoleucine, are very distinctive between *T. thermophilus* and *E. coli* (Figure 2C). Similarly, the fatty acid compositions of *T. thermophilus* were also different from *E. coli*; the linear chain fatty acids composition are almost negligible in *T. thermophilus* while the branched-chain fatty acids such as iso-C17:0 and iso-C15:0, which are not present in *E. coli*, contribute the bulk of total lipid compositions (Figure 2D). Collectively, these results highlight the need for the careful estimation of biomass equation while modeling thermophiles.

### Model validation using minimal and complex media during batch cultures

**Figure 3:**
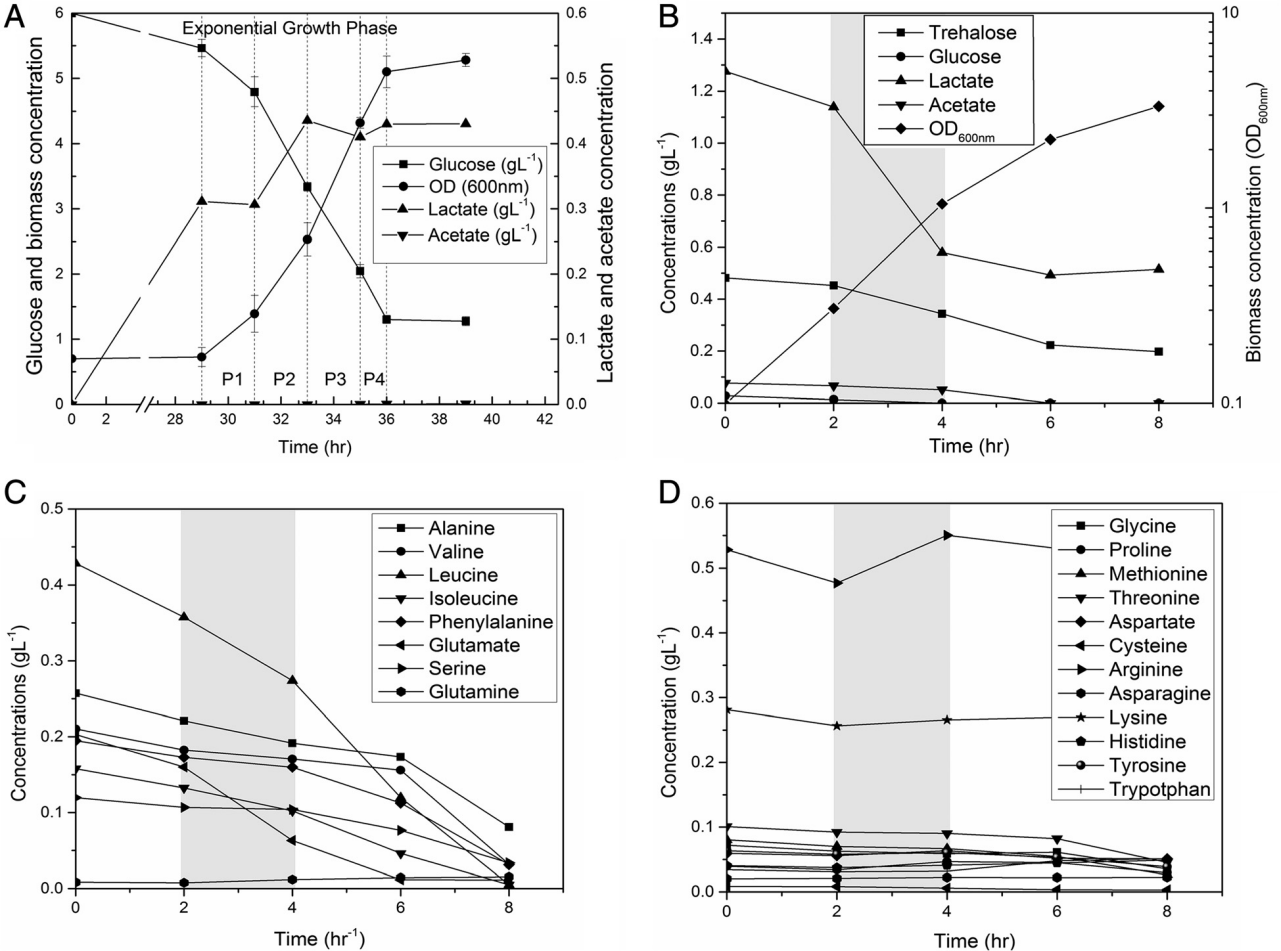
**Batch fermentation profile of optical density and various nutrients in glucose minimal and complex medium. (A)** Profiles of optical density and residual concentrations of the glucose, acetate and lactate in glucose minimal medium, **(B)** optical density and residual concentrations of glucose, trehalose, lactate and acetate in complex medium, **(C)** concentrations of amino acids which were rapidly consumed in complex media and **(D)** amino acids which were not completely consumed. Highlighted regions correspond to exponential growth phases of the cultures and the corresponding nutrient consumption/secretion profiles were used for *in silico* simulations.

We validated *i*TT548 using data from the batch cultures of *T. thermophilus* growing in glucose minimal and complex media. In case of glucose minimal medium, cells were cultured in a DMM containing 0.6% (w/v) glucose at 70°C. The residual concentration of glucose and the cell density were monitored (Figure 3A). Initially, a prolonged lag phase of 2 d was observed, followed by an exponential growth phase of 8 h before the stationary phase was reached. The cell cultures also indicated the presence of acetate, lactate and ethanol in trace amounts during lag and exponential growth phases. In order to analyse the growth behaviour of *T. thermophilus* in a rich medium, the cells were grown in a complex TM medium at 70°C. It should be noted that this medium was supplemented with all 20 amino acids. The nutrient consumption profiles indicated that glucose was consumed first. Subsequently, other carbohydrates such as trehalose and amino acids were assimilated (Figure 3B). Notably, cells did not consume all the amino acid supplemented in the medium and preferred branched chain amino acids ahead of other amino acids, possibly to synthesize the branched chain fatty acids (Figures 3C and D). Furthermore, the complex medium did not show any appreciable lag phase and the cells grew almost twice as fast as the minimal media.

**Figure 4:**
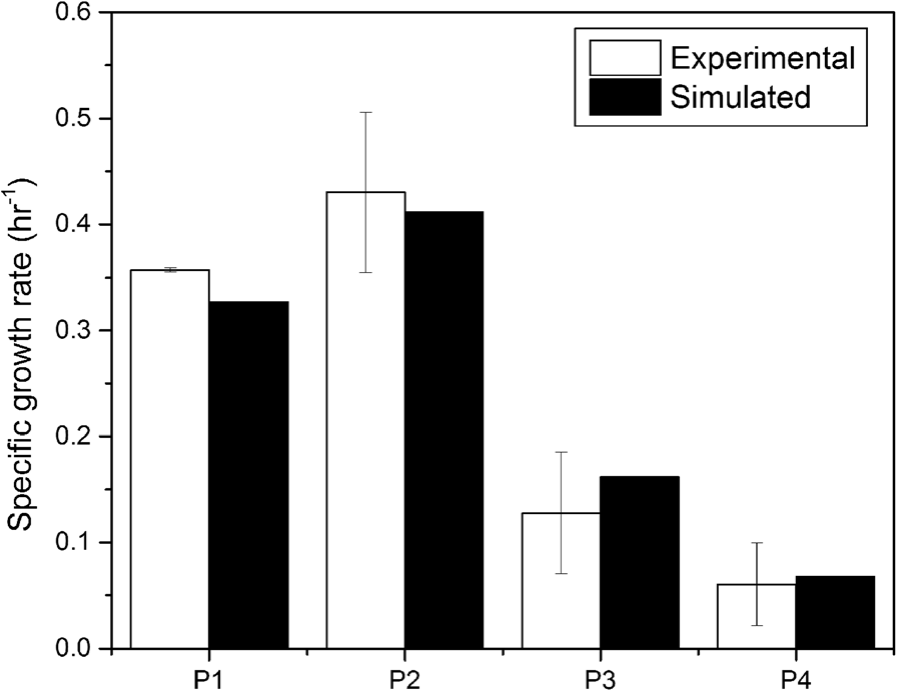
**Comparison of *in silico *** **growth rate with experimentally observed growth rate during exponential phase of the cell culture in glucose minimal medium.**

To simulate the cellular growth in the minimal medium, the biomass equation was maximized in the flux analysis simulations while simultaneously constraining the glucose uptake rates measured during the exponential phase based on the assumption that wild-type organisms typically evolve towards the maximization of cellular growth during exponential phase [38]. The exchange fluxes of NH_3_, phosphate, sulphite, H_2_O, Fe^2+^, Mg and H^+^ were left unconstrained to provide basic nutrients and minerals for cell growth. The oxygen uptake rate was constrained at the average specific uptake rate of 10 mmol g^-1^ DCW hr^-1^ based on previous publication [39]. Additionally, the lactate exchange flux was also constrained at the measured uptake/secretion rates in each phase. A growth associated maintenance (GAM) value of 58.34 mmol g^-1^ DCW h^-1^, from *E. coli* GSMM, and a NGAM requirement of 14 mmol g^-1^ DCW h^-1^, calculated based on established methods [14], were also used for the simulations (see Additional file 3 for detailed calculation of NGAM calculations). Here, it should be noted that while comparing the NGAM requirement of *T. thermophilus* with *E. coli*[14], it possess a very high value (14 compared to that of 8 mmol g^-1^ DCW h^-1^) possibly due to the differences in H^+^ permeability of their cytoplasmic membrane at optimal growth temperature; *T. thermophilus* cytoplasmic membrane is highly permeable to H^+^, and thus, leaks protons without ATP synthesis via ATPase [40]. Notably, simulation results were highly consistent with observed growth rates (Figure 4). For complex media, once again the biomass equation was maximized while simultaneously constraining the uptake/secretion rates of all nutrients (glucose, trehalose, lactate, acetate and amino acids) during the exponential growth phase. The *in silico* predicted cell growth of 0.66 h^-1^ was very close (within the acceptable error range of 10%) to the experimentally observed specific growth rate of 0.64 h^-1^, thus clearly indicating the high predictive ability of *i*TT548 even in complex media.

### *In silico* comparative metabolic flux analysis of minimal and complex media

When comparing the cellular growth rates of *T. thermophilus* in minimal and complex media, not surprisingly, it was significantly higher in the latter owing to the availability of rich carbon and nitrogen sources in the form of various amino acids. Since the microbe can take up some of the amino acids directly from the medium, it could be possible that the protein biosynthetic demand can be partially fulfilled. In order to confirm such hypothesis, we compared the simulated metabolic fluxes between minimal (phase P1) and complex media, and observed much lower fluxes through the relevant biosynthetic reactions of amino acids in the complex medium (Table 2). Herein, it should be noted that we also conducted the flux variability analysis [41] to re-assure the confidence of the simulated metabolic states in each environmental condition.

**Table 2:** Comparison of metabolic reaction fluxes of amino acids biosynthetic reactions between minimal and complex media

**Reaction ID**	**Enzyme name (Gene)**	**Reaction details**	**Complex media**	**Minimal media**
			**(LB < L < UB)**	**(LB < L < UB)**
**R192**	**Alanine dehydrogenase (*ald*)**	**H[c] + NADH[c] + PYR[c] + NH4[c] < = > L_ALA[c] + NAD[c] + H2O[c]**	**-0.031 < -0.031 < -0.031**	**0.234 < 0.234 < 2.416**
R521	Asparagine synthase (*asnB*)	L_ASP[c] + L_GLN[c] + ATP[c] + H2O[c] < = > L_GLU[c] + L_ASN[c] + PPi[c] + H[c] + AMP[c]	-1.808 < -1.808 < -1.783	-0.537 < -0.537 < -0.522
R198	Aspartate aminotransferase (*aspC*)	L_ASP[c] + AKG[c] < = > L_GLU[c] + OAC[c]	-1.782 < -1.782 < -1.782	-0.416 < -0.416 < -0.416
R205	Glutamine synthetase (*gln*)	L_GLU[c] + ATP[c] + NH4[c] - > L_GLN[c] + ADP[c] + Pi[c] + H[c]	-1.793 < -1.793 < -1.793	-0.474 < -0.474 < -0.474
R202	Glutamate dehydrogenase (*gdh*)	L_GLU[c] + NADP[c] + H2O[c] < = > H[c] + NADPH[c] + AKG[c] + NH4[c]	-9.830 < -9.145 < 4.713	-9.288 < -8.813 < 2.977
**R227**	**Cysteine synthase (*cysM*)**	**OAC_SER[c] + H2S[c] - > L_CYS[c] + H[c] + AC[c]**	**0 < 0 < 0**	**0 < 0.005 < 0.005**
**R235**	**Methionine synthase (*metH*)**	**L_HCYS[c] + 5MTHF[c] - > L_MET[c] + H[c] + THF[c]**	**0 < 0 < 0**	**0 < 0.009 < 0.009**
R272	N2-acetyl-L-lysine deacetylase (l*ysK*)	H2O[c] + N2AC_L_LYS[c] - > L_LYS[c] + AC[c]	0.229 < 0.229 < 0.229	0.05 < 0.05 < 0.05
R651	3-phosphoserine phosphatase (*serB*)	H2O[c] + PPTD_SER[c] - > L_SER[c] + Pi[c]	0 < 0 < 0	0 < 1.072 < 2.545
R655	Serine hydroxymethyltransferase (*glyA*)	L_SER[c] + THF[c] - > L_GLY[c] + H2O[c] + METHF[c]	0 < 0 < 0	0 < 0.512 < 6.42
R213	Serine hydroxymethyltransferase (*glyA*)	L_GLY[c] + H2O[c] + 5_10_MNTHF[c] < = > L_SER[c] + THF[c]	0.135 < 0.135 < 0.135	-1.787 < -0.442 < 3.993
R212	Tryptophan synthase (*trpA*)	L_SER[c] + IND_GLYP[c] < = > L_TRP[c] + H2O[c] + GAP[c]	0.054 < 0.054 < 0.054	0.026 < 0.026 < 0.026
R221	Threonine synthase (*thrC*)	H2O[c] + OP_HSER[c] - > L_THR[c] + Pi[c]	0.0714 < 0.0714 < 0.0714	0.063 < 0.063 < 0.063
**R245**	**Branched-chain amino acid aminotransferase (*ilvE*)**	**L_VAL[c] + AKG[c] < = > L_GLU[c] + 2K3M_BUT[c]**	**0.113 < 0.113 < 0.113**	**-0.068 < -0.068 < -0.068**
**R244**	**Branched-chain amino acid aminotransferase (*ilvE*)**	**L_GLU[c] + 4M_2OPENTN[c] < = > L_LEU[c] + AKG[c]**	**-1.179 < -1.179 < -1.179**	**0.154 < 0.154 < 0.154**
**R246**	**Branched-chain amino acid aminotransferase (*ilvE*)**	**L_ILE[c] + AKG[c] < = > L_GLU[c] + 3M_2OPENTN[c]**	**0.478 < 0.478 < 0.478**	**-0.024 < -0.024 < -0.024**
R341	Pyrroline-5-carboxylate reductase (*ProC*)	L_PRO[c] + NADP[c] < = > 2 H[c] + NADPH[c] + PYRR5CARB[c]	-0.432 < -0.432 < -0.432	-0.119 < -0.119 < -0.119
R195	Argininosuccinate lyase (*argH*)	L_ARG_SUCC[c] - > L_ARG[c] + FUM[c]	1.144 < 1.144 < 1.144	0.132 < 0.132 < 0.132
**R297**	**Aspartate aminotransferase (*aat*)**	**L_PHE[c] + AKG[c] < = > L_GLU[c] + PPYR[c]**	**0 < 0 < 0**	**-0.054 < -0.054 < -0.054**
**R284**	Histidinol dehydrogenase (*hisD*)	2 NAD[c] + H2O[c] + L_HISN[c] - > L_HIS[c] + 3 H[c] + 2 NADH[c]	0.025 < 0.025 < 0.025	0.015 < 0.015 < 0.015

The reactions which are highlighted in bold possess less flux in complex media. FVA was conducted to re-assure the simulated fluxes and the results are provided with the attainable lower and upper bounds identified from this analysis.

Interestingly, although all 20 amino acids were supplemented in the complex medium, the cells consumed only a few of them preferably. In order to understand this cellular behavior, we compared the uptake rate of individual amino acids with their actual biosynthetic demands. Methionine and cysteine were consumed from the medium according to their biosynthetic demands while alanine, valine, leucine, isoleucine and glutamate were consumed in excess of their individual biosynthetic requirements (Table 3). Further analysis revealed that the surplus valine/leucine/isoleucine and alanine/glutamate were utilized to synthesize the branched chain fatty acids and other amino acids, respectively. Notably, leucine was consumed much more than its biosynthetic demand (almost 7 times higher) mainly to synthesize iso-15 and iso-17 fatty acids, which constitute the major composition in *T. thermophilus*. Remarkably, this amino acid utilization pattern is completely different from *E. coli* grown in complex media, which consumed serine, aspartate, glycine and threonine ahead of other amino acids [42]. The subsequent *in silico* analysis in *E. coli* revealed that the rapidly depleted serine was primarily converted into pyruvate via serine deaminase, and then to acetate for producing more ATP as similarly observed in several other microbes such as *C. glutamicum* and *Lactococcus lactis*. In this regard, it is interesting to note that *T. thermophilus* possess unique nutrient consumption pattern, preferably synthesizing branched chain fatty acids rather than improving energy production further. Overall, as the *in silico* analysis highlights that all consumed amino acids contribute either directly or indirectly towards biomass synthesis, strategies to increase the uptake of non-consumed amino acids such as tyrosine, lysine, tryptophan and histidine can be postulated to enhance the cellular growth in complex medium.

**Table 3:** Consumption or production pattern of amino acids in complex media

**Amino acids**	**Supply from medium**	**Biosynthetic demand**	**Contribution**		
			**To others**	**From others**	**Remarks**
Alanine	0.7399	0.355	0.3853		Surplus alanine contributes to glycine synthesis
Glycine	0.116	0.308		0.1918	Some are produced from alanine
Valine	0.224	0.109	0.1151		Excess valine is utilized in synthesis of anteiso-17:1 and anteiso-15:0 fatty acids
Leucine	1.427	0.244	1.1827		Excess leucine is utilized in iso-15:0 and iso-17:0 fatty acids synthesis
Isoleucine	0.5181	0.039	0.4794		Excess isoleucine is utilized in synthesis of anteiso fatty acid synthesis
Proline	-0.24	0.189		0.4291	Synthesized from glutamate
Methionine	0.015	0.015			Utilized from media as per biosynthetic demand
Serine	0.057	0.135		0.0785	Synthesized from glycine
Threonine	0.03	0.100		0.0699	Synthesized from aspartate
Phenylalanine	0.086	0.086			Utilized from media as per biosynthetic demand
Aspartate	-0.12	0.103		0.2232	Synthesized from oxaloacetate and glutamate
Cysteine	0.0087	0.009			Utilized from media as per biosynthetic demand
Glutamate	1.48	0.176	1.3041		Excess glutamate is utilized in several other amino acid synthesis
Arginine	-0.93	0.211		1.1409	Synthesized from aspartate
Asparagine	-0.023	0.103		0.1262	Synthesized from aspartate
Lysine	-0.141	0.087		0.2283	Synthesized from oxoglutarate
Glutamine	0	0.177		0.1771	Synthesized from glutamate
Histidine	0	0.024		0.0245	Synthetsized from PRPP
Tyrosine	-0.03204	0.069		0.1007	Synthesized from PEP, E4P
Tryptophan	-0.01116	0.043		0.0538	Synthesized from serine

### Analysis of essential genes *T. thermophilus*

**Figure 5:**
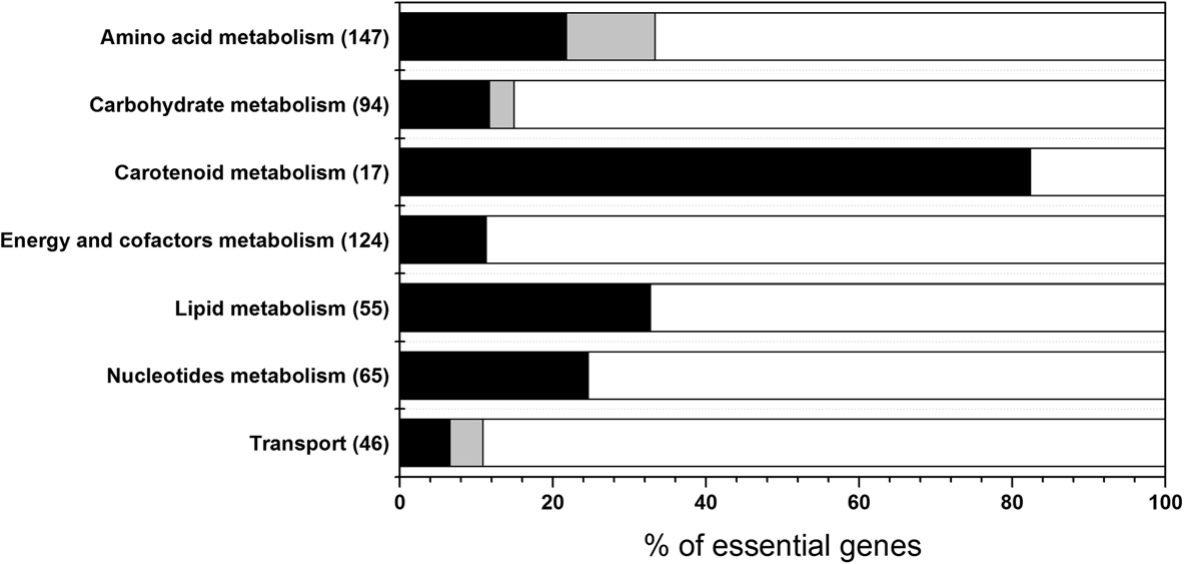
**Distribution of essential genes in *T. thermophilus *metabolic subsystems.** Black, grey and white colors indicate the completely-, conditionally- and non-essential genes, respectively. The numbers within the parenthesis represent the number of genes in each subsystem.

We analysed the essentiality of individual genes of the *T. thermophilus* under glucose minimal and complex TT medium conditions using *i*TT548 model by deleting every gene one at a time (see Methods). The genes were then categorized into three classes: (i) completely essential – genes which are required for cellular growth in both the media, (ii) conditionally essential – required only in one of the media and (iii) non-essential – dispensable in both the media. The gene essentiality study revealed that 23.5% and 19.5% of the total 548 genes in *i*TT548 are essential for cell growth in minimal and complex media, respectively (see Additional file 4 for complete list of essential genes). A total of 107 genes were essential in both conditions and an additional 21 genes were essential only in minimal media. In order to further understand the knockout of which functional category of genes are more crucial for cell viability in either condition, we identified the distribution of essential genes across various metabolic processes (Figure 5). Interestingly, the carotenoids metabolism contained most of the lethal genes (82%), suggesting that this unique pathway synthesizing the thermozeaxanthin and thermobiszeaxanthin does not have many alternative routes and is quite rigid in *T. thermophilus*. Following carotenoids metabolism, the nucleotides and lipids metabolism has second and third highest number of completely essential genes (32.7% and 24.6%, respectively). On the other hand, examination of conditionally essential genes, i.e. genes which are essential only in minimal media, revealed that amino acid metabolism contains almost all such genes (Figure 5). Since the complex media is supplemented with all the amino acids, some of them were directly consumed from the media without utilizing their biosynthetic pathways, thus classifying the genes from those pathways as non-essential under such conditions. However, at the same time, distribution of certain completely essential genes in amino acid metabolism also indicates that biosynthetic pathways of tryptophan, proline and tyrosine are crucial for the cell growth since they should be synthesized within the cell albeit their availability in the complex medium. Interestingly, our gene deletion analyses also showed a high number of completely essential genes within the oxidative phosphorylation and TCA cycle. Oxygen is the key electron acceptor in cellular metabolism which can accept electrons from other redox cofactors involved in the TCA cycle and generates energy through oxidative phosphorylation. If oxygen is devoid or any of the oxidative phosphorylation and TCA cycle reactions are perturbed, generally, most of the bacteria regenerate the redox cofactors by switching to fermentative growth with the help of substrate level phosphorylation. In this regard, since *T. thermophilus* lacks the PTS and utilizes ABC transporters even for glucose uptake, it could be possible that the microbe cannot switch to fermentative metabolism completely and thus, requires oxygen to generate sufficient energy for cell growth via TCA cycle and oxidative phosphorylation.

## Discussion

Thermophilic microbes represent a unique class of organisms with a distinct cell wall assembly that enables them to maintain the cellular membrane integrity even at very high temperatures. It is reported that many thermophilic organisms, including *T. maritima* and *S. solfataricus*, synthesize ether lipids from long chain dicarboxylic fatty acids and fatty alcohols [43]. However, *Thermus* sp. do not synthesize ether lipids but produce unique carotenoid molecules such as thermozeaxanthin and thermobizeaxanthin, and embed them in the lipid bi-layer to attain the required cellular membrane fluidity at high temperatures [10]. In this regard, *i*TT548 completely captures all the biosynthetic pathways of thermozeaxanthins, in addition to the metabolic routes of other biomass precursors such as amino acids, nucleotides and lipids. Similarly, *i*TT548 also contains the unique biosynthetic pathways of several unusual polyamines which help in stabilizing the nucleotide strands and proteins synthesis at high temperatures. It has been earlier reported that *T. thermophilus* is unique in polyamine synthesis: even the extreme thermophiles such as *S. solfataricus* produces relatively shorter polyamines [36]. Collectively, these results clearly show the detailed metabolic coverage of *i*TT548 of thermophiles when comparing with its preceding GSMMs.

**Figure 6:**
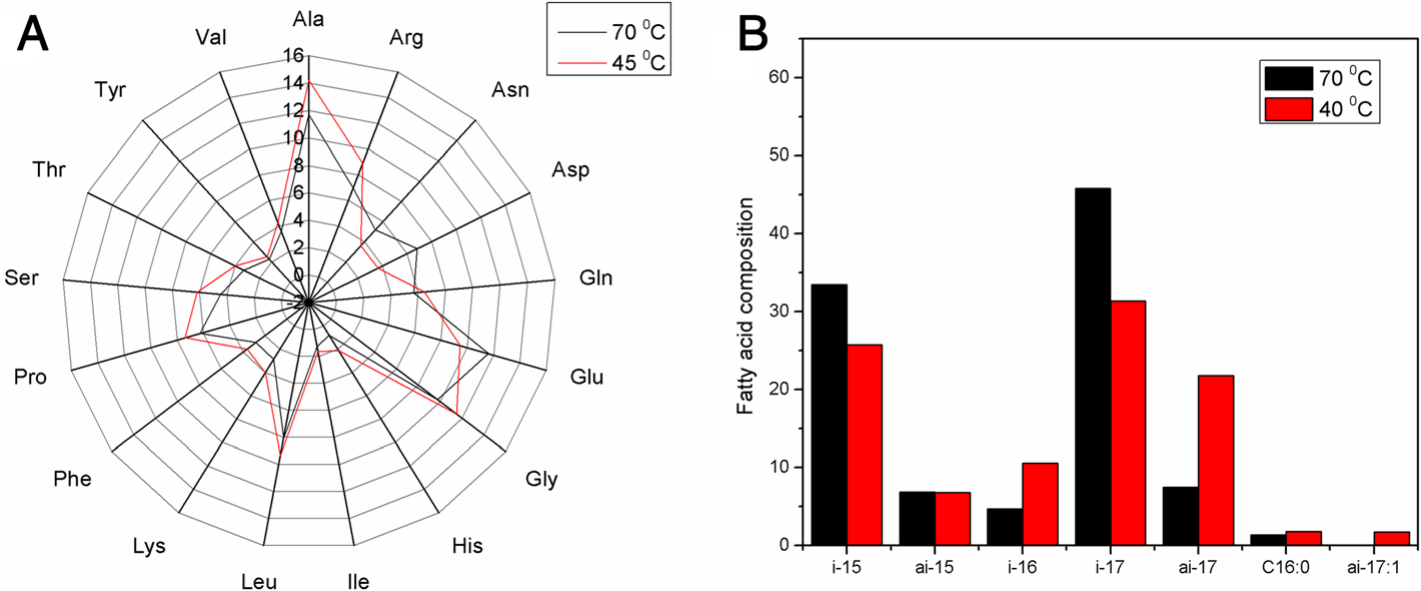
**Influence of temperature on *T. thermophilus *biomass composition. (A)** amino acid composition (mol%) at 70°C and 45°C and **(B)** fatty acid composition (mol%) at 70°C and 40°C.

Furthermore, this work includes a prudently drafted biomass equation that is specific to thermophiles, especially *Thermus* sp. As mentioned earlier, the comparative analysis of *T. thermophilus* and *E. coli* biomass compositions have highlighted significant differences between amino acid and fatty acid compositions. Noticeably, the *T. thermophilus* biomass analysis revealed that the concentration of some of the thermolabile amino acids such as threonine and histidine are substantially lesser than *E. coli* whereas the proline concentration is much higher. It should be highlighted that these observations are in good agreement with earlier reports which suggested the selective usage of amino acid residues as one of the key adaptive strategy employed by thermophiles [44,45]. Arguably, the cellular compositions in thermophilic microbes may change depending on growth temperature; the current biomass equation was derived based on compositional analysis of *T. thermophilus* grown at 70°C. In order to clarify the temperature dependent compositional change in biomass, we measured amino acid compositions in *T. thermophilus* at 45°C. Their comparison with compositional data at 70°C clearly indicated that there is no significant difference in both individual and overall amino acid concentrations (Figure 6A). Similarly, we also compared the fatty acid compositions between 40°C and 70°C using the data from literature [30]. Very interestingly, unlike amino acid comparison, fatty acid compositions, both overall and individual were much lower at 40°C (Figure 6B). Although we were not able to make a complete comparison between low and high growth temperatures since no data was available on other cellular constituents such as peptidoglycans and thermotolerant carotenoids at low temperature range, we can still hypothesize that thermophiles are most likely to adjust their biomass composition selectively to better adapt to the growth environment. Therefore, the use of appropriate biomass equations for simulating the cellular growth in corresponding temperature ranges is crucial for reliable prediction.

The gene deletion analyses of *i*TT548 have revealed several interesting traits about the function of deleted genes with respect to overall cellular metabolism of *T. thermophilus*. Among them, the most notable is the relatively high percentage of essential genes when compared to *E. coli* (23% to that of 13%), possibly due to the smaller Open Reading Frames (ORF) content (only 2,263 as compared to 4,623) despite possessing all the necessary modules for the cell to be viable at high temperatures. Furthermore, this observation also highlights the fact that since *T. thermophilus* thrives at higher temperatures than most other microbes, its fitness might be relatively less competitive with more rigid network organization. Interestingly, the gene essentiality analyses also indicated that the carotenoids metabolism is functionally quite fragile since almost all of its genes are essential for cellular growth. However, it should be noted that the gene deletion analysis results are sensitive to several parameters such as *in silico* medium setup and biomass composition. In this regard, the current biomass composition is obtain from *T. thermophilus* at optimum growth temperatures, i.e. 70°C, and thus the gene deletion results of the current study are only applicable to this condition.

## Conclusions

We presented the genome-scale metabolic network of *T. thermophilus*, *i*TT548, the first ever representing thermophiles, containing 548 unique genes, 796 reactions and 635 unique metabolites. As a unique feature of *T. thermophilus*, *i*TT548 contains necessary metabolic pathways for synthesizing several unique carotenoids and polyamines which help them in habituating high temperatures. The reconstructed metabolic model was subsequently validated with the batch culture experiments on glucose minimal and complex medium where the *in silico* growth predictions of the *i*TT548 were in good agreement with the observed experimental results. The comparative flux analysis between minimal and complex media highlighted that the consumption and utilization of branched chain amino acids directly in the relevant fatty acids anabolic pathways, thus resulting in higher growth rates in the rich medium. A gene essentiality study was also conducted through *in silico* simulation studies in both minimal and complex media, highlighting a very high percentage of lethal genes in comparison with *E. coli*, suggesting that the metabolic backbone of *T. thermophilus* could to be quite rigid. Overall, the metabolic network presented in the current study is expected to be a significant contribution towards systems analysis of thermophiles where the metabolic model can be utilized along with high throughput datasets for the better understanding of organism.

## Methods

### Microorganism and culture conditions

*T. thermophilus* HB27 strain was used as a model organism. For fermentation in complex medium, a single colony was cultivated overnight at 70°C with 150 rpm in 5 mL of the TM medium [46], and the culture was transferred to a 500 mL baffled-flask containing 100 mL of TM broth. In case of cultivation in defined glucose minimal medium, the overnight seed grown at 70°C with 150 rpm in 5 mL of the TM medium was then transferred to a 500 mL baffled-flask containing 100 mL of defined minimal medium (DMM) with 0.6% (w/v) glucose and cultivated for 24 hours. Then, 5 mL of flask culture in DMM was inoculated to 100 mL of fresh DMM. During fermentations, cell growth was monitored by measuring the optical density at 600 nm. The dry cell weight (DCW) was then estimated by a predetermined conversion factor of 0.34.

### Analytical methods

Concentrations of glucose, organic acids and ethanol in the culture broth were measured by high performance liquid chromatography (HPLC) (Waters, Milford, MA) equipped with an HPX-87H column (Bio-Rad, Hercules, CA), a dual λ absorbance detector. The collected samples were centrifuged at 14,000 *g* and 4°C for 5 min and the supernatant was analyzed with the column using 5 mM sulfuric acid as a mobile phase at 0.6 mL^-1^ min. Concentrations of amino acids were determined by gas chromatograph/mass spectrophotometer (GC/MS) (Agilent, Santa Ciara, CA) equipped with an HP-5MS column (Agilent), as previously reported [47]. In brief, the samples were centrifuged, dried and derivatized with methyl-N-t-butyldimethylsilyl-trifluoro-acetamide (MBDSTFA) in DMF at 80°C for 30 min. After centrifugation at 14,000 g for 5 min, the supernatant was injected to GC/MS in split injection mode (1:10 split ratio).

### Metabolic network reconstruction

The genome-scale metabolic network of *T. thermophilus* HB27 was reconstructed using the published genome annotation [28] and the information collected from various biological and genomic databases on the basis of the established procedure [48]. First, an initial draft model was constructed by compiling the annotated metabolic genes and their corresponding biochemical reactions from KEGG [49] and MetaCyc [50]. Then, these reactions were corrected for any elemental imbalances and mapped with appropriate genes to devise proper gene-protein-reaction (GPR) relationships. Additionally, some spontaneous as well as non-gene-associated reactions including metabolite transport were also incorporated into the model based on the physiological evidence from literature and databases. The connectivity of the draft network was then checked using the GapFind algorithm to find the gaps [51]. The identified missing links were filled either by introduction of sink reactions to allow for material exchange between the cell and its surrounding environment or by adding reactions from other similar microbes to close the knowledge gaps.

### Biomass composition

Cellular biomass composition is an important prerequisite for the *in silico* flux analysis, especially during the exponential growth phase, where the primary cellular objective is to maximize growth. Amino acid composition of *T. thermophilus* HB27 was estimated by hydrolyzing the cell pellets with 6 N HCl for 24 h at 130°C, and subsequently analysing the hydrolysates using HPLC equipped with UV-detector and C18 column. Cell wall and lipid compositions were obtained from previous publications on *Thermus* sp. [30,31,35,52]. The overall DNA and RNA composition was assumed to be same as *E. coli*[14] since no data was available on *Thermus* sp. The individual weights of nucleotides in the DNA and RNA were calculated based on the reported G + C content of 69.4% [28]. Detailed information on biomass composition calculations could be found in Additional file 3.

### Constraints-based flux analysis

We implemented constraints-based flux analysis to simulate the *T. thermophilus* metabolism under varying environmental conditions. The biomass reaction was maximized to simulate the exponential growth phase as described elsewhere [53-55]. Mathematically, the optimization problem, i.e. maximization of biomass subjected to stoichiometric and capacity constraints, can be formulated as follows:

$$\text{max}Z=\sum_{j}c_{j}v_{j}$$

$$s.t.\;\sum_{j}S_{\mathrm{ij}}v_{j}=0\;\forall \;\mathrm{metabolite}\;i$$

$$v_{j}^{\text{min}}\leq v_{j}\leq v_{j}^{\text{max}}\;\forall \;\mathrm{reaction}\;j$$

where *S*_*ij*_ refers to the stoichiometric coefficient of metabolite *i* involved in reaction *j*, *v*_*j*_ denotes to the flux or specific rate of metabolic reaction *j*, $v_{j}^{\text{min}}$ and $v_{j}^{\text{max}}$ represent the lower and upper limits on the flux of reaction *j*, respectively; and *Z* corresponds to the cellular objective as a linear function of all the metabolic reactions where the relative weights are determined by the coefficient *c*_*j*_. In this study, the constraints-based flux analysis problems were solved using COBRA toolbox [56].

### Flux variability analysis

As constraints-based flux analysis is an optimization based technique, it is often possible to have multiple flux distributions attaining the same physiological state. Therefore, in order to confirm the plausibility of internal metabolic fluxes simulated in minimal and complex media by flux analysis, we performed the flux variability analysis (FVA) to identify the possible range of all fluxes while simulating a particular phenotypic state. Mathematically, the optimization problem specific to FVA can be represented as follows:

$$\begin{array}{l} \text{max}/\text{min}\;v_{j}\\ \;s.t.\;\sum_{j}S_{\mathrm{ij}}v_{j}=0\\ \;\sum_{j}c_{j}v_{j}=Z_{\mathrm{obj}}\\ \;v_{j}^{\text{min}}\leq v_{j}\leq v_{j}^{\text{max}}\;\mathrm{for}\;j=1,\ldots ,n \end{array}$$

where *Z*_*obj*_ denotes the value of objective calculated by flux analysis and *n* is the number of fluxes. The upper range of fluxes is identified by maximizing the objective whereas the lower range is obtained by minimizing the same. In this study, the FVA was implemented using COBRA toolbox.

### Gene deletion analysis

Gene deletion simulations were performed by maximizing the cellular biomass while constraining flux through the corresponding reaction(s) to be zero via the GPR relationships under defined nutrient uptake rates. In case of glucose minimal medium, only glucose was fueled as carbon source. On the other hand, glucose, trehalose and amino acids such as valine, leucine and isoleucine were supplied as carbon source based on the complex media based on nutrient consumption profile. The simulation results were subsequently analyzed to identify the essential genes where a gene is classified to be essential if the resulting cell growth prediction for the corresponding mutant is less than or equal to 5% of wild-type. Note that all the gene deletion analysis in this study was performed using COBRA toolbox.

## Competing interests

The authors declare that they have no competing interests.

## Authors’ contributions

NRL, ML, JBP and DYL conceived and designed the study. NRL and JWS performed the batch culture experiments. NRL and SA created the draft model. ML refined the model and performed simulations. NRL, ML, IAK, JBP and DYL wrote the manuscript. JBP and DYL coordinated and directed the project. All authors have read and approved the final manuscript.

## Supplementary Material

Additional file 1 **Details of *i*TT548 containing all genes, reactions, metabolites.** A list of reactions added during gap-filling and possible new annotations identified in this study are also provided. Click here for file

Additional file 2 **SBML file of *i*** **TT548.** Click here for file

Additional file 3 **Biomass composition of *T. thermophilus *** **HB27 and NGAM calculations.** Click here for file

Additional file 4 List of essential genes in glucose minimal and complex media. Click here for file
