# Supplementary material for: Genome-scale metabolic network reconstruction and in silico flux analysis of the thermophilic bacterium Thermus thermophilus HB27
Source: Microb Cell Fact. 2014 Apr 28;13:61. doi: 10.1186/1475-2859-13-61 (PMC4021367; doi:10.1186/1475-2859-13-61)
Supplement: Additional file 3 — Biomass composition of T. thermophilus HB27 and NGAM calculations. [file 1475-2859-13-61-S3.pdf]

Additional File 3: Biomass Composition and NGAM calculations

Overall Composition

Overall cellular composition data were obtained from literature sources and HPLC analysis. The nucleotide composition was taken from *Escherichia coli i*AF1260 model [1].

Table 1. Cellular composition

Components	Weight percentage	Source
Protein	49.4500	HPLC analysis and literature
Lipid	18.0000	[2]
Petidoglycan	1.2500	[3]
Glycogen	3.0000	[4]
DNA	3.1000	Assumed same as *E. coli*
RNA	21.0000	Assumed same as *E. coli*
Soluble Pool	3.8000	Assumed same as *Z. mobilis*
Total	99.6000	

Protein Composition

Protein composition was estimated HPLC analysis and literature source.

Table 2. Protein composition

Amino acids	mg/100 gDCW	MW (g/mol)	mmol/gDCW	Source
L_ala	4841.2408	89.0900	**0.5434**	HPLC analysis
L_arg	5628.5887	174.2000	**0.3231**	HPLC analysis
L_asn	2105.5200	132.1170	**0.1594**	HPLC analysis
L_asp	2105.5200	133.1000	**0.1582**	HPLC analysis
L_cys	162.3544	121.1600	**0.0134**	[4]
L_gln	3966.9700	146.1400	**0.2714**	HPLC analysis
L_glu	3966.9700	147.1300	**0.2696**	HPLC analysis
L_gly	3396.4233	75.0700	**0.4524**	HPLC analysis
L_his	581.9890	155.1500	**0.0375**	HPLC analysis
L_ile	777.2783	131.1700	**0.0593**	HPLC analysis
L_leu	4911.4870	131.1700	**0.3744**	HPLC analysis
L_lys	1954.5421	146.1900	**0.1337**	HPLC analysis
L_met	334.2304	149.2100	**0.0224**	[4]

L_phe	2184.5943	165.1900	**0.1322**	HPLC analysis
L_pro	3336.5369	115.1300	**0.2898**	HPLC analysis
L_ser	2181.8860	105.0900	**0.2076**	HPLC analysis
L_thr	1823.6985	119.1200	**0.1531**	HPLC analysis
L_trp	1335.6315	204.2250	**0.0654**	[4]
L_tyr	1905.3602	181.1900	**0.1052**	HPLC analysis
L_val	1953.9112	117.1500	**0.1668**	HPLC analysis
Total	49454.7326		**3.9384**	

DNA Composition

According to the genome sequence, G/C content of *T. thermophilus* is 69.4%. This value is then used to derive the ratio of the nucleic acids in the DNA. The molecular weight given in the following table is the weight if the nucleotide monophosphate minus the weight of water molecule, which is lost during esterification.

Table 3. DNA composition

Nucleotide	DNA (mol/mol)	MW (g/mmol)	DNA (g/mol)	DNA (g/g)	g/gDCW	mmol/ gDCW
dATP	0.153	487.151	74.5341	0.1545	0.0048	**0.0098**
dCTP	0.347	461.109	160.0048	0.3318	0.0103	**0.0223**
dTTP	0.153	478.136	73.1548	0.1517	0.0047	**0.0098**
dGTP	0.347	503.15	174.5931	0.3620	0.0112	**0.0223**
Total			482.2868			**0.0643**

RNA Composition

We assumed that RNA consisted of 5% mRNA, 81% rRNA, 14% tRNA taken from total composition of *E. coli*. The nucleotide comositon of mRNA was considered similar with genomic DNA. The nucleotide composition of rRNA and tRNA was calculated from the sequences of rRNA and tRNA in TIGR database. As the case for DNA, the molecular weight given in the following table is the weight if the nucleotide monophosphate minus the weight of water molecule, which is lost during esterification.

Table 4. RNA composition

RNA	RNA (mol/mol)	MW (g/mmol)	RNA (g/mol)	RNA (g/g)	g/gDCW	mmol/ gDCW
ATP	0.262	503.1500	131.8253	0.2836	0.0596	**0.1184**
CTP	0.1297	479.1240	62.1424	0.1337	0.0281	**0.0586**
UTP	0.216	480.1080	103.7033	0.2231	0.0469	**0.0976**

GTP	0.322	519.1490	167.1660	0.3596	0.0755	**0.1455**
Total			464.8370			**0.4200**

Peptidoglycan composition

N-acetylmuramate and N-acetyl-D-glucosamine are arranged alternately to form the backbone of the peptidoglycan molecule. N-acetylmuramate molecules are cross-linked with a tail of four amino acids (L-Ala, D-Glu, DAP, D-Ala). Molecular weight presented in the following table excludes the weight of water to account for the bond formation. DAP are subtracted two mol of water to account for cross-linking.

Table 5. Peptidoglycan composition

Peptidoglycan	(mol/mol)	MW (g/mmol)	(g/mol)	(g/g)	g/gDCW	mmol/ gDCW	Source
GlcNAc	1.266	221.21	280.0519	0.1952	0.0024	**0.0110**	[5]
MurNAc	1.266	293.27	371.2798	0.2588	0.0032	**0.0110**	[5]
Orn	1.264	132.16	167.0502	0.1165	0.0015	**0.0110**	[5]
D-Ala	2.975	89.09	265.0428	0.1848	0.0023	**0.0259**	[5]
Gly	2.198	75.07	165.0039	0.1150	0.0014	**0.0192**	[5]
D-Glu	1.264	147.13	185.9723	0.1297	0.0016	**0.0110**	[5]
Total			1434.4009			**0.0892**	

Lipid composition

Table 6. Overall Lipid composition

Lipid	content (wt %)
Phospholipids	30.0000
Carotenoids	60.0000
MGDG	5.0000
DGDG	5.0000

Table 7. Fatty acids composition

Fatty Acids in Lipids	MW (g/mol)	content %(w/w)	Content (w/w)	Average MW calc	Content (mmol/gDCW)	mol fraction	Source
i-15	241.3901	31.5	0.315	76.0378815	**0.001304942**	0.334732005	[6]
ai-15	241.3901	6.5	0.065	15.6903565	**0.000269274**	0.069071684	[6]
i-16	255.4167	4.7	0.047	12.0045849	**0.000184013**	0.047201381	[6]
C16:0	255.4167	1.4	0.014	3.5758338	**5.48124E-05**	0.014059986	[6]
C16:1	253.4008	0.15	0.0015	0.3801012	**5.91948E-06**	0.001518411	[6]
i-17	267.4274	47.8	0.478	127.8302972	**0.001787401**	0.45848817	[6]
ai-17	270.4500	7.9000	0.0790	21.3656	**0.0003**	0.0749	[6]

Total			256.8846051	**0.003898467**

Table 8. Individual lipid composition

Lipid class	Content %(w/w)	MW of Backbone (g/mol)	# of FA	MW (g/mol)	mmol/gDCW	Source
PE	4.0000	181.2800	2.0000	181.28	**0.003107694**	[7]
PG	3.0000	212.1400	2.0000	212.14	**0.002231684**	[7]
PI	10.0000	300.2000	2.0000	300.2	**0.006634158**	[7]
CLPN	3.0000	332.1830	4.0000	332.183	**0.001191421**	[7]
PX	80.0000	298.1600	3.0000	298.1600	**0.040418639**	[7]
tZXT	100.0000	713.0000	1.0000	969.8846	**0.111353453**	[8]
MGDG	50.0000	308.2300	2.0000	308.23	**0.010948916**	[1]
DGDG	50.0000	470.3900	2.0000	470.3900	**0.009144862**	[1]

Small molecules pool composition

For simplification, it was assumed that the selected small molecules are equally presented (w/w) in the pool.

Table 9. Small molecules pool composition

Molecules	MW (g/mol)	Pool of small molecules (g/g)	Pool of small molecules (mmol/gDCW)
NAD	664.43335	0.111111111	**0.167**
NADP	744.4132	0.111111111	**0.149**
COA	767.5354	0.111111111	**0.145**
ACP	10689	0.111111111	**0.010**
PTRC	88.15156	0.111111111	**1.260**
SPMD	145.24599	0.111111111	**0.765**
THF	445.4295	0.111111111	**0.249**
FMN	456.34396	0.111111111	**0.243**
FAD	785.5501	0.111111111	**0.141**

Overall Biomass Equation

0.1582 L_ASP[c] + 0.2696 L_GLU[c] + 0.2076 L_SER[c] + 0.0375 L_HIS[c] + 0.4716 L_GLY[c] + 0.1531 L_THR[c] + 0.3231 L_ARG[c] + 0.5434 L_ALA[c] + 0.1052 L_TYR[c] + 0.1668 L_VAL[c] + 0.1322 L_PHE[c] + 0.0593 L_ILE[c] + 0.3744 L_LEU[c] + 0.1337 L_LYS[c] + 0.2898 L_PRO[c] + 0.1582 L_ASN[c] + 0.0134 L_CYS[c] + 0.2714 L_GLN[c] + 0.0224 L_MET[c] + 0.0654 L_TRP[c] + 0.0110 UDP_ACMURM[c] + 0.0110 UDP_AGLUAM[c] + 0.0110 D_GLU[c] + 0.0110 ORT[c] + 0.0259 D_ALA[c] + 0.0098 dTTP[c] + 0.0223 dGTP[c] + 0.0223 dCTP[c] + 0.0098 dATP[c] + 0.0586 CTP[c] + 0.1455 GTP[c] + 0.0976 UTP[c] + 0.01095 MGDG[c] + 0.0091 DGDG[c] + 0.1113 tZXT[c] + 0.0031 PETHA[c]

+ 0.0022 PGLY[c] + 0.0066 PINS[c] + 0.0012 CDLPN[c] + 0.0404 PX[c] + 59.81 ATP[c] + 0.001787 NAD[c] + 0.000576 CoA[c] + 0.149 NADP[c] + 0.000223 RBFLV[c] -> 59.81 ADP[c] + 59.81 Pi[c] + 0.3659 PPi[c]

NGAM calculation

In this study, we determined the NGAM requirement for our batch culture experiment using a conventional method of finding the y-intercept of the plot of glucose uptake rate against growth rate.

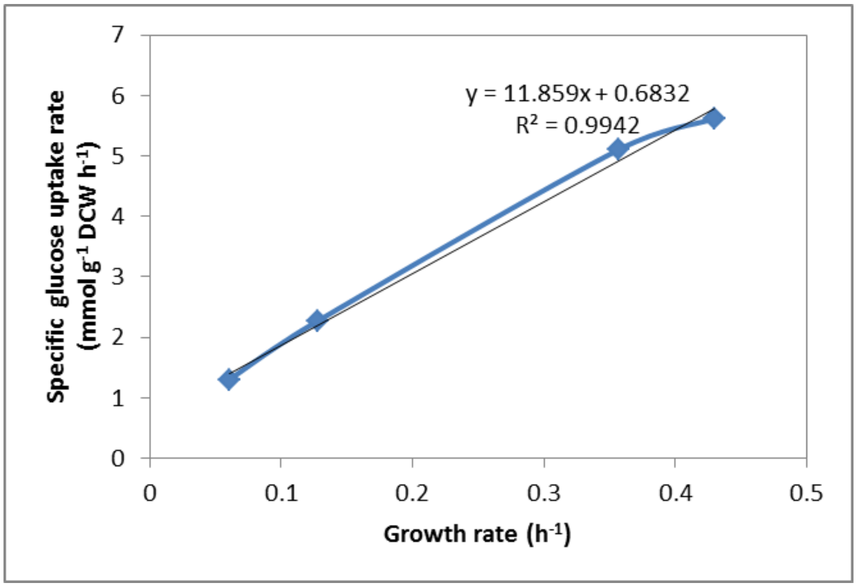

By maximizing ATP turnover under the glucose uptake constraint of 1 mmol/gDCW-hr, the ATP yield is evaluated as $Y_{ATP, max}$ = 20.92 mol ATP/ mol glucose. Using this value and the y-intercept (0.6832 mmol glucose/gDCW-hr), we can calculate the NGAM requirement to be about 14.29 mmol ATP/gDCW-hr.

REFERENCES

1. AM Feist et al. (2007) A genome-scale metabolic reconstruction for *Escherichia coli* K-12 MG1655 that accounts for 1260 ORFs and thermodynamic information, Mol Syst Biol. 2007; 3: 121
2. PH Ray et al. (1971) Effect of Growth Temperature on the Lipid Composition of Thermus aquaticus, J Bacteriol 108: 227-235
3. JC Quintela et al. (1995) Structure of peptidoglycan from Thermus thermophilus HB8, J Bacteriol 177: 4947-4962
4. B Boeck and R Schinzel (1998) Growth dependence of α-glucan phosphorylase activity in Thermus thermophilus, Res Microbiol 149: 171-176
5. Description of Thermus thermophilus (Yoshida and Oshima) comb. Bon., a Nonsporulating Thermophilic Bacterium from a Japanese Thermal Spa".

6. Structure of peptidoglycan from Thermus thermophilus HB8
7. Effect of growth temperature on fatty acid composition of ten thermus strains, Appl Environ Microbiol. 1992 May;58(5):1656-60.
8. PH Ray et al. (1971) Effect of Growth Temperature on the Lipid Composition of Thermus aquaticus, J Bacteriol 108: 227-235
9. Mandelli et al. (2012) Evaluation of biomass production, carotenoid level and antioxidant capacity produced by Thermus filiformis using fractional factorial design, Brazilian Journal of Microbiology 126-134
