# Supplementary material for: Genome-scale metabolic network reconstruction and in silico flux analysis of the thermophilic bacterium Thermus thermophilus HB27
Source: Microb Cell Fact. 2014 Apr 28;13:61. doi: 10.1186/1475-2859-13-61 (PMC4021367; doi:10.1186/1475-2859-13-61)
Supplement: Additional file 4 — List of essential genes in glucose minimal and complex media. [file 1475-2859-13-61-S4.pdf]

Additional File 4: Essential Genes

Completely essential (Essential in both the media)

TTC1592
TTC1089
TTC1090
TTC1091
TTC1092
TTC1896
TTC1898
TTC0932
TTC1408
TTC1409
TTC0561
TTC1798
TTC1930
TT_P0161
TT_P0162
TTC1197
TTC0427
TTC0247
TTC1706
TTC0426
TTC1380
TTC1381
TTC0089
TTC1243
TTC1701
TTC1702
TTC1960
TTC1533
TTC0729
TTC0730
TTC1756
TTC1757
TTC0238
TTC1768
TTC0534
TTC1550
TTC1012
TTC0043
TTC1396
TTC1866
TTC1080

TTC0801
TTC0062
TTC1079
TTC0061
TTC1652
TTC0370
TTC1280
TTC1944
TTC1546
TTC1547
TTC1393
TTC0838
TTC0500
TTC1020
TTC0989
TTC1019
TTC0088
TTC1492
TTC1493
TTC0800
TTC1929
TTC1491
TTC0017
TTC0691
TTC0721
TTC1854
TTC1378
TTC0983
TTC0815
TTC0505
TTC1614
TTC0504
TTC1815
TTC1816
TTC1438
TTC1677
TTC1983
TTC1986
TT_P0057
TT_P0066
TTC0447
TTC1543
TTC0769
TTC0770
TTC1671

TTC1672
TTC0905
TTC0906
TTC0907
TTC0908
TTC0909
TTC0910
TTC0911
TTC0912
TTC0260
TTC0616
TTC0688
TTC1463
TTC0048
TTC1678
TT_P0059
TT_P0062
TT_P0061
TTC0898
TTC1724
TTC1725

Conditionally essential (Essential only in minimal media)

TTC0978
TTC0374
TTC1172
TTC0117
TTC1028
TTC0115
TTC0177
TTC0166
TTC1636
TTC0407
TTC0253
TTC1870
TTC0867
TTC0850
TTC0871
TTC0851
TTC0852
TTC1656
TTC0447
TTC1875
TTC0328
