# Supplementary material for: Genome-scale metabolic network reconstruction and in silico flux analysis of the thermophilic bacterium Thermus thermophilus HB27
Source: Microb Cell Fact. 2014 Apr 28;13:61. doi: 10.1186/1475-2859-13-61 (PMC4021367; doi:10.1186/1475-2859-13-61)
Supplement: Additional file 2 — SBML file of i TT548. [file 1475-2859-13-61-S2.pdf]

Additional File 2: *i*TT548 model in sbml format


```
<?xml version="1.0" encoding="UTF-8"?>

<sbml xmlns="http://www.sbml.org/sbml/level2" level="2" version="1">

 <model>

  <listOfUnitDefinitions>

   <unitDefinition id="mmol_per_gDW_per_hr">

    <listOfUnits>

     <unit kind="mole" exponent="1" scale="-3" multiplier="1" offset="0"/>

     <unit kind="gram" exponent="-1" scale="0" multiplier="1" offset="0"/>

     <unit kind="second" exponent="-1" scale="0" multiplier="0.000277777777777778" offset="0"/>

    </listOfUnits>

   </unitDefinition>

  </listOfUnitDefinitions>

  <listOfCompartments>

   <compartment id="c" name="Cytoplasm" spatialDimensions="3" constant="true"/>

   <compartment id="e" name="Extracellular" spatialDimensions="3" constant="true"/>

  </listOfCompartments>

  <listOfSpecies>

   <species id="M_L_ASP_c" name="L-Aspartate" compartment="c" hasOnlySubstanceUnits="false"
boundaryCondition="false" constant="false">

    <notes>

     <body xmlns="http://www.w3.org/1999/xhtml">

      <p>FORMULA: C4H7NO4</p>

      <p>CHARGE: 0</p>

     </body>

    </notes>

   </species>

   <species id="M_L_GLU_c" name="L-Glutamate" compartment="c" hasOnlySubstanceUnits="false"
boundaryCondition="false" constant="false">
```

```
<notes>
  <body xmlns="http://www.w3.org/1999/xhtml">
    <p>FORMULA: C5H9NO4</p>
    <p>CHARGE: 0</p>
  </body>
</notes>
</species>
<species id="M_L_SER_c" name="L-Serine" compartment="c" hasOnlySubstanceUnits="false" boundaryCondition="false" constant="false">
<notes>
  <body xmlns="http://www.w3.org/1999/xhtml">
    <p>FORMULA: C3H7NO3</p>
    <p>CHARGE: 0</p>
  </body>
</notes>
</species>
<species id="M_L_HIS_c" name="L-Histidine" compartment="c" hasOnlySubstanceUnits="false" boundaryCondition="false" constant="false">
<notes>
  <body xmlns="http://www.w3.org/1999/xhtml">
    <p>FORMULA: C6H9N3O2</p>
    <p>CHARGE: 0</p>
  </body>
</notes>
</species>
<species id="M_L_GLY_c" name="Glycine" compartment="c" hasOnlySubstanceUnits="false" boundaryCondition="false" constant="false">
<notes>
  <body xmlns="http://www.w3.org/1999/xhtml">
    <p>FORMULA: C2H5NO2</p>
```

<p>CHARGE: 0</p>

</body>

</notes>

</species>

<species id="M_L_THR_c" name="L-Threonine" compartment="c" hasOnlySubstanceUnits="false" boundaryCondition="false" constant="false">

<notes>

<body xmlns="http://www.w3.org/1999/xhtml">

<p>FORMULA: C4H9NO3</p>

<p>CHARGE: 0</p>

</body>

</notes>

</species>

<species id="M_L_ARG_c" name="L-Arginine" compartment="c" hasOnlySubstanceUnits="false" boundaryCondition="false" constant="false">

<notes>

<body xmlns="http://www.w3.org/1999/xhtml">

<p>FORMULA: C6H14N4O2</p>

<p>CHARGE: 0</p>

</body>

</notes>

</species>

<species id="M_L_ALA_c" name="L-Alanine" compartment="c" hasOnlySubstanceUnits="false" boundaryCondition="false" constant="false">

<notes>

<body xmlns="http://www.w3.org/1999/xhtml">

<p>FORMULA: C3H7NO2</p>

<p>CHARGE: 0</p>

</body>

</notes>

</species>

<species id="M_L_TYR_c" name="L-Tyrosine" compartment="c" hasOnlySubstanceUnits="false" boundaryCondition="false" constant="false">

<notes>

<body xmlns="http://www.w3.org/1999/xhtml">

<p>FORMULA: C9H11NO3</p>

<p>CHARGE: 0</p>

</body>

</notes>

</species>

<species id="M_L_VAL_c" name="L-Valine" compartment="c" hasOnlySubstanceUnits="false" boundaryCondition="false" constant="false">

<notes>

<body xmlns="http://www.w3.org/1999/xhtml">

<p>FORMULA: C5H11NO2</p>

<p>CHARGE: 0</p>

</body>

</notes>

</species>

<species id="M_L_PHE_c" name="L-Phenylalanine" compartment="c" hasOnlySubstanceUnits="false" boundaryCondition="false" constant="false">

<notes>

<body xmlns="http://www.w3.org/1999/xhtml">

<p>FORMULA: C9H11NO2</p>

<p>CHARGE: 0</p>

</body>

</notes>

</species>

<species id="M_L_ILE_c" name="L-Isoleucine" compartment="c" hasOnlySubstanceUnits="false" boundaryCondition="false" constant="false">

<notes>

<body xmlns="http://www.w3.org/1999/xhtml">

<p>FORMULA: C6H13NO2</p>

<p>CHARGE: 0</p>

</body>

</notes>

</species>

<species id="M_L_LEU_c" name="L-Leucine" compartment="c" hasOnlySubstanceUnits="false" boundaryCondition="false" constant="false">

<notes>

<body xmlns="http://www.w3.org/1999/xhtml">

<p>FORMULA: C6H13NO2</p>

<p>CHARGE: 0</p>

</body>

</notes>

</species>

<species id="M_L_LYS_c" name="L-Lysine" compartment="c" hasOnlySubstanceUnits="false" boundaryCondition="false" constant="false">

<notes>

<body xmlns="http://www.w3.org/1999/xhtml">

<p>FORMULA: C6H14N2O2</p>

<p>CHARGE: 0</p>

</body>

</notes>

</species>

<species id="M_L_PRO_c" name="L-Proline" compartment="c" hasOnlySubstanceUnits="false" boundaryCondition="false" constant="false">

<notes>

<body xmlns="http://www.w3.org/1999/xhtml">

<p>FORMULA: C5H9NO2</p>

```
          <p>CHARGE: 0</p>
        </body>
      </notes>
    </species>
    <species id="M_L_ASN_c" name="L-Asparagine" compartment="c" hasOnlySubstanceUnits="false" boundaryCondition="false" constant="false">
      <notes>
        <body xmlns="http://www.w3.org/1999/xhtml">
          <p>FORMULA: C4H8N2O3</p>
          <p>CHARGE: 0</p>
        </body>
      </notes>
    </species>
    <species id="M_L_CYS_c" name="L-Cysteine" compartment="c" hasOnlySubstanceUnits="false" boundaryCondition="false" constant="false">
      <notes>
        <body xmlns="http://www.w3.org/1999/xhtml">
          <p>FORMULA: C3H7NO2S</p>
          <p>CHARGE: 0</p>
        </body>
      </notes>
    </species>
    <species id="M_L_GLN_c" name="L-Glutamine" compartment="c" hasOnlySubstanceUnits="false" boundaryCondition="false" constant="false">
      <notes>
        <body xmlns="http://www.w3.org/1999/xhtml">
          <p>FORMULA: C5H10N2O3</p>
          <p>CHARGE: 0</p>
        </body>
      </notes>
```

</species>

<species id="M_L_MET_c" name="L-Methionine" compartment="c" hasOnlySubstanceUnits="false" boundaryCondition="false" constant="false">

<notes>

<body xmlns="http://www.w3.org/1999/xhtml">

<p>FORMULA: C5H11NO2S</p>

<p>CHARGE: 0</p>

</body>

</notes>

</species>

<species id="M_L_TRP_c" name="L-Tryptophan" compartment="c" hasOnlySubstanceUnits="false" boundaryCondition="false" constant="false">

<notes>

<body xmlns="http://www.w3.org/1999/xhtml">

<p>FORMULA: C11H12N2O2</p>

<p>CHARGE: 0</p>

</body>

</notes>

</species>

<species id="M_UDP_ACMURM_c" name="UDP-N-acetylmuramate" compartment="c" hasOnlySubstanceUnits="false" boundaryCondition="false" constant="false">

<notes>

<body xmlns="http://www.w3.org/1999/xhtml">

<p>FORMULA: C20H31N3O19P2</p>

<p>CHARGE: 0</p>

</body>

</notes>

</species>

<species id="M_UDP_AGLUAM_c" name="UDP-N-acetyl-D-glucosamine" compartment="c" hasOnlySubstanceUnits="false" boundaryCondition="false" constant="false">

<notes>

<body xmlns="http://www.w3.org/1999/xhtml">

<p>FORMULA: C17H27N3O17P2</p>

<p>CHARGE: 0</p>

</body>

</notes>

</species>

<species id="M_D_GLU_c" name="D-Glutamate" compartment="c" hasOnlySubstanceUnits="false" boundaryCondition="false" constant="false">

<notes>

<body xmlns="http://www.w3.org/1999/xhtml">

<p>FORMULA: C5H9NO4</p>

<p>CHARGE: 0</p>

</body>

</notes>

</species>

<species id="M_ORT_c" name="L-Ornithine" compartment="c" hasOnlySubstanceUnits="false" boundaryCondition="false" constant="false">

<notes>

<body xmlns="http://www.w3.org/1999/xhtml">

<p>FORMULA: C5H12N2O2</p>

<p>CHARGE: 0</p>

</body>

</notes>

</species>

<species id="M_D_ALA_c" name="D-Alanine" compartment="c" hasOnlySubstanceUnits="false" boundaryCondition="false" constant="false">

<notes>

<body xmlns="http://www.w3.org/1999/xhtml">

<p>FORMULA: C3H7NO2</p>

<p>CHARGE: 0</p>

</body>

</notes>

</species>

<species id="M_dTTP_c" name="Deoxythymidine triphosphate" compartment="c" hasOnlySubstanceUnits="false" boundaryCondition="false" constant="false">

<notes>

<body xmlns="http://www.w3.org/1999/xhtml">

<p>FORMULA: C10H17N2O14P3</p>

<p>CHARGE: 0</p>

</body>

</notes>

</species>

<species id="M_dGTP_c" name="2'-Deoxyguanosine 5'-triphosphate (dGTP)" compartment="c" hasOnlySubstanceUnits="false" boundaryCondition="false" constant="false">

<notes>

<body xmlns="http://www.w3.org/1999/xhtml">

<p>FORMULA: C10H16N5O13P3</p>

<p>CHARGE: 0</p>

</body>

</notes>

</species>

<species id="M_dCTP_c" name="Deoxycytidine 5'-triphosphate (dCTP)" compartment="c" hasOnlySubstanceUnits="false" boundaryCondition="false" constant="false">

<notes>

<body xmlns="http://www.w3.org/1999/xhtml">

<p>FORMULA: C9H16N3O13P3</p>

<p>CHARGE: 0</p>

</body>

</notes>

</species>

<species id="M_dATP_c" name="Deoxyadenosine 5'-triphosphate (dATP)" compartment="c" hasOnlySubstanceUnits="false" boundaryCondition="false" constant="false">

<notes>

<body xmlns="http://www.w3.org/1999/xhtml">

<p>FORMULA: C10H16N5O12P3</p>

<p>CHARGE: 0</p>

</body>

</notes>

</species>

<species id="M_CTP_c" name="Cytidine 5'-triphosphate (CTP)" compartment="c" hasOnlySubstanceUnits="false" boundaryCondition="false" constant="false">

<notes>

<body xmlns="http://www.w3.org/1999/xhtml">

<p>FORMULA: C9H16N3O14P3</p>

<p>CHARGE: 0</p>

</body>

</notes>

</species>

<species id="M_GTP_c" name="Guanosine 5'-triphosphate (GTP)" compartment="c" hasOnlySubstanceUnits="false" boundaryCondition="false" constant="false">

<notes>

<body xmlns="http://www.w3.org/1999/xhtml">

<p>FORMULA: C10H16N5O14P3</p>

<p>CHARGE: 0</p>

</body>

</notes>

</species>

<species id="M_UTP_c" name="Uridine 5'-triphosphate (UTP)" compartment="c" hasOnlySubstanceUnits="false" boundaryCondition="false" constant="false">

<notes>

<body xmlns="http://www.w3.org/1999/xhtml">

<p>FORMULA: C9H15N2O15P3</p>

<p>CHARGE: 0</p>

</body>

</notes>

</species>

<species id="M_MGDG_c" name="monoglucosyl-1,2 diacylglycerol" compartment="c" hasOnlySubstanceUnits="false" boundaryCondition="false" constant="false">

<notes>

<body xmlns="http://www.w3.org/1999/xhtml">

<p>FORMULA: </p>

<p>CHARGE: 0</p>

</body>

</notes>

</species>

<species id="M_DGDG_c" name="diglucosyl-1,2 diacylglycerol" compartment="c" hasOnlySubstanceUnits="false" boundaryCondition="false" constant="false">

<notes>

<body xmlns="http://www.w3.org/1999/xhtml">

<p>FORMULA: </p>

<p>CHARGE: 0</p>

</body>

</notes>

</species>

<species id="M_tZXT_c" name="Thermozeaxanthin" compartment="c" hasOnlySubstanceUnits="false" boundaryCondition="false" constant="false">

<notes>

<body xmlns="http://www.w3.org/1999/xhtml">

<p>FORMULA: C59H90O8</p>

<p>CHARGE: 0</p>

</body>

</notes>

</species>

<species id="M_PETHA_c" name="Phosphatidylethanolamine" compartment="c" hasOnlySubstanceUnits="false" boundaryCondition="false" constant="false">

<notes>

<body xmlns="http://www.w3.org/1999/xhtml">

<p>FORMULA: C7H12NO8PR2</p>

<p>CHARGE: 0</p>

</body>

</notes>

</species>

<species id="M_PGLY_c" name="phosphatidylglycerol" compartment="c" hasOnlySubstanceUnits="false" boundaryCondition="false" constant="false">

<notes>

<body xmlns="http://www.w3.org/1999/xhtml">

<p>FORMULA: </p>

<p>CHARGE: 0</p>

</body>

</notes>

</species>

<species id="M_PINS_c" name="1-phosphatidyl-inositol" compartment="c" hasOnlySubstanceUnits="false" boundaryCondition="false" constant="false">

<notes>

<body xmlns="http://www.w3.org/1999/xhtml">

<p>FORMULA: </p>

<p>CHARGE: 0</p>

</body>

</notes>

</species>

<species id="M_CDLPN_c" name="cardiolipin" compartment="c" hasOnlySubstanceUnits="false" boundaryCondition="false" constant="false">

<notes>

<body xmlns="http://www.w3.org/1999/xhtml">

<p>FORMULA: </p>

<p>CHARGE: 0</p>

</body>

</notes>

</species>

<species id="M_PX_c" name="N-acylphosphoethanolamine" compartment="c" hasOnlySubstanceUnits="false" boundaryCondition="false" constant="false">

<notes>

<body xmlns="http://www.w3.org/1999/xhtml">

<p>FORMULA: </p>

<p>CHARGE: 0</p>

</body>

</notes>

</species>

<species id="M_ATP_c" name="Adenosine 5'-triphosphate (ATP)" compartment="c" hasOnlySubstanceUnits="false" boundaryCondition="false" constant="false">

<notes>

<body xmlns="http://www.w3.org/1999/xhtml">

<p>FORMULA: C10H16N5O13P3</p>

<p>CHARGE: 0</p>

</body>

</notes>

</species>

<species id="M_ADP_c" name="Adenosine 5'-diphosphate (ADP)" compartment="c" hasOnlySubstanceUnits="false" boundaryCondition="false" constant="false">

<notes>

<body xmlns="http://www.w3.org/1999/xhtml">

<p>FORMULA: C10H15N5O10P2</p>

<p>CHARGE: 0</p>

</body>

</notes>

</species>

<species id="M_Pi_c" name="Orthophosphate" compartment="c" hasOnlySubstanceUnits="false" boundaryCondition="false" constant="false">

<notes>

<body xmlns="http://www.w3.org/1999/xhtml">

<p>FORMULA: H3PO4</p>

<p>CHARGE: 0</p>

</body>

</notes>

</species>

<species id="M_PPi_c" name="Diphosphate" compartment="c" hasOnlySubstanceUnits="false" boundaryCondition="false" constant="false">

<notes>

<body xmlns="http://www.w3.org/1999/xhtml">

<p>FORMULA: P2H4O7</p>

<p>CHARGE: 0</p>

</body>

</notes>

</species>

<species id="M_H2O_c" name="Water" compartment="c" hasOnlySubstanceUnits="false" boundaryCondition="false" constant="false">

<notes>

<body xmlns="http://www.w3.org/1999/xhtml">

<p>FORMULA: H2O</p>

<p>CHARGE: 0</p>

</body>

</notes>

</species>

<species id="M_H_c" name="Hydron" compartment="c" hasOnlySubstanceUnits="false" boundaryCondition="false" constant="false">

<notes>

<body xmlns="http://www.w3.org/1999/xhtml">

<p>FORMULA: H</p>

<p>CHARGE: 0</p>

</body>

</notes>

</species>

<species id="M_D_GLC_c" name="D-Glucose" compartment="c" hasOnlySubstanceUnits="false" boundaryCondition="false" constant="false">

<notes>

<body xmlns="http://www.w3.org/1999/xhtml">

<p>FORMULA: C6H12O6</p>

<p>CHARGE: 0</p>

</body>

</notes>

</species>

<species id="M_G6P_c" name="D-Glucose 6-phosphate" compartment="c" hasOnlySubstanceUnits="false" boundaryCondition="false" constant="false">

<notes>

<body xmlns="http://www.w3.org/1999/xhtml">

<p>FORMULA: C6H13O9P</p>

<p>CHARGE: 0</p>

</body>

</notes>

</species>

<species id="M_D_MAN_c" name="D-Mannose" compartment="c" hasOnlySubstanceUnits="false" boundaryCondition="false" constant="false">

<notes>

<body xmlns="http://www.w3.org/1999/xhtml">

<p>FORMULA: C6H12O6</p>

<p>CHARGE: 0</p>

</body>

</notes>

</species>

<species id="M_M6P_c" name="D-Mannose 6-phosphate" compartment="c" hasOnlySubstanceUnits="false" boundaryCondition="false" constant="false">

<notes>

<body xmlns="http://www.w3.org/1999/xhtml">

<p>FORMULA: C6H13O9P</p>

<p>CHARGE: 0</p>

</body>

</notes>

</species>

<species id="M_F6P_c" name="D-Fructose 6-phosphate" compartment="c" hasOnlySubstanceUnits="false" boundaryCondition="false" constant="false">

<notes>

<body xmlns="http://www.w3.org/1999/xhtml">

<p>FORMULA: C6H13O9P</p>

<p>CHARGE: 0</p>

</body>

</notes>

</species>

<species id="M_FBP_c" name="D-Fructose 1,6-bisphosphate" compartment="c" hasOnlySubstanceUnits="false" boundaryCondition="false" constant="false">

```
        <notes>
          <body xmlns="http://www.w3.org/1999/xhtml">
            <p>FORMULA: C6H14O12P2</p>
            <p>CHARGE: 0</p>
          </body>
        </notes>
      </species>
      <species id="M_DHAP_c" name="Dihydroxyacetone phosphate" compartment="c" hasOnlySubstanceUnits="false"
boundaryCondition="false" constant="false">
        <notes>
          <body xmlns="http://www.w3.org/1999/xhtml">
            <p>FORMULA: C3H7O6P</p>
            <p>CHARGE: 0</p>
          </body>
        </notes>
      </species>
      <species id="M_GAP_c" name="D-Glyceraldehyde 3-phosphate" compartment="c" hasOnlySubstanceUnits="false"
boundaryCondition="false" constant="false">
        <notes>
          <body xmlns="http://www.w3.org/1999/xhtml">
            <p>FORMULA: C3H7O6P</p>
            <p>CHARGE: 0</p>
          </body>
        </notes>
      </species>
      <species id="M_NAD_c" name="Nicotinamide adenine dinucleotide (NAD)" compartment="c"
hasOnlySubstanceUnits="false" boundaryCondition="false" constant="false">
        <notes>
          <body xmlns="http://www.w3.org/1999/xhtml">
            <p>FORMULA: C21H28N7O14P2</p>
```

<p>CHARGE: 0</p>

</body>

</notes>

</species>

<species id="M_13BPG_c" name="1,3-Bisphospho-D-glycerate" compartment="c" hasOnlySubstanceUnits="false" boundaryCondition="false" constant="false">

<notes>

<body xmlns="http://www.w3.org/1999/xhtml">

<p>FORMULA: C3H8O10P2</p>

<p>CHARGE: 0</p>

</body>

</notes>

</species>

<species id="M_NADH_c" name="Nicotinamide adenine dinucleotide (NADH)" compartment="c" hasOnlySubstanceUnits="false" boundaryCondition="false" constant="false">

<notes>

<body xmlns="http://www.w3.org/1999/xhtml">

<p>FORMULA: C21H29N7O14P2</p>

<p>CHARGE: 0</p>

</body>

</notes>

</species>

<species id="M_3PG_c" name="D-Glycerate 3-phosphate" compartment="c" hasOnlySubstanceUnits="false" boundaryCondition="false" constant="false">

<notes>

<body xmlns="http://www.w3.org/1999/xhtml">

<p>FORMULA: C3H7O7P</p>

<p>CHARGE: 0</p>

</body>

</notes>

</species>

<species id="M_2PG_c" name="D-Glycerate 2-phosphate" compartment="c" hasOnlySubstanceUnits="false" boundaryCondition="false" constant="false">

<notes>

<body xmlns="http://www.w3.org/1999/xhtml">

<p>FORMULA: C3H7O7P</p>

<p>CHARGE: 0</p>

</body>

</notes>

</species>

<species id="M_PEP_c" name="Phosphoenolpyruvate" compartment="c" hasOnlySubstanceUnits="false" boundaryCondition="false" constant="false">

<notes>

<body xmlns="http://www.w3.org/1999/xhtml">

<p>FORMULA: C3H5O6P</p>

<p>CHARGE: 0</p>

</body>

</notes>

</species>

<species id="M_PYR_c" name="Pyruvate" compartment="c" hasOnlySubstanceUnits="false" boundaryCondition="false" constant="false">

<notes>

<body xmlns="http://www.w3.org/1999/xhtml">

<p>FORMULA: C3H4O3</p>

<p>CHARGE: 0</p>

</body>

</notes>

</species>

<species id="M_OAC_c" name="Oxaloacetate" compartment="c" hasOnlySubstanceUnits="false" boundaryCondition="false" constant="false">

<notes>

<body xmlns="http://www.w3.org/1999/xhtml">

<p>FORMULA: C4H4O5</p>

<p>CHARGE: 0</p>

</body>

</notes>

</species>

<species id="M_CO2_c" name="Carbon dioxide" compartment="c" hasOnlySubstanceUnits="false" boundaryCondition="false" constant="false">

<notes>

<body xmlns="http://www.w3.org/1999/xhtml">

<p>FORMULA: CO2</p>

<p>CHARGE: 0</p>

</body>

</notes>

</species>

<species id="M_LAC_c" name="L-Lactate" compartment="c" hasOnlySubstanceUnits="false" boundaryCondition="false" constant="false">

<notes>

<body xmlns="http://www.w3.org/1999/xhtml">

<p>FORMULA: C3H6O3</p>

<p>CHARGE: 0</p>

</body>

</notes>

</species>

<species id="M_CoA_c" name="Coenzyme A (CoA)" compartment="c" hasOnlySubstanceUnits="false" boundaryCondition="false" constant="false">

<notes>

<body xmlns="http://www.w3.org/1999/xhtml">

<p>FORMULA: C21H36N7O16P3S</p>

<p>CHARGE: 0</p>

</body>

</notes>

</species>

<species id="M_AC_c" name="Acetate" compartment="c" hasOnlySubstanceUnits="false" boundaryCondition="false" constant="false">

<notes>

<body xmlns="http://www.w3.org/1999/xhtml">

<p>FORMULA: C2H4O2</p>

<p>CHARGE: 0</p>

</body>

</notes>

</species>

<species id="M_AMP_c" name="Adenosine 5'-monophosphate (AMP)" compartment="c" hasOnlySubstanceUnits="false" boundaryCondition="false" constant="false">

<notes>

<body xmlns="http://www.w3.org/1999/xhtml">

<p>FORMULA: C10H14N5O7P</p>

<p>CHARGE: 0</p>

</body>

</notes>

</species>

<species id="M_ACoA_c" name="Acetyl-CoA" compartment="c" hasOnlySubstanceUnits="false" boundaryCondition="false" constant="false">

<notes>

<body xmlns="http://www.w3.org/1999/xhtml">

<p>FORMULA: C23H38N7O17P3S</p>

<p>CHARGE: 0</p>

</body>

</notes>

</species>

<species id="M_AALD_c" name="Acetaldehyde" compartment="c" hasOnlySubstanceUnits="false" boundaryCondition="false" constant="false">

<notes>

<body xmlns="http://www.w3.org/1999/xhtml">

<p>FORMULA: C2H4O</p>

<p>CHARGE: 0</p>

</body>

</notes>

</species>

<species id="M_ETH_c" name="Ethanol" compartment="c" hasOnlySubstanceUnits="false" boundaryCondition="false" constant="false">

<notes>

<body xmlns="http://www.w3.org/1999/xhtml">

<p>FORMULA: C2H6O</p>

<p>CHARGE: 0</p>

</body>

</notes>

</species>

<species id="M_CIT_c" name="Citrate" compartment="c" hasOnlySubstanceUnits="false" boundaryCondition="false" constant="false">

<notes>

<body xmlns="http://www.w3.org/1999/xhtml">

<p>FORMULA: C6H8O7</p>

<p>CHARGE: 0</p>

</body>

</notes>

</species>

<species id="M_cACO_c" name="cis-Aconitate" compartment="c" hasOnlySubstanceUnits="false" boundaryCondition="false" constant="false">

<notes>

<body xmlns="http://www.w3.org/1999/xhtml">

<p>FORMULA: C6H6O6</p>

<p>CHARGE: 0</p>

</body>

</notes>

</species>

<species id="M_ICIT_c" name="Isocitrate" compartment="c" hasOnlySubstanceUnits="false" boundaryCondition="false" constant="false">

<notes>

<body xmlns="http://www.w3.org/1999/xhtml">

<p>FORMULA: C6H8O7</p>

<p>CHARGE: 0</p>

</body>

</notes>

</species>

<species id="M_NADP_c" name="Nicotinamide adenine dinucleotide phosphate (NADP)" compartment="c" hasOnlySubstanceUnits="false" boundaryCondition="false" constant="false">

<notes>

<body xmlns="http://www.w3.org/1999/xhtml">

<p>FORMULA: C21H29N7O17P3</p>

<p>CHARGE: 0</p>

</body>

</notes>

</species>

<species id="M_OXLSUCC_c" name="Oxalosuccinate" compartment="c" hasOnlySubstanceUnits="false" boundaryCondition="false" constant="false">

<notes>

<body xmlns="http://www.w3.org/1999/xhtml">

<p>FORMULA: C6H6O7</p>

<p>CHARGE: 0</p>

</body>

</notes>

</species>

<species id="M_NADPH_c" name="Reduced nicotinamide adenine dinucleotide phosphate (NADPH)" compartment="c" hasOnlySubstanceUnits="false" boundaryCondition="false" constant="false">

<notes>

<body xmlns="http://www.w3.org/1999/xhtml">

<p>FORMULA: C21H30N7O17P3</p>

<p>CHARGE: 0</p>

</body>

</notes>

</species>

<species id="M_AKG_c" name="alpha-Ketoglutaric acid" compartment="c" hasOnlySubstanceUnits="false" boundaryCondition="false" constant="false">

<notes>

<body xmlns="http://www.w3.org/1999/xhtml">

<p>FORMULA: C5H6O5</p>

<p>CHARGE: 0</p>

</body>

</notes>

</species>

<species id="M_SUCCoA_c" name="Succinyl-CoA" compartment="c" hasOnlySubstanceUnits="false" boundaryCondition="false" constant="false">

<notes>

<body xmlns="http://www.w3.org/1999/xhtml">

<p>FORMULA: C25H40N7O19P3S</p>

<p>CHARGE: 0</p>

</body>

</notes>

</species>

<species id="M_SUCC_c" name="Succinate" compartment="c" hasOnlySubstanceUnits="false" boundaryCondition="false" constant="false">

<notes>

<body xmlns="http://www.w3.org/1999/xhtml">

<p>FORMULA: C4H6O4</p>

<p>CHARGE: 0</p>

</body>

</notes>

</species>

<species id="M_FAD_c" name="Flavin adenine dinucleotide (FAD)" compartment="c" hasOnlySubstanceUnits="false" boundaryCondition="false" constant="false">

<notes>

<body xmlns="http://www.w3.org/1999/xhtml">

<p>FORMULA: C27H33N9O15P2</p>

<p>CHARGE: 0</p>

</body>

</notes>

</species>

<species id="M_FADH2_c" name="Reduced Flavin adenine dinucleotide (FADH2)" compartment="c" hasOnlySubstanceUnits="false" boundaryCondition="false" constant="false">

<notes>

<body xmlns="http://www.w3.org/1999/xhtml">

<p>FORMULA: C27H35N9O15P2</p>

<p>CHARGE: 0</p>

</body>

</notes>

</species>

<species id="M_FUM_c" name="Fumarate" compartment="c" hasOnlySubstanceUnits="false" boundaryCondition="false" constant="false">

<notes>

<body xmlns="http://www.w3.org/1999/xhtml">

<p>FORMULA: C4H4O4</p>

<p>CHARGE: 0</p>

</body>

</notes>

</species>

<species id="M_MAL_c" name="L-Malate" compartment="c" hasOnlySubstanceUnits="false" boundaryCondition="false" constant="false">

<notes>

<body xmlns="http://www.w3.org/1999/xhtml">

<p>FORMULA: C4H6O5</p>

<p>CHARGE: 0</p>

</body>

</notes>

</species>

<species id="M_Q_c" name="Ubiquinone" compartment="c" hasOnlySubstanceUnits="false" boundaryCondition="false" constant="false">

<notes>

<body xmlns="http://www.w3.org/1999/xhtml">

<p>FORMULA: C14H18O4(C5H8)n</p>

<p>CHARGE: 0</p>

</body>

</notes>

</species>

<species id="M_GLC_LACN_c" name="D-Glucono-1,5-lactone" compartment="c" hasOnlySubstanceUnits="false" boundaryCondition="false" constant="false">

<notes>

<body xmlns="http://www.w3.org/1999/xhtml">

<p>FORMULA: C6H10O6</p>

<p>CHARGE: 0</p>

</body>

</notes>

</species>

<species id="M_QH2_c" name="Ubiquinol" compartment="c" hasOnlySubstanceUnits="false" boundaryCondition="false" constant="false">

<notes>

<body xmlns="http://www.w3.org/1999/xhtml">

<p>FORMULA: C14H20O4(C5H8)n</p>

<p>CHARGE: 0</p>

</body>

</notes>

</species>

<species id="M_E4P_c" name="D-Erythrose 4-phosphate" compartment="c" hasOnlySubstanceUnits="false" boundaryCondition="false" constant="false">

<notes>

<body xmlns="http://www.w3.org/1999/xhtml">

<p>FORMULA: C4H9O7P</p>

<p>CHARGE: 0</p>

</body>

</notes>

</species>

<species id="M_XYL5P_c" name="D-Xylulose 5-phosphate" compartment="c" hasOnlySubstanceUnits="false" boundaryCondition="false" constant="false">

<notes>

<body xmlns="http://www.w3.org/1999/xhtml">

<p>FORMULA: C5H11O8P</p>

<p>CHARGE: 0</p>

</body>

</notes>

</species>

<species id="M_SEDHEP7P_c" name="D-Sedoheptulose 7-phosphate" compartment="c" hasOnlySubstanceUnits="false" boundaryCondition="false" constant="false">

<notes>

<body xmlns="http://www.w3.org/1999/xhtml">

<p>FORMULA: C7H15O10P</p>

<p>CHARGE: 0</p>

</body>

</notes>

</species>

<species id="M_R5P_c" name="D-Ribose 5-phosphate" compartment="c" hasOnlySubstanceUnits="false" boundaryCondition="false" constant="false">

<notes>

<body xmlns="http://www.w3.org/1999/xhtml">

<p>FORMULA: C5H11O8P</p>

<p>CHARGE: 0</p>

</body>

</notes>

</species>

<species id="M_RBL5P_c" name="D-Ribulose 5-phosphate" compartment="c" hasOnlySubstanceUnits="false" boundaryCondition="false" constant="false">

<notes>

<body xmlns="http://www.w3.org/1999/xhtml">

<p>FORMULA: C5H11O8P</p>

<p>CHARGE: 0</p>

</body>

</notes>

</species>

<species id="M_PRPP_c" name="5-Phospho-alpha-D-ribose 1-diphosphate" compartment="c" hasOnlySubstanceUnits="false" boundaryCondition="false" constant="false">

<notes>
<body xmlns="http://www.w3.org/1999/xhtml">
<p>FORMULA: C5H13O14P3</p>
<p>CHARGE: 0</p>
</body>
</notes>
</species>
<species id="M_R1P_c" name="D-Ribose 1-phosphate" compartment="c" hasOnlySubstanceUnits="false" boundaryCondition="false" constant="false">
<notes>
<body xmlns="http://www.w3.org/1999/xhtml">
<p>FORMULA: C5H11O8P</p>
<p>CHARGE: 0</p>
</body>
</notes>
</species>
<species id="M_D_RIB_c" name="D-Ribose" compartment="c" hasOnlySubstanceUnits="false" boundaryCondition="false" constant="false">
<notes>
<body xmlns="http://www.w3.org/1999/xhtml">
<p>FORMULA: C5H10O5</p>
<p>CHARGE: 0</p>
</body>
</notes>
</species>
<species id="M_dR5P_c" name="2-Deoxy-D-ribose 5-phosphate" compartment="c" hasOnlySubstanceUnits="false" boundaryCondition="false" constant="false">
<notes>
<body xmlns="http://www.w3.org/1999/xhtml">
<p>FORMULA: C5H11O7P</p>

<p>CHARGE: 0</p>

</body>

</notes>

</species>

<species id="M_dR1P_c" name="2-Deoxy-D-ribose 1-phosphate" compartment="c" hasOnlySubstanceUnits="false" boundaryCondition="false" constant="false">

<notes>

<body xmlns="http://www.w3.org/1999/xhtml">

<p>FORMULA: C5H11O7P</p>

<p>CHARGE: 0</p>

</body>

</notes>

</species>

<species id="M_GLYALD_c" name="D-Glyceraldehyde" compartment="c" hasOnlySubstanceUnits="false" boundaryCondition="false" constant="false">

<notes>

<body xmlns="http://www.w3.org/1999/xhtml">

<p>FORMULA: C3H6O3</p>

<p>CHARGE: 0</p>

</body>

</notes>

</species>

<species id="M_D_GLCT_c" name="D-Glycerate" compartment="c" hasOnlySubstanceUnits="false" boundaryCondition="false" constant="false">

<notes>

<body xmlns="http://www.w3.org/1999/xhtml">

<p>FORMULA: C3H6O4</p>

<p>CHARGE: 0</p>

</body>

</notes>

</species>

<species id="M_DHY_DOP_GLUCN_c" name="2-Dehydro-3-deoxy-6-phospho-D-gluconate" compartment="c" hasOnlySubstanceUnits="false" boundaryCondition="false" constant="false">

<notes>

<body xmlns="http://www.w3.org/1999/xhtml">

<p>FORMULA: C6H11O9P</p>

<p>CHARGE: 0</p>

</body>

</notes>

</species>

<species id="M_2K3DO_GLCN_c" name="2-Dehydro-3-deoxy-D-gluconate" compartment="c" hasOnlySubstanceUnits="false" boundaryCondition="false" constant="false">

<notes>

<body xmlns="http://www.w3.org/1999/xhtml">

<p>FORMULA: C6H10O6</p>

<p>CHARGE: 0</p>

</body>

</notes>

</species>

<species id="M_DHY_DOHEXNA_c" name="(4S)-4,6-Dihydroxy-2,5-dioxohexanoate" compartment="c" hasOnlySubstanceUnits="false" boundaryCondition="false" constant="false">

<notes>

<body xmlns="http://www.w3.org/1999/xhtml">

<p>FORMULA: C6H8O6</p>

<p>CHARGE: 0</p>

</body>

</notes>

</species>

<species id="M_F1P_c" name="D-Fructose 1-phosphate" compartment="c" hasOnlySubstanceUnits="false" boundaryCondition="false" constant="false">

<notes>

<body xmlns="http://www.w3.org/1999/xhtml">

<p>FORMULA: C6H13O9P</p>

<p>CHARGE: 0</p>

</body>

</notes>

</species>

<species id="M_FUCL1P_c" name="L-Fuculose 1-phosphate" compartment="c" hasOnlySubstanceUnits="false" boundaryCondition="false" constant="false">

<notes>

<body xmlns="http://www.w3.org/1999/xhtml">

<p>FORMULA: C6H13O8P</p>

<p>CHARGE: 0</p>

</body>

</notes>

</species>

<species id="M_LALD_c" name="(S)-Lactaldehyde" compartment="c" hasOnlySubstanceUnits="false" boundaryCondition="false" constant="false">

<notes>

<body xmlns="http://www.w3.org/1999/xhtml">

<p>FORMULA: C3H6O2</p>

<p>CHARGE: 0</p>

</body>

</notes>

</species>

<species id="M_GDP_MAN_c" name="GDP-D-mannose" compartment="c" hasOnlySubstanceUnits="false" boundaryCondition="false" constant="false">

<notes>

<body xmlns="http://www.w3.org/1999/xhtml">

<p>FORMULA: C16H25N5O16P2</p>

```
<p>CHARGE: 0</p>
</body>
</notes>
</species>
<species id="M_GDP_c" name="Guanosine 5&apos;-diphosphate (GDP)" compartment="c" hasOnlySubstanceUnits="false" boundaryCondition="false" constant="false">
<notes>
<body xmlns="http://www.w3.org/1999/xhtml">
<p>FORMULA: C10H15N5O11P2</p>
<p>CHARGE: 0</p>
</body>
</notes>
</species>
<species id="M_MANNPGLYCT_c" name="2-(alpha-D-Mannosyl)-3-phosphoglycerate" compartment="c" hasOnlySubstanceUnits="false" boundaryCondition="false" constant="false">
<notes>
<body xmlns="http://www.w3.org/1999/xhtml">
<p>FORMULA: C9H17O12P</p>
<p>CHARGE: 0</p>
</body>
</notes>
</species>
<species id="M_AMANN_GLYCT_c" name="2(alpha-D-Mannosyl)-D-glycerate" compartment="c" hasOnlySubstanceUnits="false" boundaryCondition="false" constant="false">
<notes>
<body xmlns="http://www.w3.org/1999/xhtml">
<p>FORMULA: C9H16O9</p>
<p>CHARGE: 0</p>
</body>
</notes>
```

</species>

<species id="M_MAN1P_c" name="D-Mannose 1-phosphate" compartment="c" hasOnlySubstanceUnits="false" boundaryCondition="false" constant="false">

<notes>

<body xmlns="http://www.w3.org/1999/xhtml">

<p>FORMULA: C6H13O9P</p>

<p>CHARGE: 0</p>

</body>

</notes>

</species>

<species id="M_D_FRU_c" name="D-Fructose" compartment="c" hasOnlySubstanceUnits="false" boundaryCondition="false" constant="false">

<notes>

<body xmlns="http://www.w3.org/1999/xhtml">

<p>FORMULA: C6H12O6</p>

<p>CHARGE: 0</p>

</body>

</notes>

</species>

<species id="M_D_GAL_c" name="D-Galactose" compartment="c" hasOnlySubstanceUnits="false" boundaryCondition="false" constant="false">

<notes>

<body xmlns="http://www.w3.org/1999/xhtml">

<p>FORMULA: C6H12O6</p>

<p>CHARGE: 0</p>

</body>

</notes>

</species>

<species id="M_GAL1P_c" name="alpha-D-Galactose 1-phosphate" compartment="c" hasOnlySubstanceUnits="false" boundaryCondition="false" constant="false">

```
<notes>
  <body xmlns="http://www.w3.org/1999/xhtml">
    <p>FORMULA: C6H13O9P</p>
    <p>CHARGE: 0</p>
  </body>
</notes>
</species>
<species id="M_UDP_GLU_c" name="UDP-D-glucose" compartment="c" hasOnlySubstanceUnits="false"
boundaryCondition="false" constant="false">
<notes>
  <body xmlns="http://www.w3.org/1999/xhtml">
    <p>FORMULA: C15H24N2O17P2</p>
    <p>CHARGE: 0</p>
  </body>
</notes>
</species>
<species id="M_G1P_c" name="D-Glucose 1-phosphate" compartment="c" hasOnlySubstanceUnits="false"
boundaryCondition="false" constant="false">
<notes>
  <body xmlns="http://www.w3.org/1999/xhtml">
    <p>FORMULA: C6H13O9P</p>
    <p>CHARGE: 0</p>
  </body>
</notes>
</species>
<species id="M_UDP_GAL_c" name="UDP-D-galactose" compartment="c" hasOnlySubstanceUnits="false"
boundaryCondition="false" constant="false">
<notes>
  <body xmlns="http://www.w3.org/1999/xhtml">
    <p>FORMULA: C15H24N2O17P2</p>
```

<p>CHARGE: 0</p>

</body>

</notes>

</species>

<species id="M_GLCTN_c" name="Galactan" compartment="c" hasOnlySubstanceUnits="false" boundaryCondition="false" constant="false">

<notes>

<body xmlns="http://www.w3.org/1999/xhtml">

<p>FORMULA: (C12H20O11)n</p>

<p>CHARGE: 0</p>

</body>

</notes>

</species>

<species id="M_LACTS_c" name="Lactose" compartment="c" hasOnlySubstanceUnits="false" boundaryCondition="false" constant="false">

<notes>

<body xmlns="http://www.w3.org/1999/xhtml">

<p>FORMULA: C12H22O11</p>

<p>CHARGE: 0</p>

</body>

</notes>

</species>

<species id="M_EPMLBIOS_c" name="Epimelibiose" compartment="c" hasOnlySubstanceUnits="false" boundaryCondition="false" constant="false">

<notes>

<body xmlns="http://www.w3.org/1999/xhtml">

<p>FORMULA: C12H22O11</p>

<p>CHARGE: 0</p>

</body>

</notes>

</species>

<species id="M_AGAL_INS_c" name="Galactinol" compartment="c" hasOnlySubstanceUnits="false" boundaryCondition="false" constant="false">

<notes>

<body xmlns="http://www.w3.org/1999/xhtml">

<p>FORMULA: C12H22O11</p>

<p>CHARGE: 0</p>

</body>

</notes>

</species>

<species id="M_M_INS_c" name="D-myo-Inositol" compartment="c" hasOnlySubstanceUnits="false" boundaryCondition="false" constant="false">

<notes>

<body xmlns="http://www.w3.org/1999/xhtml">

<p>FORMULA: C6H12O6</p>

<p>CHARGE: 0</p>

</body>

</notes>

</species>

<species id="M_MLBTL_c" name="Melibiitol" compartment="c" hasOnlySubstanceUnits="false" boundaryCondition="false" constant="false">

<notes>

<body xmlns="http://www.w3.org/1999/xhtml">

<p>FORMULA: C12H24O11</p>

<p>CHARGE: 0</p>

</body>

</notes>

</species>

<species id="M_SORB_c" name="D-Sorbitol" compartment="c" hasOnlySubstanceUnits="false" boundaryCondition="false" constant="false">

<notes>

<body xmlns="http://www.w3.org/1999/xhtml">

<p>FORMULA: C6H14O6</p>

<p>CHARGE: 0</p>

</body>

</notes>

</species>

<species id="M_GALCS_GLYC_c" name="Galactosylglycerol" compartment="c" hasOnlySubstanceUnits="false" boundaryCondition="false" constant="false">

<notes>

<body xmlns="http://www.w3.org/1999/xhtml">

<p>FORMULA: C9H18O8</p>

<p>CHARGE: 0</p>

</body>

</notes>

</species>

<species id="M_GLYCRL_c" name="Glycerol" compartment="c" hasOnlySubstanceUnits="false" boundaryCondition="false" constant="false">

<notes>

<body xmlns="http://www.w3.org/1999/xhtml">

<p>FORMULA: C3H8O3</p>

<p>CHARGE: 0</p>

</body>

</notes>

</species>

<species id="M_STCYS_c" name="Stachyose" compartment="c" hasOnlySubstanceUnits="false" boundaryCondition="false" constant="false">

<notes>

<body xmlns="http://www.w3.org/1999/xhtml">

<p>FORMULA: C24H42O21</p>

<p>CHARGE: 0</p>

</body>

</notes>

</species>

<species id="M_RAFF_c" name="Raffinose" compartment="c" hasOnlySubstanceUnits="false" boundaryCondition="false" constant="false">

<notes>

<body xmlns="http://www.w3.org/1999/xhtml">

<p>FORMULA: C18H32O16</p>

<p>CHARGE: 0</p>

</body>

</notes>

</species>

<species id="M_MANNTRIS_c" name="Mannotriose" compartment="c" hasOnlySubstanceUnits="false" boundaryCondition="false" constant="false">

<notes>

<body xmlns="http://www.w3.org/1999/xhtml">

<p>FORMULA: C18H32O16</p>

<p>CHARGE: 0</p>

</body>

</notes>

</species>

<species id="M_MLBIOS_c" name="Melibiose" compartment="c" hasOnlySubstanceUnits="false" boundaryCondition="false" constant="false">

<notes>

<body xmlns="http://www.w3.org/1999/xhtml">

<p>FORMULA: C12H22O11</p>

<p>CHARGE: 0</p>

</body>

</notes>

```
</species>

<species id="M_SUC_c" name="Sucrose" compartment="c" hasOnlySubstanceUnits="false" boundaryCondition="false" constant="false">

<notes>

<body xmlns="http://www.w3.org/1999/xhtml">

<p>FORMULA: C12H22O11</p>

<p>CHARGE: 0</p>

</body>

</notes>

</species>

<species id="M_PALT_c" name="Paltinose" compartment="c" hasOnlySubstanceUnits="false" boundaryCondition="false" constant="false">

<notes>

<body xmlns="http://www.w3.org/1999/xhtml">

<p>FORMULA: </p>

<p>CHARGE: 0</p>

</body>

</notes>

</species>

<species id="M_ct_FARPPi_c" name="2-cis,6-trans-Farnesyl diphosphate" compartment="c" hasOnlySubstanceUnits="false" boundaryCondition="false" constant="false">

<notes>

<body xmlns="http://www.w3.org/1999/xhtml">

<p>FORMULA: C15H28O7P2</p>

<p>CHARGE: 0</p>

</body>

</notes>

</species>

<species id="M_IPEN_PPi_c" name="Isopentenyl diphosphate" compartment="c" hasOnlySubstanceUnits="false" boundaryCondition="false" constant="false">
```

<notes>
<body xmlns="http://www.w3.org/1999/xhtml">
<p>FORMULA: C5H12O7P2</p>
<p>CHARGE: 0</p>
</body>
</notes>
</species>
<species id="M_t_PPREPPi_c" name="all-trans-Polyprenyl diphosphate" compartment="c" hasOnlySubstanceUnits="false" boundaryCondition="false" constant="false">
<notes>
<body xmlns="http://www.w3.org/1999/xhtml">
<p>FORMULA: C5H12O7P2(C5H8)n</p>
<p>CHARGE: 0</p>
</body>
</notes>
</species>
<species id="M_CORM_c" name="Chorismate" compartment="c" hasOnlySubstanceUnits="false" boundaryCondition="false" constant="false">
<notes>
<body xmlns="http://www.w3.org/1999/xhtml">
<p>FORMULA: C10H10O6</p>
<p>CHARGE: 0</p>
</body>
</notes>
</species>
<species id="M_HY_BNZA_c" name="4-Hydroxybenzoate" compartment="c" hasOnlySubstanceUnits="false" boundaryCondition="false" constant="false">
<notes>
<body xmlns="http://www.w3.org/1999/xhtml">
<p>FORMULA: C7H6O3</p>

<p>CHARGE: 0</p>

</body>

</notes>

</species>

<species id="M_HY_PPREBNZA_c" name="4-Hydroxy-3-polyprenylbenzoate" compartment="c" hasOnlySubstanceUnits="false" boundaryCondition="false" constant="false">

<notes>

<body xmlns="http://www.w3.org/1999/xhtml">

<p>FORMULA: C12H14O3(C5H8)n</p>

<p>CHARGE: 0</p>

</body>

</notes>

</species>

<species id="M_PPRENPhOL_c" name="2-Polyprenylphenol" compartment="c" hasOnlySubstanceUnits="false" boundaryCondition="false" constant="false">

<notes>

<body xmlns="http://www.w3.org/1999/xhtml">

<p>FORMULA: C11H14O(C5H8)n</p>

<p>CHARGE: 0</p>

</body>

</notes>

</species>

<species id="M_O2_c" name="Oxygen" compartment="c" hasOnlySubstanceUnits="false" boundaryCondition="false" constant="false">

<notes>

<body xmlns="http://www.w3.org/1999/xhtml">

<p>FORMULA: O2</p>

<p>CHARGE: 0</p>

</body>

</notes>

</species>
<species id="M_PBDIOL_c" name="2-polyprenyl-6-hydroxyphenol" compartment="c" hasOnlySubstanceUnits="false" boundaryCondition="false" constant="false">
<notes>
<body xmlns="http://www.w3.org/1999/xhtml">
<p>FORMULA: </p>
<p>CHARGE: 0</p>
</body>
</notes>
</species>
<species id="M_SAM_c" name="S-Adenosyl-L-methionine (SAM)" compartment="c" hasOnlySubstanceUnits="false" boundaryCondition="false" constant="false">
<notes>
<body xmlns="http://www.w3.org/1999/xhtml">
<p>FORMULA: C15H22N6O5S</p>
<p>CHARGE: 0</p>
</body>
</notes>
</species>
<species id="M_METPPHOL_c" compartment="c" hasOnlySubstanceUnits="false" boundaryCondition="false" constant="false">
<notes>
<body xmlns="http://www.w3.org/1999/xhtml">
<p>FORMULA: </p>
<p>CHARGE: 0</p>
</body>
</notes>
</species>
<species id="M_SAHC_c" name="S-Adenosyl-L-homocysteine" compartment="c" hasOnlySubstanceUnits="false" boundaryCondition="false" constant="false">

<notes>

<body xmlns="http://www.w3.org/1999/xhtml">

<p>FORMULA: C14H20N6O5S</p>

<p>CHARGE: 0</p>

</body>

</notes>

</species>

<species id="M_PPRE_MOX_BNZQ_c" name="2-Polyprenyl-6-methoxy-1,4-benzoquinone" compartment="c" hasOnlySubstanceUnits="false" boundaryCondition="false" constant="false">

<notes>

<body xmlns="http://www.w3.org/1999/xhtml">

<p>FORMULA: C12H14O3(C5H8)n</p>

<p>CHARGE: 0</p>

</body>

</notes>

</species>

<species id="M_DEMQH2_c" name="3-Demethylubiquinol" compartment="c" hasOnlySubstanceUnits="false" boundaryCondition="false" constant="false">

<notes>

<body xmlns="http://www.w3.org/1999/xhtml">

<p>FORMULA: </p>

<p>CHARGE: 0</p>

</body>

</notes>

</species>

<species id="M_PPRE_MMOX_BNZQ_c" name="2-Polyprenyl-3-methyl-6-methoxy-1,4-benzoquinone" compartment="c" hasOnlySubstanceUnits="false" boundaryCondition="false" constant="false">

<notes>

<body xmlns="http://www.w3.org/1999/xhtml">

<p>FORMULA: C13H16O3(C5H8)n</p>

<p>CHARGE: 0</p>

</body>

</notes>

</species>

<species id="M_DMMQ_c" name="2-Demethylmenaquinone" compartment="c" hasOnlySubstanceUnits="false" boundaryCondition="false" constant="false">

<notes>

<body xmlns="http://www.w3.org/1999/xhtml">

<p>FORMULA: C15H14O2(C5H8)n</p>

<p>CHARGE: 0</p>

</body>

</notes>

</species>

<species id="M_MQ_c" name="Menaquinone" compartment="c" hasOnlySubstanceUnits="false" boundaryCondition="false" constant="false">

<notes>

<body xmlns="http://www.w3.org/1999/xhtml">

<p>FORMULA: C16H16O2(C5H8)n</p>

<p>CHARGE: 0</p>

</body>

</notes>

</species>

<species id="M_HY_SUCC_CYCHEXDE_CARBA_c" name="(1R,6R)-6-Hydroxy-2-succinylcyclohexa-2,4-diene-1-carboxylate (SHCHC)" compartment="c" hasOnlySubstanceUnits="false" boundaryCondition="false" constant="false">

<notes>

<body xmlns="http://www.w3.org/1999/xhtml">

<p>FORMULA: C11H12O6</p>

<p>CHARGE: 0</p>

</body>

</notes>

</species>

<species id="M_SUCC_BNZA_c" name="2-Succinylbenzoate" compartment="c" hasOnlySubstanceUnits="false" boundaryCondition="false" constant="false">

<notes>

<body xmlns="http://www.w3.org/1999/xhtml">

<p>FORMULA: C11H10O5</p>

<p>CHARGE: 0</p>

</body>

</notes>

</species>

<species id="M_CBTN_CCP_c" name="Carboxybiotin-carboxyl-carrier protein" compartment="c" hasOnlySubstanceUnits="false" boundaryCondition="false" constant="false">

<notes>

<body xmlns="http://www.w3.org/1999/xhtml">

<p>FORMULA: C18H26N5O6SR2</p>

<p>CHARGE: 0</p>

</body>

</notes>

</species>

<species id="M_MALCoA_c" name="Malonyl-CoA" compartment="c" hasOnlySubstanceUnits="false" boundaryCondition="false" constant="false">

<notes>

<body xmlns="http://www.w3.org/1999/xhtml">

<p>FORMULA: C24H38N7O19P3S</p>

<p>CHARGE: 0</p>

</body>

</notes>

</species>

<species id="M_HCARB_c" name="Holo-[carboxylase]" compartment="c" hasOnlySubstanceUnits="false" boundaryCondition="false" constant="false">

<notes>
<body xmlns="http://www.w3.org/1999/xhtml">
<p>FORMULA: C17H27N5O4SR2</p>
<p>CHARGE: 0</p>
</body>
</notes>
</species>
<species id="M_GLUTRCoA_c" name="Glutaryl-CoA" compartment="c" hasOnlySubstanceUnits="false" boundaryCondition="false" constant="false">
<notes>
<body xmlns="http://www.w3.org/1999/xhtml">
<p>FORMULA: C26H42N7O19P3S</p>
<p>CHARGE: 0</p>
</body>
</notes>
</species>
<species id="M_CROCoA_c" name="Crotonoyl-CoA" compartment="c" hasOnlySubstanceUnits="false" boundaryCondition="false" constant="false">
<notes>
<body xmlns="http://www.w3.org/1999/xhtml">
<p>FORMULA: C25H40N7O17P3S</p>
<p>CHARGE: 0</p>
</body>
</notes>
</species>
<species id="M_H_e" name="Hydron" compartment="e" hasOnlySubstanceUnits="false" boundaryCondition="false" constant="false">
<notes>
<body xmlns="http://www.w3.org/1999/xhtml">
<p>FORMULA: H</p>

<p>CHARGE: 0</p>

</body>

</notes>

</species>

<species id="M_CYT_C3_c" name="Ferricytochrome c" compartment="c" hasOnlySubstanceUnits="false" boundaryCondition="false" constant="false">

<notes>

<body xmlns="http://www.w3.org/1999/xhtml">

<p>FORMULA: C42H44FeN8O8S2R4</p>

<p>CHARGE: 0</p>

</body>

</notes>

</species>

<species id="M_CYT_C2_c" name="Ferrocytochrome c" compartment="c" hasOnlySubstanceUnits="false" boundaryCondition="false" constant="false">

<notes>

<body xmlns="http://www.w3.org/1999/xhtml">

<p>FORMULA: C42H44FeN8O8S2R4</p>

<p>CHARGE: 0</p>

</body>

</notes>

</species>

<species id="M_ADP_RIB_c" name="ADP-D-ribose" compartment="c" hasOnlySubstanceUnits="false" boundaryCondition="false" constant="false">

<notes>

<body xmlns="http://www.w3.org/1999/xhtml">

<p>FORMULA: C15H23N5O14P2</p>

<p>CHARGE: 0</p>

</body>

</notes>

</species>

<species id="M_PRIBSAMN_c" name="5-Phosphoribosylamine" compartment="c" hasOnlySubstanceUnits="false" boundaryCondition="false" constant="false">

<notes>

<body xmlns="http://www.w3.org/1999/xhtml">

<p>FORMULA: C5H12NO7P</p>

<p>CHARGE: 0</p>

</body>

</notes>

</species>

<species id="M_GAR_c" name="5'-Phosphoribosylglycinamide (GAR)" compartment="c" hasOnlySubstanceUnits="false" boundaryCondition="false" constant="false">

<notes>

<body xmlns="http://www.w3.org/1999/xhtml">

<p>FORMULA: C7H15N2O8P</p>

<p>CHARGE: 0</p>

</body>

</notes>

</species>

<species id="M_FGAR_c" name="5'-Phosphoribosyl-N-formylglycinamide (FGAR)" compartment="c" hasOnlySubstanceUnits="false" boundaryCondition="false" constant="false">

<notes>

<body xmlns="http://www.w3.org/1999/xhtml">

<p>FORMULA: C8H15N2O9P</p>

<p>CHARGE: 0</p>

</body>

</notes>

</species>

<species id="M_FGAM_c" name="2-(Formamido)-N1-(5'-phosphoribosyl)acetamidine" compartment="c" hasOnlySubstanceUnits="false" boundaryCondition="false" constant="false">

<notes>

<body xmlns="http://www.w3.org/1999/xhtml">

<p>FORMULA: C8H16N3O8P</p>

<p>CHARGE: 0</p>

</body>

</notes>

</species>

<species id="M_AIR_c" name="Aminoimidazole ribotide (AIR)" compartment="c" hasOnlySubstanceUnits="false" boundaryCondition="false" constant="false">

<notes>

<body xmlns="http://www.w3.org/1999/xhtml">

<p>FORMULA: C8H14N3O7P</p>

<p>CHARGE: 0</p>

</body>

</notes>

</species>

<species id="M_HCO3_c" name="Bicarbonate" compartment="c" hasOnlySubstanceUnits="false" boundaryCondition="false" constant="false">

<notes>

<body xmlns="http://www.w3.org/1999/xhtml">

<p>FORMULA: HCO3</p>

<p>CHARGE: 0</p>

</body>

</notes>

</species>

<species id="M_5CAIZ_c" name="5-Carboxyamino-1-(5-phospho-D-ribosyl)imidazole" compartment="c" hasOnlySubstanceUnits="false" boundaryCondition="false" constant="false">

<notes>

<body xmlns="http://www.w3.org/1999/xhtml">

<p>FORMULA: C9H14N3O9P</p>

<p>CHARGE: 0</p>

</body>

</notes>

</species>

<species id="M_CAIR_c" name="1-(5-Phospho-D-ribosyl)-5-amino-4-imidazolecarboxylate" compartment="c" hasOnlySubstanceUnits="false" boundaryCondition="false" constant="false">

<notes>

<body xmlns="http://www.w3.org/1999/xhtml">

<p>FORMULA: C9H14N3O9P</p>

<p>CHARGE: 0</p>

</body>

</notes>

</species>

<species id="M_SAICAR_c" name="1-(5'-Phosphoribosyl)-5-amino-4-(N-succinocarboxamide)-imidazole (SAICAR)" compartment="c" hasOnlySubstanceUnits="false" boundaryCondition="false" constant="false">

<notes>

<body xmlns="http://www.w3.org/1999/xhtml">

<p>FORMULA: C13H19N4O12P</p>

<p>CHARGE: 0</p>

</body>

</notes>

</species>

<species id="M_AICAR_c" name="1-(5'-Phosphoribosyl)-5-amino-4-imidazolecarboxamide" compartment="c" hasOnlySubstanceUnits="false" boundaryCondition="false" constant="false">

<notes>

<body xmlns="http://www.w3.org/1999/xhtml">

<p>FORMULA: C9H15N4O8P</p>

<p>CHARGE: 0</p>

</body>

</notes>

</species>

<species id="M_5A_4IMDZCARBA_c" name="5-Amino-4-imidazolecarboxyamide" compartment="c" hasOnlySubstanceUnits="false" boundaryCondition="false" constant="false">

<notes>

<body xmlns="http://www.w3.org/1999/xhtml">

<p>FORMULA: C4H6N4O</p>

<p>CHARGE: 0</p>

</body>

</notes>

</species>

<species id="M_10F_THF_c" name="10-Formyltetrahydrofolate" compartment="c" hasOnlySubstanceUnits="false" boundaryCondition="false" constant="false">

<notes>

<body xmlns="http://www.w3.org/1999/xhtml">

<p>FORMULA: C20H23N7O7</p>

<p>CHARGE: 0</p>

</body>

</notes>

</species>

<species id="M_THF_c" name="5,6,7,8-Tetrahydrofolate" compartment="c" hasOnlySubstanceUnits="false" boundaryCondition="false" constant="false">

<notes>

<body xmlns="http://www.w3.org/1999/xhtml">

<p>FORMULA: C19H23N7O6</p>

<p>CHARGE: 0</p>

</body>

</notes>

</species>

<species id="M_FAICAR_c" name="1-(5'-Phosphoribosyl)-5-formamido-4-imidazolecarboxamide (FAICAR)" compartment="c" hasOnlySubstanceUnits="false" boundaryCondition="false" constant="false">

<notes>

<body xmlns="http://www.w3.org/1999/xhtml">

<p>FORMULA: C10H15N4O9P</p>

<p>CHARGE: 0</p>

</body>

</notes>

</species>

<species id="M_IMP_c" name="Inosine monophosphate (IMP)" compartment="c" hasOnlySubstanceUnits="false" boundaryCondition="false" constant="false">

<notes>

<body xmlns="http://www.w3.org/1999/xhtml">

<p>FORMULA: C10H13N4O8P</p>

<p>CHARGE: 0</p>

</body>

</notes>

</species>

<species id="M_BADNS_PPP_c" name="P1,P4-Bis(5'-adenosyl) tetraphosphate (AppppA)" compartment="c" hasOnlySubstanceUnits="false" boundaryCondition="false" constant="false">

<notes>

<body xmlns="http://www.w3.org/1999/xhtml">

<p>FORMULA: C20H28N10O19P4</p>

<p>CHARGE: 0</p>

</body>

</notes>

</species>

<species id="M_SO4_c" name="Sulfate" compartment="c" hasOnlySubstanceUnits="false" boundaryCondition="false" constant="false">

<notes>

<body xmlns="http://www.w3.org/1999/xhtml">

<p>FORMULA: H2SO4</p>

<p>CHARGE: 0</p>

</body>

</notes>

</species>

<species id="M_APS_c" name="Adenylyl sulfate" compartment="c" hasOnlySubstanceUnits="false" boundaryCondition="false" constant="false">

<notes>

<body xmlns="http://www.w3.org/1999/xhtml">

<p>FORMULA: C10H14N5O10PS</p>

<p>CHARGE: 0</p>

</body>

</notes>

</species>

<species id="M_dIDP_c" name="2'-Deoxyinosine-5'-diphosphate (dIDP)" compartment="c" hasOnlySubstanceUnits="false" boundaryCondition="false" constant="false">

<notes>

<body xmlns="http://www.w3.org/1999/xhtml">

<p>FORMULA: C10H14N4O10P2</p>

<p>CHARGE: 0</p>

</body>

</notes>

</species>

<species id="M_dITP_c" name="2'-Deoxyinosine-5'-triphosphate (dITP)" compartment="c" hasOnlySubstanceUnits="false" boundaryCondition="false" constant="false">

<notes>

<body xmlns="http://www.w3.org/1999/xhtml">

<p>FORMULA: C10H15N4O13P3</p>

<p>CHARGE: 0</p>

</body>

</notes>

</species>

<species id="M_dIMP_c" name="2'-Deoxyinosine 5'-phosphate (dIMP)" compartment="c" hasOnlySubstanceUnits="false" boundaryCondition="false" constant="false">

<notes>

<body xmlns="http://www.w3.org/1999/xhtml">

<p>FORMULA: C10H13N4O7P</p>

<p>CHARGE: 0</p>

</body>

</notes>

</species>

<species id="M_GUNS_3PPi_5TPi_c" name="Guanosine 3'-diphosphate 5'-triphosphate" compartment="c" hasOnlySubstanceUnits="false" boundaryCondition="false" constant="false">

<notes>

<body xmlns="http://www.w3.org/1999/xhtml">

<p>FORMULA: C10H18N5O20P5</p>

<p>CHARGE: 0</p>

</body>

</notes>

</species>

<species id="M_GUNSBP_c" name="Guanosine 3'-diphosphate 5'-diphosphate" compartment="c" hasOnlySubstanceUnits="false" boundaryCondition="false" constant="false">

<notes>

<body xmlns="http://www.w3.org/1999/xhtml">

<p>FORMULA: C10H17N5O17P4</p>

<p>CHARGE: 0</p>

</body>

</notes>

</species>

<species id="M_GMP_c" name="Guanosine 5'-phosphate (GMP)" compartment="c" hasOnlySubstanceUnits="false" boundaryCondition="false" constant="false">

<notes>

<body xmlns="http://www.w3.org/1999/xhtml">

<p>FORMULA: C10H14N5O8P</p>

<p>CHARGE: 0</p>

</body>

</notes>

</species>

<species id="M_BGUNS_PPP_c" name="P1,P4-Bis(5'-guanosyl) tetraphosphate (GppppG)" compartment="c" hasOnlySubstanceUnits="false" boundaryCondition="false" constant="false">

<notes>

<body xmlns="http://www.w3.org/1999/xhtml">

<p>FORMULA: C20H28N10O21P4</p>

<p>CHARGE: 0</p>

</body>

</notes>

</species>

<species id="M_dGUNS_c" name="Deoxyguanosine" compartment="c" hasOnlySubstanceUnits="false" boundaryCondition="false" constant="false">

<notes>

<body xmlns="http://www.w3.org/1999/xhtml">

<p>FORMULA: C10H13N5O4</p>

<p>CHARGE: 0</p>

</body>

</notes>

</species>

<species id="M_TPi_c" name="Triphosphate" compartment="c" hasOnlySubstanceUnits="false" boundaryCondition="false" constant="false">

<notes>

<body xmlns="http://www.w3.org/1999/xhtml">

<p>FORMULA: P3H5O10</p>

<p>CHARGE: 0</p>

</body>

</notes>

</species>

<species id="M_dGDP_c" name="2'-Deoxyguanosine 5'-diphosphate (dGDP)" compartment="c" hasOnlySubstanceUnits="false" boundaryCondition="false" constant="false">

<notes>

<body xmlns="http://www.w3.org/1999/xhtml">

<p>FORMULA: C10H15N5O10P2</p>

<p>CHARGE: 0</p>

</body>

</notes>

</species>

<species id="M_dGMP_c" name="Deoxyguanosine monophosphate" compartment="c" hasOnlySubstanceUnits="false" boundaryCondition="false" constant="false">

<notes>

<body xmlns="http://www.w3.org/1999/xhtml">

<p>FORMULA: C10H14N5O7P</p>

<p>CHARGE: 0</p>

</body>

</notes>

</species>

<species id="M_R_TRED_c" name="Reduced thioredoxin" compartment="c" hasOnlySubstanceUnits="false" boundaryCondition="false" constant="false">

<notes>

<body xmlns="http://www.w3.org/1999/xhtml">

<p>FORMULA: C10H14N4O4S2R4</p>

<p>CHARGE: 0</p>

</body>

</notes>

</species>

<species id="M_O_TRED_c" name="Oxidized thioredoxin" compartment="c" hasOnlySubstanceUnits="false" boundaryCondition="false" constant="false">

<notes>

<body xmlns="http://www.w3.org/1999/xhtml">

<p>FORMULA: C10H12N4O4S2R4</p>

<p>CHARGE: 0</p>

</body>

</notes>

</species>

<species id="M_GUN_c" name="Guanine" compartment="c" hasOnlySubstanceUnits="false" boundaryCondition="false" constant="false">

<notes>

<body xmlns="http://www.w3.org/1999/xhtml">

<p>FORMULA: C5H5N5O</p>

<p>CHARGE: 0</p>

</body>

</notes>

</species>

<species id="M_GUNS_c" name="Guanosine" compartment="c" hasOnlySubstanceUnits="false" boundaryCondition="false" constant="false">

<notes>

<body xmlns="http://www.w3.org/1999/xhtml">

<p>FORMULA: C10H13N5O5</p>

<p>CHARGE: 0</p>

</body>

</notes>

</species>

<species id="M_XMP_c" name="Xanthosine 5'-phosphate (XMP)" compartment="c" hasOnlySubstanceUnits="false" boundaryCondition="false" constant="false">

<notes>
<body xmlns="http://www.w3.org/1999/xhtml">
<p>FORMULA: C10H13N4O9P</p>
<p>CHARGE: 0</p>
</body>
</notes>
</species>
<species id="M_NH4_c" name="Ammonia" compartment="c" hasOnlySubstanceUnits="false" boundaryCondition="false" constant="false">
<notes>
<body xmlns="http://www.w3.org/1999/xhtml">
<p>FORMULA: NH4</p>
<p>CHARGE: 0</p>
</body>
</notes>
</species>
<species id="M_XppppX_c" name="P1,P4-Bis(5'-xanthosyl) tetraphosphate (XppppX)" compartment="c" hasOnlySubstanceUnits="false" boundaryCondition="false" constant="false">
<notes>
<body xmlns="http://www.w3.org/1999/xhtml">
<p>FORMULA: C20H26N8O23P4</p>
<p>CHARGE: 0</p>
</body>
</notes>
</species>
<species id="M_XTP_c" name="XTP" compartment="c" hasOnlySubstanceUnits="false" boundaryCondition="false" constant="false">
<notes>
<body xmlns="http://www.w3.org/1999/xhtml">
<p>FORMULA: C10H15N4O15P3</p>

<p>CHARGE: 0</p>

</body>

</notes>

</species>

<species id="M_ITP_c" name="Inosine 5'-triphosphate (ITP)" compartment="c" hasOnlySubstanceUnits="false" boundaryCondition="false" constant="false">

<notes>

<body xmlns="http://www.w3.org/1999/xhtml">

<p>FORMULA: C10H15N4O14P3</p>

<p>CHARGE: 0</p>

</body>

</notes>

</species>

<species id="M_IDP_c" name="Inosine 5'-diphosphate (IDP)" compartment="c" hasOnlySubstanceUnits="false" boundaryCondition="false" constant="false">

<notes>

<body xmlns="http://www.w3.org/1999/xhtml">

<p>FORMULA: C10H14N4O11P2</p>

<p>CHARGE: 0</p>

</body>

</notes>

</species>

<species id="M_XNTHS_c" name="Xanthosine" compartment="c" hasOnlySubstanceUnits="false" boundaryCondition="false" constant="false">

<notes>

<body xmlns="http://www.w3.org/1999/xhtml">

<p>FORMULA: C10H12N4O6</p>

<p>CHARGE: 0</p>

</body>

</notes>

</species>

<species id="M_XTHN_c" name="Xanthine" compartment="c" hasOnlySubstanceUnits="false" boundaryCondition="false" constant="false">

<notes>

<body xmlns="http://www.w3.org/1999/xhtml">

<p>FORMULA: C5H4N4O2</p>

<p>CHARGE: 0</p>

</body>

</notes>

</species>

<species id="M_HXTHN_c" name="Hypoxanthine" compartment="c" hasOnlySubstanceUnits="false" boundaryCondition="false" constant="false">

<notes>

<body xmlns="http://www.w3.org/1999/xhtml">

<p>FORMULA: C5H4N4O</p>

<p>CHARGE: 0</p>

</body>

</notes>

</species>

<species id="M_INSN_c" name="Inosine" compartment="c" hasOnlySubstanceUnits="false" boundaryCondition="false" constant="false">

<notes>

<body xmlns="http://www.w3.org/1999/xhtml">

<p>FORMULA: C10H12N4O5</p>

<p>CHARGE: 0</p>

</body>

</notes>

</species>

<species id="M_ADN_SUCC_c" name="Adenylosuccinate" compartment="c" hasOnlySubstanceUnits="false" boundaryCondition="false" constant="false">

<notes>

<body xmlns="http://www.w3.org/1999/xhtml">

<p>FORMULA: C14H18N5O11P</p>

<p>CHARGE: 0</p>

</body>

</notes>

</species>

<species id="M_ADSN_c" name="Adenosine" compartment="c" hasOnlySubstanceUnits="false" boundaryCondition="false" constant="false">

<notes>

<body xmlns="http://www.w3.org/1999/xhtml">

<p>FORMULA: C10H13N5O4</p>

<p>CHARGE: 0</p>

</body>

</notes>

</species>

<species id="M_ADN_c" name="Adenine" compartment="c" hasOnlySubstanceUnits="false" boundaryCondition="false" constant="false">

<notes>

<body xmlns="http://www.w3.org/1999/xhtml">

<p>FORMULA: C5H5N5</p>

<p>CHARGE: 0</p>

</body>

</notes>

</species>

<species id="M_zCARO_c" name="zeta-Carotene" compartment="c" hasOnlySubstanceUnits="false" boundaryCondition="false" constant="false">

<notes>

<body xmlns="http://www.w3.org/1999/xhtml">

<p>FORMULA: C40H60</p>

<p>CHARGE: 0</p>

</body>

</notes>

</species>

<species id="M_dADS_c" name="Deoxyadenosine" compartment="c" hasOnlySubstanceUnits="false" boundaryCondition="false" constant="false">

<notes>

<body xmlns="http://www.w3.org/1999/xhtml">

<p>FORMULA: C10H13N5O3</p>

<p>CHARGE: 0</p>

</body>

</notes>

</species>

<species id="M_dADP_c" name="2'-Deoxyadenosine 5'-diphosphate (dADP)" compartment="c" hasOnlySubstanceUnits="false" boundaryCondition="false" constant="false">

<notes>

<body xmlns="http://www.w3.org/1999/xhtml">

<p>FORMULA: C10H15N5O9P2</p>

<p>CHARGE: 0</p>

</body>

</notes>

</species>

<species id="M_dAMP_c" name="Deoxyadenosine monophosphate (dAMP)" compartment="c" hasOnlySubstanceUnits="false" boundaryCondition="false" constant="false">

<notes>

<body xmlns="http://www.w3.org/1999/xhtml">

<p>FORMULA: C10H14N5O6P</p>

<p>CHARGE: 0</p>

</body>

</notes>

```
      </species>
      <species id="M_CARB_Pi_c" name="Carbamoyl phosphate" compartment="c" hasOnlySubstanceUnits="false" boundaryCondition="false" constant="false">
        <notes>
          <body xmlns="http://www.w3.org/1999/xhtml">
            <p>FORMULA: CH4NO5P</p>
            <p>CHARGE: 0</p>
          </body>
        </notes>
      </species>
      <species id="M_CARB_ASP_c" name="N-Carbamoyl-L-aspartate" compartment="c" hasOnlySubstanceUnits="false" boundaryCondition="false" constant="false">
        <notes>
          <body xmlns="http://www.w3.org/1999/xhtml">
            <p>FORMULA: C5H8N2O5</p>
            <p>CHARGE: 0</p>
          </body>
        </notes>
      </species>
      <species id="M_DHY_ORTA_c" name="(S)-Dihydroorotate" compartment="c" hasOnlySubstanceUnits="false" boundaryCondition="false" constant="false">
        <notes>
          <body xmlns="http://www.w3.org/1999/xhtml">
            <p>FORMULA: C5H6N2O4</p>
            <p>CHARGE: 0</p>
          </body>
        </notes>
      </species>
      <species id="M_dUTP_c" name="2&apos;-Deoxyuridine 5&apos;-triphosphate (dUTP)" compartment="c" hasOnlySubstanceUnits="false" boundaryCondition="false" constant="false">
```

<notes>

<body xmlns="http://www.w3.org/1999/xhtml">

<p>FORMULA: C9H15N2O14P3</p>

<p>CHARGE: 0</p>

</body>

</notes>

</species>

<species id="M_ORTD5P_c" name="Orotidine 5'-phosphate" compartment="c" hasOnlySubstanceUnits="false" boundaryCondition="false" constant="false">

<notes>

<body xmlns="http://www.w3.org/1999/xhtml">

<p>FORMULA: C10H13N2O11P</p>

<p>CHARGE: 0</p>

</body>

</notes>

</species>

<species id="M_ORTA_c" name="Orotate" compartment="c" hasOnlySubstanceUnits="false" boundaryCondition="false" constant="false">

<notes>

<body xmlns="http://www.w3.org/1999/xhtml">

<p>FORMULA: C5H4N2O4</p>

<p>CHARGE: 0</p>

</body>

</notes>

</species>

<species id="M_UppppU_c" name="P1,P4-Bis(5'-uridyl) tetraphosphate (UppppU)" compartment="c" hasOnlySubstanceUnits="false" boundaryCondition="false" constant="false">

<notes>

<body xmlns="http://www.w3.org/1999/xhtml">

<p>FORMULA: C18H26N4O23P4</p>

<p>CHARGE: 0</p>

</body>

</notes>

</species>

<species id="M_UMP_c" name="Uridine 5'-monophosphate (UMP)" compartment="c" hasOnlySubstanceUnits="false" boundaryCondition="false" constant="false">

<notes>

<body xmlns="http://www.w3.org/1999/xhtml">

<p>FORMULA: C9H13N2O9P</p>

<p>CHARGE: 0</p>

</body>

</notes>

</species>

<species id="M_UDP_c" name="Uridine 5'-diphosphate (UDP)" compartment="c" hasOnlySubstanceUnits="false" boundaryCondition="false" constant="false">

<notes>

<body xmlns="http://www.w3.org/1999/xhtml">

<p>FORMULA: C9H14N2O12P2</p>

<p>CHARGE: 0</p>

</body>

</notes>

</species>

<species id="M_CDP_c" name="Cytidine 5'-diphosphate (CDP)" compartment="c" hasOnlySubstanceUnits="false" boundaryCondition="false" constant="false">

<notes>

<body xmlns="http://www.w3.org/1999/xhtml">

<p>FORMULA: C9H15N3O11P2</p>

<p>CHARGE: 0</p>

</body>

</notes>

</species>

<species id="M_dCDP_c" name="2'-Deoxycytidine diphosphate (dCDP)" compartment="c" hasOnlySubstanceUnits="false" boundaryCondition="false" constant="false">

<notes>

<body xmlns="http://www.w3.org/1999/xhtml">

<p>FORMULA: C9H15N3O10P2</p>

<p>CHARGE: 0</p>

</body>

</notes>

</species>

<species id="M_dUDP_c" name="2'-Deoxyuridine 5'-diphosphate (dUDP)" compartment="c" hasOnlySubstanceUnits="false" boundaryCondition="false" constant="false">

<notes>

<body xmlns="http://www.w3.org/1999/xhtml">

<p>FORMULA: C9H14N2O11P2</p>

<p>CHARGE: 0</p>

</body>

</notes>

</species>

<species id="M_dTDP_c" name="Deoxythymidine 5'-diphosphate (dTDP)" compartment="c" hasOnlySubstanceUnits="false" boundaryCondition="false" constant="false">

<notes>

<body xmlns="http://www.w3.org/1999/xhtml">

<p>FORMULA: C10H16N2O11P2</p>

<p>CHARGE: 0</p>

</body>

</notes>

</species>

<species id="M_dUMP_c" name="Deoxyuridine 5'-phosphate (dUMP)" compartment="c" hasOnlySubstanceUnits="false" boundaryCondition="false" constant="false">

```
        <notes>
          <body xmlns="http://www.w3.org/1999/xhtml">
            <p>FORMULA: C9H13N2O8P</p>
            <p>CHARGE: 0</p>
          </body>
        </notes>
      </species>
      <species id="M_CMP_c" name="Cytidine-5&apos;-monophosphate (CMP)" compartment="c" hasOnlySubstanceUnits="false" boundaryCondition="false" constant="false">
        <notes>
          <body xmlns="http://www.w3.org/1999/xhtml">
            <p>FORMULA: C9H14N3O8P</p>
            <p>CHARGE: 0</p>
          </body>
        </notes>
      </species>
      <species id="M_dCMP_c" name="Deoxycytidine monophosphate" compartment="c" hasOnlySubstanceUnits="false" boundaryCondition="false" constant="false">
        <notes>
          <body xmlns="http://www.w3.org/1999/xhtml">
            <p>FORMULA: C9H14N3O7P</p>
            <p>CHARGE: 0</p>
          </body>
        </notes>
      </species>
      <species id="M_dTMP_c" name="Thymidine 5&apos;-phosphat (Dtmp)" compartment="c" hasOnlySubstanceUnits="false" boundaryCondition="false" constant="false">
        <notes>
          <body xmlns="http://www.w3.org/1999/xhtml">
            <p>FORMULA: C10H15N2O8P</p>
```

<p>CHARGE: 0</p>
</body>
</notes>
</species>
<species id="M_URD_c" name="Uridine" compartment="c" hasOnlySubstanceUnits="false" boundaryCondition="false" constant="false">
<notes>
<body xmlns="http://www.w3.org/1999/xhtml">
<p>FORMULA: C9H12N2O6</p>
<p>CHARGE: 0</p>
</body>
</notes>
</species>
<species id="M_CYTD_c" name="Cytidine" compartment="c" hasOnlySubstanceUnits="false" boundaryCondition="false" constant="false">
<notes>
<body xmlns="http://www.w3.org/1999/xhtml">
<p>FORMULA: C9H13N3O5</p>
<p>CHARGE: 0</p>
</body>
</notes>
</species>
<species id="M_FMN_c" name="Flavin mononucleotide (FMN)" compartment="c" hasOnlySubstanceUnits="false" boundaryCondition="false" constant="false">
<notes>
<body xmlns="http://www.w3.org/1999/xhtml">
<p>FORMULA: C17H21N4O9P</p>
<p>CHARGE: 0</p>
</body>
</notes>

</species>

<species id="M_FMNH2_c" name="Reduced FMN (FMNH2)" compartment="c" hasOnlySubstanceUnits="false" boundaryCondition="false" constant="false">

<notes>

<body xmlns="http://www.w3.org/1999/xhtml">

<p>FORMULA: C17H23N4O9P</p>

<p>CHARGE: 0</p>

</body>

</notes>

</species>

<species id="M_THYMD_c" name="Thymidine" compartment="c" hasOnlySubstanceUnits="false" boundaryCondition="false" constant="false">

<notes>

<body xmlns="http://www.w3.org/1999/xhtml">

<p>FORMULA: C10H14N2O5</p>

<p>CHARGE: 0</p>

</body>

</notes>

</species>

<species id="M_dCYTD_c" name="2'-Deoxycytidine" compartment="c" hasOnlySubstanceUnits="false" boundaryCondition="false" constant="false">

<notes>

<body xmlns="http://www.w3.org/1999/xhtml">

<p>FORMULA: C9H13N3O4</p>

<p>CHARGE: 0</p>

</body>

</notes>

</species>

<species id="M_URA_c" name="Uracil" compartment="c" hasOnlySubstanceUnits="false" boundaryCondition="false" constant="false">

<notes>

<body xmlns="http://www.w3.org/1999/xhtml">

<p>FORMULA: C4H4N2O2</p>

<p>CHARGE: 0</p>

</body>

</notes>

</species>

<species id="M_dURD_c" name="Deoxyuridine" compartment="c" hasOnlySubstanceUnits="false" boundaryCondition="false" constant="false">

<notes>

<body xmlns="http://www.w3.org/1999/xhtml">

<p>FORMULA: C9H12N2O5</p>

<p>CHARGE: 0</p>

</body>

</notes>

</species>

<species id="M_5_10_MNTHF_c" name="5,10-Methylenetetrahydrofolate" compartment="c" hasOnlySubstanceUnits="false" boundaryCondition="false" constant="false">

<notes>

<body xmlns="http://www.w3.org/1999/xhtml">

<p>FORMULA: C20H23N7O6</p>

<p>CHARGE: 0</p>

</body>

</notes>

</species>

<species id="M_THYM_c" name="Thymine" compartment="c" hasOnlySubstanceUnits="false" boundaryCondition="false" constant="false">

<notes>

<body xmlns="http://www.w3.org/1999/xhtml">

<p>FORMULA: C5H6N2O2</p>

<p>CHARGE: 0</p>

</body>

</notes>

</species>

<species id="M_DHTHYM_c" name="5,6-dihydrothymine" compartment="c" hasOnlySubstanceUnits="false" boundaryCondition="false" constant="false">

<notes>

<body xmlns="http://www.w3.org/1999/xhtml">

<p>FORMULA: C5H8N2O2</p>

<p>CHARGE: 0</p>

</body>

</notes>

</species>

<species id="M_UREIDO_BUTR_c" name="3-Ureidoisobutyrate" compartment="c" hasOnlySubstanceUnits="false" boundaryCondition="false" constant="false">

<notes>

<body xmlns="http://www.w3.org/1999/xhtml">

<p>FORMULA: C5H10N2O3</p>

<p>CHARGE: 0</p>

</body>

</notes>

</species>

<species id="M_3AIBUTNA_c" name="3-Aminoisobutanoate" compartment="c" hasOnlySubstanceUnits="false" boundaryCondition="false" constant="false">

<notes>

<body xmlns="http://www.w3.org/1999/xhtml">

<p>FORMULA: C4H9NO2</p>

<p>CHARGE: 0</p>

</body>

</notes>

</species>

<species id="M_GLOXT_c" name="Glyoxylate" compartment="c" hasOnlySubstanceUnits="false" boundaryCondition="false" constant="false">

<notes>

<body xmlns="http://www.w3.org/1999/xhtml">

<p>FORMULA: C2H2O3</p>

<p>CHARGE: 0</p>

</body>

</notes>

</species>

<species id="M_CTRLN_c" name="L-Citrulline" compartment="c" hasOnlySubstanceUnits="false" boundaryCondition="false" constant="false">

<notes>

<body xmlns="http://www.w3.org/1999/xhtml">

<p>FORMULA: C6H13N3O3</p>

<p>CHARGE: 0</p>

</body>

</notes>

</species>

<species id="M_L_ARG_SUCC_c" name="L-Argininosuccinate" compartment="c" hasOnlySubstanceUnits="false" boundaryCondition="false" constant="false">

<notes>

<body xmlns="http://www.w3.org/1999/xhtml">

<p>FORMULA: C10H18N4O6</p>

<p>CHARGE: 0</p>

</body>

</notes>

</species>

<species id="M_SUCC_SA_c" name="Succinate semialdehyde" compartment="c" hasOnlySubstanceUnits="false" boundaryCondition="false" constant="false">

```
        <notes>
          <body xmlns="http://www.w3.org/1999/xhtml">
            <p>FORMULA: C4H6O3</p>
            <p>CHARGE: 0</p>
          </body>
        </notes>
      </species>
      <species id="M_4ABUT_c" name="4-Aminobutanoate" compartment="c" hasOnlySubstanceUnits="false"
boundaryCondition="false" constant="false">
        <notes>
          <body xmlns="http://www.w3.org/1999/xhtml">
            <p>FORMULA: C4H9NO2</p>
            <p>CHARGE: 0</p>
          </body>
        </notes>
      </species>
      <species id="M_PYRR5CARB_c" name="L-1-Pyrroline-5-carboxylate" compartment="c" hasOnlySubstanceUnits="false"
boundaryCondition="false" constant="false">
        <notes>
          <body xmlns="http://www.w3.org/1999/xhtml">
            <p>FORMULA: C5H7NO2</p>
            <p>CHARGE: 0</p>
          </body>
        </notes>
      </species>
      <species id="M_GLUSAP_c" name="D-Glucosamine 6-phosphate" compartment="c" hasOnlySubstanceUnits="false"
boundaryCondition="false" constant="false">
        <notes>
          <body xmlns="http://www.w3.org/1999/xhtml">
            <p>FORMULA: C6H14NO8P</p>
```

<p>CHARGE: 0</p>

</body>

</notes>

</species>

<species id="M_HY_PYR_c" name="Hydroxypyruvate" compartment="c" hasOnlySubstanceUnits="false" boundaryCondition="false" constant="false">

<notes>

<body xmlns="http://www.w3.org/1999/xhtml">

<p>FORMULA: C3H4O4</p>

<p>CHARGE: 0</p>

</body>

</notes>

</species>

<species id="M_3POXPYR_c" name="3-Phosphonooxypyruvate" compartment="c" hasOnlySubstanceUnits="false" boundaryCondition="false" constant="false">

<notes>

<body xmlns="http://www.w3.org/1999/xhtml">

<p>FORMULA: C3H5O7P</p>

<p>CHARGE: 0</p>

</body>

</notes>

</species>

<species id="M_IND_GLYP_c" name="Indoleglycerol phosphate" compartment="c" hasOnlySubstanceUnits="false" boundaryCondition="false" constant="false">

<notes>

<body xmlns="http://www.w3.org/1999/xhtml">

<p>FORMULA: C11H14NO6P</p>

<p>CHARGE: 0</p>

</body>

</notes>

</species>

<species id="M_LPP_c" name="Lipoylprotein" compartment="c" hasOnlySubstanceUnits="false" boundaryCondition="false" constant="false">

<notes>

<body xmlns="http://www.w3.org/1999/xhtml">

<p>FORMULA: C8H14NOS2R</p>

<p>CHARGE: 0</p>

</body>

</notes>

</species>

<species id="M_AMDHY_LPP_c" name="S-Aminomethyldihydrolipoylprotein" compartment="c" hasOnlySubstanceUnits="false" boundaryCondition="false" constant="false">

<notes>

<body xmlns="http://www.w3.org/1999/xhtml">

<p>FORMULA: C9H19N2OS2R</p>

<p>CHARGE: 0</p>

</body>

</notes>

</species>

<species id="M_DHY_LPP_c" name="Dihydrolipoylprotein" compartment="c" hasOnlySubstanceUnits="false" boundaryCondition="false" constant="false">

<notes>

<body xmlns="http://www.w3.org/1999/xhtml">

<p>FORMULA: C8H16NOS2R</p>

<p>CHARGE: 0</p>

</body>

</notes>

</species>

<species id="M_A_OXBUT_c" name="L-2-Amino-3-oxobutanoate" compartment="c" hasOnlySubstanceUnits="false" boundaryCondition="false" constant="false">

```
    <notes>
      <body xmlns="http://www.w3.org/1999/xhtml">
        <p>FORMULA: C4H7NO3</p>
        <p>CHARGE: 0</p>
      </body>
    </notes>
  </species>
  <species id="M_AKB_c" name="alpha-Ketobutyric acid" compartment="c" hasOnlySubstanceUnits="false"
boundaryCondition="false" constant="false">
    <notes>
      <body xmlns="http://www.w3.org/1999/xhtml">
        <p>FORMULA: C4H6O3</p>
        <p>CHARGE: 0</p>
      </body>
    </notes>
  </species>
  <species id="M_OP_HSER_c" name="O-Phospho-L-homoserine" compartment="c" hasOnlySubstanceUnits="false"
boundaryCondition="false" constant="false">
    <notes>
      <body xmlns="http://www.w3.org/1999/xhtml">
        <p>FORMULA: C4H10NO6P</p>
        <p>CHARGE: 0</p>
      </body>
    </notes>
  </species>
  <species id="M_L_HSER_c" name="L-Homoserine" compartment="c" hasOnlySubstanceUnits="false"
boundaryCondition="false" constant="false">
    <notes>
      <body xmlns="http://www.w3.org/1999/xhtml">
        <p>FORMULA: C4H9NO3</p>
```

<p>CHARGE: 0</p>

</body>

</notes>

</species>

<species id="M_ASP_4SA_c" name="L-Aspartate 4-semialdehyde" compartment="c" hasOnlySubstanceUnits="false" boundaryCondition="false" constant="false">

<notes>

<body xmlns="http://www.w3.org/1999/xhtml">

<p>FORMULA: C4H7NO3</p>

<p>CHARGE: 0</p>

</body>

</notes>

</species>

<species id="M_4P_ASP_c" name="4-Phospho-L-aspartate" compartment="c" hasOnlySubstanceUnits="false" boundaryCondition="false" constant="false">

<notes>

<body xmlns="http://www.w3.org/1999/xhtml">

<p>FORMULA: C4H8NO7P</p>

<p>CHARGE: 0</p>

</body>

</notes>

</species>

<species id="M_OAC_SER_c" name="O-Acetyl-L-serine" compartment="c" hasOnlySubstanceUnits="false" boundaryCondition="false" constant="false">

<notes>

<body xmlns="http://www.w3.org/1999/xhtml">

<p>FORMULA: C5H9NO4</p>

<p>CHARGE: 0</p>

</body>

</notes>

</species>

<species id="M_H2S_c" name="Hydrogen sulfide" compartment="c" hasOnlySubstanceUnits="false" boundaryCondition="false" constant="false">

<notes>

<body xmlns="http://www.w3.org/1999/xhtml">

<p>FORMULA: H2S</p>

<p>CHARGE: 0</p>

</body>

</notes>

</species>

<species id="M_TSFA_c" name="Thiosulfate" compartment="c" hasOnlySubstanceUnits="false" boundaryCondition="false" constant="false">

<notes>

<body xmlns="http://www.w3.org/1999/xhtml">

<p>FORMULA: HS2O3</p>

<p>CHARGE: 0</p>

</body>

</notes>

</species>

<species id="M_SO3_c" name="Sulfite" compartment="c" hasOnlySubstanceUnits="false" boundaryCondition="false" constant="false">

<notes>

<body xmlns="http://www.w3.org/1999/xhtml">

<p>FORMULA: H2SO3</p>

<p>CHARGE: 0</p>

</body>

</notes>

</species>

<species id="M_L_CYSTA_c" name="L-Cysteate" compartment="c" hasOnlySubstanceUnits="false" boundaryCondition="false" constant="false">

<notes>

<body xmlns="http://www.w3.org/1999/xhtml">

<p>FORMULA: C3H7NO5S</p>

<p>CHARGE: 0</p>

</body>

</notes>

</species>

<species id="M_SULFO_PYR_c" name="3-Sulfopyruvate" compartment="c" hasOnlySubstanceUnits="false" boundaryCondition="false" constant="false">

<notes>

<body xmlns="http://www.w3.org/1999/xhtml">

<p>FORMULA: C3H4O6S</p>

<p>CHARGE: 0</p>

</body>

</notes>

</species>

<species id="M_SLF_ALA_c" name="3-Sulfino-L-alanine" compartment="c" hasOnlySubstanceUnits="false" boundaryCondition="false" constant="false">

<notes>

<body xmlns="http://www.w3.org/1999/xhtml">

<p>FORMULA: C3H7NO4S</p>

<p>CHARGE: 0</p>

</body>

</notes>

</species>

<species id="M_SULFI_PYR_c" name="3-Sulfinylpyruvate" compartment="c" hasOnlySubstanceUnits="false" boundaryCondition="false" constant="false">

<notes>

<body xmlns="http://www.w3.org/1999/xhtml">

<p>FORMULA: C3H4O5S</p>

<p>CHARGE: 0</p>

</body>

</notes>

</species>

<species id="M_OAC_HSER_c" name="O-Acetyl-L-homoserine" compartment="c" hasOnlySubstanceUnits="false" boundaryCondition="false" constant="false">

<notes>

<body xmlns="http://www.w3.org/1999/xhtml">

<p>FORMULA: C6H11NO4</p>

<p>CHARGE: 0</p>

</body>

</notes>

</species>

<species id="M_L_HCYS_c" name="L-Homocysteine" compartment="c" hasOnlySubstanceUnits="false" boundaryCondition="false" constant="false">

<notes>

<body xmlns="http://www.w3.org/1999/xhtml">

<p>FORMULA: C4H9NO2S</p>

<p>CHARGE: 0</p>

</body>

</notes>

</species>

<species id="M_L_CYSTHNN_c" name="L-Cystathionine" compartment="c" hasOnlySubstanceUnits="false" boundaryCondition="false" constant="false">

<notes>

<body xmlns="http://www.w3.org/1999/xhtml">

<p>FORMULA: C7H14N2O4S</p>

<p>CHARGE: 0</p>

</body>

</notes>

</species>

<species id="M_5MTHF_c" name="5-Methyltetrahydrofolate" compartment="c" hasOnlySubstanceUnits="false" boundaryCondition="false" constant="false">

<notes>

<body xmlns="http://www.w3.org/1999/xhtml">

<p>FORMULA: C20H25N7O6</p>

<p>CHARGE: 0</p>

</body>

</notes>

</species>

<species id="M_RIBS_HCYS_c" name="S-Ribosyl-L-homocysteine" compartment="c" hasOnlySubstanceUnits="false" boundaryCondition="false" constant="false">

<notes>

<body xmlns="http://www.w3.org/1999/xhtml">

<p>FORMULA: C9H17NO6S</p>

<p>CHARGE: 0</p>

</body>

</notes>

</species>

<species id="M_DHY_PENTDON_c" name="(4S)-4,5-Dihydroxypentan-2,3-dione" compartment="c" hasOnlySubstanceUnits="false" boundaryCondition="false" constant="false">

<notes>

<body xmlns="http://www.w3.org/1999/xhtml">

<p>FORMULA: C5H8O4</p>

<p>CHARGE: 0</p>

</body>

</notes>

</species>

<species id="M_SAMA_c" name="S-Adenosylmethioninamine" compartment="c" hasOnlySubstanceUnits="false" boundaryCondition="false" constant="false">

```
        <notes>
          <body xmlns="http://www.w3.org/1999/xhtml">
            <p>FORMULA: C14H23N6O3S</p>
            <p>CHARGE: 0</p>
          </body>
        </notes>
      </species>
      <species id="M_PUT_c" name="Putrescine" compartment="c" hasOnlySubstanceUnits="false" boundaryCondition="false"
constant="false">
        <notes>
          <body xmlns="http://www.w3.org/1999/xhtml">
            <p>FORMULA: C4H12N2</p>
            <p>CHARGE: 0</p>
          </body>
        </notes>
      </species>
      <species id="M_MTA_c" name="5&apos;-Methylthioadenosine" compartment="c" hasOnlySubstanceUnits="false"
boundaryCondition="false" constant="false">
        <notes>
          <body xmlns="http://www.w3.org/1999/xhtml">
            <p>FORMULA: C11H15N5O3S</p>
            <p>CHARGE: 0</p>
          </body>
        </notes>
      </species>
      <species id="M_SPRD_c" name="Spermidine" compartment="c" hasOnlySubstanceUnits="false" boundaryCondition="false"
constant="false">
        <notes>
          <body xmlns="http://www.w3.org/1999/xhtml">
            <p>FORMULA: C7H19N3</p>
```

<p>CHARGE: 0</p>

</body>

</notes>

</species>

<species id="M_SPR_c" name="Spermine" compartment="c" hasOnlySubstanceUnits="false" boundaryCondition="false" constant="false">

<notes>

<body xmlns="http://www.w3.org/1999/xhtml">

<p>FORMULA: C10H26N4</p>

<p>CHARGE: 0</p>

</body>

</notes>

</species>

<species id="M_MTH_RIB_c" name="5-Methylthio-D-ribose" compartment="c" hasOnlySubstanceUnits="false" boundaryCondition="false" constant="false">

<notes>

<body xmlns="http://www.w3.org/1999/xhtml">

<p>FORMULA: C6H12O4S</p>

<p>CHARGE: 0</p>

</body>

</notes>

</species>

<species id="M_MTh_RIB1P_c" name="S-Methyl-5-thio-D-ribose 1-phosphate" compartment="c" hasOnlySubstanceUnits="false" boundaryCondition="false" constant="false">

<notes>

<body xmlns="http://www.w3.org/1999/xhtml">

<p>FORMULA: C6H13O7PS</p>

<p>CHARGE: 0</p>

</body>

</notes>

</species>

<species id="M_MTh_RIBL1P_c" name="S-Methyl-5-thio-D-ribulose 1-phosphate" compartment="c" hasOnlySubstanceUnits="false" boundaryCondition="false" constant="false">

<notes>

<body xmlns="http://www.w3.org/1999/xhtml">

<p>FORMULA: C6H13O7PS</p>

<p>CHARGE: 0</p>

</body>

</notes>

</species>

<species id="M_4M_2OPENTN_c" name="4-Methyl-2-oxopentanoate" compartment="c" hasOnlySubstanceUnits="false" boundaryCondition="false" constant="false">

<notes>

<body xmlns="http://www.w3.org/1999/xhtml">

<p>FORMULA: C6H10O3</p>

<p>CHARGE: 0</p>

</body>

</notes>

</species>

<species id="M_2K3M_BUT_c" name="3-Methyl-2-oxobutanoate" compartment="c" hasOnlySubstanceUnits="false" boundaryCondition="false" constant="false">

<notes>

<body xmlns="http://www.w3.org/1999/xhtml">

<p>FORMULA: C5H8O3</p>

<p>CHARGE: 0</p>

</body>

</notes>

</species>

<species id="M_3M_2OPENTN_c" name="(S)-3-Methyl-2-oxopentanoate" compartment="c" hasOnlySubstanceUnits="false" boundaryCondition="false" constant="false">

<notes>
<body xmlns="http://www.w3.org/1999/xhtml">
<p>FORMULA: C6H10O3</p>
<p>CHARGE: 0</p>
</body>
</notes>
</species>
<species id="M_TPP_c" name="Thiamine diphosphate (ThPP)" compartment="c" hasOnlySubstanceUnits="false" boundaryCondition="false" constant="false">
<notes>
<body xmlns="http://www.w3.org/1999/xhtml">
<p>FORMULA: C12H19N4O7P2S</p>
<p>CHARGE: 0</p>
</body>
</notes>
</species>
<species id="M_2MHYBUT_ThPP_c" name="2-Methyl-1-hydroxybutyl-ThPP" compartment="c" hasOnlySubstanceUnits="false" boundaryCondition="false" constant="false">
<notes>
<body xmlns="http://www.w3.org/1999/xhtml">
<p>FORMULA: C17H29N4O8P2S</p>
<p>CHARGE: 0</p>
</body>
</notes>
</species>
<species id="M_3MHYBUT_ThPP_c" name="3-Methyl-1-hydroxybutyl-ThPP" compartment="c" hasOnlySubstanceUnits="false" boundaryCondition="false" constant="false">
<notes>
<body xmlns="http://www.w3.org/1999/xhtml">
<p>FORMULA: C17H29N4O8P2S</p>

<p>CHARGE: 0</p>

</body>

</notes>

</species>

<species id="M_2MHYPROP_ThPP_c" name="2-Methyl-1-hydroxypropyl-ThPP" compartment="c" hasOnlySubstanceUnits="false" boundaryCondition="false" constant="false">

<notes>

<body xmlns="http://www.w3.org/1999/xhtml">

<p>FORMULA: C16H27N4O8P2S</p>

<p>CHARGE: 0</p>

</body>

</notes>

</species>

<species id="M_ENZN6_LPL_c" name="Enzyme N6-(lipoyl)lysine" compartment="c" hasOnlySubstanceUnits="false" boundaryCondition="false" constant="false">

<notes>

<body xmlns="http://www.w3.org/1999/xhtml">

<p>FORMULA: C8H14NOS2R</p>

<p>CHARGE: 0</p>

</body>

</notes>

</species>

<species id="M_DHYLPL_MPROP_MPROP_DHLPL_c" name="[Dihydrolipoyllysine-residue (2-methylpropanoyl)transferase] S-(2-methylpropanoyl)dihydrolipoyllysine" compartment="c" hasOnlySubstanceUnits="false" boundaryCondition="false" constant="false">

<notes>

<body xmlns="http://www.w3.org/1999/xhtml">

<p>FORMULA: C12H22NO2S2R</p>

<p>CHARGE: 0</p>

</body>

</notes>

</species>

<species id="M_DHYLPL_MPROP_3MBUT_DHLPL_c" name="[Dihydrolipoyllysine-residue (2-methylpropanoyl)transferase] S-(3-methylbutanoyl)dihydrolipoyllysine" compartment="c" hasOnlySubstanceUnits="false" boundaryCondition="false" constant="false">

<notes>

<body xmlns="http://www.w3.org/1999/xhtml">

<p>FORMULA: C13H24NO2S2R</p>

<p>CHARGE: 0</p>

</body>

</notes>

</species>

<species id="M_DHYLPL_MPROP_2MBUT_DHLPL_c" name="[Dihydrolipoyllysine-residue (2-methylpropanoyl)transferase] S-(2-methylbutanoyl)dihydrolipoyllysine" compartment="c" hasOnlySubstanceUnits="false" boundaryCondition="false" constant="false">

<notes>

<body xmlns="http://www.w3.org/1999/xhtml">

<p>FORMULA: C13H24NO2S2R</p>

<p>CHARGE: 0</p>

</body>

</notes>

</species>

<species id="M_MBUTNCoA_c" name="(S)-2-Methylbutanoyl-CoA" compartment="c" hasOnlySubstanceUnits="false" boundaryCondition="false" constant="false">

<notes>

<body xmlns="http://www.w3.org/1999/xhtml">

<p>FORMULA: C26H44N7O17P3S</p>

<p>CHARGE: 0</p>

</body>

</notes>

</species>

<species id="M_MBUT_ENCoA_c" name="2-Methylbut-2-enoyl-CoA" compartment="c" hasOnlySubstanceUnits="false" boundaryCondition="false" constant="false">

<notes>

<body xmlns="http://www.w3.org/1999/xhtml">

<p>FORMULA: C26H42N7O17P3S</p>

<p>CHARGE: 0</p>

</body>

</notes>

</species>

<species id="M_2M_PROCoA_c" name="2-Methylpropanoyl-CoA" compartment="c" hasOnlySubstanceUnits="false" boundaryCondition="false" constant="false">

<notes>

<body xmlns="http://www.w3.org/1999/xhtml">

<p>FORMULA: C25H42N7O17P3S</p>

<p>CHARGE: 0</p>

</body>

</notes>

</species>

<species id="M_MACRYCoA_c" name="Methylacrylyl-CoA" compartment="c" hasOnlySubstanceUnits="false" boundaryCondition="false" constant="false">

<notes>

<body xmlns="http://www.w3.org/1999/xhtml">

<p>FORMULA: C25H40N7O17P3S</p>

<p>CHARGE: 0</p>

</body>

</notes>

</species>

<species id="M_HY_MBUTRCoA_c" name="(S)-3-Hydroxy-2-methylbutyryl-CoA" compartment="c" hasOnlySubstanceUnits="false" boundaryCondition="false" constant="false">

<notes>

<body xmlns="http://www.w3.org/1999/xhtml">

<p>FORMULA: C26H44N7O18P3S</p>

<p>CHARGE: 0</p>

</body>

</notes>

</species>

<species id="M_HYIBUTRCoA_c" name="(S)-3-Hydroxyisobutyryl-CoA" compartment="c" hasOnlySubstanceUnits="false" boundaryCondition="false" constant="false">

<notes>

<body xmlns="http://www.w3.org/1999/xhtml">

<p>FORMULA: C25H42N7O18P3S</p>

<p>CHARGE: 0</p>

</body>

</notes>

</species>

<species id="M_HYM_GLUTRCoA_c" name="(S)-3-Hydroxy-3-methylglutaryl-CoA" compartment="c" hasOnlySubstanceUnits="false" boundaryCondition="false" constant="false">

<notes>

<body xmlns="http://www.w3.org/1999/xhtml">

<p>FORMULA: C27H44N7O20P3S</p>

<p>CHARGE: 0</p>

</body>

</notes>

</species>

<species id="M_AAC_c" name="Acetoacetate" compartment="c" hasOnlySubstanceUnits="false" boundaryCondition="false" constant="false">

<notes>

<body xmlns="http://www.w3.org/1999/xhtml">

<p>FORMULA: C4H6O3</p>

<p>CHARGE: 0</p>

</body>

</notes>

</species>

<species id="M_HYIBUTRA_c" name="(S)-3-Hydroxyisobutyrate" compartment="c" hasOnlySubstanceUnits="false" boundaryCondition="false" constant="false">

<notes>

<body xmlns="http://www.w3.org/1999/xhtml">

<p>FORMULA: C4H8O3</p>

<p>CHARGE: 0</p>

</body>

</notes>

</species>

<species id="M_MMAL_SA_c" name="(S)-Methylmalonate semialdehyde" compartment="c" hasOnlySubstanceUnits="false" boundaryCondition="false" constant="false">

<notes>

<body xmlns="http://www.w3.org/1999/xhtml">

<p>FORMULA: C4H6O3</p>

<p>CHARGE: 0</p>

</body>

</notes>

</species>

<species id="M_MMAL_c" name="Methylmalonate" compartment="c" hasOnlySubstanceUnits="false" boundaryCondition="false" constant="false">

<notes>

<body xmlns="http://www.w3.org/1999/xhtml">

<p>FORMULA: C4H6O4</p>

<p>CHARGE: 0</p>

</body>

</notes>

</species>

<species id="M_MAACCoA_c" name="2-Methylacetoacetyl-CoA" compartment="c" hasOnlySubstanceUnits="false" boundaryCondition="false" constant="false">

<notes>

<body xmlns="http://www.w3.org/1999/xhtml">

<p>FORMULA: C26H42N7O18P3S</p>

<p>CHARGE: 0</p>

</body>

</notes>

</species>

<species id="M_PRCoA_c" name="Propanoyl-CoA" compartment="c" hasOnlySubstanceUnits="false" boundaryCondition="false" constant="false">

<notes>

<body xmlns="http://www.w3.org/1999/xhtml">

<p>FORMULA: C24H40N7O17P3S</p>

<p>CHARGE: 0</p>

</body>

</notes>

</species>

<species id="M_R_MMALCoA_c" name="(R)-Methylmalonyl-CoA" compartment="c" hasOnlySubstanceUnits="false" boundaryCondition="false" constant="false">

<notes>

<body xmlns="http://www.w3.org/1999/xhtml">

<p>FORMULA: C25H40N7O19P3S</p>

<p>CHARGE: 0</p>

</body>

</notes>

</species>

<species id="M_MMALCoA_c" name="(S)-Methylmalonyl-CoA" compartment="c" hasOnlySubstanceUnits="false" boundaryCondition="false" constant="false">

<notes>

<body xmlns="http://www.w3.org/1999/xhtml">

<p>FORMULA: C25H40N7O19P3S</p>

<p>CHARGE: 0</p>

</body>

</notes>

</species>

<species id="M_DHY_DPICLN_c" name="Dihydrodipicolinate" compartment="c" hasOnlySubstanceUnits="false" boundaryCondition="false" constant="false">

<notes>

<body xmlns="http://www.w3.org/1999/xhtml">

<p>FORMULA: C7H7NO4</p>

<p>CHARGE: 0</p>

</body>

</notes>

</species>

<species id="M_UDP_ACMUR_ALA_GLU_DAPIM_c" name="UDP-N-acetylmuramoyl-L-alanyl-D-gamma-glutamyl-meso-2,6-diaminopimelate" compartment="c" hasOnlySubstanceUnits="false" boundaryCondition="false" constant="false">

<notes>

<body xmlns="http://www.w3.org/1999/xhtml">

<p>FORMULA: C35H55N7O26P2</p>

<p>CHARGE: 0</p>

</body>

</notes>

</species>

<species id="M_D_ALA_D_ALA_c" name="D-Alanyl-D-alanine" compartment="c" hasOnlySubstanceUnits="false" boundaryCondition="false" constant="false">

<notes>

<body xmlns="http://www.w3.org/1999/xhtml">

<p>FORMULA: C6H12N2O3</p>

<p>CHARGE: 0</p>

</body>

</notes>

</species>

<species id="M_UDP_ACMUR_ALA_GLU_DAPIM_ALA_ALA_c" name="UDP-N-acetylmuramoyl-L-alanyl-D-glutamyl-6-carboxy-L-lysyl-D-alanyl- D-alanine" compartment="c" hasOnlySubstanceUnits="false" boundaryCondition="false" constant="false">

<notes>

<body xmlns="http://www.w3.org/1999/xhtml">

<p>FORMULA: C41H65N9O28P2</p>

<p>CHARGE: 0</p>

</body>

</notes>

</species>

<species id="M_UDP_ACMURM_ALA_GLUT_c" name="UDP-N-acetylmuramoyl-L-alanyl-D-glutamate" compartment="c" hasOnlySubstanceUnits="false" boundaryCondition="false" constant="false">

<notes>

<body xmlns="http://www.w3.org/1999/xhtml">

<p>FORMULA: C28H43N5O23P2</p>

<p>CHARGE: 0</p>

</body>

</notes>

</species>

<species id="M_M26_DAP_c" name="meso-2,6-Diaminoheptanedioate" compartment="c" hasOnlySubstanceUnits="false" boundaryCondition="false" constant="false">

<notes>

<body xmlns="http://www.w3.org/1999/xhtml">

<p>FORMULA: C7H14N2O4</p>

<p>CHARGE: 0</p>

</body>

</notes>

</species>

<species id="M_HCIT_c" name="Homocitrate" compartment="c" hasOnlySubstanceUnits="false" boundaryCondition="false" constant="false">

<notes>

<body xmlns="http://www.w3.org/1999/xhtml">

<p>FORMULA: C7H10O7</p>

<p>CHARGE: 0</p>

</body>

</notes>

</species>

<species id="M_OXLGLUTR_c" name="Oxaloglutarate" compartment="c" hasOnlySubstanceUnits="false" boundaryCondition="false" constant="false">

<notes>

<body xmlns="http://www.w3.org/1999/xhtml">

<p>FORMULA: C7H8O7</p>

<p>CHARGE: 0</p>

</body>

</notes>

</species>

<species id="M_2OADPA_c" name="2-Oxoadipate" compartment="c" hasOnlySubstanceUnits="false" boundaryCondition="false" constant="false">

<notes>

<body xmlns="http://www.w3.org/1999/xhtml">

<p>FORMULA: C6H8O5</p>

<p>CHARGE: 0</p>

</body>

</notes>

</species>

<species id="M_HICTR_c" name="Homoisocitrate" compartment="c" hasOnlySubstanceUnits="false" boundaryCondition="false" constant="false">

```
        <notes>
          <body xmlns="http://www.w3.org/1999/xhtml">
            <p>FORMULA: C7H10O7</p>
            <p>CHARGE: 0</p>
          </body>
        </notes>
      </species>
      <species id="M_AADP_c" name="L-2-Aminoadipate" compartment="c" hasOnlySubstanceUnits="false"
boundaryCondition="false" constant="false">
        <notes>
          <body xmlns="http://www.w3.org/1999/xhtml">
            <p>FORMULA: </p>
            <p>CHARGE: 0</p>
          </body>
        </notes>
      </species>
      <species id="M_AC_AADP_c" name="N2-Acetyl-L-aminoadipate" compartment="c" hasOnlySubstanceUnits="false"
boundaryCondition="false" constant="false">
        <notes>
          <body xmlns="http://www.w3.org/1999/xhtml">
            <p>FORMULA: C8H13NO5</p>
            <p>CHARGE: 0</p>
          </body>
        </notes>
      </species>
      <species id="M_AC_AADP_DELPi_c" name="N2-Acetyl-L-aminoadipyl-delta-phosphate" compartment="c"
hasOnlySubstanceUnits="false" boundaryCondition="false" constant="false">
        <notes>
          <body xmlns="http://www.w3.org/1999/xhtml">
            <p>FORMULA: C8H14NO8P</p>
```

<p>CHARGE: 0</p>

</body>

</notes>

</species>

<species id="M_AC_AADP_SA_c" name="N2-Acetyl-L-aminoadipate semialdehyde" compartment="c" hasOnlySubstanceUnits="false" boundaryCondition="false" constant="false">

<notes>

<body xmlns="http://www.w3.org/1999/xhtml">

<p>FORMULA: C8H13NO4</p>

<p>CHARGE: 0</p>

</body>

</notes>

</species>

<species id="M_N2AC_L_LYS_c" name="N2-Acetyl-L-lysine" compartment="c" hasOnlySubstanceUnits="false" boundaryCondition="false" constant="false">

<notes>

<body xmlns="http://www.w3.org/1999/xhtml">

<p>FORMULA: C8H16N2O3</p>

<p>CHARGE: 0</p>

</body>

</notes>

</species>

<species id="M_DHLPL_SUCCT_GLUTRDHLPL_c" name="[Dihydrolipoyllysine-residue succinyltransferase] S-glutaryldihydrolipoyllysine, S-Glutaryldihydrolipoamide-E" compartment="c" hasOnlySubstanceUnits="false" boundaryCondition="false" constant="false">

<notes>

<body xmlns="http://www.w3.org/1999/xhtml">

<p>FORMULA: C13H22NO4S2R</p>

<p>CHARGE: 0</p>

</body>

</notes>

</species>

<species id="M_ENZNE_DHYLPL_c" name="Enzyme N6-(dihydrolipoyl)lysine" compartment="c" hasOnlySubstanceUnits="false" boundaryCondition="false" constant="false">

<notes>

<body xmlns="http://www.w3.org/1999/xhtml">

<p>FORMULA: C8H16NOS2R</p>

<p>CHARGE: 0</p>

</body>

</notes>

</species>

<species id="M_TM_AMBUTAL_c" name="4-Trimethylammoniobutanal" compartment="c" hasOnlySubstanceUnits="false" boundaryCondition="false" constant="false">

<notes>

<body xmlns="http://www.w3.org/1999/xhtml">

<p>FORMULA: C7H16NO</p>

<p>CHARGE: 0</p>

</body>

</notes>

</species>

<species id="M_TM_AMBUTAT_c" name="4-Trimethylammoniobutanoate" compartment="c" hasOnlySubstanceUnits="false" boundaryCondition="false" constant="false">

<notes>

<body xmlns="http://www.w3.org/1999/xhtml">

<p>FORMULA: C7H16NO2</p>

<p>CHARGE: 0</p>

</body>

</notes>

</species>

<species id="M_PRIB_ATP_c" name="Phosphoribosyl-ATP" compartment="c" hasOnlySubstanceUnits="false" boundaryCondition="false" constant="false">

<notes>

<body xmlns="http://www.w3.org/1999/xhtml">

<p>FORMULA: C15H25N5O20P4</p>

<p>CHARGE: 0</p>

</body>

</notes>

</species>

<species id="M_PRIB_AMP_c" name="Phosphoribosyl-AMP" compartment="c" hasOnlySubstanceUnits="false" boundaryCondition="false" constant="false">

<notes>

<body xmlns="http://www.w3.org/1999/xhtml">

<p>FORMULA: C15H23N5O14P2</p>

<p>CHARGE: 0</p>

</body>

</notes>

</species>

<species id="M_PRINS_AFORMA_PRIBS_IMDZ_CARBA_c" name="5-(5-Phospho-D-ribosylaminoformimino)-1-(5-phosphoribosyl)- imidazole-4-carboxamide" compartment="c" hasOnlySubstanceUnits="false" boundaryCondition="false" constant="false">

<notes>

<body xmlns="http://www.w3.org/1999/xhtml">

<p>FORMULA: C15H25N5O15P2</p>

<p>CHARGE: 0</p>

</body>

</notes>

</species>

<species id="M_PRINSFORMA_PRIBS_IMDZ_CARBA_c" name="N-(5'-Phospho-D-1'-ribulosylformimino)-5-amino-1-(5''-phospho-D- ribosyl)-4-imidazolecarboxamide" compartment="c" hasOnlySubstanceUnits="false" boundaryCondition="false" constant="false">

```
        <notes>
          <body xmlns="http://www.w3.org/1999/xhtml">
            <p>FORMULA: C15H25N5O15P2</p>
            <p>CHARGE: 0</p>
          </body>
        </notes>
      </species>
      <species id="M_ERY_IMDZ_GLYCP_c" name="D-erythro-1-(Imidazol-4-yl)glycerol 3-phosphate, D-erythro-Imidazole-glycerol
3-phosphate, D-erythro-Imidazole-glycerol phosphate" compartment="c" hasOnlySubstanceUnits="false"
boundaryCondition="false" constant="false">
        <notes>
          <body xmlns="http://www.w3.org/1999/xhtml">
            <p>FORMULA: C6H11N2O6P</p>
            <p>CHARGE: 0</p>
          </body>
        </notes>
      </species>
      <species id="M_IMDZ_ACLP_c" name="Imidazole-acetol phosphate" compartment="c" hasOnlySubstanceUnits="false"
boundaryCondition="false" constant="false">
        <notes>
          <body xmlns="http://www.w3.org/1999/xhtml">
            <p>FORMULA: C6H9N2O5P</p>
            <p>CHARGE: 0</p>
          </body>
        </notes>
      </species>
      <species id="M_HISDPi_c" name="L-Histidinol phosphate" compartment="c" hasOnlySubstanceUnits="false"
boundaryCondition="false" constant="false">
        <notes>
          <body xmlns="http://www.w3.org/1999/xhtml">
```

```
          <p>FORMULA: C6H12N3O4P</p>

          <p>CHARGE: 0</p>

        </body>

      </notes>

    </species>

    <species id="M_L_HISN_c" name="L-Histidinol" compartment="c" hasOnlySubstanceUnits="false"
boundaryCondition="false" constant="false">

      <notes>

        <body xmlns="http://www.w3.org/1999/xhtml">

          <p>FORMULA: C6H11N3O</p>

          <p>CHARGE: 0</p>

        </body>

      </notes>

    </species>

    <species id="M_L_HISDAL_c" name="L-Histidinal" compartment="c" hasOnlySubstanceUnits="false"
boundaryCondition="false" constant="false">

      <notes>

        <body xmlns="http://www.w3.org/1999/xhtml">

          <p>FORMULA: C6H9N3O</p>

          <p>CHARGE: 0</p>

        </body>

      </notes>

    </species>

    <species id="M_IMDZ_AALD_c" name="Imidazole-4-acetaldehyde" compartment="c" hasOnlySubstanceUnits="false"
boundaryCondition="false" constant="false">

      <notes>

        <body xmlns="http://www.w3.org/1999/xhtml">

          <p>FORMULA: C5H6N2O</p>

          <p>CHARGE: 0</p>

        </body>
```

</notes>

</species>

<species id="M_IMDZ_AC_c" name="4-Imidazoleacetate" compartment="c" hasOnlySubstanceUnits="false" boundaryCondition="false" constant="false">

<notes>

<body xmlns="http://www.w3.org/1999/xhtml">

<p>FORMULA: C5H6N2O2</p>

<p>CHARGE: 0</p>

</body>

</notes>

</species>

<species id="M_HYPh_PYR_c" name="p-Hydroxyphenylpyruvate" compartment="c" hasOnlySubstanceUnits="false" boundaryCondition="false" constant="false">

<notes>

<body xmlns="http://www.w3.org/1999/xhtml">

<p>FORMULA: C9H8O4</p>

<p>CHARGE: 0</p>

</body>

</notes>

</species>

<species id="M_HY_PhAC_c" name="4-Hydroxyphenylacetate" compartment="c" hasOnlySubstanceUnits="false" boundaryCondition="false" constant="false">

<notes>

<body xmlns="http://www.w3.org/1999/xhtml">

<p>FORMULA: C8H8O3</p>

<p>CHARGE: 0</p>

</body>

</notes>

</species>

```
<species id="M_dHY_PhAC_c" name="3,4-Dihydroxyphenylacetate" compartment="c" hasOnlySubstanceUnits="false" boundaryCondition="false" constant="false">
  <notes>
    <body xmlns="http://www.w3.org/1999/xhtml">
      <p>FORMULA: C8H8O4</p>
      <p>CHARGE: 0</p>
    </body>
  </notes>
</species>
<species id="M_2HY_5CARBMMUC_SA_c" name="2-Hydroxy-5-carboxymethylmuconate semialdehyde" compartment="c" hasOnlySubstanceUnits="false" boundaryCondition="false" constant="false">
  <notes>
    <body xmlns="http://www.w3.org/1999/xhtml">
      <p>FORMULA: C8H8O6</p>
      <p>CHARGE: 0</p>
    </body>
  </notes>
</species>
<species id="M_CARBM_HYMUC_c" name="5-Carboxymethyl-2-hydroxymuconate" compartment="c" hasOnlySubstanceUnits="false" boundaryCondition="false" constant="false">
  <notes>
    <body xmlns="http://www.w3.org/1999/xhtml">
      <p>FORMULA: C8H8O7</p>
      <p>CHARGE: 0</p>
    </body>
  </notes>
</species>
<species id="M_CRBX_OHEPTEDA_c" name="5-Carboxy-2-oxohept-3-enedioate" compartment="c" hasOnlySubstanceUnits="false" boundaryCondition="false" constant="false">
  <notes>
```

<body xmlns="http://www.w3.org/1999/xhtml">

<p>FORMULA: C8H8O7</p>

<p>CHARGE: 0</p>

</body>

</notes>

</species>

<species id="M_HYHEPTDENA_c" name="2-Hydroxyhepta-2,4-dienedioate" compartment="c" hasOnlySubstanceUnits="false" boundaryCondition="false" constant="false">

<notes>

<body xmlns="http://www.w3.org/1999/xhtml">

<p>FORMULA: C7H8O5</p>

<p>CHARGE: 0</p>

</body>

</notes>

</species>

<species id="M_OHEPEDA_c" name="2-Oxohept-3-enedioate" compartment="c" hasOnlySubstanceUnits="false" boundaryCondition="false" constant="false">

<notes>

<body xmlns="http://www.w3.org/1999/xhtml">

<p>FORMULA: C7H8O5</p>

<p>CHARGE: 0</p>

</body>

</notes>

</species>

<species id="M_PhAC_c" name="Phenylacetate" compartment="c" hasOnlySubstanceUnits="false" boundaryCondition="false" constant="false">

<notes>

<body xmlns="http://www.w3.org/1999/xhtml">

<p>FORMULA: C8H8O2</p>

<p>CHARGE: 0</p>

</body>

</notes>

</species>

<species id="M_PhACoA_c" name="Phenylacetyl-CoA" compartment="c" hasOnlySubstanceUnits="false" boundaryCondition="false" constant="false">

<notes>

<body xmlns="http://www.w3.org/1999/xhtml">

<p>FORMULA: C29H42N7O17P3S</p>

<p>CHARGE: 0</p>

</body>

</notes>

</species>

<species id="M_PPYR_c" name="Phenylpyruvate" compartment="c" hasOnlySubstanceUnits="false" boundaryCondition="false" constant="false">

<notes>

<body xmlns="http://www.w3.org/1999/xhtml">

<p>FORMULA: C9H8O3</p>

<p>CHARGE: 0</p>

</body>

</notes>

</species>

<species id="M_PhALD_c" name="Phenylacetaldehyde" compartment="c" hasOnlySubstanceUnits="false" boundaryCondition="false" constant="false">

<notes>

<body xmlns="http://www.w3.org/1999/xhtml">

<p>FORMULA: </p>

<p>CHARGE: 0</p>

</body>

</notes>

</species>

<species id="M_dhPhACoA_c" name="2-(1,2-epoxy-1,2-dihydrophenyl)acetyl-CoA" compartment="c" hasOnlySubstanceUnits="false" boundaryCondition="false" constant="false">

<notes>

<body xmlns="http://www.w3.org/1999/xhtml">

<p>FORMULA: C29H38N7O18P3S</p>

<p>CHARGE: 0</p>

</body>

</notes>

</species>

<species id="M_dhPhAC_c" compartment="c" hasOnlySubstanceUnits="false" boundaryCondition="false" constant="false">

<notes>

<body xmlns="http://www.w3.org/1999/xhtml">

<p>FORMULA: </p>

<p>CHARGE: 0</p>

</body>

</notes>

</species>

<species id="M_OPACCoA_c" name="2-oxepin-2(3H)-ylideneacetyl-CoA" compartment="c" hasOnlySubstanceUnits="false" boundaryCondition="false" constant="false">

<notes>

<body xmlns="http://www.w3.org/1999/xhtml">

<p>FORMULA: C29H38N7O18P3S</p>

<p>CHARGE: 0</p>

</body>

</notes>

</species>

<species id="M_ODHSCoASA_c" name="3-oxo-5,6-dehydrosuberyl-CoA semialdehyde" compartment="c" hasOnlySubstanceUnits="false" boundaryCondition="false" constant="false">

<notes>

<body xmlns="http://www.w3.org/1999/xhtml">

<p>FORMULA: C29H40N7O19P3S</p>

<p>CHARGE: 0</p>

</body>

</notes>

</species>

<species id="M_ODHSCoA_c" name="3-oxo-5,6-dehydrosuberyl-CoA" compartment="c" hasOnlySubstanceUnits="false" boundaryCondition="false" constant="false">

<notes>

<body xmlns="http://www.w3.org/1999/xhtml">

<p>FORMULA: C29H39N7O20P3S</p>

<p>CHARGE: 0</p>

</body>

</notes>

</species>

<species id="M_DHADPCoA_c" name="2,3-dehydroadipyl-CoA" compartment="c" hasOnlySubstanceUnits="false" boundaryCondition="false" constant="false">

<notes>

<body xmlns="http://www.w3.org/1999/xhtml">

<p>FORMULA: C27H37N7O19P3S</p>

<p>CHARGE: 0</p>

</body>

</notes>

</species>

<species id="M_HADPCoA_c" name="3-hydroxyadipyl-CoA" compartment="c" hasOnlySubstanceUnits="false" boundaryCondition="false" constant="false">

<notes>

<body xmlns="http://www.w3.org/1999/xhtml">

<p>FORMULA: C27H39N7O20P3S</p>

<p>CHARGE: 0</p>

</body>

</notes>

</species>

<species id="M_OADPCoA_c" name="3-oxoadipyl-CoA" compartment="c" hasOnlySubstanceUnits="false" boundaryCondition="false" constant="false">

<notes>

<body xmlns="http://www.w3.org/1999/xhtml">

<p>FORMULA: C27H37N7O20P3S</p>

<p>CHARGE: 0</p>

</body>

</notes>

</species>

<species id="M_B_ALA_c" name="beta-Alanine" compartment="c" hasOnlySubstanceUnits="false" boundaryCondition="false" constant="false">

<notes>

<body xmlns="http://www.w3.org/1999/xhtml">

<p>FORMULA: C3H7NO2</p>

<p>CHARGE: 0</p>

</body>

</notes>

</species>

<species id="M_MAL_SA_c" name="Malonate semialdehyde" compartment="c" hasOnlySubstanceUnits="false" boundaryCondition="false" constant="false">

<notes>

<body xmlns="http://www.w3.org/1999/xhtml">

<p>FORMULA: C3H4O3</p>

<p>CHARGE: 0</p>

</body>

</notes>

</species>

<species id="M_PANTA_c" name="(R)-Pantoate" compartment="c" hasOnlySubstanceUnits="false" boundaryCondition="false" constant="false">

<notes>

<body xmlns="http://www.w3.org/1999/xhtml">

<p>FORMULA: C6H12O4</p>

<p>CHARGE: 0</p>

</body>

</notes>

</species>

<species id="M_PANTO_c" name="(R)-Pantothenate" compartment="c" hasOnlySubstanceUnits="false" boundaryCondition="false" constant="false">

<notes>

<body xmlns="http://www.w3.org/1999/xhtml">

<p>FORMULA: C9H17NO5</p>

<p>CHARGE: 0</p>

</body>

</notes>

</species>

<species id="M_UDP_ACMURM_ALA_c" name="UDP-N-acetylmuramoyl-L-alanine" compartment="c" hasOnlySubstanceUnits="false" boundaryCondition="false" constant="false">

<notes>

<body xmlns="http://www.w3.org/1999/xhtml">

<p>FORMULA: C23H36N4O20P2</p>

<p>CHARGE: 0</p>

</body>

</notes>

</species>

<species id="M_IPROP_MAL_c" name="(2R,3S)-3-Isopropylmalate" compartment="c" hasOnlySubstanceUnits="false" boundaryCondition="false" constant="false">

<notes>

<body xmlns="http://www.w3.org/1999/xhtml">

<p>FORMULA: C7H12O5</p>

<p>CHARGE: 0</p>

</body>

</notes>

</species>

<species id="M_RDHY_MBUTNA_c" name="(R)-2,3-Dihydroxy-3-methylbutanoate" compartment="c" hasOnlySubstanceUnits="false" boundaryCondition="false" constant="false">

<notes>

<body xmlns="http://www.w3.org/1999/xhtml">

<p>FORMULA: C5H10O4</p>

<p>CHARGE: 0</p>

</body>

</notes>

</species>

<species id="M_3HY_2M_2OBUTNA_c" name="3-Hydroxy-3-methyl-2-oxobutanoic acid" compartment="c" hasOnlySubstanceUnits="false" boundaryCondition="false" constant="false">

<notes>

<body xmlns="http://www.w3.org/1999/xhtml">

<p>FORMULA: C5H8O4</p>

<p>CHARGE: 0</p>

</body>

</notes>

</species>

<species id="M_DHY_MPENTA_c" name="(R)-2,3-Dihydroxy-3-methylpentanoate" compartment="c" hasOnlySubstanceUnits="false" boundaryCondition="false" constant="false">

<notes>

<body xmlns="http://www.w3.org/1999/xhtml">

<p>FORMULA: C6H12O4</p>

<p>CHARGE: 0</p>

</body>

</notes>

</species>

<species id="M_AC_HYBUTNA_c" name="(S)-2-Aceto-2-hydroxybutanoate" compartment="c" hasOnlySubstanceUnits="false" boundaryCondition="false" constant="false">

<notes>

<body xmlns="http://www.w3.org/1999/xhtml">

<p>FORMULA: C6H10O4</p>

<p>CHARGE: 0</p>

</body>

</notes>

</species>

<species id="M_S_ACLAC_c" name="(S)-2-Acetolactate" compartment="c" hasOnlySubstanceUnits="false" boundaryCondition="false" constant="false">

<notes>

<body xmlns="http://www.w3.org/1999/xhtml">

<p>FORMULA: C5H8O4</p>

<p>CHARGE: 0</p>

</body>

</notes>

</species>

<species id="M_IPRO_MAL_c" name="(2S)-2-Isopropylmalate" compartment="c" hasOnlySubstanceUnits="false" boundaryCondition="false" constant="false">

<notes>

<body xmlns="http://www.w3.org/1999/xhtml">

<p>FORMULA: C7H12O5</p>

<p>CHARGE: 0</p>

</body>

</notes>

</species>

<species id="M_IPRO_MALE_c" name="2-Isopropylmaleate" compartment="c" hasOnlySubstanceUnits="false" boundaryCondition="false" constant="false">

<notes>

<body xmlns="http://www.w3.org/1999/xhtml">

<p>FORMULA: C7H10O4</p>

<p>CHARGE: 0</p>

</body>

</notes>

</species>

<species id="M_IPROP_OSUCC_c" name="(2S)-2-Isopropyl-3-oxosuccinate" compartment="c" hasOnlySubstanceUnits="false" boundaryCondition="false" constant="false">

<notes>

<body xmlns="http://www.w3.org/1999/xhtml">

<p>FORMULA: C7H10O5</p>

<p>CHARGE: 0</p>

</body>

</notes>

</species>

<species id="M_AC_GLUT_c" name="N-Acetyl-L-glutamate" compartment="c" hasOnlySubstanceUnits="false" boundaryCondition="false" constant="false">

<notes>

<body xmlns="http://www.w3.org/1999/xhtml">

<p>FORMULA: C7H11NO5</p>

<p>CHARGE: 0</p>

</body>

</notes>

</species>

<species id="M_AC_GLUTPi_c" name="N-Acetyl-L-glutamate 5-phosphate" compartment="c" hasOnlySubstanceUnits="false" boundaryCondition="false" constant="false">

<notes>

<body xmlns="http://www.w3.org/1999/xhtml">

<p>FORMULA: C7H12NO8P</p>

<p>CHARGE: 0</p>

</body>

</notes>

</species>

<species id="M_AC_GLUT_SA_c" name="N-Acetyl-L-glutamate 5-semialdehyde" compartment="c" hasOnlySubstanceUnits="false" boundaryCondition="false" constant="false">

<notes>

<body xmlns="http://www.w3.org/1999/xhtml">

<p>FORMULA: C7H11NO4</p>

<p>CHARGE: 0</p>

</body>

</notes>

</species>

<species id="M_AORT_c" name="N-Acetylornithine" compartment="c" hasOnlySubstanceUnits="false" boundaryCondition="false" constant="false">

<notes>

<body xmlns="http://www.w3.org/1999/xhtml">

<p>FORMULA: C7H14N2O3</p>

<p>CHARGE: 0</p>

</body>

</notes>

</species>

<species id="M_GLUT_SA_c" name="L-Glutamate 5-semialdehyde" compartment="c" hasOnlySubstanceUnits="false" boundaryCondition="false" constant="false">

<notes>

<body xmlns="http://www.w3.org/1999/xhtml">

<p>FORMULA: C5H9NO3</p>

<p>CHARGE: 0</p>

</body>

</notes>

</species>

<species id="M_L_GLUT5P_c" name="L-Glutamyl 5-phosphate" compartment="c" hasOnlySubstanceUnits="false" boundaryCondition="false" constant="false">

<notes>

<body xmlns="http://www.w3.org/1999/xhtml">

<p>FORMULA: C5H10NO7P</p>

<p>CHARGE: 0</p>

</body>

</notes>

</species>

<species id="M_UREA_c" name="Urea, Carbamide" compartment="c" hasOnlySubstanceUnits="false" boundaryCondition="false" constant="false">

<notes>

<body xmlns="http://www.w3.org/1999/xhtml">

<p>FORMULA: CH4N2O</p>

<p>CHARGE: 0</p>

</body>

</notes>

</species>

<species id="M_UREA_CARB_c" name="Urea-1-carboxylate" compartment="c" hasOnlySubstanceUnits="false" boundaryCondition="false" constant="false">

<notes>

<body xmlns="http://www.w3.org/1999/xhtml">

<p>FORMULA: C2H4N2O3</p>

<p>CHARGE: 0</p>

</body>

</notes>

</species>

<species id="M_PYRR_3HY_5CARB_c" name="L-1-Pyrroline-3-hydroxy-5-carboxylate" compartment="c" hasOnlySubstanceUnits="false" boundaryCondition="false" constant="false">

<notes>

<body xmlns="http://www.w3.org/1999/xhtml">

<p>FORMULA: C5H7NO3</p>

<p>CHARGE: 0</p>

</body>

</notes>

</species>

<species id="M_HYGLU_SA_c" name="L-4-Hydroxyglutamate semialdehyde" compartment="c" hasOnlySubstanceUnits="false" boundaryCondition="false" constant="false">

<notes>

<body xmlns="http://www.w3.org/1999/xhtml">

<p>FORMULA: C5H9NO4</p>

<p>CHARGE: 0</p>

</body>

</notes>

</species>

<species id="M_ERY_HYGLU_c" name="L-erythro-4-Hydroxyglutamate" compartment="c" hasOnlySubstanceUnits="false" boundaryCondition="false" constant="false">

<notes>

<body xmlns="http://www.w3.org/1999/xhtml">

<p>FORMULA: C5H9NO5</p>

<p>CHARGE: 0</p>

</body>

</notes>

</species>

<species id="M_4ABUTALD_c" name="4-Aminobutyraldehyde" compartment="c" hasOnlySubstanceUnits="false" boundaryCondition="false" constant="false">

<notes>

<body xmlns="http://www.w3.org/1999/xhtml">

<p>FORMULA: C4H9NO</p>

<p>CHARGE: 0</p>

</body>

</notes>

</species>

<species id="M_tHY_PRO_c" name="trans-4-Hydroxy-L-proline" compartment="c" hasOnlySubstanceUnits="false" boundaryCondition="false" constant="false">

<notes>

<body xmlns="http://www.w3.org/1999/xhtml">

<p>FORMULA: C5H9NO3</p>

<p>CHARGE: 0</p>

</body>

</notes>

</species>

<species id="M_HY_OGLUTR_c" name="D-4-Hydroxy-2-oxoglutarate" compartment="c" hasOnlySubstanceUnits="false" boundaryCondition="false" constant="false">

<notes>

<body xmlns="http://www.w3.org/1999/xhtml">

<p>FORMULA: C5H6O6</p>

<p>CHARGE: 0</p>

</body>

</notes>

</species>

<species id="M_AGMT_c" name="Agmatine" compartment="c" hasOnlySubstanceUnits="false" boundaryCondition="false" constant="false">

<notes>

<body xmlns="http://www.w3.org/1999/xhtml">

<p>FORMULA: C5H14N4</p>

<p>CHARGE: 0</p>

</body>

</notes>

</species>

<species id="M_DHYDO_ARAB_HEPTP_c" name="2-Dehydro-3-deoxy-D-arabino-heptonate 7-phosphate" compartment="c" hasOnlySubstanceUnits="false" boundaryCondition="false" constant="false">

<notes>

<body xmlns="http://www.w3.org/1999/xhtml">

<p>FORMULA: C7H13O10P</p>

<p>CHARGE: 0</p>

</body>

</notes>

</species>

<species id="M_3DHYQNT_c" name="3-Dehydroquinate" compartment="c" hasOnlySubstanceUnits="false" boundaryCondition="false" constant="false">

<notes>

<body xmlns="http://www.w3.org/1999/xhtml">

<p>FORMULA: C7H10O6</p>

<p>CHARGE: 0</p>

</body>

</notes>

</species>

<species id="M_DHY_SHK_c" name="3-Dehydroshikimate" compartment="c" hasOnlySubstanceUnits="false" boundaryCondition="false" constant="false">

<notes>

<body xmlns="http://www.w3.org/1999/xhtml">

<p>FORMULA: C7H8O5</p>

<p>CHARGE: 0</p>

</body>

</notes>

</species>

<species id="M_SHK_c" name="Shikimate" compartment="c" hasOnlySubstanceUnits="false" boundaryCondition="false" constant="false">

<notes>

<body xmlns="http://www.w3.org/1999/xhtml">

<p>FORMULA: C7H10O5</p>

<p>CHARGE: 0</p>

</body>

</notes>

</species>

<species id="M_SHKP_c" name="Shikimate 5-phosphate" compartment="c" hasOnlySubstanceUnits="false" boundaryCondition="false" constant="false">

<notes>

<body xmlns="http://www.w3.org/1999/xhtml">

<p>FORMULA: C7H11O8P</p>

<p>CHARGE: 0</p>

</body>

</notes>

</species>

<species id="M_CVP_SHK_c" name="5-O-(1-Carboxyvinyl)-3-phosphoshikimate" compartment="c" hasOnlySubstanceUnits="false" boundaryCondition="false" constant="false">

<notes>

<body xmlns="http://www.w3.org/1999/xhtml">

<p>FORMULA: C10H13O10P</p>

<p>CHARGE: 0</p>

</body>

</notes>

</species>

<species id="M_ANTHRL_c" name="Anthranilate" compartment="c" hasOnlySubstanceUnits="false" boundaryCondition="false" constant="false">

<notes>

<body xmlns="http://www.w3.org/1999/xhtml">

<p>FORMULA: C7H7NO2</p>

<p>CHARGE: 0</p>

</body>

</notes>

</species>

<species id="M_IND_c" name="Indole" compartment="c" hasOnlySubstanceUnits="false" boundaryCondition="false" constant="false">

<notes>

<body xmlns="http://www.w3.org/1999/xhtml">

<p>FORMULA: C8H7N</p>

<p>CHARGE: 0</p>

</body>

</notes>

</species>

<species id="M_CPhA_dRBL5P_c" name="1-(2-Carboxyphenylamino)-1-deoxy-D-ribulose 5-phosphate" compartment="c" hasOnlySubstanceUnits="false" boundaryCondition="false" constant="false">

<notes>

<body xmlns="http://www.w3.org/1999/xhtml">

<p>FORMULA: C12H16NO9P</p>

<p>CHARGE: 0</p>

</body>

</notes>

</species>

<species id="M_5PRIBS_ANTRL_c" name="N-(5-Phospho-D-ribosyl)anthranilate" compartment="c" hasOnlySubstanceUnits="false" boundaryCondition="false" constant="false">

<notes>

<body xmlns="http://www.w3.org/1999/xhtml">

<p>FORMULA: C12H16NO9P</p>

<p>CHARGE: 0</p>

</body>

</notes>

</species>

<species id="M_PPHA_c" name="Prephenate" compartment="c" hasOnlySubstanceUnits="false" boundaryCondition="false" constant="false">

<notes>

<body xmlns="http://www.w3.org/1999/xhtml">

<p>FORMULA: C10H10O6</p>

<p>CHARGE: 0</p>

</body>

</notes>

</species>

<species id="M_CELBS_c" name="Cellobiose" compartment="c" hasOnlySubstanceUnits="false" boundaryCondition="false" constant="false">

<notes>

<body xmlns="http://www.w3.org/1999/xhtml">

<p>FORMULA: C12H22O11</p>

<p>CHARGE: 0</p>

</body>

</notes>

</species>

<species id="M_ADP_GLU_c" name="ADP-glucose" compartment="c" hasOnlySubstanceUnits="false" boundaryCondition="false" constant="false">

<notes>

<body xmlns="http://www.w3.org/1999/xhtml">

<p>FORMULA: C16H25N5O15P2</p>

<p>CHARGE: 0</p>

</body>

</notes>

</species>

```
<species id="M_GLY_c" name="Glycogen" compartment="c" hasOnlySubstanceUnits="false" boundaryCondition="false" constant="false">
  <notes>
    <body xmlns="http://www.w3.org/1999/xhtml">
      <p>FORMULA: (C6H10O5)n</p>
      <p>CHARGE: 0</p>
    </body>
  </notes>
</species>
<species id="M_MALT_c" name="Maltose" compartment="c" hasOnlySubstanceUnits="false" boundaryCondition="false" constant="false">
  <notes>
    <body xmlns="http://www.w3.org/1999/xhtml">
      <p>FORMULA: C12H22O11</p>
      <p>CHARGE: 0</p>
    </body>
  </notes>
</species>
<species id="M_IMALT_c" name="Isomaltose" compartment="c" hasOnlySubstanceUnits="false" boundaryCondition="false" constant="false">
  <notes>
    <body xmlns="http://www.w3.org/1999/xhtml">
      <p>FORMULA: </p>
      <p>CHARGE: 0</p>
    </body>
  </notes>
</species>
<species id="M_AC_GLUSAP_c" name="N-Acetyl-D-glucosamine 6-phosphate" compartment="c" hasOnlySubstanceUnits="false" boundaryCondition="false" constant="false">
  <notes>
```

<body xmlns="http://www.w3.org/1999/xhtml">

<p>FORMULA: C8H16NO9P</p>

<p>CHARGE: 0</p>

</body>

</notes>

</species>

<species id="M_AC_AGLUCSAPi_c" name="N-Acetyl-alpha-D-glucosamine 1-phosphate" compartment="c" hasOnlySubstanceUnits="false" boundaryCondition="false" constant="false">

<notes>

<body xmlns="http://www.w3.org/1999/xhtml">

<p>FORMULA: C8H16NO9P</p>

<p>CHARGE: 0</p>

</body>

</notes>

</species>

<species id="M_GLUCSA1P_c" name="alpha-D-Glucosamine 1-phosphate" compartment="c" hasOnlySubstanceUnits="false" boundaryCondition="false" constant="false">

<notes>

<body xmlns="http://www.w3.org/1999/xhtml">

<p>FORMULA: C6H14NO8P</p>

<p>CHARGE: 0</p>

</body>

</notes>

</species>

<species id="M_UDP_AC_MANNA_c" name="UDP-N-acetyl-D-mannosamine" compartment="c" hasOnlySubstanceUnits="false" boundaryCondition="false" constant="false">

<notes>

<body xmlns="http://www.w3.org/1999/xhtml">

<p>FORMULA: C17H27N3O17P2</p>

<p>CHARGE: 0</p>

</body>

</notes>

</species>

<species id="M_UDP_AC_CARBVIN_GLUCSA_c" name="UDP-N-acetyl-3-(1-carboxyvinyl)-D-glucosamine" compartment="c" hasOnlySubstanceUnits="false" boundaryCondition="false" constant="false">

<notes>

<body xmlns="http://www.w3.org/1999/xhtml">

<p>FORMULA: C20H29N3O19P2</p>

<p>CHARGE: 0</p>

</body>

</notes>

</species>

<species id="M_mINS3P_c" name="1D-myo-Inositol 3-phosphate" compartment="c" hasOnlySubstanceUnits="false" boundaryCondition="false" constant="false">

<notes>

<body xmlns="http://www.w3.org/1999/xhtml">

<p>FORMULA: C6H13O9P</p>

<p>CHARGE: 0</p>

</body>

</notes>

</species>

<species id="M_GLCP_c" name="sn-Glycerol 3-phosphate" compartment="c" hasOnlySubstanceUnits="false" boundaryCondition="false" constant="false">

<notes>

<body xmlns="http://www.w3.org/1999/xhtml">

<p>FORMULA: C3H9O6P</p>

<p>CHARGE: 0</p>

</body>

</notes>

</species>

<species id="M_I150CoA_c" name="Iso-C15:0 CoA" compartment="c" hasOnlySubstanceUnits="false" boundaryCondition="false" constant="false">

<notes>

<body xmlns="http://www.w3.org/1999/xhtml">

<p>FORMULA: C36H60N7O17P3S1</p>

<p>CHARGE: 0</p>

</body>

</notes>

</species>

<species id="M_AI150CoA_c" name="Anteiso-C15:0 CoA" compartment="c" hasOnlySubstanceUnits="false" boundaryCondition="false" constant="false">

<notes>

<body xmlns="http://www.w3.org/1999/xhtml">

<p>FORMULA: C36H60N7O17P3S1</p>

<p>CHARGE: 0</p>

</body>

</notes>

</species>

<species id="M_I160CoA_c" name="Iso-C16:0 CoA" compartment="c" hasOnlySubstanceUnits="false" boundaryCondition="false" constant="false">

<notes>

<body xmlns="http://www.w3.org/1999/xhtml">

<p>FORMULA: C37H62N7O17P3S1</p>

<p>CHARGE: 0</p>

</body>

</notes>

</species>

<species id="M_C160CoA_c" name="Palmitoyl-CoA (n-C16:0CoA)" compartment="c" hasOnlySubstanceUnits="false" boundaryCondition="false" constant="false">

<notes>

<body xmlns="http://www.w3.org/1999/xhtml">

<p>FORMULA: C37H66N7O17P3S</p>

<p>CHARGE: 0</p>

</body>

</notes>

</species>

<species id="M_C161CoA_c" name="(2E)-Hexadecenoyl-CoA (n-C16:1CoA)" compartment="c" hasOnlySubstanceUnits="false" boundaryCondition="false" constant="false">

<notes>

<body xmlns="http://www.w3.org/1999/xhtml">

<p>FORMULA: C37H64N7O17P3S</p>

<p>CHARGE: 0</p>

</body>

</notes>

</species>

<species id="M_I170CoA_c" name="Iso-C17:0 CoA" compartment="c" hasOnlySubstanceUnits="false" boundaryCondition="false" constant="false">

<notes>

<body xmlns="http://www.w3.org/1999/xhtml">

<p>FORMULA: C38H64N7O17P3S1</p>

<p>CHARGE: 0</p>

</body>

</notes>

</species>

<species id="M_AI171CoA_c" name="Anteiso-C17:1 CoA" compartment="c" hasOnlySubstanceUnits="false" boundaryCondition="false" constant="false">

<notes>

<body xmlns="http://www.w3.org/1999/xhtml">

<p>FORMULA: C38H62N7O17P3S1</p>

<p>CHARGE: 0</p>

</body>

</notes>

</species>

<species id="M_GLY3P_c" name="1-Acyl-sn-glycerol 3-phosphate" compartment="c" hasOnlySubstanceUnits="false" boundaryCondition="false" constant="false">

<notes>

<body xmlns="http://www.w3.org/1999/xhtml">

<p>FORMULA: C4H8O7PR</p>

<p>CHARGE: 0</p>

</body>

</notes>

</species>

<species id="M_PPTD_c" name="Phosphatidate" compartment="c" hasOnlySubstanceUnits="false" boundaryCondition="false" constant="false">

<notes>

<body xmlns="http://www.w3.org/1999/xhtml">

<p>FORMULA: C5H7O8PR2</p>

<p>CHARGE: 0</p>

</body>

</notes>

</species>

<species id="M_PMETHA_c" name="Phosphatidyl-N-methylethanolamine" compartment="c" hasOnlySubstanceUnits="false" boundaryCondition="false" constant="false">

<notes>

<body xmlns="http://www.w3.org/1999/xhtml">

<p>FORMULA: C8H14NO8PR2</p>

<p>CHARGE: 0</p>

</body>

</notes>

</species>

<species id="M_PDMETHA_c" name="phosphatidyl-N-dimethylethanolamine" compartment="c" hasOnlySubstanceUnits="false" boundaryCondition="false" constant="false">

<notes>

<body xmlns="http://www.w3.org/1999/xhtml">

<p>FORMULA: </p>

<p>CHARGE: 0</p>

</body>

</notes>

</species>

<species id="M_PCHOL_c" name="phosphatidylcholine" compartment="c" hasOnlySubstanceUnits="false" boundaryCondition="false" constant="false">

<notes>

<body xmlns="http://www.w3.org/1999/xhtml">

<p>FORMULA: </p>

<p>CHARGE: 0</p>

</body>

</notes>

</species>

<species id="M_LYPCHOL_c" name="Lysophosphatidylcholine" compartment="c" hasOnlySubstanceUnits="false" boundaryCondition="false" constant="false">

<notes>

<body xmlns="http://www.w3.org/1999/xhtml">

<p>FORMULA: </p>

<p>CHARGE: 0</p>

</body>

</notes>

</species>

<species id="M_DAG_c" name="1,2-Diacyl-sn-glycerol" compartment="c" hasOnlySubstanceUnits="false" boundaryCondition="false" constant="false">

<notes>

<body xmlns="http://www.w3.org/1999/xhtml">

<p>FORMULA: C5H6O5R2</p>

<p>CHARGE: 0</p>

</body>

</notes>

</species>

<species id="M_PPTD_SER_c" name="Phosphatidyl-L-serine" compartment="c" hasOnlySubstanceUnits="false" boundaryCondition="false" constant="false">

<notes>

<body xmlns="http://www.w3.org/1999/xhtml">

<p>FORMULA: C8H12NO10PR2</p>

<p>CHARGE: 0</p>

</body>

</notes>

</species>

<species id="M_CDP_DAG_c" name="CDP-1,2-diacylglycerol" compartment="c" hasOnlySubstanceUnits="false" boundaryCondition="false" constant="false">

<notes>

<body xmlns="http://www.w3.org/1999/xhtml">

<p>FORMULA: C14H19N3O15P2R2</p>

<p>CHARGE: 0</p>

</body>

</notes>

</species>

<species id="M_PGLYP_c" name="phosphatidylglycerophosphate" compartment="c" hasOnlySubstanceUnits="false" boundaryCondition="false" constant="false">

<notes>

<body xmlns="http://www.w3.org/1999/xhtml">

<p>FORMULA: </p>

<p>CHARGE: 0</p>

</body>

</notes>

</species>

<species id="M_PMINS3P_c" name="1-phosphatidyl-1D-myo-inositol 3-phosphate" compartment="c" hasOnlySubstanceUnits="false" boundaryCondition="false" constant="false">

<notes>

<body xmlns="http://www.w3.org/1999/xhtml">

<p>FORMULA: </p>

<p>CHARGE: 0</p>

</body>

</notes>

</species>

<species id="M_DGALSCRAMD_c" name="Digalactosylceramide" compartment="c" hasOnlySubstanceUnits="false" boundaryCondition="false" constant="false">

<notes>

<body xmlns="http://www.w3.org/1999/xhtml">

<p>FORMULA: C31H56NO13R</p>

<p>CHARGE: 0</p>

</body>

</notes>

</species>

<species id="M_GALS_CA_c" name="D-Galactosylceramide" compartment="c" hasOnlySubstanceUnits="false" boundaryCondition="false" constant="false">

<notes>

<body xmlns="http://www.w3.org/1999/xhtml">

<p>FORMULA: C25H46NO8R</p>

<p>CHARGE: 0</p>

</body>

</notes>

</species>

<species id="M_UDP_ACMUR_ALA_GLU_LYS_c" name="UDP-N-acetylmuramoyl-L-alanyl-gamma-D-glutamyl-L-lysine" compartment="c" hasOnlySubstanceUnits="false" boundaryCondition="false" constant="false">

<notes>

<body xmlns="http://www.w3.org/1999/xhtml">

<p>FORMULA: C34H55N7O24P2</p>

<p>CHARGE: 0</p>

</body>

</notes>

</species>

<species id="M_UDP_MURAC_ALA_GLU_LYS_ALA_ALA_c" name="UDPMurAc(oyl-L-Ala-D-gamma-Glu-L-Lys-D-Ala-D-Ala)" compartment="c" hasOnlySubstanceUnits="false" boundaryCondition="false" constant="false">

<notes>

<body xmlns="http://www.w3.org/1999/xhtml">

<p>FORMULA: C40H65N9O26P2</p>

<p>CHARGE: 0</p>

</body>

</notes>

</species>

<species id="M_dt_pc_UNDECP_c" name="Undecaprenyl phosphate" compartment="c" hasOnlySubstanceUnits="false" boundaryCondition="false" constant="false">

<notes>

<body xmlns="http://www.w3.org/1999/xhtml">

<p>FORMULA: C55H91O4P</p>

<p>CHARGE: 0</p>

</body>

</notes>

</species>

<species id="M_MURAC_ALA_GLU_LYS_ALA_ALA_DP_UNDECP_c" name="MurAc(oyl-L-Ala-D-gamma-Glu-L-Lys-D-Ala-D-Ala)-diphospho- undecaprenol" compartment="c" hasOnlySubstanceUnits="false" boundaryCondition="false" constant="false">

<notes>

<body xmlns="http://www.w3.org/1999/xhtml">

<p>FORMULA: C86H143N7O21P2</p>

<p>CHARGE: 0</p>

</body>

</notes>

</species>

<species id="M_Undp_ACMUR_ALA_GLU_DAPIM_AALA_c" name="Undecaprenyl-diphospho-N-acetylmuramoyl-L-alanyl-D-glutamyl-meso-2,6-diaminopimeloyl-D-alanyl-D-alanine" compartment="c" hasOnlySubstanceUnits="false" boundaryCondition="false" constant="false">

<notes>

<body xmlns="http://www.w3.org/1999/xhtml">

<p>FORMULA: C87H143N7O23P2</p>

<p>CHARGE: 0</p>

</body>

</notes>

</species>

<species id="M_Undp_ACMUR_ACGLSA_ALA_GLU_LYS_AALA_c" name="Undecaprenyl-diphospho-N-acetylmuramoyl-(N-acetylglucosamine)-L- alanyl-gamma-D-glutamyl-L-lysyl-D-alanyl-D-alanine, GlcNAc-(1->4)-Mur2Ac(oyl-L-Ala-gamma-D-Glu-L-Lys-D-Ala-D-Ala)- diphosphoundecaprenol" compartment="c" hasOnlySubstanceUnits="false" boundaryCondition="false" constant="false">

<notes>

<body xmlns="http://www.w3.org/1999/xhtml">

<p>FORMULA: C94H156N8O26P2</p>

<p>CHARGE: 0</p>

</body>

</notes>

</species>

<species id="M_Undp_ACMUR_ACGLSA_ALA_GLU_DAPIM_AALA_c" name="Undecaprenyl-diphospho-N-acetylmuramoyl-(N-acetylglucosamine)-L- alanyl-D-glutamyl-meso-2,6-diaminopimeloyl-D-alanyl-D-alanine" compartment="c" hasOnlySubstanceUnits="false" boundaryCondition="false" constant="false">

<notes>

<body xmlns="http://www.w3.org/1999/xhtml">

<p>FORMULA: C95H156N8O28P2</p>

<p>CHARGE: 0</p>

</body>

</notes>

</species>

<species id="M_dt_pc_UNDECPPi_c" name="di-trans,poly-cis-Undecaprenyl diphosphate" compartment="c" hasOnlySubstanceUnits="false" boundaryCondition="false" constant="false">

<notes>

<body xmlns="http://www.w3.org/1999/xhtml">

<p>FORMULA: C55H92O7P2</p>

<p>CHARGE: 0</p>

</body>

</notes>

</species>

<species id="M_MGLX_c" name="Methylglyoxal" compartment="c" hasOnlySubstanceUnits="false" boundaryCondition="false" constant="false">

<notes>

<body xmlns="http://www.w3.org/1999/xhtml">

<p>FORMULA: C3H4O2</p>

<p>CHARGE: 0</p>

</body>

</notes>

</species>

<species id="M_GLUTAT_c" name="glutathione" compartment="c" hasOnlySubstanceUnits="false" boundaryCondition="false" constant="false">

<notes>

<body xmlns="http://www.w3.org/1999/xhtml">

<p>FORMULA: </p>

<p>CHARGE: 0</p>

</body>

</notes>

</species>

<species id="M_LACGLUT_c" name="Lactoylglutathione" compartment="c" hasOnlySubstanceUnits="false" boundaryCondition="false" constant="false">

<notes>

<body xmlns="http://www.w3.org/1999/xhtml">

<p>FORMULA: </p>

<p>CHARGE: 0</p>

</body>

</notes>

</species>

<species id="M_ACPi_c" name="Acetyl phosphate" compartment="c" hasOnlySubstanceUnits="false" boundaryCondition="false" constant="false">

<notes>

<body xmlns="http://www.w3.org/1999/xhtml">

<p>FORMULA: C2H5O5P</p>

<p>CHARGE: 0</p>

</body>

</notes>

</species>

<species id="M_ACADNA_c" name="Acetyl adenylate" compartment="c" hasOnlySubstanceUnits="false" boundaryCondition="false" constant="false">

<notes>

<body xmlns="http://www.w3.org/1999/xhtml">

<p>FORMULA: C12H16N5O8P</p>

<p>CHARGE: 0</p>

</body>

</notes>

</species>

<species id="M_HY_AC_c" name="Glycolate" compartment="c" hasOnlySubstanceUnits="false" boundaryCondition="false" constant="false">

<notes>

<body xmlns="http://www.w3.org/1999/xhtml">

<p>FORMULA: C2H4O3</p>

<p>CHARGE: 0</p>

</body>

</notes>

</species>

<species id="M_FOR_c" name="Formate" compartment="c" hasOnlySubstanceUnits="false" boundaryCondition="false" constant="false">

<notes>

<body xmlns="http://www.w3.org/1999/xhtml">

<p>FORMULA: CH2O2</p>

<p>CHARGE: 0</p>

</body>

</notes>

</species>

<species id="M_METHF_c" name="5,10-Methenyltetrahydrofolate" compartment="c" hasOnlySubstanceUnits="false" boundaryCondition="false" constant="false">

<notes>

<body xmlns="http://www.w3.org/1999/xhtml">

<p>FORMULA: C20H22N7O6</p>

<p>CHARGE: 0</p>

</body>

</notes>

</species>

<species id="M_4HY_2OGLUTR_c" name="4-Hydroxy-2-oxoglutarate" compartment="c" hasOnlySubstanceUnits="false" boundaryCondition="false" constant="false">

<notes>

<body xmlns="http://www.w3.org/1999/xhtml">

<p>FORMULA: C5H6O6</p>

<p>CHARGE: 0</p>

</body>

</notes>

</species>

<species id="M_H2O2_c" name="Hydrogen peroxide" compartment="c" hasOnlySubstanceUnits="false" boundaryCondition="false" constant="false">

<notes>

<body xmlns="http://www.w3.org/1999/xhtml">

<p>FORMULA: H2O2</p>

<p>CHARGE: 0</p>

</body>

</notes>

</species>

<species id="M_2P_GLYCA_c" name="2-Phosphoglycolate" compartment="c" hasOnlySubstanceUnits="false" boundaryCondition="false" constant="false">

<notes>

<body xmlns="http://www.w3.org/1999/xhtml">

<p>FORMULA: C2H5O6P</p>

<p>CHARGE: 0</p>

</body>

</notes>

</species>

<species id="M_2HYBUTNA_c" name="2-Hydroxybutyrate" compartment="c" hasOnlySubstanceUnits="false" boundaryCondition="false" constant="false">

<notes>

<body xmlns="http://www.w3.org/1999/xhtml">

<p>FORMULA: C4H8O3</p>

<p>CHARGE: 0</p>

</body>

</notes>

</species>

<species id="M_PROP_ADNSPi_c" name="Propionyladenylate" compartment="c" hasOnlySubstanceUnits="false" boundaryCondition="false" constant="false">

<notes>

<body xmlns="http://www.w3.org/1999/xhtml">

<p>FORMULA: C13H18N5O8P</p>

<p>CHARGE: 0</p>

</body>

</notes>

</species>

<species id="M_PROP_c" name="Propanoate" compartment="c" hasOnlySubstanceUnits="false" boundaryCondition="false" constant="false">

<notes>

<body xmlns="http://www.w3.org/1999/xhtml">

<p>FORMULA: C3H6O2</p>

<p>CHARGE: 0</p>

</body>

</notes>

</species>

<species id="M_FOLNA_c" name="5-Formyltetrahydrofolate" compartment="c" hasOnlySubstanceUnits="false" boundaryCondition="false" constant="false">

<notes>

<body xmlns="http://www.w3.org/1999/xhtml">

<p>FORMULA: C20H23N7O7</p>

<p>CHARGE: 0</p>

</body>

</notes>

</species>

<species id="M_ENZ_CYS_c" name="Thiamine biosynthesis intermediate 2" compartment="c" hasOnlySubstanceUnits="false" boundaryCondition="false" constant="false">

<notes>

<body xmlns="http://www.w3.org/1999/xhtml">

<p>FORMULA: C4H6N2O2SR2</p>

<p>CHARGE: 0</p>

</body>

</notes>

</species>

<species id="M_ENZ_SLUFA_CYS_c" name="[Enzyme]-S-sulfanylcysteine" compartment="c" hasOnlySubstanceUnits="false" boundaryCondition="false" constant="false">

<notes>

<body xmlns="http://www.w3.org/1999/xhtml">

<p>FORMULA: C4H6N2O2S2R2</p>

<p>CHARGE: 0</p>

</body>

</notes>

</species>

<species id="M_4A_M5PM_PYRM_c" name="4-Amino-2-methyl-5-phosphomethylpyrimidine, 4-Amino-5-phosphomethyl-2-methylpyrimidine" compartment="c" hasOnlySubstanceUnits="false" boundaryCondition="false" constant="false">

<notes>

<body xmlns="http://www.w3.org/1999/xhtml">

<p>FORMULA: C6H10N3O4P</p>

<p>CHARGE: 0</p>

</body>

</notes>

</species>

<species id="M_2M4A_HYMPYRMPPi_c" name="2-Methyl-4-amino-5-hydroxymethylpyrimidine diphosphate" compartment="c" hasOnlySubstanceUnits="false" boundaryCondition="false" constant="false">

<notes>

<body xmlns="http://www.w3.org/1999/xhtml">

<p>FORMULA: C6H11N3O7P2</p>

<p>CHARGE: 0</p>

</body>

</notes>

</species>

<species id="M_TOPYRM_c" name="Toxopyrimidine" compartment="c" hasOnlySubstanceUnits="false" boundaryCondition="false" constant="false">

<notes>

<body xmlns="http://www.w3.org/1999/xhtml">

<p>FORMULA: C6H9N3O</p>

<p>CHARGE: 0</p>

</body>

</notes>

</species>

<species id="M_4M_PE_THZ_c" name="4-Methyl-5-(2-phosphoethyl)-thiazole" compartment="c" hasOnlySubstanceUnits="false" boundaryCondition="false" constant="false">

<notes>

<body xmlns="http://www.w3.org/1999/xhtml">

<p>FORMULA: C6H10NO4PS</p>

<p>CHARGE: 0</p>

</body>

</notes>

</species>

<species id="M_TMP_c" name="Thiamine monophosphate (TMP)" compartment="c" hasOnlySubstanceUnits="false" boundaryCondition="false" constant="false">

<notes>

<body xmlns="http://www.w3.org/1999/xhtml">

<p>FORMULA: C12H18N4O4PS</p>

<p>CHARGE: 0</p>

```
        </body>

      </notes>

    </species>

    <species id="M_IMAC_c" name="2-iminoacetate" compartment="c" hasOnlySubstanceUnits="false"
boundaryCondition="false" constant="false">

      <notes>

        <body xmlns="http://www.w3.org/1999/xhtml">

          <p>FORMULA: C2H2NO2</p>

          <p>CHARGE: 0</p>

        </body>

      </notes>

    </species>

    <species id="M_dXYLL5P_c" name="1-Deoxy-D-xylulose 5-phosphate" compartment="c" hasOnlySubstanceUnits="false"
boundaryCondition="false" constant="false">

      <notes>

        <body xmlns="http://www.w3.org/1999/xhtml">

          <p>FORMULA: C5H11O7P</p>

          <p>CHARGE: 0</p>

        </body>

      </notes>

    </species>

    <species id="M_THZ_c" name="Thiazole" compartment="c" hasOnlySubstanceUnits="false" boundaryCondition="false"
constant="false">

      <notes>

        <body xmlns="http://www.w3.org/1999/xhtml">

          <p>FORMULA: </p>

          <p>CHARGE: 0</p>

        </body>

      </notes>

    </species>
```

<species id="M_DHY_BUTNPi_c" name="3,4-Dihydroxy-2-butanone 4-phosphate" compartment="c" hasOnlySubstanceUnits="false" boundaryCondition="false" constant="false">

<notes>

<body xmlns="http://www.w3.org/1999/xhtml">

<p>FORMULA: C4H9O6P</p>

<p>CHARGE: 0</p>

</body>

</notes>

</species>

<species id="M_DA_PRIBSA_PYMDN_c" name="2,5-Diamino-6-(5'-phosphoribosylamino)-4-pyrimidineone" compartment="c" hasOnlySubstanceUnits="false" boundaryCondition="false" constant="false">

<notes>

<body xmlns="http://www.w3.org/1999/xhtml">

<p>FORMULA: C9H16N5O8P</p>

<p>CHARGE: 0</p>

</body>

</notes>

</species>

<species id="M_AP_RIB_URA_c" name="5-Amino-6-(5'-phosphoribosylamino)uracil" compartment="c" hasOnlySubstanceUnits="false" boundaryCondition="false" constant="false">

<notes>

<body xmlns="http://www.w3.org/1999/xhtml">

<p>FORMULA: C9H15N4O9P</p>

<p>CHARGE: 0</p>

</body>

</notes>

</species>

<species id="M_APRIBTA_URA_c" name="5-Amino-6-(5'-phospho-D-ribitylamino)uracil" compartment="c" hasOnlySubstanceUnits="false" boundaryCondition="false" constant="false">

<notes>

<body xmlns="http://www.w3.org/1999/xhtml">

<p>FORMULA: C9H17N4O9P</p>

<p>CHARGE: 0</p>

</body>

</notes>

</species>

<species id="M_RBFLV_c" name="Riboflavin (Vitamin B2)" compartment="c" hasOnlySubstanceUnits="false" boundaryCondition="false" constant="false">

<notes>

<body xmlns="http://www.w3.org/1999/xhtml">

<p>FORMULA: C17H20N4O6</p>

<p>CHARGE: 0</p>

</body>

</notes>

</species>

<species id="M_NC_D_RNUC_c" name="Nicotinate D-ribonucleotide" compartment="c" hasOnlySubstanceUnits="false" boundaryCondition="false" constant="false">

<notes>

<body xmlns="http://www.w3.org/1999/xhtml">

<p>FORMULA: C11H15NO9P</p>

<p>CHARGE: 0</p>

</body>

</notes>

</species>

<species id="M_CBMCo_c" name="Cobamide coenzyme" compartment="c" hasOnlySubstanceUnits="false" boundaryCondition="false" constant="false">

<notes>

<body xmlns="http://www.w3.org/1999/xhtml">

<p>FORMULA: C72H100CoN18O17P</p>

<p>CHARGE: 0</p>

</body>

</notes>

</species>

<species id="M_NIA_c" name="Nicotinate" compartment="c" hasOnlySubstanceUnits="false" boundaryCondition="false" constant="false">

<notes>

<body xmlns="http://www.w3.org/1999/xhtml">

<p>FORMULA: C6H5NO2</p>

<p>CHARGE: 0</p>

</body>

</notes>

</species>

<species id="M_PRIBS_DMBENZ_IMDZ_c" name="N1-(5-Phospho-alpha-D-ribosyl)-5,6-dimethylbenzimidazole" compartment="c" hasOnlySubstanceUnits="false" boundaryCondition="false" constant="false">

<notes>

<body xmlns="http://www.w3.org/1999/xhtml">

<p>FORMULA: C14H19N2O7P</p>

<p>CHARGE: 0</p>

</body>

</notes>

</species>

<species id="M_5AM_RIBAM_URA_c" name="5-Amino-6-(1-D-ribitylamino)uracil" compartment="c" hasOnlySubstanceUnits="false" boundaryCondition="false" constant="false">

<notes>

<body xmlns="http://www.w3.org/1999/xhtml">

<p>FORMULA: C9H16N4O6</p>

<p>CHARGE: 0</p>

</body>

</notes>

</species>

<species id="M_DM_DR_LUM_c" name="6,7-Dimethyl-8-(1-D-ribityl)lumazine" compartment="c" hasOnlySubstanceUnits="false" boundaryCondition="false" constant="false">

<notes>

<body xmlns="http://www.w3.org/1999/xhtml">

<p>FORMULA: C13H18N4O6</p>

<p>CHARGE: 0</p>

</body>

</notes>

</species>

<species id="M_OPHY_THR_c" name="O-Phospho-4-hydroxy-L-threonine" compartment="c" hasOnlySubstanceUnits="false" boundaryCondition="false" constant="false">

<notes>

<body xmlns="http://www.w3.org/1999/xhtml">

<p>FORMULA: C4H10NO7P</p>

<p>CHARGE: 0</p>

</body>

</notes>

</species>

<species id="M_HY_THR_c" name="4-Hydroxy-L-threonine" compartment="c" hasOnlySubstanceUnits="false" boundaryCondition="false" constant="false">

<notes>

<body xmlns="http://www.w3.org/1999/xhtml">

<p>FORMULA: C4H9NO4</p>

<p>CHARGE: 0</p>

</body>

</notes>

</species>

<species id="M_RIBS_NCTA_c" name="N-Ribosylnicotinamide" compartment="c" hasOnlySubstanceUnits="false" boundaryCondition="false" constant="false">

<notes>

<body xmlns="http://www.w3.org/1999/xhtml">

<p>FORMULA: C11H15N2O5</p>

<p>CHARGE: 0</p>

</body>

</notes>

</species>

<species id="M_NAMD_c" name="Nicotinamide" compartment="c" hasOnlySubstanceUnits="false" boundaryCondition="false" constant="false">

<notes>

<body xmlns="http://www.w3.org/1999/xhtml">

<p>FORMULA: C6H6N2O</p>

<p>CHARGE: 0</p>

</body>

</notes>

</species>

<species id="M_NICRIB_c" name="Nicotinate D-ribonucleoside" compartment="c" hasOnlySubstanceUnits="false" boundaryCondition="false" constant="false">

<notes>

<body xmlns="http://www.w3.org/1999/xhtml">

<p>FORMULA: C11H14NO6</p>

<p>CHARGE: 0</p>

</body>

</notes>

</species>

<species id="M_NMN_c" name="Nicotinamide mononucleotide" compartment="c" hasOnlySubstanceUnits="false" boundaryCondition="false" constant="false">

<notes>

<body xmlns="http://www.w3.org/1999/xhtml">

<p>FORMULA: C11H15N2O8P</p>

<p>CHARGE: 0</p>

</body>

</notes>

</species>

<species id="M_QA_c" name="Quinolinate" compartment="c" hasOnlySubstanceUnits="false" boundaryCondition="false" constant="false">

<notes>

<body xmlns="http://www.w3.org/1999/xhtml">

<p>FORMULA: C7H5NO4</p>

<p>CHARGE: 0</p>

</body>

</notes>

</species>

<species id="M_DA_NAD_c" name="Deamino-NAD+" compartment="c" hasOnlySubstanceUnits="false" boundaryCondition="false" constant="false">

<notes>

<body xmlns="http://www.w3.org/1999/xhtml">

<p>FORMULA: C21H27N6O15P2</p>

<p>CHARGE: 0</p>

</body>

</notes>

</species>

<species id="M_IMASP_c" name="Iminoaspartate" compartment="c" hasOnlySubstanceUnits="false" boundaryCondition="false" constant="false">

<notes>

<body xmlns="http://www.w3.org/1999/xhtml">

<p>FORMULA: C4H5NO4</p>

<p>CHARGE: 0</p>

</body>

</notes>

</species>

<species id="M_PIM_c" name="Pimelate" compartment="c" hasOnlySubstanceUnits="false" boundaryCondition="false" constant="false">

<notes>

<body xmlns="http://www.w3.org/1999/xhtml">

<p>FORMULA: C7H12O4</p>

<p>CHARGE: 0</p>

</body>

</notes>

</species>

<species id="M_PIMCoA_c" name="Pimeloyl-CoA" compartment="c" hasOnlySubstanceUnits="false" boundaryCondition="false" constant="false">

<notes>

<body xmlns="http://www.w3.org/1999/xhtml">

<p>FORMULA: C28H46N7O19P3S</p>

<p>CHARGE: 0</p>

</body>

</notes>

</species>

<species id="M_DTBTN_c" name="Dethiobiotin" compartment="c" hasOnlySubstanceUnits="false" boundaryCondition="false" constant="false">

<notes>

<body xmlns="http://www.w3.org/1999/xhtml">

<p>FORMULA: C10H18N2O3</p>

<p>CHARGE: 0</p>

</body>

</notes>

</species>

<species id="M_S_c" name="Sulfur" compartment="c" hasOnlySubstanceUnits="false" boundaryCondition="false" constant="false">

<notes>

```
        <body xmlns="http://www.w3.org/1999/xhtml">
          <p>FORMULA: S</p>
          <p>CHARGE: 0</p>
        </body>
      </notes>
    </species>
    <species id="M_BTN_c" name="Biotin" compartment="c" hasOnlySubstanceUnits="false" boundaryCondition="false" constant="false">
      <notes>
        <body xmlns="http://www.w3.org/1999/xhtml">
          <p>FORMULA: C10H16N2O3S</p>
          <p>CHARGE: 0</p>
        </body>
      </notes>
    </species>
    <species id="M_dADNS_c" name="5&apos;-Deoxyadenosine" compartment="c" hasOnlySubstanceUnits="false" boundaryCondition="false" constant="false">
      <notes>
        <body xmlns="http://www.w3.org/1999/xhtml">
          <p>FORMULA: C10H13N5O3</p>
          <p>CHARGE: 0</p>
        </body>
      </notes>
    </species>
    <species id="M_BTN_AMP_c" name="Biotinyl-5&apos;-AMP" compartment="c" hasOnlySubstanceUnits="false" boundaryCondition="false" constant="false">
      <notes>
        <body xmlns="http://www.w3.org/1999/xhtml">
          <p>FORMULA: C20H28N7O9PS</p>
          <p>CHARGE: 0</p>
```

</body>

</notes>

</species>

<species id="M_APCARB_c" name="Apo-[carboxylase]" compartment="c" hasOnlySubstanceUnits="false" boundaryCondition="false" constant="false">

<notes>

<body xmlns="http://www.w3.org/1999/xhtml">

<p>FORMULA: C7H13N3O2R2</p>

<p>CHARGE: 0</p>

</body>

</notes>

</species>

<species id="M_OCT_ACP_c" name="Octanoyl-[acp]" compartment="c" hasOnlySubstanceUnits="false" boundaryCondition="false" constant="false">

<notes>

<body xmlns="http://www.w3.org/1999/xhtml">

<p>FORMULA: C8H15OSR</p>

<p>CHARGE: 0</p>

</body>

</notes>

</species>

<species id="M_LP_ACP_c" name="Lipoyl-[acp]" compartment="c" hasOnlySubstanceUnits="false" boundaryCondition="false" constant="false">

<notes>

<body xmlns="http://www.w3.org/1999/xhtml">

<p>FORMULA: C8H13OS3R</p>

<p>CHARGE: 0</p>

</body>

</notes>

</species>

<species id="M_PN6_OCTLYS_c" name="Protein N6-(octanoyl)lysine" compartment="c" hasOnlySubstanceUnits="false" boundaryCondition="false" constant="false">

<notes>

<body xmlns="http://www.w3.org/1999/xhtml">

<p>FORMULA: C8H16NOR</p>

<p>CHARGE: 0</p>

</body>

</notes>

</species>

<species id="M_PN6_LPLYS_c" name="Protein N6-(lipoyl)lysine" compartment="c" hasOnlySubstanceUnits="false" boundaryCondition="false" constant="false">

<notes>

<body xmlns="http://www.w3.org/1999/xhtml">

<p>FORMULA: C8H14NOS2R</p>

<p>CHARGE: 0</p>

</body>

</notes>

</species>

<species id="M_APPRO_c" name="Apoprotein" compartment="c" hasOnlySubstanceUnits="false" boundaryCondition="false" constant="false">

<notes>

<body xmlns="http://www.w3.org/1999/xhtml">

<p>FORMULA: NH2R</p>

<p>CHARGE: 0</p>

</body>

</notes>

</species>

<species id="M_ACP_c" name="Acyl-carrier protein (ACP)" compartment="c" hasOnlySubstanceUnits="false" boundaryCondition="false" constant="false">

<notes>

<body xmlns="http://www.w3.org/1999/xhtml">

<p>FORMULA: HSR</p>

<p>CHARGE: 0</p>

</body>

</notes>

</species>

<species id="M_DHY_MBUTNA_c" name="2,3-Dihydroxy-3-methylbutanoate" compartment="c" hasOnlySubstanceUnits="false" boundaryCondition="false" constant="false">

<notes>

<body xmlns="http://www.w3.org/1999/xhtml">

<p>FORMULA: C5H10O4</p>

<p>CHARGE: 0</p>

</body>

</notes>

</species>

<species id="M_DHY_PAN_c" name="2-Dehydropantoate" compartment="c" hasOnlySubstanceUnits="false" boundaryCondition="false" constant="false">

<notes>

<body xmlns="http://www.w3.org/1999/xhtml">

<p>FORMULA: C6H10O4</p>

<p>CHARGE: 0</p>

</body>

</notes>

</species>

<species id="M_PPANT_c" name="(R)-4'-Phosphopantothenate" compartment="c" hasOnlySubstanceUnits="false" boundaryCondition="false" constant="false">

<notes>

<body xmlns="http://www.w3.org/1999/xhtml">

<p>FORMULA: C9H18NO8P</p>

<p>CHARGE: 0</p>

</body>

</notes>

</species>

<species id="M_PPANTN_CYS_c" name="(R)-4'-Phosphopantothenoyl-L-cysteine" compartment="c" hasOnlySubstanceUnits="false" boundaryCondition="false" constant="false">

<notes>

<body xmlns="http://www.w3.org/1999/xhtml">

<p>FORMULA: C12H23N2O9PS</p>

<p>CHARGE: 0</p>

</body>

</notes>

</species>

<species id="M_PANTE4Pi_c" name="D-Pantetheine 4'-phosphate" compartment="c" hasOnlySubstanceUnits="false" boundaryCondition="false" constant="false">

<notes>

<body xmlns="http://www.w3.org/1999/xhtml">

<p>FORMULA: C11H23N2O7PS</p>

<p>CHARGE: 0</p>

</body>

</notes>

</species>

<species id="M_DPCoA_c" name="Dephospho-CoA" compartment="c" hasOnlySubstanceUnits="false" boundaryCondition="false" constant="false">

<notes>

<body xmlns="http://www.w3.org/1999/xhtml">

<p>FORMULA: C21H35N7O13P2S</p>

<p>CHARGE: 0</p>

</body>

</notes>

</species>

<species id="M_FAPYRM_NTP_c" name="Formamidopyrimidine nucleoside triphosphate" compartment="c" hasOnlySubstanceUnits="false" boundaryCondition="false" constant="false">

<notes>

<body xmlns="http://www.w3.org/1999/xhtml">

<p>FORMULA: C10H18N5O15P3</p>

<p>CHARGE: 0</p>

</body>

</notes>

</species>

<species id="M_DAPYRM_NTP_c" name="2,5-Diaminopyrimidine nucleoside triphosphate" compartment="c" hasOnlySubstanceUnits="false" boundaryCondition="false" constant="false">

<notes>

<body xmlns="http://www.w3.org/1999/xhtml">

<p>FORMULA: C9H18N5O14P3</p>

<p>CHARGE: 0</p>

</body>

</notes>

</species>

<species id="M_DA_TPiTHY_OPENTAOPYRM_c" name="2,5-Diamino-6-(5'-triphosphoryl-3',4'-trihydroxy-2'-oxopentyl)- amino-4-oxopyrimidine" compartment="c" hasOnlySubstanceUnits="false" boundaryCondition="false" constant="false">

<notes>

<body xmlns="http://www.w3.org/1999/xhtml">

<p>FORMULA: C9H18N5O14P3</p>

<p>CHARGE: 0</p>

</body>

</notes>

</species>

<species id="M_AHYRY_THYPROP_DHYPTRN_TPi_c" name="2-Amino-4-hydroxy-6-(erythro-1,2,3-trihydroxypropyl)dihydropteridine triphosphate" compartment="c" hasOnlySubstanceUnits="false" boundaryCondition="false" constant="false">

<notes>

<body xmlns="http://www.w3.org/1999/xhtml">

<p>FORMULA: C9H16N5O13P3</p>

<p>CHARGE: 0</p>

</body>

</notes>

</species>

<species id="M_DHY_NPTRN_c" name="Dihydroneopterin, 7,8-Dihydroneopterin" compartment="c" hasOnlySubstanceUnits="false" boundaryCondition="false" constant="false">

<notes>

<body xmlns="http://www.w3.org/1999/xhtml">

<p>FORMULA: C9H13N5O4</p>

<p>CHARGE: 0</p>

</body>

</notes>

</species>

<species id="M_GLYCALD_c" name="Glycolaldehyde" compartment="c" hasOnlySubstanceUnits="false" boundaryCondition="false" constant="false">

<notes>

<body xmlns="http://www.w3.org/1999/xhtml">

<p>FORMULA: C2H4O2</p>

<p>CHARGE: 0</p>

</body>

</notes>

</species>

<species id="M_AHY_HYM_DHYPTR_c" name="2-Amino-4-hydroxy-6-hydroxymethyl-7,8-dihydropteridine" compartment="c" hasOnlySubstanceUnits="false" boundaryCondition="false" constant="false">

<notes>

<body xmlns="http://www.w3.org/1999/xhtml">

<p>FORMULA: C7H9N5O2</p>

<p>CHARGE: 0</p>

</body>

</notes>

</species>

<species id="M_ADHY_DPOM_PTRDN_c" name="2-Amino-7,8-dihydro-4-hydroxy-6-(diphosphooxymethyl)pteridine" compartment="c" hasOnlySubstanceUnits="false" boundaryCondition="false" constant="false">

<notes>

<body xmlns="http://www.w3.org/1999/xhtml">

<p>FORMULA: C7H11N5O8P2</p>

<p>CHARGE: 0</p>

</body>

</notes>

</species>

<species id="M_ABEE_c" name="p-Aminobenzoate" compartment="c" hasOnlySubstanceUnits="false" boundaryCondition="false" constant="false">

<notes>

<body xmlns="http://www.w3.org/1999/xhtml">

<p>FORMULA: C7H7NO2</p>

<p>CHARGE: 0</p>

</body>

</notes>

</species>

<species id="M_DHY_PTR_c" name="Dihydropteroate" compartment="c" hasOnlySubstanceUnits="false" boundaryCondition="false" constant="false">

<notes>

<body xmlns="http://www.w3.org/1999/xhtml">

<p>FORMULA: C14H14N6O3</p>

<p>CHARGE: 0</p>

</body>

</notes>

</species>

<species id="M_THF_GLUT_c" name="THF-L-glutamate" compartment="c" hasOnlySubstanceUnits="false" boundaryCondition="false" constant="false">

<notes>

<body xmlns="http://www.w3.org/1999/xhtml">

<p>FORMULA: C24H30N8O9</p>

<p>CHARGE: 0</p>

</body>

</notes>

</species>

<species id="M_DHF_c" name="Dihydrofolate" compartment="c" hasOnlySubstanceUnits="false" boundaryCondition="false" constant="false">

<notes>

<body xmlns="http://www.w3.org/1999/xhtml">

<p>FORMULA: C19H21N7O6</p>

<p>CHARGE: 0</p>

</body>

</notes>

</species>

<species id="M_MEROLPi_c" name="2-C-Methyl-D-erythritol 4-phosphate" compartment="c" hasOnlySubstanceUnits="false" boundaryCondition="false" constant="false">

<notes>

<body xmlns="http://www.w3.org/1999/xhtml">

<p>FORMULA: C5H13O7P</p>

<p>CHARGE: 0</p>

</body>

</notes>

</species>

<species id="M_CYTPPi_MEROL_c" name="4-(Cytidine 5'-diphospho)-2-C-methyl-D-erythritol" compartment="c" hasOnlySubstanceUnits="false" boundaryCondition="false" constant="false">

<notes>

<body xmlns="http://www.w3.org/1999/xhtml">

<p>FORMULA: C14H25N3O14P2</p>

<p>CHARGE: 0</p>

</body>

</notes>

</species>

<species id="M_PCYTPPi_MEROL_c" name="2-Phospho-4-(cytidine 5'-diphospho)-2-C-methyl-D-erythritol" compartment="c" hasOnlySubstanceUnits="false" boundaryCondition="false" constant="false">

<notes>

<body xmlns="http://www.w3.org/1999/xhtml">

<p>FORMULA: C14H26N3O17P3</p>

<p>CHARGE: 0</p>

</body>

</notes>

</species>

<species id="M_MEROL_CYCPPi_c" name="2-C-Methyl-D-erythritol 2,4-cyclodiphosphate" compartment="c" hasOnlySubstanceUnits="false" boundaryCondition="false" constant="false">

<notes>

<body xmlns="http://www.w3.org/1999/xhtml">

<p>FORMULA: C5H12O9P2</p>

<p>CHARGE: 0</p>

</body>

</notes>

</species>

<species id="M_R_FRDX_c" name="Reduced ferredoxin" compartment="c" hasOnlySubstanceUnits="false" boundaryCondition="false" constant="false">

<notes>

<body xmlns="http://www.w3.org/1999/xhtml">

<p>FORMULA: </p>

<p>CHARGE: 0</p>

</body>

</notes>

</species>

<species id="M_HY_MBUTEPPi_c" name="1-Hydroxy-2-methyl-2-butenyl 4-diphosphate; (E)-4-Hydroxy-3-methyl-but-2-enyl diphosphate; (E)-4-Hydroxy-3-methylbut-2-en-1-yl diphosphate" compartment="c" hasOnlySubstanceUnits="false" boundaryCondition="false" constant="false">

<notes>

<body xmlns="http://www.w3.org/1999/xhtml">

<p>FORMULA: C5H12O8P2</p>

<p>CHARGE: 0</p>

</body>

</notes>

</species>

<species id="M_O_FRDX_c" name="Oxidized ferredoxin" compartment="c" hasOnlySubstanceUnits="false" boundaryCondition="false" constant="false">

<notes>

<body xmlns="http://www.w3.org/1999/xhtml">

<p>FORMULA: </p>

<p>CHARGE: 0</p>

</body>

</notes>

</species>

<species id="M_DMAPP_c" name="Dimethylallyl diphosphate" compartment="c" hasOnlySubstanceUnits="false" boundaryCondition="false" constant="false">

<notes>

<body xmlns="http://www.w3.org/1999/xhtml">

<p>FORMULA: C5H12O7P2</p>

<p>CHARGE: 0</p>

</body>

</notes>

</species>

<species id="M_GRN_PPi_c" name="Geranyl diphosphate" compartment="c" hasOnlySubstanceUnits="false" boundaryCondition="false" constant="false">

<notes>

<body xmlns="http://www.w3.org/1999/xhtml">

<p>FORMULA: C10H20O7P2</p>

<p>CHARGE: 0</p>

</body>

</notes>

</species>

<species id="M_FAR_PPi_c" name="trans,trans-Farnesyl diphosphate" compartment="c" hasOnlySubstanceUnits="false" boundaryCondition="false" constant="false">

<notes>

<body xmlns="http://www.w3.org/1999/xhtml">

<p>FORMULA: C15H28O7P2</p>

<p>CHARGE: 0</p>

</body>

</notes>

</species>

<species id="M_GRN_GRNPPi_c" name="Geranylgeranyl diphosphate" compartment="c" hasOnlySubstanceUnits="false" boundaryCondition="false" constant="false">

<notes>

<body xmlns="http://www.w3.org/1999/xhtml">

<p>FORMULA: C20H36O7P2</p>

<p>CHARGE: 0</p>

</body>

</notes>

</species>

<species id="M_CRVN_c" name="(+)-(S)-Carvone" compartment="c" hasOnlySubstanceUnits="false" boundaryCondition="false" constant="false">

<notes>

<body xmlns="http://www.w3.org/1999/xhtml">

<p>FORMULA: C10H14O</p>

<p>CHARGE: 0</p>

</body>

</notes>

</species>

<species id="M_IDHY_CRVN_c" name=" (1R,4S)-Iso-dihydrocarvone" compartment="c" hasOnlySubstanceUnits="false" boundaryCondition="false" constant="false">

<notes>

<body xmlns="http://www.w3.org/1999/xhtml">

<p>FORMULA: C10H16O</p>

<p>CHARGE: 0</p>

</body>

</notes>

</species>

<species id="M_PHTEN_c" name="Phytoene" compartment="c" hasOnlySubstanceUnits="false" boundaryCondition="false" constant="false">

<notes>

<body xmlns="http://www.w3.org/1999/xhtml">

<p>FORMULA: C40H64</p>

<p>CHARGE: 0</p>

</body>

</notes>

</species>

<species id="M_PHFLN_c" name="Phytofluene" compartment="c" hasOnlySubstanceUnits="false" boundaryCondition="false" constant="false">

<notes>

<body xmlns="http://www.w3.org/1999/xhtml">

<p>FORMULA: C40H62</p>

<p>CHARGE: 0</p>

</body>

</notes>

</species>

<species id="M_NRSPENE_c" name="Neurosporene" compartment="c" hasOnlySubstanceUnits="false" boundaryCondition="false" constant="false">

<notes>

<body xmlns="http://www.w3.org/1999/xhtml">

<p>FORMULA: C40H58</p>

<p>CHARGE: 0</p>

</body>

</notes>

</species>

<species id="M_LCPN_c" name="Lycopene" compartment="c" hasOnlySubstanceUnits="false" boundaryCondition="false" constant="false">

<notes>

<body xmlns="http://www.w3.org/1999/xhtml">

<p>FORMULA: C40H56</p>

<p>CHARGE: 0</p>

</body>

</notes>

</species>

<species id="M_ENITRN_c" name="Ethylnitronate" compartment="c" hasOnlySubstanceUnits="false" boundaryCondition="false" constant="false">

<notes>

<body xmlns="http://www.w3.org/1999/xhtml">

<p>FORMULA: C2H4NO2</p>

<p>CHARGE: 0</p>

</body>

</notes>

</species>

<species id="M_NO2_c" name="Nitrite" compartment="c" hasOnlySubstanceUnits="false" boundaryCondition="false" constant="false">

<notes>

<body xmlns="http://www.w3.org/1999/xhtml">

<p>FORMULA: HNO2</p>

<p>CHARGE: 0</p>

</body>

</notes>

</species>

<species id="M_PAPS_c" name="3'-Phosphoadenylyl sulfate (PAPS)" compartment="c" hasOnlySubstanceUnits="false" boundaryCondition="false" constant="false">

<notes>

<body xmlns="http://www.w3.org/1999/xhtml">

<p>FORMULA: C10H15N5O13P2S</p>

<p>CHARGE: 0</p>

</body>

</notes>

</species>

<species id="M_PAP_c" name="Phosphoadenosine phosphate" compartment="c" hasOnlySubstanceUnits="false" boundaryCondition="false" constant="false">

<notes>

<body xmlns="http://www.w3.org/1999/xhtml">

<p>FORMULA: C10H15N5O10P2</p>

<p>CHARGE: 0</p>

</body>

</notes>

</species>

<species id="M_CYSTHN_c" name="Cystathionine" compartment="c" hasOnlySubstanceUnits="false" boundaryCondition="false" constant="false">

<notes>

<body xmlns="http://www.w3.org/1999/xhtml">

<p>FORMULA: C7H14N2O4S</p>

<p>CHARGE: 0</p>

</body>

</notes>

</species>

<species id="M_H4MPT_c" name="5,6,7,8-Tetrahydromethanopterin (H4MPT)" compartment="c" hasOnlySubstanceUnits="false" boundaryCondition="false" constant="false">

<notes>

<body xmlns="http://www.w3.org/1999/xhtml">

<p>FORMULA: C30H45N6O16P</p>

<p>CHARGE: 0</p>

</body>

</notes>

</species>

<species id="M_METHMPTR_c" name="5,10-Methylenetetrahydromethanopterin" compartment="c" hasOnlySubstanceUnits="false" boundaryCondition="false" constant="false">

<notes>

<body xmlns="http://www.w3.org/1999/xhtml">

<p>FORMULA: C31H45N6O16P</p>

<p>CHARGE: 0</p>

</body>

</notes>

</species>

<species id="M_PSULFLAC_c" name="(2R)-O-Phospho-3-sulfolactate" compartment="c" hasOnlySubstanceUnits="false" boundaryCondition="false" constant="false">

<notes>

<body xmlns="http://www.w3.org/1999/xhtml">

<p>FORMULA: C3H7O9PS</p>

<p>CHARGE: 0</p>

</body>

</notes>

</species>

<species id="M_SULFLAC_c" name="(2R)-3-Sulfolactate" compartment="c" hasOnlySubstanceUnits="false" boundaryCondition="false" constant="false">

<notes>

<body xmlns="http://www.w3.org/1999/xhtml">

<p>FORMULA: C3H6O6S</p>

<p>CHARGE: 0</p>

</body>

</notes>

</species>

<species id="M_UPPHYRGN_I_c" name="Uroporphyrinogen I" compartment="c" hasOnlySubstanceUnits="false" boundaryCondition="false" constant="false">

<notes>

<body xmlns="http://www.w3.org/1999/xhtml">

<p>FORMULA: C40H44N4O16</p>

<p>CHARGE: 0</p>

</body>

</notes>

</species>

<species id="M_CPPHYRGN_I_c" name="Coproporphyrinogen I" compartment="c" hasOnlySubstanceUnits="false" boundaryCondition="false" constant="false">

<notes>

<body xmlns="http://www.w3.org/1999/xhtml">

<p>FORMULA: C36H44N4O8</p>

<p>CHARGE: 0</p>

</body>

</notes>

</species>

<species id="M_UPPHYRGN_III_c" name="Uroporphyrinogen III" compartment="c" hasOnlySubstanceUnits="false" boundaryCondition="false" constant="false">

<notes>

<body xmlns="http://www.w3.org/1999/xhtml">

<p>FORMULA: C40H44N4O16</p>

<p>CHARGE: 0</p>

</body>

</notes>

</species>

<species id="M_CPPPHYRGN_c" name="Coproporphyrinogen III" compartment="c" hasOnlySubstanceUnits="false" boundaryCondition="false" constant="false">

<notes>

<body xmlns="http://www.w3.org/1999/xhtml">

<p>FORMULA: C36H44N4O8</p>

<p>CHARGE: 0</p>

</body>

</notes>

</species>

<species id="M_ALEVU_c" name="5-Aminolevulinate" compartment="c" hasOnlySubstanceUnits="false" boundaryCondition="false" constant="false">

<notes>

<body xmlns="http://www.w3.org/1999/xhtml">

<p>FORMULA: C5H9NO3</p>

<p>CHARGE: 0</p>

</body>

</notes>

</species>

<species id="M_PPBLGN_c" name="Porphobilinogen" compartment="c" hasOnlySubstanceUnits="false" boundaryCondition="false" constant="false">

<notes>

<body xmlns="http://www.w3.org/1999/xhtml">

<p>FORMULA: C10H14N2O4</p>

<p>CHARGE: 0</p>

</body>

</notes>

</species>

<species id="M_HY_MBILN_c" name="Hydroxymethylbilane" compartment="c" hasOnlySubstanceUnits="false" boundaryCondition="false" constant="false">

<notes>

<body xmlns="http://www.w3.org/1999/xhtml">

<p>FORMULA: C40H46N4O17</p>

<p>CHARGE: 0</p>

</body>

</notes>

</species>

<species id="M_PPPHYRGN_c" name="Protoporphyrinogen IX" compartment="c" hasOnlySubstanceUnits="false" boundaryCondition="false" constant="false">

<notes>

<body xmlns="http://www.w3.org/1999/xhtml">

<p>FORMULA: C34H40N4O4</p>

<p>CHARGE: 0</p>

</body>

</notes>

</species>

<species id="M_PPPHYR_c" name="Protoporphyrin IX" compartment="c" hasOnlySubstanceUnits="false" boundaryCondition="false" constant="false">

<notes>

<body xmlns="http://www.w3.org/1999/xhtml">

<p>FORMULA: C34H34N4O4</p>

<p>CHARGE: 0</p>

</body>

</notes>

</species>

<species id="M_Fe2_c" name="Ferrous ion" compartment="c" hasOnlySubstanceUnits="false" boundaryCondition="false" constant="false">

<notes>

<body xmlns="http://www.w3.org/1999/xhtml">

<p>FORMULA: Fe</p>

<p>CHARGE: 0</p>

</body>

</notes>

</species>

<species id="M_HEME_c" name="Heme" compartment="c" hasOnlySubstanceUnits="false" boundaryCondition="false" constant="false">

<notes>

<body xmlns="http://www.w3.org/1999/xhtml">

<p>FORMULA: C34H32FeN4O4</p>

<p>CHARGE: 0</p>

</body>

</notes>

</species>

<species id="M_GLU_SA_c" name="L-Glutamate 1-semialdehyde" compartment="c" hasOnlySubstanceUnits="false" boundaryCondition="false" constant="false">

<notes>

<body xmlns="http://www.w3.org/1999/xhtml">

<p>FORMULA: C5H9NO3</p>

<p>CHARGE: 0</p>

```
        </body>
      </notes>
    </species>
    <species id="M_PRCRN_c" name="Precorrin 2" compartment="c" hasOnlySubstanceUnits="false"
boundaryCondition="false" constant="false">
      <notes>
        <body xmlns="http://www.w3.org/1999/xhtml">
          <p>FORMULA: C42H48N4O16</p>
          <p>CHARGE: 0</p>
        </body>
      </notes>
    </species>
    <species id="M_Mg_c" name="Magnesium" compartment="c" hasOnlySubstanceUnits="false" boundaryCondition="false"
constant="false">
      <notes>
        <body xmlns="http://www.w3.org/1999/xhtml">
          <p>FORMULA: Mg</p>
          <p>CHARGE: 0</p>
        </body>
      </notes>
    </species>
    <species id="M_Mg_PPPHYR_c" name="Magnesium protoporphyrin" compartment="c" hasOnlySubstanceUnits="false"
boundaryCondition="false" constant="false">
      <notes>
        <body xmlns="http://www.w3.org/1999/xhtml">
          <p>FORMULA: C34H32MgN4O4</p>
          <p>CHARGE: 0</p>
        </body>
      </notes>
    </species>
```

<species id="M_HEMO_c" name="Heme O" compartment="c" hasOnlySubstanceUnits="false" boundaryCondition="false" constant="false">

<notes>

<body xmlns="http://www.w3.org/1999/xhtml">

<p>FORMULA: C49H58FeN4O5</p>

<p>CHARGE: 0</p>

</body>

</notes>

</species>

<species id="M_SRHYCLR_c" name="Sirohydrochlorin" compartment="c" hasOnlySubstanceUnits="false" boundaryCondition="false" constant="false">

<notes>

<body xmlns="http://www.w3.org/1999/xhtml">

<p>FORMULA: C42H46N4O16</p>

<p>CHARGE: 0</p>

</body>

</notes>

</species>

<species id="M_SHEME_c" name="Siroheme" compartment="c" hasOnlySubstanceUnits="false" boundaryCondition="false" constant="false">

<notes>

<body xmlns="http://www.w3.org/1999/xhtml">

<p>FORMULA: C42H44FeN4O16</p>

<p>CHARGE: 0</p>

</body>

</notes>

</species>

<species id="M_PRCRN3A_c" name="Precorrin 3A" compartment="c" hasOnlySubstanceUnits="false" boundaryCondition="false" constant="false">

<notes>

<body xmlns="http://www.w3.org/1999/xhtml">

<p>FORMULA: C43H50N4O16</p>

<p>CHARGE: 0</p>

</body>

</notes>

</species>

<species id="M_CoSRHYCLR_c" name="Cobalt-sirohydrochlorin" compartment="c" hasOnlySubstanceUnits="false" boundaryCondition="false" constant="false">

<notes>

<body xmlns="http://www.w3.org/1999/xhtml">

<p>FORMULA: C42H44CoN4O16</p>

<p>CHARGE: 0</p>

</body>

</notes>

</species>

<species id="M_CoFIII_c" name="Cobalt-factor III" compartment="c" hasOnlySubstanceUnits="false" boundaryCondition="false" constant="false">

<notes>

<body xmlns="http://www.w3.org/1999/xhtml">

<p>FORMULA: C43H46CoN4O16</p>

<p>CHARGE: 0</p>

</body>

</notes>

</species>

<species id="M_CoPRCRN3_c" name="Cobalt-precorrin 3" compartment="c" hasOnlySubstanceUnits="false" boundaryCondition="false" constant="false">

<notes>

<body xmlns="http://www.w3.org/1999/xhtml">

<p>FORMULA: C43H48CoN4O16</p>

<p>CHARGE: 0</p>

</body>

</notes>

</species>

<species id="M_CoPRCRN4_c" name="Cobalt-precorrin 4" compartment="c" hasOnlySubstanceUnits="false" boundaryCondition="false" constant="false">

<notes>

<body xmlns="http://www.w3.org/1999/xhtml">

<p>FORMULA: C44H50CoN4O16</p>

<p>CHARGE: 0</p>

</body>

</notes>

</species>

<species id="M_PRCRN3B_c" name="Precorrin 3B" compartment="c" hasOnlySubstanceUnits="false" boundaryCondition="false" constant="false">

<notes>

<body xmlns="http://www.w3.org/1999/xhtml">

<p>FORMULA: C43H50N4O17</p>

<p>CHARGE: 0</p>

</body>

</notes>

</species>

<species id="M_PRCRN4_c" name="Precorrin 4" compartment="c" hasOnlySubstanceUnits="false" boundaryCondition="false" constant="false">

<notes>

<body xmlns="http://www.w3.org/1999/xhtml">

<p>FORMULA: C44H52N4O17</p>

<p>CHARGE: 0</p>

</body>

</notes>

</species>

<species id="M_PRCRN5_c" name="Precorrin 5" compartment="c" hasOnlySubstanceUnits="false" boundaryCondition="false" constant="false">

<notes>

<body xmlns="http://www.w3.org/1999/xhtml">

<p>FORMULA: C45H54N4O17</p>

<p>CHARGE: 0</p>

</body>

</notes>

</species>

<species id="M_CoPRCRN5A_c" name="Cobalt-precorrin 5A" compartment="c" hasOnlySubstanceUnits="false" boundaryCondition="false" constant="false">

<notes>

<body xmlns="http://www.w3.org/1999/xhtml">

<p>FORMULA: C45H52CoN4O16</p>

<p>CHARGE: 0</p>

</body>

</notes>

</species>

<species id="M_PRCRN6B_c" name="Precorrin 6B" compartment="c" hasOnlySubstanceUnits="false" boundaryCondition="false" constant="false">

<notes>

<body xmlns="http://www.w3.org/1999/xhtml">

<p>FORMULA: C44H56N4O16</p>

<p>CHARGE: 0</p>

</body>

</notes>

</species>

<species id="M_PRCRN8X_c" name="Precorrin 8" compartment="c" hasOnlySubstanceUnits="false" boundaryCondition="false" constant="false">

<notes>

<body xmlns="http://www.w3.org/1999/xhtml">

<p>FORMULA: C45H60N4O14</p>

<p>CHARGE: 0</p>

</body>

</notes>

</species>

<species id="M_HOBYRNA_c" name="Hydrogenobyrinate" compartment="c" hasOnlySubstanceUnits="false" boundaryCondition="false" constant="false">

<notes>

<body xmlns="http://www.w3.org/1999/xhtml">

<p>FORMULA: C45H60N4O14</p>

<p>CHARGE: 0</p>

</body>

</notes>

</species>

<species id="M_CoPRCRN8_c" name="Cobalt-precorrin 8" compartment="c" hasOnlySubstanceUnits="false" boundaryCondition="false" constant="false">

<notes>

<body xmlns="http://www.w3.org/1999/xhtml">

<p>FORMULA: C45H59CoN4O14</p>

<p>CHARGE: 0</p>

</body>

</notes>

</species>

<species id="M_CBRYNA_c" name="Cobyrinate" compartment="c" hasOnlySubstanceUnits="false" boundaryCondition="false" constant="false">

<notes>

<body xmlns="http://www.w3.org/1999/xhtml">

<p>FORMULA: C45H59CoN4O14</p>

<p>CHARGE: 0</p>

</body>

</notes>

</species>

<species id="M_HOBYRNDA_c" name="Hydrogenobyrinate diamide" compartment="c" hasOnlySubstanceUnits="false" boundaryCondition="false" constant="false">

<notes>

<body xmlns="http://www.w3.org/1999/xhtml">

<p>FORMULA: C45H62N6O12</p>

<p>CHARGE: 0</p>

</body>

</notes>

</species>

<species id="M_Co2ABYRDA_c" name="Cob(II)yrinate diamide" compartment="c" hasOnlySubstanceUnits="false" boundaryCondition="false" constant="false">

<notes>

<body xmlns="http://www.w3.org/1999/xhtml">

<p>FORMULA: C45H61CoN6O12</p>

<p>CHARGE: 0</p>

</body>

</notes>

</species>

<species id="M_ADNS_CABYRDA_c" name="Adenosyl cobyrinate diamide" compartment="c" hasOnlySubstanceUnits="false" boundaryCondition="false" constant="false">

<notes>

<body xmlns="http://www.w3.org/1999/xhtml">

<p>FORMULA: C55H73CoN11O15</p>

<p>CHARGE: 0</p>

</body>

</notes>

</species>

<species id="M_ADNS_CABYRHA_c" name="Adenosyl cobyrinate hexaamide" compartment="c" hasOnlySubstanceUnits="false" boundaryCondition="false" constant="false">

<notes>

<body xmlns="http://www.w3.org/1999/xhtml">

<p>FORMULA: C55H77CoN15O11</p>

<p>CHARGE: 0</p>

</body>

</notes>

</species>

<species id="M_CO_LPAREN_II_RPAREN__c" name="Cobalt" compartment="c" hasOnlySubstanceUnits="false" boundaryCondition="false" constant="false">

<notes>

<body xmlns="http://www.w3.org/1999/xhtml">

<p>FORMULA: </p>

<p>CHARGE: 0</p>

</body>

</notes>

</species>

<species id="M_CABYRDA_c" name="Cob(I)yrinate diamide" compartment="c" hasOnlySubstanceUnits="false" boundaryCondition="false" constant="false">

<notes>

<body xmlns="http://www.w3.org/1999/xhtml">

<p>FORMULA: C45H61CoN6O12</p>

<p>CHARGE: 0</p>

</body>

</notes>

</species>

<species id="M_CBAMD_c" name="Cobinamide" compartment="c" hasOnlySubstanceUnits="false" boundaryCondition="false" constant="false">

<notes>

<body xmlns="http://www.w3.org/1999/xhtml">

<p>FORMULA: C48H72CoN11O8</p>

<p>CHARGE: 0</p>

</body>

</notes>

</species>

<species id="M_ADNS_CBNA_c" name="Adenosyl cobinamide" compartment="c" hasOnlySubstanceUnits="false" boundaryCondition="false" constant="false">

<notes>

<body xmlns="http://www.w3.org/1999/xhtml">

<p>FORMULA: C58H84CoN16O11</p>

<p>CHARGE: 0</p>

</body>

</notes>

</species>

<species id="M_APRNPi_c" name="D-1-Aminopropan-2-ol O-phosphate" compartment="c" hasOnlySubstanceUnits="false" boundaryCondition="false" constant="false">

<notes>

<body xmlns="http://www.w3.org/1999/xhtml">

<p>FORMULA: C3H10NO4P</p>

<p>CHARGE: 0</p>

</body>

</notes>

</species>

<species id="M_ADNS_CBNAPi_c" name="Adenosyl cobinamide phosphate" compartment="c" hasOnlySubstanceUnits="false" boundaryCondition="false" constant="false">

<notes>

<body xmlns="http://www.w3.org/1999/xhtml">

<p>FORMULA: C58H85CoN16O14P</p>

<p>CHARGE: 0</p>

```
</body>
</notes>
</species>
<species id="M_ADNS_CBNA_GDP_c" name="Adenosine-GDP-cobinamide" compartment="c" hasOnlySubstanceUnits="false" boundaryCondition="false" constant="false">
<notes>
<body xmlns="http://www.w3.org/1999/xhtml">
<p>FORMULA: C68H97CoN21O21P2</p>
<p>CHARGE: 0</p>
</body>
</notes>
</species>
<species id="M_ARIBZ_c" name="alpha-Ribazole" compartment="c" hasOnlySubstanceUnits="false" boundaryCondition="false" constant="false">
<notes>
<body xmlns="http://www.w3.org/1999/xhtml">
<p>FORMULA: C14H18N2O4</p>
<p>CHARGE: 0</p>
</body>
</notes>
</species>
<species id="M_TRE_c" name="Trehalose" compartment="c" hasOnlySubstanceUnits="false" boundaryCondition="false" constant="false">
<notes>
<body xmlns="http://www.w3.org/1999/xhtml">
<p>FORMULA: C12H22O11</p>
<p>CHARGE: 0</p>
</body>
</notes>
</species>
```

<species id="M_TRE6P_c" name="Trehalose 6-phosphate" compartment="c" hasOnlySubstanceUnits="false" boundaryCondition="false" constant="false">

<notes>

<body xmlns="http://www.w3.org/1999/xhtml">

<p>FORMULA: C12H23O14P</p>

<p>CHARGE: 0</p>

</body>

</notes>

</species>

<species id="M_IVCoA_c" name="Isovaleryl-CoA" compartment="c" hasOnlySubstanceUnits="false" boundaryCondition="false" constant="false">

<notes>

<body xmlns="http://www.w3.org/1999/xhtml">

<p>FORMULA: C26H44N7O17P3S</p>

<p>CHARGE: 0</p>

</body>

</notes>

</species>

<species id="M_MCROCoA_c" name="3-Methylcrotonyl-CoA" compartment="c" hasOnlySubstanceUnits="false" boundaryCondition="false" constant="false">

<notes>

<body xmlns="http://www.w3.org/1999/xhtml">

<p>FORMULA: C26H42N7O17P3S</p>

<p>CHARGE: 0</p>

</body>

</notes>

</species>

<species id="M_3MGLUTNCoA_c" name="3-Methylglutaconyl-CoA " compartment="c" hasOnlySubstanceUnits="false" boundaryCondition="false" constant="false">

<notes>

<body xmlns="http://www.w3.org/1999/xhtml">

<p>FORMULA: C27H42N7O19P3S</p>

<p>CHARGE: 0</p>

</body>

</notes>

</species>

<species id="M_ACACoA_c" name="Acetoacetyl-CoA" compartment="c" hasOnlySubstanceUnits="false" boundaryCondition="false" constant="false">

<notes>

<body xmlns="http://www.w3.org/1999/xhtml">

<p>FORMULA: C25H40N7O18P3S</p>

<p>CHARGE: 0</p>

</body>

</notes>

</species>

<species id="M_MQL_c" name="Menaquinol" compartment="c" hasOnlySubstanceUnits="false" boundaryCondition="false" constant="false">

<notes>

<body xmlns="http://www.w3.org/1999/xhtml">

<p>FORMULA: C16H18O2(C5H8)n</p>

<p>CHARGE: 0</p>

</body>

</notes>

</species>

<species id="M_A_PROPOL_c" name="(R)-1-Amino-2-propanol" compartment="c" hasOnlySubstanceUnits="false" boundaryCondition="false" constant="false">

<notes>

<body xmlns="http://www.w3.org/1999/xhtml">

<p>FORMULA: C3H9NO</p>

<p>CHARGE: 0</p>

</body>

</notes>

</species>

<species id="M_4A4CORM_c" name="(2S)-2-Amino-4-deoxychorismate" compartment="c" hasOnlySubstanceUnits="false" boundaryCondition="false" constant="false">

<notes>

<body xmlns="http://www.w3.org/1999/xhtml">

<p>FORMULA: C10H11NO5</p>

<p>CHARGE: 0</p>

</body>

</notes>

</species>

<species id="M_MALYCoA_c" name="(3S)-3-carboxy-3-hydroxypropionyl-CoA" compartment="c" hasOnlySubstanceUnits="false" boundaryCondition="false" constant="false">

<notes>

<body xmlns="http://www.w3.org/1999/xhtml">

<p>FORMULA: C25H40N7O20P3S</p>

<p>CHARGE: 0</p>

</body>

</notes>

</species>

<species id="M_AI171_c" name="14-Methyl-hexadecenoate (anteiso-17:1)" compartment="c" hasOnlySubstanceUnits="false" boundaryCondition="false" constant="false">

<notes>

<body xmlns="http://www.w3.org/1999/xhtml">

<p>FORMULA: </p>

<p>CHARGE: 0</p>

</body>

</notes>

</species>

<species id="M_AI150_c" name="12-Methyl-tetradecanoate (anteiso-15:0)" compartment="c" hasOnlySubstanceUnits="false" boundaryCondition="false" constant="false">

<notes>

<body xmlns="http://www.w3.org/1999/xhtml">

<p>FORMULA: </p>

<p>CHARGE: 0</p>

</body>

</notes>

</species>

<species id="M_I150_c" name="13-Methyltetradecanoate (iso-15:0)" compartment="c" hasOnlySubstanceUnits="false" boundaryCondition="false" constant="false">

<notes>

<body xmlns="http://www.w3.org/1999/xhtml">

<p>FORMULA: </p>

<p>CHARGE: 0</p>

</body>

</notes>

</species>

<species id="M_I160_c" name="14-Methylpentadecanoate (iso-16:0)" compartment="c" hasOnlySubstanceUnits="false" boundaryCondition="false" constant="false">

<notes>

<body xmlns="http://www.w3.org/1999/xhtml">

<p>FORMULA: </p>

<p>CHARGE: 0</p>

</body>

</notes>

</species>

<species id="M_I170_c" name="15-Methyl hexadecenoate (iso-17:0)" compartment="c" hasOnlySubstanceUnits="false" boundaryCondition="false" constant="false">

<notes>

<body xmlns="http://www.w3.org/1999/xhtml">

<p>FORMULA: </p>

<p>CHARGE: 0</p>

</body>

</notes>

</species>

<species id="M_I130_c" name="11-Methyldodecanoate (iso-13:0)" compartment="c" hasOnlySubstanceUnits="false" boundaryCondition="false" constant="false">

<notes>

<body xmlns="http://www.w3.org/1999/xhtml">

<p>FORMULA: </p>

<p>CHARGE: 0</p>

</body>

</notes>

</species>

<species id="M_I110_c" name="9-methyldecanoate (iso-11:0)" compartment="c" hasOnlySubstanceUnits="false" boundaryCondition="false" constant="false">

<notes>

<body xmlns="http://www.w3.org/1999/xhtml">

<p>FORMULA: </p>

<p>CHARGE: 0</p>

</body>

</notes>

</species>

<species id="M_C160_c" name="Hexadecanoate" compartment="c" hasOnlySubstanceUnits="false" boundaryCondition="false" constant="false">

<notes>

<body xmlns="http://www.w3.org/1999/xhtml">

<p>FORMULA: C16H32O2</p>

<p>CHARGE: 0</p>

```
        </body>
      </notes>
    </species>
    <species id="M_C161_c" name="Palmitoleate" compartment="c" hasOnlySubstanceUnits="false" boundaryCondition="false" constant="false">
      <notes>
        <body xmlns="http://www.w3.org/1999/xhtml">
          <p>FORMULA: C16H30O2</p>
          <p>CHARGE: 0</p>
        </body>
      </notes>
    </species>
    <species id="M_gCARO_c" name="gamma-Carotene" compartment="c" hasOnlySubstanceUnits="false" boundaryCondition="false" constant="false">
      <notes>
        <body xmlns="http://www.w3.org/1999/xhtml">
          <p>FORMULA: C40H56</p>
          <p>CHARGE: 0</p>
        </body>
      </notes>
    </species>
    <species id="M_bCARO_c" name="beta-Carotene" compartment="c" hasOnlySubstanceUnits="false" boundaryCondition="false" constant="false">
      <notes>
        <body xmlns="http://www.w3.org/1999/xhtml">
          <p>FORMULA: C40H56</p>
          <p>CHARGE: 0</p>
        </body>
      </notes>
    </species>
```

```
<species id="M_CRTX_c" name="beta-Cryptoxanthin" compartment="c" hasOnlySubstanceUnits="false" boundaryCondition="false" constant="false">
  <notes>
    <body xmlns="http://www.w3.org/1999/xhtml">
      <p>FORMULA: C40H56O</p>
      <p>CHARGE: 0</p>
    </body>
  </notes>
</species>
<species id="M_ZXT_c" name="Zeaxanthin" compartment="c" hasOnlySubstanceUnits="false" boundaryCondition="false" constant="false">
  <notes>
    <body xmlns="http://www.w3.org/1999/xhtml">
      <p>FORMULA: C40H56O2</p>
      <p>CHARGE: 0</p>
    </body>
  </notes>
</species>
<species id="M_tCRTX_c" name="Thermocryptoxanthin" compartment="c" hasOnlySubstanceUnits="false" boundaryCondition="false" constant="false">
  <notes>
    <body xmlns="http://www.w3.org/1999/xhtml">
      <p>FORMULA: C57H86O7</p>
      <p>CHARGE: 0</p>
    </body>
  </notes>
</species>
<species id="M_bCRTX_GLU_c" name="beta-Cryptoxanthin glucoside" compartment="c" hasOnlySubstanceUnits="false" boundaryCondition="false" constant="false">
  <notes>
```

<body xmlns="http://www.w3.org/1999/xhtml">
<p>FORMULA: </p>
<p>CHARGE: 0</p>
</body>
</notes>
</species>
<species id="M_ZXT_GLU_c" name="Zeaxanthin glucoside" compartment="c" hasOnlySubstanceUnits="false" boundaryCondition="false" constant="false">
<notes>
<body xmlns="http://www.w3.org/1999/xhtml">
<p>FORMULA: </p>
<p>CHARGE: 0</p>
</body>
</notes>
</species>
<species id="M_tZXT_GLU_c" name="Thermozeaxanthin glucoside" compartment="c" hasOnlySubstanceUnits="false" boundaryCondition="false" constant="false">
<notes>
<body xmlns="http://www.w3.org/1999/xhtml">
<p>FORMULA: </p>
<p>CHARGE: 0</p>
</body>
</notes>
</species>
<species id="M_tbZXT_c" name="Thermobiszeaxanthin" compartment="c" hasOnlySubstanceUnits="false" boundaryCondition="false" constant="false">
<notes>
<body xmlns="http://www.w3.org/1999/xhtml">
<p>FORMULA: C78H124O14</p>
<p>CHARGE: 0</p>

</body>

</notes>

</species>

<species id="M_RHY_BUTCoA_c" name="(R)-3-Hydroxybutanoyl-CoA" compartment="c" hasOnlySubstanceUnits="false" boundaryCondition="false" constant="false">

<notes>

<body xmlns="http://www.w3.org/1999/xhtml">

<p>FORMULA: C25H42N7O18P3S</p>

<p>CHARGE: 0</p>

</body>

</notes>

</species>

<species id="M_KVCoA_c" name="beta-ketovaleryl-CoA" compartment="c" hasOnlySubstanceUnits="false" boundaryCondition="false" constant="false">

<notes>

<body xmlns="http://www.w3.org/1999/xhtml">

<p>FORMULA: C26H38N7O18P3S</p>

<p>CHARGE: 0</p>

</body>

</notes>

</species>

<species id="M_HVCoA_c" name="(R)-3-hydroxyvaleryl-CoA" compartment="c" hasOnlySubstanceUnits="false" boundaryCondition="false" constant="false">

<notes>

<body xmlns="http://www.w3.org/1999/xhtml">

<p>FORMULA: C26H40N7O18P3S</p>

<p>CHARGE: 0</p>

</body>

</notes>

</species>

<species id="M_PHB_c" name="Polyhydroxybutyrate" compartment="c" hasOnlySubstanceUnits="false" boundaryCondition="false" constant="false">

<notes>

<body xmlns="http://www.w3.org/1999/xhtml">

<p>FORMULA: </p>

<p>CHARGE: 0</p>

</body>

</notes>

</species>

<species id="M_PHV_c" name="Polyhydroxyvalerate" compartment="c" hasOnlySubstanceUnits="false" boundaryCondition="false" constant="false">

<notes>

<body xmlns="http://www.w3.org/1999/xhtml">

<p>FORMULA: </p>

<p>CHARGE: 0</p>

</body>

</notes>

</species>

<species id="M_HY_OCTCoA_c" name="(S)-3-Hydroxyoctanoyl-CoA" compartment="c" hasOnlySubstanceUnits="false" boundaryCondition="false" constant="false">

<notes>

<body xmlns="http://www.w3.org/1999/xhtml">

<p>FORMULA: C29H50N7O18P3S</p>

<p>CHARGE: 0</p>

</body>

</notes>

</species>

<species id="M_PHO_c" name="Polyhydroxyoctanoate" compartment="c" hasOnlySubstanceUnits="false" boundaryCondition="false" constant="false">

<notes>

<body xmlns="http://www.w3.org/1999/xhtml">

<p>FORMULA: </p>

<p>CHARGE: 0</p>

</body>

</notes>

</species>

<species id="M_HY_DECCoA_c" name="(S)-3-Hydroxydecanoyl-CoA" compartment="c" hasOnlySubstanceUnits="false" boundaryCondition="false" constant="false">

<notes>

<body xmlns="http://www.w3.org/1999/xhtml">

<p>FORMULA: C31H54N7O18P3S</p>

<p>CHARGE: 0</p>

</body>

</notes>

</species>

<species id="M_PHD_c" name="Polyhydroxydecanoate" compartment="c" hasOnlySubstanceUnits="false" boundaryCondition="false" constant="false">

<notes>

<body xmlns="http://www.w3.org/1999/xhtml">

<p>FORMULA: </p>

<p>CHARGE: 0</p>

</body>

</notes>

</species>

<species id="M_HY_DODECCoA_c" name="(S)-3-Hydroxydodecanoyl-CoA" compartment="c" hasOnlySubstanceUnits="false" boundaryCondition="false" constant="false">

<notes>

<body xmlns="http://www.w3.org/1999/xhtml">

<p>FORMULA: C33H58N7O18P3S</p>

<p>CHARGE: 0</p>

</body>

</notes>

</species>

<species id="M_PHDD_c" name="Polyhydodroxydecanoate" compartment="c" hasOnlySubstanceUnits="false" boundaryCondition="false" constant="false">

<notes>

<body xmlns="http://www.w3.org/1999/xhtml">

<p>FORMULA: </p>

<p>CHARGE: 0</p>

</body>

</notes>

</species>

<species id="M_GLCN_c" name="D-Gluconate" compartment="c" hasOnlySubstanceUnits="false" boundaryCondition="false" constant="false">

<notes>

<body xmlns="http://www.w3.org/1999/xhtml">

<p>FORMULA: C6H12O7</p>

<p>CHARGE: 0</p>

</body>

</notes>

</species>

<species id="M_GLCN6P_c" name="6-Phospho-D-gluconate" compartment="c" hasOnlySubstanceUnits="false" boundaryCondition="false" constant="false">

<notes>

<body xmlns="http://www.w3.org/1999/xhtml">

<p>FORMULA: C6H13O10P</p>

<p>CHARGE: 0</p>

</body>

</notes>

</species>

<species id="M_DHYGLCN_c" name="5-Dehydro-D-gluconate" compartment="c" hasOnlySubstanceUnits="false" boundaryCondition="false" constant="false">

<notes>

<body xmlns="http://www.w3.org/1999/xhtml">

<p>FORMULA: C6H10O7</p>

<p>CHARGE: 0</p>

</body>

</notes>

</species>

<species id="M_D_GLC_e" name="D-Glucose" compartment="e" hasOnlySubstanceUnits="false" boundaryCondition="false" constant="false">

<notes>

<body xmlns="http://www.w3.org/1999/xhtml">

<p>FORMULA: C6H12O6</p>

<p>CHARGE: 0</p>

</body>

</notes>

</species>

<species id="M_H2O_e" name="Water" compartment="e" hasOnlySubstanceUnits="false" boundaryCondition="false" constant="false">

<notes>

<body xmlns="http://www.w3.org/1999/xhtml">

<p>FORMULA: H2O</p>

<p>CHARGE: 0</p>

</body>

</notes>

</species>

<species id="M_O2_e" name="Oxygen" compartment="e" hasOnlySubstanceUnits="false" boundaryCondition="false" constant="false">

<notes>

```
&lt;body xmlns="http://www.w3.org/1999/xhtml"&gt;
<p>FORMULA: O2</p>
<p>CHARGE: 0</p>
&lt;/body&gt;
&lt;/notes&gt;
&lt;/species&gt;
&lt;species id="M_SO4_e" name="Sulfate" compartment="e" hasOnlySubstanceUnits="false" boundaryCondition="false" constant="false"&gt;
&lt;notes&gt;
&lt;body xmlns="http://www.w3.org/1999/xhtml"&gt;
<p>FORMULA: H2SO4</p>
<p>CHARGE: 0</p>
&lt;/body&gt;
&lt;/notes&gt;
&lt;/species&gt;
&lt;species id="M_Pi_e" name="Orthophosphate" compartment="e" hasOnlySubstanceUnits="false" boundaryCondition="false" constant="false"&gt;
&lt;notes&gt;
&lt;body xmlns="http://www.w3.org/1999/xhtml"&gt;
<p>FORMULA: H3PO4</p>
<p>CHARGE: 0</p>
&lt;/body&gt;
&lt;/notes&gt;
&lt;/species&gt;
&lt;species id="M_CO2_e" name="Carbon dioxide" compartment="e" hasOnlySubstanceUnits="false" boundaryCondition="false" constant="false"&gt;
&lt;notes&gt;
&lt;body xmlns="http://www.w3.org/1999/xhtml"&gt;
<p>FORMULA: CO2</p>
<p>CHARGE: 0</p>
```

</body>

</notes>

</species>

<species id="M_AC_e" name="Acetate" compartment="e" hasOnlySubstanceUnits="false" boundaryCondition="false" constant="false">

<notes>

<body xmlns="http://www.w3.org/1999/xhtml">

<p>FORMULA: C2H4O2</p>

<p>CHARGE: 0</p>

</body>

</notes>

</species>

<species id="M_D_ALA_D_ALA_e" name="D-Alanyl-D-alanine" compartment="e" hasOnlySubstanceUnits="false" boundaryCondition="false" constant="false">

<notes>

<body xmlns="http://www.w3.org/1999/xhtml">

<p>FORMULA: C6H12N2O3</p>

<p>CHARGE: 0</p>

</body>

</notes>

</species>

<species id="M_L_ARG_e" name="L-Arginine" compartment="e" hasOnlySubstanceUnits="false" boundaryCondition="false" constant="false">

<notes>

<body xmlns="http://www.w3.org/1999/xhtml">

<p>FORMULA: C6H14N4O2</p>

<p>CHARGE: 0</p>

</body>

</notes>

</species>

<species id="M_L_ASN_e" name="L-Asparagine" compartment="e" hasOnlySubstanceUnits="false" boundaryCondition="false" constant="false">

<notes>

<body xmlns="http://www.w3.org/1999/xhtml">

<p>FORMULA: C4H8N2O3</p>

<p>CHARGE: 0</p>

</body>

</notes>

</species>

<species id="M_CIT_e" name="Citrate" compartment="e" hasOnlySubstanceUnits="false" boundaryCondition="false" constant="false">

<notes>

<body xmlns="http://www.w3.org/1999/xhtml">

<p>FORMULA: C6H8O7</p>

<p>CHARGE: 0</p>

</body>

</notes>

</species>

<species id="M_ETH_e" name="Ethanol" compartment="e" hasOnlySubstanceUnits="false" boundaryCondition="false" constant="false">

<notes>

<body xmlns="http://www.w3.org/1999/xhtml">

<p>FORMULA: C2H6O</p>

<p>CHARGE: 0</p>

</body>

</notes>

</species>

<species id="M_F1P_e" name="D-Fructose 1-phosphate" compartment="e" hasOnlySubstanceUnits="false" boundaryCondition="false" constant="false">

<notes>

<body xmlns="http://www.w3.org/1999/xhtml">

<p>FORMULA: C6H13O9P</p>

<p>CHARGE: 0</p>

</body>

</notes>

</species>

<species id="M_FUCL1P_e" name="L-Fuculose 1-phosphate" compartment="e" hasOnlySubstanceUnits="false" boundaryCondition="false" constant="false">

<notes>

<body xmlns="http://www.w3.org/1999/xhtml">

<p>FORMULA: C6H13O8P</p>

<p>CHARGE: 0</p>

</body>

</notes>

</species>

<species id="M_Fe2_e" name="Ferrous ion" compartment="e" hasOnlySubstanceUnits="false" boundaryCondition="false" constant="false">

<notes>

<body xmlns="http://www.w3.org/1999/xhtml">

<p>FORMULA: Fe</p>

<p>CHARGE: 0</p>

</body>

</notes>

</species>

<species id="M_D_FRU_e" name="D-Fructose" compartment="e" hasOnlySubstanceUnits="false" boundaryCondition="false" constant="false">

<notes>

<body xmlns="http://www.w3.org/1999/xhtml">

<p>FORMULA: C6H12O6</p>

<p>CHARGE: 0</p>

</body>

</notes>

</species>

<species id="M_FUM_e" name="Fumarate" compartment="e" hasOnlySubstanceUnits="false" boundaryCondition="false" constant="false">

<notes>

<body xmlns="http://www.w3.org/1999/xhtml">

<p>FORMULA: C4H4O4</p>

<p>CHARGE: 0</p>

</body>

</notes>

</species>

<species id="M_GLUCSA1P_e" name="alpha-D-Glucosamine 1-phosphate" compartment="e" hasOnlySubstanceUnits="false" boundaryCondition="false" constant="false">

<notes>

<body xmlns="http://www.w3.org/1999/xhtml">

<p>FORMULA: C6H14NO8P</p>

<p>CHARGE: 0</p>

</body>

</notes>

</species>

<species id="M_GLUSAP_e" name="D-Glucosamine 6-phosphate" compartment="e" hasOnlySubstanceUnits="false" boundaryCondition="false" constant="false">

<notes>

<body xmlns="http://www.w3.org/1999/xhtml">

<p>FORMULA: C6H14NO8P</p>

<p>CHARGE: 0</p>

</body>

</notes>

</species>

```
<species id="M_GLYCALD_e" name="Glycolaldehyde" compartment="e" hasOnlySubstanceUnits="false" boundaryCondition="false" constant="false">
  <notes>
    <body xmlns="http://www.w3.org/1999/xhtml">
      <p>FORMULA: C2H4O2</p>
      <p>CHARGE: 0</p>
    </body>
  </notes>
</species>
<species id="M_D_GLU_e" name="D-Glutamate" compartment="e" hasOnlySubstanceUnits="false" boundaryCondition="false" constant="false">
  <notes>
    <body xmlns="http://www.w3.org/1999/xhtml">
      <p>FORMULA: C5H9NO4</p>
      <p>CHARGE: 0</p>
    </body>
  </notes>
</species>
<species id="M_HY_AC_e" name="Glycolate" compartment="e" hasOnlySubstanceUnits="false" boundaryCondition="false" constant="false">
  <notes>
    <body xmlns="http://www.w3.org/1999/xhtml">
      <p>FORMULA: C2H4O3</p>
      <p>CHARGE: 0</p>
    </body>
  </notes>
</species>
<species id="M_D_GLCT_e" name="D-Glycerate" compartment="e" hasOnlySubstanceUnits="false" boundaryCondition="false" constant="false">
  <notes>
```

<body xmlns="http://www.w3.org/1999/xhtml">
<p>FORMULA: C3H6O4</p>
<p>CHARGE: 0</p>
</body>
</notes>
</species>
<species id="M_GLYCRL_e" name="Glycerol" compartment="e" hasOnlySubstanceUnits="false" boundaryCondition="false" constant="false">
<notes>
<body xmlns="http://www.w3.org/1999/xhtml">
<p>FORMULA: C3H8O3</p>
<p>CHARGE: 0</p>
</body>
</notes>
</species>
<species id="M_L_GLY_e" name="Glycine" compartment="e" hasOnlySubstanceUnits="false" boundaryCondition="false" constant="false">
<notes>
<body xmlns="http://www.w3.org/1999/xhtml">
<p>FORMULA: C2H5NO2</p>
<p>CHARGE: 0</p>
</body>
</notes>
</species>
<species id="M_H2S_e" name="Hydrogen sulfide" compartment="e" hasOnlySubstanceUnits="false" boundaryCondition="false" constant="false">
<notes>
<body xmlns="http://www.w3.org/1999/xhtml">
<p>FORMULA: H2S</p>
<p>CHARGE: 0</p>

</body>

</notes>

</species>

<species id="M_L_HCYS_e" name="L-Homocysteine" compartment="e" hasOnlySubstanceUnits="false" boundaryCondition="false" constant="false">

<notes>

<body xmlns="http://www.w3.org/1999/xhtml">

<p>FORMULA: C4H9NO2S</p>

<p>CHARGE: 0</p>

</body>

</notes>

</species>

<species id="M_L_ILE_e" name="L-Isoleucine" compartment="e" hasOnlySubstanceUnits="false" boundaryCondition="false" constant="false">

<notes>

<body xmlns="http://www.w3.org/1999/xhtml">

<p>FORMULA: C6H13NO2</p>

<p>CHARGE: 0</p>

</body>

</notes>

</species>

<species id="M_L_LYS_e" name="L-Lysine" compartment="e" hasOnlySubstanceUnits="false" boundaryCondition="false" constant="false">

<notes>

<body xmlns="http://www.w3.org/1999/xhtml">

<p>FORMULA: C6H14N2O2</p>

<p>CHARGE: 0</p>

</body>

</notes>

</species>

<species id="M_MAL_e" name="L-Malate" compartment="e" hasOnlySubstanceUnits="false" boundaryCondition="false" constant="false">

<notes>

<body xmlns="http://www.w3.org/1999/xhtml">

<p>FORMULA: C4H6O5</p>

<p>CHARGE: 0</p>

</body>

</notes>

</species>

<species id="M_MALT_e" name="Maltose" compartment="e" hasOnlySubstanceUnits="false" boundaryCondition="false" constant="false">

<notes>

<body xmlns="http://www.w3.org/1999/xhtml">

<p>FORMULA: C12H22O11</p>

<p>CHARGE: 0</p>

</body>

</notes>

</species>

<species id="M_IMALT_e" name="Isomaltose" compartment="e" hasOnlySubstanceUnits="false" boundaryCondition="false" constant="false">

<notes>

<body xmlns="http://www.w3.org/1999/xhtml">

<p>FORMULA: </p>

<p>CHARGE: 0</p>

</body>

</notes>

</species>

<species id="M_MAN1P_e" name="D-Mannose 1-phosphate" compartment="e" hasOnlySubstanceUnits="false" boundaryCondition="false" constant="false">

<notes>

```
        <body xmlns="http://www.w3.org/1999/xhtml">
          <p>FORMULA: C6H13O9P</p>
          <p>CHARGE: 0</p>
        </body>
      </notes>
    </species>
    <species id="M_M6P_e" name="D-Mannose 6-phosphate" compartment="e" hasOnlySubstanceUnits="false"
boundaryCondition="false" constant="false">
      <notes>
        <body xmlns="http://www.w3.org/1999/xhtml">
          <p>FORMULA: C6H13O9P</p>
          <p>CHARGE: 0</p>
        </body>
      </notes>
    </species>
    <species id="M_D_MAN_e" name="D-Mannose" compartment="e" hasOnlySubstanceUnits="false"
boundaryCondition="false" constant="false">
      <notes>
        <body xmlns="http://www.w3.org/1999/xhtml">
          <p>FORMULA: C6H12O6</p>
          <p>CHARGE: 0</p>
        </body>
      </notes>
    </species>
    <species id="M_L_PHE_e" name="L-Phenylalanine" compartment="e" hasOnlySubstanceUnits="false"
boundaryCondition="false" constant="false">
      <notes>
        <body xmlns="http://www.w3.org/1999/xhtml">
          <p>FORMULA: C9H11NO2</p>
          <p>CHARGE: 0</p>
```

</body>

</notes>

</species>

<species id="M_L_PRO_e" name="L-Proline" compartment="e" hasOnlySubstanceUnits="false" boundaryCondition="false" constant="false">

<notes>

<body xmlns="http://www.w3.org/1999/xhtml">

<p>FORMULA: C5H9NO2</p>

<p>CHARGE: 0</p>

</body>

</notes>

</species>

<species id="M_D_RIB_e" name="D-Ribose" compartment="e" hasOnlySubstanceUnits="false" boundaryCondition="false" constant="false">

<notes>

<body xmlns="http://www.w3.org/1999/xhtml">

<p>FORMULA: C5H10O5</p>

<p>CHARGE: 0</p>

</body>

</notes>

</species>

<species id="M_SORB_e" name="D-Sorbitol" compartment="e" hasOnlySubstanceUnits="false" boundaryCondition="false" constant="false">

<notes>

<body xmlns="http://www.w3.org/1999/xhtml">

<p>FORMULA: C6H14O6</p>

<p>CHARGE: 0</p>

</body>

</notes>

</species>

<species id="M_L_SER_e" name="L-Serine" compartment="e" hasOnlySubstanceUnits="false" boundaryCondition="false" constant="false">

<notes>

<body xmlns="http://www.w3.org/1999/xhtml">

<p>FORMULA: C3H7NO3</p>

<p>CHARGE: 0</p>

</body>

</notes>

</species>

<species id="M_SO3_e" name="Sulfite" compartment="e" hasOnlySubstanceUnits="false" boundaryCondition="false" constant="false">

<notes>

<body xmlns="http://www.w3.org/1999/xhtml">

<p>FORMULA: H2SO3</p>

<p>CHARGE: 0</p>

</body>

</notes>

</species>

<species id="M_SUCC_e" name="Succinate" compartment="e" hasOnlySubstanceUnits="false" boundaryCondition="false" constant="false">

<notes>

<body xmlns="http://www.w3.org/1999/xhtml">

<p>FORMULA: C4H6O4</p>

<p>CHARGE: 0</p>

</body>

</notes>

</species>

<species id="M_SUC_e" name="Sucrose" compartment="e" hasOnlySubstanceUnits="false" boundaryCondition="false" constant="false">

<notes>

<body xmlns="http://www.w3.org/1999/xhtml">

<p>FORMULA: C12H22O11</p>

<p>CHARGE: 0</p>

</body>

</notes>

</species>

<species id="M_L_ALA_e" name="L-Alanine" compartment="e" hasOnlySubstanceUnits="false" boundaryCondition="false" constant="false">

<notes>

<body xmlns="http://www.w3.org/1999/xhtml">

<p>FORMULA: C3H7NO2</p>

<p>CHARGE: 0</p>

</body>

</notes>

</species>

<species id="M_L_ASP_e" name="L-Aspartate" compartment="e" hasOnlySubstanceUnits="false" boundaryCondition="false" constant="false">

<notes>

<body xmlns="http://www.w3.org/1999/xhtml">

<p>FORMULA: C4H7NO4</p>

<p>CHARGE: 0</p>

</body>

</notes>

</species>

<species id="M_L_CYS_e" name="L-Cysteine" compartment="e" hasOnlySubstanceUnits="false" boundaryCondition="false" constant="false">

<notes>

<body xmlns="http://www.w3.org/1999/xhtml">

<p>FORMULA: C3H7NO2S</p>

<p>CHARGE: 0</p>

</body>

</notes>

</species>

<species id="M_L_GLU_e" name="L-Glutamate" compartment="e" hasOnlySubstanceUnits="false" boundaryCondition="false" constant="false">

<notes>

<body xmlns="http://www.w3.org/1999/xhtml">

<p>FORMULA: C5H9NO4</p>

<p>CHARGE: 0</p>

</body>

</notes>

</species>

<species id="M_L_MET_e" name="L-Methionine" compartment="e" hasOnlySubstanceUnits="false" boundaryCondition="false" constant="false">

<notes>

<body xmlns="http://www.w3.org/1999/xhtml">

<p>FORMULA: C5H11NO2S</p>

<p>CHARGE: 0</p>

</body>

</notes>

</species>

<species id="M_L_THR_e" name="L-Threonine" compartment="e" hasOnlySubstanceUnits="false" boundaryCondition="false" constant="false">

<notes>

<body xmlns="http://www.w3.org/1999/xhtml">

<p>FORMULA: C4H9NO3</p>

<p>CHARGE: 0</p>

</body>

</notes>

</species>

<species id="M_L_LEU_e" name="L-Leucine" compartment="e" hasOnlySubstanceUnits="false" boundaryCondition="false" constant="false">

<notes>

<body xmlns="http://www.w3.org/1999/xhtml">

<p>FORMULA: C6H13NO2</p>

<p>CHARGE: 0</p>

</body>

</notes>

</species>

<species id="M_L_TRP_e" name="L-Tryptophan" compartment="e" hasOnlySubstanceUnits="false" boundaryCondition="false" constant="false">

<notes>

<body xmlns="http://www.w3.org/1999/xhtml">

<p>FORMULA: C11H12N2O2</p>

<p>CHARGE: 0</p>

</body>

</notes>

</species>

<species id="M_L_TYR_e" name="L-Tyrosine" compartment="e" hasOnlySubstanceUnits="false" boundaryCondition="false" constant="false">

<notes>

<body xmlns="http://www.w3.org/1999/xhtml">

<p>FORMULA: C9H11NO3</p>

<p>CHARGE: 0</p>

</body>

</notes>

</species>

<species id="M_L_VAL_e" name="L-Valine" compartment="e" hasOnlySubstanceUnits="false" boundaryCondition="false" constant="false">

<notes>

```
        <body xmlns="http://www.w3.org/1999/xhtml">
          <p>FORMULA: C5H11NO2</p>
          <p>CHARGE: 0</p>
        </body>
      </notes>
    </species>
    <species id="M_HCO3_e" name="Bicarbonate" compartment="e" hasOnlySubstanceUnits="false" boundaryCondition="false" constant="false">
      <notes>
        <body xmlns="http://www.w3.org/1999/xhtml">
          <p>FORMULA: HCO3</p>
          <p>CHARGE: 0</p>
        </body>
      </notes>
    </species>
    <species id="M_LAC_e" name="L-Lactate" compartment="e" hasOnlySubstanceUnits="false" boundaryCondition="false" constant="false">
      <notes>
        <body xmlns="http://www.w3.org/1999/xhtml">
          <p>FORMULA: C3H6O3</p>
          <p>CHARGE: 0</p>
        </body>
      </notes>
    </species>
    <species id="M_NH4_e" name="Ammonia" compartment="e" hasOnlySubstanceUnits="false" boundaryCondition="false" constant="false">
      <notes>
        <body xmlns="http://www.w3.org/1999/xhtml">
          <p>FORMULA: NH4</p>
          <p>CHARGE: 0</p>
```

</body>

</notes>

</species>

<species id="M_TRE_e" name="Trehalose" compartment="e" hasOnlySubstanceUnits="false" boundaryCondition="false" constant="false">

<notes>

<body xmlns="http://www.w3.org/1999/xhtml">

<p>FORMULA: C12H22O11</p>

<p>CHARGE: 0</p>

</body>

</notes>

</species>

<species id="M_Mg_e" name="Magnesium" compartment="e" hasOnlySubstanceUnits="false" boundaryCondition="false" constant="false">

<notes>

<body xmlns="http://www.w3.org/1999/xhtml">

<p>FORMULA: Mg</p>

<p>CHARGE: 0</p>

</body>

</notes>

</species>

<species id="M_HEME_e" name="Heme" compartment="e" hasOnlySubstanceUnits="false" boundaryCondition="false" constant="false">

<notes>

<body xmlns="http://www.w3.org/1999/xhtml">

<p>FORMULA: C34H32FeN4O4</p>

<p>CHARGE: 0</p>

</body>

</notes>

</species>

<species id="M_GLCN_e" name="D-Gluconate" compartment="e" hasOnlySubstanceUnits="false" boundaryCondition="false" constant="false">

<notes>

<body xmlns="http://www.w3.org/1999/xhtml">

<p>FORMULA: C6H12O7</p>

<p>CHARGE: 0</p>

</body>

</notes>

</species>

<species id="M_CELBS_e" name="Cellobiose" compartment="e" hasOnlySubstanceUnits="false" boundaryCondition="false" constant="false">

<notes>

<body xmlns="http://www.w3.org/1999/xhtml">

<p>FORMULA: C12H22O11</p>

<p>CHARGE: 0</p>

</body>

</notes>

</species>

<species id="M_PALT_e" name="Paltinose" compartment="e" hasOnlySubstanceUnits="false" boundaryCondition="false" constant="false">

<notes>

<body xmlns="http://www.w3.org/1999/xhtml">

<p>FORMULA: </p>

<p>CHARGE: 0</p>

</body>

</notes>

</species>

<species id="M_D_GAL_e" name="D-Galactose" compartment="e" hasOnlySubstanceUnits="false" boundaryCondition="false" constant="false">

<notes>

<body xmlns="http://www.w3.org/1999/xhtml">

<p>FORMULA: C6H12O6</p>

<p>CHARGE: 0</p>

</body>

</notes>

</species>

<species id="M_LACTS_e" name="Lactose" compartment="e" hasOnlySubstanceUnits="false" boundaryCondition="false" constant="false">

<notes>

<body xmlns="http://www.w3.org/1999/xhtml">

<p>FORMULA: </p>

<p>CHARGE: 0</p>

</body>

</notes>

</species>

<species id="M_STCYS_e" name="Stachyose" compartment="e" hasOnlySubstanceUnits="false" boundaryCondition="false" constant="false">

<notes>

<body xmlns="http://www.w3.org/1999/xhtml">

<p>FORMULA: </p>

<p>CHARGE: 0</p>

</body>

</notes>

</species>

<species id="M_PHB_e" name="polyhydroxybutyrate" compartment="e" hasOnlySubstanceUnits="false" boundaryCondition="false" constant="false">

<notes>

<body xmlns="http://www.w3.org/1999/xhtml">

<p>FORMULA: </p>

<p>CHARGE: 0</p>

</body>

</notes>

</species>

<species id="M_PHV_e" name="Polyhydroxyvalerate" compartment="e" hasOnlySubstanceUnits="false" boundaryCondition="false" constant="false">

<notes>

<body xmlns="http://www.w3.org/1999/xhtml">

<p>FORMULA: </p>

<p>CHARGE: 0</p>

</body>

</notes>

</species>

<species id="M_PHO_e" name="Polyhydroxyoctanoate" compartment="e" hasOnlySubstanceUnits="false" boundaryCondition="false" constant="false">

<notes>

<body xmlns="http://www.w3.org/1999/xhtml">

<p>FORMULA: </p>

<p>CHARGE: 0</p>

</body>

</notes>

</species>

<species id="M_PHD_e" name="Polyhydroxydecanoate" compartment="e" hasOnlySubstanceUnits="false" boundaryCondition="false" constant="false">

<notes>

<body xmlns="http://www.w3.org/1999/xhtml">

<p>FORMULA: </p>

<p>CHARGE: 0</p>

</body>

</notes>

</species>

<species id="M_PHDD_e" name="Polyhydodroxydecanoate" compartment="e" hasOnlySubstanceUnits="false" boundaryCondition="false" constant="false">

<notes>

<body xmlns="http://www.w3.org/1999/xhtml">

<p>FORMULA: </p>

<p>CHARGE: 0</p>

</body>

</notes>

</species>

</listOfSpecies>

<listOfReactions>

<reaction id="R_R001" name="Biomass reaction (70 C)" reversible="false">

<notes>

<body xmlns="http://www.w3.org/1999/xhtml">

<p>GENE_ASSOCIATION: </p>

<p>SUBSYSTEM: Exchange Reactions</p>

<p>EC Number: </p>

<p>Confidence Level: 1</p>

<p>AUTHORS: </p>

<p/>

</body>

</notes>

<listOfReactants>

<speciesReference species="M_L_ASP_c" stoichiometry="0.1582"/>

<speciesReference species="M_L_GLU_c" stoichiometry="0.2696"/>

<speciesReference species="M_L_SER_c" stoichiometry="0.2076"/>

<speciesReference species="M_L_HIS_c" stoichiometry="0.0375"/>

<speciesReference species="M_L_GLY_c" stoichiometry="0.4716"/>

<speciesReference species="M_L_THR_c" stoichiometry="0.1531"/>

<speciesReference species="M_L_ARG_c" stoichiometry="0.3231"/>

<speciesReference species="M_L_ALA_c" stoichiometry="0.5434"/>

<speciesReference species="M_L_TYR_c" stoichiometry="0.1052"/>

<speciesReference species="M_L_VAL_c" stoichiometry="0.1668"/>

<speciesReference species="M_L_PHE_c" stoichiometry="0.1322"/>

<speciesReference species="M_L_ILE_c" stoichiometry="0.0593"/>

<speciesReference species="M_L_LEU_c" stoichiometry="0.3744"/>

<speciesReference species="M_L_LYS_c" stoichiometry="0.1337"/>

<speciesReference species="M_L_PRO_c" stoichiometry="0.2898"/>

<speciesReference species="M_L_ASN_c" stoichiometry="0.1582"/>

<speciesReference species="M_L_CYS_c" stoichiometry="0.0134"/>

<speciesReference species="M_L_GLN_c" stoichiometry="0.2714"/>

<speciesReference species="M_L_MET_c" stoichiometry="0.0224"/>

<speciesReference species="M_L_TRP_c" stoichiometry="0.0654"/>

<speciesReference species="M_UDP_ACMURM_c" stoichiometry="0.011"/>

<speciesReference species="M_UDP_AGLUAM_c" stoichiometry="0.011"/>

<speciesReference species="M_D_GLU_c" stoichiometry="0.011"/>

<speciesReference species="M_ORT_c" stoichiometry="0.011"/>

<speciesReference species="M_D_ALA_c" stoichiometry="0.0259"/>

<speciesReference species="M_dTTP_c" stoichiometry="0.0098"/>

<speciesReference species="M_dGTP_c" stoichiometry="0.0223"/>

<speciesReference species="M_dCTP_c" stoichiometry="0.0223"/>

<speciesReference species="M_dATP_c" stoichiometry="0.0098"/>

<speciesReference species="M_CTP_c" stoichiometry="0.0586"/>

<speciesReference species="M_GTP_c" stoichiometry="0.1455"/>

<speciesReference species="M_UTP_c" stoichiometry="0.0976"/>

<speciesReference species="M_MGDG_c" stoichiometry="0.01095"/>

<speciesReference species="M_DGDG_c" stoichiometry="0.0091"/>

<speciesReference species="M_tZXT_c" stoichiometry="0.1113"/>

```
    <speciesReference species="M_PETHA_c" stoichiometry="0.0031"/>
    <speciesReference species="M_PGLY_c" stoichiometry="0.0022"/>
    <speciesReference species="M_PINS_c" stoichiometry="0.0066"/>
    <speciesReference species="M_CDLPN_c" stoichiometry="0.0012"/>
    <speciesReference species="M_PX_c" stoichiometry="0.0404"/>
    <speciesReference species="M_ATP_c" stoichiometry="59.81"/>
  </listOfReactants>
  <listOfProducts>
    <speciesReference species="M_ADP_c" stoichiometry="59.81"/>
    <speciesReference species="M_Pi_c" stoichiometry="59.81"/>
    <speciesReference species="M_PPi_c" stoichiometry="0.3659"/>
  </listOfProducts>
  <kineticLaw>
    <math xmlns="http://www.w3.org/1998/Math/MathML">
      <ci> FLUX_VALUE </ci>
    </math>
    <listOfParameters>
      <parameter id="LOWER_BOUND" value="0" units="mmol_per_gDW_per_hr" constant="true"/>
      <parameter id="UPPER_BOUND" value="1000" units="mmol_per_gDW_per_hr" constant="true"/>
      <parameter id="FLUX_VALUE" value="0" units="mmol_per_gDW_per_hr" constant="true"/>
      <parameter id="OBJECTIVE_COEFFICIENT" value="1" units="mmol_per_gDW_per_hr" constant="true"/>
    </listOfParameters>
  </kineticLaw>
</reaction>
<reaction id="R_R002" name="ATP Maintenance" reversible="false">
  <notes>
    <body xmlns="http://www.w3.org/1999/xhtml">
      <p>GENE_ASSOCIATION: </p>
      <p>SUBSYSTEM: Uassigned</p>
```

```
      <p>EC Number: </p>
      <p>Confidence Level: 1</p>
      <p>AUTHORS: </p>
      <p/>
    </body>
  </notes>
  <listOfReactants>
    <speciesReference species="M_ATP_c" stoichiometry="1"/>
    <speciesReference species="M_H2O_c" stoichiometry="1"/>
  </listOfReactants>
  <listOfProducts>
    <speciesReference species="M_ADP_c" stoichiometry="1"/>
    <speciesReference species="M_Pi_c" stoichiometry="1"/>
    <speciesReference species="M_H_c" stoichiometry="1"/>
  </listOfProducts>
  <kineticLaw>
    <math xmlns="http://www.w3.org/1998/Math/MathML">
      <ci> FLUX_VALUE </ci>
    </math>
    <listOfParameters>
      <parameter id="LOWER_BOUND" value="14.29" units="mmol_per_gDW_per_hr" constant="true"/>
      <parameter id="UPPER_BOUND" value="1000" units="mmol_per_gDW_per_hr" constant="true"/>
      <parameter id="FLUX_VALUE" value="0" units="mmol_per_gDW_per_hr" constant="true"/>
      <parameter id="OBJECTIVE_COEFFICIENT" value="0" units="mmol_per_gDW_per_hr" constant="true"/>
    </listOfParameters>
  </kineticLaw>
</reaction>
<reaction id="R_R003" name="ATP:D-glucose 6-phosphotransferase" reversible="false">
  <notes>
```

```
  <body xmlns="http://www.w3.org/1999/xhtml">
    <p>GENE_ASSOCIATION: TTC1688</p>
    <p>SUBSYSTEM: Glycolysis/Gluconeogenesis</p>
    <p>EC Number: 2.7.1.2</p>
    <p>Confidence Level: 1</p>
    <p>AUTHORS: Carbohydrate metabolism</p>
    <p/>
  </body>
</notes>
<listOfReactants>
  <speciesReference species="M_ATP_c" stoichiometry="1"/>
  <speciesReference species="M_D_GLC_c" stoichiometry="1"/>
</listOfReactants>
<listOfProducts>
  <speciesReference species="M_ADP_c" stoichiometry="1"/>
  <speciesReference species="M_H_c" stoichiometry="1"/>
  <speciesReference species="M_G6P_c" stoichiometry="1"/>
</listOfProducts>
<kineticLaw>
  <math xmlns="http://www.w3.org/1998/Math/MathML">
    <ci> FLUX_VALUE </ci>
  </math>
  <listOfParameters>
    <parameter id="LOWER_BOUND" value="0" units="mmol_per_gDW_per_hr" constant="true"/>
    <parameter id="UPPER_BOUND" value="1000" units="mmol_per_gDW_per_hr" constant="true"/>
    <parameter id="FLUX_VALUE" value="0" units="mmol_per_gDW_per_hr" constant="true"/>
    <parameter id="OBJECTIVE_COEFFICIENT" value="0" units="mmol_per_gDW_per_hr" constant="true"/>
  </listOfParameters>
</kineticLaw>
```

```
</reaction>
<reaction id="R_R004" name="ATP:D-glucose 6-phosphotransferase" reversible="false">
 <notes>
  <body xmlns="http://www.w3.org/1999/xhtml">
   <p>GENE_ASSOCIATION: TTC1688</p>
   <p>SUBSYSTEM: Glycolysis/Gluconeogenesis</p>
   <p>EC Number: 2.7.1.2</p>
   <p>Confidence Level: 1</p>
   <p>AUTHORS: Carbohydrate metabolism</p>
   <p/>
  </body>
 </notes>
 <listOfReactants>
  <speciesReference species="M_ATP_c" stoichiometry="1"/>
  <speciesReference species="M_D_MAN_c" stoichiometry="1"/>
 </listOfReactants>
 <listOfProducts>
  <speciesReference species="M_ADP_c" stoichiometry="1"/>
  <speciesReference species="M_H_c" stoichiometry="1"/>
  <speciesReference species="M_M6P_c" stoichiometry="1"/>
 </listOfProducts>
 <kineticLaw>
  <math xmlns="http://www.w3.org/1998/Math/MathML">
   <ci> FLUX_VALUE </ci>
  </math>
  <listOfParameters>
   <parameter id="LOWER_BOUND" value="0" units="mmol_per_gDW_per_hr" constant="true"/>
   <parameter id="UPPER_BOUND" value="1000" units="mmol_per_gDW_per_hr" constant="true"/>
   <parameter id="FLUX_VALUE" value="0" units="mmol_per_gDW_per_hr" constant="true"/>
```

```
      <parameter id="OBJECTIVE_COEFFICIENT" value="0" units="mmol_per_gDW_per_hr" constant="true"/>
    </listOfParameters>
  </kineticLaw>
</reaction>
<reaction id="R_R005" name="D-glucose-6-phosphate aldose-ketose-isomerase" reversible="true">
  <notes>
    <body xmlns="http://www.w3.org/1999/xhtml">
      <p>GENE_ASSOCIATION: TTC1710</p>
      <p>SUBSYSTEM: Glycolysis/Gluconeogenesis</p>
      <p>EC Number: 5.3.1.9</p>
      <p>Confidence Level: 1</p>
      <p>AUTHORS: Carbohydrate metabolism</p>
      <p/>
    </body>
  </notes>
  <listOfReactants>
    <speciesReference species="M_G6P_c" stoichiometry="1"/>
  </listOfReactants>
  <listOfProducts>
    <speciesReference species="M_F6P_c" stoichiometry="1"/>
  </listOfProducts>
  <kineticLaw>
    <math xmlns="http://www.w3.org/1998/Math/MathML">
      <ci> FLUX_VALUE </ci>
    </math>
    <listOfParameters>
      <parameter id="LOWER_BOUND" value="-1000" units="mmol_per_gDW_per_hr" constant="true"/>
      <parameter id="UPPER_BOUND" value="1000" units="mmol_per_gDW_per_hr" constant="true"/>
      <parameter id="FLUX_VALUE" value="0" units="mmol_per_gDW_per_hr" constant="true"/>
```

```xml
      <parameter id="OBJECTIVE_COEFFICIENT" value="0" units="mmol_per_gDW_per_hr" constant="true"/>
    </listOfParameters>
  </kineticLaw>
</reaction>
<reaction id="R_R006" name="ATP:D-fructose-6-phosphate 1-phosphotransferase" reversible="false">
  <notes>
    <body xmlns="http://www.w3.org/1999/xhtml">
      <p>GENE_ASSOCIATION: TTC1597</p>
      <p>SUBSYSTEM: Glycolysis/Gluconeogenesis</p>
      <p>EC Number: 2.7.1.11</p>
      <p>Confidence Level: 1</p>
      <p>AUTHORS: Carbohydrate metabolism</p>
      <p/>
    </body>
  </notes>
  <listOfReactants>
    <speciesReference species="M_ATP_c" stoichiometry="1"/>
    <speciesReference species="M_F6P_c" stoichiometry="1"/>
  </listOfReactants>
  <listOfProducts>
    <speciesReference species="M_ADP_c" stoichiometry="1"/>
    <speciesReference species="M_H_c" stoichiometry="1"/>
    <speciesReference species="M_FBP_c" stoichiometry="1"/>
  </listOfProducts>
  <kineticLaw>
    <math xmlns="http://www.w3.org/1998/Math/MathML">
      <ci> FLUX_VALUE </ci>
    </math>
    <listOfParameters>
```

```xml
<parameter id="LOWER_BOUND" value="0" units="mmol_per_gDW_per_hr" constant="true"/>

<parameter id="UPPER_BOUND" value="1000" units="mmol_per_gDW_per_hr" constant="true"/>

<parameter id="FLUX_VALUE" value="0" units="mmol_per_gDW_per_hr" constant="true"/>

<parameter id="OBJECTIVE_COEFFICIENT" value="0" units="mmol_per_gDW_per_hr" constant="true"/>

</listOfParameters>

</kineticLaw>

</reaction>

<reaction id="R_R007" name="D-fructose-1,6-bisphosphate D-glyceraldehyde-3-phosphate-lyase (glycerone-phosphate-forming)" reversible="true">

<notes>

<body xmlns="http://www.w3.org/1999/xhtml">

<p>GENE_ASSOCIATION: TTC1414</p>

<p>SUBSYSTEM: Glycolysis/Gluconeogenesis</p>

<p>EC Number: 4.1.2.13</p>

<p>Confidence Level: 1</p>

<p>AUTHORS: Carbohydrate metabolism</p>

<p/>

</body>

</notes>

<listOfReactants>

<speciesReference species="M_FBP_c" stoichiometry="1"/>

</listOfReactants>

<listOfProducts>

<speciesReference species="M_DHAP_c" stoichiometry="1"/>

<speciesReference species="M_GAP_c" stoichiometry="1"/>

</listOfProducts>

<kineticLaw>

<math xmlns="http://www.w3.org/1998/Math/MathML">

<ci> FLUX_VALUE </ci>
```

```
    </math>
    <listOfParameters>
      <parameter id="LOWER_BOUND" value="-1000" units="mmol_per_gDW_per_hr" constant="true"/>
      <parameter id="UPPER_BOUND" value="1000" units="mmol_per_gDW_per_hr" constant="true"/>
      <parameter id="FLUX_VALUE" value="0" units="mmol_per_gDW_per_hr" constant="true"/>
      <parameter id="OBJECTIVE_COEFFICIENT" value="0" units="mmol_per_gDW_per_hr" constant="true"/>
    </listOfParameters>
  </kineticLaw>
</reaction>
<reaction id="R_R008" name="D-glyceraldehyde-3-phosphate aldose-ketose-isomerase" reversible="true">
  <notes>
    <body xmlns="http://www.w3.org/1999/xhtml">
      <p>GENE_ASSOCIATION: TTC0581</p>
      <p>SUBSYSTEM: Glycolysis/Gluconeogenesis</p>
      <p>EC Number: 5.3.1.1</p>
      <p>Confidence Level: 1</p>
      <p>AUTHORS: Carbohydrate metabolism</p>
      <p/>
    </body>
  </notes>
  <listOfReactants>
    <speciesReference species="M_GAP_c" stoichiometry="1"/>
  </listOfReactants>
  <listOfProducts>
    <speciesReference species="M_DHAP_c" stoichiometry="1"/>
  </listOfProducts>
  <kineticLaw>
    <math xmlns="http://www.w3.org/1998/Math/MathML">
      <ci> FLUX_VALUE </ci>
```

```
</math>
<listOfParameters>
<parameter id="LOWER_BOUND" value="-1000" units="mmol_per_gDW_per_hr" constant="true"/>
<parameter id="UPPER_BOUND" value="1000" units="mmol_per_gDW_per_hr" constant="true"/>
<parameter id="FLUX_VALUE" value="0" units="mmol_per_gDW_per_hr" constant="true"/>
<parameter id="OBJECTIVE_COEFFICIENT" value="0" units="mmol_per_gDW_per_hr" constant="true"/>
</listOfParameters>
</kineticLaw>
</reaction>
<reaction id="R_R009" name="D-glyceraldehyde-3-phosphate:NAD+ oxidoreductase (phosphorylating)" reversible="true">
<notes>
<body xmlns="http://www.w3.org/1999/xhtml">
<p>GENE_ASSOCIATION: TTC0549</p>
<p>SUBSYSTEM: Glycolysis/Gluconeogenesis</p>
<p>EC Number: 1.2.1.12</p>
<p>Confidence Level: 1</p>
<p>AUTHORS: Carbohydrate metabolism</p>
<p/>
</body>
</notes>
<listOfReactants>
<speciesReference species="M_Pi_c" stoichiometry="1"/>
<speciesReference species="M_GAP_c" stoichiometry="1"/>
<speciesReference species="M_NAD_c" stoichiometry="1"/>
</listOfReactants>
<listOfProducts>
<speciesReference species="M_H_c" stoichiometry="1"/>
<speciesReference species="M_13BPG_c" stoichiometry="1"/>
<speciesReference species="M_NADH_c" stoichiometry="1"/>
```

```
    </listOfProducts>
    <kineticLaw>
      <math xmlns="http://www.w3.org/1998/Math/MathML">
        <ci> FLUX_VALUE </ci>
      </math>
      <listOfParameters>
        <parameter id="LOWER_BOUND" value="-1000" units="mmol_per_gDW_per_hr" constant="true"/>
        <parameter id="UPPER_BOUND" value="1000" units="mmol_per_gDW_per_hr" constant="true"/>
        <parameter id="FLUX_VALUE" value="0" units="mmol_per_gDW_per_hr" constant="true"/>
        <parameter id="OBJECTIVE_COEFFICIENT" value="0" units="mmol_per_gDW_per_hr" constant="true"/>
      </listOfParameters>
    </kineticLaw>
  </reaction>
  <reaction id="R_R010" name="ATP:3-phospho-D-glycerate 1-phosphotransferase" reversible="true">
    <notes>
      <body xmlns="http://www.w3.org/1999/xhtml">
        <p>GENE_ASSOCIATION: TTC0550</p>
        <p>SUBSYSTEM: Glycolysis/Gluconeogenesis</p>
        <p>EC Number: 2.7.2.3</p>
        <p>Confidence Level: 1</p>
        <p>AUTHORS: Carbohydrate metabolism</p>
        <p/>
      </body>
    </notes>
    <listOfReactants>
      <speciesReference species="M_ATP_c" stoichiometry="1"/>
      <speciesReference species="M_3PG_c" stoichiometry="1"/>
    </listOfReactants>
    <listOfProducts>
```

```
    <speciesReference species="M_ADP_c" stoichiometry="1"/>
    <speciesReference species="M_13BPG_c" stoichiometry="1"/>
  </listOfProducts>
  <kineticLaw>
    <math xmlns="http://www.w3.org/1998/Math/MathML">
      <ci> FLUX_VALUE </ci>
    </math>
    <listOfParameters>
      <parameter id="LOWER_BOUND" value="-1000" units="mmol_per_gDW_per_hr" constant="true"/>
      <parameter id="UPPER_BOUND" value="1000" units="mmol_per_gDW_per_hr" constant="true"/>
      <parameter id="FLUX_VALUE" value="0" units="mmol_per_gDW_per_hr" constant="true"/>
      <parameter id="OBJECTIVE_COEFFICIENT" value="0" units="mmol_per_gDW_per_hr" constant="true"/>
    </listOfParameters>
  </kineticLaw>
</reaction>
<reaction id="R_R011" name="2-Phospho-D-glycerate 2,3-phosphomutase" reversible="true">
  <notes>
    <body xmlns="http://www.w3.org/1999/xhtml">
      <p>GENE_ASSOCIATION: (TTC1888 or TTC1618 or TTC1956 or TT_P0006)</p>
      <p>SUBSYSTEM: Glycolysis/Gluconeogenesis</p>
      <p>EC Number: 5.4.2.1</p>
      <p>Confidence Level: 1</p>
      <p>AUTHORS: Carbohydrate metabolism</p>
      <p/>
    </body>
  </notes>
  <listOfReactants>
    <speciesReference species="M_2PG_c" stoichiometry="1"/>
  </listOfReactants>
```

```
  <listOfProducts>
    <speciesReference species="M_3PG_c" stoichiometry="1"/>
  </listOfProducts>
  <kineticLaw>
    <math xmlns="http://www.w3.org/1998/Math/MathML">
      <ci> FLUX_VALUE </ci>
    </math>
    <listOfParameters>
      <parameter id="LOWER_BOUND" value="-1000" units="mmol_per_gDW_per_hr" constant="true"/>
      <parameter id="UPPER_BOUND" value="1000" units="mmol_per_gDW_per_hr" constant="true"/>
      <parameter id="FLUX_VALUE" value="0" units="mmol_per_gDW_per_hr" constant="true"/>
      <parameter id="OBJECTIVE_COEFFICIENT" value="0" units="mmol_per_gDW_per_hr" constant="true"/>
    </listOfParameters>
  </kineticLaw>
</reaction>
<reaction id="R_R012" name="2-phospho-D-glycerate hydro-lyase (phosphoenolpyruvate-forming)" reversible="true">
  <notes>
    <body xmlns="http://www.w3.org/1999/xhtml">
      <p>GENE_ASSOCIATION: TTC1610</p>
      <p>SUBSYSTEM: Glycolysis/Gluconeogenesis</p>
      <p>EC Number: 4.2.1.11</p>
      <p>Confidence Level: 1</p>
      <p>AUTHORS: Carbohydrate metabolism</p>
      <p/>
    </body>
  </notes>
  <listOfReactants>
    <speciesReference species="M_H2O_c" stoichiometry="1"/>
    <speciesReference species="M_PEP_c" stoichiometry="1"/>
```

```
    </listOfReactants>
    <listOfProducts>
      <speciesReference species="M_2PG_c" stoichiometry="1"/>
    </listOfProducts>
    <kineticLaw>
      <math xmlns="http://www.w3.org/1998/Math/MathML">
        <ci> FLUX_VALUE </ci>
      </math>
      <listOfParameters>
        <parameter id="LOWER_BOUND" value="-1000" units="mmol_per_gDW_per_hr" constant="true"/>
        <parameter id="UPPER_BOUND" value="1000" units="mmol_per_gDW_per_hr" constant="true"/>
        <parameter id="FLUX_VALUE" value="0" units="mmol_per_gDW_per_hr" constant="true"/>
        <parameter id="OBJECTIVE_COEFFICIENT" value="0" units="mmol_per_gDW_per_hr" constant="true"/>
      </listOfParameters>
    </kineticLaw>
  </reaction>
  <reaction id="R_R013" name="ATP:pyruvate 2-O-phosphotransferase" reversible="false">
    <notes>
      <body xmlns="http://www.w3.org/1999/xhtml">
        <p>GENE_ASSOCIATION: TTC1611</p>
        <p>SUBSYSTEM: Glycolysis/Gluconeogenesis</p>
        <p>EC Number: 2.7.1.40</p>
        <p>Confidence Level: 1</p>
        <p>AUTHORS: Carbohydrate metabolism</p>
        <p/>
      </body>
    </notes>
    <listOfReactants>
      <speciesReference species="M_ADP_c" stoichiometry="1"/>
```

```
          <speciesReference species="M_H_c" stoichiometry="1"/>
          <speciesReference species="M_PEP_c" stoichiometry="1"/>
        </listOfReactants>
        <listOfProducts>
          <speciesReference species="M_ATP_c" stoichiometry="1"/>
          <speciesReference species="M_PYR_c" stoichiometry="1"/>
        </listOfProducts>
        <kineticLaw>
          <math xmlns="http://www.w3.org/1998/Math/MathML">
            <ci> FLUX_VALUE </ci>
          </math>
          <listOfParameters>
            <parameter id="LOWER_BOUND" value="0" units="mmol_per_gDW_per_hr" constant="true"/>
            <parameter id="UPPER_BOUND" value="1000" units="mmol_per_gDW_per_hr" constant="true"/>
            <parameter id="FLUX_VALUE" value="0" units="mmol_per_gDW_per_hr" constant="true"/>
            <parameter id="OBJECTIVE_COEFFICIENT" value="0" units="mmol_per_gDW_per_hr" constant="true"/>
          </listOfParameters>
        </kineticLaw>
      </reaction>
      <reaction id="R_R014" name="ATP:oxaloacetate carboxy-lyase (transphosphorylating;phosphoenolpyruvate-forming)"
reversible="false">
        <notes>
          <body xmlns="http://www.w3.org/1999/xhtml">
            <p>GENE_ASSOCIATION: TTC1709</p>
            <p>SUBSYSTEM: Glycolysis/Gluconeogenesis</p>
            <p>EC Number: 4.1.1.49</p>
            <p>Confidence Level: 1</p>
            <p>AUTHORS: Carbohydrate metabolism</p>
            <p/>
```

```
    </body>
  </notes>
  <listOfReactants>
    <speciesReference species="M_ATP_c" stoichiometry="1"/>
    <speciesReference species="M_OAC_c" stoichiometry="1"/>
  </listOfReactants>
  <listOfProducts>
    <speciesReference species="M_ADP_c" stoichiometry="1"/>
    <speciesReference species="M_PEP_c" stoichiometry="1"/>
    <speciesReference species="M_CO2_c" stoichiometry="1"/>
  </listOfProducts>
  <kineticLaw>
    <math xmlns="http://www.w3.org/1998/Math/MathML">
      <ci> FLUX_VALUE </ci>
    </math>
    <listOfParameters>
      <parameter id="LOWER_BOUND" value="0" units="mmol_per_gDW_per_hr" constant="true"/>
      <parameter id="UPPER_BOUND" value="1000" units="mmol_per_gDW_per_hr" constant="true"/>
      <parameter id="FLUX_VALUE" value="0" units="mmol_per_gDW_per_hr" constant="true"/>
      <parameter id="OBJECTIVE_COEFFICIENT" value="0" units="mmol_per_gDW_per_hr" constant="true"/>
    </listOfParameters>
  </kineticLaw>
</reaction>
<reaction id="R_R015" name="(S)-Lactate:NAD+ oxidoreductase" reversible="false">
  <notes>
    <body xmlns="http://www.w3.org/1999/xhtml">
      <p>GENE_ASSOCIATION: TTC0748</p>
      <p>SUBSYSTEM: Glycolysis/Gluconeogenesis</p>
      <p>EC Number: 1.1.1.27</p>
```

```
      <p>Confidence Level: 1</p>
      <p>AUTHORS: Carbohydrate metabolism</p>
      <p/>
    </body>
  </notes>
  <listOfReactants>
    <speciesReference species="M_H_c" stoichiometry="1"/>
    <speciesReference species="M_NADH_c" stoichiometry="1"/>
    <speciesReference species="M_PYR_c" stoichiometry="1"/>
  </listOfReactants>
  <listOfProducts>
    <speciesReference species="M_NAD_c" stoichiometry="1"/>
    <speciesReference species="M_LAC_c" stoichiometry="1"/>
  </listOfProducts>
  <kineticLaw>
    <math xmlns="http://www.w3.org/1998/Math/MathML">
      <ci> FLUX_VALUE </ci>
    </math>
    <listOfParameters>
      <parameter id="LOWER_BOUND" value="0" units="mmol_per_gDW_per_hr" constant="true"/>
      <parameter id="UPPER_BOUND" value="0" units="mmol_per_gDW_per_hr" constant="true"/>
      <parameter id="FLUX_VALUE" value="0" units="mmol_per_gDW_per_hr" constant="true"/>
      <parameter id="OBJECTIVE_COEFFICIENT" value="0" units="mmol_per_gDW_per_hr" constant="true"/>
    </listOfParameters>
  </kineticLaw>
</reaction>
<reaction id="R_R016" name="Acetate:CoA ligase (AMP-forming)" reversible="false">
  <notes>
    <body xmlns="http://www.w3.org/1999/xhtml">
```

```xml
    <p>GENE_ASSOCIATION: (TTC0884 and TTC0885 and TTC0886 and TTC0919)</p>
    <p>SUBSYSTEM: Glycolysis/Gluconeogenesis</p>
    <p>EC Number: 6.2.1.1</p>
    <p>Confidence Level: 1</p>
    <p>AUTHORS: Carbohydrate metabolism</p>
    <p/>
  </body>
</notes>
<listOfReactants>
  <speciesReference species="M_ATP_c" stoichiometry="1"/>
  <speciesReference species="M_CoA_c" stoichiometry="1"/>
  <speciesReference species="M_AC_c" stoichiometry="1"/>
</listOfReactants>
<listOfProducts>
  <speciesReference species="M_PPi_c" stoichiometry="1"/>
  <speciesReference species="M_AMP_c" stoichiometry="1"/>
  <speciesReference species="M_ACoA_c" stoichiometry="1"/>
</listOfProducts>
<kineticLaw>
  <math xmlns="http://www.w3.org/1998/Math/MathML">
    <ci> FLUX_VALUE </ci>
  </math>
  <listOfParameters>
    <parameter id="LOWER_BOUND" value="0" units="mmol_per_gDW_per_hr" constant="true"/>
    <parameter id="UPPER_BOUND" value="1000" units="mmol_per_gDW_per_hr" constant="true"/>
    <parameter id="FLUX_VALUE" value="0" units="mmol_per_gDW_per_hr" constant="true"/>
    <parameter id="OBJECTIVE_COEFFICIENT" value="0" units="mmol_per_gDW_per_hr" constant="true"/>
  </listOfParameters>
</kineticLaw>
```

```
</reaction>
<reaction id="R_R017" name="Acetaldehyde:NAD+ oxidoreductase" reversible="false">
 <notes>
  <body xmlns="http://www.w3.org/1999/xhtml">
   <p>GENE_ASSOCIATION: (TTC0513 or TTC0604)</p>
   <p>SUBSYSTEM: Glycolysis/Gluconeogenesis</p>
   <p>EC Number: 1.2.1.3</p>
   <p>Confidence Level: 1</p>
   <p>AUTHORS: Carbohydrate metabolism</p>
   <p/>
  </body>
 </notes>
 <listOfReactants>
  <speciesReference species="M_H2O_c" stoichiometry="1"/>
  <speciesReference species="M_NAD_c" stoichiometry="1"/>
  <speciesReference species="M_AALD_c" stoichiometry="1"/>
 </listOfReactants>
 <listOfProducts>
  <speciesReference species="M_H_c" stoichiometry="2"/>
  <speciesReference species="M_NADH_c" stoichiometry="1"/>
  <speciesReference species="M_AC_c" stoichiometry="1"/>
 </listOfProducts>
 <kineticLaw>
  <math xmlns="http://www.w3.org/1998/Math/MathML">
   <ci> FLUX_VALUE </ci>
  </math>
  <listOfParameters>
   <parameter id="LOWER_BOUND" value="0" units="mmol_per_gDW_per_hr" constant="true"/>
   <parameter id="UPPER_BOUND" value="1000" units="mmol_per_gDW_per_hr" constant="true"/>
```

```
      <parameter id="FLUX_VALUE" value="0" units="mmol_per_gDW_per_hr" constant="true"/>
      <parameter id="OBJECTIVE_COEFFICIENT" value="0" units="mmol_per_gDW_per_hr" constant="true"/>
    </listOfParameters>
  </kineticLaw>
</reaction>
<reaction id="R_R018" name="ethanol:NAD+ oxidoreductase" reversible="true">
  <notes>
    <body xmlns="http://www.w3.org/1999/xhtml">
      <p>GENE_ASSOCIATION: TTC1572</p>
      <p>SUBSYSTEM: Glycolysis/Gluconeogenesis</p>
      <p>EC Number: 1.1.1.1</p>
      <p>Confidence Level: 1</p>
      <p>AUTHORS: Carbohydrate metabolism</p>
      <p/>
    </body>
  </notes>
  <listOfReactants>
    <speciesReference species="M_NAD_c" stoichiometry="1"/>
    <speciesReference species="M_ETH_c" stoichiometry="1"/>
  </listOfReactants>
  <listOfProducts>
    <speciesReference species="M_H_c" stoichiometry="1"/>
    <speciesReference species="M_NADH_c" stoichiometry="1"/>
    <speciesReference species="M_AALD_c" stoichiometry="1"/>
  </listOfProducts>
  <kineticLaw>
    <math xmlns="http://www.w3.org/1998/Math/MathML">
      <ci> FLUX_VALUE </ci>
    </math>
```

```
    <listOfParameters>
      <parameter id="LOWER_BOUND" value="-1000" units="mmol_per_gDW_per_hr" constant="true"/>
      <parameter id="UPPER_BOUND" value="1000" units="mmol_per_gDW_per_hr" constant="true"/>
      <parameter id="FLUX_VALUE" value="0" units="mmol_per_gDW_per_hr" constant="true"/>
      <parameter id="OBJECTIVE_COEFFICIENT" value="0" units="mmol_per_gDW_per_hr" constant="true"/>
    </listOfParameters>
  </kineticLaw>
</reaction>
<reaction id="R_R019" name="acetyl-CoA:oxaloacetate C-acetyltransferase (thioester-hydrolysing)" reversible="false">
  <notes>
    <body xmlns="http://www.w3.org/1999/xhtml">
      <p>GENE_ASSOCIATION: TTC0978</p>
      <p>SUBSYSTEM: Citric Acid Cycle</p>
      <p>EC Number: 2.3.3.1</p>
      <p>Confidence Level: 1</p>
      <p>AUTHORS: Carbohydrate metabolism</p>
      <p/>
    </body>
  </notes>
  <listOfReactants>
    <speciesReference species="M_H2O_c" stoichiometry="1"/>
    <speciesReference species="M_OAC_c" stoichiometry="1"/>
    <speciesReference species="M_ACoA_c" stoichiometry="1"/>
  </listOfReactants>
  <listOfProducts>
    <speciesReference species="M_H_c" stoichiometry="1"/>
    <speciesReference species="M_CoA_c" stoichiometry="1"/>
    <speciesReference species="M_CIT_c" stoichiometry="1"/>
  </listOfProducts>
```

```
  <kineticLaw>
   <math xmlns="http://www.w3.org/1998/Math/MathML">
    <ci> FLUX_VALUE </ci>
   </math>
   <listOfParameters>
    <parameter id="LOWER_BOUND" value="0" units="mmol_per_gDW_per_hr" constant="true"/>
    <parameter id="UPPER_BOUND" value="1000" units="mmol_per_gDW_per_hr" constant="true"/>
    <parameter id="FLUX_VALUE" value="0" units="mmol_per_gDW_per_hr" constant="true"/>
    <parameter id="OBJECTIVE_COEFFICIENT" value="0" units="mmol_per_gDW_per_hr" constant="true"/>
   </listOfParameters>
  </kineticLaw>
 </reaction>
 <reaction id="R_R020" name="citrate hydro-lyase (cis-aconitate-forming)" reversible="true">
  <notes>
   <body xmlns="http://www.w3.org/1999/xhtml">
    <p>GENE_ASSOCIATION: TTC0374</p>
    <p>SUBSYSTEM: Citric Acid Cycle</p>
    <p>EC Number: 4.2.1.3</p>
    <p>Confidence Level: 1</p>
    <p>AUTHORS: Carbohydrate metabolism</p>
    <p/>
   </body>
  </notes>
  <listOfReactants>
   <speciesReference species="M_CIT_c" stoichiometry="1"/>
  </listOfReactants>
  <listOfProducts>
   <speciesReference species="M_H2O_c" stoichiometry="1"/>
   <speciesReference species="M_cACO_c" stoichiometry="1"/>
```

```
    </listOfProducts>
    <kineticLaw>
      <math xmlns="http://www.w3.org/1998/Math/MathML">
        <ci> FLUX_VALUE </ci>
      </math>
      <listOfParameters>
        <parameter id="LOWER_BOUND" value="-1000" units="mmol_per_gDW_per_hr" constant="true"/>
        <parameter id="UPPER_BOUND" value="1000" units="mmol_per_gDW_per_hr" constant="true"/>
        <parameter id="FLUX_VALUE" value="0" units="mmol_per_gDW_per_hr" constant="true"/>
        <parameter id="OBJECTIVE_COEFFICIENT" value="0" units="mmol_per_gDW_per_hr" constant="true"/>
      </listOfParameters>
    </kineticLaw>
  </reaction>
  <reaction id="R_R021" name="isocitrate hydro-lyase (cis-aconitate-forming)" reversible="true">
    <notes>
      <body xmlns="http://www.w3.org/1999/xhtml">
        <p>GENE_ASSOCIATION: TTC0374</p>
        <p>SUBSYSTEM: Citric Acid Cycle</p>
        <p>EC Number: 4.2.1.3</p>
        <p>Confidence Level: 1</p>
        <p>AUTHORS: Carbohydrate metabolism</p>
        <p/>
      </body>
    </notes>
    <listOfReactants>
      <speciesReference species="M_ICIT_c" stoichiometry="1"/>
    </listOfReactants>
    <listOfProducts>
      <speciesReference species="M_H2O_c" stoichiometry="1"/>
```

```
<speciesReference species="M_cACO_c" stoichiometry="1"/>
</listOfProducts>
<kineticLaw>
<math xmlns="http://www.w3.org/1998/Math/MathML">
<ci> FLUX_VALUE </ci>
</math>
<listOfParameters>
<parameter id="LOWER_BOUND" value="-1000" units="mmol_per_gDW_per_hr" constant="true"/>
<parameter id="UPPER_BOUND" value="1000" units="mmol_per_gDW_per_hr" constant="true"/>
<parameter id="FLUX_VALUE" value="0" units="mmol_per_gDW_per_hr" constant="true"/>
<parameter id="OBJECTIVE_COEFFICIENT" value="0" units="mmol_per_gDW_per_hr" constant="true"/>
</listOfParameters>
</kineticLaw>
</reaction>
<reaction id="R_R022" name="Isocitrate:NADP+ oxidoreductase" reversible="false">
<notes>
<body xmlns="http://www.w3.org/1999/xhtml">
<p>GENE_ASSOCIATION: TTC1172</p>
<p>SUBSYSTEM: Citric Acid Cycle</p>
<p>EC Number: 1.1.1.42</p>
<p>Confidence Level: 1</p>
<p>AUTHORS: Carbohydrate metabolism</p>
<p/>
</body>
</notes>
<listOfReactants>
<speciesReference species="M_ICIT_c" stoichiometry="1"/>
<speciesReference species="M_NADP_c" stoichiometry="1"/>
</listOfReactants>
```

```
  <listOfProducts>
    <speciesReference species="M_H_c" stoichiometry="1"/>
    <speciesReference species="M_OXLSUCC_c" stoichiometry="1"/>
    <speciesReference species="M_NADPH_c" stoichiometry="1"/>
  </listOfProducts>
  <kineticLaw>
    <math xmlns="http://www.w3.org/1998/Math/MathML">
      <ci> FLUX_VALUE </ci>
    </math>
    <listOfParameters>
      <parameter id="LOWER_BOUND" value="0" units="mmol_per_gDW_per_hr" constant="true"/>
      <parameter id="UPPER_BOUND" value="1000" units="mmol_per_gDW_per_hr" constant="true"/>
      <parameter id="FLUX_VALUE" value="0" units="mmol_per_gDW_per_hr" constant="true"/>
      <parameter id="OBJECTIVE_COEFFICIENT" value="0" units="mmol_per_gDW_per_hr" constant="true"/>
    </listOfParameters>
  </kineticLaw>
</reaction>
<reaction id="R_R023" name="oxalosuccinate carboxy-lyase (2-oxoglutarate-forming)" reversible="false">
  <notes>
    <body xmlns="http://www.w3.org/1999/xhtml">
      <p>GENE_ASSOCIATION: TTC1172</p>
      <p>SUBSYSTEM: Citric Acid Cycle</p>
      <p>EC Number: 1.1.1.42</p>
      <p>Confidence Level: 1</p>
      <p>AUTHORS: Carbohydrate metabolism</p>
      <p/>
    </body>
  </notes>
  <listOfReactants>
```

```
      <speciesReference species="M_H_c" stoichiometry="1"/>
      <speciesReference species="M_OXLSUCC_c" stoichiometry="1"/>
    </listOfReactants>
    <listOfProducts>
      <speciesReference species="M_CO2_c" stoichiometry="1"/>
      <speciesReference species="M_AKG_c" stoichiometry="1"/>
    </listOfProducts>
    <kineticLaw>
      <math xmlns="http://www.w3.org/1998/Math/MathML">
        <ci> FLUX_VALUE </ci>
      </math>
      <listOfParameters>
        <parameter id="LOWER_BOUND" value="0" units="mmol_per_gDW_per_hr" constant="true"/>
        <parameter id="UPPER_BOUND" value="1000" units="mmol_per_gDW_per_hr" constant="true"/>
        <parameter id="FLUX_VALUE" value="0" units="mmol_per_gDW_per_hr" constant="true"/>
        <parameter id="OBJECTIVE_COEFFICIENT" value="0" units="mmol_per_gDW_per_hr" constant="true"/>
      </listOfParameters>
    </kineticLaw>
  </reaction>
  <reaction id="R_R024" name="2-oxoglutarate:ferredoxin oxidoreductase (decarboxylating)" reversible="false">
    <notes>
      <body xmlns="http://www.w3.org/1999/xhtml">
        <p>GENE_ASSOCIATION: (TTC1591 and TTC1592)</p>
        <p>SUBSYSTEM: Citric Acid Cycle</p>
        <p>EC Number: 1.2.7.3</p>
        <p>Confidence Level: 1</p>
        <p>AUTHORS: Carbohydrate metabolism</p>
        <p/>
      </body>
```

```
  </notes>
  <listOfReactants>
    <speciesReference species="M_NAD_c" stoichiometry="1"/>
    <speciesReference species="M_CoA_c" stoichiometry="1"/>
    <speciesReference species="M_AKG_c" stoichiometry="1"/>
  </listOfReactants>
  <listOfProducts>
    <speciesReference species="M_NADH_c" stoichiometry="1"/>
    <speciesReference species="M_CO2_c" stoichiometry="1"/>
    <speciesReference species="M_SUCCoA_c" stoichiometry="1"/>
  </listOfProducts>
  <kineticLaw>
    <math xmlns="http://www.w3.org/1998/Math/MathML">
      <ci> FLUX_VALUE </ci>
    </math>
    <listOfParameters>
      <parameter id="LOWER_BOUND" value="0" units="mmol_per_gDW_per_hr" constant="true"/>
      <parameter id="UPPER_BOUND" value="1000" units="mmol_per_gDW_per_hr" constant="true"/>
      <parameter id="FLUX_VALUE" value="0" units="mmol_per_gDW_per_hr" constant="true"/>
      <parameter id="OBJECTIVE_COEFFICIENT" value="0" units="mmol_per_gDW_per_hr" constant="true"/>
    </listOfParameters>
  </kineticLaw>
</reaction>
<reaction id="R_R025" name="Succinate:CoA ligase (ADP-forming)" reversible="true">
  <notes>
    <body xmlns="http://www.w3.org/1999/xhtml">
      <p>GENE_ASSOCIATION: (TTC0169 and TTC0170)</p>
      <p>SUBSYSTEM: Citric Acid Cycle</p>
      <p>EC Number: 6.2.1.5</p>
```

```
      <p>Confidence Level: 1</p>
      <p>AUTHORS: Carbohydrate metabolism</p>
      <p/>
    </body>
  </notes>
  <listOfReactants>
    <speciesReference species="M_ATP_c" stoichiometry="1"/>
    <speciesReference species="M_CoA_c" stoichiometry="1"/>
    <speciesReference species="M_SUCC_c" stoichiometry="1"/>
  </listOfReactants>
  <listOfProducts>
    <speciesReference species="M_ADP_c" stoichiometry="1"/>
    <speciesReference species="M_Pi_c" stoichiometry="1"/>
    <speciesReference species="M_SUCCoA_c" stoichiometry="1"/>
  </listOfProducts>
  <kineticLaw>
    <math xmlns="http://www.w3.org/1998/Math/MathML">
      <ci> FLUX_VALUE </ci>
    </math>
    <listOfParameters>
      <parameter id="LOWER_BOUND" value="-1000" units="mmol_per_gDW_per_hr" constant="true"/>
      <parameter id="UPPER_BOUND" value="1000" units="mmol_per_gDW_per_hr" constant="true"/>
      <parameter id="FLUX_VALUE" value="0" units="mmol_per_gDW_per_hr" constant="true"/>
      <parameter id="OBJECTIVE_COEFFICIENT" value="0" units="mmol_per_gDW_per_hr" constant="true"/>
    </listOfParameters>
  </kineticLaw>
</reaction>
<reaction id="R_R026" name="Succinate:(acceptor) oxidoreductase" reversible="true">
  <notes>
```

```xml
    <body xmlns="http://www.w3.org/1999/xhtml">
      <p>GENE_ASSOCIATION: (TTC1089 and TTC1090 and TTC1091 and TTC1092)</p>
      <p>SUBSYSTEM: Citric Acid Cycle</p>
      <p>EC Number: 1.3.99.1</p>
      <p>Confidence Level: 1</p>
      <p>AUTHORS: Carbohydrate metabolism</p>
      <p/>
    </body>
  </notes>
  <listOfReactants>
    <speciesReference species="M_SUCC_c" stoichiometry="1"/>
    <speciesReference species="M_FAD_c" stoichiometry="1"/>
  </listOfReactants>
  <listOfProducts>
    <speciesReference species="M_FADH2_c" stoichiometry="1"/>
    <speciesReference species="M_FUM_c" stoichiometry="1"/>
  </listOfProducts>
  <kineticLaw>
    <math xmlns="http://www.w3.org/1998/Math/MathML">
      <ci> FLUX_VALUE </ci>
    </math>
    <listOfParameters>
      <parameter id="LOWER_BOUND" value="-1000" units="mmol_per_gDW_per_hr" constant="true"/>
      <parameter id="UPPER_BOUND" value="1000" units="mmol_per_gDW_per_hr" constant="true"/>
      <parameter id="FLUX_VALUE" value="0" units="mmol_per_gDW_per_hr" constant="true"/>
      <parameter id="OBJECTIVE_COEFFICIENT" value="0" units="mmol_per_gDW_per_hr" constant="true"/>
    </listOfParameters>
  </kineticLaw>
</reaction>
```

```xml
<reaction id="R_R027" name="(S)-malate hydro-lyase (fumarate-forming)" reversible="true">
 <notes>
  <body xmlns="http://www.w3.org/1999/xhtml">
   <p>GENE_ASSOCIATION: TTC0190</p>
   <p>SUBSYSTEM: Citric Acid Cycle</p>
   <p>EC Number: 4.2.1.2</p>
   <p>Confidence Level: 1</p>
   <p>AUTHORS: Carbohydrate metabolism</p>
   <p/>
  </body>
 </notes>
 <listOfReactants>
  <speciesReference species="M_MAL_c" stoichiometry="1"/>
 </listOfReactants>
 <listOfProducts>
  <speciesReference species="M_H2O_c" stoichiometry="1"/>
  <speciesReference species="M_FUM_c" stoichiometry="1"/>
 </listOfProducts>
 <kineticLaw>
  <math xmlns="http://www.w3.org/1998/Math/MathML">
   <ci> FLUX_VALUE </ci>
  </math>
  <listOfParameters>
   <parameter id="LOWER_BOUND" value="-1000" units="mmol_per_gDW_per_hr" constant="true"/>
   <parameter id="UPPER_BOUND" value="1000" units="mmol_per_gDW_per_hr" constant="true"/>
   <parameter id="FLUX_VALUE" value="0" units="mmol_per_gDW_per_hr" constant="true"/>
   <parameter id="OBJECTIVE_COEFFICIENT" value="0" units="mmol_per_gDW_per_hr" constant="true"/>
  </listOfParameters>
 </kineticLaw>
```

```
</reaction>
<reaction id="R_R028" name="(S)-malate:NAD+ oxidoreductase" reversible="true">
 <notes>
  <body xmlns="http://www.w3.org/1999/xhtml">
   <p>GENE_ASSOCIATION: TTC0168</p>
   <p>SUBSYSTEM: Citric Acid Cycle</p>
   <p>EC Number: 1.1.1.37</p>
   <p>Confidence Level: 1</p>
   <p>AUTHORS: Carbohydrate metabolism</p>
   <p/>
  </body>
 </notes>
 <listOfReactants>
  <speciesReference species="M_NAD_c" stoichiometry="1"/>
  <speciesReference species="M_MAL_c" stoichiometry="1"/>
 </listOfReactants>
 <listOfProducts>
  <speciesReference species="M_H_c" stoichiometry="1"/>
  <speciesReference species="M_NADH_c" stoichiometry="1"/>
  <speciesReference species="M_OAC_c" stoichiometry="1"/>
 </listOfProducts>
 <kineticLaw>
  <math xmlns="http://www.w3.org/1998/Math/MathML">
   <ci> FLUX_VALUE </ci>
  </math>
  <listOfParameters>
   <parameter id="LOWER_BOUND" value="-1000" units="mmol_per_gDW_per_hr" constant="true"/>
   <parameter id="UPPER_BOUND" value="1000" units="mmol_per_gDW_per_hr" constant="true"/>
   <parameter id="FLUX_VALUE" value="0" units="mmol_per_gDW_per_hr" constant="true"/>
```

```
      <parameter id="OBJECTIVE_COEFFICIENT" value="0" units="mmol_per_gDW_per_hr" constant="true"/>
    </listOfParameters>
  </kineticLaw>
</reaction>
<reaction id="R_R029" name="D-glucose:ubiquinone oxidoreductase" reversible="false">
  <notes>
    <body xmlns="http://www.w3.org/1999/xhtml">
      <p>GENE_ASSOCIATION: TTC0202</p>
      <p>SUBSYSTEM: Pentose Phosphate Pathway</p>
      <p>EC Number: 1.1.5.2</p>
      <p>Confidence Level: 1</p>
      <p>AUTHORS: Carbohydrate metabolism</p>
      <p/>
    </body>
  </notes>
  <listOfReactants>
    <speciesReference species="M_D_GLC_c" stoichiometry="1"/>
    <speciesReference species="M_Q_c" stoichiometry="1"/>
  </listOfReactants>
  <listOfProducts>
    <speciesReference species="M_GLC_LACN_c" stoichiometry="1"/>
    <speciesReference species="M_QH2_c" stoichiometry="1"/>
  </listOfProducts>
  <kineticLaw>
    <math xmlns="http://www.w3.org/1998/Math/MathML">
      <ci> FLUX_VALUE </ci>
    </math>
    <listOfParameters>
      <parameter id="LOWER_BOUND" value="0" units="mmol_per_gDW_per_hr" constant="true"/>
```

```
<parameter id="UPPER_BOUND" value="1000" units="mmol_per_gDW_per_hr" constant="true"/>
<parameter id="FLUX_VALUE" value="0" units="mmol_per_gDW_per_hr" constant="true"/>
<parameter id="OBJECTIVE_COEFFICIENT" value="0" units="mmol_per_gDW_per_hr" constant="true"/>
</listOfParameters>
</kineticLaw>
</reaction>
<reaction id="R_R030" name="D-Fructose 6-phosphate:D-glyceraldehyde-3-phosphate glycolaldehyde transferase" reversible="true">
<notes>
<body xmlns="http://www.w3.org/1999/xhtml">
<p>GENE_ASSOCIATION: TTC1896</p>
<p>SUBSYSTEM: Pentose Phosphate Pathway</p>
<p>EC Number: 2.2.1.1</p>
<p>Confidence Level: 1</p>
<p>AUTHORS: Carbohydrate metabolism</p>
<p/>
</body>
</notes>
<listOfReactants>
<speciesReference species="M_F6P_c" stoichiometry="1"/>
<speciesReference species="M_GAP_c" stoichiometry="1"/>
</listOfReactants>
<listOfProducts>
<speciesReference species="M_E4P_c" stoichiometry="1"/>
<speciesReference species="M_XYL5P_c" stoichiometry="1"/>
</listOfProducts>
<kineticLaw>
<math xmlns="http://www.w3.org/1998/Math/MathML">
<ci> FLUX_VALUE </ci>
```

```xml
</math>

<listOfParameters>

<parameter id="LOWER_BOUND" value="-1000" units="mmol_per_gDW_per_hr" constant="true"/>

<parameter id="UPPER_BOUND" value="1000" units="mmol_per_gDW_per_hr" constant="true"/>

<parameter id="FLUX_VALUE" value="0" units="mmol_per_gDW_per_hr" constant="true"/>

<parameter id="OBJECTIVE_COEFFICIENT" value="0" units="mmol_per_gDW_per_hr" constant="true"/>

</listOfParameters>

</kineticLaw>

</reaction>

<reaction id="R_R031" name="sedoheptulose-7-phosphate:D-glyceraldehyde-3-phosphate glyceronetransferase" reversible="true">

<notes>

<body xmlns="http://www.w3.org/1999/xhtml">

<p>GENE_ASSOCIATION: TTC0701</p>

<p>SUBSYSTEM: Pentose Phosphate Pathway</p>

<p>EC Number: 2.2.1.2</p>

<p>Confidence Level: 1</p>

<p>AUTHORS: Carbohydrate metabolism</p>

<p/>

</body>

</notes>

<listOfReactants>

<speciesReference species="M_GAP_c" stoichiometry="1"/>

<speciesReference species="M_SEDHEP7P_c" stoichiometry="1"/>

</listOfReactants>

<listOfProducts>

<speciesReference species="M_F6P_c" stoichiometry="1"/>

<speciesReference species="M_E4P_c" stoichiometry="1"/>

</listOfProducts>
```

```
        <kineticLaw>
          <math xmlns="http://www.w3.org/1998/Math/MathML">
            <ci> FLUX_VALUE </ci>
          </math>
          <listOfParameters>
            <parameter id="LOWER_BOUND" value="-1000" units="mmol_per_gDW_per_hr" constant="true"/>
            <parameter id="UPPER_BOUND" value="1000" units="mmol_per_gDW_per_hr" constant="true"/>
            <parameter id="FLUX_VALUE" value="0" units="mmol_per_gDW_per_hr" constant="true"/>
            <parameter id="OBJECTIVE_COEFFICIENT" value="0" units="mmol_per_gDW_per_hr" constant="true"/>
          </listOfParameters>
        </kineticLaw>
      </reaction>
      <reaction id="R_R032" name="Sedoheptulose-7-phosphate:D-glyceraldehyde-3-phosphate glycolaldehyde transferase"
reversible="true">
        <notes>
          <body xmlns="http://www.w3.org/1999/xhtml">
            <p>GENE_ASSOCIATION: TTC1896</p>
            <p>SUBSYSTEM: Pentose Phosphate Pathway</p>
            <p>EC Number: 2.2.1.1</p>
            <p>Confidence Level: 1</p>
            <p>AUTHORS: Carbohydrate metabolism</p>
            <p/>
          </body>
        </notes>
        <listOfReactants>
          <speciesReference species="M_GAP_c" stoichiometry="1"/>
          <speciesReference species="M_SEDHEP7P_c" stoichiometry="1"/>
        </listOfReactants>
        <listOfProducts>
```

```
    <speciesReference species="M_XYL5P_c" stoichiometry="1"/>
    <speciesReference species="M_R5P_c" stoichiometry="1"/>
  </listOfProducts>
  <kineticLaw>
    <math xmlns="http://www.w3.org/1998/Math/MathML">
      <ci> FLUX_VALUE </ci>
    </math>
    <listOfParameters>
      <parameter id="LOWER_BOUND" value="-1000" units="mmol_per_gDW_per_hr" constant="true"/>
      <parameter id="UPPER_BOUND" value="1000" units="mmol_per_gDW_per_hr" constant="true"/>
      <parameter id="FLUX_VALUE" value="0" units="mmol_per_gDW_per_hr" constant="true"/>
      <parameter id="OBJECTIVE_COEFFICIENT" value="0" units="mmol_per_gDW_per_hr" constant="true"/>
    </listOfParameters>
  </kineticLaw>
</reaction>
<reaction id="R_R033" name="D-Ribulose-5-phosphate 3-epimerase" reversible="true">
  <notes>
    <body xmlns="http://www.w3.org/1999/xhtml">
      <p>GENE_ASSOCIATION: TTC1898</p>
      <p>SUBSYSTEM: Pentose Phosphate Pathway</p>
      <p>EC Number: 5.1.3.1</p>
      <p>Confidence Level: 1</p>
      <p>AUTHORS: Carbohydrate metabolism</p>
      <p/>
    </body>
  </notes>
  <listOfReactants>
    <speciesReference species="M_RBL5P_c" stoichiometry="1"/>
  </listOfReactants>
```

```
  <listOfProducts>
    <speciesReference species="M_XYL5P_c" stoichiometry="1"/>
  </listOfProducts>
  <kineticLaw>
    <math xmlns="http://www.w3.org/1998/Math/MathML">
      <ci> FLUX_VALUE </ci>
    </math>
    <listOfParameters>
      <parameter id="LOWER_BOUND" value="-1000" units="mmol_per_gDW_per_hr" constant="true"/>
      <parameter id="UPPER_BOUND" value="1000" units="mmol_per_gDW_per_hr" constant="true"/>
      <parameter id="FLUX_VALUE" value="0" units="mmol_per_gDW_per_hr" constant="true"/>
      <parameter id="OBJECTIVE_COEFFICIENT" value="0" units="mmol_per_gDW_per_hr" constant="true"/>
    </listOfParameters>
  </kineticLaw>
</reaction>
<reaction id="R_R034" name="D-ribose-5-phosphate aldose-ketose-isomerase" reversible="true">
  <notes>
    <body xmlns="http://www.w3.org/1999/xhtml">
      <p>GENE_ASSOCIATION: TTC0932</p>
      <p>SUBSYSTEM: Pentose Phosphate Pathway</p>
      <p>EC Number: 5.3.1.6</p>
      <p>Confidence Level: 1</p>
      <p>AUTHORS: Carbohydrate metabolism</p>
      <p/>
    </body>
  </notes>
  <listOfReactants>
    <speciesReference species="M_R5P_c" stoichiometry="1"/>
  </listOfReactants>
```

```
  <listOfProducts>
   <speciesReference species="M_RBL5P_c" stoichiometry="1"/>
  </listOfProducts>
  <kineticLaw>
   <math xmlns="http://www.w3.org/1998/Math/MathML">
    <ci> FLUX_VALUE </ci>
   </math>
   <listOfParameters>
    <parameter id="LOWER_BOUND" value="-1000" units="mmol_per_gDW_per_hr" constant="true"/>
    <parameter id="UPPER_BOUND" value="1000" units="mmol_per_gDW_per_hr" constant="true"/>
    <parameter id="FLUX_VALUE" value="0" units="mmol_per_gDW_per_hr" constant="true"/>
    <parameter id="OBJECTIVE_COEFFICIENT" value="0" units="mmol_per_gDW_per_hr" constant="true"/>
   </listOfParameters>
  </kineticLaw>
 </reaction>
 <reaction id="R_R035" name="ATP:D-ribose-5-phosphate diphosphotransferase" reversible="true">
  <notes>
   <body xmlns="http://www.w3.org/1999/xhtml">
    <p>GENE_ASSOCIATION: (TTC1184 or TTC1274)</p>
    <p>SUBSYSTEM: Pentose Phosphate Pathway</p>
    <p>EC Number: 2.7.6.1</p>
    <p>Confidence Level: 1</p>
    <p>AUTHORS: Carbohydrate metabolism</p>
    <p/>
   </body>
  </notes>
  <listOfReactants>
   <speciesReference species="M_ATP_c" stoichiometry="1"/>
   <speciesReference species="M_R5P_c" stoichiometry="1"/>
```

```
  </listOfReactants>
  <listOfProducts>
    <speciesReference species="M_H_c" stoichiometry="1"/>
    <speciesReference species="M_AMP_c" stoichiometry="1"/>
    <speciesReference species="M_PRPP_c" stoichiometry="1"/>
  </listOfProducts>
  <kineticLaw>
    <math xmlns="http://www.w3.org/1998/Math/MathML">
      <ci> FLUX_VALUE </ci>
    </math>
    <listOfParameters>
      <parameter id="LOWER_BOUND" value="-1000" units="mmol_per_gDW_per_hr" constant="true"/>
      <parameter id="UPPER_BOUND" value="1000" units="mmol_per_gDW_per_hr" constant="true"/>
      <parameter id="FLUX_VALUE" value="0" units="mmol_per_gDW_per_hr" constant="true"/>
      <parameter id="OBJECTIVE_COEFFICIENT" value="0" units="mmol_per_gDW_per_hr" constant="true"/>
    </listOfParameters>
  </kineticLaw>
</reaction>
<reaction id="R_R036" name="D-Ribose 1,5-phosphomutase" reversible="true">
  <notes>
    <body xmlns="http://www.w3.org/1999/xhtml">
      <p>GENE_ASSOCIATION: (TTC1630 or TTC1659)</p>
      <p>SUBSYSTEM: Pentose Phosphate Pathway</p>
      <p>EC Number: 5.4.2.2 or 5.4.2.7</p>
      <p>Confidence Level: 1</p>
      <p>AUTHORS: Carbohydrate metabolism</p>
      <p/>
    </body>
  </notes>
```

```
  <listOfReactants>
    <speciesReference species="M_R1P_c" stoichiometry="1"/>
  </listOfReactants>
  <listOfProducts>
    <speciesReference species="M_R5P_c" stoichiometry="1"/>
  </listOfProducts>
  <kineticLaw>
    <math xmlns="http://www.w3.org/1998/Math/MathML">
      <ci> FLUX_VALUE </ci>
    </math>
    <listOfParameters>
      <parameter id="LOWER_BOUND" value="-1000" units="mmol_per_gDW_per_hr" constant="true"/>
      <parameter id="UPPER_BOUND" value="1000" units="mmol_per_gDW_per_hr" constant="true"/>
      <parameter id="FLUX_VALUE" value="0" units="mmol_per_gDW_per_hr" constant="true"/>
      <parameter id="OBJECTIVE_COEFFICIENT" value="0" units="mmol_per_gDW_per_hr" constant="true"/>
    </listOfParameters>
  </kineticLaw>
</reaction>
<reaction id="R_R037" name="ATP:D-ribose 5-phosphotransferase" reversible="false">
  <notes>
    <body xmlns="http://www.w3.org/1999/xhtml">
      <p>GENE_ASSOCIATION: TTC0063</p>
      <p>SUBSYSTEM: Pentose Phosphate Pathway</p>
      <p>EC Number: 2.7.1.15</p>
      <p>Confidence Level: 1</p>
      <p>AUTHORS: Carbohydrate metabolism</p>
      <p/>
    </body>
  </notes>
```

```
        <listOfReactants>
          <speciesReference species="M_ATP_c" stoichiometry="1"/>
          <speciesReference species="M_D_RIB_c" stoichiometry="1"/>
        </listOfReactants>
        <listOfProducts>
          <speciesReference species="M_ADP_c" stoichiometry="1"/>
          <speciesReference species="M_H_c" stoichiometry="1"/>
          <speciesReference species="M_R5P_c" stoichiometry="1"/>
        </listOfProducts>
        <kineticLaw>
          <math xmlns="http://www.w3.org/1998/Math/MathML">
            <ci> FLUX_VALUE </ci>
          </math>
          <listOfParameters>
            <parameter id="LOWER_BOUND" value="0" units="mmol_per_gDW_per_hr" constant="true"/>
            <parameter id="UPPER_BOUND" value="1000" units="mmol_per_gDW_per_hr" constant="true"/>
            <parameter id="FLUX_VALUE" value="0" units="mmol_per_gDW_per_hr" constant="true"/>
            <parameter id="OBJECTIVE_COEFFICIENT" value="0" units="mmol_per_gDW_per_hr" constant="true"/>
          </listOfParameters>
        </kineticLaw>
      </reaction>
      <reaction id="R_R038" name="2-deoxy-D-ribose-5-phosphate acetaldehyde-lyase (D-glyceraldehyde-3-phosphate-forming)"
reversible="false">
        <notes>
          <body xmlns="http://www.w3.org/1999/xhtml">
            <p>GENE_ASSOCIATION: TTC0823</p>
            <p>SUBSYSTEM: Pentose Phosphate Pathway</p>
            <p>EC Number: 4.1.2.4</p>
            <p>Confidence Level: 1</p>
```

```
      <p>AUTHORS: Carbohydrate metabolism</p>
      <p/>
    </body>
  </notes>
  <listOfReactants>
    <speciesReference species="M_dR5P_c" stoichiometry="1"/>
  </listOfReactants>
  <listOfProducts>
    <speciesReference species="M_GAP_c" stoichiometry="1"/>
    <speciesReference species="M_AALD_c" stoichiometry="1"/>
  </listOfProducts>
  <kineticLaw>
    <math xmlns="http://www.w3.org/1998/Math/MathML">
      <ci> FLUX_VALUE </ci>
    </math>
    <listOfParameters>
      <parameter id="LOWER_BOUND" value="0" units="mmol_per_gDW_per_hr" constant="true"/>
      <parameter id="UPPER_BOUND" value="1000" units="mmol_per_gDW_per_hr" constant="true"/>
      <parameter id="FLUX_VALUE" value="0" units="mmol_per_gDW_per_hr" constant="true"/>
      <parameter id="OBJECTIVE_COEFFICIENT" value="0" units="mmol_per_gDW_per_hr" constant="true"/>
    </listOfParameters>
  </kineticLaw>
</reaction>
<reaction id="R_R039" name="2-deoxy-D-ribose 1-phosphate 1,5-phosphomutase" reversible="true">
  <notes>
    <body xmlns="http://www.w3.org/1999/xhtml">
      <p>GENE_ASSOCIATION: TTC1659</p>
      <p>SUBSYSTEM: Pentose Phosphate Pathway</p>
      <p>EC Number: 5.4.2.7</p>
```

```
      <p>Confidence Level: 1</p>
      <p>AUTHORS: Carbohydrate metabolism</p>
      <p/>
    </body>
  </notes>
  <listOfReactants>
    <speciesReference species="M_dR1P_c" stoichiometry="1"/>
  </listOfReactants>
  <listOfProducts>
    <speciesReference species="M_dR5P_c" stoichiometry="1"/>
  </listOfProducts>
  <kineticLaw>
    <math xmlns="http://www.w3.org/1998/Math/MathML">
      <ci> FLUX_VALUE </ci>
    </math>
    <listOfParameters>
      <parameter id="LOWER_BOUND" value="-1000" units="mmol_per_gDW_per_hr" constant="true"/>
      <parameter id="UPPER_BOUND" value="1000" units="mmol_per_gDW_per_hr" constant="true"/>
      <parameter id="FLUX_VALUE" value="0" units="mmol_per_gDW_per_hr" constant="true"/>
      <parameter id="OBJECTIVE_COEFFICIENT" value="0" units="mmol_per_gDW_per_hr" constant="true"/>
    </listOfParameters>
  </kineticLaw>
</reaction>
<reaction id="R_R040" name="glyceraldehyde ferredoxin oxidoreductase" reversible="true">
  <notes>
    <body xmlns="http://www.w3.org/1999/xhtml">
      <p>GENE_ASSOCIATION: TTC1834</p>
      <p>SUBSYSTEM: Pentose Phosphate Pathway</p>
      <p>EC Number: 1.2.7.5</p>
```

```
          <p>Confidence Level: 1</p>
          <p>AUTHORS: Carbohydrate metabolism</p>
          <p/>
        </body>
      </notes>
      <listOfReactants>
        <speciesReference species="M_H2O_c" stoichiometry="1"/>
        <speciesReference species="M_NAD_c" stoichiometry="1"/>
        <speciesReference species="M_GLYALD_c" stoichiometry="1"/>
      </listOfReactants>
      <listOfProducts>
        <speciesReference species="M_H_c" stoichiometry="2"/>
        <speciesReference species="M_NADH_c" stoichiometry="1"/>
        <speciesReference species="M_D_GLCT_c" stoichiometry="1"/>
      </listOfProducts>
      <kineticLaw>
        <math xmlns="http://www.w3.org/1998/Math/MathML">
          <ci> FLUX_VALUE </ci>
        </math>
        <listOfParameters>
          <parameter id="LOWER_BOUND" value="-1000" units="mmol_per_gDW_per_hr" constant="true"/>
          <parameter id="UPPER_BOUND" value="1000" units="mmol_per_gDW_per_hr" constant="true"/>
          <parameter id="FLUX_VALUE" value="0" units="mmol_per_gDW_per_hr" constant="true"/>
          <parameter id="OBJECTIVE_COEFFICIENT" value="0" units="mmol_per_gDW_per_hr" constant="true"/>
        </listOfParameters>
      </kineticLaw>
    </reaction>
    <reaction id="R_R041" name="2-dehydro-3-deoxy-D-gluconate-6-phosphate D-glyceraldehyde-3-phosphate-lyase"
reversible="false">
```

```
  <notes>
   <body xmlns="http://www.w3.org/1999/xhtml">
    <p>GENE_ASSOCIATION: TT_P0030</p>
    <p>SUBSYSTEM: Pentose Phosphate Pathway</p>
    <p>EC Number: 4.1.2.14</p>
    <p>Confidence Level: 1</p>
    <p>AUTHORS: Carbohydrate metabolism</p>
    <p/>
   </body>
  </notes>
  <listOfReactants>
   <speciesReference species="M_DHY_DOP_GLUCN_c" stoichiometry="1"/>
  </listOfReactants>
  <listOfProducts>
   <speciesReference species="M_GAP_c" stoichiometry="1"/>
   <speciesReference species="M_PYR_c" stoichiometry="1"/>
  </listOfProducts>
  <kineticLaw>
   <math xmlns="http://www.w3.org/1998/Math/MathML">
    <ci> FLUX_VALUE </ci>
   </math>
   <listOfParameters>
    <parameter id="LOWER_BOUND" value="0" units="mmol_per_gDW_per_hr" constant="true"/>
    <parameter id="UPPER_BOUND" value="1000" units="mmol_per_gDW_per_hr" constant="true"/>
    <parameter id="FLUX_VALUE" value="0" units="mmol_per_gDW_per_hr" constant="true"/>
    <parameter id="OBJECTIVE_COEFFICIENT" value="0" units="mmol_per_gDW_per_hr" constant="true"/>
   </listOfParameters>
  </kineticLaw>
 </reaction>
```

```xml
<reaction id="R_R042" name="2-Dehydro-3-deoxy-D-gluconate:NAD+ 5-oxidoreductase" reversible="true">
 <notes>
  <body xmlns="http://www.w3.org/1999/xhtml">
   <p>GENE_ASSOCIATION: TT_P0037</p>
   <p>SUBSYSTEM: Pentose Phosphate Pathway</p>
   <p>EC Number: 1.1.1.125</p>
   <p>Confidence Level: 1</p>
   <p>AUTHORS: Carbohydrate metabolism</p>
   <p/>
  </body>
 </notes>
 <listOfReactants>
  <speciesReference species="M_NAD_c" stoichiometry="1"/>
  <speciesReference species="M_2K3DO_GLCN_c" stoichiometry="1"/>
 </listOfReactants>
 <listOfProducts>
  <speciesReference species="M_H_c" stoichiometry="1"/>
  <speciesReference species="M_NADH_c" stoichiometry="1"/>
  <speciesReference species="M_DHY_DOHEXNA_c" stoichiometry="1"/>
 </listOfProducts>
 <kineticLaw>
  <math xmlns="http://www.w3.org/1998/Math/MathML">
   <ci> FLUX_VALUE </ci>
  </math>
  <listOfParameters>
   <parameter id="LOWER_BOUND" value="-1000" units="mmol_per_gDW_per_hr" constant="true"/>
   <parameter id="UPPER_BOUND" value="1000" units="mmol_per_gDW_per_hr" constant="true"/>
   <parameter id="FLUX_VALUE" value="0" units="mmol_per_gDW_per_hr" constant="true"/>
   <parameter id="OBJECTIVE_COEFFICIENT" value="0" units="mmol_per_gDW_per_hr" constant="true"/>
```

```
    </listOfParameters>
  </kineticLaw>
</reaction>
<reaction id="R_R043" name="D-fructose 1-phosphate D-glyceraldehyde-3-phosphate-lyase" reversible="true">
  <notes>
    <body xmlns="http://www.w3.org/1999/xhtml">
      <p>GENE_ASSOCIATION: TTC1414</p>
      <p>SUBSYSTEM: Alternate Carbon Metabolism</p>
      <p>EC Number: 4.1.2.13</p>
      <p>Confidence Level: 1</p>
      <p>AUTHORS: Carbohydrate metabolism</p>
      <p/>
    </body>
  </notes>
  <listOfReactants>
    <speciesReference species="M_F1P_c" stoichiometry="1"/>
  </listOfReactants>
  <listOfProducts>
    <speciesReference species="M_DHAP_c" stoichiometry="1"/>
    <speciesReference species="M_GLYALD_c" stoichiometry="1"/>
  </listOfProducts>
  <kineticLaw>
    <math xmlns="http://www.w3.org/1998/Math/MathML">
      <ci> FLUX_VALUE </ci>
    </math>
    <listOfParameters>
      <parameter id="LOWER_BOUND" value="-1000" units="mmol_per_gDW_per_hr" constant="true"/>
      <parameter id="UPPER_BOUND" value="1000" units="mmol_per_gDW_per_hr" constant="true"/>
      <parameter id="FLUX_VALUE" value="0" units="mmol_per_gDW_per_hr" constant="true"/>
```

```xml
<parameter id="OBJECTIVE_COEFFICIENT" value="0" units="mmol_per_gDW_per_hr" constant="true"/>
</listOfParameters>
</kineticLaw>
</reaction>
<reaction id="R_R044" name="L-Fuculose-1-phosphate lactaldehyde-lyase" reversible="true">
<notes>
<body xmlns="http://www.w3.org/1999/xhtml">
<p>GENE_ASSOCIATION: TTC1459</p>
<p>SUBSYSTEM: Alternate Carbon Metabolism</p>
<p>EC Number: 4.1.2.17</p>
<p>Confidence Level: 1</p>
<p>AUTHORS: Carbohydrate metabolism</p>
<p/>
</body>
</notes>
<listOfReactants>
<speciesReference species="M_FUCL1P_c" stoichiometry="1"/>
</listOfReactants>
<listOfProducts>
<speciesReference species="M_DHAP_c" stoichiometry="1"/>
<speciesReference species="M_LALD_c" stoichiometry="1"/>
</listOfProducts>
<kineticLaw>
<math xmlns="http://www.w3.org/1998/Math/MathML">
<ci> FLUX_VALUE </ci>
</math>
<listOfParameters>
<parameter id="LOWER_BOUND" value="-1000" units="mmol_per_gDW_per_hr" constant="true"/>
<parameter id="UPPER_BOUND" value="1000" units="mmol_per_gDW_per_hr" constant="true"/>
```

```
      <parameter id="FLUX_VALUE" value="0" units="mmol_per_gDW_per_hr" constant="true"/>
      <parameter id="OBJECTIVE_COEFFICIENT" value="0" units="mmol_per_gDW_per_hr" constant="true"/>
    </listOfParameters>
  </kineticLaw>
</reaction>
<reaction id="R_R045" name="L-lactaldehyde:NAD+ oxidoreductase" reversible="true">
  <notes>
    <body xmlns="http://www.w3.org/1999/xhtml">
      <p>GENE_ASSOCIATION: TTC0513</p>
      <p>SUBSYSTEM: Alternate Carbon Metabolism</p>
      <p>EC Number: 1.2.1.22</p>
      <p>Confidence Level: 1</p>
      <p>AUTHORS: Carbohydrate metabolism</p>
      <p/>
    </body>
  </notes>
  <listOfReactants>
    <speciesReference species="M_H2O_c" stoichiometry="1"/>
    <speciesReference species="M_NAD_c" stoichiometry="1"/>
    <speciesReference species="M_LALD_c" stoichiometry="1"/>
  </listOfReactants>
  <listOfProducts>
    <speciesReference species="M_H_c" stoichiometry="2"/>
    <speciesReference species="M_NADH_c" stoichiometry="1"/>
    <speciesReference species="M_LAC_c" stoichiometry="1"/>
  </listOfProducts>
  <kineticLaw>
    <math xmlns="http://www.w3.org/1998/Math/MathML">
      <ci> FLUX_VALUE </ci>
```

```
</math>
<listOfParameters>
<parameter id="LOWER_BOUND" value="-1000" units="mmol_per_gDW_per_hr" constant="true"/>
<parameter id="UPPER_BOUND" value="1000" units="mmol_per_gDW_per_hr" constant="true"/>
<parameter id="FLUX_VALUE" value="0" units="mmol_per_gDW_per_hr" constant="true"/>
<parameter id="OBJECTIVE_COEFFICIENT" value="0" units="mmol_per_gDW_per_hr" constant="true"/>
</listOfParameters>
</kineticLaw>
</reaction>
<reaction id="R_R046" name="GDP-mannose:3-phosphoglycerate 3-a-D-mannosyltransferase" reversible="true">
<notes>
<body xmlns="http://www.w3.org/1999/xhtml">
<p>GENE_ASSOCIATION: TTC0588</p>
<p>SUBSYSTEM: Alternate Carbon Metabolism</p>
<p>EC Number: 2.4.1.217</p>
<p>Confidence Level: 1</p>
<p>AUTHORS: Carbohydrate metabolism</p>
<p/>
</body>
</notes>
<listOfReactants>
<speciesReference species="M_3PG_c" stoichiometry="1"/>
<speciesReference species="M_GDP_MAN_c" stoichiometry="1"/>
</listOfReactants>
<listOfProducts>
<speciesReference species="M_GDP_c" stoichiometry="1"/>
<speciesReference species="M_MANNPGLYCT_c" stoichiometry="1"/>
</listOfProducts>
<kineticLaw>
```

```
    <math xmlns="http://www.w3.org/1998/Math/MathML">
      <ci> FLUX_VALUE </ci>
    </math>
    <listOfParameters>
      <parameter id="LOWER_BOUND" value="-1000" units="mmol_per_gDW_per_hr" constant="true"/>
      <parameter id="UPPER_BOUND" value="1000" units="mmol_per_gDW_per_hr" constant="true"/>
      <parameter id="FLUX_VALUE" value="0" units="mmol_per_gDW_per_hr" constant="true"/>
      <parameter id="OBJECTIVE_COEFFICIENT" value="0" units="mmol_per_gDW_per_hr" constant="true"/>
    </listOfParameters>
  </kineticLaw>
</reaction>
<reaction id="R_R047" name="alpha-D-mannosyl-3-phosphoglycerate phosphohydrolase" reversible="true">
  <notes>
    <body xmlns="http://www.w3.org/1999/xhtml">
      <p>GENE_ASSOCIATION: TTC0589</p>
      <p>SUBSYSTEM: Alternate Carbon Metabolism</p>
      <p>EC Number: 3.1.3.70</p>
      <p>Confidence Level: 1</p>
      <p>AUTHORS: Carbohydrate metabolism</p>
      <p/>
    </body>
  </notes>
  <listOfReactants>
    <speciesReference species="M_H2O_c" stoichiometry="1"/>
    <speciesReference species="M_MANNPGLYCT_c" stoichiometry="1"/>
  </listOfReactants>
  <listOfProducts>
    <speciesReference species="M_Pi_c" stoichiometry="1"/>
    <speciesReference species="M_AMANN_GLYCT_c" stoichiometry="1"/>
```

```
    </listOfProducts>
    <kineticLaw>
      <math xmlns="http://www.w3.org/1998/Math/MathML">
        <ci> FLUX_VALUE </ci>
      </math>
      <listOfParameters>
        <parameter id="LOWER_BOUND" value="-1000" units="mmol_per_gDW_per_hr" constant="true"/>
        <parameter id="UPPER_BOUND" value="1000" units="mmol_per_gDW_per_hr" constant="true"/>
        <parameter id="FLUX_VALUE" value="0" units="mmol_per_gDW_per_hr" constant="true"/>
        <parameter id="OBJECTIVE_COEFFICIENT" value="0" units="mmol_per_gDW_per_hr" constant="true"/>
      </listOfParameters>
    </kineticLaw>
  </reaction>
  <reaction id="R_R048" name="D-mannose 6-phosphate 1,6-phosphomutase" reversible="true">
    <notes>
      <body xmlns="http://www.w3.org/1999/xhtml">
        <p>GENE_ASSOCIATION: (TTC1630 or TTC0291 or TTC1063)</p>
        <p>SUBSYSTEM: Alternate Carbon Metabolism</p>
        <p>EC Number: 5.4.2.8</p>
        <p>Confidence Level: 1</p>
        <p>AUTHORS: Carbohydrate metabolism</p>
        <p/>
      </body>
    </notes>
    <listOfReactants>
      <speciesReference species="M_M6P_c" stoichiometry="1"/>
    </listOfReactants>
    <listOfProducts>
      <speciesReference species="M_MAN1P_c" stoichiometry="1"/>
```

```
  </listOfProducts>
  <kineticLaw>
    <math xmlns="http://www.w3.org/1998/Math/MathML">
      <ci> FLUX_VALUE </ci>
    </math>
    <listOfParameters>
      <parameter id="LOWER_BOUND" value="-1000" units="mmol_per_gDW_per_hr" constant="true"/>
      <parameter id="UPPER_BOUND" value="1000" units="mmol_per_gDW_per_hr" constant="true"/>
      <parameter id="FLUX_VALUE" value="0" units="mmol_per_gDW_per_hr" constant="true"/>
      <parameter id="OBJECTIVE_COEFFICIENT" value="0" units="mmol_per_gDW_per_hr" constant="true"/>
    </listOfParameters>
  </kineticLaw>
</reaction>
<reaction id="R_R049" name="GDP:D-mannose-1-phosphate guanylyltransferase" reversible="false">
  <notes>
    <body xmlns="http://www.w3.org/1999/xhtml">
      <p>GENE_ASSOCIATION: TTC1388</p>
      <p>SUBSYSTEM: Alternate Carbon Metabolism</p>
      <p>EC Number: 2.7.7.22</p>
      <p>Confidence Level: 1</p>
      <p>AUTHORS: Carbohydrate metabolism</p>
      <p/>
    </body>
  </notes>
  <listOfReactants>
    <speciesReference species="M_H_c" stoichiometry="1"/>
    <speciesReference species="M_GDP_c" stoichiometry="1"/>
    <speciesReference species="M_MAN1P_c" stoichiometry="1"/>
  </listOfReactants>
```

```
<listOfProducts>
  <speciesReference species="M_Pi_c" stoichiometry="1"/>
  <speciesReference species="M_GDP_MAN_c" stoichiometry="1"/>
</listOfProducts>
<kineticLaw>
  <math xmlns="http://www.w3.org/1998/Math/MathML">
    <ci> FLUX_VALUE </ci>
  </math>
  <listOfParameters>
    <parameter id="LOWER_BOUND" value="0" units="mmol_per_gDW_per_hr" constant="true"/>
    <parameter id="UPPER_BOUND" value="1000" units="mmol_per_gDW_per_hr" constant="true"/>
    <parameter id="FLUX_VALUE" value="0" units="mmol_per_gDW_per_hr" constant="true"/>
    <parameter id="OBJECTIVE_COEFFICIENT" value="0" units="mmol_per_gDW_per_hr" constant="true"/>
  </listOfParameters>
</kineticLaw>
</reaction>
<reaction id="R_R050" name="ATP:D-fructose 6-phosphotransferase" reversible="false">
<notes>
  <body xmlns="http://www.w3.org/1999/xhtml">
    <p>GENE_ASSOCIATION: TTC0630</p>
    <p>SUBSYSTEM: Alternate Carbon Metabolism</p>
    <p>EC Number: 2.7.1.4</p>
    <p>Confidence Level: 1</p>
    <p>AUTHORS: Carbohydrate metabolism</p>
    <p/>
  </body>
</notes>
<listOfReactants>
  <speciesReference species="M_ATP_c" stoichiometry="1"/>
```

```
    <speciesReference species="M_D_FRU_c" stoichiometry="1"/>
  </listOfReactants>
  <listOfProducts>
    <speciesReference species="M_ADP_c" stoichiometry="1"/>
    <speciesReference species="M_H_c" stoichiometry="1"/>
    <speciesReference species="M_F6P_c" stoichiometry="1"/>
  </listOfProducts>
  <kineticLaw>
    <math xmlns="http://www.w3.org/1998/Math/MathML">
      <ci> FLUX_VALUE </ci>
    </math>
    <listOfParameters>
      <parameter id="LOWER_BOUND" value="0" units="mmol_per_gDW_per_hr" constant="true"/>
      <parameter id="UPPER_BOUND" value="1000" units="mmol_per_gDW_per_hr" constant="true"/>
      <parameter id="FLUX_VALUE" value="0" units="mmol_per_gDW_per_hr" constant="true"/>
      <parameter id="OBJECTIVE_COEFFICIENT" value="0" units="mmol_per_gDW_per_hr" constant="true"/>
    </listOfParameters>
  </kineticLaw>
</reaction>
<reaction id="R_R051" name="D-mannose-6-phosphate aldose-ketose-isomerase" reversible="true">
  <notes>
    <body xmlns="http://www.w3.org/1999/xhtml">
      <p>GENE_ASSOCIATION: TTC0980</p>
      <p>SUBSYSTEM: Alternate Carbon Metabolism</p>
      <p>EC Number: 5.3.1.8</p>
      <p>Confidence Level: 1</p>
      <p>AUTHORS: Carbohydrate metabolism</p>
      <p/>
    </body>
```

```
  </notes>
  <listOfReactants>
    <speciesReference species="M_M6P_c" stoichiometry="1"/>
  </listOfReactants>
  <listOfProducts>
    <speciesReference species="M_F6P_c" stoichiometry="1"/>
  </listOfProducts>
  <kineticLaw>
    <math xmlns="http://www.w3.org/1998/Math/MathML">
      <ci> FLUX_VALUE </ci>
    </math>
    <listOfParameters>
      <parameter id="LOWER_BOUND" value="-1000" units="mmol_per_gDW_per_hr" constant="true"/>
      <parameter id="UPPER_BOUND" value="1000" units="mmol_per_gDW_per_hr" constant="true"/>
      <parameter id="FLUX_VALUE" value="0" units="mmol_per_gDW_per_hr" constant="true"/>
      <parameter id="OBJECTIVE_COEFFICIENT" value="0" units="mmol_per_gDW_per_hr" constant="true"/>
    </listOfParameters>
  </kineticLaw>
</reaction>
<reaction id="R_R052" name="ATP:D-galactose 1-phosphotransferase" reversible="true">
  <notes>
    <body xmlns="http://www.w3.org/1999/xhtml">
      <p>GENE_ASSOCIATION: TTC0226</p>
      <p>SUBSYSTEM: Alternate Carbon Metabolism</p>
      <p>EC Number: 2.7.1.6</p>
      <p>Confidence Level: 1</p>
      <p>AUTHORS: Carbohydrate metabolism</p>
      <p/>
    </body>
```

```xml
  </notes>
  <listOfReactants>
    <speciesReference species="M_ATP_c" stoichiometry="1"/>
    <speciesReference species="M_D_GAL_c" stoichiometry="1"/>
  </listOfReactants>
  <listOfProducts>
    <speciesReference species="M_ADP_c" stoichiometry="1"/>
    <speciesReference species="M_H_c" stoichiometry="1"/>
    <speciesReference species="M_GAL1P_c" stoichiometry="1"/>
  </listOfProducts>
  <kineticLaw>
    <math xmlns="http://www.w3.org/1998/Math/MathML">
      <ci> FLUX_VALUE </ci>
    </math>
    <listOfParameters>
      <parameter id="LOWER_BOUND" value="-1000" units="mmol_per_gDW_per_hr" constant="true"/>
      <parameter id="UPPER_BOUND" value="1000" units="mmol_per_gDW_per_hr" constant="true"/>
      <parameter id="FLUX_VALUE" value="0" units="mmol_per_gDW_per_hr" constant="true"/>
      <parameter id="OBJECTIVE_COEFFICIENT" value="0" units="mmol_per_gDW_per_hr" constant="true"/>
    </listOfParameters>
  </kineticLaw>
</reaction>
<reaction id="R_R053" name="UDP-glucose:alpha-D-galactose-1-phosphate uridylyltransferase" reversible="true">
  <notes>
    <body xmlns="http://www.w3.org/1999/xhtml">
      <p>GENE_ASSOCIATION: TT_P0071</p>
      <p>SUBSYSTEM: Alternate Carbon Metabolism</p>
      <p>EC Number: 2.7.7.12</p>
      <p>Confidence Level: 1</p>
```

```
      <p>AUTHORS: Carbohydrate metabolism</p>
      <p/>
    </body>
  </notes>
  <listOfReactants>
    <speciesReference species="M_GAL1P_c" stoichiometry="1"/>
    <speciesReference species="M_UDP_GLU_c" stoichiometry="1"/>
  </listOfReactants>
  <listOfProducts>
    <speciesReference species="M_G1P_c" stoichiometry="1"/>
    <speciesReference species="M_UDP_GAL_c" stoichiometry="1"/>
  </listOfProducts>
  <kineticLaw>
    <math xmlns="http://www.w3.org/1998/Math/MathML">
      <ci> FLUX_VALUE </ci>
    </math>
    <listOfParameters>
      <parameter id="LOWER_BOUND" value="-1000" units="mmol_per_gDW_per_hr" constant="true"/>
      <parameter id="UPPER_BOUND" value="1000" units="mmol_per_gDW_per_hr" constant="true"/>
      <parameter id="FLUX_VALUE" value="0" units="mmol_per_gDW_per_hr" constant="true"/>
      <parameter id="OBJECTIVE_COEFFICIENT" value="0" units="mmol_per_gDW_per_hr" constant="true"/>
    </listOfParameters>
  </kineticLaw>
</reaction>
<reaction id="R_R054" name="UDP-glucose 4-epimerase" reversible="true">
  <notes>
    <body xmlns="http://www.w3.org/1999/xhtml">
      <p>GENE_ASSOCIATION: TTC0222</p>
      <p>SUBSYSTEM: Alternate Carbon Metabolism</p>
```

```
      <p>EC Number: 5.1.3.2</p>
      <p>Confidence Level: 1</p>
      <p>AUTHORS: Carbohydrate metabolism</p>
      <p/>
    </body>
  </notes>
  <listOfReactants>
    <speciesReference species="M_UDP_GLU_c" stoichiometry="1"/>
  </listOfReactants>
  <listOfProducts>
    <speciesReference species="M_UDP_GAL_c" stoichiometry="1"/>
  </listOfProducts>
  <kineticLaw>
    <math xmlns="http://www.w3.org/1998/Math/MathML">
      <ci> FLUX_VALUE </ci>
    </math>
    <listOfParameters>
      <parameter id="LOWER_BOUND" value="-1000" units="mmol_per_gDW_per_hr" constant="true"/>
      <parameter id="UPPER_BOUND" value="1000" units="mmol_per_gDW_per_hr" constant="true"/>
      <parameter id="FLUX_VALUE" value="0" units="mmol_per_gDW_per_hr" constant="true"/>
      <parameter id="OBJECTIVE_COEFFICIENT" value="0" units="mmol_per_gDW_per_hr" constant="true"/>
    </listOfParameters>
  </kineticLaw>
</reaction>
<reaction id="R_R055" name="Galactan galactohydrolase" reversible="true">
  <notes>
    <body xmlns="http://www.w3.org/1999/xhtml">
      <p>GENE_ASSOCIATION: (TT_P0220 or TT_P0222)</p>
      <p>SUBSYSTEM: Alternate Carbon Metabolism</p>
```

```
      <p>EC Number: 3.2.1.23</p>
      <p>Confidence Level: 1</p>
      <p>AUTHORS: Carbohydrate metabolism</p>
      <p/>
    </body>
  </notes>
  <listOfReactants>
    <speciesReference species="M_H2O_c" stoichiometry="1"/>
    <speciesReference species="M_GLCTN_c" stoichiometry="1"/>
  </listOfReactants>
  <listOfProducts>
    <speciesReference species="M_D_GAL_c" stoichiometry="2"/>
  </listOfProducts>
  <kineticLaw>
    <math xmlns="http://www.w3.org/1998/Math/MathML">
      <ci> FLUX_VALUE </ci>
    </math>
    <listOfParameters>
      <parameter id="LOWER_BOUND" value="-1000" units="mmol_per_gDW_per_hr" constant="true"/>
      <parameter id="UPPER_BOUND" value="1000" units="mmol_per_gDW_per_hr" constant="true"/>
      <parameter id="FLUX_VALUE" value="0" units="mmol_per_gDW_per_hr" constant="true"/>
      <parameter id="OBJECTIVE_COEFFICIENT" value="0" units="mmol_per_gDW_per_hr" constant="true"/>
    </listOfParameters>
  </kineticLaw>
</reaction>
<reaction id="R_R056" name="Lactose galactohydrolase" reversible="false">
  <notes>
    <body xmlns="http://www.w3.org/1999/xhtml">
      <p>GENE_ASSOCIATION: (TT_P0220 or TT_P0222)</p>
```

```xml
      <p>SUBSYSTEM: Alternate Carbon Metabolism</p>
      <p>EC Number: 3.2.1.23</p>
      <p>Confidence Level: 1</p>
      <p>AUTHORS: Carbohydrate metabolism</p>
      <p/>
    </body>
  </notes>
  <listOfReactants>
    <speciesReference species="M_H2O_c" stoichiometry="1"/>
    <speciesReference species="M_LACTS_c" stoichiometry="1"/>
  </listOfReactants>
  <listOfProducts>
    <speciesReference species="M_D_GLC_c" stoichiometry="1"/>
    <speciesReference species="M_D_GAL_c" stoichiometry="1"/>
  </listOfProducts>
  <kineticLaw>
    <math xmlns="http://www.w3.org/1998/Math/MathML">
      <ci> FLUX_VALUE </ci>
    </math>
    <listOfParameters>
      <parameter id="LOWER_BOUND" value="0" units="mmol_per_gDW_per_hr" constant="true"/>
      <parameter id="UPPER_BOUND" value="1000" units="mmol_per_gDW_per_hr" constant="true"/>
      <parameter id="FLUX_VALUE" value="0" units="mmol_per_gDW_per_hr" constant="true"/>
      <parameter id="OBJECTIVE_COEFFICIENT" value="0" units="mmol_per_gDW_per_hr" constant="true"/>
    </listOfParameters>
  </kineticLaw>
</reaction>
<reaction id="R_R057" name="alpha-D-glucose 1,6-phosphomutase" reversible="true">
  <notes>
```

```xml
<body xmlns="http://www.w3.org/1999/xhtml">
<p>GENE_ASSOCIATION: (TTC1630 or TTC0291 or TTC1063)</p>
<p>SUBSYSTEM: Alternate Carbon Metabolism</p>
<p>EC Number: 5.4.2.2</p>
<p>Confidence Level: 1</p>
<p>AUTHORS: Carbohydrate metabolism</p>
<p/>
</body>
</notes>
<listOfReactants>
<speciesReference species="M_G1P_c" stoichiometry="1"/>
</listOfReactants>
<listOfProducts>
<speciesReference species="M_G6P_c" stoichiometry="1"/>
</listOfProducts>
<kineticLaw>
<math xmlns="http://www.w3.org/1998/Math/MathML">
<ci> FLUX_VALUE </ci>
</math>
<listOfParameters>
<parameter id="LOWER_BOUND" value="-1000" units="mmol_per_gDW_per_hr" constant="true"/>
<parameter id="UPPER_BOUND" value="1000" units="mmol_per_gDW_per_hr" constant="true"/>
<parameter id="FLUX_VALUE" value="0" units="mmol_per_gDW_per_hr" constant="true"/>
<parameter id="OBJECTIVE_COEFFICIENT" value="0" units="mmol_per_gDW_per_hr" constant="true"/>
</listOfParameters>
</kineticLaw>
</reaction>
<reaction id="R_R058" name="Epimelibiose galactohydrolase" reversible="true">
<notes>
```

```
    <body xmlns="http://www.w3.org/1999/xhtml">
      <p>GENE_ASSOCIATION: TT_P0072</p>
      <p>SUBSYSTEM: Alternate Carbon Metabolism</p>
      <p>EC Number: 3.2.1.22</p>
      <p>Confidence Level: 1</p>
      <p>AUTHORS: Carbohydrate metabolism</p>
      <p/>
    </body>
  </notes>
  <listOfReactants>
    <speciesReference species="M_H2O_c" stoichiometry="1"/>
    <speciesReference species="M_EPMLBIOS_c" stoichiometry="1"/>
  </listOfReactants>
  <listOfProducts>
    <speciesReference species="M_D_MAN_c" stoichiometry="1"/>
    <speciesReference species="M_D_GAL_c" stoichiometry="1"/>
  </listOfProducts>
  <kineticLaw>
    <math xmlns="http://www.w3.org/1998/Math/MathML">
      <ci> FLUX_VALUE </ci>
    </math>
    <listOfParameters>
      <parameter id="LOWER_BOUND" value="-1000" units="mmol_per_gDW_per_hr" constant="true"/>
      <parameter id="UPPER_BOUND" value="1000" units="mmol_per_gDW_per_hr" constant="true"/>
      <parameter id="FLUX_VALUE" value="0" units="mmol_per_gDW_per_hr" constant="true"/>
      <parameter id="OBJECTIVE_COEFFICIENT" value="0" units="mmol_per_gDW_per_hr" constant="true"/>
    </listOfParameters>
  </kineticLaw>
</reaction>
```

```
<reaction id="R_R059" name="3-O-alpha-D-Galactosyl-1D-myo-inositol galactohydrolase" reversible="true">
 <notes>
  <body xmlns="http://www.w3.org/1999/xhtml">
   <p>GENE_ASSOCIATION: TT_P0072</p>
   <p>SUBSYSTEM: Alternate Carbon Metabolism</p>
   <p>EC Number: 3.2.1.22</p>
   <p>Confidence Level: 1</p>
   <p>AUTHORS: Carbohydrate metabolism</p>
   <p/>
  </body>
 </notes>
 <listOfReactants>
  <speciesReference species="M_H2O_c" stoichiometry="1"/>
  <speciesReference species="M_AGAL_INS_c" stoichiometry="1"/>
 </listOfReactants>
 <listOfProducts>
  <speciesReference species="M_D_GAL_c" stoichiometry="1"/>
  <speciesReference species="M_M_INS_c" stoichiometry="1"/>
 </listOfProducts>
 <kineticLaw>
  <math xmlns="http://www.w3.org/1998/Math/MathML">
   <ci> FLUX_VALUE </ci>
  </math>
  <listOfParameters>
   <parameter id="LOWER_BOUND" value="-1000" units="mmol_per_gDW_per_hr" constant="true"/>
   <parameter id="UPPER_BOUND" value="1000" units="mmol_per_gDW_per_hr" constant="true"/>
   <parameter id="FLUX_VALUE" value="0" units="mmol_per_gDW_per_hr" constant="true"/>
   <parameter id="OBJECTIVE_COEFFICIENT" value="0" units="mmol_per_gDW_per_hr" constant="true"/>
  </listOfParameters>
```

```
  </kineticLaw>
</reaction>
<reaction id="R_R060" name="Melibiitol galactohydrolase" reversible="true">
  <notes>
    <body xmlns="http://www.w3.org/1999/xhtml">
      <p>GENE_ASSOCIATION: TT_P0072</p>
      <p>SUBSYSTEM: Alternate Carbon Metabolism</p>
      <p>EC Number: 3.2.1.22</p>
      <p>Confidence Level: 1</p>
      <p>AUTHORS: Carbohydrate metabolism</p>
      <p/>
    </body>
  </notes>
  <listOfReactants>
    <speciesReference species="M_H2O_c" stoichiometry="1"/>
    <speciesReference species="M_MLBTL_c" stoichiometry="1"/>
  </listOfReactants>
  <listOfProducts>
    <speciesReference species="M_D_GAL_c" stoichiometry="1"/>
    <speciesReference species="M_SORB_c" stoichiometry="1"/>
  </listOfProducts>
  <kineticLaw>
    <math xmlns="http://www.w3.org/1998/Math/MathML">
      <ci> FLUX_VALUE </ci>
    </math>
    <listOfParameters>
      <parameter id="LOWER_BOUND" value="-1000" units="mmol_per_gDW_per_hr" constant="true"/>
      <parameter id="UPPER_BOUND" value="1000" units="mmol_per_gDW_per_hr" constant="true"/>
      <parameter id="FLUX_VALUE" value="0" units="mmol_per_gDW_per_hr" constant="true"/>
```

```
      <parameter id="OBJECTIVE_COEFFICIENT" value="0" units="mmol_per_gDW_per_hr" constant="true"/>
    </listOfParameters>
  </kineticLaw>
</reaction>
<reaction id="R_R061" name="Galactosylglycerol galactohydrolase" reversible="true">
  <notes>
    <body xmlns="http://www.w3.org/1999/xhtml">
      <p>GENE_ASSOCIATION: TT_P0072</p>
      <p>SUBSYSTEM: Alternate Carbon Metabolism</p>
      <p>EC Number: 3.2.1.22</p>
      <p>Confidence Level: 1</p>
      <p>AUTHORS: Carbohydrate metabolism</p>
      <p/>
    </body>
  </notes>
  <listOfReactants>
    <speciesReference species="M_H2O_c" stoichiometry="1"/>
    <speciesReference species="M_GALCS_GLYC_c" stoichiometry="1"/>
  </listOfReactants>
  <listOfProducts>
    <speciesReference species="M_D_GAL_c" stoichiometry="1"/>
    <speciesReference species="M_GLYCRL_c" stoichiometry="1"/>
  </listOfProducts>
  <kineticLaw>
    <math xmlns="http://www.w3.org/1998/Math/MathML">
      <ci> FLUX_VALUE </ci>
    </math>
    <listOfParameters>
      <parameter id="LOWER_BOUND" value="-1000" units="mmol_per_gDW_per_hr" constant="true"/>
```

```
      <parameter id="UPPER_BOUND" value="1000" units="mmol_per_gDW_per_hr" constant="true"/>
      <parameter id="FLUX_VALUE" value="0" units="mmol_per_gDW_per_hr" constant="true"/>
      <parameter id="OBJECTIVE_COEFFICIENT" value="0" units="mmol_per_gDW_per_hr" constant="true"/>
    </listOfParameters>
  </kineticLaw>
</reaction>
<reaction id="R_R062" name="alkaline alpha-galactosidase" reversible="true">
  <notes>
    <body xmlns="http://www.w3.org/1999/xhtml">
      <p>GENE_ASSOCIATION: TT_P0072</p>
      <p>SUBSYSTEM: Alternate Carbon Metabolism</p>
      <p>EC Number: 3.2.1.22</p>
      <p>Confidence Level: 1</p>
      <p>AUTHORS: Carbohydrate metabolism</p>
      <p/>
    </body>
  </notes>
  <listOfReactants>
    <speciesReference species="M_H2O_c" stoichiometry="1"/>
    <speciesReference species="M_STCYS_c" stoichiometry="1"/>
  </listOfReactants>
  <listOfProducts>
    <speciesReference species="M_D_GAL_c" stoichiometry="1"/>
    <speciesReference species="M_RAFF_c" stoichiometry="1"/>
  </listOfProducts>
  <kineticLaw>
    <math xmlns="http://www.w3.org/1998/Math/MathML">
      <ci> FLUX_VALUE </ci>
    </math>
```

```
    <listOfParameters>
      <parameter id="LOWER_BOUND" value="-1000" units="mmol_per_gDW_per_hr" constant="true"/>
      <parameter id="UPPER_BOUND" value="1000" units="mmol_per_gDW_per_hr" constant="true"/>
      <parameter id="FLUX_VALUE" value="0" units="mmol_per_gDW_per_hr" constant="true"/>
      <parameter id="OBJECTIVE_COEFFICIENT" value="0" units="mmol_per_gDW_per_hr" constant="true"/>
    </listOfParameters>
  </kineticLaw>
</reaction>
<reaction id="R_R063" name="Manninotriose galactohydrolase" reversible="true">
  <notes>
    <body xmlns="http://www.w3.org/1999/xhtml">
      <p>GENE_ASSOCIATION: TT_P0072</p>
      <p>SUBSYSTEM: Alternate Carbon Metabolism</p>
      <p>EC Number: 3.2.1.22</p>
      <p>Confidence Level: 1</p>
      <p>AUTHORS: Carbohydrate metabolism</p>
      <p/>
    </body>
  </notes>
  <listOfReactants>
    <speciesReference species="M_H2O_c" stoichiometry="1"/>
    <speciesReference species="M_MANNTRIS_c" stoichiometry="1"/>
  </listOfReactants>
  <listOfProducts>
    <speciesReference species="M_D_GAL_c" stoichiometry="1"/>
    <speciesReference species="M_MLBIOS_c" stoichiometry="1"/>
  </listOfProducts>
  <kineticLaw>
    <math xmlns="http://www.w3.org/1998/Math/MathML">
```

```xml
      <ci> FLUX_VALUE </ci>
    </math>
    <listOfParameters>
      <parameter id="LOWER_BOUND" value="-1000" units="mmol_per_gDW_per_hr" constant="true"/>
      <parameter id="UPPER_BOUND" value="1000" units="mmol_per_gDW_per_hr" constant="true"/>
      <parameter id="FLUX_VALUE" value="0" units="mmol_per_gDW_per_hr" constant="true"/>
      <parameter id="OBJECTIVE_COEFFICIENT" value="0" units="mmol_per_gDW_per_hr" constant="true"/>
    </listOfParameters>
  </kineticLaw>
</reaction>
<reaction id="R_R064" name="Raffinose galactohydrolase" reversible="true">
  <notes>
    <body xmlns="http://www.w3.org/1999/xhtml">
      <p>GENE_ASSOCIATION: TT_P0072</p>
      <p>SUBSYSTEM: Alternate Carbon Metabolism</p>
      <p>EC Number: 3.2.1.22</p>
      <p>Confidence Level: 1</p>
      <p>AUTHORS: Carbohydrate metabolism</p>
      <p/>
    </body>
  </notes>
  <listOfReactants>
    <speciesReference species="M_H2O_c" stoichiometry="1"/>
    <speciesReference species="M_RAFF_c" stoichiometry="1"/>
  </listOfReactants>
  <listOfProducts>
    <speciesReference species="M_D_GAL_c" stoichiometry="1"/>
    <speciesReference species="M_SUC_c" stoichiometry="1"/>
  </listOfProducts>
```

```
  <kineticLaw>
    <math xmlns="http://www.w3.org/1998/Math/MathML">
      <ci> FLUX_VALUE </ci>
    </math>
    <listOfParameters>
      <parameter id="LOWER_BOUND" value="-1000" units="mmol_per_gDW_per_hr" constant="true"/>
      <parameter id="UPPER_BOUND" value="1000" units="mmol_per_gDW_per_hr" constant="true"/>
      <parameter id="FLUX_VALUE" value="0" units="mmol_per_gDW_per_hr" constant="true"/>
      <parameter id="OBJECTIVE_COEFFICIENT" value="0" units="mmol_per_gDW_per_hr" constant="true"/>
    </listOfParameters>
  </kineticLaw>
</reaction>
<reaction id="R_R065" name="melibiose galactohydrolase" reversible="true">
  <notes>
    <body xmlns="http://www.w3.org/1999/xhtml">
      <p>GENE_ASSOCIATION: TT_P0072</p>
      <p>SUBSYSTEM: Alternate Carbon Metabolism</p>
      <p>EC Number: 3.2.1.22</p>
      <p>Confidence Level: 1</p>
      <p>AUTHORS: Carbohydrate metabolism</p>
      <p/>
    </body>
  </notes>
  <listOfReactants>
    <speciesReference species="M_H2O_c" stoichiometry="1"/>
    <speciesReference species="M_MLBIOS_c" stoichiometry="1"/>
  </listOfReactants>
  <listOfProducts>
    <speciesReference species="M_D_GLC_c" stoichiometry="1"/>
```

```
    <speciesReference species="M_D_GAL_c" stoichiometry="1"/>
  </listOfProducts>
  <kineticLaw>
    <math xmlns="http://www.w3.org/1998/Math/MathML">
      <ci> FLUX_VALUE </ci>
    </math>
    <listOfParameters>
      <parameter id="LOWER_BOUND" value="-1000" units="mmol_per_gDW_per_hr" constant="true"/>
      <parameter id="UPPER_BOUND" value="1000" units="mmol_per_gDW_per_hr" constant="true"/>
      <parameter id="FLUX_VALUE" value="0" units="mmol_per_gDW_per_hr" constant="true"/>
      <parameter id="OBJECTIVE_COEFFICIENT" value="0" units="mmol_per_gDW_per_hr" constant="true"/>
    </listOfParameters>
  </kineticLaw>
</reaction>
<reaction id="R_R066" name="sucrose glucohydrolase" reversible="false">
  <notes>
    <body xmlns="http://www.w3.org/1999/xhtml">
      <p>GENE_ASSOCIATION: (TTC0107 or TTC1283 or TT_P0221)</p>
      <p>SUBSYSTEM: Alternate Carbon Metabolism</p>
      <p>EC Number: 3.2.1.20</p>
      <p>Confidence Level: 1</p>
      <p>AUTHORS: Carbohydrate metabolism</p>
      <p/>
    </body>
  </notes>
  <listOfReactants>
    <speciesReference species="M_H2O_c" stoichiometry="1"/>
    <speciesReference species="M_SUC_c" stoichiometry="1"/>
  </listOfReactants>
```

```
  <listOfProducts>
    <speciesReference species="M_D_GLC_c" stoichiometry="1"/>
    <speciesReference species="M_D_FRU_c" stoichiometry="1"/>
  </listOfProducts>
  <kineticLaw>
    <math xmlns="http://www.w3.org/1998/Math/MathML">
      <ci> FLUX_VALUE </ci>
    </math>
    <listOfParameters>
      <parameter id="LOWER_BOUND" value="0" units="mmol_per_gDW_per_hr" constant="true"/>
      <parameter id="UPPER_BOUND" value="1000" units="mmol_per_gDW_per_hr" constant="true"/>
      <parameter id="FLUX_VALUE" value="0" units="mmol_per_gDW_per_hr" constant="true"/>
      <parameter id="OBJECTIVE_COEFFICIENT" value="0" units="mmol_per_gDW_per_hr" constant="true"/>
    </listOfParameters>
  </kineticLaw>
</reaction>
<reaction id="R_R067" name="palatinose glucohydrolase" reversible="false">
  <notes>
    <body xmlns="http://www.w3.org/1999/xhtml">
      <p>GENE_ASSOCIATION: (TTC0107 or TTC1283 or TT_P0221)</p>
      <p>SUBSYSTEM: Alternate Carbon Metabolism</p>
      <p>EC Number: 3.2.1.20</p>
      <p>Confidence Level: 1</p>
      <p>AUTHORS: Carbohydrate metabolism</p>
      <p/>
    </body>
  </notes>
  <listOfReactants>
    <speciesReference species="M_H2O_c" stoichiometry="1"/>
```

```xml
    <speciesReference species="M_PALT_c" stoichiometry="1"/>
  </listOfReactants>
  <listOfProducts>
    <speciesReference species="M_D_GLC_c" stoichiometry="1"/>
    <speciesReference species="M_D_FRU_c" stoichiometry="1"/>
  </listOfProducts>
  <kineticLaw>
    <math xmlns="http://www.w3.org/1998/Math/MathML">
      <ci> FLUX_VALUE </ci>
    </math>
    <listOfParameters>
      <parameter id="LOWER_BOUND" value="0" units="mmol_per_gDW_per_hr" constant="true"/>
      <parameter id="UPPER_BOUND" value="1000" units="mmol_per_gDW_per_hr" constant="true"/>
      <parameter id="FLUX_VALUE" value="0" units="mmol_per_gDW_per_hr" constant="true"/>
      <parameter id="OBJECTIVE_COEFFICIENT" value="0" units="mmol_per_gDW_per_hr" constant="true"/>
    </listOfParameters>
  </kineticLaw>
</reaction>
<reaction id="R_R068" name="octaprenyl-diphosphate synthase" reversible="true">
  <notes>
    <body xmlns="http://www.w3.org/1999/xhtml">
      <p>GENE_ASSOCIATION: TTC1291</p>
      <p>SUBSYSTEM: Cofactor and Prosthetic Group Biosynthesis</p>
      <p>EC Number: 2.5.1.90</p>
      <p>Confidence Level: 1</p>
      <p>AUTHORS: Energy and cofactors metabolism</p>
      <p/>
    </body>
  </notes>
```

```
  <listOfReactants>
    <speciesReference species="M_ct_FARPPi_c" stoichiometry="1"/>
    <speciesReference species="M_IPEN_PPi_c" stoichiometry="5"/>
  </listOfReactants>
  <listOfProducts>
    <speciesReference species="M_PPi_c" stoichiometry="5"/>
    <speciesReference species="M_t_PPREPPi_c" stoichiometry="1"/>
  </listOfProducts>
  <kineticLaw>
    <math xmlns="http://www.w3.org/1998/Math/MathML">
      <ci> FLUX_VALUE </ci>
    </math>
    <listOfParameters>
      <parameter id="LOWER_BOUND" value="-1000" units="mmol_per_gDW_per_hr" constant="true"/>
      <parameter id="UPPER_BOUND" value="1000" units="mmol_per_gDW_per_hr" constant="true"/>
      <parameter id="FLUX_VALUE" value="0" units="mmol_per_gDW_per_hr" constant="true"/>
      <parameter id="OBJECTIVE_COEFFICIENT" value="0" units="mmol_per_gDW_per_hr" constant="true"/>
    </listOfParameters>
  </kineticLaw>
</reaction>
<reaction id="R_R069" name="chorismate lyase" reversible="false">
  <notes>
    <body xmlns="http://www.w3.org/1999/xhtml">
      <p>GENE_ASSOCIATION: </p>
      <p>SUBSYSTEM: Cofactor and Prosthetic Group Biosynthesis</p>
      <p>EC Number: 4.1.3.40</p>
      <p>Confidence Level: 1</p>
      <p>AUTHORS: Energy and cofactors metabolism</p>
      <p/>
```

```
    </body>
  </notes>
  <listOfReactants>
    <speciesReference species="M_CORM_c" stoichiometry="1"/>
  </listOfReactants>
  <listOfProducts>
    <speciesReference species="M_PYR_c" stoichiometry="1"/>
    <speciesReference species="M_HY_BNZA_c" stoichiometry="1"/>
  </listOfProducts>
  <kineticLaw>
    <math xmlns="http://www.w3.org/1998/Math/MathML">
      <ci> FLUX_VALUE </ci>
    </math>
    <listOfParameters>
      <parameter id="LOWER_BOUND" value="0" units="mmol_per_gDW_per_hr" constant="true"/>
      <parameter id="UPPER_BOUND" value="1000" units="mmol_per_gDW_per_hr" constant="true"/>
      <parameter id="FLUX_VALUE" value="0" units="mmol_per_gDW_per_hr" constant="true"/>
      <parameter id="OBJECTIVE_COEFFICIENT" value="0" units="mmol_per_gDW_per_hr" constant="true"/>
    </listOfParameters>
  </kineticLaw>
</reaction>
<reaction id="R_R070" name="Polyisopentenylpyrolinate:4-hydroxybenzoate nonaprenyltransferase" reversible="true">
  <notes>
    <body xmlns="http://www.w3.org/1999/xhtml">
      <p>GENE_ASSOCIATION: TTC0240 </p>
      <p>SUBSYSTEM: Cofactor and Prosthetic Group Biosynthesis</p>
      <p>EC Number: 2.5.1.39</p>
      <p>Confidence Level: 1</p>
      <p>AUTHORS: Energy and cofactors metabolism</p>
```

```
      <p/>
    </body>
  </notes>
  <listOfReactants>
    <speciesReference species="M_t_PPREPPi_c" stoichiometry="1"/>
    <speciesReference species="M_HY_BNZA_c" stoichiometry="1"/>
  </listOfReactants>
  <listOfProducts>
    <speciesReference species="M_PPi_c" stoichiometry="1"/>
    <speciesReference species="M_HY_PPREBNZA_c" stoichiometry="1"/>
  </listOfProducts>
  <kineticLaw>
    <math xmlns="http://www.w3.org/1998/Math/MathML">
      <ci> FLUX_VALUE </ci>
    </math>
    <listOfParameters>
      <parameter id="LOWER_BOUND" value="-1000" units="mmol_per_gDW_per_hr" constant="true"/>
      <parameter id="UPPER_BOUND" value="1000" units="mmol_per_gDW_per_hr" constant="true"/>
      <parameter id="FLUX_VALUE" value="0" units="mmol_per_gDW_per_hr" constant="true"/>
      <parameter id="OBJECTIVE_COEFFICIENT" value="0" units="mmol_per_gDW_per_hr" constant="true"/>
    </listOfParameters>
  </kineticLaw>
</reaction>
<reaction id="R_R071" name="4-hydroxy-3-polyprenylbenzoate carboxy-lyase" reversible="true">
  <notes>
    <body xmlns="http://www.w3.org/1999/xhtml">
      <p>GENE_ASSOCIATION: TTC1553</p>
      <p>SUBSYSTEM: Cofactor and Prosthetic Group Biosynthesis</p>
      <p>EC Number: 4.1.1.-</p>
```

```
      <p>Confidence Level: 1</p>
      <p>AUTHORS: Energy and cofactors metabolism</p>
      <p/>
    </body>
  </notes>
  <listOfReactants>
    <speciesReference species="M_CO2_c" stoichiometry="1"/>
    <speciesReference species="M_PPRENPhOL_c" stoichiometry="1"/>
  </listOfReactants>
  <listOfProducts>
    <speciesReference species="M_HY_PPREBNZA_c" stoichiometry="1"/>
  </listOfProducts>
  <kineticLaw>
    <math xmlns="http://www.w3.org/1998/Math/MathML">
      <ci> FLUX_VALUE </ci>
    </math>
    <listOfParameters>
      <parameter id="LOWER_BOUND" value="-1000" units="mmol_per_gDW_per_hr" constant="true"/>
      <parameter id="UPPER_BOUND" value="1000" units="mmol_per_gDW_per_hr" constant="true"/>
      <parameter id="FLUX_VALUE" value="0" units="mmol_per_gDW_per_hr" constant="true"/>
      <parameter id="OBJECTIVE_COEFFICIENT" value="0" units="mmol_per_gDW_per_hr" constant="true"/>
    </listOfParameters>
  </kineticLaw>
</reaction>
<reaction id="R_R072" name="polyprenylphenol hydroxylase" reversible="false">
  <notes>
    <body xmlns="http://www.w3.org/1999/xhtml">
      <p>GENE_ASSOCIATION: </p>
      <p>SUBSYSTEM: Cofactor and Prosthetic Group Biosynthesis</p>
```

```
      <p>EC Number: 1.14.13.-</p>
      <p>Confidence Level: 1</p>
      <p>AUTHORS: Energy and cofactors metabolism</p>
      <p/>
    </body>
  </notes>
  <listOfReactants>
    <speciesReference species="M_H_c" stoichiometry="1"/>
    <speciesReference species="M_NADPH_c" stoichiometry="1"/>
    <speciesReference species="M_PPRENPhOL_c" stoichiometry="1"/>
    <speciesReference species="M_O2_c" stoichiometry="1"/>
  </listOfReactants>
  <listOfProducts>
    <speciesReference species="M_H2O_c" stoichiometry="1"/>
    <speciesReference species="M_NADP_c" stoichiometry="1"/>
    <speciesReference species="M_PBDIOL_c" stoichiometry="1"/>
  </listOfProducts>
  <kineticLaw>
    <math xmlns="http://www.w3.org/1998/Math/MathML">
      <ci> FLUX_VALUE </ci>
    </math>
    <listOfParameters>
      <parameter id="LOWER_BOUND" value="0" units="mmol_per_gDW_per_hr" constant="true"/>
      <parameter id="UPPER_BOUND" value="1000" units="mmol_per_gDW_per_hr" constant="true"/>
      <parameter id="FLUX_VALUE" value="0" units="mmol_per_gDW_per_hr" constant="true"/>
      <parameter id="OBJECTIVE_COEFFICIENT" value="0" units="mmol_per_gDW_per_hr" constant="true"/>
    </listOfParameters>
  </kineticLaw>
</reaction>
```

```
<reaction id="R_R073" name="2-polyprenyl-6-hydroxyphenol methylase" reversible="false">
 <notes>
  <body xmlns="http://www.w3.org/1999/xhtml">
   <p>GENE_ASSOCIATION: </p>
   <p>SUBSYSTEM: Cofactor and Prosthetic Group Biosynthesis</p>
   <p>EC Number: 2.1.1.222</p>
   <p>Confidence Level: 1</p>
   <p>AUTHORS: Energy and cofactors metabolism</p>
   <p/>
  </body>
 </notes>
 <listOfReactants>
  <speciesReference species="M_PBDIOL_c" stoichiometry="1"/>
  <speciesReference species="M_SAM_c" stoichiometry="1"/>
 </listOfReactants>
 <listOfProducts>
  <speciesReference species="M_H_c" stoichiometry="1"/>
  <speciesReference species="M_METPPHOL_c" stoichiometry="1"/>
  <speciesReference species="M_SAHC_c" stoichiometry="1"/>
 </listOfProducts>
 <kineticLaw>
  <math xmlns="http://www.w3.org/1998/Math/MathML">
   <ci> FLUX_VALUE </ci>
  </math>
  <listOfParameters>
   <parameter id="LOWER_BOUND" value="0" units="mmol_per_gDW_per_hr" constant="true"/>
   <parameter id="UPPER_BOUND" value="1000" units="mmol_per_gDW_per_hr" constant="true"/>
   <parameter id="FLUX_VALUE" value="0" units="mmol_per_gDW_per_hr" constant="true"/>
   <parameter id="OBJECTIVE_COEFFICIENT" value="0" units="mmol_per_gDW_per_hr" constant="true"/>
```

```
    &lt;/listOfParameters&gt;
  &lt;/kineticLaw&gt;
&lt;/reaction&gt;
&lt;reaction id="R_R074" name="2-methoxy-6-all-trans-polyprenylphenol hydroxylase" reversible="false"&gt;
  &lt;notes&gt;
    &lt;body xmlns="http://www.w3.org/1999/xhtml"&gt;
      <p>GENE_ASSOCIATION: </p>
      <p>SUBSYSTEM: Cofactor and Prosthetic Group Biosynthesis</p>
      <p>EC Number: 1.14.13.-</p>
      <p>Confidence Level: 1</p>
      <p>AUTHORS: Energy and cofactors metabolism</p>
      <p/>
    &lt;/body&gt;
  &lt;/notes&gt;
  &lt;listOfReactants&gt;
    &lt;speciesReference species="M_H_c" stoichiometry="1"/&gt;
    &lt;speciesReference species="M_NADPH_c" stoichiometry="1"/&gt;
    &lt;speciesReference species="M_O2_c" stoichiometry="1"/&gt;
    &lt;speciesReference species="M_METPPHOL_c" stoichiometry="1"/&gt;
  &lt;/listOfReactants&gt;
  &lt;listOfProducts&gt;
    &lt;speciesReference species="M_H2O_c" stoichiometry="1"/&gt;
    &lt;speciesReference species="M_NADP_c" stoichiometry="1"/&gt;
    &lt;speciesReference species="M_PPRE_MOX_BNZQ_c" stoichiometry="1"/&gt;
  &lt;/listOfProducts&gt;
  &lt;kineticLaw&gt;
    &lt;math xmlns="http://www.w3.org/1998/Math/MathML"&gt;
      &lt;ci&gt; FLUX_VALUE &lt;/ci&gt;
    &lt;/math&gt;
```

```xml
    <listOfParameters>
      <parameter id="LOWER_BOUND" value="0" units="mmol_per_gDW_per_hr" constant="true"/>
      <parameter id="UPPER_BOUND" value="1000" units="mmol_per_gDW_per_hr" constant="true"/>
      <parameter id="FLUX_VALUE" value="0" units="mmol_per_gDW_per_hr" constant="true"/>
      <parameter id="OBJECTIVE_COEFFICIENT" value="0" units="mmol_per_gDW_per_hr" constant="true"/>
    </listOfParameters>
  </kineticLaw>
</reaction>
<reaction id="R_R075" name="2-Polyprenyl-6-methoxy-1,4-benzoquinone hydroxylase" reversible="false">
  <notes>
    <body xmlns="http://www.w3.org/1999/xhtml">
      <p>GENE_ASSOCIATION: </p>
      <p>SUBSYSTEM: Cofactor and Prosthetic Group Biosynthesis</p>
      <p>EC Number: 1.14.13.-</p>
      <p>Confidence Level: 1</p>
      <p>AUTHORS: Energy and cofactors metabolism</p>
      <p/>
    </body>
  </notes>
  <listOfReactants>
    <speciesReference species="M_NADPH_c" stoichiometry="1"/>
    <speciesReference species="M_O2_c" stoichiometry="1"/>
    <speciesReference species="M_PPRE_MOX_BNZQ_c" stoichiometry="1"/>
  </listOfReactants>
  <listOfProducts>
    <speciesReference species="M_H2O_c" stoichiometry="1"/>
    <speciesReference species="M_NADP_c" stoichiometry="1"/>
    <speciesReference species="M_DEMQH2_c" stoichiometry="1"/>
  </listOfProducts>
```

```xml
<kineticLaw>
  <math xmlns="http://www.w3.org/1998/Math/MathML">
    <ci> FLUX_VALUE </ci>
  </math>
  <listOfParameters>
    <parameter id="LOWER_BOUND" value="0" units="mmol_per_gDW_per_hr" constant="true"/>
    <parameter id="UPPER_BOUND" value="1000" units="mmol_per_gDW_per_hr" constant="true"/>
    <parameter id="FLUX_VALUE" value="0" units="mmol_per_gDW_per_hr" constant="true"/>
    <parameter id="OBJECTIVE_COEFFICIENT" value="0" units="mmol_per_gDW_per_hr" constant="true"/>
  </listOfParameters>
</kineticLaw>
</reaction>
<reaction id="R_R076" name="3-Demethylubiquinol methylase" reversible="false">
<notes>
  <body xmlns="http://www.w3.org/1999/xhtml">
    <p>GENE_ASSOCIATION: </p>
    <p>SUBSYSTEM: Cofactor and Prosthetic Group Biosynthesis</p>
    <p>EC Number: 2.1.1.64</p>
    <p>Confidence Level: 1</p>
    <p>AUTHORS: Energy and cofactors metabolism</p>
    <p/>
  </body>
</notes>
<listOfReactants>
  <speciesReference species="M_SAM_c" stoichiometry="1"/>
  <speciesReference species="M_DEMQH2_c" stoichiometry="1"/>
</listOfReactants>
<listOfProducts>
  <speciesReference species="M_H_c" stoichiometry="1"/>
```

```
<speciesReference species="M_QH2_c" stoichiometry="1"/>
<speciesReference species="M_SAHC_c" stoichiometry="1"/>
</listOfProducts>
<kineticLaw>
<math xmlns="http://www.w3.org/1998/Math/MathML">
<ci> FLUX_VALUE </ci>
</math>
<listOfParameters>
<parameter id="LOWER_BOUND" value="0" units="mmol_per_gDW_per_hr" constant="true"/>
<parameter id="UPPER_BOUND" value="1000" units="mmol_per_gDW_per_hr" constant="true"/>
<parameter id="FLUX_VALUE" value="0" units="mmol_per_gDW_per_hr" constant="true"/>
<parameter id="OBJECTIVE_COEFFICIENT" value="0" units="mmol_per_gDW_per_hr" constant="true"/>
</listOfParameters>
</kineticLaw>
</reaction>
<reaction id="R_R077" name="ubiquinone/menaquinone biosynthesis methyltransferase" reversible="true">
<notes>
<body xmlns="http://www.w3.org/1999/xhtml">
<p>GENE_ASSOCIATION: TTC1503</p>
<p>SUBSYSTEM: Cofactor and Prosthetic Group Biosynthesis</p>
<p>EC Number: 2.1.1.201</p>
<p>Confidence Level: 1</p>
<p>AUTHORS: Energy and cofactors metabolism</p>
<p/>
</body>
</notes>
<listOfReactants>
<speciesReference species="M_SAM_c" stoichiometry="1"/>
<speciesReference species="M_PPRE_MOX_BNZQ_c" stoichiometry="1"/>
```

```
  </listOfReactants>
  <listOfProducts>
    <speciesReference species="M_SAHC_c" stoichiometry="1"/>
    <speciesReference species="M_PPRE_MMOX_BNZQ_c" stoichiometry="1"/>
  </listOfProducts>
  <kineticLaw>
    <math xmlns="http://www.w3.org/1998/Math/MathML">
      <ci> FLUX_VALUE </ci>
    </math>
    <listOfParameters>
      <parameter id="LOWER_BOUND" value="-1000" units="mmol_per_gDW_per_hr" constant="true"/>
      <parameter id="UPPER_BOUND" value="1000" units="mmol_per_gDW_per_hr" constant="true"/>
      <parameter id="FLUX_VALUE" value="0" units="mmol_per_gDW_per_hr" constant="true"/>
      <parameter id="OBJECTIVE_COEFFICIENT" value="0" units="mmol_per_gDW_per_hr" constant="true"/>
    </listOfParameters>
  </kineticLaw>
</reaction>
<reaction id="R_R078" name="2-Polyprenyl-3-methyl-6-methoxy-1,4-benzoquinone hydroxylase" reversible="false">
  <notes>
    <body xmlns="http://www.w3.org/1999/xhtml">
      <p>GENE_ASSOCIATION: </p>
      <p>SUBSYSTEM: Cofactor and Prosthetic Group Biosynthesis</p>
      <p>EC Number: 1.14.13.-</p>
      <p>Confidence Level: 1</p>
      <p>AUTHORS: Energy and cofactors metabolism</p>
      <p/>
    </body>
  </notes>
  <listOfReactants>
```

```
    <speciesReference species="M_NADPH_c" stoichiometry="1"/>
    <speciesReference species="M_O2_c" stoichiometry="1"/>
    <speciesReference species="M_PPRE_MMOX_BNZQ_c" stoichiometry="1"/>
  </listOfReactants>
  <listOfProducts>
    <speciesReference species="M_H2O_c" stoichiometry="1"/>
    <speciesReference species="M_NADP_c" stoichiometry="1"/>
    <speciesReference species="M_DMMQ_c" stoichiometry="1"/>
  </listOfProducts>
  <kineticLaw>
    <math xmlns="http://www.w3.org/1998/Math/MathML">
      <ci> FLUX_VALUE </ci>
    </math>
    <listOfParameters>
      <parameter id="LOWER_BOUND" value="0" units="mmol_per_gDW_per_hr" constant="true"/>
      <parameter id="UPPER_BOUND" value="1000" units="mmol_per_gDW_per_hr" constant="true"/>
      <parameter id="FLUX_VALUE" value="0" units="mmol_per_gDW_per_hr" constant="true"/>
      <parameter id="OBJECTIVE_COEFFICIENT" value="0" units="mmol_per_gDW_per_hr" constant="true"/>
    </listOfParameters>
  </kineticLaw>
</reaction>
<reaction id="R_R079" name="S-adenosyl-L-methione:demethylmenaquinone methyltransferase" reversible="true">
  <notes>
    <body xmlns="http://www.w3.org/1999/xhtml">
      <p>GENE_ASSOCIATION: TTC1503</p>
      <p>SUBSYSTEM: Cofactor and Prosthetic Group Biosynthesis</p>
      <p>EC Number: 2.1.1.163</p>
      <p>Confidence Level: 1</p>
      <p>AUTHORS: Energy and cofactors metabolism</p>
```

```
      <p/>
    </body>
  </notes>
  <listOfReactants>
    <speciesReference species="M_SAM_c" stoichiometry="1"/>
    <speciesReference species="M_DMMQ_c" stoichiometry="1"/>
  </listOfReactants>
  <listOfProducts>
    <speciesReference species="M_SAHC_c" stoichiometry="1"/>
    <speciesReference species="M_MQ_c" stoichiometry="1"/>
  </listOfProducts>
  <kineticLaw>
    <math xmlns="http://www.w3.org/1998/Math/MathML">
      <ci> FLUX_VALUE </ci>
    </math>
    <listOfParameters>
      <parameter id="LOWER_BOUND" value="-1000" units="mmol_per_gDW_per_hr" constant="true"/>
      <parameter id="UPPER_BOUND" value="1000" units="mmol_per_gDW_per_hr" constant="true"/>
      <parameter id="FLUX_VALUE" value="0" units="mmol_per_gDW_per_hr" constant="true"/>
      <parameter id="OBJECTIVE_COEFFICIENT" value="0" units="mmol_per_gDW_per_hr" constant="true"/>
    </listOfParameters>
  </kineticLaw>
</reaction>
<reaction id="R_R080" name="(1R,6R)-6-hydroxy-2-succinylcyclohexa-2,4-diene-1-carboxylate hydrolyase (2-
succinylbenzoate-forming)" reversible="false">
  <notes>
    <body xmlns="http://www.w3.org/1999/xhtml">
      <p>GENE_ASSOCIATION: TTC0519</p>
      <p>SUBSYSTEM: Cofactor and Prosthetic Group Biosynthesis</p>
```

```xml
<p>EC Number: 4.2.1.113</p>
<p>Confidence Level: 1</p>
<p>AUTHORS: Energy and cofactors metabolism</p>
<p/>
</body>
</notes>
<listOfReactants>
<speciesReference species="M_HY_SUCC_CYCHEXDE_CARBA_c" stoichiometry="1"/>
</listOfReactants>
<listOfProducts>
<speciesReference species="M_H2O_c" stoichiometry="1"/>
<speciesReference species="M_SUCC_BNZA_c" stoichiometry="1"/>
</listOfProducts>
<kineticLaw>
<math xmlns="http://www.w3.org/1998/Math/MathML">
<ci> FLUX_VALUE </ci>
</math>
<listOfParameters>
<parameter id="LOWER_BOUND" value="0" units="mmol_per_gDW_per_hr" constant="true"/>
<parameter id="UPPER_BOUND" value="1000" units="mmol_per_gDW_per_hr" constant="true"/>
<parameter id="FLUX_VALUE" value="0" units="mmol_per_gDW_per_hr" constant="true"/>
<parameter id="OBJECTIVE_COEFFICIENT" value="0" units="mmol_per_gDW_per_hr" constant="true"/>
</listOfParameters>
</kineticLaw>
</reaction>
<reaction id="R_R081" name="Acetyl-CoA:carbon-dioxide ligase (ADP-forming)" reversible="true">
<notes>
<body xmlns="http://www.w3.org/1999/xhtml">
<p>GENE_ASSOCIATION: (TTC0758 and TTC1408 and TTC1409)</p>
```

```
          <p>SUBSYSTEM: Cell Envelope Biosynthesis</p>
          <p>EC Number: 6.4.1.2</p>
          <p>Confidence Level: 1</p>
          <p>AUTHORS: Lipid metabolism</p>
          <p/>
        </body>
      </notes>
      <listOfReactants>
        <speciesReference species="M_ACoA_c" stoichiometry="1"/>
        <speciesReference species="M_CBTN_CCP_c" stoichiometry="1"/>
      </listOfReactants>
      <listOfProducts>
        <speciesReference species="M_MALCoA_c" stoichiometry="1"/>
        <speciesReference species="M_HCARB_c" stoichiometry="1"/>
      </listOfProducts>
      <kineticLaw>
        <math xmlns="http://www.w3.org/1998/Math/MathML">
          <ci> FLUX_VALUE </ci>
        </math>
        <listOfParameters>
          <parameter id="LOWER_BOUND" value="-1000" units="mmol_per_gDW_per_hr" constant="true"/>
          <parameter id="UPPER_BOUND" value="1000" units="mmol_per_gDW_per_hr" constant="true"/>
          <parameter id="FLUX_VALUE" value="0" units="mmol_per_gDW_per_hr" constant="true"/>
          <parameter id="OBJECTIVE_COEFFICIENT" value="0" units="mmol_per_gDW_per_hr" constant="true"/>
        </listOfParameters>
      </kineticLaw>
    </reaction>
    <reaction id="R_R082" name="glutaryl-CoA:electron-transfer flavoprotein 2,3-oxidoreductase (decarboxylating)"
reversible="false">
```

```xml
<notes>
  <body xmlns="http://www.w3.org/1999/xhtml">
    <p>GENE_ASSOCIATION: TTC0435</p>
    <p>SUBSYSTEM: Membrane Lipid Metabolism</p>
    <p>EC Number: 1.3.8.6</p>
    <p>Confidence Level: 1</p>
    <p>AUTHORS: Lipid metabolism</p>
    <p/>
  </body>
</notes>
<listOfReactants>
  <speciesReference species="M_H_c" stoichiometry="1"/>
  <speciesReference species="M_FAD_c" stoichiometry="1"/>
  <speciesReference species="M_GLUTRCoA_c" stoichiometry="1"/>
</listOfReactants>
<listOfProducts>
  <speciesReference species="M_CO2_c" stoichiometry="1"/>
  <speciesReference species="M_FADH2_c" stoichiometry="1"/>
  <speciesReference species="M_CROCoA_c" stoichiometry="1"/>
</listOfProducts>
<kineticLaw>
  <math xmlns="http://www.w3.org/1998/Math/MathML">
    <ci> FLUX_VALUE </ci>
  </math>
  <listOfParameters>
    <parameter id="LOWER_BOUND" value="0" units="mmol_per_gDW_per_hr" constant="true"/>
    <parameter id="UPPER_BOUND" value="1000" units="mmol_per_gDW_per_hr" constant="true"/>
    <parameter id="FLUX_VALUE" value="0" units="mmol_per_gDW_per_hr" constant="true"/>
    <parameter id="OBJECTIVE_COEFFICIENT" value="0" units="mmol_per_gDW_per_hr" constant="true"/>
```

```
      </listOfParameters>
    </kineticLaw>
  </reaction>
  <reaction id="R_R083" name="NADH:ubiquinone oxidoreductase" reversible="false">
    <notes>
      <body xmlns="http://www.w3.org/1999/xhtml">
        <p>GENE_ASSOCIATION: (TTC1907 and TTC1908 and TTC1909 and TTC1910 and TTC1911 and TTC1912 and TTC1913 and TTC1914 and TTC1915 and TTC1916 and TTC1917 and TTC1918 and TTC1919 and TTC1920 and TT_P0054)</p>
        <p>SUBSYSTEM: Oxidative Phosphorylation</p>
        <p>EC Number: 1.6.5.3</p>
        <p>Confidence Level: 1</p>
        <p>AUTHORS: Energy and cofactors metabolism</p>
        <p/>
      </body>
    </notes>
    <listOfReactants>
      <speciesReference species="M_H_c" stoichiometry="4"/>
      <speciesReference species="M_NADH_c" stoichiometry="1"/>
      <speciesReference species="M_Q_c" stoichiometry="1"/>
    </listOfReactants>
    <listOfProducts>
      <speciesReference species="M_NAD_c" stoichiometry="1"/>
      <speciesReference species="M_QH2_c" stoichiometry="1"/>
      <speciesReference species="M_H_e" stoichiometry="3"/>
    </listOfProducts>
    <kineticLaw>
      <math xmlns="http://www.w3.org/1998/Math/MathML">
        <ci> FLUX_VALUE </ci>
      </math>
```

```
    <listOfParameters>
      <parameter id="LOWER_BOUND" value="0" units="mmol_per_gDW_per_hr" constant="true"/>
      <parameter id="UPPER_BOUND" value="1000" units="mmol_per_gDW_per_hr" constant="true"/>
      <parameter id="FLUX_VALUE" value="0" units="mmol_per_gDW_per_hr" constant="true"/>
      <parameter id="OBJECTIVE_COEFFICIENT" value="0" units="mmol_per_gDW_per_hr" constant="true"/>
    </listOfParameters>
  </kineticLaw>
</reaction>
<reaction id="R_R084" name="Ubiquinol:ferricytochrome-c oxidoreductase" reversible="true">
  <notes>
    <body xmlns="http://www.w3.org/1999/xhtml">
      <p>GENE_ASSOCIATION: </p>
      <p>SUBSYSTEM: Oxidative Phosphorylation</p>
      <p>EC Number: 1.10.2.2</p>
      <p>Confidence Level: 1</p>
      <p>AUTHORS: Energy and cofactors metabolism</p>
      <p/>
    </body>
  </notes>
  <listOfReactants>
    <speciesReference species="M_QH2_c" stoichiometry="1"/>
    <speciesReference species="M_CYT_C3_c" stoichiometry="2"/>
  </listOfReactants>
  <listOfProducts>
    <speciesReference species="M_Q_c" stoichiometry="1"/>
    <speciesReference species="M_CYT_C2_c" stoichiometry="2"/>
  </listOfProducts>
  <kineticLaw>
    <math xmlns="http://www.w3.org/1998/Math/MathML">
```

```
      <ci> FLUX_VALUE </ci>
    </math>
    <listOfParameters>
      <parameter id="LOWER_BOUND" value="-1000" units="mmol_per_gDW_per_hr" constant="true"/>
      <parameter id="UPPER_BOUND" value="1000" units="mmol_per_gDW_per_hr" constant="true"/>
      <parameter id="FLUX_VALUE" value="0" units="mmol_per_gDW_per_hr" constant="true"/>
      <parameter id="OBJECTIVE_COEFFICIENT" value="0" units="mmol_per_gDW_per_hr" constant="true"/>
    </listOfParameters>
  </kineticLaw>
</reaction>
<reaction id="R_R085" name="diphosphate phosphohydrolase" reversible="false">
  <notes>
    <body xmlns="http://www.w3.org/1999/xhtml">
      <p>GENE_ASSOCIATION: TTC1600</p>
      <p>SUBSYSTEM: Anaplerotic Reactions</p>
      <p>EC Number: 3.6.1.1</p>
      <p>Confidence Level: 1</p>
      <p>AUTHORS: Energy and cofactors metabolism</p>
      <p/>
    </body>
  </notes>
  <listOfReactants>
    <speciesReference species="M_PPi_c" stoichiometry="1"/>
    <speciesReference species="M_H2O_c" stoichiometry="1"/>
  </listOfReactants>
  <listOfProducts>
    <speciesReference species="M_Pi_c" stoichiometry="2"/>
    <speciesReference species="M_H_c" stoichiometry="1"/>
  </listOfProducts>
```

```xml
  <kineticLaw>
    <math xmlns="http://www.w3.org/1998/Math/MathML">
      <ci> FLUX_VALUE </ci>
    </math>
    <listOfParameters>
      <parameter id="LOWER_BOUND" value="0" units="mmol_per_gDW_per_hr" constant="true"/>
      <parameter id="UPPER_BOUND" value="1000" units="mmol_per_gDW_per_hr" constant="true"/>
      <parameter id="FLUX_VALUE" value="0" units="mmol_per_gDW_per_hr" constant="true"/>
      <parameter id="OBJECTIVE_COEFFICIENT" value="0" units="mmol_per_gDW_per_hr" constant="true"/>
    </listOfParameters>
  </kineticLaw>
</reaction>
<reaction id="R_R086" name="ATP phosphohydrolase" reversible="false">
  <notes>
    <body xmlns="http://www.w3.org/1999/xhtml">
      <p>GENE_ASSOCIATION: (TT_P0081 or TT_P0211 or TTC1823 or TTC1844 or TTC1290)</p>
      <p>SUBSYSTEM: Nucleotide Salvage Pathway</p>
      <p>EC Number: 3.6.3.14</p>
      <p>Confidence Level: 1</p>
      <p>AUTHORS: Nucleotides metabolism</p>
      <p/>
    </body>
  </notes>
  <listOfReactants>
    <speciesReference species="M_ATP_c" stoichiometry="1"/>
    <speciesReference species="M_H2O_c" stoichiometry="1"/>
  </listOfReactants>
  <listOfProducts>
    <speciesReference species="M_ADP_c" stoichiometry="1"/>
```

```
    <speciesReference species="M_Pi_c" stoichiometry="1"/>
    <speciesReference species="M_H_c" stoichiometry="1"/>
  </listOfProducts>
  <kineticLaw>
    <math xmlns="http://www.w3.org/1998/Math/MathML">
      <ci> FLUX_VALUE </ci>
    </math>
    <listOfParameters>
      <parameter id="LOWER_BOUND" value="0" units="mmol_per_gDW_per_hr" constant="true"/>
      <parameter id="UPPER_BOUND" value="1000" units="mmol_per_gDW_per_hr" constant="true"/>
      <parameter id="FLUX_VALUE" value="0" units="mmol_per_gDW_per_hr" constant="true"/>
      <parameter id="OBJECTIVE_COEFFICIENT" value="0" units="mmol_per_gDW_per_hr" constant="true"/>
    </listOfParameters>
  </kineticLaw>
</reaction>
<reaction id="R_R087" name="ADP-ribose ribophosphohydrolase" reversible="false">
  <notes>
    <body xmlns="http://www.w3.org/1999/xhtml">
      <p>GENE_ASSOCIATION: (TTC0160 or TTC0511)</p>
      <p>SUBSYSTEM: Nucleotide Salvage Pathway</p>
      <p>EC Number: 3.6.1.13</p>
      <p>Confidence Level: 1</p>
      <p>AUTHORS: Nucleotides metabolism</p>
      <p/>
    </body>
  </notes>
  <listOfReactants>
    <speciesReference species="M_H2O_c" stoichiometry="1"/>
    <speciesReference species="M_ADP_RIB_c" stoichiometry="1"/>
```

```xml
</listOfReactants>

<listOfProducts>

<speciesReference species="M_H_c" stoichiometry="2"/>

<speciesReference species="M_AMP_c" stoichiometry="1"/>

<speciesReference species="M_R5P_c" stoichiometry="1"/>

</listOfProducts>

<kineticLaw>

<math xmlns="http://www.w3.org/1998/Math/MathML">

<ci> FLUX_VALUE </ci>

</math>

<listOfParameters>

<parameter id="LOWER_BOUND" value="0" units="mmol_per_gDW_per_hr" constant="true"/>

<parameter id="UPPER_BOUND" value="1000" units="mmol_per_gDW_per_hr" constant="true"/>

<parameter id="FLUX_VALUE" value="0" units="mmol_per_gDW_per_hr" constant="true"/>

<parameter id="OBJECTIVE_COEFFICIENT" value="0" units="mmol_per_gDW_per_hr" constant="true"/>

</listOfParameters>

</kineticLaw>

</reaction>

<reaction id="R_R088" name="5-phosphoribosylamine:diphosphate phospho-alpha-D-ribosyltransferase (glutamate-amidating)" reversible="false">

<notes>

<body xmlns="http://www.w3.org/1999/xhtml">

<p>GENE_ASSOCIATION: TTC1156</p>

<p>SUBSYSTEM: Nucleotide Salvage Pathway</p>

<p>EC Number: 2.4.2.14</p>

<p>Confidence Level: 1</p>

<p>AUTHORS: Nucleotides metabolism</p>

<p/>

</body>
```

```xml
  </notes>
  <listOfReactants>
    <speciesReference species="M_L_GLN_c" stoichiometry="1"/>
    <speciesReference species="M_H2O_c" stoichiometry="1"/>
    <speciesReference species="M_PRPP_c" stoichiometry="1"/>
  </listOfReactants>
  <listOfProducts>
    <speciesReference species="M_L_GLU_c" stoichiometry="1"/>
    <speciesReference species="M_PPi_c" stoichiometry="1"/>
    <speciesReference species="M_PRIBSAMN_c" stoichiometry="1"/>
  </listOfProducts>
  <kineticLaw>
    <math xmlns="http://www.w3.org/1998/Math/MathML">
      <ci> FLUX_VALUE </ci>
    </math>
    <listOfParameters>
      <parameter id="LOWER_BOUND" value="0" units="mmol_per_gDW_per_hr" constant="true"/>
      <parameter id="UPPER_BOUND" value="1000" units="mmol_per_gDW_per_hr" constant="true"/>
      <parameter id="FLUX_VALUE" value="0" units="mmol_per_gDW_per_hr" constant="true"/>
      <parameter id="OBJECTIVE_COEFFICIENT" value="0" units="mmol_per_gDW_per_hr" constant="true"/>
    </listOfParameters>
  </kineticLaw>
</reaction>
<reaction id="R_R089" name="5-Phospho-D-ribosylamine:glycine ligase (ADP-forming)" reversible="false">
  <notes>
    <body xmlns="http://www.w3.org/1999/xhtml">
      <p>GENE_ASSOCIATION: TTC0460</p>
      <p>SUBSYSTEM: Nucleotide Salvage Pathway</p>
      <p>EC Number: 6.3.4.13</p>
```

```
      <p>Confidence Level: 1</p>
      <p>AUTHORS: Nucleotides metabolism</p>
      <p/>
    </body>
  </notes>
  <listOfReactants>
    <speciesReference species="M_L_GLY_c" stoichiometry="1"/>
    <speciesReference species="M_ATP_c" stoichiometry="1"/>
    <speciesReference species="M_PRIBSAMN_c" stoichiometry="1"/>
  </listOfReactants>
  <listOfProducts>
    <speciesReference species="M_ADP_c" stoichiometry="1"/>
    <speciesReference species="M_Pi_c" stoichiometry="1"/>
    <speciesReference species="M_H_c" stoichiometry="1"/>
    <speciesReference species="M_GAR_c" stoichiometry="1"/>
  </listOfProducts>
  <kineticLaw>
    <math xmlns="http://www.w3.org/1998/Math/MathML">
      <ci> FLUX_VALUE </ci>
    </math>
    <listOfParameters>
      <parameter id="LOWER_BOUND" value="0" units="mmol_per_gDW_per_hr" constant="true"/>
      <parameter id="UPPER_BOUND" value="1000" units="mmol_per_gDW_per_hr" constant="true"/>
      <parameter id="FLUX_VALUE" value="0" units="mmol_per_gDW_per_hr" constant="true"/>
      <parameter id="OBJECTIVE_COEFFICIENT" value="0" units="mmol_per_gDW_per_hr" constant="true"/>
    </listOfParameters>
  </kineticLaw>
</reaction>
```

```
<reaction id="R_R090" name="5&apos;-Phosphoribosylformylglycinamide:L-glutamine amido-ligase (ADP-forming)"
reversible="false">
  <notes>
    <body xmlns="http://www.w3.org/1999/xhtml">
      <p>GENE_ASSOCIATION: (TTC1152 or TTC1153 or TTC1155 or TTC1383)</p>
      <p>SUBSYSTEM: Nucleotide Salvage Pathway</p>
      <p>EC Number: 6.3.5.3</p>
      <p>Confidence Level: 1</p>
      <p>AUTHORS: Nucleotides metabolism</p>
      <p/>
    </body>
  </notes>
  <listOfReactants>
    <speciesReference species="M_L_GLN_c" stoichiometry="1"/>
    <speciesReference species="M_ATP_c" stoichiometry="1"/>
    <speciesReference species="M_H2O_c" stoichiometry="1"/>
    <speciesReference species="M_FGAR_c" stoichiometry="1"/>
  </listOfReactants>
  <listOfProducts>
    <speciesReference species="M_L_GLU_c" stoichiometry="1"/>
    <speciesReference species="M_ADP_c" stoichiometry="1"/>
    <speciesReference species="M_Pi_c" stoichiometry="1"/>
    <speciesReference species="M_H_c" stoichiometry="1"/>
    <speciesReference species="M_FGAM_c" stoichiometry="1"/>
  </listOfProducts>
  <kineticLaw>
    <math xmlns="http://www.w3.org/1998/Math/MathML">
      <ci> FLUX_VALUE </ci>
    </math>
```

```xml
<listOfParameters>
<parameter id="LOWER_BOUND" value="0" units="mmol_per_gDW_per_hr" constant="true"/>
<parameter id="UPPER_BOUND" value="1000" units="mmol_per_gDW_per_hr" constant="true"/>
<parameter id="FLUX_VALUE" value="0" units="mmol_per_gDW_per_hr" constant="true"/>
<parameter id="OBJECTIVE_COEFFICIENT" value="0" units="mmol_per_gDW_per_hr" constant="true"/>
</listOfParameters>
</kineticLaw>
</reaction>
<reaction id="R_R091" name="2-(Formamido)-N1-(5-phosphoribosyl)acetamidine cyclo-ligase (ADP-forming)" reversible="false">
<notes>
<body xmlns="http://www.w3.org/1999/xhtml">
<p>GENE_ASSOCIATION: TTC1619</p>
<p>SUBSYSTEM: Nucleotide Salvage Pathway</p>
<p>EC Number: 6.3.3.1</p>
<p>Confidence Level: 1</p>
<p>AUTHORS: Nucleotides metabolism</p>
<p/>
</body>
</notes>
<listOfReactants>
<speciesReference species="M_ATP_c" stoichiometry="1"/>
<speciesReference species="M_FGAM_c" stoichiometry="1"/>
</listOfReactants>
<listOfProducts>
<speciesReference species="M_ADP_c" stoichiometry="1"/>
<speciesReference species="M_Pi_c" stoichiometry="1"/>
<speciesReference species="M_H_c" stoichiometry="2"/>
<speciesReference species="M_AIR_c" stoichiometry="1"/>
```

```
        </listOfProducts>
        <kineticLaw>
          <math xmlns="http://www.w3.org/1998/Math/MathML">
            <ci> FLUX_VALUE </ci>
          </math>
          <listOfParameters>
            <parameter id="LOWER_BOUND" value="0" units="mmol_per_gDW_per_hr" constant="true"/>
            <parameter id="UPPER_BOUND" value="1000" units="mmol_per_gDW_per_hr" constant="true"/>
            <parameter id="FLUX_VALUE" value="0" units="mmol_per_gDW_per_hr" constant="true"/>
            <parameter id="OBJECTIVE_COEFFICIENT" value="0" units="mmol_per_gDW_per_hr" constant="true"/>
          </listOfParameters>
        </kineticLaw>
      </reaction>
      <reaction id="R_R092" name="5-amino-1-(5-phospho-D-ribosyl)imidazole:carbon-dioxide ligase (ADP-forming)"
reversible="false">
        <notes>
          <body xmlns="http://www.w3.org/1999/xhtml">
            <p>GENE_ASSOCIATION: TTC0137</p>
            <p>SUBSYSTEM: Nucleotide Salvage Pathway</p>
            <p>EC Number: 6.3.4.18</p>
            <p>Confidence Level: 1</p>
            <p>AUTHORS: Nucleotides metabolism</p>
            <p/>
          </body>
        </notes>
        <listOfReactants>
          <speciesReference species="M_ATP_c" stoichiometry="1"/>
          <speciesReference species="M_AIR_c" stoichiometry="1"/>
          <speciesReference species="M_HCO3_c" stoichiometry="1"/>
```

```xml
</listOfReactants>
<listOfProducts>
<speciesReference species="M_ADP_c" stoichiometry="1"/>
<speciesReference species="M_Pi_c" stoichiometry="1"/>
<speciesReference species="M_H_c" stoichiometry="1"/>
<speciesReference species="M_5CAIZ_c" stoichiometry="1"/>
</listOfProducts>
<kineticLaw>
<math xmlns="http://www.w3.org/1998/Math/MathML">
<ci> FLUX_VALUE </ci>
</math>
<listOfParameters>
<parameter id="LOWER_BOUND" value="0" units="mmol_per_gDW_per_hr" constant="true"/>
<parameter id="UPPER_BOUND" value="1000" units="mmol_per_gDW_per_hr" constant="true"/>
<parameter id="FLUX_VALUE" value="0" units="mmol_per_gDW_per_hr" constant="true"/>
<parameter id="OBJECTIVE_COEFFICIENT" value="0" units="mmol_per_gDW_per_hr" constant="true"/>
</listOfParameters>
</kineticLaw>
</reaction>
<reaction id="R_R093" name="5-carboxyamino-1-(5-phospho-D-ribosyl)imidazole carboxymutase" reversible="true">
<notes>
<body xmlns="http://www.w3.org/1999/xhtml">
<p>GENE_ASSOCIATION: TTC0138</p>
<p>SUBSYSTEM: Nucleotide Salvage Pathway</p>
<p>EC Number: 5.4.99.18</p>
<p>Confidence Level: 1</p>
<p>AUTHORS: Nucleotides metabolism</p>
<p/>
</body>
```

```xml
</notes>

<listOfReactants>

<speciesReference species="M_5CAIZ_c" stoichiometry="1"/>

</listOfReactants>

<listOfProducts>

<speciesReference species="M_CAIR_c" stoichiometry="1"/>

</listOfProducts>

<kineticLaw>

<math xmlns="http://www.w3.org/1998/Math/MathML">

<ci> FLUX_VALUE </ci>

</math>

<listOfParameters>

<parameter id="LOWER_BOUND" value="-1000" units="mmol_per_gDW_per_hr" constant="true"/>

<parameter id="UPPER_BOUND" value="1000" units="mmol_per_gDW_per_hr" constant="true"/>

<parameter id="FLUX_VALUE" value="0" units="mmol_per_gDW_per_hr" constant="true"/>

<parameter id="OBJECTIVE_COEFFICIENT" value="0" units="mmol_per_gDW_per_hr" constant="true"/>

</listOfParameters>

</kineticLaw>

</reaction>

<reaction id="R_R094" name="1-(5-Phosphoribosyl)-5-amino-4-carboxyimidazole:L-aspartate ligase (ADP-forming)" reversible="true">

<notes>

<body xmlns="http://www.w3.org/1999/xhtml">

<p>GENE_ASSOCIATION: TTC1151</p>

<p>SUBSYSTEM: Nucleotide Salvage Pathway</p>

<p>EC Number: 6.3.2.6</p>

<p>Confidence Level: 1</p>

<p>AUTHORS: Nucleotides metabolism</p>

<p/>
```

```
</body>
</notes>
<listOfReactants>
<speciesReference species="M_L_ASP_c" stoichiometry="1"/>
<speciesReference species="M_ATP_c" stoichiometry="1"/>
<speciesReference species="M_CAIR_c" stoichiometry="1"/>
</listOfReactants>
<listOfProducts>
<speciesReference species="M_ADP_c" stoichiometry="1"/>
<speciesReference species="M_Pi_c" stoichiometry="1"/>
<speciesReference species="M_H_c" stoichiometry="1"/>
<speciesReference species="M_SAICAR_c" stoichiometry="1"/>
</listOfProducts>
<kineticLaw>
<math xmlns="http://www.w3.org/1998/Math/MathML">
<ci> FLUX_VALUE </ci>
</math>
<listOfParameters>
<parameter id="LOWER_BOUND" value="-1000" units="mmol_per_gDW_per_hr" constant="true"/>
<parameter id="UPPER_BOUND" value="1000" units="mmol_per_gDW_per_hr" constant="true"/>
<parameter id="FLUX_VALUE" value="0" units="mmol_per_gDW_per_hr" constant="true"/>
<parameter id="OBJECTIVE_COEFFICIENT" value="0" units="mmol_per_gDW_per_hr" constant="true"/>
</listOfParameters>
</kineticLaw>
</reaction>
<reaction id="R_R095" name="1-(5&apos;-Phosphoribosyl)-5-amino-4-(N-succinocarboxamide)-imidazole AMP-lyase" reversible="true">
<notes>
<body xmlns="http://www.w3.org/1999/xhtml">
```

```
<p>GENE_ASSOCIATION: TTC1149</p>
<p>SUBSYSTEM: Nucleotide Salvage Pathway</p>
<p>EC Number: 4.3.2.2</p>
<p>Confidence Level: 1</p>
<p>AUTHORS: Nucleotides metabolism</p>
<p/>
</body>
</notes>
<listOfReactants>
<speciesReference species="M_SAICAR_c" stoichiometry="1"/>
</listOfReactants>
<listOfProducts>
<speciesReference species="M_FUM_c" stoichiometry="1"/>
<speciesReference species="M_AICAR_c" stoichiometry="1"/>
</listOfProducts>
<kineticLaw>
<math xmlns="http://www.w3.org/1998/Math/MathML">
<ci> FLUX_VALUE </ci>
</math>
<listOfParameters>
<parameter id="LOWER_BOUND" value="-1000" units="mmol_per_gDW_per_hr" constant="true"/>
<parameter id="UPPER_BOUND" value="1000" units="mmol_per_gDW_per_hr" constant="true"/>
<parameter id="FLUX_VALUE" value="0" units="mmol_per_gDW_per_hr" constant="true"/>
<parameter id="OBJECTIVE_COEFFICIENT" value="0" units="mmol_per_gDW_per_hr" constant="true"/>
</listOfParameters>
</kineticLaw>
</reaction>
<reaction id="R_R096" name="1-(5&apos;-Phosphoribosyl)-5-amino-4-imidazolecarboxamide:pyrophosphate phosphoribosyltransferase" reversible="true">
```

```xml
<notes>
  <body xmlns="http://www.w3.org/1999/xhtml">
    <p>GENE_ASSOCIATION: (TTC1249 or TTC1250)</p>
    <p>SUBSYSTEM: Nucleotide Salvage Pathway</p>
    <p>EC Number: 2.4.2.7</p>
    <p>Confidence Level: 1</p>
    <p>AUTHORS: Nucleotides metabolism</p>
    <p/>
  </body>
</notes>
<listOfReactants>
  <speciesReference species="M_PPi_c" stoichiometry="1"/>
  <speciesReference species="M_AICAR_c" stoichiometry="1"/>
</listOfReactants>
<listOfProducts>
  <speciesReference species="M_PRPP_c" stoichiometry="1"/>
  <speciesReference species="M_5A_4IMDZCARBA_c" stoichiometry="1"/>
</listOfProducts>
<kineticLaw>
  <math xmlns="http://www.w3.org/1998/Math/MathML">
    <ci> FLUX_VALUE </ci>
  </math>
  <listOfParameters>
    <parameter id="LOWER_BOUND" value="-1000" units="mmol_per_gDW_per_hr" constant="true"/>
    <parameter id="UPPER_BOUND" value="1000" units="mmol_per_gDW_per_hr" constant="true"/>
    <parameter id="FLUX_VALUE" value="0" units="mmol_per_gDW_per_hr" constant="true"/>
    <parameter id="OBJECTIVE_COEFFICIENT" value="0" units="mmol_per_gDW_per_hr" constant="true"/>
  </listOfParameters>
</kineticLaw>
```

```xml
</reaction>

<reaction id="R_R097" name="10-Formyltetrahydrofolate:5&apos;-phosphoribosyl-5-amino-4-imidazolecarboxamide formyltransferase" reversible="true">

<notes>

<body xmlns="http://www.w3.org/1999/xhtml">

<p>GENE_ASSOCIATION: TTC0561</p>

<p>SUBSYSTEM: Nucleotide Salvage Pathway</p>

<p>EC Number: 2.1.2.3</p>

<p>Confidence Level: 1</p>

<p>AUTHORS: Nucleotides metabolism</p>

<p/>

</body>

</notes>

<listOfReactants>

<speciesReference species="M_AICAR_c" stoichiometry="1"/>

<speciesReference species="M_10F_THF_c" stoichiometry="1"/>

</listOfReactants>

<listOfProducts>

<speciesReference species="M_THF_c" stoichiometry="1"/>

<speciesReference species="M_FAICAR_c" stoichiometry="1"/>

</listOfProducts>

<kineticLaw>

<math xmlns="http://www.w3.org/1998/Math/MathML">

<ci> FLUX_VALUE </ci>

</math>

<listOfParameters>

<parameter id="LOWER_BOUND" value="-1000" units="mmol_per_gDW_per_hr" constant="true"/>

<parameter id="UPPER_BOUND" value="1000" units="mmol_per_gDW_per_hr" constant="true"/>

<parameter id="FLUX_VALUE" value="0" units="mmol_per_gDW_per_hr" constant="true"/>
```

```
      <parameter id="OBJECTIVE_COEFFICIENT" value="0" units="mmol_per_gDW_per_hr" constant="true"/>
    </listOfParameters>
  </kineticLaw>
</reaction>
<reaction id="R_R098" name="IMP 1,2-hydrolase (decyclizing)" reversible="true">
  <notes>
    <body xmlns="http://www.w3.org/1999/xhtml">
      <p>GENE_ASSOCIATION: TTC0561</p>
      <p>SUBSYSTEM: Nucleotide Salvage Pathway</p>
      <p>EC Number: 3.5.4.10</p>
      <p>Confidence Level: 1</p>
      <p>AUTHORS: Nucleotides metabolism</p>
      <p/>
    </body>
  </notes>
  <listOfReactants>
    <speciesReference species="M_H2O_c" stoichiometry="1"/>
    <speciesReference species="M_IMP_c" stoichiometry="1"/>
  </listOfReactants>
  <listOfProducts>
    <speciesReference species="M_FAICAR_c" stoichiometry="1"/>
  </listOfProducts>
  <kineticLaw>
    <math xmlns="http://www.w3.org/1998/Math/MathML">
      <ci> FLUX_VALUE </ci>
    </math>
    <listOfParameters>
      <parameter id="LOWER_BOUND" value="-1000" units="mmol_per_gDW_per_hr" constant="true"/>
      <parameter id="UPPER_BOUND" value="1000" units="mmol_per_gDW_per_hr" constant="true"/>
```

```xml
      <parameter id="FLUX_VALUE" value="0" units="mmol_per_gDW_per_hr" constant="true"/>
      <parameter id="OBJECTIVE_COEFFICIENT" value="0" units="mmol_per_gDW_per_hr" constant="true"/>
    </listOfParameters>
  </kineticLaw>
</reaction>
<reaction id="R_R099" name="P1,P4-bis(5&apos;-adenosyl)-tetraphosphate adenylohydrolase" reversible="false">
  <notes>
    <body xmlns="http://www.w3.org/1999/xhtml">
      <p>GENE_ASSOCIATION: (TTC1519 or TTC1859)</p>
      <p>SUBSYSTEM: Nucleotide Salvage Pathway</p>
      <p>EC Number: 3.6.1.17</p>
      <p>Confidence Level: 1</p>
      <p>AUTHORS: Nucleotides metabolism</p>
      <p/>
    </body>
  </notes>
  <listOfReactants>
    <speciesReference species="M_H2O_c" stoichiometry="1"/>
    <speciesReference species="M_BADNS_PPP_c" stoichiometry="1"/>
  </listOfReactants>
  <listOfProducts>
    <speciesReference species="M_ATP_c" stoichiometry="1"/>
    <speciesReference species="M_AMP_c" stoichiometry="1"/>
  </listOfProducts>
  <kineticLaw>
    <math xmlns="http://www.w3.org/1998/Math/MathML">
      <ci> FLUX_VALUE </ci>
    </math>
    <listOfParameters>
```

```
      <parameter id="LOWER_BOUND" value="0" units="mmol_per_gDW_per_hr" constant="true"/>
      <parameter id="UPPER_BOUND" value="1000" units="mmol_per_gDW_per_hr" constant="true"/>
      <parameter id="FLUX_VALUE" value="0" units="mmol_per_gDW_per_hr" constant="true"/>
      <parameter id="OBJECTIVE_COEFFICIENT" value="0" units="mmol_per_gDW_per_hr" constant="true"/>
    </listOfParameters>
  </kineticLaw>
</reaction>
<reaction id="R_R100" name="ATP:sulfate adenylyltransferase" reversible="false">
  <notes>
    <body xmlns="http://www.w3.org/1999/xhtml">
      <p>GENE_ASSOCIATION: TTC0307</p>
      <p>SUBSYSTEM: Nucleotide Salvage Pathway</p>
      <p>EC Number: 2.7.7.4</p>
      <p>Confidence Level: 1</p>
      <p>AUTHORS: Nucleotides metabolism</p>
      <p/>
    </body>
  </notes>
  <listOfReactants>
    <speciesReference species="M_ATP_c" stoichiometry="1"/>
    <speciesReference species="M_H_c" stoichiometry="1"/>
    <speciesReference species="M_SO4_c" stoichiometry="1"/>
  </listOfReactants>
  <listOfProducts>
    <speciesReference species="M_PPi_c" stoichiometry="1"/>
    <speciesReference species="M_APS_c" stoichiometry="1"/>
  </listOfProducts>
  <kineticLaw>
    <math xmlns="http://www.w3.org/1998/Math/MathML">
```

```
      <ci> FLUX_VALUE </ci>
    </math>
    <listOfParameters>
      <parameter id="LOWER_BOUND" value="0" units="mmol_per_gDW_per_hr" constant="true"/>
      <parameter id="UPPER_BOUND" value="1000" units="mmol_per_gDW_per_hr" constant="true"/>
      <parameter id="FLUX_VALUE" value="0" units="mmol_per_gDW_per_hr" constant="true"/>
      <parameter id="OBJECTIVE_COEFFICIENT" value="0" units="mmol_per_gDW_per_hr" constant="true"/>
    </listOfParameters>
  </kineticLaw>
</reaction>
<reaction id="R_R101" name="ATP:dIDP phosphotransferase" reversible="true">
  <notes>
    <body xmlns="http://www.w3.org/1999/xhtml">
      <p>GENE_ASSOCIATION: TTC1798</p>
      <p>SUBSYSTEM: Nucleotide Salvage Pathway</p>
      <p>EC Number: 2.7.4.6</p>
      <p>Confidence Level: 1</p>
      <p>AUTHORS: Nucleotides metabolism</p>
      <p/>
    </body>
  </notes>
  <listOfReactants>
    <speciesReference species="M_ATP_c" stoichiometry="1"/>
    <speciesReference species="M_dIDP_c" stoichiometry="1"/>
  </listOfReactants>
  <listOfProducts>
    <speciesReference species="M_ADP_c" stoichiometry="1"/>
    <speciesReference species="M_dITP_c" stoichiometry="1"/>
  </listOfProducts>
```

```
  <kineticLaw>
    <math xmlns="http://www.w3.org/1998/Math/MathML">
      <ci> FLUX_VALUE </ci>
    </math>
    <listOfParameters>
      <parameter id="LOWER_BOUND" value="-1000" units="mmol_per_gDW_per_hr" constant="true"/>
      <parameter id="UPPER_BOUND" value="1000" units="mmol_per_gDW_per_hr" constant="true"/>
      <parameter id="FLUX_VALUE" value="0" units="mmol_per_gDW_per_hr" constant="true"/>
      <parameter id="OBJECTIVE_COEFFICIENT" value="0" units="mmol_per_gDW_per_hr" constant="true"/>
    </listOfParameters>
  </kineticLaw>
</reaction>
<reaction id="R_R102" name="2&apos;-Deoxyinosine-5&apos;-triphosphate pyrophosphohydrolase" reversible="false">
  <notes>
    <body xmlns="http://www.w3.org/1999/xhtml">
      <p>GENE_ASSOCIATION: (TTC1290 or TTC1583)</p>
      <p>SUBSYSTEM: Nucleotide Salvage Pathway</p>
      <p>EC Number: 3.6.1.19</p>
      <p>Confidence Level: 1</p>
      <p>AUTHORS: Nucleotides metabolism</p>
      <p/>
    </body>
  </notes>
  <listOfReactants>
    <speciesReference species="M_H2O_c" stoichiometry="1"/>
    <speciesReference species="M_dITP_c" stoichiometry="1"/>
  </listOfReactants>
  <listOfProducts>
    <speciesReference species="M_PPi_c" stoichiometry="1"/>
```

```
        <speciesReference species="M_H_c" stoichiometry="1"/>
        <speciesReference species="M_dIMP_c" stoichiometry="1"/>
      </listOfProducts>
      <kineticLaw>
        <math xmlns="http://www.w3.org/1998/Math/MathML">
          <ci> FLUX_VALUE </ci>
        </math>
        <listOfParameters>
          <parameter id="LOWER_BOUND" value="0" units="mmol_per_gDW_per_hr" constant="true"/>
          <parameter id="UPPER_BOUND" value="1000" units="mmol_per_gDW_per_hr" constant="true"/>
          <parameter id="FLUX_VALUE" value="0" units="mmol_per_gDW_per_hr" constant="true"/>
          <parameter id="OBJECTIVE_COEFFICIENT" value="0" units="mmol_per_gDW_per_hr" constant="true"/>
        </listOfParameters>
      </kineticLaw>
    </reaction>
    <reaction id="R_R103" name="guanosine 3&apos;-diphosphate 5&apos;-triphosphate 5&apos;-phosphohydrolase"
reversible="false">
      <notes>
        <body xmlns="http://www.w3.org/1999/xhtml">
          <p>GENE_ASSOCIATION: TTC0636</p>
          <p>SUBSYSTEM: Nucleotide Salvage Pathway</p>
          <p>EC Number: 3.6.1.40</p>
          <p>Confidence Level: 1</p>
          <p>AUTHORS: Nucleotides metabolism</p>
          <p/>
        </body>
      </notes>
      <listOfReactants>
        <speciesReference species="M_H2O_c" stoichiometry="1"/>
```

```
    <speciesReference species="M_GUNS_3PPi_5TPi_c" stoichiometry="1"/>
  </listOfReactants>
  <listOfProducts>
    <speciesReference species="M_Pi_c" stoichiometry="1"/>
    <speciesReference species="M_H_c" stoichiometry="1"/>
    <speciesReference species="M_GUNSBP_c" stoichiometry="1"/>
  </listOfProducts>
  <kineticLaw>
    <math xmlns="http://www.w3.org/1998/Math/MathML">
      <ci> FLUX_VALUE </ci>
    </math>
    <listOfParameters>
      <parameter id="LOWER_BOUND" value="0" units="mmol_per_gDW_per_hr" constant="true"/>
      <parameter id="UPPER_BOUND" value="1000" units="mmol_per_gDW_per_hr" constant="true"/>
      <parameter id="FLUX_VALUE" value="0" units="mmol_per_gDW_per_hr" constant="true"/>
      <parameter id="OBJECTIVE_COEFFICIENT" value="0" units="mmol_per_gDW_per_hr" constant="true"/>
    </listOfParameters>
  </kineticLaw>
</reaction>
<reaction id="R_R104" name="P1,P4-bis(5&apos;-guanosyl)-tetraphosphate guanylylhydrolase" reversible="false">
  <notes>
    <body xmlns="http://www.w3.org/1999/xhtml">
      <p>GENE_ASSOCIATION: (TTC1519 or TTC1859)</p>
      <p>SUBSYSTEM: Nucleotide Salvage Pathway</p>
      <p>EC Number: 3.6.1.17</p>
      <p>Confidence Level: 1</p>
      <p>AUTHORS: Nucleotides metabolism</p>
      <p/>
    </body>
```

```xml
  </notes>
  <listOfReactants>
    <speciesReference species="M_GTP_c" stoichiometry="1"/>
    <speciesReference species="M_GMP_c" stoichiometry="1"/>
  </listOfReactants>
  <listOfProducts>
    <speciesReference species="M_H2O_c" stoichiometry="1"/>
    <speciesReference species="M_H_c" stoichiometry="2"/>
    <speciesReference species="M_BGUNS_PPP_c" stoichiometry="1"/>
  </listOfProducts>
  <kineticLaw>
    <math xmlns="http://www.w3.org/1998/Math/MathML">
      <ci> FLUX_VALUE </ci>
    </math>
    <listOfParameters>
      <parameter id="LOWER_BOUND" value="0" units="mmol_per_gDW_per_hr" constant="true"/>
      <parameter id="UPPER_BOUND" value="1000" units="mmol_per_gDW_per_hr" constant="true"/>
      <parameter id="FLUX_VALUE" value="0" units="mmol_per_gDW_per_hr" constant="true"/>
      <parameter id="OBJECTIVE_COEFFICIENT" value="0" units="mmol_per_gDW_per_hr" constant="true"/>
    </listOfParameters>
  </kineticLaw>
</reaction>
<reaction id="R_R105" name="dGTP triphosphohydrolase" reversible="false">
  <notes>
    <body xmlns="http://www.w3.org/1999/xhtml">
      <p>GENE_ASSOCIATION: TTC0044</p>
      <p>SUBSYSTEM: Nucleotide Salvage Pathway</p>
      <p>EC Number: 3.1.5.1</p>
      <p>Confidence Level: 1</p>
```

```
          <p>AUTHORS: Nucleotides metabolism</p>
          <p/>
        </body>
      </notes>
      <listOfReactants>
        <speciesReference species="M_dGTP_c" stoichiometry="1"/>
        <speciesReference species="M_H2O_c" stoichiometry="1"/>
      </listOfReactants>
      <listOfProducts>
        <speciesReference species="M_dGUNS_c" stoichiometry="1"/>
        <speciesReference species="M_TPi_c" stoichiometry="1"/>
      </listOfProducts>
      <kineticLaw>
        <math xmlns="http://www.w3.org/1998/Math/MathML">
          <ci> FLUX_VALUE </ci>
        </math>
        <listOfParameters>
          <parameter id="LOWER_BOUND" value="0" units="mmol_per_gDW_per_hr" constant="true"/>
          <parameter id="UPPER_BOUND" value="1000" units="mmol_per_gDW_per_hr" constant="true"/>
          <parameter id="FLUX_VALUE" value="0" units="mmol_per_gDW_per_hr" constant="true"/>
          <parameter id="OBJECTIVE_COEFFICIENT" value="0" units="mmol_per_gDW_per_hr" constant="true"/>
        </listOfParameters>
      </kineticLaw>
    </reaction>
    <reaction id="R_R106" name="guanosine-3&apos;,5&apos;-bis(diphosphate) 3&apos;-diphosphohydrolase"
reversible="false">
      <notes>
        <body xmlns="http://www.w3.org/1999/xhtml">
          <p>GENE_ASSOCIATION: TTC1355</p>
```

```
      <p>SUBSYSTEM: Nucleotide Salvage Pathway</p>
      <p>EC Number: 3.1.7.2</p>
      <p>Confidence Level: 1</p>
      <p>AUTHORS: Nucleotides metabolism</p>
      <p/>
    </body>
  </notes>
  <listOfReactants>
    <speciesReference species="M_H2O_c" stoichiometry="1"/>
    <speciesReference species="M_GUNSBP_c" stoichiometry="1"/>
  </listOfReactants>
  <listOfProducts>
    <speciesReference species="M_PPi_c" stoichiometry="1"/>
    <speciesReference species="M_GDP_c" stoichiometry="1"/>
  </listOfProducts>
  <kineticLaw>
    <math xmlns="http://www.w3.org/1998/Math/MathML">
      <ci> FLUX_VALUE </ci>
    </math>
    <listOfParameters>
      <parameter id="LOWER_BOUND" value="0" units="mmol_per_gDW_per_hr" constant="true"/>
      <parameter id="UPPER_BOUND" value="1000" units="mmol_per_gDW_per_hr" constant="true"/>
      <parameter id="FLUX_VALUE" value="0" units="mmol_per_gDW_per_hr" constant="true"/>
      <parameter id="OBJECTIVE_COEFFICIENT" value="0" units="mmol_per_gDW_per_hr" constant="true"/>
    </listOfParameters>
  </kineticLaw>
</reaction>
<reaction id="R_R107" name="ATP:GDP phosphotransferase" reversible="false">
  <notes>
```

```
   <body xmlns="http://www.w3.org/1999/xhtml">
    <p>GENE_ASSOCIATION: TTC1798</p>
    <p>SUBSYSTEM: Nucleotide Salvage Pathway</p>
    <p>EC Number: 2.7.4.6</p>
    <p>Confidence Level: 1</p>
    <p>AUTHORS: Nucleotides metabolism</p>
    <p/>
   </body>
  </notes>
  <listOfReactants>
   <speciesReference species="M_ATP_c" stoichiometry="1"/>
   <speciesReference species="M_GDP_c" stoichiometry="1"/>
  </listOfReactants>
  <listOfProducts>
   <speciesReference species="M_GTP_c" stoichiometry="1"/>
   <speciesReference species="M_ADP_c" stoichiometry="1"/>
  </listOfProducts>
  <kineticLaw>
   <math xmlns="http://www.w3.org/1998/Math/MathML">
    <ci> FLUX_VALUE </ci>
   </math>
   <listOfParameters>
    <parameter id="LOWER_BOUND" value="0" units="mmol_per_gDW_per_hr" constant="true"/>
    <parameter id="UPPER_BOUND" value="1000" units="mmol_per_gDW_per_hr" constant="true"/>
    <parameter id="FLUX_VALUE" value="0" units="mmol_per_gDW_per_hr" constant="true"/>
    <parameter id="OBJECTIVE_COEFFICIENT" value="0" units="mmol_per_gDW_per_hr" constant="true"/>
   </listOfParameters>
  </kineticLaw>
</reaction>
```

```
<reaction id="R_R108" name="ATP:dGDP phosphotransferase" reversible="true">
 <notes>
  <body xmlns="http://www.w3.org/1999/xhtml">
   <p>GENE_ASSOCIATION: TTC1798</p>
   <p>SUBSYSTEM: Nucleotide Salvage Pathway</p>
   <p>EC Number: 2.7.4.6</p>
   <p>Confidence Level: 1</p>
   <p>AUTHORS: Nucleotides metabolism</p>
   <p/>
  </body>
 </notes>
 <listOfReactants>
  <speciesReference species="M_ATP_c" stoichiometry="1"/>
  <speciesReference species="M_dGDP_c" stoichiometry="1"/>
 </listOfReactants>
 <listOfProducts>
  <speciesReference species="M_dGTP_c" stoichiometry="1"/>
  <speciesReference species="M_ADP_c" stoichiometry="1"/>
 </listOfProducts>
 <kineticLaw>
  <math xmlns="http://www.w3.org/1998/Math/MathML">
   <ci> FLUX_VALUE </ci>
  </math>
  <listOfParameters>
   <parameter id="LOWER_BOUND" value="0" units="mmol_per_gDW_per_hr" constant="true"/>
   <parameter id="UPPER_BOUND" value="0" units="mmol_per_gDW_per_hr" constant="true"/>
   <parameter id="FLUX_VALUE" value="0" units="mmol_per_gDW_per_hr" constant="true"/>
   <parameter id="OBJECTIVE_COEFFICIENT" value="0" units="mmol_per_gDW_per_hr" constant="true"/>
  </listOfParameters>
```

```
  </kineticLaw>
</reaction>
<reaction id="R_R109" name="dGTP:pyruvate 2-O-phosphotransferase" reversible="false">
  <notes>
    <body xmlns="http://www.w3.org/1999/xhtml">
      <p>GENE_ASSOCIATION: TTC1611</p>
      <p>SUBSYSTEM: Nucleotide Salvage Pathway</p>
      <p>EC Number: 2.7.1.40</p>
      <p>Confidence Level: 1</p>
      <p>AUTHORS: Nucleotides metabolism</p>
      <p/>
    </body>
  </notes>
  <listOfReactants>
    <speciesReference species="M_PEP_c" stoichiometry="1"/>
    <speciesReference species="M_dGDP_c" stoichiometry="1"/>
  </listOfReactants>
  <listOfProducts>
    <speciesReference species="M_dGTP_c" stoichiometry="1"/>
    <speciesReference species="M_PYR_c" stoichiometry="1"/>
  </listOfProducts>
  <kineticLaw>
    <math xmlns="http://www.w3.org/1998/Math/MathML">
      <ci> FLUX_VALUE </ci>
    </math>
    <listOfParameters>
      <parameter id="LOWER_BOUND" value="0" units="mmol_per_gDW_per_hr" constant="true"/>
      <parameter id="UPPER_BOUND" value="1000" units="mmol_per_gDW_per_hr" constant="true"/>
      <parameter id="FLUX_VALUE" value="0" units="mmol_per_gDW_per_hr" constant="true"/>
```

```xml
<parameter id="OBJECTIVE_COEFFICIENT" value="0" units="mmol_per_gDW_per_hr" constant="true"/>
</listOfParameters>
</kineticLaw>
</reaction>
<reaction id="R_R110" name="2&apos;-Deoxyguanosine 5&apos;-triphosphate diphosphohydrolase" reversible="false">
<notes>
<body xmlns="http://www.w3.org/1999/xhtml">
<p>GENE_ASSOCIATION: (TTC1290 or TTC1583)</p>
<p>SUBSYSTEM: Nucleotide Salvage Pathway</p>
<p>EC Number: 3.6.1.19</p>
<p>Confidence Level: 1</p>
<p>AUTHORS: Nucleotides metabolism</p>
<p/>
</body>
</notes>
<listOfReactants>
<speciesReference species="M_dGTP_c" stoichiometry="1"/>
<speciesReference species="M_H2O_c" stoichiometry="1"/>
</listOfReactants>
<listOfProducts>
<speciesReference species="M_PPi_c" stoichiometry="1"/>
<speciesReference species="M_H_c" stoichiometry="1"/>
<speciesReference species="M_dGMP_c" stoichiometry="1"/>
</listOfProducts>
<kineticLaw>
<math xmlns="http://www.w3.org/1998/Math/MathML">
<ci> FLUX_VALUE </ci>
</math>
<listOfParameters>
```

```xml
      <parameter id="LOWER_BOUND" value="0" units="mmol_per_gDW_per_hr" constant="true"/>
      <parameter id="UPPER_BOUND" value="1000" units="mmol_per_gDW_per_hr" constant="true"/>
      <parameter id="FLUX_VALUE" value="0" units="mmol_per_gDW_per_hr" constant="true"/>
      <parameter id="OBJECTIVE_COEFFICIENT" value="0" units="mmol_per_gDW_per_hr" constant="true"/>
    </listOfParameters>
  </kineticLaw>
</reaction>
<reaction id="R_R111" name="2&apos;-Deoxyguanosine 5&apos;-monophosphate phosphohydrolase" reversible="false">
  <notes>
    <body xmlns="http://www.w3.org/1999/xhtml">
      <p>GENE_ASSOCIATION: (TTC0964 or TTC1625 or TT_P0028)</p>
      <p>SUBSYSTEM: Nucleotide Salvage Pathway</p>
      <p>EC Number: 3.1.3.5</p>
      <p>Confidence Level: 1</p>
      <p>AUTHORS: Nucleotides metabolism</p>
      <p/>
    </body>
  </notes>
  <listOfReactants>
    <speciesReference species="M_H2O_c" stoichiometry="1"/>
    <speciesReference species="M_dGMP_c" stoichiometry="1"/>
  </listOfReactants>
  <listOfProducts>
    <speciesReference species="M_Pi_c" stoichiometry="1"/>
    <speciesReference species="M_dGUNS_c" stoichiometry="1"/>
  </listOfProducts>
  <kineticLaw>
    <math xmlns="http://www.w3.org/1998/Math/MathML">
      <ci> FLUX_VALUE </ci>
```

```
      </math>
      <listOfParameters>
        <parameter id="LOWER_BOUND" value="0" units="mmol_per_gDW_per_hr" constant="true"/>
        <parameter id="UPPER_BOUND" value="1000" units="mmol_per_gDW_per_hr" constant="true"/>
        <parameter id="FLUX_VALUE" value="0" units="mmol_per_gDW_per_hr" constant="true"/>
        <parameter id="OBJECTIVE_COEFFICIENT" value="0" units="mmol_per_gDW_per_hr" constant="true"/>
      </listOfParameters>
    </kineticLaw>
  </reaction>
  <reaction id="R_R112" name="2&apos;-Deoxyguanosine 5&apos;-diphosphate:oxidized-thioredoxin 2&apos;-
oxidoreductase" reversible="false">
    <notes>
      <body xmlns="http://www.w3.org/1999/xhtml">
        <p>GENE_ASSOCIATION: (TTC1930 and TT_P0161 and TT_P0162)</p>
        <p>SUBSYSTEM: Nucleotide Salvage Pathway</p>
        <p>EC Number: 1.17.4.1</p>
        <p>Confidence Level: 1</p>
        <p>AUTHORS: Nucleotides metabolism</p>
        <p/>
      </body>
    </notes>
    <listOfReactants>
      <speciesReference species="M_GDP_c" stoichiometry="1"/>
      <speciesReference species="M_R_TRED_c" stoichiometry="1"/>
    </listOfReactants>
    <listOfProducts>
      <speciesReference species="M_H2O_c" stoichiometry="1"/>
      <speciesReference species="M_dGDP_c" stoichiometry="1"/>
      <speciesReference species="M_O_TRED_c" stoichiometry="1"/>
```

```
</listOfProducts>
<kineticLaw>
  <math xmlns="http://www.w3.org/1998/Math/MathML">
    <ci> FLUX_VALUE </ci>
  </math>
  <listOfParameters>
    <parameter id="LOWER_BOUND" value="0" units="mmol_per_gDW_per_hr" constant="true"/>
    <parameter id="UPPER_BOUND" value="1000" units="mmol_per_gDW_per_hr" constant="true"/>
    <parameter id="FLUX_VALUE" value="0" units="mmol_per_gDW_per_hr" constant="true"/>
    <parameter id="OBJECTIVE_COEFFICIENT" value="0" units="mmol_per_gDW_per_hr" constant="true"/>
  </listOfParameters>
</kineticLaw>
</reaction>
<reaction id="R_R113" name="ATP:dGMP phosphotransferase" reversible="true">
<notes>
  <body xmlns="http://www.w3.org/1999/xhtml">
    <p>GENE_ASSOCIATION: TTC1197</p>
    <p>SUBSYSTEM: Nucleotide Salvage Pathway</p>
    <p>EC Number: 2.7.4.8</p>
    <p>Confidence Level: 1</p>
    <p>AUTHORS: Nucleotides metabolism</p>
    <p/>
  </body>
</notes>
<listOfReactants>
  <speciesReference species="M_ATP_c" stoichiometry="1"/>
  <speciesReference species="M_dGMP_c" stoichiometry="1"/>
</listOfReactants>
<listOfProducts>
```

```
    <speciesReference species="M_ADP_c" stoichiometry="1"/>
    <speciesReference species="M_dGDP_c" stoichiometry="1"/>
  </listOfProducts>
  <kineticLaw>
    <math xmlns="http://www.w3.org/1998/Math/MathML">
      <ci> FLUX_VALUE </ci>
    </math>
    <listOfParameters>
      <parameter id="LOWER_BOUND" value="-1000" units="mmol_per_gDW_per_hr" constant="true"/>
      <parameter id="UPPER_BOUND" value="1000" units="mmol_per_gDW_per_hr" constant="true"/>
      <parameter id="FLUX_VALUE" value="0" units="mmol_per_gDW_per_hr" constant="true"/>
      <parameter id="OBJECTIVE_COEFFICIENT" value="0" units="mmol_per_gDW_per_hr" constant="true"/>
    </listOfParameters>
  </kineticLaw>
</reaction>
<reaction id="R_R114" name="Deoxyguanosine:orthophosphate ribosyltransferase" reversible="true">
  <notes>
    <body xmlns="http://www.w3.org/1999/xhtml">
      <p>GENE_ASSOCIATION: ((TTC0194 or TTC1070) or TTC1412)</p>
      <p>SUBSYSTEM: Nucleotide Salvage Pathway</p>
      <p>EC Number: 2.4.2.1 or 2.4.2.4</p>
      <p>Confidence Level: 1</p>
      <p>AUTHORS: Nucleotides metabolism</p>
      <p/>
    </body>
  </notes>
  <listOfReactants>
    <speciesReference species="M_Pi_c" stoichiometry="1"/>
    <speciesReference species="M_dGUNS_c" stoichiometry="1"/>
```

```
  </listOfReactants>
  <listOfProducts>
   <speciesReference species="M_dR1P_c" stoichiometry="1"/>
   <speciesReference species="M_GUN_c" stoichiometry="1"/>
  </listOfProducts>
  <kineticLaw>
   <math xmlns="http://www.w3.org/1998/Math/MathML">
    <ci> FLUX_VALUE </ci>
   </math>
   <listOfParameters>
    <parameter id="LOWER_BOUND" value="-1000" units="mmol_per_gDW_per_hr" constant="true"/>
    <parameter id="UPPER_BOUND" value="1000" units="mmol_per_gDW_per_hr" constant="true"/>
    <parameter id="FLUX_VALUE" value="0" units="mmol_per_gDW_per_hr" constant="true"/>
    <parameter id="OBJECTIVE_COEFFICIENT" value="0" units="mmol_per_gDW_per_hr" constant="true"/>
   </listOfParameters>
  </kineticLaw>
 </reaction>
 <reaction id="R_R115" name="GTP diphosphohydrolase (diphosphate-forming)" reversible="false">
  <notes>
   <body xmlns="http://www.w3.org/1999/xhtml">
    <p>GENE_ASSOCIATION: (TTC1290 or TTC1583)</p>
    <p>SUBSYSTEM: Nucleotide Salvage Pathway</p>
    <p>EC Number: 3.6.1.19</p>
    <p>Confidence Level: 1</p>
    <p>AUTHORS: Nucleotides metabolism</p>
    <p/>
   </body>
  </notes>
  <listOfReactants>
```

```xml
    <speciesReference species="M_GTP_c" stoichiometry="1"/>
    <speciesReference species="M_H2O_c" stoichiometry="1"/>
  </listOfReactants>
  <listOfProducts>
    <speciesReference species="M_PPi_c" stoichiometry="1"/>
    <speciesReference species="M_H_c" stoichiometry="1"/>
    <speciesReference species="M_GMP_c" stoichiometry="1"/>
  </listOfProducts>
  <kineticLaw>
    <math xmlns="http://www.w3.org/1998/Math/MathML">
      <ci> FLUX_VALUE </ci>
    </math>
    <listOfParameters>
      <parameter id="LOWER_BOUND" value="0" units="mmol_per_gDW_per_hr" constant="true"/>
      <parameter id="UPPER_BOUND" value="1000" units="mmol_per_gDW_per_hr" constant="true"/>
      <parameter id="FLUX_VALUE" value="0" units="mmol_per_gDW_per_hr" constant="true"/>
      <parameter id="OBJECTIVE_COEFFICIENT" value="0" units="mmol_per_gDW_per_hr" constant="true"/>
    </listOfParameters>
  </kineticLaw>
</reaction>
<reaction id="R_R116" name="ATP:GMP phosphotransferase" reversible="true">
  <notes>
    <body xmlns="http://www.w3.org/1999/xhtml">
      <p>GENE_ASSOCIATION: TTC1197</p>
      <p>SUBSYSTEM: Nucleotide Salvage Pathway</p>
      <p>EC Number: 2.7.4.8</p>
      <p>Confidence Level: 1</p>
      <p>AUTHORS: Nucleotides metabolism</p>
      <p/>
```

```
    </body>
  </notes>
  <listOfReactants>
    <speciesReference species="M_ATP_c" stoichiometry="1"/>
    <speciesReference species="M_GMP_c" stoichiometry="1"/>
  </listOfReactants>
  <listOfProducts>
    <speciesReference species="M_ADP_c" stoichiometry="1"/>
    <speciesReference species="M_GDP_c" stoichiometry="1"/>
  </listOfProducts>
  <kineticLaw>
    <math xmlns="http://www.w3.org/1998/Math/MathML">
      <ci> FLUX_VALUE </ci>
    </math>
    <listOfParameters>
      <parameter id="LOWER_BOUND" value="-1000" units="mmol_per_gDW_per_hr" constant="true"/>
      <parameter id="UPPER_BOUND" value="1000" units="mmol_per_gDW_per_hr" constant="true"/>
      <parameter id="FLUX_VALUE" value="0" units="mmol_per_gDW_per_hr" constant="true"/>
      <parameter id="OBJECTIVE_COEFFICIENT" value="0" units="mmol_per_gDW_per_hr" constant="true"/>
    </listOfParameters>
  </kineticLaw>
</reaction>
<reaction id="R_R117" name="Guanosine 5&apos;-monophosphate phosphohydrolase" reversible="false">
  <notes>
    <body xmlns="http://www.w3.org/1999/xhtml">
      <p>GENE_ASSOCIATION: (TTC0964 or TTC1625 or TT_P0028)</p>
      <p>SUBSYSTEM: Nucleotide Salvage Pathway</p>
      <p>EC Number: 3.1.3.5</p>
      <p>Confidence Level: 1</p>
```

```
      <p>AUTHORS: Nucleotides metabolism</p>
      <p/>
    </body>
  </notes>
  <listOfReactants>
    <speciesReference species="M_H2O_c" stoichiometry="1"/>
    <speciesReference species="M_GMP_c" stoichiometry="1"/>
  </listOfReactants>
  <listOfProducts>
    <speciesReference species="M_Pi_c" stoichiometry="1"/>
    <speciesReference species="M_GUNS_c" stoichiometry="1"/>
  </listOfProducts>
  <kineticLaw>
    <math xmlns="http://www.w3.org/1998/Math/MathML">
      <ci> FLUX_VALUE </ci>
    </math>
    <listOfParameters>
      <parameter id="LOWER_BOUND" value="0" units="mmol_per_gDW_per_hr" constant="true"/>
      <parameter id="UPPER_BOUND" value="1000" units="mmol_per_gDW_per_hr" constant="true"/>
      <parameter id="FLUX_VALUE" value="0" units="mmol_per_gDW_per_hr" constant="true"/>
      <parameter id="OBJECTIVE_COEFFICIENT" value="0" units="mmol_per_gDW_per_hr" constant="true"/>
    </listOfParameters>
  </kineticLaw>
</reaction>
<reaction id="R_R118" name="guanosine:phosphate alpha-D-ribosyltransferase" reversible="true">
  <notes>
    <body xmlns="http://www.w3.org/1999/xhtml">
      <p>GENE_ASSOCIATION: (TTC0194 or TTC1070)</p>
      <p>SUBSYSTEM: Nucleotide Salvage Pathway</p>
```

```xml
      <p>EC Number: 2.4.2.1</p>
      <p>Confidence Level: 1</p>
      <p>AUTHORS: Nucleotides metabolism</p>
      <p/>
    </body>
  </notes>
  <listOfReactants>
    <speciesReference species="M_Pi_c" stoichiometry="1"/>
    <speciesReference species="M_GUNS_c" stoichiometry="1"/>
  </listOfReactants>
  <listOfProducts>
    <speciesReference species="M_R1P_c" stoichiometry="1"/>
    <speciesReference species="M_GUN_c" stoichiometry="1"/>
  </listOfProducts>
  <kineticLaw>
    <math xmlns="http://www.w3.org/1998/Math/MathML">
      <ci> FLUX_VALUE </ci>
    </math>
    <listOfParameters>
      <parameter id="LOWER_BOUND" value="-1000" units="mmol_per_gDW_per_hr" constant="true"/>
      <parameter id="UPPER_BOUND" value="1000" units="mmol_per_gDW_per_hr" constant="true"/>
      <parameter id="FLUX_VALUE" value="0" units="mmol_per_gDW_per_hr" constant="true"/>
      <parameter id="OBJECTIVE_COEFFICIENT" value="0" units="mmol_per_gDW_per_hr" constant="true"/>
    </listOfParameters>
  </kineticLaw>
</reaction>
<reaction id="R_R119" name="GMP:diphosphate 5-phospho-alpha-D-ribosyltransferase" reversible="true">
  <notes>
    <body xmlns="http://www.w3.org/1999/xhtml">
```

```
      <p>GENE_ASSOCIATION: ((TTC1249 or TTC1250) or TTC1766)</p>
      <p>SUBSYSTEM: Nucleotide Salvage Pathway</p>
      <p>EC Number: 2.4.2.7 or 2.4.2.8</p>
      <p>Confidence Level: 1</p>
      <p>AUTHORS: Nucleotides metabolism</p>
      <p/>
    </body>
  </notes>
  <listOfReactants>
    <speciesReference species="M_PRPP_c" stoichiometry="1"/>
    <speciesReference species="M_GUN_c" stoichiometry="1"/>
  </listOfReactants>
  <listOfProducts>
    <speciesReference species="M_PPi_c" stoichiometry="1"/>
    <speciesReference species="M_GMP_c" stoichiometry="1"/>
  </listOfProducts>
  <kineticLaw>
    <math xmlns="http://www.w3.org/1998/Math/MathML">
      <ci> FLUX_VALUE </ci>
    </math>
    <listOfParameters>
      <parameter id="LOWER_BOUND" value="-1000" units="mmol_per_gDW_per_hr" constant="true"/>
      <parameter id="UPPER_BOUND" value="1000" units="mmol_per_gDW_per_hr" constant="true"/>
      <parameter id="FLUX_VALUE" value="0" units="mmol_per_gDW_per_hr" constant="true"/>
      <parameter id="OBJECTIVE_COEFFICIENT" value="0" units="mmol_per_gDW_per_hr" constant="true"/>
    </listOfParameters>
  </kineticLaw>
</reaction>
<reaction id="R_R120" name="Xanthosine-5&apos;-phosphate:ammonia ligase (AMP-forming)" reversible="false">
```

```
<notes>
  <body xmlns="http://www.w3.org/1999/xhtml">
    <p>GENE_ASSOCIATION: TTC1187</p>
    <p>SUBSYSTEM: Nucleotide Salvage Pathway</p>
    <p>EC Number: 6.3.5.2</p>
    <p>Confidence Level: 1</p>
    <p>AUTHORS: Nucleotides metabolism</p>
    <p/>
  </body>
</notes>
<listOfReactants>
  <speciesReference species="M_ATP_c" stoichiometry="1"/>
  <speciesReference species="M_XMP_c" stoichiometry="1"/>
  <speciesReference species="M_NH4_c" stoichiometry="1"/>
</listOfReactants>
<listOfProducts>
  <speciesReference species="M_PPi_c" stoichiometry="1"/>
  <speciesReference species="M_H_c" stoichiometry="2"/>
  <speciesReference species="M_AMP_c" stoichiometry="1"/>
  <speciesReference species="M_GMP_c" stoichiometry="1"/>
</listOfProducts>
<kineticLaw>
  <math xmlns="http://www.w3.org/1998/Math/MathML">
    <ci> FLUX_VALUE </ci>
  </math>
  <listOfParameters>
    <parameter id="LOWER_BOUND" value="0" units="mmol_per_gDW_per_hr" constant="true"/>
    <parameter id="UPPER_BOUND" value="1000" units="mmol_per_gDW_per_hr" constant="true"/>
    <parameter id="FLUX_VALUE" value="0" units="mmol_per_gDW_per_hr" constant="true"/>
```

```xml
<parameter id="OBJECTIVE_COEFFICIENT" value="0" units="mmol_per_gDW_per_hr" constant="true"/>
</listOfParameters>
</kineticLaw>
</reaction>
<reaction id="R_R121" name="P1,P4-Bis(5&apos;-nucleosyl)-tetraphosphate nucleotidohydrolase" reversible="false">
<notes>
<body xmlns="http://www.w3.org/1999/xhtml">
<p>GENE_ASSOCIATION: (TTC1519 or TTC1859)</p>
<p>SUBSYSTEM: Nucleotide Salvage Pathway</p>
<p>EC Number: 3.6.1.17</p>
<p>Confidence Level: 1</p>
<p>AUTHORS: Nucleotides metabolism</p>
<p/>
</body>
</notes>
<listOfReactants>
<speciesReference species="M_H2O_c" stoichiometry="1"/>
<speciesReference species="M_XppppX_c" stoichiometry="1"/>
</listOfReactants>
<listOfProducts>
<speciesReference species="M_XMP_c" stoichiometry="1"/>
<speciesReference species="M_XTP_c" stoichiometry="1"/>
</listOfProducts>
<kineticLaw>
<math xmlns="http://www.w3.org/1998/Math/MathML">
<ci> FLUX_VALUE </ci>
</math>
<listOfParameters>
<parameter id="LOWER_BOUND" value="0" units="mmol_per_gDW_per_hr" constant="true"/>
```

```
      <parameter id="UPPER_BOUND" value="1000" units="mmol_per_gDW_per_hr" constant="true"/>
      <parameter id="FLUX_VALUE" value="0" units="mmol_per_gDW_per_hr" constant="true"/>
      <parameter id="OBJECTIVE_COEFFICIENT" value="0" units="mmol_per_gDW_per_hr" constant="true"/>
    </listOfParameters>
  </kineticLaw>
</reaction>
<reaction id="R_R122" name="Inosine 5&apos;-triphosphate pyrophosphohydrolase" reversible="false">
  <notes>
    <body xmlns="http://www.w3.org/1999/xhtml">
      <p>GENE_ASSOCIATION: (TTC1290 or TTC1583)</p>
      <p>SUBSYSTEM: Nucleotide Salvage Pathway</p>
      <p>EC Number: 3.6.1.19</p>
      <p>Confidence Level: 1</p>
      <p>AUTHORS: Nucleotides metabolism</p>
      <p/>
    </body>
  </notes>
  <listOfReactants>
    <speciesReference species="M_H2O_c" stoichiometry="1"/>
    <speciesReference species="M_ITP_c" stoichiometry="1"/>
  </listOfReactants>
  <listOfProducts>
    <speciesReference species="M_PPi_c" stoichiometry="1"/>
    <speciesReference species="M_H_c" stoichiometry="1"/>
    <speciesReference species="M_IMP_c" stoichiometry="1"/>
  </listOfProducts>
  <kineticLaw>
    <math xmlns="http://www.w3.org/1998/Math/MathML">
      <ci> FLUX_VALUE </ci>
```

```
      </math>
      <listOfParameters>
        <parameter id="LOWER_BOUND" value="0" units="mmol_per_gDW_per_hr" constant="true"/>
        <parameter id="UPPER_BOUND" value="1000" units="mmol_per_gDW_per_hr" constant="true"/>
        <parameter id="FLUX_VALUE" value="0" units="mmol_per_gDW_per_hr" constant="true"/>
        <parameter id="OBJECTIVE_COEFFICIENT" value="0" units="mmol_per_gDW_per_hr" constant="true"/>
      </listOfParameters>
    </kineticLaw>
  </reaction>
  <reaction id="R_R123" name="ATP:IDP phosphotransferase" reversible="true">
    <notes>
      <body xmlns="http://www.w3.org/1999/xhtml">
        <p>GENE_ASSOCIATION: TTC1798</p>
        <p>SUBSYSTEM: Nucleotide Salvage Pathway</p>
        <p>EC Number: 2.7.4.6</p>
        <p>Confidence Level: 1</p>
        <p>AUTHORS: Nucleotides metabolism</p>
        <p/>
      </body>
    </notes>
    <listOfReactants>
      <speciesReference species="M_ATP_c" stoichiometry="1"/>
      <speciesReference species="M_IDP_c" stoichiometry="1"/>
    </listOfReactants>
    <listOfProducts>
      <speciesReference species="M_ADP_c" stoichiometry="1"/>
      <speciesReference species="M_ITP_c" stoichiometry="1"/>
    </listOfProducts>
    <kineticLaw>
```

```xml
      <math xmlns="http://www.w3.org/1998/Math/MathML">
        <ci> FLUX_VALUE </ci>
      </math>
      <listOfParameters>
        <parameter id="LOWER_BOUND" value="-1000" units="mmol_per_gDW_per_hr" constant="true"/>
        <parameter id="UPPER_BOUND" value="1000" units="mmol_per_gDW_per_hr" constant="true"/>
        <parameter id="FLUX_VALUE" value="0" units="mmol_per_gDW_per_hr" constant="true"/>
        <parameter id="OBJECTIVE_COEFFICIENT" value="0" units="mmol_per_gDW_per_hr" constant="true"/>
      </listOfParameters>
    </kineticLaw>
  </reaction>
  <reaction id="R_R124" name="XTP pyrophosphohydrolase" reversible="false">
    <notes>
      <body xmlns="http://www.w3.org/1999/xhtml">
        <p>GENE_ASSOCIATION: (TTC1290 or TTC1583)</p>
        <p>SUBSYSTEM: Nucleotide Salvage Pathway</p>
        <p>EC Number: 3.6.1.19</p>
        <p>Confidence Level: 1</p>
        <p>AUTHORS: Nucleotides metabolism</p>
        <p/>
      </body>
    </notes>
    <listOfReactants>
      <speciesReference species="M_H2O_c" stoichiometry="1"/>
      <speciesReference species="M_XTP_c" stoichiometry="1"/>
    </listOfReactants>
    <listOfProducts>
      <speciesReference species="M_PPi_c" stoichiometry="1"/>
      <speciesReference species="M_H_c" stoichiometry="1"/>
```

```
      <speciesReference species="M_XMP_c" stoichiometry="1"/>
    </listOfProducts>
    <kineticLaw>
      <math xmlns="http://www.w3.org/1998/Math/MathML">
        <ci> FLUX_VALUE </ci>
      </math>
      <listOfParameters>
        <parameter id="LOWER_BOUND" value="0" units="mmol_per_gDW_per_hr" constant="true"/>
        <parameter id="UPPER_BOUND" value="1000" units="mmol_per_gDW_per_hr" constant="true"/>
        <parameter id="FLUX_VALUE" value="0" units="mmol_per_gDW_per_hr" constant="true"/>
        <parameter id="OBJECTIVE_COEFFICIENT" value="0" units="mmol_per_gDW_per_hr" constant="true"/>
      </listOfParameters>
    </kineticLaw>
  </reaction>
  <reaction id="R_R125" name="Xanthosine 5&apos;-phosphate phosphohydrolase" reversible="false">
    <notes>
      <body xmlns="http://www.w3.org/1999/xhtml">
        <p>GENE_ASSOCIATION: (TTC0964 or TTC1625 or TT_P0028)</p>
        <p>SUBSYSTEM: Nucleotide Salvage Pathway</p>
        <p>EC Number: 3.1.3.5</p>
        <p>Confidence Level: 1</p>
        <p>AUTHORS: Nucleotides metabolism</p>
        <p/>
      </body>
    </notes>
    <listOfReactants>
      <speciesReference species="M_H2O_c" stoichiometry="1"/>
      <speciesReference species="M_XMP_c" stoichiometry="1"/>
    </listOfReactants>
```

```xml
  <listOfProducts>
   <speciesReference species="M_Pi_c" stoichiometry="1"/>
   <speciesReference species="M_XNTHS_c" stoichiometry="1"/>
  </listOfProducts>
  <kineticLaw>
   <math xmlns="http://www.w3.org/1998/Math/MathML">
    <ci> FLUX_VALUE </ci>
   </math>
   <listOfParameters>
    <parameter id="LOWER_BOUND" value="0" units="mmol_per_gDW_per_hr" constant="true"/>
    <parameter id="UPPER_BOUND" value="1000" units="mmol_per_gDW_per_hr" constant="true"/>
    <parameter id="FLUX_VALUE" value="0" units="mmol_per_gDW_per_hr" constant="true"/>
    <parameter id="OBJECTIVE_COEFFICIENT" value="0" units="mmol_per_gDW_per_hr" constant="true"/>
   </listOfParameters>
  </kineticLaw>
 </reaction>
 <reaction id="R_R126" name="XMP:pyrophosphate phosphoribosyltransferase" reversible="false">
  <notes>
   <body xmlns="http://www.w3.org/1999/xhtml">
    <p>GENE_ASSOCIATION: TTC1766</p>
    <p>SUBSYSTEM: Nucleotide Salvage Pathway</p>
    <p>EC Number: 2.4.2.22 or 2.4.2.8</p>
    <p>Confidence Level: 1</p>
    <p>AUTHORS: Nucleotides metabolism</p>
    <p/>
   </body>
  </notes>
  <listOfReactants>
   <speciesReference species="M_PRPP_c" stoichiometry="1"/>
```

```
<speciesReference species="M_XTHN_c" stoichiometry="1"/>
</listOfReactants>
<listOfProducts>
<speciesReference species="M_PPi_c" stoichiometry="1"/>
<speciesReference species="M_XMP_c" stoichiometry="1"/>
</listOfProducts>
<kineticLaw>
<math xmlns="http://www.w3.org/1998/Math/MathML">
<ci> FLUX_VALUE </ci>
</math>
<listOfParameters>
<parameter id="LOWER_BOUND" value="0" units="mmol_per_gDW_per_hr" constant="true"/>
<parameter id="UPPER_BOUND" value="1000" units="mmol_per_gDW_per_hr" constant="true"/>
<parameter id="FLUX_VALUE" value="0" units="mmol_per_gDW_per_hr" constant="true"/>
<parameter id="OBJECTIVE_COEFFICIENT" value="0" units="mmol_per_gDW_per_hr" constant="true"/>
</listOfParameters>
</kineticLaw>
</reaction>
<reaction id="R_R127" name="Xanthosine:orthophosphate ribosyltransferase" reversible="true">
<notes>
<body xmlns="http://www.w3.org/1999/xhtml">
<p>GENE_ASSOCIATION: (TTC0194 or TTC1070)</p>
<p>SUBSYSTEM: Nucleotide Salvage Pathway</p>
<p>EC Number: 2.4.2.1</p>
<p>Confidence Level: 1</p>
<p>AUTHORS: Nucleotides metabolism</p>
<p/>
</body>
</notes>
```

```
  <listOfReactants>
    <speciesReference species="M_Pi_c" stoichiometry="1"/>
    <speciesReference species="M_XNTHS_c" stoichiometry="1"/>
  </listOfReactants>
  <listOfProducts>
    <speciesReference species="M_R1P_c" stoichiometry="1"/>
    <speciesReference species="M_XTHN_c" stoichiometry="1"/>
  </listOfProducts>
  <kineticLaw>
    <math xmlns="http://www.w3.org/1998/Math/MathML">
      <ci> FLUX_VALUE </ci>
    </math>
    <listOfParameters>
      <parameter id="LOWER_BOUND" value="-1000" units="mmol_per_gDW_per_hr" constant="true"/>
      <parameter id="UPPER_BOUND" value="1000" units="mmol_per_gDW_per_hr" constant="true"/>
      <parameter id="FLUX_VALUE" value="0" units="mmol_per_gDW_per_hr" constant="true"/>
      <parameter id="OBJECTIVE_COEFFICIENT" value="0" units="mmol_per_gDW_per_hr" constant="true"/>
    </listOfParameters>
  </kineticLaw>
</reaction>
<reaction id="R_R128" name="IMP:NAD+ oxidoreductase" reversible="false">
  <notes>
    <body xmlns="http://www.w3.org/1999/xhtml">
      <p>GENE_ASSOCIATION: (TTC0064 or TTC1634)</p>
      <p>SUBSYSTEM: Nucleotide Salvage Pathway</p>
      <p>EC Number: 1.1.1.205</p>
      <p>Confidence Level: 1</p>
      <p>AUTHORS: Nucleotides metabolism</p>
      <p/>
```

```xml
</body>
</notes>
<listOfReactants>
<speciesReference species="M_H2O_c" stoichiometry="1"/>
<speciesReference species="M_NAD_c" stoichiometry="1"/>
<speciesReference species="M_IMP_c" stoichiometry="1"/>
</listOfReactants>
<listOfProducts>
<speciesReference species="M_H_c" stoichiometry="1"/>
<speciesReference species="M_NADH_c" stoichiometry="1"/>
<speciesReference species="M_XMP_c" stoichiometry="1"/>
</listOfProducts>
<kineticLaw>
<math xmlns="http://www.w3.org/1998/Math/MathML">
<ci> FLUX_VALUE </ci>
</math>
<listOfParameters>
<parameter id="LOWER_BOUND" value="0" units="mmol_per_gDW_per_hr" constant="true"/>
<parameter id="UPPER_BOUND" value="1000" units="mmol_per_gDW_per_hr" constant="true"/>
<parameter id="FLUX_VALUE" value="0" units="mmol_per_gDW_per_hr" constant="true"/>
<parameter id="OBJECTIVE_COEFFICIENT" value="0" units="mmol_per_gDW_per_hr" constant="true"/>
</listOfParameters>
</kineticLaw>
</reaction>
<reaction id="R_R129" name="IMP:diphosphate phospho-D-ribosyltransferase" reversible="true">
<notes>
<body xmlns="http://www.w3.org/1999/xhtml">
<p>GENE_ASSOCIATION: TTC1766</p>
<p>SUBSYSTEM: Nucleotide Salvage Pathway</p>
```

```xml
      <p>EC Number: 2.4.2.8</p>
      <p>Confidence Level: 1</p>
      <p>AUTHORS: Nucleotides metabolism</p>
      <p/>
    </body>
  </notes>
  <listOfReactants>
    <speciesReference species="M_PRPP_c" stoichiometry="1"/>
    <speciesReference species="M_HXTHN_c" stoichiometry="1"/>
  </listOfReactants>
  <listOfProducts>
    <speciesReference species="M_PPi_c" stoichiometry="1"/>
    <speciesReference species="M_IMP_c" stoichiometry="1"/>
  </listOfProducts>
  <kineticLaw>
    <math xmlns="http://www.w3.org/1998/Math/MathML">
      <ci> FLUX_VALUE </ci>
    </math>
    <listOfParameters>
      <parameter id="LOWER_BOUND" value="-1000" units="mmol_per_gDW_per_hr" constant="true"/>
      <parameter id="UPPER_BOUND" value="1000" units="mmol_per_gDW_per_hr" constant="true"/>
      <parameter id="FLUX_VALUE" value="0" units="mmol_per_gDW_per_hr" constant="true"/>
      <parameter id="OBJECTIVE_COEFFICIENT" value="0" units="mmol_per_gDW_per_hr" constant="true"/>
    </listOfParameters>
  </kineticLaw>
</reaction>
<reaction id="R_R130" name="Inosine 5&apos;-monophosphate phosphohydrolase" reversible="false">
  <notes>
    <body xmlns="http://www.w3.org/1999/xhtml">
```

```
    <p>GENE_ASSOCIATION: (TTC0964 or TTC1625 or TT_P0028)</p>
    <p>SUBSYSTEM: Nucleotide Salvage Pathway</p>
    <p>EC Number: 3.1.3.5</p>
    <p>Confidence Level: 1</p>
    <p>AUTHORS: Nucleotides metabolism</p>
    <p/>
   </body>
  </notes>
  <listOfReactants>
   <speciesReference species="M_H2O_c" stoichiometry="1"/>
   <speciesReference species="M_IMP_c" stoichiometry="1"/>
  </listOfReactants>
  <listOfProducts>
   <speciesReference species="M_Pi_c" stoichiometry="1"/>
   <speciesReference species="M_INSN_c" stoichiometry="1"/>
  </listOfProducts>
  <kineticLaw>
   <math xmlns="http://www.w3.org/1998/Math/MathML">
    <ci> FLUX_VALUE </ci>
   </math>
   <listOfParameters>
    <parameter id="LOWER_BOUND" value="0" units="mmol_per_gDW_per_hr" constant="true"/>
    <parameter id="UPPER_BOUND" value="1000" units="mmol_per_gDW_per_hr" constant="true"/>
    <parameter id="FLUX_VALUE" value="0" units="mmol_per_gDW_per_hr" constant="true"/>
    <parameter id="OBJECTIVE_COEFFICIENT" value="0" units="mmol_per_gDW_per_hr" constant="true"/>
   </listOfParameters>
  </kineticLaw>
 </reaction>
 <reaction id="R_R131" name="inosine:phosphate alpha-D-ribosyltransferase" reversible="true">
```

```xml
<notes>
  <body xmlns="http://www.w3.org/1999/xhtml">
    <p>GENE_ASSOCIATION: (TTC0194 or TTC1070)</p>
    <p>SUBSYSTEM: Nucleotide Salvage Pathway</p>
    <p>EC Number: 2.4.2.1</p>
    <p>Confidence Level: 1</p>
    <p>AUTHORS: Nucleotides metabolism</p>
    <p/>
  </body>
</notes>
<listOfReactants>
  <speciesReference species="M_Pi_c" stoichiometry="1"/>
  <speciesReference species="M_INSN_c" stoichiometry="1"/>
</listOfReactants>
<listOfProducts>
  <speciesReference species="M_R1P_c" stoichiometry="1"/>
  <speciesReference species="M_HXTHN_c" stoichiometry="1"/>
</listOfProducts>
<kineticLaw>
  <math xmlns="http://www.w3.org/1998/Math/MathML">
    <ci> FLUX_VALUE </ci>
  </math>
  <listOfParameters>
    <parameter id="LOWER_BOUND" value="-1000" units="mmol_per_gDW_per_hr" constant="true"/>
    <parameter id="UPPER_BOUND" value="1000" units="mmol_per_gDW_per_hr" constant="true"/>
    <parameter id="FLUX_VALUE" value="0" units="mmol_per_gDW_per_hr" constant="true"/>
    <parameter id="OBJECTIVE_COEFFICIENT" value="0" units="mmol_per_gDW_per_hr" constant="true"/>
  </listOfParameters>
</kineticLaw>
```

```
</reaction>
<reaction id="R_R132" name="IMP:L-aspartate ligase (GDP-forming)" reversible="false">
 <notes>
  <body xmlns="http://www.w3.org/1999/xhtml">
   <p>GENE_ASSOCIATION: TTC1764</p>
   <p>SUBSYSTEM: Nucleotide Salvage Pathway</p>
   <p>EC Number: 6.3.4.4</p>
   <p>Confidence Level: 1</p>
   <p>AUTHORS: Nucleotides metabolism</p>
   <p/>
  </body>
 </notes>
 <listOfReactants>
  <speciesReference species="M_L_ASP_c" stoichiometry="1"/>
  <speciesReference species="M_GTP_c" stoichiometry="1"/>
  <speciesReference species="M_IMP_c" stoichiometry="1"/>
 </listOfReactants>
 <listOfProducts>
  <speciesReference species="M_Pi_c" stoichiometry="1"/>
  <speciesReference species="M_H_c" stoichiometry="2"/>
  <speciesReference species="M_GDP_c" stoichiometry="1"/>
  <speciesReference species="M_ADN_SUCC_c" stoichiometry="1"/>
 </listOfProducts>
 <kineticLaw>
  <math xmlns="http://www.w3.org/1998/Math/MathML">
   <ci> FLUX_VALUE </ci>
  </math>
  <listOfParameters>
   <parameter id="LOWER_BOUND" value="0" units="mmol_per_gDW_per_hr" constant="true"/>
```

```
      <parameter id="UPPER_BOUND" value="1000" units="mmol_per_gDW_per_hr" constant="true"/>
      <parameter id="FLUX_VALUE" value="0" units="mmol_per_gDW_per_hr" constant="true"/>
      <parameter id="OBJECTIVE_COEFFICIENT" value="0" units="mmol_per_gDW_per_hr" constant="true"/>
    </listOfParameters>
  </kineticLaw>
</reaction>
<reaction id="R_R133" name="N6-(1,2-dicarboxyethyl)AMP AMP-lyase (fumarate-forming)" reversible="false">
  <notes>
    <body xmlns="http://www.w3.org/1999/xhtml">
      <p>GENE_ASSOCIATION: TTC1149</p>
      <p>SUBSYSTEM: Nucleotide Salvage Pathway</p>
      <p>EC Number: 4.3.2.2</p>
      <p>Confidence Level: 1</p>
      <p>AUTHORS: Nucleotides metabolism</p>
      <p/>
    </body>
  </notes>
  <listOfReactants>
    <speciesReference species="M_ADN_SUCC_c" stoichiometry="1"/>
  </listOfReactants>
  <listOfProducts>
    <speciesReference species="M_AMP_c" stoichiometry="1"/>
    <speciesReference species="M_FUM_c" stoichiometry="1"/>
  </listOfProducts>
  <kineticLaw>
    <math xmlns="http://www.w3.org/1998/Math/MathML">
      <ci> FLUX_VALUE </ci>
    </math>
    <listOfParameters>
```

```
      <parameter id="LOWER_BOUND" value="0" units="mmol_per_gDW_per_hr" constant="true"/>
      <parameter id="UPPER_BOUND" value="1000" units="mmol_per_gDW_per_hr" constant="true"/>
      <parameter id="FLUX_VALUE" value="0" units="mmol_per_gDW_per_hr" constant="true"/>
      <parameter id="OBJECTIVE_COEFFICIENT" value="0" units="mmol_per_gDW_per_hr" constant="true"/>
    </listOfParameters>
  </kineticLaw>
</reaction>
<reaction id="R_R134" name="Adenosine 5&apos;-monophosphate phosphohydrolase" reversible="false">
  <notes>
    <body xmlns="http://www.w3.org/1999/xhtml">
      <p>GENE_ASSOCIATION: (TTC0964 or TTC1625 or TT_P0028)</p>
      <p>SUBSYSTEM: Nucleotide Salvage Pathway</p>
      <p>EC Number: 3.1.3.5</p>
      <p>Confidence Level: 1</p>
      <p>AUTHORS: Nucleotides metabolism</p>
      <p/>
    </body>
  </notes>
  <listOfReactants>
    <speciesReference species="M_H2O_c" stoichiometry="1"/>
    <speciesReference species="M_AMP_c" stoichiometry="1"/>
  </listOfReactants>
  <listOfProducts>
    <speciesReference species="M_Pi_c" stoichiometry="1"/>
    <speciesReference species="M_ADSN_c" stoichiometry="1"/>
  </listOfProducts>
  <kineticLaw>
    <math xmlns="http://www.w3.org/1998/Math/MathML">
      <ci> FLUX_VALUE </ci>
```

```
    </math>
    <listOfParameters>
      <parameter id="LOWER_BOUND" value="0" units="mmol_per_gDW_per_hr" constant="true"/>
      <parameter id="UPPER_BOUND" value="1000" units="mmol_per_gDW_per_hr" constant="true"/>
      <parameter id="FLUX_VALUE" value="0" units="mmol_per_gDW_per_hr" constant="true"/>
      <parameter id="OBJECTIVE_COEFFICIENT" value="0" units="mmol_per_gDW_per_hr" constant="true"/>
    </listOfParameters>
  </kineticLaw>
</reaction>
<reaction id="R_R135" name="Adenosine:phosphate alpha-D-ribosyltransferase" reversible="true">
  <notes>
    <body xmlns="http://www.w3.org/1999/xhtml">
      <p>GENE_ASSOCIATION: (TTC0194 or TTC1070)</p>
      <p>SUBSYSTEM: Nucleotide Salvage Pathway</p>
      <p>EC Number: 2.4.2.1</p>
      <p>Confidence Level: 1</p>
      <p>AUTHORS: Nucleotides metabolism</p>
      <p/>
    </body>
  </notes>
  <listOfReactants>
    <speciesReference species="M_Pi_c" stoichiometry="1"/>
    <speciesReference species="M_ADSN_c" stoichiometry="1"/>
  </listOfReactants>
  <listOfProducts>
    <speciesReference species="M_R1P_c" stoichiometry="1"/>
    <speciesReference species="M_ADN_c" stoichiometry="1"/>
  </listOfProducts>
  <kineticLaw>
```

```
<math xmlns="http://www.w3.org/1998/Math/MathML">
  <ci> FLUX_VALUE </ci>
</math>
<listOfParameters>
  <parameter id="LOWER_BOUND" value="-1000" units="mmol_per_gDW_per_hr" constant="true"/>
  <parameter id="UPPER_BOUND" value="1000" units="mmol_per_gDW_per_hr" constant="true"/>
  <parameter id="FLUX_VALUE" value="0" units="mmol_per_gDW_per_hr" constant="true"/>
  <parameter id="OBJECTIVE_COEFFICIENT" value="0" units="mmol_per_gDW_per_hr" constant="true"/>
</listOfParameters>
</kineticLaw>
</reaction>
<reaction id="R_R136" name="Deoxyinosine:orthophosphate ribosyltransferase" reversible="false">
<notes>
  <body xmlns="http://www.w3.org/1999/xhtml">
    <p>GENE_ASSOCIATION: ((TTC0194 or TTC1070) or TTC1412)</p>
    <p>SUBSYSTEM: Nucleotide Salvage Pathway</p>
    <p>EC Number: 2.4.2.1 or 2.4.2.4</p>
    <p>Confidence Level: 1</p>
    <p>AUTHORS: Nucleotides metabolism</p>
    <p/>
  </body>
</notes>
<listOfReactants>
  <speciesReference species="M_Pi_c" stoichiometry="1"/>
  <speciesReference species="M_zCARO_c" stoichiometry="1"/>
</listOfReactants>
<listOfProducts>
  <speciesReference species="M_dR1P_c" stoichiometry="1"/>
  <speciesReference species="M_HXTHN_c" stoichiometry="1"/>
```

```
</listOfProducts>
<kineticLaw>
 <math xmlns="http://www.w3.org/1998/Math/MathML">
  <ci> FLUX_VALUE </ci>
 </math>
 <listOfParameters>
  <parameter id="LOWER_BOUND" value="0" units="mmol_per_gDW_per_hr" constant="true"/>
  <parameter id="UPPER_BOUND" value="1000" units="mmol_per_gDW_per_hr" constant="true"/>
  <parameter id="FLUX_VALUE" value="0" units="mmol_per_gDW_per_hr" constant="true"/>
  <parameter id="OBJECTIVE_COEFFICIENT" value="0" units="mmol_per_gDW_per_hr" constant="true"/>
 </listOfParameters>
</kineticLaw>
</reaction>
<reaction id="R_R137" name="Deoxyadenosine:orthophosphate ribosyltransferase" reversible="true">
<notes>
 <body xmlns="http://www.w3.org/1999/xhtml">
  <p>GENE_ASSOCIATION: (TTC0194 or TTC1070)</p>
  <p>SUBSYSTEM: Nucleotide Salvage Pathway</p>
  <p>EC Number: 2.4.2.1</p>
  <p>Confidence Level: 1</p>
  <p>AUTHORS: Nucleotides metabolism</p>
  <p/>
 </body>
</notes>
<listOfReactants>
 <speciesReference species="M_Pi_c" stoichiometry="1"/>
 <speciesReference species="M_dADS_c" stoichiometry="1"/>
</listOfReactants>
<listOfProducts>
```

```
    <speciesReference species="M_dR1P_c" stoichiometry="1"/>
    <speciesReference species="M_ADN_c" stoichiometry="1"/>
  </listOfProducts>
  <kineticLaw>
    <math xmlns="http://www.w3.org/1998/Math/MathML">
      <ci> FLUX_VALUE </ci>
    </math>
    <listOfParameters>
      <parameter id="LOWER_BOUND" value="-1000" units="mmol_per_gDW_per_hr" constant="true"/>
      <parameter id="UPPER_BOUND" value="1000" units="mmol_per_gDW_per_hr" constant="true"/>
      <parameter id="FLUX_VALUE" value="0" units="mmol_per_gDW_per_hr" constant="true"/>
      <parameter id="OBJECTIVE_COEFFICIENT" value="0" units="mmol_per_gDW_per_hr" constant="true"/>
    </listOfParameters>
  </kineticLaw>
</reaction>
<reaction id="R_R138" name="ATP:ADP phosphatransferase" reversible="true">
  <notes>
    <body xmlns="http://www.w3.org/1999/xhtml">
      <p>GENE_ASSOCIATION: TTC1798</p>
      <p>SUBSYSTEM: Nucleotide Salvage Pathway</p>
      <p>EC Number: 2.7.4.6</p>
      <p>Confidence Level: 1</p>
      <p>AUTHORS: Nucleotides metabolism</p>
      <p/>
    </body>
  </notes>
  <listOfProducts>
    <speciesReference species="M_ATP_c" stoichiometry="1"/>
    <speciesReference species="M_ADP_c" stoichiometry="1"/>
```

```
  </listOfProducts>
  <kineticLaw>
    <math xmlns="http://www.w3.org/1998/Math/MathML">
      <ci> FLUX_VALUE </ci>
    </math>
    <listOfParameters>
      <parameter id="LOWER_BOUND" value="-1000" units="mmol_per_gDW_per_hr" constant="true"/>
      <parameter id="UPPER_BOUND" value="1000" units="mmol_per_gDW_per_hr" constant="true"/>
      <parameter id="FLUX_VALUE" value="0" units="mmol_per_gDW_per_hr" constant="true"/>
      <parameter id="OBJECTIVE_COEFFICIENT" value="0" units="mmol_per_gDW_per_hr" constant="true"/>
    </listOfParameters>
  </kineticLaw>
</reaction>
<reaction id="R_R139" name="ATP:AMP phosphotransferase" reversible="false">
  <notes>
    <body xmlns="http://www.w3.org/1999/xhtml">
      <p>GENE_ASSOCIATION: TTC1307</p>
      <p>SUBSYSTEM: Nucleotide Salvage Pathway</p>
      <p>EC Number: 2.7.4.3</p>
      <p>Confidence Level: 1</p>
      <p>AUTHORS: Nucleotides metabolism</p>
      <p/>
    </body>
  </notes>
  <listOfReactants>
    <speciesReference species="M_ATP_c" stoichiometry="1"/>
    <speciesReference species="M_AMP_c" stoichiometry="1"/>
  </listOfReactants>
  <listOfProducts>
```

```
          <speciesReference species="M_ADP_c" stoichiometry="2"/>
        </listOfProducts>
        <kineticLaw>
          <math xmlns="http://www.w3.org/1998/Math/MathML">
            <ci> FLUX_VALUE </ci>
          </math>
          <listOfParameters>
            <parameter id="LOWER_BOUND" value="0" units="mmol_per_gDW_per_hr" constant="true"/>
            <parameter id="UPPER_BOUND" value="1000" units="mmol_per_gDW_per_hr" constant="true"/>
            <parameter id="FLUX_VALUE" value="0" units="mmol_per_gDW_per_hr" constant="true"/>
            <parameter id="OBJECTIVE_COEFFICIENT" value="0" units="mmol_per_gDW_per_hr" constant="true"/>
          </listOfParameters>
        </kineticLaw>
      </reaction>
      <reaction id="R_R140" name="2&apos;-Deoxyadenosine 5&apos;-diphosphate:oxidized-thioredoxin 2&apos;-
oxidoreductase" reversible="true">
        <notes>
          <body xmlns="http://www.w3.org/1999/xhtml">
            <p>GENE_ASSOCIATION: (TTC1930 and TT_P0161 and TT_P0162)</p>
            <p>SUBSYSTEM: Nucleotide Salvage Pathway</p>
            <p>EC Number: 1.17.4.1</p>
            <p>Confidence Level: 1</p>
            <p>AUTHORS: Nucleotides metabolism</p>
            <p/>
          </body>
        </notes>
        <listOfReactants>
          <speciesReference species="M_ADP_c" stoichiometry="1"/>
          <speciesReference species="M_R_TRED_c" stoichiometry="1"/>
```

```
  </listOfReactants>
  <listOfProducts>
    <speciesReference species="M_H2O_c" stoichiometry="1"/>
    <speciesReference species="M_O_TRED_c" stoichiometry="1"/>
    <speciesReference species="M_dADP_c" stoichiometry="1"/>
  </listOfProducts>
  <kineticLaw>
    <math xmlns="http://www.w3.org/1998/Math/MathML">
      <ci> FLUX_VALUE </ci>
    </math>
    <listOfParameters>
      <parameter id="LOWER_BOUND" value="-1000" units="mmol_per_gDW_per_hr" constant="true"/>
      <parameter id="UPPER_BOUND" value="1000" units="mmol_per_gDW_per_hr" constant="true"/>
      <parameter id="FLUX_VALUE" value="0" units="mmol_per_gDW_per_hr" constant="true"/>
      <parameter id="OBJECTIVE_COEFFICIENT" value="0" units="mmol_per_gDW_per_hr" constant="true"/>
    </listOfParameters>
  </kineticLaw>
</reaction>
<reaction id="R_R141" name="ATP:dADP phosphotransferase" reversible="false">
  <notes>
    <body xmlns="http://www.w3.org/1999/xhtml">
      <p>GENE_ASSOCIATION: TTC1798</p>
      <p>SUBSYSTEM: Nucleotide Salvage Pathway</p>
      <p>EC Number: 2.7.4.6</p>
      <p>Confidence Level: 1</p>
      <p>AUTHORS: Nucleotides metabolism</p>
      <p/>
    </body>
  </notes>
```

```
  <listOfReactants>
    <speciesReference species="M_ATP_c" stoichiometry="1"/>
    <speciesReference species="M_dADP_c" stoichiometry="1"/>
  </listOfReactants>
  <listOfProducts>
    <speciesReference species="M_dATP_c" stoichiometry="1"/>
    <speciesReference species="M_ADP_c" stoichiometry="1"/>
  </listOfProducts>
  <kineticLaw>
    <math xmlns="http://www.w3.org/1998/Math/MathML">
      <ci> FLUX_VALUE </ci>
    </math>
    <listOfParameters>
      <parameter id="LOWER_BOUND" value="0" units="mmol_per_gDW_per_hr" constant="true"/>
      <parameter id="UPPER_BOUND" value="1000" units="mmol_per_gDW_per_hr" constant="true"/>
      <parameter id="FLUX_VALUE" value="0" units="mmol_per_gDW_per_hr" constant="true"/>
      <parameter id="OBJECTIVE_COEFFICIENT" value="0" units="mmol_per_gDW_per_hr" constant="true"/>
    </listOfParameters>
  </kineticLaw>
</reaction>
<reaction id="R_R142" name="ATP:dAMP phosphotransferase" reversible="true">
  <notes>
    <body xmlns="http://www.w3.org/1999/xhtml">
      <p>GENE_ASSOCIATION: TTC1307</p>
      <p>SUBSYSTEM: Nucleotide Salvage Pathway</p>
      <p>EC Number: 2.7.4.3</p>
      <p>Confidence Level: 1</p>
      <p>AUTHORS: Nucleotides metabolism</p>
      <p/>
```

```xml
    </body>
  </notes>
  <listOfReactants>
    <speciesReference species="M_ATP_c" stoichiometry="1"/>
    <speciesReference species="M_dAMP_c" stoichiometry="1"/>
  </listOfReactants>
  <listOfProducts>
    <speciesReference species="M_ADP_c" stoichiometry="1"/>
    <speciesReference species="M_dADP_c" stoichiometry="1"/>
  </listOfProducts>
  <kineticLaw>
    <math xmlns="http://www.w3.org/1998/Math/MathML">
      <ci> FLUX_VALUE </ci>
    </math>
    <listOfParameters>
      <parameter id="LOWER_BOUND" value="-1000" units="mmol_per_gDW_per_hr" constant="true"/>
      <parameter id="UPPER_BOUND" value="1000" units="mmol_per_gDW_per_hr" constant="true"/>
      <parameter id="FLUX_VALUE" value="0" units="mmol_per_gDW_per_hr" constant="true"/>
      <parameter id="OBJECTIVE_COEFFICIENT" value="0" units="mmol_per_gDW_per_hr" constant="true"/>
    </listOfParameters>
  </kineticLaw>
</reaction>
<reaction id="R_R143" name="2&apos;-Deoxyadenosine 5&apos;-monophosphate phosphohydrolase" reversible="true">
  <notes>
    <body xmlns="http://www.w3.org/1999/xhtml">
      <p>GENE_ASSOCIATION: (TTC0964 or TTC1625 or TT_P0028)</p>
      <p>SUBSYSTEM: Nucleotide Salvage Pathway</p>
      <p>EC Number: 3.1.3.5</p>
      <p>Confidence Level: 1</p>
```

```
      <p>AUTHORS: Nucleotides metabolism</p>
      <p/>
    </body>
  </notes>
  <listOfReactants>
    <speciesReference species="M_H2O_c" stoichiometry="1"/>
    <speciesReference species="M_dAMP_c" stoichiometry="1"/>
  </listOfReactants>
  <listOfProducts>
    <speciesReference species="M_Pi_c" stoichiometry="1"/>
    <speciesReference species="M_dADS_c" stoichiometry="1"/>
  </listOfProducts>
  <kineticLaw>
    <math xmlns="http://www.w3.org/1998/Math/MathML">
      <ci> FLUX_VALUE </ci>
    </math>
    <listOfParameters>
      <parameter id="LOWER_BOUND" value="-1000" units="mmol_per_gDW_per_hr" constant="true"/>
      <parameter id="UPPER_BOUND" value="1000" units="mmol_per_gDW_per_hr" constant="true"/>
      <parameter id="FLUX_VALUE" value="0" units="mmol_per_gDW_per_hr" constant="true"/>
      <parameter id="OBJECTIVE_COEFFICIENT" value="0" units="mmol_per_gDW_per_hr" constant="true"/>
    </listOfParameters>
  </kineticLaw>
</reaction>
<reaction id="R_R144" name="carbamoyl-phosphate:L-aspartate carbamoyltransferase" reversible="false">
  <notes>
    <body xmlns="http://www.w3.org/1999/xhtml">
      <p>GENE_ASSOCIATION: TTC0427</p>
      <p>SUBSYSTEM: Nucleotide Salvage Pathway</p>
```

```xml
<p>EC Number: 2.1.3.2</p>
<p>Confidence Level: 1</p>
<p>AUTHORS: Nucleotides metabolism</p>
<p/>
</body>
</notes>
<listOfReactants>
<speciesReference species="M_L_ASP_c" stoichiometry="1"/>
<speciesReference species="M_CARB_Pi_c" stoichiometry="1"/>
</listOfReactants>
<listOfProducts>
<speciesReference species="M_Pi_c" stoichiometry="1"/>
<speciesReference species="M_H_c" stoichiometry="1"/>
<speciesReference species="M_CARB_ASP_c" stoichiometry="1"/>
</listOfProducts>
<kineticLaw>
<math xmlns="http://www.w3.org/1998/Math/MathML">
<ci> FLUX_VALUE </ci>
</math>
<listOfParameters>
<parameter id="LOWER_BOUND" value="0" units="mmol_per_gDW_per_hr" constant="true"/>
<parameter id="UPPER_BOUND" value="1000" units="mmol_per_gDW_per_hr" constant="true"/>
<parameter id="FLUX_VALUE" value="0" units="mmol_per_gDW_per_hr" constant="true"/>
<parameter id="OBJECTIVE_COEFFICIENT" value="0" units="mmol_per_gDW_per_hr" constant="true"/>
</listOfParameters>
</kineticLaw>
</reaction>
<reaction id="R_R145" name="hydrogen-carbonate:L-glutamine amido-ligase (ADP-forming, carbamate-phosphorylating)" reversible="false">
```

```xml
<notes>
  <body xmlns="http://www.w3.org/1999/xhtml">
    <p>GENE_ASSOCIATION: (TTC0247 and TTC1706)</p>
    <p>SUBSYSTEM: Nucleotide Salvage Pathway</p>
    <p>EC Number: 6.3.5.5</p>
    <p>Confidence Level: 1</p>
    <p>AUTHORS: Nucleotides metabolism</p>
    <p/>
  </body>
</notes>
<listOfReactants>
  <speciesReference species="M_L_GLN_c" stoichiometry="1"/>
  <speciesReference species="M_ATP_c" stoichiometry="2"/>
  <speciesReference species="M_H2O_c" stoichiometry="1"/>
  <speciesReference species="M_HCO3_c" stoichiometry="1"/>
</listOfReactants>
<listOfProducts>
  <speciesReference species="M_L_GLU_c" stoichiometry="1"/>
  <speciesReference species="M_ADP_c" stoichiometry="2"/>
  <speciesReference species="M_Pi_c" stoichiometry="1"/>
  <speciesReference species="M_H_c" stoichiometry="2"/>
  <speciesReference species="M_CARB_Pi_c" stoichiometry="1"/>
</listOfProducts>
<kineticLaw>
  <math xmlns="http://www.w3.org/1998/Math/MathML">
    <ci> FLUX_VALUE </ci>
  </math>
  <listOfParameters>
    <parameter id="LOWER_BOUND" value="0" units="mmol_per_gDW_per_hr" constant="true"/>
```

```
      <parameter id="UPPER_BOUND" value="1000" units="mmol_per_gDW_per_hr" constant="true"/>
      <parameter id="FLUX_VALUE" value="0" units="mmol_per_gDW_per_hr" constant="true"/>
      <parameter id="OBJECTIVE_COEFFICIENT" value="0" units="mmol_per_gDW_per_hr" constant="true"/>
    </listOfParameters>
  </kineticLaw>
</reaction>
<reaction id="R_R146" name="(S)-dihydroorotate amidohydrolase" reversible="true">
  <notes>
    <body xmlns="http://www.w3.org/1999/xhtml">
      <p>GENE_ASSOCIATION: TTC0426</p>
      <p>SUBSYSTEM: Nucleotide Salvage Pathway</p>
      <p>EC Number: 3.5.2.3</p>
      <p>Confidence Level: 1</p>
      <p>AUTHORS: Nucleotides metabolism</p>
      <p/>
    </body>
  </notes>
  <listOfReactants>
    <speciesReference species="M_H2O_c" stoichiometry="1"/>
    <speciesReference species="M_DHY_ORTA_c" stoichiometry="1"/>
  </listOfReactants>
  <listOfProducts>
    <speciesReference species="M_H_c" stoichiometry="1"/>
    <speciesReference species="M_CARB_ASP_c" stoichiometry="1"/>
  </listOfProducts>
  <kineticLaw>
    <math xmlns="http://www.w3.org/1998/Math/MathML">
      <ci> FLUX_VALUE </ci>
    </math>
```

```
      <listOfParameters>
        <parameter id="LOWER_BOUND" value="-1000" units="mmol_per_gDW_per_hr" constant="true"/>
        <parameter id="UPPER_BOUND" value="1000" units="mmol_per_gDW_per_hr" constant="true"/>
        <parameter id="FLUX_VALUE" value="0" units="mmol_per_gDW_per_hr" constant="true"/>
        <parameter id="OBJECTIVE_COEFFICIENT" value="0" units="mmol_per_gDW_per_hr" constant="true"/>
      </listOfParameters>
    </kineticLaw>
  </reaction>
  <reaction id="R_R147" name="CTP aminohydrolase" reversible="false">
    <notes>
      <body xmlns="http://www.w3.org/1999/xhtml">
        <p>GENE_ASSOCIATION: TTC1864</p>
        <p>SUBSYSTEM: Nucleotide Salvage Pathway</p>
        <p>EC Number: 3.5.4.13</p>
        <p>Confidence Level: 1</p>
        <p>AUTHORS: Nucleotides metabolism</p>
        <p/>
      </body>
    </notes>
    <listOfReactants>
      <speciesReference species="M_CTP_c" stoichiometry="1"/>
      <speciesReference species="M_H2O_c" stoichiometry="1"/>
    </listOfReactants>
    <listOfProducts>
      <speciesReference species="M_UTP_c" stoichiometry="1"/>
      <speciesReference species="M_NH4_c" stoichiometry="1"/>
    </listOfProducts>
    <kineticLaw>
      <math xmlns="http://www.w3.org/1998/Math/MathML">
```

```
      <ci> FLUX_VALUE </ci>
    </math>
    <listOfParameters>
      <parameter id="LOWER_BOUND" value="0" units="mmol_per_gDW_per_hr" constant="true"/>
      <parameter id="UPPER_BOUND" value="1000" units="mmol_per_gDW_per_hr" constant="true"/>
      <parameter id="FLUX_VALUE" value="0" units="mmol_per_gDW_per_hr" constant="true"/>
      <parameter id="OBJECTIVE_COEFFICIENT" value="0" units="mmol_per_gDW_per_hr" constant="true"/>
    </listOfParameters>
  </kineticLaw>
</reaction>
<reaction id="R_R148" name="NADPH:oxidized-thioredoxin oxidoreductase" reversible="false">
  <notes>
    <body xmlns="http://www.w3.org/1999/xhtml">
      <p>GENE_ASSOCIATION: (TTC0096 or TTC0853 or TTC1555)</p>
      <p>SUBSYSTEM: Oxidative Phosphorylation</p>
      <p>EC Number: 1.8.1.9</p>
      <p>Confidence Level: 1</p>
      <p>AUTHORS: Energy and cofactors metabolism</p>
      <p/>
    </body>
  </notes>
  <listOfReactants>
    <speciesReference species="M_H_c" stoichiometry="1"/>
    <speciesReference species="M_NADPH_c" stoichiometry="1"/>
    <speciesReference species="M_O_TRED_c" stoichiometry="1"/>
  </listOfReactants>
  <listOfProducts>
    <speciesReference species="M_NADP_c" stoichiometry="1"/>
    <speciesReference species="M_R_TRED_c" stoichiometry="1"/>
```

```
  </listOfProducts>
  <kineticLaw>
    <math xmlns="http://www.w3.org/1998/Math/MathML">
      <ci> FLUX_VALUE </ci>
    </math>
    <listOfParameters>
      <parameter id="LOWER_BOUND" value="0" units="mmol_per_gDW_per_hr" constant="true"/>
      <parameter id="UPPER_BOUND" value="1000" units="mmol_per_gDW_per_hr" constant="true"/>
      <parameter id="FLUX_VALUE" value="0" units="mmol_per_gDW_per_hr" constant="true"/>
      <parameter id="OBJECTIVE_COEFFICIENT" value="0" units="mmol_per_gDW_per_hr" constant="true"/>
    </listOfParameters>
  </kineticLaw>
</reaction>
<reaction id="R_R149" name="UTP:ammonia ligase (ADP-forming)" reversible="false">
  <notes>
    <body xmlns="http://www.w3.org/1999/xhtml">
      <p>GENE_ASSOCIATION: TTC1102</p>
      <p>SUBSYSTEM: Nucleotide Salvage Pathway</p>
      <p>EC Number: 6.3.4.2</p>
      <p>Confidence Level: 1</p>
      <p>AUTHORS: Nucleotides metabolism</p>
      <p/>
    </body>
  </notes>
  <listOfReactants>
    <speciesReference species="M_L_GLN_c" stoichiometry="1"/>
    <speciesReference species="M_UTP_c" stoichiometry="1"/>
    <speciesReference species="M_ATP_c" stoichiometry="1"/>
    <speciesReference species="M_H2O_c" stoichiometry="1"/>
```

```
  </listOfReactants>
  <listOfProducts>
    <speciesReference species="M_L_GLU_c" stoichiometry="1"/>
    <speciesReference species="M_CTP_c" stoichiometry="1"/>
    <speciesReference species="M_ADP_c" stoichiometry="1"/>
    <speciesReference species="M_Pi_c" stoichiometry="1"/>
    <speciesReference species="M_H_c" stoichiometry="2"/>
  </listOfProducts>
  <kineticLaw>
    <math xmlns="http://www.w3.org/1998/Math/MathML">
      <ci> FLUX_VALUE </ci>
    </math>
    <listOfParameters>
      <parameter id="LOWER_BOUND" value="0" units="mmol_per_gDW_per_hr" constant="true"/>
      <parameter id="UPPER_BOUND" value="1000" units="mmol_per_gDW_per_hr" constant="true"/>
      <parameter id="FLUX_VALUE" value="0" units="mmol_per_gDW_per_hr" constant="true"/>
      <parameter id="OBJECTIVE_COEFFICIENT" value="0" units="mmol_per_gDW_per_hr" constant="true"/>
    </listOfParameters>
  </kineticLaw>
</reaction>
<reaction id="R_R150" reversible="false">
  <notes>
    <body xmlns="http://www.w3.org/1999/xhtml">
      <p>GENE_ASSOCIATION: </p>
      <p>SUBSYSTEM: Nucleotide Salvage Pathway</p>
      <p>EC Number: </p>
      <p>Confidence Level: 1</p>
      <p>AUTHORS: Nucleotides metabolism</p>
      <p/>
```

```
    </body>
   </notes>
   <listOfReactants>
    <speciesReference species="M_L_GLN_c" stoichiometry="1"/>
    <speciesReference species="M_UTP_c" stoichiometry="1"/>
    <speciesReference species="M_ATP_c" stoichiometry="1"/>
    <speciesReference species="M_H2O_c" stoichiometry="1"/>
   </listOfReactants>
   <listOfProducts>
    <speciesReference species="M_L_GLU_c" stoichiometry="1"/>
    <speciesReference species="M_PPi_c" stoichiometry="1"/>
    <speciesReference species="M_H_c" stoichiometry="2"/>
    <speciesReference species="M_AMP_c" stoichiometry="1"/>
    <speciesReference species="M_GMP_c" stoichiometry="1"/>
   </listOfProducts>
   <kineticLaw>
    <math xmlns="http://www.w3.org/1998/Math/MathML">
     <ci> FLUX_VALUE </ci>
    </math>
    <listOfParameters>
     <parameter id="LOWER_BOUND" value="0" units="mmol_per_gDW_per_hr" constant="true"/>
     <parameter id="UPPER_BOUND" value="1000" units="mmol_per_gDW_per_hr" constant="true"/>
     <parameter id="FLUX_VALUE" value="0" units="mmol_per_gDW_per_hr" constant="true"/>
     <parameter id="OBJECTIVE_COEFFICIENT" value="0" units="mmol_per_gDW_per_hr" constant="true"/>
    </listOfParameters>
   </kineticLaw>
  </reaction>
  <reaction id="R_R151" name="dCTP aminohydrolase" reversible="false">
   <notes>
```

```
  <body xmlns="http://www.w3.org/1999/xhtml">
    <p>GENE_ASSOCIATION: TTC1864</p>
    <p>SUBSYSTEM: Nucleotide Salvage Pathway</p>
    <p>EC Number: 3.5.4.13</p>
    <p>Confidence Level: 1</p>
    <p>AUTHORS: Nucleotides metabolism</p>
    <p/>
  </body>
</notes>
<listOfReactants>
  <speciesReference species="M_dCTP_c" stoichiometry="1"/>
  <speciesReference species="M_H2O_c" stoichiometry="1"/>
  <speciesReference species="M_H_c" stoichiometry="1"/>
</listOfReactants>
<listOfProducts>
  <speciesReference species="M_NH4_c" stoichiometry="1"/>
  <speciesReference species="M_dUTP_c" stoichiometry="1"/>
</listOfProducts>
<kineticLaw>
  <math xmlns="http://www.w3.org/1998/Math/MathML">
    <ci> FLUX_VALUE </ci>
  </math>
  <listOfParameters>
    <parameter id="LOWER_BOUND" value="0" units="mmol_per_gDW_per_hr" constant="true"/>
    <parameter id="UPPER_BOUND" value="1000" units="mmol_per_gDW_per_hr" constant="true"/>
    <parameter id="FLUX_VALUE" value="0" units="mmol_per_gDW_per_hr" constant="true"/>
    <parameter id="OBJECTIVE_COEFFICIENT" value="0" units="mmol_per_gDW_per_hr" constant="true"/>
  </listOfParameters>
</kineticLaw>
```

```xml
</reaction>
<reaction id="R_R152" name="Orotidine-5&apos;-phosphate:diphosphate phospho-alpha-D-ribosyl-transferase" reversible="true">
<notes>
<body xmlns="http://www.w3.org/1999/xhtml">
<p>GENE_ASSOCIATION: TTC1380</p>
<p>SUBSYSTEM: Nucleotide Salvage Pathway</p>
<p>EC Number: 2.4.2.10</p>
<p>Confidence Level: 1</p>
<p>AUTHORS: Nucleotides metabolism</p>
<p/>
</body>
</notes>
<listOfReactants>
<speciesReference species="M_PPi_c" stoichiometry="1"/>
<speciesReference species="M_ORTD5P_c" stoichiometry="1"/>
</listOfReactants>
<listOfProducts>
<speciesReference species="M_PRPP_c" stoichiometry="1"/>
<speciesReference species="M_ORTA_c" stoichiometry="1"/>
</listOfProducts>
<kineticLaw>
<math xmlns="http://www.w3.org/1998/Math/MathML">
<ci> FLUX_VALUE </ci>
</math>
<listOfParameters>
<parameter id="LOWER_BOUND" value="-1000" units="mmol_per_gDW_per_hr" constant="true"/>
<parameter id="UPPER_BOUND" value="1000" units="mmol_per_gDW_per_hr" constant="true"/>
<parameter id="FLUX_VALUE" value="0" units="mmol_per_gDW_per_hr" constant="true"/>
```

```
      <parameter id="OBJECTIVE_COEFFICIENT" value="0" units="mmol_per_gDW_per_hr" constant="true"/>
    </listOfParameters>
  </kineticLaw>
</reaction>
<reaction id="R_R153" name="P1,P4-bis(5&apos;-uridyl)-tetraphosphate uridylohydrolase" reversible="false">
  <notes>
    <body xmlns="http://www.w3.org/1999/xhtml">
      <p>GENE_ASSOCIATION: (TTC1519 or TTC1859)</p>
      <p>SUBSYSTEM: Nucleotide Salvage Pathway</p>
      <p>EC Number: 3.6.1.17</p>
      <p>Confidence Level: 1</p>
      <p>AUTHORS: Nucleotides metabolism</p>
      <p/>
    </body>
  </notes>
  <listOfReactants>
    <speciesReference species="M_H2O_c" stoichiometry="1"/>
    <speciesReference species="M_UppppU_c" stoichiometry="1"/>
  </listOfReactants>
  <listOfProducts>
    <speciesReference species="M_UTP_c" stoichiometry="1"/>
    <speciesReference species="M_UMP_c" stoichiometry="1"/>
  </listOfProducts>
  <kineticLaw>
    <math xmlns="http://www.w3.org/1998/Math/MathML">
      <ci> FLUX_VALUE </ci>
    </math>
    <listOfParameters>
      <parameter id="LOWER_BOUND" value="0" units="mmol_per_gDW_per_hr" constant="true"/>
```

```
      <parameter id="UPPER_BOUND" value="1000" units="mmol_per_gDW_per_hr" constant="true"/>
      <parameter id="FLUX_VALUE" value="0" units="mmol_per_gDW_per_hr" constant="true"/>
      <parameter id="OBJECTIVE_COEFFICIENT" value="0" units="mmol_per_gDW_per_hr" constant="true"/>
    </listOfParameters>
  </kineticLaw>
</reaction>
<reaction id="R_R154" name="ATP:UDP phosphotransferase" reversible="true">
  <notes>
    <body xmlns="http://www.w3.org/1999/xhtml">
      <p>GENE_ASSOCIATION: TTC1798</p>
      <p>SUBSYSTEM: Nucleotide Salvage Pathway</p>
      <p>EC Number: 2.7.4.6</p>
      <p>Confidence Level: 1</p>
      <p>AUTHORS: Nucleotides metabolism</p>
      <p/>
    </body>
  </notes>
  <listOfReactants>
    <speciesReference species="M_ATP_c" stoichiometry="1"/>
    <speciesReference species="M_UDP_c" stoichiometry="1"/>
  </listOfReactants>
  <listOfProducts>
    <speciesReference species="M_UTP_c" stoichiometry="1"/>
    <speciesReference species="M_ADP_c" stoichiometry="1"/>
  </listOfProducts>
  <kineticLaw>
    <math xmlns="http://www.w3.org/1998/Math/MathML">
      <ci> FLUX_VALUE </ci>
    </math>
```

```
    <listOfParameters>
      <parameter id="LOWER_BOUND" value="-1000" units="mmol_per_gDW_per_hr" constant="true"/>
      <parameter id="UPPER_BOUND" value="1000" units="mmol_per_gDW_per_hr" constant="true"/>
      <parameter id="FLUX_VALUE" value="0" units="mmol_per_gDW_per_hr" constant="true"/>
      <parameter id="OBJECTIVE_COEFFICIENT" value="0" units="mmol_per_gDW_per_hr" constant="true"/>
    </listOfParameters>
  </kineticLaw>
</reaction>
<reaction id="R_R155" name="ATP:CDP phosphotransferase" reversible="true">
  <notes>
    <body xmlns="http://www.w3.org/1999/xhtml">
      <p>GENE_ASSOCIATION: TTC1798</p>
      <p>SUBSYSTEM: Nucleotide Salvage Pathway</p>
      <p>EC Number: 2.7.4.6</p>
      <p>Confidence Level: 1</p>
      <p>AUTHORS: Nucleotides metabolism</p>
      <p/>
    </body>
  </notes>
  <listOfReactants>
    <speciesReference species="M_ATP_c" stoichiometry="1"/>
    <speciesReference species="M_CDP_c" stoichiometry="1"/>
  </listOfReactants>
  <listOfProducts>
    <speciesReference species="M_CTP_c" stoichiometry="1"/>
    <speciesReference species="M_ADP_c" stoichiometry="1"/>
  </listOfProducts>
  <kineticLaw>
    <math xmlns="http://www.w3.org/1998/Math/MathML">
```

```
      <ci> FLUX_VALUE </ci>
    </math>
    <listOfParameters>
      <parameter id="LOWER_BOUND" value="-1000" units="mmol_per_gDW_per_hr" constant="true"/>
      <parameter id="UPPER_BOUND" value="1000" units="mmol_per_gDW_per_hr" constant="true"/>
      <parameter id="FLUX_VALUE" value="0" units="mmol_per_gDW_per_hr" constant="true"/>
      <parameter id="OBJECTIVE_COEFFICIENT" value="0" units="mmol_per_gDW_per_hr" constant="true"/>
    </listOfParameters>
  </kineticLaw>
</reaction>
<reaction id="R_R156" name="ATP:dCDP phosphotransferase" reversible="true">
  <notes>
    <body xmlns="http://www.w3.org/1999/xhtml">
      <p>GENE_ASSOCIATION: TTC1798</p>
      <p>SUBSYSTEM: Nucleotide Salvage Pathway</p>
      <p>EC Number: 2.7.4.6</p>
      <p>Confidence Level: 1</p>
      <p>AUTHORS: Nucleotides metabolism</p>
      <p/>
    </body>
  </notes>
  <listOfReactants>
    <speciesReference species="M_ATP_c" stoichiometry="1"/>
    <speciesReference species="M_dCDP_c" stoichiometry="1"/>
  </listOfReactants>
  <listOfProducts>
    <speciesReference species="M_dCTP_c" stoichiometry="1"/>
    <speciesReference species="M_ADP_c" stoichiometry="1"/>
  </listOfProducts>
```

```
    <kineticLaw>
      <math xmlns="http://www.w3.org/1998/Math/MathML">
        <ci> FLUX_VALUE </ci>
      </math>
      <listOfParameters>
        <parameter id="LOWER_BOUND" value="-1000" units="mmol_per_gDW_per_hr" constant="true"/>
        <parameter id="UPPER_BOUND" value="1000" units="mmol_per_gDW_per_hr" constant="true"/>
        <parameter id="FLUX_VALUE" value="0" units="mmol_per_gDW_per_hr" constant="true"/>
        <parameter id="OBJECTIVE_COEFFICIENT" value="0" units="mmol_per_gDW_per_hr" constant="true"/>
      </listOfParameters>
    </kineticLaw>
  </reaction>
  <reaction id="R_R157" name="ATP:dUDP phosphotransferase" reversible="true">
    <notes>
      <body xmlns="http://www.w3.org/1999/xhtml">
        <p>GENE_ASSOCIATION: TTC1798</p>
        <p>SUBSYSTEM: Nucleotide Salvage Pathway</p>
        <p>EC Number: 2.7.4.6</p>
        <p>Confidence Level: 1</p>
        <p>AUTHORS: Nucleotides metabolism</p>
        <p/>
      </body>
    </notes>
    <listOfReactants>
      <speciesReference species="M_ATP_c" stoichiometry="1"/>
      <speciesReference species="M_dUDP_c" stoichiometry="1"/>
    </listOfReactants>
    <listOfProducts>
      <speciesReference species="M_ADP_c" stoichiometry="1"/>
```

```xml
<speciesReference species="M_dUTP_c" stoichiometry="1"/>
</listOfProducts>
<kineticLaw>
<math xmlns="http://www.w3.org/1998/Math/MathML">
<ci> FLUX_VALUE </ci>
</math>
<listOfParameters>
<parameter id="LOWER_BOUND" value="-1000" units="mmol_per_gDW_per_hr" constant="true"/>
<parameter id="UPPER_BOUND" value="1000" units="mmol_per_gDW_per_hr" constant="true"/>
<parameter id="FLUX_VALUE" value="0" units="mmol_per_gDW_per_hr" constant="true"/>
<parameter id="OBJECTIVE_COEFFICIENT" value="0" units="mmol_per_gDW_per_hr" constant="true"/>
</listOfParameters>
</kineticLaw>
</reaction>
<reaction id="R_R158" name="ATP:dTDP phosphotransferase" reversible="true">
<notes>
<body xmlns="http://www.w3.org/1999/xhtml">
<p>GENE_ASSOCIATION: TTC1798</p>
<p>SUBSYSTEM: Nucleotide Salvage Pathway</p>
<p>EC Number: 2.7.4.6</p>
<p>Confidence Level: 1</p>
<p>AUTHORS: Nucleotides metabolism</p>
<p/>
</body>
</notes>
<listOfReactants>
<speciesReference species="M_ATP_c" stoichiometry="1"/>
<speciesReference species="M_dTDP_c" stoichiometry="1"/>
</listOfReactants>
```

```xml
  <listOfProducts>
    <speciesReference species="M_dTTP_c" stoichiometry="1"/>
    <speciesReference species="M_ADP_c" stoichiometry="1"/>
  </listOfProducts>
  <kineticLaw>
    <math xmlns="http://www.w3.org/1998/Math/MathML">
      <ci> FLUX_VALUE </ci>
    </math>
    <listOfParameters>
      <parameter id="LOWER_BOUND" value="-1000" units="mmol_per_gDW_per_hr" constant="true"/>
      <parameter id="UPPER_BOUND" value="1000" units="mmol_per_gDW_per_hr" constant="true"/>
      <parameter id="FLUX_VALUE" value="0" units="mmol_per_gDW_per_hr" constant="true"/>
      <parameter id="OBJECTIVE_COEFFICIENT" value="0" units="mmol_per_gDW_per_hr" constant="true"/>
    </listOfParameters>
  </kineticLaw>
</reaction>
<reaction id="R_R159" name="Uridine triphosphate pyrophosphohydrolase" reversible="false">
  <notes>
    <body xmlns="http://www.w3.org/1999/xhtml">
      <p>GENE_ASSOCIATION: (TTC1290 or TTC1583)</p>
      <p>SUBSYSTEM: Nucleotide Salvage Pathway</p>
      <p>EC Number: 3.6.1.19</p>
      <p>Confidence Level: 1</p>
      <p>AUTHORS: Nucleotides metabolism</p>
      <p/>
    </body>
  </notes>
  <listOfReactants>
    <speciesReference species="M_UTP_c" stoichiometry="1"/>
```

```
    <speciesReference species="M_H2O_c" stoichiometry="2"/>
  </listOfReactants>
  <listOfProducts>
    <speciesReference species="M_Pi_c" stoichiometry="2"/>
    <speciesReference species="M_H_c" stoichiometry="2"/>
    <speciesReference species="M_UMP_c" stoichiometry="1"/>
  </listOfProducts>
  <kineticLaw>
    <math xmlns="http://www.w3.org/1998/Math/MathML">
      <ci> FLUX_VALUE </ci>
    </math>
    <listOfParameters>
      <parameter id="LOWER_BOUND" value="0" units="mmol_per_gDW_per_hr" constant="true"/>
      <parameter id="UPPER_BOUND" value="1000" units="mmol_per_gDW_per_hr" constant="true"/>
      <parameter id="FLUX_VALUE" value="0" units="mmol_per_gDW_per_hr" constant="true"/>
      <parameter id="OBJECTIVE_COEFFICIENT" value="0" units="mmol_per_gDW_per_hr" constant="true"/>
    </listOfParameters>
  </kineticLaw>
</reaction>
<reaction id="R_R160" name="dUTP nucleotidohydrolase" reversible="false">
  <notes>
    <body xmlns="http://www.w3.org/1999/xhtml">
      <p>GENE_ASSOCIATION: (TTC1290 or TTC1583)</p>
      <p>SUBSYSTEM: Nucleotide Salvage Pathway</p>
      <p>EC Number: 3.6.1.19</p>
      <p>Confidence Level: 1</p>
      <p>AUTHORS: Nucleotides metabolism</p>
      <p/>
    </body>
```

```
        </notes>
        <listOfReactants>
          <speciesReference species="M_H2O_c" stoichiometry="1"/>
          <speciesReference species="M_dUTP_c" stoichiometry="1"/>
        </listOfReactants>
        <listOfProducts>
          <speciesReference species="M_PPi_c" stoichiometry="1"/>
          <speciesReference species="M_H_c" stoichiometry="1"/>
          <speciesReference species="M_dUMP_c" stoichiometry="1"/>
        </listOfProducts>
        <kineticLaw>
          <math xmlns="http://www.w3.org/1998/Math/MathML">
            <ci> FLUX_VALUE </ci>
          </math>
          <listOfParameters>
            <parameter id="LOWER_BOUND" value="0" units="mmol_per_gDW_per_hr" constant="true"/>
            <parameter id="UPPER_BOUND" value="1000" units="mmol_per_gDW_per_hr" constant="true"/>
            <parameter id="FLUX_VALUE" value="0" units="mmol_per_gDW_per_hr" constant="true"/>
            <parameter id="OBJECTIVE_COEFFICIENT" value="0" units="mmol_per_gDW_per_hr" constant="true"/>
          </listOfParameters>
        </kineticLaw>
      </reaction>
      <reaction id="R_R161" name="2&apos;-Deoxycytidine diphosphate:oxidized-thioredoxin 2&apos;-oxidoreductase"
reversible="false">
        <notes>
          <body xmlns="http://www.w3.org/1999/xhtml">
            <p>GENE_ASSOCIATION: (TTC1930 and TT_P0161 and TT_P0162)</p>
            <p>SUBSYSTEM: Nucleotide Salvage Pathway</p>
            <p>EC Number: 1.17.4.1</p>
```

```xml
<p>Confidence Level: 1</p>

<p>AUTHORS: Nucleotides metabolism</p>

<p/>

</body>

</notes>

<listOfReactants>

<speciesReference species="M_R_TRED_c" stoichiometry="1"/>

<speciesReference species="M_CDP_c" stoichiometry="1"/>

</listOfReactants>

<listOfProducts>

<speciesReference species="M_H2O_c" stoichiometry="1"/>

<speciesReference species="M_O_TRED_c" stoichiometry="1"/>

<speciesReference species="M_dCDP_c" stoichiometry="1"/>

</listOfProducts>

<kineticLaw>

<math xmlns="http://www.w3.org/1998/Math/MathML">

<ci> FLUX_VALUE </ci>

</math>

<listOfParameters>

<parameter id="LOWER_BOUND" value="0" units="mmol_per_gDW_per_hr" constant="true"/>

<parameter id="UPPER_BOUND" value="1000" units="mmol_per_gDW_per_hr" constant="true"/>

<parameter id="FLUX_VALUE" value="0" units="mmol_per_gDW_per_hr" constant="true"/>

<parameter id="OBJECTIVE_COEFFICIENT" value="0" units="mmol_per_gDW_per_hr" constant="true"/>

</listOfParameters>

</kineticLaw>

</reaction>

<reaction id="R_R162" name="2&apos;-Deoxyuridine 5&apos;-diphosphate:oxidized-thioredoxin 2&apos;-oxidoreductase" reversible="false">

<notes>
```

```
  <body xmlns="http://www.w3.org/1999/xhtml">
    <p>GENE_ASSOCIATION: (TTC1930 and TT_P0161 and TT_P0162)</p>
    <p>SUBSYSTEM: Nucleotide Salvage Pathway</p>
    <p>EC Number: 1.17.4.1</p>
    <p>Confidence Level: 1</p>
    <p>AUTHORS: Nucleotides metabolism</p>
    <p/>
  </body>
</notes>
<listOfReactants>
  <speciesReference species="M_R_TRED_c" stoichiometry="1"/>
  <speciesReference species="M_UDP_c" stoichiometry="1"/>
</listOfReactants>
<listOfProducts>
  <speciesReference species="M_H2O_c" stoichiometry="1"/>
  <speciesReference species="M_O_TRED_c" stoichiometry="1"/>
  <speciesReference species="M_dUDP_c" stoichiometry="1"/>
</listOfProducts>
<kineticLaw>
  <math xmlns="http://www.w3.org/1998/Math/MathML">
    <ci> FLUX_VALUE </ci>
  </math>
  <listOfParameters>
    <parameter id="LOWER_BOUND" value="0" units="mmol_per_gDW_per_hr" constant="true"/>
    <parameter id="UPPER_BOUND" value="1000" units="mmol_per_gDW_per_hr" constant="true"/>
    <parameter id="FLUX_VALUE" value="0" units="mmol_per_gDW_per_hr" constant="true"/>
    <parameter id="OBJECTIVE_COEFFICIENT" value="0" units="mmol_per_gDW_per_hr" constant="true"/>
  </listOfParameters>
</kineticLaw>
```

```xml
</reaction>
<reaction id="R_R163" name="orotidine-5&apos;-phosphate carboxy-lyase (UMP-forming)" reversible="false">
 <notes>
  <body xmlns="http://www.w3.org/1999/xhtml">
   <p>GENE_ASSOCIATION: TTC1381</p>
   <p>SUBSYSTEM: Nucleotide Salvage Pathway</p>
   <p>EC Number: 4.1.1.23</p>
   <p>Confidence Level: 1</p>
   <p>AUTHORS: Nucleotides metabolism</p>
   <p/>
  </body>
 </notes>
 <listOfReactants>
  <speciesReference species="M_H_c" stoichiometry="1"/>
  <speciesReference species="M_ORTD5P_c" stoichiometry="1"/>
 </listOfReactants>
 <listOfProducts>
  <speciesReference species="M_CO2_c" stoichiometry="1"/>
  <speciesReference species="M_UMP_c" stoichiometry="1"/>
 </listOfProducts>
 <kineticLaw>
  <math xmlns="http://www.w3.org/1998/Math/MathML">
   <ci> FLUX_VALUE </ci>
  </math>
  <listOfParameters>
   <parameter id="LOWER_BOUND" value="0" units="mmol_per_gDW_per_hr" constant="true"/>
   <parameter id="UPPER_BOUND" value="1000" units="mmol_per_gDW_per_hr" constant="true"/>
   <parameter id="FLUX_VALUE" value="0" units="mmol_per_gDW_per_hr" constant="true"/>
   <parameter id="OBJECTIVE_COEFFICIENT" value="0" units="mmol_per_gDW_per_hr" constant="true"/>
```

```
    </listOfParameters>
  </kineticLaw>
</reaction>
<reaction id="R_R164" name="ATP:UMP phosphotransferase" reversible="true">
  <notes>
    <body xmlns="http://www.w3.org/1999/xhtml">
      <p>GENE_ASSOCIATION: (TTC0089 or TTC0507)</p>
      <p>SUBSYSTEM: Nucleotide Salvage Pathway</p>
      <p>EC Number: 2.7.4.14 or 2.7.4.22</p>
      <p>Confidence Level: 1</p>
      <p>AUTHORS: Nucleotides metabolism</p>
      <p/>
    </body>
  </notes>
  <listOfReactants>
    <speciesReference species="M_ATP_c" stoichiometry="1"/>
    <speciesReference species="M_UMP_c" stoichiometry="1"/>
  </listOfReactants>
  <listOfProducts>
    <speciesReference species="M_ADP_c" stoichiometry="1"/>
    <speciesReference species="M_UDP_c" stoichiometry="1"/>
  </listOfProducts>
  <kineticLaw>
    <math xmlns="http://www.w3.org/1998/Math/MathML">
      <ci> FLUX_VALUE </ci>
    </math>
    <listOfParameters>
      <parameter id="LOWER_BOUND" value="-1000" units="mmol_per_gDW_per_hr" constant="true"/>
      <parameter id="UPPER_BOUND" value="1000" units="mmol_per_gDW_per_hr" constant="true"/>
```

```
      <parameter id="FLUX_VALUE" value="0" units="mmol_per_gDW_per_hr" constant="true"/>
      <parameter id="OBJECTIVE_COEFFICIENT" value="0" units="mmol_per_gDW_per_hr" constant="true"/>
    </listOfParameters>
  </kineticLaw>
</reaction>
<reaction id="R_R165" name="ATP:CMP phosphotransferase" reversible="true">
  <notes>
    <body xmlns="http://www.w3.org/1999/xhtml">
      <p>GENE_ASSOCIATION: TTC0089</p>
      <p>SUBSYSTEM: Nucleotide Salvage Pathway</p>
      <p>EC Number: 2.7.4.14</p>
      <p>Confidence Level: 1</p>
      <p>AUTHORS: Nucleotides metabolism</p>
      <p/>
    </body>
  </notes>
  <listOfReactants>
    <speciesReference species="M_ATP_c" stoichiometry="1"/>
    <speciesReference species="M_CMP_c" stoichiometry="1"/>
  </listOfReactants>
  <listOfProducts>
    <speciesReference species="M_ADP_c" stoichiometry="1"/>
    <speciesReference species="M_CDP_c" stoichiometry="1"/>
  </listOfProducts>
  <kineticLaw>
    <math xmlns="http://www.w3.org/1998/Math/MathML">
      <ci> FLUX_VALUE </ci>
    </math>
    <listOfParameters>
```

```xml
<parameter id="LOWER_BOUND" value="-1000" units="mmol_per_gDW_per_hr" constant="true"/>
<parameter id="UPPER_BOUND" value="1000" units="mmol_per_gDW_per_hr" constant="true"/>
<parameter id="FLUX_VALUE" value="0" units="mmol_per_gDW_per_hr" constant="true"/>
<parameter id="OBJECTIVE_COEFFICIENT" value="0" units="mmol_per_gDW_per_hr" constant="true"/>
</listOfParameters>
</kineticLaw>
</reaction>
<reaction id="R_R166" name="ATP:dCMP phosphotransferase" reversible="true">
<notes>
<body xmlns="http://www.w3.org/1999/xhtml">
<p>GENE_ASSOCIATION: TTC0089</p>
<p>SUBSYSTEM: Nucleotide Salvage Pathway</p>
<p>EC Number: 2.7.4.15</p>
<p>Confidence Level: 1</p>
<p>AUTHORS: Nucleotides metabolism</p>
<p/>
</body>
</notes>
<listOfReactants>
<speciesReference species="M_ATP_c" stoichiometry="1"/>
<speciesReference species="M_dCMP_c" stoichiometry="1"/>
</listOfReactants>
<listOfProducts>
<speciesReference species="M_ADP_c" stoichiometry="1"/>
<speciesReference species="M_dCDP_c" stoichiometry="1"/>
</listOfProducts>
<kineticLaw>
<math xmlns="http://www.w3.org/1998/Math/MathML">
<ci> FLUX_VALUE </ci>
```

```
    </math>
    <listOfParameters>
      <parameter id="LOWER_BOUND" value="-1000" units="mmol_per_gDW_per_hr" constant="true"/>
      <parameter id="UPPER_BOUND" value="1000" units="mmol_per_gDW_per_hr" constant="true"/>
      <parameter id="FLUX_VALUE" value="0" units="mmol_per_gDW_per_hr" constant="true"/>
      <parameter id="OBJECTIVE_COEFFICIENT" value="0" units="mmol_per_gDW_per_hr" constant="true"/>
    </listOfParameters>
  </kineticLaw>
</reaction>
<reaction id="R_R167" name="ATP:dUMP phosphotransferase" reversible="true">
  <notes>
    <body xmlns="http://www.w3.org/1999/xhtml">
      <p>GENE_ASSOCIATION: TTC1243</p>
      <p>SUBSYSTEM: Nucleotide Salvage Pathway</p>
      <p>EC Number: 2.7.4.9</p>
      <p>Confidence Level: 1</p>
      <p>AUTHORS: Nucleotides metabolism</p>
      <p/>
    </body>
  </notes>
  <listOfReactants>
    <speciesReference species="M_ATP_c" stoichiometry="1"/>
    <speciesReference species="M_dUMP_c" stoichiometry="1"/>
  </listOfReactants>
  <listOfProducts>
    <speciesReference species="M_ADP_c" stoichiometry="1"/>
    <speciesReference species="M_dUDP_c" stoichiometry="1"/>
  </listOfProducts>
  <kineticLaw>
```

```
    <math xmlns="http://www.w3.org/1998/Math/MathML">
      <ci> FLUX_VALUE </ci>
    </math>
    <listOfParameters>
      <parameter id="LOWER_BOUND" value="-1000" units="mmol_per_gDW_per_hr" constant="true"/>
      <parameter id="UPPER_BOUND" value="1000" units="mmol_per_gDW_per_hr" constant="true"/>
      <parameter id="FLUX_VALUE" value="0" units="mmol_per_gDW_per_hr" constant="true"/>
      <parameter id="OBJECTIVE_COEFFICIENT" value="0" units="mmol_per_gDW_per_hr" constant="true"/>
    </listOfParameters>
  </kineticLaw>
</reaction>
<reaction id="R_R168" name="ATP:dTMP phosphotransferase" reversible="true">
  <notes>
    <body xmlns="http://www.w3.org/1999/xhtml">
      <p>GENE_ASSOCIATION: TTC1243</p>
      <p>SUBSYSTEM: Nucleotide Salvage Pathway</p>
      <p>EC Number: 2.7.4.9</p>
      <p>Confidence Level: 1</p>
      <p>AUTHORS: Nucleotides metabolism</p>
      <p/>
    </body>
  </notes>
  <listOfReactants>
    <speciesReference species="M_ATP_c" stoichiometry="1"/>
    <speciesReference species="M_dTMP_c" stoichiometry="1"/>
  </listOfReactants>
  <listOfProducts>
    <speciesReference species="M_ADP_c" stoichiometry="1"/>
    <speciesReference species="M_dTDP_c" stoichiometry="1"/>
```

```xml
  </listOfProducts>
  <kineticLaw>
    <math xmlns="http://www.w3.org/1998/Math/MathML">
      <ci> FLUX_VALUE </ci>
    </math>
    <listOfParameters>
      <parameter id="LOWER_BOUND" value="-1000" units="mmol_per_gDW_per_hr" constant="true"/>
      <parameter id="UPPER_BOUND" value="1000" units="mmol_per_gDW_per_hr" constant="true"/>
      <parameter id="FLUX_VALUE" value="0" units="mmol_per_gDW_per_hr" constant="true"/>
      <parameter id="OBJECTIVE_COEFFICIENT" value="0" units="mmol_per_gDW_per_hr" constant="true"/>
    </listOfParameters>
  </kineticLaw>
</reaction>
<reaction id="R_R169" name="ATP:uridine 5&apos;-phosphotransferase" reversible="true">
  <notes>
    <body xmlns="http://www.w3.org/1999/xhtml">
      <p>GENE_ASSOCIATION: TTC0210</p>
      <p>SUBSYSTEM: Nucleotide Salvage Pathway</p>
      <p>EC Number: 2.7.1.48</p>
      <p>Confidence Level: 1</p>
      <p>AUTHORS: Nucleotides metabolism</p>
      <p/>
    </body>
  </notes>
  <listOfReactants>
    <speciesReference species="M_ATP_c" stoichiometry="1"/>
    <speciesReference species="M_URD_c" stoichiometry="1"/>
  </listOfReactants>
  <listOfProducts>
```

```
    <speciesReference species="M_ADP_c" stoichiometry="1"/>
    <speciesReference species="M_H_c" stoichiometry="1"/>
    <speciesReference species="M_UMP_c" stoichiometry="1"/>
  </listOfProducts>
  <kineticLaw>
    <math xmlns="http://www.w3.org/1998/Math/MathML">
      <ci> FLUX_VALUE </ci>
    </math>
    <listOfParameters>
      <parameter id="LOWER_BOUND" value="0" units="mmol_per_gDW_per_hr" constant="true"/>
      <parameter id="UPPER_BOUND" value="0" units="mmol_per_gDW_per_hr" constant="true"/>
      <parameter id="FLUX_VALUE" value="0" units="mmol_per_gDW_per_hr" constant="true"/>
      <parameter id="OBJECTIVE_COEFFICIENT" value="0" units="mmol_per_gDW_per_hr" constant="true"/>
    </listOfParameters>
  </kineticLaw>
</reaction>
<reaction id="R_R170" name="ATP:cytidine 5&apos;-phosphotransferase" reversible="false">
  <notes>
    <body xmlns="http://www.w3.org/1999/xhtml">
      <p>GENE_ASSOCIATION: TTC0210</p>
      <p>SUBSYSTEM: Nucleotide Salvage Pathway</p>
      <p>EC Number: 2.7.1.48</p>
      <p>Confidence Level: 1</p>
      <p>AUTHORS: Nucleotides metabolism</p>
      <p/>
    </body>
  </notes>
  <listOfReactants>
    <speciesReference species="M_ATP_c" stoichiometry="1"/>
```

```
<speciesReference species="M_CYTD_c" stoichiometry="1"/>
</listOfReactants>
<listOfProducts>
<speciesReference species="M_ADP_c" stoichiometry="1"/>
<speciesReference species="M_H_c" stoichiometry="1"/>
<speciesReference species="M_CMP_c" stoichiometry="1"/>
</listOfProducts>
<kineticLaw>
<math xmlns="http://www.w3.org/1998/Math/MathML">
<ci> FLUX_VALUE </ci>
</math>
<listOfParameters>
<parameter id="LOWER_BOUND" value="0" units="mmol_per_gDW_per_hr" constant="true"/>
<parameter id="UPPER_BOUND" value="1000" units="mmol_per_gDW_per_hr" constant="true"/>
<parameter id="FLUX_VALUE" value="0" units="mmol_per_gDW_per_hr" constant="true"/>
<parameter id="OBJECTIVE_COEFFICIENT" value="0" units="mmol_per_gDW_per_hr" constant="true"/>
</listOfParameters>
</kineticLaw>
</reaction>
<reaction id="R_R171" name="GTP:cytidine 5&apos;-phosphotransferase" reversible="false">
<notes>
<body xmlns="http://www.w3.org/1999/xhtml">
<p>GENE_ASSOCIATION: TTC0210</p>
<p>SUBSYSTEM: Nucleotide Salvage Pathway</p>
<p>EC Number: 2.7.1.48</p>
<p>Confidence Level: 1</p>
<p>AUTHORS: Nucleotides metabolism</p>
<p/>
</body>
```

```
  </notes>
  <listOfReactants>
    <speciesReference species="M_GTP_c" stoichiometry="1"/>
    <speciesReference species="M_CYTD_c" stoichiometry="1"/>
  </listOfReactants>
  <listOfProducts>
    <speciesReference species="M_H_c" stoichiometry="1"/>
    <speciesReference species="M_GDP_c" stoichiometry="1"/>
    <speciesReference species="M_CMP_c" stoichiometry="1"/>
  </listOfProducts>
  <kineticLaw>
    <math xmlns="http://www.w3.org/1998/Math/MathML">
      <ci> FLUX_VALUE </ci>
    </math>
    <listOfParameters>
      <parameter id="LOWER_BOUND" value="0" units="mmol_per_gDW_per_hr" constant="true"/>
      <parameter id="UPPER_BOUND" value="1000" units="mmol_per_gDW_per_hr" constant="true"/>
      <parameter id="FLUX_VALUE" value="0" units="mmol_per_gDW_per_hr" constant="true"/>
      <parameter id="OBJECTIVE_COEFFICIENT" value="0" units="mmol_per_gDW_per_hr" constant="true"/>
    </listOfParameters>
  </kineticLaw>
</reaction>
<reaction id="R_R172" name="guanosine nucleotides de novo biosynthesis" reversible="false">
  <notes>
    <body xmlns="http://www.w3.org/1999/xhtml">
      <p>GENE_ASSOCIATION: </p>
      <p>SUBSYSTEM: Nucleotide Salvage Pathway</p>
      <p>EC Number: </p>
      <p>Confidence Level: 1</p>
```

```
      <p>AUTHORS: Nucleotides metabolism</p>
      <p/>
    </body>
  </notes>
  <listOfReactants>
    <speciesReference species="M_GTP_c" stoichiometry="1"/>
    <speciesReference species="M_FMN_c" stoichiometry="1"/>
  </listOfReactants>
  <listOfProducts>
    <speciesReference species="M_dGTP_c" stoichiometry="1"/>
    <speciesReference species="M_H2O_c" stoichiometry="1"/>
    <speciesReference species="M_FMNH2_c" stoichiometry="1"/>
  </listOfProducts>
  <kineticLaw>
    <math xmlns="http://www.w3.org/1998/Math/MathML">
      <ci> FLUX_VALUE </ci>
    </math>
    <listOfParameters>
      <parameter id="LOWER_BOUND" value="0" units="mmol_per_gDW_per_hr" constant="true"/>
      <parameter id="UPPER_BOUND" value="1000" units="mmol_per_gDW_per_hr" constant="true"/>
      <parameter id="FLUX_VALUE" value="0" units="mmol_per_gDW_per_hr" constant="true"/>
      <parameter id="OBJECTIVE_COEFFICIENT" value="0" units="mmol_per_gDW_per_hr" constant="true"/>
    </listOfParameters>
  </kineticLaw>
</reaction>
<reaction id="R_R173" name="UTP:uridine 5&apos;-phosphotransferase" reversible="false">
  <notes>
    <body xmlns="http://www.w3.org/1999/xhtml">
      <p>GENE_ASSOCIATION: TTC0210</p>
```

```
      <p>SUBSYSTEM: Nucleotide Salvage Pathway</p>
      <p>EC Number: 2.7.1.48</p>
      <p>Confidence Level: 1</p>
      <p>AUTHORS: Nucleotides metabolism</p>
      <p/>
    </body>
  </notes>
  <listOfReactants>
    <speciesReference species="M_UTP_c" stoichiometry="1"/>
    <speciesReference species="M_URD_c" stoichiometry="1"/>
  </listOfReactants>
  <listOfProducts>
    <speciesReference species="M_UMP_c" stoichiometry="1"/>
    <speciesReference species="M_UDP_c" stoichiometry="1"/>
  </listOfProducts>
  <kineticLaw>
    <math xmlns="http://www.w3.org/1998/Math/MathML">
      <ci> FLUX_VALUE </ci>
    </math>
    <listOfParameters>
      <parameter id="LOWER_BOUND" value="0" units="mmol_per_gDW_per_hr" constant="true"/>
      <parameter id="UPPER_BOUND" value="1000" units="mmol_per_gDW_per_hr" constant="true"/>
      <parameter id="FLUX_VALUE" value="0" units="mmol_per_gDW_per_hr" constant="true"/>
      <parameter id="OBJECTIVE_COEFFICIENT" value="0" units="mmol_per_gDW_per_hr" constant="true"/>
    </listOfParameters>
  </kineticLaw>
</reaction>
<reaction id="R_R174" name="GTP:uridine 5&apos;-phosphotransferase" reversible="false">
  <notes>
```

```
  <body xmlns="http://www.w3.org/1999/xhtml">
    <p>GENE_ASSOCIATION: TTC0210</p>
    <p>SUBSYSTEM: Nucleotide Salvage Pathway</p>
    <p>EC Number: 2.7.1.48</p>
    <p>Confidence Level: 1</p>
    <p>AUTHORS: Nucleotides metabolism</p>
    <p/>
  </body>
</notes>
<listOfReactants>
  <speciesReference species="M_GTP_c" stoichiometry="1"/>
  <speciesReference species="M_URD_c" stoichiometry="1"/>
</listOfReactants>
<listOfProducts>
  <speciesReference species="M_H_c" stoichiometry="1"/>
  <speciesReference species="M_GDP_c" stoichiometry="1"/>
  <speciesReference species="M_UMP_c" stoichiometry="1"/>
</listOfProducts>
<kineticLaw>
  <math xmlns="http://www.w3.org/1998/Math/MathML">
    <ci> FLUX_VALUE </ci>
  </math>
  <listOfParameters>
    <parameter id="LOWER_BOUND" value="0" units="mmol_per_gDW_per_hr" constant="true"/>
    <parameter id="UPPER_BOUND" value="1000" units="mmol_per_gDW_per_hr" constant="true"/>
    <parameter id="FLUX_VALUE" value="0" units="mmol_per_gDW_per_hr" constant="true"/>
    <parameter id="OBJECTIVE_COEFFICIENT" value="0" units="mmol_per_gDW_per_hr" constant="true"/>
  </listOfParameters>
</kineticLaw>
```

```
</reaction>

<reaction id="R_R175" name="ITP:uridine 5&apos;-phosphotransferase" reversible="false">

  <notes>

    <body xmlns="http://www.w3.org/1999/xhtml">

      <p>GENE_ASSOCIATION: TTC0210</p>

      <p>SUBSYSTEM: Nucleotide Salvage Pathway</p>

      <p>EC Number: 2.7.1.48</p>

      <p>Confidence Level: 1</p>

      <p>AUTHORS: Nucleotides metabolism</p>

      <p/>

    </body>

  </notes>

  <listOfReactants>

    <speciesReference species="M_ITP_c" stoichiometry="1"/>

    <speciesReference species="M_URD_c" stoichiometry="1"/>

  </listOfReactants>

  <listOfProducts>

    <speciesReference species="M_IDP_c" stoichiometry="1"/>

    <speciesReference species="M_UMP_c" stoichiometry="1"/>

  </listOfProducts>

  <kineticLaw>

    <math xmlns="http://www.w3.org/1998/Math/MathML">

      <ci> FLUX_VALUE </ci>

    </math>

    <listOfParameters>

      <parameter id="LOWER_BOUND" value="0" units="mmol_per_gDW_per_hr" constant="true"/>

      <parameter id="UPPER_BOUND" value="1000" units="mmol_per_gDW_per_hr" constant="true"/>

      <parameter id="FLUX_VALUE" value="0" units="mmol_per_gDW_per_hr" constant="true"/>

      <parameter id="OBJECTIVE_COEFFICIENT" value="0" units="mmol_per_gDW_per_hr" constant="true"/>
```

</listOfParameters>
</kineticLaw>
</reaction>
<reaction id="R_R176" name="dGTP:uridine 5'-phosphotransferase" reversible="false">
<notes>
<body xmlns="http://www.w3.org/1999/xhtml">
<p>GENE_ASSOCIATION: TTC0210</p>
<p>SUBSYSTEM: Nucleotide Salvage Pathway</p>
<p>EC Number: 2.7.1.48</p>
<p>Confidence Level: 1</p>
<p>AUTHORS: Nucleotides metabolism</p>
<p/>
</body>
</notes>
<listOfReactants>
<speciesReference species="M_dGTP_c" stoichiometry="1"/>
<speciesReference species="M_URD_c" stoichiometry="1"/>
</listOfReactants>
<listOfProducts>
<speciesReference species="M_dGDP_c" stoichiometry="1"/>
<speciesReference species="M_UMP_c" stoichiometry="1"/>
</listOfProducts>
<kineticLaw>
<math xmlns="http://www.w3.org/1998/Math/MathML">
<ci> FLUX_VALUE </ci>
</math>
<listOfParameters>

```
<parameter id="FLUX_VALUE" value="0" units="mmol_per_gDW_per_hr" constant="true"/>
<parameter id="OBJECTIVE_COEFFICIENT" value="0" units="mmol_per_gDW_per_hr" constant="true"/>
</listOfParameters>
</kineticLaw>
</reaction>
<reaction id="R_R177" name="dTTP:uridine 5&apos;-phosphotransferase" reversible="false">
<notes>
<body xmlns="http://www.w3.org/1999/xhtml">
<p>GENE_ASSOCIATION: TTC0210</p>
<p>SUBSYSTEM: Nucleotide Salvage Pathway</p>
<p>EC Number: 2.7.1.48</p>
<p>Confidence Level: 1</p>
<p>AUTHORS: Nucleotides metabolism</p>
<p/>
</body>
</notes>
<listOfReactants>
<speciesReference species="M_dTTP_c" stoichiometry="1"/>
<speciesReference species="M_URD_c" stoichiometry="1"/>
</listOfReactants>
<listOfProducts>
<speciesReference species="M_UMP_c" stoichiometry="1"/>
<speciesReference species="M_dTDP_c" stoichiometry="1"/>
</listOfProducts>
<kineticLaw>
<math xmlns="http://www.w3.org/1998/Math/MathML">
<ci> FLUX_VALUE </ci>
</math>
<listOfParameters>
```

```
      <parameter id="LOWER_BOUND" value="0" units="mmol_per_gDW_per_hr" constant="true"/>
      <parameter id="UPPER_BOUND" value="1000" units="mmol_per_gDW_per_hr" constant="true"/>
      <parameter id="FLUX_VALUE" value="0" units="mmol_per_gDW_per_hr" constant="true"/>
      <parameter id="OBJECTIVE_COEFFICIENT" value="0" units="mmol_per_gDW_per_hr" constant="true"/>
    </listOfParameters>
  </kineticLaw>
</reaction>
<reaction id="R_R178" name="dCTP:uridine 5&apos;-phosphotransferase" reversible="false">
  <notes>
    <body xmlns="http://www.w3.org/1999/xhtml">
      <p>GENE_ASSOCIATION: TTC0210</p>
      <p>SUBSYSTEM: Nucleotide Salvage Pathway</p>
      <p>EC Number: 2.7.1.48</p>
      <p>Confidence Level: 1</p>
      <p>AUTHORS: Nucleotides metabolism</p>
      <p/>
    </body>
  </notes>
  <listOfReactants>
    <speciesReference species="M_dCTP_c" stoichiometry="1"/>
    <speciesReference species="M_URD_c" stoichiometry="1"/>
  </listOfReactants>
  <listOfProducts>
    <speciesReference species="M_UMP_c" stoichiometry="1"/>
    <speciesReference species="M_dCDP_c" stoichiometry="1"/>
  </listOfProducts>
  <kineticLaw>
    <math xmlns="http://www.w3.org/1998/Math/MathML">
      <ci> FLUX_VALUE </ci>
```

```
      </math>
      <listOfParameters>
        <parameter id="LOWER_BOUND" value="0" units="mmol_per_gDW_per_hr" constant="true"/>
        <parameter id="UPPER_BOUND" value="1000" units="mmol_per_gDW_per_hr" constant="true"/>
        <parameter id="FLUX_VALUE" value="0" units="mmol_per_gDW_per_hr" constant="true"/>
        <parameter id="OBJECTIVE_COEFFICIENT" value="0" units="mmol_per_gDW_per_hr" constant="true"/>
      </listOfParameters>
    </kineticLaw>
  </reaction>
  <reaction id="R_R179" name="dUTP:uridine 5&apos;-phosphotransferase" reversible="false">
    <notes>
      <body xmlns="http://www.w3.org/1999/xhtml">
        <p>GENE_ASSOCIATION: TTC0210</p>
        <p>SUBSYSTEM: Nucleotide Salvage Pathway</p>
        <p>EC Number: 2.7.1.48</p>
        <p>Confidence Level: 1</p>
        <p>AUTHORS: Nucleotides metabolism</p>
        <p/>
      </body>
    </notes>
    <listOfReactants>
      <speciesReference species="M_dUTP_c" stoichiometry="1"/>
      <speciesReference species="M_URD_c" stoichiometry="1"/>
    </listOfReactants>
    <listOfProducts>
      <speciesReference species="M_UMP_c" stoichiometry="1"/>
      <speciesReference species="M_dUDP_c" stoichiometry="1"/>
    </listOfProducts>
    <kineticLaw>
```

```
    <math xmlns="http://www.w3.org/1998/Math/MathML">
      <ci> FLUX_VALUE </ci>
    </math>
    <listOfParameters>
      <parameter id="LOWER_BOUND" value="0" units="mmol_per_gDW_per_hr" constant="true"/>
      <parameter id="UPPER_BOUND" value="1000" units="mmol_per_gDW_per_hr" constant="true"/>
      <parameter id="FLUX_VALUE" value="0" units="mmol_per_gDW_per_hr" constant="true"/>
      <parameter id="OBJECTIVE_COEFFICIENT" value="0" units="mmol_per_gDW_per_hr" constant="true"/>
    </listOfParameters>
  </kineticLaw>
</reaction>
<reaction id="R_R180" name="Uridine 5&apos;-monophosphate phosphohydrolase" reversible="false">
  <notes>
    <body xmlns="http://www.w3.org/1999/xhtml">
      <p>GENE_ASSOCIATION: (TTC0964 or TTC1625 or TT_P0028)</p>
      <p>SUBSYSTEM: Nucleotide Salvage Pathway</p>
      <p>EC Number: 3.1.3.5</p>
      <p>Confidence Level: 1</p>
      <p>AUTHORS: Nucleotides metabolism</p>
      <p/>
    </body>
  </notes>
  <listOfReactants>
    <speciesReference species="M_H2O_c" stoichiometry="1"/>
    <speciesReference species="M_UMP_c" stoichiometry="1"/>
  </listOfReactants>
  <listOfProducts>
    <speciesReference species="M_Pi_c" stoichiometry="1"/>
    <speciesReference species="M_URD_c" stoichiometry="1"/>
```

```
  </listOfProducts>
  <kineticLaw>
    <math xmlns="http://www.w3.org/1998/Math/MathML">
      <ci> FLUX_VALUE </ci>
    </math>
    <listOfParameters>
      <parameter id="LOWER_BOUND" value="0" units="mmol_per_gDW_per_hr" constant="true"/>
      <parameter id="UPPER_BOUND" value="1000" units="mmol_per_gDW_per_hr" constant="true"/>
      <parameter id="FLUX_VALUE" value="0" units="mmol_per_gDW_per_hr" constant="true"/>
      <parameter id="OBJECTIVE_COEFFICIENT" value="0" units="mmol_per_gDW_per_hr" constant="true"/>
    </listOfParameters>
  </kineticLaw>
</reaction>
<reaction id="R_R181" name="Thymidylate 5&apos;-phosphohydrolase" reversible="false">
  <notes>
    <body xmlns="http://www.w3.org/1999/xhtml">
      <p>GENE_ASSOCIATION: (TTC0964 or TTC1625 or TT_P0028)</p>
      <p>SUBSYSTEM: Nucleotide Salvage Pathway</p>
      <p>EC Number: 3.1.3.5</p>
      <p>Confidence Level: 1</p>
      <p>AUTHORS: Nucleotides metabolism</p>
      <p/>
    </body>
  </notes>
  <listOfReactants>
    <speciesReference species="M_H2O_c" stoichiometry="1"/>
    <speciesReference species="M_dTMP_c" stoichiometry="1"/>
  </listOfReactants>
  <listOfProducts>
```

```
<speciesReference species="M_Pi_c" stoichiometry="1"/>
<speciesReference species="M_THYMD_c" stoichiometry="1"/>
</listOfProducts>
<kineticLaw>
<math xmlns="http://www.w3.org/1998/Math/MathML">
<ci> FLUX_VALUE </ci>
</math>
<listOfParameters>
<parameter id="LOWER_BOUND" value="0" units="mmol_per_gDW_per_hr" constant="true"/>
<parameter id="UPPER_BOUND" value="1000" units="mmol_per_gDW_per_hr" constant="true"/>
<parameter id="FLUX_VALUE" value="0" units="mmol_per_gDW_per_hr" constant="true"/>
<parameter id="OBJECTIVE_COEFFICIENT" value="0" units="mmol_per_gDW_per_hr" constant="true"/>
</listOfParameters>
</kineticLaw>
</reaction>
<reaction id="R_R182" name="2&apos;-Deoxycytidine 5&apos;-monophosphate phosphohydrolase" reversible="false">
<notes>
<body xmlns="http://www.w3.org/1999/xhtml">
<p>GENE_ASSOCIATION: (TTC0964 or TTC1625 or TT_P0028)</p>
<p>SUBSYSTEM: Nucleotide Salvage Pathway</p>
<p>EC Number: 3.1.3.5</p>
<p>Confidence Level: 1</p>
<p>AUTHORS: Nucleotides metabolism</p>
<p/>
</body>
</notes>
<listOfReactants>
<speciesReference species="M_H2O_c" stoichiometry="1"/>
<speciesReference species="M_dCMP_c" stoichiometry="1"/>
```

```
  </listOfReactants>
  <listOfProducts>
    <speciesReference species="M_Pi_c" stoichiometry="1"/>
    <speciesReference species="M_dCYTD_c" stoichiometry="1"/>
  </listOfProducts>
  <kineticLaw>
    <math xmlns="http://www.w3.org/1998/Math/MathML">
      <ci> FLUX_VALUE </ci>
    </math>
    <listOfParameters>
      <parameter id="LOWER_BOUND" value="0" units="mmol_per_gDW_per_hr" constant="true"/>
      <parameter id="UPPER_BOUND" value="1000" units="mmol_per_gDW_per_hr" constant="true"/>
      <parameter id="FLUX_VALUE" value="0" units="mmol_per_gDW_per_hr" constant="true"/>
      <parameter id="OBJECTIVE_COEFFICIENT" value="0" units="mmol_per_gDW_per_hr" constant="true"/>
    </listOfParameters>
  </kineticLaw>
</reaction>
<reaction id="R_R183" name="UMP:diphosphate phospho-alpha-D-ribosyltransferase" reversible="true">
  <notes>
    <body xmlns="http://www.w3.org/1999/xhtml">
      <p>GENE_ASSOCIATION: (TTC0428 or TTC0946)</p>
      <p>SUBSYSTEM: Nucleotide Salvage Pathway</p>
      <p>EC Number: 2.4.2.9</p>
      <p>Confidence Level: 1</p>
      <p>AUTHORS: Nucleotides metabolism</p>
      <p/>
    </body>
  </notes>
  <listOfReactants>
```

```
    <speciesReference species="M_PPi_c" stoichiometry="1"/>
    <speciesReference species="M_UMP_c" stoichiometry="1"/>
  </listOfReactants>
  <listOfProducts>
    <speciesReference species="M_PRPP_c" stoichiometry="1"/>
    <speciesReference species="M_URA_c" stoichiometry="1"/>
  </listOfProducts>
  <kineticLaw>
    <math xmlns="http://www.w3.org/1998/Math/MathML">
      <ci> FLUX_VALUE </ci>
    </math>
    <listOfParameters>
      <parameter id="LOWER_BOUND" value="-1000" units="mmol_per_gDW_per_hr" constant="true"/>
      <parameter id="UPPER_BOUND" value="1000" units="mmol_per_gDW_per_hr" constant="true"/>
      <parameter id="FLUX_VALUE" value="0" units="mmol_per_gDW_per_hr" constant="true"/>
      <parameter id="OBJECTIVE_COEFFICIENT" value="0" units="mmol_per_gDW_per_hr" constant="true"/>
    </listOfParameters>
  </kineticLaw>
</reaction>
<reaction id="R_R184" name="Cytidine aminohydrolase" reversible="true">
  <notes>
    <body xmlns="http://www.w3.org/1999/xhtml">
      <p>GENE_ASSOCIATION: TTC0383</p>
      <p>SUBSYSTEM: Nucleotide Salvage Pathway</p>
      <p>EC Number: 3.5.4.5</p>
      <p>Confidence Level: 1</p>
      <p>AUTHORS: Nucleotides metabolism</p>
      <p/>
    </body>
```

```
  </notes>
  <listOfReactants>
    <speciesReference species="M_H2O_c" stoichiometry="1"/>
    <speciesReference species="M_H_c" stoichiometry="1"/>
    <speciesReference species="M_CYTD_c" stoichiometry="1"/>
  </listOfReactants>
  <listOfProducts>
    <speciesReference species="M_NH4_c" stoichiometry="1"/>
    <speciesReference species="M_URD_c" stoichiometry="1"/>
  </listOfProducts>
  <kineticLaw>
    <math xmlns="http://www.w3.org/1998/Math/MathML">
      <ci> FLUX_VALUE </ci>
    </math>
    <listOfParameters>
      <parameter id="LOWER_BOUND" value="-1000" units="mmol_per_gDW_per_hr" constant="true"/>
      <parameter id="UPPER_BOUND" value="1000" units="mmol_per_gDW_per_hr" constant="true"/>
      <parameter id="FLUX_VALUE" value="0" units="mmol_per_gDW_per_hr" constant="true"/>
      <parameter id="OBJECTIVE_COEFFICIENT" value="0" units="mmol_per_gDW_per_hr" constant="true"/>
    </listOfParameters>
  </kineticLaw>
</reaction>
<reaction id="R_R185" name="Deoxycytidine aminohydrolase" reversible="false">
  <notes>
    <body xmlns="http://www.w3.org/1999/xhtml">
      <p>GENE_ASSOCIATION: TTC0383</p>
      <p>SUBSYSTEM: Nucleotide Salvage Pathway</p>
      <p>EC Number: 3.5.4.5</p>
      <p>Confidence Level: 1</p>
```

```xml
      <p>AUTHORS: Nucleotides metabolism</p>
      <p/>
    </body>
  </notes>
  <listOfReactants>
    <speciesReference species="M_H2O_c" stoichiometry="1"/>
    <speciesReference species="M_H_c" stoichiometry="1"/>
    <speciesReference species="M_dCYTD_c" stoichiometry="1"/>
  </listOfReactants>
  <listOfProducts>
    <speciesReference species="M_NH4_c" stoichiometry="1"/>
    <speciesReference species="M_dURD_c" stoichiometry="1"/>
  </listOfProducts>
  <kineticLaw>
    <math xmlns="http://www.w3.org/1998/Math/MathML">
      <ci> FLUX_VALUE </ci>
    </math>
    <listOfParameters>
      <parameter id="LOWER_BOUND" value="0" units="mmol_per_gDW_per_hr" constant="true"/>
      <parameter id="UPPER_BOUND" value="1000" units="mmol_per_gDW_per_hr" constant="true"/>
      <parameter id="FLUX_VALUE" value="0" units="mmol_per_gDW_per_hr" constant="true"/>
      <parameter id="OBJECTIVE_COEFFICIENT" value="0" units="mmol_per_gDW_per_hr" constant="true"/>
    </listOfParameters>
  </kineticLaw>
</reaction>
<reaction id="R_R186" name="5,10-methylenetetrahydrofolate,NADPH:dUMP C-methyltransferase" reversible="false">
  <notes>
    <body xmlns="http://www.w3.org/1999/xhtml">
      <p>GENE_ASSOCIATION: TTC0731</p>
```

```
      <p>SUBSYSTEM: Nucleotide Salvage Pathway</p>
      <p>EC Number: 2.1.1.148</p>
      <p>Confidence Level: 1</p>
      <p>AUTHORS: Nucleotides metabolism</p>
      <p/>
    </body>
  </notes>
  <listOfReactants>
    <speciesReference species="M_NADPH_c" stoichiometry="1"/>
    <speciesReference species="M_dUMP_c" stoichiometry="1"/>
    <speciesReference species="M_5_10_MNTHF_c" stoichiometry="1"/>
  </listOfReactants>
  <listOfProducts>
    <speciesReference species="M_NADP_c" stoichiometry="1"/>
    <speciesReference species="M_THF_c" stoichiometry="1"/>
    <speciesReference species="M_dTMP_c" stoichiometry="1"/>
  </listOfProducts>
  <kineticLaw>
    <math xmlns="http://www.w3.org/1998/Math/MathML">
      <ci> FLUX_VALUE </ci>
    </math>
    <listOfParameters>
      <parameter id="LOWER_BOUND" value="0" units="mmol_per_gDW_per_hr" constant="true"/>
      <parameter id="UPPER_BOUND" value="1000" units="mmol_per_gDW_per_hr" constant="true"/>
      <parameter id="FLUX_VALUE" value="0" units="mmol_per_gDW_per_hr" constant="true"/>
      <parameter id="OBJECTIVE_COEFFICIENT" value="0" units="mmol_per_gDW_per_hr" constant="true"/>
    </listOfParameters>
  </kineticLaw>
</reaction>
```

```
<reaction id="R_R187" name="deoxyuridine:orthophosphate 2-deoxy-D-ribosyltransferase" reversible="false">
 <notes>
  <body xmlns="http://www.w3.org/1999/xhtml">
   <p>GENE_ASSOCIATION: ((TTC0194 or TTC1070) or TTC1412)</p>
   <p>SUBSYSTEM: Nucleotide Salvage Pathway</p>
   <p>EC Number: 2.4.2.1 or 2.4.2.4</p>
   <p>Confidence Level: 1</p>
   <p>AUTHORS: Nucleotides metabolism</p>
   <p/>
  </body>
 </notes>
 <listOfReactants>
  <speciesReference species="M_Pi_c" stoichiometry="1"/>
  <speciesReference species="M_dURD_c" stoichiometry="1"/>
 </listOfReactants>
 <listOfProducts>
  <speciesReference species="M_dR1P_c" stoichiometry="1"/>
  <speciesReference species="M_URA_c" stoichiometry="1"/>
 </listOfProducts>
 <kineticLaw>
  <math xmlns="http://www.w3.org/1998/Math/MathML">
   <ci> FLUX_VALUE </ci>
  </math>
  <listOfParameters>
   <parameter id="LOWER_BOUND" value="0" units="mmol_per_gDW_per_hr" constant="true"/>
   <parameter id="UPPER_BOUND" value="1000" units="mmol_per_gDW_per_hr" constant="true"/>
   <parameter id="FLUX_VALUE" value="0" units="mmol_per_gDW_per_hr" constant="true"/>
   <parameter id="OBJECTIVE_COEFFICIENT" value="0" units="mmol_per_gDW_per_hr" constant="true"/>
  </listOfParameters>
```

```
  </kineticLaw>
</reaction>
<reaction id="R_R188" name="thymidine:phosphate deoxy-alpha-D-ribosyltransferase" reversible="true">
  <notes>
    <body xmlns="http://www.w3.org/1999/xhtml">
      <p>GENE_ASSOCIATION: TTC1412</p>
      <p>SUBSYSTEM: Nucleotide Salvage Pathway</p>
      <p>EC Number: 2.4.2.4</p>
      <p>Confidence Level: 1</p>
      <p>AUTHORS: Nucleotides metabolism</p>
      <p/>
    </body>
  </notes>
  <listOfReactants>
    <speciesReference species="M_Pi_c" stoichiometry="1"/>
    <speciesReference species="M_THYMD_c" stoichiometry="1"/>
  </listOfReactants>
  <listOfProducts>
    <speciesReference species="M_dR1P_c" stoichiometry="1"/>
    <speciesReference species="M_THYM_c" stoichiometry="1"/>
  </listOfProducts>
  <kineticLaw>
    <math xmlns="http://www.w3.org/1998/Math/MathML">
      <ci> FLUX_VALUE </ci>
    </math>
    <listOfParameters>
      <parameter id="LOWER_BOUND" value="-1000" units="mmol_per_gDW_per_hr" constant="true"/>
      <parameter id="UPPER_BOUND" value="1000" units="mmol_per_gDW_per_hr" constant="true"/>
      <parameter id="FLUX_VALUE" value="0" units="mmol_per_gDW_per_hr" constant="true"/>
```

```
      <parameter id="OBJECTIVE_COEFFICIENT" value="0" units="mmol_per_gDW_per_hr" constant="true"/>
    </listOfParameters>
  </kineticLaw>
</reaction>
<reaction id="R_R189" name="dihydropyrimidine dehydrogenase" reversible="false">
  <notes>
    <body xmlns="http://www.w3.org/1999/xhtml">
      <p>GENE_ASSOCIATION: </p>
      <p>SUBSYSTEM: Nucleotide Salvage Pathway</p>
      <p>EC Number: 1.3.1.2</p>
      <p>Confidence Level: 1</p>
      <p>AUTHORS: Nucleotides metabolism</p>
      <p/>
    </body>
  </notes>
  <listOfReactants>
    <speciesReference species="M_H_c" stoichiometry="1"/>
    <speciesReference species="M_NADPH_c" stoichiometry="1"/>
    <speciesReference species="M_THYM_c" stoichiometry="1"/>
  </listOfReactants>
  <listOfProducts>
    <speciesReference species="M_NADP_c" stoichiometry="1"/>
    <speciesReference species="M_DHTHYM_c" stoichiometry="1"/>
  </listOfProducts>
  <kineticLaw>
    <math xmlns="http://www.w3.org/1998/Math/MathML">
      <ci> FLUX_VALUE </ci>
    </math>
    <listOfParameters>
```

```
      <parameter id="LOWER_BOUND" value="0" units="mmol_per_gDW_per_hr" constant="true"/>
      <parameter id="UPPER_BOUND" value="1000" units="mmol_per_gDW_per_hr" constant="true"/>
      <parameter id="FLUX_VALUE" value="0" units="mmol_per_gDW_per_hr" constant="true"/>
      <parameter id="OBJECTIVE_COEFFICIENT" value="0" units="mmol_per_gDW_per_hr" constant="true"/>
    </listOfParameters>
  </kineticLaw>
</reaction>
<reaction id="R_R190" name="dihydropyrimidinase" reversible="false">
  <notes>
    <body xmlns="http://www.w3.org/1999/xhtml">
      <p>GENE_ASSOCIATION: TTC0426</p>
      <p>SUBSYSTEM: Nucleotide Salvage Pathway</p>
      <p>EC Number: 3.5.2.2</p>
      <p>Confidence Level: 1</p>
      <p>AUTHORS: Nucleotides metabolism</p>
      <p/>
    </body>
  </notes>
  <listOfReactants>
    <speciesReference species="M_H2O_c" stoichiometry="1"/>
    <speciesReference species="M_DHTHYM_c" stoichiometry="1"/>
  </listOfReactants>
  <listOfProducts>
    <speciesReference species="M_H_c" stoichiometry="1"/>
    <speciesReference species="M_UREIDO_BUTR_c" stoichiometry="1"/>
  </listOfProducts>
  <kineticLaw>
    <math xmlns="http://www.w3.org/1998/Math/MathML">
      <ci> FLUX_VALUE </ci>
```

```xml
      </math>
      <listOfParameters>
        <parameter id="LOWER_BOUND" value="0" units="mmol_per_gDW_per_hr" constant="true"/>
        <parameter id="UPPER_BOUND" value="1000" units="mmol_per_gDW_per_hr" constant="true"/>
        <parameter id="FLUX_VALUE" value="0" units="mmol_per_gDW_per_hr" constant="true"/>
        <parameter id="OBJECTIVE_COEFFICIENT" value="0" units="mmol_per_gDW_per_hr" constant="true"/>
      </listOfParameters>
    </kineticLaw>
  </reaction>
  <reaction id="R_R191" name="3-Ureidoisobutyrate amidohydrolase" reversible="false">
    <notes>
      <body xmlns="http://www.w3.org/1999/xhtml">
        <p>GENE_ASSOCIATION: TTC1539</p>
        <p>SUBSYSTEM: Nucleotide Salvage Pathway</p>
        <p>EC Number: 3.5.1.6</p>
        <p>Confidence Level: 1</p>
        <p>AUTHORS: Nucleotides metabolism</p>
        <p/>
      </body>
    </notes>
    <listOfReactants>
      <speciesReference species="M_H2O_c" stoichiometry="1"/>
      <speciesReference species="M_UREIDO_BUTR_c" stoichiometry="1"/>
    </listOfReactants>
    <listOfProducts>
      <speciesReference species="M_CO2_c" stoichiometry="1"/>
      <speciesReference species="M_NH4_c" stoichiometry="1"/>
      <speciesReference species="M_3AIBUTNA_c" stoichiometry="1"/>
    </listOfProducts>
```

```
  <kineticLaw>
    <math xmlns="http://www.w3.org/1998/Math/MathML">
      <ci> FLUX_VALUE </ci>
    </math>
    <listOfParameters>
      <parameter id="LOWER_BOUND" value="0" units="mmol_per_gDW_per_hr" constant="true"/>
      <parameter id="UPPER_BOUND" value="1000" units="mmol_per_gDW_per_hr" constant="true"/>
      <parameter id="FLUX_VALUE" value="0" units="mmol_per_gDW_per_hr" constant="true"/>
      <parameter id="OBJECTIVE_COEFFICIENT" value="0" units="mmol_per_gDW_per_hr" constant="true"/>
    </listOfParameters>
  </kineticLaw>
</reaction>
<reaction id="R_R192" name="L-Alanine:NAD+ oxidoreductase (deaminating)" reversible="true">
  <notes>
    <body xmlns="http://www.w3.org/1999/xhtml">
      <p>GENE_ASSOCIATION: (TTC1082 or TTC1770)</p>
      <p>SUBSYSTEM: Alanine and Aspartate Metabolism</p>
      <p>EC Number: 1.4.1.1</p>
      <p>Confidence Level: 1</p>
      <p>AUTHORS: Amino acid metabolism</p>
      <p/>
    </body>
  </notes>
  <listOfReactants>
    <speciesReference species="M_H_c" stoichiometry="1"/>
    <speciesReference species="M_NADH_c" stoichiometry="1"/>
    <speciesReference species="M_PYR_c" stoichiometry="1"/>
    <speciesReference species="M_NH4_c" stoichiometry="1"/>
  </listOfReactants>
```

```
 <listOfProducts>
  <speciesReference species="M_L_ALA_c" stoichiometry="1"/>
  <speciesReference species="M_H2O_c" stoichiometry="1"/>
  <speciesReference species="M_NAD_c" stoichiometry="1"/>
 </listOfProducts>
 <kineticLaw>
  <math xmlns="http://www.w3.org/1998/Math/MathML">
   <ci> FLUX_VALUE </ci>
  </math>
  <listOfParameters>
   <parameter id="LOWER_BOUND" value="-1000" units="mmol_per_gDW_per_hr" constant="true"/>
   <parameter id="UPPER_BOUND" value="1000" units="mmol_per_gDW_per_hr" constant="true"/>
   <parameter id="FLUX_VALUE" value="0" units="mmol_per_gDW_per_hr" constant="true"/>
   <parameter id="OBJECTIVE_COEFFICIENT" value="0" units="mmol_per_gDW_per_hr" constant="true"/>
  </listOfParameters>
 </kineticLaw>
</reaction>
<reaction id="R_R193" name="L-Alanine:glyoxylate aminotransferase" reversible="false">
 <notes>
  <body xmlns="http://www.w3.org/1999/xhtml">
   <p>GENE_ASSOCIATION: TTC1813</p>
   <p>SUBSYSTEM: Alanine and Aspartate Metabolism</p>
   <p>EC Number: 2.6.1.44</p>
   <p>Confidence Level: 1</p>
   <p>AUTHORS: Amino acid metabolism</p>
   <p/>
  </body>
 </notes>
 <listOfReactants>
```

```
    <speciesReference species="M_L_ALA_c" stoichiometry="1"/>
    <speciesReference species="M_GLOXT_c" stoichiometry="1"/>
  </listOfReactants>
  <listOfProducts>
    <speciesReference species="M_L_GLY_c" stoichiometry="1"/>
    <speciesReference species="M_PYR_c" stoichiometry="1"/>
  </listOfProducts>
  <kineticLaw>
    <math xmlns="http://www.w3.org/1998/Math/MathML">
      <ci> FLUX_VALUE </ci>
    </math>
    <listOfParameters>
      <parameter id="LOWER_BOUND" value="0" units="mmol_per_gDW_per_hr" constant="true"/>
      <parameter id="UPPER_BOUND" value="1000" units="mmol_per_gDW_per_hr" constant="true"/>
      <parameter id="FLUX_VALUE" value="0" units="mmol_per_gDW_per_hr" constant="true"/>
      <parameter id="OBJECTIVE_COEFFICIENT" value="0" units="mmol_per_gDW_per_hr" constant="true"/>
    </listOfParameters>
  </kineticLaw>
</reaction>
<reaction id="R_R194" name="L-Citrulline:L-aspartate ligase (AMP-forming)" reversible="true">
  <notes>
    <body xmlns="http://www.w3.org/1999/xhtml">
      <p>GENE_ASSOCIATION: TTC1701</p>
      <p>SUBSYSTEM: Arginine and Proline Metabolism</p>
      <p>EC Number: 6.3.4.5</p>
      <p>Confidence Level: 1</p>
      <p>AUTHORS: Amino acid metabolism</p>
      <p/>
    </body>
```

```
  </notes>
  <listOfReactants>
    <speciesReference species="M_L_ASP_c" stoichiometry="1"/>
    <speciesReference species="M_ATP_c" stoichiometry="1"/>
    <speciesReference species="M_CTRLN_c" stoichiometry="1"/>
  </listOfReactants>
  <listOfProducts>
    <speciesReference species="M_PPi_c" stoichiometry="1"/>
    <speciesReference species="M_H_c" stoichiometry="1"/>
    <speciesReference species="M_AMP_c" stoichiometry="1"/>
    <speciesReference species="M_L_ARG_SUCC_c" stoichiometry="1"/>
  </listOfProducts>
  <kineticLaw>
    <math xmlns="http://www.w3.org/1998/Math/MathML">
      <ci> FLUX_VALUE </ci>
    </math>
    <listOfParameters>
      <parameter id="LOWER_BOUND" value="-1000" units="mmol_per_gDW_per_hr" constant="true"/>
      <parameter id="UPPER_BOUND" value="1000" units="mmol_per_gDW_per_hr" constant="true"/>
      <parameter id="FLUX_VALUE" value="0" units="mmol_per_gDW_per_hr" constant="true"/>
      <parameter id="OBJECTIVE_COEFFICIENT" value="0" units="mmol_per_gDW_per_hr" constant="true"/>
    </listOfParameters>
  </kineticLaw>
</reaction>
<reaction id="R_R195" name="2-(Nomega-L-arginino)succinate arginine-lyase (fumarate-forming)" reversible="false">
  <notes>
    <body xmlns="http://www.w3.org/1999/xhtml">
      <p>GENE_ASSOCIATION: TTC1702</p>
      <p>SUBSYSTEM: Arginine and Proline Metabolism</p>
```

```
      <p>EC Number: 4.3.2.1</p>
      <p>Confidence Level: 1</p>
      <p>AUTHORS: Amino acid metabolism</p>
      <p/>
    </body>
  </notes>
  <listOfReactants>
    <speciesReference species="M_L_ARG_SUCC_c" stoichiometry="1"/>
  </listOfReactants>
  <listOfProducts>
    <speciesReference species="M_L_ARG_c" stoichiometry="1"/>
    <speciesReference species="M_FUM_c" stoichiometry="1"/>
  </listOfProducts>
  <kineticLaw>
    <math xmlns="http://www.w3.org/1998/Math/MathML">
      <ci> FLUX_VALUE </ci>
    </math>
    <listOfParameters>
      <parameter id="LOWER_BOUND" value="0" units="mmol_per_gDW_per_hr" constant="true"/>
      <parameter id="UPPER_BOUND" value="1000" units="mmol_per_gDW_per_hr" constant="true"/>
      <parameter id="FLUX_VALUE" value="0" units="mmol_per_gDW_per_hr" constant="true"/>
      <parameter id="OBJECTIVE_COEFFICIENT" value="0" units="mmol_per_gDW_per_hr" constant="true"/>
    </listOfParameters>
  </kineticLaw>
</reaction>
<reaction id="R_R196" name="L-aspartate:ammonia ligase (AMP-forming)" reversible="true">
  <notes>
    <body xmlns="http://www.w3.org/1999/xhtml">
      <p>GENE_ASSOCIATION: TTC0282</p>
```

```xml
    <p>SUBSYSTEM: Alanine and Aspartate Metabolism</p>
    <p>EC Number: 6.3.5.4</p>
    <p>Confidence Level: 1</p>
    <p>AUTHORS: Amino acid metabolism</p>
    <p/>
  </body>
</notes>
<listOfReactants>
  <speciesReference species="M_L_ASP_c" stoichiometry="1"/>
  <speciesReference species="M_ATP_c" stoichiometry="1"/>
  <speciesReference species="M_NH4_c" stoichiometry="1"/>
</listOfReactants>
<listOfProducts>
  <speciesReference species="M_L_ASN_c" stoichiometry="1"/>
  <speciesReference species="M_PPi_c" stoichiometry="1"/>
  <speciesReference species="M_H_c" stoichiometry="1"/>
  <speciesReference species="M_AMP_c" stoichiometry="1"/>
</listOfProducts>
<kineticLaw>
  <math xmlns="http://www.w3.org/1998/Math/MathML">
    <ci> FLUX_VALUE </ci>
  </math>
  <listOfParameters>
    <parameter id="LOWER_BOUND" value="-1000" units="mmol_per_gDW_per_hr" constant="true"/>
    <parameter id="UPPER_BOUND" value="1000" units="mmol_per_gDW_per_hr" constant="true"/>
    <parameter id="FLUX_VALUE" value="0" units="mmol_per_gDW_per_hr" constant="true"/>
    <parameter id="OBJECTIVE_COEFFICIENT" value="0" units="mmol_per_gDW_per_hr" constant="true"/>
  </listOfParameters>
</kineticLaw>
```

```
</reaction>
<reaction id="R_R197" name="L-asparginase" reversible="false">
  <notes>
    <body xmlns="http://www.w3.org/1999/xhtml">
      <p>GENE_ASSOCIATION: TTC1938</p>
      <p>SUBSYSTEM: Alanine and Aspartate Metabolism</p>
      <p>EC Number: 3.5.1.1</p>
      <p>Confidence Level: 1</p>
      <p>AUTHORS: Amino acid metabolism</p>
      <p/>
    </body>
  </notes>
  <listOfReactants>
    <speciesReference species="M_L_ASN_c" stoichiometry="1"/>
    <speciesReference species="M_H2O_c" stoichiometry="1"/>
  </listOfReactants>
  <listOfProducts>
    <speciesReference species="M_L_ASP_c" stoichiometry="1"/>
    <speciesReference species="M_H_c" stoichiometry="1"/>
    <speciesReference species="M_NH4_c" stoichiometry="1"/>
  </listOfProducts>
  <kineticLaw>
    <math xmlns="http://www.w3.org/1998/Math/MathML">
      <ci> FLUX_VALUE </ci>
    </math>
    <listOfParameters>
      <parameter id="LOWER_BOUND" value="0" units="mmol_per_gDW_per_hr" constant="true"/>
      <parameter id="UPPER_BOUND" value="1000" units="mmol_per_gDW_per_hr" constant="true"/>
      <parameter id="FLUX_VALUE" value="0" units="mmol_per_gDW_per_hr" constant="true"/>
```

```
      <parameter id="OBJECTIVE_COEFFICIENT" value="0" units="mmol_per_gDW_per_hr" constant="true"/>
    </listOfParameters>
  </kineticLaw>
</reaction>
<reaction id="R_R198" name="L-Aspartate:2-oxoglutarate aminotransferase" reversible="true">
  <notes>
    <body xmlns="http://www.w3.org/1999/xhtml">
      <p>GENE_ASSOCIATION: (TTC1960 or TTC1641)</p>
      <p>SUBSYSTEM: Alanine and Aspartate Metabolism</p>
      <p>EC Number: 2.6.1.1</p>
      <p>Confidence Level: 1</p>
      <p>AUTHORS: Amino acid metabolism</p>
      <p/>
    </body>
  </notes>
  <listOfReactants>
    <speciesReference species="M_L_ASP_c" stoichiometry="1"/>
    <speciesReference species="M_AKG_c" stoichiometry="1"/>
  </listOfReactants>
  <listOfProducts>
    <speciesReference species="M_L_GLU_c" stoichiometry="1"/>
    <speciesReference species="M_OAC_c" stoichiometry="1"/>
  </listOfProducts>
  <kineticLaw>
    <math xmlns="http://www.w3.org/1998/Math/MathML">
      <ci> FLUX_VALUE </ci>
    </math>
    <listOfParameters>
      <parameter id="LOWER_BOUND" value="-1000" units="mmol_per_gDW_per_hr" constant="true"/>
```

```
      <parameter id="UPPER_BOUND" value="1000" units="mmol_per_gDW_per_hr" constant="true"/>
      <parameter id="FLUX_VALUE" value="0" units="mmol_per_gDW_per_hr" constant="true"/>
      <parameter id="OBJECTIVE_COEFFICIENT" value="0" units="mmol_per_gDW_per_hr" constant="true"/>
    </listOfParameters>
  </kineticLaw>
</reaction>
<reaction id="R_R199" name="Succinate-semialdehyde:NADP+ oxidoreductase" reversible="false">
  <notes>
    <body xmlns="http://www.w3.org/1999/xhtml">
      <p>GENE_ASSOCIATION: TTC0634</p>
      <p>SUBSYSTEM: Arginine and Proline Metabolism</p>
      <p>EC Number: 1.2.1.16</p>
      <p>Confidence Level: 1</p>
      <p>AUTHORS: Amino acid metabolism</p>
      <p/>
    </body>
  </notes>
  <listOfReactants>
    <speciesReference species="M_H2O_c" stoichiometry="1"/>
    <speciesReference species="M_NADP_c" stoichiometry="1"/>
    <speciesReference species="M_SUCC_SA_c" stoichiometry="1"/>
  </listOfReactants>
  <listOfProducts>
    <speciesReference species="M_H_c" stoichiometry="2"/>
    <speciesReference species="M_NADPH_c" stoichiometry="1"/>
    <speciesReference species="M_SUCC_c" stoichiometry="1"/>
  </listOfProducts>
  <kineticLaw>
    <math xmlns="http://www.w3.org/1998/Math/MathML">
```

```
      <ci> FLUX_VALUE </ci>
    </math>
    <listOfParameters>
      <parameter id="LOWER_BOUND" value="0" units="mmol_per_gDW_per_hr" constant="true"/>
      <parameter id="UPPER_BOUND" value="1000" units="mmol_per_gDW_per_hr" constant="true"/>
      <parameter id="FLUX_VALUE" value="0" units="mmol_per_gDW_per_hr" constant="true"/>
      <parameter id="OBJECTIVE_COEFFICIENT" value="0" units="mmol_per_gDW_per_hr" constant="true"/>
    </listOfParameters>
  </kineticLaw>
</reaction>
<reaction id="R_R200" name="4-aminobutanoate:2-oxoglutarate aminotransferase" reversible="false">
  <notes>
    <body xmlns="http://www.w3.org/1999/xhtml">
      <p>GENE_ASSOCIATION: TTC0510</p>
      <p>SUBSYSTEM: Arginine and Proline Metabolism</p>
      <p>EC Number: 2.6.1.19</p>
      <p>Confidence Level: 1</p>
      <p>AUTHORS: Amino acid metabolism</p>
      <p/>
    </body>
  </notes>
  <listOfReactants>
    <speciesReference species="M_AKG_c" stoichiometry="1"/>
    <speciesReference species="M_4ABUT_c" stoichiometry="1"/>
  </listOfReactants>
  <listOfProducts>
    <speciesReference species="M_L_GLU_c" stoichiometry="1"/>
    <speciesReference species="M_SUCC_SA_c" stoichiometry="1"/>
  </listOfProducts>
```

```xml
  <kineticLaw>
    <math xmlns="http://www.w3.org/1998/Math/MathML">
      <ci> FLUX_VALUE </ci>
    </math>
    <listOfParameters>
      <parameter id="LOWER_BOUND" value="0" units="mmol_per_gDW_per_hr" constant="true"/>
      <parameter id="UPPER_BOUND" value="1000" units="mmol_per_gDW_per_hr" constant="true"/>
      <parameter id="FLUX_VALUE" value="0" units="mmol_per_gDW_per_hr" constant="true"/>
      <parameter id="OBJECTIVE_COEFFICIENT" value="0" units="mmol_per_gDW_per_hr" constant="true"/>
    </listOfParameters>
  </kineticLaw>
</reaction>
<reaction id="R_R201" name="L-glutamate:NAD+ oxidoreductase (deaminating)" reversible="true">
  <notes>
    <body xmlns="http://www.w3.org/1999/xhtml">
      <p>GENE_ASSOCIATION: (TTC1211 or TTC1212)</p>
      <p>SUBSYSTEM: Glutamate Metabolism</p>
      <p>EC Number: 1.4.1.3</p>
      <p>Confidence Level: 1</p>
      <p>AUTHORS: Amino acid metabolism</p>
      <p/>
    </body>
  </notes>
  <listOfReactants>
    <speciesReference species="M_L_GLU_c" stoichiometry="1"/>
    <speciesReference species="M_H2O_c" stoichiometry="1"/>
    <speciesReference species="M_NAD_c" stoichiometry="1"/>
  </listOfReactants>
  <listOfProducts>
```

```
    <speciesReference species="M_H_c" stoichiometry="1"/>
    <speciesReference species="M_NADH_c" stoichiometry="1"/>
    <speciesReference species="M_AKG_c" stoichiometry="1"/>
    <speciesReference species="M_NH4_c" stoichiometry="1"/>
  </listOfProducts>
  <kineticLaw>
    <math xmlns="http://www.w3.org/1998/Math/MathML">
      <ci> FLUX_VALUE </ci>
    </math>
    <listOfParameters>
      <parameter id="LOWER_BOUND" value="-1000" units="mmol_per_gDW_per_hr" constant="true"/>
      <parameter id="UPPER_BOUND" value="1000" units="mmol_per_gDW_per_hr" constant="true"/>
      <parameter id="FLUX_VALUE" value="0" units="mmol_per_gDW_per_hr" constant="true"/>
      <parameter id="OBJECTIVE_COEFFICIENT" value="0" units="mmol_per_gDW_per_hr" constant="true"/>
    </listOfParameters>
  </kineticLaw>
</reaction>
<reaction id="R_R202" name="L-Glutamate:NADP+ oxidoreductase (deaminating)" reversible="true">
  <notes>
    <body xmlns="http://www.w3.org/1999/xhtml">
      <p>GENE_ASSOCIATION: (TTC1104 or (TTC1211 or TTC1212))</p>
      <p>SUBSYSTEM: Glutamate Metabolism</p>
      <p>EC Number: (1.4.1.13 or 1.4.1.14)</p>
      <p>Confidence Level: 1</p>
      <p>AUTHORS: Amino acid metabolism</p>
      <p/>
    </body>
  </notes>
  <listOfReactants>
```

```
    <speciesReference species="M_L_GLU_c" stoichiometry="1"/>
    <speciesReference species="M_H2O_c" stoichiometry="1"/>
    <speciesReference species="M_NADP_c" stoichiometry="1"/>
  </listOfReactants>
  <listOfProducts>
    <speciesReference species="M_H_c" stoichiometry="1"/>
    <speciesReference species="M_NADPH_c" stoichiometry="1"/>
    <speciesReference species="M_AKG_c" stoichiometry="1"/>
    <speciesReference species="M_NH4_c" stoichiometry="1"/>
  </listOfProducts>
  <kineticLaw>
    <math xmlns="http://www.w3.org/1998/Math/MathML">
      <ci> FLUX_VALUE </ci>
    </math>
    <listOfParameters>
      <parameter id="LOWER_BOUND" value="-1000" units="mmol_per_gDW_per_hr" constant="true"/>
      <parameter id="UPPER_BOUND" value="1000" units="mmol_per_gDW_per_hr" constant="true"/>
      <parameter id="FLUX_VALUE" value="0" units="mmol_per_gDW_per_hr" constant="true"/>
      <parameter id="OBJECTIVE_COEFFICIENT" value="0" units="mmol_per_gDW_per_hr" constant="true"/>
    </listOfParameters>
  </kineticLaw>
</reaction>
<reaction id="R_R203" name="L-Glutamate:NADP+ oxidoreductase (transaminating)" reversible="false">
  <notes>
    <body xmlns="http://www.w3.org/1999/xhtml">
      <p>GENE_ASSOCIATION: TTC1104</p>
      <p>SUBSYSTEM: Glutamate Metabolism </p>
      <p>EC Number: 1.4.1.13</p>
      <p>Confidence Level: 1</p>
```

```
      <p>AUTHORS: Amino acid metabolism</p>
      <p/>
    </body>
  </notes>
  <listOfReactants>
    <speciesReference species="M_L_GLN_c" stoichiometry="1"/>
    <speciesReference species="M_H_c" stoichiometry="1"/>
    <speciesReference species="M_NADPH_c" stoichiometry="1"/>
    <speciesReference species="M_AKG_c" stoichiometry="1"/>
  </listOfReactants>
  <listOfProducts>
    <speciesReference species="M_L_GLU_c" stoichiometry="2"/>
    <speciesReference species="M_NADP_c" stoichiometry="1"/>
  </listOfProducts>
  <kineticLaw>
    <math xmlns="http://www.w3.org/1998/Math/MathML">
      <ci> FLUX_VALUE </ci>
    </math>
    <listOfParameters>
      <parameter id="LOWER_BOUND" value="0" units="mmol_per_gDW_per_hr" constant="true"/>
      <parameter id="UPPER_BOUND" value="1000" units="mmol_per_gDW_per_hr" constant="true"/>
      <parameter id="FLUX_VALUE" value="0" units="mmol_per_gDW_per_hr" constant="true"/>
      <parameter id="OBJECTIVE_COEFFICIENT" value="0" units="mmol_per_gDW_per_hr" constant="true"/>
    </listOfParameters>
  </kineticLaw>
</reaction>
<reaction id="R_R204" name="(S)-1-pyrroline-5-carboxylate:NAD+ oxidoreductase" reversible="false">
  <notes>
    <body xmlns="http://www.w3.org/1999/xhtml">
```

```xml
    <p>GENE_ASSOCIATION: TTC1213</p>

    <p>SUBSYSTEM: Arginine and Proline Metabolism</p>

    <p>EC Number: 1.5.1.12</p>

    <p>Confidence Level: 1</p>

    <p>AUTHORS: Amino acid metabolism</p>

    <p/>

  </body>

</notes>

<listOfReactants>

  <speciesReference species="M_H2O_c" stoichiometry="2"/>

  <speciesReference species="M_NAD_c" stoichiometry="1"/>

  <speciesReference species="M_PYRR5CARB_c" stoichiometry="1"/>

</listOfReactants>

<listOfProducts>

  <speciesReference species="M_L_GLU_c" stoichiometry="1"/>

  <speciesReference species="M_H_c" stoichiometry="1"/>

  <speciesReference species="M_NADH_c" stoichiometry="1"/>

</listOfProducts>

<kineticLaw>

  <math xmlns="http://www.w3.org/1998/Math/MathML">

    <ci> FLUX_VALUE </ci>

  </math>

  <listOfParameters>

    <parameter id="LOWER_BOUND" value="0" units="mmol_per_gDW_per_hr" constant="true"/>

    <parameter id="UPPER_BOUND" value="1000" units="mmol_per_gDW_per_hr" constant="true"/>

    <parameter id="FLUX_VALUE" value="0" units="mmol_per_gDW_per_hr" constant="true"/>

    <parameter id="OBJECTIVE_COEFFICIENT" value="0" units="mmol_per_gDW_per_hr" constant="true"/>

  </listOfParameters>

</kineticLaw>
```

```xml
</reaction>
<reaction id="R_R205" name="L-Glutamate:ammonia ligase (ADP-forming)" reversible="false">
 <notes>
  <body xmlns="http://www.w3.org/1999/xhtml">
   <p>GENE_ASSOCIATION: TTC0965</p>
   <p>SUBSYSTEM: Glutamate Metabolism </p>
   <p>EC Number: 6.3.1.2</p>
   <p>Confidence Level: 1</p>
   <p>AUTHORS: Amino acid metabolism</p>
   <p/>
  </body>
 </notes>
 <listOfReactants>
  <speciesReference species="M_L_GLU_c" stoichiometry="1"/>
  <speciesReference species="M_ATP_c" stoichiometry="1"/>
  <speciesReference species="M_NH4_c" stoichiometry="1"/>
 </listOfReactants>
 <listOfProducts>
  <speciesReference species="M_L_GLN_c" stoichiometry="1"/>
  <speciesReference species="M_ADP_c" stoichiometry="1"/>
  <speciesReference species="M_Pi_c" stoichiometry="1"/>
  <speciesReference species="M_H_c" stoichiometry="1"/>
 </listOfProducts>
 <kineticLaw>
  <math xmlns="http://www.w3.org/1998/Math/MathML">
   <ci> FLUX_VALUE </ci>
  </math>
  <listOfParameters>
   <parameter id="LOWER_BOUND" value="0" units="mmol_per_gDW_per_hr" constant="true"/>
```

```
      <parameter id="UPPER_BOUND" value="1000" units="mmol_per_gDW_per_hr" constant="true"/>
      <parameter id="FLUX_VALUE" value="0" units="mmol_per_gDW_per_hr" constant="true"/>
      <parameter id="OBJECTIVE_COEFFICIENT" value="0" units="mmol_per_gDW_per_hr" constant="true"/>
    </listOfParameters>
  </kineticLaw>
</reaction>
<reaction id="R_R206" name="L-glutamine:D-fructose-6-phosphate isomerase (deaminating)" reversible="false">
  <notes>
    <body xmlns="http://www.w3.org/1999/xhtml">
      <p>GENE_ASSOCIATION: TTC1533</p>
      <p>SUBSYSTEM: Glutamate Metabolism </p>
      <p>EC Number: 2.6.1.16</p>
      <p>Confidence Level: 1</p>
      <p>AUTHORS: Amino acid metabolism</p>
      <p/>
    </body>
  </notes>
  <listOfReactants>
    <speciesReference species="M_L_GLN_c" stoichiometry="1"/>
    <speciesReference species="M_F6P_c" stoichiometry="1"/>
  </listOfReactants>
  <listOfProducts>
    <speciesReference species="M_L_GLU_c" stoichiometry="1"/>
    <speciesReference species="M_GLUSAP_c" stoichiometry="1"/>
  </listOfProducts>
  <kineticLaw>
    <math xmlns="http://www.w3.org/1998/Math/MathML">
      <ci> FLUX_VALUE </ci>
    </math>
```

```
<listOfParameters>
<parameter id="LOWER_BOUND" value="0" units="mmol_per_gDW_per_hr" constant="true"/>
<parameter id="UPPER_BOUND" value="1000" units="mmol_per_gDW_per_hr" constant="true"/>
<parameter id="FLUX_VALUE" value="0" units="mmol_per_gDW_per_hr" constant="true"/>
<parameter id="OBJECTIVE_COEFFICIENT" value="0" units="mmol_per_gDW_per_hr" constant="true"/>
</listOfParameters>
</kineticLaw>
</reaction>
<reaction id="R_R207" name="D-Glycerate:NAD+ 2-oxidoreductase" reversible="false">
<notes>
<body xmlns="http://www.w3.org/1999/xhtml">
<p>GENE_ASSOCIATION: (TTC0431 or TTC0124)</p>
<p>SUBSYSTEM: Alternate Carbon Metabolism</p>
<p>EC Number: (1.1.1.26 or 1.1.1.81)</p>
<p>Confidence Level: 1</p>
<p>AUTHORS: Carbohydrate metabolism</p>
<p/>
</body>
</notes>
<listOfReactants>
<speciesReference species="M_H_c" stoichiometry="1"/>
<speciesReference species="M_NADH_c" stoichiometry="1"/>
<speciesReference species="M_HY_PYR_c" stoichiometry="1"/>
</listOfReactants>
<listOfProducts>
<speciesReference species="M_NAD_c" stoichiometry="1"/>
<speciesReference species="M_D_GLCT_c" stoichiometry="1"/>
</listOfProducts>
<kineticLaw>
```

```xml
<math xmlns="http://www.w3.org/1998/Math/MathML">
  <ci> FLUX_VALUE </ci>
</math>
<listOfParameters>
  <parameter id="LOWER_BOUND" value="0" units="mmol_per_gDW_per_hr" constant="true"/>
  <parameter id="UPPER_BOUND" value="1000" units="mmol_per_gDW_per_hr" constant="true"/>
  <parameter id="FLUX_VALUE" value="0" units="mmol_per_gDW_per_hr" constant="true"/>
  <parameter id="OBJECTIVE_COEFFICIENT" value="0" units="mmol_per_gDW_per_hr" constant="true"/>
</listOfParameters>
</kineticLaw>
</reaction>
<reaction id="R_R208" name="D-Glycerate:NADP+ 2-oxidoreductase" reversible="false">
 <notes>
  <body xmlns="http://www.w3.org/1999/xhtml">
   <p>GENE_ASSOCIATION: TTC0124</p>
   <p>SUBSYSTEM: Alternate Carbon Metabolism</p>
   <p>EC Number: 1.1.1.81</p>
   <p>Confidence Level: 1</p>
   <p>AUTHORS: Carbohydrate metabolism</p>
   <p/>
  </body>
 </notes>
 <listOfReactants>
  <speciesReference species="M_H_c" stoichiometry="1"/>
  <speciesReference species="M_NADPH_c" stoichiometry="1"/>
  <speciesReference species="M_HY_PYR_c" stoichiometry="1"/>
 </listOfReactants>
 <listOfProducts>
  <speciesReference species="M_NADP_c" stoichiometry="1"/>
```

```
    <speciesReference species="M_D_GLCT_c" stoichiometry="1"/>
  </listOfProducts>
  <kineticLaw>
    <math xmlns="http://www.w3.org/1998/Math/MathML">
      <ci> FLUX_VALUE </ci>
    </math>
    <listOfParameters>
      <parameter id="LOWER_BOUND" value="0" units="mmol_per_gDW_per_hr" constant="true"/>
      <parameter id="UPPER_BOUND" value="1000" units="mmol_per_gDW_per_hr" constant="true"/>
      <parameter id="FLUX_VALUE" value="0" units="mmol_per_gDW_per_hr" constant="true"/>
      <parameter id="OBJECTIVE_COEFFICIENT" value="0" units="mmol_per_gDW_per_hr" constant="true"/>
    </listOfParameters>
  </kineticLaw>
</reaction>
<reaction id="R_R209" name="L-Serine:pyruvate aminotransferase" reversible="false">
  <notes>
    <body xmlns="http://www.w3.org/1999/xhtml">
      <p>GENE_ASSOCIATION: TTC1813</p>
      <p>SUBSYSTEM: Glycine and Serine Metabolism</p>
      <p>EC Number: 2.6.1.51</p>
      <p>Confidence Level: 1</p>
      <p>AUTHORS: Amino acid metabolism</p>
      <p/>
    </body>
  </notes>
  <listOfReactants>
    <speciesReference species="M_L_SER_c" stoichiometry="1"/>
    <speciesReference species="M_PYR_c" stoichiometry="1"/>
  </listOfReactants>
```

```
  <listOfProducts>
   <speciesReference species="M_L_ALA_c" stoichiometry="1"/>
   <speciesReference species="M_HY_PYR_c" stoichiometry="1"/>
  </listOfProducts>
  <kineticLaw>
   <math xmlns="http://www.w3.org/1998/Math/MathML">
    <ci> FLUX_VALUE </ci>
   </math>
   <listOfParameters>
    <parameter id="LOWER_BOUND" value="0" units="mmol_per_gDW_per_hr" constant="true"/>
    <parameter id="UPPER_BOUND" value="1000" units="mmol_per_gDW_per_hr" constant="true"/>
    <parameter id="FLUX_VALUE" value="0" units="mmol_per_gDW_per_hr" constant="true"/>
    <parameter id="OBJECTIVE_COEFFICIENT" value="0" units="mmol_per_gDW_per_hr" constant="true"/>
   </listOfParameters>
  </kineticLaw>
 </reaction>
 <reaction id="R_R210" name="3-Phospho-D-glycerate:NAD+ 2-oxidoreductase" reversible="false">
  <notes>
   <body xmlns="http://www.w3.org/1999/xhtml">
    <p>GENE_ASSOCIATION: (TTC0586 or TTC1209)</p>
    <p>SUBSYSTEM: Glycine and Serine Metabolism</p>
    <p>EC Number: 1.1.1.95</p>
    <p>Confidence Level: 1</p>
    <p>AUTHORS: Amino acid metabolism</p>
    <p/>
   </body>
  </notes>
  <listOfReactants>
   <speciesReference species="M_NAD_c" stoichiometry="1"/>
```

```xml
    <speciesReference species="M_3PG_c" stoichiometry="1"/>
  </listOfReactants>
  <listOfProducts>
    <speciesReference species="M_H_c" stoichiometry="1"/>
    <speciesReference species="M_NADH_c" stoichiometry="1"/>
    <speciesReference species="M_3POXPYR_c" stoichiometry="1"/>
  </listOfProducts>
  <kineticLaw>
    <math xmlns="http://www.w3.org/1998/Math/MathML">
      <ci> FLUX_VALUE </ci>
    </math>
    <listOfParameters>
      <parameter id="LOWER_BOUND" value="0" units="mmol_per_gDW_per_hr" constant="true"/>
      <parameter id="UPPER_BOUND" value="1000" units="mmol_per_gDW_per_hr" constant="true"/>
      <parameter id="FLUX_VALUE" value="0" units="mmol_per_gDW_per_hr" constant="true"/>
      <parameter id="OBJECTIVE_COEFFICIENT" value="0" units="mmol_per_gDW_per_hr" constant="true"/>
    </listOfParameters>
  </kineticLaw>
</reaction>
<reaction id="R_R211" name="L-serine ammonia-lyase" reversible="false">
  <notes>
    <body xmlns="http://www.w3.org/1999/xhtml">
      <p>GENE_ASSOCIATION: ((TTC0387 or TTC0667) or TTC1708)</p>
      <p>SUBSYSTEM: Glycine and Serine Metabolism</p>
      <p>EC Number: (4.3.1.17 or 4.3.1.19)</p>
      <p>Confidence Level: 1</p>
      <p>AUTHORS: Amino acid metabolism</p>
      <p/>
    </body>
```

```
  </notes>
  <listOfReactants>
    <speciesReference species="M_L_SER_c" stoichiometry="1"/>
  </listOfReactants>
  <listOfProducts>
    <speciesReference species="M_PYR_c" stoichiometry="1"/>
    <speciesReference species="M_NH4_c" stoichiometry="1"/>
  </listOfProducts>
  <kineticLaw>
    <math xmlns="http://www.w3.org/1998/Math/MathML">
      <ci> FLUX_VALUE </ci>
    </math>
    <listOfParameters>
      <parameter id="LOWER_BOUND" value="0" units="mmol_per_gDW_per_hr" constant="true"/>
      <parameter id="UPPER_BOUND" value="1000" units="mmol_per_gDW_per_hr" constant="true"/>
      <parameter id="FLUX_VALUE" value="0" units="mmol_per_gDW_per_hr" constant="true"/>
      <parameter id="OBJECTIVE_COEFFICIENT" value="0" units="mmol_per_gDW_per_hr" constant="true"/>
    </listOfParameters>
  </kineticLaw>
</reaction>
<reaction id="R_R212" name="L-serine hydro-lyase [adding 1-C-(indol-3-yl)glycerol 3-phosphate" reversible="true">
  <notes>
    <body xmlns="http://www.w3.org/1999/xhtml">
      <p>GENE_ASSOCIATION: (TTC0729 and TTC0730)</p>
      <p>SUBSYSTEM: Tyrosine, Tryptophan, and Phenylalanine Metabolism</p>
      <p>EC Number: 4.2.1.20</p>
      <p>Confidence Level: 1</p>
      <p>AUTHORS: Amino acid metabolism</p>
      <p/>
```

```xml
  </body>
 </notes>
 <listOfReactants>
  <speciesReference species="M_L_SER_c" stoichiometry="1"/>
  <speciesReference species="M_IND_GLYP_c" stoichiometry="1"/>
 </listOfReactants>
 <listOfProducts>
  <speciesReference species="M_L_TRP_c" stoichiometry="1"/>
  <speciesReference species="M_H2O_c" stoichiometry="1"/>
  <speciesReference species="M_GAP_c" stoichiometry="1"/>
 </listOfProducts>
 <kineticLaw>
  <math xmlns="http://www.w3.org/1998/Math/MathML">
   <ci> FLUX_VALUE </ci>
  </math>
  <listOfParameters>
   <parameter id="LOWER_BOUND" value="-1000" units="mmol_per_gDW_per_hr" constant="true"/>
   <parameter id="UPPER_BOUND" value="1000" units="mmol_per_gDW_per_hr" constant="true"/>
   <parameter id="FLUX_VALUE" value="0" units="mmol_per_gDW_per_hr" constant="true"/>
   <parameter id="OBJECTIVE_COEFFICIENT" value="0" units="mmol_per_gDW_per_hr" constant="true"/>
  </listOfParameters>
 </kineticLaw>
</reaction>
<reaction id="R_R213" name="5,10-Methylenetetrahydrofolate:glycine hydroxymethyltransferase" reversible="true">
 <notes>
  <body xmlns="http://www.w3.org/1999/xhtml">
   <p>GENE_ASSOCIATION: TTC1160</p>
   <p>SUBSYSTEM: Glycine and Serine Metabolism</p>
   <p>EC Number: 2.1.2.1</p>
```

```
<p>Confidence Level: 1</p>

<p>AUTHORS: Amino acid metabolism</p>

<p/>

</body>

</notes>

<listOfReactants>

<speciesReference species="M_L_GLY_c" stoichiometry="1"/>

<speciesReference species="M_H2O_c" stoichiometry="1"/>

<speciesReference species="M_5_10_MNTHF_c" stoichiometry="1"/>

</listOfReactants>

<listOfProducts>

<speciesReference species="M_L_SER_c" stoichiometry="1"/>

<speciesReference species="M_THF_c" stoichiometry="1"/>

</listOfProducts>

<kineticLaw>

<math xmlns="http://www.w3.org/1998/Math/MathML">

<ci> FLUX_VALUE </ci>

</math>

<listOfParameters>

<parameter id="LOWER_BOUND" value="-1000" units="mmol_per_gDW_per_hr" constant="true"/>

<parameter id="UPPER_BOUND" value="1000" units="mmol_per_gDW_per_hr" constant="true"/>

<parameter id="FLUX_VALUE" value="0" units="mmol_per_gDW_per_hr" constant="true"/>

<parameter id="OBJECTIVE_COEFFICIENT" value="0" units="mmol_per_gDW_per_hr" constant="true"/>

</listOfParameters>

</kineticLaw>

</reaction>

<reaction id="R_R214" name="glycine:lipoylprotein oxidoreductase (decarboxylating and acceptor-aminomethylating)"
reversible="false">

<notes>
```

```
    <body xmlns="http://www.w3.org/1999/xhtml">
      <p>GENE_ASSOCIATION: (TTC0150 or TTC0151)</p>
      <p>SUBSYSTEM: Glycine and Serine Metabolism</p>
      <p>EC Number: 1.4.4.2</p>
      <p>Confidence Level: 1</p>
      <p>AUTHORS: Amino acid metabolism</p>
      <p/>
    </body>
  </notes>
  <listOfReactants>
    <speciesReference species="M_L_GLY_c" stoichiometry="1"/>
    <speciesReference species="M_LPP_c" stoichiometry="1"/>
  </listOfReactants>
  <listOfProducts>
    <speciesReference species="M_CO2_c" stoichiometry="1"/>
    <speciesReference species="M_AMDHY_LPP_c" stoichiometry="1"/>
  </listOfProducts>
  <kineticLaw>
    <math xmlns="http://www.w3.org/1998/Math/MathML">
      <ci> FLUX_VALUE </ci>
    </math>
    <listOfParameters>
      <parameter id="LOWER_BOUND" value="0" units="mmol_per_gDW_per_hr" constant="true"/>
      <parameter id="UPPER_BOUND" value="1000" units="mmol_per_gDW_per_hr" constant="true"/>
      <parameter id="FLUX_VALUE" value="0" units="mmol_per_gDW_per_hr" constant="true"/>
      <parameter id="OBJECTIVE_COEFFICIENT" value="0" units="mmol_per_gDW_per_hr" constant="true"/>
    </listOfParameters>
  </kineticLaw>
</reaction>
```

```xml
<reaction id="R_R215" name="S-aminomethyldihydrolipoylprotein:(6S)-tetrahydrofolate aminomethyltransferase (ammonia-forming)" reversible="false">
<notes>
<body xmlns="http://www.w3.org/1999/xhtml">
<p>GENE_ASSOCIATION: TTC0148</p>
<p>SUBSYSTEM: Glycine and Serine Metabolism</p>
<p>EC Number: 2.1.2.10</p>
<p>Confidence Level: 1</p>
<p>AUTHORS: Amino acid metabolism</p>
<p/>
</body>
</notes>
<listOfReactants>
<speciesReference species="M_THF_c" stoichiometry="1"/>
<speciesReference species="M_AMDHY_LPP_c" stoichiometry="1"/>
</listOfReactants>
<listOfProducts>
<speciesReference species="M_NH4_c" stoichiometry="1"/>
<speciesReference species="M_5_10_MNTHF_c" stoichiometry="1"/>
<speciesReference species="M_DHY_LPP_c" stoichiometry="1"/>
</listOfProducts>
<kineticLaw>
<math xmlns="http://www.w3.org/1998/Math/MathML">
<ci> FLUX_VALUE </ci>
</math>
<listOfParameters>
<parameter id="LOWER_BOUND" value="0" units="mmol_per_gDW_per_hr" constant="true"/>
<parameter id="UPPER_BOUND" value="1000" units="mmol_per_gDW_per_hr" constant="true"/>
<parameter id="FLUX_VALUE" value="0" units="mmol_per_gDW_per_hr" constant="true"/>
```

```
      <parameter id="OBJECTIVE_COEFFICIENT" value="0" units="mmol_per_gDW_per_hr" constant="true"/>
    </listOfParameters>
  </kineticLaw>
</reaction>
<reaction id="R_R216" name="dihydrolipoylprotein:NAD+ oxidoreductase" reversible="false">
  <notes>
    <body xmlns="http://www.w3.org/1999/xhtml">
      <p>GENE_ASSOCIATION: (TTC1700 or TTC1753)</p>
      <p>SUBSYSTEM: Glycine and Serine Metabolism</p>
      <p>EC Number: 1.8.1.4</p>
      <p>Confidence Level: 1</p>
      <p>AUTHORS: Amino acid metabolism</p>
      <p/>
    </body>
  </notes>
  <listOfReactants>
    <speciesReference species="M_NAD_c" stoichiometry="1"/>
    <speciesReference species="M_DHY_LPP_c" stoichiometry="1"/>
  </listOfReactants>
  <listOfProducts>
    <speciesReference species="M_H_c" stoichiometry="1"/>
    <speciesReference species="M_NADH_c" stoichiometry="1"/>
    <speciesReference species="M_LPP_c" stoichiometry="1"/>
  </listOfProducts>
  <kineticLaw>
    <math xmlns="http://www.w3.org/1998/Math/MathML">
      <ci> FLUX_VALUE </ci>
    </math>
    <listOfParameters>
```

```xml
      <parameter id="LOWER_BOUND" value="0" units="mmol_per_gDW_per_hr" constant="true"/>
      <parameter id="UPPER_BOUND" value="1000" units="mmol_per_gDW_per_hr" constant="true"/>
      <parameter id="FLUX_VALUE" value="0" units="mmol_per_gDW_per_hr" constant="true"/>
      <parameter id="OBJECTIVE_COEFFICIENT" value="0" units="mmol_per_gDW_per_hr" constant="true"/>
    </listOfParameters>
  </kineticLaw>
</reaction>
<reaction id="R_R217" name="L-threonine acetaldehyde-lyase (glycine-forming)" reversible="false">
  <notes>
    <body xmlns="http://www.w3.org/1999/xhtml">
      <p>GENE_ASSOCIATION: TTC0397</p>
      <p>SUBSYSTEM: Threonine and Lysine Metabolism</p>
      <p>EC Number: 4.1.2.5</p>
      <p>Confidence Level: 1</p>
      <p>AUTHORS: Amino acid metabolism</p>
      <p/>
    </body>
  </notes>
  <listOfReactants>
    <speciesReference species="M_L_THR_c" stoichiometry="1"/>
  </listOfReactants>
  <listOfProducts>
    <speciesReference species="M_L_GLY_c" stoichiometry="1"/>
    <speciesReference species="M_AALD_c" stoichiometry="1"/>
  </listOfProducts>
  <kineticLaw>
    <math xmlns="http://www.w3.org/1998/Math/MathML">
      <ci> FLUX_VALUE </ci>
    </math>
```

```
<listOfParameters>
  <parameter id="LOWER_BOUND" value="0" units="mmol_per_gDW_per_hr" constant="true"/>
  <parameter id="UPPER_BOUND" value="1000" units="mmol_per_gDW_per_hr" constant="true"/>
  <parameter id="FLUX_VALUE" value="0" units="mmol_per_gDW_per_hr" constant="true"/>
  <parameter id="OBJECTIVE_COEFFICIENT" value="0" units="mmol_per_gDW_per_hr" constant="true"/>
</listOfParameters>
</kineticLaw>
</reaction>
<reaction id="R_R218" name="Acetyl-CoA:glycine C-acetyltransferase" reversible="true">
<notes>
<body xmlns="http://www.w3.org/1999/xhtml">
  <p>GENE_ASSOCIATION: TTC1219</p>
  <p>SUBSYSTEM: Glycine and Serine Metabolism</p>
  <p>EC Number: 2.3.1.29</p>
  <p>Confidence Level: 1</p>
  <p>AUTHORS: Amino acid metabolism</p>
  <p/>
</body>
</notes>
<listOfReactants>
  <speciesReference species="M_CoA_c" stoichiometry="1"/>
  <speciesReference species="M_A_OXBUT_c" stoichiometry="1"/>
</listOfReactants>
<listOfProducts>
  <speciesReference species="M_L_GLY_c" stoichiometry="1"/>
  <speciesReference species="M_ACoA_c" stoichiometry="1"/>
</listOfProducts>
<kineticLaw>
  <math xmlns="http://www.w3.org/1998/Math/MathML">
```

```
      <ci> FLUX_VALUE </ci>
    </math>
    <listOfParameters>
      <parameter id="LOWER_BOUND" value="-1000" units="mmol_per_gDW_per_hr" constant="true"/>
      <parameter id="UPPER_BOUND" value="1000" units="mmol_per_gDW_per_hr" constant="true"/>
      <parameter id="FLUX_VALUE" value="0" units="mmol_per_gDW_per_hr" constant="true"/>
      <parameter id="OBJECTIVE_COEFFICIENT" value="0" units="mmol_per_gDW_per_hr" constant="true"/>
    </listOfParameters>
  </kineticLaw>
</reaction>
<reaction id="R_R219" name="L-threonine:NAD+ oxidoreductase" reversible="false">
  <notes>
    <body xmlns="http://www.w3.org/1999/xhtml">
      <p>GENE_ASSOCIATION: TTC0201</p>
      <p>SUBSYSTEM: Glycine and Serine Metabolism</p>
      <p>EC Number: 1.1.1.103</p>
      <p>Confidence Level: 1</p>
      <p>AUTHORS: Amino acid metabolism</p>
      <p/>
    </body>
  </notes>
  <listOfReactants>
    <speciesReference species="M_L_THR_c" stoichiometry="1"/>
    <speciesReference species="M_NAD_c" stoichiometry="1"/>
  </listOfReactants>
  <listOfProducts>
    <speciesReference species="M_H_c" stoichiometry="1"/>
    <speciesReference species="M_NADH_c" stoichiometry="1"/>
    <speciesReference species="M_A_OXBUT_c" stoichiometry="1"/>
```

```
  </listOfProducts>
  <kineticLaw>
    <math xmlns="http://www.w3.org/1998/Math/MathML">
      <ci> FLUX_VALUE </ci>
    </math>
    <listOfParameters>
      <parameter id="LOWER_BOUND" value="0" units="mmol_per_gDW_per_hr" constant="true"/>
      <parameter id="UPPER_BOUND" value="1000" units="mmol_per_gDW_per_hr" constant="true"/>
      <parameter id="FLUX_VALUE" value="0" units="mmol_per_gDW_per_hr" constant="true"/>
      <parameter id="OBJECTIVE_COEFFICIENT" value="0" units="mmol_per_gDW_per_hr" constant="true"/>
    </listOfParameters>
  </kineticLaw>
</reaction>
<reaction id="R_R220" name="L-threonine ammonia-lyase (2-oxobutanoate-forming)" reversible="false">
  <notes>
    <body xmlns="http://www.w3.org/1999/xhtml">
      <p>GENE_ASSOCIATION: TTC1708</p>
      <p>SUBSYSTEM: Threonine and Lysine Metabolism</p>
      <p>EC Number: 4.3.1.19</p>
      <p>Confidence Level: 1</p>
      <p>AUTHORS: Amino acid metabolism</p>
      <p/>
    </body>
  </notes>
  <listOfReactants>
    <speciesReference species="M_L_THR_c" stoichiometry="1"/>
  </listOfReactants>
  <listOfProducts>
    <speciesReference species="M_NH4_c" stoichiometry="1"/>
```

```
    <speciesReference species="M_AKB_c" stoichiometry="1"/>
   </listOfProducts>
   <kineticLaw>
    <math xmlns="http://www.w3.org/1998/Math/MathML">
     <ci> FLUX_VALUE </ci>
    </math>
    <listOfParameters>
     <parameter id="LOWER_BOUND" value="0" units="mmol_per_gDW_per_hr" constant="true"/>
     <parameter id="UPPER_BOUND" value="1000" units="mmol_per_gDW_per_hr" constant="true"/>
     <parameter id="FLUX_VALUE" value="0" units="mmol_per_gDW_per_hr" constant="true"/>
     <parameter id="OBJECTIVE_COEFFICIENT" value="0" units="mmol_per_gDW_per_hr" constant="true"/>
    </listOfParameters>
   </kineticLaw>
  </reaction>
  <reaction id="R_R221" name="O-phospho-L-homoserine phosphate-lyase (adding water;L-threonine-forming)"
reversible="false">
   <notes>
    <body xmlns="http://www.w3.org/1999/xhtml">
     <p>GENE_ASSOCIATION: TTC0117</p>
     <p>SUBSYSTEM: Threonine and Lysine Metabolism</p>
     <p>EC Number: 4.2.3.1</p>
     <p>Confidence Level: 1</p>
     <p>AUTHORS: Amino acid metabolism</p>
     <p/>
    </body>
   </notes>
   <listOfReactants>
    <speciesReference species="M_H2O_c" stoichiometry="1"/>
    <speciesReference species="M_OP_HSER_c" stoichiometry="1"/>
```

```
  </listOfReactants>
  <listOfProducts>
    <speciesReference species="M_L_THR_c" stoichiometry="1"/>
    <speciesReference species="M_Pi_c" stoichiometry="1"/>
  </listOfProducts>
  <kineticLaw>
    <math xmlns="http://www.w3.org/1998/Math/MathML">
      <ci> FLUX_VALUE </ci>
    </math>
    <listOfParameters>
      <parameter id="LOWER_BOUND" value="0" units="mmol_per_gDW_per_hr" constant="true"/>
      <parameter id="UPPER_BOUND" value="1000" units="mmol_per_gDW_per_hr" constant="true"/>
      <parameter id="FLUX_VALUE" value="0" units="mmol_per_gDW_per_hr" constant="true"/>
      <parameter id="OBJECTIVE_COEFFICIENT" value="0" units="mmol_per_gDW_per_hr" constant="true"/>
    </listOfParameters>
  </kineticLaw>
</reaction>
<reaction id="R_R222" name="ATP:L-homoserine O-phosphotransferase" reversible="false">
  <notes>
    <body xmlns="http://www.w3.org/1999/xhtml">
      <p>GENE_ASSOCIATION: TTC1028</p>
      <p>SUBSYSTEM: Threonine and Lysine Metabolism</p>
      <p>EC Number: 2.7.1.39</p>
      <p>Confidence Level: 1</p>
      <p>AUTHORS: Amino acid metabolism</p>
      <p/>
    </body>
  </notes>
  <listOfReactants>
```

```
      <speciesReference species="M_ATP_c" stoichiometry="1"/>
      <speciesReference species="M_L_HSER_c" stoichiometry="1"/>
    </listOfReactants>
    <listOfProducts>
      <speciesReference species="M_ADP_c" stoichiometry="1"/>
      <speciesReference species="M_H_c" stoichiometry="1"/>
      <speciesReference species="M_OP_HSER_c" stoichiometry="1"/>
    </listOfProducts>
    <kineticLaw>
      <math xmlns="http://www.w3.org/1998/Math/MathML">
        <ci> FLUX_VALUE </ci>
      </math>
      <listOfParameters>
        <parameter id="LOWER_BOUND" value="0" units="mmol_per_gDW_per_hr" constant="true"/>
        <parameter id="UPPER_BOUND" value="1000" units="mmol_per_gDW_per_hr" constant="true"/>
        <parameter id="FLUX_VALUE" value="0" units="mmol_per_gDW_per_hr" constant="true"/>
        <parameter id="OBJECTIVE_COEFFICIENT" value="0" units="mmol_per_gDW_per_hr" constant="true"/>
      </listOfParameters>
    </kineticLaw>
  </reaction>
  <reaction id="R_R223" name="L-Homoserine:NAD+ oxidoreductase" reversible="false">
    <notes>
      <body xmlns="http://www.w3.org/1999/xhtml">
        <p>GENE_ASSOCIATION: TTC0115</p>
        <p>SUBSYSTEM: Threonine and Lysine Metabolism</p>
        <p>EC Number: 1.1.1.3</p>
        <p>Confidence Level: 1</p>
        <p>AUTHORS: Amino acid metabolism</p>
        <p/>
```

```
  </body>
 </notes>
 <listOfReactants>
  <speciesReference species="M_H_c" stoichiometry="1"/>
  <speciesReference species="M_NADH_c" stoichiometry="1"/>
  <speciesReference species="M_ASP_4SA_c" stoichiometry="1"/>
 </listOfReactants>
 <listOfProducts>
  <speciesReference species="M_NAD_c" stoichiometry="1"/>
  <speciesReference species="M_L_HSER_c" stoichiometry="1"/>
 </listOfProducts>
 <kineticLaw>
  <math xmlns="http://www.w3.org/1998/Math/MathML">
   <ci> FLUX_VALUE </ci>
  </math>
  <listOfParameters>
   <parameter id="LOWER_BOUND" value="0" units="mmol_per_gDW_per_hr" constant="true"/>
   <parameter id="UPPER_BOUND" value="1000" units="mmol_per_gDW_per_hr" constant="true"/>
   <parameter id="FLUX_VALUE" value="0" units="mmol_per_gDW_per_hr" constant="true"/>
   <parameter id="OBJECTIVE_COEFFICIENT" value="0" units="mmol_per_gDW_per_hr" constant="true"/>
  </listOfParameters>
 </kineticLaw>
</reaction>
<reaction id="R_R224" name="L-homoserine:NADP+ oxidoreductase" reversible="false">
 <notes>
  <body xmlns="http://www.w3.org/1999/xhtml">
   <p>GENE_ASSOCIATION: TTC0115</p>
   <p>SUBSYSTEM: Threonine and Lysine Metabolism</p>
   <p>EC Number: 1.1.1.3</p>
```

```
      <p>Confidence Level: 1</p>
      <p>AUTHORS: Amino acid metabolism</p>
      <p/>
    </body>
  </notes>
  <listOfReactants>
    <speciesReference species="M_H_c" stoichiometry="1"/>
    <speciesReference species="M_NADPH_c" stoichiometry="1"/>
    <speciesReference species="M_ASP_4SA_c" stoichiometry="1"/>
  </listOfReactants>
  <listOfProducts>
    <speciesReference species="M_NADP_c" stoichiometry="1"/>
    <speciesReference species="M_L_HSER_c" stoichiometry="1"/>
  </listOfProducts>
  <kineticLaw>
    <math xmlns="http://www.w3.org/1998/Math/MathML">
      <ci> FLUX_VALUE </ci>
    </math>
    <listOfParameters>
      <parameter id="LOWER_BOUND" value="0" units="mmol_per_gDW_per_hr" constant="true"/>
      <parameter id="UPPER_BOUND" value="1000" units="mmol_per_gDW_per_hr" constant="true"/>
      <parameter id="FLUX_VALUE" value="0" units="mmol_per_gDW_per_hr" constant="true"/>
      <parameter id="OBJECTIVE_COEFFICIENT" value="0" units="mmol_per_gDW_per_hr" constant="true"/>
    </listOfParameters>
  </kineticLaw>
</reaction>
<reaction id="R_R225" name="L-Aspartate-4-semialdehyde:NADP+ oxidoreductase (phosphorylating)" reversible="true">
  <notes>
    <body xmlns="http://www.w3.org/1999/xhtml">
```

```
    <p>GENE_ASSOCIATION: TTC0177</p>
    <p>SUBSYSTEM: Threonine and Lysine Metabolism</p>
    <p>EC Number: 1.2.1.11</p>
    <p>Confidence Level: 1</p>
    <p>AUTHORS: Amino acid metabolism</p>
    <p/>
  </body>
</notes>
<listOfReactants>
  <speciesReference species="M_H_c" stoichiometry="1"/>
  <speciesReference species="M_NADPH_c" stoichiometry="1"/>
  <speciesReference species="M_4P_ASP_c" stoichiometry="1"/>
</listOfReactants>
<listOfProducts>
  <speciesReference species="M_Pi_c" stoichiometry="1"/>
  <speciesReference species="M_NADP_c" stoichiometry="1"/>
  <speciesReference species="M_ASP_4SA_c" stoichiometry="1"/>
</listOfProducts>
<kineticLaw>
  <math xmlns="http://www.w3.org/1998/Math/MathML">
    <ci> FLUX_VALUE </ci>
  </math>
  <listOfParameters>
    <parameter id="LOWER_BOUND" value="-1000" units="mmol_per_gDW_per_hr" constant="true"/>
    <parameter id="UPPER_BOUND" value="1000" units="mmol_per_gDW_per_hr" constant="true"/>
    <parameter id="FLUX_VALUE" value="0" units="mmol_per_gDW_per_hr" constant="true"/>
    <parameter id="OBJECTIVE_COEFFICIENT" value="0" units="mmol_per_gDW_per_hr" constant="true"/>
  </listOfParameters>
</kineticLaw>
```

```xml
</reaction>
<reaction id="R_R226" name="ATP:L-aspartate 4-phosphotransferase" reversible="true">
 <notes>
  <body xmlns="http://www.w3.org/1999/xhtml">
   <p>GENE_ASSOCIATION: TTC0166</p>
   <p>SUBSYSTEM: Threonine and Lysine Metabolism</p>
   <p>EC Number: 2.7.2.4</p>
   <p>Confidence Level: 1</p>
   <p>AUTHORS: Amino acid metabolism</p>
   <p/>
  </body>
 </notes>
 <listOfReactants>
  <speciesReference species="M_L_ASP_c" stoichiometry="1"/>
  <speciesReference species="M_ATP_c" stoichiometry="1"/>
 </listOfReactants>
 <listOfProducts>
  <speciesReference species="M_ADP_c" stoichiometry="1"/>
  <speciesReference species="M_4P_ASP_c" stoichiometry="1"/>
 </listOfProducts>
 <kineticLaw>
  <math xmlns="http://www.w3.org/1998/Math/MathML">
   <ci> FLUX_VALUE </ci>
  </math>
  <listOfParameters>
   <parameter id="LOWER_BOUND" value="-1000" units="mmol_per_gDW_per_hr" constant="true"/>
   <parameter id="UPPER_BOUND" value="1000" units="mmol_per_gDW_per_hr" constant="true"/>
   <parameter id="FLUX_VALUE" value="0" units="mmol_per_gDW_per_hr" constant="true"/>
   <parameter id="OBJECTIVE_COEFFICIENT" value="0" units="mmol_per_gDW_per_hr" constant="true"/>
```

```
      </listOfParameters>
    </kineticLaw>
  </reaction>
  <reaction id="R_R227" name="O3-acetyl-L-serine:hydrogen-sulfide 2-amino-2-carboxyethyltransferase" reversible="false">
    <notes>
      <body xmlns="http://www.w3.org/1999/xhtml">
        <p>GENE_ASSOCIATION: TTC1636</p>
        <p>SUBSYSTEM: Cysteine and methionine metabolism</p>
        <p>EC Number: 2.5.1.47</p>
        <p>Confidence Level: 1</p>
        <p>AUTHORS: Amino acid metabolism</p>
        <p/>
      </body>
    </notes>
    <listOfReactants>
      <speciesReference species="M_OAC_SER_c" stoichiometry="1"/>
      <speciesReference species="M_H2S_c" stoichiometry="1"/>
    </listOfReactants>
    <listOfProducts>
      <speciesReference species="M_L_CYS_c" stoichiometry="1"/>
      <speciesReference species="M_H_c" stoichiometry="1"/>
      <speciesReference species="M_AC_c" stoichiometry="1"/>
    </listOfProducts>
    <kineticLaw>
      <math xmlns="http://www.w3.org/1998/Math/MathML">
        <ci> FLUX_VALUE </ci>
      </math>
      <listOfParameters>
        <parameter id="LOWER_BOUND" value="0" units="mmol_per_gDW_per_hr" constant="true"/>
```

```
<parameter id="UPPER_BOUND" value="1000" units="mmol_per_gDW_per_hr" constant="true"/>

<parameter id="FLUX_VALUE" value="0" units="mmol_per_gDW_per_hr" constant="true"/>

<parameter id="OBJECTIVE_COEFFICIENT" value="0" units="mmol_per_gDW_per_hr" constant="true"/>

</listOfParameters>

</kineticLaw>

</reaction>

<reaction id="R_R228" name="O3-acetyl-L-serine:thiosulfate 2-amino-2-carboxyethyltransferase (reducing, L-cysteine-forming)" reversible="false">

<notes>

<body xmlns="http://www.w3.org/1999/xhtml">

<p>GENE_ASSOCIATION: TTC1636</p>

<p>SUBSYSTEM: Cysteine and methionine metabolism</p>

<p>EC Number: 2.5.1.47</p>

<p>Confidence Level: 1</p>

<p>AUTHORS: Amino acid metabolism</p>

<p/>

</body>

</notes>

<listOfReactants>

<speciesReference species="M_H_c" stoichiometry="1"/>

<speciesReference species="M_R_TRED_c" stoichiometry="1"/>

<speciesReference species="M_OAC_SER_c" stoichiometry="1"/>

<speciesReference species="M_TSFA_c" stoichiometry="1"/>

</listOfReactants>

<listOfProducts>

<speciesReference species="M_L_CYS_c" stoichiometry="1"/>

<speciesReference species="M_AC_c" stoichiometry="1"/>

<speciesReference species="M_O_TRED_c" stoichiometry="1"/>

<speciesReference species="M_SO3_c" stoichiometry="1"/>
```

```xml
</listOfProducts>
<kineticLaw>
  <math xmlns="http://www.w3.org/1998/Math/MathML">
    <ci> FLUX_VALUE </ci>
  </math>
  <listOfParameters>
    <parameter id="LOWER_BOUND" value="0" units="mmol_per_gDW_per_hr" constant="true"/>
    <parameter id="UPPER_BOUND" value="1000" units="mmol_per_gDW_per_hr" constant="true"/>
    <parameter id="FLUX_VALUE" value="0" units="mmol_per_gDW_per_hr" constant="true"/>
    <parameter id="OBJECTIVE_COEFFICIENT" value="0" units="mmol_per_gDW_per_hr" constant="true"/>
  </listOfParameters>
</kineticLaw>
</reaction>
<reaction id="R_R229" name="L-cysteate:2-oxoglutarate aminotransferase" reversible="true">
<notes>
  <body xmlns="http://www.w3.org/1999/xhtml">
    <p>GENE_ASSOCIATION: TTC1960</p>
    <p>SUBSYSTEM: Cysteine and methionine metabolism</p>
    <p>EC Number: 2.6.1.1</p>
    <p>Confidence Level: 1</p>
    <p>AUTHORS: Amino acid metabolism</p>
    <p/>
  </body>
</notes>
<listOfReactants>
  <speciesReference species="M_AKG_c" stoichiometry="1"/>
  <speciesReference species="M_L_CYSTA_c" stoichiometry="1"/>
</listOfReactants>
<listOfProducts>
```

```
    <speciesReference species="M_L_GLU_c" stoichiometry="1"/>
    <speciesReference species="M_SULFO_PYR_c" stoichiometry="1"/>
  </listOfProducts>
  <kineticLaw>
    <math xmlns="http://www.w3.org/1998/Math/MathML">
      <ci> FLUX_VALUE </ci>
    </math>
    <listOfParameters>
      <parameter id="LOWER_BOUND" value="-1000" units="mmol_per_gDW_per_hr" constant="true"/>
      <parameter id="UPPER_BOUND" value="1000" units="mmol_per_gDW_per_hr" constant="true"/>
      <parameter id="FLUX_VALUE" value="0" units="mmol_per_gDW_per_hr" constant="true"/>
      <parameter id="OBJECTIVE_COEFFICIENT" value="0" units="mmol_per_gDW_per_hr" constant="true"/>
    </listOfParameters>
  </kineticLaw>
</reaction>
<reaction id="R_R230" name="3-sulfino-L-alanine:2-oxoglutarate aminotransferase" reversible="true">
  <notes>
    <body xmlns="http://www.w3.org/1999/xhtml">
      <p>GENE_ASSOCIATION: TTC1960</p>
      <p>SUBSYSTEM: Cysteine and methionine metabolism</p>
      <p>EC Number: 2.6.1.1</p>
      <p>Confidence Level: 1</p>
      <p>AUTHORS: Amino acid metabolism</p>
      <p/>
    </body>
  </notes>
  <listOfReactants>
    <speciesReference species="M_AKG_c" stoichiometry="1"/>
    <speciesReference species="M_SLF_ALA_c" stoichiometry="1"/>
```

```
  </listOfReactants>
  <listOfProducts>
   <speciesReference species="M_L_GLU_c" stoichiometry="1"/>
   <speciesReference species="M_SULFI_PYR_c" stoichiometry="1"/>
  </listOfProducts>
  <kineticLaw>
   <math xmlns="http://www.w3.org/1998/Math/MathML">
    <ci> FLUX_VALUE </ci>
   </math>
   <listOfParameters>
    <parameter id="LOWER_BOUND" value="-1000" units="mmol_per_gDW_per_hr" constant="true"/>
    <parameter id="UPPER_BOUND" value="1000" units="mmol_per_gDW_per_hr" constant="true"/>
    <parameter id="FLUX_VALUE" value="0" units="mmol_per_gDW_per_hr" constant="true"/>
    <parameter id="OBJECTIVE_COEFFICIENT" value="0" units="mmol_per_gDW_per_hr" constant="true"/>
   </listOfParameters>
  </kineticLaw>
 </reaction>
 <reaction id="R_R231" name="L-Cysteine hydrogen-sulfide-lyase (deaminating" reversible="false">
  <notes>
   <body xmlns="http://www.w3.org/1999/xhtml">
    <p>GENE_ASSOCIATION: TTC1256</p>
    <p>SUBSYSTEM: Cysteine and methionine metabolism</p>
    <p>EC Number: 4.4.1.8</p>
    <p>Confidence Level: 1</p>
    <p>AUTHORS: Amino acid metabolism</p>
    <p/>
   </body>
  </notes>
  <listOfReactants>
```

```
    <speciesReference species="M_L_CYS_c" stoichiometry="1"/>
    <speciesReference species="M_H2O_c" stoichiometry="1"/>
  </listOfReactants>
  <listOfProducts>
    <speciesReference species="M_PYR_c" stoichiometry="1"/>
    <speciesReference species="M_NH4_c" stoichiometry="1"/>
    <speciesReference species="M_H2S_c" stoichiometry="1"/>
  </listOfProducts>
  <kineticLaw>
    <math xmlns="http://www.w3.org/1998/Math/MathML">
      <ci> FLUX_VALUE </ci>
    </math>
    <listOfParameters>
      <parameter id="LOWER_BOUND" value="0" units="mmol_per_gDW_per_hr" constant="true"/>
      <parameter id="UPPER_BOUND" value="1000" units="mmol_per_gDW_per_hr" constant="true"/>
      <parameter id="FLUX_VALUE" value="0" units="mmol_per_gDW_per_hr" constant="true"/>
      <parameter id="OBJECTIVE_COEFFICIENT" value="0" units="mmol_per_gDW_per_hr" constant="true"/>
    </listOfParameters>
  </kineticLaw>
</reaction>
<reaction id="R_R232" name="Acetyl-CoA:L-homoserine O-acetyltransferase" reversible="false">
  <notes>
    <body xmlns="http://www.w3.org/1999/xhtml">
      <p>GENE_ASSOCIATION: TTC0407</p>
      <p>SUBSYSTEM: Cysteine and methionine metabolism</p>
      <p>EC Number: 2.3.1.31</p>
      <p>Confidence Level: 1</p>
      <p>AUTHORS: Amino acid metabolism</p>
      <p/>
```

</body>

</notes>

<listOfReactants>

<speciesReference species="M_ACoA_c" stoichiometry="1"/>

<speciesReference species="M_L_HSER_c" stoichiometry="1"/>

</listOfReactants>

<listOfProducts>

<speciesReference species="M_CoA_c" stoichiometry="1"/>

<speciesReference species="M_OAC_HSER_c" stoichiometry="1"/>

</listOfProducts>

<kineticLaw>

<math xmlns="http://www.w3.org/1998/Math/MathML">

<ci> FLUX_VALUE </ci>

</math>

<listOfParameters>

<parameter id="LOWER_BOUND" value="0" units="mmol_per_gDW_per_hr" constant="true"/>

<parameter id="UPPER_BOUND" value="1000" units="mmol_per_gDW_per_hr" constant="true"/>

<parameter id="FLUX_VALUE" value="0" units="mmol_per_gDW_per_hr" constant="true"/>

<parameter id="OBJECTIVE_COEFFICIENT" value="0" units="mmol_per_gDW_per_hr" constant="true"/>

</listOfParameters>

</kineticLaw>

</reaction>

<reaction id="R_R233" name="O-acetyl-L-homoserine:hydrogen sulfide S-(3-amino-3-carboxypropyl)transferase" reversible="false">

<notes>

<body xmlns="http://www.w3.org/1999/xhtml">

<p>GENE_ASSOCIATION: (TTC0408 or TTC0792)</p>

<p>SUBSYSTEM: Cysteine and methionine metabolism</p>

<p>EC Number: 2.5.1.49</p>

```
      <p>Confidence Level: 1</p>
      <p>AUTHORS: Amino acid metabolism</p>
      <p/>
    </body>
  </notes>
  <listOfReactants>
    <speciesReference species="M_H2S_c" stoichiometry="1"/>
    <speciesReference species="M_OAC_HSER_c" stoichiometry="1"/>
  </listOfReactants>
  <listOfProducts>
    <speciesReference species="M_H_c" stoichiometry="1"/>
    <speciesReference species="M_AC_c" stoichiometry="1"/>
    <speciesReference species="M_L_HCYS_c" stoichiometry="1"/>
  </listOfProducts>
  <kineticLaw>
    <math xmlns="http://www.w3.org/1998/Math/MathML">
      <ci> FLUX_VALUE </ci>
    </math>
    <listOfParameters>
      <parameter id="LOWER_BOUND" value="0" units="mmol_per_gDW_per_hr" constant="true"/>
      <parameter id="UPPER_BOUND" value="1000" units="mmol_per_gDW_per_hr" constant="true"/>
      <parameter id="FLUX_VALUE" value="0" units="mmol_per_gDW_per_hr" constant="true"/>
      <parameter id="OBJECTIVE_COEFFICIENT" value="0" units="mmol_per_gDW_per_hr" constant="true"/>
    </listOfParameters>
  </kineticLaw>
</reaction>
<reaction id="R_R234" name="L-cystathionine L-homocysteine-lyase (deaminating" reversible="false">
  <notes>
    <body xmlns="http://www.w3.org/1999/xhtml">
```

```
      <p>GENE_ASSOCIATION: TTC1256</p>
      <p>SUBSYSTEM: Cysteine and methionine metabolism</p>
      <p>EC Number: 4.4.1.8</p>
      <p>Confidence Level: 1</p>
      <p>AUTHORS: Amino acid metabolism</p>
      <p/>
    </body>
  </notes>
  <listOfReactants>
    <speciesReference species="M_H2O_c" stoichiometry="1"/>
    <speciesReference species="M_L_CYSTHNN_c" stoichiometry="1"/>
  </listOfReactants>
  <listOfProducts>
    <speciesReference species="M_PYR_c" stoichiometry="1"/>
    <speciesReference species="M_NH4_c" stoichiometry="1"/>
    <speciesReference species="M_L_HCYS_c" stoichiometry="1"/>
  </listOfProducts>
  <kineticLaw>
    <math xmlns="http://www.w3.org/1998/Math/MathML">
      <ci> FLUX_VALUE </ci>
    </math>
    <listOfParameters>
      <parameter id="LOWER_BOUND" value="0" units="mmol_per_gDW_per_hr" constant="true"/>
      <parameter id="UPPER_BOUND" value="1000" units="mmol_per_gDW_per_hr" constant="true"/>
      <parameter id="FLUX_VALUE" value="0" units="mmol_per_gDW_per_hr" constant="true"/>
      <parameter id="OBJECTIVE_COEFFICIENT" value="0" units="mmol_per_gDW_per_hr" constant="true"/>
    </listOfParameters>
  </kineticLaw>
</reaction>
```

```
<reaction id="R_R235" name="5-Methyltetrahydrofolate:L-homocysteine S-methyltransferase" reversible="false">
 <notes>
  <body xmlns="http://www.w3.org/1999/xhtml">
   <p>GENE_ASSOCIATION: TTC0253</p>
   <p>SUBSYSTEM: Cysteine and methionine metabolism</p>
   <p>EC Number: 2.1.1.13</p>
   <p>Confidence Level: 1</p>
   <p>AUTHORS: Amino acid metabolism</p>
   <p/>
  </body>
 </notes>
 <listOfReactants>
  <speciesReference species="M_L_HCYS_c" stoichiometry="1"/>
  <speciesReference species="M_5MTHF_c" stoichiometry="1"/>
 </listOfReactants>
 <listOfProducts>
  <speciesReference species="M_L_MET_c" stoichiometry="1"/>
  <speciesReference species="M_H_c" stoichiometry="1"/>
  <speciesReference species="M_THF_c" stoichiometry="1"/>
 </listOfProducts>
 <kineticLaw>
  <math xmlns="http://www.w3.org/1998/Math/MathML">
   <ci> FLUX_VALUE </ci>
  </math>
  <listOfParameters>
   <parameter id="LOWER_BOUND" value="0" units="mmol_per_gDW_per_hr" constant="true"/>
   <parameter id="UPPER_BOUND" value="1000" units="mmol_per_gDW_per_hr" constant="true"/>
   <parameter id="FLUX_VALUE" value="0" units="mmol_per_gDW_per_hr" constant="true"/>
   <parameter id="OBJECTIVE_COEFFICIENT" value="0" units="mmol_per_gDW_per_hr" constant="true"/>
```

```xml
    </listOfParameters>
  </kineticLaw>
</reaction>
<reaction id="R_R236" name="S-Adenosyl-L-homocysteine homocysteinylribohydrolase" reversible="false">
  <notes>
    <body xmlns="http://www.w3.org/1999/xhtml">
      <p>GENE_ASSOCIATION: TTC1185</p>
      <p>SUBSYSTEM: Cysteine and methionine metabolism</p>
      <p>EC Number: 3.2.2.9</p>
      <p>Confidence Level: 1</p>
      <p>AUTHORS: Amino acid metabolism</p>
      <p/>
    </body>
  </notes>
  <listOfReactants>
    <speciesReference species="M_H2O_c" stoichiometry="1"/>
    <speciesReference species="M_SAHC_c" stoichiometry="1"/>
  </listOfReactants>
  <listOfProducts>
    <speciesReference species="M_ADN_c" stoichiometry="1"/>
    <speciesReference species="M_RIBS_HCYS_c" stoichiometry="1"/>
  </listOfProducts>
  <kineticLaw>
    <math xmlns="http://www.w3.org/1998/Math/MathML">
      <ci> FLUX_VALUE </ci>
    </math>
    <listOfParameters>
      <parameter id="LOWER_BOUND" value="0" units="mmol_per_gDW_per_hr" constant="true"/>
      <parameter id="UPPER_BOUND" value="1000" units="mmol_per_gDW_per_hr" constant="true"/>
```

```
<parameter id="FLUX_VALUE" value="0" units="mmol_per_gDW_per_hr" constant="true"/>
<parameter id="OBJECTIVE_COEFFICIENT" value="0" units="mmol_per_gDW_per_hr" constant="true"/>
</listOfParameters>
</kineticLaw>
</reaction>
<reaction id="R_R237" name="S-(5-deoxy-D-ribos-5-yl)-L-homocysteine homocysteine-lyase [(4S)-4,5-dihydroxypentan-2,3-dione-forming]" reversible="false">
<notes>
<body xmlns="http://www.w3.org/1999/xhtml">
<p>GENE_ASSOCIATION: TTC1186</p>
<p>SUBSYSTEM: Cysteine and methionine metabolism</p>
<p>EC Number: 4.4.1.21</p>
<p>Confidence Level: 1</p>
<p>AUTHORS: Amino acid metabolism</p>
<p/>
</body>
</notes>
<listOfReactants>
<speciesReference species="M_RIBS_HCYS_c" stoichiometry="1"/>
</listOfReactants>
<listOfProducts>
<speciesReference species="M_L_HCYS_c" stoichiometry="1"/>
<speciesReference species="M_DHY_PENTDON_c" stoichiometry="1"/>
</listOfProducts>
<kineticLaw>
<math xmlns="http://www.w3.org/1998/Math/MathML">
<ci> FLUX_VALUE </ci>
</math>
<listOfParameters>
```

```
      <parameter id="LOWER_BOUND" value="0" units="mmol_per_gDW_per_hr" constant="true"/>
      <parameter id="UPPER_BOUND" value="1000" units="mmol_per_gDW_per_hr" constant="true"/>
      <parameter id="FLUX_VALUE" value="0" units="mmol_per_gDW_per_hr" constant="true"/>
      <parameter id="OBJECTIVE_COEFFICIENT" value="0" units="mmol_per_gDW_per_hr" constant="true"/>
    </listOfParameters>
  </kineticLaw>
</reaction>
<reaction id="R_R238" name="ATP:L-methionine S-adenosyltransferase" reversible="false">
  <notes>
    <body xmlns="http://www.w3.org/1999/xhtml">
      <p>GENE_ASSOCIATION: TTC1279</p>
      <p>SUBSYSTEM: Cysteine and methionine metabolism</p>
      <p>EC Number: 2.5.1.6</p>
      <p>Confidence Level: 1</p>
      <p>AUTHORS: Amino acid metabolism</p>
      <p/>
    </body>
  </notes>
  <listOfReactants>
    <speciesReference species="M_L_MET_c" stoichiometry="1"/>
    <speciesReference species="M_ATP_c" stoichiometry="1"/>
    <speciesReference species="M_H2O_c" stoichiometry="1"/>
  </listOfReactants>
  <listOfProducts>
    <speciesReference species="M_Pi_c" stoichiometry="1"/>
    <speciesReference species="M_PPi_c" stoichiometry="1"/>
    <speciesReference species="M_SAM_c" stoichiometry="1"/>
  </listOfProducts>
  <kineticLaw>
```

```xml
<math xmlns="http://www.w3.org/1998/Math/MathML">

<ci> FLUX_VALUE </ci>

</math>

<listOfParameters>

<parameter id="LOWER_BOUND" value="0" units="mmol_per_gDW_per_hr" constant="true"/>

<parameter id="UPPER_BOUND" value="1000" units="mmol_per_gDW_per_hr" constant="true"/>

<parameter id="FLUX_VALUE" value="0" units="mmol_per_gDW_per_hr" constant="true"/>

<parameter id="OBJECTIVE_COEFFICIENT" value="0" units="mmol_per_gDW_per_hr" constant="true"/>

</listOfParameters>

</kineticLaw>

</reaction>

<reaction id="R_R239" name="S-adenosyl-L-methionine carboxy-lyase [(5-deoxy-5-adenosyl)(3-aminopropyl)methylsulfonium-forming]" reversible="false">

<notes>

<body xmlns="http://www.w3.org/1999/xhtml">

<p>GENE_ASSOCIATION: (TTC0473 or TTC1093)</p>

<p>SUBSYSTEM: Cysteine and methionine metabolism</p>

<p>EC Number: 4.1.1.50</p>

<p>Confidence Level: 1</p>

<p>AUTHORS: Amino acid metabolism</p>

<p/>

</body>

</notes>

<listOfReactants>

<speciesReference species="M_H_c" stoichiometry="1"/>

<speciesReference species="M_SAM_c" stoichiometry="1"/>

</listOfReactants>

<listOfProducts>

<speciesReference species="M_CO2_c" stoichiometry="1"/>
```

```xml
  <speciesReference species="M_SAMA_c" stoichiometry="1"/>
 </listOfProducts>
 <kineticLaw>
  <math xmlns="http://www.w3.org/1998/Math/MathML">
   <ci> FLUX_VALUE </ci>
  </math>
  <listOfParameters>
   <parameter id="LOWER_BOUND" value="0" units="mmol_per_gDW_per_hr" constant="true"/>
   <parameter id="UPPER_BOUND" value="1000" units="mmol_per_gDW_per_hr" constant="true"/>
   <parameter id="FLUX_VALUE" value="0" units="mmol_per_gDW_per_hr" constant="true"/>
   <parameter id="OBJECTIVE_COEFFICIENT" value="0" units="mmol_per_gDW_per_hr" constant="true"/>
  </listOfParameters>
 </kineticLaw>
</reaction>
<reaction id="R_R240" name="S-adenosylmethioninamine:putrescine 3-aminopropyltransferase" reversible="false">
 <notes>
  <body xmlns="http://www.w3.org/1999/xhtml">
   <p>GENE_ASSOCIATION: TTC0472</p>
   <p>SUBSYSTEM: Cysteine and methionine metabolism</p>
   <p>EC Number: 2.5.1.16</p>
   <p>Confidence Level: 1</p>
   <p>AUTHORS: Amino acid metabolism</p>
   <p/>
  </body>
 </notes>
 <listOfReactants>
  <speciesReference species="M_SAMA_c" stoichiometry="1"/>
  <speciesReference species="M_PUT_c" stoichiometry="1"/>
 </listOfReactants>
```

```xml
  <listOfProducts>
    <speciesReference species="M_H_c" stoichiometry="1"/>
    <speciesReference species="M_MTA_c" stoichiometry="1"/>
    <speciesReference species="M_SPRD_c" stoichiometry="1"/>
  </listOfProducts>
  <kineticLaw>
    <math xmlns="http://www.w3.org/1998/Math/MathML">
      <ci> FLUX_VALUE </ci>
    </math>
    <listOfParameters>
      <parameter id="LOWER_BOUND" value="0" units="mmol_per_gDW_per_hr" constant="true"/>
      <parameter id="UPPER_BOUND" value="1000" units="mmol_per_gDW_per_hr" constant="true"/>
      <parameter id="FLUX_VALUE" value="0" units="mmol_per_gDW_per_hr" constant="true"/>
      <parameter id="OBJECTIVE_COEFFICIENT" value="0" units="mmol_per_gDW_per_hr" constant="true"/>
    </listOfParameters>
  </kineticLaw>
</reaction>
<reaction id="R_R241" name="S-adenosylmethioninamine:spermidine 3-aminopropyltransferase" reversible="true">
  <notes>
    <body xmlns="http://www.w3.org/1999/xhtml">
      <p>GENE_ASSOCIATION: TTC0472</p>
      <p>SUBSYSTEM: Cysteine and methionine metabolism</p>
      <p>EC Number: 2.5.1.16</p>
      <p>Confidence Level: 1</p>
      <p>AUTHORS: Amino acid metabolism</p>
      <p/>
    </body>
  </notes>
  <listOfReactants>
```

```
    <speciesReference species="M_SAMA_c" stoichiometry="1"/>
    <speciesReference species="M_SPRD_c" stoichiometry="1"/>
  </listOfReactants>
  <listOfProducts>
    <speciesReference species="M_H_c" stoichiometry="1"/>
    <speciesReference species="M_MTA_c" stoichiometry="1"/>
    <speciesReference species="M_SPR_c" stoichiometry="1"/>
  </listOfProducts>
  <kineticLaw>
    <math xmlns="http://www.w3.org/1998/Math/MathML">
      <ci> FLUX_VALUE </ci>
    </math>
    <listOfParameters>
      <parameter id="LOWER_BOUND" value="-1000" units="mmol_per_gDW_per_hr" constant="true"/>
      <parameter id="UPPER_BOUND" value="1000" units="mmol_per_gDW_per_hr" constant="true"/>
      <parameter id="FLUX_VALUE" value="0" units="mmol_per_gDW_per_hr" constant="true"/>
      <parameter id="OBJECTIVE_COEFFICIENT" value="0" units="mmol_per_gDW_per_hr" constant="true"/>
    </listOfParameters>
  </kineticLaw>
</reaction>
<reaction id="R_R242" name="Methylthioadenosine methylthioribohydrolase" reversible="false">
  <notes>
    <body xmlns="http://www.w3.org/1999/xhtml">
      <p>GENE_ASSOCIATION: TTC1185</p>
      <p>SUBSYSTEM: Cysteine and methionine metabolism</p>
      <p>EC Number: 3.2.2.9</p>
      <p>Confidence Level: 1</p>
      <p>AUTHORS: Amino acid metabolism</p>
      <p/>
```

```xml
    </body>
  </notes>
  <listOfReactants>
    <speciesReference species="M_H2O_c" stoichiometry="1"/>
    <speciesReference species="M_MTA_c" stoichiometry="1"/>
  </listOfReactants>
  <listOfProducts>
    <speciesReference species="M_ADN_c" stoichiometry="1"/>
    <speciesReference species="M_MTH_RIB_c" stoichiometry="1"/>
  </listOfProducts>
  <kineticLaw>
    <math xmlns="http://www.w3.org/1998/Math/MathML">
      <ci> FLUX_VALUE </ci>
    </math>
    <listOfParameters>
      <parameter id="LOWER_BOUND" value="0" units="mmol_per_gDW_per_hr" constant="true"/>
      <parameter id="UPPER_BOUND" value="1000" units="mmol_per_gDW_per_hr" constant="true"/>
      <parameter id="FLUX_VALUE" value="0" units="mmol_per_gDW_per_hr" constant="true"/>
      <parameter id="OBJECTIVE_COEFFICIENT" value="0" units="mmol_per_gDW_per_hr" constant="true"/>
    </listOfParameters>
  </kineticLaw>
</reaction>
<reaction id="R_R243" name="5-Methylthio-5-deoxy-D-ribose-1-phosphate ketol-isomerase" reversible="true">
  <notes>
    <body xmlns="http://www.w3.org/1999/xhtml">
      <p>GENE_ASSOCIATION: TTC0707</p>
      <p>SUBSYSTEM: Cysteine and methionine metabolism</p>
      <p>EC Number: 5.3.1.23</p>
      <p>Confidence Level: 1</p>
```

```
      <p>AUTHORS: Amino acid metabolism</p>
      <p/>
    </body>
  </notes>
  <listOfReactants>
    <speciesReference species="M_MTh_RIB1P_c" stoichiometry="1"/>
  </listOfReactants>
  <listOfProducts>
    <speciesReference species="M_MTh_RIBL1P_c" stoichiometry="1"/>
  </listOfProducts>
  <kineticLaw>
    <math xmlns="http://www.w3.org/1998/Math/MathML">
      <ci> FLUX_VALUE </ci>
    </math>
    <listOfParameters>
      <parameter id="LOWER_BOUND" value="-1000" units="mmol_per_gDW_per_hr" constant="true"/>
      <parameter id="UPPER_BOUND" value="1000" units="mmol_per_gDW_per_hr" constant="true"/>
      <parameter id="FLUX_VALUE" value="0" units="mmol_per_gDW_per_hr" constant="true"/>
      <parameter id="OBJECTIVE_COEFFICIENT" value="0" units="mmol_per_gDW_per_hr" constant="true"/>
    </listOfParameters>
  </kineticLaw>
</reaction>
<reaction id="R_R244" name="L-Leucine:2-oxoglutarate aminotransferase" reversible="true">
  <notes>
    <body xmlns="http://www.w3.org/1999/xhtml">
      <p>GENE_ASSOCIATION: TTC1870</p>
      <p>SUBSYSTEM: Valine, Leucine, and Isoleucine Metabolism</p>
      <p>EC Number: 2.6.1.42</p>
      <p>Confidence Level: 1</p>
```

```
      <p>AUTHORS: Amino acid metabolism</p>
      <p/>
    </body>
  </notes>
  <listOfReactants>
    <speciesReference species="M_L_GLU_c" stoichiometry="1"/>
    <speciesReference species="M_4M_2OPENTN_c" stoichiometry="1"/>
  </listOfReactants>
  <listOfProducts>
    <speciesReference species="M_L_LEU_c" stoichiometry="1"/>
    <speciesReference species="M_AKG_c" stoichiometry="1"/>
  </listOfProducts>
  <kineticLaw>
    <math xmlns="http://www.w3.org/1998/Math/MathML">
      <ci> FLUX_VALUE </ci>
    </math>
    <listOfParameters>
      <parameter id="LOWER_BOUND" value="-1000" units="mmol_per_gDW_per_hr" constant="true"/>
      <parameter id="UPPER_BOUND" value="1000" units="mmol_per_gDW_per_hr" constant="true"/>
      <parameter id="FLUX_VALUE" value="0" units="mmol_per_gDW_per_hr" constant="true"/>
      <parameter id="OBJECTIVE_COEFFICIENT" value="0" units="mmol_per_gDW_per_hr" constant="true"/>
    </listOfParameters>
  </kineticLaw>
</reaction>
<reaction id="R_R245" name="L-Valine:2-oxoglutarate aminotransferase" reversible="true">
  <notes>
    <body xmlns="http://www.w3.org/1999/xhtml">
      <p>GENE_ASSOCIATION: TTC1870</p>
      <p>SUBSYSTEM: Valine, Leucine, and Isoleucine Metabolism</p>
```

```
      <p>EC Number: 2.6.1.42</p>
      <p>Confidence Level: 1</p>
      <p>AUTHORS: Amino acid metabolism</p>
      <p/>
    </body>
  </notes>
  <listOfReactants>
    <speciesReference species="M_L_VAL_c" stoichiometry="1"/>
    <speciesReference species="M_AKG_c" stoichiometry="1"/>
  </listOfReactants>
  <listOfProducts>
    <speciesReference species="M_L_GLU_c" stoichiometry="1"/>
    <speciesReference species="M_2K3M_BUT_c" stoichiometry="1"/>
  </listOfProducts>
  <kineticLaw>
    <math xmlns="http://www.w3.org/1998/Math/MathML">
      <ci> FLUX_VALUE </ci>
    </math>
    <listOfParameters>
      <parameter id="LOWER_BOUND" value="-1000" units="mmol_per_gDW_per_hr" constant="true"/>
      <parameter id="UPPER_BOUND" value="1000" units="mmol_per_gDW_per_hr" constant="true"/>
      <parameter id="FLUX_VALUE" value="0" units="mmol_per_gDW_per_hr" constant="true"/>
      <parameter id="OBJECTIVE_COEFFICIENT" value="0" units="mmol_per_gDW_per_hr" constant="true"/>
    </listOfParameters>
  </kineticLaw>
</reaction>
<reaction id="R_R246" name="L-Isoleucine:2-oxoglutarate aminotransferase" reversible="true">
  <notes>
    <body xmlns="http://www.w3.org/1999/xhtml">
```

```
    <p>GENE_ASSOCIATION: TTC1870</p>
    <p>SUBSYSTEM: Valine, Leucine, and Isoleucine Metabolism</p>
    <p>EC Number: 2.6.1.42</p>
    <p>Confidence Level: 1</p>
    <p>AUTHORS: Amino acid metabolism</p>
    <p/>
   </body>
  </notes>
  <listOfReactants>
   <speciesReference species="M_L_ILE_c" stoichiometry="1"/>
   <speciesReference species="M_AKG_c" stoichiometry="1"/>
  </listOfReactants>
  <listOfProducts>
   <speciesReference species="M_L_GLU_c" stoichiometry="1"/>
   <speciesReference species="M_3M_2OPENTN_c" stoichiometry="1"/>
  </listOfProducts>
  <kineticLaw>
   <math xmlns="http://www.w3.org/1998/Math/MathML">
    <ci> FLUX_VALUE </ci>
   </math>
   <listOfParameters>
    <parameter id="LOWER_BOUND" value="-1000" units="mmol_per_gDW_per_hr" constant="true"/>
    <parameter id="UPPER_BOUND" value="1000" units="mmol_per_gDW_per_hr" constant="true"/>
    <parameter id="FLUX_VALUE" value="0" units="mmol_per_gDW_per_hr" constant="true"/>
    <parameter id="OBJECTIVE_COEFFICIENT" value="0" units="mmol_per_gDW_per_hr" constant="true"/>
   </listOfParameters>
  </kineticLaw>
 </reaction>
 <reaction id="R_R247" name="branched-chain alpha-keto acid dehydrogenase" reversible="false">
```

```xml
<notes>
  <body xmlns="http://www.w3.org/1999/xhtml">
    <p>GENE_ASSOCIATION: (TTC1756 and TTC1757)</p>
    <p>SUBSYSTEM: Valine, Leucine, and Isoleucine Metabolism</p>
    <p>EC Number: 1.2.4.4</p>
    <p>Confidence Level: 1</p>
    <p>AUTHORS: Amino acid metabolism</p>
    <p/>
  </body>
</notes>
<listOfReactants>
  <speciesReference species="M_3M_2OPENTN_c" stoichiometry="1"/>
  <speciesReference species="M_TPP_c" stoichiometry="1"/>
</listOfReactants>
<listOfProducts>
  <speciesReference species="M_CO2_c" stoichiometry="1"/>
  <speciesReference species="M_2MHYBUT_ThPP_c" stoichiometry="1"/>
</listOfProducts>
<kineticLaw>
  <math xmlns="http://www.w3.org/1998/Math/MathML">
    <ci> FLUX_VALUE </ci>
  </math>
  <listOfParameters>
    <parameter id="LOWER_BOUND" value="0" units="mmol_per_gDW_per_hr" constant="true"/>
    <parameter id="UPPER_BOUND" value="1000" units="mmol_per_gDW_per_hr" constant="true"/>
    <parameter id="FLUX_VALUE" value="0" units="mmol_per_gDW_per_hr" constant="true"/>
    <parameter id="OBJECTIVE_COEFFICIENT" value="0" units="mmol_per_gDW_per_hr" constant="true"/>
  </listOfParameters>
</kineticLaw>
```

```xml
</reaction>
<reaction id="R_R248" name="branched-chain alpha-keto acid dehydrogenase" reversible="false">
  <notes>
    <body xmlns="http://www.w3.org/1999/xhtml">
      <p>GENE_ASSOCIATION: (TTC1756 and TTC1757)</p>
      <p>SUBSYSTEM: Valine, Leucine, and Isoleucine Metabolism</p>
      <p>EC Number: 1.2.4.4</p>
      <p>Confidence Level: 1</p>
      <p>AUTHORS: Amino acid metabolism</p>
      <p/>
    </body>
  </notes>
  <listOfReactants>
    <speciesReference species="M_4M_2OPENTN_c" stoichiometry="1"/>
    <speciesReference species="M_TPP_c" stoichiometry="1"/>
  </listOfReactants>
  <listOfProducts>
    <speciesReference species="M_CO2_c" stoichiometry="1"/>
    <speciesReference species="M_3MHYBUT_ThPP_c" stoichiometry="1"/>
  </listOfProducts>
  <kineticLaw>
    <math xmlns="http://www.w3.org/1998/Math/MathML">
      <ci> FLUX_VALUE </ci>
    </math>
    <listOfParameters>
      <parameter id="LOWER_BOUND" value="0" units="mmol_per_gDW_per_hr" constant="true"/>
      <parameter id="UPPER_BOUND" value="1000" units="mmol_per_gDW_per_hr" constant="true"/>
      <parameter id="FLUX_VALUE" value="0" units="mmol_per_gDW_per_hr" constant="true"/>
      <parameter id="OBJECTIVE_COEFFICIENT" value="0" units="mmol_per_gDW_per_hr" constant="true"/>
```

```
    </listOfParameters>
  </kineticLaw>
</reaction>
<reaction id="R_R249" name="branched-chain alpha-keto acid dehydrogenase" reversible="false">
  <notes>
    <body xmlns="http://www.w3.org/1999/xhtml">
      <p>GENE_ASSOCIATION: (TTC1756 and TTC1757)</p>
      <p>SUBSYSTEM: Valine, Leucine, and Isoleucine Metabolism</p>
      <p>EC Number: 1.2.4.4</p>
      <p>Confidence Level: 1</p>
      <p>AUTHORS: Amino acid metabolism</p>
      <p/>
    </body>
  </notes>
  <listOfReactants>
    <speciesReference species="M_2K3M_BUT_c" stoichiometry="1"/>
    <speciesReference species="M_TPP_c" stoichiometry="1"/>
  </listOfReactants>
  <listOfProducts>
    <speciesReference species="M_CO2_c" stoichiometry="1"/>
    <speciesReference species="M_2MHYPROP_ThPP_c" stoichiometry="1"/>
  </listOfProducts>
  <kineticLaw>
    <math xmlns="http://www.w3.org/1998/Math/MathML">
      <ci> FLUX_VALUE </ci>
    </math>
    <listOfParameters>
      <parameter id="LOWER_BOUND" value="0" units="mmol_per_gDW_per_hr" constant="true"/>
      <parameter id="UPPER_BOUND" value="1000" units="mmol_per_gDW_per_hr" constant="true"/>
```

```xml
      <parameter id="FLUX_VALUE" value="0" units="mmol_per_gDW_per_hr" constant="true"/>

      <parameter id="OBJECTIVE_COEFFICIENT" value="0" units="mmol_per_gDW_per_hr" constant="true"/>

    </listOfParameters>

  </kineticLaw>

</reaction>

<reaction id="R_R250" name="branched-chain alpha-keto acid dehydrogenase" reversible="false">

  <notes>

    <body xmlns="http://www.w3.org/1999/xhtml">

      <p>GENE_ASSOCIATION: (TTC1756 and TTC1757)</p>

      <p>SUBSYSTEM: Valine, Leucine, and Isoleucine Metabolism</p>

      <p>EC Number: 1.2.4.4</p>

      <p>Confidence Level: 1</p>

      <p>AUTHORS: Amino acid metabolism</p>

      <p/>

    </body>

  </notes>

  <listOfReactants>

    <speciesReference species="M_2MHYPROP_ThPP_c" stoichiometry="1"/>

    <speciesReference species="M_ENZN6_LPL_c" stoichiometry="1"/>

  </listOfReactants>

  <listOfProducts>

    <speciesReference species="M_TPP_c" stoichiometry="1"/>

    <speciesReference species="M_DHYLPL_MPROP_MPROP_DHLPL_c" stoichiometry="1"/>

  </listOfProducts>

  <kineticLaw>

    <math xmlns="http://www.w3.org/1998/Math/MathML">

      <ci> FLUX_VALUE </ci>

    </math>

    <listOfParameters>
```

```xml
<parameter id="LOWER_BOUND" value="0" units="mmol_per_gDW_per_hr" constant="true"/>
<parameter id="UPPER_BOUND" value="1000" units="mmol_per_gDW_per_hr" constant="true"/>
<parameter id="FLUX_VALUE" value="0" units="mmol_per_gDW_per_hr" constant="true"/>
<parameter id="OBJECTIVE_COEFFICIENT" value="0" units="mmol_per_gDW_per_hr" constant="true"/>
</listOfParameters>
</kineticLaw>
</reaction>
<reaction id="R_R251" name="branched-chain alpha-keto acid dehydrogenase" reversible="false">
<notes>
<body xmlns="http://www.w3.org/1999/xhtml">
<p>GENE_ASSOCIATION: (TTC1756 and TTC1757)</p>
<p>SUBSYSTEM: Valine, Leucine, and Isoleucine Metabolism</p>
<p>EC Number: 1.2.4.4</p>
<p>Confidence Level: 1</p>
<p>AUTHORS: Amino acid metabolism</p>
<p/>
</body>
</notes>
<listOfReactants>
<speciesReference species="M_3MHYBUT_ThPP_c" stoichiometry="1"/>
<speciesReference species="M_ENZN6_LPL_c" stoichiometry="1"/>
</listOfReactants>
<listOfProducts>
<speciesReference species="M_TPP_c" stoichiometry="1"/>
<speciesReference species="M_DHYLPL_MPROP_3MBUT_DHLPL_c" stoichiometry="1"/>
</listOfProducts>
<kineticLaw>
<math xmlns="http://www.w3.org/1998/Math/MathML">
<ci> FLUX_VALUE </ci>
```

```xml
      </math>
      <listOfParameters>
        <parameter id="LOWER_BOUND" value="0" units="mmol_per_gDW_per_hr" constant="true"/>
        <parameter id="UPPER_BOUND" value="1000" units="mmol_per_gDW_per_hr" constant="true"/>
        <parameter id="FLUX_VALUE" value="0" units="mmol_per_gDW_per_hr" constant="true"/>
        <parameter id="OBJECTIVE_COEFFICIENT" value="0" units="mmol_per_gDW_per_hr" constant="true"/>
      </listOfParameters>
    </kineticLaw>
  </reaction>
  <reaction id="R_R252" name="branched-chain alpha-keto acid dehydrogenase" reversible="false">
    <notes>
      <body xmlns="http://www.w3.org/1999/xhtml">
        <p>GENE_ASSOCIATION: (TTC1756 and TTC1757)</p>
        <p>SUBSYSTEM: Valine, Leucine, and Isoleucine Metabolism</p>
        <p>EC Number: 1.2.4.4</p>
        <p>Confidence Level: 1</p>
        <p>AUTHORS: Amino acid metabolism</p>
        <p/>
      </body>
    </notes>
    <listOfReactants>
      <speciesReference species="M_2MHYBUT_ThPP_c" stoichiometry="1"/>
      <speciesReference species="M_ENZN6_LPL_c" stoichiometry="1"/>
    </listOfReactants>
    <listOfProducts>
      <speciesReference species="M_TPP_c" stoichiometry="1"/>
      <speciesReference species="M_DHYLPL_MPROP_2MBUT_DHLPL_c" stoichiometry="1"/>
    </listOfProducts>
    <kineticLaw>
```

```
    <math xmlns="http://www.w3.org/1998/Math/MathML">
      <ci> FLUX_VALUE </ci>
    </math>
    <listOfParameters>
      <parameter id="LOWER_BOUND" value="0" units="mmol_per_gDW_per_hr" constant="true"/>
      <parameter id="UPPER_BOUND" value="1000" units="mmol_per_gDW_per_hr" constant="true"/>
      <parameter id="FLUX_VALUE" value="0" units="mmol_per_gDW_per_hr" constant="true"/>
      <parameter id="OBJECTIVE_COEFFICIENT" value="0" units="mmol_per_gDW_per_hr" constant="true"/>
    </listOfParameters>
  </kineticLaw>
</reaction>
<reaction id="R_R253" name="(S)-2-methylbutanoyl-CoA:acceptor 2,3-oxidoreductase" reversible="false">
  <notes>
    <body xmlns="http://www.w3.org/1999/xhtml">
      <p>GENE_ASSOCIATION: TTC0238</p>
      <p>SUBSYSTEM: Valine, Leucine, and Isoleucine Metabolism</p>
      <p>EC Number: 1.3.8.1</p>
      <p>Confidence Level: 1</p>
      <p>AUTHORS: Amino acid metabolism</p>
      <p/>
    </body>
  </notes>
  <listOfReactants>
    <speciesReference species="M_FAD_c" stoichiometry="1"/>
    <speciesReference species="M_MBUTNCoA_c" stoichiometry="1"/>
  </listOfReactants>
  <listOfProducts>
    <speciesReference species="M_FADH2_c" stoichiometry="1"/>
    <speciesReference species="M_MBUT_ENCoA_c" stoichiometry="1"/>
```

```
  </listOfProducts>
  <kineticLaw>
    <math xmlns="http://www.w3.org/1998/Math/MathML">
      <ci> FLUX_VALUE </ci>
    </math>
    <listOfParameters>
      <parameter id="LOWER_BOUND" value="0" units="mmol_per_gDW_per_hr" constant="true"/>
      <parameter id="UPPER_BOUND" value="1000" units="mmol_per_gDW_per_hr" constant="true"/>
      <parameter id="FLUX_VALUE" value="0" units="mmol_per_gDW_per_hr" constant="true"/>
      <parameter id="OBJECTIVE_COEFFICIENT" value="0" units="mmol_per_gDW_per_hr" constant="true"/>
    </listOfParameters>
  </kineticLaw>
</reaction>
<reaction id="R_R254" name="2-methylpropanoyl-CoA:(acceptor) 2,3-oxidoreductase" reversible="true">
  <notes>
    <body xmlns="http://www.w3.org/1999/xhtml">
      <p>GENE_ASSOCIATION: TTC0238</p>
      <p>SUBSYSTEM: Valine, Leucine, and Isoleucine Metabolism</p>
      <p>EC Number: 1.3.8.1</p>
      <p>Confidence Level: 1</p>
      <p>AUTHORS: Amino acid metabolism</p>
      <p/>
    </body>
  </notes>
  <listOfReactants>
    <speciesReference species="M_FAD_c" stoichiometry="1"/>
    <speciesReference species="M_2M_PROCoA_c" stoichiometry="1"/>
  </listOfReactants>
  <listOfProducts>
```

```
    <speciesReference species="M_FADH2_c" stoichiometry="1"/>
    <speciesReference species="M_MACRYCoA_c" stoichiometry="1"/>
  </listOfProducts>
  <kineticLaw>
    <math xmlns="http://www.w3.org/1998/Math/MathML">
      <ci> FLUX_VALUE </ci>
    </math>
    <listOfParameters>
      <parameter id="LOWER_BOUND" value="-1000" units="mmol_per_gDW_per_hr" constant="true"/>
      <parameter id="UPPER_BOUND" value="1000" units="mmol_per_gDW_per_hr" constant="true"/>
      <parameter id="FLUX_VALUE" value="0" units="mmol_per_gDW_per_hr" constant="true"/>
      <parameter id="OBJECTIVE_COEFFICIENT" value="0" units="mmol_per_gDW_per_hr" constant="true"/>
    </listOfParameters>
  </kineticLaw>
</reaction>
<reaction id="R_R255" name="(2S,3S)-3-Hydroxy-2-methylbutanoyl-CoA hydro-liase" reversible="false">
  <notes>
    <body xmlns="http://www.w3.org/1999/xhtml">
      <p>GENE_ASSOCIATION: TTC1768</p>
      <p>SUBSYSTEM: Valine, Leucine, and Isoleucine Metabolism</p>
      <p>EC Number: 4.2.1.17</p>
      <p>Confidence Level: 1</p>
      <p>AUTHORS: Amino acid metabolism</p>
      <p/>
    </body>
  </notes>
  <listOfReactants>
    <speciesReference species="M_H2O_c" stoichiometry="1"/>
    <speciesReference species="M_MBUT_ENCoA_c" stoichiometry="1"/>
```

```
    </listOfReactants>
    <listOfProducts>
      <speciesReference species="M_HY_MBUTRCoA_c" stoichiometry="1"/>
    </listOfProducts>
    <kineticLaw>
      <math xmlns="http://www.w3.org/1998/Math/MathML">
        <ci> FLUX_VALUE </ci>
      </math>
      <listOfParameters>
        <parameter id="LOWER_BOUND" value="0" units="mmol_per_gDW_per_hr" constant="true"/>
        <parameter id="UPPER_BOUND" value="1000" units="mmol_per_gDW_per_hr" constant="true"/>
        <parameter id="FLUX_VALUE" value="0" units="mmol_per_gDW_per_hr" constant="true"/>
        <parameter id="OBJECTIVE_COEFFICIENT" value="0" units="mmol_per_gDW_per_hr" constant="true"/>
      </listOfParameters>
    </kineticLaw>
  </reaction>
  <reaction id="R_R256" name="(S)-3-Hydroxyisobutyryl-CoA hydro-lyase" reversible="false">
    <notes>
      <body xmlns="http://www.w3.org/1999/xhtml">
        <p>GENE_ASSOCIATION: TTC1768</p>
        <p>SUBSYSTEM: Valine, Leucine, and Isoleucine Metabolism</p>
        <p>EC Number: 4.2.1.17</p>
        <p>Confidence Level: 1</p>
        <p>AUTHORS: Amino acid metabolism</p>
        <p/>
      </body>
    </notes>
    <listOfReactants>
      <speciesReference species="M_H2O_c" stoichiometry="1"/>
```

```
          <speciesReference species="M_MACRYCoA_c" stoichiometry="1"/>
        </listOfReactants>
        <listOfProducts>
          <speciesReference species="M_HYIBUTRCoA_c" stoichiometry="1"/>
        </listOfProducts>
        <kineticLaw>
          <math xmlns="http://www.w3.org/1998/Math/MathML">
            <ci> FLUX_VALUE </ci>
          </math>
          <listOfParameters>
            <parameter id="LOWER_BOUND" value="0" units="mmol_per_gDW_per_hr" constant="true"/>
            <parameter id="UPPER_BOUND" value="1000" units="mmol_per_gDW_per_hr" constant="true"/>
            <parameter id="FLUX_VALUE" value="0" units="mmol_per_gDW_per_hr" constant="true"/>
            <parameter id="OBJECTIVE_COEFFICIENT" value="0" units="mmol_per_gDW_per_hr" constant="true"/>
          </listOfParameters>
        </kineticLaw>
      </reaction>
      <reaction id="R_R257" name="(S)-3-hydroxy-3-methylglutaryl-CoA acetoacetate-lyase (acetyl-CoA-forming)"
reversible="false">
        <notes>
          <body xmlns="http://www.w3.org/1999/xhtml">
            <p>GENE_ASSOCIATION: TTC1162</p>
            <p>SUBSYSTEM: Valine, Leucine, and Isoleucine Metabolism</p>
            <p>EC Number: 4.1.3.4</p>
            <p>Confidence Level: 1</p>
            <p>AUTHORS: Amino acid metabolism</p>
            <p/>
          </body>
        </notes>
```

```
  <listOfReactants>
    <speciesReference species="M_HYM_GLUTRCoA_c" stoichiometry="1"/>
  </listOfReactants>
  <listOfProducts>
    <speciesReference species="M_ACoA_c" stoichiometry="1"/>
    <speciesReference species="M_AAC_c" stoichiometry="1"/>
  </listOfProducts>
  <kineticLaw>
    <math xmlns="http://www.w3.org/1998/Math/MathML">
      <ci> FLUX_VALUE </ci>
    </math>
    <listOfParameters>
      <parameter id="LOWER_BOUND" value="0" units="mmol_per_gDW_per_hr" constant="true"/>
      <parameter id="UPPER_BOUND" value="1000" units="mmol_per_gDW_per_hr" constant="true"/>
      <parameter id="FLUX_VALUE" value="0" units="mmol_per_gDW_per_hr" constant="true"/>
      <parameter id="OBJECTIVE_COEFFICIENT" value="0" units="mmol_per_gDW_per_hr" constant="true"/>
    </listOfParameters>
  </kineticLaw>
</reaction>
<reaction id="R_R258" name="3-Hydroxy-2-methylpropanoate:NAD+ oxidoreductase" reversible="false">
  <notes>
    <body xmlns="http://www.w3.org/1999/xhtml">
      <p>GENE_ASSOCIATION: (TTC1749 or TTC0534)</p>
      <p>SUBSYSTEM: Valine, Leucine, and Isoleucine Metabolism</p>
      <p>EC Number: 1.1.1.31 or 1.1.1.35</p>
      <p>Confidence Level: 1</p>
      <p>AUTHORS: Amino acid metabolism</p>
      <p/>
    </body>
```

```xml
  </notes>
  <listOfReactants>
    <speciesReference species="M_NAD_c" stoichiometry="1"/>
    <speciesReference species="M_HYIBUTRA_c" stoichiometry="1"/>
  </listOfReactants>
  <listOfProducts>
    <speciesReference species="M_H_c" stoichiometry="1"/>
    <speciesReference species="M_NADH_c" stoichiometry="1"/>
    <speciesReference species="M_MMAL_SA_c" stoichiometry="1"/>
  </listOfProducts>
  <kineticLaw>
    <math xmlns="http://www.w3.org/1998/Math/MathML">
      <ci> FLUX_VALUE </ci>
    </math>
    <listOfParameters>
      <parameter id="LOWER_BOUND" value="0" units="mmol_per_gDW_per_hr" constant="true"/>
      <parameter id="UPPER_BOUND" value="1000" units="mmol_per_gDW_per_hr" constant="true"/>
      <parameter id="FLUX_VALUE" value="0" units="mmol_per_gDW_per_hr" constant="true"/>
      <parameter id="OBJECTIVE_COEFFICIENT" value="0" units="mmol_per_gDW_per_hr" constant="true"/>
    </listOfParameters>
  </kineticLaw>
</reaction>
<reaction id="R_R259" name="(S)-Methylmalonate semialdehyde:NAD+ oxidoreductase" reversible="false">
  <notes>
    <body xmlns="http://www.w3.org/1999/xhtml">
      <p>GENE_ASSOCIATION: TTC0513</p>
      <p>SUBSYSTEM: Valine, Leucine, and Isoleucine Metabolism</p>
      <p>EC Number: 1.2.1.3</p>
      <p>Confidence Level: 1</p>
```

```xml
      <p>AUTHORS: Amino acid metabolism</p>
      <p/>
    </body>
  </notes>
  <listOfReactants>
    <speciesReference species="M_H2O_c" stoichiometry="1"/>
    <speciesReference species="M_NAD_c" stoichiometry="1"/>
    <speciesReference species="M_MMAL_SA_c" stoichiometry="1"/>
  </listOfReactants>
  <listOfProducts>
    <speciesReference species="M_H_c" stoichiometry="1"/>
    <speciesReference species="M_NADH_c" stoichiometry="1"/>
    <speciesReference species="M_MMAL_c" stoichiometry="1"/>
  </listOfProducts>
  <kineticLaw>
    <math xmlns="http://www.w3.org/1998/Math/MathML">
      <ci> FLUX_VALUE </ci>
    </math>
    <listOfParameters>
      <parameter id="LOWER_BOUND" value="0" units="mmol_per_gDW_per_hr" constant="true"/>
      <parameter id="UPPER_BOUND" value="1000" units="mmol_per_gDW_per_hr" constant="true"/>
      <parameter id="FLUX_VALUE" value="0" units="mmol_per_gDW_per_hr" constant="true"/>
      <parameter id="OBJECTIVE_COEFFICIENT" value="0" units="mmol_per_gDW_per_hr" constant="true"/>
    </listOfParameters>
  </kineticLaw>
</reaction>
<reaction id="R_R260" name="acetyl-CoA:propanoyl-CoA 2-C-acetyltransferase" reversible="false">
  <notes>
    <body xmlns="http://www.w3.org/1999/xhtml">
```

```
<p>GENE_ASSOCIATION: ((TTC0535 or TTC0623) or (TTC0191 or TTC0330))</p>
<p>SUBSYSTEM: Valine, Leucine, and Isoleucine Metabolism</p>
<p>EC Number: 2.3.1.16 or 2.3.1.9</p>
<p>Confidence Level: 1</p>
<p>AUTHORS: Amino acid metabolism</p>
<p/>
</body>
</notes>
<listOfReactants>
<speciesReference species="M_CoA_c" stoichiometry="1"/>
<speciesReference species="M_MAACCoA_c" stoichiometry="1"/>
</listOfReactants>
<listOfProducts>
<speciesReference species="M_ACoA_c" stoichiometry="1"/>
<speciesReference species="M_PRCoA_c" stoichiometry="1"/>
</listOfProducts>
<kineticLaw>
<math xmlns="http://www.w3.org/1998/Math/MathML">
<ci> FLUX_VALUE </ci>
</math>
<listOfParameters>
<parameter id="LOWER_BOUND" value="0" units="mmol_per_gDW_per_hr" constant="true"/>
<parameter id="UPPER_BOUND" value="1000" units="mmol_per_gDW_per_hr" constant="true"/>
<parameter id="FLUX_VALUE" value="0" units="mmol_per_gDW_per_hr" constant="true"/>
<parameter id="OBJECTIVE_COEFFICIENT" value="0" units="mmol_per_gDW_per_hr" constant="true"/>
</listOfParameters>
</kineticLaw>
</reaction>
<reaction id="R_R261" name="(R)-Methylmalonyl-CoA CoA-carbonylmutase" reversible="true">
```

```xml
  <notes>
    <body xmlns="http://www.w3.org/1999/xhtml">
      <p>GENE_ASSOCIATION: (TTC0676 and (TTC0677 or TTC0882))</p>
      <p>SUBSYSTEM: Valine, Leucine, and Isoleucine Metabolism</p>
      <p>EC Number: 5.4.99.2</p>
      <p>Confidence Level: 1</p>
      <p>AUTHORS: Amino acid metabolism</p>
      <p/>
    </body>
  </notes>
  <listOfReactants>
    <speciesReference species="M_R_MMALCoA_c" stoichiometry="1"/>
  </listOfReactants>
  <listOfProducts>
    <speciesReference species="M_SUCCoA_c" stoichiometry="1"/>
  </listOfProducts>
  <kineticLaw>
    <math xmlns="http://www.w3.org/1998/Math/MathML">
      <ci> FLUX_VALUE </ci>
    </math>
    <listOfParameters>
      <parameter id="LOWER_BOUND" value="-1000" units="mmol_per_gDW_per_hr" constant="true"/>
      <parameter id="UPPER_BOUND" value="1000" units="mmol_per_gDW_per_hr" constant="true"/>
      <parameter id="FLUX_VALUE" value="0" units="mmol_per_gDW_per_hr" constant="true"/>
      <parameter id="OBJECTIVE_COEFFICIENT" value="0" units="mmol_per_gDW_per_hr" constant="true"/>
    </listOfParameters>
  </kineticLaw>
</reaction>
<reaction id="R_R262" name="Propanoyl-CoA:carbon-dioxide ligase (ADP-forming)" reversible="false">
```

```xml
<notes>
  <body xmlns="http://www.w3.org/1999/xhtml">
    <p>GENE_ASSOCIATION: (TTC0783 and TTC1192)</p>
    <p>SUBSYSTEM: Valine, Leucine, and Isoleucine Metabolism</p>
    <p>EC Number: 6.4.1.3</p>
    <p>Confidence Level: 1</p>
    <p>AUTHORS: Amino acid metabolism</p>
    <p/>
  </body>
</notes>
<listOfReactants>
  <speciesReference species="M_ATP_c" stoichiometry="1"/>
  <speciesReference species="M_HCO3_c" stoichiometry="1"/>
  <speciesReference species="M_PRCoA_c" stoichiometry="1"/>
</listOfReactants>
<listOfProducts>
  <speciesReference species="M_ADP_c" stoichiometry="1"/>
  <speciesReference species="M_Pi_c" stoichiometry="1"/>
  <speciesReference species="M_H_c" stoichiometry="1"/>
  <speciesReference species="M_MMALCoA_c" stoichiometry="1"/>
</listOfProducts>
<kineticLaw>
  <math xmlns="http://www.w3.org/1998/Math/MathML">
    <ci> FLUX_VALUE </ci>
  </math>
  <listOfParameters>
    <parameter id="LOWER_BOUND" value="0" units="mmol_per_gDW_per_hr" constant="true"/>
    <parameter id="UPPER_BOUND" value="1000" units="mmol_per_gDW_per_hr" constant="true"/>
    <parameter id="FLUX_VALUE" value="0" units="mmol_per_gDW_per_hr" constant="true"/>
```

```xml
      <parameter id="OBJECTIVE_COEFFICIENT" value="0" units="mmol_per_gDW_per_hr" constant="true"/>
    </listOfParameters>
  </kineticLaw>
</reaction>
<reaction id="R_R263" name="4-hydroxy-tetrahydrodipicolinate synthase" reversible="false">
  <notes>
    <body xmlns="http://www.w3.org/1999/xhtml">
      <p>GENE_ASSOCIATION: TTC0591</p>
      <p>SUBSYSTEM: Threonine and Lysine Metabolism</p>
      <p>EC Number: 4.2.1.52</p>
      <p>Confidence Level: 1</p>
      <p>AUTHORS: Amino acid metabolism</p>
      <p/>
    </body>
  </notes>
  <listOfReactants>
    <speciesReference species="M_PYR_c" stoichiometry="1"/>
    <speciesReference species="M_ASP_4SA_c" stoichiometry="1"/>
  </listOfReactants>
  <listOfProducts>
    <speciesReference species="M_H2O_c" stoichiometry="2"/>
    <speciesReference species="M_H_c" stoichiometry="1"/>
    <speciesReference species="M_DHY_DPICLN_c" stoichiometry="1"/>
  </listOfProducts>
  <kineticLaw>
    <math xmlns="http://www.w3.org/1998/Math/MathML">
      <ci> FLUX_VALUE </ci>
    </math>
    <listOfParameters>
```

```
<parameter id="LOWER_BOUND" value="0" units="mmol_per_gDW_per_hr" constant="true"/>
<parameter id="UPPER_BOUND" value="1000" units="mmol_per_gDW_per_hr" constant="true"/>
<parameter id="FLUX_VALUE" value="0" units="mmol_per_gDW_per_hr" constant="true"/>
<parameter id="OBJECTIVE_COEFFICIENT" value="0" units="mmol_per_gDW_per_hr" constant="true"/>
</listOfParameters>
</kineticLaw>
</reaction>
<reaction id="R_R264" name="UDP-N-acetylmuramoyl-L-alanyl-D-glutamyl-meso-2,6-diaminoheptanedioate:D-alanyl-D-alanine ligase(ADP-forming)" reversible="false">
<notes>
<body xmlns="http://www.w3.org/1999/xhtml">
<p>GENE_ASSOCIATION: TTC0714</p>
<p>SUBSYSTEM: Cell Envelope Biosynthesis</p>
<p>EC Number: 6.3.2.10</p>
<p>Confidence Level: 1</p>
<p>AUTHORS: Lipid metabolism</p>
<p/>
</body>
</notes>
<listOfReactants>
<speciesReference species="M_ATP_c" stoichiometry="1"/>
<speciesReference species="M_UDP_ACMUR_ALA_GLU_DAPIM_c" stoichiometry="1"/>
<speciesReference species="M_D_ALA_D_ALA_c" stoichiometry="1"/>
</listOfReactants>
<listOfProducts>
<speciesReference species="M_ADP_c" stoichiometry="1"/>
<speciesReference species="M_Pi_c" stoichiometry="1"/>
<speciesReference species="M_H_c" stoichiometry="1"/>
<speciesReference species="M_UDP_ACMUR_ALA_GLU_DAPIM_ALA_ALA_c" stoichiometry="1"/>
```

```
        </listOfProducts>
        <kineticLaw>
          <math xmlns="http://www.w3.org/1998/Math/MathML">
            <ci> FLUX_VALUE </ci>
          </math>
          <listOfParameters>
            <parameter id="LOWER_BOUND" value="0" units="mmol_per_gDW_per_hr" constant="true"/>
            <parameter id="UPPER_BOUND" value="1000" units="mmol_per_gDW_per_hr" constant="true"/>
            <parameter id="FLUX_VALUE" value="0" units="mmol_per_gDW_per_hr" constant="true"/>
            <parameter id="OBJECTIVE_COEFFICIENT" value="0" units="mmol_per_gDW_per_hr" constant="true"/>
          </listOfParameters>
        </kineticLaw>
      </reaction>
      <reaction id="R_R265" name="UDP-N-acetylmuramoyl-L-alanyl-D-glutamate:(L)-meso-2,6-diaminoheptanedioate gamma-
ligase (ADP-forming)" reversible="false">
        <notes>
          <body xmlns="http://www.w3.org/1999/xhtml">
            <p>GENE_ASSOCIATION: TTC1904</p>
            <p>SUBSYSTEM: Cell Envelope Biosynthesis</p>
            <p>EC Number: 6.3.2.13</p>
            <p>Confidence Level: 1</p>
            <p>AUTHORS: Lipid metabolism</p>
            <p/>
          </body>
        </notes>
        <listOfReactants>
          <speciesReference species="M_ATP_c" stoichiometry="1"/>
          <speciesReference species="M_UDP_ACMURM_ALA_GLUT_c" stoichiometry="1"/>
          <speciesReference species="M_M26_DAP_c" stoichiometry="1"/>
```

```xml
</listOfReactants>

<listOfProducts>

<speciesReference species="M_ADP_c" stoichiometry="1"/>

<speciesReference species="M_Pi_c" stoichiometry="1"/>

<speciesReference species="M_H_c" stoichiometry="1"/>

<speciesReference species="M_UDP_ACMUR_ALA_GLU_DAPIM_c" stoichiometry="1"/>

</listOfProducts>

<kineticLaw>

<math xmlns="http://www.w3.org/1998/Math/MathML">

<ci> FLUX_VALUE </ci>

</math>

<listOfParameters>

<parameter id="LOWER_BOUND" value="0" units="mmol_per_gDW_per_hr" constant="true"/>

<parameter id="UPPER_BOUND" value="1000" units="mmol_per_gDW_per_hr" constant="true"/>

<parameter id="FLUX_VALUE" value="0" units="mmol_per_gDW_per_hr" constant="true"/>

<parameter id="OBJECTIVE_COEFFICIENT" value="0" units="mmol_per_gDW_per_hr" constant="true"/>

</listOfParameters>

</kineticLaw>

</reaction>

<reaction id="R_R266" name="acetyl-CoA:2-oxoglutarate C-acetyltransferase (thioester-hydrolysing, carboxymethyl forming)" reversible="false">

<notes>

<body xmlns="http://www.w3.org/1999/xhtml">

<p>GENE_ASSOCIATION: TTC1550</p>

<p>SUBSYSTEM: Threonine and Lysine Metabolism</p>

<p>EC Number: 2.3.3.14</p>

<p>Confidence Level: 1</p>

<p>AUTHORS: Amino acid metabolism</p>

<p/>
```

```
      </body>
    </notes>
    <listOfReactants>
      <speciesReference species="M_H2O_c" stoichiometry="1"/>
      <speciesReference species="M_ACoA_c" stoichiometry="1"/>
      <speciesReference species="M_AKG_c" stoichiometry="1"/>
    </listOfReactants>
    <listOfProducts>
      <speciesReference species="M_CoA_c" stoichiometry="1"/>
      <speciesReference species="M_HCIT_c" stoichiometry="1"/>
    </listOfProducts>
    <kineticLaw>
      <math xmlns="http://www.w3.org/1998/Math/MathML">
        <ci> FLUX_VALUE </ci>
      </math>
      <listOfParameters>
        <parameter id="LOWER_BOUND" value="0" units="mmol_per_gDW_per_hr" constant="true"/>
        <parameter id="UPPER_BOUND" value="1000" units="mmol_per_gDW_per_hr" constant="true"/>
        <parameter id="FLUX_VALUE" value="0" units="mmol_per_gDW_per_hr" constant="true"/>
        <parameter id="OBJECTIVE_COEFFICIENT" value="0" units="mmol_per_gDW_per_hr" constant="true"/>
      </listOfParameters>
    </kineticLaw>
  </reaction>
  <reaction id="R_R267" name="(1R,2S)-1-hydroxybutane-1,2,4-tricarboxylate:NAD+ oxidoreductase (decarboxylating)" reversible="true">
    <notes>
      <body xmlns="http://www.w3.org/1999/xhtml">
        <p>GENE_ASSOCIATION: TTC1012</p>
        <p>SUBSYSTEM: Threonine and Lysine Metabolism</p>
```

```
          <p>EC Number: 1.1.1.87</p>
          <p>Confidence Level: 1</p>
          <p>AUTHORS: Amino acid metabolism</p>
          <p/>
        </body>
      </notes>
      <listOfReactants>
        <speciesReference species="M_OXLGLUTR_c" stoichiometry="1"/>
      </listOfReactants>
      <listOfProducts>
        <speciesReference species="M_CO2_c" stoichiometry="1"/>
        <speciesReference species="M_2OADPA_c" stoichiometry="1"/>
      </listOfProducts>
      <kineticLaw>
        <math xmlns="http://www.w3.org/1998/Math/MathML">
          <ci> FLUX_VALUE </ci>
        </math>
        <listOfParameters>
          <parameter id="LOWER_BOUND" value="-1000" units="mmol_per_gDW_per_hr" constant="true"/>
          <parameter id="UPPER_BOUND" value="1000" units="mmol_per_gDW_per_hr" constant="true"/>
          <parameter id="FLUX_VALUE" value="0" units="mmol_per_gDW_per_hr" constant="true"/>
          <parameter id="OBJECTIVE_COEFFICIENT" value="0" units="mmol_per_gDW_per_hr" constant="true"/>
        </listOfParameters>
      </kineticLaw>
    </reaction>
    <reaction id="R_R268" name="(1R,2S)-1-hydroxybutane-1,2,4-tricarboxylate:NAD+ oxidoreductase (decarboxylating)"
reversible="true">
      <notes>
        <body xmlns="http://www.w3.org/1999/xhtml">
```

```xml
      <p>GENE_ASSOCIATION: TTC1012</p>
      <p>SUBSYSTEM: Threonine and Lysine Metabolism</p>
      <p>EC Number: 1.1.1.87</p>
      <p>Confidence Level: 1</p>
      <p>AUTHORS: Amino acid metabolism</p>
      <p/>
    </body>
  </notes>
  <listOfReactants>
    <speciesReference species="M_NAD_c" stoichiometry="1"/>
    <speciesReference species="M_HICTR_c" stoichiometry="1"/>
  </listOfReactants>
  <listOfProducts>
    <speciesReference species="M_H_c" stoichiometry="1"/>
    <speciesReference species="M_NADH_c" stoichiometry="1"/>
    <speciesReference species="M_OXLGLUTR_c" stoichiometry="1"/>
  </listOfProducts>
  <kineticLaw>
    <math xmlns="http://www.w3.org/1998/Math/MathML">
      <ci> FLUX_VALUE </ci>
    </math>
    <listOfParameters>
      <parameter id="LOWER_BOUND" value="-1000" units="mmol_per_gDW_per_hr" constant="true"/>
      <parameter id="UPPER_BOUND" value="1000" units="mmol_per_gDW_per_hr" constant="true"/>
      <parameter id="FLUX_VALUE" value="0" units="mmol_per_gDW_per_hr" constant="true"/>
      <parameter id="OBJECTIVE_COEFFICIENT" value="0" units="mmol_per_gDW_per_hr" constant="true"/>
    </listOfParameters>
  </kineticLaw>
</reaction>
```

```xml
<reaction id="R_R269" name="L-2-aminoadipate:2-oxoglutarate aminotransferase" reversible="true">
  <notes>
    <body xmlns="http://www.w3.org/1999/xhtml">
      <p>GENE_ASSOCIATION: TTC0043</p>
      <p>SUBSYSTEM: Threonine and Lysine Metabolism</p>
      <p>EC Number: 2.6.1.39</p>
      <p>Confidence Level: 1</p>
      <p>AUTHORS: Amino acid metabolism</p>
      <p/>
    </body>
  </notes>
  <listOfReactants>
    <speciesReference species="M_AKG_c" stoichiometry="1"/>
    <speciesReference species="M_AADP_c" stoichiometry="1"/>
  </listOfReactants>
  <listOfProducts>
    <speciesReference species="M_L_GLU_c" stoichiometry="1"/>
    <speciesReference species="M_2OADPA_c" stoichiometry="1"/>
  </listOfProducts>
  <kineticLaw>
    <math xmlns="http://www.w3.org/1998/Math/MathML">
      <ci> FLUX_VALUE </ci>
    </math>
    <listOfParameters>
      <parameter id="LOWER_BOUND" value="-1000" units="mmol_per_gDW_per_hr" constant="true"/>
      <parameter id="UPPER_BOUND" value="1000" units="mmol_per_gDW_per_hr" constant="true"/>
      <parameter id="FLUX_VALUE" value="0" units="mmol_per_gDW_per_hr" constant="true"/>
      <parameter id="OBJECTIVE_COEFFICIENT" value="0" units="mmol_per_gDW_per_hr" constant="true"/>
    </listOfParameters>
```

```
  </kineticLaw>
</reaction>
<reaction id="R_R270" name="N-acetylaminoadipate kinase" reversible="false">
  <notes>
    <body xmlns="http://www.w3.org/1999/xhtml">
      <p>GENE_ASSOCIATION: (TTC1541 or TTC1586)</p>
      <p>SUBSYSTEM: Threonine and Lysine Metabolism</p>
      <p>EC Number: 2.7.2.- </p>
      <p>Confidence Level: 1</p>
      <p>AUTHORS: Amino acid metabolism</p>
      <p/>
    </body>
  </notes>
  <listOfReactants>
    <speciesReference species="M_ATP_c" stoichiometry="1"/>
    <speciesReference species="M_AC_AADP_c" stoichiometry="1"/>
  </listOfReactants>
  <listOfProducts>
    <speciesReference species="M_ADP_c" stoichiometry="1"/>
    <speciesReference species="M_AC_AADP_DELPi_c" stoichiometry="1"/>
  </listOfProducts>
  <kineticLaw>
    <math xmlns="http://www.w3.org/1998/Math/MathML">
      <ci> FLUX_VALUE </ci>
    </math>
    <listOfParameters>
      <parameter id="LOWER_BOUND" value="0" units="mmol_per_gDW_per_hr" constant="true"/>
      <parameter id="UPPER_BOUND" value="1000" units="mmol_per_gDW_per_hr" constant="true"/>
      <parameter id="FLUX_VALUE" value="0" units="mmol_per_gDW_per_hr" constant="true"/>
```

```
      <parameter id="OBJECTIVE_COEFFICIENT" value="0" units="mmol_per_gDW_per_hr" constant="true"/>
    </listOfParameters>
  </kineticLaw>
</reaction>
<reaction id="R_R271" name="N-acetyl-alpha-aminoadipate-phosphate reductase" reversible="false">
  <notes>
    <body xmlns="http://www.w3.org/1999/xhtml">
      <p>GENE_ASSOCIATION: (TTC0386 or TTC1542)</p>
      <p>SUBSYSTEM: Threonine and Lysine Metabolism</p>
      <p>EC Number: 1.2.1.38</p>
      <p>Confidence Level: 1</p>
      <p>AUTHORS: Amino acid metabolism</p>
      <p/>
    </body>
  </notes>
  <listOfReactants>
    <speciesReference species="M_H_c" stoichiometry="1"/>
    <speciesReference species="M_NADPH_c" stoichiometry="1"/>
    <speciesReference species="M_AC_AADP_DELPi_c" stoichiometry="1"/>
  </listOfReactants>
  <listOfProducts>
    <speciesReference species="M_Pi_c" stoichiometry="1"/>
    <speciesReference species="M_NADP_c" stoichiometry="1"/>
    <speciesReference species="M_AC_AADP_SA_c" stoichiometry="1"/>
  </listOfProducts>
  <kineticLaw>
    <math xmlns="http://www.w3.org/1998/Math/MathML">
      <ci> FLUX_VALUE </ci>
    </math>
```

```
    <listOfParameters>
      <parameter id="LOWER_BOUND" value="0" units="mmol_per_gDW_per_hr" constant="true"/>
      <parameter id="UPPER_BOUND" value="1000" units="mmol_per_gDW_per_hr" constant="true"/>
      <parameter id="FLUX_VALUE" value="0" units="mmol_per_gDW_per_hr" constant="true"/>
      <parameter id="OBJECTIVE_COEFFICIENT" value="0" units="mmol_per_gDW_per_hr" constant="true"/>
    </listOfParameters>
  </kineticLaw>
</reaction>
<reaction id="R_R272" name="N2-acetyl-L-lysine deacetylase" reversible="false">
  <notes>
    <body xmlns="http://www.w3.org/1999/xhtml">
      <p>GENE_ASSOCIATION: TTC1396</p>
      <p>SUBSYSTEM: Threonine and Lysine Metabolism</p>
      <p>EC Number: 3.5.1.-</p>
      <p>Confidence Level: 1</p>
      <p>AUTHORS: Amino acid metabolism</p>
      <p/>
    </body>
  </notes>
  <listOfReactants>
    <speciesReference species="M_H2O_c" stoichiometry="1"/>
    <speciesReference species="M_N2AC_L_LYS_c" stoichiometry="1"/>
  </listOfReactants>
  <listOfProducts>
    <speciesReference species="M_L_LYS_c" stoichiometry="1"/>
    <speciesReference species="M_AC_c" stoichiometry="1"/>
  </listOfProducts>
  <kineticLaw>
    <math xmlns="http://www.w3.org/1998/Math/MathML">
```

```xml
<ci> FLUX_VALUE </ci>

</math>

<listOfParameters>

<parameter id="LOWER_BOUND" value="0" units="mmol_per_gDW_per_hr" constant="true"/>

<parameter id="UPPER_BOUND" value="1000" units="mmol_per_gDW_per_hr" constant="true"/>

<parameter id="FLUX_VALUE" value="0" units="mmol_per_gDW_per_hr" constant="true"/>

<parameter id="OBJECTIVE_COEFFICIENT" value="0" units="mmol_per_gDW_per_hr" constant="true"/>

</listOfParameters>

</kineticLaw>

</reaction>

<reaction id="R_R273" name="2-Oxoadipate:lipoamde 2-oxidoreductase(decarboxylating and acceptor-succinylating)"
reversible="false">

<notes>

<body xmlns="http://www.w3.org/1999/xhtml">

<p>GENE_ASSOCIATION: TTC1698</p>

<p>SUBSYSTEM: Threonine and Lysine Metabolism</p>

<p>EC Number: 1.2.4.2</p>

<p>Confidence Level: 1</p>

<p>AUTHORS: Amino acid metabolism</p>

<p/>

</body>

</notes>

<listOfReactants>

<speciesReference species="M_ENZN6_LPL_c" stoichiometry="1"/>

<speciesReference species="M_2OADPA_c" stoichiometry="1"/>

</listOfReactants>

<listOfProducts>

<speciesReference species="M_CO2_c" stoichiometry="1"/>

<speciesReference species="M_DHLPL_SUCCT_GLUTRDHLPL_c" stoichiometry="1"/>
```

```
    </listOfProducts>
    <kineticLaw>
      <math xmlns="http://www.w3.org/1998/Math/MathML">
        <ci> FLUX_VALUE </ci>
      </math>
      <listOfParameters>
        <parameter id="LOWER_BOUND" value="0" units="mmol_per_gDW_per_hr" constant="true"/>
        <parameter id="UPPER_BOUND" value="1000" units="mmol_per_gDW_per_hr" constant="true"/>
        <parameter id="FLUX_VALUE" value="0" units="mmol_per_gDW_per_hr" constant="true"/>
        <parameter id="OBJECTIVE_COEFFICIENT" value="0" units="mmol_per_gDW_per_hr" constant="true"/>
      </listOfParameters>
    </kineticLaw>
  </reaction>
  <reaction id="R_R274" name="Glutaryl-CoA:dihydrolipoamide S-succinyltransferase" reversible="true">
    <notes>
      <body xmlns="http://www.w3.org/1999/xhtml">
        <p>GENE_ASSOCIATION: TTC1699</p>
        <p>SUBSYSTEM: Threonine and Lysine Metabolism</p>
        <p>EC Number: 2.3.1.61</p>
        <p>Confidence Level: 1</p>
        <p>AUTHORS: Amino acid metabolism</p>
        <p/>
      </body>
    </notes>
    <listOfReactants>
      <speciesReference species="M_GLUTRCoA_c" stoichiometry="1"/>
      <speciesReference species="M_ENZNE_DHYLPL_c" stoichiometry="1"/>
    </listOfReactants>
    <listOfProducts>
```

```xml
<speciesReference species="M_CoA_c" stoichiometry="1"/>
<speciesReference species="M_DHLPL_SUCCT_GLUTRDHLPL_c" stoichiometry="1"/>
</listOfProducts>
<kineticLaw>
<math xmlns="http://www.w3.org/1998/Math/MathML">
<ci> FLUX_VALUE </ci>
</math>
<listOfParameters>
<parameter id="LOWER_BOUND" value="-1000" units="mmol_per_gDW_per_hr" constant="true"/>
<parameter id="UPPER_BOUND" value="1000" units="mmol_per_gDW_per_hr" constant="true"/>
<parameter id="FLUX_VALUE" value="0" units="mmol_per_gDW_per_hr" constant="true"/>
<parameter id="OBJECTIVE_COEFFICIENT" value="0" units="mmol_per_gDW_per_hr" constant="true"/>
</listOfParameters>
</kineticLaw>
</reaction>
<reaction id="R_R275" name="4-Trimethylammoniobutanal:NAD+ 1-oxidoreductase" reversible="false">
<notes>
<body xmlns="http://www.w3.org/1999/xhtml">
<p>GENE_ASSOCIATION: TTC0513</p>
<p>SUBSYSTEM: Threonine and Lysine Metabolism</p>
<p>EC Number: 1.2.1.3</p>
<p>Confidence Level: 1</p>
<p>AUTHORS: Amino acid metabolism</p>
<p/>
</body>
</notes>
<listOfReactants>
<speciesReference species="M_H2O_c" stoichiometry="1"/>
<speciesReference species="M_NAD_c" stoichiometry="1"/>
```

```xml
<speciesReference species="M_TM_AMBUTAL_c" stoichiometry="1"/>
</listOfReactants>
<listOfProducts>
<speciesReference species="M_H_c" stoichiometry="1"/>
<speciesReference species="M_NADH_c" stoichiometry="1"/>
<speciesReference species="M_TM_AMBUTAT_c" stoichiometry="1"/>
</listOfProducts>
<kineticLaw>
<math xmlns="http://www.w3.org/1998/Math/MathML">
<ci> FLUX_VALUE </ci>
</math>
<listOfParameters>
<parameter id="LOWER_BOUND" value="0" units="mmol_per_gDW_per_hr" constant="true"/>
<parameter id="UPPER_BOUND" value="1000" units="mmol_per_gDW_per_hr" constant="true"/>
<parameter id="FLUX_VALUE" value="0" units="mmol_per_gDW_per_hr" constant="true"/>
<parameter id="OBJECTIVE_COEFFICIENT" value="0" units="mmol_per_gDW_per_hr" constant="true"/>
</listOfParameters>
</kineticLaw>
</reaction>
<reaction id="R_R276" name="1-(5-phospho-D-ribosyl)-ATP:diphosphate phospho-alpha-D-ribosyl-transferase" reversible="true">
<notes>
<body xmlns="http://www.w3.org/1999/xhtml">
<p>GENE_ASSOCIATION: TTC1866</p>
<p>SUBSYSTEM: Histidine Metabolism</p>
<p>EC Number: 2.4.2.17</p>
<p>Confidence Level: 1</p>
<p>AUTHORS: Amino acid metabolism</p>
<p/>
```

```xml
    </body>
  </notes>
  <listOfReactants>
    <speciesReference species="M_PPi_c" stoichiometry="1"/>
    <speciesReference species="M_PRIB_ATP_c" stoichiometry="1"/>
  </listOfReactants>
  <listOfProducts>
    <speciesReference species="M_ATP_c" stoichiometry="1"/>
    <speciesReference species="M_PRPP_c" stoichiometry="1"/>
  </listOfProducts>
  <kineticLaw>
    <math xmlns="http://www.w3.org/1998/Math/MathML">
      <ci> FLUX_VALUE </ci>
    </math>
    <listOfParameters>
      <parameter id="LOWER_BOUND" value="-1000" units="mmol_per_gDW_per_hr" constant="true"/>
      <parameter id="UPPER_BOUND" value="1000" units="mmol_per_gDW_per_hr" constant="true"/>
      <parameter id="FLUX_VALUE" value="0" units="mmol_per_gDW_per_hr" constant="true"/>
      <parameter id="OBJECTIVE_COEFFICIENT" value="0" units="mmol_per_gDW_per_hr" constant="true"/>
    </listOfParameters>
  </kineticLaw>
</reaction>
<reaction id="R_R277" name="Phosphoribosyl-ATP pyrophosphohydrolase" reversible="false">
  <notes>
    <body xmlns="http://www.w3.org/1999/xhtml">
      <p>GENE_ASSOCIATION: TTC1080</p>
      <p>SUBSYSTEM: Histidine Metabolism</p>
      <p>EC Number: 3.6.1.31</p>
      <p>Confidence Level: 1</p>
```

```xml
      <p>AUTHORS: Amino acid metabolism</p>
      <p/>
    </body>
  </notes>
  <listOfReactants>
    <speciesReference species="M_H2O_c" stoichiometry="1"/>
    <speciesReference species="M_PRIB_ATP_c" stoichiometry="1"/>
  </listOfReactants>
  <listOfProducts>
    <speciesReference species="M_PPi_c" stoichiometry="1"/>
    <speciesReference species="M_H_c" stoichiometry="1"/>
    <speciesReference species="M_PRIB_AMP_c" stoichiometry="1"/>
  </listOfProducts>
  <kineticLaw>
    <math xmlns="http://www.w3.org/1998/Math/MathML">
      <ci> FLUX_VALUE </ci>
    </math>
    <listOfParameters>
      <parameter id="LOWER_BOUND" value="0" units="mmol_per_gDW_per_hr" constant="true"/>
      <parameter id="UPPER_BOUND" value="1000" units="mmol_per_gDW_per_hr" constant="true"/>
      <parameter id="FLUX_VALUE" value="0" units="mmol_per_gDW_per_hr" constant="true"/>
      <parameter id="OBJECTIVE_COEFFICIENT" value="0" units="mmol_per_gDW_per_hr" constant="true"/>
    </listOfParameters>
  </kineticLaw>
</reaction>
<reaction id="R_R278" name="1-(5-phospho-D-ribosyl)-AMP 1,6-hydrolase" reversible="false">
  <notes>
    <body xmlns="http://www.w3.org/1999/xhtml">
      <p>GENE_ASSOCIATION: TTC1080</p>
```

```
<p>SUBSYSTEM: Histidine Metabolism</p>

<p>EC Number: 3.5.4.19</p>

<p>Confidence Level: 1</p>

<p>AUTHORS: Amino acid metabolism</p>

<p/>

</body>

</notes>

<listOfReactants>

<speciesReference species="M_H2O_c" stoichiometry="1"/>

<speciesReference species="M_PRIB_AMP_c" stoichiometry="1"/>

</listOfReactants>

<listOfProducts>

<speciesReference species="M_PRINS_AFORMA_PRIBS_IMDZ_CARBA_c" stoichiometry="1"/>

</listOfProducts>

<kineticLaw>

<math xmlns="http://www.w3.org/1998/Math/MathML">

<ci> FLUX_VALUE </ci>

</math>

<listOfParameters>

<parameter id="LOWER_BOUND" value="0" units="mmol_per_gDW_per_hr" constant="true"/>

<parameter id="UPPER_BOUND" value="1000" units="mmol_per_gDW_per_hr" constant="true"/>

<parameter id="FLUX_VALUE" value="0" units="mmol_per_gDW_per_hr" constant="true"/>

<parameter id="OBJECTIVE_COEFFICIENT" value="0" units="mmol_per_gDW_per_hr" constant="true"/>

</listOfParameters>

</kineticLaw>

</reaction>

<reaction id="R_R279" name="N-(5&apos;-Phospho-D-ribosylformimino)-5-amino-1- (5&apos;&apos;-phospho-D-ribosyl)-4-
imidazolecarboxamide ketol-isomerase" reversible="true">

<notes>
```

```
<body xmlns="http://www.w3.org/1999/xhtml">
<p>GENE_ASSOCIATION: TTC0801</p>
<p>SUBSYSTEM: Histidine Metabolism</p>
<p>EC Number: 5.3.1.16</p>
<p>Confidence Level: 1</p>
<p>AUTHORS: Amino acid metabolism</p>
<p/>
</body>
</notes>
<listOfReactants>
<speciesReference species="M_PRINS_AFORMA_PRIBS_IMDZ_CARBA_c" stoichiometry="1"/>
</listOfReactants>
<listOfProducts>
<speciesReference species="M_PRINSFORMA_PRIBS_IMDZ_CARBA_c" stoichiometry="1"/>
</listOfProducts>
<kineticLaw>
<math xmlns="http://www.w3.org/1998/Math/MathML">
<ci> FLUX_VALUE </ci>
</math>
<listOfParameters>
<parameter id="LOWER_BOUND" value="-1000" units="mmol_per_gDW_per_hr" constant="true"/>
<parameter id="UPPER_BOUND" value="1000" units="mmol_per_gDW_per_hr" constant="true"/>
<parameter id="FLUX_VALUE" value="0" units="mmol_per_gDW_per_hr" constant="true"/>
<parameter id="OBJECTIVE_COEFFICIENT" value="0" units="mmol_per_gDW_per_hr" constant="true"/>
</listOfParameters>
</kineticLaw>
</reaction>
<reaction id="R_R280" name="Imidazole-glycerol-3-phosphate synthase" reversible="false">
<notes>
```

```
  <body xmlns="http://www.w3.org/1999/xhtml">
    <p>GENE_ASSOCIATION: (TTC0062 and TTC1079)</p>
    <p>SUBSYSTEM: Histidine Metabolism</p>
    <p>EC Number: 2.4.2.- or 4.1.3.-</p>
    <p>Confidence Level: 1</p>
    <p>AUTHORS: Amino acid metabolism</p>
    <p/>
  </body>
</notes>
<listOfReactants>
  <speciesReference species="M_L_GLN_c" stoichiometry="1"/>
  <speciesReference species="M_PRINSFORMA_PRIBS_IMDZ_CARBA_c" stoichiometry="1"/>
</listOfReactants>
<listOfProducts>
  <speciesReference species="M_L_GLU_c" stoichiometry="1"/>
  <speciesReference species="M_H_c" stoichiometry="1"/>
  <speciesReference species="M_AICAR_c" stoichiometry="1"/>
  <speciesReference species="M_ERY_IMDZ_GLYCP_c" stoichiometry="1"/>
</listOfProducts>
<kineticLaw>
  <math xmlns="http://www.w3.org/1998/Math/MathML">
    <ci> FLUX_VALUE </ci>
  </math>
  <listOfParameters>
    <parameter id="LOWER_BOUND" value="0" units="mmol_per_gDW_per_hr" constant="true"/>
    <parameter id="UPPER_BOUND" value="1000" units="mmol_per_gDW_per_hr" constant="true"/>
    <parameter id="FLUX_VALUE" value="0" units="mmol_per_gDW_per_hr" constant="true"/>
    <parameter id="OBJECTIVE_COEFFICIENT" value="0" units="mmol_per_gDW_per_hr" constant="true"/>
  </listOfParameters>
```

```
    </kineticLaw>
  </reaction>
  <reaction id="R_R281" name="D-erythro-1-(Imidazol-4-yl)glycerol 3-phosphate hydro-lyase" reversible="false">
    <notes>
      <body xmlns="http://www.w3.org/1999/xhtml">
        <p>GENE_ASSOCIATION: TTC0061</p>
        <p>SUBSYSTEM: Histidine Metabolism</p>
        <p>EC Number: 4.2.1.19</p>
        <p>Confidence Level: 1</p>
        <p>AUTHORS: Amino acid metabolism</p>
        <p/>
      </body>
    </notes>
    <listOfReactants>
      <speciesReference species="M_ERY_IMDZ_GLYCP_c" stoichiometry="1"/>
    </listOfReactants>
    <listOfProducts>
      <speciesReference species="M_H2O_c" stoichiometry="1"/>
      <speciesReference species="M_IMDZ_ACLP_c" stoichiometry="1"/>
    </listOfProducts>
    <kineticLaw>
      <math xmlns="http://www.w3.org/1998/Math/MathML">
        <ci> FLUX_VALUE </ci>
      </math>
      <listOfParameters>
        <parameter id="LOWER_BOUND" value="0" units="mmol_per_gDW_per_hr" constant="true"/>
        <parameter id="UPPER_BOUND" value="1000" units="mmol_per_gDW_per_hr" constant="true"/>
        <parameter id="FLUX_VALUE" value="0" units="mmol_per_gDW_per_hr" constant="true"/>
        <parameter id="OBJECTIVE_COEFFICIENT" value="0" units="mmol_per_gDW_per_hr" constant="true"/>
```

```xml
    </listOfParameters>
  </kineticLaw>
</reaction>
<reaction id="R_R282" name="5-Amino-2-oxopentanoate:2-oxoglutarate aminotransferase" reversible="true">
  <notes>
    <body xmlns="http://www.w3.org/1999/xhtml">
      <p>GENE_ASSOCIATION: (TTC0060 or TT_P0022)</p>
      <p>SUBSYSTEM: Histidine Metabolism</p>
      <p>EC Number: 2.6.1.9</p>
      <p>Confidence Level: 1</p>
      <p>AUTHORS: Amino acid metabolism</p>
      <p/>
    </body>
  </notes>
  <listOfReactants>
    <speciesReference species="M_AKG_c" stoichiometry="1"/>
    <speciesReference species="M_HISDPi_c" stoichiometry="1"/>
  </listOfReactants>
  <listOfProducts>
    <speciesReference species="M_L_GLU_c" stoichiometry="1"/>
    <speciesReference species="M_IMDZ_ACLP_c" stoichiometry="1"/>
  </listOfProducts>
  <kineticLaw>
    <math xmlns="http://www.w3.org/1998/Math/MathML">
      <ci> FLUX_VALUE </ci>
    </math>
    <listOfParameters>
      <parameter id="LOWER_BOUND" value="-1000" units="mmol_per_gDW_per_hr" constant="true"/>
      <parameter id="UPPER_BOUND" value="1000" units="mmol_per_gDW_per_hr" constant="true"/>
```

```
      <parameter id="FLUX_VALUE" value="0" units="mmol_per_gDW_per_hr" constant="true"/>
      <parameter id="OBJECTIVE_COEFFICIENT" value="0" units="mmol_per_gDW_per_hr" constant="true"/>
    </listOfParameters>
  </kineticLaw>
</reaction>
<reaction id="R_R283" name="L-Histidinol-phosphate phosphohydrolase" reversible="false">
  <notes>
    <body xmlns="http://www.w3.org/1999/xhtml">
      <p>GENE_ASSOCIATION: TTC1652</p>
      <p>SUBSYSTEM: Histidine Metabolism</p>
      <p>EC Number: 3.1.3.15</p>
      <p>Confidence Level: 1</p>
      <p>AUTHORS: Amino acid metabolism</p>
      <p/>
    </body>
  </notes>
  <listOfReactants>
    <speciesReference species="M_H2O_c" stoichiometry="1"/>
    <speciesReference species="M_HISDPi_c" stoichiometry="1"/>
  </listOfReactants>
  <listOfProducts>
    <speciesReference species="M_Pi_c" stoichiometry="1"/>
    <speciesReference species="M_L_HISN_c" stoichiometry="1"/>
  </listOfProducts>
  <kineticLaw>
    <math xmlns="http://www.w3.org/1998/Math/MathML">
      <ci> FLUX_VALUE </ci>
    </math>
    <listOfParameters>
```

```
      <parameter id="LOWER_BOUND" value="0" units="mmol_per_gDW_per_hr" constant="true"/>
      <parameter id="UPPER_BOUND" value="1000" units="mmol_per_gDW_per_hr" constant="true"/>
      <parameter id="FLUX_VALUE" value="0" units="mmol_per_gDW_per_hr" constant="true"/>
      <parameter id="OBJECTIVE_COEFFICIENT" value="0" units="mmol_per_gDW_per_hr" constant="true"/>
    </listOfParameters>
  </kineticLaw>
</reaction>
<reaction id="R_R284" name="L-Histidinol:NAD+ oxidoreductase" reversible="false">
  <notes>
    <body xmlns="http://www.w3.org/1999/xhtml">
      <p>GENE_ASSOCIATION: TTC0370</p>
      <p>SUBSYSTEM: Histidine Metabolism</p>
      <p>EC Number: 1.1.1.23</p>
      <p>Confidence Level: 1</p>
      <p>AUTHORS: Amino acid metabolism</p>
      <p/>
    </body>
  </notes>
  <listOfReactants>
    <speciesReference species="M_H2O_c" stoichiometry="1"/>
    <speciesReference species="M_NAD_c" stoichiometry="2"/>
    <speciesReference species="M_L_HISN_c" stoichiometry="1"/>
  </listOfReactants>
  <listOfProducts>
    <speciesReference species="M_L_HIS_c" stoichiometry="1"/>
    <speciesReference species="M_H_c" stoichiometry="3"/>
    <speciesReference species="M_NADH_c" stoichiometry="2"/>
  </listOfProducts>
  <kineticLaw>
```

```
<math xmlns="http://www.w3.org/1998/Math/MathML">
<ci> FLUX_VALUE </ci>
</math>
<listOfParameters>
<parameter id="LOWER_BOUND" value="0" units="mmol_per_gDW_per_hr" constant="true"/>
<parameter id="UPPER_BOUND" value="1000" units="mmol_per_gDW_per_hr" constant="true"/>
<parameter id="FLUX_VALUE" value="0" units="mmol_per_gDW_per_hr" constant="true"/>
<parameter id="OBJECTIVE_COEFFICIENT" value="0" units="mmol_per_gDW_per_hr" constant="true"/>
</listOfParameters>
</kineticLaw>
</reaction>
<reaction id="R_R285" name="L-histidinal:NAD+ oxidoreductase" reversible="false">
<notes>
<body xmlns="http://www.w3.org/1999/xhtml">
<p>GENE_ASSOCIATION: TTC0370</p>
<p>SUBSYSTEM: Histidine Metabolism</p>
<p>EC Number: 1.1.1.23</p>
<p>Confidence Level: 1</p>
<p>AUTHORS: Amino acid metabolism</p>
<p/>
</body>
</notes>
<listOfReactants>
<speciesReference species="M_H2O_c" stoichiometry="1"/>
<speciesReference species="M_NAD_c" stoichiometry="1"/>
<speciesReference species="M_L_HISDAL_c" stoichiometry="1"/>
</listOfReactants>
<listOfProducts>
<speciesReference species="M_L_HIS_c" stoichiometry="1"/>
```

```
    <speciesReference species="M_H_c" stoichiometry="1"/>
    <speciesReference species="M_NADH_c" stoichiometry="1"/>
  </listOfProducts>
  <kineticLaw>
    <math xmlns="http://www.w3.org/1998/Math/MathML">
      <ci> FLUX_VALUE </ci>
    </math>
    <listOfParameters>
      <parameter id="LOWER_BOUND" value="0" units="mmol_per_gDW_per_hr" constant="true"/>
      <parameter id="UPPER_BOUND" value="1000" units="mmol_per_gDW_per_hr" constant="true"/>
      <parameter id="FLUX_VALUE" value="0" units="mmol_per_gDW_per_hr" constant="true"/>
      <parameter id="OBJECTIVE_COEFFICIENT" value="0" units="mmol_per_gDW_per_hr" constant="true"/>
    </listOfParameters>
  </kineticLaw>
</reaction>
<reaction id="R_R286" name="Imidazole acetaldehyde:NAD+ oxidoreductase" reversible="false">
  <notes>
    <body xmlns="http://www.w3.org/1999/xhtml">
      <p>GENE_ASSOCIATION: TTC0513</p>
      <p>SUBSYSTEM: Histidine Metabolism</p>
      <p>EC Number: 1.2.1.3</p>
      <p>Confidence Level: 1</p>
      <p>AUTHORS: Amino acid metabolism</p>
      <p/>
    </body>
  </notes>
  <listOfReactants>
    <speciesReference species="M_H2O_c" stoichiometry="1"/>
    <speciesReference species="M_NAD_c" stoichiometry="1"/>
```

```
    <speciesReference species="M_IMDZ_AALD_c" stoichiometry="1"/>
  </listOfReactants>
  <listOfProducts>
    <speciesReference species="M_H_c" stoichiometry="1"/>
    <speciesReference species="M_NADH_c" stoichiometry="1"/>
    <speciesReference species="M_IMDZ_AC_c" stoichiometry="1"/>
  </listOfProducts>
  <kineticLaw>
    <math xmlns="http://www.w3.org/1998/Math/MathML">
      <ci> FLUX_VALUE </ci>
    </math>
    <listOfParameters>
      <parameter id="LOWER_BOUND" value="0" units="mmol_per_gDW_per_hr" constant="true"/>
      <parameter id="UPPER_BOUND" value="1000" units="mmol_per_gDW_per_hr" constant="true"/>
      <parameter id="FLUX_VALUE" value="0" units="mmol_per_gDW_per_hr" constant="true"/>
      <parameter id="OBJECTIVE_COEFFICIENT" value="0" units="mmol_per_gDW_per_hr" constant="true"/>
    </listOfParameters>
  </kineticLaw>
</reaction>
<reaction id="R_R287" name="p-hydroxyphenylpyruvate oxidase" reversible="true">
  <notes>
    <body xmlns="http://www.w3.org/1999/xhtml">
      <p>GENE_ASSOCIATION: </p>
      <p>SUBSYSTEM: Tyrosine, Tryptophan, and Phenylalanine Metabolism</p>
      <p>EC Number: 1.2.3.13</p>
      <p>Confidence Level: 1</p>
      <p>AUTHORS: Amino acid metabolism</p>
      <p/>
    </body>
```

```
</notes>
<listOfReactants>
  <speciesReference species="M_H_c" stoichiometry="2"/>
  <speciesReference species="M_O2_c" stoichiometry="1"/>
  <speciesReference species="M_HYPh_PYR_c" stoichiometry="1"/>
</listOfReactants>
<listOfProducts>
  <speciesReference species="M_H2O_c" stoichiometry="1"/>
  <speciesReference species="M_CO2_c" stoichiometry="1"/>
  <speciesReference species="M_HY_PhAC_c" stoichiometry="1"/>
</listOfProducts>
<kineticLaw>
  <math xmlns="http://www.w3.org/1998/Math/MathML">
    <ci> FLUX_VALUE </ci>
  </math>
  <listOfParameters>
    <parameter id="LOWER_BOUND" value="-1000" units="mmol_per_gDW_per_hr" constant="true"/>
    <parameter id="UPPER_BOUND" value="1000" units="mmol_per_gDW_per_hr" constant="true"/>
    <parameter id="FLUX_VALUE" value="0" units="mmol_per_gDW_per_hr" constant="true"/>
    <parameter id="OBJECTIVE_COEFFICIENT" value="0" units="mmol_per_gDW_per_hr" constant="true"/>
  </listOfParameters>
</kineticLaw>
</reaction>
<reaction id="R_R288" name="4-hydroxyphenylacetate,NADH:oxygen oxidoreductase (3-hydroxylating)" reversible="true">
<notes>
  <body xmlns="http://www.w3.org/1999/xhtml">
    <p>GENE_ASSOCIATION: TTC0594</p>
    <p>SUBSYSTEM: Tyrosine, Tryptophan, and Phenylalanine Metabolism</p>
    <p>EC Number: 1.14.14.9</p>
```

```
      <p>Confidence Level: 1</p>
      <p>AUTHORS: Amino acid metabolism</p>
      <p/>
    </body>
  </notes>
  <listOfReactants>
    <speciesReference species="M_H_c" stoichiometry="1"/>
    <speciesReference species="M_NADH_c" stoichiometry="1"/>
    <speciesReference species="M_O2_c" stoichiometry="1"/>
    <speciesReference species="M_HY_PhAC_c" stoichiometry="1"/>
  </listOfReactants>
  <listOfProducts>
    <speciesReference species="M_H2O_c" stoichiometry="1"/>
    <speciesReference species="M_NAD_c" stoichiometry="1"/>
    <speciesReference species="M_dHY_PhAC_c" stoichiometry="1"/>
  </listOfProducts>
  <kineticLaw>
    <math xmlns="http://www.w3.org/1998/Math/MathML">
      <ci> FLUX_VALUE </ci>
    </math>
    <listOfParameters>
      <parameter id="LOWER_BOUND" value="-1000" units="mmol_per_gDW_per_hr" constant="true"/>
      <parameter id="UPPER_BOUND" value="1000" units="mmol_per_gDW_per_hr" constant="true"/>
      <parameter id="FLUX_VALUE" value="0" units="mmol_per_gDW_per_hr" constant="true"/>
      <parameter id="OBJECTIVE_COEFFICIENT" value="0" units="mmol_per_gDW_per_hr" constant="true"/>
    </listOfParameters>
  </kineticLaw>
</reaction>
<reaction id="R_R289" name="3,4-dihydroxyphenylacetate dioxygenase" reversible="false">
```

```
<notes>
  <body xmlns="http://www.w3.org/1999/xhtml">
    <p>GENE_ASSOCIATION: </p>
    <p>SUBSYSTEM: Tyrosine, Tryptophan, and Phenylalanine Metabolism</p>
    <p>EC Number: 1.13.11.15</p>
    <p>Confidence Level: 1</p>
    <p>AUTHORS: Amino acid metabolism</p>
    <p/>
  </body>
</notes>
<listOfReactants>
  <speciesReference species="M_O2_c" stoichiometry="1"/>
  <speciesReference species="M_dHY_PhAC_c" stoichiometry="1"/>
</listOfReactants>
<listOfProducts>
  <speciesReference species="M_H_c" stoichiometry="1"/>
  <speciesReference species="M_2HY_5CARBMMUC_SA_c" stoichiometry="1"/>
</listOfProducts>
<kineticLaw>
  <math xmlns="http://www.w3.org/1998/Math/MathML">
    <ci> FLUX_VALUE </ci>
  </math>
  <listOfParameters>
    <parameter id="LOWER_BOUND" value="0" units="mmol_per_gDW_per_hr" constant="true"/>
    <parameter id="UPPER_BOUND" value="1000" units="mmol_per_gDW_per_hr" constant="true"/>
    <parameter id="FLUX_VALUE" value="0" units="mmol_per_gDW_per_hr" constant="true"/>
    <parameter id="OBJECTIVE_COEFFICIENT" value="0" units="mmol_per_gDW_per_hr" constant="true"/>
  </listOfParameters>
</kineticLaw>
```

```
</reaction>
<reaction id="R_R290" name="5-carboxymethyl-2-hydroxymuconic-semialdehyde:NAD+ oxidoreductase" reversible="true">
 <notes>
  <body xmlns="http://www.w3.org/1999/xhtml">
   <p>GENE_ASSOCIATION: TTC0593</p>
   <p>SUBSYSTEM: Tyrosine, Tryptophan, and Phenylalanine Metabolism</p>
   <p>EC Number: 1.2.1.60</p>
   <p>Confidence Level: 1</p>
   <p>AUTHORS: Amino acid metabolism</p>
   <p/>
  </body>
 </notes>
 <listOfReactants>
  <speciesReference species="M_H2O_c" stoichiometry="1"/>
  <speciesReference species="M_NAD_c" stoichiometry="1"/>
  <speciesReference species="M_2HY_5CARBMMUC_SA_c" stoichiometry="1"/>
 </listOfReactants>
 <listOfProducts>
  <speciesReference species="M_H_c" stoichiometry="1"/>
  <speciesReference species="M_NADH_c" stoichiometry="1"/>
  <speciesReference species="M_CARBM_HYMUC_c" stoichiometry="1"/>
 </listOfProducts>
 <kineticLaw>
  <math xmlns="http://www.w3.org/1998/Math/MathML">
   <ci> FLUX_VALUE </ci>
  </math>
  <listOfParameters>
   <parameter id="LOWER_BOUND" value="-1000" units="mmol_per_gDW_per_hr" constant="true"/>
   <parameter id="UPPER_BOUND" value="1000" units="mmol_per_gDW_per_hr" constant="true"/>
```

```
      <parameter id="FLUX_VALUE" value="0" units="mmol_per_gDW_per_hr" constant="true"/>
      <parameter id="OBJECTIVE_COEFFICIENT" value="0" units="mmol_per_gDW_per_hr" constant="true"/>
    </listOfParameters>
  </kineticLaw>
</reaction>
<reaction id="R_R291" name="5-carboxymethyl-2-hydroxymuconate isomerase" reversible="true">
  <notes>
    <body xmlns="http://www.w3.org/1999/xhtml">
      <p>GENE_ASSOCIATION: </p>
      <p>SUBSYSTEM: Tyrosine, Tryptophan, and Phenylalanine Metabolism</p>
      <p>EC Number: 5.3.3.10</p>
      <p>Confidence Level: 1</p>
      <p>AUTHORS: Amino acid metabolism</p>
      <p/>
    </body>
  </notes>
  <listOfReactants>
    <speciesReference species="M_CARBM_HYMUC_c" stoichiometry="1"/>
  </listOfReactants>
  <listOfProducts>
    <speciesReference species="M_CO2_c" stoichiometry="1"/>
    <speciesReference species="M_CRBX_OHEPTEDA_c" stoichiometry="1"/>
  </listOfProducts>
  <kineticLaw>
    <math xmlns="http://www.w3.org/1998/Math/MathML">
      <ci> FLUX_VALUE </ci>
    </math>
    <listOfParameters>
      <parameter id="LOWER_BOUND" value="-1000" units="mmol_per_gDW_per_hr" constant="true"/>
```

```
      <parameter id="UPPER_BOUND" value="1000" units="mmol_per_gDW_per_hr" constant="true"/>
      <parameter id="FLUX_VALUE" value="0" units="mmol_per_gDW_per_hr" constant="true"/>
      <parameter id="OBJECTIVE_COEFFICIENT" value="0" units="mmol_per_gDW_per_hr" constant="true"/>
    </listOfParameters>
  </kineticLaw>
</reaction>
<reaction id="R_R292" name="5-oxopent-3-ene-1,2,5-tricarboxylate carboxy-lyase" reversible="false">
  <notes>
    <body xmlns="http://www.w3.org/1999/xhtml">
      <p>GENE_ASSOCIATION: TTC0592</p>
      <p>SUBSYSTEM: Tyrosine, Tryptophan, and Phenylalanine Metabolism</p>
      <p>EC Number: 4.1.1.68</p>
      <p>Confidence Level: 1</p>
      <p>AUTHORS: Amino acid metabolism</p>
      <p/>
    </body>
  </notes>
  <listOfReactants>
    <speciesReference species="M_CRBX_OHEPTEDA_c" stoichiometry="1"/>
  </listOfReactants>
  <listOfProducts>
    <speciesReference species="M_CO2_c" stoichiometry="1"/>
    <speciesReference species="M_HYHEPTDENA_c" stoichiometry="1"/>
  </listOfProducts>
  <kineticLaw>
    <math xmlns="http://www.w3.org/1998/Math/MathML">
      <ci> FLUX_VALUE </ci>
    </math>
    <listOfParameters>
```

```
      <parameter id="LOWER_BOUND" value="0" units="mmol_per_gDW_per_hr" constant="true"/>
      <parameter id="UPPER_BOUND" value="1000" units="mmol_per_gDW_per_hr" constant="true"/>
      <parameter id="FLUX_VALUE" value="0" units="mmol_per_gDW_per_hr" constant="true"/>
      <parameter id="OBJECTIVE_COEFFICIENT" value="0" units="mmol_per_gDW_per_hr" constant="true"/>
    </listOfParameters>
  </kineticLaw>
</reaction>
<reaction id="R_R293" name="2-hydroxyhepta-2,4-dienedioate hydratase" reversible="true">
  <notes>
    <body xmlns="http://www.w3.org/1999/xhtml">
      <p>GENE_ASSOCIATION: </p>
      <p>SUBSYSTEM: Tyrosine, Tryptophan, and Phenylalanine Metabolism</p>
      <p>EC Number: 5.3.3.-</p>
      <p>Confidence Level: 1</p>
      <p>AUTHORS: Amino acid metabolism</p>
      <p/>
    </body>
  </notes>
  <listOfReactants>
    <speciesReference species="M_OHEPEDA_c" stoichiometry="1"/>
  </listOfReactants>
  <listOfProducts>
    <speciesReference species="M_H2O_c" stoichiometry="1"/>
    <speciesReference species="M_HYHEPTDENA_c" stoichiometry="1"/>
  </listOfProducts>
  <kineticLaw>
    <math xmlns="http://www.w3.org/1998/Math/MathML">
      <ci> FLUX_VALUE </ci>
    </math>
```

```
<listOfParameters>
  <parameter id="LOWER_BOUND" value="-1000" units="mmol_per_gDW_per_hr" constant="true"/>
  <parameter id="UPPER_BOUND" value="1000" units="mmol_per_gDW_per_hr" constant="true"/>
  <parameter id="FLUX_VALUE" value="0" units="mmol_per_gDW_per_hr" constant="true"/>
  <parameter id="OBJECTIVE_COEFFICIENT" value="0" units="mmol_per_gDW_per_hr" constant="true"/>
</listOfParameters>
</kineticLaw>
</reaction>
<reaction id="R_R294" name="4-hydroxy-2-ketopimelate aldolase" reversible="false">
<notes>
  <body xmlns="http://www.w3.org/1999/xhtml">
    <p>GENE_ASSOCIATION: </p>
    <p>SUBSYSTEM: Tyrosine, Tryptophan, and Phenylalanine Metabolism</p>
    <p>EC Number: 4.1.2.-</p>
    <p>Confidence Level: 1</p>
    <p>AUTHORS: Amino acid metabolism</p>
    <p/>
  </body>
</notes>
<listOfReactants>
  <speciesReference species="M_OHEPEDA_c" stoichiometry="1"/>
</listOfReactants>
<listOfProducts>
  <speciesReference species="M_PYR_c" stoichiometry="1"/>
  <speciesReference species="M_SUCC_SA_c" stoichiometry="1"/>
</listOfProducts>
<kineticLaw>
  <math xmlns="http://www.w3.org/1998/Math/MathML">
    <ci> FLUX_VALUE </ci>
```

```xml
</math>
<listOfParameters>
<parameter id="LOWER_BOUND" value="0" units="mmol_per_gDW_per_hr" constant="true"/>
<parameter id="UPPER_BOUND" value="1000" units="mmol_per_gDW_per_hr" constant="true"/>
<parameter id="FLUX_VALUE" value="0" units="mmol_per_gDW_per_hr" constant="true"/>
<parameter id="OBJECTIVE_COEFFICIENT" value="0" units="mmol_per_gDW_per_hr" constant="true"/>
</listOfParameters>
</kineticLaw>
</reaction>
<reaction id="R_R295" name="L-tyrosine:2-oxoglutarate aminotransferase" reversible="true">
<notes>
<body xmlns="http://www.w3.org/1999/xhtml">
<p>GENE_ASSOCIATION: TTC1960</p>
<p>SUBSYSTEM: Tyrosine, Tryptophan, and Phenylalanine Metabolism</p>
<p>EC Number: 2.6.1.1</p>
<p>Confidence Level: 1</p>
<p>AUTHORS: Amino acid metabolism</p>
<p/>
</body>
</notes>
<listOfReactants>
<speciesReference species="M_L_TYR_c" stoichiometry="1"/>
<speciesReference species="M_AKG_c" stoichiometry="1"/>
</listOfReactants>
<listOfProducts>
<speciesReference species="M_L_GLU_c" stoichiometry="1"/>
<speciesReference species="M_HYPh_PYR_c" stoichiometry="1"/>
</listOfProducts>
<kineticLaw>
```

```
<math xmlns="http://www.w3.org/1998/Math/MathML">
  <ci> FLUX_VALUE </ci>
</math>
<listOfParameters>
  <parameter id="LOWER_BOUND" value="-1000" units="mmol_per_gDW_per_hr" constant="true"/>
  <parameter id="UPPER_BOUND" value="1000" units="mmol_per_gDW_per_hr" constant="true"/>
  <parameter id="FLUX_VALUE" value="0" units="mmol_per_gDW_per_hr" constant="true"/>
  <parameter id="OBJECTIVE_COEFFICIENT" value="0" units="mmol_per_gDW_per_hr" constant="true"/>
</listOfParameters>
</kineticLaw>
</reaction>
<reaction id="R_R296" name="Phenylacetate:CoA ligase" reversible="false">
 <notes>
  <body xmlns="http://www.w3.org/1999/xhtml">
   <p>GENE_ASSOCIATION: TTC0602</p>
   <p>SUBSYSTEM: Alternate Carbon Metabolism</p>
   <p>EC Number: 6.2.1.30</p>
   <p>Confidence Level: 1</p>
   <p>AUTHORS: Carbohydrate metabolism</p>
   <p/>
  </body>
 </notes>
 <listOfReactants>
  <speciesReference species="M_ATP_c" stoichiometry="1"/>
  <speciesReference species="M_CoA_c" stoichiometry="1"/>
  <speciesReference species="M_PhAC_c" stoichiometry="1"/>
 </listOfReactants>
 <listOfProducts>
  <speciesReference species="M_PPi_c" stoichiometry="1"/>
```

```xml
<speciesReference species="M_AMP_c" stoichiometry="1"/>
<speciesReference species="M_PhACoA_c" stoichiometry="1"/>
</listOfProducts>
<kineticLaw>
<math xmlns="http://www.w3.org/1998/Math/MathML">
<ci> FLUX_VALUE </ci>
</math>
<listOfParameters>
<parameter id="LOWER_BOUND" value="0" units="mmol_per_gDW_per_hr" constant="true"/>
<parameter id="UPPER_BOUND" value="1000" units="mmol_per_gDW_per_hr" constant="true"/>
<parameter id="FLUX_VALUE" value="0" units="mmol_per_gDW_per_hr" constant="true"/>
<parameter id="OBJECTIVE_COEFFICIENT" value="0" units="mmol_per_gDW_per_hr" constant="true"/>
</listOfParameters>
</kineticLaw>
</reaction>
<reaction id="R_R297" name="L-Phenylalanine:2-oxoglutarate aminotransferase" reversible="true">
<notes>
<body xmlns="http://www.w3.org/1999/xhtml">
<p>GENE_ASSOCIATION: TTC1960</p>
<p>SUBSYSTEM: Tyrosine, Tryptophan, and Phenylalanine Metabolism</p>
<p>EC Number: 2.6.1.1</p>
<p>Confidence Level: 1</p>
<p>AUTHORS: Amino acid metabolism</p>
<p/>
</body>
</notes>
<listOfReactants>
<speciesReference species="M_L_PHE_c" stoichiometry="1"/>
<speciesReference species="M_AKG_c" stoichiometry="1"/>
```

```
  </listOfReactants>
  <listOfProducts>
    <speciesReference species="M_L_GLU_c" stoichiometry="1"/>
    <speciesReference species="M_PPYR_c" stoichiometry="1"/>
  </listOfProducts>
  <kineticLaw>
    <math xmlns="http://www.w3.org/1998/Math/MathML">
      <ci> FLUX_VALUE </ci>
    </math>
    <listOfParameters>
      <parameter id="LOWER_BOUND" value="-1000" units="mmol_per_gDW_per_hr" constant="true"/>
      <parameter id="UPPER_BOUND" value="1000" units="mmol_per_gDW_per_hr" constant="true"/>
      <parameter id="FLUX_VALUE" value="0" units="mmol_per_gDW_per_hr" constant="true"/>
      <parameter id="OBJECTIVE_COEFFICIENT" value="0" units="mmol_per_gDW_per_hr" constant="true"/>
    </listOfParameters>
  </kineticLaw>
</reaction>
<reaction id="R_R298" name="Phenylpyruvate decarboxylase" reversible="false">
  <notes>
    <body xmlns="http://www.w3.org/1999/xhtml">
      <p>GENE_ASSOCIATION: </p>
      <p>SUBSYSTEM: Tyrosine, Tryptophan, and Phenylalanine Metabolism</p>
      <p>EC Number: 4.1.1.43</p>
      <p>Confidence Level: 1</p>
      <p>AUTHORS: Amino acid metabolism</p>
      <p/>
    </body>
  </notes>
  <listOfReactants>
```

```xml
    <speciesReference species="M_H_c" stoichiometry="1"/>
    <speciesReference species="M_PPYR_c" stoichiometry="1"/>
  </listOfReactants>
  <listOfProducts>
    <speciesReference species="M_CO2_c" stoichiometry="1"/>
    <speciesReference species="M_PhALD_c" stoichiometry="1"/>
  </listOfProducts>
  <kineticLaw>
    <math xmlns="http://www.w3.org/1998/Math/MathML">
      <ci> FLUX_VALUE </ci>
    </math>
    <listOfParameters>
      <parameter id="LOWER_BOUND" value="0" units="mmol_per_gDW_per_hr" constant="true"/>
      <parameter id="UPPER_BOUND" value="1000" units="mmol_per_gDW_per_hr" constant="true"/>
      <parameter id="FLUX_VALUE" value="0" units="mmol_per_gDW_per_hr" constant="true"/>
      <parameter id="OBJECTIVE_COEFFICIENT" value="0" units="mmol_per_gDW_per_hr" constant="true"/>
    </listOfParameters>
  </kineticLaw>
</reaction>
<reaction id="R_R299" name="Phenylacetaldehyde dehydrogease" reversible="false">
  <notes>
    <body xmlns="http://www.w3.org/1999/xhtml">
      <p>GENE_ASSOCIATION: </p>
      <p>SUBSYSTEM: Tyrosine, Tryptophan, and Phenylalanine Metabolism</p>
      <p>EC Number: 1.2.1.39</p>
      <p>Confidence Level: 1</p>
      <p>AUTHORS: Amino acid metabolism</p>
      <p/>
    </body>
```

```
  </notes>
  <listOfReactants>
    <speciesReference species="M_H2O_c" stoichiometry="1"/>
    <speciesReference species="M_NAD_c" stoichiometry="1"/>
    <speciesReference species="M_PhALD_c" stoichiometry="1"/>
  </listOfReactants>
  <listOfProducts>
    <speciesReference species="M_H_c" stoichiometry="2"/>
    <speciesReference species="M_NADH_c" stoichiometry="1"/>
    <speciesReference species="M_PhAC_c" stoichiometry="1"/>
  </listOfProducts>
  <kineticLaw>
    <math xmlns="http://www.w3.org/1998/Math/MathML">
      <ci> FLUX_VALUE </ci>
    </math>
    <listOfParameters>
      <parameter id="LOWER_BOUND" value="0" units="mmol_per_gDW_per_hr" constant="true"/>
      <parameter id="UPPER_BOUND" value="1000" units="mmol_per_gDW_per_hr" constant="true"/>
      <parameter id="FLUX_VALUE" value="0" units="mmol_per_gDW_per_hr" constant="true"/>
      <parameter id="OBJECTIVE_COEFFICIENT" value="0" units="mmol_per_gDW_per_hr" constant="true"/>
    </listOfParameters>
  </kineticLaw>
</reaction>
<reaction id="R_R300" name="phenylacetate-CoA oxygenase" reversible="false">
  <notes>
    <body xmlns="http://www.w3.org/1999/xhtml">
      <p>GENE_ASSOCIATION: TTC0608</p>
      <p>SUBSYSTEM: Tyrosine, Tryptophan, and Phenylalanine Metabolism</p>
      <p>EC Number: 1.14.13.149</p>
```

```
<p>Confidence Level: 1</p>
<p>AUTHORS: Amino acid metabolism</p>
<p/>
</body>
</notes>
<listOfReactants>
<speciesReference species="M_H_c" stoichiometry="1"/>
<speciesReference species="M_NADPH_c" stoichiometry="1"/>
<speciesReference species="M_O2_c" stoichiometry="1"/>
<speciesReference species="M_PhACoA_c" stoichiometry="1"/>
</listOfReactants>
<listOfProducts>
<speciesReference species="M_H2O_c" stoichiometry="1"/>
<speciesReference species="M_NADP_c" stoichiometry="1"/>
<speciesReference species="M_dhPhACoA_c" stoichiometry="1"/>
</listOfProducts>
<kineticLaw>
<math xmlns="http://www.w3.org/1998/Math/MathML">
<ci> FLUX_VALUE </ci>
</math>
<listOfParameters>
<parameter id="LOWER_BOUND" value="0" units="mmol_per_gDW_per_hr" constant="true"/>
<parameter id="UPPER_BOUND" value="1000" units="mmol_per_gDW_per_hr" constant="true"/>
<parameter id="FLUX_VALUE" value="0" units="mmol_per_gDW_per_hr" constant="true"/>
<parameter id="OBJECTIVE_COEFFICIENT" value="0" units="mmol_per_gDW_per_hr" constant="true"/>
</listOfParameters>
</kineticLaw>
</reaction>
<reaction id="R_R301" name="1,2-epoxyphenylacetyl-CoA isomerase" reversible="true">
```

```
<notes>
  <body xmlns="http://www.w3.org/1999/xhtml">
    <p>GENE_ASSOCIATION: TTC1697</p>
    <p>SUBSYSTEM: Tyrosine, Tryptophan, and Phenylalanine Metabolism</p>
    <p>EC Number: 5.3.3.18</p>
    <p>Confidence Level: 1</p>
    <p>AUTHORS: Amino acid metabolism</p>
    <p/>
  </body>
</notes>
<listOfReactants>
  <speciesReference species="M_dhPhAC_c" stoichiometry="1"/>
</listOfReactants>
<listOfProducts>
  <speciesReference species="M_OPACCoA_c" stoichiometry="1"/>
</listOfProducts>
<kineticLaw>
  <math xmlns="http://www.w3.org/1998/Math/MathML">
    <ci> FLUX_VALUE </ci>
  </math>
  <listOfParameters>
    <parameter id="LOWER_BOUND" value="-1000" units="mmol_per_gDW_per_hr" constant="true"/>
    <parameter id="UPPER_BOUND" value="1000" units="mmol_per_gDW_per_hr" constant="true"/>
    <parameter id="FLUX_VALUE" value="0" units="mmol_per_gDW_per_hr" constant="true"/>
    <parameter id="OBJECTIVE_COEFFICIENT" value="0" units="mmol_per_gDW_per_hr" constant="true"/>
  </listOfParameters>
</kineticLaw>
</reaction>
<reaction id="R_R302" name="oxepin-CoA hydrolase" reversible="false">
```

```xml
  <notes>
    <body xmlns="http://www.w3.org/1999/xhtml">
      <p>GENE_ASSOCIATION: TTC0604</p>
      <p>SUBSYSTEM: Tyrosine, Tryptophan, and Phenylalanine Metabolism</p>
      <p>EC Number: 3.7.1.16</p>
      <p>Confidence Level: 1</p>
      <p>AUTHORS: Amino acid metabolism</p>
      <p/>
    </body>
  </notes>
  <listOfReactants>
    <speciesReference species="M_H2O_c" stoichiometry="1"/>
    <speciesReference species="M_OPACCoA_c" stoichiometry="1"/>
  </listOfReactants>
  <listOfProducts>
    <speciesReference species="M_ODHSCoASA_c" stoichiometry="1"/>
  </listOfProducts>
  <kineticLaw>
    <math xmlns="http://www.w3.org/1998/Math/MathML">
      <ci> FLUX_VALUE </ci>
    </math>
    <listOfParameters>
      <parameter id="LOWER_BOUND" value="0" units="mmol_per_gDW_per_hr" constant="true"/>
      <parameter id="UPPER_BOUND" value="1000" units="mmol_per_gDW_per_hr" constant="true"/>
      <parameter id="FLUX_VALUE" value="0" units="mmol_per_gDW_per_hr" constant="true"/>
      <parameter id="OBJECTIVE_COEFFICIENT" value="0" units="mmol_per_gDW_per_hr" constant="true"/>
    </listOfParameters>
  </kineticLaw>
</reaction>
```

```
<reaction id="R_R303" name="3-oxo-5,6-dehydrosuberyl-CoA semialdehyde dehydrogenase" reversible="false">
 <notes>
  <body xmlns="http://www.w3.org/1999/xhtml">
   <p>GENE_ASSOCIATION: TTC0604</p>
   <p>SUBSYSTEM: Tyrosine, Tryptophan, and Phenylalanine Metabolism</p>
   <p>EC Number: 1.17.1.17</p>
   <p>Confidence Level: 1</p>
   <p>AUTHORS: Amino acid metabolism</p>
   <p/>
  </body>
 </notes>
 <listOfReactants>
  <speciesReference species="M_H2O_c" stoichiometry="1"/>
  <speciesReference species="M_NADP_c" stoichiometry="1"/>
  <speciesReference species="M_ODHSCoASA_c" stoichiometry="1"/>
 </listOfReactants>
 <listOfProducts>
  <speciesReference species="M_H_c" stoichiometry="2"/>
  <speciesReference species="M_NADPH_c" stoichiometry="1"/>
  <speciesReference species="M_ODHSCoA_c" stoichiometry="1"/>
 </listOfProducts>
 <kineticLaw>
  <math xmlns="http://www.w3.org/1998/Math/MathML">
   <ci> FLUX_VALUE </ci>
  </math>
  <listOfParameters>
   <parameter id="LOWER_BOUND" value="0" units="mmol_per_gDW_per_hr" constant="true"/>
   <parameter id="UPPER_BOUND" value="1000" units="mmol_per_gDW_per_hr" constant="true"/>
   <parameter id="FLUX_VALUE" value="0" units="mmol_per_gDW_per_hr" constant="true"/>
```

```
      <parameter id="OBJECTIVE_COEFFICIENT" value="0" units="mmol_per_gDW_per_hr" constant="true"/>
    </listOfParameters>
  </kineticLaw>
</reaction>
<reaction id="R_R304" name="beta-ketoadipyl CoA thiolase" reversible="false">
  <notes>
    <body xmlns="http://www.w3.org/1999/xhtml">
      <p>GENE_ASSOCIATION: TTC0623</p>
      <p>SUBSYSTEM: Tyrosine, Tryptophan, and Phenylalanine Metabolism</p>
      <p>EC Number: 2.3.1.-</p>
      <p>Confidence Level: 1</p>
      <p>AUTHORS: Amino acid metabolism</p>
      <p/>
    </body>
  </notes>
  <listOfReactants>
    <speciesReference species="M_CoA_c" stoichiometry="1"/>
    <speciesReference species="M_ODHSCoA_c" stoichiometry="1"/>
  </listOfReactants>
  <listOfProducts>
    <speciesReference species="M_ACoA_c" stoichiometry="1"/>
    <speciesReference species="M_DHADPCoA_c" stoichiometry="1"/>
  </listOfProducts>
  <kineticLaw>
    <math xmlns="http://www.w3.org/1998/Math/MathML">
      <ci> FLUX_VALUE </ci>
    </math>
    <listOfParameters>
      <parameter id="LOWER_BOUND" value="0" units="mmol_per_gDW_per_hr" constant="true"/>
```

```xml
<parameter id="UPPER_BOUND" value="1000" units="mmol_per_gDW_per_hr" constant="true"/>

<parameter id="FLUX_VALUE" value="0" units="mmol_per_gDW_per_hr" constant="true"/>

<parameter id="OBJECTIVE_COEFFICIENT" value="0" units="mmol_per_gDW_per_hr" constant="true"/>

</listOfParameters>

</kineticLaw>

</reaction>

<reaction id="R_R305" name="enoyl-CoA hydratase " reversible="false">

<notes>

<body xmlns="http://www.w3.org/1999/xhtml">

<p>GENE_ASSOCIATION: (TTC0604 or TTC1697 or TTC1768)</p>

<p>SUBSYSTEM: Tyrosine, Tryptophan, and Phenylalanine Metabolism</p>

<p>EC Number: 4.2.1.17</p>

<p>Confidence Level: 1</p>

<p>AUTHORS: Amino acid metabolism</p>

<p/>

</body>

</notes>

<listOfReactants>

<speciesReference species="M_DHADPCoA_c" stoichiometry="1"/>

</listOfReactants>

<listOfProducts>

<speciesReference species="M_H2O_c" stoichiometry="1"/>

<speciesReference species="M_HADPCoA_c" stoichiometry="1"/>

</listOfProducts>

<kineticLaw>

<math xmlns="http://www.w3.org/1998/Math/MathML">

<ci> FLUX_VALUE </ci>

</math>

<listOfParameters>
```

```
        <parameter id="LOWER_BOUND" value="0" units="mmol_per_gDW_per_hr" constant="true"/>
        <parameter id="UPPER_BOUND" value="1000" units="mmol_per_gDW_per_hr" constant="true"/>
        <parameter id="FLUX_VALUE" value="0" units="mmol_per_gDW_per_hr" constant="true"/>
        <parameter id="OBJECTIVE_COEFFICIENT" value="0" units="mmol_per_gDW_per_hr" constant="true"/>
      </listOfParameters>
    </kineticLaw>
  </reaction>
  <reaction id="R_R306" name="3-hydroxyacyl-CoA dehydrogenase" reversible="false">
    <notes>
      <body xmlns="http://www.w3.org/1999/xhtml">
        <p>GENE_ASSOCIATION: (TTC0331 or TTC0534)</p>
        <p>SUBSYSTEM: Tyrosine, Tryptophan, and Phenylalanine Metabolism</p>
        <p>EC Number: 1.1.1.35</p>
        <p>Confidence Level: 1</p>
        <p>AUTHORS: Amino acid metabolism</p>
        <p/>
      </body>
    </notes>
    <listOfReactants>
      <speciesReference species="M_NAD_c" stoichiometry="1"/>
      <speciesReference species="M_HADPCoA_c" stoichiometry="1"/>
    </listOfReactants>
    <listOfProducts>
      <speciesReference species="M_H_c" stoichiometry="1"/>
      <speciesReference species="M_NADH_c" stoichiometry="1"/>
      <speciesReference species="M_OADPCoA_c" stoichiometry="1"/>
    </listOfProducts>
    <kineticLaw>
      <math xmlns="http://www.w3.org/1998/Math/MathML">
```

```
      <ci> FLUX_VALUE </ci>
    </math>
    <listOfParameters>
      <parameter id="LOWER_BOUND" value="0" units="mmol_per_gDW_per_hr" constant="true"/>
      <parameter id="UPPER_BOUND" value="1000" units="mmol_per_gDW_per_hr" constant="true"/>
      <parameter id="FLUX_VALUE" value="0" units="mmol_per_gDW_per_hr" constant="true"/>
      <parameter id="OBJECTIVE_COEFFICIENT" value="0" units="mmol_per_gDW_per_hr" constant="true"/>
    </listOfParameters>
  </kineticLaw>
</reaction>
<reaction id="R_R307" name="beta-ketoadipyl CoA thiolase" reversible="false">
  <notes>
    <body xmlns="http://www.w3.org/1999/xhtml">
      <p>GENE_ASSOCIATION: TTC0623</p>
      <p>SUBSYSTEM: Tyrosine, Tryptophan, and Phenylalanine Metabolism</p>
      <p>EC Number: 2.3.1.174</p>
      <p>Confidence Level: 1</p>
      <p>AUTHORS: Amino acid metabolism</p>
      <p/>
    </body>
  </notes>
  <listOfReactants>
    <speciesReference species="M_CoA_c" stoichiometry="1"/>
    <speciesReference species="M_OADPCoA_c" stoichiometry="1"/>
  </listOfReactants>
  <listOfProducts>
    <speciesReference species="M_ACoA_c" stoichiometry="1"/>
    <speciesReference species="M_SUCCoA_c" stoichiometry="1"/>
  </listOfProducts>
```

```
<kineticLaw>
 <math xmlns="http://www.w3.org/1998/Math/MathML">
  <ci> FLUX_VALUE </ci>
 </math>
 <listOfParameters>
  <parameter id="LOWER_BOUND" value="0" units="mmol_per_gDW_per_hr" constant="true"/>
  <parameter id="UPPER_BOUND" value="1000" units="mmol_per_gDW_per_hr" constant="true"/>
  <parameter id="FLUX_VALUE" value="0" units="mmol_per_gDW_per_hr" constant="true"/>
  <parameter id="OBJECTIVE_COEFFICIENT" value="0" units="mmol_per_gDW_per_hr" constant="true"/>
 </listOfParameters>
</kineticLaw>
</reaction>
<reaction id="R_R308" name="2-oxoadipate dehydrogenase complex" reversible="false">
 <notes>
  <body xmlns="http://www.w3.org/1999/xhtml">
   <p>GENE_ASSOCIATION: (TTC1698 or (TTC1700 or TTC1753) or TTC1699)</p>
   <p>SUBSYSTEM: Tyrosine, Tryptophan, and Phenylalanine Metabolism</p>
   <p>EC Number: 1.2.4.2 or 1.8.1.4 or 2.3.1.61</p>
   <p>Confidence Level: 1</p>
   <p>AUTHORS: Amino acid metabolism</p>
   <p/>
  </body>
 </notes>
 <listOfReactants>
  <speciesReference species="M_NAD_c" stoichiometry="1"/>
  <speciesReference species="M_CoA_c" stoichiometry="1"/>
  <speciesReference species="M_2OADPA_c" stoichiometry="1"/>
 </listOfReactants>
 <listOfProducts>
```

```
    <speciesReference species="M_H_c" stoichiometry="1"/>
    <speciesReference species="M_NADH_c" stoichiometry="1"/>
    <speciesReference species="M_CO2_c" stoichiometry="1"/>
    <speciesReference species="M_GLUTRCoA_c" stoichiometry="1"/>
  </listOfProducts>
  <kineticLaw>
    <math xmlns="http://www.w3.org/1998/Math/MathML">
      <ci> FLUX_VALUE </ci>
    </math>
    <listOfParameters>
      <parameter id="LOWER_BOUND" value="0" units="mmol_per_gDW_per_hr" constant="true"/>
      <parameter id="UPPER_BOUND" value="1000" units="mmol_per_gDW_per_hr" constant="true"/>
      <parameter id="FLUX_VALUE" value="0" units="mmol_per_gDW_per_hr" constant="true"/>
      <parameter id="OBJECTIVE_COEFFICIENT" value="0" units="mmol_per_gDW_per_hr" constant="true"/>
    </listOfParameters>
  </kineticLaw>
</reaction>
<reaction id="R_R309" name="L-aspartate 1-carboxy-lyase (beta-alanine-forming)" reversible="false">
  <notes>
    <body xmlns="http://www.w3.org/1999/xhtml">
      <p>GENE_ASSOCIATION: TTC0241</p>
      <p>SUBSYSTEM: Cofactor and Prosthetic Group Biosynthesis</p>
      <p>EC Number: 4.1.1.11</p>
      <p>Confidence Level: 1</p>
      <p>AUTHORS: Energy and cofactors metabolism</p>
      <p/>
    </body>
  </notes>
  <listOfReactants>
```

```
      <speciesReference species="M_L_ASP_c" stoichiometry="1"/>
      <speciesReference species="M_H_c" stoichiometry="1"/>
    </listOfReactants>
    <listOfProducts>
      <speciesReference species="M_CO2_c" stoichiometry="1"/>
      <speciesReference species="M_B_ALA_c" stoichiometry="1"/>
    </listOfProducts>
    <kineticLaw>
      <math xmlns="http://www.w3.org/1998/Math/MathML">
        <ci> FLUX_VALUE </ci>
      </math>
      <listOfParameters>
        <parameter id="LOWER_BOUND" value="0" units="mmol_per_gDW_per_hr" constant="true"/>
        <parameter id="UPPER_BOUND" value="1000" units="mmol_per_gDW_per_hr" constant="true"/>
        <parameter id="FLUX_VALUE" value="0" units="mmol_per_gDW_per_hr" constant="true"/>
        <parameter id="OBJECTIVE_COEFFICIENT" value="0" units="mmol_per_gDW_per_hr" constant="true"/>
      </listOfParameters>
    </kineticLaw>
  </reaction>
  <reaction id="R_R310" name="beta-alanine:2-oxoglutarate aminotransferase" reversible="true">
    <notes>
      <body xmlns="http://www.w3.org/1999/xhtml">
        <p>GENE_ASSOCIATION: TTC0510</p>
        <p>SUBSYSTEM: Beta-Alanine metabolism </p>
        <p>EC Number: 2.6.1.19</p>
        <p>Confidence Level: 1</p>
        <p>AUTHORS: Amino acid metabolism</p>
        <p/>
      </body>
```

```
  </notes>
  <listOfReactants>
    <speciesReference species="M_AKG_c" stoichiometry="1"/>
    <speciesReference species="M_B_ALA_c" stoichiometry="1"/>
  </listOfReactants>
  <listOfProducts>
    <speciesReference species="M_L_GLU_c" stoichiometry="1"/>
    <speciesReference species="M_MAL_SA_c" stoichiometry="1"/>
  </listOfProducts>
  <kineticLaw>
    <math xmlns="http://www.w3.org/1998/Math/MathML">
      <ci> FLUX_VALUE </ci>
    </math>
    <listOfParameters>
      <parameter id="LOWER_BOUND" value="-1000" units="mmol_per_gDW_per_hr" constant="true"/>
      <parameter id="UPPER_BOUND" value="1000" units="mmol_per_gDW_per_hr" constant="true"/>
      <parameter id="FLUX_VALUE" value="0" units="mmol_per_gDW_per_hr" constant="true"/>
      <parameter id="OBJECTIVE_COEFFICIENT" value="0" units="mmol_per_gDW_per_hr" constant="true"/>
    </listOfParameters>
  </kineticLaw>
</reaction>
<reaction id="R_R311" name="malonate-semialdehyde dehydrogenase" reversible="false">
  <notes>
    <body xmlns="http://www.w3.org/1999/xhtml">
      <p>GENE_ASSOCIATION: </p>
      <p>SUBSYSTEM: Beta-Alanine metabolism </p>
      <p>EC Number: 1.2.1.-</p>
      <p>Confidence Level: 1</p>
      <p>AUTHORS: Amino acid metabolism</p>
```

```xml
<p/>

</body>

</notes>

<listOfReactants>

<speciesReference species="M_NAD_c" stoichiometry="1"/>

<speciesReference species="M_CoA_c" stoichiometry="1"/>

<speciesReference species="M_MAL_SA_c" stoichiometry="1"/>

</listOfReactants>

<listOfProducts>

<speciesReference species="M_NADH_c" stoichiometry="1"/>

<speciesReference species="M_CO2_c" stoichiometry="1"/>

<speciesReference species="M_ACoA_c" stoichiometry="1"/>

</listOfProducts>

<kineticLaw>

<math xmlns="http://www.w3.org/1998/Math/MathML">

<ci> FLUX_VALUE </ci>

</math>

<listOfParameters>

<parameter id="LOWER_BOUND" value="0" units="mmol_per_gDW_per_hr" constant="true"/>

<parameter id="UPPER_BOUND" value="1000" units="mmol_per_gDW_per_hr" constant="true"/>

<parameter id="FLUX_VALUE" value="0" units="mmol_per_gDW_per_hr" constant="true"/>

<parameter id="OBJECTIVE_COEFFICIENT" value="0" units="mmol_per_gDW_per_hr" constant="true"/>

</listOfParameters>

</kineticLaw>

</reaction>

<reaction id="R_R312" name="(R)-Pantoate:beta-alanine ligase (AMP-forming)" reversible="false">

<notes>

<body xmlns="http://www.w3.org/1999/xhtml">

<p>GENE_ASSOCIATION: TTC1416</p>
```

```
    <p>SUBSYSTEM: Cofactor and Prosthetic Group Biosynthesis</p>
    <p>EC Number: 6.3.2.1</p>
    <p>Confidence Level: 1</p>
    <p>AUTHORS: Energy and cofactors metabolism</p>
    <p/>
  </body>
</notes>
<listOfReactants>
  <speciesReference species="M_ATP_c" stoichiometry="1"/>
  <speciesReference species="M_B_ALA_c" stoichiometry="1"/>
  <speciesReference species="M_PANTA_c" stoichiometry="1"/>
</listOfReactants>
<listOfProducts>
  <speciesReference species="M_PPi_c" stoichiometry="1"/>
  <speciesReference species="M_H_c" stoichiometry="1"/>
  <speciesReference species="M_AMP_c" stoichiometry="1"/>
  <speciesReference species="M_PANTO_c" stoichiometry="1"/>
</listOfProducts>
<kineticLaw>
  <math xmlns="http://www.w3.org/1998/Math/MathML">
    <ci> FLUX_VALUE </ci>
  </math>
  <listOfParameters>
    <parameter id="LOWER_BOUND" value="0" units="mmol_per_gDW_per_hr" constant="true"/>
    <parameter id="UPPER_BOUND" value="1000" units="mmol_per_gDW_per_hr" constant="true"/>
    <parameter id="FLUX_VALUE" value="0" units="mmol_per_gDW_per_hr" constant="true"/>
    <parameter id="OBJECTIVE_COEFFICIENT" value="0" units="mmol_per_gDW_per_hr" constant="true"/>
  </listOfParameters>
</kineticLaw>
```

```
</reaction>
<reaction id="R_R313" name="UDP-N-acetylmuramate:L-alanine ligase (ADP-forming)" reversible="false">
 <notes>
  <body xmlns="http://www.w3.org/1999/xhtml">
   <p>GENE_ASSOCIATION: TTC0720</p>
   <p>SUBSYSTEM: Cell Envelope Biosynthesis</p>
   <p>EC Number: 6.3.2.8</p>
   <p>Confidence Level: 1</p>
   <p>AUTHORS: Lipid metabolism</p>
   <p/>
  </body>
 </notes>
 <listOfReactants>
  <speciesReference species="M_L_ALA_c" stoichiometry="1"/>
  <speciesReference species="M_UDP_ACMURM_c" stoichiometry="1"/>
  <speciesReference species="M_ATP_c" stoichiometry="1"/>
 </listOfReactants>
 <listOfProducts>
  <speciesReference species="M_ADP_c" stoichiometry="1"/>
  <speciesReference species="M_Pi_c" stoichiometry="1"/>
  <speciesReference species="M_H_c" stoichiometry="1"/>
  <speciesReference species="M_UDP_ACMURM_ALA_c" stoichiometry="1"/>
 </listOfProducts>
 <kineticLaw>
  <math xmlns="http://www.w3.org/1998/Math/MathML">
   <ci> FLUX_VALUE </ci>
  </math>
  <listOfParameters>
   <parameter id="LOWER_BOUND" value="0" units="mmol_per_gDW_per_hr" constant="true"/>
```

```xml
      <parameter id="UPPER_BOUND" value="1000" units="mmol_per_gDW_per_hr" constant="true"/>
      <parameter id="FLUX_VALUE" value="0" units="mmol_per_gDW_per_hr" constant="true"/>
      <parameter id="OBJECTIVE_COEFFICIENT" value="0" units="mmol_per_gDW_per_hr" constant="true"/>
    </listOfParameters>
  </kineticLaw>
</reaction>
<reaction id="R_R314" name="UDP-N-acetylmuramoyl-L-alanine:D-glutamate ligase(ADP-forming)" reversible="false">
  <notes>
    <body xmlns="http://www.w3.org/1999/xhtml">
      <p>GENE_ASSOCIATION: TTC0717</p>
      <p>SUBSYSTEM: Cell Envelope Biosynthesis</p>
      <p>EC Number: 6.3.2.9</p>
      <p>Confidence Level: 1</p>
      <p>AUTHORS: Lipid metabolism</p>
      <p/>
    </body>
  </notes>
  <listOfReactants>
    <speciesReference species="M_D_GLU_c" stoichiometry="1"/>
    <speciesReference species="M_ATP_c" stoichiometry="1"/>
    <speciesReference species="M_UDP_ACMURM_ALA_c" stoichiometry="1"/>
  </listOfReactants>
  <listOfProducts>
    <speciesReference species="M_ADP_c" stoichiometry="1"/>
    <speciesReference species="M_Pi_c" stoichiometry="1"/>
    <speciesReference species="M_H_c" stoichiometry="1"/>
    <speciesReference species="M_UDP_ACMURM_ALA_GLUT_c" stoichiometry="1"/>
  </listOfProducts>
  <kineticLaw>
```

```
<math xmlns="http://www.w3.org/1998/Math/MathML">
  <ci> FLUX_VALUE </ci>
</math>
<listOfParameters>
  <parameter id="LOWER_BOUND" value="0" units="mmol_per_gDW_per_hr" constant="true"/>
  <parameter id="UPPER_BOUND" value="1000" units="mmol_per_gDW_per_hr" constant="true"/>
  <parameter id="FLUX_VALUE" value="0" units="mmol_per_gDW_per_hr" constant="true"/>
  <parameter id="OBJECTIVE_COEFFICIENT" value="0" units="mmol_per_gDW_per_hr" constant="true"/>
</listOfParameters>
</kineticLaw>
</reaction>
<reaction id="R_R315" name="glutamate racemase" reversible="true">
 <notes>
  <body xmlns="http://www.w3.org/1999/xhtml">
   <p>GENE_ASSOCIATION: TTC1280</p>
   <p>SUBSYSTEM: Cell Envelope Biosynthesis</p>
   <p>EC Number: 5.1.1.3</p>
   <p>Confidence Level: 1</p>
   <p>AUTHORS: Lipid metabolism</p>
   <p/>
  </body>
 </notes>
 <listOfReactants>
  <speciesReference species="M_L_GLU_c" stoichiometry="1"/>
 </listOfReactants>
 <listOfProducts>
  <speciesReference species="M_D_GLU_c" stoichiometry="1"/>
 </listOfProducts>
 <kineticLaw>
```

```
    <math xmlns="http://www.w3.org/1998/Math/MathML">
      <ci> FLUX_VALUE </ci>
    </math>
    <listOfParameters>
      <parameter id="LOWER_BOUND" value="-1000" units="mmol_per_gDW_per_hr" constant="true"/>
      <parameter id="UPPER_BOUND" value="1000" units="mmol_per_gDW_per_hr" constant="true"/>
      <parameter id="FLUX_VALUE" value="0" units="mmol_per_gDW_per_hr" constant="true"/>
      <parameter id="OBJECTIVE_COEFFICIENT" value="0" units="mmol_per_gDW_per_hr" constant="true"/>
    </listOfParameters>
  </kineticLaw>
</reaction>
<reaction id="R_R316" name="alanine racemase" reversible="true">
  <notes>
    <body xmlns="http://www.w3.org/1999/xhtml">
      <p>GENE_ASSOCIATION: TTC1944</p>
      <p>SUBSYSTEM: Alanine and Aspartate Metabolism</p>
      <p>EC Number: 5.1.1.1</p>
      <p>Confidence Level: 1</p>
      <p>AUTHORS: Amino acid metabolism</p>
      <p/>
    </body>
  </notes>
  <listOfReactants>
    <speciesReference species="M_L_ALA_c" stoichiometry="1"/>
  </listOfReactants>
  <listOfProducts>
    <speciesReference species="M_D_ALA_c" stoichiometry="1"/>
  </listOfProducts>
  <kineticLaw>
```

```
    <math xmlns="http://www.w3.org/1998/Math/MathML">
      <ci> FLUX_VALUE </ci>
    </math>
    <listOfParameters>
      <parameter id="LOWER_BOUND" value="-1000" units="mmol_per_gDW_per_hr" constant="true"/>
      <parameter id="UPPER_BOUND" value="1000" units="mmol_per_gDW_per_hr" constant="true"/>
      <parameter id="FLUX_VALUE" value="0" units="mmol_per_gDW_per_hr" constant="true"/>
      <parameter id="OBJECTIVE_COEFFICIENT" value="0" units="mmol_per_gDW_per_hr" constant="true"/>
    </listOfParameters>
  </kineticLaw>
</reaction>
<reaction id="R_R317" name="D-alanine:D-alanine ligase (ADP-forming)" reversible="true">
  <notes>
    <body xmlns="http://www.w3.org/1999/xhtml">
      <p>GENE_ASSOCIATION: TTC1223</p>
      <p>SUBSYSTEM: Cell Envelope Biosynthesis</p>
      <p>EC Number: 6.3.2.4</p>
      <p>Confidence Level: 1</p>
      <p>AUTHORS: Lipid metabolism</p>
      <p/>
    </body>
  </notes>
  <listOfReactants>
    <speciesReference species="M_D_ALA_c" stoichiometry="2"/>
    <speciesReference species="M_ATP_c" stoichiometry="1"/>
  </listOfReactants>
  <listOfProducts>
    <speciesReference species="M_ADP_c" stoichiometry="1"/>
    <speciesReference species="M_Pi_c" stoichiometry="1"/>
```

```
    <speciesReference species="M_H_c" stoichiometry="1"/>
    <speciesReference species="M_D_ALA_D_ALA_c" stoichiometry="1"/>
  </listOfProducts>
  <kineticLaw>
    <math xmlns="http://www.w3.org/1998/Math/MathML">
      <ci> FLUX_VALUE </ci>
    </math>
    <listOfParameters>
      <parameter id="LOWER_BOUND" value="-1000" units="mmol_per_gDW_per_hr" constant="true"/>
      <parameter id="UPPER_BOUND" value="1000" units="mmol_per_gDW_per_hr" constant="true"/>
      <parameter id="FLUX_VALUE" value="0" units="mmol_per_gDW_per_hr" constant="true"/>
      <parameter id="OBJECTIVE_COEFFICIENT" value="0" units="mmol_per_gDW_per_hr" constant="true"/>
    </listOfParameters>
  </kineticLaw>
</reaction>
<reaction id="R_R318" name="(2R,3S)-3-methylmalate:NAD+ oxidoreductase" reversible="false">
  <notes>
    <body xmlns="http://www.w3.org/1999/xhtml">
      <p>GENE_ASSOCIATION: TTC0867</p>
      <p>SUBSYSTEM: Valine, Leucine, and Isoleucine Metabolism</p>
      <p>EC Number: 1.1.1.85</p>
      <p>Confidence Level: 1</p>
      <p>AUTHORS: Amino acid metabolism</p>
      <p/>
    </body>
  </notes>
  <listOfReactants>
    <speciesReference species="M_NAD_c" stoichiometry="1"/>
    <speciesReference species="M_IPROP_MAL_c" stoichiometry="1"/>
```

```xml
</listOfReactants>
<listOfProducts>
  <speciesReference species="M_H_c" stoichiometry="1"/>
  <speciesReference species="M_NADH_c" stoichiometry="1"/>
  <speciesReference species="M_CO2_c" stoichiometry="1"/>
  <speciesReference species="M_AKB_c" stoichiometry="1"/>
</listOfProducts>
<kineticLaw>
  <math xmlns="http://www.w3.org/1998/Math/MathML">
    <ci> FLUX_VALUE </ci>
  </math>
  <listOfParameters>
    <parameter id="LOWER_BOUND" value="0" units="mmol_per_gDW_per_hr" constant="true"/>
    <parameter id="UPPER_BOUND" value="1000" units="mmol_per_gDW_per_hr" constant="true"/>
    <parameter id="FLUX_VALUE" value="0" units="mmol_per_gDW_per_hr" constant="true"/>
    <parameter id="OBJECTIVE_COEFFICIENT" value="0" units="mmol_per_gDW_per_hr" constant="true"/>
  </listOfParameters>
</kineticLaw>
</reaction>
<reaction id="R_R319" name="(R)-2,3-Dihydroxy-3-methylbutanoate:NADP+ oxidoreductase (isomerizing)" reversible="true">
<notes>
  <body xmlns="http://www.w3.org/1999/xhtml">
    <p>GENE_ASSOCIATION: TTC0850</p>
    <p>SUBSYSTEM: Valine, Leucine, and Isoleucine Metabolism</p>
    <p>EC Number: 1.1.1.86</p>
    <p>Confidence Level: 1</p>
    <p>AUTHORS: Amino acid metabolism</p>
    <p/>
```

```xml
</body>

</notes>

<listOfReactants>

<speciesReference species="M_NADP_c" stoichiometry="1"/>

<speciesReference species="M_RDHY_MBUTNA_c" stoichiometry="1"/>

</listOfReactants>

<listOfProducts>

<speciesReference species="M_H_c" stoichiometry="1"/>

<speciesReference species="M_NADPH_c" stoichiometry="1"/>

<speciesReference species="M_3HY_2M_2OBUTNA_c" stoichiometry="1"/>

</listOfProducts>

<kineticLaw>

<math xmlns="http://www.w3.org/1998/Math/MathML">

<ci> FLUX_VALUE </ci>

</math>

<listOfParameters>

<parameter id="LOWER_BOUND" value="-1000" units="mmol_per_gDW_per_hr" constant="true"/>

<parameter id="UPPER_BOUND" value="1000" units="mmol_per_gDW_per_hr" constant="true"/>

<parameter id="FLUX_VALUE" value="0" units="mmol_per_gDW_per_hr" constant="true"/>

<parameter id="OBJECTIVE_COEFFICIENT" value="0" units="mmol_per_gDW_per_hr" constant="true"/>

</listOfParameters>

</kineticLaw>

</reaction>

<reaction id="R_R320" name="(R)-2,3-Dihydroxy-3-methylpentanoate:NADP+ oxidoreductase (isomerizing)" reversible="true">

<notes>

<body xmlns="http://www.w3.org/1999/xhtml">

<p>GENE_ASSOCIATION: TTC0850</p>

<p>SUBSYSTEM: Valine, Leucine, and Isoleucine Metabolism</p>
```

```
      <p>EC Number: 1.1.1.86</p>
      <p>Confidence Level: 1</p>
      <p>AUTHORS: Amino acid metabolism</p>
      <p/>
    </body>
  </notes>
  <listOfReactants>
    <speciesReference species="M_NADP_c" stoichiometry="1"/>
    <speciesReference species="M_DHY_MPENTA_c" stoichiometry="1"/>
  </listOfReactants>
  <listOfProducts>
    <speciesReference species="M_H_c" stoichiometry="1"/>
    <speciesReference species="M_NADPH_c" stoichiometry="1"/>
    <speciesReference species="M_AC_HYBUTNA_c" stoichiometry="1"/>
  </listOfProducts>
  <kineticLaw>
    <math xmlns="http://www.w3.org/1998/Math/MathML">
      <ci> FLUX_VALUE </ci>
    </math>
    <listOfParameters>
      <parameter id="LOWER_BOUND" value="-1000" units="mmol_per_gDW_per_hr" constant="true"/>
      <parameter id="UPPER_BOUND" value="1000" units="mmol_per_gDW_per_hr" constant="true"/>
      <parameter id="FLUX_VALUE" value="0" units="mmol_per_gDW_per_hr" constant="true"/>
      <parameter id="OBJECTIVE_COEFFICIENT" value="0" units="mmol_per_gDW_per_hr" constant="true"/>
    </listOfParameters>
  </kineticLaw>
</reaction>
<reaction id="R_R321" name="(S)-2-Acetolactate methylmutase" reversible="true">
  <notes>
```

```xml
  <body xmlns="http://www.w3.org/1999/xhtml">
    <p>GENE_ASSOCIATION: TTC0850</p>
    <p>SUBSYSTEM: Valine, Leucine, and Isoleucine Metabolism</p>
    <p>EC Number: 1.1.1.86</p>
    <p>Confidence Level: 1</p>
    <p>AUTHORS: Amino acid metabolism</p>
    <p/>
  </body>
 </notes>
 <listOfReactants>
  <speciesReference species="M_S_ACLAC_c" stoichiometry="1"/>
 </listOfReactants>
 <listOfProducts>
  <speciesReference species="M_3HY_2M_2OBUTNA_c" stoichiometry="1"/>
 </listOfProducts>
 <kineticLaw>
  <math xmlns="http://www.w3.org/1998/Math/MathML">
    <ci> FLUX_VALUE </ci>
  </math>
  <listOfParameters>
    <parameter id="LOWER_BOUND" value="-1000" units="mmol_per_gDW_per_hr" constant="true"/>
    <parameter id="UPPER_BOUND" value="1000" units="mmol_per_gDW_per_hr" constant="true"/>
    <parameter id="FLUX_VALUE" value="0" units="mmol_per_gDW_per_hr" constant="true"/>
    <parameter id="OBJECTIVE_COEFFICIENT" value="0" units="mmol_per_gDW_per_hr" constant="true"/>
  </listOfParameters>
 </kineticLaw>
</reaction>
<reaction id="R_R322" name="(R)-2,3-Dihydroxy-3-methylpentanoate hydro-lyase" reversible="false">
 <notes>
```

```xml
    <body xmlns="http://www.w3.org/1999/xhtml">
      <p>GENE_ASSOCIATION: TTC0871</p>
      <p>SUBSYSTEM: Valine, Leucine, and Isoleucine Metabolism</p>
      <p>EC Number: 4.2.1.9</p>
      <p>Confidence Level: 1</p>
      <p>AUTHORS: Amino acid metabolism</p>
      <p/>
    </body>
  </notes>
  <listOfReactants>
    <speciesReference species="M_DHY_MPENTA_c" stoichiometry="1"/>
  </listOfReactants>
  <listOfProducts>
    <speciesReference species="M_H2O_c" stoichiometry="1"/>
    <speciesReference species="M_3M_2OPENTN_c" stoichiometry="1"/>
  </listOfProducts>
  <kineticLaw>
    <math xmlns="http://www.w3.org/1998/Math/MathML">
      <ci> FLUX_VALUE </ci>
    </math>
    <listOfParameters>
      <parameter id="LOWER_BOUND" value="0" units="mmol_per_gDW_per_hr" constant="true"/>
      <parameter id="UPPER_BOUND" value="1000" units="mmol_per_gDW_per_hr" constant="true"/>
      <parameter id="FLUX_VALUE" value="0" units="mmol_per_gDW_per_hr" constant="true"/>
      <parameter id="OBJECTIVE_COEFFICIENT" value="0" units="mmol_per_gDW_per_hr" constant="true"/>
    </listOfParameters>
  </kineticLaw>
</reaction>
<reaction id="R_R323" name="(R)-2,3-Dihydroxy-3-methylbutanoate hydro-lyase" reversible="false">
```

```
<notes>
  <body xmlns="http://www.w3.org/1999/xhtml">
    <p>GENE_ASSOCIATION: TTC0871</p>
    <p>SUBSYSTEM: Valine, Leucine, and Isoleucine Metabolism</p>
    <p>EC Number: 4.2.1.9</p>
    <p>Confidence Level: 1</p>
    <p>AUTHORS: Amino acid metabolism</p>
    <p/>
  </body>
</notes>
<listOfReactants>
  <speciesReference species="M_RDHY_MBUTNA_c" stoichiometry="1"/>
</listOfReactants>
<listOfProducts>
  <speciesReference species="M_H2O_c" stoichiometry="1"/>
  <speciesReference species="M_2K3M_BUT_c" stoichiometry="1"/>
</listOfProducts>
<kineticLaw>
  <math xmlns="http://www.w3.org/1998/Math/MathML">
    <ci> FLUX_VALUE </ci>
  </math>
  <listOfParameters>
    <parameter id="LOWER_BOUND" value="0" units="mmol_per_gDW_per_hr" constant="true"/>
    <parameter id="UPPER_BOUND" value="1000" units="mmol_per_gDW_per_hr" constant="true"/>
    <parameter id="FLUX_VALUE" value="0" units="mmol_per_gDW_per_hr" constant="true"/>
    <parameter id="OBJECTIVE_COEFFICIENT" value="0" units="mmol_per_gDW_per_hr" constant="true"/>
  </listOfParameters>
</kineticLaw>
</reaction>
```

```
<reaction id="R_R324" name="acetyl-CoA:3-methyl-2-oxobutanoate C-acetyltransferase (thioester-hydrolysing, carboxymethyl-forming)" reversible="false">
  <notes>
    <body xmlns="http://www.w3.org/1999/xhtml">
      <p>GENE_ASSOCIATION: (TTC0847 or TTC0849)</p>
      <p>SUBSYSTEM: Valine, Leucine, and Isoleucine Metabolism</p>
      <p>EC Number: 2.3.3.13</p>
      <p>Confidence Level: 1</p>
      <p>AUTHORS: Amino acid metabolism</p>
      <p/>
    </body>
  </notes>
  <listOfReactants>
    <speciesReference species="M_H2O_c" stoichiometry="1"/>
    <speciesReference species="M_ACoA_c" stoichiometry="1"/>
    <speciesReference species="M_2K3M_BUT_c" stoichiometry="1"/>
  </listOfReactants>
  <listOfProducts>
    <speciesReference species="M_H_c" stoichiometry="1"/>
    <speciesReference species="M_CoA_c" stoichiometry="1"/>
    <speciesReference species="M_IPRO_MAL_c" stoichiometry="1"/>
  </listOfProducts>
  <kineticLaw>
    <math xmlns="http://www.w3.org/1998/Math/MathML">
      <ci> FLUX_VALUE </ci>
    </math>
    <listOfParameters>
      <parameter id="LOWER_BOUND" value="0" units="mmol_per_gDW_per_hr" constant="true"/>
      <parameter id="UPPER_BOUND" value="1000" units="mmol_per_gDW_per_hr" constant="true"/>
```

```
<parameter id="FLUX_VALUE" value="0" units="mmol_per_gDW_per_hr" constant="true"/>

<parameter id="OBJECTIVE_COEFFICIENT" value="0" units="mmol_per_gDW_per_hr" constant="true"/>

</listOfParameters>

</kineticLaw>

</reaction>

<reaction id="R_R325" name="2-Isopropylmalate hydro-lyase" reversible="true">

<notes>

<body xmlns="http://www.w3.org/1999/xhtml">

<p>GENE_ASSOCIATION: ((TTC0865 and TTC0866) or (TTC1546 and TTC1547))</p>

<p>SUBSYSTEM: Valine, Leucine, and Isoleucine Metabolism</p>

<p>EC Number: 4.2.1.33</p>

<p>Confidence Level: 1</p>

<p>AUTHORS: Amino acid metabolism</p>

<p/>

</body>

</notes>

<listOfReactants>

<speciesReference species="M_IPRO_MAL_c" stoichiometry="1"/>

</listOfReactants>

<listOfProducts>

<speciesReference species="M_H2O_c" stoichiometry="1"/>

<speciesReference species="M_IPRO_MALE_c" stoichiometry="1"/>

</listOfProducts>

<kineticLaw>

<math xmlns="http://www.w3.org/1998/Math/MathML">

<ci> FLUX_VALUE </ci>

</math>

<listOfParameters>

<parameter id="LOWER_BOUND" value="-1000" units="mmol_per_gDW_per_hr" constant="true"/>
```

```
      <parameter id="UPPER_BOUND" value="1000" units="mmol_per_gDW_per_hr" constant="true"/>
      <parameter id="FLUX_VALUE" value="0" units="mmol_per_gDW_per_hr" constant="true"/>
      <parameter id="OBJECTIVE_COEFFICIENT" value="0" units="mmol_per_gDW_per_hr" constant="true"/>
    </listOfParameters>
  </kineticLaw>
</reaction>
<reaction id="R_R326" name="3-Isopropylmalate hydro-lyase" reversible="true">
  <notes>
    <body xmlns="http://www.w3.org/1999/xhtml">
      <p>GENE_ASSOCIATION: ((TTC0865 and TTC0866) or (TTC1546 and TTC1547))</p>
      <p>SUBSYSTEM: Valine, Leucine, and Isoleucine Metabolism</p>
      <p>EC Number: 4.2.1.33</p>
      <p>Confidence Level: 1</p>
      <p>AUTHORS: Amino acid metabolism</p>
      <p/>
    </body>
  </notes>
  <listOfReactants>
    <speciesReference species="M_IPROP_MAL_c" stoichiometry="1"/>
  </listOfReactants>
  <listOfProducts>
    <speciesReference species="M_H2O_c" stoichiometry="1"/>
    <speciesReference species="M_IPRO_MALE_c" stoichiometry="1"/>
  </listOfProducts>
  <kineticLaw>
    <math xmlns="http://www.w3.org/1998/Math/MathML">
      <ci> FLUX_VALUE </ci>
    </math>
    <listOfParameters>
```

```
      <parameter id="LOWER_BOUND" value="-1000" units="mmol_per_gDW_per_hr" constant="true"/>
      <parameter id="UPPER_BOUND" value="1000" units="mmol_per_gDW_per_hr" constant="true"/>
      <parameter id="FLUX_VALUE" value="0" units="mmol_per_gDW_per_hr" constant="true"/>
      <parameter id="OBJECTIVE_COEFFICIENT" value="0" units="mmol_per_gDW_per_hr" constant="true"/>
    </listOfParameters>
  </kineticLaw>
</reaction>
<reaction id="R_R327" name="(2R,3S)-3-isopropylmalate:NAD+ oxidoreductase" reversible="true">
  <notes>
    <body xmlns="http://www.w3.org/1999/xhtml">
      <p>GENE_ASSOCIATION: TTC0867</p>
      <p>SUBSYSTEM: Valine, Leucine, and Isoleucine Metabolism</p>
      <p>EC Number: 1.1.1.85</p>
      <p>Confidence Level: 1</p>
      <p>AUTHORS: Amino acid metabolism</p>
      <p/>
    </body>
  </notes>
  <listOfReactants>
    <speciesReference species="M_NAD_c" stoichiometry="1"/>
    <speciesReference species="M_IPROP_MAL_c" stoichiometry="1"/>
  </listOfReactants>
  <listOfProducts>
    <speciesReference species="M_H_c" stoichiometry="1"/>
    <speciesReference species="M_NADH_c" stoichiometry="1"/>
    <speciesReference species="M_IPROP_OSUCC_c" stoichiometry="1"/>
  </listOfProducts>
  <kineticLaw>
    <math xmlns="http://www.w3.org/1998/Math/MathML">
```

```xml
      <ci> FLUX_VALUE </ci>
    </math>
    <listOfParameters>
      <parameter id="LOWER_BOUND" value="-1000" units="mmol_per_gDW_per_hr" constant="true"/>
      <parameter id="UPPER_BOUND" value="1000" units="mmol_per_gDW_per_hr" constant="true"/>
      <parameter id="FLUX_VALUE" value="0" units="mmol_per_gDW_per_hr" constant="true"/>
      <parameter id="OBJECTIVE_COEFFICIENT" value="0" units="mmol_per_gDW_per_hr" constant="true"/>
    </listOfParameters>
  </kineticLaw>
</reaction>
<reaction id="R_R328" name="acetyl-CoA:L-glutamate N-acetyltransferase" reversible="false">
  <notes>
    <body xmlns="http://www.w3.org/1999/xhtml">
      <p>GENE_ASSOCIATION: (TTC0835 or TTC1703)</p>
      <p>SUBSYSTEM: Arginine and Proline Metabolism</p>
      <p>EC Number: 2.3.1.1</p>
      <p>Confidence Level: 1</p>
      <p>AUTHORS: Amino acid metabolism</p>
      <p/>
    </body>
  </notes>
  <listOfReactants>
    <speciesReference species="M_L_GLU_c" stoichiometry="1"/>
    <speciesReference species="M_ACoA_c" stoichiometry="1"/>
  </listOfReactants>
  <listOfProducts>
    <speciesReference species="M_H_c" stoichiometry="1"/>
    <speciesReference species="M_CoA_c" stoichiometry="1"/>
    <speciesReference species="M_AC_GLUT_c" stoichiometry="1"/>
```

```
  </listOfProducts>
  <kineticLaw>
    <math xmlns="http://www.w3.org/1998/Math/MathML">
      <ci> FLUX_VALUE </ci>
    </math>
    <listOfParameters>
      <parameter id="LOWER_BOUND" value="0" units="mmol_per_gDW_per_hr" constant="true"/>
      <parameter id="UPPER_BOUND" value="1000" units="mmol_per_gDW_per_hr" constant="true"/>
      <parameter id="FLUX_VALUE" value="0" units="mmol_per_gDW_per_hr" constant="true"/>
      <parameter id="OBJECTIVE_COEFFICIENT" value="0" units="mmol_per_gDW_per_hr" constant="true"/>
    </listOfParameters>
  </kineticLaw>
</reaction>
<reaction id="R_R329" name="ATP:N-acetyl-L-glutamate 5-phosphotransferase" reversible="true">
  <notes>
    <body xmlns="http://www.w3.org/1999/xhtml">
      <p>GENE_ASSOCIATION: (TTC1541 or TTC1586)</p>
      <p>SUBSYSTEM: Arginine and Proline Metabolism</p>
      <p>EC Number: 2.7.2.8</p>
      <p>Confidence Level: 1</p>
      <p>AUTHORS: Amino acid metabolism</p>
      <p/>
    </body>
  </notes>
  <listOfReactants>
    <speciesReference species="M_ATP_c" stoichiometry="1"/>
    <speciesReference species="M_AC_GLUT_c" stoichiometry="1"/>
  </listOfReactants>
  <listOfProducts>
```

```
          <speciesReference species="M_ADP_c" stoichiometry="1"/>
          <speciesReference species="M_AC_GLUTPi_c" stoichiometry="1"/>
        </listOfProducts>
        <kineticLaw>
          <math xmlns="http://www.w3.org/1998/Math/MathML">
            <ci> FLUX_VALUE </ci>
          </math>
          <listOfParameters>
            <parameter id="LOWER_BOUND" value="-1000" units="mmol_per_gDW_per_hr" constant="true"/>
            <parameter id="UPPER_BOUND" value="1000" units="mmol_per_gDW_per_hr" constant="true"/>
            <parameter id="FLUX_VALUE" value="0" units="mmol_per_gDW_per_hr" constant="true"/>
            <parameter id="OBJECTIVE_COEFFICIENT" value="0" units="mmol_per_gDW_per_hr" constant="true"/>
          </listOfParameters>
        </kineticLaw>
      </reaction>
      <reaction id="R_R330" name="N-acetyl-L-glutamate-5-semialdehyde:NADP+ 5-oxidoreductase (phosphrylating)"
reversible="true">
        <notes>
          <body xmlns="http://www.w3.org/1999/xhtml">
            <p>GENE_ASSOCIATION: (TTC0836 or TTC1542)</p>
            <p>SUBSYSTEM: Arginine and Proline Metabolism</p>
            <p>EC Number: 1.2.1.38</p>
            <p>Confidence Level: 1</p>
            <p>AUTHORS: Amino acid metabolism</p>
            <p/>
          </body>
        </notes>
        <listOfReactants>
          <speciesReference species="M_H_c" stoichiometry="1"/>
```

```
      <speciesReference species="M_NADPH_c" stoichiometry="1"/>
      <speciesReference species="M_AC_GLUTPi_c" stoichiometry="1"/>
    </listOfReactants>
    <listOfProducts>
      <speciesReference species="M_Pi_c" stoichiometry="1"/>
      <speciesReference species="M_NADP_c" stoichiometry="1"/>
      <speciesReference species="M_AC_GLUT_SA_c" stoichiometry="1"/>
    </listOfProducts>
    <kineticLaw>
      <math xmlns="http://www.w3.org/1998/Math/MathML">
        <ci> FLUX_VALUE </ci>
      </math>
      <listOfParameters>
        <parameter id="LOWER_BOUND" value="-1000" units="mmol_per_gDW_per_hr" constant="true"/>
        <parameter id="UPPER_BOUND" value="1000" units="mmol_per_gDW_per_hr" constant="true"/>
        <parameter id="FLUX_VALUE" value="0" units="mmol_per_gDW_per_hr" constant="true"/>
        <parameter id="OBJECTIVE_COEFFICIENT" value="0" units="mmol_per_gDW_per_hr" constant="true"/>
      </listOfParameters>
    </kineticLaw>
  </reaction>
  <reaction id="R_R331" name="N2-Acetyl-L-ornithine:2-oxoglutarate aminotransferase" reversible="true">
    <notes>
      <body xmlns="http://www.w3.org/1999/xhtml">
        <p>GENE_ASSOCIATION: TTC1393</p>
        <p>SUBSYSTEM: Arginine and Proline Metabolism</p>
        <p>EC Number: 2.6.1.11</p>
        <p>Confidence Level: 1</p>
        <p>AUTHORS: Amino acid metabolism</p>
        <p/>
```

```xml
    </body>
  </notes>
  <listOfReactants>
    <speciesReference species="M_AKG_c" stoichiometry="1"/>
    <speciesReference species="M_AORT_c" stoichiometry="1"/>
  </listOfReactants>
  <listOfProducts>
    <speciesReference species="M_L_GLU_c" stoichiometry="1"/>
    <speciesReference species="M_AC_GLUT_SA_c" stoichiometry="1"/>
  </listOfProducts>
  <kineticLaw>
    <math xmlns="http://www.w3.org/1998/Math/MathML">
      <ci> FLUX_VALUE </ci>
    </math>
    <listOfParameters>
      <parameter id="LOWER_BOUND" value="-1000" units="mmol_per_gDW_per_hr" constant="true"/>
      <parameter id="UPPER_BOUND" value="1000" units="mmol_per_gDW_per_hr" constant="true"/>
      <parameter id="FLUX_VALUE" value="0" units="mmol_per_gDW_per_hr" constant="true"/>
      <parameter id="OBJECTIVE_COEFFICIENT" value="0" units="mmol_per_gDW_per_hr" constant="true"/>
    </listOfParameters>
  </kineticLaw>
</reaction>
<reaction id="R_R332" name="L-Glutamate 5-semialdehyde:NAD+ oxidoreductase" reversible="false">
  <notes>
    <body xmlns="http://www.w3.org/1999/xhtml">
      <p>GENE_ASSOCIATION: TTC1213</p>
      <p>SUBSYSTEM: Arginine and Proline Metabolism</p>
      <p>EC Number: 1.5.1.12</p>
      <p>Confidence Level: 1</p>
```

```xml
      <p>AUTHORS: Amino acid metabolism</p>
      <p/>
    </body>
  </notes>
  <listOfReactants>
    <speciesReference species="M_L_GLU_c" stoichiometry="1"/>
    <speciesReference species="M_H_c" stoichiometry="1"/>
    <speciesReference species="M_NADH_c" stoichiometry="1"/>
  </listOfReactants>
  <listOfProducts>
    <speciesReference species="M_H2O_c" stoichiometry="1"/>
    <speciesReference species="M_NAD_c" stoichiometry="1"/>
    <speciesReference species="M_GLUT_SA_c" stoichiometry="1"/>
  </listOfProducts>
  <kineticLaw>
    <math xmlns="http://www.w3.org/1998/Math/MathML">
      <ci> FLUX_VALUE </ci>
    </math>
    <listOfParameters>
      <parameter id="LOWER_BOUND" value="0" units="mmol_per_gDW_per_hr" constant="true"/>
      <parameter id="UPPER_BOUND" value="1000" units="mmol_per_gDW_per_hr" constant="true"/>
      <parameter id="FLUX_VALUE" value="0" units="mmol_per_gDW_per_hr" constant="true"/>
      <parameter id="OBJECTIVE_COEFFICIENT" value="0" units="mmol_per_gDW_per_hr" constant="true"/>
    </listOfParameters>
  </kineticLaw>
</reaction>
<reaction id="R_R333" name="L-glutamate-5-semialdehyde:NADP+ 5-oxidoreductase (phosphorylating)" reversible="false">
  <notes>
    <body xmlns="http://www.w3.org/1999/xhtml">
```

```
    <p>GENE_ASSOCIATION: TTC1564</p>
    <p>SUBSYSTEM: Arginine and Proline Metabolism</p>
    <p>EC Number: 1.2.1.41</p>
    <p>Confidence Level: 1</p>
    <p>AUTHORS: Amino acid metabolism</p>
    <p/>
  </body>
</notes>
<listOfReactants>
  <speciesReference species="M_H_c" stoichiometry="1"/>
  <speciesReference species="M_NADPH_c" stoichiometry="1"/>
  <speciesReference species="M_L_GLUT5P_c" stoichiometry="1"/>
</listOfReactants>
<listOfProducts>
  <speciesReference species="M_Pi_c" stoichiometry="1"/>
  <speciesReference species="M_NADP_c" stoichiometry="1"/>
  <speciesReference species="M_GLUT_SA_c" stoichiometry="1"/>
</listOfProducts>
<kineticLaw>
  <math xmlns="http://www.w3.org/1998/Math/MathML">
    <ci> FLUX_VALUE </ci>
  </math>
  <listOfParameters>
    <parameter id="LOWER_BOUND" value="0" units="mmol_per_gDW_per_hr" constant="true"/>
    <parameter id="UPPER_BOUND" value="1000" units="mmol_per_gDW_per_hr" constant="true"/>
    <parameter id="FLUX_VALUE" value="0" units="mmol_per_gDW_per_hr" constant="true"/>
    <parameter id="OBJECTIVE_COEFFICIENT" value="0" units="mmol_per_gDW_per_hr" constant="true"/>
  </listOfParameters>
</kineticLaw>
```

```
</reaction>
<reaction id="R_R334" name="ATP:L-glutamate 5-phosphotransferase" reversible="false">
 <notes>
  <body xmlns="http://www.w3.org/1999/xhtml">
   <p>GENE_ASSOCIATION: TTC1563</p>
   <p>SUBSYSTEM: Arginine and Proline Metabolism</p>
   <p>EC Number: 2.7.2.11</p>
   <p>Confidence Level: 1</p>
   <p>AUTHORS: Amino acid metabolism</p>
   <p/>
  </body>
 </notes>
 <listOfReactants>
  <speciesReference species="M_L_GLU_c" stoichiometry="1"/>
  <speciesReference species="M_ATP_c" stoichiometry="1"/>
 </listOfReactants>
 <listOfProducts>
  <speciesReference species="M_ADP_c" stoichiometry="1"/>
  <speciesReference species="M_L_GLUT5P_c" stoichiometry="1"/>
 </listOfProducts>
 <kineticLaw>
  <math xmlns="http://www.w3.org/1998/Math/MathML">
   <ci> FLUX_VALUE </ci>
  </math>
  <listOfParameters>
   <parameter id="LOWER_BOUND" value="0" units="mmol_per_gDW_per_hr" constant="true"/>
   <parameter id="UPPER_BOUND" value="1000" units="mmol_per_gDW_per_hr" constant="true"/>
   <parameter id="FLUX_VALUE" value="0" units="mmol_per_gDW_per_hr" constant="true"/>
   <parameter id="OBJECTIVE_COEFFICIENT" value="0" units="mmol_per_gDW_per_hr" constant="true"/>
```

```
    </listOfParameters>
  </kineticLaw>
</reaction>
<reaction id="R_R335" name="N2-Acetyl-L-ornithine:L-glutamate N-acetyltransferase" reversible="true">
  <notes>
    <body xmlns="http://www.w3.org/1999/xhtml">
      <p>GENE_ASSOCIATION: TTC0835</p>
      <p>SUBSYSTEM: Arginine and Proline Metabolism</p>
      <p>EC Number: 2.3.1.35</p>
      <p>Confidence Level: 1</p>
      <p>AUTHORS: Amino acid metabolism</p>
      <p/>
    </body>
  </notes>
  <listOfReactants>
    <speciesReference species="M_L_GLU_c" stoichiometry="1"/>
    <speciesReference species="M_AORT_c" stoichiometry="1"/>
  </listOfReactants>
  <listOfProducts>
    <speciesReference species="M_ORT_c" stoichiometry="1"/>
    <speciesReference species="M_AC_GLUT_c" stoichiometry="1"/>
  </listOfProducts>
  <kineticLaw>
    <math xmlns="http://www.w3.org/1998/Math/MathML">
      <ci> FLUX_VALUE </ci>
    </math>
    <listOfParameters>
      <parameter id="LOWER_BOUND" value="-1000" units="mmol_per_gDW_per_hr" constant="true"/>
      <parameter id="UPPER_BOUND" value="1000" units="mmol_per_gDW_per_hr" constant="true"/>
```

```
      <parameter id="FLUX_VALUE" value="0" units="mmol_per_gDW_per_hr" constant="true"/>
      <parameter id="OBJECTIVE_COEFFICIENT" value="0" units="mmol_per_gDW_per_hr" constant="true"/>
    </listOfParameters>
  </kineticLaw>
</reaction>
<reaction id="R_R336" name="N2-Acetyl-L-ornithine amidohydrolase" reversible="false">
  <notes>
    <body xmlns="http://www.w3.org/1999/xhtml">
      <p>GENE_ASSOCIATION: (TTC0133 or TTC1460)</p>
      <p>SUBSYSTEM: Arginine and Proline Metabolism</p>
      <p>EC Number: 3.5.1.14</p>
      <p>Confidence Level: 1</p>
      <p>AUTHORS: Amino acid metabolism</p>
      <p/>
    </body>
  </notes>
  <listOfReactants>
    <speciesReference species="M_H2O_c" stoichiometry="1"/>
    <speciesReference species="M_AORT_c" stoichiometry="1"/>
  </listOfReactants>
  <listOfProducts>
    <speciesReference species="M_ORT_c" stoichiometry="1"/>
    <speciesReference species="M_AC_c" stoichiometry="1"/>
  </listOfProducts>
  <kineticLaw>
    <math xmlns="http://www.w3.org/1998/Math/MathML">
      <ci> FLUX_VALUE </ci>
    </math>
    <listOfParameters>
```

```
      <parameter id="LOWER_BOUND" value="0" units="mmol_per_gDW_per_hr" constant="true"/>
      <parameter id="UPPER_BOUND" value="1000" units="mmol_per_gDW_per_hr" constant="true"/>
      <parameter id="FLUX_VALUE" value="0" units="mmol_per_gDW_per_hr" constant="true"/>
      <parameter id="OBJECTIVE_COEFFICIENT" value="0" units="mmol_per_gDW_per_hr" constant="true"/>
    </listOfParameters>
  </kineticLaw>
</reaction>
<reaction id="R_R337" name="Carbamoyl-phosphate:L-ornithine carbamoyltransferase" reversible="true">
  <notes>
    <body xmlns="http://www.w3.org/1999/xhtml">
      <p>GENE_ASSOCIATION: TTC0838</p>
      <p>SUBSYSTEM: Arginine and Proline Metabolism</p>
      <p>EC Number: 2.1.3.3</p>
      <p>Confidence Level: 1</p>
      <p>AUTHORS: Amino acid metabolism</p>
      <p/>
    </body>
  </notes>
  <listOfReactants>
    <speciesReference species="M_ORT_c" stoichiometry="1"/>
    <speciesReference species="M_CARB_Pi_c" stoichiometry="1"/>
  </listOfReactants>
  <listOfProducts>
    <speciesReference species="M_Pi_c" stoichiometry="1"/>
    <speciesReference species="M_H_c" stoichiometry="1"/>
    <speciesReference species="M_CTRLN_c" stoichiometry="1"/>
  </listOfProducts>
  <kineticLaw>
    <math xmlns="http://www.w3.org/1998/Math/MathML">
```

```xml
<ci> FLUX_VALUE </ci>
</math>
<listOfParameters>
<parameter id="LOWER_BOUND" value="-1000" units="mmol_per_gDW_per_hr" constant="true"/>
<parameter id="UPPER_BOUND" value="1000" units="mmol_per_gDW_per_hr" constant="true"/>
<parameter id="FLUX_VALUE" value="0" units="mmol_per_gDW_per_hr" constant="true"/>
<parameter id="OBJECTIVE_COEFFICIENT" value="0" units="mmol_per_gDW_per_hr" constant="true"/>
</listOfParameters>
</kineticLaw>
</reaction>
<reaction id="R_R338" name="L-Arginine amidinohydrolase" reversible="true">
<notes>
<body xmlns="http://www.w3.org/1999/xhtml">
<p>GENE_ASSOCIATION: TTC1132</p>
<p>SUBSYSTEM: Arginine and Proline Metabolism</p>
<p>EC Number: 3.5.3.1</p>
<p>Confidence Level: 1</p>
<p>AUTHORS: Amino acid metabolism</p>
<p/>
</body>
</notes>
<listOfReactants>
<speciesReference species="M_L_ARG_c" stoichiometry="1"/>
<speciesReference species="M_H2O_c" stoichiometry="1"/>
</listOfReactants>
<listOfProducts>
<speciesReference species="M_ORT_c" stoichiometry="1"/>
<speciesReference species="M_UREA_c" stoichiometry="1"/>
</listOfProducts>
```

```
  <kineticLaw>
    <math xmlns="http://www.w3.org/1998/Math/MathML">
      <ci> FLUX_VALUE </ci>
    </math>
    <listOfParameters>
      <parameter id="LOWER_BOUND" value="-1000" units="mmol_per_gDW_per_hr" constant="true"/>
      <parameter id="UPPER_BOUND" value="1000" units="mmol_per_gDW_per_hr" constant="true"/>
      <parameter id="FLUX_VALUE" value="0" units="mmol_per_gDW_per_hr" constant="true"/>
      <parameter id="OBJECTIVE_COEFFICIENT" value="0" units="mmol_per_gDW_per_hr" constant="true"/>
    </listOfParameters>
  </kineticLaw>
</reaction>
<reaction id="R_R339" name="urea-1-carboxylate amidohydrolase" reversible="false">
  <notes>
    <body xmlns="http://www.w3.org/1999/xhtml">
      <p>GENE_ASSOCIATION: TTC0624</p>
      <p>SUBSYSTEM: Arginine and Proline Metabolism</p>
      <p>EC Number: 3.5.1.54</p>
      <p>Confidence Level: 1</p>
      <p>AUTHORS: Amino acid metabolism</p>
      <p/>
    </body>
  </notes>
  <listOfReactants>
    <speciesReference species="M_H2O_c" stoichiometry="1"/>
    <speciesReference species="M_H_c" stoichiometry="1"/>
    <speciesReference species="M_UREA_CARB_c" stoichiometry="1"/>
  </listOfReactants>
  <listOfProducts>
```

```
    <speciesReference species="M_CO2_c" stoichiometry="2"/>
    <speciesReference species="M_NH4_c" stoichiometry="2"/>
  </listOfProducts>
  <kineticLaw>
    <math xmlns="http://www.w3.org/1998/Math/MathML">
      <ci> FLUX_VALUE </ci>
    </math>
    <listOfParameters>
      <parameter id="LOWER_BOUND" value="0" units="mmol_per_gDW_per_hr" constant="true"/>
      <parameter id="UPPER_BOUND" value="1000" units="mmol_per_gDW_per_hr" constant="true"/>
      <parameter id="FLUX_VALUE" value="0" units="mmol_per_gDW_per_hr" constant="true"/>
      <parameter id="OBJECTIVE_COEFFICIENT" value="0" units="mmol_per_gDW_per_hr" constant="true"/>
    </listOfParameters>
  </kineticLaw>
</reaction>
<reaction id="R_R340" name="L-Proline:NAD+ 5-oxidoreductase" reversible="true">
  <notes>
    <body xmlns="http://www.w3.org/1999/xhtml">
      <p>GENE_ASSOCIATION: TTC0500</p>
      <p>SUBSYSTEM: Arginine and Proline Metabolism</p>
      <p>EC Number: 1.5.1.2</p>
      <p>Confidence Level: 1</p>
      <p>AUTHORS: Amino acid metabolism</p>
      <p/>
    </body>
  </notes>
  <listOfReactants>
    <speciesReference species="M_L_PRO_c" stoichiometry="1"/>
    <speciesReference species="M_NAD_c" stoichiometry="1"/>
```

```xml
  </listOfReactants>
  <listOfProducts>
    <speciesReference species="M_H_c" stoichiometry="2"/>
    <speciesReference species="M_NADH_c" stoichiometry="1"/>
    <speciesReference species="M_PYRR5CARB_c" stoichiometry="1"/>
  </listOfProducts>
  <kineticLaw>
    <math xmlns="http://www.w3.org/1998/Math/MathML">
      <ci> FLUX_VALUE </ci>
    </math>
    <listOfParameters>
      <parameter id="LOWER_BOUND" value="-1000" units="mmol_per_gDW_per_hr" constant="true"/>
      <parameter id="UPPER_BOUND" value="1000" units="mmol_per_gDW_per_hr" constant="true"/>
      <parameter id="FLUX_VALUE" value="0" units="mmol_per_gDW_per_hr" constant="true"/>
      <parameter id="OBJECTIVE_COEFFICIENT" value="0" units="mmol_per_gDW_per_hr" constant="true"/>
    </listOfParameters>
  </kineticLaw>
</reaction>
<reaction id="R_R341" name="L-Proline:NADP+ 5-oxidoreductase" reversible="true">
  <notes>
    <body xmlns="http://www.w3.org/1999/xhtml">
      <p>GENE_ASSOCIATION: TTC0500</p>
      <p>SUBSYSTEM: Arginine and Proline Metabolism</p>
      <p>EC Number: 1.5.1.2</p>
      <p>Confidence Level: 1</p>
      <p>AUTHORS: Amino acid metabolism</p>
      <p/>
    </body>
  </notes>
```

```
  <listOfReactants>
    <speciesReference species="M_L_PRO_c" stoichiometry="1"/>
    <speciesReference species="M_NADP_c" stoichiometry="1"/>
  </listOfReactants>
  <listOfProducts>
    <speciesReference species="M_H_c" stoichiometry="2"/>
    <speciesReference species="M_NADPH_c" stoichiometry="1"/>
    <speciesReference species="M_PYRR5CARB_c" stoichiometry="1"/>
  </listOfProducts>
  <kineticLaw>
    <math xmlns="http://www.w3.org/1998/Math/MathML">
      <ci> FLUX_VALUE </ci>
    </math>
    <listOfParameters>
      <parameter id="LOWER_BOUND" value="-1000" units="mmol_per_gDW_per_hr" constant="true"/>
      <parameter id="UPPER_BOUND" value="1000" units="mmol_per_gDW_per_hr" constant="true"/>
      <parameter id="FLUX_VALUE" value="0" units="mmol_per_gDW_per_hr" constant="true"/>
      <parameter id="OBJECTIVE_COEFFICIENT" value="0" units="mmol_per_gDW_per_hr" constant="true"/>
    </listOfParameters>
  </kineticLaw>
</reaction>
<reaction id="R_R342" name="4-hydroxyproline degradation I " reversible="true">
  <notes>
    <body xmlns="http://www.w3.org/1999/xhtml">
      <p>GENE_ASSOCIATION: </p>
      <p>SUBSYSTEM: Arginine and Proline Metabolism</p>
      <p>EC Number: spontaneous</p>
      <p>Confidence Level: 1</p>
      <p>AUTHORS: Amino acid metabolism</p>
```

```
      <p/>
    </body>
  </notes>
  <listOfReactants>
    <speciesReference species="M_PYRR_3HY_5CARB_c" stoichiometry="1"/>
  </listOfReactants>
  <listOfProducts>
    <speciesReference species="M_H2O_c" stoichiometry="2"/>
    <speciesReference species="M_H_c" stoichiometry="2"/>
    <speciesReference species="M_HYGLU_SA_c" stoichiometry="1"/>
  </listOfProducts>
  <kineticLaw>
    <math xmlns="http://www.w3.org/1998/Math/MathML">
      <ci> FLUX_VALUE </ci>
    </math>
    <listOfParameters>
      <parameter id="LOWER_BOUND" value="-1000" units="mmol_per_gDW_per_hr" constant="true"/>
      <parameter id="UPPER_BOUND" value="1000" units="mmol_per_gDW_per_hr" constant="true"/>
      <parameter id="FLUX_VALUE" value="0" units="mmol_per_gDW_per_hr" constant="true"/>
      <parameter id="OBJECTIVE_COEFFICIENT" value="0" units="mmol_per_gDW_per_hr" constant="true"/>
    </listOfParameters>
  </kineticLaw>
</reaction>
<reaction id="R_R343" name="L-erythro-4-Hydroxyglutamate:NAD+ oxidoreductase" reversible="false">
  <notes>
    <body xmlns="http://www.w3.org/1999/xhtml">
      <p>GENE_ASSOCIATION: (TTC1213 or TTC1214)</p>
      <p>SUBSYSTEM: Arginine and Proline Metabolism</p>
      <p>EC Number: (1.5.1.12 or 1.5.99.8)</p>
```

```
      <p>Confidence Level: 1</p>
      <p>AUTHORS: Amino acid metabolism</p>
      <p/>
    </body>
  </notes>
  <listOfReactants>
    <speciesReference species="M_H2O_c" stoichiometry="1"/>
    <speciesReference species="M_NAD_c" stoichiometry="1"/>
    <speciesReference species="M_HYGLU_SA_c" stoichiometry="1"/>
  </listOfReactants>
  <listOfProducts>
    <speciesReference species="M_H_c" stoichiometry="1"/>
    <speciesReference species="M_NADH_c" stoichiometry="1"/>
    <speciesReference species="M_ERY_HYGLU_c" stoichiometry="1"/>
  </listOfProducts>
  <kineticLaw>
    <math xmlns="http://www.w3.org/1998/Math/MathML">
      <ci> FLUX_VALUE </ci>
    </math>
    <listOfParameters>
      <parameter id="LOWER_BOUND" value="0" units="mmol_per_gDW_per_hr" constant="true"/>
      <parameter id="UPPER_BOUND" value="1000" units="mmol_per_gDW_per_hr" constant="true"/>
      <parameter id="FLUX_VALUE" value="0" units="mmol_per_gDW_per_hr" constant="true"/>
      <parameter id="OBJECTIVE_COEFFICIENT" value="0" units="mmol_per_gDW_per_hr" constant="true"/>
    </listOfParameters>
  </kineticLaw>
</reaction>
<reaction id="R_R344" name="L-1-Pyrroline-3-hydroxy-5-carboxylate:NAD+ oxidoreductase" reversible="false">
  <notes>
```

```xml
<body xmlns="http://www.w3.org/1999/xhtml">
  <p>GENE_ASSOCIATION: TTC1213</p>
  <p>SUBSYSTEM: Arginine and Proline Metabolism</p>
  <p>EC Number: 1.5.1.12</p>
  <p>Confidence Level: 1</p>
  <p>AUTHORS: Amino acid metabolism</p>
  <p/>
</body>
</notes>
<listOfReactants>
  <speciesReference species="M_H2O_c" stoichiometry="2"/>
  <speciesReference species="M_NAD_c" stoichiometry="1"/>
  <speciesReference species="M_PYRR_3HY_5CARB_c" stoichiometry="1"/>
</listOfReactants>
<listOfProducts>
  <speciesReference species="M_H_c" stoichiometry="1"/>
  <speciesReference species="M_NADH_c" stoichiometry="1"/>
  <speciesReference species="M_ERY_HYGLU_c" stoichiometry="1"/>
</listOfProducts>
<kineticLaw>
  <math xmlns="http://www.w3.org/1998/Math/MathML">
    <ci> FLUX_VALUE </ci>
  </math>
  <listOfParameters>
    <parameter id="LOWER_BOUND" value="0" units="mmol_per_gDW_per_hr" constant="true"/>
    <parameter id="UPPER_BOUND" value="1000" units="mmol_per_gDW_per_hr" constant="true"/>
    <parameter id="FLUX_VALUE" value="0" units="mmol_per_gDW_per_hr" constant="true"/>
    <parameter id="OBJECTIVE_COEFFICIENT" value="0" units="mmol_per_gDW_per_hr" constant="true"/>
  </listOfParameters>
```

```
  </kineticLaw>
</reaction>
<reaction id="R_R345" name="putrescine:2-oxoglutarate aminotransferase" reversible="false">
  <notes>
    <body xmlns="http://www.w3.org/1999/xhtml">
      <p>GENE_ASSOCIATION: TTC0553</p>
      <p>SUBSYSTEM: Arginine and Proline Metabolism</p>
      <p>EC Number: 2.6.1.82</p>
      <p>Confidence Level: 1</p>
      <p>AUTHORS: Amino acid metabolism</p>
      <p/>
    </body>
  </notes>
  <listOfReactants>
    <speciesReference species="M_AKG_c" stoichiometry="1"/>
    <speciesReference species="M_PUT_c" stoichiometry="1"/>
  </listOfReactants>
  <listOfProducts>
    <speciesReference species="M_L_GLU_c" stoichiometry="1"/>
    <speciesReference species="M_4ABUTALD_c" stoichiometry="1"/>
  </listOfProducts>
  <kineticLaw>
    <math xmlns="http://www.w3.org/1998/Math/MathML">
      <ci> FLUX_VALUE </ci>
    </math>
    <listOfParameters>
      <parameter id="LOWER_BOUND" value="0" units="mmol_per_gDW_per_hr" constant="true"/>
      <parameter id="UPPER_BOUND" value="1000" units="mmol_per_gDW_per_hr" constant="true"/>
      <parameter id="FLUX_VALUE" value="0" units="mmol_per_gDW_per_hr" constant="true"/>
```

```
      <parameter id="OBJECTIVE_COEFFICIENT" value="0" units="mmol_per_gDW_per_hr" constant="true"/>
    </listOfParameters>
  </kineticLaw>
</reaction>
<reaction id="R_R346" name="4-aminobutanal:NAD+ 1-oxidoreductase" reversible="false">
  <notes>
    <body xmlns="http://www.w3.org/1999/xhtml">
      <p>GENE_ASSOCIATION: TTC0513</p>
      <p>SUBSYSTEM: Arginine and Proline Metabolism</p>
      <p>EC Number: 1.2.1.3</p>
      <p>Confidence Level: 1</p>
      <p>AUTHORS: Amino acid metabolism</p>
      <p/>
    </body>
  </notes>
  <listOfReactants>
    <speciesReference species="M_H2O_c" stoichiometry="1"/>
    <speciesReference species="M_NAD_c" stoichiometry="1"/>
    <speciesReference species="M_4ABUTALD_c" stoichiometry="1"/>
  </listOfReactants>
  <listOfProducts>
    <speciesReference species="M_H_c" stoichiometry="2"/>
    <speciesReference species="M_NADH_c" stoichiometry="1"/>
    <speciesReference species="M_4ABUT_c" stoichiometry="1"/>
  </listOfProducts>
  <kineticLaw>
    <math xmlns="http://www.w3.org/1998/Math/MathML">
      <ci> FLUX_VALUE </ci>
    </math>
```

```xml
    <listOfParameters>
      <parameter id="LOWER_BOUND" value="0" units="mmol_per_gDW_per_hr" constant="true"/>
      <parameter id="UPPER_BOUND" value="1000" units="mmol_per_gDW_per_hr" constant="true"/>
      <parameter id="FLUX_VALUE" value="0" units="mmol_per_gDW_per_hr" constant="true"/>
      <parameter id="OBJECTIVE_COEFFICIENT" value="0" units="mmol_per_gDW_per_hr" constant="true"/>
    </listOfParameters>
  </kineticLaw>
</reaction>
<reaction id="R_R347" name="trans-4-Hydroxy-L-proline:NAD+ 5-oxidoreductase" reversible="true">
  <notes>
    <body xmlns="http://www.w3.org/1999/xhtml">
      <p>GENE_ASSOCIATION: TTC0500</p>
      <p>SUBSYSTEM: Arginine and Proline Metabolism</p>
      <p>EC Number: 1.5.1.2</p>
      <p>Confidence Level: 1</p>
      <p>AUTHORS: Amino acid metabolism</p>
      <p/>
    </body>
  </notes>
  <listOfReactants>
    <speciesReference species="M_H_c" stoichiometry="1"/>
    <speciesReference species="M_NADH_c" stoichiometry="1"/>
    <speciesReference species="M_PYRR_3HY_5CARB_c" stoichiometry="1"/>
  </listOfReactants>
  <listOfProducts>
    <speciesReference species="M_NAD_c" stoichiometry="1"/>
    <speciesReference species="M_tHY_PRO_c" stoichiometry="1"/>
  </listOfProducts>
  <kineticLaw>
```

```
    <math xmlns="http://www.w3.org/1998/Math/MathML">
     <ci> FLUX_VALUE </ci>
    </math>
    <listOfParameters>
     <parameter id="LOWER_BOUND" value="-1000" units="mmol_per_gDW_per_hr" constant="true"/>
     <parameter id="UPPER_BOUND" value="1000" units="mmol_per_gDW_per_hr" constant="true"/>
     <parameter id="FLUX_VALUE" value="0" units="mmol_per_gDW_per_hr" constant="true"/>
     <parameter id="OBJECTIVE_COEFFICIENT" value="0" units="mmol_per_gDW_per_hr" constant="true"/>
    </listOfParameters>
   </kineticLaw>
  </reaction>
  <reaction id="R_R348" name="trans-4-Hydroxy-L-proline:NADP+ 5-oxidoreductase" reversible="true">
   <notes>
    <body xmlns="http://www.w3.org/1999/xhtml">
     <p>GENE_ASSOCIATION: TTC0500</p>
     <p>SUBSYSTEM: Arginine and Proline Metabolism</p>
     <p>EC Number: 1.5.1.2</p>
     <p>Confidence Level: 1</p>
     <p>AUTHORS: Amino acid metabolism</p>
     <p/>
    </body>
   </notes>
   <listOfReactants>
    <speciesReference species="M_H_c" stoichiometry="1"/>
    <speciesReference species="M_NADPH_c" stoichiometry="1"/>
    <speciesReference species="M_PYRR_3HY_5CARB_c" stoichiometry="1"/>
   </listOfReactants>
   <listOfProducts>
    <speciesReference species="M_NADP_c" stoichiometry="1"/>
```

```xml
    <speciesReference species="M_tHY_PRO_c" stoichiometry="1"/>
  </listOfProducts>
  <kineticLaw>
    <math xmlns="http://www.w3.org/1998/Math/MathML">
      <ci> FLUX_VALUE </ci>
    </math>
    <listOfParameters>
      <parameter id="LOWER_BOUND" value="-1000" units="mmol_per_gDW_per_hr" constant="true"/>
      <parameter id="UPPER_BOUND" value="1000" units="mmol_per_gDW_per_hr" constant="true"/>
      <parameter id="FLUX_VALUE" value="0" units="mmol_per_gDW_per_hr" constant="true"/>
      <parameter id="OBJECTIVE_COEFFICIENT" value="0" units="mmol_per_gDW_per_hr" constant="true"/>
    </listOfParameters>
  </kineticLaw>
</reaction>
<reaction id="R_R349" name="proline 4-hydroxylase" reversible="true">
  <notes>
    <body xmlns="http://www.w3.org/1999/xhtml">
      <p>GENE_ASSOCIATION: </p>
      <p>SUBSYSTEM: Arginine and Proline Metabolism</p>
      <p>EC Number: 1.14.11.-</p>
      <p>Confidence Level: 1</p>
      <p>AUTHORS: Amino acid metabolism</p>
      <p/>
    </body>
  </notes>
  <listOfReactants>
    <speciesReference species="M_L_PRO_c" stoichiometry="1"/>
    <speciesReference species="M_AKG_c" stoichiometry="1"/>
    <speciesReference species="M_O2_c" stoichiometry="1"/>
```

```xml
</listOfReactants>
<listOfProducts>
<speciesReference species="M_CO2_c" stoichiometry="1"/>
<speciesReference species="M_SUCC_c" stoichiometry="1"/>
<speciesReference species="M_tHY_PRO_c" stoichiometry="1"/>
</listOfProducts>
<kineticLaw>
<math xmlns="http://www.w3.org/1998/Math/MathML">
<ci> FLUX_VALUE </ci>
</math>
<listOfParameters>
<parameter id="LOWER_BOUND" value="-1000" units="mmol_per_gDW_per_hr" constant="true"/>
<parameter id="UPPER_BOUND" value="1000" units="mmol_per_gDW_per_hr" constant="true"/>
<parameter id="FLUX_VALUE" value="0" units="mmol_per_gDW_per_hr" constant="true"/>
<parameter id="OBJECTIVE_COEFFICIENT" value="0" units="mmol_per_gDW_per_hr" constant="true"/>
</listOfParameters>
</kineticLaw>
</reaction>
<reaction id="R_R350" name="L-erythro-4-hydroxyglutamate:2-oxoglutarate aminotransferase" reversible="false">
<notes>
<body xmlns="http://www.w3.org/1999/xhtml">
<p>GENE_ASSOCIATION: TTC1960</p>
<p>SUBSYSTEM: Arginine and Proline Metabolism</p>
<p>EC Number: 2.6.1.1</p>
<p>Confidence Level: 1</p>
<p>AUTHORS: Amino acid metabolism</p>
<p/>
</body>
</notes>
```

```
  <listOfReactants>
    <speciesReference species="M_AKG_c" stoichiometry="1"/>
    <speciesReference species="M_ERY_HYGLU_c" stoichiometry="1"/>
  </listOfReactants>
  <listOfProducts>
    <speciesReference species="M_L_GLU_c" stoichiometry="1"/>
    <speciesReference species="M_HY_OGLUTR_c" stoichiometry="1"/>
  </listOfProducts>
  <kineticLaw>
    <math xmlns="http://www.w3.org/1998/Math/MathML">
      <ci> FLUX_VALUE </ci>
    </math>
    <listOfParameters>
      <parameter id="LOWER_BOUND" value="0" units="mmol_per_gDW_per_hr" constant="true"/>
      <parameter id="UPPER_BOUND" value="1000" units="mmol_per_gDW_per_hr" constant="true"/>
      <parameter id="FLUX_VALUE" value="0" units="mmol_per_gDW_per_hr" constant="true"/>
      <parameter id="OBJECTIVE_COEFFICIENT" value="0" units="mmol_per_gDW_per_hr" constant="true"/>
    </listOfParameters>
  </kineticLaw>
</reaction>
<reaction id="R_R351" name="D-4-Hydroxy-2-oxoglutarate glyoxylate-lyase" reversible="false">
  <notes>
    <body xmlns="http://www.w3.org/1999/xhtml">
      <p>GENE_ASSOCIATION: TT_P0030</p>
      <p>SUBSYSTEM: Arginine and Proline Metabolism</p>
      <p>EC Number:  4.1.2.14</p>
      <p>Confidence Level: 1</p>
      <p>AUTHORS: Amino acid metabolism</p>
      <p/>
```

```
    </body>
   </notes>
   <listOfReactants>
    <speciesReference species="M_HY_OGLUTR_c" stoichiometry="1"/>
   </listOfReactants>
   <listOfProducts>
    <speciesReference species="M_PYR_c" stoichiometry="1"/>
    <speciesReference species="M_GLOXT_c" stoichiometry="1"/>
   </listOfProducts>
   <kineticLaw>
    <math xmlns="http://www.w3.org/1998/Math/MathML">
     <ci> FLUX_VALUE </ci>
    </math>
    <listOfParameters>
     <parameter id="LOWER_BOUND" value="0" units="mmol_per_gDW_per_hr" constant="true"/>
     <parameter id="UPPER_BOUND" value="1000" units="mmol_per_gDW_per_hr" constant="true"/>
     <parameter id="FLUX_VALUE" value="0" units="mmol_per_gDW_per_hr" constant="true"/>
     <parameter id="OBJECTIVE_COEFFICIENT" value="0" units="mmol_per_gDW_per_hr" constant="true"/>
    </listOfParameters>
   </kineticLaw>
  </reaction>
  <reaction id="R_R352" name="Agmatine amidinohydrolase" reversible="false">
   <notes>
    <body xmlns="http://www.w3.org/1999/xhtml">
     <p>GENE_ASSOCIATION: TTC0764</p>
     <p>SUBSYSTEM: Arginine and Proline Metabolism</p>
     <p>EC Number: 3.5.3.11</p>
     <p>Confidence Level: 1</p>
     <p>AUTHORS: Amino acid metabolism</p>
```

```
      <p/>
    </body>
  </notes>
  <listOfReactants>
    <speciesReference species="M_H2O_c" stoichiometry="1"/>
    <speciesReference species="M_AGMT_c" stoichiometry="1"/>
  </listOfReactants>
  <listOfProducts>
    <speciesReference species="M_PUT_c" stoichiometry="1"/>
    <speciesReference species="M_UREA_c" stoichiometry="1"/>
  </listOfProducts>
  <kineticLaw>
    <math xmlns="http://www.w3.org/1998/Math/MathML">
      <ci> FLUX_VALUE </ci>
    </math>
    <listOfParameters>
      <parameter id="LOWER_BOUND" value="0" units="mmol_per_gDW_per_hr" constant="true"/>
      <parameter id="UPPER_BOUND" value="1000" units="mmol_per_gDW_per_hr" constant="true"/>
      <parameter id="FLUX_VALUE" value="0" units="mmol_per_gDW_per_hr" constant="true"/>
      <parameter id="OBJECTIVE_COEFFICIENT" value="0" units="mmol_per_gDW_per_hr" constant="true"/>
    </listOfParameters>
  </kineticLaw>
</reaction>
<reaction id="R_R353" name="L-arginine carboxy-lyase (agmatine-forming)" reversible="false">
  <notes>
    <body xmlns="http://www.w3.org/1999/xhtml">
      <p>GENE_ASSOCIATION: TTC1277</p>
      <p>SUBSYSTEM: Arginine and Proline Metabolism</p>
      <p>EC Number: 4.1.1.19</p>
```

```xml
<p>Confidence Level: 1</p>

<p>AUTHORS: Amino acid metabolism</p>

<p/>

</body>

</notes>

<listOfReactants>

<speciesReference species="M_L_ARG_c" stoichiometry="1"/>

<speciesReference species="M_H_c" stoichiometry="1"/>

</listOfReactants>

<listOfProducts>

<speciesReference species="M_CO2_c" stoichiometry="1"/>

<speciesReference species="M_AGMT_c" stoichiometry="1"/>

</listOfProducts>

<kineticLaw>

<math xmlns="http://www.w3.org/1998/Math/MathML">

<ci> FLUX_VALUE </ci>

</math>

<listOfParameters>

<parameter id="LOWER_BOUND" value="0" units="mmol_per_gDW_per_hr" constant="true"/>

<parameter id="UPPER_BOUND" value="1000" units="mmol_per_gDW_per_hr" constant="true"/>

<parameter id="FLUX_VALUE" value="0" units="mmol_per_gDW_per_hr" constant="true"/>

<parameter id="OBJECTIVE_COEFFICIENT" value="0" units="mmol_per_gDW_per_hr" constant="true"/>

</listOfParameters>

</kineticLaw>

</reaction>

<reaction id="R_R354" name="2-Dehydro-3-deoxy-D-arabino-heptonate 7-phosphate phosphate-lyase (cyclyzing)" reversible="false">

<notes>

<body xmlns="http://www.w3.org/1999/xhtml">
```

```
      <p>GENE_ASSOCIATION: TTC1020</p>
      <p>SUBSYSTEM: Tyrosine, Tryptophan, and Phenylalanine Metabolism</p>
      <p>EC Number: 4.2.3.4</p>
      <p>Confidence Level: 1</p>
      <p>AUTHORS: Amino acid metabolism</p>
      <p/>
    </body>
  </notes>
  <listOfReactants>
    <speciesReference species="M_DHYDO_ARAB_HEPTP_c" stoichiometry="1"/>
  </listOfReactants>
  <listOfProducts>
    <speciesReference species="M_Pi_c" stoichiometry="1"/>
    <speciesReference species="M_3DHYQNT_c" stoichiometry="1"/>
  </listOfProducts>
  <kineticLaw>
    <math xmlns="http://www.w3.org/1998/Math/MathML">
      <ci> FLUX_VALUE </ci>
    </math>
    <listOfParameters>
      <parameter id="LOWER_BOUND" value="0" units="mmol_per_gDW_per_hr" constant="true"/>
      <parameter id="UPPER_BOUND" value="1000" units="mmol_per_gDW_per_hr" constant="true"/>
      <parameter id="FLUX_VALUE" value="0" units="mmol_per_gDW_per_hr" constant="true"/>
      <parameter id="OBJECTIVE_COEFFICIENT" value="0" units="mmol_per_gDW_per_hr" constant="true"/>
    </listOfParameters>
  </kineticLaw>
</reaction>
<reaction id="R_R355" name="3-Dehydroquinate hydro-lyase" reversible="false">
  <notes>
```

```
    <body xmlns="http://www.w3.org/1999/xhtml">
      <p>GENE_ASSOCIATION: TTC0989</p>
      <p>SUBSYSTEM: Tyrosine, Tryptophan, and Phenylalanine Metabolism</p>
      <p>EC Number: 4.2.1.10</p>
      <p>Confidence Level: 1</p>
      <p>AUTHORS: Amino acid metabolism</p>
      <p/>
    </body>
  </notes>
  <listOfReactants>
    <speciesReference species="M_3DHYQNT_c" stoichiometry="1"/>
  </listOfReactants>
  <listOfProducts>
    <speciesReference species="M_H2O_c" stoichiometry="1"/>
    <speciesReference species="M_DHY_SHK_c" stoichiometry="1"/>
  </listOfProducts>
  <kineticLaw>
    <math xmlns="http://www.w3.org/1998/Math/MathML">
      <ci> FLUX_VALUE </ci>
    </math>
    <listOfParameters>
      <parameter id="LOWER_BOUND" value="0" units="mmol_per_gDW_per_hr" constant="true"/>
      <parameter id="UPPER_BOUND" value="1000" units="mmol_per_gDW_per_hr" constant="true"/>
      <parameter id="FLUX_VALUE" value="0" units="mmol_per_gDW_per_hr" constant="true"/>
      <parameter id="OBJECTIVE_COEFFICIENT" value="0" units="mmol_per_gDW_per_hr" constant="true"/>
    </listOfParameters>
  </kineticLaw>
</reaction>
<reaction id="R_R356" name="ATP:shikimate 3-phosphotransferase" reversible="false">
```

```xml
<notes>
  <body xmlns="http://www.w3.org/1999/xhtml">
    <p>GENE_ASSOCIATION: TTC1019</p>
    <p>SUBSYSTEM: Tyrosine, Tryptophan, and Phenylalanine Metabolism</p>
    <p>EC Number: 2.7.1.71</p>
    <p>Confidence Level: 1</p>
    <p>AUTHORS: Amino acid metabolism</p>
    <p/>
  </body>
</notes>
<listOfReactants>
  <speciesReference species="M_ATP_c" stoichiometry="1"/>
  <speciesReference species="M_SHK_c" stoichiometry="1"/>
</listOfReactants>
<listOfProducts>
  <speciesReference species="M_ADP_c" stoichiometry="1"/>
  <speciesReference species="M_H_c" stoichiometry="1"/>
  <speciesReference species="M_SHKP_c" stoichiometry="1"/>
</listOfProducts>
<kineticLaw>
  <math xmlns="http://www.w3.org/1998/Math/MathML">
    <ci> FLUX_VALUE </ci>
  </math>
  <listOfParameters>
    <parameter id="LOWER_BOUND" value="0" units="mmol_per_gDW_per_hr" constant="true"/>
    <parameter id="UPPER_BOUND" value="1000" units="mmol_per_gDW_per_hr" constant="true"/>
    <parameter id="FLUX_VALUE" value="0" units="mmol_per_gDW_per_hr" constant="true"/>
    <parameter id="OBJECTIVE_COEFFICIENT" value="0" units="mmol_per_gDW_per_hr" constant="true"/>
  </listOfParameters>
```

```
    </kineticLaw>
   </reaction>
   <reaction id="R_R357" name="Phosphoenolpyruvate:3-phosphoshikimate 5-O-(1-carboxyvinyl)-transferase"
reversible="true">
    <notes>
     <body xmlns="http://www.w3.org/1999/xhtml">
      <p>GENE_ASSOCIATION: TTC0088</p>
      <p>SUBSYSTEM: Tyrosine, Tryptophan, and Phenylalanine Metabolism</p>
      <p>EC Number: 2.5.1.19</p>
      <p>Confidence Level: 1</p>
      <p>AUTHORS: Amino acid metabolism</p>
      <p/>
     </body>
    </notes>
    <listOfReactants>
     <speciesReference species="M_PEP_c" stoichiometry="1"/>
     <speciesReference species="M_SHKP_c" stoichiometry="1"/>
    </listOfReactants>
    <listOfProducts>
     <speciesReference species="M_Pi_c" stoichiometry="1"/>
     <speciesReference species="M_CVP_SHK_c" stoichiometry="1"/>
    </listOfProducts>
    <kineticLaw>
     <math xmlns="http://www.w3.org/1998/Math/MathML">
      <ci> FLUX_VALUE </ci>
     </math>
     <listOfParameters>
      <parameter id="LOWER_BOUND" value="-1000" units="mmol_per_gDW_per_hr" constant="true"/>
      <parameter id="UPPER_BOUND" value="1000" units="mmol_per_gDW_per_hr" constant="true"/>
```

<parameter id="FLUX_VALUE" value="0" units="mmol_per_gDW_per_hr" constant="true"/>

<parameter id="OBJECTIVE_COEFFICIENT" value="0" units="mmol_per_gDW_per_hr" constant="true"/>

</listOfParameters>

</kineticLaw>

</reaction>

<reaction id="R_R358" name="5-O-(1-Carboxyvinyl)-3-phosphoshikimate phosphate-lyase (chorismate-forming)" reversible="false">

<notes>

<body xmlns="http://www.w3.org/1999/xhtml">

<p>GENE_ASSOCIATION: (TTC0518 or TTC1018)</p>

<p>SUBSYSTEM: Tyrosine, Tryptophan, and Phenylalanine Metabolism</p>

<p>EC Number: 4.2.3.5</p>

<p>Confidence Level: 1</p>

<p>AUTHORS: Amino acid metabolism</p>

<p/>

</body>

</notes>

<listOfReactants>

<speciesReference species="M_CVP_SHK_c" stoichiometry="1"/>

</listOfReactants>

<listOfProducts>

<speciesReference species="M_Pi_c" stoichiometry="1"/>

<speciesReference species="M_CORM_c" stoichiometry="1"/>

</listOfProducts>

<kineticLaw>

<math xmlns="http://www.w3.org/1998/Math/MathML">

<ci> FLUX_VALUE </ci>

</math>

<listOfParameters>

```xml
<parameter id="LOWER_BOUND" value="0" units="mmol_per_gDW_per_hr" constant="true"/>
<parameter id="UPPER_BOUND" value="1000" units="mmol_per_gDW_per_hr" constant="true"/>
<parameter id="FLUX_VALUE" value="0" units="mmol_per_gDW_per_hr" constant="true"/>
<parameter id="OBJECTIVE_COEFFICIENT" value="0" units="mmol_per_gDW_per_hr" constant="true"/>
</listOfParameters>
</kineticLaw>
</reaction>
<reaction id="R_R359" name="Chorismate pyruvate-lyase (amino-accepting)" reversible="false">
<notes>
<body xmlns="http://www.w3.org/1999/xhtml">
<p>GENE_ASSOCIATION: (TTC1492 and TTC1493)</p>
<p>SUBSYSTEM: Tyrosine, Tryptophan, and Phenylalanine Metabolism</p>
<p>EC Number: 4.1.3.27</p>
<p>Confidence Level: 1</p>
<p>AUTHORS: Amino acid metabolism</p>
<p/>
</body>
</notes>
<listOfReactants>
<speciesReference species="M_L_GLN_c" stoichiometry="1"/>
<speciesReference species="M_CORM_c" stoichiometry="1"/>
</listOfReactants>
<listOfProducts>
<speciesReference species="M_L_GLU_c" stoichiometry="1"/>
<speciesReference species="M_H_c" stoichiometry="1"/>
<speciesReference species="M_PYR_c" stoichiometry="1"/>
<speciesReference species="M_ANTHRL_c" stoichiometry="1"/>
</listOfProducts>
<kineticLaw>
```

```xml
<math xmlns="http://www.w3.org/1998/Math/MathML">
  <ci> FLUX_VALUE </ci>
</math>
<listOfParameters>
  <parameter id="LOWER_BOUND" value="0" units="mmol_per_gDW_per_hr" constant="true"/>
  <parameter id="UPPER_BOUND" value="1000" units="mmol_per_gDW_per_hr" constant="true"/>
  <parameter id="FLUX_VALUE" value="0" units="mmol_per_gDW_per_hr" constant="true"/>
  <parameter id="OBJECTIVE_COEFFICIENT" value="0" units="mmol_per_gDW_per_hr" constant="true"/>
</listOfParameters>
</kineticLaw>
</reaction>
<reaction id="R_R360" name="L-serine hydro-lyase (adding indole" reversible="false">
 <notes>
  <body xmlns="http://www.w3.org/1999/xhtml">
   <p>GENE_ASSOCIATION: (TTC0729 and TTC0730)</p>
   <p>SUBSYSTEM: Tyrosine, Tryptophan, and Phenylalanine Metabolism</p>
   <p>EC Number: 4.2.1.20</p>
   <p>Confidence Level: 1</p>
   <p>AUTHORS: Amino acid metabolism</p>
   <p/>
  </body>
 </notes>
 <listOfReactants>
  <speciesReference species="M_L_SER_c" stoichiometry="1"/>
  <speciesReference species="M_IND_c" stoichiometry="1"/>
 </listOfReactants>
 <listOfProducts>
  <speciesReference species="M_L_TRP_c" stoichiometry="1"/>
  <speciesReference species="M_H2O_c" stoichiometry="1"/>
```

```xml
</listOfProducts>

<kineticLaw>

<math xmlns="http://www.w3.org/1998/Math/MathML">

<ci> FLUX_VALUE </ci>

</math>

<listOfParameters>

<parameter id="LOWER_BOUND" value="0" units="mmol_per_gDW_per_hr" constant="true"/>

<parameter id="UPPER_BOUND" value="1000" units="mmol_per_gDW_per_hr" constant="true"/>

<parameter id="FLUX_VALUE" value="0" units="mmol_per_gDW_per_hr" constant="true"/>

<parameter id="OBJECTIVE_COEFFICIENT" value="0" units="mmol_per_gDW_per_hr" constant="true"/>

</listOfParameters>

</kineticLaw>

</reaction>

<reaction id="R_R361" name="(1S,2R)-1-C-(indol-3-yl)glycerol 3-phosphate D-glyceraldehyde-3-phosphate-lyase" reversible="false">

<notes>

<body xmlns="http://www.w3.org/1999/xhtml">

<p>GENE_ASSOCIATION: (TTC0729 and TTC0730)</p>

<p>SUBSYSTEM: Tyrosine, Tryptophan, and Phenylalanine Metabolism</p>

<p>EC Number: 4.2.1.20</p>

<p>Confidence Level: 1</p>

<p>AUTHORS: Amino acid metabolism</p>

<p/>

</body>

</notes>

<listOfReactants>

<speciesReference species="M_IND_GLYP_c" stoichiometry="1"/>

</listOfReactants>

<listOfProducts>
```

```
<speciesReference species="M_GAP_c" stoichiometry="1"/>
<speciesReference species="M_IND_c" stoichiometry="1"/>
</listOfProducts>
<kineticLaw>
<math xmlns="http://www.w3.org/1998/Math/MathML">
<ci> FLUX_VALUE </ci>
</math>
<listOfParameters>
<parameter id="LOWER_BOUND" value="0" units="mmol_per_gDW_per_hr" constant="true"/>
<parameter id="UPPER_BOUND" value="1000" units="mmol_per_gDW_per_hr" constant="true"/>
<parameter id="FLUX_VALUE" value="0" units="mmol_per_gDW_per_hr" constant="true"/>
<parameter id="OBJECTIVE_COEFFICIENT" value="0" units="mmol_per_gDW_per_hr" constant="true"/>
</listOfParameters>
</kineticLaw>
</reaction>
<reaction id="R_R362" name="1-(2-Carboxyphenylamino)-1-deoxy-D-ribulose-5-phosphate carboxy-lyase(cyclizing)"
reversible="false">
<notes>
<body xmlns="http://www.w3.org/1999/xhtml">
<p>GENE_ASSOCIATION: TTC0800</p>
<p>SUBSYSTEM: Tyrosine, Tryptophan, and Phenylalanine Metabolism</p>
<p>EC Number: 4.1.1.48</p>
<p>Confidence Level: 1</p>
<p>AUTHORS: Amino acid metabolism</p>
<p/>
</body>
</notes>
<listOfReactants>
<speciesReference species="M_H_c" stoichiometry="1"/>
```

```
    <speciesReference species="M_CPhA_dRBL5P_c" stoichiometry="1"/>
  </listOfReactants>
  <listOfProducts>
    <speciesReference species="M_H2O_c" stoichiometry="1"/>
    <speciesReference species="M_CO2_c" stoichiometry="1"/>
    <speciesReference species="M_IND_GLYP_c" stoichiometry="1"/>
  </listOfProducts>
  <kineticLaw>
    <math xmlns="http://www.w3.org/1998/Math/MathML">
      <ci> FLUX_VALUE </ci>
    </math>
    <listOfParameters>
      <parameter id="LOWER_BOUND" value="0" units="mmol_per_gDW_per_hr" constant="true"/>
      <parameter id="UPPER_BOUND" value="1000" units="mmol_per_gDW_per_hr" constant="true"/>
      <parameter id="FLUX_VALUE" value="0" units="mmol_per_gDW_per_hr" constant="true"/>
      <parameter id="OBJECTIVE_COEFFICIENT" value="0" units="mmol_per_gDW_per_hr" constant="true"/>
    </listOfParameters>
  </kineticLaw>
</reaction>
<reaction id="R_R363" name="N-(5-Phospho-beta-D-ribosyl)anthranilate ketol-isomerase" reversible="true">
  <notes>
    <body xmlns="http://www.w3.org/1999/xhtml">
      <p>GENE_ASSOCIATION: TTC1929</p>
      <p>SUBSYSTEM: Tyrosine, Tryptophan, and Phenylalanine Metabolism</p>
      <p>EC Number: 5.3.1.24</p>
      <p>Confidence Level: 1</p>
      <p>AUTHORS: Amino acid metabolism</p>
      <p/>
    </body>
```

```xml
</notes>
<listOfReactants>
  <speciesReference species="M_5PRIBS_ANTRL_c" stoichiometry="1"/>
</listOfReactants>
<listOfProducts>
  <speciesReference species="M_CPhA_dRBL5P_c" stoichiometry="1"/>
</listOfProducts>
<kineticLaw>
  <math xmlns="http://www.w3.org/1998/Math/MathML">
    <ci> FLUX_VALUE </ci>
  </math>
  <listOfParameters>
    <parameter id="LOWER_BOUND" value="-1000" units="mmol_per_gDW_per_hr" constant="true"/>
    <parameter id="UPPER_BOUND" value="1000" units="mmol_per_gDW_per_hr" constant="true"/>
    <parameter id="FLUX_VALUE" value="0" units="mmol_per_gDW_per_hr" constant="true"/>
    <parameter id="OBJECTIVE_COEFFICIENT" value="0" units="mmol_per_gDW_per_hr" constant="true"/>
  </listOfParameters>
</kineticLaw>
</reaction>
<reaction id="R_R364" name="N-(5-Phospho-D-ribosyl)anthranilate:pyrophosphate phosphoribosyl-transferase" reversible="true">
<notes>
  <body xmlns="http://www.w3.org/1999/xhtml">
    <p>GENE_ASSOCIATION: TTC1491</p>
    <p>SUBSYSTEM: Tyrosine, Tryptophan, and Phenylalanine Metabolism</p>
    <p>EC Number: 2.4.2.18</p>
    <p>Confidence Level: 1</p>
    <p>AUTHORS: Amino acid metabolism</p>
    <p/>
```

```xml
    </body>
  </notes>
  <listOfReactants>
    <speciesReference species="M_PPi_c" stoichiometry="1"/>
    <speciesReference species="M_5PRIBS_ANTRL_c" stoichiometry="1"/>
  </listOfReactants>
  <listOfProducts>
    <speciesReference species="M_PRPP_c" stoichiometry="1"/>
    <speciesReference species="M_ANTHRL_c" stoichiometry="1"/>
  </listOfProducts>
  <kineticLaw>
    <math xmlns="http://www.w3.org/1998/Math/MathML">
      <ci> FLUX_VALUE </ci>
    </math>
    <listOfParameters>
      <parameter id="LOWER_BOUND" value="-1000" units="mmol_per_gDW_per_hr" constant="true"/>
      <parameter id="UPPER_BOUND" value="1000" units="mmol_per_gDW_per_hr" constant="true"/>
      <parameter id="FLUX_VALUE" value="0" units="mmol_per_gDW_per_hr" constant="true"/>
      <parameter id="OBJECTIVE_COEFFICIENT" value="0" units="mmol_per_gDW_per_hr" constant="true"/>
    </listOfParameters>
  </kineticLaw>
</reaction>
<reaction id="R_R365" name="Chorismate pyruvatemutase" reversible="false">
  <notes>
    <body xmlns="http://www.w3.org/1999/xhtml">
      <p>GENE_ASSOCIATION: (TTC0020 or TTC0514)</p>
      <p>SUBSYSTEM: Tyrosine, Tryptophan, and Phenylalanine Metabolism</p>
      <p>EC Number: 5.4.99.5</p>
      <p>Confidence Level: 1</p>
```

```xml
<p>AUTHORS: Amino acid metabolism</p>
<p/>
</body>
</notes>
<listOfReactants>
<speciesReference species="M_CORM_c" stoichiometry="1"/>
</listOfReactants>
<listOfProducts>
<speciesReference species="M_PPHA_c" stoichiometry="1"/>
</listOfProducts>
<kineticLaw>
<math xmlns="http://www.w3.org/1998/Math/MathML">
<ci> FLUX_VALUE </ci>
</math>
<listOfParameters>
<parameter id="LOWER_BOUND" value="0" units="mmol_per_gDW_per_hr" constant="true"/>
<parameter id="UPPER_BOUND" value="1000" units="mmol_per_gDW_per_hr" constant="true"/>
<parameter id="FLUX_VALUE" value="0" units="mmol_per_gDW_per_hr" constant="true"/>
<parameter id="OBJECTIVE_COEFFICIENT" value="0" units="mmol_per_gDW_per_hr" constant="true"/>
</listOfParameters>
</kineticLaw>
</reaction>
<reaction id="R_R366" name="Phosphoenolpyruvate:D-erythrose-4-phosphate C-(1-carboxyvinyl)transferase (phosphate hydrolysing, 2-carboxy-2-oxoethyl-forming)" reversible="false">
<notes>
<body xmlns="http://www.w3.org/1999/xhtml">
<p>GENE_ASSOCIATION: (TTC0020 or TTC0448)</p>
<p>SUBSYSTEM: Tyrosine, Tryptophan, and Phenylalanine Metabolism</p>
<p>EC Number: 2.5.1.54</p>
```

```xml
<p>Confidence Level: 1</p>
<p>AUTHORS: Amino acid metabolism</p>
<p/>
</body>
</notes>
<listOfReactants>
<speciesReference species="M_H2O_c" stoichiometry="1"/>
<speciesReference species="M_PEP_c" stoichiometry="1"/>
<speciesReference species="M_E4P_c" stoichiometry="1"/>
</listOfReactants>
<listOfProducts>
<speciesReference species="M_Pi_c" stoichiometry="1"/>
<speciesReference species="M_DHYDO_ARAB_HEPTP_c" stoichiometry="1"/>
</listOfProducts>
<kineticLaw>
<math xmlns="http://www.w3.org/1998/Math/MathML">
<ci> FLUX_VALUE </ci>
</math>
<listOfParameters>
<parameter id="LOWER_BOUND" value="0" units="mmol_per_gDW_per_hr" constant="true"/>
<parameter id="UPPER_BOUND" value="1000" units="mmol_per_gDW_per_hr" constant="true"/>
<parameter id="FLUX_VALUE" value="0" units="mmol_per_gDW_per_hr" constant="true"/>
<parameter id="OBJECTIVE_COEFFICIENT" value="0" units="mmol_per_gDW_per_hr" constant="true"/>
</listOfParameters>
</kineticLaw>
</reaction>
<reaction id="R_R367" name="cellobiose glucohydrolase" reversible="false">
<notes>
<body xmlns="http://www.w3.org/1999/xhtml">
```

```xml
      <p>GENE_ASSOCIATION: TT_P0042</p>

      <p>SUBSYSTEM: Alternate Carbon Metabolism</p>

      <p>EC Number: 3.2.1.21</p>

      <p>Confidence Level: 1</p>

      <p>AUTHORS: Carbohydrate metabolism</p>

      <p/>

    </body>

  </notes>

  <listOfReactants>

    <speciesReference species="M_H2O_c" stoichiometry="1"/>

    <speciesReference species="M_CELBS_c" stoichiometry="1"/>

  </listOfReactants>

  <listOfProducts>

    <speciesReference species="M_D_GLC_c" stoichiometry="2"/>

  </listOfProducts>

  <kineticLaw>

    <math xmlns="http://www.w3.org/1998/Math/MathML">

      <ci> FLUX_VALUE </ci>

    </math>

    <listOfParameters>

      <parameter id="LOWER_BOUND" value="0" units="mmol_per_gDW_per_hr" constant="true"/>

      <parameter id="UPPER_BOUND" value="1000" units="mmol_per_gDW_per_hr" constant="true"/>

      <parameter id="FLUX_VALUE" value="0" units="mmol_per_gDW_per_hr" constant="true"/>

      <parameter id="OBJECTIVE_COEFFICIENT" value="0" units="mmol_per_gDW_per_hr" constant="true"/>

    </listOfParameters>

  </kineticLaw>

</reaction>

<reaction id="R_R368" name="ATP:alpha-D-glucose-1-phosphate adenylyltransferase" reversible="false">

  <notes>
```

```
  <body xmlns="http://www.w3.org/1999/xhtml">
    <p>GENE_ASSOCIATION: (TTC1976 or TTC1977)</p>
    <p>SUBSYSTEM: Glycolysis/Gluconeogenesis</p>
    <p>EC Number: 2.7.7.27</p>
    <p>Confidence Level: 1</p>
    <p>AUTHORS: Carbohydrate metabolism</p>
    <p/>
  </body>
</notes>
<listOfReactants>
  <speciesReference species="M_ATP_c" stoichiometry="1"/>
  <speciesReference species="M_H_c" stoichiometry="1"/>
  <speciesReference species="M_G1P_c" stoichiometry="1"/>
</listOfReactants>
<listOfProducts>
  <speciesReference species="M_PPi_c" stoichiometry="1"/>
  <speciesReference species="M_ADP_GLU_c" stoichiometry="1"/>
</listOfProducts>
<kineticLaw>
  <math xmlns="http://www.w3.org/1998/Math/MathML">
    <ci> FLUX_VALUE </ci>
  </math>
  <listOfParameters>
    <parameter id="LOWER_BOUND" value="0" units="mmol_per_gDW_per_hr" constant="true"/>
    <parameter id="UPPER_BOUND" value="1000" units="mmol_per_gDW_per_hr" constant="true"/>
    <parameter id="FLUX_VALUE" value="0" units="mmol_per_gDW_per_hr" constant="true"/>
    <parameter id="OBJECTIVE_COEFFICIENT" value="0" units="mmol_per_gDW_per_hr" constant="true"/>
  </listOfParameters>
</kineticLaw>
```

```xml
</reaction>
<reaction id="R_R369" name="ADP-glucose:1,4-alpha-D-glucan 4-alpha-D-glucosyltransferase" reversible="true">
 <notes>
  <body xmlns="http://www.w3.org/1999/xhtml">
   <p>GENE_ASSOCIATION: TTC1980</p>
   <p>SUBSYSTEM: Alternate Carbon Metabolism</p>
   <p>EC Number: 2.4.1.21</p>
   <p>Confidence Level: 1</p>
   <p>AUTHORS: Carbohydrate metabolism</p>
   <p/>
  </body>
 </notes>
 <listOfReactants>
  <speciesReference species="M_ADP_GLU_c" stoichiometry="1"/>
 </listOfReactants>
 <listOfProducts>
  <speciesReference species="M_ADP_c" stoichiometry="1"/>
  <speciesReference species="M_H_c" stoichiometry="1"/>
  <speciesReference species="M_GLY_c" stoichiometry="1"/>
 </listOfProducts>
 <kineticLaw>
  <math xmlns="http://www.w3.org/1998/Math/MathML">
   <ci> FLUX_VALUE </ci>
  </math>
  <listOfParameters>
   <parameter id="LOWER_BOUND" value="-1000" units="mmol_per_gDW_per_hr" constant="true"/>
   <parameter id="UPPER_BOUND" value="1000" units="mmol_per_gDW_per_hr" constant="true"/>
   <parameter id="FLUX_VALUE" value="0" units="mmol_per_gDW_per_hr" constant="true"/>
   <parameter id="OBJECTIVE_COEFFICIENT" value="0" units="mmol_per_gDW_per_hr" constant="true"/>
```

```xml
    </listOfParameters>
  </kineticLaw>
</reaction>
<reaction id="R_R370" name="1,4-alpha-D-Glucan:orthophosphate alpha-D-glucosyltransferase" reversible="false">
  <notes>
    <body xmlns="http://www.w3.org/1999/xhtml">
      <p>GENE_ASSOCIATION: (TTC0808 and TTC1540 and TTC0897)</p>
      <p>SUBSYSTEM: Alternate Carbon Metabolism</p>
      <p>EC Number: 2.4.1.1</p>
      <p>Confidence Level: 1</p>
      <p>AUTHORS: Carbohydrate metabolism</p>
      <p/>
    </body>
  </notes>
  <listOfReactants>
    <speciesReference species="M_Pi_c" stoichiometry="1"/>
    <speciesReference species="M_GLY_c" stoichiometry="1"/>
  </listOfReactants>
  <listOfProducts>
    <speciesReference species="M_G1P_c" stoichiometry="1"/>
  </listOfProducts>
  <kineticLaw>
    <math xmlns="http://www.w3.org/1998/Math/MathML">
      <ci> FLUX_VALUE </ci>
    </math>
    <listOfParameters>
      <parameter id="LOWER_BOUND" value="0" units="mmol_per_gDW_per_hr" constant="true"/>
      <parameter id="UPPER_BOUND" value="1000" units="mmol_per_gDW_per_hr" constant="true"/>
      <parameter id="FLUX_VALUE" value="0" units="mmol_per_gDW_per_hr" constant="true"/>
```

```
      <parameter id="OBJECTIVE_COEFFICIENT" value="0" units="mmol_per_gDW_per_hr" constant="true"/>
    </listOfParameters>
  </kineticLaw>
</reaction>
<reaction id="R_R371" name="maltose glucohydrolase" reversible="false">
  <notes>
    <body xmlns="http://www.w3.org/1999/xhtml">
      <p>GENE_ASSOCIATION: (TTC0107 or TTC1283 or TT_P0221)</p>
      <p>SUBSYSTEM: Alternate Carbon Metabolism</p>
      <p>EC Number: 3.2.1.20</p>
      <p>Confidence Level: 1</p>
      <p>AUTHORS: Carbohydrate metabolism</p>
      <p/>
    </body>
  </notes>
  <listOfReactants>
    <speciesReference species="M_H2O_c" stoichiometry="1"/>
    <speciesReference species="M_MALT_c" stoichiometry="1"/>
  </listOfReactants>
  <listOfProducts>
    <speciesReference species="M_D_GLC_c" stoichiometry="2"/>
  </listOfProducts>
  <kineticLaw>
    <math xmlns="http://www.w3.org/1998/Math/MathML">
      <ci> FLUX_VALUE </ci>
    </math>
    <listOfParameters>
      <parameter id="LOWER_BOUND" value="0" units="mmol_per_gDW_per_hr" constant="true"/>
      <parameter id="UPPER_BOUND" value="1000" units="mmol_per_gDW_per_hr" constant="true"/>
```

```
      <parameter id="FLUX_VALUE" value="0" units="mmol_per_gDW_per_hr" constant="true"/>
      <parameter id="OBJECTIVE_COEFFICIENT" value="0" units="mmol_per_gDW_per_hr" constant="true"/>
    </listOfParameters>
  </kineticLaw>
</reaction>
<reaction id="R_R372" name="maltose glucohydrolase" reversible="false">
  <notes>
    <body xmlns="http://www.w3.org/1999/xhtml">
      <p>GENE_ASSOCIATION: (TTC0107 or TTC1283 or TT_P0221)</p>
      <p>SUBSYSTEM: Alternate Carbon Metabolism</p>
      <p>EC Number: 3.2.1.20</p>
      <p>Confidence Level: 1</p>
      <p>AUTHORS: Carbohydrate metabolism</p>
      <p/>
    </body>
  </notes>
  <listOfReactants>
    <speciesReference species="M_H2O_c" stoichiometry="1"/>
    <speciesReference species="M_IMALT_c" stoichiometry="1"/>
  </listOfReactants>
  <listOfProducts>
    <speciesReference species="M_D_GLC_c" stoichiometry="2"/>
  </listOfProducts>
  <kineticLaw>
    <math xmlns="http://www.w3.org/1998/Math/MathML">
      <ci> FLUX_VALUE </ci>
    </math>
    <listOfParameters>
      <parameter id="LOWER_BOUND" value="0" units="mmol_per_gDW_per_hr" constant="true"/>
```

```
<parameter id="UPPER_BOUND" value="1000" units="mmol_per_gDW_per_hr" constant="true"/>
<parameter id="FLUX_VALUE" value="0" units="mmol_per_gDW_per_hr" constant="true"/>
<parameter id="OBJECTIVE_COEFFICIENT" value="0" units="mmol_per_gDW_per_hr" constant="true"/>
</listOfParameters>
</kineticLaw>
</reaction>
<reaction id="R_R373" name="N-Acetyl-D-glucosamine-6-phosphate amidohydrolase" reversible="false">
<notes>
<body xmlns="http://www.w3.org/1999/xhtml">
<p>GENE_ASSOCIATION: TT_P0025</p>
<p>SUBSYSTEM: Alternate Carbon Metabolism</p>
<p>EC Number: 3.5.1.25</p>
<p>Confidence Level: 1</p>
<p>AUTHORS: Carbohydrate metabolism</p>
<p/>
</body>
</notes>
<listOfReactants>
<speciesReference species="M_H2O_c" stoichiometry="1"/>
<speciesReference species="M_AC_GLUSAP_c" stoichiometry="1"/>
</listOfReactants>
<listOfProducts>
<speciesReference species="M_AC_c" stoichiometry="1"/>
<speciesReference species="M_GLUSAP_c" stoichiometry="1"/>
</listOfProducts>
<kineticLaw>
<math xmlns="http://www.w3.org/1998/Math/MathML">
<ci> FLUX_VALUE </ci>
</math>
```

```
    <listOfParameters>
      <parameter id="LOWER_BOUND" value="0" units="mmol_per_gDW_per_hr" constant="true"/>
      <parameter id="UPPER_BOUND" value="1000" units="mmol_per_gDW_per_hr" constant="true"/>
      <parameter id="FLUX_VALUE" value="0" units="mmol_per_gDW_per_hr" constant="true"/>
      <parameter id="OBJECTIVE_COEFFICIENT" value="0" units="mmol_per_gDW_per_hr" constant="true"/>
    </listOfParameters>
  </kineticLaw>
</reaction>
<reaction id="R_R374" name="UTP:N-acetyl-alpha-D-glucosamine-1-phosphate uridylyltransferase" reversible="false">
  <notes>
    <body xmlns="http://www.w3.org/1999/xhtml">
      <p>GENE_ASSOCIATION: TTC0017</p>
      <p>SUBSYSTEM: Cell Envelope Biosynthesis</p>
      <p>EC Number: 2.7.7.23</p>
      <p>Confidence Level: 1</p>
      <p>AUTHORS: Lipid metabolism</p>
      <p/>
    </body>
  </notes>
  <listOfReactants>
    <speciesReference species="M_UTP_c" stoichiometry="1"/>
    <speciesReference species="M_H_c" stoichiometry="1"/>
    <speciesReference species="M_AC_AGLUCSAPi_c" stoichiometry="1"/>
  </listOfReactants>
  <listOfProducts>
    <speciesReference species="M_UDP_AGLUAM_c" stoichiometry="1"/>
    <speciesReference species="M_PPi_c" stoichiometry="1"/>
  </listOfProducts>
  <kineticLaw>
```

```
    <math xmlns="http://www.w3.org/1998/Math/MathML">
      <ci> FLUX_VALUE </ci>
    </math>
    <listOfParameters>
      <parameter id="LOWER_BOUND" value="0" units="mmol_per_gDW_per_hr" constant="true"/>
      <parameter id="UPPER_BOUND" value="1000" units="mmol_per_gDW_per_hr" constant="true"/>
      <parameter id="FLUX_VALUE" value="0" units="mmol_per_gDW_per_hr" constant="true"/>
      <parameter id="OBJECTIVE_COEFFICIENT" value="0" units="mmol_per_gDW_per_hr" constant="true"/>
    </listOfParameters>
  </kineticLaw>
</reaction>
<reaction id="R_R375" name="Acetyl-CoA:D-glucosamine-1-phosphate N-acetyltransferase" reversible="false">
  <notes>
    <body xmlns="http://www.w3.org/1999/xhtml">
      <p>GENE_ASSOCIATION: TTC0017</p>
      <p>SUBSYSTEM: Cell Envelope Biosynthesis</p>
      <p>EC Number: 2.3.1.157</p>
      <p>Confidence Level: 1</p>
      <p>AUTHORS: Lipid metabolism</p>
      <p/>
    </body>
  </notes>
  <listOfReactants>
    <speciesReference species="M_ACoA_c" stoichiometry="1"/>
    <speciesReference species="M_GLUCSA1P_c" stoichiometry="1"/>
  </listOfReactants>
  <listOfProducts>
    <speciesReference species="M_H_c" stoichiometry="1"/>
    <speciesReference species="M_CoA_c" stoichiometry="1"/>
```

```xml
<speciesReference species="M_AC_AGLUCSAPi_c" stoichiometry="1"/>
</listOfProducts>
<kineticLaw>
<math xmlns="http://www.w3.org/1998/Math/MathML">
<ci> FLUX_VALUE </ci>
</math>
<listOfParameters>
<parameter id="LOWER_BOUND" value="0" units="mmol_per_gDW_per_hr" constant="true"/>
<parameter id="UPPER_BOUND" value="1000" units="mmol_per_gDW_per_hr" constant="true"/>
<parameter id="FLUX_VALUE" value="0" units="mmol_per_gDW_per_hr" constant="true"/>
<parameter id="OBJECTIVE_COEFFICIENT" value="0" units="mmol_per_gDW_per_hr" constant="true"/>
</listOfParameters>
</kineticLaw>
</reaction>
<reaction id="R_R376" name="UDP-N-acetyl-D-glucosamine 2-epimerase" reversible="false">
<notes>
<body xmlns="http://www.w3.org/1999/xhtml">
<p>GENE_ASSOCIATION: TTC0948</p>
<p>SUBSYSTEM: Cell Envelope Biosynthesis</p>
<p>EC Number: 5.1.3.14</p>
<p>Confidence Level: 1</p>
<p>AUTHORS: Lipid metabolism</p>
<p/>
</body>
</notes>
<listOfReactants>
<speciesReference species="M_UDP_AGLUAM_c" stoichiometry="1"/>
</listOfReactants>
<listOfProducts>
```

```
  <speciesReference species="M_UDP_AC_MANNA_c" stoichiometry="1"/>
 </listOfProducts>
 <kineticLaw>
  <math xmlns="http://www.w3.org/1998/Math/MathML">
   <ci> FLUX_VALUE </ci>
  </math>
  <listOfParameters>
   <parameter id="LOWER_BOUND" value="0" units="mmol_per_gDW_per_hr" constant="true"/>
   <parameter id="UPPER_BOUND" value="1000" units="mmol_per_gDW_per_hr" constant="true"/>
   <parameter id="FLUX_VALUE" value="0" units="mmol_per_gDW_per_hr" constant="true"/>
   <parameter id="OBJECTIVE_COEFFICIENT" value="0" units="mmol_per_gDW_per_hr" constant="true"/>
  </listOfParameters>
 </kineticLaw>
</reaction>
<reaction id="R_R377" name="D-Glucosamine 1-phosphate 1,6-phosphomutase" reversible="true">
 <notes>
  <body xmlns="http://www.w3.org/1999/xhtml">
   <p>GENE_ASSOCIATION: TTC0691</p>
   <p>SUBSYSTEM: Cell Envelope Biosynthesis</p>
   <p>EC Number: 5.4.2.10</p>
   <p>Confidence Level: 1</p>
   <p>AUTHORS: Lipid metabolism</p>
   <p/>
  </body>
 </notes>
 <listOfReactants>
  <speciesReference species="M_GLUSAP_c" stoichiometry="1"/>
 </listOfReactants>
 <listOfProducts>
```

```
    <speciesReference species="M_GLUCSA1P_c" stoichiometry="1"/>
  </listOfProducts>
  <kineticLaw>
    <math xmlns="http://www.w3.org/1998/Math/MathML">
      <ci> FLUX_VALUE </ci>
    </math>
    <listOfParameters>
      <parameter id="LOWER_BOUND" value="-1000" units="mmol_per_gDW_per_hr" constant="true"/>
      <parameter id="UPPER_BOUND" value="1000" units="mmol_per_gDW_per_hr" constant="true"/>
      <parameter id="FLUX_VALUE" value="0" units="mmol_per_gDW_per_hr" constant="true"/>
      <parameter id="OBJECTIVE_COEFFICIENT" value="0" units="mmol_per_gDW_per_hr" constant="true"/>
    </listOfParameters>
  </kineticLaw>
</reaction>
<reaction id="R_R378" name="UDP-N-acetylmuramate:NADP+ oxidoreductase" reversible="false">
  <notes>
    <body xmlns="http://www.w3.org/1999/xhtml">
      <p>GENE_ASSOCIATION: TTC0721</p>
      <p>SUBSYSTEM: Cell Envelope Biosynthesis</p>
      <p>EC Number: 1.1.1.158</p>
      <p>Confidence Level: 1</p>
      <p>AUTHORS: Lipid metabolism</p>
      <p/>
    </body>
  </notes>
  <listOfReactants>
    <speciesReference species="M_H_c" stoichiometry="1"/>
    <speciesReference species="M_NADPH_c" stoichiometry="1"/>
    <speciesReference species="M_UDP_AC_CARBVIN_GLUCSA_c" stoichiometry="1"/>
```

```xml
</listOfReactants>

<listOfProducts>

<speciesReference species="M_UDP_ACMURM_c" stoichiometry="1"/>

<speciesReference species="M_NADP_c" stoichiometry="1"/>

</listOfProducts>

<kineticLaw>

<math xmlns="http://www.w3.org/1998/Math/MathML">

<ci> FLUX_VALUE </ci>

</math>

<listOfParameters>

<parameter id="LOWER_BOUND" value="0" units="mmol_per_gDW_per_hr" constant="true"/>

<parameter id="UPPER_BOUND" value="1000" units="mmol_per_gDW_per_hr" constant="true"/>

<parameter id="FLUX_VALUE" value="0" units="mmol_per_gDW_per_hr" constant="true"/>

<parameter id="OBJECTIVE_COEFFICIENT" value="0" units="mmol_per_gDW_per_hr" constant="true"/>

</listOfParameters>

</kineticLaw>

</reaction>

<reaction id="R_R379" name="Phosphoenolpyruvate:UDP-N-acetyl-D-glucosamine 1-carboxyvinyl-transferase" reversible="false">

<notes>

<body xmlns="http://www.w3.org/1999/xhtml">

<p>GENE_ASSOCIATION: TTC1854</p>

<p>SUBSYSTEM: Cell Envelope Biosynthesis</p>

<p>EC Number: 2.5.1.7</p>

<p>Confidence Level: 1</p>

<p>AUTHORS: Lipid metabolism</p>

<p/>

</body>

</notes>
```

```
  <listOfReactants>
    <speciesReference species="M_UDP_AGLUAM_c" stoichiometry="1"/>
    <speciesReference species="M_PEP_c" stoichiometry="1"/>
  </listOfReactants>
  <listOfProducts>
    <speciesReference species="M_Pi_c" stoichiometry="1"/>
    <speciesReference species="M_UDP_AC_CARBVIN_GLUCSA_c" stoichiometry="1"/>
  </listOfProducts>
  <kineticLaw>
    <math xmlns="http://www.w3.org/1998/Math/MathML">
      <ci> FLUX_VALUE </ci>
    </math>
    <listOfParameters>
      <parameter id="LOWER_BOUND" value="0" units="mmol_per_gDW_per_hr" constant="true"/>
      <parameter id="UPPER_BOUND" value="1000" units="mmol_per_gDW_per_hr" constant="true"/>
      <parameter id="FLUX_VALUE" value="0" units="mmol_per_gDW_per_hr" constant="true"/>
      <parameter id="OBJECTIVE_COEFFICIENT" value="0" units="mmol_per_gDW_per_hr" constant="true"/>
    </listOfParameters>
  </kineticLaw>
</reaction>
<reaction id="R_R380" name="1D-myo-Inositol 4-phosphate phosphohydrolase" reversible="false">
  <notes>
    <body xmlns="http://www.w3.org/1999/xhtml">
      <p>GENE_ASSOCIATION: </p>
      <p>SUBSYSTEM: Inositol phosphate metabolism</p>
      <p>EC Number: 5.5.1.4</p>
      <p>Confidence Level: 1</p>
      <p>AUTHORS: Lipid metabolism</p>
      <p/>
```

```xml
</body>
</notes>
<listOfReactants>
<speciesReference species="M_G6P_c" stoichiometry="1"/>
</listOfReactants>
<listOfProducts>
<speciesReference species="M_mINS3P_c" stoichiometry="1"/>
</listOfProducts>
<kineticLaw>
<math xmlns="http://www.w3.org/1998/Math/MathML">
<ci> FLUX_VALUE </ci>
</math>
<listOfParameters>
<parameter id="LOWER_BOUND" value="0" units="mmol_per_gDW_per_hr" constant="true"/>
<parameter id="UPPER_BOUND" value="1000" units="mmol_per_gDW_per_hr" constant="true"/>
<parameter id="FLUX_VALUE" value="0" units="mmol_per_gDW_per_hr" constant="true"/>
<parameter id="OBJECTIVE_COEFFICIENT" value="0" units="mmol_per_gDW_per_hr" constant="true"/>
</listOfParameters>
</kineticLaw>
</reaction>
<reaction id="R_R381" name="1D-myo-Inositol 3-phosphate phosphohydrolase" reversible="false">
<notes>
<body xmlns="http://www.w3.org/1999/xhtml">
<p>GENE_ASSOCIATION: TTC1928</p>
<p>SUBSYSTEM: Inositol phosphate metabolism</p>
<p>EC Number: 3.1.3.25</p>
<p>Confidence Level: 1</p>
<p>AUTHORS: Lipid metabolism</p>
<p/>
```

```
    </body>
   </notes>
   <listOfReactants>
    <speciesReference species="M_H2O_c" stoichiometry="1"/>
    <speciesReference species="M_mINS3P_c" stoichiometry="1"/>
   </listOfReactants>
   <listOfProducts>
    <speciesReference species="M_Pi_c" stoichiometry="1"/>
    <speciesReference species="M_M_INS_c" stoichiometry="1"/>
   </listOfProducts>
   <kineticLaw>
    <math xmlns="http://www.w3.org/1998/Math/MathML">
     <ci> FLUX_VALUE </ci>
    </math>
    <listOfParameters>
     <parameter id="LOWER_BOUND" value="0" units="mmol_per_gDW_per_hr" constant="true"/>
     <parameter id="UPPER_BOUND" value="1000" units="mmol_per_gDW_per_hr" constant="true"/>
     <parameter id="FLUX_VALUE" value="0" units="mmol_per_gDW_per_hr" constant="true"/>
     <parameter id="OBJECTIVE_COEFFICIENT" value="0" units="mmol_per_gDW_per_hr" constant="true"/>
    </listOfParameters>
   </kineticLaw>
  </reaction>
  <reaction id="R_R382" name="sn-Glycerol-3-phosphate:NAD+ 2-oxidoreductase" reversible="false">
   <notes>
    <body xmlns="http://www.w3.org/1999/xhtml">
     <p>GENE_ASSOCIATION: TTC1378</p>
     <p>SUBSYSTEM: Alternate Carbon Metabolism</p>
     <p>EC Number: 1.1.1.94</p>
     <p>Confidence Level: 1</p>
```

```
      <p>AUTHORS: Carbohydrate metabolism</p>
      <p/>
    </body>
  </notes>
  <listOfReactants>
    <speciesReference species="M_H_c" stoichiometry="1"/>
    <speciesReference species="M_DHAP_c" stoichiometry="1"/>
    <speciesReference species="M_NADH_c" stoichiometry="1"/>
  </listOfReactants>
  <listOfProducts>
    <speciesReference species="M_NAD_c" stoichiometry="1"/>
    <speciesReference species="M_GLCP_c" stoichiometry="1"/>
  </listOfProducts>
  <kineticLaw>
    <math xmlns="http://www.w3.org/1998/Math/MathML">
      <ci> FLUX_VALUE </ci>
    </math>
    <listOfParameters>
      <parameter id="LOWER_BOUND" value="0" units="mmol_per_gDW_per_hr" constant="true"/>
      <parameter id="UPPER_BOUND" value="1000" units="mmol_per_gDW_per_hr" constant="true"/>
      <parameter id="FLUX_VALUE" value="0" units="mmol_per_gDW_per_hr" constant="true"/>
      <parameter id="OBJECTIVE_COEFFICIENT" value="0" units="mmol_per_gDW_per_hr" constant="true"/>
    </listOfParameters>
  </kineticLaw>
</reaction>
<reaction id="R_R383" name="sn-Glycerol-3-phosphate:NADP+ 2-oxidoreductase" reversible="false">
  <notes>
    <body xmlns="http://www.w3.org/1999/xhtml">
      <p>GENE_ASSOCIATION: TTC1378</p>
```

```xml
<p>SUBSYSTEM: Alternate Carbon Metabolism</p>

<p>EC Number: 1.1.1.94</p>

<p>Confidence Level: 1</p>

<p>AUTHORS: Carbohydrate metabolism</p>

<p/>

</body>

</notes>

<listOfReactants>

<speciesReference species="M_H_c" stoichiometry="1"/>

<speciesReference species="M_DHAP_c" stoichiometry="1"/>

<speciesReference species="M_NADPH_c" stoichiometry="1"/>

</listOfReactants>

<listOfProducts>

<speciesReference species="M_NADP_c" stoichiometry="1"/>

<speciesReference species="M_GLCP_c" stoichiometry="1"/>

</listOfProducts>

<kineticLaw>

<math xmlns="http://www.w3.org/1998/Math/MathML">

<ci> FLUX_VALUE </ci>

</math>

<listOfParameters>

<parameter id="LOWER_BOUND" value="0" units="mmol_per_gDW_per_hr" constant="true"/>

<parameter id="UPPER_BOUND" value="1000" units="mmol_per_gDW_per_hr" constant="true"/>

<parameter id="FLUX_VALUE" value="0" units="mmol_per_gDW_per_hr" constant="true"/>

<parameter id="OBJECTIVE_COEFFICIENT" value="0" units="mmol_per_gDW_per_hr" constant="true"/>

</listOfParameters>

</kineticLaw>

</reaction>

<reaction id="R_R384" name="acyl-CoA:sn-glycerol-3-phosphate 1-O-acyltransferase" reversible="false">
```

```xml
<notes>
  <body xmlns="http://www.w3.org/1999/xhtml">
    <p>GENE_ASSOCIATION: (TTC0841 or TTC1384)</p>
    <p>SUBSYSTEM: Glycerolipid metabolism</p>
    <p>EC Number: 2.3.1.15</p>
    <p>Confidence Level: 1</p>
    <p>AUTHORS: Lipid metabolism</p>
    <p/>
  </body>
</notes>
<listOfReactants>
  <speciesReference species="M_GLCP_c" stoichiometry="1"/>
  <speciesReference species="M_I150CoA_c" stoichiometry="0.334"/>
  <speciesReference species="M_AI150CoA_c" stoichiometry="0.069"/>
  <speciesReference species="M_I160CoA_c" stoichiometry="0.047"/>
  <speciesReference species="M_C160CoA_c" stoichiometry="0.014"/>
  <speciesReference species="M_C161CoA_c" stoichiometry="0.0015"/>
  <speciesReference species="M_I170CoA_c" stoichiometry="0.45"/>
  <speciesReference species="M_AI171CoA_c" stoichiometry="0.075"/>
</listOfReactants>
<listOfProducts>
  <speciesReference species="M_CoA_c" stoichiometry="1"/>
  <speciesReference species="M_GLY3P_c" stoichiometry="1"/>
</listOfProducts>
<kineticLaw>
  <math xmlns="http://www.w3.org/1998/Math/MathML">
    <ci> FLUX_VALUE </ci>
  </math>
  <listOfParameters>
```

```xml
<parameter id="LOWER_BOUND" value="0" units="mmol_per_gDW_per_hr" constant="true"/>
<parameter id="UPPER_BOUND" value="1000" units="mmol_per_gDW_per_hr" constant="true"/>
<parameter id="FLUX_VALUE" value="0" units="mmol_per_gDW_per_hr" constant="true"/>
<parameter id="OBJECTIVE_COEFFICIENT" value="0" units="mmol_per_gDW_per_hr" constant="true"/>
</listOfParameters>
</kineticLaw>
</reaction>
<reaction id="R_R385" name="acyl-CoA:1-acyl-sn-glycerol-3-phosphate 2-O-acyltransferase" reversible="false">
<notes>
<body xmlns="http://www.w3.org/1999/xhtml">
<p>GENE_ASSOCIATION: TTC0983</p>
<p>SUBSYSTEM: Glycerolipid metabolism</p>
<p>EC Number: 2.3.1.51</p>
<p>Confidence Level: 1</p>
<p>AUTHORS: Lipid metabolism</p>
<p/>
</body>
</notes>
<listOfReactants>
<speciesReference species="M_I150CoA_c" stoichiometry="0.334"/>
<speciesReference species="M_AI150CoA_c" stoichiometry="0.069"/>
<speciesReference species="M_I160CoA_c" stoichiometry="0.047"/>
<speciesReference species="M_C160CoA_c" stoichiometry="0.014"/>
<speciesReference species="M_C161CoA_c" stoichiometry="0.0015"/>
<speciesReference species="M_I170CoA_c" stoichiometry="0.45"/>
<speciesReference species="M_AI171CoA_c" stoichiometry="0.075"/>
<speciesReference species="M_GLY3P_c" stoichiometry="1"/>
</listOfReactants>
<listOfProducts>
```

```
<speciesReference species="M_CoA_c" stoichiometry="1"/>
<speciesReference species="M_PPTD_c" stoichiometry="1"/>
</listOfProducts>
<kineticLaw>
<math xmlns="http://www.w3.org/1998/Math/MathML">
<ci> FLUX_VALUE </ci>
</math>
<listOfParameters>
<parameter id="LOWER_BOUND" value="0" units="mmol_per_gDW_per_hr" constant="true"/>
<parameter id="UPPER_BOUND" value="1000" units="mmol_per_gDW_per_hr" constant="true"/>
<parameter id="FLUX_VALUE" value="0" units="mmol_per_gDW_per_hr" constant="true"/>
<parameter id="OBJECTIVE_COEFFICIENT" value="0" units="mmol_per_gDW_per_hr" constant="true"/>
</listOfParameters>
</kineticLaw>
</reaction>
<reaction id="R_R386" name="S-adenosyl-L-methionine:phosphatidylethanolamine N-methyltransferase"
reversible="false">
<notes>
<body xmlns="http://www.w3.org/1999/xhtml">
<p>GENE_ASSOCIATION: TTC1366</p>
<p>SUBSYSTEM: Glycerophospholipid metabolism</p>
<p>EC Number: 2.1.1.17</p>
<p>Confidence Level: 1</p>
<p>AUTHORS: Lipid metabolism</p>
<p/>
</body>
</notes>
<listOfReactants>
<speciesReference species="M_PETHA_c" stoichiometry="1"/>
```

```
    <speciesReference species="M_SAM_c" stoichiometry="1"/>
  </listOfReactants>
  <listOfProducts>
    <speciesReference species="M_SAHC_c" stoichiometry="1"/>
    <speciesReference species="M_PMETHA_c" stoichiometry="1"/>
  </listOfProducts>
  <kineticLaw>
    <math xmlns="http://www.w3.org/1998/Math/MathML">
      <ci> FLUX_VALUE </ci>
    </math>
    <listOfParameters>
      <parameter id="LOWER_BOUND" value="0" units="mmol_per_gDW_per_hr" constant="true"/>
      <parameter id="UPPER_BOUND" value="1000" units="mmol_per_gDW_per_hr" constant="true"/>
      <parameter id="FLUX_VALUE" value="0" units="mmol_per_gDW_per_hr" constant="true"/>
      <parameter id="OBJECTIVE_COEFFICIENT" value="0" units="mmol_per_gDW_per_hr" constant="true"/>
    </listOfParameters>
  </kineticLaw>
</reaction>
<reaction id="R_R387" name="phosphatidyl-N-methylethanolamine N-methyltransferase" reversible="false">
  <notes>
    <body xmlns="http://www.w3.org/1999/xhtml">
      <p>GENE_ASSOCIATION: </p>
      <p>SUBSYSTEM: Glycerophospholipid metabolism</p>
      <p>EC Number: 2.1.1.71</p>
      <p>Confidence Level: 1</p>
      <p>AUTHORS: Lipid metabolism</p>
      <p/>
    </body>
  </notes>
```

```
  <listOfReactants>
    <speciesReference species="M_SAM_c" stoichiometry="1"/>
    <speciesReference species="M_PMETHA_c" stoichiometry="1"/>
  </listOfReactants>
  <listOfProducts>
    <speciesReference species="M_SAHC_c" stoichiometry="1"/>
    <speciesReference species="M_PDMETHA_c" stoichiometry="1"/>
  </listOfProducts>
  <kineticLaw>
    <math xmlns="http://www.w3.org/1998/Math/MathML">
      <ci> FLUX_VALUE </ci>
    </math>
    <listOfParameters>
      <parameter id="LOWER_BOUND" value="0" units="mmol_per_gDW_per_hr" constant="true"/>
      <parameter id="UPPER_BOUND" value="1000" units="mmol_per_gDW_per_hr" constant="true"/>
      <parameter id="FLUX_VALUE" value="0" units="mmol_per_gDW_per_hr" constant="true"/>
      <parameter id="OBJECTIVE_COEFFICIENT" value="0" units="mmol_per_gDW_per_hr" constant="true"/>
    </listOfParameters>
  </kineticLaw>
</reaction>
<reaction id="R_R388" name="phosphatidyl-N-dimethylethanolamine N-methyltransferase" reversible="false">
  <notes>
    <body xmlns="http://www.w3.org/1999/xhtml">
      <p>GENE_ASSOCIATION: </p>
      <p>SUBSYSTEM: Glycerophospholipid metabolism</p>
      <p>EC Number: 2.1.1.71</p>
      <p>Confidence Level: 1</p>
      <p>AUTHORS: Lipid metabolism</p>
      <p/>
```

```
    </body>
  </notes>
  <listOfReactants>
    <speciesReference species="M_SAM_c" stoichiometry="1"/>
    <speciesReference species="M_PDMETHA_c" stoichiometry="1"/>
  </listOfReactants>
  <listOfProducts>
    <speciesReference species="M_SAHC_c" stoichiometry="1"/>
    <speciesReference species="M_PCHOL_c" stoichiometry="1"/>
  </listOfProducts>
  <kineticLaw>
    <math xmlns="http://www.w3.org/1998/Math/MathML">
      <ci> FLUX_VALUE </ci>
    </math>
    <listOfParameters>
      <parameter id="LOWER_BOUND" value="0" units="mmol_per_gDW_per_hr" constant="true"/>
      <parameter id="UPPER_BOUND" value="1000" units="mmol_per_gDW_per_hr" constant="true"/>
      <parameter id="FLUX_VALUE" value="0" units="mmol_per_gDW_per_hr" constant="true"/>
      <parameter id="OBJECTIVE_COEFFICIENT" value="0" units="mmol_per_gDW_per_hr" constant="true"/>
    </listOfParameters>
  </kineticLaw>
</reaction>
<reaction id="R_R389" name="Transacylase" reversible="false">
  <notes>
    <body xmlns="http://www.w3.org/1999/xhtml">
      <p>GENE_ASSOCIATION: </p>
      <p>SUBSYSTEM: Glycerophospholipid metabolism</p>
      <p>EC Number: </p>
      <p>Confidence Level: 1</p>
```

```
      <p>AUTHORS: Lipid metabolism</p>
      <p/>
    </body>
  </notes>
  <listOfReactants>
    <speciesReference species="M_PETHA_c" stoichiometry="1"/>
    <speciesReference species="M_PCHOL_c" stoichiometry="1"/>
  </listOfReactants>
  <listOfProducts>
    <speciesReference species="M_PX_c" stoichiometry="1"/>
    <speciesReference species="M_LYPCHOL_c" stoichiometry="1"/>
  </listOfProducts>
  <kineticLaw>
    <math xmlns="http://www.w3.org/1998/Math/MathML">
      <ci> FLUX_VALUE </ci>
    </math>
    <listOfParameters>
      <parameter id="LOWER_BOUND" value="0" units="mmol_per_gDW_per_hr" constant="true"/>
      <parameter id="UPPER_BOUND" value="1000" units="mmol_per_gDW_per_hr" constant="true"/>
      <parameter id="FLUX_VALUE" value="0" units="mmol_per_gDW_per_hr" constant="true"/>
      <parameter id="OBJECTIVE_COEFFICIENT" value="0" units="mmol_per_gDW_per_hr" constant="true"/>
    </listOfParameters>
  </kineticLaw>
</reaction>
<reaction id="R_R390" name="acyltransferase" reversible="false">
  <notes>
    <body xmlns="http://www.w3.org/1999/xhtml">
      <p>GENE_ASSOCIATION: </p>
      <p>SUBSYSTEM: Glycerophospholipid metabolism</p>
```

```xml
    <p>EC Number: </p>
    <p>Confidence Level: 1</p>
    <p>AUTHORS: Lipid metabolism</p>
    <p/>
  </body>
</notes>
<listOfReactants>
  <speciesReference species="M_I150CoA_c" stoichiometry="0.334"/>
  <speciesReference species="M_AI150CoA_c" stoichiometry="0.069"/>
  <speciesReference species="M_I160CoA_c" stoichiometry="0.047"/>
  <speciesReference species="M_C160CoA_c" stoichiometry="0.014"/>
  <speciesReference species="M_C161CoA_c" stoichiometry="0.0015"/>
  <speciesReference species="M_I170CoA_c" stoichiometry="0.45"/>
  <speciesReference species="M_AI171CoA_c" stoichiometry="0.075"/>
  <speciesReference species="M_LYPCHOL_c" stoichiometry="1"/>
</listOfReactants>
<listOfProducts>
  <speciesReference species="M_CoA_c" stoichiometry="1"/>
  <speciesReference species="M_PCHOL_c" stoichiometry="1"/>
</listOfProducts>
<kineticLaw>
  <math xmlns="http://www.w3.org/1998/Math/MathML">
    <ci> FLUX_VALUE </ci>
  </math>
  <listOfParameters>
    <parameter id="LOWER_BOUND" value="0" units="mmol_per_gDW_per_hr" constant="true"/>
    <parameter id="UPPER_BOUND" value="1000" units="mmol_per_gDW_per_hr" constant="true"/>
    <parameter id="FLUX_VALUE" value="0" units="mmol_per_gDW_per_hr" constant="true"/>
    <parameter id="OBJECTIVE_COEFFICIENT" value="0" units="mmol_per_gDW_per_hr" constant="true"/>
```

```
    </listOfParameters>
  </kineticLaw>
</reaction>
<reaction id="R_R391" name="phosphatidic acid phosphatase" reversible="false">
  <notes>
    <body xmlns="http://www.w3.org/1999/xhtml">
      <p>GENE_ASSOCIATION: </p>
      <p>SUBSYSTEM: Glycerophospholipid metabolism</p>
      <p>EC Number: </p>
      <p>Confidence Level: 1</p>
      <p>AUTHORS: Lipid metabolism</p>
      <p/>
    </body>
  </notes>
  <listOfReactants>
    <speciesReference species="M_H2O_c" stoichiometry="1"/>
    <speciesReference species="M_PPTD_c" stoichiometry="1"/>
  </listOfReactants>
  <listOfProducts>
    <speciesReference species="M_Pi_c" stoichiometry="1"/>
    <speciesReference species="M_DAG_c" stoichiometry="1"/>
  </listOfProducts>
  <kineticLaw>
    <math xmlns="http://www.w3.org/1998/Math/MathML">
      <ci> FLUX_VALUE </ci>
    </math>
    <listOfParameters>
      <parameter id="LOWER_BOUND" value="0" units="mmol_per_gDW_per_hr" constant="true"/>
      <parameter id="UPPER_BOUND" value="1000" units="mmol_per_gDW_per_hr" constant="true"/>
```

```
      <parameter id="FLUX_VALUE" value="0" units="mmol_per_gDW_per_hr" constant="true"/>
      <parameter id="OBJECTIVE_COEFFICIENT" value="0" units="mmol_per_gDW_per_hr" constant="true"/>
    </listOfParameters>
  </kineticLaw>
</reaction>
<reaction id="R_R392" name="ATP:1,2-diacylglycerol 3-phosphotransferase" reversible="false">
  <notes>
    <body xmlns="http://www.w3.org/1999/xhtml">
      <p>GENE_ASSOCIATION: TTC0682</p>
      <p>SUBSYSTEM: Glycerolipid metabolism</p>
      <p>EC Number: 2.7.1.107</p>
      <p>Confidence Level: 1</p>
      <p>AUTHORS: Lipid metabolism</p>
      <p/>
    </body>
  </notes>
  <listOfReactants>
    <speciesReference species="M_ATP_c" stoichiometry="1"/>
    <speciesReference species="M_DAG_c" stoichiometry="1"/>
  </listOfReactants>
  <listOfProducts>
    <speciesReference species="M_ADP_c" stoichiometry="1"/>
    <speciesReference species="M_PPTD_c" stoichiometry="1"/>
  </listOfProducts>
  <kineticLaw>
    <math xmlns="http://www.w3.org/1998/Math/MathML">
      <ci> FLUX_VALUE </ci>
    </math>
    <listOfParameters>
```

```
<parameter id="LOWER_BOUND" value="0" units="mmol_per_gDW_per_hr" constant="true"/>
<parameter id="UPPER_BOUND" value="1000" units="mmol_per_gDW_per_hr" constant="true"/>
<parameter id="FLUX_VALUE" value="0" units="mmol_per_gDW_per_hr" constant="true"/>
<parameter id="OBJECTIVE_COEFFICIENT" value="0" units="mmol_per_gDW_per_hr" constant="true"/>
</listOfParameters>
</kineticLaw>
</reaction>
<reaction id="R_R393" name="UDP-glucosyltransferase (diglucosyl)" reversible="false">
<notes>
<body xmlns="http://www.w3.org/1999/xhtml">
<p>GENE_ASSOCIATION: </p>
<p>SUBSYSTEM: Glycerolipid metabolism</p>
<p>EC Number: </p>
<p>Confidence Level: 1</p>
<p>AUTHORS: Lipid metabolism</p>
<p/>
</body>
</notes>
<listOfReactants>
<speciesReference species="M_UDP_GLU_c" stoichiometry="1"/>
<speciesReference species="M_DAG_c" stoichiometry="1"/>
</listOfReactants>
<listOfProducts>
<speciesReference species="M_MGDG_c" stoichiometry="1"/>
<speciesReference species="M_H_c" stoichiometry="1"/>
<speciesReference species="M_UDP_c" stoichiometry="1"/>
</listOfProducts>
<kineticLaw>
<math xmlns="http://www.w3.org/1998/Math/MathML">
```

```
      <ci> FLUX_VALUE </ci>
    </math>
    <listOfParameters>
      <parameter id="LOWER_BOUND" value="0" units="mmol_per_gDW_per_hr" constant="true"/>
      <parameter id="UPPER_BOUND" value="1000" units="mmol_per_gDW_per_hr" constant="true"/>
      <parameter id="FLUX_VALUE" value="0" units="mmol_per_gDW_per_hr" constant="true"/>
      <parameter id="OBJECTIVE_COEFFICIENT" value="0" units="mmol_per_gDW_per_hr" constant="true"/>
    </listOfParameters>
  </kineticLaw>
</reaction>
<reaction id="R_R394" name="UDP-glucosyltransferase (monoglucosyl)" reversible="false">
  <notes>
    <body xmlns="http://www.w3.org/1999/xhtml">
      <p>GENE_ASSOCIATION: </p>
      <p>SUBSYSTEM: Glycerolipid metabolism</p>
      <p>EC Number: </p>
      <p>Confidence Level: 1</p>
      <p>AUTHORS: Lipid metabolism</p>
      <p/>
    </body>
  </notes>
  <listOfReactants>
    <speciesReference species="M_MGDG_c" stoichiometry="1"/>
    <speciesReference species="M_UDP_GLU_c" stoichiometry="1"/>
  </listOfReactants>
  <listOfProducts>
    <speciesReference species="M_DGDG_c" stoichiometry="1"/>
    <speciesReference species="M_H_c" stoichiometry="1"/>
    <speciesReference species="M_UDP_c" stoichiometry="1"/>
```

```
  </listOfProducts>
  <kineticLaw>
    <math xmlns="http://www.w3.org/1998/Math/MathML">
      <ci> FLUX_VALUE </ci>
    </math>
    <listOfParameters>
      <parameter id="LOWER_BOUND" value="0" units="mmol_per_gDW_per_hr" constant="true"/>
      <parameter id="UPPER_BOUND" value="1000" units="mmol_per_gDW_per_hr" constant="true"/>
      <parameter id="FLUX_VALUE" value="0" units="mmol_per_gDW_per_hr" constant="true"/>
      <parameter id="OBJECTIVE_COEFFICIENT" value="0" units="mmol_per_gDW_per_hr" constant="true"/>
    </listOfParameters>
  </kineticLaw>
</reaction>
<reaction id="R_R395" name="Phsophatidyl-L-serine carboxy-lyase" reversible="false">
  <notes>
    <body xmlns="http://www.w3.org/1999/xhtml">
      <p>GENE_ASSOCIATION: TTC0815</p>
      <p>SUBSYSTEM: Glycerophospholipid metabolism</p>
      <p>EC Number: 4.1.1.65</p>
      <p>Confidence Level: 1</p>
      <p>AUTHORS: Lipid metabolism</p>
      <p/>
    </body>
  </notes>
  <listOfReactants>
    <speciesReference species="M_PPTD_SER_c" stoichiometry="1"/>
  </listOfReactants>
  <listOfProducts>
    <speciesReference species="M_PETHA_c" stoichiometry="1"/>
```

```
    <speciesReference species="M_CO2_c" stoichiometry="1"/>
  </listOfProducts>
  <kineticLaw>
    <math xmlns="http://www.w3.org/1998/Math/MathML">
      <ci> FLUX_VALUE </ci>
    </math>
    <listOfParameters>
      <parameter id="LOWER_BOUND" value="0" units="mmol_per_gDW_per_hr" constant="true"/>
      <parameter id="UPPER_BOUND" value="1000" units="mmol_per_gDW_per_hr" constant="true"/>
      <parameter id="FLUX_VALUE" value="0" units="mmol_per_gDW_per_hr" constant="true"/>
      <parameter id="OBJECTIVE_COEFFICIENT" value="0" units="mmol_per_gDW_per_hr" constant="true"/>
    </listOfParameters>
  </kineticLaw>
</reaction>
<reaction id="R_R396" name="CTP:phosphatidate cytidyltransferase" reversible="false">
  <notes>
    <body xmlns="http://www.w3.org/1999/xhtml">
      <p>GENE_ASSOCIATION: TTC0505</p>
      <p>SUBSYSTEM: Glycerophospholipid metabolism</p>
      <p>EC Number: 2.7.7.41</p>
      <p>Confidence Level: 1</p>
      <p>AUTHORS: Lipid metabolism</p>
      <p/>
    </body>
  </notes>
  <listOfReactants>
    <speciesReference species="M_CTP_c" stoichiometry="1"/>
    <speciesReference species="M_PPTD_c" stoichiometry="1"/>
  </listOfReactants>
```

```xml
<listOfProducts>
  <speciesReference species="M_PPi_c" stoichiometry="1"/>
  <speciesReference species="M_CDP_DAG_c" stoichiometry="1"/>
</listOfProducts>
<kineticLaw>
  <math xmlns="http://www.w3.org/1998/Math/MathML">
    <ci> FLUX_VALUE </ci>
  </math>
  <listOfParameters>
    <parameter id="LOWER_BOUND" value="0" units="mmol_per_gDW_per_hr" constant="true"/>
    <parameter id="UPPER_BOUND" value="1000" units="mmol_per_gDW_per_hr" constant="true"/>
    <parameter id="FLUX_VALUE" value="0" units="mmol_per_gDW_per_hr" constant="true"/>
    <parameter id="OBJECTIVE_COEFFICIENT" value="0" units="mmol_per_gDW_per_hr" constant="true"/>
  </listOfParameters>
</kineticLaw>
</reaction>
<reaction id="R_R397" name="CDPdiacylglycerol:sn-glycerol-3-phosphate 3-phosphatidyltransferase" reversible="false">
<notes>
  <body xmlns="http://www.w3.org/1999/xhtml">
    <p>GENE_ASSOCIATION: </p>
    <p>SUBSYSTEM: Glycerophospholipid metabolism</p>
    <p>EC Number: </p>
    <p>Confidence Level: 1</p>
    <p>AUTHORS: Lipid metabolism</p>
    <p/>
  </body>
</notes>
<listOfReactants>
  <speciesReference species="M_GLCP_c" stoichiometry="1"/>
```

```xml
<speciesReference species="M_CDP_DAG_c" stoichiometry="1"/>
</listOfReactants>
<listOfProducts>
<speciesReference species="M_H_c" stoichiometry="1"/>
<speciesReference species="M_CMP_c" stoichiometry="1"/>
<speciesReference species="M_PGLYP_c" stoichiometry="1"/>
</listOfProducts>
<kineticLaw>
<math xmlns="http://www.w3.org/1998/Math/MathML">
<ci> FLUX_VALUE </ci>
</math>
<listOfParameters>
<parameter id="LOWER_BOUND" value="0" units="mmol_per_gDW_per_hr" constant="true"/>
<parameter id="UPPER_BOUND" value="1000" units="mmol_per_gDW_per_hr" constant="true"/>
<parameter id="FLUX_VALUE" value="0" units="mmol_per_gDW_per_hr" constant="true"/>
<parameter id="OBJECTIVE_COEFFICIENT" value="0" units="mmol_per_gDW_per_hr" constant="true"/>
</listOfParameters>
</kineticLaw>
</reaction>
<reaction id="R_R398" name="Phosphatidylglycerophosphate phosphohydrolase" reversible="false">
<notes>
<body xmlns="http://www.w3.org/1999/xhtml">
<p>GENE_ASSOCIATION: </p>
<p>SUBSYSTEM: Glycerophospholipid metabolism</p>
<p>EC Number: 3.1.3.27</p>
<p>Confidence Level: 1</p>
<p>AUTHORS: Lipid metabolism</p>
<p/>
</body>
```

```xml
  </notes>
  <listOfReactants>
    <speciesReference species="M_H2O_c" stoichiometry="1"/>
    <speciesReference species="M_PGLYP_c" stoichiometry="1"/>
  </listOfReactants>
  <listOfProducts>
    <speciesReference species="M_PGLY_c" stoichiometry="1"/>
    <speciesReference species="M_Pi_c" stoichiometry="1"/>
  </listOfProducts>
  <kineticLaw>
    <math xmlns="http://www.w3.org/1998/Math/MathML">
      <ci> FLUX_VALUE </ci>
    </math>
    <listOfParameters>
      <parameter id="LOWER_BOUND" value="0" units="mmol_per_gDW_per_hr" constant="true"/>
      <parameter id="UPPER_BOUND" value="1000" units="mmol_per_gDW_per_hr" constant="true"/>
      <parameter id="FLUX_VALUE" value="0" units="mmol_per_gDW_per_hr" constant="true"/>
      <parameter id="OBJECTIVE_COEFFICIENT" value="0" units="mmol_per_gDW_per_hr" constant="true"/>
    </listOfParameters>
  </kineticLaw>
</reaction>
<reaction id="R_R399" name="phosphatidylinositol phosphate synthase" reversible="false">
  <notes>
    <body xmlns="http://www.w3.org/1999/xhtml">
      <p>GENE_ASSOCIATION: </p>
      <p>SUBSYSTEM: Glycerophospholipid metabolism</p>
      <p>EC Number: </p>
      <p>Confidence Level: 1</p>
      <p>AUTHORS: Lipid metabolism</p>
```

```
      <p/>
    </body>
  </notes>
  <listOfReactants>
    <speciesReference species="M_mINS3P_c" stoichiometry="1"/>
    <speciesReference species="M_CDP_DAG_c" stoichiometry="1"/>
  </listOfReactants>
  <listOfProducts>
    <speciesReference species="M_H_c" stoichiometry="1"/>
    <speciesReference species="M_CMP_c" stoichiometry="1"/>
    <speciesReference species="M_PMINS3P_c" stoichiometry="1"/>
  </listOfProducts>
  <kineticLaw>
    <math xmlns="http://www.w3.org/1998/Math/MathML">
      <ci> FLUX_VALUE </ci>
    </math>
    <listOfParameters>
      <parameter id="LOWER_BOUND" value="0" units="mmol_per_gDW_per_hr" constant="true"/>
      <parameter id="UPPER_BOUND" value="1000" units="mmol_per_gDW_per_hr" constant="true"/>
      <parameter id="FLUX_VALUE" value="0" units="mmol_per_gDW_per_hr" constant="true"/>
      <parameter id="OBJECTIVE_COEFFICIENT" value="0" units="mmol_per_gDW_per_hr" constant="true"/>
    </listOfParameters>
  </kineticLaw>
</reaction>
<reaction id="R_R400" name="1-phosphatidyl-1D-myo-inositol 3-phosphatase" reversible="false">
  <notes>
    <body xmlns="http://www.w3.org/1999/xhtml">
      <p>GENE_ASSOCIATION: </p>
      <p>SUBSYSTEM: Glycerophospholipid metabolism</p>
```

```
      <p>EC Number: 3.1.3.64</p>
      <p>Confidence Level: 1</p>
      <p>AUTHORS: Lipid metabolism</p>
      <p/>
    </body>
  </notes>
  <listOfReactants>
    <speciesReference species="M_H2O_c" stoichiometry="1"/>
    <speciesReference species="M_PMINS3P_c" stoichiometry="1"/>
  </listOfReactants>
  <listOfProducts>
    <speciesReference species="M_PINS_c" stoichiometry="1"/>
    <speciesReference species="M_Pi_c" stoichiometry="1"/>
  </listOfProducts>
  <kineticLaw>
    <math xmlns="http://www.w3.org/1998/Math/MathML">
      <ci> FLUX_VALUE </ci>
    </math>
    <listOfParameters>
      <parameter id="LOWER_BOUND" value="0" units="mmol_per_gDW_per_hr" constant="true"/>
      <parameter id="UPPER_BOUND" value="1000" units="mmol_per_gDW_per_hr" constant="true"/>
      <parameter id="FLUX_VALUE" value="0" units="mmol_per_gDW_per_hr" constant="true"/>
      <parameter id="OBJECTIVE_COEFFICIENT" value="0" units="mmol_per_gDW_per_hr" constant="true"/>
    </listOfParameters>
  </kineticLaw>
</reaction>
<reaction id="R_R401" name="cardiolipin synthase" reversible="false">
  <notes>
    <body xmlns="http://www.w3.org/1999/xhtml">
```

```
<p>GENE_ASSOCIATION: </p>

<p>SUBSYSTEM: Glycerophospholipid metabolism</p>

<p>EC Number: </p>

<p>Confidence Level: 1</p>

<p>AUTHORS: Lipid metabolism</p>

<p/>

</body>

</notes>

<listOfReactants>

<speciesReference species="M_PGLY_c" stoichiometry="2"/>

</listOfReactants>

<listOfProducts>

<speciesReference species="M_CDLPN_c" stoichiometry="1"/>

<speciesReference species="M_GLYCRL_c" stoichiometry="1"/>

</listOfProducts>

<kineticLaw>

<math xmlns="http://www.w3.org/1998/Math/MathML">

<ci> FLUX_VALUE </ci>

</math>

<listOfParameters>

<parameter id="LOWER_BOUND" value="0" units="mmol_per_gDW_per_hr" constant="true"/>

<parameter id="UPPER_BOUND" value="1000" units="mmol_per_gDW_per_hr" constant="true"/>

<parameter id="FLUX_VALUE" value="0" units="mmol_per_gDW_per_hr" constant="true"/>

<parameter id="OBJECTIVE_COEFFICIENT" value="0" units="mmol_per_gDW_per_hr" constant="true"/>

</listOfParameters>

</kineticLaw>

</reaction>

<reaction id="R_R402" name="Digalactosylceramide galactohydrolase" reversible="false">

<notes>
```

```
    <body xmlns="http://www.w3.org/1999/xhtml">
      <p>GENE_ASSOCIATION: TT_P0072</p>
      <p>SUBSYSTEM: Sphingolipid metabolism</p>
      <p>EC Number: 3.2.1.22</p>
      <p>Confidence Level: 1</p>
      <p>AUTHORS: Lipid metabolism</p>
      <p/>
    </body>
  </notes>
  <listOfReactants>
    <speciesReference species="M_H2O_c" stoichiometry="1"/>
    <speciesReference species="M_DGALSCRAMD_c" stoichiometry="1"/>
  </listOfReactants>
  <listOfProducts>
    <speciesReference species="M_D_GAL_c" stoichiometry="1"/>
    <speciesReference species="M_GALS_CA_c" stoichiometry="1"/>
  </listOfProducts>
  <kineticLaw>
    <math xmlns="http://www.w3.org/1998/Math/MathML">
      <ci> FLUX_VALUE </ci>
    </math>
    <listOfParameters>
      <parameter id="LOWER_BOUND" value="0" units="mmol_per_gDW_per_hr" constant="true"/>
      <parameter id="UPPER_BOUND" value="1000" units="mmol_per_gDW_per_hr" constant="true"/>
      <parameter id="FLUX_VALUE" value="0" units="mmol_per_gDW_per_hr" constant="true"/>
      <parameter id="OBJECTIVE_COEFFICIENT" value="0" units="mmol_per_gDW_per_hr" constant="true"/>
    </listOfParameters>
  </kineticLaw>
</reaction>
```

```
<reaction id="R_R403" name="UDP-N-acetylmuramoyl-L-alanyl-D-glutamyl-L-lysine:alanyl-D-alanine ligase (ADP-forming)" reversible="false">
  <notes>
    <body xmlns="http://www.w3.org/1999/xhtml">
      <p>GENE_ASSOCIATION: TTC0714</p>
      <p>SUBSYSTEM: Cell Envelope Biosynthesis</p>
      <p>EC Number: 6.3.2.10</p>
      <p>Confidence Level: 1</p>
      <p>AUTHORS: Lipid metabolism</p>
      <p/>
    </body>
  </notes>
  <listOfReactants>
    <speciesReference species="M_ATP_c" stoichiometry="1"/>
    <speciesReference species="M_D_ALA_D_ALA_c" stoichiometry="1"/>
    <speciesReference species="M_UDP_ACMUR_ALA_GLU_LYS_c" stoichiometry="1"/>
  </listOfReactants>
  <listOfProducts>
    <speciesReference species="M_ADP_c" stoichiometry="1"/>
    <speciesReference species="M_Pi_c" stoichiometry="1"/>
    <speciesReference species="M_UDP_MURAC_ALA_GLU_LYS_ALA_ALA_c" stoichiometry="1"/>
  </listOfProducts>
  <kineticLaw>
    <math xmlns="http://www.w3.org/1998/Math/MathML">
      <ci> FLUX_VALUE </ci>
    </math>
    <listOfParameters>
      <parameter id="LOWER_BOUND" value="0" units="mmol_per_gDW_per_hr" constant="true"/>
      <parameter id="UPPER_BOUND" value="1000" units="mmol_per_gDW_per_hr" constant="true"/>
```

```xml
<parameter id="FLUX_VALUE" value="0" units="mmol_per_gDW_per_hr" constant="true"/>

<parameter id="OBJECTIVE_COEFFICIENT" value="0" units="mmol_per_gDW_per_hr" constant="true"/>

</listOfParameters>

</kineticLaw>

</reaction>

<reaction id="R_R404" name="UDPMurAc(oyl-L-Ala-D-gamma-Glu-L-Lys-D-Ala-D-Ala):undecaprenyl-phosphate phospho-N-acetylmuramoyl-pentapeptide-transferase" reversible="false">

<notes>

<body xmlns="http://www.w3.org/1999/xhtml">

<p>GENE_ASSOCIATION: TTC0716</p>

<p>SUBSYSTEM: Cell Envelope Biosynthesis</p>

<p>EC Number: 2.7.8.13</p>

<p>Confidence Level: 1</p>

<p>AUTHORS: Lipid metabolism</p>

<p/>

</body>

</notes>

<listOfReactants>

<speciesReference species="M_UDP_MURAC_ALA_GLU_LYS_ALA_ALA_c" stoichiometry="1"/>

<speciesReference species="M_dt_pc_UNDECP_c" stoichiometry="1"/>

</listOfReactants>

<listOfProducts>

<speciesReference species="M_UMP_c" stoichiometry="1"/>

<speciesReference species="M_MURAC_ALA_GLU_LYS_ALA_ALA_DP_UNDECP_c" stoichiometry="1"/>

</listOfProducts>

<kineticLaw>

<math xmlns="http://www.w3.org/1998/Math/MathML">

<ci> FLUX_VALUE </ci>

</math>
```

```
<listOfParameters>
  <parameter id="LOWER_BOUND" value="0" units="mmol_per_gDW_per_hr" constant="true"/>
  <parameter id="UPPER_BOUND" value="1000" units="mmol_per_gDW_per_hr" constant="true"/>
  <parameter id="FLUX_VALUE" value="0" units="mmol_per_gDW_per_hr" constant="true"/>
  <parameter id="OBJECTIVE_COEFFICIENT" value="0" units="mmol_per_gDW_per_hr" constant="true"/>
</listOfParameters>
</kineticLaw>
</reaction>
<reaction id="R_R405" name="UDP-N-acetylmuramoyl-L-alanyl-gamma-D-glutamyl-meso-2,6-diaminopimeloyl-D-alanyl-D-alanine:undecaprenyl-phosphate phospho-N-acetylmuramoyl-pentapeptide-transferase" reversible="false">
<notes>
  <body xmlns="http://www.w3.org/1999/xhtml">
    <p>GENE_ASSOCIATION: TTC0716</p>
    <p>SUBSYSTEM: Cell Envelope Biosynthesis</p>
    <p>EC Number: 2.7.8.13</p>
    <p>Confidence Level: 1</p>
    <p>AUTHORS: Lipid metabolism</p>
    <p/>
  </body>
</notes>
<listOfReactants>
  <speciesReference species="M_UDP_ACMUR_ALA_GLU_DAPIM_ALA_ALA_c" stoichiometry="1"/>
  <speciesReference species="M_dt_pc_UNDECP_c" stoichiometry="1"/>
</listOfReactants>
<listOfProducts>
  <speciesReference species="M_UMP_c" stoichiometry="1"/>
  <speciesReference species="M_Undp_ACMUR_ALA_GLU_DAPIM_AALA_c" stoichiometry="1"/>
</listOfProducts>
<kineticLaw>
```

```xml
<math xmlns="http://www.w3.org/1998/Math/MathML">
  <ci> FLUX_VALUE </ci>
</math>
<listOfParameters>
  <parameter id="LOWER_BOUND" value="0" units="mmol_per_gDW_per_hr" constant="true"/>
  <parameter id="UPPER_BOUND" value="1000" units="mmol_per_gDW_per_hr" constant="true"/>
  <parameter id="FLUX_VALUE" value="0" units="mmol_per_gDW_per_hr" constant="true"/>
  <parameter id="OBJECTIVE_COEFFICIENT" value="0" units="mmol_per_gDW_per_hr" constant="true"/>
</listOfParameters>
</kineticLaw>
</reaction>
<reaction id="R_R406" name="UDP-N-acetyl-D-glucosamine:N-acetyl-alpha-D-muramyl(oyl-L-Ala-gamma-D-Glu-L-Lys-D-Ala-D-Ala)-diphosphoundecaprenol 4-beta-N-acetylglucosaminlytransferase" reversible="false">
  <notes>
    <body xmlns="http://www.w3.org/1999/xhtml">
      <p>GENE_ASSOCIATION: TTC0719</p>
      <p>SUBSYSTEM: Cell Envelope Biosynthesis</p>
      <p>EC Number: 2.4.1.227</p>
      <p>Confidence Level: 1</p>
      <p>AUTHORS: Lipid metabolism</p>
      <p/>
    </body>
  </notes>
  <listOfReactants>
    <speciesReference species="M_UDP_AGLUAM_c" stoichiometry="1"/>
    <speciesReference species="M_MURAC_ALA_GLU_LYS_ALA_ALA_DP_UNDECP_c" stoichiometry="1"/>
  </listOfReactants>
  <listOfProducts>
    <speciesReference species="M_UDP_c" stoichiometry="1"/>
```

```xml
<speciesReference species="M_Undp_ACMUR_ACGLSA_ALA_GLU_LYS_AALA_c" stoichiometry="1"/>
</listOfProducts>
<kineticLaw>
<math xmlns="http://www.w3.org/1998/Math/MathML">
<ci> FLUX_VALUE </ci>
</math>
<listOfParameters>
<parameter id="LOWER_BOUND" value="0" units="mmol_per_gDW_per_hr" constant="true"/>
<parameter id="UPPER_BOUND" value="1000" units="mmol_per_gDW_per_hr" constant="true"/>
<parameter id="FLUX_VALUE" value="0" units="mmol_per_gDW_per_hr" constant="true"/>
<parameter id="OBJECTIVE_COEFFICIENT" value="0" units="mmol_per_gDW_per_hr" constant="true"/>
</listOfParameters>
</kineticLaw>
</reaction>
<reaction id="R_R407" name="UDP-N-acetyl-D-glucosamine:undecaprenyl-diphospho-N-acetylmuramoyl-L-alanyl-gamma-D-glutamyl-meso-2,6-diaminopimeloyl-D-alanyl-D-alanine 4-beta-N-acetylglucosaminlytransferase" reversible="false">
<notes>
<body xmlns="http://www.w3.org/1999/xhtml">
<p>GENE_ASSOCIATION: TTC0719</p>
<p>SUBSYSTEM: Cell Envelope Biosynthesis</p>
<p>EC Number: 2.4.1.227</p>
<p>Confidence Level: 1</p>
<p>AUTHORS: Lipid metabolism</p>
<p/>
</body>
</notes>
<listOfReactants>
<speciesReference species="M_UDP_AGLUAM_c" stoichiometry="1"/>
<speciesReference species="M_Undp_ACMUR_ALA_GLU_DAPIM_AALA_c" stoichiometry="1"/>
```

```
  </listOfReactants>
  <listOfProducts>
    <speciesReference species="M_H_c" stoichiometry="1"/>
    <speciesReference species="M_UDP_c" stoichiometry="1"/>
    <speciesReference species="M_Undp_ACMUR_ACGLSA_ALA_GLU_DAPIM_AALA_c" stoichiometry="1"/>
  </listOfProducts>
  <kineticLaw>
    <math xmlns="http://www.w3.org/1998/Math/MathML">
      <ci> FLUX_VALUE </ci>
    </math>
    <listOfParameters>
      <parameter id="LOWER_BOUND" value="0" units="mmol_per_gDW_per_hr" constant="true"/>
      <parameter id="UPPER_BOUND" value="1000" units="mmol_per_gDW_per_hr" constant="true"/>
      <parameter id="FLUX_VALUE" value="0" units="mmol_per_gDW_per_hr" constant="true"/>
      <parameter id="OBJECTIVE_COEFFICIENT" value="0" units="mmol_per_gDW_per_hr" constant="true"/>
    </listOfParameters>
  </kineticLaw>
</reaction>
<reaction id="R_R408" name="undecaprenyl-diphosphate phosphohydrolase" reversible="false">
  <notes>
    <body xmlns="http://www.w3.org/1999/xhtml">
      <p>GENE_ASSOCIATION: TTC1814</p>
      <p>SUBSYSTEM: Cell Envelope Biosynthesis</p>
      <p>EC Number: 3.6.1.27</p>
      <p>Confidence Level: 1</p>
      <p>AUTHORS: Lipid metabolism</p>
      <p/>
    </body>
  </notes>
```

```xml
  <listOfReactants>
    <speciesReference species="M_H2O_c" stoichiometry="1"/>
    <speciesReference species="M_dt_pc_UNDECPPi_c" stoichiometry="1"/>
  </listOfReactants>
  <listOfProducts>
    <speciesReference species="M_Pi_c" stoichiometry="1"/>
    <speciesReference species="M_H_c" stoichiometry="1"/>
    <speciesReference species="M_dt_pc_UNDECP_c" stoichiometry="1"/>
  </listOfProducts>
  <kineticLaw>
    <math xmlns="http://www.w3.org/1998/Math/MathML">
      <ci> FLUX_VALUE </ci>
    </math>
    <listOfParameters>
      <parameter id="LOWER_BOUND" value="0" units="mmol_per_gDW_per_hr" constant="true"/>
      <parameter id="UPPER_BOUND" value="1000" units="mmol_per_gDW_per_hr" constant="true"/>
      <parameter id="FLUX_VALUE" value="0" units="mmol_per_gDW_per_hr" constant="true"/>
      <parameter id="OBJECTIVE_COEFFICIENT" value="0" units="mmol_per_gDW_per_hr" constant="true"/>
    </listOfParameters>
  </kineticLaw>
</reaction>
<reaction id="R_R409" name="Lactoylglutathione lyase" reversible="false">
  <notes>
    <body xmlns="http://www.w3.org/1999/xhtml">
      <p>GENE_ASSOCIATION: TTC0449</p>
      <p>SUBSYSTEM: Methylglyoxal Metabolism</p>
      <p>EC Number: 4.4.1.5</p>
      <p>Confidence Level: 1</p>
      <p>AUTHORS: Carbohydrate metabolism</p>
```

```
      <p/>
    </body>
  </notes>
  <listOfReactants>
    <speciesReference species="M_MGLX_c" stoichiometry="1"/>
    <speciesReference species="M_GLUTAT_c" stoichiometry="1"/>
  </listOfReactants>
  <listOfProducts>
    <speciesReference species="M_LACGLUT_c" stoichiometry="1"/>
  </listOfProducts>
  <kineticLaw>
    <math xmlns="http://www.w3.org/1998/Math/MathML">
      <ci> FLUX_VALUE </ci>
    </math>
    <listOfParameters>
      <parameter id="LOWER_BOUND" value="0" units="mmol_per_gDW_per_hr" constant="true"/>
      <parameter id="UPPER_BOUND" value="1000" units="mmol_per_gDW_per_hr" constant="true"/>
      <parameter id="FLUX_VALUE" value="0" units="mmol_per_gDW_per_hr" constant="true"/>
      <parameter id="OBJECTIVE_COEFFICIENT" value="0" units="mmol_per_gDW_per_hr" constant="true"/>
    </listOfParameters>
  </kineticLaw>
</reaction>
<reaction id="R_R410" name="hydroxyacylglutathione hydrolase" reversible="false">
  <notes>
    <body xmlns="http://www.w3.org/1999/xhtml">
      <p>GENE_ASSOCIATION: TTC0485</p>
      <p>SUBSYSTEM: Methylglyoxal Metabolism</p>
      <p>EC Number: 3.1.2.6</p>
      <p>Confidence Level: 1</p>
```

```
      <p>AUTHORS: Carbohydrate metabolism</p>
      <p/>
    </body>
  </notes>
  <listOfReactants>
    <speciesReference species="M_H2O_c" stoichiometry="1"/>
    <speciesReference species="M_LACGLUT_c" stoichiometry="1"/>
  </listOfReactants>
  <listOfProducts>
    <speciesReference species="M_H_c" stoichiometry="1"/>
    <speciesReference species="M_LAC_c" stoichiometry="1"/>
    <speciesReference species="M_GLUTAT_c" stoichiometry="1"/>
  </listOfProducts>
  <kineticLaw>
    <math xmlns="http://www.w3.org/1998/Math/MathML">
      <ci> FLUX_VALUE </ci>
    </math>
    <listOfParameters>
      <parameter id="LOWER_BOUND" value="0" units="mmol_per_gDW_per_hr" constant="true"/>
      <parameter id="UPPER_BOUND" value="1000" units="mmol_per_gDW_per_hr" constant="true"/>
      <parameter id="FLUX_VALUE" value="0" units="mmol_per_gDW_per_hr" constant="true"/>
      <parameter id="OBJECTIVE_COEFFICIENT" value="0" units="mmol_per_gDW_per_hr" constant="true"/>
    </listOfParameters>
  </kineticLaw>
</reaction>
<reaction id="R_R411" name="glycerone-phosphate phosphate-lyase (methylglyoxal-forming)" reversible="false">
  <notes>
    <body xmlns="http://www.w3.org/1999/xhtml">
      <p>GENE_ASSOCIATION: TTC1443</p>
```

```
      <p>SUBSYSTEM: Methylglyoxal Metabolism</p>
      <p>EC Number: 4.2.3.3</p>
      <p>Confidence Level: 1</p>
      <p>AUTHORS: Carbohydrate metabolism</p>
      <p/>
    </body>
  </notes>
  <listOfReactants>
    <speciesReference species="M_DHAP_c" stoichiometry="1"/>
  </listOfReactants>
  <listOfProducts>
    <speciesReference species="M_Pi_c" stoichiometry="1"/>
    <speciesReference species="M_MGLX_c" stoichiometry="1"/>
  </listOfProducts>
  <kineticLaw>
    <math xmlns="http://www.w3.org/1998/Math/MathML">
      <ci> FLUX_VALUE </ci>
    </math>
    <listOfParameters>
      <parameter id="LOWER_BOUND" value="0" units="mmol_per_gDW_per_hr" constant="true"/>
      <parameter id="UPPER_BOUND" value="1000" units="mmol_per_gDW_per_hr" constant="true"/>
      <parameter id="FLUX_VALUE" value="0" units="mmol_per_gDW_per_hr" constant="true"/>
      <parameter id="OBJECTIVE_COEFFICIENT" value="0" units="mmol_per_gDW_per_hr" constant="true"/>
    </listOfParameters>
  </kineticLaw>
</reaction>
<reaction id="R_R412" name="ATP:pyruvate,phosphate phosphotransferase" reversible="true">
  <notes>
    <body xmlns="http://www.w3.org/1999/xhtml">
```

```
    <p>GENE_ASSOCIATION: TTC0304</p>
    <p>SUBSYSTEM: Pyruvate metabolism</p>
    <p>EC Number: 2.7.9.1</p>
    <p>Confidence Level: 1</p>
    <p>AUTHORS: Carbohydrate metabolism</p>
    <p/>
  </body>
</notes>
<listOfReactants>
  <speciesReference species="M_ATP_c" stoichiometry="1"/>
  <speciesReference species="M_Pi_c" stoichiometry="1"/>
  <speciesReference species="M_PYR_c" stoichiometry="1"/>
</listOfReactants>
<listOfProducts>
  <speciesReference species="M_PPi_c" stoichiometry="1"/>
  <speciesReference species="M_H_c" stoichiometry="1"/>
  <speciesReference species="M_PEP_c" stoichiometry="1"/>
  <speciesReference species="M_AMP_c" stoichiometry="1"/>
</listOfProducts>
<kineticLaw>
  <math xmlns="http://www.w3.org/1998/Math/MathML">
    <ci> FLUX_VALUE </ci>
  </math>
  <listOfParameters>
    <parameter id="LOWER_BOUND" value="-1000" units="mmol_per_gDW_per_hr" constant="true"/>
    <parameter id="UPPER_BOUND" value="1000" units="mmol_per_gDW_per_hr" constant="true"/>
    <parameter id="FLUX_VALUE" value="0" units="mmol_per_gDW_per_hr" constant="true"/>
    <parameter id="OBJECTIVE_COEFFICIENT" value="0" units="mmol_per_gDW_per_hr" constant="true"/>
  </listOfParameters>
```

```
  </kineticLaw>
</reaction>
<reaction id="R_R413" name="ATP:pyruvate,water phosphotransferase" reversible="false">
  <notes>
    <body xmlns="http://www.w3.org/1999/xhtml">
      <p>GENE_ASSOCIATION: TTC1136</p>
      <p>SUBSYSTEM: Glycolysis/Gluconeogenesis</p>
      <p>EC Number: 2.7.9.2</p>
      <p>Confidence Level: 1</p>
      <p>AUTHORS: Carbohydrate metabolism</p>
      <p/>
    </body>
  </notes>
  <listOfReactants>
    <speciesReference species="M_ATP_c" stoichiometry="1"/>
    <speciesReference species="M_H2O_c" stoichiometry="1"/>
    <speciesReference species="M_PYR_c" stoichiometry="1"/>
  </listOfReactants>
  <listOfProducts>
    <speciesReference species="M_Pi_c" stoichiometry="1"/>
    <speciesReference species="M_H_c" stoichiometry="2"/>
    <speciesReference species="M_PEP_c" stoichiometry="1"/>
    <speciesReference species="M_AMP_c" stoichiometry="1"/>
  </listOfProducts>
  <kineticLaw>
    <math xmlns="http://www.w3.org/1998/Math/MathML">
      <ci> FLUX_VALUE </ci>
    </math>
    <listOfParameters>
```

```
      <parameter id="LOWER_BOUND" value="0" units="mmol_per_gDW_per_hr" constant="true"/>
      <parameter id="UPPER_BOUND" value="1000" units="mmol_per_gDW_per_hr" constant="true"/>
      <parameter id="FLUX_VALUE" value="0" units="mmol_per_gDW_per_hr" constant="true"/>
      <parameter id="OBJECTIVE_COEFFICIENT" value="0" units="mmol_per_gDW_per_hr" constant="true"/>
    </listOfParameters>
  </kineticLaw>
</reaction>
<reaction id="R_R414" name="(S)-lactate:oxygen 2-oxidoreductase (decarboxylating)" reversible="true">
  <notes>
    <body xmlns="http://www.w3.org/1999/xhtml">
      <p>GENE_ASSOCIATION: TTC1744</p>
      <p>SUBSYSTEM: Pyruvate metabolism</p>
      <p>EC Number: 1.13.12.4</p>
      <p>Confidence Level: 1</p>
      <p>AUTHORS: Carbohydrate metabolism</p>
      <p/>
    </body>
  </notes>
  <listOfReactants>
    <speciesReference species="M_LAC_c" stoichiometry="1"/>
    <speciesReference species="M_O2_c" stoichiometry="1"/>
  </listOfReactants>
  <listOfProducts>
    <speciesReference species="M_H2O_c" stoichiometry="1"/>
    <speciesReference species="M_CO2_c" stoichiometry="1"/>
    <speciesReference species="M_AC_c" stoichiometry="1"/>
  </listOfProducts>
  <kineticLaw>
    <math xmlns="http://www.w3.org/1998/Math/MathML">
```

```
      <ci> FLUX_VALUE </ci>
    </math>
    <listOfParameters>
      <parameter id="LOWER_BOUND" value="0" units="mmol_per_gDW_per_hr" constant="true"/>
      <parameter id="UPPER_BOUND" value="0" units="mmol_per_gDW_per_hr" constant="true"/>
      <parameter id="FLUX_VALUE" value="0" units="mmol_per_gDW_per_hr" constant="true"/>
      <parameter id="OBJECTIVE_COEFFICIENT" value="0" units="mmol_per_gDW_per_hr" constant="true"/>
    </listOfParameters>
  </kineticLaw>
</reaction>
<reaction id="R_R415" name="Acetyl phosphate phosphohydrolase" reversible="false">
  <notes>
    <body xmlns="http://www.w3.org/1999/xhtml">
      <p>GENE_ASSOCIATION: TTC0199</p>
      <p>SUBSYSTEM: Pyruvate metabolism</p>
      <p>EC Number: 3.6.1.7</p>
      <p>Confidence Level: 1</p>
      <p>AUTHORS: Carbohydrate metabolism</p>
      <p/>
    </body>
  </notes>
  <listOfReactants>
    <speciesReference species="M_H2O_c" stoichiometry="1"/>
    <speciesReference species="M_ACPi_c" stoichiometry="1"/>
  </listOfReactants>
  <listOfProducts>
    <speciesReference species="M_Pi_c" stoichiometry="1"/>
    <speciesReference species="M_H_c" stoichiometry="1"/>
    <speciesReference species="M_AC_c" stoichiometry="1"/>
```

```
  </listOfProducts>
  <kineticLaw>
    <math xmlns="http://www.w3.org/1998/Math/MathML">
      <ci> FLUX_VALUE </ci>
    </math>
    <listOfParameters>
      <parameter id="LOWER_BOUND" value="0" units="mmol_per_gDW_per_hr" constant="true"/>
      <parameter id="UPPER_BOUND" value="1000" units="mmol_per_gDW_per_hr" constant="true"/>
      <parameter id="FLUX_VALUE" value="0" units="mmol_per_gDW_per_hr" constant="true"/>
      <parameter id="OBJECTIVE_COEFFICIENT" value="0" units="mmol_per_gDW_per_hr" constant="true"/>
    </listOfParameters>
  </kineticLaw>
</reaction>
<reaction id="R_R416" name="acetyl adenylate:CoA acetyltransferase" reversible="true">
  <notes>
    <body xmlns="http://www.w3.org/1999/xhtml">
      <p>GENE_ASSOCIATION: (TTC0884 or TTC0885 or TTC0886 or TTC0919)</p>
      <p>SUBSYSTEM: Pyruvate metabolism</p>
      <p>EC Number: 6.2.1.1</p>
      <p>Confidence Level: 1</p>
      <p>AUTHORS: Carbohydrate metabolism</p>
      <p/>
    </body>
  </notes>
  <listOfReactants>
    <speciesReference species="M_CoA_c" stoichiometry="1"/>
    <speciesReference species="M_ACADNA_c" stoichiometry="1"/>
  </listOfReactants>
  <listOfProducts>
```

```
    <speciesReference species="M_AMP_c" stoichiometry="1"/>
    <speciesReference species="M_ACoA_c" stoichiometry="1"/>
  </listOfProducts>
  <kineticLaw>
    <math xmlns="http://www.w3.org/1998/Math/MathML">
      <ci> FLUX_VALUE </ci>
    </math>
    <listOfParameters>
      <parameter id="LOWER_BOUND" value="-1000" units="mmol_per_gDW_per_hr" constant="true"/>
      <parameter id="UPPER_BOUND" value="1000" units="mmol_per_gDW_per_hr" constant="true"/>
      <parameter id="FLUX_VALUE" value="0" units="mmol_per_gDW_per_hr" constant="true"/>
      <parameter id="OBJECTIVE_COEFFICIENT" value="0" units="mmol_per_gDW_per_hr" constant="true"/>
    </listOfParameters>
  </kineticLaw>
</reaction>
<reaction id="R_R417" name="ATP:acetate adenylyltransferase" reversible="true">
  <notes>
    <body xmlns="http://www.w3.org/1999/xhtml">
      <p>GENE_ASSOCIATION: (TTC0884 or TTC0885 or TTC0886 or TTC0919)</p>
      <p>SUBSYSTEM: Pyruvate metabolism</p>
      <p>EC Number: 6.2.1.1</p>
      <p>Confidence Level: 1</p>
      <p>AUTHORS: Carbohydrate metabolism</p>
      <p/>
    </body>
  </notes>
  <listOfReactants>
    <speciesReference species="M_ATP_c" stoichiometry="1"/>
    <speciesReference species="M_AC_c" stoichiometry="1"/>
```

```
  </listOfReactants>
  <listOfProducts>
    <speciesReference species="M_PPi_c" stoichiometry="1"/>
    <speciesReference species="M_ACADNA_c" stoichiometry="1"/>
  </listOfProducts>
  <kineticLaw>
    <math xmlns="http://www.w3.org/1998/Math/MathML">
      <ci> FLUX_VALUE </ci>
    </math>
    <listOfParameters>
      <parameter id="LOWER_BOUND" value="0" units="mmol_per_gDW_per_hr" constant="true"/>
      <parameter id="UPPER_BOUND" value="0" units="mmol_per_gDW_per_hr" constant="true"/>
      <parameter id="FLUX_VALUE" value="0" units="mmol_per_gDW_per_hr" constant="true"/>
      <parameter id="OBJECTIVE_COEFFICIENT" value="0" units="mmol_per_gDW_per_hr" constant="true"/>
    </listOfParameters>
  </kineticLaw>
</reaction>
<reaction id="R_R418" name="(S)-malate:NAD+ oxidoreductase (decarboxylating)" reversible="false">
  <notes>
    <body xmlns="http://www.w3.org/1999/xhtml">
      <p>GENE_ASSOCIATION: TTC0144</p>
      <p>SUBSYSTEM: Pyruvate metabolism</p>
      <p>EC Number: 1.1.1.38</p>
      <p>Confidence Level: 1</p>
      <p>AUTHORS: Carbohydrate metabolism</p>
      <p/>
    </body>
  </notes>
  <listOfReactants>
```

```
      <speciesReference species="M_NAD_c" stoichiometry="1"/>
      <speciesReference species="M_MAL_c" stoichiometry="1"/>
    </listOfReactants>
    <listOfProducts>
      <speciesReference species="M_NADH_c" stoichiometry="1"/>
      <speciesReference species="M_PYR_c" stoichiometry="1"/>
      <speciesReference species="M_CO2_c" stoichiometry="1"/>
    </listOfProducts>
    <kineticLaw>
      <math xmlns="http://www.w3.org/1998/Math/MathML">
        <ci> FLUX_VALUE </ci>
      </math>
      <listOfParameters>
        <parameter id="LOWER_BOUND" value="0" units="mmol_per_gDW_per_hr" constant="true"/>
        <parameter id="UPPER_BOUND" value="1000" units="mmol_per_gDW_per_hr" constant="true"/>
        <parameter id="FLUX_VALUE" value="0" units="mmol_per_gDW_per_hr" constant="true"/>
        <parameter id="OBJECTIVE_COEFFICIENT" value="0" units="mmol_per_gDW_per_hr" constant="true"/>
      </listOfParameters>
    </kineticLaw>
  </reaction>
  <reaction id="R_R419" name="Glycolate oxidase" reversible="false">
    <notes>
      <body xmlns="http://www.w3.org/1999/xhtml">
        <p>GENE_ASSOCIATION: TTC1940</p>
        <p>SUBSYSTEM: Pyruvate metabolism</p>
        <p>EC Number: 1.1.19.14</p>
        <p>Confidence Level: 1</p>
        <p>AUTHORS: Carbohydrate metabolism</p>
        <p/>
```

```
      </body>
    </notes>
    <listOfReactants>
      <speciesReference species="M_NAD_c" stoichiometry="1"/>
      <speciesReference species="M_HY_AC_c" stoichiometry="1"/>
    </listOfReactants>
    <listOfProducts>
      <speciesReference species="M_H_c" stoichiometry="1"/>
      <speciesReference species="M_NADH_c" stoichiometry="1"/>
      <speciesReference species="M_GLOXT_c" stoichiometry="1"/>
    </listOfProducts>
    <kineticLaw>
      <math xmlns="http://www.w3.org/1998/Math/MathML">
        <ci> FLUX_VALUE </ci>
      </math>
      <listOfParameters>
        <parameter id="LOWER_BOUND" value="0" units="mmol_per_gDW_per_hr" constant="true"/>
        <parameter id="UPPER_BOUND" value="1000" units="mmol_per_gDW_per_hr" constant="true"/>
        <parameter id="FLUX_VALUE" value="0" units="mmol_per_gDW_per_hr" constant="true"/>
        <parameter id="OBJECTIVE_COEFFICIENT" value="0" units="mmol_per_gDW_per_hr" constant="true"/>
      </listOfParameters>
    </kineticLaw>
  </reaction>
  <reaction id="R_R420" name="acetyl-CoA:glyoxylate C-acetyltransferase (thioester-hydrolysing, carboxymethyl-forming)"
reversible="false">
    <notes>
      <body xmlns="http://www.w3.org/1999/xhtml">
        <p>GENE_ASSOCIATION: TTC0130</p>
        <p>SUBSYSTEM: Pyruvate metabolism</p>
```

```xml
      <p>EC Number: 2.3.3.9</p>
      <p>Confidence Level: 1</p>
      <p>AUTHORS: Carbohydrate metabolism</p>
      <p/>
    </body>
  </notes>
  <listOfReactants>
    <speciesReference species="M_H2O_c" stoichiometry="1"/>
    <speciesReference species="M_ACoA_c" stoichiometry="1"/>
    <speciesReference species="M_GLOXT_c" stoichiometry="1"/>
  </listOfReactants>
  <listOfProducts>
    <speciesReference species="M_H_c" stoichiometry="1"/>
    <speciesReference species="M_CoA_c" stoichiometry="1"/>
    <speciesReference species="M_MAL_c" stoichiometry="1"/>
  </listOfProducts>
  <kineticLaw>
    <math xmlns="http://www.w3.org/1998/Math/MathML">
      <ci> FLUX_VALUE </ci>
    </math>
    <listOfParameters>
      <parameter id="LOWER_BOUND" value="0" units="mmol_per_gDW_per_hr" constant="true"/>
      <parameter id="UPPER_BOUND" value="1000" units="mmol_per_gDW_per_hr" constant="true"/>
      <parameter id="FLUX_VALUE" value="0" units="mmol_per_gDW_per_hr" constant="true"/>
      <parameter id="OBJECTIVE_COEFFICIENT" value="0" units="mmol_per_gDW_per_hr" constant="true"/>
    </listOfParameters>
  </kineticLaw>
</reaction>
<reaction id="R_R421" name="Formate:tetrahydrofolate ligase (ADP-forming)" reversible="true">
```

```
<notes>
  <body xmlns="http://www.w3.org/1999/xhtml">
    <p>GENE_ASSOCIATION: TTC1707</p>
    <p>SUBSYSTEM: Folate Metabolism</p>
    <p>EC Number: 6.3.4.3</p>
    <p>Confidence Level: 1</p>
    <p>AUTHORS: Energy and cofactors metabolism</p>
    <p/>
  </body>
</notes>
<listOfReactants>
  <speciesReference species="M_ATP_c" stoichiometry="1"/>
  <speciesReference species="M_THF_c" stoichiometry="1"/>
  <speciesReference species="M_FOR_c" stoichiometry="1"/>
</listOfReactants>
<listOfProducts>
  <speciesReference species="M_ADP_c" stoichiometry="1"/>
  <speciesReference species="M_Pi_c" stoichiometry="1"/>
  <speciesReference species="M_10F_THF_c" stoichiometry="1"/>
</listOfProducts>
<kineticLaw>
  <math xmlns="http://www.w3.org/1998/Math/MathML">
    <ci> FLUX_VALUE </ci>
  </math>
  <listOfParameters>
    <parameter id="LOWER_BOUND" value="-1000" units="mmol_per_gDW_per_hr" constant="true"/>
    <parameter id="UPPER_BOUND" value="1000" units="mmol_per_gDW_per_hr" constant="true"/>
    <parameter id="FLUX_VALUE" value="0" units="mmol_per_gDW_per_hr" constant="true"/>
    <parameter id="OBJECTIVE_COEFFICIENT" value="0" units="mmol_per_gDW_per_hr" constant="true"/>
```

```
    </listOfParameters>
  </kineticLaw>
</reaction>
<reaction id="R_R422" name="10-Formyltetrahydrofolate amidohydrolase" reversible="false">
  <notes>
    <body xmlns="http://www.w3.org/1999/xhtml">
      <p>GENE_ASSOCIATION: TTC0957</p>
      <p>SUBSYSTEM: Folate Metabolism</p>
      <p>EC Number: 3.5.1.10</p>
      <p>Confidence Level: 1</p>
      <p>AUTHORS: Energy and cofactors metabolism</p>
      <p/>
    </body>
  </notes>
  <listOfReactants>
    <speciesReference species="M_H2O_c" stoichiometry="1"/>
    <speciesReference species="M_10F_THF_c" stoichiometry="1"/>
  </listOfReactants>
  <listOfProducts>
    <speciesReference species="M_H_c" stoichiometry="1"/>
    <speciesReference species="M_THF_c" stoichiometry="1"/>
    <speciesReference species="M_FOR_c" stoichiometry="1"/>
  </listOfProducts>
  <kineticLaw>
    <math xmlns="http://www.w3.org/1998/Math/MathML">
      <ci> FLUX_VALUE </ci>
    </math>
    <listOfParameters>
      <parameter id="LOWER_BOUND" value="0" units="mmol_per_gDW_per_hr" constant="true"/>
```

```
      <parameter id="UPPER_BOUND" value="1000" units="mmol_per_gDW_per_hr" constant="true"/>

      <parameter id="FLUX_VALUE" value="0" units="mmol_per_gDW_per_hr" constant="true"/>

      <parameter id="OBJECTIVE_COEFFICIENT" value="0" units="mmol_per_gDW_per_hr" constant="true"/>

    </listOfParameters>

  </kineticLaw>

</reaction>

<reaction id="R_R423" name="5,10-Methenyltetrahydrofolate 5-hydrolase (decyclizing)" reversible="true">

  <notes>

    <body xmlns="http://www.w3.org/1999/xhtml">

      <p>GENE_ASSOCIATION: TTC0755</p>

      <p>SUBSYSTEM: Folate Metabolism</p>

      <p>EC Number: 3.5.4.9</p>

      <p>Confidence Level: 1</p>

      <p>AUTHORS: Energy and cofactors metabolism</p>

      <p/>

    </body>

  </notes>

  <listOfReactants>

    <speciesReference species="M_H_c" stoichiometry="1"/>

    <speciesReference species="M_10F_THF_c" stoichiometry="1"/>

  </listOfReactants>

  <listOfProducts>

    <speciesReference species="M_H2O_c" stoichiometry="1"/>

    <speciesReference species="M_METHF_c" stoichiometry="1"/>

  </listOfProducts>

  <kineticLaw>

    <math xmlns="http://www.w3.org/1998/Math/MathML">

      <ci> FLUX_VALUE </ci>

    </math>
```

```
    <listOfParameters>
      <parameter id="LOWER_BOUND" value="-1000" units="mmol_per_gDW_per_hr" constant="true"/>
      <parameter id="UPPER_BOUND" value="1000" units="mmol_per_gDW_per_hr" constant="true"/>
      <parameter id="FLUX_VALUE" value="0" units="mmol_per_gDW_per_hr" constant="true"/>
      <parameter id="OBJECTIVE_COEFFICIENT" value="0" units="mmol_per_gDW_per_hr" constant="true"/>
    </listOfParameters>
  </kineticLaw>
</reaction>
<reaction id="R_R424" name="5,10-methylenetetrahydrofolate:NADP+ oxidoreductase" reversible="true">
  <notes>
    <body xmlns="http://www.w3.org/1999/xhtml">
      <p>GENE_ASSOCIATION: TTC0755</p>
      <p>SUBSYSTEM: Folate Metabolism</p>
      <p>EC Number: 1.5.1.5</p>
      <p>Confidence Level: 1</p>
      <p>AUTHORS: Energy and cofactors metabolism</p>
      <p/>
    </body>
  </notes>
  <listOfReactants>
    <speciesReference species="M_NADH_c" stoichiometry="1"/>
    <speciesReference species="M_METHF_c" stoichiometry="1"/>
  </listOfReactants>
  <listOfProducts>
    <speciesReference species="M_NAD_c" stoichiometry="1"/>
    <speciesReference species="M_5_10_MNTHF_c" stoichiometry="1"/>
  </listOfProducts>
  <kineticLaw>
    <math xmlns="http://www.w3.org/1998/Math/MathML">
```

```xml
      <ci> FLUX_VALUE </ci>
    </math>
    <listOfParameters>
      <parameter id="LOWER_BOUND" value="-1000" units="mmol_per_gDW_per_hr" constant="true"/>
      <parameter id="UPPER_BOUND" value="1000" units="mmol_per_gDW_per_hr" constant="true"/>
      <parameter id="FLUX_VALUE" value="0" units="mmol_per_gDW_per_hr" constant="true"/>
      <parameter id="OBJECTIVE_COEFFICIENT" value="0" units="mmol_per_gDW_per_hr" constant="true"/>
    </listOfParameters>
  </kineticLaw>
</reaction>
<reaction id="R_R425" name="isocitrate glyoxylate-lyase (succinate-forming)" reversible="false">
  <notes>
    <body xmlns="http://www.w3.org/1999/xhtml">
      <p>GENE_ASSOCIATION: TTC1485</p>
      <p>SUBSYSTEM: Glyoxylate and dicarboxylate metabolism</p>
      <p>EC Number: 4.1.3.1</p>
      <p>Confidence Level: 1</p>
      <p>AUTHORS: Carbohydrate metabolism</p>
      <p/>
    </body>
  </notes>
  <listOfReactants>
    <speciesReference species="M_ICIT_c" stoichiometry="1"/>
  </listOfReactants>
  <listOfProducts>
    <speciesReference species="M_SUCC_c" stoichiometry="1"/>
    <speciesReference species="M_GLOXT_c" stoichiometry="1"/>
  </listOfProducts>
  <kineticLaw>
```

```
    <math xmlns="http://www.w3.org/1998/Math/MathML">
      <ci> FLUX_VALUE </ci>
    </math>
    <listOfParameters>
      <parameter id="LOWER_BOUND" value="0" units="mmol_per_gDW_per_hr" constant="true"/>
      <parameter id="UPPER_BOUND" value="1000" units="mmol_per_gDW_per_hr" constant="true"/>
      <parameter id="FLUX_VALUE" value="0" units="mmol_per_gDW_per_hr" constant="true"/>
      <parameter id="OBJECTIVE_COEFFICIENT" value="0" units="mmol_per_gDW_per_hr" constant="true"/>
    </listOfParameters>
  </kineticLaw>
</reaction>
<reaction id="R_R426" name="4-hydroxy-2-oxoglutarate glyoxylate-lyase (pyruvate-forming)" reversible="true">
  <notes>
    <body xmlns="http://www.w3.org/1999/xhtml">
      <p>GENE_ASSOCIATION: TT_P0030</p>
      <p>SUBSYSTEM: Glyoxylate and dicarboxylate metabolism</p>
      <p>EC Number: 4.1.3.16</p>
      <p>Confidence Level: 1</p>
      <p>AUTHORS: Carbohydrate metabolism</p>
      <p/>
    </body>
  </notes>
  <listOfReactants>
    <speciesReference species="M_4HY_2OGLUTR_c" stoichiometry="1"/>
  </listOfReactants>
  <listOfProducts>
    <speciesReference species="M_PYR_c" stoichiometry="1"/>
    <speciesReference species="M_GLOXT_c" stoichiometry="1"/>
  </listOfProducts>
```

```
<kineticLaw>
  <math xmlns="http://www.w3.org/1998/Math/MathML">
    <ci> FLUX_VALUE </ci>
  </math>
  <listOfParameters>
    <parameter id="LOWER_BOUND" value="-1000" units="mmol_per_gDW_per_hr" constant="true"/>
    <parameter id="UPPER_BOUND" value="1000" units="mmol_per_gDW_per_hr" constant="true"/>
    <parameter id="FLUX_VALUE" value="0" units="mmol_per_gDW_per_hr" constant="true"/>
    <parameter id="OBJECTIVE_COEFFICIENT" value="0" units="mmol_per_gDW_per_hr" constant="true"/>
  </listOfParameters>
</kineticLaw>
</reaction>
<reaction id="R_R427" name="Glycolate:oxygen 2-oxidoreductase" reversible="false">
<notes>
  <body xmlns="http://www.w3.org/1999/xhtml">
    <p>GENE_ASSOCIATION: (TTC0129 or TTC0443)</p>
    <p>SUBSYSTEM: Glyoxylate and dicarboxylate metabolism</p>
    <p>EC Number: 1.1.3.15</p>
    <p>Confidence Level: 1</p>
    <p>AUTHORS: Carbohydrate metabolism</p>
    <p/>
  </body>
</notes>
<listOfReactants>
  <speciesReference species="M_O2_c" stoichiometry="1"/>
  <speciesReference species="M_HY_AC_c" stoichiometry="1"/>
</listOfReactants>
<listOfProducts>
  <speciesReference species="M_GLOXT_c" stoichiometry="1"/>
```

```xml
      <speciesReference species="M_H2O2_c" stoichiometry="1"/>
    </listOfProducts>
    <kineticLaw>
      <math xmlns="http://www.w3.org/1998/Math/MathML">
        <ci> FLUX_VALUE </ci>
      </math>
      <listOfParameters>
        <parameter id="LOWER_BOUND" value="0" units="mmol_per_gDW_per_hr" constant="true"/>
        <parameter id="UPPER_BOUND" value="1000" units="mmol_per_gDW_per_hr" constant="true"/>
        <parameter id="FLUX_VALUE" value="0" units="mmol_per_gDW_per_hr" constant="true"/>
        <parameter id="OBJECTIVE_COEFFICIENT" value="0" units="mmol_per_gDW_per_hr" constant="true"/>
      </listOfParameters>
    </kineticLaw>
  </reaction>
  <reaction id="R_R428" name="Glycolate:NAD+ oxidoreductase" reversible="false">
    <notes>
      <body xmlns="http://www.w3.org/1999/xhtml">
        <p>GENE_ASSOCIATION: TTC0431</p>
        <p>SUBSYSTEM: Glyoxylate and dicarboxylate metabolism</p>
        <p>EC Number: 1.1.1.26</p>
        <p>Confidence Level: 1</p>
        <p>AUTHORS: Carbohydrate metabolism</p>
        <p/>
      </body>
    </notes>
    <listOfReactants>
      <speciesReference species="M_H_c" stoichiometry="1"/>
      <speciesReference species="M_NADH_c" stoichiometry="1"/>
      <speciesReference species="M_GLOXT_c" stoichiometry="1"/>
```

```
  </listOfReactants>
  <listOfProducts>
    <speciesReference species="M_NAD_c" stoichiometry="1"/>
    <speciesReference species="M_HY_AC_c" stoichiometry="1"/>
  </listOfProducts>
  <kineticLaw>
    <math xmlns="http://www.w3.org/1998/Math/MathML">
      <ci> FLUX_VALUE </ci>
    </math>
    <listOfParameters>
      <parameter id="LOWER_BOUND" value="0" units="mmol_per_gDW_per_hr" constant="true"/>
      <parameter id="UPPER_BOUND" value="1000" units="mmol_per_gDW_per_hr" constant="true"/>
      <parameter id="FLUX_VALUE" value="0" units="mmol_per_gDW_per_hr" constant="true"/>
      <parameter id="OBJECTIVE_COEFFICIENT" value="0" units="mmol_per_gDW_per_hr" constant="true"/>
    </listOfParameters>
  </kineticLaw>
</reaction>
<reaction id="R_R429" name="2-Phosphoglycolate phosphohydrolase" reversible="false">
  <notes>
    <body xmlns="http://www.w3.org/1999/xhtml">
      <p>GENE_ASSOCIATION: TTC1471</p>
      <p>SUBSYSTEM: Glyoxylate and dicarboxylate metabolism</p>
      <p>EC Number: 3.1.3.18</p>
      <p>Confidence Level: 1</p>
      <p>AUTHORS: Carbohydrate metabolism</p>
      <p/>
    </body>
  </notes>
  <listOfReactants>
```

```
    <speciesReference species="M_H2O_c" stoichiometry="1"/>
    <speciesReference species="M_2P_GLYCA_c" stoichiometry="1"/>
  </listOfReactants>
  <listOfProducts>
    <speciesReference species="M_Pi_c" stoichiometry="1"/>
    <speciesReference species="M_HY_AC_c" stoichiometry="1"/>
  </listOfProducts>
  <kineticLaw>
    <math xmlns="http://www.w3.org/1998/Math/MathML">
      <ci> FLUX_VALUE </ci>
    </math>
    <listOfParameters>
      <parameter id="LOWER_BOUND" value="0" units="mmol_per_gDW_per_hr" constant="true"/>
      <parameter id="UPPER_BOUND" value="1000" units="mmol_per_gDW_per_hr" constant="true"/>
      <parameter id="FLUX_VALUE" value="0" units="mmol_per_gDW_per_hr" constant="true"/>
      <parameter id="OBJECTIVE_COEFFICIENT" value="0" units="mmol_per_gDW_per_hr" constant="true"/>
    </listOfParameters>
  </kineticLaw>
</reaction>
<reaction id="R_R430" name="2-Hydroxybutyrate:NAD+ oxidoreductase" reversible="false">
  <notes>
    <body xmlns="http://www.w3.org/1999/xhtml">
      <p>GENE_ASSOCIATION: TTC0748</p>
      <p>SUBSYSTEM: Propanoate metabolism</p>
      <p>EC Number: 1.1.1.27</p>
      <p>Confidence Level: 1</p>
      <p>AUTHORS: Lipid metabolism</p>
      <p/>
    </body>
```

```
  </notes>
  <listOfReactants>
    <speciesReference species="M_NAD_c" stoichiometry="1"/>
    <speciesReference species="M_2HYBUTNA_c" stoichiometry="1"/>
  </listOfReactants>
  <listOfProducts>
    <speciesReference species="M_H_c" stoichiometry="1"/>
    <speciesReference species="M_NADH_c" stoichiometry="1"/>
    <speciesReference species="M_AKB_c" stoichiometry="1"/>
  </listOfProducts>
  <kineticLaw>
    <math xmlns="http://www.w3.org/1998/Math/MathML">
      <ci> FLUX_VALUE </ci>
    </math>
    <listOfParameters>
      <parameter id="LOWER_BOUND" value="0" units="mmol_per_gDW_per_hr" constant="true"/>
      <parameter id="UPPER_BOUND" value="1000" units="mmol_per_gDW_per_hr" constant="true"/>
      <parameter id="FLUX_VALUE" value="0" units="mmol_per_gDW_per_hr" constant="true"/>
      <parameter id="OBJECTIVE_COEFFICIENT" value="0" units="mmol_per_gDW_per_hr" constant="true"/>
    </listOfParameters>
  </kineticLaw>
</reaction>
<reaction id="R_R431" name="Propionyladenylate:CoA propionyltransferase" reversible="true">
  <notes>
    <body xmlns="http://www.w3.org/1999/xhtml">
      <p>GENE_ASSOCIATION: (TTC0884 or TTC0885 or TTC0886 or TTC0919)</p>
      <p>SUBSYSTEM: Propanoate metabolism</p>
      <p>EC Number: 6.2.1.1</p>
      <p>Confidence Level: 1</p>
```

```
<p>AUTHORS: Lipid metabolism</p>

<p/>

</body>

</notes>

<listOfReactants>

<speciesReference species="M_CoA_c" stoichiometry="1"/>

<speciesReference species="M_PROP_ADNSPi_c" stoichiometry="1"/>

</listOfReactants>

<listOfProducts>

<speciesReference species="M_AMP_c" stoichiometry="1"/>

<speciesReference species="M_PRCoA_c" stoichiometry="1"/>

</listOfProducts>

<kineticLaw>

<math xmlns="http://www.w3.org/1998/Math/MathML">

<ci> FLUX_VALUE </ci>

</math>

<listOfParameters>

<parameter id="LOWER_BOUND" value="-1000" units="mmol_per_gDW_per_hr" constant="true"/>

<parameter id="UPPER_BOUND" value="1000" units="mmol_per_gDW_per_hr" constant="true"/>

<parameter id="FLUX_VALUE" value="0" units="mmol_per_gDW_per_hr" constant="true"/>

<parameter id="OBJECTIVE_COEFFICIENT" value="0" units="mmol_per_gDW_per_hr" constant="true"/>

</listOfParameters>

</kineticLaw>

</reaction>

<reaction id="R_R432" name="ATP:propanoate adenylyltransferase" reversible="true">

<notes>

<body xmlns="http://www.w3.org/1999/xhtml">

<p>GENE_ASSOCIATION: (TTC0884 or TTC0885 or TTC0886 or TTC0919)</p>

<p>SUBSYSTEM: Propanoate metabolism</p>
```

```xml
<p>EC Number: 6.2.1.1</p>
<p>Confidence Level: 1</p>
<p>AUTHORS: Lipid metabolism</p>
<p/>
</body>
</notes>
<listOfReactants>
<speciesReference species="M_ATP_c" stoichiometry="1"/>
<speciesReference species="M_PROP_c" stoichiometry="1"/>
</listOfReactants>
<listOfProducts>
<speciesReference species="M_PPi_c" stoichiometry="1"/>
<speciesReference species="M_PROP_ADNSPi_c" stoichiometry="1"/>
</listOfProducts>
<kineticLaw>
<math xmlns="http://www.w3.org/1998/Math/MathML">
<ci> FLUX_VALUE </ci>
</math>
<listOfParameters>
<parameter id="LOWER_BOUND" value="-1000" units="mmol_per_gDW_per_hr" constant="true"/>
<parameter id="UPPER_BOUND" value="1000" units="mmol_per_gDW_per_hr" constant="true"/>
<parameter id="FLUX_VALUE" value="0" units="mmol_per_gDW_per_hr" constant="true"/>
<parameter id="OBJECTIVE_COEFFICIENT" value="0" units="mmol_per_gDW_per_hr" constant="true"/>
</listOfParameters>
</kineticLaw>
</reaction>
<reaction id="R_R433" name="pyruvate:pyruvate acetaldehydetransferase (decarboxylating)" reversible="false">
<notes>
<body xmlns="http://www.w3.org/1999/xhtml">
```

```
      <p>GENE_ASSOCIATION: (TTC0851 and TTC0852)</p>
      <p>SUBSYSTEM: Valine, Leucine, and Isoleucine Metabolism</p>
      <p>EC Number: 2.2.1.6</p>
      <p>Confidence Level: 1</p>
      <p>AUTHORS: Amino acid metabolism</p>
      <p/>
    </body>
  </notes>
  <listOfReactants>
    <speciesReference species="M_H_c" stoichiometry="1"/>
    <speciesReference species="M_PYR_c" stoichiometry="2"/>
  </listOfReactants>
  <listOfProducts>
    <speciesReference species="M_CO2_c" stoichiometry="1"/>
    <speciesReference species="M_S_ACLAC_c" stoichiometry="1"/>
  </listOfProducts>
  <kineticLaw>
    <math xmlns="http://www.w3.org/1998/Math/MathML">
      <ci> FLUX_VALUE </ci>
    </math>
    <listOfParameters>
      <parameter id="LOWER_BOUND" value="0" units="mmol_per_gDW_per_hr" constant="true"/>
      <parameter id="UPPER_BOUND" value="1000" units="mmol_per_gDW_per_hr" constant="true"/>
      <parameter id="FLUX_VALUE" value="0" units="mmol_per_gDW_per_hr" constant="true"/>
      <parameter id="OBJECTIVE_COEFFICIENT" value="0" units="mmol_per_gDW_per_hr" constant="true"/>
    </listOfParameters>
  </kineticLaw>
</reaction>
<reaction id="R_R434" name="glycine synthase" reversible="false">
```

```xml
<notes>
  <body xmlns="http://www.w3.org/1999/xhtml">
    <p>GENE_ASSOCIATION: (TTC0150 and TTC0151)</p>
    <p>SUBSYSTEM: Folate Metabolism</p>
    <p>EC Number: 1.4.4.2</p>
    <p>Confidence Level: 1</p>
    <p>AUTHORS: Energy and cofactors metabolism</p>
    <p/>
  </body>
</notes>
<listOfReactants>
  <speciesReference species="M_L_GLY_c" stoichiometry="1"/>
  <speciesReference species="M_NAD_c" stoichiometry="1"/>
  <speciesReference species="M_THF_c" stoichiometry="1"/>
</listOfReactants>
<listOfProducts>
  <speciesReference species="M_H_c" stoichiometry="1"/>
  <speciesReference species="M_NADH_c" stoichiometry="1"/>
  <speciesReference species="M_CO2_c" stoichiometry="1"/>
  <speciesReference species="M_NH4_c" stoichiometry="1"/>
  <speciesReference species="M_5_10_MNTHF_c" stoichiometry="1"/>
</listOfProducts>
<kineticLaw>
  <math xmlns="http://www.w3.org/1998/Math/MathML">
    <ci> FLUX_VALUE </ci>
  </math>
  <listOfParameters>
    <parameter id="LOWER_BOUND" value="0" units="mmol_per_gDW_per_hr" constant="true"/>
    <parameter id="UPPER_BOUND" value="1000" units="mmol_per_gDW_per_hr" constant="true"/>
```

```xml
<parameter id="FLUX_VALUE" value="0" units="mmol_per_gDW_per_hr" constant="true"/>

<parameter id="OBJECTIVE_COEFFICIENT" value="0" units="mmol_per_gDW_per_hr" constant="true"/>

</listOfParameters>

</kineticLaw>

</reaction>

<reaction id="R_R435" name="adenosine nucleotides de novo biosynthesis " reversible="false">

<notes>

<body xmlns="http://www.w3.org/1999/xhtml">

<p>GENE_ASSOCIATION: </p>

<p>SUBSYSTEM: Folate Metabolism</p>

<p>EC Number: </p>

<p>Confidence Level: 1</p>

<p>AUTHORS: Energy and cofactors metabolism</p>

<p/>

</body>

</notes>

<listOfReactants>

<speciesReference species="M_ATP_c" stoichiometry="1"/>

<speciesReference species="M_FMN_c" stoichiometry="1"/>

</listOfReactants>

<listOfProducts>

<speciesReference species="M_dATP_c" stoichiometry="1"/>

<speciesReference species="M_H2O_c" stoichiometry="1"/>

<speciesReference species="M_FMNH2_c" stoichiometry="1"/>

</listOfProducts>

<kineticLaw>

<math xmlns="http://www.w3.org/1998/Math/MathML">

<ci> FLUX_VALUE </ci>

</math>
```

```
    <listOfParameters>
      <parameter id="LOWER_BOUND" value="0" units="mmol_per_gDW_per_hr" constant="true"/>
      <parameter id="UPPER_BOUND" value="1000" units="mmol_per_gDW_per_hr" constant="true"/>
      <parameter id="FLUX_VALUE" value="0" units="mmol_per_gDW_per_hr" constant="true"/>
      <parameter id="OBJECTIVE_COEFFICIENT" value="0" units="mmol_per_gDW_per_hr" constant="true"/>
    </listOfParameters>
  </kineticLaw>
</reaction>
<reaction id="R_R436" name="5-Formyltetrahydrofolate cyclo-ligase (ADP-forming)" reversible="true">
  <notes>
    <body xmlns="http://www.w3.org/1999/xhtml">
      <p>GENE_ASSOCIATION: (TTC1247 or TTC1803)</p>
      <p>SUBSYSTEM: Folate Metabolism</p>
      <p>EC Number: 6.3.3.2</p>
      <p>Confidence Level: 1</p>
      <p>AUTHORS: Energy and cofactors metabolism</p>
      <p/>
    </body>
  </notes>
  <listOfReactants>
    <speciesReference species="M_ATP_c" stoichiometry="1"/>
    <speciesReference species="M_FOLNA_c" stoichiometry="1"/>
  </listOfReactants>
  <listOfProducts>
    <speciesReference species="M_ADP_c" stoichiometry="1"/>
    <speciesReference species="M_Pi_c" stoichiometry="1"/>
    <speciesReference species="M_METHF_c" stoichiometry="1"/>
  </listOfProducts>
  <kineticLaw>
```

```
    <math xmlns="http://www.w3.org/1998/Math/MathML">
      <ci> FLUX_VALUE </ci>
    </math>
    <listOfParameters>
      <parameter id="LOWER_BOUND" value="-1000" units="mmol_per_gDW_per_hr" constant="true"/>
      <parameter id="UPPER_BOUND" value="1000" units="mmol_per_gDW_per_hr" constant="true"/>
      <parameter id="FLUX_VALUE" value="0" units="mmol_per_gDW_per_hr" constant="true"/>
      <parameter id="OBJECTIVE_COEFFICIENT" value="0" units="mmol_per_gDW_per_hr" constant="true"/>
    </listOfParameters>
  </kineticLaw>
</reaction>
<reaction id="R_R437" name="5-methyltetrahydrofolate:NADP+ oxidoreductase" reversible="false">
  <notes>
    <body xmlns="http://www.w3.org/1999/xhtml">
      <p>GENE_ASSOCIATION: TTC1656</p>
      <p>SUBSYSTEM: Folate Metabolism</p>
      <p>EC Number: 1.5.1.20</p>
      <p>Confidence Level: 1</p>
      <p>AUTHORS: Energy and cofactors metabolism</p>
      <p/>
    </body>
  </notes>
  <listOfReactants>
    <speciesReference species="M_H_c" stoichiometry="2"/>
    <speciesReference species="M_NADPH_c" stoichiometry="1"/>
    <speciesReference species="M_5_10_MNTHF_c" stoichiometry="1"/>
  </listOfReactants>
  <listOfProducts>
    <speciesReference species="M_NADP_c" stoichiometry="1"/>
```

```
      <speciesReference species="M_5MTHF_c" stoichiometry="1"/>
    </listOfProducts>
    <kineticLaw>
      <math xmlns="http://www.w3.org/1998/Math/MathML">
        <ci> FLUX_VALUE </ci>
      </math>
      <listOfParameters>
        <parameter id="LOWER_BOUND" value="0" units="mmol_per_gDW_per_hr" constant="true"/>
        <parameter id="UPPER_BOUND" value="1000" units="mmol_per_gDW_per_hr" constant="true"/>
        <parameter id="FLUX_VALUE" value="0" units="mmol_per_gDW_per_hr" constant="true"/>
        <parameter id="OBJECTIVE_COEFFICIENT" value="0" units="mmol_per_gDW_per_hr" constant="true"/>
      </listOfParameters>
    </kineticLaw>
  </reaction>
  <reaction id="R_R438" name="5-methyltetrahydrofolate:NAD+ oxidoreductase" reversible="false">
    <notes>
      <body xmlns="http://www.w3.org/1999/xhtml">
        <p>GENE_ASSOCIATION: TTC1656</p>
        <p>SUBSYSTEM: Folate Metabolism</p>
        <p>EC Number: 1.5.1.20</p>
        <p>Confidence Level: 1</p>
        <p>AUTHORS: Energy and cofactors metabolism</p>
        <p/>
      </body>
    </notes>
    <listOfReactants>
      <speciesReference species="M_H_c" stoichiometry="2"/>
      <speciesReference species="M_NADH_c" stoichiometry="1"/>
      <speciesReference species="M_5_10_MNTHF_c" stoichiometry="1"/>
```

```xml
  </listOfReactants>
  <listOfProducts>
    <speciesReference species="M_NAD_c" stoichiometry="1"/>
    <speciesReference species="M_5MTHF_c" stoichiometry="1"/>
  </listOfProducts>
  <kineticLaw>
    <math xmlns="http://www.w3.org/1998/Math/MathML">
      <ci> FLUX_VALUE </ci>
    </math>
    <listOfParameters>
      <parameter id="LOWER_BOUND" value="0" units="mmol_per_gDW_per_hr" constant="true"/>
      <parameter id="UPPER_BOUND" value="1000" units="mmol_per_gDW_per_hr" constant="true"/>
      <parameter id="FLUX_VALUE" value="0" units="mmol_per_gDW_per_hr" constant="true"/>
      <parameter id="OBJECTIVE_COEFFICIENT" value="0" units="mmol_per_gDW_per_hr" constant="true"/>
    </listOfParameters>
  </kineticLaw>
</reaction>
<reaction id="R_R439" name="L-cysteine:[ThiI] sulfurtransferase" reversible="false">
  <notes>
    <body xmlns="http://www.w3.org/1999/xhtml">
      <p>GENE_ASSOCIATION: (TTC0087 or TTC1373)</p>
      <p>SUBSYSTEM: Cofactor and Prosthetic Group Biosynthesis</p>
      <p>EC Number: 2.8.1.7</p>
      <p>Confidence Level: 1</p>
      <p>AUTHORS: Energy and cofactors metabolism</p>
      <p/>
    </body>
  </notes>
  <listOfReactants>
```

```xml
      <speciesReference species="M_L_CYS_c" stoichiometry="1"/>
      <speciesReference species="M_ENZ_CYS_c" stoichiometry="1"/>
    </listOfReactants>
    <listOfProducts>
      <speciesReference species="M_L_ALA_c" stoichiometry="1"/>
      <speciesReference species="M_ENZ_SLUFA_CYS_c" stoichiometry="1"/>
    </listOfProducts>
    <kineticLaw>
      <math xmlns="http://www.w3.org/1998/Math/MathML">
        <ci> FLUX_VALUE </ci>
      </math>
      <listOfParameters>
        <parameter id="LOWER_BOUND" value="0" units="mmol_per_gDW_per_hr" constant="true"/>
        <parameter id="UPPER_BOUND" value="1000" units="mmol_per_gDW_per_hr" constant="true"/>
        <parameter id="FLUX_VALUE" value="0" units="mmol_per_gDW_per_hr" constant="true"/>
        <parameter id="OBJECTIVE_COEFFICIENT" value="0" units="mmol_per_gDW_per_hr" constant="true"/>
      </listOfParameters>
    </kineticLaw>
  </reaction>
  <reaction id="R_R440" name="ATP:4-amino-2-methyl-5-phosphomethylpyrimidine phosphotransferase" reversible="false">
    <notes>
      <body xmlns="http://www.w3.org/1999/xhtml">
        <p>GENE_ASSOCIATION: TTC0321</p>
        <p>SUBSYSTEM: Cofactor and Prosthetic Group Biosynthesis</p>
        <p>EC Number: 2.7.4.7</p>
        <p>Confidence Level: 1</p>
        <p>AUTHORS: Energy and cofactors metabolism</p>
        <p/>
      </body>
```

```
        </notes>
        <listOfReactants>
          <speciesReference species="M_ATP_c" stoichiometry="1"/>
          <speciesReference species="M_4A_M5PM_PYRM_c" stoichiometry="1"/>
        </listOfReactants>
        <listOfProducts>
          <speciesReference species="M_ADP_c" stoichiometry="1"/>
          <speciesReference species="M_2M4A_HYMPYRMPPi_c" stoichiometry="1"/>
        </listOfProducts>
        <kineticLaw>
          <math xmlns="http://www.w3.org/1998/Math/MathML">
            <ci> FLUX_VALUE </ci>
          </math>
          <listOfParameters>
            <parameter id="LOWER_BOUND" value="0" units="mmol_per_gDW_per_hr" constant="true"/>
            <parameter id="UPPER_BOUND" value="1000" units="mmol_per_gDW_per_hr" constant="true"/>
            <parameter id="FLUX_VALUE" value="0" units="mmol_per_gDW_per_hr" constant="true"/>
            <parameter id="OBJECTIVE_COEFFICIENT" value="0" units="mmol_per_gDW_per_hr" constant="true"/>
          </listOfParameters>
        </kineticLaw>
      </reaction>
      <reaction id="R_R441" name="ATP:4-amino-5-hydroxymethyl-2-methylpyrimidine 5-phosphotransferase"
reversible="false">
        <notes>
          <body xmlns="http://www.w3.org/1999/xhtml">
            <p>GENE_ASSOCIATION: TTC0321</p>
            <p>SUBSYSTEM: Cofactor and Prosthetic Group Biosynthesis</p>
            <p>EC Number: 2.7.1.49</p>
            <p>Confidence Level: 1</p>
```

```xml
<p>AUTHORS: Energy and cofactors metabolism</p>

<p/>

</body>

</notes>

<listOfReactants>

<speciesReference species="M_ATP_c" stoichiometry="1"/>

<speciesReference species="M_TOPYRM_c" stoichiometry="1"/>

</listOfReactants>

<listOfProducts>

<speciesReference species="M_ADP_c" stoichiometry="1"/>

<speciesReference species="M_H_c" stoichiometry="1"/>

<speciesReference species="M_4A_M5PM_PYRM_c" stoichiometry="1"/>

</listOfProducts>

<kineticLaw>

<math xmlns="http://www.w3.org/1998/Math/MathML">

<ci> FLUX_VALUE </ci>

</math>

<listOfParameters>

<parameter id="LOWER_BOUND" value="0" units="mmol_per_gDW_per_hr" constant="true"/>

<parameter id="UPPER_BOUND" value="1000" units="mmol_per_gDW_per_hr" constant="true"/>

<parameter id="FLUX_VALUE" value="0" units="mmol_per_gDW_per_hr" constant="true"/>

<parameter id="OBJECTIVE_COEFFICIENT" value="0" units="mmol_per_gDW_per_hr" constant="true"/>

</listOfParameters>

</kineticLaw>

</reaction>

<reaction id="R_R442" name="2-methyl-4-amino-5-hydroxymethylpyrimidine-diphosphate:4-methyl-5-(2-phosphoethyl)-thiazole 2-methyl-4-aminopyridine-5-methenyltransferase" reversible="false">

<notes>

<body xmlns="http://www.w3.org/1999/xhtml">
```

```
      <p>GENE_ASSOCIATION: TTC0315</p>
      <p>SUBSYSTEM: Cofactor and Prosthetic Group Biosynthesis</p>
      <p>EC Number: 2.5.1.3</p>
      <p>Confidence Level: 1</p>
      <p>AUTHORS: Energy and cofactors metabolism</p>
      <p/>
    </body>
  </notes>
  <listOfReactants>
    <speciesReference species="M_H_c" stoichiometry="1"/>
    <speciesReference species="M_2M4A_HYMPYRMPPi_c" stoichiometry="1"/>
    <speciesReference species="M_4M_PE_THZ_c" stoichiometry="1"/>
  </listOfReactants>
  <listOfProducts>
    <speciesReference species="M_PPi_c" stoichiometry="1"/>
    <speciesReference species="M_TMP_c" stoichiometry="1"/>
  </listOfProducts>
  <kineticLaw>
    <math xmlns="http://www.w3.org/1998/Math/MathML">
      <ci> FLUX_VALUE </ci>
    </math>
    <listOfParameters>
      <parameter id="LOWER_BOUND" value="0" units="mmol_per_gDW_per_hr" constant="true"/>
      <parameter id="UPPER_BOUND" value="1000" units="mmol_per_gDW_per_hr" constant="true"/>
      <parameter id="FLUX_VALUE" value="0" units="mmol_per_gDW_per_hr" constant="true"/>
      <parameter id="OBJECTIVE_COEFFICIENT" value="0" units="mmol_per_gDW_per_hr" constant="true"/>
    </listOfParameters>
  </kineticLaw>
</reaction>
```

```
<reaction id="R_R443" name="ATP:thiamin-phosphate phosphotransferase" reversible="false">
 <notes>
  <body xmlns="http://www.w3.org/1999/xhtml">
   <p>GENE_ASSOCIATION: TTC0056</p>
   <p>SUBSYSTEM: Cofactor and Prosthetic Group Biosynthesis</p>
   <p>EC Number: 2.7.4.16</p>
   <p>Confidence Level: 1</p>
   <p>AUTHORS: Energy and cofactors metabolism</p>
   <p/>
  </body>
 </notes>
 <listOfReactants>
  <speciesReference species="M_ATP_c" stoichiometry="1"/>
  <speciesReference species="M_TMP_c" stoichiometry="1"/>
 </listOfReactants>
 <listOfProducts>
  <speciesReference species="M_ADP_c" stoichiometry="1"/>
  <speciesReference species="M_TPP_c" stoichiometry="1"/>
 </listOfProducts>
 <kineticLaw>
  <math xmlns="http://www.w3.org/1998/Math/MathML">
   <ci> FLUX_VALUE </ci>
  </math>
  <listOfParameters>
   <parameter id="LOWER_BOUND" value="0" units="mmol_per_gDW_per_hr" constant="true"/>
   <parameter id="UPPER_BOUND" value="1000" units="mmol_per_gDW_per_hr" constant="true"/>
   <parameter id="FLUX_VALUE" value="0" units="mmol_per_gDW_per_hr" constant="true"/>
   <parameter id="OBJECTIVE_COEFFICIENT" value="0" units="mmol_per_gDW_per_hr" constant="true"/>
  </listOfParameters>
```

```
  </kineticLaw>
</reaction>
<reaction id="R_R444" name="Thiamin diphosphate phosphohydrolase" reversible="false">
  <notes>
    <body xmlns="http://www.w3.org/1999/xhtml">
      <p>GENE_ASSOCIATION: </p>
      <p>SUBSYSTEM: Cofactor and Prosthetic Group Biosynthesis</p>
      <p>EC Number: 3.6.1.6</p>
      <p>Confidence Level: 1</p>
      <p>AUTHORS: Energy and cofactors metabolism</p>
      <p/>
    </body>
  </notes>
  <listOfReactants>
    <speciesReference species="M_H2O_c" stoichiometry="1"/>
    <speciesReference species="M_TPP_c" stoichiometry="1"/>
  </listOfReactants>
  <listOfProducts>
    <speciesReference species="M_Pi_c" stoichiometry="1"/>
    <speciesReference species="M_H_c" stoichiometry="1"/>
    <speciesReference species="M_TMP_c" stoichiometry="1"/>
  </listOfProducts>
  <kineticLaw>
    <math xmlns="http://www.w3.org/1998/Math/MathML">
      <ci> FLUX_VALUE </ci>
    </math>
    <listOfParameters>
      <parameter id="LOWER_BOUND" value="0" units="mmol_per_gDW_per_hr" constant="true"/>
      <parameter id="UPPER_BOUND" value="1000" units="mmol_per_gDW_per_hr" constant="true"/>
```

```
      <parameter id="FLUX_VALUE" value="0" units="mmol_per_gDW_per_hr" constant="true"/>
      <parameter id="OBJECTIVE_COEFFICIENT" value="0" units="mmol_per_gDW_per_hr" constant="true"/>
    </listOfParameters>
  </kineticLaw>
</reaction>
<reaction id="R_R445" name="Glycine oxidase" reversible="false">
  <notes>
    <body xmlns="http://www.w3.org/1999/xhtml">
      <p>GENE_ASSOCIATION: TTC0318</p>
      <p>SUBSYSTEM: Cofactor and Prosthetic Group Biosynthesis</p>
      <p>EC Number: </p>
      <p>Confidence Level: 1</p>
      <p>AUTHORS: Energy and cofactors metabolism</p>
      <p/>
    </body>
  </notes>
  <listOfReactants>
    <speciesReference species="M_L_GLY_c" stoichiometry="1"/>
    <speciesReference species="M_O2_c" stoichiometry="1"/>
  </listOfReactants>
  <listOfProducts>
    <speciesReference species="M_H_c" stoichiometry="1"/>
    <speciesReference species="M_H2O2_c" stoichiometry="1"/>
    <speciesReference species="M_IMAC_c" stoichiometry="1"/>
  </listOfProducts>
  <kineticLaw>
    <math xmlns="http://www.w3.org/1998/Math/MathML">
      <ci> FLUX_VALUE </ci>
    </math>
```

```
    <listOfParameters>
      <parameter id="LOWER_BOUND" value="0" units="mmol_per_gDW_per_hr" constant="true"/>
      <parameter id="UPPER_BOUND" value="1000" units="mmol_per_gDW_per_hr" constant="true"/>
      <parameter id="FLUX_VALUE" value="0" units="mmol_per_gDW_per_hr" constant="true"/>
      <parameter id="OBJECTIVE_COEFFICIENT" value="0" units="mmol_per_gDW_per_hr" constant="true"/>
    </listOfParameters>
  </kineticLaw>
</reaction>
<reaction id="R_R446" name="Thiazole synthase" reversible="false">
  <notes>
    <body xmlns="http://www.w3.org/1999/xhtml">
      <p>GENE_ASSOCIATION: TTC0317</p>
      <p>SUBSYSTEM: Cofactor and Prosthetic Group Biosynthesis</p>
      <p>EC Number: 2.8.1.10</p>
      <p>Confidence Level: 1</p>
      <p>AUTHORS: Energy and cofactors metabolism</p>
      <p/>
    </body>
  </notes>
  <listOfReactants>
    <speciesReference species="M_L_CYS_c" stoichiometry="1"/>
    <speciesReference species="M_ATP_c" stoichiometry="1"/>
    <speciesReference species="M_IMAC_c" stoichiometry="1"/>
    <speciesReference species="M_dXYLL5P_c" stoichiometry="1"/>
  </listOfReactants>
  <listOfProducts>
    <speciesReference species="M_L_ALA_c" stoichiometry="1"/>
    <speciesReference species="M_PPi_c" stoichiometry="1"/>
    <speciesReference species="M_H2O_c" stoichiometry="2"/>
```

```
      <speciesReference species="M_AMP_c" stoichiometry="1"/>
      <speciesReference species="M_THZ_c" stoichiometry="1"/>
    </listOfProducts>
    <kineticLaw>
      <math xmlns="http://www.w3.org/1998/Math/MathML">
        <ci> FLUX_VALUE </ci>
      </math>
      <listOfParameters>
        <parameter id="LOWER_BOUND" value="0" units="mmol_per_gDW_per_hr" constant="true"/>
        <parameter id="UPPER_BOUND" value="1000" units="mmol_per_gDW_per_hr" constant="true"/>
        <parameter id="FLUX_VALUE" value="0" units="mmol_per_gDW_per_hr" constant="true"/>
        <parameter id="OBJECTIVE_COEFFICIENT" value="0" units="mmol_per_gDW_per_hr" constant="true"/>
      </listOfParameters>
    </kineticLaw>
  </reaction>
  <reaction id="R_R447" name="Thiazole isomerase" reversible="false">
    <notes>
      <body xmlns="http://www.w3.org/1999/xhtml">
        <p>GENE_ASSOCIATION: </p>
        <p>SUBSYSTEM: Cofactor and Prosthetic Group Biosynthesis</p>
        <p>EC Number: 5.3.99.10</p>
        <p>Confidence Level: 1</p>
        <p>AUTHORS: Energy and cofactors metabolism</p>
        <p/>
      </body>
    </notes>
    <listOfReactants>
      <speciesReference species="M_THZ_c" stoichiometry="1"/>
    </listOfReactants>
```

```
<listOfProducts>
  <speciesReference species="M_4M_PE_THZ_c" stoichiometry="1"/>
</listOfProducts>
<kineticLaw>
  <math xmlns="http://www.w3.org/1998/Math/MathML">
    <ci> FLUX_VALUE </ci>
  </math>
  <listOfParameters>
    <parameter id="LOWER_BOUND" value="0" units="mmol_per_gDW_per_hr" constant="true"/>
    <parameter id="UPPER_BOUND" value="1000" units="mmol_per_gDW_per_hr" constant="true"/>
    <parameter id="FLUX_VALUE" value="0" units="mmol_per_gDW_per_hr" constant="true"/>
    <parameter id="OBJECTIVE_COEFFICIENT" value="0" units="mmol_per_gDW_per_hr" constant="true"/>
  </listOfParameters>
</kineticLaw>
</reaction>
<reaction id="R_R448" name="D-ribulose 5-phosphate formate-lyase (L-3,4-dihydroxybutan-2-one 4-phosphate-forming)"
reversible="false">
<notes>
  <body xmlns="http://www.w3.org/1999/xhtml">
    <p>GENE_ASSOCIATION: TTC0697</p>
    <p>SUBSYSTEM: Cofactor and Prosthetic Group Biosynthesis</p>
    <p>EC Number: 4.1.99.12</p>
    <p>Confidence Level: 1</p>
    <p>AUTHORS: Energy and cofactors metabolism</p>
    <p/>
  </body>
</notes>
<listOfReactants>
  <speciesReference species="M_RBL5P_c" stoichiometry="1"/>
```

```
</listOfReactants>
<listOfProducts>
 <speciesReference species="M_H_c" stoichiometry="1"/>
 <speciesReference species="M_FOR_c" stoichiometry="1"/>
 <speciesReference species="M_DHY_BUTNPi_c" stoichiometry="1"/>
</listOfProducts>
<kineticLaw>
 <math xmlns="http://www.w3.org/1998/Math/MathML">
  <ci> FLUX_VALUE </ci>
 </math>
 <listOfParameters>
  <parameter id="LOWER_BOUND" value="0" units="mmol_per_gDW_per_hr" constant="true"/>
  <parameter id="UPPER_BOUND" value="1000" units="mmol_per_gDW_per_hr" constant="true"/>
  <parameter id="FLUX_VALUE" value="0" units="mmol_per_gDW_per_hr" constant="true"/>
  <parameter id="OBJECTIVE_COEFFICIENT" value="0" units="mmol_per_gDW_per_hr" constant="true"/>
 </listOfParameters>
</kineticLaw>
</reaction>
<reaction id="R_R449" name="GTP 7,8-8,9-dihydrolase (diphosphate-forming)" reversible="false">
<notes>
 <body xmlns="http://www.w3.org/1999/xhtml">
  <p>GENE_ASSOCIATION: TTC0697</p>
  <p>SUBSYSTEM: Cofactor and Prosthetic Group Biosynthesis</p>
  <p>EC Number: 3.5.4.25</p>
  <p>Confidence Level: 1</p>
  <p>AUTHORS: Energy and cofactors metabolism</p>
  <p/>
 </body>
</notes>
```

```
        <listOfReactants>
          <speciesReference species="M_GTP_c" stoichiometry="1"/>
          <speciesReference species="M_H2O_c" stoichiometry="3"/>
        </listOfReactants>
        <listOfProducts>
          <speciesReference species="M_PPi_c" stoichiometry="1"/>
          <speciesReference species="M_H_c" stoichiometry="2"/>
          <speciesReference species="M_FOR_c" stoichiometry="1"/>
          <speciesReference species="M_DA_PRIBSA_PYMDN_c" stoichiometry="1"/>
        </listOfProducts>
        <kineticLaw>
          <math xmlns="http://www.w3.org/1998/Math/MathML">
            <ci> FLUX_VALUE </ci>
          </math>
          <listOfParameters>
            <parameter id="LOWER_BOUND" value="0" units="mmol_per_gDW_per_hr" constant="true"/>
            <parameter id="UPPER_BOUND" value="1000" units="mmol_per_gDW_per_hr" constant="true"/>
            <parameter id="FLUX_VALUE" value="0" units="mmol_per_gDW_per_hr" constant="true"/>
            <parameter id="OBJECTIVE_COEFFICIENT" value="0" units="mmol_per_gDW_per_hr" constant="true"/>
          </listOfParameters>
        </kineticLaw>
      </reaction>
      <reaction id="R_R450" name="5-amino-6-(5-phosphoribitylamino)uracil:NADP+ 1&apos;-oxidoreductase"
reversible="false">
        <notes>
          <body xmlns="http://www.w3.org/1999/xhtml">
            <p>GENE_ASSOCIATION: TTC0699</p>
            <p>SUBSYSTEM: Cofactor and Prosthetic Group Biosynthesis</p>
            <p>EC Number: 1.1.1.193</p>
```

```xml
<p>Confidence Level: 1</p>
<p>AUTHORS: Energy and cofactors metabolism</p>
<p/>
</body>
</notes>
<listOfReactants>
<speciesReference species="M_H_c" stoichiometry="1"/>
<speciesReference species="M_NADPH_c" stoichiometry="1"/>
<speciesReference species="M_AP_RIB_URA_c" stoichiometry="1"/>
</listOfReactants>
<listOfProducts>
<speciesReference species="M_NADP_c" stoichiometry="1"/>
<speciesReference species="M_APRIBTA_URA_c" stoichiometry="1"/>
</listOfProducts>
<kineticLaw>
<math xmlns="http://www.w3.org/1998/Math/MathML">
<ci> FLUX_VALUE </ci>
</math>
<listOfParameters>
<parameter id="LOWER_BOUND" value="0" units="mmol_per_gDW_per_hr" constant="true"/>
<parameter id="UPPER_BOUND" value="1000" units="mmol_per_gDW_per_hr" constant="true"/>
<parameter id="FLUX_VALUE" value="0" units="mmol_per_gDW_per_hr" constant="true"/>
<parameter id="OBJECTIVE_COEFFICIENT" value="0" units="mmol_per_gDW_per_hr" constant="true"/>
</listOfParameters>
</kineticLaw>
</reaction>
<reaction id="R_R451" name="ATP:riboflavin 5&apos;-phosphotransferase" reversible="false">
<notes>
<body xmlns="http://www.w3.org/1999/xhtml">
```

```
      <p>GENE_ASSOCIATION: TTC0159</p>
      <p>SUBSYSTEM: Cofactor and Prosthetic Group Biosynthesis</p>
      <p>EC Number: 2.7.1.26</p>
      <p>Confidence Level: 1</p>
      <p>AUTHORS: Energy and cofactors metabolism</p>
      <p/>
    </body>
  </notes>
  <listOfReactants>
    <speciesReference species="M_ATP_c" stoichiometry="1"/>
    <speciesReference species="M_RBFLV_c" stoichiometry="1"/>
  </listOfReactants>
  <listOfProducts>
    <speciesReference species="M_ADP_c" stoichiometry="1"/>
    <speciesReference species="M_H_c" stoichiometry="1"/>
    <speciesReference species="M_FMN_c" stoichiometry="1"/>
  </listOfProducts>
  <kineticLaw>
    <math xmlns="http://www.w3.org/1998/Math/MathML">
      <ci> FLUX_VALUE </ci>
    </math>
    <listOfParameters>
      <parameter id="LOWER_BOUND" value="0" units="mmol_per_gDW_per_hr" constant="true"/>
      <parameter id="UPPER_BOUND" value="1000" units="mmol_per_gDW_per_hr" constant="true"/>
      <parameter id="FLUX_VALUE" value="0" units="mmol_per_gDW_per_hr" constant="true"/>
      <parameter id="OBJECTIVE_COEFFICIENT" value="0" units="mmol_per_gDW_per_hr" constant="true"/>
    </listOfParameters>
  </kineticLaw>
</reaction>
```

```xml
<reaction id="R_R452" name="ATP:FMN adenylyltransferase" reversible="false">
 <notes>
  <body xmlns="http://www.w3.org/1999/xhtml">
   <p>GENE_ASSOCIATION: TTC0159</p>
   <p>SUBSYSTEM: Cofactor and Prosthetic Group Biosynthesis</p>
   <p>EC Number: 2.7.7.2</p>
   <p>Confidence Level: 1</p>
   <p>AUTHORS: Energy and cofactors metabolism</p>
   <p/>
  </body>
 </notes>
 <listOfReactants>
  <speciesReference species="M_ATP_c" stoichiometry="1"/>
  <speciesReference species="M_H_c" stoichiometry="1"/>
  <speciesReference species="M_FMN_c" stoichiometry="1"/>
 </listOfReactants>
 <listOfProducts>
  <speciesReference species="M_PPi_c" stoichiometry="1"/>
  <speciesReference species="M_FAD_c" stoichiometry="1"/>
 </listOfProducts>
 <kineticLaw>
  <math xmlns="http://www.w3.org/1998/Math/MathML">
   <ci> FLUX_VALUE </ci>
  </math>
  <listOfParameters>
   <parameter id="LOWER_BOUND" value="0" units="mmol_per_gDW_per_hr" constant="true"/>
   <parameter id="UPPER_BOUND" value="1000" units="mmol_per_gDW_per_hr" constant="true"/>
   <parameter id="FLUX_VALUE" value="0" units="mmol_per_gDW_per_hr" constant="true"/>
   <parameter id="OBJECTIVE_COEFFICIENT" value="0" units="mmol_per_gDW_per_hr" constant="true"/>
```

```xml
    </listOfParameters>
  </kineticLaw>
</reaction>
<reaction id="R_R453" name="riboflavin-5-phosphate phosphohydrolase (acid optimum)" reversible="false">
  <notes>
    <body xmlns="http://www.w3.org/1999/xhtml">
      <p>GENE_ASSOCIATION: TTC1252</p>
      <p>SUBSYSTEM: Cofactor and Prosthetic Group Biosynthesis</p>
      <p>EC Number: 3.1.3.2</p>
      <p>Confidence Level: 1</p>
      <p>AUTHORS: Energy and cofactors metabolism</p>
      <p/>
    </body>
  </notes>
  <listOfReactants>
    <speciesReference species="M_H2O_c" stoichiometry="1"/>
    <speciesReference species="M_FMN_c" stoichiometry="1"/>
  </listOfReactants>
  <listOfProducts>
    <speciesReference species="M_Pi_c" stoichiometry="1"/>
    <speciesReference species="M_RBFLV_c" stoichiometry="1"/>
  </listOfProducts>
  <kineticLaw>
    <math xmlns="http://www.w3.org/1998/Math/MathML">
      <ci> FLUX_VALUE </ci>
    </math>
    <listOfParameters>
      <parameter id="LOWER_BOUND" value="0" units="mmol_per_gDW_per_hr" constant="true"/>
      <parameter id="UPPER_BOUND" value="1000" units="mmol_per_gDW_per_hr" constant="true"/>
```

```
<parameter id="FLUX_VALUE" value="0" units="mmol_per_gDW_per_hr" constant="true"/>

<parameter id="OBJECTIVE_COEFFICIENT" value="0" units="mmol_per_gDW_per_hr" constant="true"/>

</listOfParameters>

</kineticLaw>

</reaction>

<reaction id="R_R454" name="Nicotinate-nucleotide:dimethylbenzimidazole phospho-D-ribosyltransferase" reversible="false">

<notes>

<body xmlns="http://www.w3.org/1999/xhtml">

<p>GENE_ASSOCIATION: TT_P0005</p>

<p>SUBSYSTEM: Cofactor and Prosthetic Group Biosynthesis</p>

<p>EC Number: 2.4.2.21</p>

<p>Confidence Level: 1</p>

<p>AUTHORS: Energy and cofactors metabolism</p>

<p/>

</body>

</notes>

<listOfReactants>

<speciesReference species="M_NC_D_RNUC_c" stoichiometry="1"/>

<speciesReference species="M_CBMCo_c" stoichiometry="1"/>

</listOfReactants>

<listOfProducts>

<speciesReference species="M_H_c" stoichiometry="1"/>

<speciesReference species="M_NIA_c" stoichiometry="1"/>

<speciesReference species="M_PRIBS_DMBENZ_IMDZ_c" stoichiometry="1"/>

</listOfProducts>

<kineticLaw>

<math xmlns="http://www.w3.org/1998/Math/MathML">

<ci> FLUX_VALUE </ci>
```

```xml
</math>

<listOfParameters>

<parameter id="LOWER_BOUND" value="0" units="mmol_per_gDW_per_hr" constant="true"/>

<parameter id="UPPER_BOUND" value="1000" units="mmol_per_gDW_per_hr" constant="true"/>

<parameter id="FLUX_VALUE" value="0" units="mmol_per_gDW_per_hr" constant="true"/>

<parameter id="OBJECTIVE_COEFFICIENT" value="0" units="mmol_per_gDW_per_hr" constant="true"/>

</listOfParameters>

</kineticLaw>

</reaction>

<reaction id="R_R455" name="2,5-Diamino-6-hydroxy-4-(5-phosphoribosylamino)-pyrimidine 2-aminohydrolase" reversible="false">

<notes>

<body xmlns="http://www.w3.org/1999/xhtml">

<p>GENE_ASSOCIATION: TTC0699</p>

<p>SUBSYSTEM: Cofactor and Prosthetic Group Biosynthesis</p>

<p>EC Number: 3.5.4.26</p>

<p>Confidence Level: 1</p>

<p>AUTHORS: Energy and cofactors metabolism</p>

<p/>

</body>

</notes>

<listOfReactants>

<speciesReference species="M_H2O_c" stoichiometry="1"/>

<speciesReference species="M_H_c" stoichiometry="1"/>

<speciesReference species="M_DA_PRIBSA_PYMDN_c" stoichiometry="1"/>

</listOfReactants>

<listOfProducts>

<speciesReference species="M_NH4_c" stoichiometry="1"/>

<speciesReference species="M_AP_RIB_URA_c" stoichiometry="1"/>
```

```xml
  </listOfProducts>
  <kineticLaw>
    <math xmlns="http://www.w3.org/1998/Math/MathML">
      <ci> FLUX_VALUE </ci>
    </math>
    <listOfParameters>
      <parameter id="LOWER_BOUND" value="0" units="mmol_per_gDW_per_hr" constant="true"/>
      <parameter id="UPPER_BOUND" value="1000" units="mmol_per_gDW_per_hr" constant="true"/>
      <parameter id="FLUX_VALUE" value="0" units="mmol_per_gDW_per_hr" constant="true"/>
      <parameter id="OBJECTIVE_COEFFICIENT" value="0" units="mmol_per_gDW_per_hr" constant="true"/>
    </listOfParameters>
  </kineticLaw>
</reaction>
<reaction id="R_R456" name="5-amino-6-(D-ribitylamino)uracil butanedionetransferase" reversible="false">
  <notes>
    <body xmlns="http://www.w3.org/1999/xhtml">
      <p>GENE_ASSOCIATION: TTC1647</p>
      <p>SUBSYSTEM: Cofactor and Prosthetic Group Biosynthesis</p>
      <p>EC Number: 2.5.1.78</p>
      <p>Confidence Level: 1</p>
      <p>AUTHORS: Energy and cofactors metabolism</p>
      <p/>
    </body>
  </notes>
  <listOfReactants>
    <speciesReference species="M_DHY_BUTNPi_c" stoichiometry="1"/>
    <speciesReference species="M_5AM_RIBAM_URA_c" stoichiometry="1"/>
  </listOfReactants>
  <listOfProducts>
```

```xml
    <speciesReference species="M_Pi_c" stoichiometry="1"/>
    <speciesReference species="M_H2O_c" stoichiometry="2"/>
    <speciesReference species="M_DM_DR_LUM_c" stoichiometry="1"/>
  </listOfProducts>
  <kineticLaw>
    <math xmlns="http://www.w3.org/1998/Math/MathML">
      <ci> FLUX_VALUE </ci>
    </math>
    <listOfParameters>
      <parameter id="LOWER_BOUND" value="0" units="mmol_per_gDW_per_hr" constant="true"/>
      <parameter id="UPPER_BOUND" value="1000" units="mmol_per_gDW_per_hr" constant="true"/>
      <parameter id="FLUX_VALUE" value="0" units="mmol_per_gDW_per_hr" constant="true"/>
      <parameter id="OBJECTIVE_COEFFICIENT" value="0" units="mmol_per_gDW_per_hr" constant="true"/>
    </listOfParameters>
  </kineticLaw>
</reaction>
<reaction id="R_R457" name="O-Phospho-4-hydroxy-L-threonine phospho-lyase (adding water)" reversible="false">
  <notes>
    <body xmlns="http://www.w3.org/1999/xhtml">
      <p>GENE_ASSOCIATION: TTC0117</p>
      <p>SUBSYSTEM: Cofactor and Prosthetic Group Biosynthesis</p>
      <p>EC Number: 4.2.3.1</p>
      <p>Confidence Level: 1</p>
      <p>AUTHORS: Energy and cofactors metabolism</p>
      <p/>
    </body>
  </notes>
  <listOfReactants>
    <speciesReference species="M_H2O_c" stoichiometry="1"/>
```

```
  <speciesReference species="M_OPHY_THR_c" stoichiometry="1"/>
 </listOfReactants>
 <listOfProducts>
  <speciesReference species="M_Pi_c" stoichiometry="1"/>
  <speciesReference species="M_HY_THR_c" stoichiometry="1"/>
 </listOfProducts>
 <kineticLaw>
  <math xmlns="http://www.w3.org/1998/Math/MathML">
   <ci> FLUX_VALUE </ci>
  </math>
  <listOfParameters>
   <parameter id="LOWER_BOUND" value="0" units="mmol_per_gDW_per_hr" constant="true"/>
   <parameter id="UPPER_BOUND" value="1000" units="mmol_per_gDW_per_hr" constant="true"/>
   <parameter id="FLUX_VALUE" value="0" units="mmol_per_gDW_per_hr" constant="true"/>
   <parameter id="OBJECTIVE_COEFFICIENT" value="0" units="mmol_per_gDW_per_hr" constant="true"/>
  </listOfParameters>
 </kineticLaw>
</reaction>
<reaction id="R_R458" name="Nicotinate D-ribonucleotide:diphosphate phosphoribosyltransferase" reversible="false">
 <notes>
  <body xmlns="http://www.w3.org/1999/xhtml">
   <p>GENE_ASSOCIATION: TTC0252</p>
   <p>SUBSYSTEM: Cofactor and Prosthetic Group Biosynthesis</p>
   <p>EC Number: 2.4.2.11</p>
   <p>Confidence Level: 1</p>
   <p>AUTHORS: Energy and cofactors metabolism</p>
   <p/>
  </body>
 </notes>
```

```xml
  <listOfReactants>
    <speciesReference species="M_H_c" stoichiometry="1"/>
    <speciesReference species="M_PRPP_c" stoichiometry="1"/>
    <speciesReference species="M_NIA_c" stoichiometry="1"/>
  </listOfReactants>
  <listOfProducts>
    <speciesReference species="M_PPi_c" stoichiometry="1"/>
    <speciesReference species="M_NC_D_RNUC_c" stoichiometry="1"/>
  </listOfProducts>
  <kineticLaw>
    <math xmlns="http://www.w3.org/1998/Math/MathML">
      <ci> FLUX_VALUE </ci>
    </math>
    <listOfParameters>
      <parameter id="LOWER_BOUND" value="0" units="mmol_per_gDW_per_hr" constant="true"/>
      <parameter id="UPPER_BOUND" value="1000" units="mmol_per_gDW_per_hr" constant="true"/>
      <parameter id="FLUX_VALUE" value="0" units="mmol_per_gDW_per_hr" constant="true"/>
      <parameter id="OBJECTIVE_COEFFICIENT" value="0" units="mmol_per_gDW_per_hr" constant="true"/>
    </listOfParameters>
  </kineticLaw>
</reaction>
<reaction id="R_R459" name="N-Ribosylnicotinamide:orthophosphate ribosyltransferase" reversible="true">
  <notes>
    <body xmlns="http://www.w3.org/1999/xhtml">
      <p>GENE_ASSOCIATION: (TTC0194 or TTC1070)</p>
      <p>SUBSYSTEM: Cofactor and Prosthetic Group Biosynthesis</p>
      <p>EC Number: 2.4.2.1</p>
      <p>Confidence Level: 1</p>
      <p>AUTHORS: Energy and cofactors metabolism</p>
```

```
      <p/>
    </body>
  </notes>
  <listOfReactants>
    <speciesReference species="M_Pi_c" stoichiometry="1"/>
    <speciesReference species="M_RIBS_NCTA_c" stoichiometry="1"/>
  </listOfReactants>
  <listOfProducts>
    <speciesReference species="M_R1P_c" stoichiometry="1"/>
    <speciesReference species="M_NAMD_c" stoichiometry="1"/>
  </listOfProducts>
  <kineticLaw>
    <math xmlns="http://www.w3.org/1998/Math/MathML">
      <ci> FLUX_VALUE </ci>
    </math>
    <listOfParameters>
      <parameter id="LOWER_BOUND" value="-1000" units="mmol_per_gDW_per_hr" constant="true"/>
      <parameter id="UPPER_BOUND" value="1000" units="mmol_per_gDW_per_hr" constant="true"/>
      <parameter id="FLUX_VALUE" value="0" units="mmol_per_gDW_per_hr" constant="true"/>
      <parameter id="OBJECTIVE_COEFFICIENT" value="0" units="mmol_per_gDW_per_hr" constant="true"/>
    </listOfParameters>
  </kineticLaw>
</reaction>
<reaction id="R_R460" name="Nicotinate D-ribonucleoside:orthophosphate ribosyltransferase" reversible="true">
  <notes>
    <body xmlns="http://www.w3.org/1999/xhtml">
      <p>GENE_ASSOCIATION: (TTC0194 or TTC1070)</p>
      <p>SUBSYSTEM: Cofactor and Prosthetic Group Biosynthesis</p>
      <p>EC Number: 2.4.2.1</p>
```

```xml
<p>Confidence Level: 1</p>
<p>AUTHORS: Energy and cofactors metabolism</p>
<p/>
</body>
</notes>
<listOfReactants>
<speciesReference species="M_Pi_c" stoichiometry="1"/>
<speciesReference species="M_NICRIB_c" stoichiometry="1"/>
</listOfReactants>
<listOfProducts>
<speciesReference species="M_H_c" stoichiometry="1"/>
<speciesReference species="M_R1P_c" stoichiometry="1"/>
<speciesReference species="M_NIA_c" stoichiometry="1"/>
</listOfProducts>
<kineticLaw>
<math xmlns="http://www.w3.org/1998/Math/MathML">
<ci> FLUX_VALUE </ci>
</math>
<listOfParameters>
<parameter id="LOWER_BOUND" value="-1000" units="mmol_per_gDW_per_hr" constant="true"/>
<parameter id="UPPER_BOUND" value="1000" units="mmol_per_gDW_per_hr" constant="true"/>
<parameter id="FLUX_VALUE" value="0" units="mmol_per_gDW_per_hr" constant="true"/>
<parameter id="OBJECTIVE_COEFFICIENT" value="0" units="mmol_per_gDW_per_hr" constant="true"/>
</listOfParameters>
</kineticLaw>
</reaction>
<reaction id="R_R461" name="Nicotinamide ribonucleotide phosphohydrolase" reversible="false">
<notes>
<body xmlns="http://www.w3.org/1999/xhtml">
```

```xml
<p>GENE_ASSOCIATION: (TTC0964 or TTC1625 or TT_P0028)</p>
<p>SUBSYSTEM: Cofactor and Prosthetic Group Biosynthesis</p>
<p>EC Number: 3.1.3.5</p>
<p>Confidence Level: 1</p>
<p>AUTHORS: Energy and cofactors metabolism</p>
<p/>
</body>
</notes>
<listOfReactants>
<speciesReference species="M_H2O_c" stoichiometry="1"/>
<speciesReference species="M_NMN_c" stoichiometry="1"/>
</listOfReactants>
<listOfProducts>
<speciesReference species="M_Pi_c" stoichiometry="1"/>
<speciesReference species="M_RIBS_NCTA_c" stoichiometry="1"/>
</listOfProducts>
<kineticLaw>
<math xmlns="http://www.w3.org/1998/Math/MathML">
<ci> FLUX_VALUE </ci>
</math>
<listOfParameters>
<parameter id="LOWER_BOUND" value="0" units="mmol_per_gDW_per_hr" constant="true"/>
<parameter id="UPPER_BOUND" value="1000" units="mmol_per_gDW_per_hr" constant="true"/>
<parameter id="FLUX_VALUE" value="0" units="mmol_per_gDW_per_hr" constant="true"/>
<parameter id="OBJECTIVE_COEFFICIENT" value="0" units="mmol_per_gDW_per_hr" constant="true"/>
</listOfParameters>
</kineticLaw>
</reaction>
```

```xml
<reaction id="R_R462" name="Nicotinate-nucleotide:pyrophosphate phosphoribosyltransferase (carboxylating)" reversible="false">
  <notes>
    <body xmlns="http://www.w3.org/1999/xhtml">
      <p>GENE_ASSOCIATION: TTC0621</p>
      <p>SUBSYSTEM: Cofactor and Prosthetic Group Biosynthesis</p>
      <p>EC Number: 2.4.2.19</p>
      <p>Confidence Level: 1</p>
      <p>AUTHORS: Energy and cofactors metabolism</p>
      <p/>
    </body>
  </notes>
  <listOfReactants>
    <speciesReference species="M_H_c" stoichiometry="2"/>
    <speciesReference species="M_PRPP_c" stoichiometry="1"/>
    <speciesReference species="M_QA_c" stoichiometry="1"/>
  </listOfReactants>
  <listOfProducts>
    <speciesReference species="M_PPi_c" stoichiometry="1"/>
    <speciesReference species="M_CO2_c" stoichiometry="1"/>
    <speciesReference species="M_NC_D_RNUC_c" stoichiometry="1"/>
  </listOfProducts>
  <kineticLaw>
    <math xmlns="http://www.w3.org/1998/Math/MathML">
      <ci> FLUX_VALUE </ci>
    </math>
    <listOfParameters>
      <parameter id="LOWER_BOUND" value="0" units="mmol_per_gDW_per_hr" constant="true"/>
      <parameter id="UPPER_BOUND" value="1000" units="mmol_per_gDW_per_hr" constant="true"/>
```

```
      <parameter id="FLUX_VALUE" value="0" units="mmol_per_gDW_per_hr" constant="true"/>
      <parameter id="OBJECTIVE_COEFFICIENT" value="0" units="mmol_per_gDW_per_hr" constant="true"/>
    </listOfParameters>
  </kineticLaw>
</reaction>
<reaction id="R_R463" name="ATP:nicotinamide-nucleotide adenylyltransferase" reversible="false">
  <notes>
    <body xmlns="http://www.w3.org/1999/xhtml">
      <p>GENE_ASSOCIATION: TTC1421</p>
      <p>SUBSYSTEM: Cofactor and Prosthetic Group Biosynthesis</p>
      <p>EC Number: 2.7.7.18</p>
      <p>Confidence Level: 1</p>
      <p>AUTHORS: Energy and cofactors metabolism</p>
      <p/>
    </body>
  </notes>
  <listOfReactants>
    <speciesReference species="M_ATP_c" stoichiometry="1"/>
    <speciesReference species="M_H_c" stoichiometry="1"/>
    <speciesReference species="M_NMN_c" stoichiometry="1"/>
  </listOfReactants>
  <listOfProducts>
    <speciesReference species="M_PPi_c" stoichiometry="1"/>
    <speciesReference species="M_NAD_c" stoichiometry="1"/>
  </listOfProducts>
  <kineticLaw>
    <math xmlns="http://www.w3.org/1998/Math/MathML">
      <ci> FLUX_VALUE </ci>
    </math>
```

```
      <listOfParameters>
        <parameter id="LOWER_BOUND" value="0" units="mmol_per_gDW_per_hr" constant="true"/>
        <parameter id="UPPER_BOUND" value="1000" units="mmol_per_gDW_per_hr" constant="true"/>
        <parameter id="FLUX_VALUE" value="0" units="mmol_per_gDW_per_hr" constant="true"/>
        <parameter id="OBJECTIVE_COEFFICIENT" value="0" units="mmol_per_gDW_per_hr" constant="true"/>
      </listOfParameters>
    </kineticLaw>
  </reaction>
  <reaction id="R_R464" name="deamido-NAD+:ammonia ligase (AMP-forming)" reversible="false">
    <notes>
      <body xmlns="http://www.w3.org/1999/xhtml">
        <p>GENE_ASSOCIATION: TTC1538</p>
        <p>SUBSYSTEM: Cofactor and Prosthetic Group Biosynthesis</p>
        <p>EC Number: 6.3.1.5</p>
        <p>Confidence Level: 1</p>
        <p>AUTHORS: Energy and cofactors metabolism</p>
        <p/>
      </body>
    </notes>
    <listOfReactants>
      <speciesReference species="M_ATP_c" stoichiometry="1"/>
      <speciesReference species="M_NH4_c" stoichiometry="1"/>
      <speciesReference species="M_DA_NAD_c" stoichiometry="1"/>
    </listOfReactants>
    <listOfProducts>
      <speciesReference species="M_PPi_c" stoichiometry="1"/>
      <speciesReference species="M_H_c" stoichiometry="1"/>
      <speciesReference species="M_NAD_c" stoichiometry="1"/>
      <speciesReference species="M_AMP_c" stoichiometry="1"/>
```

```
  </listOfProducts>
  <kineticLaw>
    <math xmlns="http://www.w3.org/1998/Math/MathML">
      <ci> FLUX_VALUE </ci>
    </math>
    <listOfParameters>
      <parameter id="LOWER_BOUND" value="0" units="mmol_per_gDW_per_hr" constant="true"/>
      <parameter id="UPPER_BOUND" value="1000" units="mmol_per_gDW_per_hr" constant="true"/>
      <parameter id="FLUX_VALUE" value="0" units="mmol_per_gDW_per_hr" constant="true"/>
      <parameter id="OBJECTIVE_COEFFICIENT" value="0" units="mmol_per_gDW_per_hr" constant="true"/>
    </listOfParameters>
  </kineticLaw>
</reaction>
<reaction id="R_R465" name="NADPH:NAD+ oxidoreductase" reversible="false">
  <notes>
    <body xmlns="http://www.w3.org/1999/xhtml">
      <p>GENE_ASSOCIATION: (TTC1778 and (TTC1779 or TTC1780))</p>
      <p>SUBSYSTEM: Oxidative Phosphorylation</p>
      <p>EC Number: 1.6.1.1 and 1.6.1.2</p>
      <p>Confidence Level: 1</p>
      <p>AUTHORS: Energy and cofactors metabolism</p>
      <p/>
    </body>
  </notes>
  <listOfReactants>
    <speciesReference species="M_NAD_c" stoichiometry="1"/>
    <speciesReference species="M_NADPH_c" stoichiometry="1"/>
  </listOfReactants>
  <listOfProducts>
```

```
    <speciesReference species="M_NADH_c" stoichiometry="1"/>
    <speciesReference species="M_NADP_c" stoichiometry="1"/>
  </listOfProducts>
  <kineticLaw>
    <math xmlns="http://www.w3.org/1998/Math/MathML">
      <ci> FLUX_VALUE </ci>
    </math>
    <listOfParameters>
      <parameter id="LOWER_BOUND" value="0" units="mmol_per_gDW_per_hr" constant="true"/>
      <parameter id="UPPER_BOUND" value="1000" units="mmol_per_gDW_per_hr" constant="true"/>
      <parameter id="FLUX_VALUE" value="0" units="mmol_per_gDW_per_hr" constant="true"/>
      <parameter id="OBJECTIVE_COEFFICIENT" value="0" units="mmol_per_gDW_per_hr" constant="true"/>
    </listOfParameters>
  </kineticLaw>
</reaction>
<reaction id="R_R466" name="L-aspartate:oxygen oxidoreductase" reversible="false">
  <notes>
    <body xmlns="http://www.w3.org/1999/xhtml">
      <p>GENE_ASSOCIATION: TTC0619</p>
      <p>SUBSYSTEM: Cofactor and Prosthetic Group Biosynthesis</p>
      <p>EC Number: 1.4.3.16</p>
      <p>Confidence Level: 1</p>
      <p>AUTHORS: Energy and cofactors metabolism</p>
      <p/>
    </body>
  </notes>
  <listOfReactants>
    <speciesReference species="M_L_ASP_c" stoichiometry="1"/>
    <speciesReference species="M_O2_c" stoichiometry="1"/>
```

```
  </listOfReactants>
  <listOfProducts>
   <speciesReference species="M_H_c" stoichiometry="1"/>
   <speciesReference species="M_H2O2_c" stoichiometry="1"/>
   <speciesReference species="M_IMASP_c" stoichiometry="1"/>
  </listOfProducts>
  <kineticLaw>
   <math xmlns="http://www.w3.org/1998/Math/MathML">
    <ci> FLUX_VALUE </ci>
   </math>
   <listOfParameters>
    <parameter id="LOWER_BOUND" value="0" units="mmol_per_gDW_per_hr" constant="true"/>
    <parameter id="UPPER_BOUND" value="1000" units="mmol_per_gDW_per_hr" constant="true"/>
    <parameter id="FLUX_VALUE" value="0" units="mmol_per_gDW_per_hr" constant="true"/>
    <parameter id="OBJECTIVE_COEFFICIENT" value="0" units="mmol_per_gDW_per_hr" constant="true"/>
   </listOfParameters>
  </kineticLaw>
 </reaction>
 <reaction id="R_R467" name="glycerone phosphate:iminosuccinate alkyltransferase (cyclizing)" reversible="false">
  <notes>
   <body xmlns="http://www.w3.org/1999/xhtml">
    <p>GENE_ASSOCIATION: TTC0620</p>
    <p>SUBSYSTEM: Cofactor and Prosthetic Group Biosynthesis</p>
    <p>EC Number: 2.5.1.72</p>
    <p>Confidence Level: 1</p>
    <p>AUTHORS: Energy and cofactors metabolism</p>
    <p/>
   </body>
  </notes>
```

```
  <listOfReactants>
    <speciesReference species="M_DHAP_c" stoichiometry="1"/>
    <speciesReference species="M_IMASP_c" stoichiometry="1"/>
  </listOfReactants>
  <listOfProducts>
    <speciesReference species="M_Pi_c" stoichiometry="1"/>
    <speciesReference species="M_H2O_c" stoichiometry="2"/>
    <speciesReference species="M_QA_c" stoichiometry="1"/>
  </listOfProducts>
  <kineticLaw>
    <math xmlns="http://www.w3.org/1998/Math/MathML">
      <ci> FLUX_VALUE </ci>
    </math>
    <listOfParameters>
      <parameter id="LOWER_BOUND" value="0" units="mmol_per_gDW_per_hr" constant="true"/>
      <parameter id="UPPER_BOUND" value="1000" units="mmol_per_gDW_per_hr" constant="true"/>
      <parameter id="FLUX_VALUE" value="0" units="mmol_per_gDW_per_hr" constant="true"/>
      <parameter id="OBJECTIVE_COEFFICIENT" value="0" units="mmol_per_gDW_per_hr" constant="true"/>
    </listOfParameters>
  </kineticLaw>
</reaction>
<reaction id="R_R468" name="Nicotinate D-ribonucleotide phosphohydrolase" reversible="false">
  <notes>
    <body xmlns="http://www.w3.org/1999/xhtml">
      <p>GENE_ASSOCIATION: (TTC0964 or TTC1625 or TT_P0028)</p>
      <p>SUBSYSTEM: Cofactor and Prosthetic Group Biosynthesis</p>
      <p>EC Number: 3.1.3.5</p>
      <p>Confidence Level: 1</p>
      <p>AUTHORS: Energy and cofactors metabolism</p>
```

```
      <p/>
    </body>
  </notes>
  <listOfReactants>
    <speciesReference species="M_H2O_c" stoichiometry="1"/>
    <speciesReference species="M_NC_D_RNUC_c" stoichiometry="1"/>
  </listOfReactants>
  <listOfProducts>
    <speciesReference species="M_Pi_c" stoichiometry="1"/>
    <speciesReference species="M_NICRIB_c" stoichiometry="1"/>
  </listOfProducts>
  <kineticLaw>
    <math xmlns="http://www.w3.org/1998/Math/MathML">
      <ci> FLUX_VALUE </ci>
    </math>
    <listOfParameters>
      <parameter id="LOWER_BOUND" value="0" units="mmol_per_gDW_per_hr" constant="true"/>
      <parameter id="UPPER_BOUND" value="1000" units="mmol_per_gDW_per_hr" constant="true"/>
      <parameter id="FLUX_VALUE" value="0" units="mmol_per_gDW_per_hr" constant="true"/>
      <parameter id="OBJECTIVE_COEFFICIENT" value="0" units="mmol_per_gDW_per_hr" constant="true"/>
    </listOfParameters>
  </kineticLaw>
</reaction>
<reaction id="R_R469" name="ATP:nicotinamide-nucleotide adenylyltransferase" reversible="true">
  <notes>
    <body xmlns="http://www.w3.org/1999/xhtml">
      <p>GENE_ASSOCIATION: TTC1421</p>
      <p>SUBSYSTEM: Cofactor and Prosthetic Group Biosynthesis</p>
      <p>EC Number: 2.7.7.18</p>
```

```
      <p>Confidence Level: 1</p>
      <p>AUTHORS: Energy and cofactors metabolism</p>
      <p/>
    </body>
  </notes>
  <listOfReactants>
    <speciesReference species="M_ATP_c" stoichiometry="1"/>
    <speciesReference species="M_H_c" stoichiometry="1"/>
    <speciesReference species="M_NC_D_RNUC_c" stoichiometry="1"/>
  </listOfReactants>
  <listOfProducts>
    <speciesReference species="M_PPi_c" stoichiometry="1"/>
    <speciesReference species="M_DA_NAD_c" stoichiometry="1"/>
  </listOfProducts>
  <kineticLaw>
    <math xmlns="http://www.w3.org/1998/Math/MathML">
      <ci> FLUX_VALUE </ci>
    </math>
    <listOfParameters>
      <parameter id="LOWER_BOUND" value="-1000" units="mmol_per_gDW_per_hr" constant="true"/>
      <parameter id="UPPER_BOUND" value="1000" units="mmol_per_gDW_per_hr" constant="true"/>
      <parameter id="FLUX_VALUE" value="0" units="mmol_per_gDW_per_hr" constant="true"/>
      <parameter id="OBJECTIVE_COEFFICIENT" value="0" units="mmol_per_gDW_per_hr" constant="true"/>
    </listOfParameters>
  </kineticLaw>
</reaction>
<reaction id="R_R470" name="6-Carboxyhyxanoate:CoA ligase (AMP-forming)" reversible="true">
  <notes>
    <body xmlns="http://www.w3.org/1999/xhtml">
```

```xml
    <p>GENE_ASSOCIATION: TTC1711</p>
    <p>SUBSYSTEM: Cofactor and Prosthetic Group Biosynthesis</p>
    <p>EC Number: 6.2.1.14</p>
    <p>Confidence Level: 1</p>
    <p>AUTHORS: Energy and cofactors metabolism</p>
    <p/>
  </body>
</notes>
<listOfReactants>
  <speciesReference species="M_ATP_c" stoichiometry="1"/>
  <speciesReference species="M_CoA_c" stoichiometry="1"/>
  <speciesReference species="M_PIM_c" stoichiometry="1"/>
</listOfReactants>
<listOfProducts>
  <speciesReference species="M_PPi_c" stoichiometry="1"/>
  <speciesReference species="M_AMP_c" stoichiometry="1"/>
  <speciesReference species="M_PIMCoA_c" stoichiometry="1"/>
</listOfProducts>
<kineticLaw>
  <math xmlns="http://www.w3.org/1998/Math/MathML">
    <ci> FLUX_VALUE </ci>
  </math>
  <listOfParameters>
    <parameter id="LOWER_BOUND" value="-1000" units="mmol_per_gDW_per_hr" constant="true"/>
    <parameter id="UPPER_BOUND" value="1000" units="mmol_per_gDW_per_hr" constant="true"/>
    <parameter id="FLUX_VALUE" value="0" units="mmol_per_gDW_per_hr" constant="true"/>
    <parameter id="OBJECTIVE_COEFFICIENT" value="0" units="mmol_per_gDW_per_hr" constant="true"/>
  </listOfParameters>
</kineticLaw>
```

```
</reaction>
<reaction id="R_R471" name="dethiobiotin:sulfur sulfurtransferase" reversible="false">
 <notes>
  <body xmlns="http://www.w3.org/1999/xhtml">
   <p>GENE_ASSOCIATION: TTC0242</p>
   <p>SUBSYSTEM: Cofactor and Prosthetic Group Biosynthesis</p>
   <p>EC Number: 2.8.1.6</p>
   <p>Confidence Level: 1</p>
   <p>AUTHORS: Energy and cofactors metabolism</p>
   <p/>
  </body>
 </notes>
 <listOfReactants>
  <speciesReference species="M_SAM_c" stoichiometry="2"/>
  <speciesReference species="M_DTBTN_c" stoichiometry="1"/>
  <speciesReference species="M_S_c" stoichiometry="1"/>
 </listOfReactants>
 <listOfProducts>
  <speciesReference species="M_L_MET_c" stoichiometry="2"/>
  <speciesReference species="M_H_c" stoichiometry="1"/>
  <speciesReference species="M_BTN_c" stoichiometry="1"/>
  <speciesReference species="M_dADNS_c" stoichiometry="2"/>
 </listOfProducts>
 <kineticLaw>
  <math xmlns="http://www.w3.org/1998/Math/MathML">
   <ci> FLUX_VALUE </ci>
  </math>
  <listOfParameters>
   <parameter id="LOWER_BOUND" value="0" units="mmol_per_gDW_per_hr" constant="true"/>
```

```
      <parameter id="UPPER_BOUND" value="1000" units="mmol_per_gDW_per_hr" constant="true"/>
      <parameter id="FLUX_VALUE" value="0" units="mmol_per_gDW_per_hr" constant="true"/>
      <parameter id="OBJECTIVE_COEFFICIENT" value="0" units="mmol_per_gDW_per_hr" constant="true"/>
    </listOfParameters>
  </kineticLaw>
</reaction>
<reaction id="R_R472" name="biotin:CoA ligase (AMP-forming)" reversible="false">
  <notes>
    <body xmlns="http://www.w3.org/1999/xhtml">
      <p>GENE_ASSOCIATION: TTC1761</p>
      <p>SUBSYSTEM: Cofactor and Prosthetic Group Biosynthesis</p>
      <p>EC Number: 6.3.4.15</p>
      <p>Confidence Level: 1</p>
      <p>AUTHORS: Energy and cofactors metabolism</p>
      <p/>
    </body>
  </notes>
  <listOfReactants>
    <speciesReference species="M_ATP_c" stoichiometry="1"/>
    <speciesReference species="M_BTN_c" stoichiometry="1"/>
  </listOfReactants>
  <listOfProducts>
    <speciesReference species="M_PPi_c" stoichiometry="1"/>
    <speciesReference species="M_BTN_AMP_c" stoichiometry="1"/>
  </listOfProducts>
  <kineticLaw>
    <math xmlns="http://www.w3.org/1998/Math/MathML">
      <ci> FLUX_VALUE </ci>
    </math>
```

```
<listOfParameters>
  <parameter id="LOWER_BOUND" value="0" units="mmol_per_gDW_per_hr" constant="true"/>
  <parameter id="UPPER_BOUND" value="1000" units="mmol_per_gDW_per_hr" constant="true"/>
  <parameter id="FLUX_VALUE" value="0" units="mmol_per_gDW_per_hr" constant="true"/>
  <parameter id="OBJECTIVE_COEFFICIENT" value="0" units="mmol_per_gDW_per_hr" constant="true"/>
</listOfParameters>
</kineticLaw>
</reaction>
<reaction id="R_R473" name="Biotinyl-5&apos;-AMP:apo-[carboxylase] ligase (AMP-forming)" reversible="false">
<notes>
  <body xmlns="http://www.w3.org/1999/xhtml">
    <p>GENE_ASSOCIATION: TTC1761</p>
    <p>SUBSYSTEM: Cofactor and Prosthetic Group Biosynthesis</p>
    <p>EC Number: 6.3.4.15</p>
    <p>Confidence Level: 1</p>
    <p>AUTHORS: Energy and cofactors metabolism</p>
    <p/>
  </body>
</notes>
<listOfReactants>
  <speciesReference species="M_BTN_AMP_c" stoichiometry="1"/>
  <speciesReference species="M_APCARB_c" stoichiometry="1"/>
</listOfReactants>
<listOfProducts>
  <speciesReference species="M_AMP_c" stoichiometry="1"/>
  <speciesReference species="M_HCARB_c" stoichiometry="1"/>
</listOfProducts>
<kineticLaw>
  <math xmlns="http://www.w3.org/1998/Math/MathML">
```

```
      <ci> FLUX_VALUE </ci>
    </math>
    <listOfParameters>
      <parameter id="LOWER_BOUND" value="0" units="mmol_per_gDW_per_hr" constant="true"/>
      <parameter id="UPPER_BOUND" value="1000" units="mmol_per_gDW_per_hr" constant="true"/>
      <parameter id="FLUX_VALUE" value="0" units="mmol_per_gDW_per_hr" constant="true"/>
      <parameter id="OBJECTIVE_COEFFICIENT" value="0" units="mmol_per_gDW_per_hr" constant="true"/>
    </listOfParameters>
  </kineticLaw>
</reaction>
<reaction id="R_R474" name="octanoyl-[acp]:sulfur sulfurtransferase" reversible="false">
  <notes>
    <body xmlns="http://www.w3.org/1999/xhtml">
      <p>GENE_ASSOCIATION: TTC1747</p>
      <p>SUBSYSTEM: Lipoic acid metabolism </p>
      <p>EC Number: 2.8.1.8</p>
      <p>Confidence Level: 1</p>
      <p>AUTHORS: Lipid metabolism</p>
      <p/>
    </body>
  </notes>
  <listOfReactants>
    <speciesReference species="M_SAM_c" stoichiometry="2"/>
    <speciesReference species="M_S_c" stoichiometry="2"/>
    <speciesReference species="M_OCT_ACP_c" stoichiometry="1"/>
  </listOfReactants>
  <listOfProducts>
    <speciesReference species="M_L_MET_c" stoichiometry="2"/>
    <speciesReference species="M_dADNS_c" stoichiometry="2"/>
```

```
    <speciesReference species="M_LP_ACP_c" stoichiometry="1"/>
   </listOfProducts>
   <kineticLaw>
    <math xmlns="http://www.w3.org/1998/Math/MathML">
     <ci> FLUX_VALUE </ci>
    </math>
    <listOfParameters>
     <parameter id="LOWER_BOUND" value="0" units="mmol_per_gDW_per_hr" constant="true"/>
     <parameter id="UPPER_BOUND" value="1000" units="mmol_per_gDW_per_hr" constant="true"/>
     <parameter id="FLUX_VALUE" value="0" units="mmol_per_gDW_per_hr" constant="true"/>
     <parameter id="OBJECTIVE_COEFFICIENT" value="0" units="mmol_per_gDW_per_hr" constant="true"/>
    </listOfParameters>
   </kineticLaw>
  </reaction>
  <reaction id="R_R475" name="protein N6-(octanoyl)lysine:sulfur sulfurtransferase" reversible="false">
   <notes>
    <body xmlns="http://www.w3.org/1999/xhtml">
     <p>GENE_ASSOCIATION: TTC1747</p>
     <p>SUBSYSTEM: Lipoic acid metabolism </p>
     <p>EC Number: 2.8.1.8</p>
     <p>Confidence Level: 1</p>
     <p>AUTHORS: Lipid metabolism</p>
     <p/>
    </body>
   </notes>
   <listOfReactants>
    <speciesReference species="M_SAM_c" stoichiometry="2"/>
    <speciesReference species="M_S_c" stoichiometry="2"/>
    <speciesReference species="M_PN6_OCTLYS_c" stoichiometry="1"/>
```

```
  </listOfReactants>
  <listOfProducts>
    <speciesReference species="M_L_MET_c" stoichiometry="2"/>
    <speciesReference species="M_dADNS_c" stoichiometry="2"/>
    <speciesReference species="M_PN6_LPLYS_c" stoichiometry="1"/>
  </listOfProducts>
  <kineticLaw>
    <math xmlns="http://www.w3.org/1998/Math/MathML">
      <ci> FLUX_VALUE </ci>
    </math>
    <listOfParameters>
      <parameter id="LOWER_BOUND" value="0" units="mmol_per_gDW_per_hr" constant="true"/>
      <parameter id="UPPER_BOUND" value="1000" units="mmol_per_gDW_per_hr" constant="true"/>
      <parameter id="FLUX_VALUE" value="0" units="mmol_per_gDW_per_hr" constant="true"/>
      <parameter id="OBJECTIVE_COEFFICIENT" value="0" units="mmol_per_gDW_per_hr" constant="true"/>
    </listOfParameters>
  </kineticLaw>
</reaction>
<reaction id="R_R476" name="octanoyl-[acp]:protein N6-octanoyltransferase" reversible="false">
  <notes>
    <body xmlns="http://www.w3.org/1999/xhtml">
      <p>GENE_ASSOCIATION: TTC1746</p>
      <p>SUBSYSTEM: Lipoic acid metabolism </p>
      <p>EC Number: 2.3.1.181</p>
      <p>Confidence Level: 1</p>
      <p>AUTHORS: Lipid metabolism</p>
      <p/>
    </body>
  </notes>
```

```
 <listOfReactants>
  <speciesReference species="M_OCT_ACP_c" stoichiometry="1"/>
  <speciesReference species="M_APPRO_c" stoichiometry="1"/>
 </listOfReactants>
 <listOfProducts>
  <speciesReference species="M_PN6_OCTLYS_c" stoichiometry="1"/>
  <speciesReference species="M_ACP_c" stoichiometry="1"/>
 </listOfProducts>
 <kineticLaw>
  <math xmlns="http://www.w3.org/1998/Math/MathML">
   <ci> FLUX_VALUE </ci>
  </math>
  <listOfParameters>
   <parameter id="LOWER_BOUND" value="0" units="mmol_per_gDW_per_hr" constant="true"/>
   <parameter id="UPPER_BOUND" value="1000" units="mmol_per_gDW_per_hr" constant="true"/>
   <parameter id="FLUX_VALUE" value="0" units="mmol_per_gDW_per_hr" constant="true"/>
   <parameter id="OBJECTIVE_COEFFICIENT" value="0" units="mmol_per_gDW_per_hr" constant="true"/>
  </listOfParameters>
 </kineticLaw>
</reaction>
<reaction id="R_R477" name="lipoyl-[acp]:protein N6-lipoyltransferase" reversible="false">
 <notes>
  <body xmlns="http://www.w3.org/1999/xhtml">
   <p>GENE_ASSOCIATION: TTC1746</p>
   <p>SUBSYSTEM: Lipoic acid metabolism </p>
   <p>EC Number: 2.3.1.181</p>
   <p>Confidence Level: 1</p>
   <p>AUTHORS: Lipid metabolism</p>
   <p/>
```

```xml
</body>
</notes>
<listOfReactants>
<speciesReference species="M_LP_ACP_c" stoichiometry="1"/>
<speciesReference species="M_APPRO_c" stoichiometry="1"/>
</listOfReactants>
<listOfProducts>
<speciesReference species="M_PN6_LPLYS_c" stoichiometry="1"/>
<speciesReference species="M_ACP_c" stoichiometry="1"/>
</listOfProducts>
<kineticLaw>
<math xmlns="http://www.w3.org/1998/Math/MathML">
<ci> FLUX_VALUE </ci>
</math>
<listOfParameters>
<parameter id="LOWER_BOUND" value="0" units="mmol_per_gDW_per_hr" constant="true"/>
<parameter id="UPPER_BOUND" value="1000" units="mmol_per_gDW_per_hr" constant="true"/>
<parameter id="FLUX_VALUE" value="0" units="mmol_per_gDW_per_hr" constant="true"/>
<parameter id="OBJECTIVE_COEFFICIENT" value="0" units="mmol_per_gDW_per_hr" constant="true"/>
</listOfParameters>
</kineticLaw>
</reaction>
<reaction id="R_R478" name="2,3-Dihydroxy-3-methylbutanoate:NADP+ oxidoreductase (isomerizing)" reversible="true">
<notes>
<body xmlns="http://www.w3.org/1999/xhtml">
<p>GENE_ASSOCIATION: TTC0850</p>
<p>SUBSYSTEM: Cofactor and Prosthetic Group Biosynthesis</p>
<p>EC Number: 1.1.1.86</p>
<p>Confidence Level: 1</p>
```

```xml
      <p>AUTHORS: Energy and cofactors metabolism</p>
      <p/>
    </body>
  </notes>
  <listOfReactants>
    <speciesReference species="M_H_c" stoichiometry="1"/>
    <speciesReference species="M_NADPH_c" stoichiometry="1"/>
    <speciesReference species="M_S_ACLAC_c" stoichiometry="1"/>
  </listOfReactants>
  <listOfProducts>
    <speciesReference species="M_NADP_c" stoichiometry="1"/>
    <speciesReference species="M_DHY_MBUTNA_c" stoichiometry="1"/>
  </listOfProducts>
  <kineticLaw>
    <math xmlns="http://www.w3.org/1998/Math/MathML">
      <ci> FLUX_VALUE </ci>
    </math>
    <listOfParameters>
      <parameter id="LOWER_BOUND" value="-1000" units="mmol_per_gDW_per_hr" constant="true"/>
      <parameter id="UPPER_BOUND" value="1000" units="mmol_per_gDW_per_hr" constant="true"/>
      <parameter id="FLUX_VALUE" value="0" units="mmol_per_gDW_per_hr" constant="true"/>
      <parameter id="OBJECTIVE_COEFFICIENT" value="0" units="mmol_per_gDW_per_hr" constant="true"/>
    </listOfParameters>
  </kineticLaw>
</reaction>
<reaction id="R_R479" name="2,3-Dihydroxy-3-methylbutanoate hydro-lyase" reversible="false">
  <notes>
    <body xmlns="http://www.w3.org/1999/xhtml">
      <p>GENE_ASSOCIATION: TTC0871</p>
```

```
          <p>SUBSYSTEM: Cofactor and Prosthetic Group Biosynthesis</p>
          <p>EC Number: 4.2.1.9</p>
          <p>Confidence Level: 1</p>
          <p>AUTHORS: Energy and cofactors metabolism</p>
          <p/>
        </body>
      </notes>
      <listOfReactants>
        <speciesReference species="M_DHY_MBUTNA_c" stoichiometry="1"/>
      </listOfReactants>
      <listOfProducts>
        <speciesReference species="M_H2O_c" stoichiometry="1"/>
        <speciesReference species="M_2K3M_BUT_c" stoichiometry="1"/>
      </listOfProducts>
      <kineticLaw>
        <math xmlns="http://www.w3.org/1998/Math/MathML">
          <ci> FLUX_VALUE </ci>
        </math>
        <listOfParameters>
          <parameter id="LOWER_BOUND" value="0" units="mmol_per_gDW_per_hr" constant="true"/>
          <parameter id="UPPER_BOUND" value="1000" units="mmol_per_gDW_per_hr" constant="true"/>
          <parameter id="FLUX_VALUE" value="0" units="mmol_per_gDW_per_hr" constant="true"/>
          <parameter id="OBJECTIVE_COEFFICIENT" value="0" units="mmol_per_gDW_per_hr" constant="true"/>
        </listOfParameters>
      </kineticLaw>
    </reaction>
    <reaction id="R_R480" name="5,10-Methylenetetrahydrofolate:3-methyl-2-oxobutanoate hydroxymethyltransferase"
reversible="false">
      <notes>
```

```
  <body xmlns="http://www.w3.org/1999/xhtml">
    <p>GENE_ASSOCIATION: TTC0039</p>
    <p>SUBSYSTEM: Cofactor and Prosthetic Group Biosynthesis</p>
    <p>EC Number: 2.1.2.11</p>
    <p>Confidence Level: 1</p>
    <p>AUTHORS: Energy and cofactors metabolism</p>
    <p/>
  </body>
</notes>
<listOfReactants>
  <speciesReference species="M_H2O_c" stoichiometry="1"/>
  <speciesReference species="M_5_10_MNTHF_c" stoichiometry="1"/>
  <speciesReference species="M_2K3M_BUT_c" stoichiometry="1"/>
</listOfReactants>
<listOfProducts>
  <speciesReference species="M_THF_c" stoichiometry="1"/>
  <speciesReference species="M_DHY_PAN_c" stoichiometry="1"/>
</listOfProducts>
<kineticLaw>
  <math xmlns="http://www.w3.org/1998/Math/MathML">
    <ci> FLUX_VALUE </ci>
  </math>
  <listOfParameters>
    <parameter id="LOWER_BOUND" value="0" units="mmol_per_gDW_per_hr" constant="true"/>
    <parameter id="UPPER_BOUND" value="1000" units="mmol_per_gDW_per_hr" constant="true"/>
    <parameter id="FLUX_VALUE" value="0" units="mmol_per_gDW_per_hr" constant="true"/>
    <parameter id="OBJECTIVE_COEFFICIENT" value="0" units="mmol_per_gDW_per_hr" constant="true"/>
  </listOfParameters>
</kineticLaw>
```

```
</reaction>
<reaction id="R_R481" name="ATP:pantothenate 4&apos;-phosphotransferase" reversible="false">
  <notes>
    <body xmlns="http://www.w3.org/1999/xhtml">
      <p>GENE_ASSOCIATION: TTC1008</p>
      <p>SUBSYSTEM: Cofactor and Prosthetic Group Biosynthesis</p>
      <p>EC Number: 2.7.1.33</p>
      <p>Confidence Level: 1</p>
      <p>AUTHORS: Energy and cofactors metabolism</p>
      <p/>
    </body>
  </notes>
  <listOfReactants>
    <speciesReference species="M_ATP_c" stoichiometry="1"/>
    <speciesReference species="M_PANTO_c" stoichiometry="1"/>
  </listOfReactants>
  <listOfProducts>
    <speciesReference species="M_ADP_c" stoichiometry="1"/>
    <speciesReference species="M_H_c" stoichiometry="1"/>
    <speciesReference species="M_PPANT_c" stoichiometry="1"/>
  </listOfProducts>
  <kineticLaw>
    <math xmlns="http://www.w3.org/1998/Math/MathML">
      <ci> FLUX_VALUE </ci>
    </math>
    <listOfParameters>
      <parameter id="LOWER_BOUND" value="0" units="mmol_per_gDW_per_hr" constant="true"/>
      <parameter id="UPPER_BOUND" value="1000" units="mmol_per_gDW_per_hr" constant="true"/>
      <parameter id="FLUX_VALUE" value="0" units="mmol_per_gDW_per_hr" constant="true"/>
```

```
<parameter id="OBJECTIVE_COEFFICIENT" value="0" units="mmol_per_gDW_per_hr" constant="true"/>
</listOfParameters>
</kineticLaw>
</reaction>
<reaction id="R_R482" name="(R)-4&apos;-Phosphopantothenate:L-cysteine ligase" reversible="false">
<notes>
<body xmlns="http://www.w3.org/1999/xhtml">
<p>GENE_ASSOCIATION: TTC1195</p>
<p>SUBSYSTEM: Cofactor and Prosthetic Group Biosynthesis</p>
<p>EC Number: 6.3.2.5</p>
<p>Confidence Level: 1</p>
<p>AUTHORS: Energy and cofactors metabolism</p>
<p/>
</body>
</notes>
<listOfReactants>
<speciesReference species="M_L_CYS_c" stoichiometry="1"/>
<speciesReference species="M_CTP_c" stoichiometry="1"/>
<speciesReference species="M_PPANT_c" stoichiometry="1"/>
</listOfReactants>
<listOfProducts>
<speciesReference species="M_PPi_c" stoichiometry="1"/>
<speciesReference species="M_H_c" stoichiometry="1"/>
<speciesReference species="M_CMP_c" stoichiometry="1"/>
<speciesReference species="M_PPANTN_CYS_c" stoichiometry="1"/>
</listOfProducts>
<kineticLaw>
<math xmlns="http://www.w3.org/1998/Math/MathML">
<ci> FLUX_VALUE </ci>
```

```
    </math>
    <listOfParameters>
      <parameter id="LOWER_BOUND" value="0" units="mmol_per_gDW_per_hr" constant="true"/>
      <parameter id="UPPER_BOUND" value="1000" units="mmol_per_gDW_per_hr" constant="true"/>
      <parameter id="FLUX_VALUE" value="0" units="mmol_per_gDW_per_hr" constant="true"/>
      <parameter id="OBJECTIVE_COEFFICIENT" value="0" units="mmol_per_gDW_per_hr" constant="true"/>
    </listOfParameters>
  </kineticLaw>
</reaction>
<reaction id="R_R483" name="N-[(R)-4&apos;-Phosphopantothenoyl]-L-cysteine carboxy-lyase" reversible="false">
  <notes>
    <body xmlns="http://www.w3.org/1999/xhtml">
      <p>GENE_ASSOCIATION: TTC1195</p>
      <p>SUBSYSTEM: Cofactor and Prosthetic Group Biosynthesis</p>
      <p>EC Number: 4.1.1.36</p>
      <p>Confidence Level: 1</p>
      <p>AUTHORS: Energy and cofactors metabolism</p>
      <p/>
    </body>
  </notes>
  <listOfReactants>
    <speciesReference species="M_H_c" stoichiometry="1"/>
    <speciesReference species="M_PPANTN_CYS_c" stoichiometry="1"/>
  </listOfReactants>
  <listOfProducts>
    <speciesReference species="M_CO2_c" stoichiometry="1"/>
    <speciesReference species="M_PANTE4Pi_c" stoichiometry="1"/>
  </listOfProducts>
  <kineticLaw>
```

```
      <math xmlns="http://www.w3.org/1998/Math/MathML">
        <ci> FLUX_VALUE </ci>
      </math>
      <listOfParameters>
        <parameter id="LOWER_BOUND" value="0" units="mmol_per_gDW_per_hr" constant="true"/>
        <parameter id="UPPER_BOUND" value="1000" units="mmol_per_gDW_per_hr" constant="true"/>
        <parameter id="FLUX_VALUE" value="0" units="mmol_per_gDW_per_hr" constant="true"/>
        <parameter id="OBJECTIVE_COEFFICIENT" value="0" units="mmol_per_gDW_per_hr" constant="true"/>
      </listOfParameters>
    </kineticLaw>
  </reaction>
  <reaction id="R_R484" name="ATP:pantetheine-4&apos;-phosphate adenylyltransferase" reversible="false">
    <notes>
      <body xmlns="http://www.w3.org/1999/xhtml">
        <p>GENE_ASSOCIATION: TTC0560</p>
        <p>SUBSYSTEM: Cofactor and Prosthetic Group Biosynthesis</p>
        <p>EC Number: 2.7.7.3</p>
        <p>Confidence Level: 1</p>
        <p>AUTHORS: Energy and cofactors metabolism</p>
        <p/>
      </body>
    </notes>
    <listOfReactants>
      <speciesReference species="M_ATP_c" stoichiometry="1"/>
      <speciesReference species="M_H_c" stoichiometry="1"/>
      <speciesReference species="M_PANTE4Pi_c" stoichiometry="1"/>
    </listOfReactants>
    <listOfProducts>
      <speciesReference species="M_PPi_c" stoichiometry="1"/>
```

```xml
<speciesReference species="M_DPCoA_c" stoichiometry="1"/>
</listOfProducts>
<kineticLaw>
<math xmlns="http://www.w3.org/1998/Math/MathML">
<ci> FLUX_VALUE </ci>
</math>
<listOfParameters>
<parameter id="LOWER_BOUND" value="0" units="mmol_per_gDW_per_hr" constant="true"/>
<parameter id="UPPER_BOUND" value="1000" units="mmol_per_gDW_per_hr" constant="true"/>
<parameter id="FLUX_VALUE" value="0" units="mmol_per_gDW_per_hr" constant="true"/>
<parameter id="OBJECTIVE_COEFFICIENT" value="0" units="mmol_per_gDW_per_hr" constant="true"/>
</listOfParameters>
</kineticLaw>
</reaction>
<reaction id="R_R485" name="ATP:dephospho-CoA 3&apos;-phosphotransferase" reversible="false">
<notes>
<body xmlns="http://www.w3.org/1999/xhtml">
<p>GENE_ASSOCIATION: TTC0557</p>
<p>SUBSYSTEM: Cofactor and Prosthetic Group Biosynthesis</p>
<p>EC Number: 2.7.1.24</p>
<p>Confidence Level: 1</p>
<p>AUTHORS: Energy and cofactors metabolism</p>
<p/>
</body>
</notes>
<listOfReactants>
<speciesReference species="M_ATP_c" stoichiometry="1"/>
<speciesReference species="M_DPCoA_c" stoichiometry="1"/>
</listOfReactants>
```

```
<listOfProducts>
  <speciesReference species="M_ADP_c" stoichiometry="1"/>
  <speciesReference species="M_H_c" stoichiometry="1"/>
  <speciesReference species="M_CoA_c" stoichiometry="1"/>
</listOfProducts>
<kineticLaw>
  <math xmlns="http://www.w3.org/1998/Math/MathML">
    <ci> FLUX_VALUE </ci>
  </math>
  <listOfParameters>
    <parameter id="LOWER_BOUND" value="0" units="mmol_per_gDW_per_hr" constant="true"/>
    <parameter id="UPPER_BOUND" value="1000" units="mmol_per_gDW_per_hr" constant="true"/>
    <parameter id="FLUX_VALUE" value="0" units="mmol_per_gDW_per_hr" constant="true"/>
    <parameter id="OBJECTIVE_COEFFICIENT" value="0" units="mmol_per_gDW_per_hr" constant="true"/>
  </listOfParameters>
</kineticLaw>
</reaction>
<reaction id="R_R486" name="GTP 8,9-hydrolase" reversible="false">
<notes>
  <body xmlns="http://www.w3.org/1999/xhtml">
    <p>GENE_ASSOCIATION: TTC1517</p>
    <p>SUBSYSTEM: Cofactor and Prosthetic Group Biosynthesis</p>
    <p>EC Number: 3.5.4.16</p>
    <p>Confidence Level: 1</p>
    <p>AUTHORS: Energy and cofactors metabolism</p>
    <p/>
  </body>
</notes>
<listOfReactants>
```

```
      <speciesReference species="M_GTP_c" stoichiometry="1"/>
      <speciesReference species="M_H2O_c" stoichiometry="1"/>
    </listOfReactants>
    <listOfProducts>
      <speciesReference species="M_FAPYRM_NTP_c" stoichiometry="1"/>
    </listOfProducts>
    <kineticLaw>
      <math xmlns="http://www.w3.org/1998/Math/MathML">
        <ci> FLUX_VALUE </ci>
      </math>
      <listOfParameters>
        <parameter id="LOWER_BOUND" value="0" units="mmol_per_gDW_per_hr" constant="true"/>
        <parameter id="UPPER_BOUND" value="1000" units="mmol_per_gDW_per_hr" constant="true"/>
        <parameter id="FLUX_VALUE" value="0" units="mmol_per_gDW_per_hr" constant="true"/>
        <parameter id="OBJECTIVE_COEFFICIENT" value="0" units="mmol_per_gDW_per_hr" constant="true"/>
      </listOfParameters>
    </kineticLaw>
  </reaction>
  <reaction id="R_R487" name="formamidopyrimidine nucleoside triphosphate amidohydrolase" reversible="false">
    <notes>
      <body xmlns="http://www.w3.org/1999/xhtml">
        <p>GENE_ASSOCIATION: TTC1517</p>
        <p>SUBSYSTEM: Cofactor and Prosthetic Group Biosynthesis</p>
        <p>EC Number: 3.5.4.16</p>
        <p>Confidence Level: 1</p>
        <p>AUTHORS: Energy and cofactors metabolism</p>
        <p/>
      </body>
    </notes>
```

```
    <listOfReactants>
      <speciesReference species="M_H2O_c" stoichiometry="1"/>
      <speciesReference species="M_FAPYRM_NTP_c" stoichiometry="1"/>
    </listOfReactants>
    <listOfProducts>
      <speciesReference species="M_FOR_c" stoichiometry="1"/>
      <speciesReference species="M_DAPYRM_NTP_c" stoichiometry="1"/>
    </listOfProducts>
    <kineticLaw>
      <math xmlns="http://www.w3.org/1998/Math/MathML">
        <ci> FLUX_VALUE </ci>
      </math>
      <listOfParameters>
        <parameter id="LOWER_BOUND" value="0" units="mmol_per_gDW_per_hr" constant="true"/>
        <parameter id="UPPER_BOUND" value="1000" units="mmol_per_gDW_per_hr" constant="true"/>
        <parameter id="FLUX_VALUE" value="0" units="mmol_per_gDW_per_hr" constant="true"/>
        <parameter id="OBJECTIVE_COEFFICIENT" value="0" units="mmol_per_gDW_per_hr" constant="true"/>
      </listOfParameters>
    </kineticLaw>
  </reaction>
  <reaction id="R_R488" name="2,5-Diaminopyrimidine nucleoside triphosphate mutase" reversible="false">
    <notes>
      <body xmlns="http://www.w3.org/1999/xhtml">
        <p>GENE_ASSOCIATION: TTC1517</p>
        <p>SUBSYSTEM: Cofactor and Prosthetic Group Biosynthesis</p>
        <p>EC Number: 3.5.4.16</p>
        <p>Confidence Level: 1</p>
        <p>AUTHORS: Energy and cofactors metabolism</p>
        <p/>
```

```xml
    </body>
  </notes>
  <listOfReactants>
    <speciesReference species="M_DAPYRM_NTP_c" stoichiometry="1"/>
  </listOfReactants>
  <listOfProducts>
    <speciesReference species="M_DA_TPiTHY_OPENTAOPYRM_c" stoichiometry="1"/>
  </listOfProducts>
  <kineticLaw>
    <math xmlns="http://www.w3.org/1998/Math/MathML">
      <ci> FLUX_VALUE </ci>
    </math>
    <listOfParameters>
      <parameter id="LOWER_BOUND" value="0" units="mmol_per_gDW_per_hr" constant="true"/>
      <parameter id="UPPER_BOUND" value="1000" units="mmol_per_gDW_per_hr" constant="true"/>
      <parameter id="FLUX_VALUE" value="0" units="mmol_per_gDW_per_hr" constant="true"/>
      <parameter id="OBJECTIVE_COEFFICIENT" value="0" units="mmol_per_gDW_per_hr" constant="true"/>
    </listOfParameters>
  </kineticLaw>
</reaction>
<reaction id="R_R489" name="2-Amino-4-hydroxy-6-(erythro-1,2,3-trihydroxypropyl) dihydropteridine triphosphate hydrolase" reversible="false">
  <notes>
    <body xmlns="http://www.w3.org/1999/xhtml">
      <p>GENE_ASSOCIATION: TTC1517</p>
      <p>SUBSYSTEM: Cofactor and Prosthetic Group Biosynthesis</p>
      <p>EC Number: 3.5.4.16</p>
      <p>Confidence Level: 1</p>
      <p>AUTHORS: Energy and cofactors metabolism</p>
```

```xml
<p/>

</body>

</notes>

<listOfReactants>

<speciesReference species="M_DA_TPiTHY_OPENTAOPYRM_c" stoichiometry="1"/>

</listOfReactants>

<listOfProducts>

<speciesReference species="M_H2O_c" stoichiometry="1"/>

<speciesReference species="M_AHYRY_THYPROP_DHYPTRN_TPi_c" stoichiometry="1"/>

</listOfProducts>

<kineticLaw>

<math xmlns="http://www.w3.org/1998/Math/MathML">

<ci> FLUX_VALUE </ci>

</math>

<listOfParameters>

<parameter id="LOWER_BOUND" value="0" units="mmol_per_gDW_per_hr" constant="true"/>

<parameter id="UPPER_BOUND" value="1000" units="mmol_per_gDW_per_hr" constant="true"/>

<parameter id="FLUX_VALUE" value="0" units="mmol_per_gDW_per_hr" constant="true"/>

<parameter id="OBJECTIVE_COEFFICIENT" value="0" units="mmol_per_gDW_per_hr" constant="true"/>

</listOfParameters>

</kineticLaw>

</reaction>

<reaction id="R_R490" name="2-Amino-4-hydroxy-6-(erythro-1,2,3-trihydroxypropyl) dihydropteridine triphosphate phosphohydrolase (alkaline optimum)" reversible="false">

<notes>

<body xmlns="http://www.w3.org/1999/xhtml">

<p>GENE_ASSOCIATION: TT_P0024</p>

<p>SUBSYSTEM: Cofactor and Prosthetic Group Biosynthesis</p>

<p>EC Number: 3.1.3.1</p>
```

```
<p>Confidence Level: 1</p>
<p>AUTHORS: Energy and cofactors metabolism</p>
<p/>
</body>
</notes>
<listOfReactants>
<speciesReference species="M_H2O_c" stoichiometry="3"/>
<speciesReference species="M_AHYRY_THYPROP_DHYPTRN_TPi_c" stoichiometry="1"/>
</listOfReactants>
<listOfProducts>
<speciesReference species="M_Pi_c" stoichiometry="3"/>
<speciesReference species="M_H_c" stoichiometry="2"/>
<speciesReference species="M_DHY_NPTRN_c" stoichiometry="1"/>
</listOfProducts>
<kineticLaw>
<math xmlns="http://www.w3.org/1998/Math/MathML">
<ci> FLUX_VALUE </ci>
</math>
<listOfParameters>
<parameter id="LOWER_BOUND" value="0" units="mmol_per_gDW_per_hr" constant="true"/>
<parameter id="UPPER_BOUND" value="1000" units="mmol_per_gDW_per_hr" constant="true"/>
<parameter id="FLUX_VALUE" value="0" units="mmol_per_gDW_per_hr" constant="true"/>
<parameter id="OBJECTIVE_COEFFICIENT" value="0" units="mmol_per_gDW_per_hr" constant="true"/>
</listOfParameters>
</kineticLaw>
</reaction>
<reaction id="R_R491" name="2-amino-4-hydroxy-6-(D-erythro-1,2,3-trihydroxypropyl)-7,8-dihydropteridine glycolaldehyde-lyase" reversible="false">
<notes>
```

```xml
<body xmlns="http://www.w3.org/1999/xhtml">
<p>GENE_ASSOCIATION: TTC1796</p>
<p>SUBSYSTEM: Cofactor and Prosthetic Group Biosynthesis</p>
<p>EC Number: 4.1.2.25</p>
<p>Confidence Level: 1</p>
<p>AUTHORS: Energy and cofactors metabolism</p>
<p/>
</body>
</notes>
<listOfReactants>
<speciesReference species="M_DHY_NPTRN_c" stoichiometry="1"/>
</listOfReactants>
<listOfProducts>
<speciesReference species="M_H_c" stoichiometry="1"/>
<speciesReference species="M_GLYCALD_c" stoichiometry="1"/>
<speciesReference species="M_AHY_HYM_DHYPTR_c" stoichiometry="1"/>
</listOfProducts>
<kineticLaw>
<math xmlns="http://www.w3.org/1998/Math/MathML">
<ci> FLUX_VALUE </ci>
</math>
<listOfParameters>
<parameter id="LOWER_BOUND" value="0" units="mmol_per_gDW_per_hr" constant="true"/>
<parameter id="UPPER_BOUND" value="1000" units="mmol_per_gDW_per_hr" constant="true"/>
<parameter id="FLUX_VALUE" value="0" units="mmol_per_gDW_per_hr" constant="true"/>
<parameter id="OBJECTIVE_COEFFICIENT" value="0" units="mmol_per_gDW_per_hr" constant="true"/>
</listOfParameters>
</kineticLaw>
</reaction>
```

```
<reaction id="R_R492" name="ATP:2-amino-4-hydroxy-6-hydroxymethyl-7,8-dihydropteridine 6&apos;-pyrophosphotransferase" reversible="false">
  <notes>
    <body xmlns="http://www.w3.org/1999/xhtml">
      <p>GENE_ASSOCIATION: TTC1387</p>
      <p>SUBSYSTEM: Cofactor and Prosthetic Group Biosynthesis</p>
      <p>EC Number: 2.7.6.3</p>
      <p>Confidence Level: 1</p>
      <p>AUTHORS: Energy and cofactors metabolism</p>
      <p/>
    </body>
  </notes>
  <listOfReactants>
    <speciesReference species="M_ATP_c" stoichiometry="1"/>
    <speciesReference species="M_AHY_HYM_DHYPTR_c" stoichiometry="1"/>
  </listOfReactants>
  <listOfProducts>
    <speciesReference species="M_H_c" stoichiometry="1"/>
    <speciesReference species="M_AMP_c" stoichiometry="1"/>
    <speciesReference species="M_ADHY_DPOM_PTRDN_c" stoichiometry="1"/>
  </listOfProducts>
  <kineticLaw>
    <math xmlns="http://www.w3.org/1998/Math/MathML">
      <ci> FLUX_VALUE </ci>
    </math>
    <listOfParameters>
      <parameter id="LOWER_BOUND" value="0" units="mmol_per_gDW_per_hr" constant="true"/>
      <parameter id="UPPER_BOUND" value="1000" units="mmol_per_gDW_per_hr" constant="true"/>
      <parameter id="FLUX_VALUE" value="0" units="mmol_per_gDW_per_hr" constant="true"/>
```

```
<parameter id="OBJECTIVE_COEFFICIENT" value="0" units="mmol_per_gDW_per_hr" constant="true"/>
</listOfParameters>
</kineticLaw>
</reaction>
<reaction id="R_R493" name="2-amino-4-hydroxy-6-hydroxymethyl-7,8-dihydropteridine-diphosphate:4-aminobenzoate 2-amino-4-hydroxydihydropteridine-6-methenyltransferase" reversible="false">
<notes>
<body xmlns="http://www.w3.org/1999/xhtml">
<p>GENE_ASSOCIATION: TTC1795</p>
<p>SUBSYSTEM: Cofactor and Prosthetic Group Biosynthesis</p>
<p>EC Number: 2.5.1.15</p>
<p>Confidence Level: 1</p>
<p>AUTHORS: Energy and cofactors metabolism</p>
<p/>
</body>
</notes>
<listOfReactants>
<speciesReference species="M_ADHY_DPOM_PTRDN_c" stoichiometry="1"/>
<speciesReference species="M_ABEE_c" stoichiometry="1"/>
</listOfReactants>
<listOfProducts>
<speciesReference species="M_PPi_c" stoichiometry="1"/>
<speciesReference species="M_DHY_PTR_c" stoichiometry="1"/>
</listOfProducts>
<kineticLaw>
<math xmlns="http://www.w3.org/1998/Math/MathML">
<ci> FLUX_VALUE </ci>
</math>
<listOfParameters>
```

```xml
<parameter id="LOWER_BOUND" value="0" units="mmol_per_gDW_per_hr" constant="true"/>

<parameter id="UPPER_BOUND" value="1000" units="mmol_per_gDW_per_hr" constant="true"/>

<parameter id="FLUX_VALUE" value="0" units="mmol_per_gDW_per_hr" constant="true"/>

<parameter id="OBJECTIVE_COEFFICIENT" value="0" units="mmol_per_gDW_per_hr" constant="true"/>

</listOfParameters>

</kineticLaw>

</reaction>

<reaction id="R_R494" name="2-amino-4-hydroxy-6-hydroxymethyl-7,8-dihydropteridine:4-aminobenzoate 2-amino-4-hydroxydihydropteridine-6-methenyltransferase" reversible="false">

<notes>

<body xmlns="http://www.w3.org/1999/xhtml">

<p>GENE_ASSOCIATION: TTC1795</p>

<p>SUBSYSTEM: Cofactor and Prosthetic Group Biosynthesis</p>

<p>EC Number: 2.5.1.15</p>

<p>Confidence Level: 1</p>

<p>AUTHORS: Energy and cofactors metabolism</p>

<p/>

</body>

</notes>

<listOfReactants>

<speciesReference species="M_AHY_HYM_DHYPTR_c" stoichiometry="1"/>

<speciesReference species="M_ABEE_c" stoichiometry="1"/>

</listOfReactants>

<listOfProducts>

<speciesReference species="M_H2O_c" stoichiometry="1"/>

<speciesReference species="M_DHY_PTR_c" stoichiometry="1"/>

</listOfProducts>

<kineticLaw>

<math xmlns="http://www.w3.org/1998/Math/MathML">
```

```
      <ci> FLUX_VALUE </ci>
    </math>
    <listOfParameters>
      <parameter id="LOWER_BOUND" value="0" units="mmol_per_gDW_per_hr" constant="true"/>
      <parameter id="UPPER_BOUND" value="1000" units="mmol_per_gDW_per_hr" constant="true"/>
      <parameter id="FLUX_VALUE" value="0" units="mmol_per_gDW_per_hr" constant="true"/>
      <parameter id="OBJECTIVE_COEFFICIENT" value="0" units="mmol_per_gDW_per_hr" constant="true"/>
    </listOfParameters>
  </kineticLaw>
</reaction>
<reaction id="R_R495" name="Tetrahydrofolate:L-glutamate gamma-ligase (ADP-forming)" reversible="true">
  <notes>
    <body xmlns="http://www.w3.org/1999/xhtml">
      <p>GENE_ASSOCIATION: (TTC0975 or TTC1640)</p>
      <p>SUBSYSTEM: Cofactor and Prosthetic Group Biosynthesis</p>
      <p>EC Number: 6.3.2.17</p>
      <p>Confidence Level: 1</p>
      <p>AUTHORS: Energy and cofactors metabolism</p>
      <p/>
    </body>
  </notes>
  <listOfReactants>
    <speciesReference species="M_L_GLU_c" stoichiometry="1"/>
    <speciesReference species="M_ATP_c" stoichiometry="1"/>
    <speciesReference species="M_THF_c" stoichiometry="1"/>
  </listOfReactants>
  <listOfProducts>
    <speciesReference species="M_ADP_c" stoichiometry="1"/>
    <speciesReference species="M_Pi_c" stoichiometry="1"/>
```

```
    <speciesReference species="M_THF_GLUT_c" stoichiometry="1"/>
  </listOfProducts>
  <kineticLaw>
    <math xmlns="http://www.w3.org/1998/Math/MathML">
      <ci> FLUX_VALUE </ci>
    </math>
    <listOfParameters>
      <parameter id="LOWER_BOUND" value="-1000" units="mmol_per_gDW_per_hr" constant="true"/>
      <parameter id="UPPER_BOUND" value="1000" units="mmol_per_gDW_per_hr" constant="true"/>
      <parameter id="FLUX_VALUE" value="0" units="mmol_per_gDW_per_hr" constant="true"/>
      <parameter id="OBJECTIVE_COEFFICIENT" value="0" units="mmol_per_gDW_per_hr" constant="true"/>
    </listOfParameters>
  </kineticLaw>
</reaction>
<reaction id="R_R496" name="7,8-dihydropteroate:L-glutamate ligase (ADP-forming)" reversible="false">
  <notes>
    <body xmlns="http://www.w3.org/1999/xhtml">
      <p>GENE_ASSOCIATION: (TTC0975 or TTC1640)</p>
      <p>SUBSYSTEM: Cofactor and Prosthetic Group Biosynthesis</p>
      <p>EC Number: 6.3.2.17</p>
      <p>Confidence Level: 1</p>
      <p>AUTHORS: Energy and cofactors metabolism</p>
      <p/>
    </body>
  </notes>
  <listOfReactants>
    <speciesReference species="M_L_GLU_c" stoichiometry="1"/>
    <speciesReference species="M_ATP_c" stoichiometry="1"/>
    <speciesReference species="M_DHY_PTR_c" stoichiometry="1"/>
```

```xml
</listOfReactants>
<listOfProducts>
<speciesReference species="M_ADP_c" stoichiometry="1"/>
<speciesReference species="M_Pi_c" stoichiometry="1"/>
<speciesReference species="M_H_c" stoichiometry="1"/>
<speciesReference species="M_DHF_c" stoichiometry="1"/>
</listOfProducts>
<kineticLaw>
<math xmlns="http://www.w3.org/1998/Math/MathML">
<ci> FLUX_VALUE </ci>
</math>
<listOfParameters>
<parameter id="LOWER_BOUND" value="0" units="mmol_per_gDW_per_hr" constant="true"/>
<parameter id="UPPER_BOUND" value="1000" units="mmol_per_gDW_per_hr" constant="true"/>
<parameter id="FLUX_VALUE" value="0" units="mmol_per_gDW_per_hr" constant="true"/>
<parameter id="OBJECTIVE_COEFFICIENT" value="0" units="mmol_per_gDW_per_hr" constant="true"/>
</listOfParameters>
</kineticLaw>
</reaction>
<reaction id="R_R497" name="1-Deoxy-D-xylulose-5-phosphate pyruvate-lyase (carboxylating)" reversible="false">
<notes>
<body xmlns="http://www.w3.org/1999/xhtml">
<p>GENE_ASSOCIATION: TTC1614</p>
<p>SUBSYSTEM: Cofactor and Prosthetic Group Biosynthesis</p>
<p>EC Number: 2.2.1.7</p>
<p>Confidence Level: 1</p>
<p>AUTHORS: Carotenoid metabolism</p>
<p/>
</body>
```

```
</notes>

<listOfReactants>

<speciesReference species="M_H_c" stoichiometry="1"/>

<speciesReference species="M_GAP_c" stoichiometry="1"/>

<speciesReference species="M_PYR_c" stoichiometry="1"/>

</listOfReactants>

<listOfProducts>

<speciesReference species="M_CO2_c" stoichiometry="1"/>

<speciesReference species="M_dXYLL5P_c" stoichiometry="1"/>

</listOfProducts>

<kineticLaw>

<math xmlns="http://www.w3.org/1998/Math/MathML">

<ci> FLUX_VALUE </ci>

</math>

<listOfParameters>

<parameter id="LOWER_BOUND" value="0" units="mmol_per_gDW_per_hr" constant="true"/>

<parameter id="UPPER_BOUND" value="1000" units="mmol_per_gDW_per_hr" constant="true"/>

<parameter id="FLUX_VALUE" value="0" units="mmol_per_gDW_per_hr" constant="true"/>

<parameter id="OBJECTIVE_COEFFICIENT" value="0" units="mmol_per_gDW_per_hr" constant="true"/>

</listOfParameters>

</kineticLaw>

</reaction>

<reaction id="R_R498" name="1-Deoxy-D-xylulose-5-phosphate isomeroreductase" reversible="false">

<notes>

<body xmlns="http://www.w3.org/1999/xhtml">

<p>GENE_ASSOCIATION: TTC0504</p>

<p>SUBSYSTEM: Cofactor and Prosthetic Group Biosynthesis</p>

<p>EC Number: 1.1.1.267</p>

<p>Confidence Level: 1</p>
```

<p>AUTHORS: Carotenoid metabolism</p>

<p/>

</body>

</notes>

<listOfReactants>

<speciesReference species="M_H_c" stoichiometry="1"/>

<speciesReference species="M_NADPH_c" stoichiometry="1"/>

<speciesReference species="M_dXYLL5P_c" stoichiometry="1"/>

</listOfReactants>

<listOfProducts>

<speciesReference species="M_NADP_c" stoichiometry="1"/>

<speciesReference species="M_MEROLPi_c" stoichiometry="1"/>

</listOfProducts>

<kineticLaw>

<math xmlns="http://www.w3.org/1998/Math/MathML">

<ci> FLUX_VALUE </ci>

</math>

<listOfParameters>

<parameter id="LOWER_BOUND" value="0" units="mmol_per_gDW_per_hr" constant="true"/>

<parameter id="UPPER_BOUND" value="1000" units="mmol_per_gDW_per_hr" constant="true"/>

<parameter id="FLUX_VALUE" value="0" units="mmol_per_gDW_per_hr" constant="true"/>

<parameter id="OBJECTIVE_COEFFICIENT" value="0" units="mmol_per_gDW_per_hr" constant="true"/>

</listOfParameters>

</kineticLaw>

</reaction>

<reaction id="R_R499" name="CTP: 2-C-Methyl-D-erythritol 4-phosphate cytidylyltransferase" reversible="false">

<notes>

<body xmlns="http://www.w3.org/1999/xhtml">

<p>GENE_ASSOCIATION: TTC1815</p>

```
      <p>SUBSYSTEM: Cofactor and Prosthetic Group Biosynthesis</p>
      <p>EC Number: 2.7.7.60</p>
      <p>Confidence Level: 1</p>
      <p>AUTHORS: Carotenoid metabolism</p>
      <p/>
    </body>
  </notes>
  <listOfReactants>
    <speciesReference species="M_CTP_c" stoichiometry="1"/>
    <speciesReference species="M_H_c" stoichiometry="1"/>
    <speciesReference species="M_MEROLPi_c" stoichiometry="1"/>
  </listOfReactants>
  <listOfProducts>
    <speciesReference species="M_PPi_c" stoichiometry="1"/>
    <speciesReference species="M_CYTPPi_MEROL_c" stoichiometry="1"/>
  </listOfProducts>
  <kineticLaw>
    <math xmlns="http://www.w3.org/1998/Math/MathML">
      <ci> FLUX_VALUE </ci>
    </math>
    <listOfParameters>
      <parameter id="LOWER_BOUND" value="0" units="mmol_per_gDW_per_hr" constant="true"/>
      <parameter id="UPPER_BOUND" value="1000" units="mmol_per_gDW_per_hr" constant="true"/>
      <parameter id="FLUX_VALUE" value="0" units="mmol_per_gDW_per_hr" constant="true"/>
      <parameter id="OBJECTIVE_COEFFICIENT" value="0" units="mmol_per_gDW_per_hr" constant="true"/>
    </listOfParameters>
  </kineticLaw>
</reaction>
```

```
<reaction id="R_R500" name="ATP:4-(Cytidine 5&apos;-diphospho)-2-C-methyl-D-erythritol 2-phosphotransferase" reversible="false">
  <notes>
    <body xmlns="http://www.w3.org/1999/xhtml">
      <p>GENE_ASSOCIATION: TTC1816</p>
      <p>SUBSYSTEM: Cofactor and Prosthetic Group Biosynthesis</p>
      <p>EC Number: 2.7.1.148</p>
      <p>Confidence Level: 1</p>
      <p>AUTHORS: Carotenoid metabolism</p>
      <p/>
    </body>
  </notes>
  <listOfReactants>
    <speciesReference species="M_ATP_c" stoichiometry="1"/>
    <speciesReference species="M_CYTPPi_MEROL_c" stoichiometry="1"/>
  </listOfReactants>
  <listOfProducts>
    <speciesReference species="M_ADP_c" stoichiometry="1"/>
    <speciesReference species="M_H_c" stoichiometry="1"/>
    <speciesReference species="M_PCYTPPi_MEROL_c" stoichiometry="1"/>
  </listOfProducts>
  <kineticLaw>
    <math xmlns="http://www.w3.org/1998/Math/MathML">
      <ci> FLUX_VALUE </ci>
    </math>
    <listOfParameters>
      <parameter id="LOWER_BOUND" value="0" units="mmol_per_gDW_per_hr" constant="true"/>
      <parameter id="UPPER_BOUND" value="1000" units="mmol_per_gDW_per_hr" constant="true"/>
      <parameter id="FLUX_VALUE" value="0" units="mmol_per_gDW_per_hr" constant="true"/>
```

```xml
<parameter id="OBJECTIVE_COEFFICIENT" value="0" units="mmol_per_gDW_per_hr" constant="true"/>
</listOfParameters>
</kineticLaw>
</reaction>
<reaction id="R_R501" name="2-Phospho-4-(cytidine 5&apos;-diphospho)-2-C-methyl-D-erythritol CMP-lyase (cyclizing)" reversible="false">
<notes>
<body xmlns="http://www.w3.org/1999/xhtml">
<p>GENE_ASSOCIATION: TTC1438</p>
<p>SUBSYSTEM: Cofactor and Prosthetic Group Biosynthesis</p>
<p>EC Number: 4.6.1.12</p>
<p>Confidence Level: 1</p>
<p>AUTHORS: Carotenoid metabolism</p>
<p/>
</body>
</notes>
<listOfReactants>
<speciesReference species="M_PCYTPPi_MEROL_c" stoichiometry="1"/>
</listOfReactants>
<listOfProducts>
<speciesReference species="M_CMP_c" stoichiometry="1"/>
<speciesReference species="M_MEROL_CYCPPi_c" stoichiometry="1"/>
</listOfProducts>
<kineticLaw>
<math xmlns="http://www.w3.org/1998/Math/MathML">
<ci> FLUX_VALUE </ci>
</math>
<listOfParameters>
<parameter id="LOWER_BOUND" value="0" units="mmol_per_gDW_per_hr" constant="true"/>
```

<parameter id="UPPER_BOUND" value="1000" units="mmol_per_gDW_per_hr" constant="true"/>

<parameter id="FLUX_VALUE" value="0" units="mmol_per_gDW_per_hr" constant="true"/>

<parameter id="OBJECTIVE_COEFFICIENT" value="0" units="mmol_per_gDW_per_hr" constant="true"/>

</listOfParameters>

</kineticLaw>

</reaction>

<reaction id="R_R502" name="(E)-4-hydroxy-3-methylbut-2-en-1-yl-diphosphate:oxidized ferredoxin oxidoreductase (hydrating)" reversible="false">

<notes>

<body xmlns="http://www.w3.org/1999/xhtml">

<p>GENE_ASSOCIATION: TTC1677</p>

<p>SUBSYSTEM: Cofactor and Prosthetic Group Biosynthesis</p>

<p>EC Number: 1.17.7.1</p>

<p>Confidence Level: 1</p>

<p>AUTHORS: Carotenoid metabolism</p>

<p/>

</body>

</notes>

<listOfReactants>

<speciesReference species="M_H_c" stoichiometry="1"/>

<speciesReference species="M_MEROL_CYCPPi_c" stoichiometry="1"/>

<speciesReference species="M_R_FRDX_c" stoichiometry="2"/>

</listOfReactants>

<listOfProducts>

<speciesReference species="M_H2O_c" stoichiometry="1"/>

<speciesReference species="M_HY_MBUTEPPi_c" stoichiometry="1"/>

<speciesReference species="M_O_FRDX_c" stoichiometry="2"/>

</listOfProducts>

<kineticLaw>

```
    <math xmlns="http://www.w3.org/1998/Math/MathML">
      <ci> FLUX_VALUE </ci>
    </math>
    <listOfParameters>
      <parameter id="LOWER_BOUND" value="0" units="mmol_per_gDW_per_hr" constant="true"/>
      <parameter id="UPPER_BOUND" value="1000" units="mmol_per_gDW_per_hr" constant="true"/>
      <parameter id="FLUX_VALUE" value="0" units="mmol_per_gDW_per_hr" constant="true"/>
      <parameter id="OBJECTIVE_COEFFICIENT" value="0" units="mmol_per_gDW_per_hr" constant="true"/>
    </listOfParameters>
  </kineticLaw>
</reaction>
<reaction id="R_R503" name="isopentenyl-diphosphate:NAD(P)+ oxidoreductase" reversible="false">
  <notes>
    <body xmlns="http://www.w3.org/1999/xhtml">
      <p>GENE_ASSOCIATION: TTC1983</p>
      <p>SUBSYSTEM: Cofactor and Prosthetic Group Biosynthesis</p>
      <p>EC Number: 1.17.1.2</p>
      <p>Confidence Level: 1</p>
      <p>AUTHORS: Carotenoid metabolism</p>
      <p/>
    </body>
  </notes>
  <listOfReactants>
    <speciesReference species="M_H_c" stoichiometry="1"/>
    <speciesReference species="M_NADPH_c" stoichiometry="1"/>
    <speciesReference species="M_HY_MBUTEPPi_c" stoichiometry="1"/>
  </listOfReactants>
  <listOfProducts>
    <speciesReference species="M_H2O_c" stoichiometry="1"/>
```

```
    <speciesReference species="M_NADP_c" stoichiometry="1"/>
    <speciesReference species="M_IPEN_PPi_c" stoichiometry="1"/>
  </listOfProducts>
  <kineticLaw>
    <math xmlns="http://www.w3.org/1998/Math/MathML">
      <ci> FLUX_VALUE </ci>
    </math>
    <listOfParameters>
      <parameter id="LOWER_BOUND" value="0" units="mmol_per_gDW_per_hr" constant="true"/>
      <parameter id="UPPER_BOUND" value="1000" units="mmol_per_gDW_per_hr" constant="true"/>
      <parameter id="FLUX_VALUE" value="0" units="mmol_per_gDW_per_hr" constant="true"/>
      <parameter id="OBJECTIVE_COEFFICIENT" value="0" units="mmol_per_gDW_per_hr" constant="true"/>
    </listOfParameters>
  </kineticLaw>
</reaction>
<reaction id="R_R504" name="dimethyallyl diphosphate:NADP+ oxidoreductase" reversible="false">
  <notes>
    <body xmlns="http://www.w3.org/1999/xhtml">
      <p>GENE_ASSOCIATION: TTC1983</p>
      <p>SUBSYSTEM: Cofactor and Prosthetic Group Biosynthesis</p>
      <p>EC Number: 1.17.1.2</p>
      <p>Confidence Level: 1</p>
      <p>AUTHORS: Carotenoid metabolism</p>
      <p/>
    </body>
  </notes>
  <listOfReactants>
    <speciesReference species="M_H_c" stoichiometry="1"/>
    <speciesReference species="M_NADPH_c" stoichiometry="1"/>
```

```
      <speciesReference species="M_HY_MBUTEPPi_c" stoichiometry="1"/>
    </listOfReactants>
    <listOfProducts>
      <speciesReference species="M_H2O_c" stoichiometry="1"/>
      <speciesReference species="M_NADP_c" stoichiometry="1"/>
      <speciesReference species="M_DMAPP_c" stoichiometry="1"/>
    </listOfProducts>
    <kineticLaw>
      <math xmlns="http://www.w3.org/1998/Math/MathML">
        <ci> FLUX_VALUE </ci>
      </math>
      <listOfParameters>
        <parameter id="LOWER_BOUND" value="0" units="mmol_per_gDW_per_hr" constant="true"/>
        <parameter id="UPPER_BOUND" value="1000" units="mmol_per_gDW_per_hr" constant="true"/>
        <parameter id="FLUX_VALUE" value="0" units="mmol_per_gDW_per_hr" constant="true"/>
        <parameter id="OBJECTIVE_COEFFICIENT" value="0" units="mmol_per_gDW_per_hr" constant="true"/>
      </listOfParameters>
    </kineticLaw>
  </reaction>
  <reaction id="R_R505" name="isopentenyl-diphosphate:NAD+ oxidoreductase" reversible="false">
    <notes>
      <body xmlns="http://www.w3.org/1999/xhtml">
        <p>GENE_ASSOCIATION: TTC1983</p>
        <p>SUBSYSTEM: Cofactor and Prosthetic Group Biosynthesis</p>
        <p>EC Number: 1.17.1.2</p>
        <p>Confidence Level: 1</p>
        <p>AUTHORS: Carotenoid metabolism</p>
        <p/>
      </body>
```

```xml
  </notes>
  <listOfReactants>
    <speciesReference species="M_H_c" stoichiometry="1"/>
    <speciesReference species="M_NADH_c" stoichiometry="1"/>
    <speciesReference species="M_HY_MBUTEPPi_c" stoichiometry="1"/>
  </listOfReactants>
  <listOfProducts>
    <speciesReference species="M_H2O_c" stoichiometry="1"/>
    <speciesReference species="M_NAD_c" stoichiometry="1"/>
    <speciesReference species="M_IPEN_PPi_c" stoichiometry="1"/>
  </listOfProducts>
  <kineticLaw>
    <math xmlns="http://www.w3.org/1998/Math/MathML">
      <ci> FLUX_VALUE </ci>
    </math>
    <listOfParameters>
      <parameter id="LOWER_BOUND" value="0" units="mmol_per_gDW_per_hr" constant="true"/>
      <parameter id="UPPER_BOUND" value="1000" units="mmol_per_gDW_per_hr" constant="true"/>
      <parameter id="FLUX_VALUE" value="0" units="mmol_per_gDW_per_hr" constant="true"/>
      <parameter id="OBJECTIVE_COEFFICIENT" value="0" units="mmol_per_gDW_per_hr" constant="true"/>
    </listOfParameters>
  </kineticLaw>
</reaction>
<reaction id="R_R506" name="dimethyallyl diphosphate:NAD+ oxidoreductase" reversible="false">
  <notes>
    <body xmlns="http://www.w3.org/1999/xhtml">
      <p>GENE_ASSOCIATION: TTC1983</p>
      <p>SUBSYSTEM: Cofactor and Prosthetic Group Biosynthesis</p>
      <p>EC Number: 1.17.1.2</p>
```

```
      <p>Confidence Level: 1</p>
      <p>AUTHORS: Carotenoid metabolism</p>
      <p/>
    </body>
  </notes>
  <listOfReactants>
    <speciesReference species="M_H_c" stoichiometry="1"/>
    <speciesReference species="M_NADH_c" stoichiometry="1"/>
    <speciesReference species="M_HY_MBUTEPPi_c" stoichiometry="1"/>
  </listOfReactants>
  <listOfProducts>
    <speciesReference species="M_H2O_c" stoichiometry="1"/>
    <speciesReference species="M_NAD_c" stoichiometry="1"/>
    <speciesReference species="M_DMAPP_c" stoichiometry="1"/>
  </listOfProducts>
  <kineticLaw>
    <math xmlns="http://www.w3.org/1998/Math/MathML">
      <ci> FLUX_VALUE </ci>
    </math>
    <listOfParameters>
      <parameter id="LOWER_BOUND" value="0" units="mmol_per_gDW_per_hr" constant="true"/>
      <parameter id="UPPER_BOUND" value="1000" units="mmol_per_gDW_per_hr" constant="true"/>
      <parameter id="FLUX_VALUE" value="0" units="mmol_per_gDW_per_hr" constant="true"/>
      <parameter id="OBJECTIVE_COEFFICIENT" value="0" units="mmol_per_gDW_per_hr" constant="true"/>
    </listOfParameters>
  </kineticLaw>
</reaction>
<reaction id="R_R507" name="Z-farnesyl diphosphate synthase" reversible="false">
  <notes>
```

```
    <body xmlns="http://www.w3.org/1999/xhtml">
      <p>GENE_ASSOCIATION: TTC1551</p>
      <p>SUBSYSTEM: Cofactor and Prosthetic Group Biosynthesis</p>
      <p>EC Number: 2.5.1.68</p>
      <p>Confidence Level: 1</p>
      <p>AUTHORS: Carotenoid metabolism</p>
      <p/>
    </body>
  </notes>
  <listOfReactants>
    <speciesReference species="M_IPEN_PPi_c" stoichiometry="1"/>
    <speciesReference species="M_GRN_PPi_c" stoichiometry="1"/>
  </listOfReactants>
  <listOfProducts>
    <speciesReference species="M_PPi_c" stoichiometry="1"/>
    <speciesReference species="M_ct_FARPPi_c" stoichiometry="1"/>
  </listOfProducts>
  <kineticLaw>
    <math xmlns="http://www.w3.org/1998/Math/MathML">
      <ci> FLUX_VALUE </ci>
    </math>
    <listOfParameters>
      <parameter id="LOWER_BOUND" value="0" units="mmol_per_gDW_per_hr" constant="true"/>
      <parameter id="UPPER_BOUND" value="1000" units="mmol_per_gDW_per_hr" constant="true"/>
      <parameter id="FLUX_VALUE" value="0" units="mmol_per_gDW_per_hr" constant="true"/>
      <parameter id="OBJECTIVE_COEFFICIENT" value="0" units="mmol_per_gDW_per_hr" constant="true"/>
    </listOfParameters>
  </kineticLaw>
</reaction>
```

```
<reaction id="R_R508" name="Dimethylallyl-diphosphate:isopentenyl-diphosphate dimethylallyltranstransferase" reversible="false">
  <notes>
    <body xmlns="http://www.w3.org/1999/xhtml">
      <p>GENE_ASSOCIATION: TTC1986</p>
      <p>SUBSYSTEM: Cofactor and Prosthetic Group Biosynthesis</p>
      <p>EC Number: 2.5.1.1</p>
      <p>Confidence Level: 1</p>
      <p>AUTHORS: Carotenoid metabolism</p>
      <p/>
    </body>
  </notes>
  <listOfReactants>
    <speciesReference species="M_IPEN_PPi_c" stoichiometry="1"/>
    <speciesReference species="M_DMAPP_c" stoichiometry="1"/>
  </listOfReactants>
  <listOfProducts>
    <speciesReference species="M_PPi_c" stoichiometry="1"/>
    <speciesReference species="M_GRN_PPi_c" stoichiometry="1"/>
  </listOfProducts>
  <kineticLaw>
    <math xmlns="http://www.w3.org/1998/Math/MathML">
      <ci> FLUX_VALUE </ci>
    </math>
    <listOfParameters>
      <parameter id="LOWER_BOUND" value="0" units="mmol_per_gDW_per_hr" constant="true"/>
      <parameter id="UPPER_BOUND" value="1000" units="mmol_per_gDW_per_hr" constant="true"/>
      <parameter id="FLUX_VALUE" value="0" units="mmol_per_gDW_per_hr" constant="true"/>
      <parameter id="OBJECTIVE_COEFFICIENT" value="0" units="mmol_per_gDW_per_hr" constant="true"/>
```

```
    </listOfParameters>
  </kineticLaw>
</reaction>
<reaction id="R_R509" name="Isopentenyl-diphosphate delta3-delta2-isomerase" reversible="true">
  <notes>
    <body xmlns="http://www.w3.org/1999/xhtml">
      <p>GENE_ASSOCIATION: (TT_P0067 or TTC0228)</p>
      <p>SUBSYSTEM: Cofactor and Prosthetic Group Biosynthesis</p>
      <p>EC Number: 5.3.3.2</p>
      <p>Confidence Level: 1</p>
      <p>AUTHORS: Carotenoid metabolism</p>
      <p/>
    </body>
  </notes>
  <listOfReactants>
    <speciesReference species="M_IPEN_PPi_c" stoichiometry="1"/>
  </listOfReactants>
  <listOfProducts>
    <speciesReference species="M_DMAPP_c" stoichiometry="1"/>
  </listOfProducts>
  <kineticLaw>
    <math xmlns="http://www.w3.org/1998/Math/MathML">
      <ci> FLUX_VALUE </ci>
    </math>
    <listOfParameters>
      <parameter id="LOWER_BOUND" value="-1000" units="mmol_per_gDW_per_hr" constant="true"/>
      <parameter id="UPPER_BOUND" value="1000" units="mmol_per_gDW_per_hr" constant="true"/>
      <parameter id="FLUX_VALUE" value="0" units="mmol_per_gDW_per_hr" constant="true"/>
      <parameter id="OBJECTIVE_COEFFICIENT" value="0" units="mmol_per_gDW_per_hr" constant="true"/>
```

```xml
    </listOfParameters>
  </kineticLaw>
</reaction>
<reaction id="R_R510" name="Geranyl-diphosphate:isopentenyl-diphosphate geranyltrans-transferase" reversible="false">
  <notes>
    <body xmlns="http://www.w3.org/1999/xhtml">
      <p>GENE_ASSOCIATION: TTC1986</p>
      <p>SUBSYSTEM: Cofactor and Prosthetic Group Biosynthesis</p>
      <p>EC Number: 2.5.1.10</p>
      <p>Confidence Level: 1</p>
      <p>AUTHORS: Carotenoid metabolism</p>
      <p/>
    </body>
  </notes>
  <listOfReactants>
    <speciesReference species="M_IPEN_PPi_c" stoichiometry="1"/>
    <speciesReference species="M_GRN_PPi_c" stoichiometry="1"/>
  </listOfReactants>
  <listOfProducts>
    <speciesReference species="M_PPi_c" stoichiometry="1"/>
    <speciesReference species="M_FAR_PPi_c" stoichiometry="1"/>
  </listOfProducts>
  <kineticLaw>
    <math xmlns="http://www.w3.org/1998/Math/MathML">
      <ci> FLUX_VALUE </ci>
    </math>
    <listOfParameters>
      <parameter id="LOWER_BOUND" value="0" units="mmol_per_gDW_per_hr" constant="true"/>
      <parameter id="UPPER_BOUND" value="1000" units="mmol_per_gDW_per_hr" constant="true"/>
```

<parameter id="FLUX_VALUE" value="0" units="mmol_per_gDW_per_hr" constant="true"/>

<parameter id="OBJECTIVE_COEFFICIENT" value="0" units="mmol_per_gDW_per_hr" constant="true"/>

</listOfParameters>

</kineticLaw>

</reaction>

<reaction id="R_R511" name="trans,trans-Farnesyl-diphosphate:isopentenyl-diphosphate farnesyltranstransferase" reversible="false">

<notes>

<body xmlns="http://www.w3.org/1999/xhtml">

<p>GENE_ASSOCIATION: TTC1986</p>

<p>SUBSYSTEM: Cofactor and Prosthetic Group Biosynthesis</p>

<p>EC Number: 2.5.1.29</p>

<p>Confidence Level: 1</p>

<p>AUTHORS: Carotenoid metabolism</p>

<p/>

</body>

</notes>

<listOfReactants>

<speciesReference species="M_IPEN_PPi_c" stoichiometry="1"/>

<speciesReference species="M_FAR_PPi_c" stoichiometry="1"/>

</listOfReactants>

<listOfProducts>

<speciesReference species="M_PPi_c" stoichiometry="1"/>

<speciesReference species="M_GRN_GRNPPi_c" stoichiometry="1"/>

</listOfProducts>

<kineticLaw>

<math xmlns="http://www.w3.org/1998/Math/MathML">

<ci> FLUX_VALUE </ci>

</math>

```
    <listOfParameters>
      <parameter id="LOWER_BOUND" value="0" units="mmol_per_gDW_per_hr" constant="true"/>
      <parameter id="UPPER_BOUND" value="1000" units="mmol_per_gDW_per_hr" constant="true"/>
      <parameter id="FLUX_VALUE" value="0" units="mmol_per_gDW_per_hr" constant="true"/>
      <parameter id="OBJECTIVE_COEFFICIENT" value="0" units="mmol_per_gDW_per_hr" constant="true"/>
    </listOfParameters>
  </kineticLaw>
</reaction>
<reaction id="R_R512" name="carvone reductase" reversible="false">
  <notes>
    <body xmlns="http://www.w3.org/1999/xhtml">
      <p>GENE_ASSOCIATION: </p>
      <p>SUBSYSTEM: Limonene and pinene degradation</p>
      <p>EC Number: 1.3.99.25</p>
      <p>Confidence Level: 1</p>
      <p>AUTHORS: Carotenoid metabolism</p>
      <p/>
    </body>
  </notes>
  <listOfReactants>
    <speciesReference species="M_H_c" stoichiometry="1"/>
    <speciesReference species="M_CRVN_c" stoichiometry="1"/>
  </listOfReactants>
  <listOfProducts>
    <speciesReference species="M_IDHY_CRVN_c" stoichiometry="1"/>
  </listOfProducts>
  <kineticLaw>
    <math xmlns="http://www.w3.org/1998/Math/MathML">
      <ci> FLUX_VALUE </ci>
```

```xml
</math>

<listOfParameters>

<parameter id="LOWER_BOUND" value="0" units="mmol_per_gDW_per_hr" constant="true"/>

<parameter id="UPPER_BOUND" value="1000" units="mmol_per_gDW_per_hr" constant="true"/>

<parameter id="FLUX_VALUE" value="0" units="mmol_per_gDW_per_hr" constant="true"/>

<parameter id="OBJECTIVE_COEFFICIENT" value="0" units="mmol_per_gDW_per_hr" constant="true"/>

</listOfParameters>

</kineticLaw>

</reaction>

<reaction id="R_R513" name="geranylgeranyl-diphosphate:geranylgeranyl-diphosphate geranylgeranyltransferase" reversible="false">

<notes>

<body xmlns="http://www.w3.org/1999/xhtml">

<p>GENE_ASSOCIATION: TT_P0057</p>

<p>SUBSYSTEM: Carotenoid biosynthesis</p>

<p>EC Number: 2.5.1.32</p>

<p>Confidence Level: 1</p>

<p>AUTHORS: Carotenoid metabolism</p>

<p/>

</body>

</notes>

<listOfReactants>

<speciesReference species="M_GRN_GRNPPi_c" stoichiometry="2"/>

</listOfReactants>

<listOfProducts>

<speciesReference species="M_PPi_c" stoichiometry="2"/>

<speciesReference species="M_PHTEN_c" stoichiometry="1"/>

</listOfProducts>

<kineticLaw>
```

```
<math xmlns="http://www.w3.org/1998/Math/MathML">
  <ci> FLUX_VALUE </ci>
</math>
<listOfParameters>
  <parameter id="LOWER_BOUND" value="0" units="mmol_per_gDW_per_hr" constant="true"/>
  <parameter id="UPPER_BOUND" value="1000" units="mmol_per_gDW_per_hr" constant="true"/>
  <parameter id="FLUX_VALUE" value="0" units="mmol_per_gDW_per_hr" constant="true"/>
  <parameter id="OBJECTIVE_COEFFICIENT" value="0" units="mmol_per_gDW_per_hr" constant="true"/>
</listOfParameters>
</kineticLaw>
</reaction>
<reaction id="R_R514" name="15-cis-phytoene:plastoquinone oxidoreductase" reversible="false">
 <notes>
  <body xmlns="http://www.w3.org/1999/xhtml">
   <p>GENE_ASSOCIATION: (TT_P0047 or TT_P0066)</p>
   <p>SUBSYSTEM: Carotenoid biosynthesis</p>
   <p>EC Number: 1.3.5.5</p>
   <p>Confidence Level: 1</p>
   <p>AUTHORS: Carotenoid metabolism</p>
   <p/>
  </body>
 </notes>
 <listOfReactants>
  <speciesReference species="M_FAD_c" stoichiometry="1"/>
  <speciesReference species="M_PHTEN_c" stoichiometry="1"/>
 </listOfReactants>
 <listOfProducts>
  <speciesReference species="M_FADH2_c" stoichiometry="1"/>
  <speciesReference species="M_PHFLN_c" stoichiometry="1"/>
```

```
  </listOfProducts>
  <kineticLaw>
    <math xmlns="http://www.w3.org/1998/Math/MathML">
      <ci> FLUX_VALUE </ci>
    </math>
    <listOfParameters>
      <parameter id="LOWER_BOUND" value="0" units="mmol_per_gDW_per_hr" constant="true"/>
      <parameter id="UPPER_BOUND" value="1000" units="mmol_per_gDW_per_hr" constant="true"/>
      <parameter id="FLUX_VALUE" value="0" units="mmol_per_gDW_per_hr" constant="true"/>
      <parameter id="OBJECTIVE_COEFFICIENT" value="0" units="mmol_per_gDW_per_hr" constant="true"/>
    </listOfParameters>
  </kineticLaw>
</reaction>
<reaction id="R_R515" name="15-cis-phytoene:acceptor oxidoreductase" reversible="true">
  <notes>
    <body xmlns="http://www.w3.org/1999/xhtml">
      <p>GENE_ASSOCIATION: TT_P0066</p>
      <p>SUBSYSTEM: Carotenoid biosynthesis</p>
      <p>EC Number: 1.3.99.28</p>
      <p>Confidence Level: 1</p>
      <p>AUTHORS: Carotenoid metabolism</p>
      <p/>
    </body>
  </notes>
  <listOfReactants>
    <speciesReference species="M_FAD_c" stoichiometry="1"/>
    <speciesReference species="M_PHFLN_c" stoichiometry="1"/>
  </listOfReactants>
  <listOfProducts>
```

```xml
    <speciesReference species="M_FADH2_c" stoichiometry="1"/>
    <speciesReference species="M_zCARO_c" stoichiometry="1"/>
  </listOfProducts>
  <kineticLaw>
    <math xmlns="http://www.w3.org/1998/Math/MathML">
      <ci> FLUX_VALUE </ci>
    </math>
    <listOfParameters>
      <parameter id="LOWER_BOUND" value="-1000" units="mmol_per_gDW_per_hr" constant="true"/>
      <parameter id="UPPER_BOUND" value="1000" units="mmol_per_gDW_per_hr" constant="true"/>
      <parameter id="FLUX_VALUE" value="0" units="mmol_per_gDW_per_hr" constant="true"/>
      <parameter id="OBJECTIVE_COEFFICIENT" value="0" units="mmol_per_gDW_per_hr" constant="true"/>
    </listOfParameters>
  </kineticLaw>
</reaction>
<reaction id="R_R516" name="Neurosporene, hydrogen-donor:oxygen oxidoreductase" reversible="false">
  <notes>
    <body xmlns="http://www.w3.org/1999/xhtml">
      <p>GENE_ASSOCIATION: TT_P0066</p>
      <p>SUBSYSTEM: Carotenoid biosynthesis</p>
      <p>EC Number: 1.3.99.28</p>
      <p>Confidence Level: 1</p>
      <p>AUTHORS: Carotenoid metabolism</p>
      <p/>
    </body>
  </notes>
  <listOfReactants>
    <speciesReference species="M_FADH2_c" stoichiometry="1"/>
    <speciesReference species="M_O2_c" stoichiometry="1"/>
```

```
    <speciesReference species="M_NRSPENE_c" stoichiometry="1"/>
   </listOfReactants>
   <listOfProducts>
    <speciesReference species="M_H2O_c" stoichiometry="2"/>
    <speciesReference species="M_FAD_c" stoichiometry="1"/>
    <speciesReference species="M_LCPN_c" stoichiometry="1"/>
   </listOfProducts>
   <kineticLaw>
    <math xmlns="http://www.w3.org/1998/Math/MathML">
     <ci> FLUX_VALUE </ci>
    </math>
    <listOfParameters>
     <parameter id="LOWER_BOUND" value="0" units="mmol_per_gDW_per_hr" constant="true"/>
     <parameter id="UPPER_BOUND" value="1000" units="mmol_per_gDW_per_hr" constant="true"/>
     <parameter id="FLUX_VALUE" value="0" units="mmol_per_gDW_per_hr" constant="true"/>
     <parameter id="OBJECTIVE_COEFFICIENT" value="0" units="mmol_per_gDW_per_hr" constant="true"/>
    </listOfParameters>
   </kineticLaw>
  </reaction>
  <reaction id="R_R517" name="zeta-Carotene, hydrogen-donor:oxygen oxidoreductase" reversible="false">
   <notes>
    <body xmlns="http://www.w3.org/1999/xhtml">
     <p>GENE_ASSOCIATION: TT_P0066</p>
     <p>SUBSYSTEM: Carotenoid biosynthesis</p>
     <p>EC Number: 1.3.99.28</p>
     <p>Confidence Level: 1</p>
     <p>AUTHORS: Carotenoid metabolism</p>
     <p/>
    </body>
```

```xml
</notes>
<listOfReactants>
<speciesReference species="M_FADH2_c" stoichiometry="1"/>
<speciesReference species="M_O2_c" stoichiometry="1"/>
<speciesReference species="M_zCARO_c" stoichiometry="1"/>
</listOfReactants>
<listOfProducts>
<speciesReference species="M_H2O_c" stoichiometry="2"/>
<speciesReference species="M_FAD_c" stoichiometry="1"/>
<speciesReference species="M_NRSPENE_c" stoichiometry="1"/>
</listOfProducts>
<kineticLaw>
<math xmlns="http://www.w3.org/1998/Math/MathML">
<ci> FLUX_VALUE </ci>
</math>
<listOfParameters>
<parameter id="LOWER_BOUND" value="0" units="mmol_per_gDW_per_hr" constant="true"/>
<parameter id="UPPER_BOUND" value="1000" units="mmol_per_gDW_per_hr" constant="true"/>
<parameter id="FLUX_VALUE" value="0" units="mmol_per_gDW_per_hr" constant="true"/>
<parameter id="OBJECTIVE_COEFFICIENT" value="0" units="mmol_per_gDW_per_hr" constant="true"/>
</listOfParameters>
</kineticLaw>
</reaction>
<reaction id="R_R518" name="ethylnitronate:oxygen 2-oxidoreductase (nitrite-forming)" reversible="false">
<notes>
<body xmlns="http://www.w3.org/1999/xhtml">
<p>GENE_ASSOCIATION: TTC1901</p>
<p>SUBSYSTEM: Nitrogen metabolism</p>
<p>EC Number: 1.13.12.16</p>
```

```
      <p>Confidence Level: 1</p>
      <p>AUTHORS: Energy and cofactors metabolism</p>
      <p/>
    </body>
  </notes>
  <listOfReactants>
    <speciesReference species="M_O2_c" stoichiometry="1"/>
    <speciesReference species="M_FMNH2_c" stoichiometry="1"/>
    <speciesReference species="M_ENITRN_c" stoichiometry="1"/>
  </listOfReactants>
  <listOfProducts>
    <speciesReference species="M_H2O_c" stoichiometry="1"/>
    <speciesReference species="M_AALD_c" stoichiometry="1"/>
    <speciesReference species="M_FMN_c" stoichiometry="1"/>
    <speciesReference species="M_NO2_c" stoichiometry="1"/>
  </listOfProducts>
  <kineticLaw>
    <math xmlns="http://www.w3.org/1998/Math/MathML">
      <ci> FLUX_VALUE </ci>
    </math>
    <listOfParameters>
      <parameter id="LOWER_BOUND" value="0" units="mmol_per_gDW_per_hr" constant="true"/>
      <parameter id="UPPER_BOUND" value="1000" units="mmol_per_gDW_per_hr" constant="true"/>
      <parameter id="FLUX_VALUE" value="0" units="mmol_per_gDW_per_hr" constant="true"/>
      <parameter id="OBJECTIVE_COEFFICIENT" value="0" units="mmol_per_gDW_per_hr" constant="true"/>
    </listOfParameters>
  </kineticLaw>
</reaction>
<reaction id="R_R519" name="Ammonia:ferredoxin oxidoreductase" reversible="true">
```

```
<notes>
  <body xmlns="http://www.w3.org/1999/xhtml">
    <p>GENE_ASSOCIATION: TTC0313</p>
    <p>SUBSYSTEM: Nitrogen metabolism</p>
    <p>EC Number: 1.7.7.1</p>
    <p>Confidence Level: 1</p>
    <p>AUTHORS: Energy and cofactors metabolism</p>
    <p/>
  </body>
</notes>
<listOfReactants>
  <speciesReference species="M_H_c" stoichiometry="7"/>
  <speciesReference species="M_R_FRDX_c" stoichiometry="6"/>
  <speciesReference species="M_NO2_c" stoichiometry="1"/>
</listOfReactants>
<listOfProducts>
  <speciesReference species="M_H2O_c" stoichiometry="2"/>
  <speciesReference species="M_NH4_c" stoichiometry="1"/>
  <speciesReference species="M_O_FRDX_c" stoichiometry="6"/>
</listOfProducts>
<kineticLaw>
  <math xmlns="http://www.w3.org/1998/Math/MathML">
    <ci> FLUX_VALUE </ci>
  </math>
  <listOfParameters>
    <parameter id="LOWER_BOUND" value="-1000" units="mmol_per_gDW_per_hr" constant="true"/>
    <parameter id="UPPER_BOUND" value="1000" units="mmol_per_gDW_per_hr" constant="true"/>
    <parameter id="FLUX_VALUE" value="0" units="mmol_per_gDW_per_hr" constant="true"/>
    <parameter id="OBJECTIVE_COEFFICIENT" value="0" units="mmol_per_gDW_per_hr" constant="true"/>
```

```
    </listOfParameters>
  </kineticLaw>
</reaction>
<reaction id="R_R520" name="L-Glutamine amidohydrolase" reversible="false">
  <notes>
    <body xmlns="http://www.w3.org/1999/xhtml">
      <p>GENE_ASSOCIATION: (TTC1104 or TTC0282 or (TTC0247 or TTC1706))</p>
      <p>SUBSYSTEM: Glutamate Metabolism</p>
      <p>EC Number: 1.4.1.13 or 6.3.5.4 or 6.3.5.5</p>
      <p>Confidence Level: 1</p>
      <p>AUTHORS: Amino acid metabolism</p>
      <p/>
    </body>
  </notes>
  <listOfReactants>
    <speciesReference species="M_L_GLN_c" stoichiometry="1"/>
    <speciesReference species="M_H2O_c" stoichiometry="1"/>
  </listOfReactants>
  <listOfProducts>
    <speciesReference species="M_L_GLU_c" stoichiometry="1"/>
    <speciesReference species="M_NH4_c" stoichiometry="1"/>
  </listOfProducts>
  <kineticLaw>
    <math xmlns="http://www.w3.org/1998/Math/MathML">
      <ci> FLUX_VALUE </ci>
    </math>
    <listOfParameters>
      <parameter id="LOWER_BOUND" value="0" units="mmol_per_gDW_per_hr" constant="true"/>
      <parameter id="UPPER_BOUND" value="1000" units="mmol_per_gDW_per_hr" constant="true"/>
```

```
<parameter id="FLUX_VALUE" value="0" units="mmol_per_gDW_per_hr" constant="true"/>
<parameter id="OBJECTIVE_COEFFICIENT" value="0" units="mmol_per_gDW_per_hr" constant="true"/>
</listOfParameters>
</kineticLaw>
</reaction>
<reaction id="R_R521" name="L-aspartate:L-glutamine amido-ligase (AMP-forming)" reversible="true">
<notes>
<body xmlns="http://www.w3.org/1999/xhtml">
<p>GENE_ASSOCIATION: TTC0282</p>
<p>SUBSYSTEM: Alanine and Aspartate Metabolism</p>
<p>EC Number: 6.3.5.4</p>
<p>Confidence Level: 1</p>
<p>AUTHORS: Amino acid metabolism</p>
<p/>
</body>
</notes>
<listOfReactants>
<speciesReference species="M_L_ASP_c" stoichiometry="1"/>
<speciesReference species="M_L_GLN_c" stoichiometry="1"/>
<speciesReference species="M_ATP_c" stoichiometry="1"/>
<speciesReference species="M_H2O_c" stoichiometry="1"/>
</listOfReactants>
<listOfProducts>
<speciesReference species="M_L_GLU_c" stoichiometry="1"/>
<speciesReference species="M_L_ASN_c" stoichiometry="1"/>
<speciesReference species="M_PPi_c" stoichiometry="1"/>
<speciesReference species="M_H_c" stoichiometry="1"/>
<speciesReference species="M_AMP_c" stoichiometry="1"/>
</listOfProducts>
```

```
  <kineticLaw>
   <math xmlns="http://www.w3.org/1998/Math/MathML">
    <ci> FLUX_VALUE </ci>
   </math>
   <listOfParameters>
    <parameter id="LOWER_BOUND" value="-1000" units="mmol_per_gDW_per_hr" constant="true"/>
    <parameter id="UPPER_BOUND" value="1000" units="mmol_per_gDW_per_hr" constant="true"/>
    <parameter id="FLUX_VALUE" value="0" units="mmol_per_gDW_per_hr" constant="true"/>
    <parameter id="OBJECTIVE_COEFFICIENT" value="0" units="mmol_per_gDW_per_hr" constant="true"/>
   </listOfParameters>
  </kineticLaw>
 </reaction>
 <reaction id="R_R522" name="Sulfite:ferricytochrome-c oxidoreductase" reversible="false">
  <notes>
   <body xmlns="http://www.w3.org/1999/xhtml">
    <p>GENE_ASSOCIATION: (TTC1046 or TTC1650)</p>
    <p>SUBSYSTEM: Sulfur metabolism </p>
    <p>EC Number: 1.8.2.1</p>
    <p>Confidence Level: 1</p>
    <p>AUTHORS: Energy and cofactors metabolism</p>
    <p/>
   </body>
  </notes>
  <listOfReactants>
   <speciesReference species="M_H2O_c" stoichiometry="1"/>
   <speciesReference species="M_CYT_C3_c" stoichiometry="2"/>
   <speciesReference species="M_SO3_c" stoichiometry="1"/>
  </listOfReactants>
  <listOfProducts>
```

```
<speciesReference species="M_CYT_C2_c" stoichiometry="2"/>
<speciesReference species="M_SO4_c" stoichiometry="1"/>
</listOfProducts>
<kineticLaw>
<math xmlns="http://www.w3.org/1998/Math/MathML">
<ci> FLUX_VALUE </ci>
</math>
<listOfParameters>
<parameter id="LOWER_BOUND" value="0" units="mmol_per_gDW_per_hr" constant="true"/>
<parameter id="UPPER_BOUND" value="1000" units="mmol_per_gDW_per_hr" constant="true"/>
<parameter id="FLUX_VALUE" value="0" units="mmol_per_gDW_per_hr" constant="true"/>
<parameter id="OBJECTIVE_COEFFICIENT" value="0" units="mmol_per_gDW_per_hr" constant="true"/>
</listOfParameters>
</kineticLaw>
</reaction>
<reaction id="R_R523" name="adenosine 3&apos;,5&apos;-bisphosphate,sulfite:oxidized-thioredoxin oxidoreductase
(3&apos;-phosphoadenosine-5&apos;-phosphosulfate -forming)" reversible="false">
<notes>
<body xmlns="http://www.w3.org/1999/xhtml">
<p>GENE_ASSOCIATION: (TTC0310 or TTC0961)</p>
<p>SUBSYSTEM: Sulfur metabolism </p>
<p>EC Number: 1.8.4.8</p>
<p>Confidence Level: 1</p>
<p>AUTHORS: Energy and cofactors metabolism</p>
<p/>
</body>
</notes>
<listOfReactants>
<speciesReference species="M_R_TRED_c" stoichiometry="1"/>
```

```xml
<speciesReference species="M_PAPS_c" stoichiometry="1"/>
</listOfReactants>
<listOfProducts>
<speciesReference species="M_H_c" stoichiometry="2"/>
<speciesReference species="M_O_TRED_c" stoichiometry="1"/>
<speciesReference species="M_SO3_c" stoichiometry="1"/>
<speciesReference species="M_PAP_c" stoichiometry="1"/>
</listOfProducts>
<kineticLaw>
<math xmlns="http://www.w3.org/1998/Math/MathML">
<ci> FLUX_VALUE </ci>
</math>
<listOfParameters>
<parameter id="LOWER_BOUND" value="0" units="mmol_per_gDW_per_hr" constant="true"/>
<parameter id="UPPER_BOUND" value="1000" units="mmol_per_gDW_per_hr" constant="true"/>
<parameter id="FLUX_VALUE" value="0" units="mmol_per_gDW_per_hr" constant="true"/>
<parameter id="OBJECTIVE_COEFFICIENT" value="0" units="mmol_per_gDW_per_hr" constant="true"/>
</listOfParameters>
</kineticLaw>
</reaction>
<reaction id="R_R524" name="Cystathionine L-homocysteine-lyase (deaminating)" reversible="false">
<notes>
<body xmlns="http://www.w3.org/1999/xhtml">
<p>GENE_ASSOCIATION: TTC1256</p>
<p>SUBSYSTEM: Sulfur metabolism </p>
<p>EC Number: 4.4.1.8</p>
<p>Confidence Level: 1</p>
<p>AUTHORS: Energy and cofactors metabolism</p>
<p/>
```

```
</body>
</notes>
<listOfReactants>
<speciesReference species="M_H2O_c" stoichiometry="1"/>
<speciesReference species="M_CYSTHN_c" stoichiometry="1"/>
</listOfReactants>
<listOfProducts>
<speciesReference species="M_PYR_c" stoichiometry="1"/>
<speciesReference species="M_NH4_c" stoichiometry="1"/>
<speciesReference species="M_L_HCYS_c" stoichiometry="1"/>
</listOfProducts>
<kineticLaw>
<math xmlns="http://www.w3.org/1998/Math/MathML">
<ci> FLUX_VALUE </ci>
</math>
<listOfParameters>
<parameter id="LOWER_BOUND" value="0" units="mmol_per_gDW_per_hr" constant="true"/>
<parameter id="UPPER_BOUND" value="1000" units="mmol_per_gDW_per_hr" constant="true"/>
<parameter id="FLUX_VALUE" value="0" units="mmol_per_gDW_per_hr" constant="true"/>
<parameter id="OBJECTIVE_COEFFICIENT" value="0" units="mmol_per_gDW_per_hr" constant="true"/>
</listOfParameters>
</kineticLaw>
</reaction>
<reaction id="R_R525" name="5,10-methylenetetrahydromethanopterin:glycine hydroxymethyltransferase"
reversible="false">
<notes>
<body xmlns="http://www.w3.org/1999/xhtml">
<p>GENE_ASSOCIATION: TTC1160</p>
<p>SUBSYSTEM: Methane metabolism</p>
```

```xml
      <p>EC Number: 2.1.2.1</p>
      <p>Confidence Level: 1</p>
      <p>AUTHORS: Carbohydrate metabolism</p>
      <p/>
    </body>
  </notes>
  <listOfReactants>
    <speciesReference species="M_L_SER_c" stoichiometry="1"/>
    <speciesReference species="M_H4MPT_c" stoichiometry="1"/>
  </listOfReactants>
  <listOfProducts>
    <speciesReference species="M_L_GLY_c" stoichiometry="1"/>
    <speciesReference species="M_H2O_c" stoichiometry="1"/>
    <speciesReference species="M_METHMPTR_c" stoichiometry="1"/>
  </listOfProducts>
  <kineticLaw>
    <math xmlns="http://www.w3.org/1998/Math/MathML">
      <ci> FLUX_VALUE </ci>
    </math>
    <listOfParameters>
      <parameter id="LOWER_BOUND" value="0" units="mmol_per_gDW_per_hr" constant="true"/>
      <parameter id="UPPER_BOUND" value="1000" units="mmol_per_gDW_per_hr" constant="true"/>
      <parameter id="FLUX_VALUE" value="0" units="mmol_per_gDW_per_hr" constant="true"/>
      <parameter id="OBJECTIVE_COEFFICIENT" value="0" units="mmol_per_gDW_per_hr" constant="true"/>
    </listOfParameters>
  </kineticLaw>
</reaction>
<reaction id="R_R526" name="(R)-2-phospho-3-sulfolactate phosphohydrolase" reversible="true">
  <notes>
```

```xml
<body xmlns="http://www.w3.org/1999/xhtml">

<p>GENE_ASSOCIATION: TTC1073</p>

<p>SUBSYSTEM: Methane metabolism</p>

<p>EC Number: 3.1.3.71</p>

<p>Confidence Level: 1</p>

<p>AUTHORS: Carbohydrate metabolism</p>

<p/>

</body>

</notes>

<listOfReactants>

<speciesReference species="M_H2O_c" stoichiometry="1"/>

<speciesReference species="M_PSULFLAC_c" stoichiometry="1"/>

</listOfReactants>

<listOfProducts>

<speciesReference species="M_Pi_c" stoichiometry="1"/>

<speciesReference species="M_SULFLAC_c" stoichiometry="1"/>

</listOfProducts>

<kineticLaw>

<math xmlns="http://www.w3.org/1998/Math/MathML">

<ci> FLUX_VALUE </ci>

</math>

<listOfParameters>

<parameter id="LOWER_BOUND" value="-1000" units="mmol_per_gDW_per_hr" constant="true"/>

<parameter id="UPPER_BOUND" value="1000" units="mmol_per_gDW_per_hr" constant="true"/>

<parameter id="FLUX_VALUE" value="0" units="mmol_per_gDW_per_hr" constant="true"/>

<parameter id="OBJECTIVE_COEFFICIENT" value="0" units="mmol_per_gDW_per_hr" constant="true"/>

</listOfParameters>

</kineticLaw>

</reaction>
```

```xml
<reaction id="R_R527" name="Uroporphyrinogen I carboxy-lyase" reversible="true">
 <notes>
  <body xmlns="http://www.w3.org/1999/xhtml">
   <p>GENE_ASSOCIATION: TTC0232</p>
   <p>SUBSYSTEM: Cofactor and Prosthetic Group Biosynthesis</p>
   <p>EC Number: 4.1.1.37</p>
   <p>Confidence Level: 1</p>
   <p>AUTHORS: Energy and cofactors metabolism</p>
   <p/>
  </body>
 </notes>
 <listOfReactants>
  <speciesReference species="M_UPPHYRGN_I_c" stoichiometry="1"/>
 </listOfReactants>
 <listOfProducts>
  <speciesReference species="M_H_c" stoichiometry="4"/>
  <speciesReference species="M_CO2_c" stoichiometry="4"/>
  <speciesReference species="M_CPPHYRGN_I_c" stoichiometry="1"/>
 </listOfProducts>
 <kineticLaw>
  <math xmlns="http://www.w3.org/1998/Math/MathML">
   <ci> FLUX_VALUE </ci>
  </math>
  <listOfParameters>
   <parameter id="LOWER_BOUND" value="-1000" units="mmol_per_gDW_per_hr" constant="true"/>
   <parameter id="UPPER_BOUND" value="1000" units="mmol_per_gDW_per_hr" constant="true"/>
   <parameter id="FLUX_VALUE" value="0" units="mmol_per_gDW_per_hr" constant="true"/>
   <parameter id="OBJECTIVE_COEFFICIENT" value="0" units="mmol_per_gDW_per_hr" constant="true"/>
  </listOfParameters>
```

```xml
  </kineticLaw>
</reaction>
<reaction id="R_R528" name="Uroporphyrinogen-III carboxy-lyase" reversible="false">
  <notes>
    <body xmlns="http://www.w3.org/1999/xhtml">
      <p>GENE_ASSOCIATION: TTC0232</p>
      <p>SUBSYSTEM: Cofactor and Prosthetic Group Biosynthesis</p>
      <p>EC Number: 4.1.1.37</p>
      <p>Confidence Level: 1</p>
      <p>AUTHORS: Energy and cofactors metabolism</p>
      <p/>
    </body>
  </notes>
  <listOfReactants>
    <speciesReference species="M_H_c" stoichiometry="4"/>
    <speciesReference species="M_UPPHYRGN_III_c" stoichiometry="1"/>
  </listOfReactants>
  <listOfProducts>
    <speciesReference species="M_CO2_c" stoichiometry="4"/>
    <speciesReference species="M_CPPPHYRGN_c" stoichiometry="1"/>
  </listOfProducts>
  <kineticLaw>
    <math xmlns="http://www.w3.org/1998/Math/MathML">
      <ci> FLUX_VALUE </ci>
    </math>
    <listOfParameters>
      <parameter id="LOWER_BOUND" value="0" units="mmol_per_gDW_per_hr" constant="true"/>
      <parameter id="UPPER_BOUND" value="1000" units="mmol_per_gDW_per_hr" constant="true"/>
      <parameter id="FLUX_VALUE" value="0" units="mmol_per_gDW_per_hr" constant="true"/>
```

```
      <parameter id="OBJECTIVE_COEFFICIENT" value="0" units="mmol_per_gDW_per_hr" constant="true"/>
    </listOfParameters>
  </kineticLaw>
</reaction>
<reaction id="R_R529" name="5-aminolevulinate hydro-lyase (adding 5-aminolevulinate and cyclizing" reversible="false">
  <notes>
    <body xmlns="http://www.w3.org/1999/xhtml">
      <p>GENE_ASSOCIATION: TTC1234</p>
      <p>SUBSYSTEM: Cofactor and Prosthetic Group Biosynthesis</p>
      <p>EC Number: 4.2.1.24</p>
      <p>Confidence Level: 1</p>
      <p>AUTHORS: Energy and cofactors metabolism</p>
      <p/>
    </body>
  </notes>
  <listOfReactants>
    <speciesReference species="M_ALEVU_c" stoichiometry="2"/>
  </listOfReactants>
  <listOfProducts>
    <speciesReference species="M_H2O_c" stoichiometry="2"/>
    <speciesReference species="M_H_c" stoichiometry="1"/>
    <speciesReference species="M_PPBLGN_c" stoichiometry="1"/>
  </listOfProducts>
  <kineticLaw>
    <math xmlns="http://www.w3.org/1998/Math/MathML">
      <ci> FLUX_VALUE </ci>
    </math>
    <listOfParameters>
      <parameter id="LOWER_BOUND" value="0" units="mmol_per_gDW_per_hr" constant="true"/>
```

```xml
<parameter id="UPPER_BOUND" value="1000" units="mmol_per_gDW_per_hr" constant="true"/>

<parameter id="FLUX_VALUE" value="0" units="mmol_per_gDW_per_hr" constant="true"/>

<parameter id="OBJECTIVE_COEFFICIENT" value="0" units="mmol_per_gDW_per_hr" constant="true"/>

</listOfParameters>

</kineticLaw>

</reaction>

<reaction id="R_R530" name="porphobilinogen:(4-[2-carboxyethyl]-3-[carboxymethyl]pyrrol-2-yl)methyltransferase (hydrolysing)" reversible="false">

<notes>

<body xmlns="http://www.w3.org/1999/xhtml">

<p>GENE_ASSOCIATION: TTC1638</p>

<p>SUBSYSTEM: Cofactor and Prosthetic Group Biosynthesis</p>

<p>EC Number: 2.5.1.61</p>

<p>Confidence Level: 1</p>

<p>AUTHORS: Energy and cofactors metabolism</p>

<p/>

</body>

</notes>

<listOfReactants>

<speciesReference species="M_H2O_c" stoichiometry="1"/>

<speciesReference species="M_PPBLGN_c" stoichiometry="4"/>

</listOfReactants>

<listOfProducts>

<speciesReference species="M_NH4_c" stoichiometry="4"/>

<speciesReference species="M_HY_MBILN_c" stoichiometry="1"/>

</listOfProducts>

<kineticLaw>

<math xmlns="http://www.w3.org/1998/Math/MathML">

<ci> FLUX_VALUE </ci>
```

```
      </math>
      <listOfParameters>
        <parameter id="LOWER_BOUND" value="0" units="mmol_per_gDW_per_hr" constant="true"/>
        <parameter id="UPPER_BOUND" value="1000" units="mmol_per_gDW_per_hr" constant="true"/>
        <parameter id="FLUX_VALUE" value="0" units="mmol_per_gDW_per_hr" constant="true"/>
        <parameter id="OBJECTIVE_COEFFICIENT" value="0" units="mmol_per_gDW_per_hr" constant="true"/>
      </listOfParameters>
    </kineticLaw>
  </reaction>
  <reaction id="R_R531" name="Hydroxymethylbilane hydro-lyase(cyclizing)" reversible="false">
    <notes>
      <body xmlns="http://www.w3.org/1999/xhtml">
        <p>GENE_ASSOCIATION: (TTC0312 or TTC1143)</p>
        <p>SUBSYSTEM: Cofactor and Prosthetic Group Biosynthesis</p>
        <p>EC Number: 4.2.1.75</p>
        <p>Confidence Level: 1</p>
        <p>AUTHORS: Energy and cofactors metabolism</p>
        <p/>
      </body>
    </notes>
    <listOfReactants>
      <speciesReference species="M_HY_MBILN_c" stoichiometry="1"/>
    </listOfReactants>
    <listOfProducts>
      <speciesReference species="M_H2O_c" stoichiometry="1"/>
      <speciesReference species="M_UPPHYRGN_III_c" stoichiometry="1"/>
    </listOfProducts>
    <kineticLaw>
      <math xmlns="http://www.w3.org/1998/Math/MathML">
```

```
<ci> FLUX_VALUE </ci>
</math>
<listOfParameters>
<parameter id="LOWER_BOUND" value="0" units="mmol_per_gDW_per_hr" constant="true"/>
<parameter id="UPPER_BOUND" value="1000" units="mmol_per_gDW_per_hr" constant="true"/>
<parameter id="FLUX_VALUE" value="0" units="mmol_per_gDW_per_hr" constant="true"/>
<parameter id="OBJECTIVE_COEFFICIENT" value="0" units="mmol_per_gDW_per_hr" constant="true"/>
</listOfParameters>
</kineticLaw>
</reaction>
<reaction id="R_R532" name="coproporphyrinogen-III:S-adenosyl-L-methionine oxidoreductase(decarboxylating)" reversible="false">
<notes>
<body xmlns="http://www.w3.org/1999/xhtml">
<p>GENE_ASSOCIATION: TTC0123</p>
<p>SUBSYSTEM: Cofactor and Prosthetic Group Biosynthesis</p>
<p>EC Number: 1.3.99.22</p>
<p>Confidence Level: 1</p>
<p>AUTHORS: Energy and cofactors metabolism</p>
<p/>
</body>
</notes>
<listOfReactants>
<speciesReference species="M_SAM_c" stoichiometry="2"/>
<speciesReference species="M_CPPPHYRGN_c" stoichiometry="1"/>
</listOfReactants>
<listOfProducts>
<speciesReference species="M_L_MET_c" stoichiometry="2"/>
<speciesReference species="M_CO2_c" stoichiometry="2"/>
```

```
    <speciesReference species="M_dADNS_c" stoichiometry="2"/>
    <speciesReference species="M_PPPHYRGN_c" stoichiometry="1"/>
  </listOfProducts>
  <kineticLaw>
    <math xmlns="http://www.w3.org/1998/Math/MathML">
      <ci> FLUX_VALUE </ci>
    </math>
    <listOfParameters>
      <parameter id="LOWER_BOUND" value="0" units="mmol_per_gDW_per_hr" constant="true"/>
      <parameter id="UPPER_BOUND" value="1000" units="mmol_per_gDW_per_hr" constant="true"/>
      <parameter id="FLUX_VALUE" value="0" units="mmol_per_gDW_per_hr" constant="true"/>
      <parameter id="OBJECTIVE_COEFFICIENT" value="0" units="mmol_per_gDW_per_hr" constant="true"/>
    </listOfParameters>
  </kineticLaw>
</reaction>
<reaction id="R_R533" name="protoporphyrinogen-IX:oxygen oxidoreductase" reversible="false">
  <notes>
    <body xmlns="http://www.w3.org/1999/xhtml">
      <p>GENE_ASSOCIATION: TTC0230</p>
      <p>SUBSYSTEM: Cofactor and Prosthetic Group Biosynthesis</p>
      <p>EC Number: 1.3.3.4</p>
      <p>Confidence Level: 1</p>
      <p>AUTHORS: Energy and cofactors metabolism</p>
      <p/>
    </body>
  </notes>
  <listOfReactants>
    <speciesReference species="M_O2_c" stoichiometry="3"/>
    <speciesReference species="M_PPPHYRGN_c" stoichiometry="1"/>
```

```
  </listOfReactants>
  <listOfProducts>
    <speciesReference species="M_H2O2_c" stoichiometry="3"/>
    <speciesReference species="M_PPPHYR_c" stoichiometry="1"/>
  </listOfProducts>
  <kineticLaw>
    <math xmlns="http://www.w3.org/1998/Math/MathML">
      <ci> FLUX_VALUE </ci>
    </math>
    <listOfParameters>
      <parameter id="LOWER_BOUND" value="0" units="mmol_per_gDW_per_hr" constant="true"/>
      <parameter id="UPPER_BOUND" value="1000" units="mmol_per_gDW_per_hr" constant="true"/>
      <parameter id="FLUX_VALUE" value="0" units="mmol_per_gDW_per_hr" constant="true"/>
      <parameter id="OBJECTIVE_COEFFICIENT" value="0" units="mmol_per_gDW_per_hr" constant="true"/>
    </listOfParameters>
  </kineticLaw>
</reaction>
<reaction id="R_R534" name="protoheme ferro-lyase (protoporphyrin-forming)" reversible="false">
  <notes>
    <body xmlns="http://www.w3.org/1999/xhtml">
      <p>GENE_ASSOCIATION: TTC0231</p>
      <p>SUBSYSTEM: Cofactor and Prosthetic Group Biosynthesis</p>
      <p>EC Number: 4.99.1.1</p>
      <p>Confidence Level: 1</p>
      <p>AUTHORS: Energy and cofactors metabolism</p>
      <p/>
    </body>
  </notes>
  <listOfReactants>
```

```
    <speciesReference species="M_PPPHYR_c" stoichiometry="1"/>
    <speciesReference species="M_Fe2_c" stoichiometry="1"/>
  </listOfReactants>
  <listOfProducts>
    <speciesReference species="M_H_c" stoichiometry="2"/>
    <speciesReference species="M_HEME_c" stoichiometry="1"/>
  </listOfProducts>
  <kineticLaw>
    <math xmlns="http://www.w3.org/1998/Math/MathML">
      <ci> FLUX_VALUE </ci>
    </math>
    <listOfParameters>
      <parameter id="LOWER_BOUND" value="0" units="mmol_per_gDW_per_hr" constant="true"/>
      <parameter id="UPPER_BOUND" value="1000" units="mmol_per_gDW_per_hr" constant="true"/>
      <parameter id="FLUX_VALUE" value="0" units="mmol_per_gDW_per_hr" constant="true"/>
      <parameter id="OBJECTIVE_COEFFICIENT" value="0" units="mmol_per_gDW_per_hr" constant="true"/>
    </listOfParameters>
  </kineticLaw>
</reaction>
<reaction id="R_R535" name="(S)-4-Amino-5-oxopentanoate 4,5-aminomutase" reversible="true">
  <notes>
    <body xmlns="http://www.w3.org/1999/xhtml">
      <p>GENE_ASSOCIATION: TTC0564</p>
      <p>SUBSYSTEM: Cofactor and Prosthetic Group Biosynthesis</p>
      <p>EC Number: 5.4.3.8</p>
      <p>Confidence Level: 1</p>
      <p>AUTHORS: Energy and cofactors metabolism</p>
      <p/>
    </body>
```

```
  </notes>
  <listOfReactants>
    <speciesReference species="M_GLU_SA_c" stoichiometry="1"/>
  </listOfReactants>
  <listOfProducts>
    <speciesReference species="M_ALEVU_c" stoichiometry="1"/>
  </listOfProducts>
  <kineticLaw>
    <math xmlns="http://www.w3.org/1998/Math/MathML">
      <ci> FLUX_VALUE </ci>
    </math>
    <listOfParameters>
      <parameter id="LOWER_BOUND" value="-1000" units="mmol_per_gDW_per_hr" constant="true"/>
      <parameter id="UPPER_BOUND" value="1000" units="mmol_per_gDW_per_hr" constant="true"/>
      <parameter id="FLUX_VALUE" value="0" units="mmol_per_gDW_per_hr" constant="true"/>
      <parameter id="OBJECTIVE_COEFFICIENT" value="0" units="mmol_per_gDW_per_hr" constant="true"/>
    </listOfParameters>
  </kineticLaw>
</reaction>
<reaction id="R_R536" name="S-adenosyl-L-methionine:uroporphyrin-III C-methyltransferase" reversible="false">
  <notes>
    <body xmlns="http://www.w3.org/1999/xhtml">
      <p>GENE_ASSOCIATION: (TTC0308 or TT_P0017)</p>
      <p>SUBSYSTEM: Cofactor and Prosthetic Group Biosynthesis</p>
      <p>EC Number: 2.1.1.107</p>
      <p>Confidence Level: 1</p>
      <p>AUTHORS: Energy and cofactors metabolism</p>
      <p/>
    </body>
```

```xml
</notes>
<listOfReactants>
<speciesReference species="M_SAM_c" stoichiometry="2"/>
<speciesReference species="M_UPPHYRGN_III_c" stoichiometry="1"/>
</listOfReactants>
<listOfProducts>
<speciesReference species="M_H_c" stoichiometry="1"/>
<speciesReference species="M_SAHC_c" stoichiometry="2"/>
<speciesReference species="M_PRCRN_c" stoichiometry="1"/>
</listOfProducts>
<kineticLaw>
<math xmlns="http://www.w3.org/1998/Math/MathML">
<ci> FLUX_VALUE </ci>
</math>
<listOfParameters>
<parameter id="LOWER_BOUND" value="0" units="mmol_per_gDW_per_hr" constant="true"/>
<parameter id="UPPER_BOUND" value="1000" units="mmol_per_gDW_per_hr" constant="true"/>
<parameter id="FLUX_VALUE" value="0" units="mmol_per_gDW_per_hr" constant="true"/>
<parameter id="OBJECTIVE_COEFFICIENT" value="0" units="mmol_per_gDW_per_hr" constant="true"/>
</listOfParameters>
</kineticLaw>
</reaction>
<reaction id="R_R537" name="Magnesium-protoporphyrin IX chelatase" reversible="false">
<notes>
<body xmlns="http://www.w3.org/1999/xhtml">
<p>GENE_ASSOCIATION: TTC0268</p>
<p>SUBSYSTEM: Cofactor and Prosthetic Group Biosynthesis</p>
<p>EC Number: 6.6.1.1</p>
<p>Confidence Level: 1</p>
```

```
      <p>AUTHORS: Energy and cofactors metabolism</p>
      <p/>
    </body>
  </notes>
  <listOfReactants>
    <speciesReference species="M_ATP_c" stoichiometry="1"/>
    <speciesReference species="M_H2O_c" stoichiometry="1"/>
    <speciesReference species="M_PPPHYR_c" stoichiometry="1"/>
    <speciesReference species="M_Mg_c" stoichiometry="1"/>
  </listOfReactants>
  <listOfProducts>
    <speciesReference species="M_ADP_c" stoichiometry="1"/>
    <speciesReference species="M_Pi_c" stoichiometry="1"/>
    <speciesReference species="M_H_c" stoichiometry="3"/>
    <speciesReference species="M_Mg_PPPHYR_c" stoichiometry="1"/>
  </listOfProducts>
  <kineticLaw>
    <math xmlns="http://www.w3.org/1998/Math/MathML">
      <ci> FLUX_VALUE </ci>
    </math>
    <listOfParameters>
      <parameter id="LOWER_BOUND" value="0" units="mmol_per_gDW_per_hr" constant="true"/>
      <parameter id="UPPER_BOUND" value="1000" units="mmol_per_gDW_per_hr" constant="true"/>
      <parameter id="FLUX_VALUE" value="0" units="mmol_per_gDW_per_hr" constant="true"/>
      <parameter id="OBJECTIVE_COEFFICIENT" value="0" units="mmol_per_gDW_per_hr" constant="true"/>
    </listOfParameters>
  </kineticLaw>
</reaction>
<reaction id="R_R538" name="protoheme IX farnesyltransferase" reversible="false">
```

```
<notes>
  <body xmlns="http://www.w3.org/1999/xhtml">
    <p>GENE_ASSOCIATION: </p>
    <p>SUBSYSTEM: Cofactor and Prosthetic Group Biosynthesis</p>
    <p>EC Number: 2.5.1.-</p>
    <p>Confidence Level: 1</p>
    <p>AUTHORS: Energy and cofactors metabolism</p>
    <p/>
  </body>
</notes>
<listOfReactants>
  <speciesReference species="M_H2O_c" stoichiometry="1"/>
  <speciesReference species="M_FAR_PPi_c" stoichiometry="1"/>
  <speciesReference species="M_HEME_c" stoichiometry="1"/>
</listOfReactants>
<listOfProducts>
  <speciesReference species="M_PPi_c" stoichiometry="1"/>
  <speciesReference species="M_HEMO_c" stoichiometry="1"/>
</listOfProducts>
<kineticLaw>
  <math xmlns="http://www.w3.org/1998/Math/MathML">
    <ci> FLUX_VALUE </ci>
  </math>
  <listOfParameters>
    <parameter id="LOWER_BOUND" value="0" units="mmol_per_gDW_per_hr" constant="true"/>
    <parameter id="UPPER_BOUND" value="1000" units="mmol_per_gDW_per_hr" constant="true"/>
    <parameter id="FLUX_VALUE" value="0" units="mmol_per_gDW_per_hr" constant="true"/>
    <parameter id="OBJECTIVE_COEFFICIENT" value="0" units="mmol_per_gDW_per_hr" constant="true"/>
  </listOfParameters>
```

```
  </kineticLaw>
</reaction>
<reaction id="R_R539" name="S-Adenosyl-L-methionine:uroporphyrin-III C-methyltransferase" reversible="false">
  <notes>
    <body xmlns="http://www.w3.org/1999/xhtml">
      <p>GENE_ASSOCIATION: TTC0311</p>
      <p>SUBSYSTEM: Cofactor and Prosthetic Group Biosynthesis</p>
      <p>EC Number: 4.99.1.4</p>
      <p>Confidence Level: 1</p>
      <p>AUTHORS: Energy and cofactors metabolism</p>
      <p/>
    </body>
  </notes>
  <listOfReactants>
    <speciesReference species="M_Fe2_c" stoichiometry="1"/>
    <speciesReference species="M_SRHYCLR_c" stoichiometry="1"/>
  </listOfReactants>
  <listOfProducts>
    <speciesReference species="M_H_c" stoichiometry="3"/>
    <speciesReference species="M_SHEME_c" stoichiometry="1"/>
  </listOfProducts>
  <kineticLaw>
    <math xmlns="http://www.w3.org/1998/Math/MathML">
      <ci> FLUX_VALUE </ci>
    </math>
    <listOfParameters>
      <parameter id="LOWER_BOUND" value="0" units="mmol_per_gDW_per_hr" constant="true"/>
      <parameter id="UPPER_BOUND" value="1000" units="mmol_per_gDW_per_hr" constant="true"/>
      <parameter id="FLUX_VALUE" value="0" units="mmol_per_gDW_per_hr" constant="true"/>
```

```
      <parameter id="OBJECTIVE_COEFFICIENT" value="0" units="mmol_per_gDW_per_hr" constant="true"/>
    </listOfParameters>
  </kineticLaw>
</reaction>
<reaction id="R_R540" name="S-Adenosyl-L-methionine:uroporphyrin-III C-methyltransferase" reversible="false">
  <notes>
    <body xmlns="http://www.w3.org/1999/xhtml">
      <p>GENE_ASSOCIATION: TTC0311</p>
      <p>SUBSYSTEM: Cofactor and Prosthetic Group Biosynthesis</p>
      <p>EC Number: 1.3.1.76</p>
      <p>Confidence Level: 1</p>
      <p>AUTHORS: Energy and cofactors metabolism</p>
      <p/>
    </body>
  </notes>
  <listOfReactants>
    <speciesReference species="M_NAD_c" stoichiometry="1"/>
    <speciesReference species="M_PRCRN_c" stoichiometry="1"/>
  </listOfReactants>
  <listOfProducts>
    <speciesReference species="M_H_c" stoichiometry="1"/>
    <speciesReference species="M_NADH_c" stoichiometry="1"/>
    <speciesReference species="M_SRHYCLR_c" stoichiometry="1"/>
  </listOfProducts>
  <kineticLaw>
    <math xmlns="http://www.w3.org/1998/Math/MathML">
      <ci> FLUX_VALUE </ci>
    </math>
    <listOfParameters>
```

```
<parameter id="LOWER_BOUND" value="0" units="mmol_per_gDW_per_hr" constant="true"/>
<parameter id="UPPER_BOUND" value="1000" units="mmol_per_gDW_per_hr" constant="true"/>
<parameter id="FLUX_VALUE" value="0" units="mmol_per_gDW_per_hr" constant="true"/>
<parameter id="OBJECTIVE_COEFFICIENT" value="0" units="mmol_per_gDW_per_hr" constant="true"/>
</listOfParameters>
</kineticLaw>
</reaction>
<reaction id="R_R541" name="S-adenosyl-L-methionine:precorrin-4 C20-methyltransferase" reversible="false">
<notes>
<body xmlns="http://www.w3.org/1999/xhtml">
<p>GENE_ASSOCIATION: TT_P0011</p>
<p>SUBSYSTEM: Cofactor and Prosthetic Group Biosynthesis</p>
<p>EC Number: 2.1.1.130</p>
<p>Confidence Level: 1</p>
<p>AUTHORS: Energy and cofactors metabolism</p>
<p/>
</body>
</notes>
<listOfReactants>
<speciesReference species="M_SAM_c" stoichiometry="1"/>
<speciesReference species="M_PRCRN_c" stoichiometry="1"/>
</listOfReactants>
<listOfProducts>
<speciesReference species="M_H_c" stoichiometry="1"/>
<speciesReference species="M_SAHC_c" stoichiometry="1"/>
<speciesReference species="M_PRCRN3A_c" stoichiometry="1"/>
</listOfProducts>
<kineticLaw>
<math xmlns="http://www.w3.org/1998/Math/MathML">
```

```
      <ci> FLUX_VALUE </ci>
    </math>
    <listOfParameters>
      <parameter id="LOWER_BOUND" value="0" units="mmol_per_gDW_per_hr" constant="true"/>
      <parameter id="UPPER_BOUND" value="1000" units="mmol_per_gDW_per_hr" constant="true"/>
      <parameter id="FLUX_VALUE" value="0" units="mmol_per_gDW_per_hr" constant="true"/>
      <parameter id="OBJECTIVE_COEFFICIENT" value="0" units="mmol_per_gDW_per_hr" constant="true"/>
    </listOfParameters>
  </kineticLaw>
</reaction>
<reaction id="R_R542" name="S-adenosyl-L-methionine:cobalt-factor-II C20-methyltransferase" reversible="false">
  <notes>
    <body xmlns="http://www.w3.org/1999/xhtml">
      <p>GENE_ASSOCIATION: TT_P0011</p>
      <p>SUBSYSTEM: Cofactor and Prosthetic Group Biosynthesis</p>
      <p>EC Number: 2.1.1.151</p>
      <p>Confidence Level: 1</p>
      <p>AUTHORS: Energy and cofactors metabolism</p>
      <p/>
    </body>
  </notes>
  <listOfReactants>
    <speciesReference species="M_SAM_c" stoichiometry="1"/>
    <speciesReference species="M_CoSRHYCLR_c" stoichiometry="1"/>
  </listOfReactants>
  <listOfProducts>
    <speciesReference species="M_SAHC_c" stoichiometry="1"/>
    <speciesReference species="M_CoFIII_c" stoichiometry="1"/>
  </listOfProducts>
```

```
  <kineticLaw>
    <math xmlns="http://www.w3.org/1998/Math/MathML">
      <ci> FLUX_VALUE </ci>
    </math>
    <listOfParameters>
      <parameter id="LOWER_BOUND" value="0" units="mmol_per_gDW_per_hr" constant="true"/>
      <parameter id="UPPER_BOUND" value="1000" units="mmol_per_gDW_per_hr" constant="true"/>
      <parameter id="FLUX_VALUE" value="0" units="mmol_per_gDW_per_hr" constant="true"/>
      <parameter id="OBJECTIVE_COEFFICIENT" value="0" units="mmol_per_gDW_per_hr" constant="true"/>
    </listOfParameters>
  </kineticLaw>
</reaction>
<reaction id="R_R543" name="precorrin-3B C17-methyltransferase" reversible="false">
  <notes>
    <body xmlns="http://www.w3.org/1999/xhtml">
      <p>GENE_ASSOCIATION: TT_P0013</p>
      <p>SUBSYSTEM: Cofactor and Prosthetic Group Biosynthesis</p>
      <p>EC Number: 2.1.1.131</p>
      <p>Confidence Level: 1</p>
      <p>AUTHORS: Energy and cofactors metabolism</p>
      <p/>
    </body>
  </notes>
  <listOfReactants>
    <speciesReference species="M_H_c" stoichiometry="1"/>
    <speciesReference species="M_SAM_c" stoichiometry="1"/>
    <speciesReference species="M_CoPRCRN3_c" stoichiometry="1"/>
  </listOfReactants>
  <listOfProducts>
```

```
<speciesReference species="M_SAHC_c" stoichiometry="1"/>
<speciesReference species="M_CoPRCRN4_c" stoichiometry="1"/>
</listOfProducts>
<kineticLaw>
<math xmlns="http://www.w3.org/1998/Math/MathML">
<ci> FLUX_VALUE </ci>
</math>
<listOfParameters>
<parameter id="LOWER_BOUND" value="0" units="mmol_per_gDW_per_hr" constant="true"/>
<parameter id="UPPER_BOUND" value="1000" units="mmol_per_gDW_per_hr" constant="true"/>
<parameter id="FLUX_VALUE" value="0" units="mmol_per_gDW_per_hr" constant="true"/>
<parameter id="OBJECTIVE_COEFFICIENT" value="0" units="mmol_per_gDW_per_hr" constant="true"/>
</listOfParameters>
</kineticLaw>
</reaction>
<reaction id="R_R544" name="S-adenosyl-L-methionine:precorrin-3B C17-methyltransferase" reversible="false">
<notes>
<body xmlns="http://www.w3.org/1999/xhtml">
<p>GENE_ASSOCIATION: TT_P0013</p>
<p>SUBSYSTEM: Cofactor and Prosthetic Group Biosynthesis</p>
<p>EC Number: 2.1.1.131</p>
<p>Confidence Level: 1</p>
<p>AUTHORS: Energy and cofactors metabolism</p>
<p/>
</body>
</notes>
<listOfReactants>
<speciesReference species="M_SAM_c" stoichiometry="1"/>
<speciesReference species="M_PRCRN3B_c" stoichiometry="1"/>
```

```
  </listOfReactants>
  <listOfProducts>
    <speciesReference species="M_H_c" stoichiometry="2"/>
    <speciesReference species="M_SAHC_c" stoichiometry="1"/>
    <speciesReference species="M_PRCRN4_c" stoichiometry="1"/>
  </listOfProducts>
  <kineticLaw>
    <math xmlns="http://www.w3.org/1998/Math/MathML">
      <ci> FLUX_VALUE </ci>
    </math>
    <listOfParameters>
      <parameter id="LOWER_BOUND" value="0" units="mmol_per_gDW_per_hr" constant="true"/>
      <parameter id="UPPER_BOUND" value="1000" units="mmol_per_gDW_per_hr" constant="true"/>
      <parameter id="FLUX_VALUE" value="0" units="mmol_per_gDW_per_hr" constant="true"/>
      <parameter id="OBJECTIVE_COEFFICIENT" value="0" units="mmol_per_gDW_per_hr" constant="true"/>
    </listOfParameters>
  </kineticLaw>
</reaction>
<reaction id="R_R545" name="S-adenosyl-L-methionine:precorrin-4 C11 methyltransferase" reversible="false">
  <notes>
    <body xmlns="http://www.w3.org/1999/xhtml">
      <p>GENE_ASSOCIATION: TT_P0012</p>
      <p>SUBSYSTEM: Cofactor and Prosthetic Group Biosynthesis</p>
      <p>EC Number: 2.1.1.133</p>
      <p>Confidence Level: 1</p>
      <p>AUTHORS: Energy and cofactors metabolism</p>
      <p/>
    </body>
  </notes>
```

```
  <listOfReactants>
    <speciesReference species="M_SAM_c" stoichiometry="1"/>
    <speciesReference species="M_PRCRN4_c" stoichiometry="1"/>
  </listOfReactants>
  <listOfProducts>
    <speciesReference species="M_H_c" stoichiometry="1"/>
    <speciesReference species="M_SAHC_c" stoichiometry="1"/>
    <speciesReference species="M_PRCRN5_c" stoichiometry="1"/>
  </listOfProducts>
  <kineticLaw>
    <math xmlns="http://www.w3.org/1998/Math/MathML">
      <ci> FLUX_VALUE </ci>
    </math>
    <listOfParameters>
      <parameter id="LOWER_BOUND" value="0" units="mmol_per_gDW_per_hr" constant="true"/>
      <parameter id="UPPER_BOUND" value="1000" units="mmol_per_gDW_per_hr" constant="true"/>
      <parameter id="FLUX_VALUE" value="0" units="mmol_per_gDW_per_hr" constant="true"/>
      <parameter id="OBJECTIVE_COEFFICIENT" value="0" units="mmol_per_gDW_per_hr" constant="true"/>
    </listOfParameters>
  </kineticLaw>
</reaction>
<reaction id="R_R546" name="cobalt-precorrin-4 methyltransferase" reversible="false">
  <notes>
    <body xmlns="http://www.w3.org/1999/xhtml">
      <p>GENE_ASSOCIATION: TT_P0012</p>
      <p>SUBSYSTEM: Cofactor and Prosthetic Group Biosynthesis</p>
      <p>EC Number: 2.1.1.133</p>
      <p>Confidence Level: 1</p>
      <p>AUTHORS: Energy and cofactors metabolism</p>
```

```xml
<p/>

</body>

</notes>

<listOfReactants>

<speciesReference species="M_SAM_c" stoichiometry="1"/>

<speciesReference species="M_CoPRCRN4_c" stoichiometry="1"/>

</listOfReactants>

<listOfProducts>

<speciesReference species="M_SAHC_c" stoichiometry="1"/>

<speciesReference species="M_CoPRCRN5A_c" stoichiometry="1"/>

</listOfProducts>

<kineticLaw>

<math xmlns="http://www.w3.org/1998/Math/MathML">

<ci> FLUX_VALUE </ci>

</math>

<listOfParameters>

<parameter id="LOWER_BOUND" value="0" units="mmol_per_gDW_per_hr" constant="true"/>

<parameter id="UPPER_BOUND" value="1000" units="mmol_per_gDW_per_hr" constant="true"/>

<parameter id="FLUX_VALUE" value="0" units="mmol_per_gDW_per_hr" constant="true"/>

<parameter id="OBJECTIVE_COEFFICIENT" value="0" units="mmol_per_gDW_per_hr" constant="true"/>

</listOfParameters>

</kineticLaw>

</reaction>

<reaction id="R_R547" name="S-Adenosyl-L-methionine:1-precorrin-6Y C5,15-methyltransferase (C-12-decarboxylating)" reversible="false">

<notes>

<body xmlns="http://www.w3.org/1999/xhtml">

<p>GENE_ASSOCIATION: TT_P0010</p>

<p>SUBSYSTEM: Cofactor and Prosthetic Group Biosynthesis</p>
```

```
      <p>EC Number: 2.1.1.132</p>
      <p>Confidence Level: 1</p>
      <p>AUTHORS: Energy and cofactors metabolism</p>
      <p/>
    </body>
  </notes>
  <listOfReactants>
    <speciesReference species="M_SAM_c" stoichiometry="2"/>
    <speciesReference species="M_PRCRN6B_c" stoichiometry="1"/>
  </listOfReactants>
  <listOfProducts>
    <speciesReference species="M_H_c" stoichiometry="2"/>
    <speciesReference species="M_CO2_c" stoichiometry="1"/>
    <speciesReference species="M_SAHC_c" stoichiometry="2"/>
    <speciesReference species="M_PRCRN8X_c" stoichiometry="1"/>
  </listOfProducts>
  <kineticLaw>
    <math xmlns="http://www.w3.org/1998/Math/MathML">
      <ci> FLUX_VALUE </ci>
    </math>
    <listOfParameters>
      <parameter id="LOWER_BOUND" value="0" units="mmol_per_gDW_per_hr" constant="true"/>
      <parameter id="UPPER_BOUND" value="1000" units="mmol_per_gDW_per_hr" constant="true"/>
      <parameter id="FLUX_VALUE" value="0" units="mmol_per_gDW_per_hr" constant="true"/>
      <parameter id="OBJECTIVE_COEFFICIENT" value="0" units="mmol_per_gDW_per_hr" constant="true"/>
    </listOfParameters>
  </kineticLaw>
</reaction>
<reaction id="R_R548" name="precorrin-8X methylmutase" reversible="false">
```

```
  <notes>
    <body xmlns="http://www.w3.org/1999/xhtml">
      <p>GENE_ASSOCIATION: TT_P0009</p>
      <p>SUBSYSTEM: Cofactor and Prosthetic Group Biosynthesis</p>
      <p>EC Number: 5.4.1.2</p>
      <p>Confidence Level: 1</p>
      <p>AUTHORS: Energy and cofactors metabolism</p>
      <p/>
    </body>
  </notes>
  <listOfReactants>
    <speciesReference species="M_PRCRN8X_c" stoichiometry="1"/>
  </listOfReactants>
  <listOfProducts>
    <speciesReference species="M_HOBYRNA_c" stoichiometry="1"/>
  </listOfProducts>
  <kineticLaw>
    <math xmlns="http://www.w3.org/1998/Math/MathML">
      <ci> FLUX_VALUE </ci>
    </math>
    <listOfParameters>
      <parameter id="LOWER_BOUND" value="0" units="mmol_per_gDW_per_hr" constant="true"/>
      <parameter id="UPPER_BOUND" value="1000" units="mmol_per_gDW_per_hr" constant="true"/>
      <parameter id="FLUX_VALUE" value="0" units="mmol_per_gDW_per_hr" constant="true"/>
      <parameter id="OBJECTIVE_COEFFICIENT" value="0" units="mmol_per_gDW_per_hr" constant="true"/>
    </listOfParameters>
  </kineticLaw>
</reaction>
<reaction id="R_R549" name="precorrin-8X methylmutase" reversible="false">
```

```xml
<notes>
  <body xmlns="http://www.w3.org/1999/xhtml">
    <p>GENE_ASSOCIATION: TT_P0009</p>
    <p>SUBSYSTEM: Cofactor and Prosthetic Group Biosynthesis</p>
    <p>EC Number: 5.4.1.2</p>
    <p>Confidence Level: 1</p>
    <p>AUTHORS: Energy and cofactors metabolism</p>
    <p/>
  </body>
</notes>
<listOfReactants>
  <speciesReference species="M_CoPRCRN8_c" stoichiometry="1"/>
</listOfReactants>
<listOfProducts>
  <speciesReference species="M_CBRYNA_c" stoichiometry="1"/>
</listOfProducts>
<kineticLaw>
  <math xmlns="http://www.w3.org/1998/Math/MathML">
    <ci> FLUX_VALUE </ci>
  </math>
  <listOfParameters>
    <parameter id="LOWER_BOUND" value="0" units="mmol_per_gDW_per_hr" constant="true"/>
    <parameter id="UPPER_BOUND" value="1000" units="mmol_per_gDW_per_hr" constant="true"/>
    <parameter id="FLUX_VALUE" value="0" units="mmol_per_gDW_per_hr" constant="true"/>
    <parameter id="OBJECTIVE_COEFFICIENT" value="0" units="mmol_per_gDW_per_hr" constant="true"/>
  </listOfParameters>
</kineticLaw>
</reaction>
<reaction id="R_R550" name="cobyrinic acid A,C-diamide synthase" reversible="false">
```

```
<notes>
  <body xmlns="http://www.w3.org/1999/xhtml">
    <p>GENE_ASSOCIATION: TT_P0001</p>
    <p>SUBSYSTEM: Cofactor and Prosthetic Group Biosynthesis</p>
    <p>EC Number: 6.3.5.9</p>
    <p>Confidence Level: 1</p>
    <p>AUTHORS: Energy and cofactors metabolism</p>
    <p/>
  </body>
</notes>
<listOfReactants>
  <speciesReference species="M_L_GLN_c" stoichiometry="2"/>
  <speciesReference species="M_ATP_c" stoichiometry="2"/>
  <speciesReference species="M_H2O_c" stoichiometry="2"/>
  <speciesReference species="M_HOBYRNA_c" stoichiometry="1"/>
</listOfReactants>
<listOfProducts>
  <speciesReference species="M_L_GLU_c" stoichiometry="2"/>
  <speciesReference species="M_ADP_c" stoichiometry="2"/>
  <speciesReference species="M_Pi_c" stoichiometry="2"/>
  <speciesReference species="M_H_c" stoichiometry="2"/>
  <speciesReference species="M_HOBYRNDA_c" stoichiometry="1"/>
</listOfProducts>
<kineticLaw>
  <math xmlns="http://www.w3.org/1998/Math/MathML">
    <ci> FLUX_VALUE </ci>
  </math>
  <listOfParameters>
    <parameter id="LOWER_BOUND" value="0" units="mmol_per_gDW_per_hr" constant="true"/>
```

```
      <parameter id="UPPER_BOUND" value="1000" units="mmol_per_gDW_per_hr" constant="true"/>
      <parameter id="FLUX_VALUE" value="0" units="mmol_per_gDW_per_hr" constant="true"/>
      <parameter id="OBJECTIVE_COEFFICIENT" value="0" units="mmol_per_gDW_per_hr" constant="true"/>
    </listOfParameters>
  </kineticLaw>
</reaction>
<reaction id="R_R551" name="cobyrinate:L-glutamine amido-ligase (ADP-forming)" reversible="false">
  <notes>
    <body xmlns="http://www.w3.org/1999/xhtml">
      <p>GENE_ASSOCIATION: TT_P0001</p>
      <p>SUBSYSTEM: Cofactor and Prosthetic Group Biosynthesis</p>
      <p>EC Number: 6.3.5.11</p>
      <p>Confidence Level: 1</p>
      <p>AUTHORS: Energy and cofactors metabolism</p>
      <p/>
    </body>
  </notes>
  <listOfReactants>
    <speciesReference species="M_L_GLN_c" stoichiometry="2"/>
    <speciesReference species="M_ATP_c" stoichiometry="2"/>
    <speciesReference species="M_H2O_c" stoichiometry="2"/>
    <speciesReference species="M_CBRYNA_c" stoichiometry="1"/>
  </listOfReactants>
  <listOfProducts>
    <speciesReference species="M_L_GLU_c" stoichiometry="2"/>
    <speciesReference species="M_ADP_c" stoichiometry="2"/>
    <speciesReference species="M_Pi_c" stoichiometry="2"/>
    <speciesReference species="M_H_c" stoichiometry="3"/>
    <speciesReference species="M_Co2ABYRDA_c" stoichiometry="1"/>
```

```xml
</listOfProducts>
<kineticLaw>
  <math xmlns="http://www.w3.org/1998/Math/MathML">
    <ci> FLUX_VALUE </ci>
  </math>
  <listOfParameters>
    <parameter id="LOWER_BOUND" value="0" units="mmol_per_gDW_per_hr" constant="true"/>
    <parameter id="UPPER_BOUND" value="1000" units="mmol_per_gDW_per_hr" constant="true"/>
    <parameter id="FLUX_VALUE" value="0" units="mmol_per_gDW_per_hr" constant="true"/>
    <parameter id="OBJECTIVE_COEFFICIENT" value="0" units="mmol_per_gDW_per_hr" constant="true"/>
  </listOfParameters>
</kineticLaw>
</reaction>
<reaction id="R_R552" name="adenosylcobyric acid synthase (glutamine-hydrolysing)" reversible="false">
<notes>
  <body xmlns="http://www.w3.org/1999/xhtml">
    <p>GENE_ASSOCIATION: TT_P0023</p>
    <p>SUBSYSTEM: Cofactor and Prosthetic Group Biosynthesis</p>
    <p>EC Number: 6.3.5.10</p>
    <p>Confidence Level: 1</p>
    <p>AUTHORS: Energy and cofactors metabolism</p>
    <p/>
  </body>
</notes>
<listOfReactants>
  <speciesReference species="M_L_GLN_c" stoichiometry="4"/>
  <speciesReference species="M_ATP_c" stoichiometry="4"/>
  <speciesReference species="M_H2O_c" stoichiometry="4"/>
  <speciesReference species="M_ADNS_CABYRDA_c" stoichiometry="1"/>
```

```
    </listOfReactants>
    <listOfProducts>
      <speciesReference species="M_L_GLU_c" stoichiometry="4"/>
      <speciesReference species="M_ADP_c" stoichiometry="4"/>
      <speciesReference species="M_Pi_c" stoichiometry="4"/>
      <speciesReference species="M_H_c" stoichiometry="4"/>
      <speciesReference species="M_ADNS_CABYRHA_c" stoichiometry="1"/>
    </listOfProducts>
    <kineticLaw>
      <math xmlns="http://www.w3.org/1998/Math/MathML">
        <ci> FLUX_VALUE </ci>
      </math>
      <listOfParameters>
        <parameter id="LOWER_BOUND" value="0" units="mmol_per_gDW_per_hr" constant="true"/>
        <parameter id="UPPER_BOUND" value="1000" units="mmol_per_gDW_per_hr" constant="true"/>
        <parameter id="FLUX_VALUE" value="0" units="mmol_per_gDW_per_hr" constant="true"/>
        <parameter id="OBJECTIVE_COEFFICIENT" value="0" units="mmol_per_gDW_per_hr" constant="true"/>
      </listOfParameters>
    </kineticLaw>
  </reaction>
  <reaction id="R_R553" name="Cobalt synthesis" reversible="false">
    <notes>
      <body xmlns="http://www.w3.org/1999/xhtml">
        <p>GENE_ASSOCIATION: </p>
        <p>SUBSYSTEM: Cofactor and Prosthetic Group Biosynthesis</p>
        <p>EC Number: 6.6.1.2</p>
        <p>Confidence Level: 1</p>
        <p>AUTHORS: Energy and cofactors metabolism</p>
        <p/>
```

```
    </body>
  </notes>
  <listOfReactants>
    <speciesReference species="M_ATP_c" stoichiometry="1"/>
    <speciesReference species="M_H2O_c" stoichiometry="1"/>
    <speciesReference species="M_HOBYRNDA_c" stoichiometry="1"/>
    <speciesReference species="M_CO_LPAREN_II_RPAREN__c" stoichiometry="1"/>
  </listOfReactants>
  <listOfProducts>
    <speciesReference species="M_ADP_c" stoichiometry="1"/>
    <speciesReference species="M_Pi_c" stoichiometry="1"/>
    <speciesReference species="M_H_c" stoichiometry="3"/>
    <speciesReference species="M_Co2ABYRDA_c" stoichiometry="1"/>
  </listOfProducts>
  <kineticLaw>
    <math xmlns="http://www.w3.org/1998/Math/MathML">
      <ci> FLUX_VALUE </ci>
    </math>
    <listOfParameters>
      <parameter id="LOWER_BOUND" value="0" units="mmol_per_gDW_per_hr" constant="true"/>
      <parameter id="UPPER_BOUND" value="1000" units="mmol_per_gDW_per_hr" constant="true"/>
      <parameter id="FLUX_VALUE" value="0" units="mmol_per_gDW_per_hr" constant="true"/>
      <parameter id="OBJECTIVE_COEFFICIENT" value="0" units="mmol_per_gDW_per_hr" constant="true"/>
    </listOfParameters>
  </kineticLaw>
</reaction>
<reaction id="R_R554" name="ATP:cob(I)yrinic acid-a,c-diamide Cobeta-adenosyltransferase" reversible="false">
  <notes>
    <body xmlns="http://www.w3.org/1999/xhtml">
```

```xml
      <p>GENE_ASSOCIATION: TT_P0002</p>
      <p>SUBSYSTEM: Cofactor and Prosthetic Group Biosynthesis</p>
      <p>EC Number: 2.5.1.17</p>
      <p>Confidence Level: 1</p>
      <p>AUTHORS: Energy and cofactors metabolism</p>
      <p/>
    </body>
  </notes>
  <listOfReactants>
    <speciesReference species="M_ATP_c" stoichiometry="1"/>
    <speciesReference species="M_CABYRDA_c" stoichiometry="1"/>
  </listOfReactants>
  <listOfProducts>
    <speciesReference species="M_TPi_c" stoichiometry="1"/>
    <speciesReference species="M_ADNS_CABYRDA_c" stoichiometry="1"/>
  </listOfProducts>
  <kineticLaw>
    <math xmlns="http://www.w3.org/1998/Math/MathML">
      <ci> FLUX_VALUE </ci>
    </math>
    <listOfParameters>
      <parameter id="LOWER_BOUND" value="0" units="mmol_per_gDW_per_hr" constant="true"/>
      <parameter id="UPPER_BOUND" value="1000" units="mmol_per_gDW_per_hr" constant="true"/>
      <parameter id="FLUX_VALUE" value="0" units="mmol_per_gDW_per_hr" constant="true"/>
      <parameter id="OBJECTIVE_COEFFICIENT" value="0" units="mmol_per_gDW_per_hr" constant="true"/>
    </listOfParameters>
  </kineticLaw>
</reaction>
<reaction id="R_R555" name="ATP:cobinamide Cobeta-adenosyltransferase" reversible="true">
```

```
<notes>
  <body xmlns="http://www.w3.org/1999/xhtml">
    <p>GENE_ASSOCIATION: TT_P0002</p>
    <p>SUBSYSTEM: Cofactor and Prosthetic Group Biosynthesis</p>
    <p>EC Number: 2.5.1.17</p>
    <p>Confidence Level: 1</p>
    <p>AUTHORS: Energy and cofactors metabolism</p>
    <p/>
  </body>
</notes>
<listOfReactants>
  <speciesReference species="M_ATP_c" stoichiometry="1"/>
  <speciesReference species="M_H_c" stoichiometry="1"/>
  <speciesReference species="M_CBAMD_c" stoichiometry="1"/>
</listOfReactants>
<listOfProducts>
  <speciesReference species="M_TPi_c" stoichiometry="1"/>
  <speciesReference species="M_ADNS_CBNA_c" stoichiometry="1"/>
</listOfProducts>
<kineticLaw>
  <math xmlns="http://www.w3.org/1998/Math/MathML">
    <ci> FLUX_VALUE </ci>
  </math>
  <listOfParameters>
    <parameter id="LOWER_BOUND" value="-1000" units="mmol_per_gDW_per_hr" constant="true"/>
    <parameter id="UPPER_BOUND" value="1000" units="mmol_per_gDW_per_hr" constant="true"/>
    <parameter id="FLUX_VALUE" value="0" units="mmol_per_gDW_per_hr" constant="true"/>
    <parameter id="OBJECTIVE_COEFFICIENT" value="0" units="mmol_per_gDW_per_hr" constant="true"/>
  </listOfParameters>
```

```
  </kineticLaw>
</reaction>
<reaction id="R_R556" name="adenosylcobyric acid:(R)-1-aminopropan-2-yl phosphate ligase" reversible="false">
  <notes>
    <body xmlns="http://www.w3.org/1999/xhtml">
      <p>GENE_ASSOCIATION: TT_P0019</p>
      <p>SUBSYSTEM: Cofactor and Prosthetic Group Biosynthesis</p>
      <p>EC Number: 6.3.1.10</p>
      <p>Confidence Level: 1</p>
      <p>AUTHORS: Energy and cofactors metabolism</p>
      <p/>
    </body>
  </notes>
  <listOfReactants>
    <speciesReference species="M_ATP_c" stoichiometry="1"/>
    <speciesReference species="M_ADNS_CABYRHA_c" stoichiometry="1"/>
    <speciesReference species="M_APRNPi_c" stoichiometry="1"/>
  </listOfReactants>
  <listOfProducts>
    <speciesReference species="M_ADP_c" stoichiometry="1"/>
    <speciesReference species="M_Pi_c" stoichiometry="1"/>
    <speciesReference species="M_ADNS_CBNAPi_c" stoichiometry="1"/>
  </listOfProducts>
  <kineticLaw>
    <math xmlns="http://www.w3.org/1998/Math/MathML">
      <ci> FLUX_VALUE </ci>
    </math>
    <listOfParameters>
      <parameter id="LOWER_BOUND" value="0" units="mmol_per_gDW_per_hr" constant="true"/>
```

```
      <parameter id="UPPER_BOUND" value="1000" units="mmol_per_gDW_per_hr" constant="true"/>
      <parameter id="FLUX_VALUE" value="0" units="mmol_per_gDW_per_hr" constant="true"/>
      <parameter id="OBJECTIVE_COEFFICIENT" value="0" units="mmol_per_gDW_per_hr" constant="true"/>
    </listOfParameters>
  </kineticLaw>
</reaction>
<reaction id="R_R557" name="cobalamin [5&apos;-phosphate] synthase" reversible="false">
  <notes>
    <body xmlns="http://www.w3.org/1999/xhtml">
      <p>GENE_ASSOCIATION: TT_P0004</p>
      <p>SUBSYSTEM: Cofactor and Prosthetic Group Biosynthesis</p>
      <p>EC Number: 2.7.8.26</p>
      <p>Confidence Level: 1</p>
      <p>AUTHORS: Energy and cofactors metabolism</p>
      <p/>
    </body>
  </notes>
  <listOfReactants>
    <speciesReference species="M_ADNS_CBNA_GDP_c" stoichiometry="1"/>
    <speciesReference species="M_ARIBZ_c" stoichiometry="1"/>
  </listOfReactants>
  <listOfProducts>
    <speciesReference species="M_H_c" stoichiometry="1"/>
    <speciesReference species="M_GMP_c" stoichiometry="1"/>
    <speciesReference species="M_CBMCo_c" stoichiometry="1"/>
  </listOfProducts>
  <kineticLaw>
    <math xmlns="http://www.w3.org/1998/Math/MathML">
      <ci> FLUX_VALUE </ci>
```

```
    </math>
    <listOfParameters>
      <parameter id="LOWER_BOUND" value="0" units="mmol_per_gDW_per_hr" constant="true"/>
      <parameter id="UPPER_BOUND" value="1000" units="mmol_per_gDW_per_hr" constant="true"/>
      <parameter id="FLUX_VALUE" value="0" units="mmol_per_gDW_per_hr" constant="true"/>
      <parameter id="OBJECTIVE_COEFFICIENT" value="0" units="mmol_per_gDW_per_hr" constant="true"/>
    </listOfParameters>
  </kineticLaw>
</reaction>
<reaction id="R_R558" name="citrullinase" reversible="false">
  <notes>
    <body xmlns="http://www.w3.org/1999/xhtml">
      <p>GENE_ASSOCIATION: </p>
      <p>SUBSYSTEM: Arginine and Proline Metabolism</p>
      <p>EC Number: 3.5.1.20</p>
      <p>Confidence Level: 1</p>
      <p>AUTHORS: Amino acid metabolism</p>
      <p/>
    </body>
  </notes>
  <listOfReactants>
    <speciesReference species="M_H2O_c" stoichiometry="1"/>
    <speciesReference species="M_H_c" stoichiometry="1"/>
    <speciesReference species="M_CTRLN_c" stoichiometry="1"/>
  </listOfReactants>
  <listOfProducts>
    <speciesReference species="M_ORT_c" stoichiometry="1"/>
    <speciesReference species="M_CO2_c" stoichiometry="1"/>
    <speciesReference species="M_NH4_c" stoichiometry="1"/>
```

```
    </listOfProducts>
    <kineticLaw>
      <math xmlns="http://www.w3.org/1998/Math/MathML">
        <ci> FLUX_VALUE </ci>
      </math>
      <listOfParameters>
        <parameter id="LOWER_BOUND" value="0" units="mmol_per_gDW_per_hr" constant="true"/>
        <parameter id="UPPER_BOUND" value="1000" units="mmol_per_gDW_per_hr" constant="true"/>
        <parameter id="FLUX_VALUE" value="0" units="mmol_per_gDW_per_hr" constant="true"/>
        <parameter id="OBJECTIVE_COEFFICIENT" value="0" units="mmol_per_gDW_per_hr" constant="true"/>
      </listOfParameters>
    </kineticLaw>
  </reaction>
  <reaction id="R_R559" name="malate dehydrogenase (quinone)" reversible="false">
    <notes>
      <body xmlns="http://www.w3.org/1999/xhtml">
        <p>GENE_ASSOCIATION: </p>
        <p>SUBSYSTEM: Citric Acid Cycle</p>
        <p>EC Number: 1.1.5.4</p>
        <p>Confidence Level: 1</p>
        <p>AUTHORS: Carbohydrate metabolism</p>
        <p/>
      </body>
    </notes>
    <listOfReactants>
      <speciesReference species="M_MAL_c" stoichiometry="1"/>
      <speciesReference species="M_Q_c" stoichiometry="1"/>
    </listOfReactants>
    <listOfProducts>
```

```
    <speciesReference species="M_OAC_c" stoichiometry="1"/>
    <speciesReference species="M_QH2_c" stoichiometry="1"/>
  </listOfProducts>
  <kineticLaw>
    <math xmlns="http://www.w3.org/1998/Math/MathML">
      <ci> FLUX_VALUE </ci>
    </math>
    <listOfParameters>
      <parameter id="LOWER_BOUND" value="0" units="mmol_per_gDW_per_hr" constant="true"/>
      <parameter id="UPPER_BOUND" value="1000" units="mmol_per_gDW_per_hr" constant="true"/>
      <parameter id="FLUX_VALUE" value="0" units="mmol_per_gDW_per_hr" constant="true"/>
      <parameter id="OBJECTIVE_COEFFICIENT" value="0" units="mmol_per_gDW_per_hr" constant="true"/>
    </listOfParameters>
  </kineticLaw>
</reaction>
<reaction id="R_R560" name="prephenate dehydratase" reversible="false">
  <notes>
    <body xmlns="http://www.w3.org/1999/xhtml">
      <p>GENE_ASSOCIATION: </p>
      <p>SUBSYSTEM: </p>
      <p>EC Number: 4.2.1.51</p>
      <p>Confidence Level: 1</p>
      <p>AUTHORS: Amino acid metabolism</p>
      <p/>
    </body>
  </notes>
  <listOfReactants>
    <speciesReference species="M_H_c" stoichiometry="1"/>
    <speciesReference species="M_PPHA_c" stoichiometry="1"/>
```

```xml
</listOfReactants>

<listOfProducts>

<speciesReference species="M_H2O_c" stoichiometry="1"/>

<speciesReference species="M_CO2_c" stoichiometry="1"/>

<speciesReference species="M_PPYR_c" stoichiometry="1"/>

</listOfProducts>

<kineticLaw>

<math xmlns="http://www.w3.org/1998/Math/MathML">

<ci> FLUX_VALUE </ci>

</math>

<listOfParameters>

<parameter id="LOWER_BOUND" value="0" units="mmol_per_gDW_per_hr" constant="true"/>

<parameter id="UPPER_BOUND" value="1000" units="mmol_per_gDW_per_hr" constant="true"/>

<parameter id="FLUX_VALUE" value="0" units="mmol_per_gDW_per_hr" constant="true"/>

<parameter id="OBJECTIVE_COEFFICIENT" value="0" units="mmol_per_gDW_per_hr" constant="true"/>

</listOfParameters>

</kineticLaw>

</reaction>

<reaction id="R_R561" name="3-isopropylmalate dehydrogenase; beta-isopropylmalic enzyme; beta-isopropylmalate dehydrogenase; threo-Ds-3-isopropylmalate dehydrogenase; 3-carboxy-2-hydroxy-4-methylpentanoate:NAD+ oxidoreductase" reversible="false">

<notes>

<body xmlns="http://www.w3.org/1999/xhtml">

<p>GENE_ASSOCIATION: TTC0867</p>

<p>SUBSYSTEM: Valine, Leucine, and Isoleucine Metabolism</p>

<p>EC Number: 1.1.1.85</p>

<p>Confidence Level: 1</p>

<p>AUTHORS: Amino acid metabolism</p>

<p/>

</body>
```

```xml
  </notes>
  <listOfReactants>
    <speciesReference species="M_H_c" stoichiometry="1"/>
    <speciesReference species="M_IPROP_OSUCC_c" stoichiometry="1"/>
  </listOfReactants>
  <listOfProducts>
    <speciesReference species="M_CO2_c" stoichiometry="1"/>
    <speciesReference species="M_4M_2OPENTN_c" stoichiometry="1"/>
  </listOfProducts>
  <kineticLaw>
    <math xmlns="http://www.w3.org/1998/Math/MathML">
      <ci> FLUX_VALUE </ci>
    </math>
    <listOfParameters>
      <parameter id="LOWER_BOUND" value="0" units="mmol_per_gDW_per_hr" constant="true"/>
      <parameter id="UPPER_BOUND" value="1000" units="mmol_per_gDW_per_hr" constant="true"/>
      <parameter id="FLUX_VALUE" value="0" units="mmol_per_gDW_per_hr" constant="true"/>
      <parameter id="OBJECTIVE_COEFFICIENT" value="0" units="mmol_per_gDW_per_hr" constant="true"/>
    </listOfParameters>
  </kineticLaw>
</reaction>
<reaction id="R_R562" name="prephenate dehydrogenase (NADP+)" reversible="true">
  <notes>
    <body xmlns="http://www.w3.org/1999/xhtml">
      <p>GENE_ASSOCIATION: TTC0447</p>
      <p>SUBSYSTEM: Tyrosine, Tryptophan, and Phenylalanine Metabolism</p>
      <p>EC Number: 1.3.1.13</p>
      <p>Confidence Level: 1</p>
      <p>AUTHORS: Amino acid metabolism</p>
```

```
      <p/>
    </body>
  </notes>
  <listOfReactants>
    <speciesReference species="M_NADP_c" stoichiometry="1"/>
    <speciesReference species="M_PPHA_c" stoichiometry="1"/>
  </listOfReactants>
  <listOfProducts>
    <speciesReference species="M_H_c" stoichiometry="1"/>
    <speciesReference species="M_CO2_c" stoichiometry="1"/>
    <speciesReference species="M_NADPH_c" stoichiometry="1"/>
    <speciesReference species="M_HYPh_PYR_c" stoichiometry="1"/>
  </listOfProducts>
  <kineticLaw>
    <math xmlns="http://www.w3.org/1998/Math/MathML">
      <ci> FLUX_VALUE </ci>
    </math>
    <listOfParameters>
      <parameter id="LOWER_BOUND" value="-1000" units="mmol_per_gDW_per_hr" constant="true"/>
      <parameter id="UPPER_BOUND" value="1000" units="mmol_per_gDW_per_hr" constant="true"/>
      <parameter id="FLUX_VALUE" value="0" units="mmol_per_gDW_per_hr" constant="true"/>
      <parameter id="OBJECTIVE_COEFFICIENT" value="0" units="mmol_per_gDW_per_hr" constant="true"/>
    </listOfParameters>
  </kineticLaw>
</reaction>
<reaction id="R_R563" name="N2-acetyl-alpha-aminoadipate semialdehyde transaminase" reversible="false">
  <notes>
    <body xmlns="http://www.w3.org/1999/xhtml">
      <p>GENE_ASSOCIATION: TTC1393</p>
```

```
    <p>SUBSYSTEM: Threonine and Lysine Metabolism</p>
    <p>EC Number: 2.6.1.-</p>
    <p>Confidence Level: 1</p>
    <p>AUTHORS: Amino acid metabolism</p>
    <p/>
   </body>
  </notes>
  <listOfReactants>
   <speciesReference species="M_L_GLU_c" stoichiometry="1"/>
   <speciesReference species="M_AC_AADP_SA_c" stoichiometry="1"/>
  </listOfReactants>
  <listOfProducts>
   <speciesReference species="M_AKG_c" stoichiometry="1"/>
   <speciesReference species="M_N2AC_L_LYS_c" stoichiometry="1"/>
  </listOfProducts>
  <kineticLaw>
   <math xmlns="http://www.w3.org/1998/Math/MathML">
    <ci> FLUX_VALUE </ci>
   </math>
   <listOfParameters>
    <parameter id="LOWER_BOUND" value="0" units="mmol_per_gDW_per_hr" constant="true"/>
    <parameter id="UPPER_BOUND" value="1000" units="mmol_per_gDW_per_hr" constant="true"/>
    <parameter id="FLUX_VALUE" value="0" units="mmol_per_gDW_per_hr" constant="true"/>
    <parameter id="OBJECTIVE_COEFFICIENT" value="0" units="mmol_per_gDW_per_hr" constant="true"/>
   </listOfParameters>
  </kineticLaw>
 </reaction>
 <reaction id="R_R564" name="alpha-aminoadipate acetyltransferase" reversible="true">
  <notes>
```

```
  <body xmlns="http://www.w3.org/1999/xhtml">
    <p>GENE_ASSOCIATION: TTC1543</p>
    <p>SUBSYSTEM: Threonine and Lysine Metabolism</p>
    <p>EC Number: 6.3.2.-</p>
    <p>Confidence Level: 1</p>
    <p>AUTHORS: Amino acid metabolism</p>
    <p/>
  </body>
</notes>
<listOfReactants>
  <speciesReference species="M_ACoA_c" stoichiometry="1"/>
  <speciesReference species="M_AADP_c" stoichiometry="1"/>
</listOfReactants>
<listOfProducts>
  <speciesReference species="M_H_c" stoichiometry="1"/>
  <speciesReference species="M_CoA_c" stoichiometry="1"/>
  <speciesReference species="M_AC_AADP_c" stoichiometry="1"/>
</listOfProducts>
<kineticLaw>
  <math xmlns="http://www.w3.org/1998/Math/MathML">
    <ci> FLUX_VALUE </ci>
  </math>
  <listOfParameters>
    <parameter id="LOWER_BOUND" value="-1000" units="mmol_per_gDW_per_hr" constant="true"/>
    <parameter id="UPPER_BOUND" value="1000" units="mmol_per_gDW_per_hr" constant="true"/>
    <parameter id="FLUX_VALUE" value="0" units="mmol_per_gDW_per_hr" constant="true"/>
    <parameter id="OBJECTIVE_COEFFICIENT" value="0" units="mmol_per_gDW_per_hr" constant="true"/>
  </listOfParameters>
</kineticLaw>
```

```
</reaction>
<reaction id="R_R565" name="methanogen homoaconitase" reversible="false">
 <notes>
  <body xmlns="http://www.w3.org/1999/xhtml">
   <p>GENE_ASSOCIATION: (TTC1546 and TTC1547)</p>
   <p>SUBSYSTEM: Threonine and Lysine Metabolism</p>
   <p>EC Number: 4.2.1.114</p>
   <p>Confidence Level: 1</p>
   <p>AUTHORS: Amino acid metabolism</p>
   <p/>
  </body>
 </notes>
 <listOfReactants>
  <speciesReference species="M_HCIT_c" stoichiometry="1"/>
 </listOfReactants>
 <listOfProducts>
  <speciesReference species="M_H2O_c" stoichiometry="1"/>
  <speciesReference species="M_HICTR_c" stoichiometry="1"/>
 </listOfProducts>
 <kineticLaw>
  <math xmlns="http://www.w3.org/1998/Math/MathML">
   <ci> FLUX_VALUE </ci>
  </math>
  <listOfParameters>
   <parameter id="LOWER_BOUND" value="0" units="mmol_per_gDW_per_hr" constant="true"/>
   <parameter id="UPPER_BOUND" value="1000" units="mmol_per_gDW_per_hr" constant="true"/>
   <parameter id="FLUX_VALUE" value="0" units="mmol_per_gDW_per_hr" constant="true"/>
   <parameter id="OBJECTIVE_COEFFICIENT" value="0" units="mmol_per_gDW_per_hr" constant="true"/>
  </listOfParameters>
```

```xml
  </kineticLaw>
</reaction>
<reaction id="R_R566" name="CDP-diacylglycerol-serine O-phosphatidyltransferase" reversible="false">
  <notes>
    <body xmlns="http://www.w3.org/1999/xhtml">
      <p>GENE_ASSOCIATION: TTC0824</p>
      <p>SUBSYSTEM: Membrane Lipid Metabolism</p>
      <p>EC Number: 2.7.8.8</p>
      <p>Confidence Level: 1</p>
      <p>AUTHORS: Lipid metabolism</p>
      <p/>
    </body>
  </notes>
  <listOfReactants>
    <speciesReference species="M_L_SER_c" stoichiometry="1"/>
    <speciesReference species="M_CDP_DAG_c" stoichiometry="1"/>
  </listOfReactants>
  <listOfProducts>
    <speciesReference species="M_H_c" stoichiometry="1"/>
    <speciesReference species="M_CMP_c" stoichiometry="1"/>
    <speciesReference species="M_PPTD_SER_c" stoichiometry="1"/>
  </listOfProducts>
  <kineticLaw>
    <math xmlns="http://www.w3.org/1998/Math/MathML">
      <ci> FLUX_VALUE </ci>
    </math>
    <listOfParameters>
      <parameter id="LOWER_BOUND" value="0" units="mmol_per_gDW_per_hr" constant="true"/>
      <parameter id="UPPER_BOUND" value="1000" units="mmol_per_gDW_per_hr" constant="true"/>
```

```
      <parameter id="FLUX_VALUE" value="0" units="mmol_per_gDW_per_hr" constant="true"/>
      <parameter id="OBJECTIVE_COEFFICIENT" value="0" units="mmol_per_gDW_per_hr" constant="true"/>
    </listOfParameters>
  </kineticLaw>
</reaction>
<reaction id="R_R567" name="sulfite reductase (NADPH2)" reversible="false">
  <notes>
    <body xmlns="http://www.w3.org/1999/xhtml">
      <p>GENE_ASSOCIATION: TTC0961</p>
      <p>SUBSYSTEM: Cysteine and methionine metabolism</p>
      <p>EC Number: 1.8.2.2</p>
      <p>Confidence Level: 1</p>
      <p>AUTHORS: Amino acid metabolism</p>
      <p/>
    </body>
  </notes>
  <listOfReactants>
    <speciesReference species="M_H_c" stoichiometry="5"/>
    <speciesReference species="M_NADPH_c" stoichiometry="3"/>
    <speciesReference species="M_SO3_c" stoichiometry="1"/>
  </listOfReactants>
  <listOfProducts>
    <speciesReference species="M_H2O_c" stoichiometry="3"/>
    <speciesReference species="M_NADP_c" stoichiometry="3"/>
    <speciesReference species="M_H2S_c" stoichiometry="1"/>
  </listOfProducts>
  <kineticLaw>
    <math xmlns="http://www.w3.org/1998/Math/MathML">
      <ci> FLUX_VALUE </ci>
```

```
      </math>
      <listOfParameters>
        <parameter id="LOWER_BOUND" value="0" units="mmol_per_gDW_per_hr" constant="true"/>
        <parameter id="UPPER_BOUND" value="1000" units="mmol_per_gDW_per_hr" constant="true"/>
        <parameter id="FLUX_VALUE" value="0" units="mmol_per_gDW_per_hr" constant="true"/>
        <parameter id="OBJECTIVE_COEFFICIENT" value="0" units="mmol_per_gDW_per_hr" constant="true"/>
      </listOfParameters>
    </kineticLaw>
  </reaction>
  <reaction id="R_R568" reversible="false">
    <notes>
      <body xmlns="http://www.w3.org/1999/xhtml">
        <p>GENE_ASSOCIATION: </p>
        <p>SUBSYSTEM: Inorganic Ion Transport and Metabolism</p>
        <p>EC Number: </p>
        <p>Confidence Level: 1</p>
        <p>AUTHORS: Energy and cofactors metabolism</p>
        <p/>
      </body>
    </notes>
    <listOfReactants>
      <speciesReference species="M_O2_c" stoichiometry="2"/>
      <speciesReference species="M_H2S_c" stoichiometry="1"/>
    </listOfReactants>
    <listOfProducts>
      <speciesReference species="M_H_c" stoichiometry="2"/>
      <speciesReference species="M_SO4_c" stoichiometry="1"/>
    </listOfProducts>
    <kineticLaw>
```

```
    <math xmlns="http://www.w3.org/1998/Math/MathML">
      <ci> FLUX_VALUE </ci>
    </math>
    <listOfParameters>
      <parameter id="LOWER_BOUND" value="0" units="mmol_per_gDW_per_hr" constant="true"/>
      <parameter id="UPPER_BOUND" value="1000" units="mmol_per_gDW_per_hr" constant="true"/>
      <parameter id="FLUX_VALUE" value="0" units="mmol_per_gDW_per_hr" constant="true"/>
      <parameter id="OBJECTIVE_COEFFICIENT" value="0" units="mmol_per_gDW_per_hr" constant="true"/>
    </listOfParameters>
  </kineticLaw>
</reaction>
<reaction id="R_R569" name="catalase" reversible="false">
  <notes>
    <body xmlns="http://www.w3.org/1999/xhtml">
      <p>GENE_ASSOCIATION: TTC1872</p>
      <p>SUBSYSTEM: Unassigned</p>
      <p>EC Number: </p>
      <p>Confidence Level: 1</p>
      <p>AUTHORS: Energy and cofactors metabolism</p>
      <p/>
    </body>
  </notes>
  <listOfReactants>
    <speciesReference species="M_H2O2_c" stoichiometry="2"/>
  </listOfReactants>
  <listOfProducts>
    <speciesReference species="M_H2O_c" stoichiometry="2"/>
    <speciesReference species="M_O2_c" stoichiometry="1"/>
  </listOfProducts>
```

```xml
  <kineticLaw>
    <math xmlns="http://www.w3.org/1998/Math/MathML">
      <ci> FLUX_VALUE </ci>
    </math>
    <listOfParameters>
      <parameter id="LOWER_BOUND" value="0" units="mmol_per_gDW_per_hr" constant="true"/>
      <parameter id="UPPER_BOUND" value="1000" units="mmol_per_gDW_per_hr" constant="true"/>
      <parameter id="FLUX_VALUE" value="0" units="mmol_per_gDW_per_hr" constant="true"/>
      <parameter id="OBJECTIVE_COEFFICIENT" value="0" units="mmol_per_gDW_per_hr" constant="true"/>
    </listOfParameters>
  </kineticLaw>
</reaction>
<reaction id="R_R570" name="cytochrome c oxidase" reversible="false">
  <notes>
    <body xmlns="http://www.w3.org/1999/xhtml">
      <p>GENE_ASSOCIATION: (TTC0769 and TTC0770 and TTC1671 and TTC1672)</p>
      <p>SUBSYSTEM: Oxidative Phosphorylation</p>
      <p>EC Number: 1.9.3.1</p>
      <p>Confidence Level: 1</p>
      <p>AUTHORS: Energy and cofactors metabolism</p>
      <p/>
    </body>
  </notes>
  <listOfReactants>
    <speciesReference species="M_H_c" stoichiometry="6"/>
    <speciesReference species="M_O2_c" stoichiometry="1"/>
    <speciesReference species="M_CYT_C2_c" stoichiometry="4"/>
  </listOfReactants>
  <listOfProducts>
```

```
<speciesReference species="M_H2O_c" stoichiometry="2"/>
<speciesReference species="M_H_e" stoichiometry="6"/>
<speciesReference species="M_CYT_C3_c" stoichiometry="4"/>
</listOfProducts>
<kineticLaw>
<math xmlns="http://www.w3.org/1998/Math/MathML">
<ci> FLUX_VALUE </ci>
</math>
<listOfParameters>
<parameter id="LOWER_BOUND" value="0" units="mmol_per_gDW_per_hr" constant="true"/>
<parameter id="UPPER_BOUND" value="1000" units="mmol_per_gDW_per_hr" constant="true"/>
<parameter id="FLUX_VALUE" value="0" units="mmol_per_gDW_per_hr" constant="true"/>
<parameter id="OBJECTIVE_COEFFICIENT" value="0" units="mmol_per_gDW_per_hr" constant="true"/>
</listOfParameters>
</kineticLaw>
</reaction>
<reaction id="R_R571" name="trehalase" reversible="false">
<notes>
<body xmlns="http://www.w3.org/1999/xhtml">
<p>GENE_ASSOCIATION: TTC0614</p>
<p>SUBSYSTEM: Alternate Carbon Metabolism</p>
<p>EC Number: 3.2.1.28</p>
<p>Confidence Level: 1</p>
<p>AUTHORS: Carbohydrate metabolism</p>
<p/>
</body>
</notes>
<listOfReactants>
<speciesReference species="M_H2O_c" stoichiometry="1"/>
```

```
    <speciesReference species="M_TRE_c" stoichiometry="1"/>
  </listOfReactants>
  <listOfProducts>
    <speciesReference species="M_D_GLC_c" stoichiometry="2"/>
  </listOfProducts>
  <kineticLaw>
    <math xmlns="http://www.w3.org/1998/Math/MathML">
      <ci> FLUX_VALUE </ci>
    </math>
    <listOfParameters>
      <parameter id="LOWER_BOUND" value="0" units="mmol_per_gDW_per_hr" constant="true"/>
      <parameter id="UPPER_BOUND" value="1000" units="mmol_per_gDW_per_hr" constant="true"/>
      <parameter id="FLUX_VALUE" value="0" units="mmol_per_gDW_per_hr" constant="true"/>
      <parameter id="OBJECTIVE_COEFFICIENT" value="0" units="mmol_per_gDW_per_hr" constant="true"/>
    </listOfParameters>
  </kineticLaw>
</reaction>
<reaction id="R_R572" name="trehalose-6-phosphate hydrolase" reversible="false">
  <notes>
    <body xmlns="http://www.w3.org/1999/xhtml">
      <p>GENE_ASSOCIATION: TTC0107</p>
      <p>SUBSYSTEM: Alternate Carbon Metabolism</p>
      <p>EC Number: 3.2.1.93</p>
      <p>Confidence Level: 1</p>
      <p>AUTHORS: Carbohydrate metabolism</p>
      <p/>
    </body>
  </notes>
  <listOfReactants>
```

```
    <speciesReference species="M_H2O_c" stoichiometry="1"/>
    <speciesReference species="M_TRE6P_c" stoichiometry="1"/>
  </listOfReactants>
  <listOfProducts>
    <speciesReference species="M_D_GLC_c" stoichiometry="1"/>
    <speciesReference species="M_G6P_c" stoichiometry="1"/>
  </listOfProducts>
  <kineticLaw>
    <math xmlns="http://www.w3.org/1998/Math/MathML">
      <ci> FLUX_VALUE </ci>
    </math>
    <listOfParameters>
      <parameter id="LOWER_BOUND" value="0" units="mmol_per_gDW_per_hr" constant="true"/>
      <parameter id="UPPER_BOUND" value="1000" units="mmol_per_gDW_per_hr" constant="true"/>
      <parameter id="FLUX_VALUE" value="0" units="mmol_per_gDW_per_hr" constant="true"/>
      <parameter id="OBJECTIVE_COEFFICIENT" value="0" units="mmol_per_gDW_per_hr" constant="true"/>
    </listOfParameters>
  </kineticLaw>
</reaction>
<reaction id="R_R573" name="trehalose-6-phosphate phosphatase" reversible="false">
  <notes>
    <body xmlns="http://www.w3.org/1999/xhtml">
      <p>GENE_ASSOCIATION: </p>
      <p>SUBSYSTEM: Alternate Carbon Metabolism</p>
      <p>EC Number: 3.1.3.12</p>
      <p>Confidence Level: 1</p>
      <p>AUTHORS: Carbohydrate metabolism</p>
      <p/>
    </body>
```

```
        </notes>
        <listOfReactants>
          <speciesReference species="M_H2O_c" stoichiometry="1"/>
          <speciesReference species="M_TRE6P_c" stoichiometry="1"/>
        </listOfReactants>
        <listOfProducts>
          <speciesReference species="M_Pi_c" stoichiometry="1"/>
          <speciesReference species="M_TRE_c" stoichiometry="1"/>
        </listOfProducts>
        <kineticLaw>
          <math xmlns="http://www.w3.org/1998/Math/MathML">
            <ci> FLUX_VALUE </ci>
          </math>
          <listOfParameters>
            <parameter id="LOWER_BOUND" value="0" units="mmol_per_gDW_per_hr" constant="true"/>
            <parameter id="UPPER_BOUND" value="1000" units="mmol_per_gDW_per_hr" constant="true"/>
            <parameter id="FLUX_VALUE" value="0" units="mmol_per_gDW_per_hr" constant="true"/>
            <parameter id="OBJECTIVE_COEFFICIENT" value="0" units="mmol_per_gDW_per_hr" constant="true"/>
          </listOfParameters>
        </kineticLaw>
      </reaction>
      <reaction id="R_R574" name="ATP synthase" reversible="true">
        <notes>
          <body xmlns="http://www.w3.org/1999/xhtml">
            <p>GENE_ASSOCIATION: (TTC0905 and TTC0906 and TTC0907 and TTC0908 and TTC0909 and TTC0910 and TTC0911 and
TTC0912)</p>
            <p>SUBSYSTEM: Oxidative Phosphorylation</p>
            <p>EC Number: 3.6.3.14</p>
            <p>Confidence Level: 1</p>
```

```
<p>AUTHORS: Energy and cofactors metabolism</p>

<p/>

</body>

</notes>

<listOfReactants>

<speciesReference species="M_ADP_c" stoichiometry="1"/>

<speciesReference species="M_Pi_c" stoichiometry="1"/>

<speciesReference species="M_H_e" stoichiometry="4"/>

</listOfReactants>

<listOfProducts>

<speciesReference species="M_ATP_c" stoichiometry="1"/>

<speciesReference species="M_H2O_c" stoichiometry="1"/>

<speciesReference species="M_H_c" stoichiometry="3"/>

</listOfProducts>

<kineticLaw>

<math xmlns="http://www.w3.org/1998/Math/MathML">

<ci> FLUX_VALUE </ci>

</math>

<listOfParameters>

<parameter id="LOWER_BOUND" value="-1000" units="mmol_per_gDW_per_hr" constant="true"/>

<parameter id="UPPER_BOUND" value="1000" units="mmol_per_gDW_per_hr" constant="true"/>

<parameter id="FLUX_VALUE" value="0" units="mmol_per_gDW_per_hr" constant="true"/>

<parameter id="OBJECTIVE_COEFFICIENT" value="0" units="mmol_per_gDW_per_hr" constant="true"/>

</listOfParameters>

</kineticLaw>

</reaction>

<reaction id="R_R575" name="2-methylpropanoyl-CoA:enzyme N6-(dihydrolipoyl)lysine S-(2-methylpropanoyl)transferase"
reversible="false">

<notes>
```

```
    <body xmlns="http://www.w3.org/1999/xhtml">
      <p>GENE_ASSOCIATION: TTC1757</p>
      <p>SUBSYSTEM: Valine, Leucine, and Isoleucine Metabolism</p>
      <p>EC Number: 2.3.1.168</p>
      <p>Confidence Level: 1</p>
      <p>AUTHORS: Amino acid metabolism</p>
      <p/>
    </body>
  </notes>
  <listOfReactants>
    <speciesReference species="M_CoA_c" stoichiometry="1"/>
    <speciesReference species="M_DHYLPL_MPROP_MPROP_DHLPL_c" stoichiometry="1"/>
  </listOfReactants>
  <listOfProducts>
    <speciesReference species="M_2M_PROCoA_c" stoichiometry="1"/>
    <speciesReference species="M_ENZNE_DHYLPL_c" stoichiometry="1"/>
  </listOfProducts>
  <kineticLaw>
    <math xmlns="http://www.w3.org/1998/Math/MathML">
      <ci> FLUX_VALUE </ci>
    </math>
    <listOfParameters>
      <parameter id="LOWER_BOUND" value="0" units="mmol_per_gDW_per_hr" constant="true"/>
      <parameter id="UPPER_BOUND" value="1000" units="mmol_per_gDW_per_hr" constant="true"/>
      <parameter id="FLUX_VALUE" value="0" units="mmol_per_gDW_per_hr" constant="true"/>
      <parameter id="OBJECTIVE_COEFFICIENT" value="0" units="mmol_per_gDW_per_hr" constant="true"/>
    </listOfParameters>
  </kineticLaw>
</reaction>
```

```xml
<reaction id="R_R576" name="(S)-2-methylbutanoyl-CoA:enzyme N6-(dihydrolipoyl)lysine S-(2-methylbutanoyl)transferase" reversible="false">
  <notes>
    <body xmlns="http://www.w3.org/1999/xhtml">
      <p>GENE_ASSOCIATION: TTC1757</p>
      <p>SUBSYSTEM: Valine, Leucine, and Isoleucine Metabolism</p>
      <p>EC Number: 2.3.1.168</p>
      <p>Confidence Level: 1</p>
      <p>AUTHORS: Amino acid metabolism</p>
      <p/>
    </body>
  </notes>
  <listOfReactants>
    <speciesReference species="M_CoA_c" stoichiometry="1"/>
    <speciesReference species="M_DHYLPL_MPROP_2MBUT_DHLPL_c" stoichiometry="1"/>
  </listOfReactants>
  <listOfProducts>
    <speciesReference species="M_MBUTNCoA_c" stoichiometry="1"/>
    <speciesReference species="M_ENZNE_DHYLPL_c" stoichiometry="1"/>
  </listOfProducts>
  <kineticLaw>
    <math xmlns="http://www.w3.org/1998/Math/MathML">
      <ci> FLUX_VALUE </ci>
    </math>
    <listOfParameters>
      <parameter id="LOWER_BOUND" value="0" units="mmol_per_gDW_per_hr" constant="true"/>
      <parameter id="UPPER_BOUND" value="1000" units="mmol_per_gDW_per_hr" constant="true"/>
      <parameter id="FLUX_VALUE" value="0" units="mmol_per_gDW_per_hr" constant="true"/>
      <parameter id="OBJECTIVE_COEFFICIENT" value="0" units="mmol_per_gDW_per_hr" constant="true"/>
```

```
        </listOfParameters>
      </kineticLaw>
    </reaction>
    <reaction id="R_R577" name="3-methylbutanoyl-CoA:enzyme N6-(dihydrolipoyl)lysine S-(3-methylbutanoyl)transferase"
reversible="false">
      <notes>
        <body xmlns="http://www.w3.org/1999/xhtml">
          <p>GENE_ASSOCIATION: TTC1757</p>
          <p>SUBSYSTEM: Valine, Leucine, and Isoleucine Metabolism</p>
          <p>EC Number: 2.3.1.168</p>
          <p>Confidence Level: 1</p>
          <p>AUTHORS: Amino acid metabolism</p>
          <p/>
        </body>
      </notes>
      <listOfReactants>
        <speciesReference species="M_CoA_c" stoichiometry="1"/>
        <speciesReference species="M_DHYLPL_MPROP_3MBUT_DHLPL_c" stoichiometry="1"/>
      </listOfReactants>
      <listOfProducts>
        <speciesReference species="M_ENZNE_DHYLPL_c" stoichiometry="1"/>
        <speciesReference species="M_IVCoA_c" stoichiometry="1"/>
      </listOfProducts>
      <kineticLaw>
        <math xmlns="http://www.w3.org/1998/Math/MathML">
          <ci> FLUX_VALUE </ci>
        </math>
        <listOfParameters>
          <parameter id="LOWER_BOUND" value="0" units="mmol_per_gDW_per_hr" constant="true"/>
```

```
      <parameter id="UPPER_BOUND" value="1000" units="mmol_per_gDW_per_hr" constant="true"/>
      <parameter id="FLUX_VALUE" value="0" units="mmol_per_gDW_per_hr" constant="true"/>
      <parameter id="OBJECTIVE_COEFFICIENT" value="0" units="mmol_per_gDW_per_hr" constant="true"/>
    </listOfParameters>
  </kineticLaw>
</reaction>
<reaction id="R_R578" name="dihydrolipoyl dehydrogenase" reversible="true">
  <notes>
    <body xmlns="http://www.w3.org/1999/xhtml">
      <p>GENE_ASSOCIATION: (TTC1700 or TTC1753)</p>
      <p>SUBSYSTEM: Valine, Leucine, and Isoleucine Metabolism</p>
      <p>EC Number: 1.8.1.4</p>
      <p>Confidence Level: 1</p>
      <p>AUTHORS: Amino acid metabolism</p>
      <p/>
    </body>
  </notes>
  <listOfReactants>
    <speciesReference species="M_NAD_c" stoichiometry="1"/>
    <speciesReference species="M_ENZNE_DHYLPL_c" stoichiometry="1"/>
  </listOfReactants>
  <listOfProducts>
    <speciesReference species="M_H_c" stoichiometry="1"/>
    <speciesReference species="M_NADH_c" stoichiometry="1"/>
    <speciesReference species="M_ENZN6_LPL_c" stoichiometry="1"/>
  </listOfProducts>
  <kineticLaw>
    <math xmlns="http://www.w3.org/1998/Math/MathML">
      <ci> FLUX_VALUE </ci>
```

```xml
    </math>
    <listOfParameters>
      <parameter id="LOWER_BOUND" value="-1000" units="mmol_per_gDW_per_hr" constant="true"/>
      <parameter id="UPPER_BOUND" value="1000" units="mmol_per_gDW_per_hr" constant="true"/>
      <parameter id="FLUX_VALUE" value="0" units="mmol_per_gDW_per_hr" constant="true"/>
      <parameter id="OBJECTIVE_COEFFICIENT" value="0" units="mmol_per_gDW_per_hr" constant="true"/>
    </listOfParameters>
  </kineticLaw>
</reaction>
<reaction id="R_R579" name="3-methylbutanoyl-CoA:electron-transfer flavoprotein 2,3-oxidoreductase" reversible="true">
  <notes>
    <body xmlns="http://www.w3.org/1999/xhtml">
      <p>GENE_ASSOCIATION: (TTC0238 or TTC0536 or TTC779 or TTC1552 or TTC1575 or TT_P0074)</p>
      <p>SUBSYSTEM: Valine, Leucine, and Isoleucine Metabolism</p>
      <p>EC Number: 1.3.8.7</p>
      <p>Confidence Level: 1</p>
      <p>AUTHORS: Amino acid metabolism</p>
      <p/>
    </body>
  </notes>
  <listOfReactants>
    <speciesReference species="M_FAD_c" stoichiometry="1"/>
    <speciesReference species="M_IVCoA_c" stoichiometry="1"/>
  </listOfReactants>
  <listOfProducts>
    <speciesReference species="M_FADH2_c" stoichiometry="1"/>
    <speciesReference species="M_MCROCoA_c" stoichiometry="1"/>
  </listOfProducts>
  <kineticLaw>
```

```
<math xmlns="http://www.w3.org/1998/Math/MathML">
  <ci> FLUX_VALUE </ci>
</math>
<listOfParameters>
  <parameter id="LOWER_BOUND" value="-1000" units="mmol_per_gDW_per_hr" constant="true"/>
  <parameter id="UPPER_BOUND" value="1000" units="mmol_per_gDW_per_hr" constant="true"/>
  <parameter id="FLUX_VALUE" value="0" units="mmol_per_gDW_per_hr" constant="true"/>
  <parameter id="OBJECTIVE_COEFFICIENT" value="0" units="mmol_per_gDW_per_hr" constant="true"/>
</listOfParameters>
</kineticLaw>
</reaction>
<reaction id="R_R580" name="3-Methylcrotonoyl-CoA:carbon-dioxide ligase (ADP-forming)" reversible="false">
 <notes>
  <body xmlns="http://www.w3.org/1999/xhtml">
   <p>GENE_ASSOCIATION: TTC1192</p>
   <p>SUBSYSTEM: Valine, Leucine, and Isoleucine Metabolism</p>
   <p>EC Number: 6.4.1.4</p>
   <p>Confidence Level: 1</p>
   <p>AUTHORS: Amino acid metabolism</p>
   <p/>
  </body>
 </notes>
 <listOfReactants>
  <speciesReference species="M_ATP_c" stoichiometry="1"/>
  <speciesReference species="M_HCO3_c" stoichiometry="1"/>
  <speciesReference species="M_MCROCoA_c" stoichiometry="1"/>
 </listOfReactants>
 <listOfProducts>
  <speciesReference species="M_ADP_c" stoichiometry="1"/>
```

```xml
<speciesReference species="M_Pi_c" stoichiometry="1"/>

<speciesReference species="M_3MGLUTNCoA_c" stoichiometry="1"/>

</listOfProducts>

<kineticLaw>

<math xmlns="http://www.w3.org/1998/Math/MathML">

<ci> FLUX_VALUE </ci>

</math>

<listOfParameters>

<parameter id="LOWER_BOUND" value="0" units="mmol_per_gDW_per_hr" constant="true"/>

<parameter id="UPPER_BOUND" value="1000" units="mmol_per_gDW_per_hr" constant="true"/>

<parameter id="FLUX_VALUE" value="0" units="mmol_per_gDW_per_hr" constant="true"/>

<parameter id="OBJECTIVE_COEFFICIENT" value="0" units="mmol_per_gDW_per_hr" constant="true"/>

</listOfParameters>

</kineticLaw>

</reaction>

<reaction id="R_R581" name="(S)-3-Hydroxy-3-methylglutaryl-CoA hydro-lyase (trans-3-methylglutaconyl-CoA-forming)" reversible="false">

<notes>

<body xmlns="http://www.w3.org/1999/xhtml">

<p>GENE_ASSOCIATION: TTC0182</p>

<p>SUBSYSTEM: Valine, Leucine, and Isoleucine Metabolism</p>

<p>EC Number: 4.2.1.18</p>

<p>Confidence Level: 1</p>

<p>AUTHORS: Amino acid metabolism</p>

<p/>

</body>

</notes>

<listOfReactants>

<speciesReference species="M_H2O_c" stoichiometry="1"/>
```

```
    <speciesReference species="M_3MGLUTNCoA_c" stoichiometry="1"/>
  </listOfReactants>
  <listOfProducts>
    <speciesReference species="M_HYM_GLUTRCoA_c" stoichiometry="1"/>
  </listOfProducts>
  <kineticLaw>
    <math xmlns="http://www.w3.org/1998/Math/MathML">
      <ci> FLUX_VALUE </ci>
    </math>
    <listOfParameters>
      <parameter id="LOWER_BOUND" value="0" units="mmol_per_gDW_per_hr" constant="true"/>
      <parameter id="UPPER_BOUND" value="1000" units="mmol_per_gDW_per_hr" constant="true"/>
      <parameter id="FLUX_VALUE" value="0" units="mmol_per_gDW_per_hr" constant="true"/>
      <parameter id="OBJECTIVE_COEFFICIENT" value="0" units="mmol_per_gDW_per_hr" constant="true"/>
    </listOfParameters>
  </kineticLaw>
</reaction>
<reaction id="R_R582" name="succinyl-CoA:acetoacetate CoA-transferase" reversible="true">
  <notes>
    <body xmlns="http://www.w3.org/1999/xhtml">
      <p>GENE_ASSOCIATION: </p>
      <p>SUBSYSTEM: Citric Acid Cycle</p>
      <p>EC Number: 2.8.3.5</p>
      <p>Confidence Level: 1</p>
      <p>AUTHORS: Amino acid metabolism</p>
      <p/>
    </body>
  </notes>
  <listOfReactants>
```

```
      <speciesReference species="M_SUCCoA_c" stoichiometry="1"/>
      <speciesReference species="M_AAC_c" stoichiometry="1"/>
    </listOfReactants>
    <listOfProducts>
      <speciesReference species="M_SUCC_c" stoichiometry="1"/>
      <speciesReference species="M_ACACoA_c" stoichiometry="1"/>
    </listOfProducts>
    <kineticLaw>
      <math xmlns="http://www.w3.org/1998/Math/MathML">
        <ci> FLUX_VALUE </ci>
      </math>
      <listOfParameters>
        <parameter id="LOWER_BOUND" value="-1000" units="mmol_per_gDW_per_hr" constant="true"/>
        <parameter id="UPPER_BOUND" value="1000" units="mmol_per_gDW_per_hr" constant="true"/>
        <parameter id="FLUX_VALUE" value="0" units="mmol_per_gDW_per_hr" constant="true"/>
        <parameter id="OBJECTIVE_COEFFICIENT" value="0" units="mmol_per_gDW_per_hr" constant="true"/>
      </listOfParameters>
    </kineticLaw>
  </reaction>
  <reaction id="R_R583" name="(S)-3-Hydroxyisobutyryl-CoA hydrolase" reversible="true">
    <notes>
      <body xmlns="http://www.w3.org/1999/xhtml">
        <p>GENE_ASSOCIATION: </p>
        <p>SUBSYSTEM: Valine, Leucine, and Isoleucine Metabolism</p>
        <p>EC Number: 3.1.2.4</p>
        <p>Confidence Level: 1</p>
        <p>AUTHORS: Amino acid metabolism</p>
        <p/>
      </body>
```

```xml
</notes>
<listOfReactants>
<speciesReference species="M_H2O_c" stoichiometry="1"/>
<speciesReference species="M_HYIBUTRCoA_c" stoichiometry="1"/>
</listOfReactants>
<listOfProducts>
<speciesReference species="M_CoA_c" stoichiometry="1"/>
<speciesReference species="M_HYIBUTRA_c" stoichiometry="1"/>
</listOfProducts>
<kineticLaw>
<math xmlns="http://www.w3.org/1998/Math/MathML">
<ci> FLUX_VALUE </ci>
</math>
<listOfParameters>
<parameter id="LOWER_BOUND" value="-1000" units="mmol_per_gDW_per_hr" constant="true"/>
<parameter id="UPPER_BOUND" value="1000" units="mmol_per_gDW_per_hr" constant="true"/>
<parameter id="FLUX_VALUE" value="0" units="mmol_per_gDW_per_hr" constant="true"/>
<parameter id="OBJECTIVE_COEFFICIENT" value="0" units="mmol_per_gDW_per_hr" constant="true"/>
</listOfParameters>
</kineticLaw>
</reaction>
<reaction id="R_R584" name="(2S,3S)-3-hydroxy-2-methylbutanoyl-CoA:NAD+ oxidoreductase" reversible="false">
<notes>
<body xmlns="http://www.w3.org/1999/xhtml">
<p>GENE_ASSOCIATION: TTC0534</p>
<p>SUBSYSTEM: Valine, Leucine, and Isoleucine Metabolism</p>
<p>EC Number: 1.1.1.35</p>
<p>Confidence Level: 1</p>
<p>AUTHORS: Amino acid metabolism</p>
```

```
      <p/>
    </body>
  </notes>
  <listOfReactants>
    <speciesReference species="M_NAD_c" stoichiometry="1"/>
    <speciesReference species="M_HY_MBUTRCoA_c" stoichiometry="1"/>
  </listOfReactants>
  <listOfProducts>
    <speciesReference species="M_H_c" stoichiometry="1"/>
    <speciesReference species="M_NADH_c" stoichiometry="1"/>
    <speciesReference species="M_MAACCoA_c" stoichiometry="1"/>
  </listOfProducts>
  <kineticLaw>
    <math xmlns="http://www.w3.org/1998/Math/MathML">
      <ci> FLUX_VALUE </ci>
    </math>
    <listOfParameters>
      <parameter id="LOWER_BOUND" value="0" units="mmol_per_gDW_per_hr" constant="true"/>
      <parameter id="UPPER_BOUND" value="1000" units="mmol_per_gDW_per_hr" constant="true"/>
      <parameter id="FLUX_VALUE" value="0" units="mmol_per_gDW_per_hr" constant="true"/>
      <parameter id="OBJECTIVE_COEFFICIENT" value="0" units="mmol_per_gDW_per_hr" constant="true"/>
    </listOfParameters>
  </kineticLaw>
</reaction>
<reaction id="R_R585" name="Methylmalonyl-CoA epimerase" reversible="true">
  <notes>
    <body xmlns="http://www.w3.org/1999/xhtml">
      <p>GENE_ASSOCIATION: TTC0024</p>
      <p>SUBSYSTEM: Valine, Leucine, and Isoleucine Metabolism</p>
```

```xml
<p>EC Number: 5.1.99.1</p>

<p>Confidence Level: 1</p>

<p>AUTHORS: Amino acid metabolism</p>

<p/>

</body>

</notes>

<listOfReactants>

<speciesReference species="M_R_MMALCoA_c" stoichiometry="1"/>

</listOfReactants>

<listOfProducts>

<speciesReference species="M_MMALCoA_c" stoichiometry="1"/>

</listOfProducts>

<kineticLaw>

<math xmlns="http://www.w3.org/1998/Math/MathML">

<ci> FLUX_VALUE </ci>

</math>

<listOfParameters>

<parameter id="LOWER_BOUND" value="-1000" units="mmol_per_gDW_per_hr" constant="true"/>

<parameter id="UPPER_BOUND" value="1000" units="mmol_per_gDW_per_hr" constant="true"/>

<parameter id="FLUX_VALUE" value="0" units="mmol_per_gDW_per_hr" constant="true"/>

<parameter id="OBJECTIVE_COEFFICIENT" value="0" units="mmol_per_gDW_per_hr" constant="true"/>

</listOfParameters>

</kineticLaw>

</reaction>

<reaction id="R_R586" name="biotin carboxyl carrier protein of acetyl-CoA carboxylase, acetyl-CoA carboxylase carboxyltransferase subunit alpha, acetyl-coenzyme A carboxylase carboxyl transferase subunit beta" reversible="false">

<notes>

<body xmlns="http://www.w3.org/1999/xhtml">

<p>GENE_ASSOCIATION: (TTC1408 and TTC1409)</p>
```

```
    <p>SUBSYSTEM: Membrane Lipid Metabolism</p>
    <p>EC Number: 6.4.1.2</p>
    <p>Confidence Level: 1</p>
    <p>AUTHORS: Lipid metabolism</p>
    <p/>
  </body>
</notes>
<listOfReactants>
  <speciesReference species="M_ATP_c" stoichiometry="1"/>
  <speciesReference species="M_ACoA_c" stoichiometry="1"/>
  <speciesReference species="M_HCO3_c" stoichiometry="1"/>
</listOfReactants>
<listOfProducts>
  <speciesReference species="M_ADP_c" stoichiometry="1"/>
  <speciesReference species="M_Pi_c" stoichiometry="1"/>
  <speciesReference species="M_H_c" stoichiometry="1"/>
  <speciesReference species="M_MALCoA_c" stoichiometry="1"/>
</listOfProducts>
<kineticLaw>
  <math xmlns="http://www.w3.org/1998/Math/MathML">
    <ci> FLUX_VALUE </ci>
  </math>
  <listOfParameters>
    <parameter id="LOWER_BOUND" value="0" units="mmol_per_gDW_per_hr" constant="true"/>
    <parameter id="UPPER_BOUND" value="1000" units="mmol_per_gDW_per_hr" constant="true"/>
    <parameter id="FLUX_VALUE" value="0" units="mmol_per_gDW_per_hr" constant="true"/>
    <parameter id="OBJECTIVE_COEFFICIENT" value="0" units="mmol_per_gDW_per_hr" constant="true"/>
  </listOfParameters>
</kineticLaw>
```

```xml
</reaction>
<reaction id="R_R587" name="phosphoribosylglycinamide formyltransferase" reversible="true">
<notes>
<body xmlns="http://www.w3.org/1999/xhtml">
<p>GENE_ASSOCIATION: TTC0459</p>
<p>SUBSYSTEM: Nucleotide Salvage Pathway</p>
<p>EC Number: 2.1.2.2</p>
<p>Confidence Level: 1</p>
<p>AUTHORS: Nucleotides metabolism</p>
<p/>
</body>
</notes>
<listOfReactants>
<speciesReference species="M_GAR_c" stoichiometry="1"/>
<speciesReference species="M_10F_THF_c" stoichiometry="1"/>
</listOfReactants>
<listOfProducts>
<speciesReference species="M_H_c" stoichiometry="1"/>
<speciesReference species="M_FGAR_c" stoichiometry="1"/>
<speciesReference species="M_THF_c" stoichiometry="1"/>
</listOfProducts>
<kineticLaw>
<math xmlns="http://www.w3.org/1998/Math/MathML">
<ci> FLUX_VALUE </ci>
</math>
<listOfParameters>
<parameter id="LOWER_BOUND" value="0" units="mmol_per_gDW_per_hr" constant="true"/>
<parameter id="UPPER_BOUND" value="0" units="mmol_per_gDW_per_hr" constant="true"/>
<parameter id="FLUX_VALUE" value="0" units="mmol_per_gDW_per_hr" constant="true"/>
```

```xml
      <parameter id="OBJECTIVE_COEFFICIENT" value="0" units="mmol_per_gDW_per_hr" constant="true"/>
    </listOfParameters>
  </kineticLaw>
</reaction>
<reaction id="R_R588" name="fumarate reductase (menaquinone)" reversible="false">
  <notes>
    <body xmlns="http://www.w3.org/1999/xhtml">
      <p>GENE_ASSOCIATION: TTC1089</p>
      <p>SUBSYSTEM: Citric Acid Cycle</p>
      <p>EC Number: 1.3.5.4</p>
      <p>Confidence Level: 1</p>
      <p>AUTHORS: Carbohydrate metabolism</p>
      <p/>
    </body>
  </notes>
  <listOfReactants>
    <speciesReference species="M_FUM_c" stoichiometry="1"/>
    <speciesReference species="M_MQL_c" stoichiometry="1"/>
  </listOfReactants>
  <listOfProducts>
    <speciesReference species="M_SUCC_c" stoichiometry="1"/>
    <speciesReference species="M_MQ_c" stoichiometry="1"/>
  </listOfProducts>
  <kineticLaw>
    <math xmlns="http://www.w3.org/1998/Math/MathML">
      <ci> FLUX_VALUE </ci>
    </math>
    <listOfParameters>
      <parameter id="LOWER_BOUND" value="0" units="mmol_per_gDW_per_hr" constant="true"/>
```

```
      <parameter id="UPPER_BOUND" value="1000" units="mmol_per_gDW_per_hr" constant="true"/>
      <parameter id="FLUX_VALUE" value="0" units="mmol_per_gDW_per_hr" constant="true"/>
      <parameter id="OBJECTIVE_COEFFICIENT" value="0" units="mmol_per_gDW_per_hr" constant="true"/>
    </listOfParameters>
  </kineticLaw>
</reaction>
<reaction id="R_R589" name="succinate dehydrogenase membrane anchor subunit" reversible="false">
  <notes>
    <body xmlns="http://www.w3.org/1999/xhtml">
      <p>GENE_ASSOCIATION: TTC1092</p>
      <p>SUBSYSTEM: Oxidative Phosphorylation</p>
      <p>EC Number: 1.3.5.1</p>
      <p>Confidence Level: 1</p>
      <p>AUTHORS: Energy and cofactors metabolism</p>
      <p/>
    </body>
  </notes>
  <listOfReactants>
    <speciesReference species="M_SUCC_c" stoichiometry="1"/>
    <speciesReference species="M_Q_c" stoichiometry="1"/>
  </listOfReactants>
  <listOfProducts>
    <speciesReference species="M_FUM_c" stoichiometry="1"/>
    <speciesReference species="M_QH2_c" stoichiometry="1"/>
  </listOfProducts>
  <kineticLaw>
    <math xmlns="http://www.w3.org/1998/Math/MathML">
      <ci> FLUX_VALUE </ci>
    </math>
```

```
    <listOfParameters>
      <parameter id="LOWER_BOUND" value="0" units="mmol_per_gDW_per_hr" constant="true"/>
      <parameter id="UPPER_BOUND" value="1000" units="mmol_per_gDW_per_hr" constant="true"/>
      <parameter id="FLUX_VALUE" value="0" units="mmol_per_gDW_per_hr" constant="true"/>
      <parameter id="OBJECTIVE_COEFFICIENT" value="0" units="mmol_per_gDW_per_hr" constant="true"/>
    </listOfParameters>
  </kineticLaw>
</reaction>
<reaction id="R_R590" name="formate C-acetyltransferase" reversible="false">
  <notes>
    <body xmlns="http://www.w3.org/1999/xhtml">
      <p>GENE_ASSOCIATION: </p>
      <p>SUBSYSTEM: Pyruvate Metabolism</p>
      <p>EC Number: 2.3.1.54</p>
      <p>Confidence Level: 1</p>
      <p>AUTHORS: Carbohydrate metabolism</p>
      <p/>
    </body>
  </notes>
  <listOfReactants>
    <speciesReference species="M_PYR_c" stoichiometry="1"/>
    <speciesReference species="M_CoA_c" stoichiometry="1"/>
  </listOfReactants>
  <listOfProducts>
    <speciesReference species="M_ACoA_c" stoichiometry="1"/>
    <speciesReference species="M_FOR_c" stoichiometry="1"/>
  </listOfProducts>
  <kineticLaw>
    <math xmlns="http://www.w3.org/1998/Math/MathML">
```

```
      <ci> FLUX_VALUE </ci>
    </math>
    <listOfParameters>
      <parameter id="LOWER_BOUND" value="0" units="mmol_per_gDW_per_hr" constant="true"/>
      <parameter id="UPPER_BOUND" value="1000" units="mmol_per_gDW_per_hr" constant="true"/>
      <parameter id="FLUX_VALUE" value="0" units="mmol_per_gDW_per_hr" constant="true"/>
      <parameter id="OBJECTIVE_COEFFICIENT" value="0" units="mmol_per_gDW_per_hr" constant="true"/>
    </listOfParameters>
  </kineticLaw>
</reaction>
<reaction id="R_R591" name="protoporphyrinogen IX dehydrogenase (menaquinone)" reversible="false">
  <notes>
    <body xmlns="http://www.w3.org/1999/xhtml">
      <p>GENE_ASSOCIATION: </p>
      <p>SUBSYSTEM: Cofactor and Prosthetic Group Biosynthesis</p>
      <p>EC Number: 1.3.5.3</p>
      <p>Confidence Level: 1</p>
      <p>AUTHORS: Energy and cofactors metabolism</p>
      <p/>
    </body>
  </notes>
  <listOfReactants>
    <speciesReference species="M_MQ_c" stoichiometry="3"/>
    <speciesReference species="M_PPPHYRGN_c" stoichiometry="1"/>
  </listOfReactants>
  <listOfProducts>
    <speciesReference species="M_PPPHYR_c" stoichiometry="1"/>
    <speciesReference species="M_MQL_c" stoichiometry="3"/>
  </listOfProducts>
```

```
  <kineticLaw>
    <math xmlns="http://www.w3.org/1998/Math/MathML">
      <ci> FLUX_VALUE </ci>
    </math>
    <listOfParameters>
      <parameter id="LOWER_BOUND" value="0" units="mmol_per_gDW_per_hr" constant="true"/>
      <parameter id="UPPER_BOUND" value="1000" units="mmol_per_gDW_per_hr" constant="true"/>
      <parameter id="FLUX_VALUE" value="0" units="mmol_per_gDW_per_hr" constant="true"/>
      <parameter id="OBJECTIVE_COEFFICIENT" value="0" units="mmol_per_gDW_per_hr" constant="true"/>
    </listOfParameters>
  </kineticLaw>
</reaction>
<reaction id="R_R592" name="L-glutamate 5-semialdehyde dehydratase (spontaneous)" reversible="true">
  <notes>
    <body xmlns="http://www.w3.org/1999/xhtml">
      <p>GENE_ASSOCIATION: </p>
      <p>SUBSYSTEM: Arginine and Proline Metabolism</p>
      <p>EC Number: spontaneous</p>
      <p>Confidence Level: 1</p>
      <p>AUTHORS: Amino acid metabolism</p>
      <p/>
    </body>
  </notes>
  <listOfReactants>
    <speciesReference species="M_GLUT_SA_c" stoichiometry="1"/>
  </listOfReactants>
  <listOfProducts>
    <speciesReference species="M_H2O_c" stoichiometry="1"/>
    <speciesReference species="M_H_c" stoichiometry="1"/>
```

```xml
<speciesReference species="M_PYRR5CARB_c" stoichiometry="1"/>
</listOfProducts>
<kineticLaw>
<math xmlns="http://www.w3.org/1998/Math/MathML">
<ci> FLUX_VALUE </ci>
</math>
<listOfParameters>
<parameter id="LOWER_BOUND" value="-1000" units="mmol_per_gDW_per_hr" constant="true"/>
<parameter id="UPPER_BOUND" value="1000" units="mmol_per_gDW_per_hr" constant="true"/>
<parameter id="FLUX_VALUE" value="0" units="mmol_per_gDW_per_hr" constant="true"/>
<parameter id="OBJECTIVE_COEFFICIENT" value="0" units="mmol_per_gDW_per_hr" constant="true"/>
</listOfParameters>
</kineticLaw>
</reaction>
<reaction id="R_R593" name="serine O-acetyltransferase" reversible="false">
<notes>
<body xmlns="http://www.w3.org/1999/xhtml">
<p>GENE_ASSOCIATION: TTC1875</p>
<p>SUBSYSTEM: Cysteine and methionine metabolism</p>
<p>EC Number: 2.3.1.30</p>
<p>Confidence Level: 1</p>
<p>AUTHORS: Amino acid metabolism</p>
<p/>
</body>
</notes>
<listOfReactants>
<speciesReference species="M_L_SER_c" stoichiometry="1"/>
<speciesReference species="M_ACoA_c" stoichiometry="1"/>
</listOfReactants>
```

```
  <listOfProducts>
    <speciesReference species="M_CoA_c" stoichiometry="1"/>
    <speciesReference species="M_OAC_SER_c" stoichiometry="1"/>
  </listOfProducts>
  <kineticLaw>
    <math xmlns="http://www.w3.org/1998/Math/MathML">
      <ci> FLUX_VALUE </ci>
    </math>
    <listOfParameters>
      <parameter id="LOWER_BOUND" value="0" units="mmol_per_gDW_per_hr" constant="true"/>
      <parameter id="UPPER_BOUND" value="1000" units="mmol_per_gDW_per_hr" constant="true"/>
      <parameter id="FLUX_VALUE" value="0" units="mmol_per_gDW_per_hr" constant="true"/>
      <parameter id="OBJECTIVE_COEFFICIENT" value="0" units="mmol_per_gDW_per_hr" constant="true"/>
    </listOfParameters>
  </kineticLaw>
</reaction>
<reaction id="R_R594" name="pyruvate dehydrogenase E1 component beta subunit" reversible="false">
  <notes>
    <body xmlns="http://www.w3.org/1999/xhtml">
      <p>GENE_ASSOCIATION: (TTC0568 and TTC1801 and TTC1802 and TTC1754 and TTC1753 and TTC1700)</p>
      <p>SUBSYSTEM: Glycolysis/Gluconeogenesis</p>
      <p>EC Number: 1.2.1.-</p>
      <p>Confidence Level: 1</p>
      <p>AUTHORS: Carbohydrate metabolism</p>
      <p/>
    </body>
  </notes>
  <listOfReactants>
    <speciesReference species="M_NAD_c" stoichiometry="1"/>
```

```
    <speciesReference species="M_PYR_c" stoichiometry="1"/>
    <speciesReference species="M_CoA_c" stoichiometry="1"/>
  </listOfReactants>
  <listOfProducts>
    <speciesReference species="M_NADH_c" stoichiometry="1"/>
    <speciesReference species="M_CO2_c" stoichiometry="1"/>
    <speciesReference species="M_ACoA_c" stoichiometry="1"/>
  </listOfProducts>
  <kineticLaw>
    <math xmlns="http://www.w3.org/1998/Math/MathML">
      <ci> FLUX_VALUE </ci>
    </math>
    <listOfParameters>
      <parameter id="LOWER_BOUND" value="0" units="mmol_per_gDW_per_hr" constant="true"/>
      <parameter id="UPPER_BOUND" value="1000" units="mmol_per_gDW_per_hr" constant="true"/>
      <parameter id="FLUX_VALUE" value="0" units="mmol_per_gDW_per_hr" constant="true"/>
      <parameter id="OBJECTIVE_COEFFICIENT" value="0" units="mmol_per_gDW_per_hr" constant="true"/>
    </listOfParameters>
  </kineticLaw>
</reaction>
<reaction id="R_R595" name="acetaldehyde dehydrogenase" reversible="true">
  <notes>
    <body xmlns="http://www.w3.org/1999/xhtml">
      <p>GENE_ASSOCIATION: TTC0604</p>
      <p>SUBSYSTEM: Pyruvate Metabolism</p>
      <p>EC Number: 1.2.1.10</p>
      <p>Confidence Level: 1</p>
      <p>AUTHORS: Carbohydrate metabolism</p>
      <p/>
```

```
    </body>
  </notes>
  <listOfReactants>
    <speciesReference species="M_NAD_c" stoichiometry="1"/>
    <speciesReference species="M_CoA_c" stoichiometry="1"/>
    <speciesReference species="M_AALD_c" stoichiometry="1"/>
  </listOfReactants>
  <listOfProducts>
    <speciesReference species="M_H_c" stoichiometry="1"/>
    <speciesReference species="M_NADH_c" stoichiometry="1"/>
    <speciesReference species="M_ACoA_c" stoichiometry="1"/>
  </listOfProducts>
  <kineticLaw>
    <math xmlns="http://www.w3.org/1998/Math/MathML">
      <ci> FLUX_VALUE </ci>
    </math>
    <listOfParameters>
      <parameter id="LOWER_BOUND" value="-1000" units="mmol_per_gDW_per_hr" constant="true"/>
      <parameter id="UPPER_BOUND" value="1000" units="mmol_per_gDW_per_hr" constant="true"/>
      <parameter id="FLUX_VALUE" value="0" units="mmol_per_gDW_per_hr" constant="true"/>
      <parameter id="OBJECTIVE_COEFFICIENT" value="0" units="mmol_per_gDW_per_hr" constant="true"/>
    </listOfParameters>
  </kineticLaw>
</reaction>
<reaction id="R_R596" name="pyrimidine phosphatase" reversible="false">
  <notes>
    <body xmlns="http://www.w3.org/1999/xhtml">
      <p>GENE_ASSOCIATION: </p>
      <p>SUBSYSTEM: Cofactor and Prosthetic Group Biosynthesis</p>
```

```xml
      <p>EC Number: 3.6.1.-</p>
      <p>Confidence Level: 1</p>
      <p>AUTHORS: Energy and cofactors metabolism</p>
      <p/>
    </body>
  </notes>
  <listOfReactants>
    <speciesReference species="M_H2O_c" stoichiometry="1"/>
    <speciesReference species="M_APRIBTA_URA_c" stoichiometry="1"/>
  </listOfReactants>
  <listOfProducts>
    <speciesReference species="M_Pi_c" stoichiometry="1"/>
    <speciesReference species="M_5AM_RIBAM_URA_c" stoichiometry="1"/>
  </listOfProducts>
  <kineticLaw>
    <math xmlns="http://www.w3.org/1998/Math/MathML">
      <ci> FLUX_VALUE </ci>
    </math>
    <listOfParameters>
      <parameter id="LOWER_BOUND" value="0" units="mmol_per_gDW_per_hr" constant="true"/>
      <parameter id="UPPER_BOUND" value="1000" units="mmol_per_gDW_per_hr" constant="true"/>
      <parameter id="FLUX_VALUE" value="0" units="mmol_per_gDW_per_hr" constant="true"/>
      <parameter id="OBJECTIVE_COEFFICIENT" value="0" units="mmol_per_gDW_per_hr" constant="true"/>
    </listOfParameters>
  </kineticLaw>
</reaction>
<reaction id="R_R597" name="riboflavin synthase subunit alpha" reversible="false">
  <notes>
    <body xmlns="http://www.w3.org/1999/xhtml">
```

```
      <p>GENE_ASSOCIATION: TTC0698</p>
      <p>SUBSYSTEM: Cofactor and Prosthetic Group Biosynthesis</p>
      <p>EC Number: 2.5.1.9</p>
      <p>Confidence Level: 1</p>
      <p>AUTHORS: Energy and cofactors metabolism</p>
      <p/>
    </body>
  </notes>
  <listOfReactants>
    <speciesReference species="M_DM_DR_LUM_c" stoichiometry="2"/>
  </listOfReactants>
  <listOfProducts>
    <speciesReference species="M_RBFLV_c" stoichiometry="1"/>
    <speciesReference species="M_5AM_RIBAM_URA_c" stoichiometry="1"/>
  </listOfProducts>
  <kineticLaw>
    <math xmlns="http://www.w3.org/1998/Math/MathML">
      <ci> FLUX_VALUE </ci>
    </math>
    <listOfParameters>
      <parameter id="LOWER_BOUND" value="0" units="mmol_per_gDW_per_hr" constant="true"/>
      <parameter id="UPPER_BOUND" value="1000" units="mmol_per_gDW_per_hr" constant="true"/>
      <parameter id="FLUX_VALUE" value="0" units="mmol_per_gDW_per_hr" constant="true"/>
      <parameter id="OBJECTIVE_COEFFICIENT" value="0" units="mmol_per_gDW_per_hr" constant="true"/>
    </listOfParameters>
  </kineticLaw>
</reaction>
<reaction id="R_R598" name="NADPH quinone reductase" reversible="false">
  <notes>
```

```
<body xmlns="http://www.w3.org/1999/xhtml">
<p>GENE_ASSOCIATION: </p>
<p>SUBSYSTEM: Oxidative Phosphorylation</p>
<p>EC Number: 1.6.5.10</p>
<p>Confidence Level: 1</p>
<p>AUTHORS: Energy and cofactors metabolism</p>
<p/>
</body>
</notes>
<listOfReactants>
<speciesReference species="M_H_c" stoichiometry="1"/>
<speciesReference species="M_NADPH_c" stoichiometry="1"/>
<speciesReference species="M_Q_c" stoichiometry="1"/>
</listOfReactants>
<listOfProducts>
<speciesReference species="M_NADP_c" stoichiometry="1"/>
<speciesReference species="M_QH2_c" stoichiometry="1"/>
</listOfProducts>
<kineticLaw>
<math xmlns="http://www.w3.org/1998/Math/MathML">
<ci> FLUX_VALUE </ci>
</math>
<listOfParameters>
<parameter id="LOWER_BOUND" value="0" units="mmol_per_gDW_per_hr" constant="true"/>
<parameter id="UPPER_BOUND" value="1000" units="mmol_per_gDW_per_hr" constant="true"/>
<parameter id="FLUX_VALUE" value="0" units="mmol_per_gDW_per_hr" constant="true"/>
<parameter id="OBJECTIVE_COEFFICIENT" value="0" units="mmol_per_gDW_per_hr" constant="true"/>
</listOfParameters>
</kineticLaw>
```

```xml
</reaction>
<reaction id="R_R599" name="NADPH quinone reductase" reversible="false">
  <notes>
    <body xmlns="http://www.w3.org/1999/xhtml">
      <p>GENE_ASSOCIATION: </p>
      <p>SUBSYSTEM: Oxidative Phosphorylation</p>
      <p>EC Number: 1.6.5.10</p>
      <p>Confidence Level: 1</p>
      <p>AUTHORS: Energy and cofactors metabolism</p>
      <p/>
    </body>
  </notes>
  <listOfReactants>
    <speciesReference species="M_H_c" stoichiometry="1"/>
    <speciesReference species="M_NADPH_c" stoichiometry="1"/>
    <speciesReference species="M_MQ_c" stoichiometry="1"/>
  </listOfReactants>
  <listOfProducts>
    <speciesReference species="M_NADP_c" stoichiometry="1"/>
    <speciesReference species="M_MQL_c" stoichiometry="1"/>
  </listOfProducts>
  <kineticLaw>
    <math xmlns="http://www.w3.org/1998/Math/MathML">
      <ci> FLUX_VALUE </ci>
    </math>
    <listOfParameters>
      <parameter id="LOWER_BOUND" value="0" units="mmol_per_gDW_per_hr" constant="true"/>
      <parameter id="UPPER_BOUND" value="1000" units="mmol_per_gDW_per_hr" constant="true"/>
      <parameter id="FLUX_VALUE" value="0" units="mmol_per_gDW_per_hr" constant="true"/>
```

```
      <parameter id="OBJECTIVE_COEFFICIENT" value="0" units="mmol_per_gDW_per_hr" constant="true"/>
    </listOfParameters>
  </kineticLaw>
</reaction>
<reaction id="R_R600" name="2-dehydropantoate 2-reductase" reversible="false">
  <notes>
    <body xmlns="http://www.w3.org/1999/xhtml">
      <p>GENE_ASSOCIATION: </p>
      <p>SUBSYSTEM: Cofactor and Prosthetic Group Biosynthesis</p>
      <p>EC Number: 1.1.1.169</p>
      <p>Confidence Level: 1</p>
      <p>AUTHORS: Energy and cofactors metabolism</p>
      <p/>
    </body>
  </notes>
  <listOfReactants>
    <speciesReference species="M_H_c" stoichiometry="1"/>
    <speciesReference species="M_NADPH_c" stoichiometry="1"/>
    <speciesReference species="M_DHY_PAN_c" stoichiometry="1"/>
  </listOfReactants>
  <listOfProducts>
    <speciesReference species="M_NADP_c" stoichiometry="1"/>
    <speciesReference species="M_PANTA_c" stoichiometry="1"/>
  </listOfProducts>
  <kineticLaw>
    <math xmlns="http://www.w3.org/1998/Math/MathML">
      <ci> FLUX_VALUE </ci>
    </math>
    <listOfParameters>
```

```xml
      <parameter id="LOWER_BOUND" value="0" units="mmol_per_gDW_per_hr" constant="true"/>
      <parameter id="UPPER_BOUND" value="1000" units="mmol_per_gDW_per_hr" constant="true"/>
      <parameter id="FLUX_VALUE" value="0" units="mmol_per_gDW_per_hr" constant="true"/>
      <parameter id="OBJECTIVE_COEFFICIENT" value="0" units="mmol_per_gDW_per_hr" constant="true"/>
    </listOfParameters>
  </kineticLaw>
</reaction>
<reaction id="R_R601" name="adenine phosphoribosyltransferase" reversible="false">
  <notes>
    <body xmlns="http://www.w3.org/1999/xhtml">
      <p>GENE_ASSOCIATION: (TTC1249 or TTC1250)</p>
      <p>SUBSYSTEM: Nucleotide Salvage Pathway</p>
      <p>EC Number: 2.4.2.7</p>
      <p>Confidence Level: 1</p>
      <p>AUTHORS: Nucleotides metabolism</p>
      <p/>
    </body>
  </notes>
  <listOfReactants>
    <speciesReference species="M_PRPP_c" stoichiometry="1"/>
    <speciesReference species="M_ADN_c" stoichiometry="1"/>
  </listOfReactants>
  <listOfProducts>
    <speciesReference species="M_PPi_c" stoichiometry="1"/>
    <speciesReference species="M_AMP_c" stoichiometry="1"/>
  </listOfProducts>
  <kineticLaw>
    <math xmlns="http://www.w3.org/1998/Math/MathML">
      <ci> FLUX_VALUE </ci>
```

```
      </math>
      <listOfParameters>
        <parameter id="LOWER_BOUND" value="0" units="mmol_per_gDW_per_hr" constant="true"/>
        <parameter id="UPPER_BOUND" value="1000" units="mmol_per_gDW_per_hr" constant="true"/>
        <parameter id="FLUX_VALUE" value="0" units="mmol_per_gDW_per_hr" constant="true"/>
        <parameter id="OBJECTIVE_COEFFICIENT" value="0" units="mmol_per_gDW_per_hr" constant="true"/>
      </listOfParameters>
    </kineticLaw>
  </reaction>
  <reaction id="R_R602" name="cobalamin [5&apos;-phosphate] synthase" reversible="false">
    <notes>
      <body xmlns="http://www.w3.org/1999/xhtml">
        <p>GENE_ASSOCIATION: TT_P0004 </p>
        <p>SUBSYSTEM: Nucleotide Salvage Pathway</p>
        <p>EC Number: 2.7.8.26</p>
        <p>Confidence Level: 1</p>
        <p>AUTHORS: Nucleotides metabolism</p>
        <p/>
      </body>
    </notes>
    <listOfReactants>
      <speciesReference species="M_H_c" stoichiometry="1"/>
      <speciesReference species="M_PRIBS_DMBENZ_IMDZ_c" stoichiometry="1"/>
      <speciesReference species="M_ADNS_CBNA_GDP_c" stoichiometry="1"/>
    </listOfReactants>
    <listOfProducts>
      <speciesReference species="M_GMP_c" stoichiometry="1"/>
      <speciesReference species="M_CBMCo_c" stoichiometry="1"/>
    </listOfProducts>
```

```
  <kineticLaw>
   <math xmlns="http://www.w3.org/1998/Math/MathML">
    <ci> FLUX_VALUE </ci>
   </math>
   <listOfParameters>
    <parameter id="LOWER_BOUND" value="0" units="mmol_per_gDW_per_hr" constant="true"/>
    <parameter id="UPPER_BOUND" value="1000" units="mmol_per_gDW_per_hr" constant="true"/>
    <parameter id="FLUX_VALUE" value="0" units="mmol_per_gDW_per_hr" constant="true"/>
    <parameter id="OBJECTIVE_COEFFICIENT" value="0" units="mmol_per_gDW_per_hr" constant="true"/>
   </listOfParameters>
  </kineticLaw>
 </reaction>
 <reaction id="R_R603" name="GMP synthase" reversible="false">
  <notes>
   <body xmlns="http://www.w3.org/1999/xhtml">
    <p>GENE_ASSOCIATION: (TTC0355 or TTC1187)</p>
    <p>SUBSYSTEM: Nucleotide Salvage Pathway</p>
    <p>EC Number: 6.3.5.2</p>
    <p>Confidence Level: 1</p>
    <p>AUTHORS: Nucleotides metabolism</p>
    <p/>
   </body>
  </notes>
  <listOfReactants>
   <speciesReference species="M_L_GLN_c" stoichiometry="1"/>
   <speciesReference species="M_ATP_c" stoichiometry="1"/>
   <speciesReference species="M_H2O_c" stoichiometry="1"/>
   <speciesReference species="M_XMP_c" stoichiometry="1"/>
  </listOfReactants>
```

```xml
<listOfProducts>
  <speciesReference species="M_L_GLU_c" stoichiometry="1"/>
  <speciesReference species="M_PPi_c" stoichiometry="1"/>
  <speciesReference species="M_H_c" stoichiometry="2"/>
  <speciesReference species="M_AMP_c" stoichiometry="1"/>
  <speciesReference species="M_GMP_c" stoichiometry="1"/>
</listOfProducts>
<kineticLaw>
  <math xmlns="http://www.w3.org/1998/Math/MathML">
    <ci> FLUX_VALUE </ci>
  </math>
  <listOfParameters>
    <parameter id="LOWER_BOUND" value="0" units="mmol_per_gDW_per_hr" constant="true"/>
    <parameter id="UPPER_BOUND" value="1000" units="mmol_per_gDW_per_hr" constant="true"/>
    <parameter id="FLUX_VALUE" value="0" units="mmol_per_gDW_per_hr" constant="true"/>
    <parameter id="OBJECTIVE_COEFFICIENT" value="0" units="mmol_per_gDW_per_hr" constant="true"/>
  </listOfParameters>
</kineticLaw>
</reaction>
<reaction id="R_R604" reversible="false">
<notes>
  <body xmlns="http://www.w3.org/1999/xhtml">
    <p>GENE_ASSOCIATION: </p>
    <p>SUBSYSTEM: Nucleotide Salvage Pathway</p>
    <p>EC Number: </p>
    <p>Confidence Level: 1</p>
    <p>AUTHORS: Nucleotides metabolism</p>
    <p/>
  </body>
```

```
  </notes>
  <listOfReactants>
   <speciesReference species="M_L_GLN_c" stoichiometry="1"/>
   <speciesReference species="M_ATP_c" stoichiometry="1"/>
   <speciesReference species="M_H2O_c" stoichiometry="1"/>
   <speciesReference species="M_XMP_c" stoichiometry="1"/>
  </listOfReactants>
  <listOfProducts>
   <speciesReference species="M_L_GLU_c" stoichiometry="1"/>
   <speciesReference species="M_L_ASN_c" stoichiometry="1"/>
   <speciesReference species="M_PPi_c" stoichiometry="1"/>
   <speciesReference species="M_H_c" stoichiometry="1"/>
   <speciesReference species="M_AMP_c" stoichiometry="1"/>
  </listOfProducts>
  <kineticLaw>
   <math xmlns="http://www.w3.org/1998/Math/MathML">
    <ci> FLUX_VALUE </ci>
   </math>
   <listOfParameters>
    <parameter id="LOWER_BOUND" value="0" units="mmol_per_gDW_per_hr" constant="true"/>
    <parameter id="UPPER_BOUND" value="1000" units="mmol_per_gDW_per_hr" constant="true"/>
    <parameter id="FLUX_VALUE" value="0" units="mmol_per_gDW_per_hr" constant="true"/>
    <parameter id="OBJECTIVE_COEFFICIENT" value="0" units="mmol_per_gDW_per_hr" constant="true"/>
   </listOfParameters>
  </kineticLaw>
 </reaction>
 <reaction id="R_R605" name="GTP cyclohydrolase I" reversible="false">
  <notes>
   <body xmlns="http://www.w3.org/1999/xhtml">
```

```
      <p>GENE_ASSOCIATION: TTC1517</p>
      <p>SUBSYSTEM: Nucleotide Salvage Pathway</p>
      <p>EC Number: 3.5.4.16</p>
      <p>Confidence Level: 1</p>
      <p>AUTHORS: Nucleotides metabolism</p>
      <p/>
    </body>
  </notes>
  <listOfReactants>
    <speciesReference species="M_GTP_c" stoichiometry="1"/>
    <speciesReference species="M_H2O_c" stoichiometry="1"/>
  </listOfReactants>
  <listOfProducts>
    <speciesReference species="M_H_c" stoichiometry="1"/>
    <speciesReference species="M_FOR_c" stoichiometry="1"/>
    <speciesReference species="M_AHYRY_THYPROP_DHYPTRN_TPi_c" stoichiometry="1"/>
  </listOfProducts>
  <kineticLaw>
    <math xmlns="http://www.w3.org/1998/Math/MathML">
      <ci> FLUX_VALUE </ci>
    </math>
    <listOfParameters>
      <parameter id="LOWER_BOUND" value="0" units="mmol_per_gDW_per_hr" constant="true"/>
      <parameter id="UPPER_BOUND" value="1000" units="mmol_per_gDW_per_hr" constant="true"/>
      <parameter id="FLUX_VALUE" value="0" units="mmol_per_gDW_per_hr" constant="true"/>
      <parameter id="OBJECTIVE_COEFFICIENT" value="0" units="mmol_per_gDW_per_hr" constant="true"/>
    </listOfParameters>
  </kineticLaw>
</reaction>
```

```xml
<reaction id="R_R606" name="mannose-1-phosphate guanylyltransferase" reversible="false">
 <notes>
  <body xmlns="http://www.w3.org/1999/xhtml">
   <p>GENE_ASSOCIATION: TTC1388</p>
   <p>SUBSYSTEM: Alternate Carbon Metabolism</p>
   <p>EC Number: 2.7.7.13</p>
   <p>Confidence Level: 1</p>
   <p>AUTHORS: Carbohydrate metabolism</p>
   <p/>
  </body>
 </notes>
 <listOfReactants>
  <speciesReference species="M_GTP_c" stoichiometry="1"/>
  <speciesReference species="M_H_c" stoichiometry="1"/>
  <speciesReference species="M_MAN1P_c" stoichiometry="1"/>
 </listOfReactants>
 <listOfProducts>
  <speciesReference species="M_PPi_c" stoichiometry="1"/>
  <speciesReference species="M_GDP_MAN_c" stoichiometry="1"/>
 </listOfProducts>
 <kineticLaw>
  <math xmlns="http://www.w3.org/1998/Math/MathML">
   <ci> FLUX_VALUE </ci>
  </math>
  <listOfParameters>
   <parameter id="LOWER_BOUND" value="0" units="mmol_per_gDW_per_hr" constant="true"/>
   <parameter id="UPPER_BOUND" value="1000" units="mmol_per_gDW_per_hr" constant="true"/>
   <parameter id="FLUX_VALUE" value="0" units="mmol_per_gDW_per_hr" constant="true"/>
   <parameter id="OBJECTIVE_COEFFICIENT" value="0" units="mmol_per_gDW_per_hr" constant="true"/>
```

```
    </listOfParameters>
  </kineticLaw>
</reaction>
<reaction id="R_R607" name="GTPase" reversible="true">
  <notes>
    <body xmlns="http://www.w3.org/1999/xhtml">
      <p>GENE_ASSOCIATION: TTC1158</p>
      <p>SUBSYSTEM: Nucleotide Salvage Pathway</p>
      <p>EC Number: 3.6.5.-</p>
      <p>Confidence Level: 1</p>
      <p>AUTHORS: Nucleotides metabolism</p>
      <p/>
    </body>
  </notes>
  <listOfReactants>
    <speciesReference species="M_GTP_c" stoichiometry="1"/>
    <speciesReference species="M_H2O_c" stoichiometry="1"/>
  </listOfReactants>
  <listOfProducts>
    <speciesReference species="M_Pi_c" stoichiometry="1"/>
    <speciesReference species="M_H_c" stoichiometry="1"/>
    <speciesReference species="M_GDP_c" stoichiometry="1"/>
  </listOfProducts>
  <kineticLaw>
    <math xmlns="http://www.w3.org/1998/Math/MathML">
      <ci> FLUX_VALUE </ci>
    </math>
    <listOfParameters>
      <parameter id="LOWER_BOUND" value="-1000" units="mmol_per_gDW_per_hr" constant="true"/>
```

```
      <parameter id="UPPER_BOUND" value="1000" units="mmol_per_gDW_per_hr" constant="true"/>
      <parameter id="FLUX_VALUE" value="0" units="mmol_per_gDW_per_hr" constant="true"/>
      <parameter id="OBJECTIVE_COEFFICIENT" value="0" units="mmol_per_gDW_per_hr" constant="true"/>
    </listOfParameters>
  </kineticLaw>
</reaction>
<reaction id="R_R608" name="phosphoribosylaminoimidazole carboxylase" reversible="false">
  <notes>
    <body xmlns="http://www.w3.org/1999/xhtml">
      <p>GENE_ASSOCIATION: (TTC0137 and TTC0138)</p>
      <p>SUBSYSTEM: Nucleotide Salvage Pathway</p>
      <p>EC Number: 4.1.1.21</p>
      <p>Confidence Level: 1</p>
      <p>AUTHORS: Nucleotides metabolism</p>
      <p/>
    </body>
  </notes>
  <listOfReactants>
    <speciesReference species="M_CO2_c" stoichiometry="1"/>
    <speciesReference species="M_AIR_c" stoichiometry="1"/>
  </listOfReactants>
  <listOfProducts>
    <speciesReference species="M_H_c" stoichiometry="2"/>
    <speciesReference species="M_CAIR_c" stoichiometry="1"/>
  </listOfProducts>
  <kineticLaw>
    <math xmlns="http://www.w3.org/1998/Math/MathML">
      <ci> FLUX_VALUE </ci>
    </math>
```

```
      <listOfParameters>
        <parameter id="LOWER_BOUND" value="0" units="mmol_per_gDW_per_hr" constant="true"/>
        <parameter id="UPPER_BOUND" value="1000" units="mmol_per_gDW_per_hr" constant="true"/>
        <parameter id="FLUX_VALUE" value="0" units="mmol_per_gDW_per_hr" constant="true"/>
        <parameter id="OBJECTIVE_COEFFICIENT" value="0" units="mmol_per_gDW_per_hr" constant="true"/>
      </listOfParameters>
    </kineticLaw>
  </reaction>
  <reaction id="R_R609" name="adenosylcobinamide-phosphate synthase" reversible="true">
    <notes>
      <body xmlns="http://www.w3.org/1999/xhtml">
        <p>GENE_ASSOCIATION: TT_P0019</p>
        <p>SUBSYSTEM: Cofactor and Prosthetic Group Biosynthesis</p>
        <p>EC Number: 6.3.1.10</p>
        <p>Confidence Level: 1</p>
        <p>AUTHORS: Energy and cofactors metabolism</p>
        <p/>
      </body>
    </notes>
    <listOfReactants>
      <speciesReference species="M_ATP_c" stoichiometry="1"/>
      <speciesReference species="M_ADNS_CABYRHA_c" stoichiometry="1"/>
      <speciesReference species="M_A_PROPOL_c" stoichiometry="1"/>
    </listOfReactants>
    <listOfProducts>
      <speciesReference species="M_ADP_c" stoichiometry="1"/>
      <speciesReference species="M_Pi_c" stoichiometry="1"/>
      <speciesReference species="M_H_c" stoichiometry="1"/>
      <speciesReference species="M_ADNS_CBNAPi_c" stoichiometry="1"/>
```

```xml
</listOfProducts>
<kineticLaw>
<math xmlns="http://www.w3.org/1998/Math/MathML">
<ci> FLUX_VALUE </ci>
</math>
<listOfParameters>
<parameter id="LOWER_BOUND" value="-1000" units="mmol_per_gDW_per_hr" constant="true"/>
<parameter id="UPPER_BOUND" value="1000" units="mmol_per_gDW_per_hr" constant="true"/>
<parameter id="FLUX_VALUE" value="0" units="mmol_per_gDW_per_hr" constant="true"/>
<parameter id="OBJECTIVE_COEFFICIENT" value="0" units="mmol_per_gDW_per_hr" constant="true"/>
</listOfParameters>
</kineticLaw>
</reaction>
<reaction id="R_R610" name="2-keto-3-deoxy-gluconate kinase" reversible="false">
<notes>
<body xmlns="http://www.w3.org/1999/xhtml">
<p>GENE_ASSOCIATION: TT_P0036</p>
<p>SUBSYSTEM: Alternate Carbon Metabolism</p>
<p>EC Number: 2.7.1.45</p>
<p>Confidence Level: 1</p>
<p>AUTHORS: Carbohydrate metabolism</p>
<p/>
</body>
</notes>
<listOfReactants>
<speciesReference species="M_ATP_c" stoichiometry="1"/>
<speciesReference species="M_2K3DO_GLCN_c" stoichiometry="1"/>
</listOfReactants>
<listOfProducts>
```

```
      <speciesReference species="M_ADP_c" stoichiometry="1"/>
      <speciesReference species="M_H_c" stoichiometry="1"/>
      <speciesReference species="M_DHY_DOP_GLUCN_c" stoichiometry="1"/>
    </listOfProducts>
    <kineticLaw>
      <math xmlns="http://www.w3.org/1998/Math/MathML">
        <ci> FLUX_VALUE </ci>
      </math>
      <listOfParameters>
        <parameter id="LOWER_BOUND" value="0" units="mmol_per_gDW_per_hr" constant="true"/>
        <parameter id="UPPER_BOUND" value="1000" units="mmol_per_gDW_per_hr" constant="true"/>
        <parameter id="FLUX_VALUE" value="0" units="mmol_per_gDW_per_hr" constant="true"/>
        <parameter id="OBJECTIVE_COEFFICIENT" value="0" units="mmol_per_gDW_per_hr" constant="true"/>
      </listOfParameters>
    </kineticLaw>
  </reaction>
  <reaction id="R_R611" name="2-phosphoglycerate kinase" reversible="false">
    <notes>
      <body xmlns="http://www.w3.org/1999/xhtml">
        <p>GENE_ASSOCIATION: TTC0121</p>
        <p>SUBSYSTEM: Glycine and Serine Metabolism</p>
        <p>EC Number: 2.7.1.165</p>
        <p>Confidence Level: 1</p>
        <p>AUTHORS: Amino acid metabolism</p>
        <p/>
      </body>
    </notes>
    <listOfReactants>
      <speciesReference species="M_ATP_c" stoichiometry="1"/>
```

```
      <speciesReference species="M_D_GLCT_c" stoichiometry="1"/>
    </listOfReactants>
    <listOfProducts>
      <speciesReference species="M_ADP_c" stoichiometry="1"/>
      <speciesReference species="M_H_c" stoichiometry="1"/>
      <speciesReference species="M_2PG_c" stoichiometry="1"/>
    </listOfProducts>
    <kineticLaw>
      <math xmlns="http://www.w3.org/1998/Math/MathML">
        <ci> FLUX_VALUE </ci>
      </math>
      <listOfParameters>
        <parameter id="LOWER_BOUND" value="0" units="mmol_per_gDW_per_hr" constant="true"/>
        <parameter id="UPPER_BOUND" value="1000" units="mmol_per_gDW_per_hr" constant="true"/>
        <parameter id="FLUX_VALUE" value="0" units="mmol_per_gDW_per_hr" constant="true"/>
        <parameter id="OBJECTIVE_COEFFICIENT" value="0" units="mmol_per_gDW_per_hr" constant="true"/>
      </listOfParameters>
    </kineticLaw>
  </reaction>
  <reaction id="R_R612" name="Propionate-CoA ligase" reversible="false">
    <notes>
      <body xmlns="http://www.w3.org/1999/xhtml">
        <p>GENE_ASSOCIATION: TTC0884</p>
        <p>SUBSYSTEM: Valine, Leucine, and Isoleucine Metabolism</p>
        <p>EC Number: 6.2.1.17</p>
        <p>Confidence Level: 1</p>
        <p>AUTHORS: Amino acid metabolism</p>
        <p/>
      </body>
```

```
  </notes>
  <listOfReactants>
    <speciesReference species="M_ATP_c" stoichiometry="1"/>
    <speciesReference species="M_CoA_c" stoichiometry="1"/>
    <speciesReference species="M_PROP_c" stoichiometry="1"/>
  </listOfReactants>
  <listOfProducts>
    <speciesReference species="M_PPi_c" stoichiometry="1"/>
    <speciesReference species="M_AMP_c" stoichiometry="1"/>
    <speciesReference species="M_PRCoA_c" stoichiometry="1"/>
  </listOfProducts>
  <kineticLaw>
    <math xmlns="http://www.w3.org/1998/Math/MathML">
      <ci> FLUX_VALUE </ci>
    </math>
    <listOfParameters>
      <parameter id="LOWER_BOUND" value="0" units="mmol_per_gDW_per_hr" constant="true"/>
      <parameter id="UPPER_BOUND" value="1000" units="mmol_per_gDW_per_hr" constant="true"/>
      <parameter id="FLUX_VALUE" value="0" units="mmol_per_gDW_per_hr" constant="true"/>
      <parameter id="OBJECTIVE_COEFFICIENT" value="0" units="mmol_per_gDW_per_hr" constant="true"/>
    </listOfParameters>
  </kineticLaw>
</reaction>
<reaction id="R_R613" name="NH(3)-dependent NAD(+) synthetase" reversible="false">
  <notes>
    <body xmlns="http://www.w3.org/1999/xhtml">
      <p>GENE_ASSOCIATION: TTC1538</p>
      <p>SUBSYSTEM: Cofactor and Prosthetic Group Biosynthesis</p>
      <p>EC Number: 6.3.5.1</p>
```

```xml
<p>Confidence Level: 1</p>
<p>AUTHORS: Energy and cofactors metabolism</p>
<p/>
</body>
</notes>
<listOfReactants>
<speciesReference species="M_L_GLN_c" stoichiometry="1"/>
<speciesReference species="M_ATP_c" stoichiometry="1"/>
<speciesReference species="M_H2O_c" stoichiometry="1"/>
<speciesReference species="M_DA_NAD_c" stoichiometry="1"/>
</listOfReactants>
<listOfProducts>
<speciesReference species="M_L_GLU_c" stoichiometry="1"/>
<speciesReference species="M_PPi_c" stoichiometry="1"/>
<speciesReference species="M_H_c" stoichiometry="1"/>
<speciesReference species="M_NAD_c" stoichiometry="1"/>
<speciesReference species="M_AMP_c" stoichiometry="1"/>
</listOfProducts>
<kineticLaw>
<math xmlns="http://www.w3.org/1998/Math/MathML">
<ci> FLUX_VALUE </ci>
</math>
<listOfParameters>
<parameter id="LOWER_BOUND" value="0" units="mmol_per_gDW_per_hr" constant="true"/>
<parameter id="UPPER_BOUND" value="1000" units="mmol_per_gDW_per_hr" constant="true"/>
<parameter id="FLUX_VALUE" value="0" units="mmol_per_gDW_per_hr" constant="true"/>
<parameter id="OBJECTIVE_COEFFICIENT" value="0" units="mmol_per_gDW_per_hr" constant="true"/>
</listOfParameters>
</kineticLaw>
```

```
</reaction>
<reaction id="R_R614" name="NAD+ kinase" reversible="false">
 <notes>
  <body xmlns="http://www.w3.org/1999/xhtml">
   <p>GENE_ASSOCIATION: </p>
   <p>SUBSYSTEM: Cofactor and Prosthetic Group Biosynthesis</p>
   <p>EC Number: 2.7.1.23</p>
   <p>Confidence Level: 1</p>
   <p>AUTHORS: Energy and cofactors metabolism</p>
   <p/>
  </body>
 </notes>
 <listOfReactants>
  <speciesReference species="M_ATP_c" stoichiometry="1"/>
  <speciesReference species="M_NAD_c" stoichiometry="1"/>
 </listOfReactants>
 <listOfProducts>
  <speciesReference species="M_ADP_c" stoichiometry="1"/>
  <speciesReference species="M_H_c" stoichiometry="1"/>
  <speciesReference species="M_NADP_c" stoichiometry="1"/>
 </listOfProducts>
 <kineticLaw>
  <math xmlns="http://www.w3.org/1998/Math/MathML">
   <ci> FLUX_VALUE </ci>
  </math>
  <listOfParameters>
   <parameter id="LOWER_BOUND" value="0" units="mmol_per_gDW_per_hr" constant="true"/>
   <parameter id="UPPER_BOUND" value="1000" units="mmol_per_gDW_per_hr" constant="true"/>
   <parameter id="FLUX_VALUE" value="0" units="mmol_per_gDW_per_hr" constant="true"/>
```

```
<parameter id="OBJECTIVE_COEFFICIENT" value="0" units="mmol_per_gDW_per_hr" constant="true"/>
</listOfParameters>
</kineticLaw>
</reaction>
<reaction id="R_R615" name="acid phosphatase" reversible="false">
<notes>
<body xmlns="http://www.w3.org/1999/xhtml">
<p>GENE_ASSOCIATION: TTC1252</p>
<p>SUBSYSTEM: Cofactor and Prosthetic Group Biosynthesis</p>
<p>EC Number: 3.1.3.2</p>
<p>Confidence Level: 1</p>
<p>AUTHORS: Energy and cofactors metabolism</p>
<p/>
</body>
</notes>
<listOfReactants>
<speciesReference species="M_H2O_c" stoichiometry="1"/>
<speciesReference species="M_NADP_c" stoichiometry="1"/>
</listOfReactants>
<listOfProducts>
<speciesReference species="M_Pi_c" stoichiometry="1"/>
<speciesReference species="M_NAD_c" stoichiometry="1"/>
</listOfProducts>
<kineticLaw>
<math xmlns="http://www.w3.org/1998/Math/MathML">
<ci> FLUX_VALUE </ci>
</math>
<listOfParameters>
<parameter id="LOWER_BOUND" value="0" units="mmol_per_gDW_per_hr" constant="true"/>
```

```
      <parameter id="UPPER_BOUND" value="1000" units="mmol_per_gDW_per_hr" constant="true"/>
      <parameter id="FLUX_VALUE" value="0" units="mmol_per_gDW_per_hr" constant="true"/>
      <parameter id="OBJECTIVE_COEFFICIENT" value="0" units="mmol_per_gDW_per_hr" constant="true"/>
    </listOfParameters>
  </kineticLaw>
</reaction>
<reaction id="R_R616" name="thymidine kinase" reversible="false">
  <notes>
    <body xmlns="http://www.w3.org/1999/xhtml">
      <p>GENE_ASSOCIATION: </p>
      <p>SUBSYSTEM: Nucleotide Salvage Pathway</p>
      <p>EC Number: 2.7.1.21</p>
      <p>Confidence Level: 1</p>
      <p>AUTHORS: Nucleotides metabolism</p>
      <p/>
    </body>
  </notes>
  <listOfReactants>
    <speciesReference species="M_ATP_c" stoichiometry="1"/>
    <speciesReference species="M_THYMD_c" stoichiometry="1"/>
  </listOfReactants>
  <listOfProducts>
    <speciesReference species="M_ADP_c" stoichiometry="1"/>
    <speciesReference species="M_H_c" stoichiometry="1"/>
    <speciesReference species="M_dTMP_c" stoichiometry="1"/>
  </listOfProducts>
  <kineticLaw>
    <math xmlns="http://www.w3.org/1998/Math/MathML">
      <ci> FLUX_VALUE </ci>
```

```xml
    </math>
    <listOfParameters>
      <parameter id="LOWER_BOUND" value="0" units="mmol_per_gDW_per_hr" constant="true"/>
      <parameter id="UPPER_BOUND" value="1000" units="mmol_per_gDW_per_hr" constant="true"/>
      <parameter id="FLUX_VALUE" value="0" units="mmol_per_gDW_per_hr" constant="true"/>
      <parameter id="OBJECTIVE_COEFFICIENT" value="0" units="mmol_per_gDW_per_hr" constant="true"/>
    </listOfParameters>
  </kineticLaw>
</reaction>
<reaction id="R_R617" name="adenylyl-sulfate kinase" reversible="false">
  <notes>
    <body xmlns="http://www.w3.org/1999/xhtml">
      <p>GENE_ASSOCIATION: TTC0307</p>
      <p>SUBSYSTEM: Cysteine and methionine metabolism</p>
      <p>EC Number: 2.7.1.25</p>
      <p>Confidence Level: 1</p>
      <p>AUTHORS: Amino acid metabolism</p>
      <p/>
    </body>
  </notes>
  <listOfReactants>
    <speciesReference species="M_ATP_c" stoichiometry="1"/>
    <speciesReference species="M_APS_c" stoichiometry="1"/>
  </listOfReactants>
  <listOfProducts>
    <speciesReference species="M_ADP_c" stoichiometry="1"/>
    <speciesReference species="M_H_c" stoichiometry="1"/>
    <speciesReference species="M_PAPS_c" stoichiometry="1"/>
  </listOfProducts>
```

```
<kineticLaw>
  <math xmlns="http://www.w3.org/1998/Math/MathML">
    <ci> FLUX_VALUE </ci>
  </math>
  <listOfParameters>
    <parameter id="LOWER_BOUND" value="0" units="mmol_per_gDW_per_hr" constant="true"/>
    <parameter id="UPPER_BOUND" value="1000" units="mmol_per_gDW_per_hr" constant="true"/>
    <parameter id="FLUX_VALUE" value="0" units="mmol_per_gDW_per_hr" constant="true"/>
    <parameter id="OBJECTIVE_COEFFICIENT" value="0" units="mmol_per_gDW_per_hr" constant="true"/>
  </listOfParameters>
</kineticLaw>
</reaction>
<reaction id="R_R618" name="urea carboxylase/allophanate hydrolase" reversible="false">
<notes>
  <body xmlns="http://www.w3.org/1999/xhtml">
    <p>GENE_ASSOCIATION: TTC0624</p>
    <p>SUBSYSTEM: Arginine and Proline Metabolism</p>
    <p>EC Number: 6.3.4.6</p>
    <p>Confidence Level: 1</p>
    <p>AUTHORS: Amino acid metabolism</p>
    <p/>
  </body>
</notes>
<listOfReactants>
  <speciesReference species="M_ATP_c" stoichiometry="1"/>
  <speciesReference species="M_H2O_c" stoichiometry="1"/>
  <speciesReference species="M_CO2_c" stoichiometry="1"/>
  <speciesReference species="M_UREA_c" stoichiometry="1"/>
</listOfReactants>
```

```
  <listOfProducts>
   <speciesReference species="M_ADP_c" stoichiometry="1"/>
   <speciesReference species="M_Pi_c" stoichiometry="1"/>
   <speciesReference species="M_H_c" stoichiometry="2"/>
   <speciesReference species="M_UREA_CARB_c" stoichiometry="1"/>
  </listOfProducts>
  <kineticLaw>
   <math xmlns="http://www.w3.org/1998/Math/MathML">
    <ci> FLUX_VALUE </ci>
   </math>
   <listOfParameters>
    <parameter id="LOWER_BOUND" value="0" units="mmol_per_gDW_per_hr" constant="true"/>
    <parameter id="UPPER_BOUND" value="1000" units="mmol_per_gDW_per_hr" constant="true"/>
    <parameter id="FLUX_VALUE" value="0" units="mmol_per_gDW_per_hr" constant="true"/>
    <parameter id="OBJECTIVE_COEFFICIENT" value="0" units="mmol_per_gDW_per_hr" constant="true"/>
   </listOfParameters>
  </kineticLaw>
 </reaction>
 <reaction id="R_R619" name="carbamate kinase" reversible="false">
  <notes>
   <body xmlns="http://www.w3.org/1999/xhtml">
    <p>GENE_ASSOCIATION: </p>
    <p>SUBSYSTEM: Unassigned</p>
    <p>EC Number: 2.7.2.2</p>
    <p>Confidence Level: 1</p>
    <p>AUTHORS: Energy and cofactors metabolism</p>
    <p/>
   </body>
  </notes>
```

```
  <listOfReactants>
    <speciesReference species="M_ADP_c" stoichiometry="1"/>
    <speciesReference species="M_H_c" stoichiometry="1"/>
    <speciesReference species="M_CARB_Pi_c" stoichiometry="1"/>
  </listOfReactants>
  <listOfProducts>
    <speciesReference species="M_ATP_c" stoichiometry="1"/>
    <speciesReference species="M_CO2_c" stoichiometry="1"/>
    <speciesReference species="M_NH4_c" stoichiometry="1"/>
  </listOfProducts>
  <kineticLaw>
    <math xmlns="http://www.w3.org/1998/Math/MathML">
      <ci> FLUX_VALUE </ci>
    </math>
    <listOfParameters>
      <parameter id="LOWER_BOUND" value="0" units="mmol_per_gDW_per_hr" constant="true"/>
      <parameter id="UPPER_BOUND" value="1000" units="mmol_per_gDW_per_hr" constant="true"/>
      <parameter id="FLUX_VALUE" value="0" units="mmol_per_gDW_per_hr" constant="true"/>
      <parameter id="OBJECTIVE_COEFFICIENT" value="0" units="mmol_per_gDW_per_hr" constant="true"/>
    </listOfParameters>
  </kineticLaw>
</reaction>
<reaction id="R_R620" name="acetate kinase" reversible="false">
  <notes>
    <body xmlns="http://www.w3.org/1999/xhtml">
      <p>GENE_ASSOCIATION: TTC0137</p>
      <p>SUBSYSTEM: Pyruvate Metabolism</p>
      <p>EC Number: 2.7.2.1</p>
      <p>Confidence Level: 1</p>
```

```
      <p>AUTHORS: Carbohydrate metabolism</p>
      <p/>
    </body>
  </notes>
  <listOfReactants>
    <speciesReference species="M_ADP_c" stoichiometry="1"/>
    <speciesReference species="M_ACPi_c" stoichiometry="1"/>
  </listOfReactants>
  <listOfProducts>
    <speciesReference species="M_ATP_c" stoichiometry="1"/>
    <speciesReference species="M_AC_c" stoichiometry="1"/>
  </listOfProducts>
  <kineticLaw>
    <math xmlns="http://www.w3.org/1998/Math/MathML">
      <ci> FLUX_VALUE </ci>
    </math>
    <listOfParameters>
      <parameter id="LOWER_BOUND" value="0" units="mmol_per_gDW_per_hr" constant="true"/>
      <parameter id="UPPER_BOUND" value="0" units="mmol_per_gDW_per_hr" constant="true"/>
      <parameter id="FLUX_VALUE" value="0" units="mmol_per_gDW_per_hr" constant="true"/>
      <parameter id="OBJECTIVE_COEFFICIENT" value="0" units="mmol_per_gDW_per_hr" constant="true"/>
    </listOfParameters>
  </kineticLaw>
</reaction>
<reaction id="R_R621" name="phosphoenolpyruvate carboxylase" reversible="true">
  <notes>
    <body xmlns="http://www.w3.org/1999/xhtml">
      <p>GENE_ASSOCIATION: TTC0260</p>
      <p>SUBSYSTEM: Glycolysis/Gluconeogenesis</p>
```

```xml
<p>EC Number: 4.1.1.31</p>
<p>Confidence Level: 1</p>
<p>AUTHORS: Carbohydrate metabolism</p>
<p/>
</body>
</notes>
<listOfReactants>
<speciesReference species="M_PEP_c" stoichiometry="1"/>
<speciesReference species="M_HCO3_c" stoichiometry="1"/>
</listOfReactants>
<listOfProducts>
<speciesReference species="M_Pi_c" stoichiometry="1"/>
<speciesReference species="M_OAC_c" stoichiometry="1"/>
</listOfProducts>
<kineticLaw>
<math xmlns="http://www.w3.org/1998/Math/MathML">
<ci> FLUX_VALUE </ci>
</math>
<listOfParameters>
<parameter id="LOWER_BOUND" value="-1000" units="mmol_per_gDW_per_hr" constant="true"/>
<parameter id="UPPER_BOUND" value="1000" units="mmol_per_gDW_per_hr" constant="true"/>
<parameter id="FLUX_VALUE" value="0" units="mmol_per_gDW_per_hr" constant="true"/>
<parameter id="OBJECTIVE_COEFFICIENT" value="0" units="mmol_per_gDW_per_hr" constant="true"/>
</listOfParameters>
</kineticLaw>
</reaction>
<reaction id="R_R622" name="dihydroorotate dehydrogenase (quinone)" reversible="false">
<notes>
<body xmlns="http://www.w3.org/1999/xhtml">
```

```
      <p>GENE_ASSOCIATION: TTC0616</p>
      <p>SUBSYSTEM: Nucleotide Salvage Pathway</p>
      <p>EC Number: 1.3.5.2</p>
      <p>Confidence Level: 1</p>
      <p>AUTHORS: Nucleotides metabolism</p>
      <p/>
    </body>
  </notes>
  <listOfReactants>
    <speciesReference species="M_Q_c" stoichiometry="1"/>
    <speciesReference species="M_DHY_ORTA_c" stoichiometry="1"/>
  </listOfReactants>
  <listOfProducts>
    <speciesReference species="M_QH2_c" stoichiometry="1"/>
    <speciesReference species="M_ORTA_c" stoichiometry="1"/>
  </listOfProducts>
  <kineticLaw>
    <math xmlns="http://www.w3.org/1998/Math/MathML">
      <ci> FLUX_VALUE </ci>
    </math>
    <listOfParameters>
      <parameter id="LOWER_BOUND" value="0" units="mmol_per_gDW_per_hr" constant="true"/>
      <parameter id="UPPER_BOUND" value="1000" units="mmol_per_gDW_per_hr" constant="true"/>
      <parameter id="FLUX_VALUE" value="0" units="mmol_per_gDW_per_hr" constant="true"/>
      <parameter id="OBJECTIVE_COEFFICIENT" value="0" units="mmol_per_gDW_per_hr" constant="true"/>
    </listOfParameters>
  </kineticLaw>
</reaction>
<reaction id="R_R623" name="NAD+ diphosphatase" reversible="false">
```

```xml
<notes>
  <body xmlns="http://www.w3.org/1999/xhtml">
    <p>GENE_ASSOCIATION: TTC0964</p>
    <p>SUBSYSTEM: Cofactor and Prosthetic Group Biosynthesis</p>
    <p>EC Number: 3.6.1.22</p>
    <p>Confidence Level: 1</p>
    <p>AUTHORS: Energy and cofactors metabolism</p>
    <p/>
  </body>
</notes>
<listOfReactants>
  <speciesReference species="M_H2O_c" stoichiometry="1"/>
  <speciesReference species="M_NAD_c" stoichiometry="1"/>
</listOfReactants>
<listOfProducts>
  <speciesReference species="M_H_c" stoichiometry="2"/>
  <speciesReference species="M_AMP_c" stoichiometry="1"/>
  <speciesReference species="M_NMN_c" stoichiometry="1"/>
</listOfProducts>
<kineticLaw>
  <math xmlns="http://www.w3.org/1998/Math/MathML">
    <ci> FLUX_VALUE </ci>
  </math>
  <listOfParameters>
    <parameter id="LOWER_BOUND" value="0" units="mmol_per_gDW_per_hr" constant="true"/>
    <parameter id="UPPER_BOUND" value="1000" units="mmol_per_gDW_per_hr" constant="true"/>
    <parameter id="FLUX_VALUE" value="0" units="mmol_per_gDW_per_hr" constant="true"/>
    <parameter id="OBJECTIVE_COEFFICIENT" value="0" units="mmol_per_gDW_per_hr" constant="true"/>
  </listOfParameters>
```

```
  </kineticLaw>
</reaction>
<reaction id="R_R624" name="NMN nucleosidase" reversible="false">
  <notes>
    <body xmlns="http://www.w3.org/1999/xhtml">
      <p>GENE_ASSOCIATION: </p>
      <p>SUBSYSTEM: Cofactor and Prosthetic Group Biosynthesis</p>
      <p>EC Number: 3.2.2.14</p>
      <p>Confidence Level: 1</p>
      <p>AUTHORS: Energy and cofactors metabolism</p>
      <p/>
    </body>
  </notes>
  <listOfReactants>
    <speciesReference species="M_H2O_c" stoichiometry="1"/>
    <speciesReference species="M_NMN_c" stoichiometry="1"/>
  </listOfReactants>
  <listOfProducts>
    <speciesReference species="M_H_c" stoichiometry="1"/>
    <speciesReference species="M_R5P_c" stoichiometry="1"/>
    <speciesReference species="M_NAMD_c" stoichiometry="1"/>
  </listOfProducts>
  <kineticLaw>
    <math xmlns="http://www.w3.org/1998/Math/MathML">
      <ci> FLUX_VALUE </ci>
    </math>
    <listOfParameters>
      <parameter id="LOWER_BOUND" value="0" units="mmol_per_gDW_per_hr" constant="true"/>
      <parameter id="UPPER_BOUND" value="1000" units="mmol_per_gDW_per_hr" constant="true"/>
```

```
      <parameter id="FLUX_VALUE" value="0" units="mmol_per_gDW_per_hr" constant="true"/>
      <parameter id="OBJECTIVE_COEFFICIENT" value="0" units="mmol_per_gDW_per_hr" constant="true"/>
    </listOfParameters>
  </kineticLaw>
</reaction>
<reaction id="R_R625" name="nicotinamidase" reversible="false">
  <notes>
    <body xmlns="http://www.w3.org/1999/xhtml">
      <p>GENE_ASSOCIATION: TTC1446</p>
      <p>SUBSYSTEM: Cofactor and Prosthetic Group Biosynthesis</p>
      <p>EC Number: 3.5.1.19</p>
      <p>Confidence Level: 1</p>
      <p>AUTHORS: Energy and cofactors metabolism</p>
      <p/>
    </body>
  </notes>
  <listOfReactants>
    <speciesReference species="M_H2O_c" stoichiometry="1"/>
    <speciesReference species="M_NAMD_c" stoichiometry="1"/>
  </listOfReactants>
  <listOfProducts>
    <speciesReference species="M_NH4_c" stoichiometry="1"/>
    <speciesReference species="M_NIA_c" stoichiometry="1"/>
  </listOfProducts>
  <kineticLaw>
    <math xmlns="http://www.w3.org/1998/Math/MathML">
      <ci> FLUX_VALUE </ci>
    </math>
    <listOfParameters>
```

```
      <parameter id="LOWER_BOUND" value="0" units="mmol_per_gDW_per_hr" constant="true"/>
      <parameter id="UPPER_BOUND" value="1000" units="mmol_per_gDW_per_hr" constant="true"/>
      <parameter id="FLUX_VALUE" value="0" units="mmol_per_gDW_per_hr" constant="true"/>
      <parameter id="OBJECTIVE_COEFFICIENT" value="0" units="mmol_per_gDW_per_hr" constant="true"/>
    </listOfParameters>
  </kineticLaw>
</reaction>
<reaction id="R_R626" name="nicotinamide-nucleotide amidase" reversible="false">
  <notes>
    <body xmlns="http://www.w3.org/1999/xhtml">
      <p>GENE_ASSOCIATION: TTC1468</p>
      <p>SUBSYSTEM: Cofactor and Prosthetic Group Biosynthesis</p>
      <p>EC Number: 3.5.1.42</p>
      <p>Confidence Level: 1</p>
      <p>AUTHORS: Energy and cofactors metabolism</p>
      <p/>
    </body>
  </notes>
  <listOfReactants>
    <speciesReference species="M_H2O_c" stoichiometry="1"/>
    <speciesReference species="M_NMN_c" stoichiometry="1"/>
  </listOfReactants>
  <listOfProducts>
    <speciesReference species="M_H_c" stoichiometry="1"/>
    <speciesReference species="M_NH4_c" stoichiometry="1"/>
    <speciesReference species="M_NC_D_RNUC_c" stoichiometry="1"/>
  </listOfProducts>
  <kineticLaw>
    <math xmlns="http://www.w3.org/1998/Math/MathML">
```

```
      <ci> FLUX_VALUE </ci>
    </math>
    <listOfParameters>
      <parameter id="LOWER_BOUND" value="0" units="mmol_per_gDW_per_hr" constant="true"/>
      <parameter id="UPPER_BOUND" value="1000" units="mmol_per_gDW_per_hr" constant="true"/>
      <parameter id="FLUX_VALUE" value="0" units="mmol_per_gDW_per_hr" constant="true"/>
      <parameter id="OBJECTIVE_COEFFICIENT" value="0" units="mmol_per_gDW_per_hr" constant="true"/>
    </listOfParameters>
  </kineticLaw>
</reaction>
<reaction id="R_R627" name="nicotinate-nucleotide adenylyltransferase" reversible="true">
  <notes>
    <body xmlns="http://www.w3.org/1999/xhtml">
      <p>GENE_ASSOCIATION: TTC1421</p>
      <p>SUBSYSTEM: Cofactor and Prosthetic Group Biosynthesis</p>
      <p>EC Number: 2.7.7.18</p>
      <p>Confidence Level: 1</p>
      <p>AUTHORS: Energy and cofactors metabolism</p>
      <p/>
    </body>
  </notes>
  <listOfReactants>
    <speciesReference species="M_ATP_c" stoichiometry="1"/>
    <speciesReference species="M_H_c" stoichiometry="1"/>
    <speciesReference species="M_NC_D_RNUC_c" stoichiometry="1"/>
  </listOfReactants>
  <listOfProducts>
    <speciesReference species="M_PPi_c" stoichiometry="1"/>
    <speciesReference species="M_DA_NAD_c" stoichiometry="1"/>
```

```
  </listOfProducts>
  <kineticLaw>
    <math xmlns="http://www.w3.org/1998/Math/MathML">
      <ci> FLUX_VALUE </ci>
    </math>
    <listOfParameters>
      <parameter id="LOWER_BOUND" value="-1000" units="mmol_per_gDW_per_hr" constant="true"/>
      <parameter id="UPPER_BOUND" value="1000" units="mmol_per_gDW_per_hr" constant="true"/>
      <parameter id="FLUX_VALUE" value="0" units="mmol_per_gDW_per_hr" constant="true"/>
      <parameter id="OBJECTIVE_COEFFICIENT" value="0" units="mmol_per_gDW_per_hr" constant="true"/>
    </listOfParameters>
  </kineticLaw>
</reaction>
<reaction id="R_R628" name="NAD+ nucleosidase" reversible="false">
  <notes>
    <body xmlns="http://www.w3.org/1999/xhtml">
      <p>GENE_ASSOCIATION: </p>
      <p>SUBSYSTEM: Cofactor and Prosthetic Group Biosynthesis</p>
      <p>EC Number: 3.2.2.5</p>
      <p>Confidence Level: 1</p>
      <p>AUTHORS: Energy and cofactors metabolism</p>
      <p/>
    </body>
  </notes>
  <listOfReactants>
    <speciesReference species="M_H2O_c" stoichiometry="1"/>
    <speciesReference species="M_NAD_c" stoichiometry="1"/>
  </listOfReactants>
  <listOfProducts>
```

```
    <speciesReference species="M_H_c" stoichiometry="1"/>
    <speciesReference species="M_ADP_RIB_c" stoichiometry="1"/>
    <speciesReference species="M_NAMD_c" stoichiometry="1"/>
  </listOfProducts>
  <kineticLaw>
    <math xmlns="http://www.w3.org/1998/Math/MathML">
      <ci> FLUX_VALUE </ci>
    </math>
    <listOfParameters>
      <parameter id="LOWER_BOUND" value="0" units="mmol_per_gDW_per_hr" constant="true"/>
      <parameter id="UPPER_BOUND" value="1000" units="mmol_per_gDW_per_hr" constant="true"/>
      <parameter id="FLUX_VALUE" value="0" units="mmol_per_gDW_per_hr" constant="true"/>
      <parameter id="OBJECTIVE_COEFFICIENT" value="0" units="mmol_per_gDW_per_hr" constant="true"/>
    </listOfParameters>
  </kineticLaw>
</reaction>
<reaction id="R_R629" name="thiosulfate sulfurtransferase" reversible="true">
  <notes>
    <body xmlns="http://www.w3.org/1999/xhtml">
      <p>GENE_ASSOCIATION: (TTC0666 or TTC1258 or TTC0155)</p>
      <p>SUBSYSTEM: Cofactor and Prosthetic Group Biosynthesis</p>
      <p>EC Number: 2.8.1.-</p>
      <p>Confidence Level: 1</p>
      <p>AUTHORS: Energy and cofactors metabolism</p>
      <p/>
    </body>
  </notes>
  <listOfReactants>
    <speciesReference species="M_R_TRED_c" stoichiometry="1"/>
```

```
    <speciesReference species="M_TSFA_c" stoichiometry="1"/>
  </listOfReactants>
  <listOfProducts>
    <speciesReference species="M_H_c" stoichiometry="1"/>
    <speciesReference species="M_O_TRED_c" stoichiometry="1"/>
    <speciesReference species="M_H2S_c" stoichiometry="1"/>
    <speciesReference species="M_SO3_c" stoichiometry="1"/>
  </listOfProducts>
  <kineticLaw>
    <math xmlns="http://www.w3.org/1998/Math/MathML">
      <ci> FLUX_VALUE </ci>
    </math>
    <listOfParameters>
      <parameter id="LOWER_BOUND" value="-1000" units="mmol_per_gDW_per_hr" constant="true"/>
      <parameter id="UPPER_BOUND" value="1000" units="mmol_per_gDW_per_hr" constant="true"/>
      <parameter id="FLUX_VALUE" value="0" units="mmol_per_gDW_per_hr" constant="true"/>
      <parameter id="OBJECTIVE_COEFFICIENT" value="0" units="mmol_per_gDW_per_hr" constant="true"/>
    </listOfParameters>
  </kineticLaw>
</reaction>
<reaction id="R_R630" name="formate dehydrogenase" reversible="true">
  <notes>
    <body xmlns="http://www.w3.org/1999/xhtml">
      <p>GENE_ASSOCIATION: TT_P0138</p>
      <p>SUBSYSTEM: Cofactor and Prosthetic Group Biosynthesis</p>
      <p>EC Number: 1.2.1.43/1.2.1.2</p>
      <p>Confidence Level: 1</p>
      <p>AUTHORS: Energy and cofactors metabolism</p>
      <p/>
```

```
      </body>
    </notes>
    <listOfReactants>
      <speciesReference species="M_H_c" stoichiometry="1"/>
      <speciesReference species="M_CO2_c" stoichiometry="1"/>
      <speciesReference species="M_NADPH_c" stoichiometry="1"/>
    </listOfReactants>
    <listOfProducts>
      <speciesReference species="M_NADP_c" stoichiometry="1"/>
      <speciesReference species="M_FOR_c" stoichiometry="1"/>
    </listOfProducts>
    <kineticLaw>
      <math xmlns="http://www.w3.org/1998/Math/MathML">
        <ci> FLUX_VALUE </ci>
      </math>
      <listOfParameters>
        <parameter id="LOWER_BOUND" value="-1000" units="mmol_per_gDW_per_hr" constant="true"/>
        <parameter id="UPPER_BOUND" value="1000" units="mmol_per_gDW_per_hr" constant="true"/>
        <parameter id="FLUX_VALUE" value="0" units="mmol_per_gDW_per_hr" constant="true"/>
        <parameter id="OBJECTIVE_COEFFICIENT" value="0" units="mmol_per_gDW_per_hr" constant="true"/>
      </listOfParameters>
    </kineticLaw>
  </reaction>
  <reaction id="R_R631" name="formate-tetrahydrofolate ligase" reversible="false">
    <notes>
      <body xmlns="http://www.w3.org/1999/xhtml">
        <p>GENE_ASSOCIATION: TTC1707</p>
        <p>SUBSYSTEM: Cofactor and Prosthetic Group Biosynthesis</p>
        <p>EC Number: 6.3.4.3</p>
```

```xml
      <p>Confidence Level: 1</p>
      <p>AUTHORS: Energy and cofactors metabolism</p>
      <p/>
    </body>
  </notes>
  <listOfReactants>
    <speciesReference species="M_ATP_c" stoichiometry="1"/>
    <speciesReference species="M_THF_c" stoichiometry="1"/>
    <speciesReference species="M_FOR_c" stoichiometry="1"/>
  </listOfReactants>
  <listOfProducts>
    <speciesReference species="M_ADP_c" stoichiometry="1"/>
    <speciesReference species="M_Pi_c" stoichiometry="1"/>
    <speciesReference species="M_FOLNA_c" stoichiometry="1"/>
  </listOfProducts>
  <kineticLaw>
    <math xmlns="http://www.w3.org/1998/Math/MathML">
      <ci> FLUX_VALUE </ci>
    </math>
    <listOfParameters>
      <parameter id="LOWER_BOUND" value="0" units="mmol_per_gDW_per_hr" constant="true"/>
      <parameter id="UPPER_BOUND" value="1000" units="mmol_per_gDW_per_hr" constant="true"/>
      <parameter id="FLUX_VALUE" value="0" units="mmol_per_gDW_per_hr" constant="true"/>
      <parameter id="OBJECTIVE_COEFFICIENT" value="0" units="mmol_per_gDW_per_hr" constant="true"/>
    </listOfParameters>
  </kineticLaw>
</reaction>
<reaction id="R_R632" name="dihydrofolate reductase" reversible="false">
  <notes>
```

```
  <body xmlns="http://www.w3.org/1999/xhtml">
   <p>GENE_ASSOCIATION: </p>
   <p>SUBSYSTEM: Cofactor and Prosthetic Group Biosynthesis</p>
   <p>EC Number: 1.5.1.3</p>
   <p>Confidence Level: 1</p>
   <p>AUTHORS: Energy and cofactors metabolism</p>
   <p/>
  </body>
 </notes>
 <listOfReactants>
  <speciesReference species="M_H_c" stoichiometry="1"/>
  <speciesReference species="M_NADPH_c" stoichiometry="1"/>
  <speciesReference species="M_DHF_c" stoichiometry="1"/>
 </listOfReactants>
 <listOfProducts>
  <speciesReference species="M_NADP_c" stoichiometry="1"/>
  <speciesReference species="M_THF_c" stoichiometry="1"/>
 </listOfProducts>
 <kineticLaw>
  <math xmlns="http://www.w3.org/1998/Math/MathML">
   <ci> FLUX_VALUE </ci>
  </math>
  <listOfParameters>
   <parameter id="LOWER_BOUND" value="0" units="mmol_per_gDW_per_hr" constant="true"/>
   <parameter id="UPPER_BOUND" value="1000" units="mmol_per_gDW_per_hr" constant="true"/>
   <parameter id="FLUX_VALUE" value="0" units="mmol_per_gDW_per_hr" constant="true"/>
   <parameter id="OBJECTIVE_COEFFICIENT" value="0" units="mmol_per_gDW_per_hr" constant="true"/>
  </listOfParameters>
 </kineticLaw>
```

```
</reaction>
<reaction id="R_R633" name="2-oxoacid-ferredoxin oxidoreductase" reversible="false">
 <notes>
  <body xmlns="http://www.w3.org/1999/xhtml">
   <p>GENE_ASSOCIATION: TTC1591</p>
   <p>SUBSYSTEM: Cofactor and Prosthetic Group Biosynthesis</p>
   <p>EC Number: 1.2.7.2</p>
   <p>Confidence Level: 1</p>
   <p>AUTHORS: Energy and cofactors metabolism</p>
   <p/>
  </body>
 </notes>
 <listOfReactants>
  <speciesReference species="M_CO2_c" stoichiometry="1"/>
  <speciesReference species="M_PRCoA_c" stoichiometry="1"/>
  <speciesReference species="M_R_FRDX_c" stoichiometry="1"/>
 </listOfReactants>
 <listOfProducts>
  <speciesReference species="M_CoA_c" stoichiometry="1"/>
  <speciesReference species="M_AKB_c" stoichiometry="1"/>
  <speciesReference species="M_O_FRDX_c" stoichiometry="1"/>
 </listOfProducts>
 <kineticLaw>
  <math xmlns="http://www.w3.org/1998/Math/MathML">
   <ci> FLUX_VALUE </ci>
  </math>
  <listOfParameters>
   <parameter id="LOWER_BOUND" value="0" units="mmol_per_gDW_per_hr" constant="true"/>
   <parameter id="UPPER_BOUND" value="1000" units="mmol_per_gDW_per_hr" constant="true"/>
```

```xml
      <parameter id="FLUX_VALUE" value="0" units="mmol_per_gDW_per_hr" constant="true"/>
      <parameter id="OBJECTIVE_COEFFICIENT" value="0" units="mmol_per_gDW_per_hr" constant="true"/>
    </listOfParameters>
  </kineticLaw>
</reaction>
<reaction id="R_R634" name="methylenetetrahydrofolate reductase (ferredoxin)" reversible="false">
  <notes>
    <body xmlns="http://www.w3.org/1999/xhtml">
      <p>GENE_ASSOCIATION: TTC1656</p>
      <p>SUBSYSTEM: Cofactor and Prosthetic Group Biosynthesis</p>
      <p>EC Number: 1.5.7.1</p>
      <p>Confidence Level: 1</p>
      <p>AUTHORS: Energy and cofactors metabolism</p>
      <p/>
    </body>
  </notes>
  <listOfReactants>
    <speciesReference species="M_H_c" stoichiometry="2"/>
    <speciesReference species="M_5_10_MNTHF_c" stoichiometry="1"/>
    <speciesReference species="M_R_FRDX_c" stoichiometry="2"/>
  </listOfReactants>
  <listOfProducts>
    <speciesReference species="M_5MTHF_c" stoichiometry="1"/>
    <speciesReference species="M_O_FRDX_c" stoichiometry="2"/>
  </listOfProducts>
  <kineticLaw>
    <math xmlns="http://www.w3.org/1998/Math/MathML">
      <ci> FLUX_VALUE </ci>
    </math>
```

```xml
<listOfParameters>

<parameter id="LOWER_BOUND" value="0" units="mmol_per_gDW_per_hr" constant="true"/>

<parameter id="UPPER_BOUND" value="1000" units="mmol_per_gDW_per_hr" constant="true"/>

<parameter id="FLUX_VALUE" value="0" units="mmol_per_gDW_per_hr" constant="true"/>

<parameter id="OBJECTIVE_COEFFICIENT" value="0" units="mmol_per_gDW_per_hr" constant="true"/>

</listOfParameters>

</kineticLaw>

</reaction>

<reaction id="R_R635" name="nitrilotriacetate monooxygenase component B, NADH-dependent flavin oxidoreductase" reversible="true">

<notes>

<body xmlns="http://www.w3.org/1999/xhtml">

<p>GENE_ASSOCIATION: (TTC0052 or TTC1829)</p>

<p>SUBSYSTEM: Cofactor and Prosthetic Group Biosynthesis</p>

<p>EC Number: 1.14.13.-</p>

<p>Confidence Level: 1</p>

<p>AUTHORS: Energy and cofactors metabolism</p>

<p/>

</body>

</notes>

<listOfReactants>

<speciesReference species="M_H_c" stoichiometry="1"/>

<speciesReference species="M_NADH_c" stoichiometry="1"/>

<speciesReference species="M_FMN_c" stoichiometry="1"/>

</listOfReactants>

<listOfProducts>

<speciesReference species="M_NAD_c" stoichiometry="1"/>

<speciesReference species="M_FMNH2_c" stoichiometry="1"/>

</listOfProducts>
```

```
  <kineticLaw>
    <math xmlns="http://www.w3.org/1998/Math/MathML">
      <ci> FLUX_VALUE </ci>
    </math>
    <listOfParameters>
      <parameter id="LOWER_BOUND" value="-1000" units="mmol_per_gDW_per_hr" constant="true"/>
      <parameter id="UPPER_BOUND" value="1000" units="mmol_per_gDW_per_hr" constant="true"/>
      <parameter id="FLUX_VALUE" value="0" units="mmol_per_gDW_per_hr" constant="true"/>
      <parameter id="OBJECTIVE_COEFFICIENT" value="0" units="mmol_per_gDW_per_hr" constant="true"/>
    </listOfParameters>
  </kineticLaw>
</reaction>
<reaction id="R_R636" name="thymidylate synthase" reversible="true">
  <notes>
    <body xmlns="http://www.w3.org/1999/xhtml">
      <p>GENE_ASSOCIATION: </p>
      <p>SUBSYSTEM: Nucleotide Salvage Pathway</p>
      <p>EC Number: 2.1.1.45</p>
      <p>Confidence Level: 1</p>
      <p>AUTHORS: Nucleotides metabolism</p>
      <p/>
    </body>
  </notes>
  <listOfReactants>
    <speciesReference species="M_dUMP_c" stoichiometry="1"/>
    <speciesReference species="M_5_10_MNTHF_c" stoichiometry="1"/>
  </listOfReactants>
  <listOfProducts>
    <speciesReference species="M_dTMP_c" stoichiometry="1"/>
```

```
<speciesReference species="M_DHF_c" stoichiometry="1"/>
</listOfProducts>
<kineticLaw>
<math xmlns="http://www.w3.org/1998/Math/MathML">
<ci> FLUX_VALUE </ci>
</math>
<listOfParameters>
<parameter id="LOWER_BOUND" value="-1000" units="mmol_per_gDW_per_hr" constant="true"/>
<parameter id="UPPER_BOUND" value="1000" units="mmol_per_gDW_per_hr" constant="true"/>
<parameter id="FLUX_VALUE" value="0" units="mmol_per_gDW_per_hr" constant="true"/>
<parameter id="OBJECTIVE_COEFFICIENT" value="0" units="mmol_per_gDW_per_hr" constant="true"/>
</listOfParameters>
</kineticLaw>
</reaction>
<reaction id="R_R637" name="uridine nucleosidase" reversible="false">
<notes>
<body xmlns="http://www.w3.org/1999/xhtml">
<p>GENE_ASSOCIATION: </p>
<p>SUBSYSTEM: Nucleotide Salvage Pathway</p>
<p>EC Number: 3.2.2.3</p>
<p>Confidence Level: 1</p>
<p>AUTHORS: Nucleotides metabolism</p>
<p/>
</body>
</notes>
<listOfReactants>
<speciesReference species="M_H2O_c" stoichiometry="1"/>
<speciesReference species="M_URD_c" stoichiometry="1"/>
</listOfReactants>
```

```
  <listOfProducts>
   <speciesReference species="M_D_RIB_c" stoichiometry="1"/>
   <speciesReference species="M_URA_c" stoichiometry="1"/>
  </listOfProducts>
  <kineticLaw>
   <math xmlns="http://www.w3.org/1998/Math/MathML">
    <ci> FLUX_VALUE </ci>
   </math>
   <listOfParameters>
    <parameter id="LOWER_BOUND" value="0" units="mmol_per_gDW_per_hr" constant="true"/>
    <parameter id="UPPER_BOUND" value="1000" units="mmol_per_gDW_per_hr" constant="true"/>
    <parameter id="FLUX_VALUE" value="0" units="mmol_per_gDW_per_hr" constant="true"/>
    <parameter id="OBJECTIVE_COEFFICIENT" value="0" units="mmol_per_gDW_per_hr" constant="true"/>
   </listOfParameters>
  </kineticLaw>
 </reaction>
 <reaction id="R_R638" name="uridine phosphorylase" reversible="false">
  <notes>
   <body xmlns="http://www.w3.org/1999/xhtml">
    <p>GENE_ASSOCIATION: TTC1070</p>
    <p>SUBSYSTEM: Nucleotide Salvage Pathway</p>
    <p>EC Number: 2.4.2.3</p>
    <p>Confidence Level: 1</p>
    <p>AUTHORS: Nucleotides metabolism</p>
    <p/>
   </body>
  </notes>
  <listOfReactants>
   <speciesReference species="M_Pi_c" stoichiometry="1"/>
```

```
    <speciesReference species="M_URD_c" stoichiometry="1"/>
  </listOfReactants>
  <listOfProducts>
    <speciesReference species="M_R1P_c" stoichiometry="1"/>
    <speciesReference species="M_URA_c" stoichiometry="1"/>
  </listOfProducts>
  <kineticLaw>
    <math xmlns="http://www.w3.org/1998/Math/MathML">
      <ci> FLUX_VALUE </ci>
    </math>
    <listOfParameters>
      <parameter id="LOWER_BOUND" value="0" units="mmol_per_gDW_per_hr" constant="true"/>
      <parameter id="UPPER_BOUND" value="1000" units="mmol_per_gDW_per_hr" constant="true"/>
      <parameter id="FLUX_VALUE" value="0" units="mmol_per_gDW_per_hr" constant="true"/>
      <parameter id="OBJECTIVE_COEFFICIENT" value="0" units="mmol_per_gDW_per_hr" constant="true"/>
    </listOfParameters>
  </kineticLaw>
</reaction>
<reaction id="R_R639" name="cob(II)yrinic acid a,c-diamide reductase" reversible="false">
  <notes>
    <body xmlns="http://www.w3.org/1999/xhtml">
      <p>GENE_ASSOCIATION: TT_P0018 </p>
      <p>SUBSYSTEM: Cofactor and Prosthetic Group Biosynthesis</p>
      <p>EC Number: 1.16.8.1</p>
      <p>Confidence Level: 1</p>
      <p>AUTHORS: Energy and cofactors metabolism</p>
      <p/>
    </body>
  </notes>
```

```
  <listOfReactants>
    <speciesReference species="M_FMNH2_c" stoichiometry="1"/>
    <speciesReference species="M_Co2ABYRDA_c" stoichiometry="2"/>
  </listOfReactants>
  <listOfProducts>
    <speciesReference species="M_H_c" stoichiometry="3"/>
    <speciesReference species="M_FMN_c" stoichiometry="1"/>
    <speciesReference species="M_CABYRDA_c" stoichiometry="2"/>
  </listOfProducts>
  <kineticLaw>
    <math xmlns="http://www.w3.org/1998/Math/MathML">
      <ci> FLUX_VALUE </ci>
    </math>
    <listOfParameters>
      <parameter id="LOWER_BOUND" value="0" units="mmol_per_gDW_per_hr" constant="true"/>
      <parameter id="UPPER_BOUND" value="1000" units="mmol_per_gDW_per_hr" constant="true"/>
      <parameter id="FLUX_VALUE" value="0" units="mmol_per_gDW_per_hr" constant="true"/>
      <parameter id="OBJECTIVE_COEFFICIENT" value="0" units="mmol_per_gDW_per_hr" constant="true"/>
    </listOfParameters>
  </kineticLaw>
</reaction>
<reaction id="R_R640" name="adenosylcobinamide-phosphate guanylyltransferase" reversible="false">
  <notes>
    <body xmlns="http://www.w3.org/1999/xhtml">
      <p>GENE_ASSOCIATION: TT_P0023 </p>
      <p>SUBSYSTEM: Cofactor and Prosthetic Group Biosynthesis</p>
      <p>EC Number: 2.7.7.62</p>
      <p>Confidence Level: 1</p>
      <p>AUTHORS: Energy and cofactors metabolism</p>
```

```
      <p/>
    </body>
  </notes>
  <listOfReactants>
    <speciesReference species="M_GTP_c" stoichiometry="1"/>
    <speciesReference species="M_H_c" stoichiometry="1"/>
    <speciesReference species="M_ADNS_CBNAPi_c" stoichiometry="1"/>
  </listOfReactants>
  <listOfProducts>
    <speciesReference species="M_PPi_c" stoichiometry="1"/>
    <speciesReference species="M_ADNS_CBNA_GDP_c" stoichiometry="1"/>
  </listOfProducts>
  <kineticLaw>
    <math xmlns="http://www.w3.org/1998/Math/MathML">
      <ci> FLUX_VALUE </ci>
    </math>
    <listOfParameters>
      <parameter id="LOWER_BOUND" value="0" units="mmol_per_gDW_per_hr" constant="true"/>
      <parameter id="UPPER_BOUND" value="1000" units="mmol_per_gDW_per_hr" constant="true"/>
      <parameter id="FLUX_VALUE" value="0" units="mmol_per_gDW_per_hr" constant="true"/>
      <parameter id="OBJECTIVE_COEFFICIENT" value="0" units="mmol_per_gDW_per_hr" constant="true"/>
    </listOfParameters>
  </kineticLaw>
</reaction>
<reaction id="R_R641" name="aminodeoxychorismate synthase" reversible="false">
  <notes>
    <body xmlns="http://www.w3.org/1999/xhtml">
      <p>GENE_ASSOCIATION: TTC0355 </p>
      <p>SUBSYSTEM: Cofactor and Prosthetic Group Biosynthesis</p>
```

```xml
      <p>EC Number: 2.6.1.85</p>

      <p>Confidence Level: 1</p>

      <p>AUTHORS: Energy and cofactors metabolism</p>

      <p/>

    </body>

  </notes>

  <listOfReactants>

    <speciesReference species="M_L_GLN_c" stoichiometry="1"/>

    <speciesReference species="M_CORM_c" stoichiometry="1"/>

  </listOfReactants>

  <listOfProducts>

    <speciesReference species="M_L_GLU_c" stoichiometry="1"/>

    <speciesReference species="M_4A4CORM_c" stoichiometry="1"/>

  </listOfProducts>

  <kineticLaw>

    <math xmlns="http://www.w3.org/1998/Math/MathML">

      <ci> FLUX_VALUE </ci>

    </math>

    <listOfParameters>

      <parameter id="LOWER_BOUND" value="0" units="mmol_per_gDW_per_hr" constant="true"/>

      <parameter id="UPPER_BOUND" value="1000" units="mmol_per_gDW_per_hr" constant="true"/>

      <parameter id="FLUX_VALUE" value="0" units="mmol_per_gDW_per_hr" constant="true"/>

      <parameter id="OBJECTIVE_COEFFICIENT" value="0" units="mmol_per_gDW_per_hr" constant="true"/>

    </listOfParameters>

  </kineticLaw>

</reaction>

<reaction id="R_R642" name="4-amino-4-deoxychorismate lyase" reversible="false">

  <notes>

    <body xmlns="http://www.w3.org/1999/xhtml">
```

```xml
      <p>GENE_ASSOCIATION: TTC1477</p>
      <p>SUBSYSTEM: Cofactor and Prosthetic Group Biosynthesis</p>
      <p>EC Number: 4.1.3.38</p>
      <p>Confidence Level: 1</p>
      <p>AUTHORS: Energy and cofactors metabolism</p>
      <p/>
    </body>
  </notes>
  <listOfReactants>
    <speciesReference species="M_4A4CORM_c" stoichiometry="1"/>
  </listOfReactants>
  <listOfProducts>
    <speciesReference species="M_H_c" stoichiometry="1"/>
    <speciesReference species="M_PYR_c" stoichiometry="1"/>
    <speciesReference species="M_ABEE_c" stoichiometry="1"/>
  </listOfProducts>
  <kineticLaw>
    <math xmlns="http://www.w3.org/1998/Math/MathML">
      <ci> FLUX_VALUE </ci>
    </math>
    <listOfParameters>
      <parameter id="LOWER_BOUND" value="0" units="mmol_per_gDW_per_hr" constant="true"/>
      <parameter id="UPPER_BOUND" value="1000" units="mmol_per_gDW_per_hr" constant="true"/>
      <parameter id="FLUX_VALUE" value="0" units="mmol_per_gDW_per_hr" constant="true"/>
      <parameter id="OBJECTIVE_COEFFICIENT" value="0" units="mmol_per_gDW_per_hr" constant="true"/>
    </listOfParameters>
  </kineticLaw>
</reaction>
<reaction id="R_R643" name="putative deoxyribonucleotide triphosphate pyrophosphatase" reversible="false">
```

```xml
<notes>
  <body xmlns="http://www.w3.org/1999/xhtml">
    <p>GENE_ASSOCIATION: TTC1290</p>
    <p>SUBSYSTEM: Nucleotide Salvage Pathway</p>
    <p>EC Number: 3.6.1.15</p>
    <p>Confidence Level: 1</p>
    <p>AUTHORS: Nucleotides metabolism</p>
    <p/>
  </body>
</notes>
<listOfReactants>
  <speciesReference species="M_UTP_c" stoichiometry="1"/>
  <speciesReference species="M_H2O_c" stoichiometry="1"/>
</listOfReactants>
<listOfProducts>
  <speciesReference species="M_Pi_c" stoichiometry="1"/>
  <speciesReference species="M_H_c" stoichiometry="1"/>
  <speciesReference species="M_UDP_c" stoichiometry="1"/>
</listOfProducts>
<kineticLaw>
  <math xmlns="http://www.w3.org/1998/Math/MathML">
    <ci> FLUX_VALUE </ci>
  </math>
  <listOfParameters>
    <parameter id="LOWER_BOUND" value="0" units="mmol_per_gDW_per_hr" constant="true"/>
    <parameter id="UPPER_BOUND" value="1000" units="mmol_per_gDW_per_hr" constant="true"/>
    <parameter id="FLUX_VALUE" value="0" units="mmol_per_gDW_per_hr" constant="true"/>
    <parameter id="OBJECTIVE_COEFFICIENT" value="0" units="mmol_per_gDW_per_hr" constant="true"/>
  </listOfParameters>
```

```
  </kineticLaw>
</reaction>
<reaction id="R_R644" name="nucleoside-diphosphatase" reversible="false">
  <notes>
    <body xmlns="http://www.w3.org/1999/xhtml">
      <p>GENE_ASSOCIATION: </p>
      <p>SUBSYSTEM: Nucleotide Salvage Pathway</p>
      <p>EC Number: 3.6.1.6</p>
      <p>Confidence Level: 1</p>
      <p>AUTHORS: Nucleotides metabolism</p>
      <p/>
    </body>
  </notes>
  <listOfReactants>
    <speciesReference species="M_H2O_c" stoichiometry="1"/>
    <speciesReference species="M_UDP_c" stoichiometry="1"/>
  </listOfReactants>
  <listOfProducts>
    <speciesReference species="M_Pi_c" stoichiometry="1"/>
    <speciesReference species="M_H_c" stoichiometry="1"/>
    <speciesReference species="M_UMP_c" stoichiometry="1"/>
  </listOfProducts>
  <kineticLaw>
    <math xmlns="http://www.w3.org/1998/Math/MathML">
      <ci> FLUX_VALUE </ci>
    </math>
    <listOfParameters>
      <parameter id="LOWER_BOUND" value="0" units="mmol_per_gDW_per_hr" constant="true"/>
      <parameter id="UPPER_BOUND" value="1000" units="mmol_per_gDW_per_hr" constant="true"/>
```

```
        <parameter id="FLUX_VALUE" value="0" units="mmol_per_gDW_per_hr" constant="true"/>
        <parameter id="OBJECTIVE_COEFFICIENT" value="0" units="mmol_per_gDW_per_hr" constant="true"/>
      </listOfParameters>
    </kineticLaw>
  </reaction>
  <reaction id="R_R645" name="apyrase" reversible="false">
    <notes>
      <body xmlns="http://www.w3.org/1999/xhtml">
        <p>GENE_ASSOCIATION: </p>
        <p>SUBSYSTEM: Nucleotide Salvage Pathway</p>
        <p>EC Number: 3.6.1.5</p>
        <p>Confidence Level: 1</p>
        <p>AUTHORS: Nucleotides metabolism</p>
        <p/>
      </body>
    </notes>
    <listOfReactants>
      <speciesReference species="M_CTP_c" stoichiometry="1"/>
      <speciesReference species="M_H2O_c" stoichiometry="2"/>
    </listOfReactants>
    <listOfProducts>
      <speciesReference species="M_Pi_c" stoichiometry="2"/>
      <speciesReference species="M_H_c" stoichiometry="2"/>
      <speciesReference species="M_CMP_c" stoichiometry="1"/>
    </listOfProducts>
    <kineticLaw>
      <math xmlns="http://www.w3.org/1998/Math/MathML">
        <ci> FLUX_VALUE </ci>
      </math>
```

```xml
<listOfParameters>
<parameter id="LOWER_BOUND" value="0" units="mmol_per_gDW_per_hr" constant="true"/>
<parameter id="UPPER_BOUND" value="1000" units="mmol_per_gDW_per_hr" constant="true"/>
<parameter id="FLUX_VALUE" value="0" units="mmol_per_gDW_per_hr" constant="true"/>
<parameter id="OBJECTIVE_COEFFICIENT" value="0" units="mmol_per_gDW_per_hr" constant="true"/>
</listOfParameters>
</kineticLaw>
</reaction>
<reaction id="R_R646" name="putative deoxyribonucleotide triphosphate pyrophosphatase" reversible="false">
<notes>
<body xmlns="http://www.w3.org/1999/xhtml">
<p>GENE_ASSOCIATION: TTC1290</p>
<p>SUBSYSTEM: Nucleotide Salvage Pathway</p>
<p>EC Number: 3.6.1.15</p>
<p>Confidence Level: 1</p>
<p>AUTHORS: Nucleotides metabolism</p>
<p/>
</body>
</notes>
<listOfReactants>
<speciesReference species="M_CTP_c" stoichiometry="1"/>
<speciesReference species="M_H2O_c" stoichiometry="1"/>
</listOfReactants>
<listOfProducts>
<speciesReference species="M_Pi_c" stoichiometry="1"/>
<speciesReference species="M_H_c" stoichiometry="1"/>
<speciesReference species="M_CDP_c" stoichiometry="1"/>
</listOfProducts>
<kineticLaw>
```

```
    <math xmlns="http://www.w3.org/1998/Math/MathML">
      <ci> FLUX_VALUE </ci>
    </math>
    <listOfParameters>
      <parameter id="LOWER_BOUND" value="0" units="mmol_per_gDW_per_hr" constant="true"/>
      <parameter id="UPPER_BOUND" value="1000" units="mmol_per_gDW_per_hr" constant="true"/>
      <parameter id="FLUX_VALUE" value="0" units="mmol_per_gDW_per_hr" constant="true"/>
      <parameter id="OBJECTIVE_COEFFICIENT" value="0" units="mmol_per_gDW_per_hr" constant="true"/>
    </listOfParameters>
  </kineticLaw>
</reaction>
<reaction id="R_R647" name="nucleoside-diphosphatase" reversible="false">
  <notes>
    <body xmlns="http://www.w3.org/1999/xhtml">
      <p>GENE_ASSOCIATION: </p>
      <p>SUBSYSTEM: Nucleotide Salvage Pathway</p>
      <p>EC Number: 3.6.1.6</p>
      <p>Confidence Level: 1</p>
      <p>AUTHORS: Nucleotides metabolism</p>
      <p/>
    </body>
  </notes>
  <listOfReactants>
    <speciesReference species="M_H2O_c" stoichiometry="1"/>
    <speciesReference species="M_CDP_c" stoichiometry="1"/>
  </listOfReactants>
  <listOfProducts>
    <speciesReference species="M_Pi_c" stoichiometry="1"/>
    <speciesReference species="M_H_c" stoichiometry="1"/>
```

```
    <speciesReference species="M_CMP_c" stoichiometry="1"/>
  </listOfProducts>
  <kineticLaw>
    <math xmlns="http://www.w3.org/1998/Math/MathML">
      <ci> FLUX_VALUE </ci>
    </math>
    <listOfParameters>
      <parameter id="LOWER_BOUND" value="0" units="mmol_per_gDW_per_hr" constant="true"/>
      <parameter id="UPPER_BOUND" value="1000" units="mmol_per_gDW_per_hr" constant="true"/>
      <parameter id="FLUX_VALUE" value="0" units="mmol_per_gDW_per_hr" constant="true"/>
      <parameter id="OBJECTIVE_COEFFICIENT" value="0" units="mmol_per_gDW_per_hr" constant="true"/>
    </listOfParameters>
  </kineticLaw>
</reaction>
<reaction id="R_R648" name="ornithine aminotransferase" reversible="false">
  <notes>
    <body xmlns="http://www.w3.org/1999/xhtml">
      <p>GENE_ASSOCIATION: TTC0553</p>
      <p>SUBSYSTEM: Arginine and Proline Metabolism</p>
      <p>EC Number: 2.6.1.13</p>
      <p>Confidence Level: 1</p>
      <p>AUTHORS: Amino acid metabolism</p>
      <p/>
    </body>
  </notes>
  <listOfReactants>
    <speciesReference species="M_ORT_c" stoichiometry="1"/>
    <speciesReference species="M_AKG_c" stoichiometry="1"/>
  </listOfReactants>
```

```
  <listOfProducts>
    <speciesReference species="M_L_GLU_c" stoichiometry="1"/>
    <speciesReference species="M_GLU_SA_c" stoichiometry="1"/>
  </listOfProducts>
  <kineticLaw>
    <math xmlns="http://www.w3.org/1998/Math/MathML">
      <ci> FLUX_VALUE </ci>
    </math>
    <listOfParameters>
      <parameter id="LOWER_BOUND" value="0" units="mmol_per_gDW_per_hr" constant="true"/>
      <parameter id="UPPER_BOUND" value="1000" units="mmol_per_gDW_per_hr" constant="true"/>
      <parameter id="FLUX_VALUE" value="0" units="mmol_per_gDW_per_hr" constant="true"/>
      <parameter id="OBJECTIVE_COEFFICIENT" value="0" units="mmol_per_gDW_per_hr" constant="true"/>
    </listOfParameters>
  </kineticLaw>
</reaction>
<reaction id="R_R649" name="formate hydrogenlyase" reversible="false">
  <notes>
    <body xmlns="http://www.w3.org/1999/xhtml">
      <p>GENE_ASSOCIATION: </p>
      <p>SUBSYSTEM: Pyruvate Metabolism</p>
      <p>EC Number: </p>
      <p>Confidence Level: 1</p>
      <p>AUTHORS: Carbohydrate metabolism</p>
      <p/>
    </body>
  </notes>
  <listOfReactants>
    <speciesReference species="M_FOR_c" stoichiometry="1"/>
```

```
  </listOfReactants>
  <listOfProducts>
    <speciesReference species="M_H_c" stoichiometry="2"/>
    <speciesReference species="M_CO2_c" stoichiometry="1"/>
  </listOfProducts>
  <kineticLaw>
    <math xmlns="http://www.w3.org/1998/Math/MathML">
      <ci> FLUX_VALUE </ci>
    </math>
    <listOfParameters>
      <parameter id="LOWER_BOUND" value="0" units="mmol_per_gDW_per_hr" constant="true"/>
      <parameter id="UPPER_BOUND" value="1000" units="mmol_per_gDW_per_hr" constant="true"/>
      <parameter id="FLUX_VALUE" value="0" units="mmol_per_gDW_per_hr" constant="true"/>
      <parameter id="OBJECTIVE_COEFFICIENT" value="0" units="mmol_per_gDW_per_hr" constant="true"/>
    </listOfParameters>
  </kineticLaw>
</reaction>
<reaction id="R_R650" name="phosphoserine aminotransferase" reversible="false">
  <notes>
    <body xmlns="http://www.w3.org/1999/xhtml">
      <p>GENE_ASSOCIATION: </p>
      <p>SUBSYSTEM: Glycine and Serine Metabolism</p>
      <p>EC Number: 2.6.1.52</p>
      <p>Confidence Level: 1</p>
      <p>AUTHORS: Amino acid metabolism</p>
      <p/>
    </body>
  </notes>
  <listOfReactants>
```

```xml
      <speciesReference species="M_L_GLU_c" stoichiometry="1"/>
      <speciesReference species="M_3POXPYR_c" stoichiometry="1"/>
    </listOfReactants>
    <listOfProducts>
      <speciesReference species="M_AKG_c" stoichiometry="1"/>
      <speciesReference species="M_PPTD_SER_c" stoichiometry="1"/>
    </listOfProducts>
    <kineticLaw>
      <math xmlns="http://www.w3.org/1998/Math/MathML">
        <ci> FLUX_VALUE </ci>
      </math>
      <listOfParameters>
        <parameter id="LOWER_BOUND" value="0" units="mmol_per_gDW_per_hr" constant="true"/>
        <parameter id="UPPER_BOUND" value="1000" units="mmol_per_gDW_per_hr" constant="true"/>
        <parameter id="FLUX_VALUE" value="0" units="mmol_per_gDW_per_hr" constant="true"/>
        <parameter id="OBJECTIVE_COEFFICIENT" value="0" units="mmol_per_gDW_per_hr" constant="true"/>
      </listOfParameters>
    </kineticLaw>
  </reaction>
  <reaction id="R_R651" name="3-phosphoserine phosphatase" reversible="false">
    <notes>
      <body xmlns="http://www.w3.org/1999/xhtml">
        <p>GENE_ASSOCIATION: </p>
        <p>SUBSYSTEM: Glycine and Serine Metabolism</p>
        <p>EC Number: 3.1.3.3</p>
        <p>Confidence Level: 1</p>
        <p>AUTHORS: Amino acid metabolism</p>
        <p/>
      </body>
```

```
    </notes>
    <listOfReactants>
      <speciesReference species="M_H2O_c" stoichiometry="1"/>
      <speciesReference species="M_PPTD_SER_c" stoichiometry="1"/>
    </listOfReactants>
    <listOfProducts>
      <speciesReference species="M_L_SER_c" stoichiometry="1"/>
      <speciesReference species="M_Pi_c" stoichiometry="1"/>
    </listOfProducts>
    <kineticLaw>
      <math xmlns="http://www.w3.org/1998/Math/MathML">
        <ci> FLUX_VALUE </ci>
      </math>
      <listOfParameters>
        <parameter id="LOWER_BOUND" value="0" units="mmol_per_gDW_per_hr" constant="true"/>
        <parameter id="UPPER_BOUND" value="1000" units="mmol_per_gDW_per_hr" constant="true"/>
        <parameter id="FLUX_VALUE" value="0" units="mmol_per_gDW_per_hr" constant="true"/>
        <parameter id="OBJECTIVE_COEFFICIENT" value="0" units="mmol_per_gDW_per_hr" constant="true"/>
      </listOfParameters>
    </kineticLaw>
  </reaction>
  <reaction id="R_R652" name="valine-pyruvate aminotransferase" reversible="false">
    <notes>
      <body xmlns="http://www.w3.org/1999/xhtml">
        <p>GENE_ASSOCIATION: TTC1960</p>
        <p>SUBSYSTEM: Valine, Leucine, and Isoleucine Metabolism</p>
        <p>EC Number: 2.6.1.66</p>
        <p>Confidence Level: 1</p>
        <p>AUTHORS: Amino acid metabolism</p>
```

```
<p/>

</body>

</notes>

<listOfReactants>

<speciesReference species="M_L_VAL_c" stoichiometry="1"/>

<speciesReference species="M_PYR_c" stoichiometry="1"/>

</listOfReactants>

<listOfProducts>

<speciesReference species="M_L_ALA_c" stoichiometry="1"/>

<speciesReference species="M_2K3M_BUT_c" stoichiometry="1"/>

</listOfProducts>

<kineticLaw>

<math xmlns="http://www.w3.org/1998/Math/MathML">

<ci> FLUX_VALUE </ci>

</math>

<listOfParameters>

<parameter id="LOWER_BOUND" value="0" units="mmol_per_gDW_per_hr" constant="true"/>

<parameter id="UPPER_BOUND" value="1000" units="mmol_per_gDW_per_hr" constant="true"/>

<parameter id="FLUX_VALUE" value="0" units="mmol_per_gDW_per_hr" constant="true"/>

<parameter id="OBJECTIVE_COEFFICIENT" value="0" units="mmol_per_gDW_per_hr" constant="true"/>

</listOfParameters>

</kineticLaw>

</reaction>

<reaction id="R_R653" name="coproporphyrinogen oxidase" reversible="false">

<notes>

<body xmlns="http://www.w3.org/1999/xhtml">

<p>GENE_ASSOCIATION: </p>

<p>SUBSYSTEM: Cofactor and Prosthetic Group Biosynthesis</p>

<p>EC Number: 1.3.3.3</p>
```

```xml
      <p>Confidence Level: 1</p>
      <p>AUTHORS: Energy and cofactors metabolism</p>
      <p/>
    </body>
  </notes>
  <listOfReactants>
    <speciesReference species="M_H_c" stoichiometry="2"/>
    <speciesReference species="M_O2_c" stoichiometry="1"/>
    <speciesReference species="M_CPPPHYRGN_c" stoichiometry="1"/>
  </listOfReactants>
  <listOfProducts>
    <speciesReference species="M_H2O_c" stoichiometry="2"/>
    <speciesReference species="M_CO2_c" stoichiometry="2"/>
    <speciesReference species="M_PPPHYRGN_c" stoichiometry="1"/>
  </listOfProducts>
  <kineticLaw>
    <math xmlns="http://www.w3.org/1998/Math/MathML">
      <ci> FLUX_VALUE </ci>
    </math>
    <listOfParameters>
      <parameter id="LOWER_BOUND" value="0" units="mmol_per_gDW_per_hr" constant="true"/>
      <parameter id="UPPER_BOUND" value="1000" units="mmol_per_gDW_per_hr" constant="true"/>
      <parameter id="FLUX_VALUE" value="0" units="mmol_per_gDW_per_hr" constant="true"/>
      <parameter id="OBJECTIVE_COEFFICIENT" value="0" units="mmol_per_gDW_per_hr" constant="true"/>
    </listOfParameters>
  </kineticLaw>
</reaction>
<reaction id="R_R654" name="quinate/shikimate dehydrogenase" reversible="false">
  <notes>
```

```
  <body xmlns="http://www.w3.org/1999/xhtml">
    <p>GENE_ASSOCIATION: TTC0688 </p>
    <p>SUBSYSTEM: Tyrosine, Tryptophan, and Phenylalanine Metabolism</p>
    <p>EC Number: 1.1.1.282</p>
    <p>Confidence Level: 1</p>
    <p>AUTHORS: Amino acid metabolism</p>
    <p/>
  </body>
</notes>
<listOfReactants>
  <speciesReference species="M_H_c" stoichiometry="1"/>
  <speciesReference species="M_NADH_c" stoichiometry="1"/>
  <speciesReference species="M_DHY_SHK_c" stoichiometry="1"/>
</listOfReactants>
<listOfProducts>
  <speciesReference species="M_NAD_c" stoichiometry="1"/>
  <speciesReference species="M_SHK_c" stoichiometry="1"/>
</listOfProducts>
<kineticLaw>
  <math xmlns="http://www.w3.org/1998/Math/MathML">
    <ci> FLUX_VALUE </ci>
  </math>
  <listOfParameters>
    <parameter id="LOWER_BOUND" value="0" units="mmol_per_gDW_per_hr" constant="true"/>
    <parameter id="UPPER_BOUND" value="1000" units="mmol_per_gDW_per_hr" constant="true"/>
    <parameter id="FLUX_VALUE" value="0" units="mmol_per_gDW_per_hr" constant="true"/>
    <parameter id="OBJECTIVE_COEFFICIENT" value="0" units="mmol_per_gDW_per_hr" constant="true"/>
  </listOfParameters>
</kineticLaw>
```

```xml
</reaction>
<reaction id="R_R655" name="serine hydroxymethyltransferase" reversible="false">
<notes>
<body xmlns="http://www.w3.org/1999/xhtml">
<p>GENE_ASSOCIATION: TTC1160</p>
<p>SUBSYSTEM: Glycine and Serine Metabolism</p>
<p>EC Number: 2.1.2.1</p>
<p>Confidence Level: 1</p>
<p>AUTHORS: Amino acid metabolism</p>
<p/>
</body>
</notes>
<listOfReactants>
<speciesReference species="M_L_SER_c" stoichiometry="1"/>
<speciesReference species="M_THF_c" stoichiometry="1"/>
</listOfReactants>
<listOfProducts>
<speciesReference species="M_L_GLY_c" stoichiometry="1"/>
<speciesReference species="M_H2O_c" stoichiometry="1"/>
<speciesReference species="M_METHF_c" stoichiometry="1"/>
</listOfProducts>
<kineticLaw>
<math xmlns="http://www.w3.org/1998/Math/MathML">
<ci> FLUX_VALUE </ci>
</math>
<listOfParameters>
<parameter id="LOWER_BOUND" value="0" units="mmol_per_gDW_per_hr" constant="true"/>
<parameter id="UPPER_BOUND" value="1000" units="mmol_per_gDW_per_hr" constant="true"/>
<parameter id="FLUX_VALUE" value="0" units="mmol_per_gDW_per_hr" constant="true"/>
```

```
      <parameter id="OBJECTIVE_COEFFICIENT" value="0" units="mmol_per_gDW_per_hr" constant="true"/>
    </listOfParameters>
  </kineticLaw>
</reaction>
<reaction id="R_R656" name="serine-glyoxylate transaminase" reversible="false">
  <notes>
    <body xmlns="http://www.w3.org/1999/xhtml">
      <p>GENE_ASSOCIATION: </p>
      <p>SUBSYSTEM: Glycine and Serine Metabolism</p>
      <p>EC Number: 2.6.1.45</p>
      <p>Confidence Level: 1</p>
      <p>AUTHORS: Amino acid metabolism</p>
      <p/>
    </body>
  </notes>
  <listOfReactants>
    <speciesReference species="M_L_SER_c" stoichiometry="1"/>
    <speciesReference species="M_GLOXT_c" stoichiometry="1"/>
  </listOfReactants>
  <listOfProducts>
    <speciesReference species="M_L_GLY_c" stoichiometry="1"/>
    <speciesReference species="M_HY_PYR_c" stoichiometry="1"/>
  </listOfProducts>
  <kineticLaw>
    <math xmlns="http://www.w3.org/1998/Math/MathML">
      <ci> FLUX_VALUE </ci>
    </math>
    <listOfParameters>
      <parameter id="LOWER_BOUND" value="0" units="mmol_per_gDW_per_hr" constant="true"/>
```

```
<parameter id="UPPER_BOUND" value="1000" units="mmol_per_gDW_per_hr" constant="true"/>
<parameter id="FLUX_VALUE" value="0" units="mmol_per_gDW_per_hr" constant="true"/>
<parameter id="OBJECTIVE_COEFFICIENT" value="0" units="mmol_per_gDW_per_hr" constant="true"/>
</listOfParameters>
</kineticLaw>
</reaction>
<reaction id="R_R657" name="malate-CoA ligase" reversible="false">
<notes>
<body xmlns="http://www.w3.org/1999/xhtml">
<p>GENE_ASSOCIATION: </p>
<p>SUBSYSTEM: Glycine and Serine Metabolism</p>
<p>EC Number: 6.2.1.9</p>
<p>Confidence Level: 1</p>
<p>AUTHORS: Amino acid metabolism</p>
<p/>
</body>
</notes>
<listOfReactants>
<speciesReference species="M_ATP_c" stoichiometry="1"/>
<speciesReference species="M_CoA_c" stoichiometry="1"/>
<speciesReference species="M_MAL_c" stoichiometry="1"/>
</listOfReactants>
<listOfProducts>
<speciesReference species="M_ADP_c" stoichiometry="1"/>
<speciesReference species="M_Pi_c" stoichiometry="1"/>
<speciesReference species="M_MALYCoA_c" stoichiometry="1"/>
</listOfProducts>
<kineticLaw>
<math xmlns="http://www.w3.org/1998/Math/MathML">
```

```xml
<ci> FLUX_VALUE </ci>
</math>
<listOfParameters>
<parameter id="LOWER_BOUND" value="0" units="mmol_per_gDW_per_hr" constant="true"/>
<parameter id="UPPER_BOUND" value="1000" units="mmol_per_gDW_per_hr" constant="true"/>
<parameter id="FLUX_VALUE" value="0" units="mmol_per_gDW_per_hr" constant="true"/>
<parameter id="OBJECTIVE_COEFFICIENT" value="0" units="mmol_per_gDW_per_hr" constant="true"/>
</listOfParameters>
</kineticLaw>
</reaction>
<reaction id="R_R658" name="malyl-CoA lyase" reversible="false">
<notes>
<body xmlns="http://www.w3.org/1999/xhtml">
<p>GENE_ASSOCIATION: </p>
<p>SUBSYSTEM: Glycine and Serine Metabolism</p>
<p>EC Number: 4.1.3.24</p>
<p>Confidence Level: 1</p>
<p>AUTHORS: Amino acid metabolism</p>
<p/>
</body>
</notes>
<listOfReactants>
<speciesReference species="M_MALYCoA_c" stoichiometry="1"/>
</listOfReactants>
<listOfProducts>
<speciesReference species="M_ACoA_c" stoichiometry="1"/>
<speciesReference species="M_GLOXT_c" stoichiometry="1"/>
</listOfProducts>
<kineticLaw>
```

```
    <math xmlns="http://www.w3.org/1998/Math/MathML">
      <ci> FLUX_VALUE </ci>
    </math>
    <listOfParameters>
      <parameter id="LOWER_BOUND" value="0" units="mmol_per_gDW_per_hr" constant="true"/>
      <parameter id="UPPER_BOUND" value="1000" units="mmol_per_gDW_per_hr" constant="true"/>
      <parameter id="FLUX_VALUE" value="0" units="mmol_per_gDW_per_hr" constant="true"/>
      <parameter id="OBJECTIVE_COEFFICIENT" value="0" units="mmol_per_gDW_per_hr" constant="true"/>
    </listOfParameters>
  </kineticLaw>
</reaction>
<reaction id="R_R659" name="acyl-CoA hydrolase" reversible="false">
  <notes>
    <body xmlns="http://www.w3.org/1999/xhtml">
      <p>GENE_ASSOCIATION: TTC1452 </p>
      <p>SUBSYSTEM: Glycine and Serine Metabolism</p>
      <p>EC Number: 3.1.2.20 </p>
      <p>Confidence Level: 1</p>
      <p>AUTHORS: Amino acid metabolism</p>
      <p/>
    </body>
  </notes>
  <listOfReactants>
    <speciesReference species="M_H2O_c" stoichiometry="2"/>
    <speciesReference species="M_ACoA_c" stoichiometry="1"/>
  </listOfReactants>
  <listOfProducts>
    <speciesReference species="M_H_c" stoichiometry="5"/>
    <speciesReference species="M_CoA_c" stoichiometry="1"/>
```

```
    <speciesReference species="M_GLOXT_c" stoichiometry="1"/>
  </listOfProducts>
  <kineticLaw>
    <math xmlns="http://www.w3.org/1998/Math/MathML">
      <ci> FLUX_VALUE </ci>
    </math>
    <listOfParameters>
      <parameter id="LOWER_BOUND" value="0" units="mmol_per_gDW_per_hr" constant="true"/>
      <parameter id="UPPER_BOUND" value="1000" units="mmol_per_gDW_per_hr" constant="true"/>
      <parameter id="FLUX_VALUE" value="0" units="mmol_per_gDW_per_hr" constant="true"/>
      <parameter id="OBJECTIVE_COEFFICIENT" value="0" units="mmol_per_gDW_per_hr" constant="true"/>
    </listOfParameters>
  </kineticLaw>
</reaction>
<reaction id="R_R660" name="malate dehydrogenase" reversible="false">
  <notes>
    <body xmlns="http://www.w3.org/1999/xhtml">
      <p>GENE_ASSOCIATION: TTC0144</p>
      <p>SUBSYSTEM: Anaplerotic Reactions</p>
      <p>EC Number: 1.1.1.40</p>
      <p>Confidence Level: 1</p>
      <p>AUTHORS: Carbohydrate metabolism</p>
      <p/>
    </body>
  </notes>
  <listOfReactants>
    <speciesReference species="M_NADP_c" stoichiometry="1"/>
    <speciesReference species="M_MAL_c" stoichiometry="1"/>
  </listOfReactants>
```

```
  <listOfProducts>
   <speciesReference species="M_PYR_c" stoichiometry="1"/>
   <speciesReference species="M_CO2_c" stoichiometry="1"/>
   <speciesReference species="M_NADPH_c" stoichiometry="1"/>
  </listOfProducts>
  <kineticLaw>
   <math xmlns="http://www.w3.org/1998/Math/MathML">
    <ci> FLUX_VALUE </ci>
   </math>
   <listOfParameters>
    <parameter id="LOWER_BOUND" value="0" units="mmol_per_gDW_per_hr" constant="true"/>
    <parameter id="UPPER_BOUND" value="1000" units="mmol_per_gDW_per_hr" constant="true"/>
    <parameter id="FLUX_VALUE" value="0" units="mmol_per_gDW_per_hr" constant="true"/>
    <parameter id="OBJECTIVE_COEFFICIENT" value="0" units="mmol_per_gDW_per_hr" constant="true"/>
   </listOfParameters>
  </kineticLaw>
 </reaction>
 <reaction id="R_R661" name="fructose 1,6-bisphosphatase II " reversible="false">
  <notes>
   <body xmlns="http://www.w3.org/1999/xhtml">
    <p>GENE_ASSOCIATION: TTC1081</p>
    <p>SUBSYSTEM: Glycolysis/Gluconeogenesis</p>
    <p>EC Number: 3.1.3.11</p>
    <p>Confidence Level: 1</p>
    <p>AUTHORS: Carbohydrate metabolism</p>
    <p/>
   </body>
  </notes>
  <listOfReactants>
```

<speciesReference species="M_H2O_c" stoichiometry="1"/>
<speciesReference species="M_FBP_c" stoichiometry="1"/>
</listOfReactants>
<listOfProducts>
<speciesReference species="M_Pi_c" stoichiometry="1"/>
<speciesReference species="M_F6P_c" stoichiometry="1"/>
</listOfProducts>
<kineticLaw>
<math xmlns="http://www.w3.org/1998/Math/MathML">
<ci> FLUX_VALUE </ci>
</math>
<listOfParameters>
<parameter id="LOWER_BOUND" value="0" units="mmol_per_gDW_per_hr" constant="true"/>
<parameter id="UPPER_BOUND" value="1000" units="mmol_per_gDW_per_hr" constant="true"/>
<parameter id="FLUX_VALUE" value="0" units="mmol_per_gDW_per_hr" constant="true"/>
<parameter id="OBJECTIVE_COEFFICIENT" value="0" units="mmol_per_gDW_per_hr" constant="true"/>
</listOfParameters>
</kineticLaw>
</reaction>
<reaction id="R_R662" name="glucose-1-phosphatase" reversible="false">
<notes>
<body xmlns="http://www.w3.org/1999/xhtml">
<p>GENE_ASSOCIATION: </p>
<p>SUBSYSTEM: Glycolysis/Gluconeogenesis</p>
<p>EC Number: 3.1.3.10</p>
<p>Confidence Level: 1</p>
<p>AUTHORS: Carbohydrate metabolism</p>
<p/>
</body>

```
  </notes>
  <listOfReactants>
    <speciesReference species="M_H2O_c" stoichiometry="1"/>
    <speciesReference species="M_G1P_c" stoichiometry="1"/>
  </listOfReactants>
  <listOfProducts>
    <speciesReference species="M_Pi_c" stoichiometry="1"/>
    <speciesReference species="M_D_GLC_c" stoichiometry="1"/>
  </listOfProducts>
  <kineticLaw>
    <math xmlns="http://www.w3.org/1998/Math/MathML">
      <ci> FLUX_VALUE </ci>
    </math>
    <listOfParameters>
      <parameter id="LOWER_BOUND" value="0" units="mmol_per_gDW_per_hr" constant="true"/>
      <parameter id="UPPER_BOUND" value="1000" units="mmol_per_gDW_per_hr" constant="true"/>
      <parameter id="FLUX_VALUE" value="0" units="mmol_per_gDW_per_hr" constant="true"/>
      <parameter id="OBJECTIVE_COEFFICIENT" value="0" units="mmol_per_gDW_per_hr" constant="true"/>
    </listOfParameters>
  </kineticLaw>
</reaction>
<reaction id="R_R663" name="phosphate acetyltransferase" reversible="false">
  <notes>
    <body xmlns="http://www.w3.org/1999/xhtml">
      <p>GENE_ASSOCIATION: </p>
      <p>SUBSYSTEM: Pyruvate Metabolism</p>
      <p>EC Number: 2.3.1.8</p>
      <p>Confidence Level: 1</p>
      <p>AUTHORS: Carbohydrate metabolism</p>
```

```
      <p/>
    </body>
  </notes>
  <listOfReactants>
    <speciesReference species="M_Pi_c" stoichiometry="1"/>
    <speciesReference species="M_ACoA_c" stoichiometry="1"/>
  </listOfReactants>
  <listOfProducts>
    <speciesReference species="M_CoA_c" stoichiometry="1"/>
    <speciesReference species="M_ACPi_c" stoichiometry="1"/>
  </listOfProducts>
  <kineticLaw>
    <math xmlns="http://www.w3.org/1998/Math/MathML">
      <ci> FLUX_VALUE </ci>
    </math>
    <listOfParameters>
      <parameter id="LOWER_BOUND" value="0" units="mmol_per_gDW_per_hr" constant="true"/>
      <parameter id="UPPER_BOUND" value="0" units="mmol_per_gDW_per_hr" constant="true"/>
      <parameter id="FLUX_VALUE" value="0" units="mmol_per_gDW_per_hr" constant="true"/>
      <parameter id="OBJECTIVE_COEFFICIENT" value="0" units="mmol_per_gDW_per_hr" constant="true"/>
    </listOfParameters>
  </kineticLaw>
</reaction>
<reaction id="R_R664" name="acetolactate synthase large subunit" reversible="false">
  <notes>
    <body xmlns="http://www.w3.org/1999/xhtml">
      <p>GENE_ASSOCIATION: TTC0852</p>
      <p>SUBSYSTEM: Valine, Leucine, and Isoleucine Metabolism</p>
      <p>EC Number: 2.2.1.6</p>
```

```xml
      <p>Confidence Level: 1</p>
      <p>AUTHORS: Amino acid metabolism</p>
      <p/>
    </body>
  </notes>
  <listOfReactants>
    <speciesReference species="M_H_c" stoichiometry="1"/>
    <speciesReference species="M_PYR_c" stoichiometry="1"/>
    <speciesReference species="M_AKB_c" stoichiometry="1"/>
  </listOfReactants>
  <listOfProducts>
    <speciesReference species="M_CO2_c" stoichiometry="1"/>
    <speciesReference species="M_AC_HYBUTNA_c" stoichiometry="1"/>
  </listOfProducts>
  <kineticLaw>
    <math xmlns="http://www.w3.org/1998/Math/MathML">
      <ci> FLUX_VALUE </ci>
    </math>
    <listOfParameters>
      <parameter id="LOWER_BOUND" value="0" units="mmol_per_gDW_per_hr" constant="true"/>
      <parameter id="UPPER_BOUND" value="1000" units="mmol_per_gDW_per_hr" constant="true"/>
      <parameter id="FLUX_VALUE" value="0" units="mmol_per_gDW_per_hr" constant="true"/>
      <parameter id="OBJECTIVE_COEFFICIENT" value="0" units="mmol_per_gDW_per_hr" constant="true"/>
    </listOfParameters>
  </kineticLaw>
</reaction>
<reaction id="R_R665" name="ketol-acid reductoisomerase" reversible="false">
  <notes>
    <body xmlns="http://www.w3.org/1999/xhtml">
```

```
<p>GENE_ASSOCIATION: TTC0850</p>
<p>SUBSYSTEM: Valine, Leucine, and Isoleucine Metabolism</p>
<p>EC Number: 1.1.1.86</p>
<p>Confidence Level: 1</p>
<p>AUTHORS: Amino acid metabolism</p>
<p/>
</body>
</notes>
<listOfReactants>
<speciesReference species="M_H_c" stoichiometry="1"/>
<speciesReference species="M_NADPH_c" stoichiometry="1"/>
<speciesReference species="M_AC_HYBUTNA_c" stoichiometry="1"/>
</listOfReactants>
<listOfProducts>
<speciesReference species="M_NADP_c" stoichiometry="1"/>
<speciesReference species="M_DHY_MPENTA_c" stoichiometry="1"/>
</listOfProducts>
<kineticLaw>
<math xmlns="http://www.w3.org/1998/Math/MathML">
<ci> FLUX_VALUE </ci>
</math>
<listOfParameters>
<parameter id="LOWER_BOUND" value="0" units="mmol_per_gDW_per_hr" constant="true"/>
<parameter id="UPPER_BOUND" value="1000" units="mmol_per_gDW_per_hr" constant="true"/>
<parameter id="FLUX_VALUE" value="0" units="mmol_per_gDW_per_hr" constant="true"/>
<parameter id="OBJECTIVE_COEFFICIENT" value="0" units="mmol_per_gDW_per_hr" constant="true"/>
</listOfParameters>
</kineticLaw>
</reaction>
```

```
<reaction id="R_R666" name="2-oxoglutarate ferredoxin oxidoreductase subunit beta" reversible="true">
 <notes>
  <body xmlns="http://www.w3.org/1999/xhtml">
   <p>GENE_ASSOCIATION: TTC1592</p>
   <p>SUBSYSTEM: Citric Acid Cycle</p>
   <p>EC Number: 1.2.7.3</p>
   <p>Confidence Level: 1</p>
   <p>AUTHORS: Carbohydrate metabolism</p>
   <p/>
  </body>
 </notes>
 <listOfReactants>
  <speciesReference species="M_CoA_c" stoichiometry="1"/>
  <speciesReference species="M_AKG_c" stoichiometry="1"/>
  <speciesReference species="M_O_FRDX_c" stoichiometry="2"/>
 </listOfReactants>
 <listOfProducts>
  <speciesReference species="M_CO2_c" stoichiometry="1"/>
  <speciesReference species="M_SUCCoA_c" stoichiometry="1"/>
  <speciesReference species="M_R_FRDX_c" stoichiometry="2"/>
 </listOfProducts>
 <kineticLaw>
  <math xmlns="http://www.w3.org/1998/Math/MathML">
   <ci> FLUX_VALUE </ci>
  </math>
  <listOfParameters>
   <parameter id="LOWER_BOUND" value="-1000" units="mmol_per_gDW_per_hr" constant="true"/>
   <parameter id="UPPER_BOUND" value="1000" units="mmol_per_gDW_per_hr" constant="true"/>
   <parameter id="FLUX_VALUE" value="0" units="mmol_per_gDW_per_hr" constant="true"/>
```

```xml
<parameter id="OBJECTIVE_COEFFICIENT" value="0" units="mmol_per_gDW_per_hr" constant="true"/>

</listOfParameters>

</kineticLaw>

</reaction>

<reaction id="R_R667" name="fatty acid synthesis; lumped" reversible="false">

<notes>

<body xmlns="http://www.w3.org/1999/xhtml">

<p>GENE_ASSOCIATION: ((TTC0049 or TTC0045) and (TTC0045 or (TTC0049 or TTC0343)) and (TTC0047 or TTC0394) and TTC1463 and TTC0048)</p>

<p>SUBSYSTEM: Membrane Lipid Metabolism</p>

<p>EC Number: 2.3.1.180 and (2.3.1.179 or 2.3.1.180) and 1.1.1.100 and 4.2.1.59</p>

<p>Confidence Level: 1</p>

<p>AUTHORS: Lipid metabolism</p>

<p/>

</body>

</notes>

<listOfReactants>

<speciesReference species="M_H_c" stoichiometry="16"/>

<speciesReference species="M_NADPH_c" stoichiometry="11"/>

<speciesReference species="M_MALCoA_c" stoichiometry="6"/>

<speciesReference species="M_MBUTNCoA_c" stoichiometry="1"/>

</listOfReactants>

<listOfProducts>

<speciesReference species="M_H2O_c" stoichiometry="5"/>

<speciesReference species="M_CO2_c" stoichiometry="6"/>

<speciesReference species="M_CoA_c" stoichiometry="7"/>

<speciesReference species="M_NADP_c" stoichiometry="11"/>

<speciesReference species="M_AI171_c" stoichiometry="1"/>

</listOfProducts>
```

```
        <kineticLaw>
          <math xmlns="http://www.w3.org/1998/Math/MathML">
            <ci> FLUX_VALUE </ci>
          </math>
          <listOfParameters>
            <parameter id="LOWER_BOUND" value="0" units="mmol_per_gDW_per_hr" constant="true"/>
            <parameter id="UPPER_BOUND" value="1000" units="mmol_per_gDW_per_hr" constant="true"/>
            <parameter id="FLUX_VALUE" value="0" units="mmol_per_gDW_per_hr" constant="true"/>
            <parameter id="OBJECTIVE_COEFFICIENT" value="0" units="mmol_per_gDW_per_hr" constant="true"/>
          </listOfParameters>
        </kineticLaw>
      </reaction>
      <reaction id="R_R668" name="fatty acid synthesis; lumped" reversible="false">
        <notes>
          <body xmlns="http://www.w3.org/1999/xhtml">
            <p>GENE_ASSOCIATION: ((TTC0049 or TTC0045) and (TTC0045 or (TTC0049 or TTC0343)) and (TTC0047 or TTC0394) and
TTC1463 and TTC0048)</p>
            <p>SUBSYSTEM: Membrane Lipid Metabolism</p>
            <p>EC Number: 2.3.1.180 and (2.3.1.179 or 2.3.1.180) and 1.1.1.100 and 4.2.1.59</p>
            <p>Confidence Level: 1</p>
            <p>AUTHORS: Lipid metabolism</p>
            <p/>
          </body>
        </notes>
        <listOfReactants>
          <speciesReference species="M_H_c" stoichiometry="14"/>
          <speciesReference species="M_NADPH_c" stoichiometry="10"/>
          <speciesReference species="M_MALCoA_c" stoichiometry="5"/>
          <speciesReference species="M_MBUTNCoA_c" stoichiometry="1"/>
```

```
        </listOfReactants>
        <listOfProducts>
          <speciesReference species="M_H2O_c" stoichiometry="4"/>
          <speciesReference species="M_CO2_c" stoichiometry="5"/>
          <speciesReference species="M_CoA_c" stoichiometry="6"/>
          <speciesReference species="M_NADP_c" stoichiometry="10"/>
          <speciesReference species="M_AI150_c" stoichiometry="1"/>
        </listOfProducts>
        <kineticLaw>
          <math xmlns="http://www.w3.org/1998/Math/MathML">
            <ci> FLUX_VALUE </ci>
          </math>
          <listOfParameters>
            <parameter id="LOWER_BOUND" value="0" units="mmol_per_gDW_per_hr" constant="true"/>
            <parameter id="UPPER_BOUND" value="1000" units="mmol_per_gDW_per_hr" constant="true"/>
            <parameter id="FLUX_VALUE" value="0" units="mmol_per_gDW_per_hr" constant="true"/>
            <parameter id="OBJECTIVE_COEFFICIENT" value="0" units="mmol_per_gDW_per_hr" constant="true"/>
          </listOfParameters>
        </kineticLaw>
      </reaction>
      <reaction id="R_R669" name="fatty acid synthesis; lumped" reversible="false">
        <notes>
          <body xmlns="http://www.w3.org/1999/xhtml">
            <p>GENE_ASSOCIATION: ((TTC0049 or TTC0045) and (TTC0045 or (TTC0049 or TTC0343)) and (TTC0047 or TTC0394) and
TTC1463 and TTC0048)</p>
            <p>SUBSYSTEM: Membrane Lipid Metabolism</p>
            <p>EC Number: 2.3.1.180 and (2.3.1.179 or 2.3.1.180) and 1.1.1.100 and 4.2.1.59</p>
            <p>Confidence Level: 1</p>
            <p>AUTHORS: Lipid metabolism</p>
```

```
      <p/>
    </body>
  </notes>
  <listOfReactants>
    <speciesReference species="M_H_c" stoichiometry="14"/>
    <speciesReference species="M_NADPH_c" stoichiometry="10"/>
    <speciesReference species="M_MALCoA_c" stoichiometry="5"/>
    <speciesReference species="M_IVCoA_c" stoichiometry="1"/>
  </listOfReactants>
  <listOfProducts>
    <speciesReference species="M_H2O_c" stoichiometry="4"/>
    <speciesReference species="M_CO2_c" stoichiometry="5"/>
    <speciesReference species="M_CoA_c" stoichiometry="6"/>
    <speciesReference species="M_NADP_c" stoichiometry="10"/>
    <speciesReference species="M_I150_c" stoichiometry="1"/>
  </listOfProducts>
  <kineticLaw>
    <math xmlns="http://www.w3.org/1998/Math/MathML">
      <ci> FLUX_VALUE </ci>
    </math>
    <listOfParameters>
      <parameter id="LOWER_BOUND" value="0" units="mmol_per_gDW_per_hr" constant="true"/>
      <parameter id="UPPER_BOUND" value="1000" units="mmol_per_gDW_per_hr" constant="true"/>
      <parameter id="FLUX_VALUE" value="0" units="mmol_per_gDW_per_hr" constant="true"/>
      <parameter id="OBJECTIVE_COEFFICIENT" value="0" units="mmol_per_gDW_per_hr" constant="true"/>
    </listOfParameters>
  </kineticLaw>
</reaction>
<reaction id="R_R670" name="fatty acid synthesis; lumped" reversible="false">
```

```
<notes>
  <body xmlns="http://www.w3.org/1999/xhtml">
    <p>GENE_ASSOCIATION: ((TTC0049 or TTC0045) and (TTC0045 or (TTC0049 or TTC0343)) and (TTC0047 or TTC0394) and TTC1463 and TTC0048)</p>
    <p>SUBSYSTEM: Membrane Lipid Metabolism</p>
    <p>EC Number: 2.3.1.180 and (2.3.1.179 or 2.3.1.180) and 1.1.1.100 and 4.2.1.59</p>
    <p>Confidence Level: 1</p>
    <p>AUTHORS: Lipid metabolism</p>
    <p/>
  </body>
</notes>
<listOfReactants>
  <speciesReference species="M_H_c" stoichiometry="17"/>
  <speciesReference species="M_NADPH_c" stoichiometry="12"/>
  <speciesReference species="M_MALCoA_c" stoichiometry="6"/>
  <speciesReference species="M_2M_PROCoA_c" stoichiometry="1"/>
</listOfReactants>
<listOfProducts>
  <speciesReference species="M_H2O_c" stoichiometry="5"/>
  <speciesReference species="M_CO2_c" stoichiometry="6"/>
  <speciesReference species="M_CoA_c" stoichiometry="7"/>
  <speciesReference species="M_NADP_c" stoichiometry="12"/>
  <speciesReference species="M_I160_c" stoichiometry="1"/>
</listOfProducts>
<kineticLaw>
  <math xmlns="http://www.w3.org/1998/Math/MathML">
    <ci> FLUX_VALUE </ci>
  </math>
  <listOfParameters>
```

```
<parameter id="LOWER_BOUND" value="0" units="mmol_per_gDW_per_hr" constant="true"/>
<parameter id="UPPER_BOUND" value="1000" units="mmol_per_gDW_per_hr" constant="true"/>
<parameter id="FLUX_VALUE" value="0" units="mmol_per_gDW_per_hr" constant="true"/>
<parameter id="OBJECTIVE_COEFFICIENT" value="0" units="mmol_per_gDW_per_hr" constant="true"/>
</listOfParameters>
</kineticLaw>
</reaction>
<reaction id="R_R671" name="fatty acid synthesis; lumped" reversible="false">
<notes>
<body xmlns="http://www.w3.org/1999/xhtml">
<p>GENE_ASSOCIATION: ((TTC0049 or TTC0045) and (TTC0045 or (TTC0049 or TTC0343)) and (TTC0047 or TTC0394) and TTC1463 and TTC0048)</p>
<p>SUBSYSTEM: Membrane Lipid Metabolism</p>
<p>EC Number: 2.3.1.180 and (2.3.1.179 or 2.3.1.180) and 1.1.1.100 and 4.2.1.59</p>
<p>Confidence Level: 1</p>
<p>AUTHORS: Lipid metabolism</p>
<p/>
</body>
</notes>
<listOfReactants>
<speciesReference species="M_H_c" stoichiometry="16"/>
<speciesReference species="M_NADPH_c" stoichiometry="12"/>
<speciesReference species="M_MALCoA_c" stoichiometry="6"/>
<speciesReference species="M_IVCoA_c" stoichiometry="1"/>
</listOfReactants>
<listOfProducts>
<speciesReference species="M_H2O_c" stoichiometry="5"/>
<speciesReference species="M_CO2_c" stoichiometry="6"/>
<speciesReference species="M_CoA_c" stoichiometry="7"/>
```

```xml
<speciesReference species="M_NADP_c" stoichiometry="12"/>
<speciesReference species="M_I170_c" stoichiometry="1"/>
</listOfProducts>
<kineticLaw>
<math xmlns="http://www.w3.org/1998/Math/MathML">
<ci> FLUX_VALUE </ci>
</math>
<listOfParameters>
<parameter id="LOWER_BOUND" value="0" units="mmol_per_gDW_per_hr" constant="true"/>
<parameter id="UPPER_BOUND" value="1000" units="mmol_per_gDW_per_hr" constant="true"/>
<parameter id="FLUX_VALUE" value="0" units="mmol_per_gDW_per_hr" constant="true"/>
<parameter id="OBJECTIVE_COEFFICIENT" value="0" units="mmol_per_gDW_per_hr" constant="true"/>
</listOfParameters>
</kineticLaw>
</reaction>
<reaction id="R_R672" name="fatty acid synthesis; lumped" reversible="false">
<notes>
<body xmlns="http://www.w3.org/1999/xhtml">
<p>GENE_ASSOCIATION: ((TTC0049 or TTC0045) and (TTC0045 or (TTC0049 or TTC0343)) and (TTC0047 or TTC0394) and TTC1463 and TTC0048)</p>
<p>SUBSYSTEM: Membrane Lipid Metabolism</p>
<p>EC Number: 2.3.1.180 and (2.3.1.179 or 2.3.1.180) and 1.1.1.100 and 4.2.1.59</p>
<p>Confidence Level: 1</p>
<p>AUTHORS: Lipid metabolism</p>
<p/>
</body>
</notes>
<listOfReactants>
<speciesReference species="M_H_c" stoichiometry="12"/>
```

```
<speciesReference species="M_NADPH_c" stoichiometry="8"/>
<speciesReference species="M_MALCoA_c" stoichiometry="4"/>
<speciesReference species="M_IVCoA_c" stoichiometry="1"/>
</listOfReactants>
<listOfProducts>
<speciesReference species="M_H2O_c" stoichiometry="3"/>
<speciesReference species="M_CO2_c" stoichiometry="4"/>
<speciesReference species="M_CoA_c" stoichiometry="5"/>
<speciesReference species="M_NADP_c" stoichiometry="8"/>
<speciesReference species="M_I130_c" stoichiometry="1"/>
</listOfProducts>
<kineticLaw>
<math xmlns="http://www.w3.org/1998/Math/MathML">
<ci> FLUX_VALUE </ci>
</math>
<listOfParameters>
<parameter id="LOWER_BOUND" value="0" units="mmol_per_gDW_per_hr" constant="true"/>
<parameter id="UPPER_BOUND" value="1000" units="mmol_per_gDW_per_hr" constant="true"/>
<parameter id="FLUX_VALUE" value="0" units="mmol_per_gDW_per_hr" constant="true"/>
<parameter id="OBJECTIVE_COEFFICIENT" value="0" units="mmol_per_gDW_per_hr" constant="true"/>
</listOfParameters>
</kineticLaw>
</reaction>
<reaction id="R_R673" name="fatty acid synthesis; lumped" reversible="false">
<notes>
<body xmlns="http://www.w3.org/1999/xhtml">
<p>GENE_ASSOCIATION: ((TTC0049 or TTC0045) and (TTC0045 or (TTC0049 or TTC0343)) and (TTC0047 or TTC0394) and TTC1463 and TTC0048)</p>
<p>SUBSYSTEM: Membrane Lipid Metabolism</p>
```

```
    <p>EC Number: 2.3.1.180 and (2.3.1.179 or 2.3.1.180) and 1.1.1.100 and 4.2.1.59</p>
    <p>Confidence Level: 1</p>
    <p>AUTHORS: Lipid metabolism</p>
    <p/>
  </body>
</notes>
<listOfReactants>
  <speciesReference species="M_H_c" stoichiometry="10"/>
  <speciesReference species="M_NADPH_c" stoichiometry="6"/>
  <speciesReference species="M_MALCoA_c" stoichiometry="3"/>
  <speciesReference species="M_IVCoA_c" stoichiometry="1"/>
</listOfReactants>
<listOfProducts>
  <speciesReference species="M_H2O_c" stoichiometry="2"/>
  <speciesReference species="M_CO2_c" stoichiometry="3"/>
  <speciesReference species="M_CoA_c" stoichiometry="6"/>
  <speciesReference species="M_NADP_c" stoichiometry="6"/>
  <speciesReference species="M_I110_c" stoichiometry="1"/>
</listOfProducts>
<kineticLaw>
  <math xmlns="http://www.w3.org/1998/Math/MathML">
    <ci> FLUX_VALUE </ci>
  </math>
  <listOfParameters>
    <parameter id="LOWER_BOUND" value="0" units="mmol_per_gDW_per_hr" constant="true"/>
    <parameter id="UPPER_BOUND" value="1000" units="mmol_per_gDW_per_hr" constant="true"/>
    <parameter id="FLUX_VALUE" value="0" units="mmol_per_gDW_per_hr" constant="true"/>
    <parameter id="OBJECTIVE_COEFFICIENT" value="0" units="mmol_per_gDW_per_hr" constant="true"/>
  </listOfParameters>
```

</kineticLaw>
</reaction>
<reaction id="R_R674" name="fatty acid synthesis; lumped" reversible="false">
<notes>
<body xmlns="http://www.w3.org/1999/xhtml">
<p>GENE_ASSOCIATION: ((TTC0049 or TTC0045) and (TTC0045 or (TTC0049 or TTC0343)) and (TTC0047 or TTC0394) and TTC1463 and TTC0048 and TTC1678)</p>
<p>SUBSYSTEM: Membrane Lipid Metabolism</p>
<p>EC Number: 2.3.1.180 and (2.3.1.179 or 2.3.1.180) and 1.1.1.100 and 4.2.1.59</p>
<p>Confidence Level: 1</p>
<p>AUTHORS: Lipid metabolism</p>
<p/>
</body>
</notes>
<listOfReactants>
<speciesReference species="M_H_c" stoichiometry="20"/>
<speciesReference species="M_ACoA_c" stoichiometry="1"/>
<speciesReference species="M_NADPH_c" stoichiometry="14"/>
<speciesReference species="M_MALCoA_c" stoichiometry="7"/>
</listOfReactants>
<listOfProducts>
<speciesReference species="M_H2O_c" stoichiometry="6"/>
<speciesReference species="M_CO2_c" stoichiometry="7"/>
<speciesReference species="M_CoA_c" stoichiometry="8"/>
<speciesReference species="M_NADP_c" stoichiometry="14"/>
<speciesReference species="M_C160_c" stoichiometry="1"/>
</listOfProducts>
<kineticLaw>
<math xmlns="http://www.w3.org/1998/Math/MathML">

```
<ci> FLUX_VALUE </ci>
</math>
<listOfParameters>
<parameter id="LOWER_BOUND" value="0" units="mmol_per_gDW_per_hr" constant="true"/>
<parameter id="UPPER_BOUND" value="1000" units="mmol_per_gDW_per_hr" constant="true"/>
<parameter id="FLUX_VALUE" value="0" units="mmol_per_gDW_per_hr" constant="true"/>
<parameter id="OBJECTIVE_COEFFICIENT" value="0" units="mmol_per_gDW_per_hr" constant="true"/>
</listOfParameters>
</kineticLaw>
</reaction>
<reaction id="R_R675" name="fatty acid synthesis; lumped" reversible="false">
<notes>
<body xmlns="http://www.w3.org/1999/xhtml">
<p>GENE_ASSOCIATION: ((TTC0049 or TTC0045) and (TTC0045 or (TTC0049 or TTC0343)) and (TTC0047 or TTC0394) and TTC1463 and (TTC0238 or TTC0536 or TTC779 or TTC1552 or TTC1575 or TT_P0074) and TTC0048 and TTC1678)</p>
<p>SUBSYSTEM: Membrane Lipid Metabolism</p>
<p>EC Number: 2.3.1.180 and (2.3.1.179 or 2.3.1.180) and 1.1.1.100 and 4.2.1.59</p>
<p>Confidence Level: 1</p>
<p>AUTHORS: Lipid metabolism</p>
<p/>
</body>
</notes>
<listOfReactants>
<speciesReference species="M_H_c" stoichiometry="21"/>
<speciesReference species="M_ACoA_c" stoichiometry="1"/>
<speciesReference species="M_NADPH_c" stoichiometry="15"/>
<speciesReference species="M_O2_c" stoichiometry="1"/>
<speciesReference species="M_MALCoA_c" stoichiometry="7"/>
</listOfReactants>
```

```
<listOfProducts>
  <speciesReference species="M_H2O_c" stoichiometry="6"/>
  <speciesReference species="M_CO2_c" stoichiometry="7"/>
  <speciesReference species="M_CoA_c" stoichiometry="8"/>
  <speciesReference species="M_NADP_c" stoichiometry="15"/>
  <speciesReference species="M_C161_c" stoichiometry="1"/>
</listOfProducts>
<kineticLaw>
  <math xmlns="http://www.w3.org/1998/Math/MathML">
    <ci> FLUX_VALUE </ci>
  </math>
  <listOfParameters>
    <parameter id="LOWER_BOUND" value="0" units="mmol_per_gDW_per_hr" constant="true"/>
    <parameter id="UPPER_BOUND" value="1000" units="mmol_per_gDW_per_hr" constant="true"/>
    <parameter id="FLUX_VALUE" value="0" units="mmol_per_gDW_per_hr" constant="true"/>
    <parameter id="OBJECTIVE_COEFFICIENT" value="0" units="mmol_per_gDW_per_hr" constant="true"/>
  </listOfParameters>
</kineticLaw>
</reaction>
<reaction id="R_R676" name="fatty acid oxidation; lumped" reversible="false">
<notes>
  <body xmlns="http://www.w3.org/1999/xhtml">
    <p>GENE_ASSOCIATION: ((TTC0236 or TTC0342) and (TT_P0074 or TTC0238 or TTC0536) and (TTC0604 or TTC1697 or
TTC1768) and (TTC0331 or TTC0534) and (TTC0623 or TTC0535))</p>
    <p>SUBSYSTEM: Membrane Lipid Metabolism</p>
    <p>EC Number: 1.3.8.- and 4.2.1.17 and 1.1.1.35 and 2.3.1.16</p>
    <p>Confidence Level: 1</p>
    <p>AUTHORS: Lipid metabolism</p>
    <p/>
```

```
    </body>
  </notes>
  <listOfReactants>
    <speciesReference species="M_H2O_c" stoichiometry="7"/>
    <speciesReference species="M_NAD_c" stoichiometry="7"/>
    <speciesReference species="M_CoA_c" stoichiometry="7"/>
    <speciesReference species="M_NADPH_c" stoichiometry="1"/>
    <speciesReference species="M_O2_c" stoichiometry="7"/>
    <speciesReference species="M_C161CoA_c" stoichiometry="1"/>
  </listOfReactants>
  <listOfProducts>
    <speciesReference species="M_H_c" stoichiometry="6"/>
    <speciesReference species="M_NADH_c" stoichiometry="7"/>
    <speciesReference species="M_ACoA_c" stoichiometry="8"/>
    <speciesReference species="M_NADP_c" stoichiometry="1"/>
    <speciesReference species="M_H2O2_c" stoichiometry="7"/>
  </listOfProducts>
  <kineticLaw>
    <math xmlns="http://www.w3.org/1998/Math/MathML">
      <ci> FLUX_VALUE </ci>
    </math>
    <listOfParameters>
      <parameter id="LOWER_BOUND" value="0" units="mmol_per_gDW_per_hr" constant="true"/>
      <parameter id="UPPER_BOUND" value="1000" units="mmol_per_gDW_per_hr" constant="true"/>
      <parameter id="FLUX_VALUE" value="0" units="mmol_per_gDW_per_hr" constant="true"/>
      <parameter id="OBJECTIVE_COEFFICIENT" value="0" units="mmol_per_gDW_per_hr" constant="true"/>
    </listOfParameters>
  </kineticLaw>
</reaction>
```

```
<reaction id="R_R677" name="fatty acid oxidation; lumped" reversible="false">
  <notes>
    <body xmlns="http://www.w3.org/1999/xhtml">
      <p>GENE_ASSOCIATION: ((TTC0236 or TTC0342) and (TT_P0074 or TTC0238 or TTC0536) and (TTC0604 or TTC1697 or TTC1768) and (TTC0331 or TTC0534) and (TTC0623 or TTC0535))</p>
      <p>SUBSYSTEM: Membrane Lipid Metabolism</p>
      <p>EC Number: 1.3.8.- and 4.2.1.17 and 1.1.1.35 and 2.3.1.16</p>
      <p>Confidence Level: 1</p>
      <p>AUTHORS: Lipid metabolism</p>
      <p/>
    </body>
  </notes>
  <listOfReactants>
    <speciesReference species="M_H2O_c" stoichiometry="7"/>
    <speciesReference species="M_NAD_c" stoichiometry="7"/>
    <speciesReference species="M_CoA_c" stoichiometry="7"/>
    <speciesReference species="M_O2_c" stoichiometry="6"/>
    <speciesReference species="M_C161CoA_c" stoichiometry="1"/>
  </listOfReactants>
  <listOfProducts>
    <speciesReference species="M_H_c" stoichiometry="7"/>
    <speciesReference species="M_NADH_c" stoichiometry="7"/>
    <speciesReference species="M_ACoA_c" stoichiometry="8"/>
    <speciesReference species="M_H2O2_c" stoichiometry="6"/>
  </listOfProducts>
  <kineticLaw>
    <math xmlns="http://www.w3.org/1998/Math/MathML">
      <ci> FLUX_VALUE </ci>
    </math>
```

```xml
<listOfParameters>
<parameter id="LOWER_BOUND" value="0" units="mmol_per_gDW_per_hr" constant="true"/>
<parameter id="UPPER_BOUND" value="1000" units="mmol_per_gDW_per_hr" constant="true"/>
<parameter id="FLUX_VALUE" value="0" units="mmol_per_gDW_per_hr" constant="true"/>
<parameter id="OBJECTIVE_COEFFICIENT" value="0" units="mmol_per_gDW_per_hr" constant="true"/>
</listOfParameters>
</kineticLaw>
</reaction>
<reaction id="R_R678" name="fatty acid coA--ligase" reversible="true">
<notes>
<body xmlns="http://www.w3.org/1999/xhtml">
<p>GENE_ASSOCIATION: (TTC0079 or TTC1065 or TTC1099)</p>
<p>SUBSYSTEM: Membrane Lipid Metabolism</p>
<p>EC Number: 6.2.1.3</p>
<p>Confidence Level: 1</p>
<p>AUTHORS: Lipid metabolism</p>
<p/>
</body>
</notes>
<listOfReactants>
<speciesReference species="M_ATP_c" stoichiometry="1"/>
<speciesReference species="M_CoA_c" stoichiometry="1"/>
<speciesReference species="M_AI171_c" stoichiometry="1"/>
</listOfReactants>
<listOfProducts>
<speciesReference species="M_PPi_c" stoichiometry="1"/>
<speciesReference species="M_AMP_c" stoichiometry="1"/>
<speciesReference species="M_AI171CoA_c" stoichiometry="1"/>
</listOfProducts>
```

```
  <kineticLaw>
   <math xmlns="http://www.w3.org/1998/Math/MathML">
    <ci> FLUX_VALUE </ci>
   </math>
   <listOfParameters>
    <parameter id="LOWER_BOUND" value="-1000" units="mmol_per_gDW_per_hr" constant="true"/>
    <parameter id="UPPER_BOUND" value="1000" units="mmol_per_gDW_per_hr" constant="true"/>
    <parameter id="FLUX_VALUE" value="0" units="mmol_per_gDW_per_hr" constant="true"/>
    <parameter id="OBJECTIVE_COEFFICIENT" value="0" units="mmol_per_gDW_per_hr" constant="true"/>
   </listOfParameters>
  </kineticLaw>
 </reaction>
 <reaction id="R_R679" name="fatty acid coA--ligase" reversible="true">
  <notes>
   <body xmlns="http://www.w3.org/1999/xhtml">
    <p>GENE_ASSOCIATION: (TTC0079 or TTC1065 or TTC1099)</p>
    <p>SUBSYSTEM: Membrane Lipid Metabolism</p>
    <p>EC Number: 6.2.1.3</p>
    <p>Confidence Level: 1</p>
    <p>AUTHORS: Lipid metabolism</p>
    <p/>
   </body>
  </notes>
  <listOfReactants>
   <speciesReference species="M_ATP_c" stoichiometry="1"/>
   <speciesReference species="M_CoA_c" stoichiometry="1"/>
   <speciesReference species="M_AI150_c" stoichiometry="1"/>
  </listOfReactants>
  <listOfProducts>
```

```
    <speciesReference species="M_PPi_c" stoichiometry="1"/>
    <speciesReference species="M_AMP_c" stoichiometry="1"/>
    <speciesReference species="M_AI150CoA_c" stoichiometry="1"/>
  </listOfProducts>
  <kineticLaw>
    <math xmlns="http://www.w3.org/1998/Math/MathML">
      <ci> FLUX_VALUE </ci>
    </math>
    <listOfParameters>
      <parameter id="LOWER_BOUND" value="-1000" units="mmol_per_gDW_per_hr" constant="true"/>
      <parameter id="UPPER_BOUND" value="1000" units="mmol_per_gDW_per_hr" constant="true"/>
      <parameter id="FLUX_VALUE" value="0" units="mmol_per_gDW_per_hr" constant="true"/>
      <parameter id="OBJECTIVE_COEFFICIENT" value="0" units="mmol_per_gDW_per_hr" constant="true"/>
    </listOfParameters>
  </kineticLaw>
</reaction>
<reaction id="R_R680" name="fatty acid coA--ligase" reversible="true">
  <notes>
    <body xmlns="http://www.w3.org/1999/xhtml">
      <p>GENE_ASSOCIATION: (TTC0079 or TTC1065 or TTC1099)</p>
      <p>SUBSYSTEM: Membrane Lipid Metabolism</p>
      <p>EC Number: 6.2.1.3</p>
      <p>Confidence Level: 1</p>
      <p>AUTHORS: Lipid metabolism</p>
      <p/>
    </body>
  </notes>
  <listOfReactants>
    <speciesReference species="M_ATP_c" stoichiometry="1"/>
```

```xml
    <speciesReference species="M_CoA_c" stoichiometry="1"/>
    <speciesReference species="M_I150_c" stoichiometry="1"/>
  </listOfReactants>
  <listOfProducts>
    <speciesReference species="M_PPi_c" stoichiometry="1"/>
    <speciesReference species="M_AMP_c" stoichiometry="1"/>
    <speciesReference species="M_I150CoA_c" stoichiometry="1"/>
  </listOfProducts>
  <kineticLaw>
    <math xmlns="http://www.w3.org/1998/Math/MathML">
      <ci> FLUX_VALUE </ci>
    </math>
    <listOfParameters>
      <parameter id="LOWER_BOUND" value="-1000" units="mmol_per_gDW_per_hr" constant="true"/>
      <parameter id="UPPER_BOUND" value="1000" units="mmol_per_gDW_per_hr" constant="true"/>
      <parameter id="FLUX_VALUE" value="0" units="mmol_per_gDW_per_hr" constant="true"/>
      <parameter id="OBJECTIVE_COEFFICIENT" value="0" units="mmol_per_gDW_per_hr" constant="true"/>
    </listOfParameters>
  </kineticLaw>
</reaction>
<reaction id="R_R681" name="fatty acid coA--ligase" reversible="true">
  <notes>
    <body xmlns="http://www.w3.org/1999/xhtml">
      <p>GENE_ASSOCIATION: (TTC0079 or TTC1065 or TTC1099)</p>
      <p>SUBSYSTEM: Membrane Lipid Metabolism</p>
      <p>EC Number: 6.2.1.3</p>
      <p>Confidence Level: 1</p>
      <p>AUTHORS: Lipid metabolism</p>
      <p/>
```

```
    </body>
  </notes>
  <listOfReactants>
    <speciesReference species="M_ATP_c" stoichiometry="1"/>
    <speciesReference species="M_CoA_c" stoichiometry="1"/>
    <speciesReference species="M_I160_c" stoichiometry="1"/>
  </listOfReactants>
  <listOfProducts>
    <speciesReference species="M_PPi_c" stoichiometry="1"/>
    <speciesReference species="M_AMP_c" stoichiometry="1"/>
    <speciesReference species="M_I160CoA_c" stoichiometry="1"/>
  </listOfProducts>
  <kineticLaw>
    <math xmlns="http://www.w3.org/1998/Math/MathML">
      <ci> FLUX_VALUE </ci>
    </math>
    <listOfParameters>
      <parameter id="LOWER_BOUND" value="-1000" units="mmol_per_gDW_per_hr" constant="true"/>
      <parameter id="UPPER_BOUND" value="1000" units="mmol_per_gDW_per_hr" constant="true"/>
      <parameter id="FLUX_VALUE" value="0" units="mmol_per_gDW_per_hr" constant="true"/>
      <parameter id="OBJECTIVE_COEFFICIENT" value="0" units="mmol_per_gDW_per_hr" constant="true"/>
    </listOfParameters>
  </kineticLaw>
</reaction>
<reaction id="R_R682" name="fatty acid coA--ligase" reversible="true">
  <notes>
    <body xmlns="http://www.w3.org/1999/xhtml">
      <p>GENE_ASSOCIATION: (TTC0079 or TTC1065 or TTC1099)</p>
      <p>SUBSYSTEM: Membrane Lipid Metabolism</p>
```

```
      <p>EC Number: 6.2.1.3</p>
      <p>Confidence Level: 1</p>
      <p>AUTHORS: Lipid metabolism</p>
      <p/>
    </body>
  </notes>
  <listOfReactants>
    <speciesReference species="M_ATP_c" stoichiometry="1"/>
    <speciesReference species="M_CoA_c" stoichiometry="1"/>
    <speciesReference species="M_I170_c" stoichiometry="1"/>
  </listOfReactants>
  <listOfProducts>
    <speciesReference species="M_PPi_c" stoichiometry="1"/>
    <speciesReference species="M_AMP_c" stoichiometry="1"/>
    <speciesReference species="M_I170CoA_c" stoichiometry="1"/>
  </listOfProducts>
  <kineticLaw>
    <math xmlns="http://www.w3.org/1998/Math/MathML">
      <ci> FLUX_VALUE </ci>
    </math>
    <listOfParameters>
      <parameter id="LOWER_BOUND" value="-1000" units="mmol_per_gDW_per_hr" constant="true"/>
      <parameter id="UPPER_BOUND" value="1000" units="mmol_per_gDW_per_hr" constant="true"/>
      <parameter id="FLUX_VALUE" value="0" units="mmol_per_gDW_per_hr" constant="true"/>
      <parameter id="OBJECTIVE_COEFFICIENT" value="0" units="mmol_per_gDW_per_hr" constant="true"/>
    </listOfParameters>
  </kineticLaw>
</reaction>
<reaction id="R_R683" name="fatty acid coA--ligase" reversible="true">
```

```
<notes>
  <body xmlns="http://www.w3.org/1999/xhtml">
    <p>GENE_ASSOCIATION: (TTC0079 or TTC1065 or TTC1099)</p>
    <p>SUBSYSTEM: Membrane Lipid Metabolism</p>
    <p>EC Number: 6.2.1.3</p>
    <p>Confidence Level: 1</p>
    <p>AUTHORS: Lipid metabolism</p>
    <p/>
  </body>
</notes>
<listOfReactants>
  <speciesReference species="M_ATP_c" stoichiometry="1"/>
  <speciesReference species="M_CoA_c" stoichiometry="1"/>
  <speciesReference species="M_C160_c" stoichiometry="1"/>
</listOfReactants>
<listOfProducts>
  <speciesReference species="M_PPi_c" stoichiometry="1"/>
  <speciesReference species="M_AMP_c" stoichiometry="1"/>
  <speciesReference species="M_C160CoA_c" stoichiometry="1"/>
</listOfProducts>
<kineticLaw>
  <math xmlns="http://www.w3.org/1998/Math/MathML">
    <ci> FLUX_VALUE </ci>
  </math>
  <listOfParameters>
    <parameter id="LOWER_BOUND" value="-1000" units="mmol_per_gDW_per_hr" constant="true"/>
    <parameter id="UPPER_BOUND" value="1000" units="mmol_per_gDW_per_hr" constant="true"/>
    <parameter id="FLUX_VALUE" value="0" units="mmol_per_gDW_per_hr" constant="true"/>
    <parameter id="OBJECTIVE_COEFFICIENT" value="0" units="mmol_per_gDW_per_hr" constant="true"/>
```

```
    </listOfParameters>
  </kineticLaw>
</reaction>
<reaction id="R_R684" name="fatty acid coA--ligase" reversible="true">
  <notes>
    <body xmlns="http://www.w3.org/1999/xhtml">
      <p>GENE_ASSOCIATION: (TTC0079 or TTC1065 or TTC1099)</p>
      <p>SUBSYSTEM: Membrane Lipid Metabolism</p>
      <p>EC Number: 6.2.1.3</p>
      <p>Confidence Level: 1</p>
      <p>AUTHORS: Lipid metabolism</p>
      <p/>
    </body>
  </notes>
  <listOfReactants>
    <speciesReference species="M_ATP_c" stoichiometry="1"/>
    <speciesReference species="M_CoA_c" stoichiometry="1"/>
    <speciesReference species="M_C161_c" stoichiometry="1"/>
  </listOfReactants>
  <listOfProducts>
    <speciesReference species="M_PPi_c" stoichiometry="1"/>
    <speciesReference species="M_AMP_c" stoichiometry="1"/>
    <speciesReference species="M_C161CoA_c" stoichiometry="1"/>
  </listOfProducts>
  <kineticLaw>
    <math xmlns="http://www.w3.org/1998/Math/MathML">
      <ci> FLUX_VALUE </ci>
    </math>
    <listOfParameters>
```

```
      <parameter id="LOWER_BOUND" value="-1000" units="mmol_per_gDW_per_hr" constant="true"/>
      <parameter id="UPPER_BOUND" value="1000" units="mmol_per_gDW_per_hr" constant="true"/>
      <parameter id="FLUX_VALUE" value="0" units="mmol_per_gDW_per_hr" constant="true"/>
      <parameter id="OBJECTIVE_COEFFICIENT" value="0" units="mmol_per_gDW_per_hr" constant="true"/>
    </listOfParameters>
  </kineticLaw>
</reaction>
<reaction id="R_R685" name="lycopene epsilon-cyclase" reversible="true">
  <notes>
    <body xmlns="http://www.w3.org/1999/xhtml">
      <p>GENE_ASSOCIATION: </p>
      <p>SUBSYSTEM: Carotenoid biosynthesis</p>
      <p>EC Number: 5.5.1.18</p>
      <p>Confidence Level: 1</p>
      <p>AUTHORS: Carotenoid metabolism</p>
      <p/>
    </body>
  </notes>
  <listOfReactants>
    <speciesReference species="M_LCPN_c" stoichiometry="1"/>
  </listOfReactants>
  <listOfProducts>
    <speciesReference species="M_gCARO_c" stoichiometry="1"/>
  </listOfProducts>
  <kineticLaw>
    <math xmlns="http://www.w3.org/1998/Math/MathML">
      <ci> FLUX_VALUE </ci>
    </math>
    <listOfParameters>
```

```
      <parameter id="LOWER_BOUND" value="-1000" units="mmol_per_gDW_per_hr" constant="true"/>
      <parameter id="UPPER_BOUND" value="1000" units="mmol_per_gDW_per_hr" constant="true"/>
      <parameter id="FLUX_VALUE" value="0" units="mmol_per_gDW_per_hr" constant="true"/>
      <parameter id="OBJECTIVE_COEFFICIENT" value="0" units="mmol_per_gDW_per_hr" constant="true"/>
    </listOfParameters>
  </kineticLaw>
</reaction>
<reaction id="R_R686" name="lycopene beta-cyclase" reversible="true">
  <notes>
    <body xmlns="http://www.w3.org/1999/xhtml">
      <p>GENE_ASSOCIATION: </p>
      <p>SUBSYSTEM: Carotenoid biosynthesis</p>
      <p>EC Number: 5.5.1.18</p>
      <p>Confidence Level: 1</p>
      <p>AUTHORS: Carotenoid metabolism</p>
      <p/>
    </body>
  </notes>
  <listOfReactants>
    <speciesReference species="M_gCARO_c" stoichiometry="1"/>
  </listOfReactants>
  <listOfProducts>
    <speciesReference species="M_bCARO_c" stoichiometry="1"/>
  </listOfProducts>
  <kineticLaw>
    <math xmlns="http://www.w3.org/1998/Math/MathML">
      <ci> FLUX_VALUE </ci>
    </math>
    <listOfParameters>
```

```
      <parameter id="LOWER_BOUND" value="-1000" units="mmol_per_gDW_per_hr" constant="true"/>
      <parameter id="UPPER_BOUND" value="1000" units="mmol_per_gDW_per_hr" constant="true"/>
      <parameter id="FLUX_VALUE" value="0" units="mmol_per_gDW_per_hr" constant="true"/>
      <parameter id="OBJECTIVE_COEFFICIENT" value="0" units="mmol_per_gDW_per_hr" constant="true"/>
    </listOfParameters>
  </kineticLaw>
</reaction>
<reaction id="R_R687" name="UTP--glucose-1-phosphate uridylyltransferase" reversible="true">
  <notes>
    <body xmlns="http://www.w3.org/1999/xhtml">
      <p>GENE_ASSOCIATION: </p>
      <p>SUBSYSTEM: Alternate Carbon Metabolism</p>
      <p>EC Number: </p>
      <p>Confidence Level: 1</p>
      <p>AUTHORS: Carbohydrate metabolism</p>
      <p/>
    </body>
  </notes>
  <listOfReactants>
    <speciesReference species="M_UTP_c" stoichiometry="1"/>
    <speciesReference species="M_H_c" stoichiometry="1"/>
    <speciesReference species="M_G1P_c" stoichiometry="1"/>
  </listOfReactants>
  <listOfProducts>
    <speciesReference species="M_PPi_c" stoichiometry="1"/>
    <speciesReference species="M_UDP_GLU_c" stoichiometry="1"/>
  </listOfProducts>
  <kineticLaw>
    <math xmlns="http://www.w3.org/1998/Math/MathML">
```

```
      <ci> FLUX_VALUE </ci>
     </math>
     <listOfParameters>
      <parameter id="LOWER_BOUND" value="-1000" units="mmol_per_gDW_per_hr" constant="true"/>
      <parameter id="UPPER_BOUND" value="1000" units="mmol_per_gDW_per_hr" constant="true"/>
      <parameter id="FLUX_VALUE" value="0" units="mmol_per_gDW_per_hr" constant="true"/>
      <parameter id="OBJECTIVE_COEFFICIENT" value="0" units="mmol_per_gDW_per_hr" constant="true"/>
     </listOfParameters>
    </kineticLaw>
   </reaction>
   <reaction id="R_R688" name="beta-carotene hydroxylase" reversible="true">
    <notes>
     <body xmlns="http://www.w3.org/1999/xhtml">
      <p>GENE_ASSOCIATION: TT_P0059</p>
      <p>SUBSYSTEM: Carotenoid biosynthesis</p>
      <p>EC Number: </p>
      <p>Confidence Level: 1</p>
      <p>AUTHORS: Carotenoid metabolism</p>
      <p/>
     </body>
    </notes>
    <listOfReactants>
     <speciesReference species="M_H_c" stoichiometry="1"/>
     <speciesReference species="M_NADH_c" stoichiometry="1"/>
     <speciesReference species="M_O2_c" stoichiometry="1"/>
     <speciesReference species="M_bCARO_c" stoichiometry="1"/>
    </listOfReactants>
    <listOfProducts>
     <speciesReference species="M_H2O_c" stoichiometry="1"/>
```

```
    <speciesReference species="M_NAD_c" stoichiometry="1"/>
    <speciesReference species="M_CRTX_c" stoichiometry="1"/>
  </listOfProducts>
  <kineticLaw>
    <math xmlns="http://www.w3.org/1998/Math/MathML">
      <ci> FLUX_VALUE </ci>
    </math>
    <listOfParameters>
      <parameter id="LOWER_BOUND" value="-1000" units="mmol_per_gDW_per_hr" constant="true"/>
      <parameter id="UPPER_BOUND" value="1000" units="mmol_per_gDW_per_hr" constant="true"/>
      <parameter id="FLUX_VALUE" value="0" units="mmol_per_gDW_per_hr" constant="true"/>
      <parameter id="OBJECTIVE_COEFFICIENT" value="0" units="mmol_per_gDW_per_hr" constant="true"/>
    </listOfParameters>
  </kineticLaw>
</reaction>
<reaction id="R_R689" name="beta-cryptoxanthin hydroxylase " reversible="false">
  <notes>
    <body xmlns="http://www.w3.org/1999/xhtml">
      <p>GENE_ASSOCIATION: TT_P0059</p>
      <p>SUBSYSTEM: Carotenoid biosynthesis</p>
      <p>EC Number: </p>
      <p>Confidence Level: 1</p>
      <p>AUTHORS: Carotenoid metabolism</p>
      <p/>
    </body>
  </notes>
  <listOfReactants>
    <speciesReference species="M_H_c" stoichiometry="1"/>
    <speciesReference species="M_NADH_c" stoichiometry="1"/>
```

```xml
<speciesReference species="M_O2_c" stoichiometry="1"/>
<speciesReference species="M_CRTX_c" stoichiometry="1"/>
</listOfReactants>
<listOfProducts>
<speciesReference species="M_H2O_c" stoichiometry="1"/>
<speciesReference species="M_NAD_c" stoichiometry="1"/>
<speciesReference species="M_ZXT_c" stoichiometry="1"/>
</listOfProducts>
<kineticLaw>
<math xmlns="http://www.w3.org/1998/Math/MathML">
<ci> FLUX_VALUE </ci>
</math>
<listOfParameters>
<parameter id="LOWER_BOUND" value="0" units="mmol_per_gDW_per_hr" constant="true"/>
<parameter id="UPPER_BOUND" value="1000" units="mmol_per_gDW_per_hr" constant="true"/>
<parameter id="FLUX_VALUE" value="0" units="mmol_per_gDW_per_hr" constant="true"/>
<parameter id="OBJECTIVE_COEFFICIENT" value="0" units="mmol_per_gDW_per_hr" constant="true"/>
</listOfParameters>
</kineticLaw>
</reaction>
<reaction id="R_R690" name="thermocryptoxanthin hydroxylase " reversible="false">
<notes>
<body xmlns="http://www.w3.org/1999/xhtml">
<p>GENE_ASSOCIATION: TT_P0059</p>
<p>SUBSYSTEM: Carotenoid biosynthesis</p>
<p>EC Number: </p>
<p>Confidence Level: 1</p>
<p>AUTHORS: Carotenoid metabolism</p>
<p/>
```

```
    </body>
  </notes>
  <listOfReactants>
    <speciesReference species="M_H_c" stoichiometry="1"/>
    <speciesReference species="M_NADH_c" stoichiometry="1"/>
    <speciesReference species="M_O2_c" stoichiometry="1"/>
    <speciesReference species="M_tCRTX_c" stoichiometry="1"/>
  </listOfReactants>
  <listOfProducts>
    <speciesReference species="M_tZXT_c" stoichiometry="1"/>
    <speciesReference species="M_H2O_c" stoichiometry="1"/>
    <speciesReference species="M_NAD_c" stoichiometry="1"/>
  </listOfProducts>
  <kineticLaw>
    <math xmlns="http://www.w3.org/1998/Math/MathML">
      <ci> FLUX_VALUE </ci>
    </math>
    <listOfParameters>
      <parameter id="LOWER_BOUND" value="0" units="mmol_per_gDW_per_hr" constant="true"/>
      <parameter id="UPPER_BOUND" value="1000" units="mmol_per_gDW_per_hr" constant="true"/>
      <parameter id="FLUX_VALUE" value="0" units="mmol_per_gDW_per_hr" constant="true"/>
      <parameter id="OBJECTIVE_COEFFICIENT" value="0" units="mmol_per_gDW_per_hr" constant="true"/>
    </listOfParameters>
  </kineticLaw>
</reaction>
<reaction id="R_R691" name="beta-cryptoxanthin glucosyltransferase" reversible="false">
  <notes>
    <body xmlns="http://www.w3.org/1999/xhtml">
      <p>GENE_ASSOCIATION: TT_P0062</p>
```

```
      <p>SUBSYSTEM: Carotenoid biosynthesis</p>
      <p>EC Number: </p>
      <p>Confidence Level: 1</p>
      <p>AUTHORS: Carotenoid metabolism</p>
      <p/>
    </body>
  </notes>
  <listOfReactants>
    <speciesReference species="M_UDP_GLU_c" stoichiometry="1"/>
    <speciesReference species="M_CRTX_c" stoichiometry="1"/>
  </listOfReactants>
  <listOfProducts>
    <speciesReference species="M_H_c" stoichiometry="1"/>
    <speciesReference species="M_UDP_c" stoichiometry="1"/>
    <speciesReference species="M_bCRTX_GLU_c" stoichiometry="1"/>
  </listOfProducts>
  <kineticLaw>
    <math xmlns="http://www.w3.org/1998/Math/MathML">
      <ci> FLUX_VALUE </ci>
    </math>
    <listOfParameters>
      <parameter id="LOWER_BOUND" value="0" units="mmol_per_gDW_per_hr" constant="true"/>
      <parameter id="UPPER_BOUND" value="1000" units="mmol_per_gDW_per_hr" constant="true"/>
      <parameter id="FLUX_VALUE" value="0" units="mmol_per_gDW_per_hr" constant="true"/>
      <parameter id="OBJECTIVE_COEFFICIENT" value="0" units="mmol_per_gDW_per_hr" constant="true"/>
    </listOfParameters>
  </kineticLaw>
</reaction>
<reaction id="R_R692" name="beta-cryptoxanthin acyltransferase (C11:0)" reversible="false">
```

```
<notes>
  <body xmlns="http://www.w3.org/1999/xhtml">
    <p>GENE_ASSOCIATION: TT_P0061</p>
    <p>SUBSYSTEM: Carotenoid biosynthesis</p>
    <p>EC Number: </p>
    <p>Confidence Level: 1</p>
    <p>AUTHORS: Carotenoid metabolism</p>
    <p/>
  </body>
</notes>
<listOfReactants>
  <speciesReference species="M_I150CoA_c" stoichiometry="0.334"/>
  <speciesReference species="M_AI150CoA_c" stoichiometry="0.069"/>
  <speciesReference species="M_I160CoA_c" stoichiometry="0.047"/>
  <speciesReference species="M_C160CoA_c" stoichiometry="0.014"/>
  <speciesReference species="M_C161CoA_c" stoichiometry="0.0015"/>
  <speciesReference species="M_I170CoA_c" stoichiometry="0.45"/>
  <speciesReference species="M_AI171CoA_c" stoichiometry="0.075"/>
  <speciesReference species="M_bCRTX_GLU_c" stoichiometry="1"/>
</listOfReactants>
<listOfProducts>
  <speciesReference species="M_CoA_c" stoichiometry="1"/>
  <speciesReference species="M_tCRTX_c" stoichiometry="1"/>
</listOfProducts>
<kineticLaw>
  <math xmlns="http://www.w3.org/1998/Math/MathML">
    <ci> FLUX_VALUE </ci>
  </math>
  <listOfParameters>
```

```xml
      <parameter id="LOWER_BOUND" value="0" units="mmol_per_gDW_per_hr" constant="true"/>
      <parameter id="UPPER_BOUND" value="1000" units="mmol_per_gDW_per_hr" constant="true"/>
      <parameter id="FLUX_VALUE" value="0" units="mmol_per_gDW_per_hr" constant="true"/>
      <parameter id="OBJECTIVE_COEFFICIENT" value="0" units="mmol_per_gDW_per_hr" constant="true"/>
    </listOfParameters>
  </kineticLaw>
</reaction>
<reaction id="R_R693" name="zeaxanthin glucosyltransferase" reversible="false">
  <notes>
    <body xmlns="http://www.w3.org/1999/xhtml">
      <p>GENE_ASSOCIATION: TT_P0062</p>
      <p>SUBSYSTEM: Carotenoid biosynthesis</p>
      <p>EC Number: </p>
      <p>Confidence Level: 1</p>
      <p>AUTHORS: Carotenoid metabolism</p>
      <p/>
    </body>
  </notes>
  <listOfReactants>
    <speciesReference species="M_UDP_GLU_c" stoichiometry="1"/>
    <speciesReference species="M_ZXT_c" stoichiometry="1"/>
  </listOfReactants>
  <listOfProducts>
    <speciesReference species="M_H_c" stoichiometry="1"/>
    <speciesReference species="M_UDP_c" stoichiometry="1"/>
    <speciesReference species="M_ZXT_GLU_c" stoichiometry="1"/>
  </listOfProducts>
  <kineticLaw>
    <math xmlns="http://www.w3.org/1998/Math/MathML">
```

```
      <ci> FLUX_VALUE </ci>
    </math>
    <listOfParameters>
      <parameter id="LOWER_BOUND" value="0" units="mmol_per_gDW_per_hr" constant="true"/>
      <parameter id="UPPER_BOUND" value="1000" units="mmol_per_gDW_per_hr" constant="true"/>
      <parameter id="FLUX_VALUE" value="0" units="mmol_per_gDW_per_hr" constant="true"/>
      <parameter id="OBJECTIVE_COEFFICIENT" value="0" units="mmol_per_gDW_per_hr" constant="true"/>
    </listOfParameters>
  </kineticLaw>
</reaction>
<reaction id="R_R694" name="zeaxanthin acyltransferase (C13:0)" reversible="false">
  <notes>
    <body xmlns="http://www.w3.org/1999/xhtml">
      <p>GENE_ASSOCIATION: TT_P0061</p>
      <p>SUBSYSTEM: Carotenoid biosynthesis</p>
      <p>EC Number: </p>
      <p>Confidence Level: 1</p>
      <p>AUTHORS: Carotenoid metabolism</p>
      <p/>
    </body>
  </notes>
  <listOfReactants>
    <speciesReference species="M_I150CoA_c" stoichiometry="0.334"/>
    <speciesReference species="M_AI150CoA_c" stoichiometry="0.069"/>
    <speciesReference species="M_I160CoA_c" stoichiometry="0.047"/>
    <speciesReference species="M_C160CoA_c" stoichiometry="0.014"/>
    <speciesReference species="M_C161CoA_c" stoichiometry="0.0015"/>
    <speciesReference species="M_I170CoA_c" stoichiometry="0.45"/>
    <speciesReference species="M_AI171CoA_c" stoichiometry="0.075"/>
```

```xml
<speciesReference species="M_ZXT_GLU_c" stoichiometry="1"/>
</listOfReactants>
<listOfProducts>
<speciesReference species="M_tZXT_c" stoichiometry="1"/>
<speciesReference species="M_CoA_c" stoichiometry="1"/>
</listOfProducts>
<kineticLaw>
<math xmlns="http://www.w3.org/1998/Math/MathML">
<ci> FLUX_VALUE </ci>
</math>
<listOfParameters>
<parameter id="LOWER_BOUND" value="0" units="mmol_per_gDW_per_hr" constant="true"/>
<parameter id="UPPER_BOUND" value="1000" units="mmol_per_gDW_per_hr" constant="true"/>
<parameter id="FLUX_VALUE" value="0" units="mmol_per_gDW_per_hr" constant="true"/>
<parameter id="OBJECTIVE_COEFFICIENT" value="0" units="mmol_per_gDW_per_hr" constant="true"/>
</listOfParameters>
</kineticLaw>
</reaction>
<reaction id="R_R695" name="thermozeaxanthin glucosyltransferase" reversible="false">
<notes>
<body xmlns="http://www.w3.org/1999/xhtml">
<p>GENE_ASSOCIATION: TT_P0062</p>
<p>SUBSYSTEM: Carotenoid biosynthesis</p>
<p>EC Number: </p>
<p>Confidence Level: 1</p>
<p>AUTHORS: Carotenoid metabolism</p>
<p/>
</body>
</notes>
```

```
<listOfReactants>
  <speciesReference species="M_tZXT_c" stoichiometry="1"/>
  <speciesReference species="M_UDP_GLU_c" stoichiometry="1"/>
</listOfReactants>
<listOfProducts>
  <speciesReference species="M_H_c" stoichiometry="1"/>
  <speciesReference species="M_UDP_c" stoichiometry="1"/>
  <speciesReference species="M_tZXT_GLU_c" stoichiometry="1"/>
</listOfProducts>
<kineticLaw>
  <math xmlns="http://www.w3.org/1998/Math/MathML">
    <ci> FLUX_VALUE </ci>
  </math>
  <listOfParameters>
    <parameter id="LOWER_BOUND" value="0" units="mmol_per_gDW_per_hr" constant="true"/>
    <parameter id="UPPER_BOUND" value="1000" units="mmol_per_gDW_per_hr" constant="true"/>
    <parameter id="FLUX_VALUE" value="0" units="mmol_per_gDW_per_hr" constant="true"/>
    <parameter id="OBJECTIVE_COEFFICIENT" value="0" units="mmol_per_gDW_per_hr" constant="true"/>
  </listOfParameters>
</kineticLaw>
</reaction>
<reaction id="R_R696" name="thermozeaxanthin acyltransferase (C13-C13)" reversible="false">
<notes>
  <body xmlns="http://www.w3.org/1999/xhtml">
    <p>GENE_ASSOCIATION: TT_P0061</p>
    <p>SUBSYSTEM: Carotenoid biosynthesis</p>
    <p>EC Number: </p>
    <p>Confidence Level: 1</p>
    <p>AUTHORS: Carotenoid metabolism</p>
```

```
      <p/>
    </body>
  </notes>
  <listOfReactants>
    <speciesReference species="M_I150CoA_c" stoichiometry="0.334"/>
    <speciesReference species="M_AI150CoA_c" stoichiometry="0.069"/>
    <speciesReference species="M_I160CoA_c" stoichiometry="0.047"/>
    <speciesReference species="M_C160CoA_c" stoichiometry="0.014"/>
    <speciesReference species="M_C161CoA_c" stoichiometry="0.0015"/>
    <speciesReference species="M_I170CoA_c" stoichiometry="0.45"/>
    <speciesReference species="M_AI171CoA_c" stoichiometry="0.075"/>
    <speciesReference species="M_tZXT_GLU_c" stoichiometry="1"/>
  </listOfReactants>
  <listOfProducts>
    <speciesReference species="M_CoA_c" stoichiometry="1"/>
    <speciesReference species="M_tbZXT_c" stoichiometry="1"/>
  </listOfProducts>
  <kineticLaw>
    <math xmlns="http://www.w3.org/1998/Math/MathML">
      <ci> FLUX_VALUE </ci>
    </math>
    <listOfParameters>
      <parameter id="LOWER_BOUND" value="0" units="mmol_per_gDW_per_hr" constant="true"/>
      <parameter id="UPPER_BOUND" value="1000" units="mmol_per_gDW_per_hr" constant="true"/>
      <parameter id="FLUX_VALUE" value="0" units="mmol_per_gDW_per_hr" constant="true"/>
      <parameter id="OBJECTIVE_COEFFICIENT" value="0" units="mmol_per_gDW_per_hr" constant="true"/>
    </listOfParameters>
  </kineticLaw>
</reaction>
```

```
<reaction id="R_R697" name="glucosamine-6-phosphate deaminase" reversible="false">
 <notes>
  <body xmlns="http://www.w3.org/1999/xhtml">
   <p>GENE_ASSOCIATION: TTC1074</p>
   <p>SUBSYSTEM: </p>
   <p>EC Number: 3.5.99.6</p>
   <p>Confidence Level: 1</p>
   <p>AUTHORS: Carbohydrate metabolism</p>
   <p/>
  </body>
 </notes>
 <listOfReactants>
  <speciesReference species="M_H2O_c" stoichiometry="1"/>
  <speciesReference species="M_GLUSAP_c" stoichiometry="1"/>
 </listOfReactants>
 <listOfProducts>
  <speciesReference species="M_F6P_c" stoichiometry="1"/>
  <speciesReference species="M_NH4_c" stoichiometry="1"/>
 </listOfProducts>
 <kineticLaw>
  <math xmlns="http://www.w3.org/1998/Math/MathML">
   <ci> FLUX_VALUE </ci>
  </math>
  <listOfParameters>
   <parameter id="LOWER_BOUND" value="0" units="mmol_per_gDW_per_hr" constant="true"/>
   <parameter id="UPPER_BOUND" value="1000" units="mmol_per_gDW_per_hr" constant="true"/>
   <parameter id="FLUX_VALUE" value="0" units="mmol_per_gDW_per_hr" constant="true"/>
   <parameter id="OBJECTIVE_COEFFICIENT" value="0" units="mmol_per_gDW_per_hr" constant="true"/>
  </listOfParameters>
```

```xml
</kineticLaw>
</reaction>
<reaction id="R_R698" name="acetyl-CoA C-acetyltransferase" reversible="false">
<notes>
<body xmlns="http://www.w3.org/1999/xhtml">
<p>GENE_ASSOCIATION: (TTC0191 or TTC0330)</p>
<p>SUBSYSTEM: Butanoate metabolism</p>
<p>EC Number: 2.3.1.9</p>
<p>Confidence Level: 1</p>
<p>AUTHORS: Lipid metabolism</p>
<p/>
</body>
</notes>
<listOfReactants>
<speciesReference species="M_ACoA_c" stoichiometry="2"/>
</listOfReactants>
<listOfProducts>
<speciesReference species="M_CoA_c" stoichiometry="1"/>
<speciesReference species="M_ACACoA_c" stoichiometry="1"/>
</listOfProducts>
<kineticLaw>
<math xmlns="http://www.w3.org/1998/Math/MathML">
<ci> FLUX_VALUE </ci>
</math>
<listOfParameters>
<parameter id="LOWER_BOUND" value="0" units="mmol_per_gDW_per_hr" constant="true"/>
<parameter id="UPPER_BOUND" value="1000" units="mmol_per_gDW_per_hr" constant="true"/>
<parameter id="FLUX_VALUE" value="0" units="mmol_per_gDW_per_hr" constant="true"/>
<parameter id="OBJECTIVE_COEFFICIENT" value="0" units="mmol_per_gDW_per_hr" constant="true"/>
```

```
    </listOfParameters>
  </kineticLaw>
</reaction>
<reaction id="R_R699" name="(R)-3-Hydroxybutanoyl-CoA:NADP+ oxidoreductase" reversible="true">
  <notes>
    <body xmlns="http://www.w3.org/1999/xhtml">
      <p>GENE_ASSOCIATION: TTC0898</p>
      <p>SUBSYSTEM: Butanoate metabolism</p>
      <p>EC Number: 1.1.1.157</p>
      <p>Confidence Level: 1</p>
      <p>AUTHORS: Lipid metabolism</p>
      <p/>
    </body>
  </notes>
  <listOfReactants>
    <speciesReference species="M_H_c" stoichiometry="1"/>
    <speciesReference species="M_NADPH_c" stoichiometry="1"/>
    <speciesReference species="M_ACACoA_c" stoichiometry="1"/>
  </listOfReactants>
  <listOfProducts>
    <speciesReference species="M_NADP_c" stoichiometry="1"/>
    <speciesReference species="M_RHY_BUTCoA_c" stoichiometry="1"/>
  </listOfProducts>
  <kineticLaw>
    <math xmlns="http://www.w3.org/1998/Math/MathML">
      <ci> FLUX_VALUE </ci>
    </math>
    <listOfParameters>
      <parameter id="LOWER_BOUND" value="-1000" units="mmol_per_gDW_per_hr" constant="true"/>
```

```
      <parameter id="UPPER_BOUND" value="1000" units="mmol_per_gDW_per_hr" constant="true"/>
      <parameter id="FLUX_VALUE" value="0" units="mmol_per_gDW_per_hr" constant="true"/>
      <parameter id="OBJECTIVE_COEFFICIENT" value="0" units="mmol_per_gDW_per_hr" constant="true"/>
    </listOfParameters>
  </kineticLaw>
</reaction>
<reaction id="R_R700" name="beta-ketothiolase " reversible="false">
  <notes>
    <body xmlns="http://www.w3.org/1999/xhtml">
      <p>GENE_ASSOCIATION: (TTC0191 or TTC0330 or TTC0525 or TTC0623)</p>
      <p>SUBSYSTEM: Butanoate metabolism</p>
      <p>EC Number: </p>
      <p>Confidence Level: 1</p>
      <p>AUTHORS: Lipid metabolism</p>
      <p/>
    </body>
  </notes>
  <listOfReactants>
    <speciesReference species="M_ACoA_c" stoichiometry="1"/>
    <speciesReference species="M_PRCoA_c" stoichiometry="1"/>
  </listOfReactants>
  <listOfProducts>
    <speciesReference species="M_CoA_c" stoichiometry="1"/>
    <speciesReference species="M_KVCoA_c" stoichiometry="1"/>
  </listOfProducts>
  <kineticLaw>
    <math xmlns="http://www.w3.org/1998/Math/MathML">
      <ci> FLUX_VALUE </ci>
    </math>
```

```xml
<listOfParameters>
<parameter id="LOWER_BOUND" value="0" units="mmol_per_gDW_per_hr" constant="true"/>
<parameter id="UPPER_BOUND" value="1000" units="mmol_per_gDW_per_hr" constant="true"/>
<parameter id="FLUX_VALUE" value="0" units="mmol_per_gDW_per_hr" constant="true"/>
<parameter id="OBJECTIVE_COEFFICIENT" value="0" units="mmol_per_gDW_per_hr" constant="true"/>
</listOfParameters>
</kineticLaw>
</reaction>
<reaction id="R_R701" name="(R)-3-Hydroxybutanoyl-CoA:NADP+ oxidoreductase" reversible="true">
<notes>
<body xmlns="http://www.w3.org/1999/xhtml">
<p>GENE_ASSOCIATION: TTC0898</p>
<p>SUBSYSTEM: Butanoate metabolism</p>
<p>EC Number: </p>
<p>Confidence Level: 1</p>
<p>AUTHORS: Lipid metabolism</p>
<p/>
</body>
</notes>
<listOfReactants>
<speciesReference species="M_H_c" stoichiometry="3"/>
<speciesReference species="M_NADPH_c" stoichiometry="1"/>
<speciesReference species="M_KVCoA_c" stoichiometry="1"/>
</listOfReactants>
<listOfProducts>
<speciesReference species="M_NADP_c" stoichiometry="1"/>
<speciesReference species="M_HVCoA_c" stoichiometry="1"/>
</listOfProducts>
<kineticLaw>
```

```
      <math xmlns="http://www.w3.org/1998/Math/MathML">
        <ci> FLUX_VALUE </ci>
      </math>
      <listOfParameters>
        <parameter id="LOWER_BOUND" value="-1000" units="mmol_per_gDW_per_hr" constant="true"/>
        <parameter id="UPPER_BOUND" value="1000" units="mmol_per_gDW_per_hr" constant="true"/>
        <parameter id="FLUX_VALUE" value="0" units="mmol_per_gDW_per_hr" constant="true"/>
        <parameter id="OBJECTIVE_COEFFICIENT" value="0" units="mmol_per_gDW_per_hr" constant="true"/>
      </listOfParameters>
    </kineticLaw>
  </reaction>
  <reaction id="R_R702" name="Acyltransferase" reversible="false">
    <notes>
      <body xmlns="http://www.w3.org/1999/xhtml">
        <p>GENE_ASSOCIATION: </p>
        <p>SUBSYSTEM: Butanoate metabolism</p>
        <p>EC Number: 2.3.1.-</p>
        <p>Confidence Level: 1</p>
        <p>AUTHORS: Lipid metabolism</p>
        <p/>
      </body>
    </notes>
    <listOfReactants>
      <speciesReference species="M_RHY_BUTCoA_c" stoichiometry="2"/>
    </listOfReactants>
    <listOfProducts>
      <speciesReference species="M_CoA_c" stoichiometry="2"/>
      <speciesReference species="M_PHB_c" stoichiometry="1"/>
    </listOfProducts>
```

```
  <kineticLaw>
    <math xmlns="http://www.w3.org/1998/Math/MathML">
      <ci> FLUX_VALUE </ci>
    </math>
    <listOfParameters>
      <parameter id="LOWER_BOUND" value="0" units="mmol_per_gDW_per_hr" constant="true"/>
      <parameter id="UPPER_BOUND" value="1000" units="mmol_per_gDW_per_hr" constant="true"/>
      <parameter id="FLUX_VALUE" value="0" units="mmol_per_gDW_per_hr" constant="true"/>
      <parameter id="OBJECTIVE_COEFFICIENT" value="0" units="mmol_per_gDW_per_hr" constant="true"/>
    </listOfParameters>
  </kineticLaw>
</reaction>
<reaction id="R_R703" name="Acyltransferase" reversible="false">
  <notes>
    <body xmlns="http://www.w3.org/1999/xhtml">
      <p>GENE_ASSOCIATION: </p>
      <p>SUBSYSTEM: Butanoate metabolism</p>
      <p>EC Number: </p>
      <p>Confidence Level: 1</p>
      <p>AUTHORS: Lipid metabolism</p>
      <p/>
    </body>
  </notes>
  <listOfReactants>
    <speciesReference species="M_HVCoA_c" stoichiometry="2"/>
  </listOfReactants>
  <listOfProducts>
    <speciesReference species="M_CoA_c" stoichiometry="1"/>
    <speciesReference species="M_PHV_c" stoichiometry="1"/>
```

```
        </listOfProducts>
        <kineticLaw>
          <math xmlns="http://www.w3.org/1998/Math/MathML">
            <ci> FLUX_VALUE </ci>
          </math>
          <listOfParameters>
            <parameter id="LOWER_BOUND" value="0" units="mmol_per_gDW_per_hr" constant="true"/>
            <parameter id="UPPER_BOUND" value="1000" units="mmol_per_gDW_per_hr" constant="true"/>
            <parameter id="FLUX_VALUE" value="0" units="mmol_per_gDW_per_hr" constant="true"/>
            <parameter id="OBJECTIVE_COEFFICIENT" value="0" units="mmol_per_gDW_per_hr" constant="true"/>
          </listOfParameters>
        </kineticLaw>
      </reaction>
      <reaction id="R_R704" name="fatty acid synthesis; lumped" reversible="true">
        <notes>
          <body xmlns="http://www.w3.org/1999/xhtml">
            <p>GENE_ASSOCIATION: ((TTC0049 or TTC0045) and (TTC0045 or (TTC0049 or TTC0343)) and (TTC0047 or TTC0394) and TTC1463 and TTC0048 and TTC1678)</p>
            <p>SUBSYSTEM: Butanoate metabolism</p>
            <p>EC Number: </p>
            <p>Confidence Level: 1</p>
            <p>AUTHORS: Lipid metabolism</p>
            <p/>
          </body>
        </notes>
        <listOfReactants>
          <speciesReference species="M_H_c" stoichiometry="4"/>
          <speciesReference species="M_ACoA_c" stoichiometry="2"/>
          <speciesReference species="M_NADPH_c" stoichiometry="4"/>
```

```
    <speciesReference species="M_RHY_BUTCoA_c" stoichiometry="1"/>
   </listOfReactants>
   <listOfProducts>
    <speciesReference species="M_H2O_c" stoichiometry="2"/>
    <speciesReference species="M_CoA_c" stoichiometry="2"/>
    <speciesReference species="M_NADP_c" stoichiometry="4"/>
    <speciesReference species="M_HY_OCTCoA_c" stoichiometry="1"/>
   </listOfProducts>
   <kineticLaw>
    <math xmlns="http://www.w3.org/1998/Math/MathML">
     <ci> FLUX_VALUE </ci>
    </math>
    <listOfParameters>
     <parameter id="LOWER_BOUND" value="-1000" units="mmol_per_gDW_per_hr" constant="true"/>
     <parameter id="UPPER_BOUND" value="1000" units="mmol_per_gDW_per_hr" constant="true"/>
     <parameter id="FLUX_VALUE" value="0" units="mmol_per_gDW_per_hr" constant="true"/>
     <parameter id="OBJECTIVE_COEFFICIENT" value="0" units="mmol_per_gDW_per_hr" constant="true"/>
    </listOfParameters>
   </kineticLaw>
  </reaction>
  <reaction id="R_R705" name="Acyltransferase" reversible="false">
   <notes>
    <body xmlns="http://www.w3.org/1999/xhtml">
     <p>GENE_ASSOCIATION: </p>
     <p>SUBSYSTEM: Butanoate metabolism</p>
     <p>EC Number: </p>
     <p>Confidence Level: 1</p>
     <p>AUTHORS: Lipid metabolism</p>
     <p/>
```

```
        </body>
      </notes>
      <listOfReactants>
        <speciesReference species="M_HY_OCTCoA_c" stoichiometry="2"/>
      </listOfReactants>
      <listOfProducts>
        <speciesReference species="M_CoA_c" stoichiometry="2"/>
        <speciesReference species="M_PHO_c" stoichiometry="1"/>
      </listOfProducts>
      <kineticLaw>
        <math xmlns="http://www.w3.org/1998/Math/MathML">
          <ci> FLUX_VALUE </ci>
        </math>
        <listOfParameters>
          <parameter id="LOWER_BOUND" value="0" units="mmol_per_gDW_per_hr" constant="true"/>
          <parameter id="UPPER_BOUND" value="1000" units="mmol_per_gDW_per_hr" constant="true"/>
          <parameter id="FLUX_VALUE" value="0" units="mmol_per_gDW_per_hr" constant="true"/>
          <parameter id="OBJECTIVE_COEFFICIENT" value="0" units="mmol_per_gDW_per_hr" constant="true"/>
        </listOfParameters>
      </kineticLaw>
    </reaction>
    <reaction id="R_R706" name="fatty acid synthesis; lumped" reversible="true">
      <notes>
        <body xmlns="http://www.w3.org/1999/xhtml">
          <p>GENE_ASSOCIATION: ((TTC0049 or TTC0045) and (TTC0045 or (TTC0049 or TTC0343)) and (TTC0047 or TTC0394) and
TTC1463 and TTC0048 and TTC1678)</p>
          <p>SUBSYSTEM: Butanoate metabolism</p>
          <p>EC Number: </p>
          <p>Confidence Level: 1</p>
```

```xml
<p>AUTHORS: Lipid metabolism</p>

<p/>

</body>

</notes>

<listOfReactants>

<speciesReference species="M_H_c" stoichiometry="2"/>

<speciesReference species="M_ACoA_c" stoichiometry="1"/>

<speciesReference species="M_NADPH_c" stoichiometry="2"/>

<speciesReference species="M_HY_OCTCoA_c" stoichiometry="1"/>

</listOfReactants>

<listOfProducts>

<speciesReference species="M_H2O_c" stoichiometry="1"/>

<speciesReference species="M_CoA_c" stoichiometry="1"/>

<speciesReference species="M_NADP_c" stoichiometry="2"/>

<speciesReference species="M_HY_DECCoA_c" stoichiometry="1"/>

</listOfProducts>

<kineticLaw>

<math xmlns="http://www.w3.org/1998/Math/MathML">

<ci> FLUX_VALUE </ci>

</math>

<listOfParameters>

<parameter id="LOWER_BOUND" value="-1000" units="mmol_per_gDW_per_hr" constant="true"/>

<parameter id="UPPER_BOUND" value="1000" units="mmol_per_gDW_per_hr" constant="true"/>

<parameter id="FLUX_VALUE" value="0" units="mmol_per_gDW_per_hr" constant="true"/>

<parameter id="OBJECTIVE_COEFFICIENT" value="0" units="mmol_per_gDW_per_hr" constant="true"/>

</listOfParameters>

</kineticLaw>

</reaction>

<reaction id="R_R707" name="Acyltransferase" reversible="false">
```

```
  <notes>
    <body xmlns="http://www.w3.org/1999/xhtml">
      <p>GENE_ASSOCIATION: </p>
      <p>SUBSYSTEM: Butanoate metabolism</p>
      <p>EC Number: </p>
      <p>Confidence Level: 1</p>
      <p>AUTHORS: Lipid metabolism</p>
      <p/>
    </body>
  </notes>
  <listOfReactants>
    <speciesReference species="M_HY_DECCoA_c" stoichiometry="2"/>
  </listOfReactants>
  <listOfProducts>
    <speciesReference species="M_CoA_c" stoichiometry="2"/>
    <speciesReference species="M_PHD_c" stoichiometry="1"/>
  </listOfProducts>
  <kineticLaw>
    <math xmlns="http://www.w3.org/1998/Math/MathML">
      <ci> FLUX_VALUE </ci>
    </math>
    <listOfParameters>
      <parameter id="LOWER_BOUND" value="0" units="mmol_per_gDW_per_hr" constant="true"/>
      <parameter id="UPPER_BOUND" value="1000" units="mmol_per_gDW_per_hr" constant="true"/>
      <parameter id="FLUX_VALUE" value="0" units="mmol_per_gDW_per_hr" constant="true"/>
      <parameter id="OBJECTIVE_COEFFICIENT" value="0" units="mmol_per_gDW_per_hr" constant="true"/>
    </listOfParameters>
  </kineticLaw>
</reaction>
```

<reaction id="R_R708" name="fatty acid synthesis; lumped" reversible="true">

<notes>

<body xmlns="http://www.w3.org/1999/xhtml">

<p>GENE_ASSOCIATION: ((TTC0049 or TTC0045) and (TTC0045 or (TTC0049 or TTC0343)) and (TTC0047 or TTC0394) and TTC1463 and TTC0048 and TTC1678)</p>

<p>SUBSYSTEM: Butanoate metabolism</p>

<p>EC Number: </p>

<p>Confidence Level: 1</p>

<p>AUTHORS: Lipid metabolism</p>

<p/>

</body>

</notes>

<listOfReactants>

<speciesReference species="M_H_c" stoichiometry="2"/>

<speciesReference species="M_ACoA_c" stoichiometry="1"/>

<speciesReference species="M_NADPH_c" stoichiometry="2"/>

<speciesReference species="M_HY_DECCoA_c" stoichiometry="1"/>

</listOfReactants>

<listOfProducts>

<speciesReference species="M_H2O_c" stoichiometry="1"/>

<speciesReference species="M_CoA_c" stoichiometry="1"/>

<speciesReference species="M_NADP_c" stoichiometry="2"/>

<speciesReference species="M_HY_DODECCoA_c" stoichiometry="1"/>

</listOfProducts>

<kineticLaw>

<math xmlns="http://www.w3.org/1998/Math/MathML">

<ci> FLUX_VALUE </ci>

</math>

<listOfParameters>

```
      <parameter id="LOWER_BOUND" value="-1000" units="mmol_per_gDW_per_hr" constant="true"/>
      <parameter id="UPPER_BOUND" value="1000" units="mmol_per_gDW_per_hr" constant="true"/>
      <parameter id="FLUX_VALUE" value="0" units="mmol_per_gDW_per_hr" constant="true"/>
      <parameter id="OBJECTIVE_COEFFICIENT" value="0" units="mmol_per_gDW_per_hr" constant="true"/>
    </listOfParameters>
  </kineticLaw>
</reaction>
<reaction id="R_R709" name="Acyltransferase" reversible="false">
  <notes>
    <body xmlns="http://www.w3.org/1999/xhtml">
      <p>GENE_ASSOCIATION: </p>
      <p>SUBSYSTEM: Butanoate metabolism</p>
      <p>EC Number: </p>
      <p>Confidence Level: 1</p>
      <p>AUTHORS: Lipid metabolism</p>
      <p/>
    </body>
  </notes>
  <listOfReactants>
    <speciesReference species="M_HY_DODECCoA_c" stoichiometry="2"/>
  </listOfReactants>
  <listOfProducts>
    <speciesReference species="M_CoA_c" stoichiometry="2"/>
    <speciesReference species="M_PHDD_c" stoichiometry="1"/>
  </listOfProducts>
  <kineticLaw>
    <math xmlns="http://www.w3.org/1998/Math/MathML">
      <ci> FLUX_VALUE </ci>
    </math>
```

```xml
    <listOfParameters>
      <parameter id="LOWER_BOUND" value="0" units="mmol_per_gDW_per_hr" constant="true"/>
      <parameter id="UPPER_BOUND" value="1000" units="mmol_per_gDW_per_hr" constant="true"/>
      <parameter id="FLUX_VALUE" value="0" units="mmol_per_gDW_per_hr" constant="true"/>
      <parameter id="OBJECTIVE_COEFFICIENT" value="0" units="mmol_per_gDW_per_hr" constant="true"/>
    </listOfParameters>
  </kineticLaw>
</reaction>
<reaction id="R_R710" name="gluconokinase" reversible="false">
  <notes>
    <body xmlns="http://www.w3.org/1999/xhtml">
      <p>GENE_ASSOCIATION: </p>
      <p>SUBSYSTEM: Pentose Phosphate Pathway</p>
      <p>EC Number: 2.7.1.12</p>
      <p>Confidence Level: 1</p>
      <p>AUTHORS: Carbohydrate metabolism</p>
      <p/>
    </body>
  </notes>
  <listOfReactants>
    <speciesReference species="M_ATP_c" stoichiometry="1"/>
    <speciesReference species="M_GLCN_c" stoichiometry="1"/>
  </listOfReactants>
  <listOfProducts>
    <speciesReference species="M_ADP_c" stoichiometry="1"/>
    <speciesReference species="M_H_c" stoichiometry="1"/>
    <speciesReference species="M_GLCN6P_c" stoichiometry="1"/>
  </listOfProducts>
  <kineticLaw>
```

```
<math xmlns="http://www.w3.org/1998/Math/MathML">
  <ci> FLUX_VALUE </ci>
</math>
<listOfParameters>
  <parameter id="LOWER_BOUND" value="0" units="mmol_per_gDW_per_hr" constant="true"/>
  <parameter id="UPPER_BOUND" value="1000" units="mmol_per_gDW_per_hr" constant="true"/>
  <parameter id="FLUX_VALUE" value="0" units="mmol_per_gDW_per_hr" constant="true"/>
  <parameter id="OBJECTIVE_COEFFICIENT" value="0" units="mmol_per_gDW_per_hr" constant="true"/>
</listOfParameters>
</kineticLaw>
</reaction>
<reaction id="R_R711" name="gluconate 5-dehydrogenase (NAD+)" reversible="false">
 <notes>
  <body xmlns="http://www.w3.org/1999/xhtml">
   <p>GENE_ASSOCIATION: TTC0235</p>
   <p>SUBSYSTEM: </p>
   <p>EC Number: 1.1.1.69</p>
   <p>Confidence Level: 1</p>
   <p>AUTHORS: Carbohydrate metabolism</p>
   <p/>
  </body>
 </notes>
 <listOfReactants>
  <speciesReference species="M_H_c" stoichiometry="1"/>
  <speciesReference species="M_NADH_c" stoichiometry="1"/>
  <speciesReference species="M_DHYGLCN_c" stoichiometry="1"/>
 </listOfReactants>
 <listOfProducts>
  <speciesReference species="M_NAD_c" stoichiometry="1"/>
```

```
    <speciesReference species="M_GLCN_c" stoichiometry="1"/>
  </listOfProducts>
  <kineticLaw>
    <math xmlns="http://www.w3.org/1998/Math/MathML">
      <ci> FLUX_VALUE </ci>
    </math>
    <listOfParameters>
      <parameter id="LOWER_BOUND" value="0" units="mmol_per_gDW_per_hr" constant="true"/>
      <parameter id="UPPER_BOUND" value="1000" units="mmol_per_gDW_per_hr" constant="true"/>
      <parameter id="FLUX_VALUE" value="0" units="mmol_per_gDW_per_hr" constant="true"/>
      <parameter id="OBJECTIVE_COEFFICIENT" value="0" units="mmol_per_gDW_per_hr" constant="true"/>
    </listOfParameters>
  </kineticLaw>
</reaction>
<reaction id="R_R712" name="gluconate 5-dehydrogenase (NADP+)" reversible="false">
  <notes>
    <body xmlns="http://www.w3.org/1999/xhtml">
      <p>GENE_ASSOCIATION: TTC0235</p>
      <p>SUBSYSTEM: </p>
      <p>EC Number: 1.1.1.69</p>
      <p>Confidence Level: 1</p>
      <p>AUTHORS: Carbohydrate metabolism</p>
      <p/>
    </body>
  </notes>
  <listOfReactants>
    <speciesReference species="M_H_c" stoichiometry="1"/>
    <speciesReference species="M_NADPH_c" stoichiometry="1"/>
    <speciesReference species="M_DHYGLCN_c" stoichiometry="1"/>
```

```
  </listOfReactants>
  <listOfProducts>
    <speciesReference species="M_NADP_c" stoichiometry="1"/>
    <speciesReference species="M_GLCN_c" stoichiometry="1"/>
  </listOfProducts>
  <kineticLaw>
    <math xmlns="http://www.w3.org/1998/Math/MathML">
      <ci> FLUX_VALUE </ci>
    </math>
    <listOfParameters>
      <parameter id="LOWER_BOUND" value="0" units="mmol_per_gDW_per_hr" constant="true"/>
      <parameter id="UPPER_BOUND" value="1000" units="mmol_per_gDW_per_hr" constant="true"/>
      <parameter id="FLUX_VALUE" value="0" units="mmol_per_gDW_per_hr" constant="true"/>
      <parameter id="OBJECTIVE_COEFFICIENT" value="0" units="mmol_per_gDW_per_hr" constant="true"/>
    </listOfParameters>
  </kineticLaw>
</reaction>
<reaction id="R_R713" name="gluconolactonase" reversible="false">
  <notes>
    <body xmlns="http://www.w3.org/1999/xhtml">
      <p>GENE_ASSOCIATION: </p>
      <p>SUBSYSTEM: Pentose Phosphate Pathway</p>
      <p>EC Number: 3.1.1.17</p>
      <p>Confidence Level: 1</p>
      <p>AUTHORS: Carbohydrate metabolism</p>
      <p/>
    </body>
  </notes>
  <listOfReactants>
```

```
    <speciesReference species="M_H2O_c" stoichiometry="1"/>
    <speciesReference species="M_GLC_LACN_c" stoichiometry="1"/>
  </listOfReactants>
  <listOfProducts>
    <speciesReference species="M_H_c" stoichiometry="1"/>
    <speciesReference species="M_GLCN_c" stoichiometry="1"/>
  </listOfProducts>
  <kineticLaw>
    <math xmlns="http://www.w3.org/1998/Math/MathML">
      <ci> FLUX_VALUE </ci>
    </math>
    <listOfParameters>
      <parameter id="LOWER_BOUND" value="0" units="mmol_per_gDW_per_hr" constant="true"/>
      <parameter id="UPPER_BOUND" value="1000" units="mmol_per_gDW_per_hr" constant="true"/>
      <parameter id="FLUX_VALUE" value="0" units="mmol_per_gDW_per_hr" constant="true"/>
      <parameter id="OBJECTIVE_COEFFICIENT" value="0" units="mmol_per_gDW_per_hr" constant="true"/>
    </listOfParameters>
  </kineticLaw>
</reaction>
<reaction id="R_R714" name="phosphogluconate dehydratase" reversible="false">
  <notes>
    <body xmlns="http://www.w3.org/1999/xhtml">
      <p>GENE_ASSOCIATION: TTC0871</p>
      <p>SUBSYSTEM: Pentose Phosphate Pathway</p>
      <p>EC Number: 4.2.1.12</p>
      <p>Confidence Level: 1</p>
      <p>AUTHORS: Carbohydrate metabolism</p>
      <p/>
    </body>
```

```
    </notes>
    <listOfReactants>
      <speciesReference species="M_GLCN6P_c" stoichiometry="1"/>
    </listOfReactants>
    <listOfProducts>
      <speciesReference species="M_H2O_c" stoichiometry="1"/>
      <speciesReference species="M_DHY_DOP_GLUCN_c" stoichiometry="1"/>
    </listOfProducts>
    <kineticLaw>
      <math xmlns="http://www.w3.org/1998/Math/MathML">
        <ci> FLUX_VALUE </ci>
      </math>
      <listOfParameters>
        <parameter id="LOWER_BOUND" value="0" units="mmol_per_gDW_per_hr" constant="true"/>
        <parameter id="UPPER_BOUND" value="1000" units="mmol_per_gDW_per_hr" constant="true"/>
        <parameter id="FLUX_VALUE" value="0" units="mmol_per_gDW_per_hr" constant="true"/>
        <parameter id="OBJECTIVE_COEFFICIENT" value="0" units="mmol_per_gDW_per_hr" constant="true"/>
      </listOfParameters>
    </kineticLaw>
  </reaction>
  <reaction id="R_R715" name="NADH:ubiquinone oxidoreductase I" reversible="false">
    <notes>
      <body xmlns="http://www.w3.org/1999/xhtml">
        <p>GENE_ASSOCIATION: </p>
        <p>SUBSYSTEM: Oxidative Phosphorylation</p>
        <p>EC Number: 1.6.5.-</p>
        <p>Confidence Level: 1</p>
        <p>AUTHORS: Energy and cofactors metabolism</p>
        <p/>
```

```
    </body>
  </notes>
  <listOfReactants>
    <speciesReference species="M_H_c" stoichiometry="1"/>
    <speciesReference species="M_NADH_c" stoichiometry="1"/>
    <speciesReference species="M_MQ_c" stoichiometry="1"/>
  </listOfReactants>
  <listOfProducts>
    <speciesReference species="M_NAD_c" stoichiometry="1"/>
    <speciesReference species="M_MQL_c" stoichiometry="1"/>
  </listOfProducts>
  <kineticLaw>
    <math xmlns="http://www.w3.org/1998/Math/MathML">
      <ci> FLUX_VALUE </ci>
    </math>
    <listOfParameters>
      <parameter id="LOWER_BOUND" value="0" units="mmol_per_gDW_per_hr" constant="true"/>
      <parameter id="UPPER_BOUND" value="1000" units="mmol_per_gDW_per_hr" constant="true"/>
      <parameter id="FLUX_VALUE" value="0" units="mmol_per_gDW_per_hr" constant="true"/>
      <parameter id="OBJECTIVE_COEFFICIENT" value="0" units="mmol_per_gDW_per_hr" constant="true"/>
    </listOfParameters>
  </kineticLaw>
</reaction>
<reaction id="R_R716" name="Glucose transport via ABC transporter" reversible="false">
  <notes>
    <body xmlns="http://www.w3.org/1999/xhtml">
      <p>GENE_ASSOCIATION: ((TTC0326 or TTC0327) and TTC0328)</p>
      <p>SUBSYSTEM: Transport, Extracellular</p>
      <p>EC Number: </p>
```

```
      <p>Confidence Level: 1</p>
      <p>AUTHORS: Transport</p>
      <p/>
    </body>
  </notes>
  <listOfReactants>
    <speciesReference species="M_ATP_c" stoichiometry="1"/>
    <speciesReference species="M_H2O_c" stoichiometry="1"/>
    <speciesReference species="M_D_GLC_e" stoichiometry="1"/>
  </listOfReactants>
  <listOfProducts>
    <speciesReference species="M_ADP_c" stoichiometry="1"/>
    <speciesReference species="M_Pi_c" stoichiometry="1"/>
    <speciesReference species="M_H_c" stoichiometry="1"/>
    <speciesReference species="M_D_GLC_c" stoichiometry="1"/>
  </listOfProducts>
  <kineticLaw>
    <math xmlns="http://www.w3.org/1998/Math/MathML">
      <ci> FLUX_VALUE </ci>
    </math>
    <listOfParameters>
      <parameter id="LOWER_BOUND" value="0" units="mmol_per_gDW_per_hr" constant="true"/>
      <parameter id="UPPER_BOUND" value="1000" units="mmol_per_gDW_per_hr" constant="true"/>
      <parameter id="FLUX_VALUE" value="0" units="mmol_per_gDW_per_hr" constant="true"/>
      <parameter id="OBJECTIVE_COEFFICIENT" value="0" units="mmol_per_gDW_per_hr" constant="true"/>
    </listOfParameters>
  </kineticLaw>
</reaction>
<reaction id="R_R717" name="Water transport" reversible="true">
```

```xml
<notes>
  <body xmlns="http://www.w3.org/1999/xhtml">
    <p>GENE_ASSOCIATION: </p>
    <p>SUBSYSTEM: Transport, Extracellular</p>
    <p>EC Number: </p>
    <p>Confidence Level: 1</p>
    <p>AUTHORS: Transport</p>
    <p/>
  </body>
</notes>
<listOfReactants>
  <speciesReference species="M_H2O_e" stoichiometry="1"/>
</listOfReactants>
<listOfProducts>
  <speciesReference species="M_H2O_c" stoichiometry="1"/>
</listOfProducts>
<kineticLaw>
  <math xmlns="http://www.w3.org/1998/Math/MathML">
    <ci> FLUX_VALUE </ci>
  </math>
  <listOfParameters>
    <parameter id="LOWER_BOUND" value="-1000" units="mmol_per_gDW_per_hr" constant="true"/>
    <parameter id="UPPER_BOUND" value="1000" units="mmol_per_gDW_per_hr" constant="true"/>
    <parameter id="FLUX_VALUE" value="0" units="mmol_per_gDW_per_hr" constant="true"/>
    <parameter id="OBJECTIVE_COEFFICIENT" value="0" units="mmol_per_gDW_per_hr" constant="true"/>
  </listOfParameters>
</kineticLaw>
</reaction>
<reaction id="R_R718" name="Oxygen transport" reversible="false">
```

```xml
<notes>
  <body xmlns="http://www.w3.org/1999/xhtml">
    <p>GENE_ASSOCIATION: </p>
    <p>SUBSYSTEM: Transport, Extracellular</p>
    <p>EC Number: </p>
    <p>Confidence Level: 1</p>
    <p>AUTHORS: Transport</p>
    <p/>
  </body>
</notes>
<listOfReactants>
  <speciesReference species="M_O2_e" stoichiometry="1"/>
</listOfReactants>
<listOfProducts>
  <speciesReference species="M_O2_c" stoichiometry="1"/>
</listOfProducts>
<kineticLaw>
  <math xmlns="http://www.w3.org/1998/Math/MathML">
    <ci> FLUX_VALUE </ci>
  </math>
  <listOfParameters>
    <parameter id="LOWER_BOUND" value="0" units="mmol_per_gDW_per_hr" constant="true"/>
    <parameter id="UPPER_BOUND" value="1000" units="mmol_per_gDW_per_hr" constant="true"/>
    <parameter id="FLUX_VALUE" value="0" units="mmol_per_gDW_per_hr" constant="true"/>
    <parameter id="OBJECTIVE_COEFFICIENT" value="0" units="mmol_per_gDW_per_hr" constant="true"/>
  </listOfParameters>
</kineticLaw>
</reaction>
<reaction id="R_R719" name="Sulfate transport" reversible="false">
```

```
  <notes>
    <body xmlns="http://www.w3.org/1999/xhtml">
      <p>GENE_ASSOCIATION: </p>
      <p>SUBSYSTEM: Transport, Extracellular</p>
      <p>EC Number: </p>
      <p>Confidence Level: 1</p>
      <p>AUTHORS: Transport</p>
      <p/>
    </body>
  </notes>
  <listOfReactants>
    <speciesReference species="M_SO4_e" stoichiometry="1"/>
  </listOfReactants>
  <listOfProducts>
    <speciesReference species="M_SO4_c" stoichiometry="1"/>
  </listOfProducts>
  <kineticLaw>
    <math xmlns="http://www.w3.org/1998/Math/MathML">
      <ci> FLUX_VALUE </ci>
    </math>
    <listOfParameters>
      <parameter id="LOWER_BOUND" value="0" units="mmol_per_gDW_per_hr" constant="true"/>
      <parameter id="UPPER_BOUND" value="1000" units="mmol_per_gDW_per_hr" constant="true"/>
      <parameter id="FLUX_VALUE" value="0" units="mmol_per_gDW_per_hr" constant="true"/>
      <parameter id="OBJECTIVE_COEFFICIENT" value="0" units="mmol_per_gDW_per_hr" constant="true"/>
    </listOfParameters>
  </kineticLaw>
</reaction>
<reaction id="R_R720" name="Phosphate transport" reversible="true">
```

```xml
<notes>
  <body xmlns="http://www.w3.org/1999/xhtml">
    <p>GENE_ASSOCIATION: (TTC1724 and TTC1725)</p>
    <p>SUBSYSTEM: Transport, Extracellular</p>
    <p>EC Number: </p>
    <p>Confidence Level: 1</p>
    <p>AUTHORS: Transport</p>
    <p/>
  </body>
</notes>
<listOfReactants>
  <speciesReference species="M_H_e" stoichiometry="1"/>
  <speciesReference species="M_Pi_e" stoichiometry="1"/>
</listOfReactants>
<listOfProducts>
  <speciesReference species="M_Pi_c" stoichiometry="1"/>
  <speciesReference species="M_H_c" stoichiometry="1"/>
</listOfProducts>
<kineticLaw>
  <math xmlns="http://www.w3.org/1998/Math/MathML">
    <ci> FLUX_VALUE </ci>
  </math>
  <listOfParameters>
    <parameter id="LOWER_BOUND" value="-1000" units="mmol_per_gDW_per_hr" constant="true"/>
    <parameter id="UPPER_BOUND" value="1000" units="mmol_per_gDW_per_hr" constant="true"/>
    <parameter id="FLUX_VALUE" value="0" units="mmol_per_gDW_per_hr" constant="true"/>
    <parameter id="OBJECTIVE_COEFFICIENT" value="0" units="mmol_per_gDW_per_hr" constant="true"/>
  </listOfParameters>
</kineticLaw>
```

```
</reaction>
<reaction id="R_R721" name="Carbon dioxide transport" reversible="true">
 <notes>
  <body xmlns="http://www.w3.org/1999/xhtml">
   <p>GENE_ASSOCIATION: </p>
   <p>SUBSYSTEM: Transport, Extracellular</p>
   <p>EC Number: </p>
   <p>Confidence Level: 1</p>
   <p>AUTHORS: Transport</p>
   <p/>
  </body>
 </notes>
 <listOfReactants>
  <speciesReference species="M_CO2_c" stoichiometry="1"/>
 </listOfReactants>
 <listOfProducts>
  <speciesReference species="M_CO2_e" stoichiometry="1"/>
 </listOfProducts>
 <kineticLaw>
  <math xmlns="http://www.w3.org/1998/Math/MathML">
   <ci> FLUX_VALUE </ci>
  </math>
  <listOfParameters>
   <parameter id="LOWER_BOUND" value="-1000" units="mmol_per_gDW_per_hr" constant="true"/>
   <parameter id="UPPER_BOUND" value="1000" units="mmol_per_gDW_per_hr" constant="true"/>
   <parameter id="FLUX_VALUE" value="0" units="mmol_per_gDW_per_hr" constant="true"/>
   <parameter id="OBJECTIVE_COEFFICIENT" value="0" units="mmol_per_gDW_per_hr" constant="true"/>
  </listOfParameters>
 </kineticLaw>
```

```xml
</reaction>
<reaction id="R_R722" name="Acetate transport" reversible="true">
  <notes>
    <body xmlns="http://www.w3.org/1999/xhtml">
      <p>GENE_ASSOCIATION: </p>
      <p>SUBSYSTEM: Transport, Extracellular</p>
      <p>EC Number: </p>
      <p>Confidence Level: 1</p>
      <p>AUTHORS: Transport</p>
      <p/>
    </body>
  </notes>
  <listOfReactants>
    <speciesReference species="M_AC_e" stoichiometry="1"/>
  </listOfReactants>
  <listOfProducts>
    <speciesReference species="M_AC_c" stoichiometry="1"/>
  </listOfProducts>
  <kineticLaw>
    <math xmlns="http://www.w3.org/1998/Math/MathML">
      <ci> FLUX_VALUE </ci>
    </math>
    <listOfParameters>
      <parameter id="LOWER_BOUND" value="-1000" units="mmol_per_gDW_per_hr" constant="true"/>
      <parameter id="UPPER_BOUND" value="1000" units="mmol_per_gDW_per_hr" constant="true"/>
      <parameter id="FLUX_VALUE" value="0" units="mmol_per_gDW_per_hr" constant="true"/>
      <parameter id="OBJECTIVE_COEFFICIENT" value="0" units="mmol_per_gDW_per_hr" constant="true"/>
    </listOfParameters>
  </kineticLaw>
```

```
</reaction>
<reaction id="R_R723" name="D-Alanyl-D-alanine transport via ABC system" reversible="true">
 <notes>
  <body xmlns="http://www.w3.org/1999/xhtml">
   <p>GENE_ASSOCIATION: ((TTC1270 or TTC0972) and TTC0973)</p>
   <p>SUBSYSTEM: Transport, Extracellular</p>
   <p>EC Number: </p>
   <p>Confidence Level: 1</p>
   <p>AUTHORS: Transport</p>
   <p/>
  </body>
 </notes>
 <listOfReactants>
  <speciesReference species="M_D_ALA_D_ALA_e" stoichiometry="1"/>
 </listOfReactants>
 <listOfProducts>
  <speciesReference species="M_D_ALA_D_ALA_c" stoichiometry="1"/>
 </listOfProducts>
 <kineticLaw>
  <math xmlns="http://www.w3.org/1998/Math/MathML">
   <ci> FLUX_VALUE </ci>
  </math>
  <listOfParameters>
   <parameter id="LOWER_BOUND" value="-1000" units="mmol_per_gDW_per_hr" constant="true"/>
   <parameter id="UPPER_BOUND" value="1000" units="mmol_per_gDW_per_hr" constant="true"/>
   <parameter id="FLUX_VALUE" value="0" units="mmol_per_gDW_per_hr" constant="true"/>
   <parameter id="OBJECTIVE_COEFFICIENT" value="0" units="mmol_per_gDW_per_hr" constant="true"/>
  </listOfParameters>
 </kineticLaw>
```

```
</reaction>
<reaction id="R_R724" name="L-Arginine transport" reversible="true">
 <notes>
  <body xmlns="http://www.w3.org/1999/xhtml">
   <p>GENE_ASSOCIATION: </p>
   <p>SUBSYSTEM: Transport, Extracellular</p>
   <p>EC Number: </p>
   <p>Confidence Level: 1</p>
   <p>AUTHORS: Transport</p>
   <p/>
  </body>
 </notes>
 <listOfReactants>
  <speciesReference species="M_L_ARG_e" stoichiometry="1"/>
 </listOfReactants>
 <listOfProducts>
  <speciesReference species="M_L_ARG_c" stoichiometry="1"/>
 </listOfProducts>
 <kineticLaw>
  <math xmlns="http://www.w3.org/1998/Math/MathML">
   <ci> FLUX_VALUE </ci>
  </math>
  <listOfParameters>
   <parameter id="LOWER_BOUND" value="-1000" units="mmol_per_gDW_per_hr" constant="true"/>
   <parameter id="UPPER_BOUND" value="1000" units="mmol_per_gDW_per_hr" constant="true"/>
   <parameter id="FLUX_VALUE" value="0" units="mmol_per_gDW_per_hr" constant="true"/>
   <parameter id="OBJECTIVE_COEFFICIENT" value="0" units="mmol_per_gDW_per_hr" constant="true"/>
  </listOfParameters>
 </kineticLaw>
```

```xml
</reaction>
<reaction id="R_R725" name="L-Asparagine transport" reversible="true">
  <notes>
    <body xmlns="http://www.w3.org/1999/xhtml">
      <p>GENE_ASSOCIATION: </p>
      <p>SUBSYSTEM: Transport, Extracellular</p>
      <p>EC Number: </p>
      <p>Confidence Level: 1</p>
      <p>AUTHORS: Transport</p>
      <p/>
    </body>
  </notes>
  <listOfReactants>
    <speciesReference species="M_L_ASN_e" stoichiometry="1"/>
  </listOfReactants>
  <listOfProducts>
    <speciesReference species="M_L_ASN_c" stoichiometry="1"/>
  </listOfProducts>
  <kineticLaw>
    <math xmlns="http://www.w3.org/1998/Math/MathML">
      <ci> FLUX_VALUE </ci>
    </math>
    <listOfParameters>
      <parameter id="LOWER_BOUND" value="-1000" units="mmol_per_gDW_per_hr" constant="true"/>
      <parameter id="UPPER_BOUND" value="1000" units="mmol_per_gDW_per_hr" constant="true"/>
      <parameter id="FLUX_VALUE" value="0" units="mmol_per_gDW_per_hr" constant="true"/>
      <parameter id="OBJECTIVE_COEFFICIENT" value="0" units="mmol_per_gDW_per_hr" constant="true"/>
    </listOfParameters>
  </kineticLaw>
```

```
</reaction>
<reaction id="R_R726" name="Citrate transport" reversible="true">
 <notes>
  <body xmlns="http://www.w3.org/1999/xhtml">
   <p>GENE_ASSOCIATION: </p>
   <p>SUBSYSTEM: Transport, Extracellular</p>
   <p>EC Number: </p>
   <p>Confidence Level: 1</p>
   <p>AUTHORS: Transport</p>
   <p/>
  </body>
 </notes>
 <listOfReactants>
  <speciesReference species="M_CIT_e" stoichiometry="1"/>
 </listOfReactants>
 <listOfProducts>
  <speciesReference species="M_CIT_c" stoichiometry="1"/>
 </listOfProducts>
 <kineticLaw>
  <math xmlns="http://www.w3.org/1998/Math/MathML">
   <ci> FLUX_VALUE </ci>
  </math>
  <listOfParameters>
   <parameter id="LOWER_BOUND" value="-1000" units="mmol_per_gDW_per_hr" constant="true"/>
   <parameter id="UPPER_BOUND" value="1000" units="mmol_per_gDW_per_hr" constant="true"/>
   <parameter id="FLUX_VALUE" value="0" units="mmol_per_gDW_per_hr" constant="true"/>
   <parameter id="OBJECTIVE_COEFFICIENT" value="0" units="mmol_per_gDW_per_hr" constant="true"/>
  </listOfParameters>
 </kineticLaw>
```

```xml
</reaction>

<reaction id="R_R727" name="Ethanol transport" reversible="true">

<notes>

<body xmlns="http://www.w3.org/1999/xhtml">

<p>GENE_ASSOCIATION: </p>

<p>SUBSYSTEM: Transport, Extracellular</p>

<p>EC Number: </p>

<p>Confidence Level: 1</p>

<p>AUTHORS: Transport</p>

<p/>

</body>

</notes>

<listOfReactants>

<speciesReference species="M_ETH_e" stoichiometry="1"/>

</listOfReactants>

<listOfProducts>

<speciesReference species="M_ETH_c" stoichiometry="1"/>

</listOfProducts>

<kineticLaw>

<math xmlns="http://www.w3.org/1998/Math/MathML">

<ci> FLUX_VALUE </ci>

</math>

<listOfParameters>

<parameter id="LOWER_BOUND" value="-1000" units="mmol_per_gDW_per_hr" constant="true"/>

<parameter id="UPPER_BOUND" value="1000" units="mmol_per_gDW_per_hr" constant="true"/>

<parameter id="FLUX_VALUE" value="0" units="mmol_per_gDW_per_hr" constant="true"/>

<parameter id="OBJECTIVE_COEFFICIENT" value="0" units="mmol_per_gDW_per_hr" constant="true"/>

</listOfParameters>

</kineticLaw>
```

```xml
</reaction>
<reaction id="R_R728" name="D-Fructose 1-phosphate transport" reversible="true">
<notes>
<body xmlns="http://www.w3.org/1999/xhtml">
<p>GENE_ASSOCIATION: </p>
<p>SUBSYSTEM: Transport, Extracellular</p>
<p>EC Number: </p>
<p>Confidence Level: 1</p>
<p>AUTHORS: Transport</p>
<p/>
</body>
</notes>
<listOfReactants>
<speciesReference species="M_F1P_e" stoichiometry="1"/>
</listOfReactants>
<listOfProducts>
<speciesReference species="M_F1P_c" stoichiometry="1"/>
</listOfProducts>
<kineticLaw>
<math xmlns="http://www.w3.org/1998/Math/MathML">
<ci> FLUX_VALUE </ci>
</math>
<listOfParameters>
<parameter id="LOWER_BOUND" value="-1000" units="mmol_per_gDW_per_hr" constant="true"/>
<parameter id="UPPER_BOUND" value="1000" units="mmol_per_gDW_per_hr" constant="true"/>
<parameter id="FLUX_VALUE" value="0" units="mmol_per_gDW_per_hr" constant="true"/>
<parameter id="OBJECTIVE_COEFFICIENT" value="0" units="mmol_per_gDW_per_hr" constant="true"/>
</listOfParameters>
</kineticLaw>
```

```xml
</reaction>

<reaction id="R_R729" name="L-Fuculose 1-phosphate transport" reversible="true">

<notes>

<body xmlns="http://www.w3.org/1999/xhtml">

<p>GENE_ASSOCIATION: </p>

<p>SUBSYSTEM: Transport, Extracellular</p>

<p>EC Number: </p>

<p>Confidence Level: 1</p>

<p>AUTHORS: Transport</p>

<p/>

</body>

</notes>

<listOfReactants>

<speciesReference species="M_FUCL1P_e" stoichiometry="1"/>

</listOfReactants>

<listOfProducts>

<speciesReference species="M_FUCL1P_c" stoichiometry="1"/>

</listOfProducts>

<kineticLaw>

<math xmlns="http://www.w3.org/1998/Math/MathML">

<ci> FLUX_VALUE </ci>

</math>

<listOfParameters>

<parameter id="LOWER_BOUND" value="-1000" units="mmol_per_gDW_per_hr" constant="true"/>

<parameter id="UPPER_BOUND" value="1000" units="mmol_per_gDW_per_hr" constant="true"/>

<parameter id="FLUX_VALUE" value="0" units="mmol_per_gDW_per_hr" constant="true"/>

<parameter id="OBJECTIVE_COEFFICIENT" value="0" units="mmol_per_gDW_per_hr" constant="true"/>

</listOfParameters>

</kineticLaw>
```

```
</reaction>
<reaction id="R_R730" name="Ferrous ion transport via diffusion" reversible="true">
 <notes>
  <body xmlns="http://www.w3.org/1999/xhtml">
   <p>GENE_ASSOCIATION: (TTC0354 or TTC0776 or TTC1358)</p>
   <p>SUBSYSTEM: Transport, Extracellular</p>
   <p>EC Number: </p>
   <p>Confidence Level: 1</p>
   <p>AUTHORS: Transport</p>
   <p/>
  </body>
 </notes>
 <listOfReactants>
  <speciesReference species="M_Fe2_e" stoichiometry="1"/>
 </listOfReactants>
 <listOfProducts>
  <speciesReference species="M_Fe2_c" stoichiometry="1"/>
 </listOfProducts>
 <kineticLaw>
  <math xmlns="http://www.w3.org/1998/Math/MathML">
   <ci> FLUX_VALUE </ci>
  </math>
  <listOfParameters>
   <parameter id="LOWER_BOUND" value="-1000" units="mmol_per_gDW_per_hr" constant="true"/>
   <parameter id="UPPER_BOUND" value="1000" units="mmol_per_gDW_per_hr" constant="true"/>
   <parameter id="FLUX_VALUE" value="0" units="mmol_per_gDW_per_hr" constant="true"/>
   <parameter id="OBJECTIVE_COEFFICIENT" value="0" units="mmol_per_gDW_per_hr" constant="true"/>
  </listOfParameters>
 </kineticLaw>
```

```
</reaction>
<reaction id="R_R731" name="D-Fructose transport via ABC transporter" reversible="false">
 <notes>
  <body xmlns="http://www.w3.org/1999/xhtml">
   <p>GENE_ASSOCIATION: ((TTC0326 or TTC0327) and TTC0328)</p>
   <p>SUBSYSTEM: Transport, Extracellular</p>
   <p>EC Number: </p>
   <p>Confidence Level: 1</p>
   <p>AUTHORS: Transport</p>
   <p/>
  </body>
 </notes>
 <listOfReactants>
  <speciesReference species="M_ATP_c" stoichiometry="1"/>
  <speciesReference species="M_H2O_c" stoichiometry="1"/>
  <speciesReference species="M_D_FRU_e" stoichiometry="1"/>
 </listOfReactants>
 <listOfProducts>
  <speciesReference species="M_ADP_c" stoichiometry="1"/>
  <speciesReference species="M_Pi_c" stoichiometry="1"/>
  <speciesReference species="M_H_c" stoichiometry="1"/>
  <speciesReference species="M_D_FRU_c" stoichiometry="1"/>
 </listOfProducts>
 <kineticLaw>
  <math xmlns="http://www.w3.org/1998/Math/MathML">
   <ci> FLUX_VALUE </ci>
  </math>
  <listOfParameters>
   <parameter id="LOWER_BOUND" value="0" units="mmol_per_gDW_per_hr" constant="true"/>
```

```
      <parameter id="UPPER_BOUND" value="1000" units="mmol_per_gDW_per_hr" constant="true"/>
      <parameter id="FLUX_VALUE" value="0" units="mmol_per_gDW_per_hr" constant="true"/>
      <parameter id="OBJECTIVE_COEFFICIENT" value="0" units="mmol_per_gDW_per_hr" constant="true"/>
    </listOfParameters>
  </kineticLaw>
</reaction>
<reaction id="R_R732" name="Fumarate transport via proton symport" reversible="true">
  <notes>
    <body xmlns="http://www.w3.org/1999/xhtml">
      <p>GENE_ASSOCIATION: TT_P0033 </p>
      <p>SUBSYSTEM: Transport, Extracellular</p>
      <p>EC Number: </p>
      <p>Confidence Level: 1</p>
      <p>AUTHORS: Transport</p>
      <p/>
    </body>
  </notes>
  <listOfReactants>
    <speciesReference species="M_H_e" stoichiometry="2"/>
    <speciesReference species="M_FUM_e" stoichiometry="1"/>
  </listOfReactants>
  <listOfProducts>
    <speciesReference species="M_H_c" stoichiometry="2"/>
    <speciesReference species="M_FUM_c" stoichiometry="1"/>
  </listOfProducts>
  <kineticLaw>
    <math xmlns="http://www.w3.org/1998/Math/MathML">
      <ci> FLUX_VALUE </ci>
    </math>
```

```xml
    <listOfParameters>
      <parameter id="LOWER_BOUND" value="-1000" units="mmol_per_gDW_per_hr" constant="true"/>
      <parameter id="UPPER_BOUND" value="1000" units="mmol_per_gDW_per_hr" constant="true"/>
      <parameter id="FLUX_VALUE" value="0" units="mmol_per_gDW_per_hr" constant="true"/>
      <parameter id="OBJECTIVE_COEFFICIENT" value="0" units="mmol_per_gDW_per_hr" constant="true"/>
    </listOfParameters>
  </kineticLaw>
</reaction>
<reaction id="R_R733" name="alpha-D-Glucosamine 1-phosphate transport" reversible="true">
  <notes>
    <body xmlns="http://www.w3.org/1999/xhtml">
      <p>GENE_ASSOCIATION: </p>
      <p>SUBSYSTEM: Transport, Extracellular</p>
      <p>EC Number: </p>
      <p>Confidence Level: 1</p>
      <p>AUTHORS: Transport</p>
      <p/>
    </body>
  </notes>
  <listOfReactants>
    <speciesReference species="M_GLUCSA1P_e" stoichiometry="1"/>
  </listOfReactants>
  <listOfProducts>
    <speciesReference species="M_GLUCSA1P_c" stoichiometry="1"/>
  </listOfProducts>
  <kineticLaw>
    <math xmlns="http://www.w3.org/1998/Math/MathML">
      <ci> FLUX_VALUE </ci>
    </math>
```

```
    <listOfParameters>
      <parameter id="LOWER_BOUND" value="-1000" units="mmol_per_gDW_per_hr" constant="true"/>
      <parameter id="UPPER_BOUND" value="1000" units="mmol_per_gDW_per_hr" constant="true"/>
      <parameter id="FLUX_VALUE" value="0" units="mmol_per_gDW_per_hr" constant="true"/>
      <parameter id="OBJECTIVE_COEFFICIENT" value="0" units="mmol_per_gDW_per_hr" constant="true"/>
    </listOfParameters>
  </kineticLaw>
</reaction>
<reaction id="R_R734" name="D-Glucosamine 6-phosphate transport" reversible="true">
  <notes>
    <body xmlns="http://www.w3.org/1999/xhtml">
      <p>GENE_ASSOCIATION: </p>
      <p>SUBSYSTEM: Transport, Extracellular</p>
      <p>EC Number: </p>
      <p>Confidence Level: 1</p>
      <p>AUTHORS: Transport</p>
      <p/>
    </body>
  </notes>
  <listOfReactants>
    <speciesReference species="M_GLUSAP_e" stoichiometry="1"/>
  </listOfReactants>
  <listOfProducts>
    <speciesReference species="M_GLUSAP_c" stoichiometry="1"/>
  </listOfProducts>
  <kineticLaw>
    <math xmlns="http://www.w3.org/1998/Math/MathML">
      <ci> FLUX_VALUE </ci>
    </math>
```

```
    <listOfParameters>
      <parameter id="LOWER_BOUND" value="-1000" units="mmol_per_gDW_per_hr" constant="true"/>
      <parameter id="UPPER_BOUND" value="1000" units="mmol_per_gDW_per_hr" constant="true"/>
      <parameter id="FLUX_VALUE" value="0" units="mmol_per_gDW_per_hr" constant="true"/>
      <parameter id="OBJECTIVE_COEFFICIENT" value="0" units="mmol_per_gDW_per_hr" constant="true"/>
    </listOfParameters>
  </kineticLaw>
</reaction>
<reaction id="R_R735" name="Glycolaldehyde transport" reversible="true">
  <notes>
    <body xmlns="http://www.w3.org/1999/xhtml">
      <p>GENE_ASSOCIATION: </p>
      <p>SUBSYSTEM: Transport, Extracellular</p>
      <p>EC Number: </p>
      <p>Confidence Level: 1</p>
      <p>AUTHORS: Transport</p>
      <p/>
    </body>
  </notes>
  <listOfReactants>
    <speciesReference species="M_GLYCALD_e" stoichiometry="1"/>
  </listOfReactants>
  <listOfProducts>
    <speciesReference species="M_GLYCALD_c" stoichiometry="1"/>
  </listOfProducts>
  <kineticLaw>
    <math xmlns="http://www.w3.org/1998/Math/MathML">
      <ci> FLUX_VALUE </ci>
    </math>
```

```xml
<listOfParameters>
<parameter id="LOWER_BOUND" value="-1000" units="mmol_per_gDW_per_hr" constant="true"/>
<parameter id="UPPER_BOUND" value="1000" units="mmol_per_gDW_per_hr" constant="true"/>
<parameter id="FLUX_VALUE" value="0" units="mmol_per_gDW_per_hr" constant="true"/>
<parameter id="OBJECTIVE_COEFFICIENT" value="0" units="mmol_per_gDW_per_hr" constant="true"/>
</listOfParameters>
</kineticLaw>
</reaction>
<reaction id="R_R736" name="D-Glutamate transport" reversible="true">
<notes>
<body xmlns="http://www.w3.org/1999/xhtml">
<p>GENE_ASSOCIATION: </p>
<p>SUBSYSTEM: Transport, Extracellular</p>
<p>EC Number: </p>
<p>Confidence Level: 1</p>
<p>AUTHORS: Transport</p>
<p/>
</body>
</notes>
<listOfReactants>
<speciesReference species="M_D_GLU_e" stoichiometry="1"/>
</listOfReactants>
<listOfProducts>
<speciesReference species="M_D_GLU_c" stoichiometry="1"/>
</listOfProducts>
<kineticLaw>
<math xmlns="http://www.w3.org/1998/Math/MathML">
<ci> FLUX_VALUE </ci>
</math>
```

```xml
    <listOfParameters>
      <parameter id="LOWER_BOUND" value="-1000" units="mmol_per_gDW_per_hr" constant="true"/>
      <parameter id="UPPER_BOUND" value="1000" units="mmol_per_gDW_per_hr" constant="true"/>
      <parameter id="FLUX_VALUE" value="0" units="mmol_per_gDW_per_hr" constant="true"/>
      <parameter id="OBJECTIVE_COEFFICIENT" value="0" units="mmol_per_gDW_per_hr" constant="true"/>
    </listOfParameters>
  </kineticLaw>
</reaction>
<reaction id="R_R737" name="Glycolate transport" reversible="true">
  <notes>
    <body xmlns="http://www.w3.org/1999/xhtml">
      <p>GENE_ASSOCIATION: </p>
      <p>SUBSYSTEM: Transport, Extracellular</p>
      <p>EC Number: </p>
      <p>Confidence Level: 1</p>
      <p>AUTHORS: Transport</p>
      <p/>
    </body>
  </notes>
  <listOfReactants>
    <speciesReference species="M_HY_AC_e" stoichiometry="1"/>
  </listOfReactants>
  <listOfProducts>
    <speciesReference species="M_HY_AC_c" stoichiometry="1"/>
  </listOfProducts>
  <kineticLaw>
    <math xmlns="http://www.w3.org/1998/Math/MathML">
      <ci> FLUX_VALUE </ci>
    </math>
```

```
      <listOfParameters>
        <parameter id="LOWER_BOUND" value="-1000" units="mmol_per_gDW_per_hr" constant="true"/>
        <parameter id="UPPER_BOUND" value="1000" units="mmol_per_gDW_per_hr" constant="true"/>
        <parameter id="FLUX_VALUE" value="0" units="mmol_per_gDW_per_hr" constant="true"/>
        <parameter id="OBJECTIVE_COEFFICIENT" value="0" units="mmol_per_gDW_per_hr" constant="true"/>
      </listOfParameters>
    </kineticLaw>
  </reaction>
  <reaction id="R_R738" name="D-Glycerate transport" reversible="true">
    <notes>
      <body xmlns="http://www.w3.org/1999/xhtml">
        <p>GENE_ASSOCIATION: </p>
        <p>SUBSYSTEM: Transport, Extracellular</p>
        <p>EC Number: </p>
        <p>Confidence Level: 1</p>
        <p>AUTHORS: Transport</p>
        <p/>
      </body>
    </notes>
    <listOfReactants>
      <speciesReference species="M_D_GLCT_e" stoichiometry="1"/>
    </listOfReactants>
    <listOfProducts>
      <speciesReference species="M_D_GLCT_c" stoichiometry="1"/>
    </listOfProducts>
    <kineticLaw>
      <math xmlns="http://www.w3.org/1998/Math/MathML">
        <ci> FLUX_VALUE </ci>
      </math>
```

```
    <listOfParameters>
      <parameter id="LOWER_BOUND" value="-1000" units="mmol_per_gDW_per_hr" constant="true"/>
      <parameter id="UPPER_BOUND" value="1000" units="mmol_per_gDW_per_hr" constant="true"/>
      <parameter id="FLUX_VALUE" value="0" units="mmol_per_gDW_per_hr" constant="true"/>
      <parameter id="OBJECTIVE_COEFFICIENT" value="0" units="mmol_per_gDW_per_hr" constant="true"/>
    </listOfParameters>
  </kineticLaw>
</reaction>
<reaction id="R_R739" name="Glycerol transport" reversible="true">
  <notes>
    <body xmlns="http://www.w3.org/1999/xhtml">
      <p>GENE_ASSOCIATION: </p>
      <p>SUBSYSTEM: Transport, Extracellular</p>
      <p>EC Number: </p>
      <p>Confidence Level: 1</p>
      <p>AUTHORS: Transport</p>
      <p/>
    </body>
  </notes>
  <listOfReactants>
    <speciesReference species="M_GLYCRL_e" stoichiometry="1"/>
  </listOfReactants>
  <listOfProducts>
    <speciesReference species="M_GLYCRL_c" stoichiometry="1"/>
  </listOfProducts>
  <kineticLaw>
    <math xmlns="http://www.w3.org/1998/Math/MathML">
      <ci> FLUX_VALUE </ci>
    </math>
```

```
      <listOfParameters>
        <parameter id="LOWER_BOUND" value="-1000" units="mmol_per_gDW_per_hr" constant="true"/>
        <parameter id="UPPER_BOUND" value="1000" units="mmol_per_gDW_per_hr" constant="true"/>
        <parameter id="FLUX_VALUE" value="0" units="mmol_per_gDW_per_hr" constant="true"/>
        <parameter id="OBJECTIVE_COEFFICIENT" value="0" units="mmol_per_gDW_per_hr" constant="true"/>
      </listOfParameters>
    </kineticLaw>
  </reaction>
  <reaction id="R_R740" name="Glycine transport" reversible="true">
    <notes>
      <body xmlns="http://www.w3.org/1999/xhtml">
        <p>GENE_ASSOCIATION: </p>
        <p>SUBSYSTEM: Transport, Extracellular</p>
        <p>EC Number: </p>
        <p>Confidence Level: 1</p>
        <p>AUTHORS: Transport</p>
        <p/>
      </body>
    </notes>
    <listOfReactants>
      <speciesReference species="M_L_GLY_e" stoichiometry="1"/>
    </listOfReactants>
    <listOfProducts>
      <speciesReference species="M_L_GLY_c" stoichiometry="1"/>
    </listOfProducts>
    <kineticLaw>
      <math xmlns="http://www.w3.org/1998/Math/MathML">
        <ci> FLUX_VALUE </ci>
      </math>
```

```
    <listOfParameters>
      <parameter id="LOWER_BOUND" value="-1000" units="mmol_per_gDW_per_hr" constant="true"/>
      <parameter id="UPPER_BOUND" value="1000" units="mmol_per_gDW_per_hr" constant="true"/>
      <parameter id="FLUX_VALUE" value="0" units="mmol_per_gDW_per_hr" constant="true"/>
      <parameter id="OBJECTIVE_COEFFICIENT" value="0" units="mmol_per_gDW_per_hr" constant="true"/>
    </listOfParameters>
  </kineticLaw>
</reaction>
<reaction id="R_R741" name="Hydrogen sulfide transport" reversible="true">
  <notes>
    <body xmlns="http://www.w3.org/1999/xhtml">
      <p>GENE_ASSOCIATION: </p>
      <p>SUBSYSTEM: Transport, Extracellular</p>
      <p>EC Number: </p>
      <p>Confidence Level: 1</p>
      <p>AUTHORS: Transport</p>
      <p/>
    </body>
  </notes>
  <listOfReactants>
    <speciesReference species="M_H2S_e" stoichiometry="1"/>
  </listOfReactants>
  <listOfProducts>
    <speciesReference species="M_H2S_c" stoichiometry="1"/>
  </listOfProducts>
  <kineticLaw>
    <math xmlns="http://www.w3.org/1998/Math/MathML">
      <ci> FLUX_VALUE </ci>
    </math>
```

```xml
<listOfParameters>
<parameter id="LOWER_BOUND" value="0" units="mmol_per_gDW_per_hr" constant="true"/>
<parameter id="UPPER_BOUND" value="1000" units="mmol_per_gDW_per_hr" constant="true"/>
<parameter id="FLUX_VALUE" value="0" units="mmol_per_gDW_per_hr" constant="true"/>
<parameter id="OBJECTIVE_COEFFICIENT" value="0" units="mmol_per_gDW_per_hr" constant="true"/>
</listOfParameters>
</kineticLaw>
</reaction>
<reaction id="R_R742" name="L-Homocysteine transport" reversible="true">
<notes>
<body xmlns="http://www.w3.org/1999/xhtml">
<p>GENE_ASSOCIATION: </p>
<p>SUBSYSTEM: Transport, Extracellular</p>
<p>EC Number: </p>
<p>Confidence Level: 1</p>
<p>AUTHORS: Transport</p>
<p/>
</body>
</notes>
<listOfReactants>
<speciesReference species="M_L_HCYS_e" stoichiometry="1"/>
</listOfReactants>
<listOfProducts>
<speciesReference species="M_L_HCYS_c" stoichiometry="1"/>
</listOfProducts>
<kineticLaw>
<math xmlns="http://www.w3.org/1998/Math/MathML">
<ci> FLUX_VALUE </ci>
</math>
```

```xml
<listOfParameters>

<parameter id="LOWER_BOUND" value="-1000" units="mmol_per_gDW_per_hr" constant="true"/>

<parameter id="UPPER_BOUND" value="1000" units="mmol_per_gDW_per_hr" constant="true"/>

<parameter id="FLUX_VALUE" value="0" units="mmol_per_gDW_per_hr" constant="true"/>

<parameter id="OBJECTIVE_COEFFICIENT" value="0" units="mmol_per_gDW_per_hr" constant="true"/>

</listOfParameters>

</kineticLaw>

</reaction>

<reaction id="R_R743" name="L-Isoleucine transport via ABC system" reversible="false">

<notes>

<body xmlns="http://www.w3.org/1999/xhtml">

<p>GENE_ASSOCIATION: ((TTC0219 or TTC0334 or TTC0970 or TTC0600 or TTC0083) and (TTC0218 or TTC0335 or TTC0969 or TTC0078)) and ((TTC0220 or TTC0337 or TTC0598 or TTC0967 or TTC0080) and (TTC216 or TTC0968 or TTC0081))</p>

<p>SUBSYSTEM: Transport, Extracellular</p>

<p>EC Number: </p>

<p>Confidence Level: 1</p>

<p>AUTHORS: Transport</p>

<p/>

</body>

</notes>

<listOfReactants>

<speciesReference species="M_ATP_c" stoichiometry="1"/>

<speciesReference species="M_H2O_c" stoichiometry="1"/>

<speciesReference species="M_L_ILE_e" stoichiometry="1"/>

</listOfReactants>

<listOfProducts>

<speciesReference species="M_L_ILE_c" stoichiometry="1"/>

<speciesReference species="M_ADP_c" stoichiometry="1"/>

<speciesReference species="M_Pi_c" stoichiometry="1"/>
```

```xml
</listOfProducts>
<kineticLaw>
<math xmlns="http://www.w3.org/1998/Math/MathML">
<ci> FLUX_VALUE </ci>
</math>
<listOfParameters>
<parameter id="LOWER_BOUND" value="0" units="mmol_per_gDW_per_hr" constant="true"/>
<parameter id="UPPER_BOUND" value="1000" units="mmol_per_gDW_per_hr" constant="true"/>
<parameter id="FLUX_VALUE" value="0" units="mmol_per_gDW_per_hr" constant="true"/>
<parameter id="OBJECTIVE_COEFFICIENT" value="0" units="mmol_per_gDW_per_hr" constant="true"/>
</listOfParameters>
</kineticLaw>
</reaction>
<reaction id="R_R744" name="L-Lysine transport " reversible="true">
<notes>
<body xmlns="http://www.w3.org/1999/xhtml">
<p>GENE_ASSOCIATION: </p>
<p>SUBSYSTEM: Transport, Extracellular</p>
<p>EC Number: </p>
<p>Confidence Level: 1</p>
<p>AUTHORS: Transport</p>
<p/>
</body>
</notes>
<listOfReactants>
<speciesReference species="M_L_LYS_e" stoichiometry="1"/>
</listOfReactants>
<listOfProducts>
<speciesReference species="M_L_LYS_c" stoichiometry="1"/>
```

```
  </listOfProducts>
  <kineticLaw>
    <math xmlns="http://www.w3.org/1998/Math/MathML">
      <ci> FLUX_VALUE </ci>
    </math>
    <listOfParameters>
      <parameter id="LOWER_BOUND" value="-1000" units="mmol_per_gDW_per_hr" constant="true"/>
      <parameter id="UPPER_BOUND" value="1000" units="mmol_per_gDW_per_hr" constant="true"/>
      <parameter id="FLUX_VALUE" value="0" units="mmol_per_gDW_per_hr" constant="true"/>
      <parameter id="OBJECTIVE_COEFFICIENT" value="0" units="mmol_per_gDW_per_hr" constant="true"/>
    </listOfParameters>
  </kineticLaw>
</reaction>
<reaction id="R_R745" name="L-Malate transport via proton symport" reversible="true">
  <notes>
    <body xmlns="http://www.w3.org/1999/xhtml">
      <p>GENE_ASSOCIATION: TT_P0033 </p>
      <p>SUBSYSTEM: Transport, Extracellular</p>
      <p>EC Number: </p>
      <p>Confidence Level: 1</p>
      <p>AUTHORS: Transport</p>
      <p/>
    </body>
  </notes>
  <listOfReactants>
    <speciesReference species="M_H_e" stoichiometry="2"/>
    <speciesReference species="M_MAL_e" stoichiometry="1"/>
  </listOfReactants>
  <listOfProducts>
```

```
<speciesReference species="M_H_c" stoichiometry="2"/>
<speciesReference species="M_MAL_c" stoichiometry="1"/>
</listOfProducts>
<kineticLaw>
<math xmlns="http://www.w3.org/1998/Math/MathML">
<ci> FLUX_VALUE </ci>
</math>
<listOfParameters>
<parameter id="LOWER_BOUND" value="-1000" units="mmol_per_gDW_per_hr" constant="true"/>
<parameter id="UPPER_BOUND" value="1000" units="mmol_per_gDW_per_hr" constant="true"/>
<parameter id="FLUX_VALUE" value="0" units="mmol_per_gDW_per_hr" constant="true"/>
<parameter id="OBJECTIVE_COEFFICIENT" value="0" units="mmol_per_gDW_per_hr" constant="true"/>
</listOfParameters>
</kineticLaw>
</reaction>
<reaction id="R_R746" name="Maltose transport via ABC transporter" reversible="false">
<notes>
<body xmlns="http://www.w3.org/1999/xhtml">
<p>GENE_ASSOCIATION: (((TTC1287 or TTC1286) and TTC1288) or ((TTC1628 or TTC1629) and TTC1627) or ((TTC0612 or TTC0613) and TTC0615))</p>
<p>SUBSYSTEM: Transport, Extracellular</p>
<p>EC Number: </p>
<p>Confidence Level: 1</p>
<p>AUTHORS: Transport</p>
<p/>
</body>
</notes>
<listOfReactants>
<speciesReference species="M_ATP_c" stoichiometry="1"/>
```

```xml
    <speciesReference species="M_H2O_c" stoichiometry="1"/>
    <speciesReference species="M_MALT_e" stoichiometry="1"/>
  </listOfReactants>
  <listOfProducts>
    <speciesReference species="M_ADP_c" stoichiometry="1"/>
    <speciesReference species="M_Pi_c" stoichiometry="1"/>
    <speciesReference species="M_H_c" stoichiometry="1"/>
    <speciesReference species="M_MALT_c" stoichiometry="1"/>
  </listOfProducts>
  <kineticLaw>
    <math xmlns="http://www.w3.org/1998/Math/MathML">
      <ci> FLUX_VALUE </ci>
    </math>
    <listOfParameters>
      <parameter id="LOWER_BOUND" value="0" units="mmol_per_gDW_per_hr" constant="true"/>
      <parameter id="UPPER_BOUND" value="1000" units="mmol_per_gDW_per_hr" constant="true"/>
      <parameter id="FLUX_VALUE" value="0" units="mmol_per_gDW_per_hr" constant="true"/>
      <parameter id="OBJECTIVE_COEFFICIENT" value="0" units="mmol_per_gDW_per_hr" constant="true"/>
    </listOfParameters>
  </kineticLaw>
</reaction>
<reaction id="R_R747" name="Isomaltose transport via ABC transporter" reversible="false">
  <notes>
    <body xmlns="http://www.w3.org/1999/xhtml">
      <p>GENE_ASSOCIATION: </p>
      <p>SUBSYSTEM: Transport, Extracellular</p>
      <p>EC Number: </p>
      <p>Confidence Level: 1</p>
      <p>AUTHORS: Transport</p>
```

```
      <p/>
    </body>
  </notes>
  <listOfReactants>
    <speciesReference species="M_ATP_c" stoichiometry="1"/>
    <speciesReference species="M_H2O_c" stoichiometry="1"/>
    <speciesReference species="M_IMALT_e" stoichiometry="1"/>
  </listOfReactants>
  <listOfProducts>
    <speciesReference species="M_ADP_c" stoichiometry="1"/>
    <speciesReference species="M_Pi_c" stoichiometry="1"/>
    <speciesReference species="M_H_c" stoichiometry="1"/>
    <speciesReference species="M_IMALT_c" stoichiometry="1"/>
  </listOfProducts>
  <kineticLaw>
    <math xmlns="http://www.w3.org/1998/Math/MathML">
      <ci> FLUX_VALUE </ci>
    </math>
    <listOfParameters>
      <parameter id="LOWER_BOUND" value="0" units="mmol_per_gDW_per_hr" constant="true"/>
      <parameter id="UPPER_BOUND" value="1000" units="mmol_per_gDW_per_hr" constant="true"/>
      <parameter id="FLUX_VALUE" value="0" units="mmol_per_gDW_per_hr" constant="true"/>
      <parameter id="OBJECTIVE_COEFFICIENT" value="0" units="mmol_per_gDW_per_hr" constant="true"/>
    </listOfParameters>
  </kineticLaw>
</reaction>
<reaction id="R_R748" name="D-Mannose 1-phosphate transport" reversible="true">
  <notes>
    <body xmlns="http://www.w3.org/1999/xhtml">
```

<p>GENE_ASSOCIATION: </p>
<p>SUBSYSTEM: Transport, Extracellular</p>
<p>EC Number: </p>
<p>Confidence Level: 1</p>
<p>AUTHORS: Transport</p>
<p/>
</body>
</notes>
<listOfReactants>
<speciesReference species="M_MAN1P_e" stoichiometry="1"/>
</listOfReactants>
<listOfProducts>
<speciesReference species="M_MAN1P_c" stoichiometry="1"/>
</listOfProducts>
<kineticLaw>
<math xmlns="http://www.w3.org/1998/Math/MathML">
<ci> FLUX_VALUE </ci>
</math>
<listOfParameters>
<parameter id="LOWER_BOUND" value="-1000" units="mmol_per_gDW_per_hr" constant="true"/>
<parameter id="UPPER_BOUND" value="1000" units="mmol_per_gDW_per_hr" constant="true"/>
<parameter id="FLUX_VALUE" value="0" units="mmol_per_gDW_per_hr" constant="true"/>
<parameter id="OBJECTIVE_COEFFICIENT" value="0" units="mmol_per_gDW_per_hr" constant="true"/>
</listOfParameters>
</kineticLaw>
</reaction>
<reaction id="R_R749" name="D-Mannose 6-phosphate transport" reversible="true">
<notes>
<body xmlns="http://www.w3.org/1999/xhtml">

```
      <p>GENE_ASSOCIATION: </p>
      <p>SUBSYSTEM: Transport, Extracellular</p>
      <p>EC Number: </p>
      <p>Confidence Level: 1</p>
      <p>AUTHORS: Transport</p>
      <p/>
    </body>
  </notes>
  <listOfReactants>
    <speciesReference species="M_M6P_e" stoichiometry="1"/>
  </listOfReactants>
  <listOfProducts>
    <speciesReference species="M_M6P_c" stoichiometry="1"/>
  </listOfProducts>
  <kineticLaw>
    <math xmlns="http://www.w3.org/1998/Math/MathML">
      <ci> FLUX_VALUE </ci>
    </math>
    <listOfParameters>
      <parameter id="LOWER_BOUND" value="-1000" units="mmol_per_gDW_per_hr" constant="true"/>
      <parameter id="UPPER_BOUND" value="1000" units="mmol_per_gDW_per_hr" constant="true"/>
      <parameter id="FLUX_VALUE" value="0" units="mmol_per_gDW_per_hr" constant="true"/>
      <parameter id="OBJECTIVE_COEFFICIENT" value="0" units="mmol_per_gDW_per_hr" constant="true"/>
    </listOfParameters>
  </kineticLaw>
</reaction>
<reaction id="R_R750" name="D-Mannose transport via ABC transporter" reversible="false">
  <notes>
    <body xmlns="http://www.w3.org/1999/xhtml">
```

```
    <p>GENE_ASSOCIATION: ((TTC0326 or TTC0327) and TTC0328)</p>
    <p>SUBSYSTEM: Transport, Extracellular</p>
    <p>EC Number: </p>
    <p>Confidence Level: 1</p>
    <p>AUTHORS: Transport</p>
    <p/>
  </body>
</notes>
<listOfReactants>
  <speciesReference species="M_ATP_c" stoichiometry="1"/>
  <speciesReference species="M_H2O_c" stoichiometry="1"/>
  <speciesReference species="M_D_MAN_e" stoichiometry="1"/>
</listOfReactants>
<listOfProducts>
  <speciesReference species="M_ADP_c" stoichiometry="1"/>
  <speciesReference species="M_Pi_c" stoichiometry="1"/>
  <speciesReference species="M_H_c" stoichiometry="1"/>
  <speciesReference species="M_D_MAN_c" stoichiometry="1"/>
</listOfProducts>
<kineticLaw>
  <math xmlns="http://www.w3.org/1998/Math/MathML">
    <ci> FLUX_VALUE </ci>
  </math>
  <listOfParameters>
    <parameter id="LOWER_BOUND" value="0" units="mmol_per_gDW_per_hr" constant="true"/>
    <parameter id="UPPER_BOUND" value="1000" units="mmol_per_gDW_per_hr" constant="true"/>
    <parameter id="FLUX_VALUE" value="0" units="mmol_per_gDW_per_hr" constant="true"/>
    <parameter id="OBJECTIVE_COEFFICIENT" value="0" units="mmol_per_gDW_per_hr" constant="true"/>
  </listOfParameters>
```

```
  </kineticLaw>
</reaction>
<reaction id="R_R751" name="L-Phenylalanine transport" reversible="true">
  <notes>
    <body xmlns="http://www.w3.org/1999/xhtml">
      <p>GENE_ASSOCIATION: </p>
      <p>SUBSYSTEM: Transport, Extracellular</p>
      <p>EC Number: </p>
      <p>Confidence Level: 1</p>
      <p>AUTHORS: Transport</p>
      <p/>
    </body>
  </notes>
  <listOfReactants>
    <speciesReference species="M_L_PHE_e" stoichiometry="1"/>
  </listOfReactants>
  <listOfProducts>
    <speciesReference species="M_L_PHE_c" stoichiometry="1"/>
  </listOfProducts>
  <kineticLaw>
    <math xmlns="http://www.w3.org/1998/Math/MathML">
      <ci> FLUX_VALUE </ci>
    </math>
    <listOfParameters>
      <parameter id="LOWER_BOUND" value="-1000" units="mmol_per_gDW_per_hr" constant="true"/>
      <parameter id="UPPER_BOUND" value="1000" units="mmol_per_gDW_per_hr" constant="true"/>
      <parameter id="FLUX_VALUE" value="0" units="mmol_per_gDW_per_hr" constant="true"/>
      <parameter id="OBJECTIVE_COEFFICIENT" value="0" units="mmol_per_gDW_per_hr" constant="true"/>
    </listOfParameters>
```

```xml
</kineticLaw>
</reaction>
<reaction id="R_R752" name="L-Proline transport" reversible="true">
<notes>
<body xmlns="http://www.w3.org/1999/xhtml">
<p>GENE_ASSOCIATION: </p>
<p>SUBSYSTEM: Transport, Extracellular</p>
<p>EC Number: </p>
<p>Confidence Level: 1</p>
<p>AUTHORS: Transport</p>
<p/>
</body>
</notes>
<listOfReactants>
<speciesReference species="M_L_PRO_e" stoichiometry="1"/>
</listOfReactants>
<listOfProducts>
<speciesReference species="M_L_PRO_c" stoichiometry="1"/>
</listOfProducts>
<kineticLaw>
<math xmlns="http://www.w3.org/1998/Math/MathML">
<ci> FLUX_VALUE </ci>
</math>
<listOfParameters>
<parameter id="LOWER_BOUND" value="-1000" units="mmol_per_gDW_per_hr" constant="true"/>
<parameter id="UPPER_BOUND" value="1000" units="mmol_per_gDW_per_hr" constant="true"/>
<parameter id="FLUX_VALUE" value="0" units="mmol_per_gDW_per_hr" constant="true"/>
<parameter id="OBJECTIVE_COEFFICIENT" value="0" units="mmol_per_gDW_per_hr" constant="true"/>
</listOfParameters>
```

```xml
    </kineticLaw>
  </reaction>
  <reaction id="R_R753" name="D-Ribose transport via proton symport" reversible="true">
    <notes>
      <body xmlns="http://www.w3.org/1999/xhtml">
        <p>GENE_ASSOCIATION: (TTC0734 and (TTC1505 or TTC1504) and TTC0733)</p>
        <p>SUBSYSTEM: Transport, Extracellular</p>
        <p>EC Number: </p>
        <p>Confidence Level: 1</p>
        <p>AUTHORS: Transport</p>
        <p/>
      </body>
    </notes>
    <listOfReactants>
      <speciesReference species="M_H_e" stoichiometry="1"/>
      <speciesReference species="M_D_RIB_e" stoichiometry="1"/>
    </listOfReactants>
    <listOfProducts>
      <speciesReference species="M_H_c" stoichiometry="1"/>
      <speciesReference species="M_D_RIB_c" stoichiometry="1"/>
    </listOfProducts>
    <kineticLaw>
      <math xmlns="http://www.w3.org/1998/Math/MathML">
        <ci> FLUX_VALUE </ci>
      </math>
      <listOfParameters>
        <parameter id="LOWER_BOUND" value="-1000" units="mmol_per_gDW_per_hr" constant="true"/>
        <parameter id="UPPER_BOUND" value="1000" units="mmol_per_gDW_per_hr" constant="true"/>
        <parameter id="FLUX_VALUE" value="0" units="mmol_per_gDW_per_hr" constant="true"/>
```

```xml
      <parameter id="OBJECTIVE_COEFFICIENT" value="0" units="mmol_per_gDW_per_hr" constant="true"/>
    </listOfParameters>
  </kineticLaw>
</reaction>
<reaction id="R_R754" name="D-Sorbitol transport" reversible="true">
  <notes>
    <body xmlns="http://www.w3.org/1999/xhtml">
      <p>GENE_ASSOCIATION: </p>
      <p>SUBSYSTEM: Transport, Extracellular</p>
      <p>EC Number: </p>
      <p>Confidence Level: 1</p>
      <p>AUTHORS: Transport</p>
      <p/>
    </body>
  </notes>
  <listOfReactants>
    <speciesReference species="M_SORB_e" stoichiometry="1"/>
  </listOfReactants>
  <listOfProducts>
    <speciesReference species="M_SORB_c" stoichiometry="1"/>
  </listOfProducts>
  <kineticLaw>
    <math xmlns="http://www.w3.org/1998/Math/MathML">
      <ci> FLUX_VALUE </ci>
    </math>
    <listOfParameters>
      <parameter id="LOWER_BOUND" value="-1000" units="mmol_per_gDW_per_hr" constant="true"/>
      <parameter id="UPPER_BOUND" value="1000" units="mmol_per_gDW_per_hr" constant="true"/>
      <parameter id="FLUX_VALUE" value="0" units="mmol_per_gDW_per_hr" constant="true"/>
```

```
      <parameter id="OBJECTIVE_COEFFICIENT" value="0" units="mmol_per_gDW_per_hr" constant="true"/>
    </listOfParameters>
  </kineticLaw>
</reaction>
<reaction id="R_R755" name="L-Serine transport" reversible="true">
  <notes>
    <body xmlns="http://www.w3.org/1999/xhtml">
      <p>GENE_ASSOCIATION: </p>
      <p>SUBSYSTEM: Transport, Extracellular</p>
      <p>EC Number: </p>
      <p>Confidence Level: 1</p>
      <p>AUTHORS: Transport</p>
      <p/>
    </body>
  </notes>
  <listOfReactants>
    <speciesReference species="M_L_SER_e" stoichiometry="1"/>
  </listOfReactants>
  <listOfProducts>
    <speciesReference species="M_L_SER_c" stoichiometry="1"/>
  </listOfProducts>
  <kineticLaw>
    <math xmlns="http://www.w3.org/1998/Math/MathML">
      <ci> FLUX_VALUE </ci>
    </math>
    <listOfParameters>
      <parameter id="LOWER_BOUND" value="-1000" units="mmol_per_gDW_per_hr" constant="true"/>
      <parameter id="UPPER_BOUND" value="1000" units="mmol_per_gDW_per_hr" constant="true"/>
      <parameter id="FLUX_VALUE" value="0" units="mmol_per_gDW_per_hr" constant="true"/>
```

```
      <parameter id="OBJECTIVE_COEFFICIENT" value="0" units="mmol_per_gDW_per_hr" constant="true"/>
    </listOfParameters>
  </kineticLaw>
</reaction>
<reaction id="R_R756" name="Sulfite transport" reversible="true">
  <notes>
    <body xmlns="http://www.w3.org/1999/xhtml">
      <p>GENE_ASSOCIATION: </p>
      <p>SUBSYSTEM: Transport, Extracellular</p>
      <p>EC Number: </p>
      <p>Confidence Level: 1</p>
      <p>AUTHORS: Transport</p>
      <p/>
    </body>
  </notes>
  <listOfReactants>
    <speciesReference species="M_SO3_e" stoichiometry="1"/>
  </listOfReactants>
  <listOfProducts>
    <speciesReference species="M_SO3_c" stoichiometry="1"/>
  </listOfProducts>
  <kineticLaw>
    <math xmlns="http://www.w3.org/1998/Math/MathML">
      <ci> FLUX_VALUE </ci>
    </math>
    <listOfParameters>
      <parameter id="LOWER_BOUND" value="-1000" units="mmol_per_gDW_per_hr" constant="true"/>
      <parameter id="UPPER_BOUND" value="1000" units="mmol_per_gDW_per_hr" constant="true"/>
      <parameter id="FLUX_VALUE" value="0" units="mmol_per_gDW_per_hr" constant="true"/>
```

```xml
<parameter id="OBJECTIVE_COEFFICIENT" value="0" units="mmol_per_gDW_per_hr" constant="true"/>
</listOfParameters>
</kineticLaw>
</reaction>
<reaction id="R_R757" name="Succinate transport via proton symport" reversible="true">
<notes>
<body xmlns="http://www.w3.org/1999/xhtml">
<p>GENE_ASSOCIATION: TT_P0033 </p>
<p>SUBSYSTEM: Transport, Extracellular</p>
<p>EC Number: </p>
<p>Confidence Level: 1</p>
<p>AUTHORS: Transport</p>
<p/>
</body>
</notes>
<listOfReactants>
<speciesReference species="M_H_e" stoichiometry="2"/>
<speciesReference species="M_SUCC_e" stoichiometry="1"/>
</listOfReactants>
<listOfProducts>
<speciesReference species="M_H_c" stoichiometry="2"/>
<speciesReference species="M_SUCC_c" stoichiometry="1"/>
</listOfProducts>
<kineticLaw>
<math xmlns="http://www.w3.org/1998/Math/MathML">
<ci> FLUX_VALUE </ci>
</math>
<listOfParameters>
<parameter id="LOWER_BOUND" value="-1000" units="mmol_per_gDW_per_hr" constant="true"/>
```

```
<parameter id="UPPER_BOUND" value="1000" units="mmol_per_gDW_per_hr" constant="true"/>
<parameter id="FLUX_VALUE" value="0" units="mmol_per_gDW_per_hr" constant="true"/>
<parameter id="OBJECTIVE_COEFFICIENT" value="0" units="mmol_per_gDW_per_hr" constant="true"/>
</listOfParameters>
</kineticLaw>
</reaction>
<reaction id="R_R758" name="Sucrose transport via ABC transporter" reversible="false">
<notes>
<body xmlns="http://www.w3.org/1999/xhtml">
<p>GENE_ASSOCIATION: (((TTC1287 or TTC1286) and TTC1288) or ((TTC1628 or TTC1629) and TTC1627) or ((TTC0612 or TTC0613) and TTC0615))</p>
<p>SUBSYSTEM: Transport, Extracellular</p>
<p>EC Number: </p>
<p>Confidence Level: 1</p>
<p>AUTHORS: Transport</p>
<p/>
</body>
</notes>
<listOfReactants>
<speciesReference species="M_ATP_c" stoichiometry="1"/>
<speciesReference species="M_H2O_c" stoichiometry="1"/>
<speciesReference species="M_SUC_e" stoichiometry="1"/>
</listOfReactants>
<listOfProducts>
<speciesReference species="M_ADP_c" stoichiometry="1"/>
<speciesReference species="M_Pi_c" stoichiometry="1"/>
<speciesReference species="M_H_c" stoichiometry="1"/>
<speciesReference species="M_SUC_c" stoichiometry="1"/>
</listOfProducts>
```

```
<kineticLaw>
<math xmlns="http://www.w3.org/1998/Math/MathML">
<ci> FLUX_VALUE </ci>
</math>
<listOfParameters>
<parameter id="LOWER_BOUND" value="0" units="mmol_per_gDW_per_hr" constant="true"/>
<parameter id="UPPER_BOUND" value="1000" units="mmol_per_gDW_per_hr" constant="true"/>
<parameter id="FLUX_VALUE" value="0" units="mmol_per_gDW_per_hr" constant="true"/>
<parameter id="OBJECTIVE_COEFFICIENT" value="0" units="mmol_per_gDW_per_hr" constant="true"/>
</listOfParameters>
</kineticLaw>
</reaction>
<reaction id="R_R759" name="L-Alanine transport via ABC system" reversible="false">
<notes>
<body xmlns="http://www.w3.org/1999/xhtml">
<p>GENE_ASSOCIATION: ((TTC0219 or TTC0334 or TTC0970 or TTC0600 or TTC0083) and (TTC0218 or TTC0335 or TTC0969 or TTC0078)) and ((TTC0220 or TTC0337 or TTC0598 or TTC0967 or TTC0080) and (TTC216 or TTC0968 or TTC0081))</p>
<p>SUBSYSTEM: Transport, Extracellular</p>
<p>EC Number: </p>
<p>Confidence Level: 1</p>
<p>AUTHORS: Transport</p>
<p/>
</body>
</notes>
<listOfReactants>
<speciesReference species="M_ATP_c" stoichiometry="1"/>
<speciesReference species="M_H2O_c" stoichiometry="1"/>
<speciesReference species="M_L_ALA_e" stoichiometry="1"/>
</listOfReactants>
```

```
<listOfProducts>
  <speciesReference species="M_L_ALA_c" stoichiometry="1"/>
  <speciesReference species="M_ADP_c" stoichiometry="1"/>
  <speciesReference species="M_Pi_c" stoichiometry="1"/>
</listOfProducts>
<kineticLaw>
  <math xmlns="http://www.w3.org/1998/Math/MathML">
    <ci> FLUX_VALUE </ci>
  </math>
  <listOfParameters>
    <parameter id="LOWER_BOUND" value="0" units="mmol_per_gDW_per_hr" constant="true"/>
    <parameter id="UPPER_BOUND" value="1000" units="mmol_per_gDW_per_hr" constant="true"/>
    <parameter id="FLUX_VALUE" value="0" units="mmol_per_gDW_per_hr" constant="true"/>
    <parameter id="OBJECTIVE_COEFFICIENT" value="0" units="mmol_per_gDW_per_hr" constant="true"/>
  </listOfParameters>
</kineticLaw>
</reaction>
<reaction id="R_R760" name="L-Aspartate transport" reversible="true">
<notes>
  <body xmlns="http://www.w3.org/1999/xhtml">
    <p>GENE_ASSOCIATION: </p>
    <p>SUBSYSTEM: Transport, Extracellular</p>
    <p>EC Number: </p>
    <p>Confidence Level: 1</p>
    <p>AUTHORS: Transport</p>
    <p/>
  </body>
</notes>
<listOfReactants>
```

```xml
<speciesReference species="M_L_ASP_e" stoichiometry="1"/>
</listOfReactants>
<listOfProducts>
<speciesReference species="M_L_ASP_c" stoichiometry="1"/>
</listOfProducts>
<kineticLaw>
<math xmlns="http://www.w3.org/1998/Math/MathML">
<ci> FLUX_VALUE </ci>
</math>
<listOfParameters>
<parameter id="LOWER_BOUND" value="-1000" units="mmol_per_gDW_per_hr" constant="true"/>
<parameter id="UPPER_BOUND" value="1000" units="mmol_per_gDW_per_hr" constant="true"/>
<parameter id="FLUX_VALUE" value="0" units="mmol_per_gDW_per_hr" constant="true"/>
<parameter id="OBJECTIVE_COEFFICIENT" value="0" units="mmol_per_gDW_per_hr" constant="true"/>
</listOfParameters>
</kineticLaw>
</reaction>
<reaction id="R_R761" name="L-Cysteine transport" reversible="true">
<notes>
<body xmlns="http://www.w3.org/1999/xhtml">
<p>GENE_ASSOCIATION: </p>
<p>SUBSYSTEM: Transport, Extracellular</p>
<p>EC Number: </p>
<p>Confidence Level: 1</p>
<p>AUTHORS: Transport</p>
<p/>
</body>
</notes>
<listOfReactants>
```

```
<speciesReference species="M_L_CYS_e" stoichiometry="1"/>
</listOfReactants>
<listOfProducts>
<speciesReference species="M_L_CYS_c" stoichiometry="1"/>
</listOfProducts>
<kineticLaw>
<math xmlns="http://www.w3.org/1998/Math/MathML">
<ci> FLUX_VALUE </ci>
</math>
<listOfParameters>
<parameter id="LOWER_BOUND" value="-1000" units="mmol_per_gDW_per_hr" constant="true"/>
<parameter id="UPPER_BOUND" value="1000" units="mmol_per_gDW_per_hr" constant="true"/>
<parameter id="FLUX_VALUE" value="0" units="mmol_per_gDW_per_hr" constant="true"/>
<parameter id="OBJECTIVE_COEFFICIENT" value="0" units="mmol_per_gDW_per_hr" constant="true"/>
</listOfParameters>
</kineticLaw>
</reaction>
<reaction id="R_R762" name="L-Glutamate transport" reversible="true">
<notes>
<body xmlns="http://www.w3.org/1999/xhtml">
<p>GENE_ASSOCIATION: </p>
<p>SUBSYSTEM: Transport, Extracellular</p>
<p>EC Number: </p>
<p>Confidence Level: 1</p>
<p>AUTHORS: Transport</p>
<p/>
</body>
</notes>
<listOfReactants>
```

```
    <speciesReference species="M_L_GLU_e" stoichiometry="1"/>
  </listOfReactants>
  <listOfProducts>
    <speciesReference species="M_L_GLU_c" stoichiometry="1"/>
  </listOfProducts>
  <kineticLaw>
    <math xmlns="http://www.w3.org/1998/Math/MathML">
      <ci> FLUX_VALUE </ci>
    </math>
    <listOfParameters>
      <parameter id="LOWER_BOUND" value="-1000" units="mmol_per_gDW_per_hr" constant="true"/>
      <parameter id="UPPER_BOUND" value="1000" units="mmol_per_gDW_per_hr" constant="true"/>
      <parameter id="FLUX_VALUE" value="0" units="mmol_per_gDW_per_hr" constant="true"/>
      <parameter id="OBJECTIVE_COEFFICIENT" value="0" units="mmol_per_gDW_per_hr" constant="true"/>
    </listOfParameters>
  </kineticLaw>
</reaction>
<reaction id="R_R763" name="L-Methionine transport" reversible="true">
  <notes>
    <body xmlns="http://www.w3.org/1999/xhtml">
      <p>GENE_ASSOCIATION: </p>
      <p>SUBSYSTEM: Transport, Extracellular</p>
      <p>EC Number: </p>
      <p>Confidence Level: 1</p>
      <p>AUTHORS: Transport</p>
      <p/>
    </body>
  </notes>
  <listOfReactants>
```

```
    <speciesReference species="M_L_MET_e" stoichiometry="1"/>
  </listOfReactants>
  <listOfProducts>
    <speciesReference species="M_L_MET_c" stoichiometry="1"/>
  </listOfProducts>
  <kineticLaw>
    <math xmlns="http://www.w3.org/1998/Math/MathML">
      <ci> FLUX_VALUE </ci>
    </math>
    <listOfParameters>
      <parameter id="LOWER_BOUND" value="-1000" units="mmol_per_gDW_per_hr" constant="true"/>
      <parameter id="UPPER_BOUND" value="1000" units="mmol_per_gDW_per_hr" constant="true"/>
      <parameter id="FLUX_VALUE" value="0" units="mmol_per_gDW_per_hr" constant="true"/>
      <parameter id="OBJECTIVE_COEFFICIENT" value="0" units="mmol_per_gDW_per_hr" constant="true"/>
    </listOfParameters>
  </kineticLaw>
</reaction>
<reaction id="R_R764" name="L-Threonine transport" reversible="true">
  <notes>
    <body xmlns="http://www.w3.org/1999/xhtml">
      <p>GENE_ASSOCIATION: </p>
      <p>SUBSYSTEM: Transport, Extracellular</p>
      <p>EC Number: </p>
      <p>Confidence Level: 1</p>
      <p>AUTHORS: Transport</p>
      <p/>
    </body>
  </notes>
  <listOfReactants>
```

```xml
<speciesReference species="M_L_THR_e" stoichiometry="1"/>
</listOfReactants>
<listOfProducts>
<speciesReference species="M_L_THR_c" stoichiometry="1"/>
</listOfProducts>
<kineticLaw>
<math xmlns="http://www.w3.org/1998/Math/MathML">
<ci> FLUX_VALUE </ci>
</math>
<listOfParameters>
<parameter id="LOWER_BOUND" value="-1000" units="mmol_per_gDW_per_hr" constant="true"/>
<parameter id="UPPER_BOUND" value="1000" units="mmol_per_gDW_per_hr" constant="true"/>
<parameter id="FLUX_VALUE" value="0" units="mmol_per_gDW_per_hr" constant="true"/>
<parameter id="OBJECTIVE_COEFFICIENT" value="0" units="mmol_per_gDW_per_hr" constant="true"/>
</listOfParameters>
</kineticLaw>
</reaction>
<reaction id="R_R765" name="L-Leucine transport via ABC system" reversible="false">
<notes>
<body xmlns="http://www.w3.org/1999/xhtml">
<p>GENE_ASSOCIATION: ((TTC0219 or TTC0334 or TTC0970 or TTC0600 or TTC0083) and (TTC0218 or TTC0335 or TTC0969 or TTC0078)) and ((TTC0220 or TTC0337 or TTC0598 or TTC0967 or TTC0080) and (TTC216 or TTC0968 or TTC0081))</p>
<p>SUBSYSTEM: Transport, Extracellular</p>
<p>EC Number: </p>
<p>Confidence Level: 1</p>
<p>AUTHORS: Transport</p>
<p/>
</body>
</notes>
```

```
<listOfReactants>
  <speciesReference species="M_ATP_c" stoichiometry="1"/>
  <speciesReference species="M_H2O_c" stoichiometry="1"/>
  <speciesReference species="M_L_LEU_e" stoichiometry="1"/>
</listOfReactants>
<listOfProducts>
  <speciesReference species="M_L_LEU_c" stoichiometry="1"/>
  <speciesReference species="M_ADP_c" stoichiometry="1"/>
  <speciesReference species="M_Pi_c" stoichiometry="1"/>
</listOfProducts>
<kineticLaw>
  <math xmlns="http://www.w3.org/1998/Math/MathML">
    <ci> FLUX_VALUE </ci>
  </math>
  <listOfParameters>
    <parameter id="LOWER_BOUND" value="0" units="mmol_per_gDW_per_hr" constant="true"/>
    <parameter id="UPPER_BOUND" value="1000" units="mmol_per_gDW_per_hr" constant="true"/>
    <parameter id="FLUX_VALUE" value="0" units="mmol_per_gDW_per_hr" constant="true"/>
    <parameter id="OBJECTIVE_COEFFICIENT" value="0" units="mmol_per_gDW_per_hr" constant="true"/>
  </listOfParameters>
</kineticLaw>
</reaction>
<reaction id="R_R766" name="L-Tryptophan transport" reversible="true">
<notes>
  <body xmlns="http://www.w3.org/1999/xhtml">
    <p>GENE_ASSOCIATION: </p>
    <p>SUBSYSTEM: Transport, Extracellular</p>
    <p>EC Number: </p>
    <p>Confidence Level: 1</p>
```

```xml
<p>AUTHORS: Transport</p>
<p/>
</body>
</notes>
<listOfReactants>
<speciesReference species="M_L_TRP_e" stoichiometry="1"/>
</listOfReactants>
<listOfProducts>
<speciesReference species="M_L_TRP_c" stoichiometry="1"/>
</listOfProducts>
<kineticLaw>
<math xmlns="http://www.w3.org/1998/Math/MathML">
<ci> FLUX_VALUE </ci>
</math>
<listOfParameters>
<parameter id="LOWER_BOUND" value="-1000" units="mmol_per_gDW_per_hr" constant="true"/>
<parameter id="UPPER_BOUND" value="1000" units="mmol_per_gDW_per_hr" constant="true"/>
<parameter id="FLUX_VALUE" value="0" units="mmol_per_gDW_per_hr" constant="true"/>
<parameter id="OBJECTIVE_COEFFICIENT" value="0" units="mmol_per_gDW_per_hr" constant="true"/>
</listOfParameters>
</kineticLaw>
</reaction>
<reaction id="R_R767" name="L-Tyrosine transport" reversible="true">
<notes>
<body xmlns="http://www.w3.org/1999/xhtml">
<p>GENE_ASSOCIATION: </p>
<p>SUBSYSTEM: Transport, Extracellular</p>
<p>EC Number: </p>
<p>Confidence Level: 1</p>
```

```xml
<p>AUTHORS: Transport</p>

<p/>

</body>

</notes>

<listOfReactants>

<speciesReference species="M_L_TYR_e" stoichiometry="1"/>

</listOfReactants>

<listOfProducts>

<speciesReference species="M_L_TYR_c" stoichiometry="1"/>

</listOfProducts>

<kineticLaw>

<math xmlns="http://www.w3.org/1998/Math/MathML">

<ci> FLUX_VALUE </ci>

</math>

<listOfParameters>

<parameter id="LOWER_BOUND" value="-1000" units="mmol_per_gDW_per_hr" constant="true"/>

<parameter id="UPPER_BOUND" value="1000" units="mmol_per_gDW_per_hr" constant="true"/>

<parameter id="FLUX_VALUE" value="0" units="mmol_per_gDW_per_hr" constant="true"/>

<parameter id="OBJECTIVE_COEFFICIENT" value="0" units="mmol_per_gDW_per_hr" constant="true"/>

</listOfParameters>

</kineticLaw>

</reaction>

<reaction id="R_R768" name="L-Valine transport via ABC system" reversible="false">

<notes>

<body xmlns="http://www.w3.org/1999/xhtml">

<p>GENE_ASSOCIATION: ((TTC0219 or TTC0334 or TTC0970 or TTC0600 or TTC0083) and (TTC0218 or TTC0335 or TTC0969 or TTC0078)) and ((TTC0220 or TTC0337 or TTC0598 or TTC0967 or TTC0080) and (TTC216 or TTC0968 or TTC0081))</p>

<p>SUBSYSTEM: Transport, Extracellular</p>

<p>EC Number: </p>
```

```
      <p>Confidence Level: 1</p>
      <p>AUTHORS: Transport</p>
      <p/>
    </body>
  </notes>
  <listOfReactants>
    <speciesReference species="M_ATP_c" stoichiometry="1"/>
    <speciesReference species="M_H2O_c" stoichiometry="1"/>
    <speciesReference species="M_L_VAL_e" stoichiometry="1"/>
  </listOfReactants>
  <listOfProducts>
    <speciesReference species="M_L_VAL_c" stoichiometry="1"/>
    <speciesReference species="M_ADP_c" stoichiometry="1"/>
    <speciesReference species="M_Pi_c" stoichiometry="1"/>
  </listOfProducts>
  <kineticLaw>
    <math xmlns="http://www.w3.org/1998/Math/MathML">
      <ci> FLUX_VALUE </ci>
    </math>
    <listOfParameters>
      <parameter id="LOWER_BOUND" value="0" units="mmol_per_gDW_per_hr" constant="true"/>
      <parameter id="UPPER_BOUND" value="1000" units="mmol_per_gDW_per_hr" constant="true"/>
      <parameter id="FLUX_VALUE" value="0" units="mmol_per_gDW_per_hr" constant="true"/>
      <parameter id="OBJECTIVE_COEFFICIENT" value="0" units="mmol_per_gDW_per_hr" constant="true"/>
    </listOfParameters>
  </kineticLaw>
</reaction>
<reaction id="R_R769" name="Bicarbonate transport" reversible="true">
  <notes>
```

```
    <body xmlns="http://www.w3.org/1999/xhtml">
      <p>GENE_ASSOCIATION: </p>
      <p>SUBSYSTEM: Transport, Extracellular</p>
      <p>EC Number: </p>
      <p>Confidence Level: 1</p>
      <p>AUTHORS: Transport</p>
      <p/>
    </body>
  </notes>
  <listOfReactants>
    <speciesReference species="M_HCO3_e" stoichiometry="1"/>
  </listOfReactants>
  <listOfProducts>
    <speciesReference species="M_HCO3_c" stoichiometry="1"/>
  </listOfProducts>
  <kineticLaw>
    <math xmlns="http://www.w3.org/1998/Math/MathML">
      <ci> FLUX_VALUE </ci>
    </math>
    <listOfParameters>
      <parameter id="LOWER_BOUND" value="-1000" units="mmol_per_gDW_per_hr" constant="true"/>
      <parameter id="UPPER_BOUND" value="1000" units="mmol_per_gDW_per_hr" constant="true"/>
      <parameter id="FLUX_VALUE" value="0" units="mmol_per_gDW_per_hr" constant="true"/>
      <parameter id="OBJECTIVE_COEFFICIENT" value="0" units="mmol_per_gDW_per_hr" constant="true"/>
    </listOfParameters>
  </kineticLaw>
</reaction>
<reaction id="R_R770" name="L-Lactate transport" reversible="true">
  <notes>
```

```
    <body xmlns="http://www.w3.org/1999/xhtml">
      <p>GENE_ASSOCIATION: </p>
      <p>SUBSYSTEM: Transport, Extracellular</p>
      <p>EC Number: </p>
      <p>Confidence Level: 1</p>
      <p>AUTHORS: Transport</p>
      <p/>
    </body>
  </notes>
  <listOfReactants>
    <speciesReference species="M_LAC_e" stoichiometry="1"/>
  </listOfReactants>
  <listOfProducts>
    <speciesReference species="M_LAC_c" stoichiometry="1"/>
  </listOfProducts>
  <kineticLaw>
    <math xmlns="http://www.w3.org/1998/Math/MathML">
      <ci> FLUX_VALUE </ci>
    </math>
    <listOfParameters>
      <parameter id="LOWER_BOUND" value="-1000" units="mmol_per_gDW_per_hr" constant="true"/>
      <parameter id="UPPER_BOUND" value="1000" units="mmol_per_gDW_per_hr" constant="true"/>
      <parameter id="FLUX_VALUE" value="0" units="mmol_per_gDW_per_hr" constant="true"/>
      <parameter id="OBJECTIVE_COEFFICIENT" value="0" units="mmol_per_gDW_per_hr" constant="true"/>
    </listOfParameters>
  </kineticLaw>
</reaction>
<reaction id="R_R771" name="Ammonia transport" reversible="true">
  <notes>
```

```xml
<body xmlns="http://www.w3.org/1999/xhtml">
<p>GENE_ASSOCIATION: </p>
<p>SUBSYSTEM: Transport, Extracellular</p>
<p>EC Number: </p>
<p>Confidence Level: 1</p>
<p>AUTHORS: Transport</p>
<p/>
</body>
</notes>
<listOfReactants>
<speciesReference species="M_NH4_c" stoichiometry="1"/>
</listOfReactants>
<listOfProducts>
<speciesReference species="M_NH4_e" stoichiometry="1"/>
</listOfProducts>
<kineticLaw>
<math xmlns="http://www.w3.org/1998/Math/MathML">
<ci> FLUX_VALUE </ci>
</math>
<listOfParameters>
<parameter id="LOWER_BOUND" value="-1000" units="mmol_per_gDW_per_hr" constant="true"/>
<parameter id="UPPER_BOUND" value="1000" units="mmol_per_gDW_per_hr" constant="true"/>
<parameter id="FLUX_VALUE" value="0" units="mmol_per_gDW_per_hr" constant="true"/>
<parameter id="OBJECTIVE_COEFFICIENT" value="0" units="mmol_per_gDW_per_hr" constant="true"/>
</listOfParameters>
</kineticLaw>
</reaction>
<reaction id="R_R772" name="Trehalose transport via ATP transporter" reversible="false">
<notes>
```

```
<body xmlns="http://www.w3.org/1999/xhtml">
  <p>GENE_ASSOCIATION: (((TTC1287 or TTC1286) and TTC1288) or ((TTC1628 or TTC1629) and TTC1627) or ((TTC0612 or TTC0613) and TTC0615))</p>
  <p>SUBSYSTEM: Transport, Extracellular</p>
  <p>EC Number: </p>
  <p>Confidence Level: 1</p>
  <p>AUTHORS: Transport</p>
  <p/>
</body>
</notes>
<listOfReactants>
  <speciesReference species="M_ATP_c" stoichiometry="1"/>
  <speciesReference species="M_H2O_c" stoichiometry="1"/>
  <speciesReference species="M_TRE_e" stoichiometry="1"/>
</listOfReactants>
<listOfProducts>
  <speciesReference species="M_ADP_c" stoichiometry="1"/>
  <speciesReference species="M_Pi_c" stoichiometry="1"/>
  <speciesReference species="M_H_c" stoichiometry="1"/>
  <speciesReference species="M_TRE_c" stoichiometry="1"/>
</listOfProducts>
<kineticLaw>
  <math xmlns="http://www.w3.org/1998/Math/MathML">
    <ci> FLUX_VALUE </ci>
  </math>
  <listOfParameters>
    <parameter id="LOWER_BOUND" value="0" units="mmol_per_gDW_per_hr" constant="true"/>
    <parameter id="UPPER_BOUND" value="1000" units="mmol_per_gDW_per_hr" constant="true"/>
    <parameter id="FLUX_VALUE" value="0" units="mmol_per_gDW_per_hr" constant="true"/>
```

```
      <parameter id="OBJECTIVE_COEFFICIENT" value="0" units="mmol_per_gDW_per_hr" constant="true"/>
    </listOfParameters>
  </kineticLaw>
</reaction>
<reaction id="R_R773" name="Magnesium transport via diffusion" reversible="false">
  <notes>
    <body xmlns="http://www.w3.org/1999/xhtml">
      <p>GENE_ASSOCIATION: (TTC0695 or TTC1342)</p>
      <p>SUBSYSTEM: Transport, Extracellular</p>
      <p>EC Number: </p>
      <p>Confidence Level: 1</p>
      <p>AUTHORS: Transport</p>
      <p/>
    </body>
  </notes>
  <listOfReactants>
    <speciesReference species="M_Mg_e" stoichiometry="1"/>
  </listOfReactants>
  <listOfProducts>
    <speciesReference species="M_Mg_c" stoichiometry="1"/>
  </listOfProducts>
  <kineticLaw>
    <math xmlns="http://www.w3.org/1998/Math/MathML">
      <ci> FLUX_VALUE </ci>
    </math>
    <listOfParameters>
      <parameter id="LOWER_BOUND" value="0" units="mmol_per_gDW_per_hr" constant="true"/>
      <parameter id="UPPER_BOUND" value="1000" units="mmol_per_gDW_per_hr" constant="true"/>
      <parameter id="FLUX_VALUE" value="0" units="mmol_per_gDW_per_hr" constant="true"/>
```

```
      <parameter id="OBJECTIVE_COEFFICIENT" value="0" units="mmol_per_gDW_per_hr" constant="true"/>
    </listOfParameters>
  </kineticLaw>
</reaction>
<reaction id="R_R774" name="Heme export via diffusion" reversible="false">
  <notes>
    <body xmlns="http://www.w3.org/1999/xhtml">
      <p>GENE_ASSOCIATION: (TTC1401 and TTC1402)</p>
      <p>SUBSYSTEM: Transport, Extracellular</p>
      <p>EC Number: </p>
      <p>Confidence Level: 1</p>
      <p>AUTHORS: Transport</p>
      <p/>
    </body>
  </notes>
  <listOfReactants>
    <speciesReference species="M_HEME_c" stoichiometry="1"/>
  </listOfReactants>
  <listOfProducts>
    <speciesReference species="M_HEME_e" stoichiometry="1"/>
  </listOfProducts>
  <kineticLaw>
    <math xmlns="http://www.w3.org/1998/Math/MathML">
      <ci> FLUX_VALUE </ci>
    </math>
    <listOfParameters>
      <parameter id="LOWER_BOUND" value="0" units="mmol_per_gDW_per_hr" constant="true"/>
      <parameter id="UPPER_BOUND" value="1000" units="mmol_per_gDW_per_hr" constant="true"/>
      <parameter id="FLUX_VALUE" value="0" units="mmol_per_gDW_per_hr" constant="true"/>
```

```xml
      <parameter id="OBJECTIVE_COEFFICIENT" value="0" units="mmol_per_gDW_per_hr" constant="true"/>
    </listOfParameters>
  </kineticLaw>
</reaction>
<reaction id="R_R775" name="D-Gluconate transport" reversible="false">
  <notes>
    <body xmlns="http://www.w3.org/1999/xhtml">
      <p>GENE_ASSOCIATION: </p>
      <p>SUBSYSTEM: Transport, Extracellular</p>
      <p>EC Number: </p>
      <p>Confidence Level: 1</p>
      <p>AUTHORS: Transport</p>
      <p/>
    </body>
  </notes>
  <listOfReactants>
    <speciesReference species="M_GLCN_e" stoichiometry="1"/>
  </listOfReactants>
  <listOfProducts>
    <speciesReference species="M_GLCN_c" stoichiometry="1"/>
  </listOfProducts>
  <kineticLaw>
    <math xmlns="http://www.w3.org/1998/Math/MathML">
      <ci> FLUX_VALUE </ci>
    </math>
    <listOfParameters>
      <parameter id="LOWER_BOUND" value="0" units="mmol_per_gDW_per_hr" constant="true"/>
      <parameter id="UPPER_BOUND" value="1000" units="mmol_per_gDW_per_hr" constant="true"/>
      <parameter id="FLUX_VALUE" value="0" units="mmol_per_gDW_per_hr" constant="true"/>
```

<parameter id="OBJECTIVE_COEFFICIENT" value="0" units="mmol_per_gDW_per_hr" constant="true"/>

</listOfParameters>

</kineticLaw>

</reaction>

<reaction id="R_R776" name="Cellobiose transport via ABC system" reversible="false">

<notes>

<body xmlns="http://www.w3.org/1999/xhtml">

<p>GENE_ASSOCIATION: (((TTC1287 or TTC1286) and TTC1288) or ((TTC1628 or TTC1629) and TTC1627) or ((TTC0612 or TTC0613) and TTC0615))</p>

<p>SUBSYSTEM: Transport, Extracellular</p>

<p>EC Number: </p>

<p>Confidence Level: 1</p>

<p>AUTHORS: Transport</p>

<p/>

</body>

</notes>

<listOfReactants>

<speciesReference species="M_ATP_c" stoichiometry="1"/>

<speciesReference species="M_H2O_c" stoichiometry="1"/>

<speciesReference species="M_CELBS_e" stoichiometry="1"/>

</listOfReactants>

<listOfProducts>

<speciesReference species="M_ADP_c" stoichiometry="1"/>

<speciesReference species="M_Pi_c" stoichiometry="1"/>

<speciesReference species="M_H_c" stoichiometry="1"/>

<speciesReference species="M_CELBS_c" stoichiometry="1"/>

</listOfProducts>

<kineticLaw>

<math xmlns="http://www.w3.org/1998/Math/MathML">

```xml
<ci> FLUX_VALUE </ci>
</math>
<listOfParameters>
<parameter id="LOWER_BOUND" value="0" units="mmol_per_gDW_per_hr" constant="true"/>
<parameter id="UPPER_BOUND" value="1000" units="mmol_per_gDW_per_hr" constant="true"/>
<parameter id="FLUX_VALUE" value="0" units="mmol_per_gDW_per_hr" constant="true"/>
<parameter id="OBJECTIVE_COEFFICIENT" value="0" units="mmol_per_gDW_per_hr" constant="true"/>
</listOfParameters>
</kineticLaw>
</reaction>
<reaction id="R_R777" name="Palatinose transport via ABC system" reversible="false">
<notes>
<body xmlns="http://www.w3.org/1999/xhtml">
<p>GENE_ASSOCIATION: (((TTC1287 or TTC1286) and TTC1288) or ((TTC1628 or TTC1629) and TTC1627) or ((TTC0612 or TTC0613) and TTC0615))</p>
<p>SUBSYSTEM: Transport, Extracellular</p>
<p>EC Number: </p>
<p>Confidence Level: 1</p>
<p>AUTHORS: Transport</p>
<p/>
</body>
</notes>
<listOfReactants>
<speciesReference species="M_ATP_c" stoichiometry="1"/>
<speciesReference species="M_H2O_c" stoichiometry="1"/>
<speciesReference species="M_PALT_e" stoichiometry="1"/>
</listOfReactants>
<listOfProducts>
<speciesReference species="M_ADP_c" stoichiometry="1"/>
```

```
    <speciesReference species="M_Pi_c" stoichiometry="1"/>
    <speciesReference species="M_H_c" stoichiometry="1"/>
    <speciesReference species="M_PALT_c" stoichiometry="1"/>
  </listOfProducts>
  <kineticLaw>
    <math xmlns="http://www.w3.org/1998/Math/MathML">
      <ci> FLUX_VALUE </ci>
    </math>
    <listOfParameters>
      <parameter id="LOWER_BOUND" value="0" units="mmol_per_gDW_per_hr" constant="true"/>
      <parameter id="UPPER_BOUND" value="1000" units="mmol_per_gDW_per_hr" constant="true"/>
      <parameter id="FLUX_VALUE" value="0" units="mmol_per_gDW_per_hr" constant="true"/>
      <parameter id="OBJECTIVE_COEFFICIENT" value="0" units="mmol_per_gDW_per_hr" constant="true"/>
    </listOfParameters>
  </kineticLaw>
</reaction>
<reaction id="R_R778" name="D-Galactose transport via ABC system" reversible="false">
  <notes>
    <body xmlns="http://www.w3.org/1999/xhtml">
      <p>GENE_ASSOCIATION: ((TTC0326 or TTC0327) and TTC0328)</p>
      <p>SUBSYSTEM: Transport, Extracellular</p>
      <p>EC Number: </p>
      <p>Confidence Level: 1</p>
      <p>AUTHORS: Transport</p>
      <p/>
    </body>
  </notes>
  <listOfReactants>
    <speciesReference species="M_ATP_c" stoichiometry="1"/>
```

```xml
<speciesReference species="M_H2O_c" stoichiometry="1"/>

<speciesReference species="M_D_GAL_e" stoichiometry="1"/>

</listOfReactants>

<listOfProducts>

<speciesReference species="M_ADP_c" stoichiometry="1"/>

<speciesReference species="M_Pi_c" stoichiometry="1"/>

<speciesReference species="M_H_c" stoichiometry="1"/>

<speciesReference species="M_D_GAL_c" stoichiometry="1"/>

</listOfProducts>

<kineticLaw>

<math xmlns="http://www.w3.org/1998/Math/MathML">

<ci> FLUX_VALUE </ci>

</math>

<listOfParameters>

<parameter id="LOWER_BOUND" value="0" units="mmol_per_gDW_per_hr" constant="true"/>

<parameter id="UPPER_BOUND" value="1000" units="mmol_per_gDW_per_hr" constant="true"/>

<parameter id="FLUX_VALUE" value="0" units="mmol_per_gDW_per_hr" constant="true"/>

<parameter id="OBJECTIVE_COEFFICIENT" value="0" units="mmol_per_gDW_per_hr" constant="true"/>

</listOfParameters>

</kineticLaw>

</reaction>

<reaction id="R_R779" name="Latose transport via ABC system" reversible="false">

<notes>

<body xmlns="http://www.w3.org/1999/xhtml">

<p>GENE_ASSOCIATION: (((TTC1287 or TTC1286) and TTC1288) or ((TTC1628 or TTC1629) and TTC1627) or ((TTC0612 or TTC0613) and TTC0615))</p>

<p>SUBSYSTEM: Transport, Extracellular</p>

<p>EC Number: </p>

<p>Confidence Level: 1</p>
```

```xml
      <p>AUTHORS: Transport</p>

      <p/>

    </body>

  </notes>

  <listOfReactants>

    <speciesReference species="M_ATP_c" stoichiometry="1"/>

    <speciesReference species="M_H2O_c" stoichiometry="1"/>

    <speciesReference species="M_LACTS_e" stoichiometry="1"/>

  </listOfReactants>

  <listOfProducts>

    <speciesReference species="M_ADP_c" stoichiometry="1"/>

    <speciesReference species="M_Pi_c" stoichiometry="1"/>

    <speciesReference species="M_H_c" stoichiometry="1"/>

    <speciesReference species="M_LACTS_c" stoichiometry="1"/>

  </listOfProducts>

  <kineticLaw>

    <math xmlns="http://www.w3.org/1998/Math/MathML">

      <ci> FLUX_VALUE </ci>

    </math>

    <listOfParameters>

      <parameter id="LOWER_BOUND" value="0" units="mmol_per_gDW_per_hr" constant="true"/>

      <parameter id="UPPER_BOUND" value="1000" units="mmol_per_gDW_per_hr" constant="true"/>

      <parameter id="FLUX_VALUE" value="0" units="mmol_per_gDW_per_hr" constant="true"/>

      <parameter id="OBJECTIVE_COEFFICIENT" value="0" units="mmol_per_gDW_per_hr" constant="true"/>

    </listOfParameters>

  </kineticLaw>

</reaction>

<reaction id="R_R780" name="Stachyose transport via ABC system" reversible="false">

  <notes>
```

```xml
<body xmlns="http://www.w3.org/1999/xhtml">

<p>GENE_ASSOCIATION: (((TTC1287 or TTC1286) and TTC1288) or ((TTC1628 or TTC1629) and TTC1627) or ((TTC0612 or TTC0613) and TTC0615))</p>

<p>SUBSYSTEM: Transport, Extracellular</p>

<p>EC Number: </p>

<p>Confidence Level: 1</p>

<p>AUTHORS: Transport</p>

<p/>

</body>

</notes>

<listOfReactants>

<speciesReference species="M_ATP_c" stoichiometry="1"/>

<speciesReference species="M_H2O_c" stoichiometry="1"/>

<speciesReference species="M_STCYS_e" stoichiometry="1"/>

</listOfReactants>

<listOfProducts>

<speciesReference species="M_ADP_c" stoichiometry="1"/>

<speciesReference species="M_Pi_c" stoichiometry="1"/>

<speciesReference species="M_H_c" stoichiometry="1"/>

<speciesReference species="M_STCYS_c" stoichiometry="1"/>

</listOfProducts>

<kineticLaw>

<math xmlns="http://www.w3.org/1998/Math/MathML">

<ci> FLUX_VALUE </ci>

</math>

<listOfParameters>

<parameter id="LOWER_BOUND" value="0" units="mmol_per_gDW_per_hr" constant="true"/>

<parameter id="UPPER_BOUND" value="1000" units="mmol_per_gDW_per_hr" constant="true"/>

<parameter id="FLUX_VALUE" value="0" units="mmol_per_gDW_per_hr" constant="true"/>
```

```
<parameter id="OBJECTIVE_COEFFICIENT" value="0" units="mmol_per_gDW_per_hr" constant="true"/>
</listOfParameters>
</kineticLaw>
</reaction>
<reaction id="R_R781" name="PHB transport" reversible="false">
<notes>
<body xmlns="http://www.w3.org/1999/xhtml">
<p>GENE_ASSOCIATION: </p>
<p>SUBSYSTEM: Transport, Extracellular</p>
<p>EC Number: </p>
<p>Confidence Level: 1</p>
<p>AUTHORS: Transport</p>
<p/>
</body>
</notes>
<listOfReactants>
<speciesReference species="M_PHB_c" stoichiometry="1"/>
</listOfReactants>
<listOfProducts>
<speciesReference species="M_PHB_e" stoichiometry="1"/>
</listOfProducts>
<kineticLaw>
<math xmlns="http://www.w3.org/1998/Math/MathML">
<ci> FLUX_VALUE </ci>
</math>
<listOfParameters>
<parameter id="LOWER_BOUND" value="0" units="mmol_per_gDW_per_hr" constant="true"/>
<parameter id="UPPER_BOUND" value="1000" units="mmol_per_gDW_per_hr" constant="true"/>
<parameter id="FLUX_VALUE" value="0" units="mmol_per_gDW_per_hr" constant="true"/>
```

```
      <parameter id="OBJECTIVE_COEFFICIENT" value="0" units="mmol_per_gDW_per_hr" constant="true"/>
    </listOfParameters>
  </kineticLaw>
</reaction>
<reaction id="R_R782" name="PHV transport" reversible="false">
  <notes>
    <body xmlns="http://www.w3.org/1999/xhtml">
      <p>GENE_ASSOCIATION: </p>
      <p>SUBSYSTEM: Transport, Extracellular</p>
      <p>EC Number: </p>
      <p>Confidence Level: 1</p>
      <p>AUTHORS: Transport</p>
      <p/>
    </body>
  </notes>
  <listOfReactants>
    <speciesReference species="M_PHV_c" stoichiometry="1"/>
  </listOfReactants>
  <listOfProducts>
    <speciesReference species="M_PHV_e" stoichiometry="1"/>
  </listOfProducts>
  <kineticLaw>
    <math xmlns="http://www.w3.org/1998/Math/MathML">
      <ci> FLUX_VALUE </ci>
    </math>
    <listOfParameters>
      <parameter id="LOWER_BOUND" value="0" units="mmol_per_gDW_per_hr" constant="true"/>
      <parameter id="UPPER_BOUND" value="1000" units="mmol_per_gDW_per_hr" constant="true"/>
      <parameter id="FLUX_VALUE" value="0" units="mmol_per_gDW_per_hr" constant="true"/>
```

```
      <parameter id="OBJECTIVE_COEFFICIENT" value="0" units="mmol_per_gDW_per_hr" constant="true"/>
    </listOfParameters>
  </kineticLaw>
</reaction>
<reaction id="R_R783" name="PHO transport" reversible="false">
  <notes>
    <body xmlns="http://www.w3.org/1999/xhtml">
      <p>GENE_ASSOCIATION: </p>
      <p>SUBSYSTEM: Transport, Extracellular</p>
      <p>EC Number: </p>
      <p>Confidence Level: 1</p>
      <p>AUTHORS: Transport</p>
      <p/>
    </body>
  </notes>
  <listOfReactants>
    <speciesReference species="M_PHO_c" stoichiometry="1"/>
  </listOfReactants>
  <listOfProducts>
    <speciesReference species="M_PHO_e" stoichiometry="1"/>
  </listOfProducts>
  <kineticLaw>
    <math xmlns="http://www.w3.org/1998/Math/MathML">
      <ci> FLUX_VALUE </ci>
    </math>
    <listOfParameters>
      <parameter id="LOWER_BOUND" value="0" units="mmol_per_gDW_per_hr" constant="true"/>
      <parameter id="UPPER_BOUND" value="1000" units="mmol_per_gDW_per_hr" constant="true"/>
      <parameter id="FLUX_VALUE" value="0" units="mmol_per_gDW_per_hr" constant="true"/>
```

```
      <parameter id="OBJECTIVE_COEFFICIENT" value="0" units="mmol_per_gDW_per_hr" constant="true"/>
    </listOfParameters>
  </kineticLaw>
</reaction>
<reaction id="R_R784" name="PHD transport" reversible="false">
  <notes>
    <body xmlns="http://www.w3.org/1999/xhtml">
      <p>GENE_ASSOCIATION: </p>
      <p>SUBSYSTEM: Transport, Extracellular</p>
      <p>EC Number: </p>
      <p>Confidence Level: 1</p>
      <p>AUTHORS: Transport</p>
      <p/>
    </body>
  </notes>
  <listOfReactants>
    <speciesReference species="M_PHD_c" stoichiometry="1"/>
  </listOfReactants>
  <listOfProducts>
    <speciesReference species="M_PHD_e" stoichiometry="1"/>
  </listOfProducts>
  <kineticLaw>
    <math xmlns="http://www.w3.org/1998/Math/MathML">
      <ci> FLUX_VALUE </ci>
    </math>
    <listOfParameters>
      <parameter id="LOWER_BOUND" value="0" units="mmol_per_gDW_per_hr" constant="true"/>
      <parameter id="UPPER_BOUND" value="1000" units="mmol_per_gDW_per_hr" constant="true"/>
      <parameter id="FLUX_VALUE" value="0" units="mmol_per_gDW_per_hr" constant="true"/>
```

```xml
<parameter id="OBJECTIVE_COEFFICIENT" value="0" units="mmol_per_gDW_per_hr" constant="true"/>
</listOfParameters>
</kineticLaw>
</reaction>
<reaction id="R_R785" name="PHDD transport" reversible="false">
<notes>
<body xmlns="http://www.w3.org/1999/xhtml">
<p>GENE_ASSOCIATION: </p>
<p>SUBSYSTEM: Transport, Extracellular</p>
<p>EC Number: </p>
<p>Confidence Level: 1</p>
<p>AUTHORS: Transport</p>
<p/>
</body>
</notes>
<listOfReactants>
<speciesReference species="M_PHDD_c" stoichiometry="1"/>
</listOfReactants>
<listOfProducts>
<speciesReference species="M_PHDD_e" stoichiometry="1"/>
</listOfProducts>
<kineticLaw>
<math xmlns="http://www.w3.org/1998/Math/MathML">
<ci> FLUX_VALUE </ci>
</math>
<listOfParameters>
<parameter id="LOWER_BOUND" value="0" units="mmol_per_gDW_per_hr" constant="true"/>
<parameter id="UPPER_BOUND" value="1000" units="mmol_per_gDW_per_hr" constant="true"/>
<parameter id="FLUX_VALUE" value="0" units="mmol_per_gDW_per_hr" constant="true"/>
```

```
      <parameter id="OBJECTIVE_COEFFICIENT" value="0" units="mmol_per_gDW_per_hr" constant="true"/>
    </listOfParameters>
  </kineticLaw>
</reaction>
<reaction id="R_R786" name="Glucose exchange" reversible="true">
  <notes>
    <body xmlns="http://www.w3.org/1999/xhtml">
      <p>GENE_ASSOCIATION: </p>
      <p>SUBSYSTEM: Exchange Reactions</p>
      <p>EC Number: </p>
      <p>Confidence Level: 1</p>
      <p>AUTHORS: </p>
      <p/>
    </body>
  </notes>
  <listOfReactants>
    <speciesReference species="M_D_GLC_e" stoichiometry="1"/>
  </listOfReactants>
  <kineticLaw>
    <math xmlns="http://www.w3.org/1998/Math/MathML">
      <ci> FLUX_VALUE </ci>
    </math>
    <listOfParameters>
      <parameter id="LOWER_BOUND" value="-10" units="mmol_per_gDW_per_hr" constant="true"/>
      <parameter id="UPPER_BOUND" value="1000" units="mmol_per_gDW_per_hr" constant="true"/>
      <parameter id="FLUX_VALUE" value="0" units="mmol_per_gDW_per_hr" constant="true"/>
      <parameter id="OBJECTIVE_COEFFICIENT" value="0" units="mmol_per_gDW_per_hr" constant="true"/>
    </listOfParameters>
  </kineticLaw>
```

```xml
</reaction>
<reaction id="R_R787" name="Water exchange" reversible="true">
  <notes>
    <body xmlns="http://www.w3.org/1999/xhtml">
      <p>GENE_ASSOCIATION: </p>
      <p>SUBSYSTEM: Exchange Reactions</p>
      <p>EC Number: </p>
      <p>Confidence Level: 1</p>
      <p>AUTHORS: </p>
      <p/>
    </body>
  </notes>
  <listOfReactants>
    <speciesReference species="M_H2O_e" stoichiometry="1"/>
  </listOfReactants>
  <kineticLaw>
    <math xmlns="http://www.w3.org/1998/Math/MathML">
      <ci> FLUX_VALUE </ci>
    </math>
    <listOfParameters>
      <parameter id="LOWER_BOUND" value="-1000" units="mmol_per_gDW_per_hr" constant="true"/>
      <parameter id="UPPER_BOUND" value="1000" units="mmol_per_gDW_per_hr" constant="true"/>
      <parameter id="FLUX_VALUE" value="0" units="mmol_per_gDW_per_hr" constant="true"/>
      <parameter id="OBJECTIVE_COEFFICIENT" value="0" units="mmol_per_gDW_per_hr" constant="true"/>
    </listOfParameters>
  </kineticLaw>
</reaction>
<reaction id="R_R788" name="Oxygen exchange" reversible="true">
  <notes>
```

```xml
    <body xmlns="http://www.w3.org/1999/xhtml">
      <p>GENE_ASSOCIATION: </p>
      <p>SUBSYSTEM: Exchange Reactions</p>
      <p>EC Number: </p>
      <p>Confidence Level: 1</p>
      <p>AUTHORS: </p>
      <p/>
    </body>
  </notes>
  <listOfReactants>
    <speciesReference species="M_O2_e" stoichiometry="1"/>
  </listOfReactants>
  <kineticLaw>
    <math xmlns="http://www.w3.org/1998/Math/MathML">
      <ci> FLUX_VALUE </ci>
    </math>
    <listOfParameters>
      <parameter id="LOWER_BOUND" value="-10" units="mmol_per_gDW_per_hr" constant="true"/>
      <parameter id="UPPER_BOUND" value="1000" units="mmol_per_gDW_per_hr" constant="true"/>
      <parameter id="FLUX_VALUE" value="0" units="mmol_per_gDW_per_hr" constant="true"/>
      <parameter id="OBJECTIVE_COEFFICIENT" value="0" units="mmol_per_gDW_per_hr" constant="true"/>
    </listOfParameters>
  </kineticLaw>
</reaction>
<reaction id="R_R789" name="Sulfate exchange" reversible="true">
  <notes>
    <body xmlns="http://www.w3.org/1999/xhtml">
      <p>GENE_ASSOCIATION: </p>
      <p>SUBSYSTEM: Exchange Reactions</p>
```

```xml
      <p>EC Number: </p>
      <p>Confidence Level: 1</p>
      <p>AUTHORS: </p>
      <p/>
    </body>
  </notes>
  <listOfReactants>
    <speciesReference species="M_SO4_e" stoichiometry="1"/>
  </listOfReactants>
  <kineticLaw>
    <math xmlns="http://www.w3.org/1998/Math/MathML">
      <ci> FLUX_VALUE </ci>
    </math>
    <listOfParameters>
      <parameter id="LOWER_BOUND" value="0" units="mmol_per_gDW_per_hr" constant="true"/>
      <parameter id="UPPER_BOUND" value="1000" units="mmol_per_gDW_per_hr" constant="true"/>
      <parameter id="FLUX_VALUE" value="0" units="mmol_per_gDW_per_hr" constant="true"/>
      <parameter id="OBJECTIVE_COEFFICIENT" value="0" units="mmol_per_gDW_per_hr" constant="true"/>
    </listOfParameters>
  </kineticLaw>
</reaction>
<reaction id="R_R790" name="Phosphate exchange" reversible="true">
  <notes>
    <body xmlns="http://www.w3.org/1999/xhtml">
      <p>GENE_ASSOCIATION: </p>
      <p>SUBSYSTEM: Exchange Reactions</p>
      <p>EC Number: </p>
      <p>Confidence Level: 1</p>
      <p>AUTHORS: </p>
```

```
<p/>
</body>
</notes>
<listOfReactants>
<speciesReference species="M_Pi_e" stoichiometry="1"/>
</listOfReactants>
<kineticLaw>
<math xmlns="http://www.w3.org/1998/Math/MathML">
<ci> FLUX_VALUE </ci>
</math>
<listOfParameters>
<parameter id="LOWER_BOUND" value="-1000" units="mmol_per_gDW_per_hr" constant="true"/>
<parameter id="UPPER_BOUND" value="1000" units="mmol_per_gDW_per_hr" constant="true"/>
<parameter id="FLUX_VALUE" value="0" units="mmol_per_gDW_per_hr" constant="true"/>
<parameter id="OBJECTIVE_COEFFICIENT" value="0" units="mmol_per_gDW_per_hr" constant="true"/>
</listOfParameters>
</kineticLaw>
</reaction>
<reaction id="R_R791" name="Carbon dioxide exchange" reversible="true">
<notes>
<body xmlns="http://www.w3.org/1999/xhtml">
<p>GENE_ASSOCIATION: </p>
<p>SUBSYSTEM: Exchange Reactions</p>
<p>EC Number: </p>
<p>Confidence Level: 1</p>
<p>AUTHORS: </p>
<p/>
</body>
</notes>
```

```
  <listOfReactants>
    <speciesReference species="M_CO2_e" stoichiometry="1"/>
  </listOfReactants>
  <kineticLaw>
    <math xmlns="http://www.w3.org/1998/Math/MathML">
      <ci> FLUX_VALUE </ci>
    </math>
    <listOfParameters>
      <parameter id="LOWER_BOUND" value="0" units="mmol_per_gDW_per_hr" constant="true"/>
      <parameter id="UPPER_BOUND" value="1000" units="mmol_per_gDW_per_hr" constant="true"/>
      <parameter id="FLUX_VALUE" value="0" units="mmol_per_gDW_per_hr" constant="true"/>
      <parameter id="OBJECTIVE_COEFFICIENT" value="0" units="mmol_per_gDW_per_hr" constant="true"/>
    </listOfParameters>
  </kineticLaw>
</reaction>
<reaction id="R_R792" name="Acetate exchange" reversible="true">
  <notes>
    <body xmlns="http://www.w3.org/1999/xhtml">
      <p>GENE_ASSOCIATION: </p>
      <p>SUBSYSTEM: Exchange Reactions</p>
      <p>EC Number: </p>
      <p>Confidence Level: 1</p>
      <p>AUTHORS: </p>
      <p/>
    </body>
  </notes>
  <listOfReactants>
    <speciesReference species="M_AC_e" stoichiometry="1"/>
  </listOfReactants>
```

```xml
  <kineticLaw>
    <math xmlns="http://www.w3.org/1998/Math/MathML">
      <ci> FLUX_VALUE </ci>
    </math>
    <listOfParameters>
      <parameter id="LOWER_BOUND" value="0" units="mmol_per_gDW_per_hr" constant="true"/>
      <parameter id="UPPER_BOUND" value="1000" units="mmol_per_gDW_per_hr" constant="true"/>
      <parameter id="FLUX_VALUE" value="0" units="mmol_per_gDW_per_hr" constant="true"/>
      <parameter id="OBJECTIVE_COEFFICIENT" value="0" units="mmol_per_gDW_per_hr" constant="true"/>
    </listOfParameters>
  </kineticLaw>
</reaction>
<reaction id="R_R793" name="D-Alanyl-D-alanine exchange" reversible="true">
  <notes>
    <body xmlns="http://www.w3.org/1999/xhtml">
      <p>GENE_ASSOCIATION: </p>
      <p>SUBSYSTEM: Exchange Reactions</p>
      <p>EC Number: </p>
      <p>Confidence Level: 1</p>
      <p>AUTHORS: </p>
      <p/>
    </body>
  </notes>
  <listOfReactants>
    <speciesReference species="M_D_ALA_D_ALA_e" stoichiometry="1"/>
  </listOfReactants>
  <kineticLaw>
    <math xmlns="http://www.w3.org/1998/Math/MathML">
      <ci> FLUX_VALUE </ci>
```

```xml
</math>

<listOfParameters>

<parameter id="LOWER_BOUND" value="0" units="mmol_per_gDW_per_hr" constant="true"/>

<parameter id="UPPER_BOUND" value="1000" units="mmol_per_gDW_per_hr" constant="true"/>

<parameter id="FLUX_VALUE" value="0" units="mmol_per_gDW_per_hr" constant="true"/>

<parameter id="OBJECTIVE_COEFFICIENT" value="0" units="mmol_per_gDW_per_hr" constant="true"/>

</listOfParameters>

</kineticLaw>

</reaction>

<reaction id="R_R794" name="L-Arginine exchange" reversible="true">

<notes>

<body xmlns="http://www.w3.org/1999/xhtml">

<p>GENE_ASSOCIATION: </p>

<p>SUBSYSTEM: Exchange Reactions</p>

<p>EC Number: </p>

<p>Confidence Level: 1</p>

<p>AUTHORS: </p>

<p/>

</body>

</notes>

<listOfReactants>

<speciesReference species="M_L_ARG_e" stoichiometry="1"/>

</listOfReactants>

<kineticLaw>

<math xmlns="http://www.w3.org/1998/Math/MathML">

<ci> FLUX_VALUE </ci>

</math>

<listOfParameters>

<parameter id="LOWER_BOUND" value="0" units="mmol_per_gDW_per_hr" constant="true"/>
```

```
      <parameter id="UPPER_BOUND" value="1000" units="mmol_per_gDW_per_hr" constant="true"/>
      <parameter id="FLUX_VALUE" value="0" units="mmol_per_gDW_per_hr" constant="true"/>
      <parameter id="OBJECTIVE_COEFFICIENT" value="0" units="mmol_per_gDW_per_hr" constant="true"/>
    </listOfParameters>
  </kineticLaw>
</reaction>
<reaction id="R_R795" name="L-Asparagine exchange" reversible="true">
  <notes>
    <body xmlns="http://www.w3.org/1999/xhtml">
      <p>GENE_ASSOCIATION: </p>
      <p>SUBSYSTEM: Exchange Reactions</p>
      <p>EC Number: </p>
      <p>Confidence Level: 1</p>
      <p>AUTHORS: </p>
      <p/>
    </body>
  </notes>
  <listOfReactants>
    <speciesReference species="M_L_ASN_e" stoichiometry="1"/>
  </listOfReactants>
  <kineticLaw>
    <math xmlns="http://www.w3.org/1998/Math/MathML">
      <ci> FLUX_VALUE </ci>
    </math>
    <listOfParameters>
      <parameter id="LOWER_BOUND" value="0" units="mmol_per_gDW_per_hr" constant="true"/>
      <parameter id="UPPER_BOUND" value="1000" units="mmol_per_gDW_per_hr" constant="true"/>
      <parameter id="FLUX_VALUE" value="0" units="mmol_per_gDW_per_hr" constant="true"/>
      <parameter id="OBJECTIVE_COEFFICIENT" value="0" units="mmol_per_gDW_per_hr" constant="true"/>
```

```
    </listOfParameters>
  </kineticLaw>
</reaction>
<reaction id="R_R796" name="Citrate exchange" reversible="true">
  <notes>
    <body xmlns="http://www.w3.org/1999/xhtml">
      <p>GENE_ASSOCIATION: </p>
      <p>SUBSYSTEM: Exchange Reactions</p>
      <p>EC Number: </p>
      <p>Confidence Level: 1</p>
      <p>AUTHORS: </p>
      <p/>
    </body>
  </notes>
  <listOfReactants>
    <speciesReference species="M_CIT_e" stoichiometry="1"/>
  </listOfReactants>
  <kineticLaw>
    <math xmlns="http://www.w3.org/1998/Math/MathML">
      <ci> FLUX_VALUE </ci>
    </math>
    <listOfParameters>
      <parameter id="LOWER_BOUND" value="0" units="mmol_per_gDW_per_hr" constant="true"/>
      <parameter id="UPPER_BOUND" value="1000" units="mmol_per_gDW_per_hr" constant="true"/>
      <parameter id="FLUX_VALUE" value="0" units="mmol_per_gDW_per_hr" constant="true"/>
      <parameter id="OBJECTIVE_COEFFICIENT" value="0" units="mmol_per_gDW_per_hr" constant="true"/>
    </listOfParameters>
  </kineticLaw>
</reaction>
```

```xml
<reaction id="R_R797" name="Ethanol exchange" reversible="true">
<notes>
<body xmlns="http://www.w3.org/1999/xhtml">
<p>GENE_ASSOCIATION: </p>
<p>SUBSYSTEM: Exchange Reactions</p>
<p>EC Number: </p>
<p>Confidence Level: 1</p>
<p>AUTHORS: </p>
<p/>
</body>
</notes>
<listOfReactants>
<speciesReference species="M_ETH_e" stoichiometry="1"/>
</listOfReactants>
<kineticLaw>
<math xmlns="http://www.w3.org/1998/Math/MathML">
<ci> FLUX_VALUE </ci>
</math>
<listOfParameters>
<parameter id="LOWER_BOUND" value="0" units="mmol_per_gDW_per_hr" constant="true"/>
<parameter id="UPPER_BOUND" value="1000" units="mmol_per_gDW_per_hr" constant="true"/>
<parameter id="FLUX_VALUE" value="0" units="mmol_per_gDW_per_hr" constant="true"/>
<parameter id="OBJECTIVE_COEFFICIENT" value="0" units="mmol_per_gDW_per_hr" constant="true"/>
</listOfParameters>
</kineticLaw>
</reaction>
<reaction id="R_R798" name="D-Fructose 1-phosphate exchange" reversible="true">
<notes>
<body xmlns="http://www.w3.org/1999/xhtml">
```

```
      <p>GENE_ASSOCIATION: </p>
      <p>SUBSYSTEM: Exchange Reactions</p>
      <p>EC Number: </p>
      <p>Confidence Level: 1</p>
      <p>AUTHORS: </p>
      <p/>
    </body>
  </notes>
  <listOfReactants>
    <speciesReference species="M_F1P_e" stoichiometry="1"/>
  </listOfReactants>
  <kineticLaw>
    <math xmlns="http://www.w3.org/1998/Math/MathML">
      <ci> FLUX_VALUE </ci>
    </math>
    <listOfParameters>
      <parameter id="LOWER_BOUND" value="0" units="mmol_per_gDW_per_hr" constant="true"/>
      <parameter id="UPPER_BOUND" value="1000" units="mmol_per_gDW_per_hr" constant="true"/>
      <parameter id="FLUX_VALUE" value="0" units="mmol_per_gDW_per_hr" constant="true"/>
      <parameter id="OBJECTIVE_COEFFICIENT" value="0" units="mmol_per_gDW_per_hr" constant="true"/>
    </listOfParameters>
  </kineticLaw>
</reaction>
<reaction id="R_R799" name="L-Fuculose 1-phosphate exchange" reversible="true">
  <notes>
    <body xmlns="http://www.w3.org/1999/xhtml">
      <p>GENE_ASSOCIATION: </p>
      <p>SUBSYSTEM: Exchange Reactions</p>
      <p>EC Number: </p>
```

```xml
      <p>Confidence Level: 1</p>
      <p>AUTHORS: </p>
      <p/>
    </body>
  </notes>
  <listOfReactants>
    <speciesReference species="M_FUCL1P_e" stoichiometry="1"/>
  </listOfReactants>
  <kineticLaw>
    <math xmlns="http://www.w3.org/1998/Math/MathML">
      <ci> FLUX_VALUE </ci>
    </math>
    <listOfParameters>
      <parameter id="LOWER_BOUND" value="0" units="mmol_per_gDW_per_hr" constant="true"/>
      <parameter id="UPPER_BOUND" value="1000" units="mmol_per_gDW_per_hr" constant="true"/>
      <parameter id="FLUX_VALUE" value="0" units="mmol_per_gDW_per_hr" constant="true"/>
      <parameter id="OBJECTIVE_COEFFICIENT" value="0" units="mmol_per_gDW_per_hr" constant="true"/>
    </listOfParameters>
  </kineticLaw>
</reaction>
<reaction id="R_R800" name="Ferrous ion exchange" reversible="true">
  <notes>
    <body xmlns="http://www.w3.org/1999/xhtml">
      <p>GENE_ASSOCIATION: </p>
      <p>SUBSYSTEM: Exchange Reactions</p>
      <p>EC Number: </p>
      <p>Confidence Level: 1</p>
      <p>AUTHORS: </p>
      <p/>
```

```
    </body>
   </notes>
   <listOfReactants>
    <speciesReference species="M_Fe2_e" stoichiometry="1"/>
   </listOfReactants>
   <kineticLaw>
    <math xmlns="http://www.w3.org/1998/Math/MathML">
     <ci> FLUX_VALUE </ci>
    </math>
    <listOfParameters>
     <parameter id="LOWER_BOUND" value="-1000" units="mmol_per_gDW_per_hr" constant="true"/>
     <parameter id="UPPER_BOUND" value="1000" units="mmol_per_gDW_per_hr" constant="true"/>
     <parameter id="FLUX_VALUE" value="0" units="mmol_per_gDW_per_hr" constant="true"/>
     <parameter id="OBJECTIVE_COEFFICIENT" value="0" units="mmol_per_gDW_per_hr" constant="true"/>
    </listOfParameters>
   </kineticLaw>
  </reaction>
  <reaction id="R_R801" name="D-Fructose exchange" reversible="true">
   <notes>
    <body xmlns="http://www.w3.org/1999/xhtml">
     <p>GENE_ASSOCIATION: </p>
     <p>SUBSYSTEM: Exchange Reactions</p>
     <p>EC Number: </p>
     <p>Confidence Level: 1</p>
     <p>AUTHORS: </p>
     <p/>
    </body>
   </notes>
   <listOfReactants>
```

```
    <speciesReference species="M_D_FRU_e" stoichiometry="1"/>
  </listOfReactants>
  <kineticLaw>
    <math xmlns="http://www.w3.org/1998/Math/MathML">
      <ci> FLUX_VALUE </ci>
    </math>
    <listOfParameters>
      <parameter id="LOWER_BOUND" value="0" units="mmol_per_gDW_per_hr" constant="true"/>
      <parameter id="UPPER_BOUND" value="1000" units="mmol_per_gDW_per_hr" constant="true"/>
      <parameter id="FLUX_VALUE" value="0" units="mmol_per_gDW_per_hr" constant="true"/>
      <parameter id="OBJECTIVE_COEFFICIENT" value="0" units="mmol_per_gDW_per_hr" constant="true"/>
    </listOfParameters>
  </kineticLaw>
</reaction>
<reaction id="R_R802" name="Fumarate exchange" reversible="true">
  <notes>
    <body xmlns="http://www.w3.org/1999/xhtml">
      <p>GENE_ASSOCIATION: </p>
      <p>SUBSYSTEM: Exchange Reactions</p>
      <p>EC Number: </p>
      <p>Confidence Level: 1</p>
      <p>AUTHORS: </p>
      <p/>
    </body>
  </notes>
  <listOfReactants>
    <speciesReference species="M_FUM_e" stoichiometry="1"/>
  </listOfReactants>
  <kineticLaw>
```

```
    <math xmlns="http://www.w3.org/1998/Math/MathML">
      <ci> FLUX_VALUE </ci>
    </math>
    <listOfParameters>
      <parameter id="LOWER_BOUND" value="0" units="mmol_per_gDW_per_hr" constant="true"/>
      <parameter id="UPPER_BOUND" value="1000" units="mmol_per_gDW_per_hr" constant="true"/>
      <parameter id="FLUX_VALUE" value="0" units="mmol_per_gDW_per_hr" constant="true"/>
      <parameter id="OBJECTIVE_COEFFICIENT" value="0" units="mmol_per_gDW_per_hr" constant="true"/>
    </listOfParameters>
  </kineticLaw>
</reaction>
<reaction id="R_R803" name="alpha-D-Glucosamine 1-phosphate exchange" reversible="true">
  <notes>
    <body xmlns="http://www.w3.org/1999/xhtml">
      <p>GENE_ASSOCIATION: </p>
      <p>SUBSYSTEM: Exchange Reactions</p>
      <p>EC Number: </p>
      <p>Confidence Level: 1</p>
      <p>AUTHORS: </p>
      <p/>
    </body>
  </notes>
  <listOfReactants>
    <speciesReference species="M_GLUCSA1P_e" stoichiometry="1"/>
  </listOfReactants>
  <kineticLaw>
    <math xmlns="http://www.w3.org/1998/Math/MathML">
      <ci> FLUX_VALUE </ci>
    </math>
```

```
    <listOfParameters>
      <parameter id="LOWER_BOUND" value="0" units="mmol_per_gDW_per_hr" constant="true"/>
      <parameter id="UPPER_BOUND" value="1000" units="mmol_per_gDW_per_hr" constant="true"/>
      <parameter id="FLUX_VALUE" value="0" units="mmol_per_gDW_per_hr" constant="true"/>
      <parameter id="OBJECTIVE_COEFFICIENT" value="0" units="mmol_per_gDW_per_hr" constant="true"/>
    </listOfParameters>
  </kineticLaw>
</reaction>
<reaction id="R_R804" name="D-Glucosamine 6-phosphate exchange" reversible="true">
  <notes>
    <body xmlns="http://www.w3.org/1999/xhtml">
      <p>GENE_ASSOCIATION: </p>
      <p>SUBSYSTEM: Exchange Reactions</p>
      <p>EC Number: </p>
      <p>Confidence Level: 1</p>
      <p>AUTHORS: </p>
      <p/>
    </body>
  </notes>
  <listOfReactants>
    <speciesReference species="M_GLUSAP_e" stoichiometry="1"/>
  </listOfReactants>
  <kineticLaw>
    <math xmlns="http://www.w3.org/1998/Math/MathML">
      <ci> FLUX_VALUE </ci>
    </math>
    <listOfParameters>
      <parameter id="LOWER_BOUND" value="0" units="mmol_per_gDW_per_hr" constant="true"/>
      <parameter id="UPPER_BOUND" value="1000" units="mmol_per_gDW_per_hr" constant="true"/>
```

```xml
      <parameter id="FLUX_VALUE" value="0" units="mmol_per_gDW_per_hr" constant="true"/>
      <parameter id="OBJECTIVE_COEFFICIENT" value="0" units="mmol_per_gDW_per_hr" constant="true"/>
    </listOfParameters>
  </kineticLaw>
</reaction>
<reaction id="R_R805" name="Glycolaldehyde exchange" reversible="true">
  <notes>
    <body xmlns="http://www.w3.org/1999/xhtml">
      <p>GENE_ASSOCIATION: </p>
      <p>SUBSYSTEM: Exchange Reactions</p>
      <p>EC Number: </p>
      <p>Confidence Level: 1</p>
      <p>AUTHORS: </p>
      <p/>
    </body>
  </notes>
  <listOfReactants>
    <speciesReference species="M_GLYCALD_e" stoichiometry="1"/>
  </listOfReactants>
  <kineticLaw>
    <math xmlns="http://www.w3.org/1998/Math/MathML">
      <ci> FLUX_VALUE </ci>
    </math>
    <listOfParameters>
      <parameter id="LOWER_BOUND" value="0" units="mmol_per_gDW_per_hr" constant="true"/>
      <parameter id="UPPER_BOUND" value="1000" units="mmol_per_gDW_per_hr" constant="true"/>
      <parameter id="FLUX_VALUE" value="0" units="mmol_per_gDW_per_hr" constant="true"/>
      <parameter id="OBJECTIVE_COEFFICIENT" value="0" units="mmol_per_gDW_per_hr" constant="true"/>
    </listOfParameters>
```

```
  </kineticLaw>
</reaction>
<reaction id="R_R806" name="D-Glutamate exchange" reversible="true">
  <notes>
    <body xmlns="http://www.w3.org/1999/xhtml">
      <p>GENE_ASSOCIATION: </p>
      <p>SUBSYSTEM: Exchange Reactions</p>
      <p>EC Number: </p>
      <p>Confidence Level: 1</p>
      <p>AUTHORS: </p>
      <p/>
    </body>
  </notes>
  <listOfReactants>
    <speciesReference species="M_D_GLU_e" stoichiometry="1"/>
  </listOfReactants>
  <kineticLaw>
    <math xmlns="http://www.w3.org/1998/Math/MathML">
      <ci> FLUX_VALUE </ci>
    </math>
    <listOfParameters>
      <parameter id="LOWER_BOUND" value="0" units="mmol_per_gDW_per_hr" constant="true"/>
      <parameter id="UPPER_BOUND" value="1000" units="mmol_per_gDW_per_hr" constant="true"/>
      <parameter id="FLUX_VALUE" value="0" units="mmol_per_gDW_per_hr" constant="true"/>
      <parameter id="OBJECTIVE_COEFFICIENT" value="0" units="mmol_per_gDW_per_hr" constant="true"/>
    </listOfParameters>
  </kineticLaw>
</reaction>
<reaction id="R_R807" name="Glycolate exchange" reversible="true">
```

```xml
  <notes>
    <body xmlns="http://www.w3.org/1999/xhtml">
      <p>GENE_ASSOCIATION: </p>
      <p>SUBSYSTEM: Exchange Reactions</p>
      <p>EC Number: </p>
      <p>Confidence Level: 1</p>
      <p>AUTHORS: </p>
      <p/>
    </body>
  </notes>
  <listOfReactants>
    <speciesReference species="M_HY_AC_e" stoichiometry="1"/>
  </listOfReactants>
  <kineticLaw>
    <math xmlns="http://www.w3.org/1998/Math/MathML">
      <ci> FLUX_VALUE </ci>
    </math>
    <listOfParameters>
      <parameter id="LOWER_BOUND" value="0" units="mmol_per_gDW_per_hr" constant="true"/>
      <parameter id="UPPER_BOUND" value="1000" units="mmol_per_gDW_per_hr" constant="true"/>
      <parameter id="FLUX_VALUE" value="0" units="mmol_per_gDW_per_hr" constant="true"/>
      <parameter id="OBJECTIVE_COEFFICIENT" value="0" units="mmol_per_gDW_per_hr" constant="true"/>
    </listOfParameters>
  </kineticLaw>
</reaction>
<reaction id="R_R808" name="D-Glycerate exchange" reversible="true">
  <notes>
    <body xmlns="http://www.w3.org/1999/xhtml">
      <p>GENE_ASSOCIATION: </p>
```

```xml
      <p>SUBSYSTEM: Exchange Reactions</p>
      <p>EC Number: </p>
      <p>Confidence Level: 1</p>
      <p>AUTHORS: </p>
      <p/>
    </body>
  </notes>
  <listOfReactants>
    <speciesReference species="M_D_GLCT_e" stoichiometry="1"/>
  </listOfReactants>
  <kineticLaw>
    <math xmlns="http://www.w3.org/1998/Math/MathML">
      <ci> FLUX_VALUE </ci>
    </math>
    <listOfParameters>
      <parameter id="LOWER_BOUND" value="0" units="mmol_per_gDW_per_hr" constant="true"/>
      <parameter id="UPPER_BOUND" value="1000" units="mmol_per_gDW_per_hr" constant="true"/>
      <parameter id="FLUX_VALUE" value="0" units="mmol_per_gDW_per_hr" constant="true"/>
      <parameter id="OBJECTIVE_COEFFICIENT" value="0" units="mmol_per_gDW_per_hr" constant="true"/>
    </listOfParameters>
  </kineticLaw>
</reaction>
<reaction id="R_R809" name="Glycerol exchange" reversible="true">
  <notes>
    <body xmlns="http://www.w3.org/1999/xhtml">
      <p>GENE_ASSOCIATION: </p>
      <p>SUBSYSTEM: Exchange Reactions</p>
      <p>EC Number: </p>
      <p>Confidence Level: 1</p>
```

```
      <p>AUTHORS: </p>
      <p/>
    </body>
  </notes>
  <listOfReactants>
    <speciesReference species="M_GLYCRL_e" stoichiometry="1"/>
  </listOfReactants>
  <kineticLaw>
    <math xmlns="http://www.w3.org/1998/Math/MathML">
      <ci> FLUX_VALUE </ci>
    </math>
    <listOfParameters>
      <parameter id="LOWER_BOUND" value="0" units="mmol_per_gDW_per_hr" constant="true"/>
      <parameter id="UPPER_BOUND" value="1000" units="mmol_per_gDW_per_hr" constant="true"/>
      <parameter id="FLUX_VALUE" value="0" units="mmol_per_gDW_per_hr" constant="true"/>
      <parameter id="OBJECTIVE_COEFFICIENT" value="0" units="mmol_per_gDW_per_hr" constant="true"/>
    </listOfParameters>
  </kineticLaw>
</reaction>
<reaction id="R_R810" name="Glycine exchange" reversible="true">
  <notes>
    <body xmlns="http://www.w3.org/1999/xhtml">
      <p>GENE_ASSOCIATION: </p>
      <p>SUBSYSTEM: Exchange Reactions</p>
      <p>EC Number: </p>
      <p>Confidence Level: 1</p>
      <p>AUTHORS: </p>
      <p/>
    </body>
```

```
  </notes>
  <listOfReactants>
    <speciesReference species="M_L_GLY_e" stoichiometry="1"/>
  </listOfReactants>
  <kineticLaw>
    <math xmlns="http://www.w3.org/1998/Math/MathML">
      <ci> FLUX_VALUE </ci>
    </math>
    <listOfParameters>
      <parameter id="LOWER_BOUND" value="0" units="mmol_per_gDW_per_hr" constant="true"/>
      <parameter id="UPPER_BOUND" value="1000" units="mmol_per_gDW_per_hr" constant="true"/>
      <parameter id="FLUX_VALUE" value="0" units="mmol_per_gDW_per_hr" constant="true"/>
      <parameter id="OBJECTIVE_COEFFICIENT" value="0" units="mmol_per_gDW_per_hr" constant="true"/>
    </listOfParameters>
  </kineticLaw>
</reaction>
<reaction id="R_R811" name="Hydrogen sulfide exchange" reversible="true">
  <notes>
    <body xmlns="http://www.w3.org/1999/xhtml">
      <p>GENE_ASSOCIATION: </p>
      <p>SUBSYSTEM: Exchange Reactions</p>
      <p>EC Number: </p>
      <p>Confidence Level: 1</p>
      <p>AUTHORS: </p>
      <p/>
    </body>
  </notes>
  <listOfReactants>
    <speciesReference species="M_H2S_e" stoichiometry="1"/>
```

```
  </listOfReactants>
  <kineticLaw>
    <math xmlns="http://www.w3.org/1998/Math/MathML">
      <ci> FLUX_VALUE </ci>
    </math>
    <listOfParameters>
      <parameter id="LOWER_BOUND" value="0" units="mmol_per_gDW_per_hr" constant="true"/>
      <parameter id="UPPER_BOUND" value="1000" units="mmol_per_gDW_per_hr" constant="true"/>
      <parameter id="FLUX_VALUE" value="0" units="mmol_per_gDW_per_hr" constant="true"/>
      <parameter id="OBJECTIVE_COEFFICIENT" value="0" units="mmol_per_gDW_per_hr" constant="true"/>
    </listOfParameters>
  </kineticLaw>
</reaction>
<reaction id="R_R812" name="L-Homocysteine exchange" reversible="true">
  <notes>
    <body xmlns="http://www.w3.org/1999/xhtml">
      <p>GENE_ASSOCIATION: </p>
      <p>SUBSYSTEM: Exchange Reactions</p>
      <p>EC Number: </p>
      <p>Confidence Level: 1</p>
      <p>AUTHORS: </p>
      <p/>
    </body>
  </notes>
  <listOfReactants>
    <speciesReference species="M_L_HCYS_e" stoichiometry="1"/>
  </listOfReactants>
  <kineticLaw>
    <math xmlns="http://www.w3.org/1998/Math/MathML">
```

```xml
      <ci> FLUX_VALUE </ci>
    </math>
    <listOfParameters>
      <parameter id="LOWER_BOUND" value="0" units="mmol_per_gDW_per_hr" constant="true"/>
      <parameter id="UPPER_BOUND" value="1000" units="mmol_per_gDW_per_hr" constant="true"/>
      <parameter id="FLUX_VALUE" value="0" units="mmol_per_gDW_per_hr" constant="true"/>
      <parameter id="OBJECTIVE_COEFFICIENT" value="0" units="mmol_per_gDW_per_hr" constant="true"/>
    </listOfParameters>
  </kineticLaw>
</reaction>
<reaction id="R_R813" name="L-Isoleucine tansport" reversible="true">
  <notes>
    <body xmlns="http://www.w3.org/1999/xhtml">
      <p>GENE_ASSOCIATION: </p>
      <p>SUBSYSTEM: Exchange Reactions</p>
      <p>EC Number: </p>
      <p>Confidence Level: 1</p>
      <p>AUTHORS: </p>
      <p/>
    </body>
  </notes>
  <listOfReactants>
    <speciesReference species="M_L_ILE_e" stoichiometry="1"/>
  </listOfReactants>
  <kineticLaw>
    <math xmlns="http://www.w3.org/1998/Math/MathML">
      <ci> FLUX_VALUE </ci>
    </math>
    <listOfParameters>
```

```xml
      <parameter id="LOWER_BOUND" value="0" units="mmol_per_gDW_per_hr" constant="true"/>
      <parameter id="UPPER_BOUND" value="1000" units="mmol_per_gDW_per_hr" constant="true"/>
      <parameter id="FLUX_VALUE" value="0" units="mmol_per_gDW_per_hr" constant="true"/>
      <parameter id="OBJECTIVE_COEFFICIENT" value="0" units="mmol_per_gDW_per_hr" constant="true"/>
    </listOfParameters>
  </kineticLaw>
</reaction>
<reaction id="R_R814" name="L-Lysine exchange" reversible="true">
  <notes>
    <body xmlns="http://www.w3.org/1999/xhtml">
      <p>GENE_ASSOCIATION: </p>
      <p>SUBSYSTEM: Exchange Reactions</p>
      <p>EC Number: </p>
      <p>Confidence Level: 1</p>
      <p>AUTHORS: </p>
      <p/>
    </body>
  </notes>
  <listOfReactants>
    <speciesReference species="M_L_LYS_e" stoichiometry="1"/>
  </listOfReactants>
  <kineticLaw>
    <math xmlns="http://www.w3.org/1998/Math/MathML">
      <ci> FLUX_VALUE </ci>
    </math>
    <listOfParameters>
      <parameter id="LOWER_BOUND" value="0" units="mmol_per_gDW_per_hr" constant="true"/>
      <parameter id="UPPER_BOUND" value="1000" units="mmol_per_gDW_per_hr" constant="true"/>
      <parameter id="FLUX_VALUE" value="0" units="mmol_per_gDW_per_hr" constant="true"/>
```

```xml
<parameter id="OBJECTIVE_COEFFICIENT" value="0" units="mmol_per_gDW_per_hr" constant="true"/>
</listOfParameters>
</kineticLaw>
</reaction>
<reaction id="R_R815" name="L-Malate exchange" reversible="true">
<notes>
<body xmlns="http://www.w3.org/1999/xhtml">
<p>GENE_ASSOCIATION: </p>
<p>SUBSYSTEM: Exchange Reactions</p>
<p>EC Number: </p>
<p>Confidence Level: 1</p>
<p>AUTHORS: </p>
<p/>
</body>
</notes>
<listOfReactants>
<speciesReference species="M_MAL_e" stoichiometry="1"/>
</listOfReactants>
<kineticLaw>
<math xmlns="http://www.w3.org/1998/Math/MathML">
<ci> FLUX_VALUE </ci>
</math>
<listOfParameters>
<parameter id="LOWER_BOUND" value="0" units="mmol_per_gDW_per_hr" constant="true"/>
<parameter id="UPPER_BOUND" value="1000" units="mmol_per_gDW_per_hr" constant="true"/>
<parameter id="FLUX_VALUE" value="0" units="mmol_per_gDW_per_hr" constant="true"/>
<parameter id="OBJECTIVE_COEFFICIENT" value="0" units="mmol_per_gDW_per_hr" constant="true"/>
</listOfParameters>
</kineticLaw>
```

```
</reaction>
<reaction id="R_R816" name="Maltose exchange" reversible="true">
  <notes>
    <body xmlns="http://www.w3.org/1999/xhtml">
      <p>GENE_ASSOCIATION: </p>
      <p>SUBSYSTEM: Exchange Reactions</p>
      <p>EC Number: </p>
      <p>Confidence Level: 1</p>
      <p>AUTHORS: </p>
      <p/>
    </body>
  </notes>
  <listOfReactants>
    <speciesReference species="M_MALT_e" stoichiometry="1"/>
  </listOfReactants>
  <kineticLaw>
    <math xmlns="http://www.w3.org/1998/Math/MathML">
      <ci> FLUX_VALUE </ci>
    </math>
    <listOfParameters>
      <parameter id="LOWER_BOUND" value="0" units="mmol_per_gDW_per_hr" constant="true"/>
      <parameter id="UPPER_BOUND" value="1000" units="mmol_per_gDW_per_hr" constant="true"/>
      <parameter id="FLUX_VALUE" value="0" units="mmol_per_gDW_per_hr" constant="true"/>
      <parameter id="OBJECTIVE_COEFFICIENT" value="0" units="mmol_per_gDW_per_hr" constant="true"/>
    </listOfParameters>
  </kineticLaw>
</reaction>
<reaction id="R_R817" name="Isomaltose exchange" reversible="true">
  <notes>
```

```
    <body xmlns="http://www.w3.org/1999/xhtml">
      <p>GENE_ASSOCIATION: </p>
      <p>SUBSYSTEM: Exchange Reactions</p>
      <p>EC Number: </p>
      <p>Confidence Level: 1</p>
      <p>AUTHORS: </p>
      <p/>
    </body>
  </notes>
  <listOfReactants>
    <speciesReference species="M_IMALT_e" stoichiometry="1"/>
  </listOfReactants>
  <kineticLaw>
    <math xmlns="http://www.w3.org/1998/Math/MathML">
      <ci> FLUX_VALUE </ci>
    </math>
    <listOfParameters>
      <parameter id="LOWER_BOUND" value="0" units="mmol_per_gDW_per_hr" constant="true"/>
      <parameter id="UPPER_BOUND" value="1000" units="mmol_per_gDW_per_hr" constant="true"/>
      <parameter id="FLUX_VALUE" value="0" units="mmol_per_gDW_per_hr" constant="true"/>
      <parameter id="OBJECTIVE_COEFFICIENT" value="0" units="mmol_per_gDW_per_hr" constant="true"/>
    </listOfParameters>
  </kineticLaw>
</reaction>
<reaction id="R_R818" name="D-Mannose 1-phosphate exchange" reversible="true">
  <notes>
    <body xmlns="http://www.w3.org/1999/xhtml">
      <p>GENE_ASSOCIATION: </p>
      <p>SUBSYSTEM: Exchange Reactions</p>
```

```
      <p>EC Number: </p>
      <p>Confidence Level: 1</p>
      <p>AUTHORS: </p>
      <p/>
    </body>
  </notes>
  <listOfReactants>
    <speciesReference species="M_MAN1P_e" stoichiometry="1"/>
  </listOfReactants>
  <kineticLaw>
    <math xmlns="http://www.w3.org/1998/Math/MathML">
      <ci> FLUX_VALUE </ci>
    </math>
    <listOfParameters>
      <parameter id="LOWER_BOUND" value="0" units="mmol_per_gDW_per_hr" constant="true"/>
      <parameter id="UPPER_BOUND" value="1000" units="mmol_per_gDW_per_hr" constant="true"/>
      <parameter id="FLUX_VALUE" value="0" units="mmol_per_gDW_per_hr" constant="true"/>
      <parameter id="OBJECTIVE_COEFFICIENT" value="0" units="mmol_per_gDW_per_hr" constant="true"/>
    </listOfParameters>
  </kineticLaw>
</reaction>
<reaction id="R_R819" name="D-Mannose 6-phosphate exchange" reversible="true">
  <notes>
    <body xmlns="http://www.w3.org/1999/xhtml">
      <p>GENE_ASSOCIATION: </p>
      <p>SUBSYSTEM: Exchange Reactions</p>
      <p>EC Number: </p>
      <p>Confidence Level: 1</p>
      <p>AUTHORS: </p>
```

```xml
<p/>
</body>
</notes>
<listOfReactants>
<speciesReference species="M_M6P_e" stoichiometry="1"/>
</listOfReactants>
<kineticLaw>
<math xmlns="http://www.w3.org/1998/Math/MathML">
<ci> FLUX_VALUE </ci>
</math>
<listOfParameters>
<parameter id="LOWER_BOUND" value="0" units="mmol_per_gDW_per_hr" constant="true"/>
<parameter id="UPPER_BOUND" value="1000" units="mmol_per_gDW_per_hr" constant="true"/>
<parameter id="FLUX_VALUE" value="0" units="mmol_per_gDW_per_hr" constant="true"/>
<parameter id="OBJECTIVE_COEFFICIENT" value="0" units="mmol_per_gDW_per_hr" constant="true"/>
</listOfParameters>
</kineticLaw>
</reaction>
<reaction id="R_R820" name="D-Mannose exchange" reversible="true">
<notes>
<body xmlns="http://www.w3.org/1999/xhtml">
<p>GENE_ASSOCIATION: </p>
<p>SUBSYSTEM: Exchange Reactions</p>
<p>EC Number: </p>
<p>Confidence Level: 1</p>
<p>AUTHORS: </p>
<p/>
</body>
</notes>
```

```
    <listOfReactants>
      <speciesReference species="M_D_MAN_e" stoichiometry="1"/>
    </listOfReactants>
    <kineticLaw>
      <math xmlns="http://www.w3.org/1998/Math/MathML">
        <ci> FLUX_VALUE </ci>
      </math>
      <listOfParameters>
        <parameter id="LOWER_BOUND" value="0" units="mmol_per_gDW_per_hr" constant="true"/>
        <parameter id="UPPER_BOUND" value="1000" units="mmol_per_gDW_per_hr" constant="true"/>
        <parameter id="FLUX_VALUE" value="0" units="mmol_per_gDW_per_hr" constant="true"/>
        <parameter id="OBJECTIVE_COEFFICIENT" value="0" units="mmol_per_gDW_per_hr" constant="true"/>
      </listOfParameters>
    </kineticLaw>
  </reaction>
  <reaction id="R_R821" name="L-Phenylalanine exchange" reversible="true">
    <notes>
      <body xmlns="http://www.w3.org/1999/xhtml">
        <p>GENE_ASSOCIATION: </p>
        <p>SUBSYSTEM: Exchange Reactions</p>
        <p>EC Number: </p>
        <p>Confidence Level: 1</p>
        <p>AUTHORS: </p>
        <p/>
      </body>
    </notes>
    <listOfReactants>
      <speciesReference species="M_L_PHE_e" stoichiometry="1"/>
    </listOfReactants>
```

```
 <kineticLaw>
  <math xmlns="http://www.w3.org/1998/Math/MathML">
   <ci> FLUX_VALUE </ci>
  </math>
  <listOfParameters>
   <parameter id="LOWER_BOUND" value="0" units="mmol_per_gDW_per_hr" constant="true"/>
   <parameter id="UPPER_BOUND" value="1000" units="mmol_per_gDW_per_hr" constant="true"/>
   <parameter id="FLUX_VALUE" value="0" units="mmol_per_gDW_per_hr" constant="true"/>
   <parameter id="OBJECTIVE_COEFFICIENT" value="0" units="mmol_per_gDW_per_hr" constant="true"/>
  </listOfParameters>
 </kineticLaw>
</reaction>
<reaction id="R_R822" name="L-Proline exchange" reversible="true">
 <notes>
  <body xmlns="http://www.w3.org/1999/xhtml">
   <p>GENE_ASSOCIATION: </p>
   <p>SUBSYSTEM: Exchange Reactions</p>
   <p>EC Number: </p>
   <p>Confidence Level: 1</p>
   <p>AUTHORS: </p>
   <p/>
  </body>
 </notes>
 <listOfReactants>
  <speciesReference species="M_L_PRO_e" stoichiometry="1"/>
 </listOfReactants>
 <kineticLaw>
  <math xmlns="http://www.w3.org/1998/Math/MathML">
   <ci> FLUX_VALUE </ci>
```

```xml
      </math>
      <listOfParameters>
        <parameter id="LOWER_BOUND" value="0" units="mmol_per_gDW_per_hr" constant="true"/>
        <parameter id="UPPER_BOUND" value="1000" units="mmol_per_gDW_per_hr" constant="true"/>
        <parameter id="FLUX_VALUE" value="0" units="mmol_per_gDW_per_hr" constant="true"/>
        <parameter id="OBJECTIVE_COEFFICIENT" value="0" units="mmol_per_gDW_per_hr" constant="true"/>
      </listOfParameters>
    </kineticLaw>
  </reaction>
  <reaction id="R_R823" name="D-Ribose exchange" reversible="true">
    <notes>
      <body xmlns="http://www.w3.org/1999/xhtml">
        <p>GENE_ASSOCIATION: </p>
        <p>SUBSYSTEM: Exchange Reactions</p>
        <p>EC Number: </p>
        <p>Confidence Level: 1</p>
        <p>AUTHORS: </p>
        <p/>
      </body>
    </notes>
    <listOfReactants>
      <speciesReference species="M_D_RIB_e" stoichiometry="1"/>
    </listOfReactants>
    <kineticLaw>
      <math xmlns="http://www.w3.org/1998/Math/MathML">
        <ci> FLUX_VALUE </ci>
      </math>
      <listOfParameters>
        <parameter id="LOWER_BOUND" value="0" units="mmol_per_gDW_per_hr" constant="true"/>
```

```
      <parameter id="UPPER_BOUND" value="1000" units="mmol_per_gDW_per_hr" constant="true"/>
      <parameter id="FLUX_VALUE" value="0" units="mmol_per_gDW_per_hr" constant="true"/>
      <parameter id="OBJECTIVE_COEFFICIENT" value="0" units="mmol_per_gDW_per_hr" constant="true"/>
    </listOfParameters>
  </kineticLaw>
</reaction>
<reaction id="R_R824" name="D-Sorbitol exchange" reversible="true">
  <notes>
    <body xmlns="http://www.w3.org/1999/xhtml">
      <p>GENE_ASSOCIATION: </p>
      <p>SUBSYSTEM: Exchange Reactions</p>
      <p>EC Number: </p>
      <p>Confidence Level: 1</p>
      <p>AUTHORS: </p>
      <p/>
    </body>
  </notes>
  <listOfReactants>
    <speciesReference species="M_SORB_e" stoichiometry="1"/>
  </listOfReactants>
  <kineticLaw>
    <math xmlns="http://www.w3.org/1998/Math/MathML">
      <ci> FLUX_VALUE </ci>
    </math>
    <listOfParameters>
      <parameter id="LOWER_BOUND" value="0" units="mmol_per_gDW_per_hr" constant="true"/>
      <parameter id="UPPER_BOUND" value="1000" units="mmol_per_gDW_per_hr" constant="true"/>
      <parameter id="FLUX_VALUE" value="0" units="mmol_per_gDW_per_hr" constant="true"/>
      <parameter id="OBJECTIVE_COEFFICIENT" value="0" units="mmol_per_gDW_per_hr" constant="true"/>
```

```xml
    </listOfParameters>
  </kineticLaw>
</reaction>
<reaction id="R_R825" name="L-Serine exchange" reversible="true">
  <notes>
    <body xmlns="http://www.w3.org/1999/xhtml">
      <p>GENE_ASSOCIATION: </p>
      <p>SUBSYSTEM: Exchange Reactions</p>
      <p>EC Number: </p>
      <p>Confidence Level: 1</p>
      <p>AUTHORS: </p>
      <p/>
    </body>
  </notes>
  <listOfReactants>
    <speciesReference species="M_L_SER_e" stoichiometry="1"/>
  </listOfReactants>
  <kineticLaw>
    <math xmlns="http://www.w3.org/1998/Math/MathML">
      <ci> FLUX_VALUE </ci>
    </math>
    <listOfParameters>
      <parameter id="LOWER_BOUND" value="0" units="mmol_per_gDW_per_hr" constant="true"/>
      <parameter id="UPPER_BOUND" value="1000" units="mmol_per_gDW_per_hr" constant="true"/>
      <parameter id="FLUX_VALUE" value="0" units="mmol_per_gDW_per_hr" constant="true"/>
      <parameter id="OBJECTIVE_COEFFICIENT" value="0" units="mmol_per_gDW_per_hr" constant="true"/>
    </listOfParameters>
  </kineticLaw>
</reaction>
```

```xml
<reaction id="R_R826" name="Sulfite exchange" reversible="true">
 <notes>
  <body xmlns="http://www.w3.org/1999/xhtml">
   <p>GENE_ASSOCIATION: </p>
   <p>SUBSYSTEM: Exchange Reactions</p>
   <p>EC Number: </p>
   <p>Confidence Level: 1</p>
   <p>AUTHORS: </p>
   <p/>
  </body>
 </notes>
 <listOfReactants>
  <speciesReference species="M_SO3_e" stoichiometry="1"/>
 </listOfReactants>
 <kineticLaw>
  <math xmlns="http://www.w3.org/1998/Math/MathML">
   <ci> FLUX_VALUE </ci>
  </math>
  <listOfParameters>
   <parameter id="LOWER_BOUND" value="-1000" units="mmol_per_gDW_per_hr" constant="true"/>
   <parameter id="UPPER_BOUND" value="1000" units="mmol_per_gDW_per_hr" constant="true"/>
   <parameter id="FLUX_VALUE" value="0" units="mmol_per_gDW_per_hr" constant="true"/>
   <parameter id="OBJECTIVE_COEFFICIENT" value="0" units="mmol_per_gDW_per_hr" constant="true"/>
  </listOfParameters>
 </kineticLaw>
</reaction>
<reaction id="R_R827" name="Succinate exchange" reversible="true">
 <notes>
  <body xmlns="http://www.w3.org/1999/xhtml">
```

```
      <p>GENE_ASSOCIATION: </p>
      <p>SUBSYSTEM: Exchange Reactions</p>
      <p>EC Number: </p>
      <p>Confidence Level: 1</p>
      <p>AUTHORS: </p>
      <p/>
    </body>
  </notes>
  <listOfReactants>
    <speciesReference species="M_SUCC_e" stoichiometry="1"/>
  </listOfReactants>
  <kineticLaw>
    <math xmlns="http://www.w3.org/1998/Math/MathML">
      <ci> FLUX_VALUE </ci>
    </math>
    <listOfParameters>
      <parameter id="LOWER_BOUND" value="0" units="mmol_per_gDW_per_hr" constant="true"/>
      <parameter id="UPPER_BOUND" value="1000" units="mmol_per_gDW_per_hr" constant="true"/>
      <parameter id="FLUX_VALUE" value="0" units="mmol_per_gDW_per_hr" constant="true"/>
      <parameter id="OBJECTIVE_COEFFICIENT" value="0" units="mmol_per_gDW_per_hr" constant="true"/>
    </listOfParameters>
  </kineticLaw>
</reaction>
<reaction id="R_R828" name="Sucrose exchange" reversible="true">
  <notes>
    <body xmlns="http://www.w3.org/1999/xhtml">
      <p>GENE_ASSOCIATION: </p>
      <p>SUBSYSTEM: Exchange Reactions</p>
      <p>EC Number: </p>
```

<p>Confidence Level: 1</p>
<p>AUTHORS: </p>
<p/>
</body>
</notes>
<listOfReactants>
<speciesReference species="M_SUC_e" stoichiometry="1"/>
</listOfReactants>
<kineticLaw>
<math xmlns="http://www.w3.org/1998/Math/MathML">
<ci> FLUX_VALUE </ci>
</math>
<listOfParameters>
<parameter id="LOWER_BOUND" value="0" units="mmol_per_gDW_per_hr" constant="true"/>
<parameter id="UPPER_BOUND" value="1000" units="mmol_per_gDW_per_hr" constant="true"/>
<parameter id="FLUX_VALUE" value="0" units="mmol_per_gDW_per_hr" constant="true"/>
<parameter id="OBJECTIVE_COEFFICIENT" value="0" units="mmol_per_gDW_per_hr" constant="true"/>
</listOfParameters>
</kineticLaw>
</reaction>
<reaction id="R_R829" name="L-Alanine exchange" reversible="true">
<notes>
<body xmlns="http://www.w3.org/1999/xhtml">
<p>GENE_ASSOCIATION: </p>
<p>SUBSYSTEM: Exchange Reactions</p>
<p>EC Number: </p>
<p>Confidence Level: 1</p>
<p>AUTHORS: </p>
<p/>

```
    </body>
   </notes>
   <listOfReactants>
    <speciesReference species="M_L_ALA_e" stoichiometry="1"/>
   </listOfReactants>
   <kineticLaw>
    <math xmlns="http://www.w3.org/1998/Math/MathML">
     <ci> FLUX_VALUE </ci>
    </math>
    <listOfParameters>
     <parameter id="LOWER_BOUND" value="0" units="mmol_per_gDW_per_hr" constant="true"/>
     <parameter id="UPPER_BOUND" value="1000" units="mmol_per_gDW_per_hr" constant="true"/>
     <parameter id="FLUX_VALUE" value="0" units="mmol_per_gDW_per_hr" constant="true"/>
     <parameter id="OBJECTIVE_COEFFICIENT" value="0" units="mmol_per_gDW_per_hr" constant="true"/>
    </listOfParameters>
   </kineticLaw>
  </reaction>
  <reaction id="R_R830" name="L-Aspartate exchange" reversible="true">
   <notes>
    <body xmlns="http://www.w3.org/1999/xhtml">
     <p>GENE_ASSOCIATION: </p>
     <p>SUBSYSTEM: Exchange Reactions</p>
     <p>EC Number: </p>
     <p>Confidence Level: 1</p>
     <p>AUTHORS: </p>
     <p/>
    </body>
   </notes>
   <listOfReactants>
```

```
  <speciesReference species="M_L_ASP_e" stoichiometry="1"/>
 </listOfReactants>
 <kineticLaw>
  <math xmlns="http://www.w3.org/1998/Math/MathML">
   <ci> FLUX_VALUE </ci>
  </math>
  <listOfParameters>
   <parameter id="LOWER_BOUND" value="0" units="mmol_per_gDW_per_hr" constant="true"/>
   <parameter id="UPPER_BOUND" value="1000" units="mmol_per_gDW_per_hr" constant="true"/>
   <parameter id="FLUX_VALUE" value="0" units="mmol_per_gDW_per_hr" constant="true"/>
   <parameter id="OBJECTIVE_COEFFICIENT" value="0" units="mmol_per_gDW_per_hr" constant="true"/>
  </listOfParameters>
 </kineticLaw>
</reaction>
<reaction id="R_R831" name="L-Cysteine exchange" reversible="true">
 <notes>
  <body xmlns="http://www.w3.org/1999/xhtml">
   <p>GENE_ASSOCIATION: </p>
   <p>SUBSYSTEM: Exchange Reactions</p>
   <p>EC Number: </p>
   <p>Confidence Level: 1</p>
   <p>AUTHORS: </p>
   <p/>
  </body>
 </notes>
 <listOfReactants>
  <speciesReference species="M_L_CYS_e" stoichiometry="1"/>
 </listOfReactants>
 <kineticLaw>
```

```xml
    <math xmlns="http://www.w3.org/1998/Math/MathML">
      <ci> FLUX_VALUE </ci>
    </math>
    <listOfParameters>
      <parameter id="LOWER_BOUND" value="0" units="mmol_per_gDW_per_hr" constant="true"/>
      <parameter id="UPPER_BOUND" value="1000" units="mmol_per_gDW_per_hr" constant="true"/>
      <parameter id="FLUX_VALUE" value="0" units="mmol_per_gDW_per_hr" constant="true"/>
      <parameter id="OBJECTIVE_COEFFICIENT" value="0" units="mmol_per_gDW_per_hr" constant="true"/>
    </listOfParameters>
  </kineticLaw>
</reaction>
<reaction id="R_R832" name="L-Glutamate exchange" reversible="true">
  <notes>
    <body xmlns="http://www.w3.org/1999/xhtml">
      <p>GENE_ASSOCIATION: </p>
      <p>SUBSYSTEM: Exchange Reactions</p>
      <p>EC Number: </p>
      <p>Confidence Level: 1</p>
      <p>AUTHORS: </p>
      <p/>
    </body>
  </notes>
  <listOfReactants>
    <speciesReference species="M_L_GLU_e" stoichiometry="1"/>
  </listOfReactants>
  <kineticLaw>
    <math xmlns="http://www.w3.org/1998/Math/MathML">
      <ci> FLUX_VALUE </ci>
    </math>
```

```
    <listOfParameters>
      <parameter id="LOWER_BOUND" value="0" units="mmol_per_gDW_per_hr" constant="true"/>
      <parameter id="UPPER_BOUND" value="1000" units="mmol_per_gDW_per_hr" constant="true"/>
      <parameter id="FLUX_VALUE" value="0" units="mmol_per_gDW_per_hr" constant="true"/>
      <parameter id="OBJECTIVE_COEFFICIENT" value="0" units="mmol_per_gDW_per_hr" constant="true"/>
    </listOfParameters>
  </kineticLaw>
</reaction>
<reaction id="R_R833" name="L-Methionine exchange" reversible="true">
  <notes>
    <body xmlns="http://www.w3.org/1999/xhtml">
      <p>GENE_ASSOCIATION: </p>
      <p>SUBSYSTEM: Exchange Reactions</p>
      <p>EC Number: </p>
      <p>Confidence Level: 1</p>
      <p>AUTHORS: </p>
      <p/>
    </body>
  </notes>
  <listOfReactants>
    <speciesReference species="M_L_MET_e" stoichiometry="1"/>
  </listOfReactants>
  <kineticLaw>
    <math xmlns="http://www.w3.org/1998/Math/MathML">
      <ci> FLUX_VALUE </ci>
    </math>
    <listOfParameters>
      <parameter id="LOWER_BOUND" value="0" units="mmol_per_gDW_per_hr" constant="true"/>
      <parameter id="UPPER_BOUND" value="1000" units="mmol_per_gDW_per_hr" constant="true"/>
```

```xml
      <parameter id="FLUX_VALUE" value="0" units="mmol_per_gDW_per_hr" constant="true"/>
      <parameter id="OBJECTIVE_COEFFICIENT" value="0" units="mmol_per_gDW_per_hr" constant="true"/>
    </listOfParameters>
  </kineticLaw>
</reaction>
<reaction id="R_R834" name="L-Threonine exchange" reversible="true">
  <notes>
    <body xmlns="http://www.w3.org/1999/xhtml">
      <p>GENE_ASSOCIATION: </p>
      <p>SUBSYSTEM: Exchange Reactions</p>
      <p>EC Number: </p>
      <p>Confidence Level: 1</p>
      <p>AUTHORS: </p>
      <p/>
    </body>
  </notes>
  <listOfReactants>
    <speciesReference species="M_L_THR_e" stoichiometry="1"/>
  </listOfReactants>
  <kineticLaw>
    <math xmlns="http://www.w3.org/1998/Math/MathML">
      <ci> FLUX_VALUE </ci>
    </math>
    <listOfParameters>
      <parameter id="LOWER_BOUND" value="0" units="mmol_per_gDW_per_hr" constant="true"/>
      <parameter id="UPPER_BOUND" value="1000" units="mmol_per_gDW_per_hr" constant="true"/>
      <parameter id="FLUX_VALUE" value="0" units="mmol_per_gDW_per_hr" constant="true"/>
      <parameter id="OBJECTIVE_COEFFICIENT" value="0" units="mmol_per_gDW_per_hr" constant="true"/>
    </listOfParameters>
```

```xml
  </kineticLaw>
</reaction>
<reaction id="R_R835" name="L-Leucine exchange" reversible="true">
  <notes>
    <body xmlns="http://www.w3.org/1999/xhtml">
      <p>GENE_ASSOCIATION: </p>
      <p>SUBSYSTEM: Exchange Reactions</p>
      <p>EC Number: </p>
      <p>Confidence Level: 1</p>
      <p>AUTHORS: </p>
      <p/>
    </body>
  </notes>
  <listOfReactants>
    <speciesReference species="M_L_LEU_e" stoichiometry="1"/>
  </listOfReactants>
  <kineticLaw>
    <math xmlns="http://www.w3.org/1998/Math/MathML">
      <ci> FLUX_VALUE </ci>
    </math>
    <listOfParameters>
      <parameter id="LOWER_BOUND" value="0" units="mmol_per_gDW_per_hr" constant="true"/>
      <parameter id="UPPER_BOUND" value="1000" units="mmol_per_gDW_per_hr" constant="true"/>
      <parameter id="FLUX_VALUE" value="0" units="mmol_per_gDW_per_hr" constant="true"/>
      <parameter id="OBJECTIVE_COEFFICIENT" value="0" units="mmol_per_gDW_per_hr" constant="true"/>
    </listOfParameters>
  </kineticLaw>
</reaction>
<reaction id="R_R836" name="L-Tryptophan exchange" reversible="true">
```

```
    <notes>
      <body xmlns="http://www.w3.org/1999/xhtml">
        <p>GENE_ASSOCIATION: </p>
        <p>SUBSYSTEM: Exchange Reactions</p>
        <p>EC Number: </p>
        <p>Confidence Level: 1</p>
        <p>AUTHORS: </p>
        <p/>
      </body>
    </notes>
    <listOfReactants>
      <speciesReference species="M_L_TRP_e" stoichiometry="1"/>
    </listOfReactants>
    <kineticLaw>
      <math xmlns="http://www.w3.org/1998/Math/MathML">
        <ci> FLUX_VALUE </ci>
      </math>
      <listOfParameters>
        <parameter id="LOWER_BOUND" value="0" units="mmol_per_gDW_per_hr" constant="true"/>
        <parameter id="UPPER_BOUND" value="1000" units="mmol_per_gDW_per_hr" constant="true"/>
        <parameter id="FLUX_VALUE" value="0" units="mmol_per_gDW_per_hr" constant="true"/>
        <parameter id="OBJECTIVE_COEFFICIENT" value="0" units="mmol_per_gDW_per_hr" constant="true"/>
      </listOfParameters>
    </kineticLaw>
  </reaction>
  <reaction id="R_R837" name="L-Tyrosine exchange" reversible="true">
    <notes>
      <body xmlns="http://www.w3.org/1999/xhtml">
        <p>GENE_ASSOCIATION: </p>
```

```xml
<p>SUBSYSTEM: Exchange Reactions</p>
<p>EC Number: </p>
<p>Confidence Level: 1</p>
<p>AUTHORS: </p>
<p/>
</body>
</notes>
<listOfReactants>
<speciesReference species="M_L_TYR_e" stoichiometry="1"/>
</listOfReactants>
<kineticLaw>
<math xmlns="http://www.w3.org/1998/Math/MathML">
<ci> FLUX_VALUE </ci>
</math>
<listOfParameters>
<parameter id="LOWER_BOUND" value="0" units="mmol_per_gDW_per_hr" constant="true"/>
<parameter id="UPPER_BOUND" value="1000" units="mmol_per_gDW_per_hr" constant="true"/>
<parameter id="FLUX_VALUE" value="0" units="mmol_per_gDW_per_hr" constant="true"/>
<parameter id="OBJECTIVE_COEFFICIENT" value="0" units="mmol_per_gDW_per_hr" constant="true"/>
</listOfParameters>
</kineticLaw>
</reaction>
<reaction id="R_R838" name="L-Valine exchange" reversible="true">
<notes>
<body xmlns="http://www.w3.org/1999/xhtml">
<p>GENE_ASSOCIATION: </p>
<p>SUBSYSTEM: Exchange Reactions</p>
<p>EC Number: </p>
<p>Confidence Level: 1</p>
```

<p>AUTHORS: </p>
<p/>
</body>
</notes>
<listOfReactants>
<speciesReference species="M_L_VAL_e" stoichiometry="1"/>
</listOfReactants>
<kineticLaw>
<math xmlns="http://www.w3.org/1998/Math/MathML">
<ci> FLUX_VALUE </ci>
</math>
<listOfParameters>
</listOfParameters>
</kineticLaw>
</reaction>
<reaction id="R_R839" name="Bicarbonate exchange" reversible="true">
<notes>
<body xmlns="http://www.w3.org/1999/xhtml">
<p>GENE_ASSOCIATION: </p>
<p>SUBSYSTEM: Exchange Reactions</p>
<p>EC Number: </p>
<p>Confidence Level: 1</p>
<p>AUTHORS: </p>
<p/>
</body>

```
  </notes>
  <listOfReactants>
   <speciesReference species="M_HCO3_e" stoichiometry="1"/>
  </listOfReactants>
  <kineticLaw>
   <math xmlns="http://www.w3.org/1998/Math/MathML">
    <ci> FLUX_VALUE </ci>
   </math>
   <listOfParameters>
    <parameter id="LOWER_BOUND" value="0" units="mmol_per_gDW_per_hr" constant="true"/>
    <parameter id="UPPER_BOUND" value="1000" units="mmol_per_gDW_per_hr" constant="true"/>
    <parameter id="FLUX_VALUE" value="0" units="mmol_per_gDW_per_hr" constant="true"/>
    <parameter id="OBJECTIVE_COEFFICIENT" value="0" units="mmol_per_gDW_per_hr" constant="true"/>
   </listOfParameters>
  </kineticLaw>
 </reaction>
 <reaction id="R_R840" name="L-Lactate exchange" reversible="true">
  <notes>
   <body xmlns="http://www.w3.org/1999/xhtml">
    <p>GENE_ASSOCIATION: </p>
    <p>SUBSYSTEM: Exchange Reactions</p>
    <p>EC Number: </p>
    <p>Confidence Level: 1</p>
    <p>AUTHORS: </p>
    <p/>
   </body>
  </notes>
  <listOfReactants>
   <speciesReference species="M_LAC_e" stoichiometry="1"/>
```

```xml
  </listOfReactants>
  <kineticLaw>
    <math xmlns="http://www.w3.org/1998/Math/MathML">
      <ci> FLUX_VALUE </ci>
    </math>
    <listOfParameters>
      <parameter id="LOWER_BOUND" value="0" units="mmol_per_gDW_per_hr" constant="true"/>
      <parameter id="UPPER_BOUND" value="1000" units="mmol_per_gDW_per_hr" constant="true"/>
      <parameter id="FLUX_VALUE" value="0" units="mmol_per_gDW_per_hr" constant="true"/>
      <parameter id="OBJECTIVE_COEFFICIENT" value="0" units="mmol_per_gDW_per_hr" constant="true"/>
    </listOfParameters>
  </kineticLaw>
</reaction>
<reaction id="R_R841" name="Ammonia exchange" reversible="true">
  <notes>
    <body xmlns="http://www.w3.org/1999/xhtml">
      <p>GENE_ASSOCIATION: </p>
      <p>SUBSYSTEM: Exchange Reactions</p>
      <p>EC Number: </p>
      <p>Confidence Level: 1</p>
      <p>AUTHORS: </p>
      <p/>
    </body>
  </notes>
  <listOfReactants>
    <speciesReference species="M_NH4_e" stoichiometry="1"/>
  </listOfReactants>
  <kineticLaw>
    <math xmlns="http://www.w3.org/1998/Math/MathML">
```

```
    <ci> FLUX_VALUE </ci>
   </math>
   <listOfParameters>
    <parameter id="LOWER_BOUND" value="-1000" units="mmol_per_gDW_per_hr" constant="true"/>
    <parameter id="UPPER_BOUND" value="1000" units="mmol_per_gDW_per_hr" constant="true"/>
    <parameter id="FLUX_VALUE" value="0" units="mmol_per_gDW_per_hr" constant="true"/>
    <parameter id="OBJECTIVE_COEFFICIENT" value="0" units="mmol_per_gDW_per_hr" constant="true"/>
   </listOfParameters>
  </kineticLaw>
</reaction>
<reaction id="R_R842" name="Trehalose exchange" reversible="true">
  <notes>
   <body xmlns="http://www.w3.org/1999/xhtml">
    <p>GENE_ASSOCIATION: </p>
    <p>SUBSYSTEM: Exchange Reactions</p>
    <p>EC Number: </p>
    <p>Confidence Level: 1</p>
    <p>AUTHORS: </p>
    <p/>
   </body>
  </notes>
  <listOfReactants>
   <speciesReference species="M_TRE_e" stoichiometry="1"/>
  </listOfReactants>
  <kineticLaw>
   <math xmlns="http://www.w3.org/1998/Math/MathML">
    <ci> FLUX_VALUE </ci>
   </math>
   <listOfParameters>
```

```xml
<parameter id="LOWER_BOUND" value="0" units="mmol_per_gDW_per_hr" constant="true"/>
<parameter id="UPPER_BOUND" value="1000" units="mmol_per_gDW_per_hr" constant="true"/>
<parameter id="FLUX_VALUE" value="0" units="mmol_per_gDW_per_hr" constant="true"/>
<parameter id="OBJECTIVE_COEFFICIENT" value="0" units="mmol_per_gDW_per_hr" constant="true"/>
</listOfParameters>
</kineticLaw>
</reaction>
<reaction id="R_R843" name="Magnesium exchange" reversible="true">
<notes>
<body xmlns="http://www.w3.org/1999/xhtml">
<p>GENE_ASSOCIATION: </p>
<p>SUBSYSTEM: Exchange Reactions</p>
<p>EC Number: </p>
<p>Confidence Level: 1</p>
<p>AUTHORS: </p>
<p/>
</body>
</notes>
<listOfReactants>
<speciesReference species="M_Mg_e" stoichiometry="1"/>
</listOfReactants>
<kineticLaw>
<math xmlns="http://www.w3.org/1998/Math/MathML">
<ci> FLUX_VALUE </ci>
</math>
<listOfParameters>
<parameter id="LOWER_BOUND" value="-1000" units="mmol_per_gDW_per_hr" constant="true"/>
<parameter id="UPPER_BOUND" value="1000" units="mmol_per_gDW_per_hr" constant="true"/>
<parameter id="FLUX_VALUE" value="0" units="mmol_per_gDW_per_hr" constant="true"/>
```

```
      <parameter id="OBJECTIVE_COEFFICIENT" value="0" units="mmol_per_gDW_per_hr" constant="true"/>
    </listOfParameters>
  </kineticLaw>
</reaction>
<reaction id="R_R844" name="HEME exchange" reversible="true">
  <notes>
    <body xmlns="http://www.w3.org/1999/xhtml">
      <p>GENE_ASSOCIATION: </p>
      <p>SUBSYSTEM: Exchange Reactions</p>
      <p>EC Number: </p>
      <p>Confidence Level: 1</p>
      <p>AUTHORS: </p>
      <p/>
    </body>
  </notes>
  <listOfReactants>
    <speciesReference species="M_HEME_e" stoichiometry="1"/>
  </listOfReactants>
  <kineticLaw>
    <math xmlns="http://www.w3.org/1998/Math/MathML">
      <ci> FLUX_VALUE </ci>
    </math>
    <listOfParameters>
      <parameter id="LOWER_BOUND" value="0" units="mmol_per_gDW_per_hr" constant="true"/>
      <parameter id="UPPER_BOUND" value="1000" units="mmol_per_gDW_per_hr" constant="true"/>
      <parameter id="FLUX_VALUE" value="0" units="mmol_per_gDW_per_hr" constant="true"/>
      <parameter id="OBJECTIVE_COEFFICIENT" value="0" units="mmol_per_gDW_per_hr" constant="true"/>
    </listOfParameters>
  </kineticLaw>
```

```
</reaction>
<reaction id="R_R845" name="Gluconate exchange" reversible="true">
  <notes>
    <body xmlns="http://www.w3.org/1999/xhtml">
      <p>GENE_ASSOCIATION: </p>
      <p>SUBSYSTEM: Exchange Reactions</p>
      <p>EC Number: </p>
      <p>Confidence Level: 1</p>
      <p>AUTHORS: </p>
      <p/>
    </body>
  </notes>
  <listOfReactants>
    <speciesReference species="M_GLCN_e" stoichiometry="1"/>
  </listOfReactants>
  <kineticLaw>
    <math xmlns="http://www.w3.org/1998/Math/MathML">
      <ci> FLUX_VALUE </ci>
    </math>
    <listOfParameters>
      <parameter id="LOWER_BOUND" value="0" units="mmol_per_gDW_per_hr" constant="true"/>
      <parameter id="UPPER_BOUND" value="1000" units="mmol_per_gDW_per_hr" constant="true"/>
      <parameter id="FLUX_VALUE" value="0" units="mmol_per_gDW_per_hr" constant="true"/>
      <parameter id="OBJECTIVE_COEFFICIENT" value="0" units="mmol_per_gDW_per_hr" constant="true"/>
    </listOfParameters>
  </kineticLaw>
</reaction>
<reaction id="R_R846" name="Cellobiose exchange" reversible="true">
  <notes>
```

```xml
    <body xmlns="http://www.w3.org/1999/xhtml">
      <p>GENE_ASSOCIATION: </p>
      <p>SUBSYSTEM: Exchange Reactions</p>
      <p>EC Number: </p>
      <p>Confidence Level: 1</p>
      <p>AUTHORS: </p>
      <p/>
    </body>
  </notes>
  <listOfReactants>
    <speciesReference species="M_CELBS_e" stoichiometry="1"/>
  </listOfReactants>
  <kineticLaw>
    <math xmlns="http://www.w3.org/1998/Math/MathML">
      <ci> FLUX_VALUE </ci>
    </math>
    <listOfParameters>
      <parameter id="LOWER_BOUND" value="0" units="mmol_per_gDW_per_hr" constant="true"/>
      <parameter id="UPPER_BOUND" value="1000" units="mmol_per_gDW_per_hr" constant="true"/>
      <parameter id="FLUX_VALUE" value="0" units="mmol_per_gDW_per_hr" constant="true"/>
      <parameter id="OBJECTIVE_COEFFICIENT" value="0" units="mmol_per_gDW_per_hr" constant="true"/>
    </listOfParameters>
  </kineticLaw>
</reaction>
<reaction id="R_R847" name="Palatinose exchange" reversible="true">
  <notes>
    <body xmlns="http://www.w3.org/1999/xhtml">
      <p>GENE_ASSOCIATION: </p>
      <p>SUBSYSTEM: Exchange Reactions</p>
```

```
      <p>EC Number: </p>
      <p>Confidence Level: 1</p>
      <p>AUTHORS: </p>
      <p/>
    </body>
  </notes>
  <listOfReactants>
    <speciesReference species="M_PALT_e" stoichiometry="1"/>
  </listOfReactants>
  <kineticLaw>
    <math xmlns="http://www.w3.org/1998/Math/MathML">
      <ci> FLUX_VALUE </ci>
    </math>
    <listOfParameters>
      <parameter id="LOWER_BOUND" value="0" units="mmol_per_gDW_per_hr" constant="true"/>
      <parameter id="UPPER_BOUND" value="1000" units="mmol_per_gDW_per_hr" constant="true"/>
      <parameter id="FLUX_VALUE" value="0" units="mmol_per_gDW_per_hr" constant="true"/>
      <parameter id="OBJECTIVE_COEFFICIENT" value="0" units="mmol_per_gDW_per_hr" constant="true"/>
    </listOfParameters>
  </kineticLaw>
</reaction>
<reaction id="R_R848" name="D-Galactose exchange" reversible="true">
  <notes>
    <body xmlns="http://www.w3.org/1999/xhtml">
      <p>GENE_ASSOCIATION: </p>
      <p>SUBSYSTEM: Exchange Reactions</p>
      <p>EC Number: </p>
      <p>Confidence Level: 1</p>
      <p>AUTHORS: </p>
```

```xml
<p/>
</body>
</notes>
<listOfReactants>
<speciesReference species="M_D_GAL_e" stoichiometry="1"/>
</listOfReactants>
<kineticLaw>
<math xmlns="http://www.w3.org/1998/Math/MathML">
<ci> FLUX_VALUE </ci>
</math>
<listOfParameters>
<parameter id="LOWER_BOUND" value="0" units="mmol_per_gDW_per_hr" constant="true"/>
<parameter id="UPPER_BOUND" value="1000" units="mmol_per_gDW_per_hr" constant="true"/>
<parameter id="FLUX_VALUE" value="0" units="mmol_per_gDW_per_hr" constant="true"/>
<parameter id="OBJECTIVE_COEFFICIENT" value="0" units="mmol_per_gDW_per_hr" constant="true"/>
</listOfParameters>
</kineticLaw>
</reaction>
<reaction id="R_R849" name="PHB exchange" reversible="true">
<notes>
<body xmlns="http://www.w3.org/1999/xhtml">
<p>GENE_ASSOCIATION: </p>
<p>SUBSYSTEM: Exchange Reactions</p>
<p>EC Number: </p>
<p>Confidence Level: 1</p>
<p>AUTHORS: </p>
<p/>
</body>
</notes>
```

```xml
  <listOfReactants>
    <speciesReference species="M_PHB_e" stoichiometry="1"/>
  </listOfReactants>
  <kineticLaw>
    <math xmlns="http://www.w3.org/1998/Math/MathML">
      <ci> FLUX_VALUE </ci>
    </math>
    <listOfParameters>
      <parameter id="LOWER_BOUND" value="0" units="mmol_per_gDW_per_hr" constant="true"/>
      <parameter id="UPPER_BOUND" value="1000" units="mmol_per_gDW_per_hr" constant="true"/>
      <parameter id="FLUX_VALUE" value="0" units="mmol_per_gDW_per_hr" constant="true"/>
      <parameter id="OBJECTIVE_COEFFICIENT" value="0" units="mmol_per_gDW_per_hr" constant="true"/>
    </listOfParameters>
  </kineticLaw>
</reaction>
<reaction id="R_R850" name="PHV exchange" reversible="true">
  <notes>
    <body xmlns="http://www.w3.org/1999/xhtml">
      <p>GENE_ASSOCIATION: </p>
      <p>SUBSYSTEM: Exchange Reactions</p>
      <p>EC Number: </p>
      <p>Confidence Level: 1</p>
      <p>AUTHORS: </p>
      <p/>
    </body>
  </notes>
  <listOfReactants>
    <speciesReference species="M_PHV_e" stoichiometry="1"/>
  </listOfReactants>
```

```
<kineticLaw>
 <math xmlns="http://www.w3.org/1998/Math/MathML">
  <ci> FLUX_VALUE </ci>
 </math>
 <listOfParameters>
  <parameter id="LOWER_BOUND" value="0" units="mmol_per_gDW_per_hr" constant="true"/>
  <parameter id="UPPER_BOUND" value="1000" units="mmol_per_gDW_per_hr" constant="true"/>
  <parameter id="FLUX_VALUE" value="0" units="mmol_per_gDW_per_hr" constant="true"/>
  <parameter id="OBJECTIVE_COEFFICIENT" value="0" units="mmol_per_gDW_per_hr" constant="true"/>
 </listOfParameters>
</kineticLaw>
</reaction>
<reaction id="R_R851" name="PHO exchange" reversible="true">
<notes>
 <body xmlns="http://www.w3.org/1999/xhtml">
  <p>GENE_ASSOCIATION: </p>
  <p>SUBSYSTEM: Exchange Reactions</p>
  <p>EC Number: </p>
  <p>Confidence Level: 1</p>
  <p>AUTHORS: </p>
  <p/>
 </body>
</notes>
<listOfReactants>
 <speciesReference species="M_PHO_e" stoichiometry="1"/>
</listOfReactants>
<kineticLaw>
 <math xmlns="http://www.w3.org/1998/Math/MathML">
  <ci> FLUX_VALUE </ci>
```

```
    </math>
    <listOfParameters>
      <parameter id="LOWER_BOUND" value="0" units="mmol_per_gDW_per_hr" constant="true"/>
      <parameter id="UPPER_BOUND" value="1000" units="mmol_per_gDW_per_hr" constant="true"/>
      <parameter id="FLUX_VALUE" value="0" units="mmol_per_gDW_per_hr" constant="true"/>
      <parameter id="OBJECTIVE_COEFFICIENT" value="0" units="mmol_per_gDW_per_hr" constant="true"/>
    </listOfParameters>
  </kineticLaw>
</reaction>
<reaction id="R_R852" name="PHD exchange" reversible="true">
  <notes>
    <body xmlns="http://www.w3.org/1999/xhtml">
      <p>GENE_ASSOCIATION: </p>
      <p>SUBSYSTEM: Exchange Reactions</p>
      <p>EC Number: </p>
      <p>Confidence Level: 1</p>
      <p>AUTHORS: </p>
      <p/>
    </body>
  </notes>
  <listOfReactants>
    <speciesReference species="M_PHD_e" stoichiometry="1"/>
  </listOfReactants>
  <kineticLaw>
    <math xmlns="http://www.w3.org/1998/Math/MathML">
      <ci> FLUX_VALUE </ci>
    </math>
    <listOfParameters>
      <parameter id="LOWER_BOUND" value="0" units="mmol_per_gDW_per_hr" constant="true"/>
```

```
        <parameter id="UPPER_BOUND" value="1000" units="mmol_per_gDW_per_hr" constant="true"/>
        <parameter id="FLUX_VALUE" value="0" units="mmol_per_gDW_per_hr" constant="true"/>
        <parameter id="OBJECTIVE_COEFFICIENT" value="0" units="mmol_per_gDW_per_hr" constant="true"/>
      </listOfParameters>
    </kineticLaw>
  </reaction>
  <reaction id="R_R853" name="PHDD exchange" reversible="true">
    <notes>
      <body xmlns="http://www.w3.org/1999/xhtml">
        <p>GENE_ASSOCIATION: </p>
        <p>SUBSYSTEM: Exchange Reactions</p>
        <p>EC Number: </p>
        <p>Confidence Level: 1</p>
        <p>AUTHORS: </p>
        <p/>
      </body>
    </notes>
    <listOfReactants>
      <speciesReference species="M_PHDD_e" stoichiometry="1"/>
    </listOfReactants>
    <kineticLaw>
      <math xmlns="http://www.w3.org/1998/Math/MathML">
        <ci> FLUX_VALUE </ci>
      </math>
      <listOfParameters>
        <parameter id="LOWER_BOUND" value="0" units="mmol_per_gDW_per_hr" constant="true"/>
        <parameter id="UPPER_BOUND" value="1000" units="mmol_per_gDW_per_hr" constant="true"/>
        <parameter id="FLUX_VALUE" value="0" units="mmol_per_gDW_per_hr" constant="true"/>
        <parameter id="OBJECTIVE_COEFFICIENT" value="0" units="mmol_per_gDW_per_hr" constant="true"/>
```

```xml
    </listOfParameters>
  </kineticLaw>
</reaction>
<reaction id="R_R854" name="H exchange" reversible="true">
  <notes>
    <body xmlns="http://www.w3.org/1999/xhtml">
      <p>GENE_ASSOCIATION: </p>
      <p>SUBSYSTEM: Exchange Reactions</p>
      <p>EC Number: </p>
      <p>Confidence Level: 1</p>
      <p>AUTHORS: </p>
      <p/>
    </body>
  </notes>
  <listOfReactants>
    <speciesReference species="M_H_e" stoichiometry="1"/>
  </listOfReactants>
  <kineticLaw>
    <math xmlns="http://www.w3.org/1998/Math/MathML">
      <ci> FLUX_VALUE </ci>
    </math>
    <listOfParameters>
      <parameter id="LOWER_BOUND" value="-1000" units="mmol_per_gDW_per_hr" constant="true"/>
      <parameter id="UPPER_BOUND" value="1000" units="mmol_per_gDW_per_hr" constant="true"/>
      <parameter id="FLUX_VALUE" value="0" units="mmol_per_gDW_per_hr" constant="true"/>
      <parameter id="OBJECTIVE_COEFFICIENT" value="0" units="mmol_per_gDW_per_hr" constant="true"/>
    </listOfParameters>
  </kineticLaw>
</reaction>
```

```xml
<reaction id="R_R855" name="Lactose exchange" reversible="true">
 <notes>
  <body xmlns="http://www.w3.org/1999/xhtml">
   <p>GENE_ASSOCIATION: </p>
   <p>SUBSYSTEM: Exchange Reactions</p>
   <p>EC Number: </p>
   <p>Confidence Level: 1</p>
   <p>AUTHORS: </p>
   <p/>
  </body>
 </notes>
 <listOfReactants>
  <speciesReference species="M_LACTS_e" stoichiometry="1"/>
 </listOfReactants>
 <kineticLaw>
  <math xmlns="http://www.w3.org/1998/Math/MathML">
   <ci> FLUX_VALUE </ci>
  </math>
  <listOfParameters>
   <parameter id="LOWER_BOUND" value="0" units="mmol_per_gDW_per_hr" constant="true"/>
   <parameter id="UPPER_BOUND" value="1000" units="mmol_per_gDW_per_hr" constant="true"/>
   <parameter id="FLUX_VALUE" value="0" units="mmol_per_gDW_per_hr" constant="true"/>
   <parameter id="OBJECTIVE_COEFFICIENT" value="0" units="mmol_per_gDW_per_hr" constant="true"/>
  </listOfParameters>
 </kineticLaw>
</reaction>
<reaction id="R_R856" name="Stachyose exchange" reversible="true">
 <notes>
  <body xmlns="http://www.w3.org/1999/xhtml">
```

```
          <p>GENE_ASSOCIATION: </p>
          <p>SUBSYSTEM: Exchange Reactions</p>
          <p>EC Number: </p>
          <p>Confidence Level: 1</p>
          <p>AUTHORS: </p>
          <p/>
        </body>
      </notes>
      <listOfReactants>
        <speciesReference species="M_STCYS_e" stoichiometry="1"/>
      </listOfReactants>
      <kineticLaw>
        <math xmlns="http://www.w3.org/1998/Math/MathML">
          <ci> FLUX_VALUE </ci>
        </math>
        <listOfParameters>
          <parameter id="LOWER_BOUND" value="0" units="mmol_per_gDW_per_hr" constant="true"/>
          <parameter id="UPPER_BOUND" value="1000" units="mmol_per_gDW_per_hr" constant="true"/>
          <parameter id="FLUX_VALUE" value="0" units="mmol_per_gDW_per_hr" constant="true"/>
          <parameter id="OBJECTIVE_COEFFICIENT" value="0" units="mmol_per_gDW_per_hr" constant="true"/>
        </listOfParameters>
      </kineticLaw>
    </reaction>
  </listOfReactions>
 </model>
</sbml>
```
